# Yeast-Driven and Bioimpedance-Sensitive Biohybrid Soft Robots

**DOI:** 10.34133/cbsystems.0233

**Published:** 2025-04-25

**Authors:** MennaAllah Soliman, Frederick Forbes, Dana D. Damian

**Affiliations:** ^1^ School of Electrical and Electronic Engineering, University of Sheffield, Sheffield S1 3JD, UK.; ^2^ Insigneo Institute for *in silico* Medicine, University of Sheffield, Sheffield, UK.; ^3^ Sheffield Robotics, University of Sheffield, Sheffield, UK.

## Abstract

Biohybrid robots integrate biological components with synthetic materials to harness the unique capabilities of living systems for robotic functions. This study focuses on leveraging yeast fermentation dynamics to enable actuation and sensing in soft robotic systems. By leveraging yeast’s natural ability to produce carbon dioxide and generate pressure during fermentation, we demonstrate the feasibility of creating biohybrid robots with lifelike behavior and adaptability. Our research integrates bioimpedance sensing into track yeast behavior and metabolic dynamics in real time. We developed an adjustable single-resistor oscillator circuit by using a digital potentiometer to measure impedance frequency and model the yeast growth rate. Experimental results reveal the sensitivity of the single-resistor oscillator circuit to variations in yeast concentration and demonstrate the correlation between yeast behavior and actuation power. Furthermore, we highlight the potential of yeast-driven robots for various applications by demonstrating a yeast-driven soft limb capable of rotating 140° tested at different temperatures, an inflatable membrane actuator functioning as a tactile sensor detecting forces up to 4.5 N, a palpation probe for differentiating tissue stiffness, and a gripper capable of manipulating objects. This work lays the foundation for advancing biohybrid robotics by integrating yeast fermentation dynamics with bioimpedance sensing, enhancing the functionality of robotic systems.

## Introduction

Soft robots, inspired by the adaptability of biological organisms, combine sensing, actuation, and control to create highly flexible and versatile robotic systems [1]. However, replicating the intricate communication and intelligence living cells exhibit remains a challenge [2]. Rossiter [3] asserts the necessity of conceptualizing future robots as robotic organisms rather than machines, advocating for a paradigm shift in robotics systems. Recent advancements, such as those highlighted by Zhou et al. [4], emphasize the integration of actuation and sensing to achieve intelligent soft robots capable of adapting to their environments. Biohybrid robots integrate biological cells with artificial components to harness cells’ inherent actuation and sensing capabilities [5,6]. These microsystems are designed for microenvironment operation, including ex vivo and in vivo settings [7], and have the potential for intelligent drug delivery, with applications in medicine and healthcare and challenges for clinical translation [8]. Furthermore, stimuli-responsive living materials and biosensing technologies open new boundaries in environmental adaptation and controllability over biohybrid robots [9]. Biohybrid robots can be categorized into 4 groups based on their composition and design: tissue-based biohybrids [10], microbial biohybrids [11], DNA biohybrid robots [12], and cyborg robots [13]. Among these categories, tissue-based biohybrid robots are the most well-known.

Tissue-based biohybrid robots are constructed by incorporating tissue such as cardiomyocytes obtained from neonatal rats or derived from human induced pluripotent stem cells onto robotic or soft structures. One notable example is xenobots, a method for creating in vitro biological robots introduced by Blackiston et al. [14]. Recently, Kim et al. [15] presented a new type of robot that combines living cells with electronic components to create motion. These biohybrid machines can be wirelessly controlled and perform walking, turning, and object transportation functions. The study by Filippi et al. [16] integrated artificial structures with living biosystems and was advancing toward autonomous control and state awareness through innovations like piezoresistive sensors that detect and respond to muscle contractions, thus enhancing their functionality and intelligence. Advancements in control mechanisms for muscle-powered robots, highlighting the potential of biological actuators in soft robotics, are discussed by Bawa and Raman [17]. Additionally, the progression from biomimicry to biohybrid system design emphasizing dynamic adaptation in engineered systems is explored by Raman and Bashir [18]. Incorporating such control and design principles has broadened the scope of biohybrid robotics, particularly in creating adaptable and intelligent systems.

Microbial biohybrid robots consist of unicellular microorganisms, predominantly bacteria, integrated with micro/nanoscale functional components. They have demonstrated controlled actuation for cargo transport and assembly, and recent advancements have led to the development of specialized microrobotic systems for therapeutic cargo delivery [11], biofilm treatment [19], and high-throughput micro-object manipulation [20]. Another group used yeast and demonstrated thin film ZnO/Si surface acoustic waves to manipulate microparticles and yeast cells in a microchamber, enabling 3-dimensional patterning in the study by Tao et al. [21]. Using yeast as a biosensing platform, the study by Crnković et al. [22] demonstrated that a living yeast biosensor can effectively detect and differentiate single-amino-acid changes in peptide ligands, suggesting potential applications in diagnostics and scalable communication systems.

Critical challenges biohybrid robots face include biohybrid robot design and modeling, scalable fabrication, autonomous control, and translating them beyond the lab [23]. These complexities appear due to the multifaceted nature of biological and robotic components and their dynamic interactions. Designing such robots necessitates a profound understanding of biological behaviors and robotic principles, demanding the harmonious integration of these domains while considering nonlinear biological responses and uncertainties in modeling [24]. Achieving autonomous control in unpredictable biological dynamics demands advanced control algorithms that can adapt to variations. Translating biohybrid robots beyond controlled lab environments involves navigating challenges related to stability to live in uncontrolled environments and ethical considerations [25]. While cell-based biohybrid robots rely on muscle tissue for actuation, our study demonstrates a microbial approach using yeast fermentation for actuation and sensing. This approach explores the potential of yeast as a scalable, renewable, and biocompatible actuation source, differing from cell-driven systems. By leveraging yeast’s unique metabolic properties, we aim to expand the possibilities for biohybrid systems in applications that demand environmental adaptability and simplicity in design.

The first step for biohybrid control is feedback from biological component status and how to sense it. Bioimpedance is a noninvasive technique used to measure the electrical properties of biological tissues. The living organism’s components act as electrical impedance elements by applying electrical current to the tissue [26]. Bioimpedance technology offers a promising solution by enabling the development of biohybrid robots that can sense their internal state and detect external environmental effects, enhancing their observability and controllability. Different electrical models, including integer-based [27] and fractional order models [28], represent the electrical behavior of biological tissues. Bioimpedance was used in soft robotics as a sensor for external parameters, and a portable soft gripper was designed in a study [29] to measure the quality of fruits, such as apples, without damaging their tissues, as bioimpedance and soft materials are nonharmful to fruits. Additionally, a single-resistor oscillator (SCRO) circuit combined with an analog multiplexer was employed in a different study [30] to design soft E-skin using aloe vera gel.

**Fig. 1.**
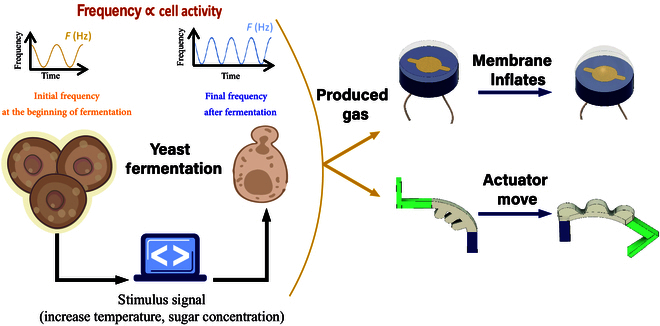
Bioimpedance sensing for a biohybrid robot, where yeast fermentation plays a crucial role in actuation and sensing. During fermentation, yeast cells undergo division, which affects the yeast culture’s impedance. Concurrently, carbon dioxide is produced as a byproduct of fermentation, which the robot utilizes as a power source for motion. The bioimpedance frequency serves as a feedback sensor for monitoring the biological components in biohybrid.

In this study, our contributions include employing bioimpedance technology to characterize the electrical properties of biological tissues and cells using an adjustable SCRO as a sensor, which uses a digital potentiometer to measure the impedance frequency for a biohybrid system described in Fig. 1. We investigated the relationship between actuator motion and the state of yeast cells using impedance frequency readings from the SCRO circuit, aiming to leverage yeast as a potential proprioceptive and exteroceptive sensor for biohybrid robotic systems. Changes in impedance frequency reflect alterations in the yeast’s metabolic activity and cell structure, providing a direct means to track gas production and growth dynamics. The SCRO offers several advantages over traditional bioimpedance measurement methods, including simplicity, cost-effectiveness, and compactness, making it particularly suitable for portable and scalable systems. Furthermore, its design allows for miniaturization, enabling its use in biomedical applications, such as implantable biosensors for tracking cellular or tissue activity. Unlike conventional impedance analyzers, the SCRO provides continuous, noninvasive monitoring of impedance frequency changes, directly reflecting yeast metabolic activity. Additionally, our implementation features a digital potentiometer, allowing precise sensitivity adjustments to accommodate varying fermentation conditions.

In addition, we studied the effect of yeast growth on mechanical properties such as pressure, volume, and force and established correlations with yeast frequency. We also modeled the relationship between pressure and yeast frequency to correlate the actuation power with the dynamic behavior of yeast. Furthermore, we studied the regeneration of the actuation power using yeast and its impact on frequency sensing. Yeast offers several advantages compared to traditional actuation and sensing systems. Unlike conventional systems requiring pumps, valves, or batteries, yeast fermentation provides a natural and biodegradable source of actuation power through carbon dioxide generation. This untethered approach simplifies the design by eliminating the need for additional mechanical components and external energy sources. Moreover, yeast systems are highly adaptive and cost-effective, making them suitable for scalable and sustainable robotic applications both inside the body as biomedical applications and in environmental settings. Bioimpedance allows yeast to act as both an actuation element and a sensor, providing a dual-purpose capability without the need for additional external sensors. Our study analyzed yeast’s actuation and sensing capabilities across 2 soft robotic systems: an inflatable membrane actuator and a soft limb actuator. We evaluated the influence of temperature on controlling a yeast-actuated soft limb and its effect on yeast impedance. Additionally, we explore using an inflated membrane actuator filled with yeast to study how external forces affect internal pressure and how impedance can detect these changes, enabling its application as tactile sensing. This method allows the use of yeast impedance to monitor not only the internal states of robotic systems but also environmental changes. This study highlights the potential of yeast-driven systems in practical applications by demonstrating a yeast-driven probe for tissue palpation, capable of differentiating tissue stiffness, and a yeast-driven gripper for object grasping, showcasing their sensitivity and mechanical responsiveness through bioimpedance sensing.

## Materials and Methods

### Yeast’s metabolism characteristics

In microbiology, yeast strains, such as *Saccharomyces cerevisiae*, are easily cultivated in nutrient-rich media containing carbon and nitrogen sources, vitamins, salts, and essential minerals at a temperature of around 30 to 40 °C. The growth follows a recognizable pattern—beginning with a lag phase after injection, followed by an exponential growth phase where cells multiply rapidly and produce more carbon dioxide. The exponential growth can be mathematically described, with the growth rate varying with temperature in Eq. (1).

$$N=N_{0}e^{\mathrm{kt}}$$ (1)

$$k=\text{ln}\left(2\right)/T$$ (2)

N is the number of yeast cells over time, and $N_{0}$ is the initial number. k is the growth constant represented in Eq. (2), where *T* is the doubling time of the culture. After several doubling times, yeast enters a stationary phase in which ethanol is produced, characterized by slowed growth due to nutrient depletion. Cells in this phase undergo physiological changes, including carbohydrate accumulation and enhanced cell wall resilience. This phase can persist until conditions improve, but if not, cells enter the death phase [31]. The exponential model describes the yeast growth phase, as shown in Eqs. (1) and (2). A detailed visualization of this process, including the impedance frequency changes over time, is provided in Fig. S3. There are 2 primary types of yeast fermentation: aerobic fermentation, which occurs in open air, and anaerobic fermentation. Anaerobic fermentation occurs when yeast ferments in a closed chamber, producing carbon dioxide pressure as a byproduct of yeast growth. In this study, we utilize anaerobic fermentation as an actuation power while simultaneously monitoring the dynamics of yeast growth, which are then transferred to actuator dynamics for further analysis.

### Oscillator circuit and bioimpedance model for yeast dynamics

Bioimpedance presents a noninvasive approach for tracking various parameters of yeast fermentation, such as cell density, growth rate, and environmental factors (e.g., temperature or pH). It enables continuous monitoring of yeast behavior as it metabolizes nutrients and produces metabolites. The methodology involves measuring changes in impedance frequency over time between 2 electrodes immersed in the fermentation medium using the oscillator circuit. As yeast cells undergo growth and metabolic processes, they alter the electrical properties of the medium, leading to changes in impedance that the electrodes can detect. The electrical properties of the biological component are modeled using the Cole model of bioimpedance shown in Eq. (3). This model effectively characterizes biological tissue by combining resistance and capacitance.

Under low-current conditions, the cell wall behaves as a capacitance, while the intracellular liquid exhibits low resistance, referred to as $R_{\mathrm{cw}}$. In contrast, the extracellular liquid displays high-resistance characteristics referred to as $R_{\mathrm{Ex}}$ with τ as the time constant and α as the Warburg coefficient. The Warburg coefficient represents the impedance contribution from diffusion-controlled processes, such as the movement of ions or molecules within the fermentation medium. In our bioimpedance model, this coefficient captures the diffusion dynamics associated with yeast metabolic activity and contributes to understanding the system’s electrochemical behavior over time. This model helps elucidate yeast cells’ complex electrical behavior during fermentation processes.

$$Z\left(\omega \right)=R_{\mathrm{Ex}}+\frac{R_{\mathrm{cw}}-R_{\mathrm{Ex}}}{1+{\left(\mathrm{j\omega \tau}\right)}^{\alpha }}$$ (3)

**Fig. 2.**
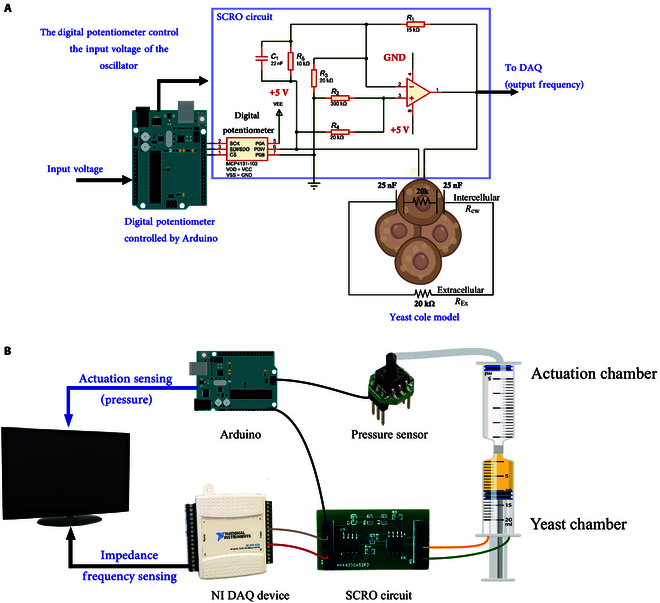
Bioimpedance and pressure measurement system for yeast-driven actuation during anaerobic fermentation. (A) Indirect bioimpedance measurement system using a single-resistor oscillator (SCRO) and a yeast solution equivalent circuit, where *R*_cw_ is the capacitance and resistance of the cell wall and inside the cell liquid, while *R*_Ex_ is the resistance of the extracellular liquid. (B) Experimental setup for measuring the frequency and pressure of yeast during anaerobic fermentation. The yeast chamber is connected to the designed SCRO circuit to read impedance frequency, and the produced gas is trapped inside a 5-ml actuation chamber. This chamber is connected to a pressure sensor to detect changes in pressure due to yeast growth and impedance changes. GND, ground; DAQ, data acquisition; NI, National Instruments.

The yeast impedance measurement methodology followed is an indirect approach, as introduced by Mohsen et al. [32]. This method employs an SCRO circuit in Fig. 2A. Its variable components are adjusted based on the Cole model of yeast growth rate shown in Table 1 and Eq. (3), depicted in Eqs. (4) and (5).

$$\begin{array}{c} \begin{array}{c} \begin{array}{l} \text{Real}\;=\;21C_{\mathrm{cw}}^{2}R_{\mathrm{cw}}\left(900.49\;\times \;10^{3}R_{\mathrm{Ex}}-12.14\;\times \;10^{7}\right)\omega^{\alpha +1}\text{cos}\left(\frac{\left(\alpha \;+\;1\right)\pi }{2}\right)\\ \;+C_{\mathrm{cw}}R_{\mathrm{cw}}\left(44.7R_{\mathrm{Ex}}-2.4\right)\omega^{\alpha }\text{cos}\left(\frac{\alpha \pi }{2}\right)+4.47\;\times \;10^{-6}R_{\mathrm{Ex}}R_{\mathrm{cw}}\\ \;+44.7R_{\mathrm{Ex}}+100\;\times \;10^{-9}R_{\mathrm{cw}}-\;2.4=0 \end{array} \end{array} \end{array}$$ (4)

$$\begin{array}{c} \begin{array}{c} \begin{array}{c} \begin{array}{l} \text{Imag}.=\\ 21C_{\mathrm{cw}}^{2}R_{\mathrm{cw}}\left(900.49\;\times \;10^{3}R_{\mathrm{Ex}}-12.14\;\times \;10^{7}\right)\omega^{\alpha +1}\text{sin}\left(\frac{\left(\alpha +1\right)\pi }{2}\right)+{\text{CcwR}}_{\mathrm{cw}}\\ \left(44.7R_{\mathrm{Ex}}-2.4\right)\omega^{\alpha }\text{sin}\left(\frac{\alpha \pi }{2}\right)+21C_{\mathrm{cw}}\left(2.03R_{\mathrm{Ex}}+2.03R_{\mathrm{cw}}\;+\;0.2\right)\omega =0 \end{array} \end{array} \end{array} \end{array}$$ (5)

**Table 1.** Yeast solution Cole model parameters [33]

Yeast	Cole model parameters		
	*C*_cw_ (nF)	*R*_cw_ (Ω)	*R*_Ex_ (Ω)
5% living	17.68	2.82	25.74
10% living	15.40	3.14	20.68
20% living	7.42	5.62	40.52

Notably, the digital potentiometer in the study is adjusted to achieve oscillation around the bioimpedance frequency. Central to this concept is the inverse relationship between capacitance and frequency. By applying an appropriate voltage to the oscillator, frequency amplification is achieved, enabling effective measurement. In this work, the digital potentiometer in the SCRO circuit has been replaced with a digital potentiometer controlled by an Arduino microcontroller. The Arduino adjusts the resistance values in real time to achieve oscillation in the circuit, enabling precise tuning for optimal bioimpedance measurements. This approach ensures sensitivity and adaptability in dynamic conditions, making it particularly effective for monitoring yeast fermentation processes. In this study, the SCRO circuit was simulated using the yeast Cole model parameters reported by Wang et al. [33] (see Table 1) to account for the bioimpedance characteristics of yeast at different growth stages. The digital potentiometer ($R_{v}$) was iteratively adjusted during the simulations to achieve oscillation within the desired frequency range. The resulting SCRO component values, spanning 10 to 15 kΩ, correspond to the impedance range of yeast during fermentation and are summarized in Table 2.

**Table 2.** SCRO oscillator parameters

*R*_1_ (kΩ)	*R*_2_ (kΩ)	*R*_3_ (kΩ)	*R*_4_ (kΩ)	*R*_5_ (kΩ)	*C* (nF)	*R*_v_ (kΩ)
15	300	20	20	10	22	10–15

By utilizing yeast dynamics (Eqs. (1) and (2)), represented by the biological factors of fermentation, along with the SCRO circuit equations (Eqs. (4) and (5)), we model the yeast growth rate to generate actuation power as an electric model. This model correlates impedance frequency with actuator outputs, such as pressure or deflection angle, enabling the system to predict and control desired behaviors. For instance, the desired behavior in a soft robotic membrane actuator might involve achieving a target pressure for inflation. Similarly, the desired behavior in a soft limb actuator could be a controllable deflection angle (e.g., 140°). This capability makes bioimpedance a dual-purpose sensor: it monitors the yeast’s state and provides feedback to predict actuator performance.

### Yeast-based biohybrid actuation power

This investigation focuses on the potential of yeast-produced gas to actuate a soft pneumatic actuator. Central to this mechanism is the gas volume generated by yeast and the resulting pressure exerted. The experimental setup, shown in Fig. 2B, is designed to measure pressure within a fixed volume, utilizing a 5-ml syringe as the actuation chamber and applying the ideal gas law to analyze the process. The yeast chamber is linked to the actuation chamber, which is connected to a pressure sensor (XGZP6847A Pressure Sensor Module) with a range of 0 to 7 bar. The pressure sensor reads pressure with an Arduino Uno every second. The yeast chamber was constructed from a 10-ml fully closed syringe, with impedance electrodes positioned 3 cm apart at the bottom. These electrodes are connected to the SCRO circuit described above to measure the impedance frequency. The circuit is interfaced with Arduino Uno to regulate the digital potentiometer and provide a 5-V power supply. A National Instruments data acquisition system acquired the impedance frequency readings using the DAQ Express software. The experiment measured pressure and yeast impedance frequency to investigate the correlation between physical power (pressure) and yeast growth kinetics. The pressure dynamics paralleled yeast growth behavior, showing an initial lag phase followed by a rapid increase during exponential growth. By correlating the frequency impedance, which represents yeast growth, with the pressure changes observed in the experiment, we demonstrated a direct relationship between yeast activity and gas production within the chamber.

#### Yeast impedance frequency

The initial phase of the study aims to assess the SCRO circuit’s capability to detect yeast dynamics. For testing, a yeast mixture consisting of 10 ml of water at 38 to 40 °C mixed with 2.25 g of yeast (22.5% concentration) is utilized. We exploit the sugar concentration to examine its impact on yeast frequency and output actuation power, employing concentrations of 3% (0.3 g), 6% (0.6 g), 12% (1.2 g), and 21% (2.1 g) sugar in yeast mixture preparation as shown in Fig. S1.

The yeast mixture is added into the yeast chamber, as illustrated in Fig. 2B, where it undergoes anaerobic fermentation, generating carbon dioxide and inducing pressure in the actuator chamber, monitored by the pressure sensor. The experiment includes 5 trials for each sugar concentration, each lasting 10 min. These parameters are determined based on findings from the yeast literature [34], as excessive sugar can be lethal to yeast, while insufficient sugar may impede yeast reactivity.

**Fig. 3.**
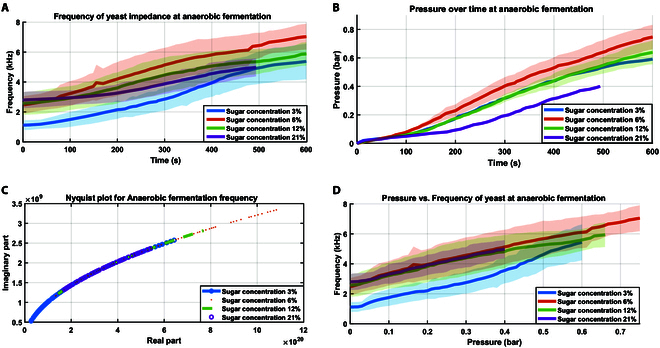
Yeast impedance frequency and yeast pressure at different sugar concentrations during anaerobic fermentation inside the actuation chamber. (A) Change in frequency over time at different sugar concentrations. The shaded area represents the 95% confidence level over 5 trials for each concentration, each with a 10-min duration, except for the 21% concentration due to foam filling the actuation chamber around 8 min. (B) Change in pressure over time for 5 trials, each with a 10-min duration, except for the 21% concentration due to foam filling the actuation chamber around 8 min affecting pressure reading. The solid line represents the mean, and the shaded areas represent the standard deviation over 5 trials for each concentration. (C) Nyquist plot of impedance frequency’s real and imaginary parts at different sugar concentrations. (D) Change in impedance frequency inside the yeast chamber versus pressure inside the actuation chamber over 5 trials, each with a 10-min duration. The shaded area indicates the 95% confidence level for the 5 trials.

Fig. 3A shows the frequency changes over time for 5 trials, with the mean and standard deviation. Initially, the frequency increases with higher sugar concentrations, indicating increased yeast activity and growth rate. However, after 1.5 min, the frequency for the 21% concentration drops below those of the 12% and 6% concentrations. Notably, the 6% concentration consistently exhibits a higher frequency than the 12% and 21% concentrations.

The yeast frequency for 3%, 12%, and 21% sugar concentrations are evaluated in aerobic fermentation, as depicted in Fig. S2, by measuring yeast frequency in a Petri dish equipped with an impedance electrode. Results show increased frequency with escalating sugar concentration. The disparity observed in the frequency results between aerobic and anaerobic fermentation can be attributed to the influence of pressure from the anaerobic fermentation on yeast-produced carbon dioxide. In anaerobic fermentation, per Henry’s law, a portion of the dissolved carbon dioxide gas in the yeast mixture transforms into foam as it is in a closed container, affecting the output pressure and the measured frequency [35]. This phenomenon may occur due to the altered physical and chemical conditions within the fermentation environment, leading to variations in gas dissolution and subsequent foam formation. This explanation aligns with scientific principles governing gas–liquid interactions and provides insight into the observed differences in frequency results between the 2 fermentation conditions.

In Fig. 3B, the imaginary and real parts of the frequency were calculated using Eqs. (4) and (5), respectively, with a 0.9 alpha coefficient based on the yeast Cole model. Due to the experimental setup, which involves capturing yeast frequency data for 10 min from the yeast’s significantly longer lifespan (which can last for months), the lifespan frequency of yeast shown in Fig. S3, the Nyquist plot displays only a partial curve due to a restricted frequency range. Despite this limitation, the Nyquist plot reveals that the sugar concentrations exhibit similar frequency ranges. In contrast, the rate of frequency change over time differs, as illustrated in Fig. 3A. This distinction underscores the distinct dynamics associated with varying sugar concentrations during fermentation. Eventually, the SCRO circuit effectively detects and represents the changes occurring within the yeast, highlighting its ability to capture the nuanced dynamics of yeast fermentation.

#### Pressure profile with the yeast growth rate

The pressure was measured concurrently with frequency over 5 trials for sugar concentrations of 3%, 6%, 12%, and 21% during anaerobic fermentation. The accumulation of pressure in the actuation chamber was monitored by a pressure sensor over 10 min, as displayed in Fig. 3B. Notably, the 3% concentration reached 0.6 bar, the 12% one reached 0.65 bar, the 6% one peaked at 0.75 bar, and the 21% concentration attained a maximum pressure of 0.4 bar. These pressure values obtained from yeast fermentation proved sufficient for serving as actuation power for the soft actuator. In Fig. 3B, it is evident that the 21% concentration exhibited the lowest pressure over time, while the 3%, 6%, and 12% concentrations showed no significant difference in output pressure. However, a significant development occurred as the pressure approached 0.1 bar, as illustrated in Fig. S4. At this juncture, yeast foam began infiltrating the actuation chamber, influenced by the gas bubbles within the yeast chamber. In particular, the experiment with the 21% concentration had to be halted prematurely at around 8.5 min, as the yeast foam inside the actuation chamber exceeded its capacity, affecting the experimental setup. The relationship between pressure change and frequency is depicted in Fig. 3D, exhibiting similar behavior to the frequency change over time shown in Fig. 3A. This observation indicates that frequency can effectively detect pressure changes, facilitating the establishment of a model correlating yeast state with output actuation power at constant volume.

**Fig. 4.**
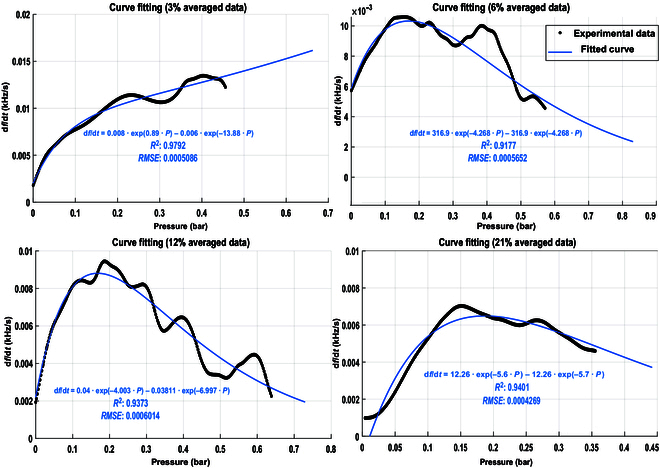
Mathematical model of pressure vs. rate of change in impedance frequency at different sugar concentrations. The equations describe the relationship between the averaged data over 5 trials of the rate of change in frequency versus the change in pressure for each concentration. These equations relate the growth rate of yeast as a biological actuation element to the pressure output for actuation power using the rate of change in impedance frequency as a sensing part. RMSE, root mean square error.

The relationship between pressure and the frequency rate of change over time for each sugar concentration is illustrated in Fig. 4. The experimental data average was fitted to a second-order exponential equation, resembling the behavior of yeast dynamics described in Eq. (1). The coefficient of determination (*R*^2^) values were found to be 0.97, 0.91, 0.93, and 0.94 for sugar concentrations of 3%, 6%, 12%, and 21%, respectively. These *R*^2^ values signify a correlation between pressure and the rate of yeast growth, as represented by the rate of change in impedance frequency (d*f*/d*t*). The 12% model was validated by evaluating its ability to predict pressure based on the rate of change in frequency, as shown in Fig. S5. This approach mirrors real-world application scenarios where the rate of change in frequency can be used to estimate the pressure of the actuator without needing a dedicated pressure sensor, thereby enabling control of the actuator’s motion. By combining these models with the yeast impedance equations (Eqs. 4 and (5)), we can elucidate the dynamics of pressure as actuation power in relation to yeast growth dynamics. This integration highlights the potential of the SCRO circuit as a sensor for the biohybrid system, capable of accurately monitoring and predicting yeast behavior and its influence on actuation power.

### Yeast-powered biohybrid soft robotics characteristics

#### Yeast-driven inflatable membrane actuator: Volume and force

**Fig. 5.**
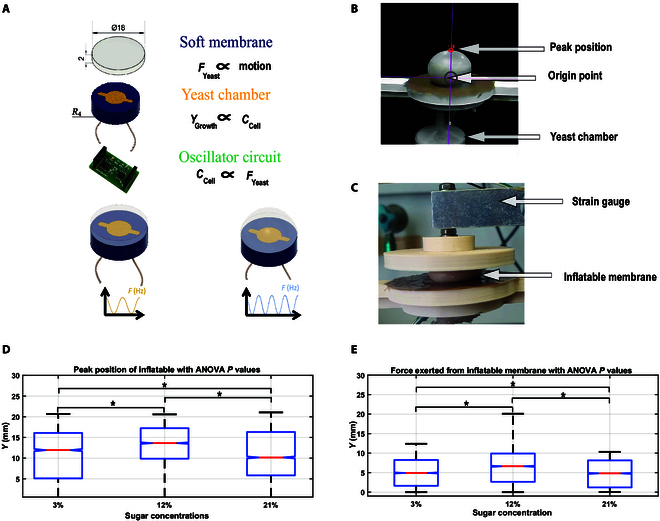
Soft robotic system: The inflatable membrane with yeast fermentation changes the capacitance of cell walls, which produces gas, altering the impedance frequency and resulting in the dynamic motion of the soft membrane. (A) The actuator design with an experimental setup was used to test yeast actuation power at different sugar concentrations. (B) The inflatable membrane actuator’s peak position changes due to pressure variations in the experimental setup. (C) The output force of the inflatable membrane actuator is exerted on the strain gauge fixed 5 mm above the actuator experimental setup. (D) Peak position of the inflatable membrane actuator under different sugar concentrations of yeast solution. (E) Force exerted by the inflatable membrane actuator under different sugar concentrations of yeast solution. * indicates a significant change between the 3 sugar concentrations using the analysis of variance (ANOVA) test (*P* value <0.05).

This experiment investigated actuation metrics in a simple soft actuator, specifically an inflatable membrane actuator from Jones and Damian [36]. This actuator, made from silicone rubber (Ecoflex 00-50), features an inner diameter of 8 mm and a membrane thickness of 1 mm. The inflatable membrane actuator replaced the actuator chamber from the previous pressure experiment and is connected to the yeast chamber, as shown in Fig. 5A. Two key experiments were conducted. The first experiment focused on measuring the peak position of the actuator across varying sugar concentrations (3%, 12%, and 21%) within the yeast solution under no-load conditions. The peak position was measured through recorded videos by a vision tracker system, as illustrated in Fig. 5B. The peak position was monitored using the Tracker video analysis and modeling tool software [37]. This peak position measurement reflects the actuator’s volume alteration in response to yeast gas effects, albeit considering the complexities arising from the actuator’s hyperelastic properties that impede straightforward volume measurements.

As illustrated in Fig. 5D, the experimental results of 3 trials demonstrate a direct correlation between increased sugar concentration and enhanced gas production, amplifying the actuator’s peak position from 3% to 12%. However, the peak position decreased from 12% to 21%, confirming the pressure experiment’s findings. The inflation dynamics of the actuator revealed an intriguing interplay between carbon dioxide and the yeast solution. While the actuator was primarily inflated by carbon dioxide, the process led to a foamlike mixture with the yeast solution. This phenomenon stemmed from the mechanics of the inflation process, where gas bubbles within the yeast solution attempted to traverse into the actuator from the yeast chamber.

The subsequent experiment measured the force exerted by the inflatable membrane actuator, specifically at its tip. This experiment was realized by firmly affixing the inflatable membrane actuator at 5 mm from the strain gauge (strain gauge load cell) to allow the actuator to inflate. The strain gauge served a dual purpose by measuring the force and functioning as a motion constraint for the actuator, as shown in Fig. 5C. This configuration ensured that the actuator’s displacement did not exceed the designated limit, thus maintaining the integrity of the experimental setup. The observed force reached a range of 10 N, and its rate of change demonstrated escalation with the increase in sugar concentration from 3% to 12%. Nonetheless, assessing the force under a 21% sugar concentration posed a challenge. This was attributed to the instability of the power generated at this concentration level. Notably, the force readings at 21% were lower than those at 12%, as shown in Fig. 5E. Consequently, this interaction resulted in a portion of the yeast solution finding its way inside the actuator. The fully inflated actuator showcased a distinct configuration, with gas entrapped over a foamlike liquid yeast layer. This unique observation accentuates the complexity of the yeast-based actuation system, where multiple factors, including gas diffusion and fluid dynamics, contribute to the overall behavior of the actuator.

#### Regeneration of actuation power in yeast-driven inflated membrane actuators

This experiment investigated the possibility of regenerating the actuation power without additional yeast or sugar. A peristaltic pump was integrated as a reverse system for the inflated membrane actuator from Fig. 5 to pump foam outside the actuator. This setup facilitated the eviction of gas and foam from the actuator after yeast growth. The pump was programmed to deflate the actuator for 1 s whenever the frequency rate of change deviated by 1 kHz, a threshold determined from the observations in the pressure experiment shown in Fig. 3. Meanwhile, the yeast inflated the membrane actuator for approximately 15 min. A 10-ml yeast mixture with a consistent 12% sugar concentration was employed throughout the experiment due to its demonstrated stability in previous experiments, showing reasonable pressure, volume, and force characteristics.

**Fig. 6.**
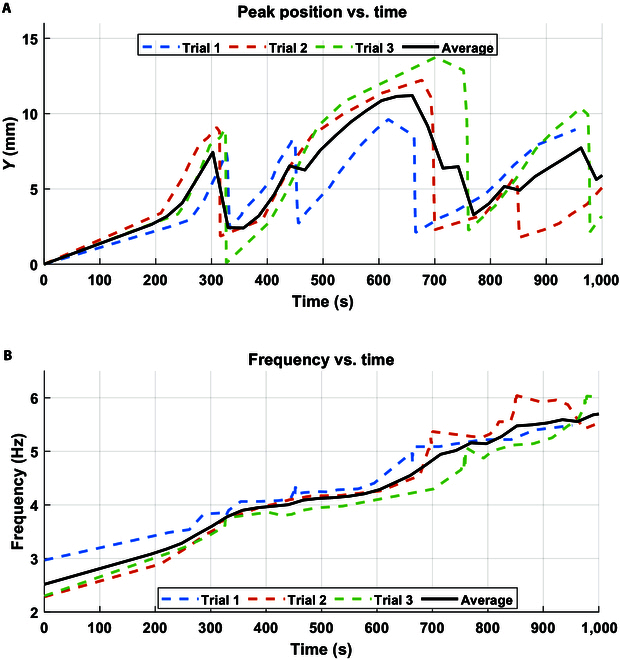
Assessing the regeneration of a 10-ml yeast solution with a 12% sugar concentration for actuating and peristaltic pump deactivating the inflated membrane actuator. (A) Cycles of the peak position of the inflatable membrane over time for 3 trials, averaging 3 cycles over approximately 16 min. (B) Impedance frequency readings of the yeast inside the chamber during the experiment.

Observations revealed that yeast could initiate inflation for approximately 3 cycles across 3 trials, as depicted in Fig. 6A with observing the frequency change over time shown in Fig. 6B. In the first cycle, inflation reached around 9 mm, while in the second cycle, inflation ranged between 12 and 14 mm, attributed to the lag phase of yeast growth. Notably, the actuator’s peak positions were more consistent across cycles in trial 1, allowing for more cycles than in trial 3, where higher peak positions in cycle 2 made it more difficult to maintain consistent actuation power. However, after these initial cycles, the pump effectively removed all yeast from the actuator, halting further inflation. Furthermore, each successive cycle gradually diminished the yeast’s ability to reinflate the actuator. This decline can be attributed to the peristaltic pump’s removal of yeast and the resulting decrease in yeast density, evidenced by a reduction in solution mass. Residual foam left from the pump’s operation contributed to this change in yeast density. Maintaining yeast-driven actuation over multiple cycles holds significant promise for advancing this biohybrid actuation mechanism.

## Results

**Fig. 7.**
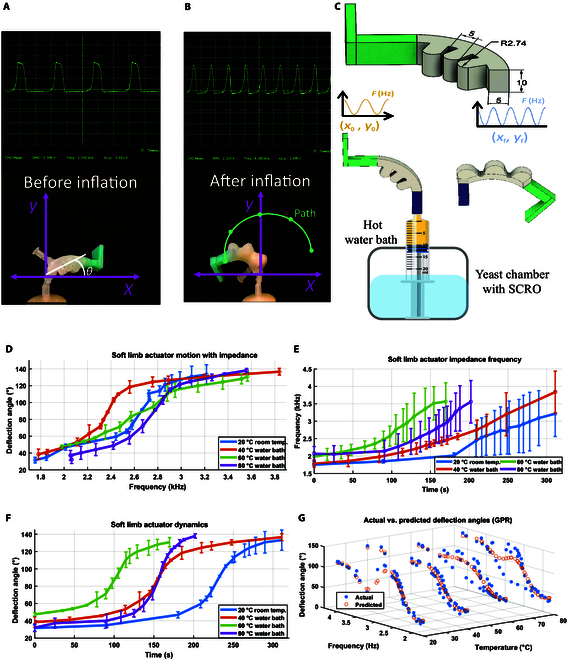
Soft robotic systems: soft limb actuator. (A) Soft limb actuator prior to inflation, showing the deflection angle (θ) and impedance frequency inside the yeast chamber. (B) Path of the soft limb actuator after inflation, highlighting the increase in impedance frequency. (C) Design of the soft limb actuator and experimental setup for measuring the actuator path at different temperatures using a water bath. (D) Deflection angle versus corresponding yeast frequency at various temperatures, with bars representing the standard deviation over 3 trials for each temperature. (E) Changes in frequency over time during limb motion. (F) Change in deflection over time. (G) Relationship between yeast temperature and the soft limb dynamics with yeast impedance using Gaussian process regression (GPR).

The practicality of utilizing yeast for actuation and sensing in real soft robotics systems was examined using a soft limb and an inflatable membrane actuator. The underlying concept aimed to correlate impedance frequency with the motion of these soft robotics systems and establish a relationship between yeast growth, as indicated by frequency, and robot motion. In both setups, experiments utilized a 12% sugar concentration yeast mixture. The first system comprises a soft limb designed with 3 air pillow geometries featuring curved oval shapes with a width of 5 mm each. These air pillows were arranged in a curved configuration to achieve approximately a 180° rotation, with the geometry selected based on the familiar shape of air pillows. The soft limbs are fabricated using Ecoflex 00-50 material, while the foot and holder parts are made of Mold Star 15 material to attach the limb to the yeast chamber shown in Fig. 7C. The second system consists of an inflatable membrane actuator with a yeast chamber made of Mold Star 30, which is a stiffer material to prevent chamber inflation, featuring an 8-mm diameter to accommodate a 2-mm membrane for actuation as shown in Fig. 5A. The yeast chamber in the inflated membrane actuator is equipped with 2 electrodes connected to the SCRO impedance sensor. The manufacture and molds of the 2 actuators are shown in Fig. S6.

### Yeast-powered biohybrid flexion of a soft limb

The yeast-based actuation was investigated in a soft flexion actuator under different temperatures. The work envelope, depicting the utmost extent of motion achievable by the actuator through yeast-powered actuation, is graphically illustrated in Fig. 7. This envelope, determined by utilizing the impedance frequency of yeast as an observation parameter for yeast growth rate as a proprioceptive sensor, offers insights into the actuator’s maximum coordinate points. Notably, the actuator demonstrates an impressive deformability angle range spanning from 20° to 140° in 5 min. The limb’s work envelope was monitored using the Tracker video analysis and modeling tool software [37] as shown in Fig. 7A and B.

The impact of varying medium temperatures on the actuator’s behavior was thoroughly investigated, as shown in Fig. 7D and F. The yeast actuator limb was mounted on a yeast chamber in hot water bath at temperatures of 40, 60, and 80 °C, compared to room temperature at 20 °C, illustrated in Fig. 7C. The alteration in temperature shows a substantial influence on the actuator’s performance. A noticeable trend emerged as the temperature increased from 20 to 40 to 60 °C, causing a significant acceleration in the actuator’s speed of motion, as shown in Fig. 7F, where it took 300 s to complete the path at 20 °C and only 150 s at 60 °C. However, the dynamics shifted when the actuator was subjected to an 80 °C environment, where it took 200 s to complete the path. In this case, the exceptionally high temperature had the opposite effect, slowing down the motion of the actuator. This deceleration can be attributed to the temperature’s adverse impact on yeast metabolism, impeding natural processes. The initial temperature-induced acceleration is likely due to the heightened metabolic activity of yeast in response to elevated thermal conditions, which can be sensed with impedance, as shown in Fig. 7D and E. These plots demonstrate how temperature variations impact yeast impedance, affecting the actuator’s performance, which is sensed by the bioimpedance SCRO circuit.

Complementing this analysis, the relationship between actuator dynamics and yeast temperature can be utilized to control robot motion in real-time applications by using yeast impedance as a sensor. To achieve this, we modeled the relationship between temperature (as a control signal) and actuator deflection angle, with impedance frequency serving as the sensing element, using Gaussian process regression, as shown in Fig. 7G. The model explains 83% of the deflection angle variance based on the temperature and frequency data, indicating a good fit and suggesting that the Gaussian process regression model effectively captures most of the relationship between these variables. The model’s validity was confirmed by predicting the actuator’s angles on untrained data, as shown in Fig. S7.

### Yeast as a tactile sensor

**Fig. 8.**
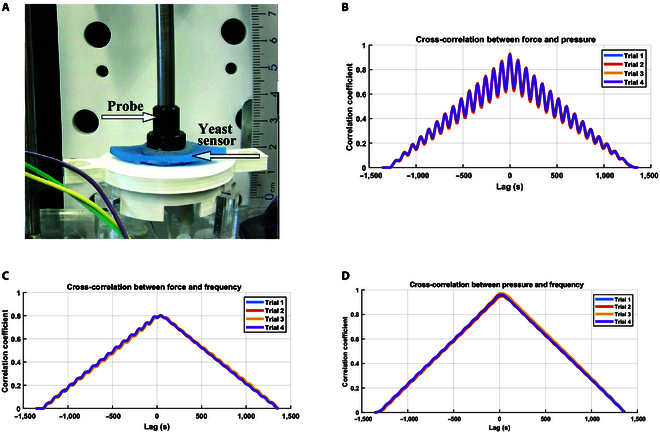
Tactile sensing using yeast inside the inflatable membrane. (A) Experiment setup for data acquisition, featuring yeast injected into the inflatable membrane actuator, equipped with SCRO electrodes, where a linear actuator with a strain gauge applies a sinusoidal force wave after the actuator’s inflation. (B) Cross-correlation between the input force sine signal and the output pressure across 4 trials shows a positive correlation at zero lag, while peaks indicate the time lag between the 2 signals. (C) Cross-correlation between the input force sine signal and yeast impedance frequency as a sensing variable across 4 trials, with a positive correlation at zero lag and peaks representing time lags. (D) Cross-correlation between the pressure sine signal and output frequency across 4 trials, demonstrating a positive correlation at zero lag.

In this experiment, we investigated the dynamic response of a yeast-inflated membrane actuator subjected to a sine-wave force. The inflated membrane actuator was filled with 3 ml of 12% sugar concentration yeast and consequently identified its sensing capability. The primary objective was to observe how the pressure and frequency of the actuator changed in response to the applied force. The experiment was conducted over 4 trials, each capturing time-varying force, pressure, and frequency data of the actuator. Each trial consisted of 15 cycles of sine-wave force application with a maximum force of 4.5 N, and data were recorded at uniform time intervals. The applied stimulus (sine-wave force) was identical across the 4 trials. However, biological variability in yeast activity and environmental conditions led to differences in the pressure and frequency responses. These variations are intrinsic to bio-driven systems and highlight the importance of conducting multiple trials to capture the system’s dynamic behavior. Key variables measured included the applied force, the resulting pressure within the actuator, and the frequency response of the actuator at equilibrium. The actuator reached an equilibrium phase when the pressure reached 0.1 bar, and the actuator inflated to approximately 0.5 cm. The experiment used a controlled linear actuator attached to a strain gauge to apply a controlled force signal over the inflated membrane actuator filled with yeast and connected to the SCRO circuit and pressure sensor, as shown in Fig. 8A. We used fast Fourier transform (FFT) analysis on each trial’s recorded force, pressure, and frequency signals. The FFT results revealed distinct peaks corresponding to the frequency of the applied sine-wave force and its harmonics, as shown in Fig. S8.

To investigate the frequency characteristics of the actuator response, we performed a cross-correlation analysis to examine the relationship between the applied force and the resulting pressure and frequency responses of the actuator. Cross-correlation measures the similarity between 2 signals as a function of the time lag between them, providing insights into the temporal relationship and phase shift between the signals. By calculating the cross-correlation between the applied force and the pressure, as well as between the applied force and the frequency, we aimed to understand the time delays and phase differences in the actuator response. The cross-correlation results revealed significant positive correlations between the applied force and the pressure response and between the applied force and the frequency response, with distinct peaks at specific time lags in Fig. 8. These peaks indicate the time delays at which the pressure and frequency responses are most similar to the applied force. These time lags suggest that the actuator’s response is not instantaneous but follows the applied force with a certain delay, probably due to the yeast cells’ dynamics and the SCRO delay.

**Fig. 9.**
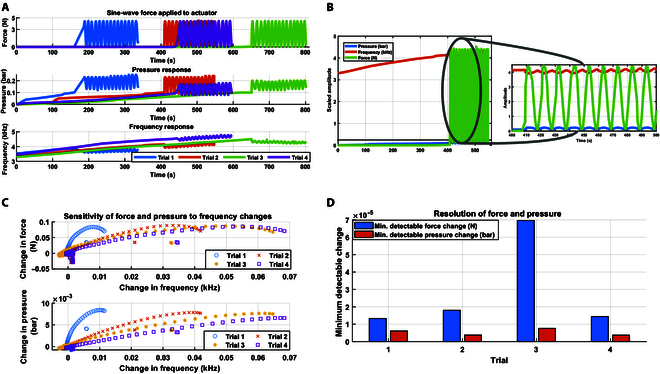
Effect of pressure change on yeast frequency due to external force. (A) Time-series data of the applied sine-wave force, pressure, and frequency response for 4 trials after the pressure inside the actuator reaches a specified value. (B) A comparison of the scale magnitude of the 3 signals from 1 trial illustrates the inverse relationship between force and pressure and the corresponding impedance frequency. (C) The sensitivity of force and pressure to changes in frequency. (D) Resolution: minimum detectable changes in force and pressure for frequency change for each trial.

In particular, pressure and force signals are directly proportional. At the same time, yeast frequency has an inverse relation with both pressure and force, decreasing as pressure increases due to changes in the arrangement of yeast cells. These relations for 4 trials are represented in Fig. 9A, and the comparison between the amplitude of the time series is shown in Fig. 9B for 1 trial. The plot presented in Fig. 9C reveals the sensitivity of force and pressure to changes in frequency as indicated by the slope of the curves. The initial changes in frequency do not correspond to changes in force and show only a slight change in pressure, representing the equilibrium phase where the membrane is allowed to deflect. The first trial displays the shortest curve due to higher noise levels than the other 3 trials. The plot demonstrates that force and pressure exhibit variable sensitivity across different frequencies. The minimum detectable changes in force and pressure are illustrated in Fig. 9D. The resolution of the sensor system demonstrates the SCRO circuit’s capability to detect variations in the actuator’s environment. We calculate the resolution in force and pressure by determining the smallest nonzero difference in these 2 parameters, resulting in a measurable change in frequency. Notably, the maximum resolution for force was 7 × 10^−5^ N, while for pressure, it was 9 × 10^−6^ bar. The data for each trial are filtered using a Butterworth filter and then smoothed using a moving average. The comparative analysis of sensitivity and resolution provides valuable insights into how effectively frequency variations can be used to sense changes in force and pressure.

Despite the inverse relationship between pressure and frequency, the cross-correlation may yield positive values as shown in Fig. 8D. This can occur due to shared dynamics as both pressure and frequency respond to the same input, which is force, leading to the same temporal patterns, even if their magnitude moves in opposite directions. In addition, the positive cross-correlation indicates that while pressure and frequency may exhibit an inverse relationship with respect to their values, their temporal patterns of change are aligned in specific contexts. These findings highlight the potential of using yeast bioimpedance as an exteroceptive sensor to detect external changes in yeast-driven robots. The yeast-driven inflatable membrane’s ability to detect changes in volume and pressure through bioimpedance frequency allows the robot to sense when it has encountered an obstacle, facilitating effective control and avoidance.

### Yeast-driven systems for tissue palpation and gripping applications

**Fig. 10.**
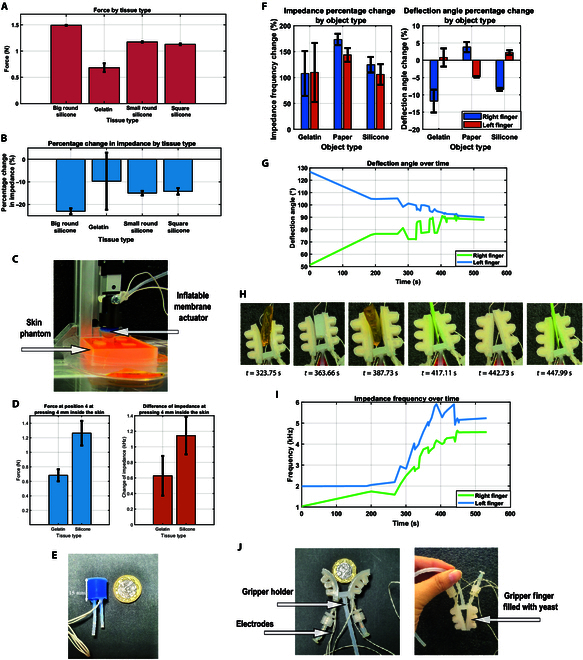
Using the yeast-driven probe and gripper. (A) Mean applied force across different tissue types during palpation. Error bars represent the standard deviation of measurements. (B) Percentage change in impedance across different tissue types during palpation. Error bars represent the standard deviation of measurements. (C) The experiment setup shows an inflated membrane pressing on the skin. (D) The difference between gelatin and silicone force and impedance at pressing. Error bars represent the standard deviation of measurements. (E) Yeast-driven probe size. (F) Percentage change in deflection angle and its effect on impedance change in a yeast-driven gripper system gripping different objects. (G) Path profile of the gripper fingers highlighting gripping phases as peaks in the deflection angle. (H) Time-stamped photos of the yeast-driven gripper holding objects, reflected in changes in impedance shown in (H) and deflection angles during gripping phases in (G). (I) Impedance changes correspond to the gripping phases for various objects (gelatin, silicone, and paper). (J) Comparison of the yeast-driven gripper to a 1-pound coin and fully closed gripper filled with yeast.

This section presents yeast-driven systems’ capabilities in biohybrid robotics, focusing on tissue palpation and object grasping. Using yeast fermentation for actuation and sensing, the experiments show how these systems can detect differences in tissue stiffness and manage objects of varying properties, illustrating their practical potential for soft robotics and biomedical applications. In this experiment, we investigated the capability of a yeast-driven inflatable membrane actuator to differentiate between tissue types by performing a palpation task. We used the previously tested inflated membrane actuator. We increased the yeast chamber to 15 mm, allowing it to be filled with approximately 2.5 ml of 12% yeast concentration, as shown in Fig. 10E, to create a yeast-driven probe. To simulate differences in tissue stiffness, a skin phantom was created using 5% concentration gelatin, representing healthy tissue, and silicone rubber (Ecoflex 00-50), representing cancerous tissue [38,39]. The actuator was filled with yeast and allowed to ferment for 13 min until the internal pressure stabilized at 0.12 bar. This pressure was maintained consistently throughout the experiment, optimizing the actuator’s sensitivity to variations in tissue stiffness.

The experimental protocol began with inflating the yeast-driven actuator until it reached a stable internal pressure of 0.12 bar. A linear actuator was then used to move the inflated membrane toward the skin phantom surface until a change in load was detected, indicating contact with the phantom. Subsequently, the actuator was displaced 4 mm into the phantom to simulate tissue palpation, ensuring consistent penetration across materials for comparison. For 3 cycles, the probe was pressed 4 mm into the phantom, which was less than the inflated membrane height. At the beginning of the experiment, the internal pressure of the actuator was consistently maintained at 0.12 bar. This process was repeated until the actuator’s internal pressure increased from 0.12 to 0.13 bar due to active yeast fermentation. During the repeated measurements across different materials within the phantom, the gradual pressure increase from 0.12 to 0.13 bar did not significantly affect the sensitivity of the actuator. However, this pressure change is crucial for maintaining the yeast fermentation-driven probe and ensuring consistent system performance throughout the experiment.

The key parameters measured during the experiment included the force exerted on the tissue phantom and the corresponding percentage change in impedance, which served as a sensing metric, as shown in Fig. 10. The results demonstrated a clear distinction between the tissue types: gelatin exhibited a minor percentage change in impedance compared to silicone, reflecting the differences in stiffness between the 2 materials. For example, the mean applied force during palpation was higher for silicone tissues compared to gelatin, as shown in Fig. 10A, with gelatin requiring lower force due to its softer composition. Similarly, the percentage change in impedance was more pronounced in silicone tissues, demonstrating the probe’s sensitivity to stiffness differences, as illustrated in Fig. 10B. The comparison of force and impedance measurements between gelatin and silicone tissues during pressing further emphasized the actuator’s ability to distinguish between the 2, as shown in Fig. 10D. These findings highlight the effectiveness of the yeast-driven actuator in detecting and differentiating tissue stiffness variations. The experiment setup, depicted in Fig. 10C, shows the inflated membrane actuator pressing on the skin phantom.

Second, we designed the yeast-driven gripper system using the soft limb actuator as gripper fingers. This system demonstrated variations in deflection angles and changes in impedance during object manipulation. The gripper fingers filled with approximately 1.5 ml of 12% yeast mixture, as shown in Fig. 10J, were tested by gripping objects made of gelatin and silicone with identical weight and thickness, as illustrated in Fig. S9. The gripper closed fully and grabbed a piece of paper after around 10 min of yeast-driven actuation Fig. 10J. The analysis revealed distinct gripping phases, marked by peaks in the deflection angle, as shown in the path profile of the gripper fingers in Fig. 10G. These peaks correspond to mechanical transitions during object engagement, highlighting the gripper’s sensitivity to grasping. Simultaneously, changes in impedance during gripping, as shown in Fig. 10I, provide insights into the gripper’s response to deflection changes and its adaptability to the physical properties of the objects being handled. Time-stamped photos of the gripper holding objects in Fig. 10H link these behavioral changes to specific operation phases, offering a synchronized view of impedance dynamics, deflection angle changes, and mechanical performance.

To evaluate the accuracy and response time of the driving system, the mean rate of change for the deflection angle was calculated as the derivative of the deflection angle with respect to time. The results indicate that the right finger exhibited a faster rate of change (0.51136/s). In comparison, the left finger demonstrated a slower rate of change (−0.15096/s), suggesting different mechanical responses and yeast fermentation between the 2 fingers. Similarly, the mean rate of impedance change, determined as the derivative of impedance over time, was higher for the right finger (0.019 Hz/s) than for the left finger (0.01212 Hz/s), reflecting faster impedance dynamics in the right finger. These metrics were derived from the time-series data of the gripper’s deflection angles and impedance, with baseline values established during the “none” state, where the gripper was not holding any object.

This comprehensive analysis highlights the dynamic behavior of the yeast-driven gripper, showcasing its responsiveness and mechanical sensitivity during object manipulation. While the response speed is relatively slow, the system’s response enables it to sense and grasp various objects. These findings underscore the potential of yeast-driven grippers for advanced biohybrid robotic applications requiring careful handling and high sensitivity.

## Discussion

This study explored the feasibility of using yeast as an actuation and sensing mechanism in soft robotics. We investigated the potential of yeast fermentation to generate mechanical power through pressure, which can be sensed using yeast bioimpedance. Through experiments with inflated membrane actuators and soft limb designs, we demonstrated the ability of yeast-driven systems to function as actuators, proprioceptive sensors, and exteroceptive sensors. Our results indicate that yeast fermentation and bioimpedance technology can effectively power and monitor microbial biohybrid robotic systems. Additionally, our research highlights the development of a circuit capable of measuring impedance in biological matter and adjusting by using a digital potentiometer, emphasizing its sensitivity to variations in yeast concentration.

First, the study investigated the potential of yeast as a source of mechanical power and the ability of a bioimpedance circuit to detect pressure changes. The experiments demonstrated that yeast can generate sufficient pressure for actuation, with pressure levels directly correlating with yeast impedance at a fixed volume. These findings underscore the potential for dynamic, real-time tracking of yeast-driven actuation, enabling timely adjustments for optimized fermentation performance. By integrating yeast dynamics equations with SCRO circuit equations, we successfully modeled yeast growth rate and actuation power, paving the way for enhanced sensing and actuation capabilities in biohybrid systems. Additionally, we observed a direct relationship between sugar concentration, actuator peak position, and the exerted force of the soft inflatable membrane actuator, indicating the influence of fermentation dynamics on actuator behavior. Furthermore, integrating a peristaltic pump allowed for the regeneration of actuation power and showed the potential for sustained yeast-driven actuation over multiple cycles. Although we tested the effects of temperature and sugar concentration, humidity was not examined due to the closed nature of the yeast chamber, which maintains anaerobic fermentation conditions where humidity is not a significant factor. The yeast-driven system exhibits a slower response speed than traditional actuation methods due to the gradual nature of fermentation. However, this characteristic makes it ideal for applications requiring adaptive and energy-efficient responses, such as environmental monitoring and biomedical systems. For example, it can provide long-term sensing of parameters like pressure, temperature, or gas composition in dynamic environments or enable controlled, gentle movements for handling delicate objects like biological tissue in soft robotics. In medical applications, it shows potential for adaptive prosthetics, tactile sensing for soft tissue evaluation, implants, and biosensing platforms to monitor body conditions over time. Additionally, yeast offers advantages like biodegradability and untethered operation, eliminating the need for external valves and pumps. At the same time, its dual functionality as both an actuator and a sensor further enhances its versatility in robotic systems.

This paper explores using 2 types of soft actuators—a soft limb and an inflatable membrane—to test the capability of yeast to actuate robots while sensing both internal and external states using bioimpedance. The experiments with soft limb designs revealed a complex interaction between yeast fermentation and robot motion, particularly regarding deflection angle under varying temperatures. By correlating impedance frequency with robot movement, we established a method for monitoring yeast dynamics and predicting actuator behavior as a proprioceptive sensor. The results indicate that temperature variations significantly affect the acceleration and deceleration of limb motion, suggesting that temperature can effectively control yeast actuation. Our findings also demonstrate that yeast impedance frequency is a reliable indicator of yeast state, providing valuable insights into actuator performance and dynamics. In our experiments involving an inflatable membrane actuator without a separate yeast chamber, we gained insights into the practical application of yeast-driven actuation by incorporating yeast directly into the actuator. The inflatable membrane actuator effectively responded to external forces, with noticeable changes in pressure and frequency. Cross-correlation analysis showed significant positive correlations between applied force, pressure, and frequency responses. While pressure and force were directly proportional, frequency showed an inverse relationship with both. FFT analysis confirmed distinct peaks corresponding to the applied force frequency and its harmonics. These findings underscore the potential of using yeast bioimpedance as an exteroceptive sensor to detect external changes in biohybrid robotic systems.

We demonstrated the potential of yeast-driven biohybrid robots through 2 applications: a tissue stiffness probe and a gripper. These demonstrations highlight the sensitivity and capability of yeast fermentation for actuation and sensing, particularly in handling delicate tasks. Despite a relatively slower response speed than conventional methods, the inherent sensitivity and dual functionality of yeast-driven systems underscore their potential for soft robotics and biomedical applications demanding delicate handling and precise sensing capabilities. If miniaturized, such systems could be effectively applied in biomedical contexts, including tissue evaluation and precise object manipulation, with the advantage that yeast-driven systems could potentially be ingestible and biodegradable.

Understanding the factors that influence yeast-driven actuation is crucial for optimizing performance. One key factor is the design and configuration of the electrodes used for bioimpedance sensing. We observed that the length and material of the electrodes and the amount of yeast mixture affected the reading frequency. Adding a digital potentiometer to the SCRO circuit to address these variations proved effective. More significant amounts of yeast mixture need a higher value of the digital potentiometer, requiring adjustments in circuit parameters to maintain oscillation frequency. Conversely, smaller amounts necessitate changes to achieve optimal circuit performance. Additionally, the distance between electrodes is crucial for accurately capturing yeast dynamics, influencing the sensitivity and resolution of the sensing system; therefore, we fixed the electrodes in the same position during experiments. Our findings also revealed that yeast actuation is driven by carbon dioxide production and the flow of yeast foam into the actuator chamber. This foam formation, resulting from gas bubbles within the yeast solution interacting with the actuator’s membrane, contributes to the actuator’s inflation and movement.

While yeast-driven actuation demonstrates significant potential, several challenges and limitations must be addressed to optimize its use. For example, the high growth rate of yeast can cause rapid fluctuations in frequency readings and actuator performance, complicating precise control and long-term operation of yeast-driven robots. One critical area for future research is the deactivation process of yeast-driven actuation. Although our study used a peristaltic pump for deactivation, developing a more efficient system without electronics is possible. Introducing a self-release valve made from soft materials could facilitate controlled deflation of the actuator, eliminating the need for electronic components. This innovation could improve the efficiency of yeast-driven robotic systems.

Yeast-driven biohybrid systems offer unique advantages, including scalability, cost-effectiveness, and straightforward handling, making them well-suited for in-body and external applications. The dual functionality of these systems, as both an actuator and a sensor, along with their biodegradability, highlights their potential as versatile options in biohybrid robotics. Unlike cell-driven systems, which provide faster and more precise actuation at a higher cost and complexity and are needed to work in lab medium, or bacteria-driven systems, which excel in microscale applications but lack scalability and require extensive genetic modification to ensure safety in biomedical applications, yeast systems simplify design and have minimal operational demands, enabling them to work both inside the body and outside the laboratory. This comparison underscores the complementary roles of yeast-driven systems in expanding the capabilities of biohybrid robotics (see Table 3).

**Table 3.** Comparison of biohybrid robot systems

Feature	Yeast-driven	Cell-driven [17]	Bacteria-driven [11]
Driving mechanism	CO_2_ production via fermentation	Contraction of muscle cells	Flagellar motion or metabolic activity
Control difficulty	Moderate (temperature, sugar regulation)	High (requires precise stimulus control)	Moderate (chemical or light stimuli)
Scalability	High (low-cost and easy to culture)	Low (complex cell culture requirements)	Moderate (scalable but contamination risk)
Response speed	Slow	Moderate to fast	Fast
Environment	Actuation, sensing, environmental tasks, biomedical uses inside the body	High-precision biohybrid robotics	Microrobots, therapeutic delivery
Ability to operate	Works inside the body and outside the lab	Typically limited to controlled lab environments	Works outside the lab and requires extensive genetic modification to ensure safety in biomedical application
Limitations	Slow response speed	High cost, complex control	Limited force generation, contamination risk

In conclusion, yeast-driven actuators hold immense potential for powering and controlling soft robotics systems. This study investigates the feasibility of using yeast for actuation and sensing in soft robotics, demonstrating that yeast fermentation can generate mechanical power and be sensed through bioimpedance. We used yeast-driven systems as actuators and sensors, showing their potential in proprioceptive and exteroceptive applications. Experiments with inflated membrane actuators and soft limbs revealed that yeast-generated pressure correlates with impedance changes, while temperature variations influence actuator performance. Additionally, incorporating yeast directly into the actuator and using bioimpedance for real-time monitoring highlighted the effectiveness of this approach for detecting external changes and optimizing robot control. Our findings pave the way for enhanced biohybrid systems with integrated sensing and actuation capabilities.

## Data Availability

All data are available from the corresponding authors upon reasonable request.
