# Supplementary material for: Yeast-Driven and Bioimpedance-Sensitive Biohybrid Soft Robots
Source: Cyborg Bionic Syst. 2025 Apr 25;6:0233. doi: 10.34133/cbsystems.0233 (PMC12022396; doi:10.34133/cbsystems.0233)
Supplement: Supplementary 1 — Figs. S1 to S9 Data File S1 Movies S1 and S2 [file cbsystems.0233.f1.zip › Data file S1..pdf]

## Exp1_Pressure vs frequency for anaerobic fermentation:

| 3% | | | | | | | | | | | | | | | | | | | |
|---|---|---|---|---|---|---|---|---|---|---|---|---|---|---|---|---|---|---|---|
| T1 | | | | T2 | | | | T3 | | | | T4 | | | | T5 | | | |
| P ( bar) | F (kHz) | t(s) | df_dt | P ( bar) | F (kHz) | t(s) | df_dt | P ( bar) | F (kHz) | t(s) | df_dt | P ( bar) | F (kHz) | t(s) | df_dt | P ( bar) | F (kHz) | t(s) | df_dt |
| 0.0000 | 0.8790 | 0.0000 | 0.0033 | 0.0000 | 1.6670 | 0.0000 | 0.0006 | 0.0000 | 1.0800 | 0.0000 | 0.0017 | 0.0000 | 0.8590 | 0.0000 | 0.0010 | 0.0000 | 1.0800 | 0.0000 | 0.0017 |
| 0.0200 | 0.8850 | 1.8000 | 0.0032 | 0.0200 | 1.6810 | 22.3167 | -0.0009 | 0.0200 | 1.0940 | 8.4667 | 0.0018 | 0.0200 | 0.8810 | 23.1167 | 0.0014 | 0.0200 | 1.0940 | 8.4667 | 0.0049 |
| 0.0300 | 0.9160 | 11.3500 | 0.0036 | 0.0300 | 1.6640 | 41.0333 | 0.0156 | 0.0300 | 1.1420 | 35.7833 | 0.0033 | 0.0300 | 0.9340 | 61.4667 | 0.0026 | 0.0300 | 1.2280 | 35.7833 | 0.0016 |
| 0.0400 | 1.0190 | 39.7167 | 0.0047 | 0.0400 | 1.9650 | 60.3333 | 0.0139 | 0.0400 | 1.2550 | 69.5667 | 0.0046 | 0.0400 | 1.0040 | 87.9667 | 0.0027 | 0.0400 | 1.2810 | 69.5667 | 0.0020 |
| 0.0500 | 1.1370 | 65.0000 | 0.0054 | 0.0500 | 2.2090 | 77.8333 | 0.0112 | 0.0500 | 1.3600 | 92.5333 | 0.0052 | 0.0500 | 1.0370 | 100.2000 | 0.0027 | 0.0500 | 1.3260 | 92.5333 | 0.0046 |
| 0.0600 | 1.2340 | 83.0500 | 0.0059 | 0.0600 | 2.3440 | 89.9000 | 0.0058 | 0.0600 | 1.4560 | 110.9000 | 0.0056 | 0.0600 | 1.0670 | 111.2500 | 0.0042 | 0.0600 | 1.4110 | 110.9000 | 0.0091 |
| 0.0700 | 1.2980 | 93.8833 | 0.0060 | 0.0700 | 2.4310 | 104.9833 | 0.0148 | 0.0700 | 1.5150 | 121.4000 | 0.0059 | 0.0700 | 1.1380 | 128.0833 | 0.0038 | 0.0700 | 1.5070 | 121.4000 | 0.0055 |
| 0.0800 | 1.3270 | 98.7000 | 0.0060 | 0.0800 | 2.5290 | 111.6167 | 0.0044 | 0.0800 | 1.5730 | 131.2500 | 0.0059 | 0.0800 | 1.2150 | 148.1000 | 0.0028 | 0.0800 | 1.5610 | 131.2500 | 0.0074 |
| 0.0900 | 1.3700 | 105.9167 | 0.0075 | 0.0900 | 2.5820 | 123.6833 | 0.0151 | 0.0900 | 1.6470 | 143.7167 | 0.0061 | 0.0900 | 1.2260 | 152.0000 | 0.0035 | 0.0900 | 1.6530 | 143.7167 | 0.0052 |
| 0.1000 | 1.3970 | 109.5333 | 0.0064 | 0.1000 | 2.6820 | 130.3167 | 0.0104 | 0.1000 | 1.6990 | 152.2500 | 0.0062 | 0.1000 | 1.2640 | 163.0000 | 0.0056 | 0.1000 | 1.6970 | 152.2500 | 0.0044 |
| 0.1100 | 1.4590 | 119.1500 | 0.0070 | 0.1100 | 2.7760 | 139.3667 | 0.0081 | 0.1100 | 1.7400 | 158.8167 | 0.0052 | 0.1100 | 1.3830 | 184.3333 | 0.0054 | 0.1100 | 1.7260 | 158.8167 | 0.0063 |
| 0.1200 | 1.5260 | 128.7833 | 0.0074 | 0.1200 | 2.8490 | 148.4167 | 0.0098 | 0.1200 | 1.7700 | 164.5667 | 0.0082 | 0.1200 | 1.4110 | 189.5500 | 0.0049 | 0.1200 | 1.7620 | 164.5667 | 0.0105 |
| 0.1300 | 1.6550 | 146.2333 | 0.0070 | 0.1300 | 2.9260 | 156.2667 | 0.0120 | 0.1300 | 1.7980 | 168.0000 | 0.0065 | 0.1300 | 1.4650 | 200.6167 | 0.0053 | 0.1300 | 1.7980 | 168.0000 | 0.0059 |
| 0.1400 | 1.6970 | 152.2500 | 0.0083 | 0.1400 | 3.0050 | 162.8333 | 0.0040 | 0.1400 | 1.8610 | 177.7667 | 0.0064 | 0.1400 | 1.5030 | 207.7833 | 0.0041 | 0.1400 | 1.8560 | 177.7667 | 0.0079 |
| 0.1500 | 1.7110 | 153.9333 | 0.0077 | 0.1500 | 3.0220 | 167.0833 | 0.0084 | 0.1500 | 1.9240 | 187.6833 | 0.0062 | 0.1500 | 1.5380 | 216.2500 | 0.0055 | 0.1500 | 1.9340 | 187.6833 | 0.0054 |
| 0.1600 | 1.7910 | 164.3000 | 0.0073 | 0.1600 | 3.0880 | 174.9833 | 0.0129 | 0.1600 | 1.9730 | 195.6167 | 0.0059 | 0.1600 | 1.5740 | 222.7667 | 0.0053 | 0.1600 | 1.9770 | 195.6167 | 0.0047 |
| 0.1700 | 1.8750 | 175.7333 | 0.0066 | 0.1700 | 3.1820 | 182.2833 | -0.0010 | 0.1700 | 2.0080 | 201.5667 | 0.0063 | 0.1700 | 1.6050 | 228.6333 | 0.0036 | 0.1700 | 2.0050 | 201.5667 | 0.0086 |
| 0.1800 | 1.9270 | 183.6333 | 0.0103 | 0.1800 | 3.1770 | 187.1333 | -0.0274 | 0.1800 | 2.0330 | 205.5333 | 0.0058 | 0.1800 | 1.6120 | 230.5833 | 0.0049 | 0.1800 | 2.0390 | 205.5333 | 0.0055 |
| 0.1900 | 1.9520 | 186.0667 | 0.0062 | 0.1900 | 3.0940 | 190.1667 | 0.0008 | 0.1900 | 2.0560 | 209.5000 | 0.0056 | 0.1900 | 1.6440 | 237.1000 | 0.0046 | 0.1900 | 2.0610 | 209.5000 | 0.0036 |
| 0.2000 | 1.9860 | 191.5167 | 0.0068 | 0.2000 | 3.1000 | 198.0667 | 0.0240 | 0.2000 | 2.1150 | 220.0667 | 0.0054 | 0.2000 | 1.7040 | 250.1333 | 0.0047 | 0.2000 | 2.0990 | 220.0667 | 0.0079 |
| 0.2100 | 2.0190 | 196.3667 | 0.0070 | 0.2100 | 3.4360 | 212.0500 | 0.0129 | 0.2100 | 2.1650 | 229.3333 | 0.0055 | 0.2100 | 1.7530 | 260.5500 | 0.0048 | 0.2100 | 2.1720 | 229.3333 | 0.0019 |
| 0.2200 | 2.0740 | 204.2333 | 0.0068 | 0.2200 | 3.5540 | 221.1833 | -0.0201 | 0.2200 | 2.2050 | 236.6000 | 0.0058 | 0.2200 | 1.7780 | 265.7667 | 0.0032 | 0.2200 | 2.1860 | 236.6000 | 0.0078 |
| 0.2300 | 2.1360 | 213.3333 | 0.0063 | 0.2300 | 3.4690 | 225.4167 | 0.0294 | 0.2300 | 2.2470 | 243.8667 | 0.0054 | 0.2300 | 1.7990 | 272.3000 | 0.0106 | 0.2300 | 2.2430 | 243.8667 | 0.0056 |
| 0.2400 | 2.1780 | 220.0000 | 0.0061 | 0.2400 | 3.5940 | 229.6667 | 0.0142 | 0.2400 | 2.2720 | 248.4833 | 0.0048 | 0.2400 | 1.8680 | 278.7833 | 0.0101 | 0.2400 | 2.2690 | 248.4833 | 0.0069 |
| 0.2500 | 2.2150 | 226.0500 | 0.0055 | 0.2500 | 3.6460 | 233.3167 | 0.0167 | 0.2500 | 2.2880 | 251.8000 | 0.0055 | 0.2500 | 1.9140 | 283.3500 | 0.0127 | 0.2500 | 2.2920 | 251.8000 | 0.0030 |
| 0.2600 | 2.2450 | 231.5167 | 0.0066 | 0.2600 | 3.7780 | 241.2333 | 0.0109 | 0.2600 | 2.3350 | 260.3833 | 0.0051 | 0.2600 | 2.0380 | 293.1167 | 0.0121 | 0.2600 | 2.3180 | 260.3833 | 0.0063 |
| 0.2700 | 2.2690 | 235.1500 | 0.0054 | 0.2700 | 3.9040 | 252.7500 | -1.1377 | 0.2700 | 2.3860 | 270.3000 | 0.0053 | 0.2700 | 2.1480 | 302.2333 | 0.0064 | 0.2700 | 2.3800 | 270.3000 | 0.0125 |
| 0.2800 | 2.3150 | 243.6333 | 0.0059 | 0.2800 | 3.8850 | 252.7667 | 0.0027 | 0.2800 | 2.4000 | 272.9500 | 0.0049 | 0.2800 | 2.1900 | 308.7500 | 0.0006 | 0.2800 | 2.4130 | 272.9500 | 0.0023 |
| 0.2900 | 2.4520 | 266.6667 | 0.0099 | 0.2900 | 3.9150 | 263.7000 | 0.0367 | 0.2900 | 2.4520 | 283.5167 | 0.0061 | 0.2900 | 2.1950 | 316.5667 | 0.0123 | 0.2900 | 2.4370 | 283.5167 | 0.0251 |
| 0.3000 | 2.4760 | 269.0833 | 0.0119 | 0.3000 | 4.0490 | 267.3500 | 0.0135 | 0.3000 | 2.4600 | 284.8333 | 0.0047 | 0.3000 | 2.2910 | 324.3833 | 0.0110 | 0.3000 | 2.4700 | 284.8333 | 0.0021 |
| 0.3100 | 2.5770 | 277.5667 | 0.0129 | 0.3100 | 4.1390 | 274.0333 | 0.0069 | 0.3100 | 2.5040 | 294.1000 | 0.0045 | 0.3100 | 2.3340 | 328.3000 | 0.0100 | 0.3100 | 2.4890 | 294.1000 | 0.0042 |
| 0.3200 | 2.6790 | 285.4500 | 0.0109 | 0.3200 | 4.2390 | 288.6000 | 0.0094 | 0.3200 | 2.5520 | 304.6667 | 0.0044 | 0.3200 | 2.3990 | 334.8167 | 0.0130 | 0.3200 | 2.5330 | 304.6667 | 0.0065 |
| 0.3300 | 2.7320 | 290.3000 | 0.0106 | 0.3300 | 4.3020 | 295.2833 | 0.0047 | 0.3300 | 2.5810 | 311.2667 | 0.0078 | 0.3300 | 2.5770 | 348.4833 | 0.0127 | 0.3300 | 2.5760 | 311.2667 | 0.0049 |
| 0.3400 | 2.8030 | 296.9667 | 0.0108 | 0.3400 | 4.3250 | 300.1500 | 0.0119 | 0.3400 | 2.6480 | 319.8667 | 0.0104 | 0.3400 | 2.6430 | 353.7000 | 0.0129 | 0.3400 | 2.6180 | 319.8667 | 0.0109 |
| 0.3500 | 2.8950 | 305.4500 | 0.0113 | 0.3500 | 4.3900 | 305.6167 | 0.0160 | 0.3500 | 2.7100 | 325.8333 | 0.0091 | 0.3500 | 2.7020 | 358.2667 | 0.0112 | 0.3500 | 2.6830 | 325.8333 | 0.0086 |
| 0.3600 | 2.9430 | 309.7000 | 0.0099 | 0.3600 | 4.4970 | 312.3000 | 0.0194 | 0.3600 | 2.7640 | 331.7667 | 0.0106 | 0.3600 | 2.8190 | 368.6833 | 0.0120 | 0.3600 | 2.7340 | 331.7667 | 0.0185 |
| 0.3700 | 2.9790 | 313.3333 | 0.0123 | 0.3700 | 4.6380 | 319.5833 | 0.0242 | 0.3700 | 2.7990 | 335.0667 | -0.0186 | 0.3700 | 2.9590 | 380.3833 | 0.0121 | 0.3700 | 2.7950 | 335.0667 | 0.0074 |
| 0.3800 | 3.0910 | 322.4167 | 0.0112 | 0.3800 | 4.9870 | 334.0333 | 0.0082 | 0.3800 | 2.6640 | 342.3333 | 0.0238 | 0.3800 | 3.0850 | 390.8333 | 0.0102 | 0.3800 | 2.8490 | 342.3333 | 0.0087 |
| 0.3900 | 3.2330 | 335.1500 | 0.0111 | 0.3900 | 5.0130 | 337.2167 | 0.0060 | 0.3900 | 3.0110 | 356.8833 | 0.0101 | 0.3900 | 3.1380 | 396.0333 | 0.0123 | 0.3900 | 2.9750 | 356.8833 | 0.0121 |
| 0.4000 | 3.3140 | 342.4167 | 0.0117 | 0.4000 | 5.0600 | 345.1000 | 0.0203 | 0.4000 | 3.0710 | 362.8167 | 0.0091 | 0.4000 | 3.2660 | 406.4667 | 0.0128 | 0.4000 | 3.0470 | 362.8167 | 0.0154 |
| 0.4100 | 3.4430 | 353.4667 | 0.0094 | 0.4100 | 5.2450 | 354.2333 | 0.0035 | 0.4100 | 3.1310 | 369.4333 | 0.0100 | 0.4100 | 3.2910 | 408.4167 | 0.0127 | 0.4100 | 3.1490 | 369.4333 | 0.0044 |
| 0.4200 | 3.4890 | 358.3833 | 0.0124 | 0.4200 | 5.2660 | 360.3000 | 0.0037 | 0.4200 | 3.2430 | 380.6667 | 0.0103 | 0.4200 | 3.3570 | 413.6333 | 0.0123 | 0.4200 | 3.1980 | 380.6667 | 0.0055 |
| 0.4300 | 3.5380 | 362.3333 | 0.0108 | 0.4300 | 5.2840 | 365.1667 | 0.0243 | 0.4300 | 3.3040 | 386.6167 | 0.0102 | 0.4300 | 3.5330 | 427.9500 | 0.0125 | 0.4300 | 3.2310 | 386.6167 | 0.0045 |
| 0.4400 | 3.6980 | 377.1000 | 0.0290 | 0.4400 | 5.6540 | 380.3667 | 0.0010 | 0.4400 | 3.4990 | 405.7833 | 0.0118 | 0.4400 | 3.7290 | 443.5833 | 0.0135 | 0.4400 | 3.3170 | 405.7833 | 0.0455 |
| 0.4500 | 3.7550 | 379.0667 | 0.0071 | 0.4500 | 5.6650 | 391.9167 | 0.0266 | 0.4500 | 3.5380 | 409.0833 | 0.0095 | 0.4500 | 3.8610 | 453.3667 | 0.0108 | 0.4500 | 3.4670 | 409.0833 | 0.0206 |
| 0.4600 | 3.7830 | 383.0000 | 0.0229 | 0.4600 | 6.0370 | 405.8833 | -0.0044 | 0.4600 | 3.5880 | 414.3667 | 0.0088 | 0.4600 | 3.9730 | 463.7833 | 0.0110 | 0.4600 | 3.5760 | 414.3667 | 0.0063 |
| 0.4700 | 3.8510 | 385.9667 | 0.0132 | 0.4700 | 6.0210 | 409.5167 | 0.0184 | 0.4700 | 3.6870 | 425.6000 | 0.0082 | 0.4700 | 4.0520 | 470.9500 | 0.0177 | 0.4700 | 3.6470 | 425.6000 | 0.0106 |
| 0.4800 | 3.9940 | 396.7833 | 0.0105 | 0.4800 | 6.2220 | 420.4500 | 0.0059 | 0.4800 | 3.7250 | 430.2333 | 0.0076 | 0.4800 | 4.2480 | 482.0167 | 0.0116 | 0.4800 | 3.6960 | 430.2333 | 0.0125 |
| 0.4900 | 4.3260 | 428.3000 | 0.0200 | 0.4900 | 6.2970 | 433.2000 | 0.0030 | 0.4900 | 3.7800 | 437.5167 | 0.0076 | 0.4900 | 4.3540 | 491.1333 | 0.0103 | 0.4900 | 3.7870 | 437.5167 | 0.0010 |
| 0.5000 | 4.4440 | 434.2000 | 0.0105 | 0.5000 | 6.3210 | 441.1167 | 0.0047 | 0.5000 | 3.9210 | 456.0667 | 0.0079 | 0.5000 | 4.5950 | 514.5833 | 0.0102 | 0.5000 | 3.8050 | 456.0667 | 0.0118 |
| 0.5100 | 4.5580 | 445.0333 | 0.0049 | 0.5100 | 6.3930 | 456.3000 | 0.0170 | 0.5100 | 4.0610 | 473.8333 | 0.0082 | 0.5100 | 4.6810 | 523.0500 | 0.0090 | 0.5100 | 4.0150 | 473.8333 | 0.0093 |
| 0.5200 | 4.6490 | 463.7500 | 0.0085 | 0.5200 | 6.6310 | 470.2667 | 0.0129 | 0.5200 | 4.1420 | 483.7667 | 0.0022 | 0.5200 | 4.7630 | 532.1833 | 0.0086 | 0.5200 | 4.1070 | 483.7667 | 0.0049 |
| 0.5300 | 4.7240 | 472.6000 | 0.0096 | 0.5300 | 6.7490 | 479.3833 | 0.0040 | 0.5300 | 4.1640 | 493.6667 | 0.0062 | 0.5300 | 4.8020 | 536.7333 | 0.0057 | 0.5300 | 4.1560 | 493.6667 | 0.0033 |
| 0.5400 | 4.8570 | 486.3833 | 0.0081 | 0.5400 | 6.8220 | 497.6167 | 0.0182 | 0.5400 | 4.2050 | 500.2833 | 0.0039 | 0.5400 | 4.8840 | 551.0667 | 0.0032 | 0.5400 | 4.1780 | 500.2833 | 0.0059 |
| 0.5500 | 4.9370 | 496.2333 | 0.0082 | 0.5500 | 7.0100 | 507.9333 | 0.0082 | 0.5500 | 4.2520 | 512.2000 | 0.0049 | 0.5500 | 4.9410 | 568.6500 | 0.0013 | 0.5500 | 4.2480 | 512.2000 | 0.0052 |

| | | | | | | | | | | | | | | | | | | | |
|---|---|---|---|---|---|---|---|---|---|---|---|---|---|---|---|---|---|---|---|
| 0.5600 | 5.0940 | 515.4167 | 0.0084 | 0.5600 | 7.1000 | 518.8833 | -0.0016 | 0.5600 | 4.3010 | 522.1000 | 0.0045 | 0.5600 | 4.9620 | 584.2833 | 0.0027 | 0.5600 | 4.2990 | 522.1000 | 0.0040 |
| 0.5700 | 5.2330 | 532.0167 | 0.0092 | 0.5700 | 7.0630 | 541.3500 | 0.0066 | 0.5700 | 4.3870 | 541.3000 | 0.0011 | 0.5700 | 4.9990 | 597.9667 | 0.0030 | 0.5700 | 4.3760 | 541.3000 | 0.0010 |
| 0.5800 | 5.3060 | 539.9667 | 0.0037 | 0.5800 | 7.2640 | 571.7333 | 0.0086 | 0.5800 | 4.4300 | 581.4667 | 0.0022 | 0.5800 | 5.0590 | 618.1667 | 0.0036 | 0.5800 | 4.4160 | 581.4667 | 0.0024 |
| 0.5900 | 5.4340 | 574.6167 | 0.0027 | 0.5900 | 7.3480 | 581.4667 | 0.0036 | 0.5900 | 4.4610 | 595.4333 | 0.0164 | 0.5900 | 5.1560 | 645.4500 | 0.1400 | 0.5900 | 4.4500 | 595.4333 | 0.0053 |
| 0.6000 | 5.5050 | 600.6000 | 0.0092 | 0.6000 | 7.4170 | 600.5400 | 0.0124 | 0.6000 | 4.5360 | 600.0000 | 0.0076 | 0.6000 | 5.2330 | 646.0000 | 0.0081 | 0.6000 | 4.4740 | 600.0000 | 0.0075 |

| 6% | | | | | | | | | | | | | | | | | | | |
|---|---|---|---|---|---|---|---|---|---|---|---|---|---|---|---|---|---|---|---|
| T1 | | | | T2 | | | | T3 | | | | T4 | | | | T5 | | | |
| P ( bar) | F (kHz) | t(s) | df_dt | P ( bar) | F (kHz) | t(s) | df_dt | P ( bar) | F (kHz) | t(s) | df_dt | P ( bar) | F (kHz) | t(s) | df_dt | P ( bar) | F (kHz) | t(s) | df_dt |
| 0.0000 | 1.5780 | 0.0000 | 0.0029 | 0.0000 | 2.8310 | 0.0000 | 0.0061 | 0.0000 | 2.3760 | 0.0000 | 0.0076 | 0.0000 | 2.4433 | 6.7000 | 0.0059 | 0.0000 | 3.1220 | 0.0000 | 0.0061 |
| 0.0200 | 1.7480 | 59.3500 | 0.0042 | 0.0300 | 2.9300 | 16.1500 | 0.0067 | 0.0300 | 2.5360 | 21.1667 | 0.0087 | 0.0200 | 2.8400 | 74.1000 | 0.0088 | 0.0300 | 3.2170 | 15.5667 | 0.0054 |
| 0.0300 | 1.8000 | 71.7667 | 0.0043 | 0.0400 | 3.0230 | 30.1167 | 0.0083 | 0.0500 | 2.6940 | 39.3000 | 0.0075 | 0.0300 | 3.0280 | 95.3667 | 0.0132 | 0.0400 | 3.3390 | 37.9667 | 0.0072 |
| 0.0400 | 1.8510 | 83.5000 | 0.0050 | 0.0500 | 3.1380 | 43.9167 | 0.0089 | 0.0600 | 2.7030 | 40.5000 | 0.0110 | 0.0500 | 3.3500 | 119.8500 | 0.1132 | 0.0500 | 3.5700 | 69.9167 | 0.0086 |
| 0.0500 | 1.9000 | 93.3000 | 0.0056 | 0.0600 | 3.1640 | 46.8500 | 0.0106 | 0.0700 | 2.7230 | 42.3167 | 0.0076 | 0.0600 | 3.7140 | 123.0667 | 0.0101 | 0.0600 | 3.7200 | 87.4000 | 0.0085 |
| 0.0600 | 1.9510 | 102.4333 | 0.0061 | 0.0800 | 3.4640 | 75.1500 | 0.0145 | 0.0800 | 2.8510 | 59.2500 | 0.0083 | 0.0700 | 3.8700 | 138.5333 | 0.0020 | 0.0800 | 3.8780 | 106.0833 | 0.0090 |
| 0.0700 | 2.0190 | 113.5167 | 0.0066 | 0.0900 | 3.5910 | 83.9333 | 0.0132 | 0.0900 | 2.8910 | 64.0833 | 0.0073 | 0.0900 | 3.8830 | 144.9833 | 0.0160 | 0.0900 | 3.9970 | 119.3500 | 0.0100 |
| 0.0800 | 2.1360 | 131.1333 | 0.0063 | 0.1000 | 3.6680 | 89.7833 | 0.0112 | 0.1000 | 2.9130 | 67.1000 | 0.0075 | 0.1000 | 3.9550 | 149.4833 | 0.0090 | 0.1000 | 4.0270 | 122.3500 | 0.0103 |
| 0.0900 | 2.1770 | 137.6500 | 0.0070 | 0.1100 | 3.7770 | 99.5500 | 0.0114 | 0.1300 | 3.0680 | 87.6500 | 0.0076 | 0.1100 | 4.0940 | 164.9500 | 0.0099 | 0.1200 | 4.1580 | 135.0167 | 0.0138 |
| 0.1000 | 2.2090 | 142.2167 | 0.0049 | 0.1300 | 3.8550 | 106.3833 | 0.0115 | 0.1500 | 3.1370 | 96.7167 | 0.0078 | 0.1200 | 4.1760 | 173.2500 | 0.0203 | 0.1300 | 4.2740 | 143.4500 | 0.0144 |
| 0.1100 | 2.2280 | 146.1333 | 0.0066 | 0.1400 | 3.9890 | 118.0833 | 0.0109 | 0.1600 | 3.1940 | 103.9833 | 0.0077 | 0.1300 | 4.2550 | 177.1500 | 0.0087 | 0.1400 | 4.4220 | 153.7000 | 0.0142 |
| 0.1200 | 2.2840 | 154.6167 | 0.0072 | 0.1500 | 4.0740 | 125.9000 | 0.0115 | 0.1800 | 3.3290 | 121.5167 | 0.0062 | 0.1500 | 4.3060 | 183.0000 | 0.0086 | 0.1500 | 4.4820 | 157.9167 | 0.1656 |
| 0.1300 | 2.3170 | 159.1833 | 0.0065 | 0.1600 | 4.1190 | 129.8000 | 0.0106 | 0.2100 | 3.4410 | 139.6500 | 0.0057 | 0.1600 | 4.3170 | 184.2833 | 0.0022 | 0.1600 | 5.5720 | 164.5000 | -0.0155 |
| 0.1400 | 2.3840 | 169.5167 | 0.0065 | 0.1700 | 4.1810 | 135.6500 | 0.0092 | 0.2200 | 3.5300 | 155.3667 | 0.0079 | 0.1700 | 4.3270 | 188.8333 | 0.0210 | 0.2200 | 5.0530 | 197.8833 | 0.0107 |
| 0.1500 | 2.4180 | 174.7667 | 0.0073 | 0.1800 | 4.2080 | 138.5833 | 0.0113 | 0.2300 | 3.5490 | 157.7833 | 0.0056 | 0.1800 | 4.3680 | 190.7833 | 0.0159 | 0.2300 | 5.1050 | 202.7333 | 0.0096 |
| 0.1600 | 2.4610 | 180.6833 | 0.0046 | 0.1900 | 4.2300 | 140.5333 | 0.0111 | 0.2600 | 3.5890 | 164.9833 | 0.0060 | 0.1900 | 4.5850 | 204.4000 | 0.0084 | 0.2500 | 5.2040 | 213.0500 | 0.0138 |
| 0.1700 | 2.4730 | 183.3000 | 0.0066 | 0.2000 | 4.4030 | 156.1500 | 0.0111 | 0.2700 | 3.6290 | 171.6833 | 0.0089 | 0.2000 | 4.6940 | 217.3833 | 0.0082 | 0.2600 | 5.3720 | 225.2000 | 0.0130 |
| 0.1800 | 2.5210 | 190.5333 | 0.0053 | 0.2100 | 4.4860 | 163.6000 | 0.0164 | 0.2800 | 3.7540 | 185.6833 | 0.0160 | 0.2100 | 4.8000 | 230.3667 | 0.0135 | 0.2700 | 5.4590 | 231.8667 | 0.0111 |
| 0.1900 | 2.5590 | 197.7667 | 0.0057 | 0.2200 | 4.5670 | 168.5333 | 0.0112 | 0.2900 | 3.7930 | 188.1167 | 0.0104 | 0.2200 | 4.8700 | 235.5500 | 0.0126 | 0.2900 | 5.5530 | 240.3667 | 0.0078 |
| 0.2000 | 2.6230 | 208.9333 | 0.0059 | 0.2300 | 4.6110 | 172.4667 | 0.0132 | 0.3000 | 3.8120 | 189.9500 | 0.0073 | 0.2300 | 4.9110 | 238.8000 | 0.0123 | 0.3000 | 5.5720 | 242.8000 | 0.0097 |
| 0.2100 | 2.6500 | 213.5333 | 0.0047 | 0.2400 | 4.7280 | 181.3333 | 0.0109 | 0.3200 | 3.8920 | 200.9000 | 0.0081 | 0.2400 | 4.9350 | 240.7500 | 0.0062 | 0.3100 | 5.6310 | 248.8667 | 0.0121 |
| 0.2200 | 2.6780 | 219.4500 | 0.0061 | 0.2500 | 4.8030 | 188.2333 | 0.0457 | 0.3300 | 3.9810 | 211.8667 | 0.0088 | 0.2500 | 4.9710 | 246.5833 | -0.0240 | 0.3200 | 5.6750 | 252.5167 | 0.0092 |
| 0.2300 | 2.7140 | 225.3500 | 0.0086 | 0.2600 | 5.1630 | 196.1167 | 0.0152 | 0.3400 | 4.0240 | 216.7333 | 0.0092 | 0.2600 | 4.7840 | 254.3667 | 0.0122 | 0.3300 | 5.7310 | 258.5833 | 0.0086 |
| 0.2400 | 2.7480 | 229.3000 | 0.0091 | 0.2700 | 5.2980 | 205.0000 | 0.0027 | 0.3600 | 4.1020 | 225.2500 | 0.0073 | 0.2800 | 4.8710 | 261.5167 | 0.0108 | 0.3400 | 5.7990 | 266.4667 | 0.0063 |
| 0.2500 | 2.7720 | 231.9333 | 0.0109 | 0.2800 | 5.3140 | 210.9000 | 0.0104 | 0.3700 | 4.2080 | 239.8667 | 0.0127 | 0.2900 | 4.8990 | 264.1000 | 0.0034 | 0.3500 | 5.8410 | 273.1500 | 0.0098 |
| 0.2600 | 2.9290 | 246.3833 | 0.0083 | 0.2900 | 5.3450 | 213.8667 | 0.0085 | 0.3800 | 4.2390 | 242.3000 | 0.0033 | 0.3000 | 4.9100 | 267.3500 | 0.0118 | 0.3700 | 5.9480 | 284.0667 | 0.0071 |
| 0.2700 | 2.9670 | 250.9833 | 0.0130 | 0.3000 | 5.3950 | 219.7833 | 0.0148 | 0.4000 | 4.2570 | 247.7833 | 0.0056 | 0.3200 | 5.0940 | 282.9167 | 0.0015 | 0.3800 | 6.0130 | 293.1833 | 0.0127 |
| 0.2800 | 3.0440 | 256.9000 | 0.0118 | 0.3100 | 5.5850 | 232.5833 | 0.0128 | 0.4100 | 4.2740 | 250.8167 | 0.0094 | 0.3300 | 5.0990 | 286.1667 | 0.0153 | 0.3900 | 6.0590 | 296.8167 | 0.0074 |
| 0.2900 | 3.0750 | 259.5167 | 0.0117 | 0.3200 | 5.6480 | 237.5167 | 0.0085 | 0.4200 | 4.4110 | 265.4333 | 0.0029 | 0.3400 | 5.1880 | 292.0000 | 0.0028 | 0.4100 | 6.0860 | 300.4667 | 0.0050 |
| 0.3000 | 3.1670 | 267.4000 | 0.0089 | 0.3300 | 5.7640 | 251.1500 | 0.0151 | 0.4300 | 4.4180 | 267.8667 | 0.0079 | 0.3500 | 5.2080 | 299.1500 | 0.0123 | 0.4300 | 6.1190 | 307.1333 | 0.0086 |
| 0.3100 | 3.2080 | 272.0000 | 0.0094 | 0.3400 | 5.8280 | 255.4000 | 0.0105 | 0.4500 | 4.5760 | 287.9667 | 0.0233 | 0.3600 | 5.3040 | 306.9333 | 0.0048 | 0.4400 | 6.1610 | 312.0000 | 0.0058 |
| 0.3200 | 3.3010 | 281.8667 | 0.0168 | 0.3500 | 5.8920 | 261.4667 | 0.0108 | 0.4600 | 4.5900 | 288.5667 | 0.0059 | 0.3800 | 5.3350 | 313.4167 | 0.0128 | 0.4500 | 6.2380 | 325.3500 | 0.0089 |
| 0.3300 | 3.4550 | 291.0500 | 0.0135 | 0.3600 | 6.0030 | 271.7833 | 0.0008 | 0.4700 | 4.6080 | 291.6167 | 0.0058 | 0.3900 | 5.3600 | 315.3667 | 0.0100 | 0.4600 | 6.2650 | 328.3833 | 0.0081 |
| 0.3400 | 3.5170 | 295.6500 | 0.0165 | 0.3700 | 6.0060 | 275.4167 | 0.0113 | 0.4800 | 4.6540 | 299.5333 | 0.0040 | 0.4000 | 5.4570 | 325.1000 | 0.0069 | 0.4700 | 6.3390 | 337.4833 | 0.0060 |
| 0.3500 | 3.5930 | 300.2500 | 0.0166 | 0.3800 | 6.2330 | 295.4333 | 0.0108 | 0.4900 | 4.7310 | 319.0167 | 0.0049 | 0.4200 | 5.5830 | 343.2667 | 0.0231 | 0.4800 | 6.3830 | 344.7667 | 0.0105 |
| 0.3600 | 3.6260 | 302.2333 | 0.0126 | 0.3900 | 6.3050 | 302.1000 | 0.0071 | 0.5000 | 4.7520 | 323.2667 | 0.0046 | 0.4300 | 5.6130 | 344.5667 | 0.0078 | 0.4900 | 6.4150 | 347.8167 | 0.0031 |
| 0.3700 | 3.7500 | 312.0833 | 0.0114 | 0.4000 | 6.3570 | 309.3833 | 0.0088 | 0.5100 | 4.7800 | 329.3667 | 0.0011 | 0.4400 | 5.6640 | 351.0667 | 0.0074 | 0.5000 | 6.4470 | 358.1333 | 0.0069 |
| 0.3800 | 3.8470 | 320.6167 | 0.0082 | 0.4100 | 6.4320 | 317.8833 | 0.0053 | 0.5200 | 4.7840 | 333.0167 | 0.0092 | 0.4500 | 5.7790 | 366.6333 | 0.0098 | 0.5200 | 6.4890 | 364.2000 | 0.0086 |
| 0.3900 | 3.8900 | 325.8833 | 0.0089 | 0.4200 | 6.4640 | 323.9500 | 0.0088 | 0.5300 | 4.8230 | 337.2667 | 0.0082 | 0.4600 | 5.8490 | 373.7667 | 0.0089 | 0.5300 | 6.5100 | 366.6333 | 0.0051 |
| 0.4000 | 3.9600 | 333.7667 | 0.0076 | 0.4300 | 6.5710 | 336.0833 | 0.0082 | 0.5400 | 4.8380 | 339.1000 | 0.0051 | 0.4700 | 5.8780 | 377.0167 | 0.0082 | 0.5500 | 6.5810 | 380.5833 | 0.0049 |
| 0.4100 | 4.0100 | 340.3333 | 0.0012 | 0.4400 | 6.6910 | 350.6500 | 0.0086 | 0.5500 | 4.8660 | 344.5833 | 0.0027 | 0.4800 | 5.9260 | 382.8500 | 0.0103 | 0.5600 | 6.6410 | 392.7333 | 0.0048 |
| 0.4200 | 4.0140 | 343.6167 | 0.0106 | 0.4500 | 6.8160 | 365.2000 | -0.0040 | 0.5600 | 4.9140 | 362.2333 | 0.0039 | 0.4900 | 5.9660 | 386.7500 | 0.0054 | 0.5800 | 6.6670 | 398.1833 | 0.0045 |
| 0.4300 | 4.1180 | 353.4667 | 0.0118 | 0.4600 | 6.7630 | 378.5500 | 0.0038 | 0.5700 | 5.0100 | 386.5833 | -0.0006 | 0.5000 | 5.9870 | 390.6500 | 0.0060 | 0.5900 | 6.6970 | 404.8667 | 0.0040 |
| 0.4400 | 4.3190 | 370.5500 | 0.0138 | 0.4700 | 6.8070 | 390.0667 | 0.0062 | 0.5900 | 5.0030 | 397.5500 | -0.0019 | 0.5100 | 6.0260 | 397.1333 | 0.0060 | 0.6000 | 6.7330 | 413.9667 | 0.0032 |
| 0.4500 | 4.4550 | 380.4167 | 0.0116 | 0.4800 | 6.9010 | 405.2333 | 0.0034 | 0.6000 | 4.9950 | 401.8000 | 0.0097 | 0.5300 | 6.1040 | 410.1167 | 0.0067 | 0.6200 | 6.7580 | 421.8667 | -0.0003 |
| 0.4600 | 4.5690 | 390.2667 | 0.0107 | 0.4900 | 6.9260 | 412.5167 | 0.0043 | 0.6100 | 5.0010 | 402.4167 | -0.0002 | 0.5400 | 6.1300 | 414.0000 | 0.0071 | 0.6300 | 6.7570 | 425.5000 | 0.0032 |
| 0.4700 | 4.6110 | 394.2000 | 0.0097 | 0.5000 | 7.0540 | 442.2500 | 0.0018 | 0.6200 | 4.9960 | 434.0667 | 0.0048 | 0.5500 | 6.1990 | 423.7333 | 0.0059 | 0.6500 | 6.8230 | 446.1500 | 0.0025 |
| 0.4800 | 4.6880 | 402.1000 | 0.0094 | 0.5100 | 7.0950 | 464.6833 | 0.0044 | 0.6300 | 5.0370 | 442.6000 | -0.0011 | 0.5600 | 6.2450 | 431.5167 | 0.0068 | 0.6600 | 6.8350 | 451.0000 | 0.0036 |
| 0.4900 | 4.8600 | 420.4833 | 0.0075 | 0.5200 | 7.1300 | 472.5667 | 0.0028 | 0.6500 | 5.0170 | 460.2167 | 0.0040 | 0.5700 | 6.3510 | 447.1000 | 0.0067 | 0.6700 | 6.8460 | 454.0333 | 0.0033 |
| 0.5000 | 4.9580 | 433.6333 | 0.0015 | 0.5300 | 7.1670 | 485.9167 | 0.0027 | 0.6600 | 5.0210 | 461.2167 | 0.0024 | 0.5800 | 6.3770 | 450.9833 | 0.0079 | 0.6800 | 6.8520 | 455.8500 | 0.0025 |
| 0.5100 | 4.9600 | 434.9500 | 0.0088 | 0.5400 | 7.2220 | 505.9333 | 0.0030 | 0.6700 | 5.0260 | 463.3167 | 0.0000 | 0.5900 | 6.4230 | 456.8333 | 0.0057 | 0.7000 | 6.8960 | 473.4500 | 0.0033 |

| | | | | | | | | | | | | | | | | | | | |
|---|---|---|---|---|---|---|---|---|---|---|---|---|---|---|---|---|---|---|---|
| 0.5200 | 5.0290 | 442.8333 | 0.0065 | 0.5500 | 7.2640 | 519.9000 | 0.0113 | 0.6800 | 5.0270 | 483.4667 | 0.2154 | 0.6000 | 6.4600 | 463.3167 | 0.0067 | 0.7100 | 6.9060 | 476.5000 | 0.0023 |
| 0.5300 | 5.0970 | 453.3333 | 0.0061 | 0.5600 | 7.3600 | 528.3833 | 0.0015 | 0.6900 | 6.0000 | 487.9833 | 0.0068 | 0.6200 | 6.5080 | 470.4500 | 0.0059 | 0.7300 | 6.9410 | 491.6667 | 0.0021 |
| 0.5400 | 5.1530 | 462.5333 | 0.0072 | 0.5700 | 7.3700 | 535.0667 | -0.0072 | 0.7000 | 6.0240 | 491.5167 | 0.0107 | 0.6300 | 6.5880 | 484.0833 | 0.0068 | 0.7400 | 6.9710 | 505.6333 | 0.0005 |
| 0.5500 | 5.2050 | 469.7833 | 0.0043 | 0.5800 | 7.3180 | 542.3333 | -0.0264 | 0.7100 | 6.1590 | 504.1000 | 0.0701 | 0.6400 | 6.6810 | 497.7167 | 0.0026 | 0.7500 | 6.9750 | 512.9167 | -0.0022 |
| 0.5600 | 5.2190 | 473.0500 | 0.0051 | 0.5900 | 7.2380 | 545.3667 | 0.0026 | 0.7200 | 6.5480 | 509.6500 | 0.0311 | 0.6500 | 6.7060 | 507.4500 | 0.0056 | 0.7600 | 6.9630 | 518.3667 | 0.0002 |
| 0.5700 | 5.2960 | 488.1667 | 0.0059 | 0.6000 | 7.2460 | 548.4000 | -0.0010 | 0.7300 | 6.9870 | 523.7500 | 0.0035 | 0.6700 | 6.7900 | 522.3667 | 0.0053 | 0.7700 | 6.9640 | 524.4500 | 0.0006 |
| 0.5800 | 5.3740 | 501.3000 | 0.0041 | 0.6100 | 7.2410 | 553.2667 | 0.0054 | 0.7400 | 7.0150 | 531.8000 | 0.0132 | 0.6800 | 6.8420 | 532.1000 | 0.0018 | 0.7800 | 6.9650 | 526.2667 | 0.0013 |
| 0.5900 | 5.4040 | 508.5333 | 0.0029 | 0.6200 | 7.2770 | 559.9333 | -0.0020 | 0.7500 | 7.1480 | 541.9000 | 0.0144 | 0.7100 | 6.8880 | 557.4167 | -0.0017 | 0.7900 | 6.9880 | 543.8667 | 0.0016 |
| 0.6000 | 5.4400 | 521.0167 | 0.0060 | 0.6300 | 7.2580 | 569.6333 | 0.0013 | 0.7600 | 7.3650 | 556.9833 | 0.0302 | 0.7200 | 6.8770 | 563.9000 | -0.0034 | 0.8000 | 6.9980 | 549.9333 | 0.0011 |
| 0.6100 | 5.5150 | 533.4833 | 0.0050 | 0.6400 | 7.2710 | 579.3500 | 0.0040 | 0.7700 | 7.5780 | 564.0333 | 0.0256 | 0.7300 | 6.8480 | 572.3333 | -0.0004 | 0.8100 | 7.0130 | 563.3000 | 0.0002 |
| 0.6200 | 5.6200 | 554.5167 | 0.0060 | 0.6500 | 7.2830 | 582.3833 | -0.0007 | 0.7800 | 7.8750 | 575.6167 | 8.4036 | 0.7400 | 6.8380 | 596.3500 | 0.0050 | 0.8200 | 7.0150 | 572.4000 | -0.0009 |
| 0.6300 | 5.6750 | 563.7167 | 0.0047 | 0.6600 | 7.2810 | 585.3167 | -0.0018 | 0.7900 | 8.0145 | 575.6333 | 0.0070 | 0.7500 | 6.8510 | 598.9333 | 0.0021 | 0.8300 | 7.0050 | 583.9333 | 0.0007 |
| 0.6400 | 5.7730 | 584.7500 | 0.0012 | 0.6700 | 7.2690 | 591.9167 | 0.0003 | 0.8000 | 8.1480 | 594.7500 | 0.0103 | 0.7600 | 6.8590 | 602.8333 | 0.0014 | 0.8400 | 7.0120 | 593.6500 | 0.0041 |
| 0.6500 | 5.8100 | 615.6167 | 0.0094 | 0.6800 | 7.2720 | 601.1833 | 0.0121 | 0.8100 | 8.2360 | 603.3167 | 0.0137 | 0.7700 | 6.8870 | 622.3000 | 0.0111 | 0.8500 | 7.0440 | 601.5333 | 0.0117 |

| 12% | | | | | | | | | | | | | | | | | | | |
|---|---|---|---|---|---|---|---|---|---|---|---|---|---|---|---|---|---|---|---|
| T1 | | | | T2 | | | | T3 | | | | T4 | | | | T5 | | | |
| P ( bar) | F (kHz) | t(s) | df_dt | P ( bar) | F (kHz) | t(s) | df_dt | P ( bar) | F (kHz) | t(s) | df_dt | P ( bar) | F (kHz) | t(s) | df_dt | P ( bar) | F (kHz) | t(s) | df_dt |
| 0.0000 | 1.8770 | 0.0000 | 0.0010 | 0.0000 | 2.0300 | 0.0000 | 0.0020 | 0.0000 | 3.1380 | 0.0000 | 0.0025 | 0.0000 | 3.6040 | 0.0000 | 0.0014 | 0.0000 | 2.1980 | 0.0000 | 0.0023 |
| 0.0200 | 1.8820 | 5.0833 | 0.0011 | 0.0200 | 2.0630 | 16.8833 | 0.0023 | 0.0200 | 3.1400 | 0.8000 | 0.0053 | 0.0200 | 3.6880 | 62.0500 | 0.0057 | 0.0200 | 2.2800 | 36.1500 | 0.0035 |
| 0.0300 | 1.8870 | 9.5500 | 0.0012 | 0.0300 | 2.1570 | 58.2833 | 0.0029 | 0.0300 | 3.2130 | 14.6500 | 0.0086 | 0.0300 | 3.8300 | 87.0000 | 0.0061 | 0.0300 | 2.4110 | 73.6000 | 0.0066 |
| 0.0400 | 1.9150 | 32.0500 | 0.0019 | 0.0400 | 2.2640 | 95.3167 | 0.0021 | 0.0500 | 3.3460 | 30.1167 | 0.0163 | 0.0400 | 3.8860 | 96.1333 | 0.0072 | 0.0400 | 2.4750 | 83.2833 | 0.0047 |
| 0.0500 | 1.9850 | 68.5667 | 0.0029 | 0.0500 | 2.2840 | 105.0333 | 0.0050 | 0.0600 | 3.3990 | 33.3667 | 0.0106 | 0.0500 | 3.9730 | 108.3000 | 0.0061 | 0.0500 | 2.6110 | 112.3333 | 0.0506 |
| 0.0600 | 2.1280 | 117.9167 | 0.0039 | 0.0600 | 2.3540 | 119.0000 | 0.0040 | 0.0700 | 3.5030 | 43.1333 | 0.0089 | 0.0600 | 4.0100 | 114.3833 | 0.0091 | 0.0600 | 2.6760 | 113.6167 | 0.0041 |
| 0.0700 | 2.2170 | 140.9833 | 0.0042 | 0.0700 | 2.4260 | 137.2167 | 0.0027 | 0.0800 | 3.5900 | 52.9000 | 0.0159 | 0.0700 | 4.0820 | 122.2833 | 0.0134 | 0.0700 | 2.7210 | 124.6000 | 0.0002 |
| 0.0800 | 2.3160 | 164.5667 | 0.0050 | 0.0800 | 2.4460 | 144.5000 | 0.0041 | 0.0900 | 3.7580 | 63.4833 | 0.0106 | 0.0800 | 4.2770 | 136.8833 | 0.0089 | 0.0800 | 2.7220 | 131.0500 | 0.0036 |
| 0.0900 | 2.3710 | 175.5500 | 0.0047 | 0.0900 | 2.5980 | 181.5833 | 0.0043 | 0.1000 | 3.8100 | 68.3667 | 0.0104 | 0.0900 | 4.3260 | 142.3667 | 0.0179 | 0.0900 | 2.7570 | 140.7333 | 0.0055 |
| 0.1000 | 2.4350 | 189.1000 | 0.0048 | 0.1000 | 2.6140 | 185.2667 | 0.0016 | 0.1100 | 3.8860 | 75.6833 | 0.0177 | 0.1000 | 4.4020 | 146.6167 | 0.0127 | 0.1000 | 2.8000 | 148.4833 | 0.0050 |
| 0.1100 | 2.4660 | 195.5500 | 0.0050 | 0.1100 | 2.6200 | 188.9333 | 0.0099 | 0.1200 | 3.9870 | 81.3833 | 0.0088 | 0.1100 | 4.5180 | 155.7500 | 0.0141 | 0.1100 | 2.8580 | 160.1000 | 0.0036 |
| 0.1200 | 2.5110 | 204.5833 | 0.0050 | 0.1200 | 2.6320 | 190.1500 | 0.0026 | 0.1300 | 4.0370 | 87.0833 | 0.0104 | 0.1200 | 4.5780 | 160.0167 | 0.0140 | 0.1200 | 2.8830 | 167.0833 | 0.0102 |
| 0.1300 | 2.5560 | 213.6167 | 0.0048 | 0.1300 | 2.6430 | 194.4333 | 0.0065 | 0.1400 | 4.1390 | 96.8500 | 0.0185 | 0.1300 | 4.6460 | 164.8833 | 0.0119 | 0.1300 | 2.9160 | 170.3333 | 0.0031 |
| 0.1400 | 2.6450 | 232.3333 | 0.0049 | 0.1400 | 2.6590 | 196.8833 | 0.0016 | 0.1500 | 4.2440 | 102.5333 | 0.0111 | 0.1400 | 4.7910 | 177.0667 | 0.0073 | 0.1400 | 2.9520 | 182.0333 | 0.0015 |
| 0.1500 | 2.6800 | 239.4333 | 0.0053 | 0.1500 | 2.6680 | 202.3833 | 0.0059 | 0.1600 | 4.3070 | 108.2333 | 0.0129 | 0.1500 | 4.8090 | 179.5167 | 0.0136 | 0.1500 | 2.9590 | 186.5833 | 0.0006 |
| 0.1600 | 2.6970 | 242.6667 | 0.0049 | 0.1600 | 2.7360 | 214.0000 | 0.0110 | 0.1800 | 4.4120 | 116.3833 | 0.0010 | 0.1600 | 4.9260 | 188.1000 | 0.0127 | 0.1600 | 2.9630 | 193.0833 | 0.0043 |
| 0.1700 | 2.7480 | 253.0000 | 0.0049 | 0.1700 | 2.8370 | 223.1667 | 0.0122 | 0.1900 | 4.4190 | 123.5000 | 0.0201 | 0.1700 | 4.9880 | 193.0000 | 0.0089 | 0.1700 | 3.0110 | 204.1333 | 0.0070 |
| 0.1800 | 2.7670 | 256.8667 | 0.0045 | 0.1800 | 3.1210 | 246.4000 | 0.0098 | 0.2000 | 4.6190 | 133.4667 | 0.0099 | 0.1800 | 5.0370 | 198.5167 | 0.0118 | 0.1800 | 3.0610 | 211.2833 | 0.0190 |
| 0.1900 | 2.8140 | 267.2000 | 0.0045 | 0.1900 | 3.1690 | 251.3000 | 0.0100 | 0.2200 | 4.7240 | 144.0500 | 0.0103 | 0.1900 | 5.0950 | 203.4167 | 0.0160 | 0.1900 | 3.1600 | 216.4833 | 0.0031 |
| 0.2000 | 2.8810 | 282.0333 | 0.0054 | 0.2000 | 3.3100 | 265.3500 | 0.0211 | 0.2300 | 4.7910 | 150.5500 | 0.0097 | 0.2000 | 5.2620 | 213.8333 | 0.0027 | 0.2000 | 3.1800 | 222.9833 | 0.0003 |
| 0.2100 | 2.9020 | 285.9167 | 0.0043 | 0.2100 | 3.3230 | 265.9667 | 0.0101 | 0.2400 | 4.8620 | 157.8833 | 0.0180 | 0.2100 | 5.2770 | 219.3500 | 0.0140 | 0.2100 | 3.1820 | 230.8000 | 0.0036 |
| 0.2200 | 2.9240 | 291.0667 | 0.0044 | 0.2200 | 3.4830 | 281.8667 | 0.0106 | 0.2500 | 5.0920 | 170.6500 | 0.0093 | 0.2200 | 5.3370 | 223.6333 | 0.0126 | 0.2200 | 3.2080 | 237.9500 | 0.0070 |
| 0.2300 | 2.9550 | 298.1667 | 0.0041 | 0.2300 | 3.5220 | 285.5333 | 0.0108 | 0.2700 | 5.2370 | 186.2500 | 0.0114 | 0.2300 | 5.4140 | 229.7667 | 0.0102 | 0.2300 | 3.2580 | 245.1000 | 0.0133 |
| 0.2400 | 2.9870 | 305.9167 | 0.0045 | 0.2400 | 3.5550 | 288.5833 | 0.0100 | 0.2800 | 5.3050 | 192.2333 | 0.0050 | 0.2400 | 5.4700 | 235.2833 | 0.0084 | 0.2400 | 3.3100 | 249.0000 | 0.0043 |
| 0.2500 | 3.0160 | 312.3667 | 0.0035 | 0.2500 | 3.7200 | 305.1000 | 0.0077 | 0.2900 | 5.3270 | 196.6000 | 0.0032 | 0.2500 | 5.5160 | 240.7833 | 0.0082 | 0.2500 | 3.3490 | 258.1000 | 0.0003 |
| 0.2600 | 3.0630 | 325.9167 | 0.0051 | 0.2600 | 3.7530 | 309.3833 | 0.0082 | 0.3000 | 5.3410 | 200.9667 | 0.0097 | 0.2600 | 5.6060 | 251.8167 | 0.0104 | 0.2600 | 3.3510 | 263.9500 | 0.0092 |
| 0.2700 | 3.0990 | 333.0167 | 0.0072 | 0.2700 | 3.8230 | 317.9333 | 0.0100 | 0.3200 | 5.4940 | 216.7000 | 0.0019 | 0.2700 | 5.6380 | 254.8833 | 0.0019 | 0.2700 | 3.3930 | 268.5000 | 0.0150 |
| 0.2800 | 3.1500 | 340.1167 | 0.0084 | 0.2800 | 3.8720 | 322.8333 | 0.0089 | 0.3300 | 5.5040 | 221.9500 | 0.0082 | 0.2800 | 5.6450 | 258.5667 | 0.0113 | 0.2800 | 3.5000 | 275.6500 | 0.0082 |
| 0.2900 | 3.1990 | 345.9333 | 0.0095 | 0.2900 | 3.9430 | 330.7833 | 0.0093 | 0.3500 | 5.5900 | 232.4333 | 0.0076 | 0.2900 | 5.7210 | 265.3000 | 0.0088 | 0.2900 | 3.5800 | 285.4000 | 0.0059 |
| 0.3000 | 3.2420 | 350.4500 | 0.0081 | 0.3000 | 3.9600 | 332.6167 | 0.0079 | 0.3600 | 5.6100 | 235.0500 | 0.0041 | 0.3000 | 5.7640 | 270.2000 | 0.0080 | 0.3000 | 3.6340 | 294.5000 | 0.0158 |
| 0.3100 | 3.2940 | 356.9000 | 0.0083 | 0.3100 | 4.0130 | 339.3333 | 0.0049 | 0.3700 | 5.6350 | 241.1667 | 0.0062 | 0.3100 | 5.8180 | 276.9333 | 0.0071 | 0.3100 | 3.7060 | 299.0500 | 0.0054 |
| 0.3200 | 3.4070 | 370.4500 | 0.0097 | 0.3200 | 4.0280 | 342.4000 | 0.0071 | 0.3800 | 5.7220 | 255.1500 | 0.0076 | 0.3200 | 5.8960 | 287.9667 | -0.0012 | 0.3200 | 3.7200 | 301.6500 | 0.0062 |
| 0.3300 | 3.5140 | 381.4333 | 0.0089 | 0.3300 | 4.0760 | 349.1167 | 0.0055 | 0.3900 | 5.7620 | 260.3833 | 0.0015 | 0.3300 | 5.8910 | 292.2500 | 0.0051 | 0.3300 | 3.7600 | 308.1500 | 0.0008 |
| 0.3400 | 3.5540 | 385.9500 | 0.0097 | 0.3400 | 4.1530 | 363.1833 | 0.0077 | 0.4100 | 5.7760 | 270.0000 | 0.0092 | 0.3400 | 5.9130 | 296.5500 | 0.0045 | 0.3400 | 3.7700 | 320.5000 | 0.0006 |
| 0.3500 | 3.6040 | 391.1000 | 0.0031 | 0.3500 | 4.2140 | 371.1333 | 0.0041 | 0.4200 | 5.8000 | 272.6167 | 0.0051 | 0.3500 | 5.9350 | 301.4500 | 0.0088 | 0.3500 | 3.7900 | 354.3167 | 0.0462 |
| 0.3600 | 3.6260 | 398.2000 | 0.0137 | 0.3600 | 4.2570 | 381.5333 | 0.0004 | 0.4300 | 5.8400 | 280.4833 | 0.0037 | 0.3600 | 6.0000 | 308.8000 | 0.0003 | 0.3600 | 3.9100 | 356.9167 | 0.0140 |
| 0.3700 | 3.7320 | 405.9500 | -0.0033 | 0.3700 | 4.2590 | 386.4167 | 0.0047 | 0.4500 | 5.9020 | 297.0833 | 0.0031 | 0.3700 | 6.0010 | 312.4833 | 0.0061 | 0.3700 | 4.0100 | 364.0667 | 0.0036 |
| 0.3800 | 3.6790 | 422.0833 | 0.0515 | 0.3800 | 4.2790 | 390.7000 | 0.0051 | 0.4700 | 5.9370 | 308.4500 | 0.0138 | 0.3800 | 6.0680 | 323.5000 | 0.0059 | 0.3800 | 4.0400 | 372.5167 | 0.0205 |
| 0.3900 | 3.9450 | 427.2500 | 0.0084 | 0.3900 | 4.3630 | 407.2000 | 0.0065 | 0.4800 | 5.9490 | 309.3167 | 0.0024 | 0.3900 | 6.0970 | 328.4000 | 0.0117 | 0.3900 | 4.1600 | 378.3667 | 0.0037 |
| 0.4000 | 4.0480 | 439.5167 | 0.0098 | 0.4000 | 4.5380 | 434.1000 | 0.0019 | 0.5000 | 6.0060 | 332.9167 | 0.0051 | 0.4000 | 6.1400 | 332.0833 | 0.0062 | 0.4000 | 4.1770 | 382.9167 | 0.0065 |
| 0.4100 | 4.1240 | 447.2500 | 0.0098 | 0.4100 | 4.5620 | 446.9500 | 0.0050 | 0.5100 | 6.0150 | 334.6667 | 0.0011 | 0.4100 | 6.2010 | 341.8833 | 0.0046 | 0.4100 | 4.2700 | 397.2167 | 0.0096 |
| 0.4200 | 4.2320 | 458.2333 | 0.0091 | 0.4200 | 4.6020 | 454.9000 | 0.0027 | 0.5400 | 6.0380 | 355.6333 | 0.0008 | 0.4200 | 6.2150 | 344.9500 | 0.0041 | 0.4200 | 4.3200 | 402.4167 | 0.0028 |

| | | | | | | | | | | | | | | | | | | | |
|---|---|---|---|---|---|---|---|---|---|---|---|---|---|---|---|---|---|---|---|
| 0.4300 | 4.3320 | 469.2000 | 0.0079 | 0.4300 | 4.6120 | 458.5667 | 0.0022 | 0.5500 | 6.0420 | 360.8833 | 0.0024 | 0.4400 | 6.2810 | 360.8833 | 0.0037 | 0.4300 | 4.3400 | 409.5667 | 0.0023 |
| 0.4400 | 4.3980 | 477.5833 | 0.0093 | 0.4400 | 4.6160 | 460.4000 | 0.0006 | 0.5600 | 6.0780 | 375.7333 | 0.0027 | 0.4500 | 6.3080 | 368.2333 | -0.0028 | 0.4400 | 4.3700 | 422.5833 | 0.0038 |
| 0.4500 | 4.4700 | 485.3333 | 0.0074 | 0.4500 | 4.6250 | 476.3000 | 0.0000 | 0.5700 | 6.0850 | 378.3500 | 0.0005 | 0.4600 | 6.2960 | 372.5167 | 0.0061 | 0.4500 | 4.3900 | 427.7833 | 0.0060 |
| 0.4600 | 4.6080 | 504.0500 | 0.0064 | 0.4600 | 4.6250 | 478.7333 | 0.0018 | 0.5800 | 6.0900 | 387.9667 | 0.0019 | 0.4700 | 6.3480 | 381.1000 | 0.0027 | 0.4600 | 4.4250 | 433.6333 | 0.0023 |
| 0.4700 | 4.6620 | 512.4333 | 0.0043 | 0.4700 | 4.6530 | 494.6333 | 0.0003 | 0.5900 | 6.1080 | 397.5833 | 0.0026 | 0.4800 | 6.3660 | 387.8333 | 0.0002 | 0.4700 | 4.4580 | 447.9333 | 0.0145 |
| 0.4800 | 4.7260 | 527.2833 | 0.0062 | 0.4800 | 4.6570 | 506.8667 | 0.0026 | 0.6000 | 6.1240 | 403.7167 | 0.0008 | 0.5000 | 6.3710 | 409.9000 | 0.0031 | 0.4800 | 4.5520 | 454.4333 | 0.0042 |
| 0.4900 | 4.7820 | 536.3167 | 0.0043 | 0.4900 | 4.6680 | 511.1500 | 0.0016 | 0.6100 | 6.1330 | 414.5500 | 0.0008 | 0.5100 | 6.3880 | 415.4167 | -0.0003 | 0.4900 | 4.6100 | 468.0833 | 0.0047 |
| 0.5000 | 4.8700 | 556.9667 | 0.0041 | 0.5000 | 4.6870 | 522.7667 | 0.0012 | 0.6200 | 6.1430 | 426.8167 | 0.0012 | 0.5600 | 6.3760 | 463.2000 | -0.0013 | 0.5000 | 4.6500 | 476.5333 | 0.0110 |
| 0.5100 | 4.9200 | 569.2333 | 0.0031 | 0.5100 | 4.7040 | 536.8333 | 0.0034 | 0.6400 | 6.1580 | 439.6833 | 0.0002 | 0.5700 | 6.3620 | 473.6167 | 0.0047 | 0.5100 | 4.7000 | 481.0833 | 0.0173 |
| 0.5200 | 4.9260 | 571.1667 | 0.0038 | 0.5200 | 4.7310 | 544.7667 | 0.0020 | 0.6600 | 6.1630 | 461.6333 | 0.0009 | 0.5800 | 6.3880 | 479.1333 | -0.0006 | 0.5200 | 4.8800 | 491.4833 | 0.0047 |
| 0.5300 | 4.9730 | 583.4333 | 0.0052 | 0.5300 | 4.7470 | 552.7167 | 0.0012 | 0.6700 | 6.1770 | 477.5333 | 0.0018 | 0.5900 | 6.3820 | 489.5500 | 0.0038 | 0.5300 | 4.9600 | 508.4000 | 0.0119 |
| 0.5400 | 5.0300 | 594.4000 | 0.0039 | 0.5400 | 4.7530 | 557.6167 | 0.0021 | 0.6800 | 6.1850 | 482.0667 | 0.0007 | 0.6000 | 6.4190 | 499.3500 | 0.0023 | 0.5400 | 5.1070 | 520.7500 | 0.0126 |
| 0.5500 | 5.0550 | 600.8667 | 0.0057 | 0.5500 | 4.7700 | 565.5667 | 0.0010 | 0.7100 | 6.1970 | 498.7167 | 0.0003 | 0.6100 | 6.4370 | 507.0667 | 0.0048 | 0.5500 | 5.3200 | 537.6500 | 0.0098 |
| 0.5600 | 5.1320 | 614.4167 | 0.0021 | 0.5600 | 4.7750 | 570.4500 | 0.0035 | 0.7400 | 6.2080 | 534.2833 | 0.0002 | 0.6200 | 6.5000 | 520.1000 | 0.0027 | 0.5600 | 5.5800 | 564.3000 | 0.0176 |
| 0.5700 | 5.1520 | 624.1000 | 0.0037 | 0.5700 | 4.8390 | 588.8000 | 0.0014 | 0.7500 | 6.2100 | 544.1167 | 0.0014 | 0.6500 | 6.5640 | 543.7000 | 0.0039 | 0.5700 | 5.8200 | 577.9500 | 0.0437 |
| 0.5800 | 5.2090 | 639.5833 | 0.0001 | 0.5800 | 4.8630 | 605.9167 | 0.0011 | 0.7600 | 6.2230 | 553.2000 | 0.0011 | 0.6600 | 6.6300 | 560.7833 | 0.0027 | 0.5800 | 6.3600 | 590.3000 | 0.0034 |
| 0.5900 | 5.2100 | 655.0666 | 0.0008 | 0.5900 | 4.8810 | 623.0334 | 0.0023 | 0.7900 | 6.2600 | 588.0167 | 0.0002 | 0.6800 | 6.6970 | 585.2000 | 0.0031 | 0.5900 | 6.4600 | 619.5667 | 0.0036 |
| 0.6000 | 5.2220 | 670.5499 | 0.0078 | 0.6000 | 4.9200 | 640.1501 | 0.0077 | 0.8000 | 6.2630 | 600.1333 | 0.0104 | 0.7000 | 6.7480 | 601.4833 | 0.0112 | 0.6000 | 6.5500 | 644.2667 | 0.0102 |

| 21% | | | | | | | | | | | | | | | | | | | |
|---|---|---|---|---|---|---|---|---|---|---|---|---|---|---|---|---|---|---|---|
| T1 | | | | T2 | | | | T3 | | | | T4 | | | | T5 | | | |
| P ( bar) | F (kHz) | t(s) | df_dt | P ( bar) | F (kHz) | t(s) | df_dt | P ( bar) | F (kHz) | t(s) | df_dt | P ( bar) | F (kHz) | t(s) | df_dt | P ( bar) | F (kHz) | t(s) | df_dt |
| 0.0000 | 2.4140 | 0.0000 | 0.0008 | 0.0000 | 2.5040 | 0.0000 | 0.0024 | 0.0000 | 2.6750 | 0.0000 | 0.0012 | 0.0000 | 3.7520 | 0.0000 | 0.0018 | 0.0000 | 2.6300 | 0.0000 | 0.0005336 |
| 0.0200 | 2.4220 | 10.3000 | 0.0009 | 0.0200 | 2.5720 | 28.5167 | 0.0023 | 0.0200 | 2.6780 | 2.5000 | -0.0040 | 0.0200 | 3.7840 | 18.0500 | 0.0028 | 0.0200 | 2.6390 | 16.8667 | 0.00071322 |
| 0.0300 | 2.5070 | 107.1833 | 0.0020 | 0.0300 | 2.6970 | 83.5500 | 0.0017 | 0.0300 | 2.6730 | 3.7500 | 0.0007 | 0.0300 | 3.7940 | 21.6667 | 0.0022 | 0.0300 | 2.6470 | 28.0833 | 0.00100398 |
| 0.0400 | 2.5930 | 150.0667 | 0.0023 | 0.0400 | 2.7160 | 94.8167 | 0.0021 | 0.0400 | 2.6780 | 10.6000 | 0.0001 | 0.0400 | 3.8020 | 25.2833 | 0.0025 | 0.0400 | 2.6680 | 49.0000 | 0.0016841 |
| 0.0500 | 2.6470 | 173.4000 | 0.0026 | 0.0500 | 2.8070 | 138.5833 | 0.0024 | 0.0500 | 2.6820 | 41.4500 | 0.0012 | 0.0500 | 3.8110 | 28.8833 | 0.0034 | 0.0500 | 2.7780 | 114.3167 | 0.00223167 |
| 0.0600 | 2.7370 | 207.5333 | 0.0028 | 0.0600 | 2.8670 | 163.1167 | 0.0027 | 0.0600 | 2.7400 | 91.0667 | 0.0019 | 0.0600 | 3.8420 | 37.9167 | 0.0050 | 0.0600 | 2.8130 | 130.0000 | 0.00211082 |
| 0.0700 | 2.7850 | 224.9333 | 0.0032 | 0.0700 | 2.9100 | 178.9500 | 0.0031 | 0.0700 | 2.7950 | 120.5667 | 0.0025 | 0.0700 | 4.0120 | 72.2167 | 0.0080 | 0.0700 | 2.8530 | 148.9500 | 0.00261997 |
| 0.0800 | 2.8730 | 252.1000 | 0.0069 | 0.0800 | 2.9750 | 199.6500 | 0.0032 | 0.0800 | 2.9420 | 179.6167 | 0.0027 | 0.0800 | 4.2000 | 95.7000 | 0.0070 | 0.0800 | 2.9540 | 187.5000 | 0.00304147 |
| 0.0900 | 2.9160 | 258.3667 | 0.0065 | 0.0900 | 3.0090 | 210.3333 | 0.0032 | 0.0900 | 2.9830 | 194.7833 | 0.0031 | 0.0900 | 4.2380 | 101.1167 | 0.0082 | 0.0900 | 3.0200 | 209.2000 | 0.00329114 |
| 0.1000 | 2.9790 | 268.1167 | 0.0067 | 0.1000 | 3.0520 | 223.7000 | 0.0015 | 0.1000 | 3.0280 | 209.3333 | 0.0032 | 0.1000 | 4.4740 | 130.0000 | 0.0076 | 0.1000 | 3.0590 | 221.0500 | 0.00531646 |
| 0.1100 | 3.0350 | 276.4667 | 0.0072 | 0.1100 | 3.0550 | 225.7000 | 0.0034 | 0.1100 | 3.0760 | 224.5167 | 0.0022 | 0.1100 | 4.5430 | 139.0333 | 0.0078 | 0.1100 | 3.1010 | 228.9500 | 0.00678363 |
| 0.1200 | 3.1560 | 293.1833 | 0.0062 | 0.1200 | 3.0890 | 235.7167 | 0.0032 | 0.1200 | 3.0930 | 232.1000 | 0.0031 | 0.1200 | 4.5760 | 143.2500 | 0.0156 | 0.1200 | 3.1590 | 237.5000 | 0.00721519 |
| 0.1300 | 3.2080 | 301.5333 | 0.0080 | 0.1300 | 3.1230 | 246.4000 | 0.0032 | 0.1300 | 3.1280 | 243.4833 | 0.0047 | 0.1300 | 4.6040 | 145.0500 | 0.0045 | 0.1300 | 3.2160 | 245.4000 | 0.00698592 |
| 0.1400 | 3.2860 | 311.2833 | 0.0078 | 0.1400 | 3.1680 | 260.4167 | 0.0031 | 0.1400 | 3.2840 | 277.0000 | 0.0075 | 0.1400 | 4.6420 | 153.4833 | 0.0115 | 0.1400 | 3.3400 | 263.1500 | 0.00729727 |
| 0.1500 | 3.4330 | 330.1000 | 0.0086 | 0.1500 | 3.2100 | 273.7667 | 0.0051 | 0.1500 | 3.3220 | 282.0667 | 0.0071 | 0.1500 | 4.7530 | 163.1000 | 0.0096 | 0.1500 | 3.4120 | 273.0167 | 0.00822785 |
| 0.1600 | 3.4870 | 336.3667 | 0.0077 | 0.1600 | 3.2340 | 278.4500 | 0.0023 | 0.1600 | 3.4070 | 294.0833 | 0.0073 | 0.1600 | 4.8690 | 175.1667 | 0.0089 | 0.1600 | 3.4770 | 280.9167 | 0.00721519 |
| 0.1700 | 3.5460 | 344.0167 | 0.0083 | 0.1700 | 3.2540 | 287.1333 | 0.0034 | 0.1700 | 3.4720 | 302.9333 | 0.0076 | 0.1700 | 4.9230 | 181.2333 | 0.0074 | 0.1700 | 3.5340 | 288.8167 | 0.00670058 |
| 0.1800 | 3.6210 | 353.0833 | 0.0074 | 0.1800 | 3.2630 | 289.8000 | 0.0032 | 0.1800 | 3.5250 | 309.8833 | 0.0087 | 0.1800 | 4.9590 | 186.0833 | 0.0033 | 0.1800 | 3.5780 | 295.3833 | 0.00823099 |
| 0.1900 | 3.6880 | 362.1333 | 0.0078 | 0.1900 | 3.2990 | 301.1500 | 0.0034 | 0.1900 | 3.5800 | 316.2167 | 0.0080 | 0.1900 | 4.9770 | 191.5333 | 0.0105 | 0.1900 | 3.6160 | 300.0000 | 0.00768 |
| 0.2000 | 3.8180 | 378.8500 | 0.0060 | 0.2000 | 3.3460 | 315.1667 | 0.0020 | 0.2000 | 3.6810 | 328.8667 | 0.0077 | 0.2000 | 5.1110 | 204.2667 | 0.0012 | 0.2000 | 3.7120 | 312.5000 | 0.00729727 |
| 0.2100 | 3.8470 | 383.7167 | 0.0071 | 0.2100 | 3.3660 | 325.1833 | 0.0030 | 0.2100 | 3.7590 | 338.9833 | 0.0081 | 0.2100 | 5.1140 | 206.6833 | 0.0119 | 0.2100 | 3.7840 | 322.3667 | 0.00736842 |
| 0.2200 | 3.9210 | 394.1667 | 0.0075 | 0.2200 | 3.3800 | 329.8667 | 0.0023 | 0.2200 | 3.8000 | 344.0333 | 0.0082 | 0.2200 | 5.1500 | 209.7167 | 0.0070 | 0.2200 | 3.8470 | 330.9167 | 0.00632911 |
| 0.2300 | 4.0040 | 405.3000 | 0.0081 | 0.2300 | 3.4000 | 338.5500 | 0.0032 | 0.2300 | 3.8620 | 351.6333 | 0.0076 | 0.2300 | 5.2650 | 226.0833 | 0.0381 | 0.2300 | 3.8970 | 338.8167 | 0.00987354 |
| 0.2400 | 4.0490 | 410.8833 | 0.0075 | 0.2400 | 3.4150 | 343.2167 | 0.0055 | 0.2400 | 3.9100 | 357.9500 | 0.0062 | 0.2400 | 5.3570 | 228.5000 | 0.0010 | 0.2400 | 3.9230 | 341.4500 | 0.00635948 |
| 0.2500 | 4.1060 | 418.5333 | 0.0062 | 0.2500 | 3.4740 | 353.9000 | 0.0066 | 0.2500 | 3.9490 | 364.2833 | 0.0069 | 0.2500 | 5.3600 | 231.5333 | 0.0005 | 0.2500 | 3.9690 | 348.6833 | 0.00677927 |
| 0.2600 | 4.2140 | 435.9500 | 0.0077 | 0.2600 | 3.5760 | 369.2667 | 0.0081 | 0.2600 | 4.0490 | 378.8167 | 0.0065 | 0.2600 | 5.3650 | 240.6333 | 0.0089 | 0.2600 | 4.0760 | 364.4667 | 0.00544304 |
| 0.2700 | 4.2620 | 442.2167 | 0.0060 | 0.2700 | 3.6190 | 374.6000 | 0.0062 | 0.2700 | 4.1400 | 392.7333 | 0.0114 | 0.2700 | 5.4510 | 250.3333 | 0.0007 | 0.2700 | 4.1190 | 372.3667 | 0.00641304 |
| 0.2800 | 4.3080 | 449.8833 | 0.0061 | 0.2800 | 3.6690 | 382.6167 | 0.0060 | 0.2800 | 4.1690 | 395.2667 | 0.0057 | 0.2800 | 5.4540 | 254.5667 | 0.0104 | 0.2800 | 4.1780 | 381.5667 | 0.00622788 |
| 0.2900 | 4.3590 | 458.2333 | 0.0069 | 0.2900 | 3.6810 | 384.6167 | 0.0070 | 0.2900 | 4.2120 | 402.8500 | 0.0059 | 0.2900 | 5.5420 | 263.0500 | -0.0014 | 0.2900 | 4.2190 | 388.1500 | 0.00608447 |
| 0.3000 | 4.4310 | 468.6833 | 0.0052 | 0.3000 | 3.7370 | 392.6333 | 0.0065 | 0.3000 | 4.2380 | 407.2833 | 0.0071 | 0.3000 | 5.5370 | 266.7000 | 0.0195 | 0.3000 | 4.2550 | 394.0667 | 0.00589891 |
| 0.3100 | 4.4490 | 472.1667 | 0.0061 | 0.3100 | 3.7980 | 401.9833 | 0.0068 | 0.3100 | 4.3050 | 416.7667 | 0.0066 | 0.3100 | 5.7970 | 280.0333 | 0.0023 | 0.3100 | 4.2900 | 400.0000 | 0.00523945 |
| 0.3200 | 4.5800 | 493.7500 | 0.0057 | 0.3200 | 3.8480 | 409.3333 | 0.0066 | 0.3200 | 4.4350 | 436.3667 | 0.0078 | 0.3200 | 5.8180 | 289.1167 | 0.0016 | 0.3200 | 4.3520 | 411.8333 | 0.00701754 |
| 0.3300 | 4.6200 | 500.7167 | 0.0054 | 0.3300 | 3.9630 | 426.6833 | 0.0069 | 0.3300 | 4.4940 | 443.9667 | 0.0005 | 0.3300 | 5.8230 | 292.1500 | 0.0049 | 0.3300 | 4.4120 | 420.3833 | 0.00572277 |
| 0.3400 | 4.6800 | 511.8500 | 0.0032 | 0.3400 | 4.0090 | 433.3667 | 0.0052 | 0.3400 | 4.5020 | 461.0333 | 0.0042 | 0.3400 | 5.8730 | 302.4500 | 0.0009 | 0.3400 | 4.4760 | 431.5667 | 0.00531648 |
| 0.3500 | 4.7160 | 523.0000 | 0.0069 | 0.3500 | 4.0580 | 442.7167 | 0.0076 | 0.3500 | 4.5530 | 473.0500 | 0.0039 | 0.3500 | 5.8770 | 306.7000 | 0.0015 | 0.3500 | 4.5110 | 438.1500 | 0.0048 |
| 0.3600 | 4.8030 | 535.5333 | 0.0048 | 0.3600 | 4.1240 | 451.4000 | 0.0062 | 0.3600 | 4.5800 | 480.0167 | 0.0025 | 0.3600 | 5.8850 | 312.1500 | 0.0036 | 0.3600 | 4.5710 | 450.6500 | 0.00783912 |
| 0.3700 | 4.8660 | 548.7667 | 0.0034 | 0.3700 | 4.1570 | 456.7333 | 0.0069 | 0.3700 | 4.6040 | 489.5000 | 0.0037 | 0.3700 | 5.8960 | 315.1833 | 0.0060 | 0.3700 | 4.5970 | 453.9667 | 0.00557748 |
| 0.3800 | 4.9420 | 571.0500 | -0.0010 | 0.3800 | 4.2900 | 476.1000 | 0.0063 | 0.3800 | 4.6950 | 514.1667 | 0.0010 | 0.3800 | 5.9360 | 321.8500 | 0.0014 | 0.3800 | 4.7290 | 477.6333 | 0.00457143 |

| | | | | | | | | | | | | | | | | | | | |
|---|---|---|---|---|---|---|---|---|---|---|---|---|---|---|---|---|---|---|---|
| 0.3900 | 4.9270 | 586.3667 | 0.0018 | 0.3900 | 4.3490 | 485.4500 | 0.0061 | 0.3900 | 4.7110 | 529.9667 | 0.0037 | 0.3900 | 5.9550 | 335.1833 | 0.0073 | 0.3900 | 4.7530 | 482.8833 | 0.00481013 |
| 0.4000 | 4.9520 | 600.3000 | 0.0082 | 0.4000 | 4.3900 | 492.1333 | 0.0058 | 0.4000 | 4.7530 | 541.3500 | 0.0034 | 0.4000 | 5.9860 | 339.4333 | -0.0044 | 0.4000 | 4.8100 | 494.7333 | 0.00227848 |
| | | | | 0.4100 | 4.4640 | 504.8167 | 0.0008 | 0.4100 | 4.7810 | 549.5833 | 0.0023 | 0.4100 | 5.9700 | 343.0667 | 0.1205 | 0.4100 | 4.8190 | 498.6833 | 0.0020908 |
| | | | | 0.4200 | 4.4850 | 532.1833 | -0.0056 | 0.4200 | 4.8000 | 557.8000 | 0.0034 | 0.4200 | 6.4800 | 347.3000 | 0.0093 | 0.4200 | 4.8520 | 514.4667 | 0.00037975 |
| | | | | 0.4300 | 4.4400 | 540.2000 | 0.0028 | 0.4300 | 4.8470 | 571.7167 | 0.0017 | 0.4300 | 6.5810 | 358.2167 | 0.0011 | 0.4300 | 4.8550 | 522.3667 | 0.0002765 |
| | | | | 0.4400 | 4.4900 | 558.2333 | 0.0032 | 0.4400 | 4.8640 | 581.8333 | 0.0026 | 0.4400 | 6.5850 | 361.8500 | 0.0032 | 0.4400 | 4.8590 | 536.8333 | 0.00192 |
| | | | | 0.4500 | 4.5220 | 568.2500 | 0.0041 | 0.4500 | 4.8840 | 589.4167 | 0.0021 | 0.4500 | 6.6420 | 379.4333 | 0.0014 | 0.4500 | 4.8830 | 549.3333 | 0.00052632 |
| | | | | 0.4600 | 4.6060 | 588.9500 | 0.0041 | 0.4600 | 4.9370 | 614.0833 | 0.0040 | 0.4600 | 6.6470 | 383.0667 | 0.0019 | 0.4600 | 4.8920 | 566.4333 | 0.00176652 |
| | | | | 0.4700 | 4.6580 | 601.6333 | 0.0077 | 0.4700 | 4.9870 | 626.7333 | 0.0080 | 0.4700 | 6.6550 | 387.3167 | 0.0172 | 0.4700 | 4.9780 | 615.1167 | 0.00809277 |

## Exp2_Peak Position of inflatable membrane:

| Sugar Concentration 21% | | | | | | Sugar Concentration 12% | | | | | | Sugar Concentration 3% | | | | | |
|---|---|---|---|---|---|---|---|---|---|---|---|---|---|---|---|---|---|
| T2 | | T5 | | T4 | | T2 | | T3 | | T4 | | T1 | | T2 | | T3 | |
| t (s) | Y (mm) | t (s) | Y (mm) | t (s) | Y (mm) | t (s) | Y (mm) | t (s) | Y (mm) | t (s) | Y (mm) | t (s) | Y (mm) | t (s) | Y (mm) | t (s) | Y (mm) |
| 0.00 | -0.02 | 0.00 | 0.00 | 0.00 | 0.00 | 0.00 | 0.00 | 0.00 | 0.00 | 0.00 | 0.00 | 0.00 | 0.00 | 0.00 | 0.00 | 0.00 | 0.00 |
| 0.07 | -0.25 | 0.08 | 4.49 | 0.17 | 0.11 | 0.08 | 0.19 | 0.08 | 0.12 | 0.08 | 0.09 | 0.17 | -0.14 | 0.08 | 0.02 | 0.17 | 0.06 |
| 0.15 | -0.08 | 0.17 | 4.45 | 0.33 | 0.19 | 0.16 | 0.19 | 0.16 | 0.19 | 0.16 | 0.08 | 0.33 | 0.04 | 0.16 | 0.07 | 0.33 | 0.05 |
| 0.24 | -0.31 | 0.25 | 4.61 | 0.50 | 0.18 | 0.25 | 0.21 | 0.25 | 0.02 | 0.25 | -0.11 | 0.50 | 0.01 | 0.24 | -0.04 | 0.50 | 0.10 |
| 0.32 | -0.08 | 0.33 | 4.59 | 0.67 | -0.01 | 0.33 | 0.22 | 0.33 | 0.01 | 0.33 | 0.11 | 0.67 | -0.14 | 0.33 | -0.03 | 0.67 | 0.13 |
| 0.40 | -0.31 | 0.42 | 4.55 | 0.83 | 0.22 | 0.41 | -0.04 | 0.41 | 0.21 | 0.41 | 0.11 | 0.83 | 0.04 | 0.41 | -0.07 | 0.83 | 0.10 |
| 0.49 | -0.21 | 0.50 | 1.89 | 1.00 | 0.06 | 0.50 | 0.24 | 0.50 | -0.03 | 0.50 | -0.13 | 1.00 | 0.00 | 0.49 | -0.07 | 1.00 | -0.02 |
| 0.57 | -0.17 | 0.58 | 2.77 | 1.17 | 0.14 | 0.58 | -0.05 | 0.58 | 0.28 | 0.58 | -0.13 | 1.17 | -0.14 | 0.58 | -0.03 | 1.17 | 0.15 |
| 0.65 | -0.19 | 0.67 | 3.30 | 1.33 | -0.01 | 0.66 | 0.28 | 0.66 | 0.03 | 0.66 | 0.16 | 1.33 | 0.01 | 0.66 | -0.10 | 1.33 | 0.09 |
| 0.74 | -0.20 | 0.75 | 4.54 | 1.50 | 0.02 | 0.75 | 0.03 | 0.75 | 0.20 | 0.75 | -0.14 | 1.50 | -0.03 | 0.74 | -0.01 | 1.50 | -0.01 |
| 0.82 | -0.18 | 0.83 | 4.58 | 1.67 | 0.17 | 0.83 | 0.26 | 0.83 | 0.00 | 0.83 | -0.10 | 1.67 | -0.13 | 0.83 | -0.09 | 1.67 | 0.00 |
| 0.90 | -0.19 | 0.92 | 4.52 | 1.83 | 0.20 | 0.91 | 0.22 | 0.91 | 0.18 | 0.91 | -0.05 | 1.83 | 0.03 | 0.91 | -0.02 | 1.83 | 0.08 |
| 0.99 | -0.14 | 1.00 | 4.53 | 2.00 | -0.04 | 1.00 | 0.20 | 1.00 | 0.08 | 1.00 | 0.05 | 2.00 | -0.09 | 0.99 | -0.03 | 2.00 | 0.00 |
| 1.07 | -0.16 | 1.08 | 4.68 | 2.17 | 0.01 | 1.08 | -0.08 | 1.08 | 0.14 | 1.08 | 0.04 | 2.17 | -0.14 | 1.08 | -0.03 | 2.17 | 0.17 |
| 1.15 | -0.16 | 1.17 | 4.47 | 2.33 | 0.20 | 1.16 | -0.04 | 1.16 | 0.01 | 1.16 | -0.07 | 2.33 | 0.01 | 1.16 | -0.01 | 2.33 | 0.09 |
| 1.24 | -0.17 | 1.25 | 4.66 | 2.50 | 0.20 | 1.25 | 0.25 | 1.25 | 0.08 | 1.25 | 0.06 | 2.50 | -0.11 | 1.24 | 0.04 | 2.50 | 0.01 |
| 1.32 | -0.17 | 1.33 | 4.48 | 2.67 | -0.03 | 1.33 | 0.25 | 1.33 | 0.11 | 1.33 | 0.01 | 2.67 | -0.14 | 1.33 | 0.01 | 2.67 | 0.02 |
| 1.40 | -0.16 | 1.42 | 4.55 | 2.83 | 0.18 | 1.41 | -0.06 | 1.41 | 0.16 | 1.41 | 0.05 | 2.83 | 0.03 | 1.41 | -0.06 | 2.83 | 0.06 |
| 1.49 | -0.16 | 1.50 | 4.53 | 3.00 | -0.02 | 1.50 | 0.28 | 1.50 | 0.12 | 1.50 | -0.09 | 3.00 | -0.08 | 1.49 | -0.03 | 3.00 | 0.15 |
| 1.57 | -0.15 | 1.58 | 4.55 | 3.17 | 0.00 | 1.58 | 0.00 | 1.58 | 0.01 | 1.58 | -0.11 | 3.17 | -0.14 | 1.58 | 0.00 | 3.17 | 0.04 |
| 1.65 | -0.15 | 1.67 | 4.49 | 3.33 | 0.19 | 1.66 | 0.25 | 1.66 | 0.01 | 1.66 | 0.11 | 3.33 | 0.00 | 1.66 | 0.01 | 3.33 | 0.09 |
| 1.74 | -0.15 | 1.75 | 4.68 | 3.50 | -0.01 | 1.75 | -0.02 | 1.75 | 0.11 | 1.75 | -0.10 | 3.50 | -0.11 | 1.74 | 0.02 | 3.50 | -0.01 |
| 1.82 | -0.15 | 1.83 | 4.43 | 3.67 | 0.21 | 1.83 | 0.25 | 1.83 | 0.11 | 1.83 | -0.03 | 3.67 | -0.15 | 1.83 | 0.03 | 3.67 | 0.06 |
| 1.90 | -0.13 | 1.92 | 4.47 | 3.83 | 0.18 | 1.91 | 0.25 | 1.91 | 0.21 | 1.91 | 0.05 | 3.83 | 0.01 | 1.91 | -0.04 | 3.83 | 0.11 |
| 1.99 | -0.13 | 2.00 | 4.65 | 4.00 | -0.02 | 2.00 | 0.21 | 2.00 | 0.22 | 2.00 | 0.08 | 4.00 | -0.14 | 1.99 | -0.04 | 4.00 | 0.02 |
| 2.07 | -0.15 | 2.08 | 4.48 | 4.17 | 0.21 | 2.08 | 0.11 | 2.08 | 0.23 | 2.08 | -0.12 | 4.17 | -0.14 | 2.08 | 0.03 | 4.17 | 0.07 |
| 2.15 | -0.13 | 2.17 | 4.50 | 4.33 | -0.03 | 2.16 | 0.16 | 2.16 | 0.09 | 2.16 | 0.07 | 4.33 | 0.02 | 2.16 | 0.05 | 4.33 | 0.15 |
| 2.24 | -0.13 | 2.25 | 4.66 | 4.50 | 0.16 | 2.25 | 0.25 | 2.25 | 0.10 | 2.25 | -0.10 | 4.50 | -0.06 | 2.24 | 0.07 | 4.50 | 0.07 |
| 2.32 | -0.15 | 2.33 | 4.46 | 4.67 | -0.04 | 2.33 | 0.05 | 2.33 | 0.14 | 2.33 | -0.02 | 4.67 | -0.12 | 2.33 | 0.05 | 4.67 | 0.07 |
| 2.40 | -0.14 | 2.42 | 4.52 | 4.83 | 0.19 | 2.41 | 0.01 | 2.41 | 0.27 | 2.41 | 0.04 | 4.83 | 0.02 | 2.41 | -0.01 | 4.83 | 0.19 |
| 2.49 | -0.16 | 2.50 | 4.52 | 5.00 | 0.14 | 2.50 | -0.01 | 2.50 | 0.29 | 2.50 | 0.10 | 5.00 | -0.12 | 2.49 | -0.02 | 5.00 | 0.17 |
| 2.57 | -0.11 | 2.58 | 4.61 | 5.17 | -0.07 | 2.58 | 0.28 | 2.58 | 0.26 | 2.58 | -0.14 | 5.17 | -0.11 | 2.58 | -0.01 | 5.17 | 0.11 |
| 2.65 | -0.13 | 2.67 | 4.49 | 5.33 | -0.02 | 2.66 | 0.02 | 2.66 | 0.25 | 2.66 | 0.08 | 5.33 | -0.01 | 2.66 | 0.03 | 5.33 | 0.20 |
| 2.74 | -0.13 | 2.75 | 4.51 | 5.50 | 0.06 | 2.75 | 0.21 | 2.75 | 0.28 | 2.75 | -0.07 | 5.50 | -0.12 | 2.74 | 0.05 | 5.50 | 0.21 |
| 2.82 | -0.12 | 2.83 | 4.65 | 5.67 | 0.24 | 2.83 | 0.25 | 2.83 | 0.19 | 2.83 | 0.01 | 5.67 | -0.09 | 2.83 | 0.04 | 5.67 | 0.14 |
| 2.90 | -0.13 | 2.92 | 4.55 | 5.83 | 0.02 | 2.91 | 0.15 | 2.91 | 0.22 | 2.91 | -0.06 | 5.83 | -0.03 | 2.91 | -0.02 | 5.83 | 0.22 |

| | | | | | | | | | | | | | | | | | |
|---|---|---|---|---|---|---|---|---|---|---|---|---|---|---|---|---|---|
| 2.99 | -0.12 | 3.00 | 4.56 | 6.00 | 0.10 | 3.00 | 0.20 | 3.00 | 0.22 | 3.00 | 0.12 | 6.00 | -0.15 | 2.99 | 0.00 | 6.00 | 0.21 |
| 3.07 | -0.12 | 3.08 | 4.58 | 6.17 | 0.05 | 3.08 | 0.29 | 3.08 | 0.29 | 3.08 | 0.11 | 6.17 | -0.07 | 3.08 | -0.01 | 6.17 | 0.21 |
| 3.15 | -0.12 | 3.17 | 4.60 | 6.33 | 0.00 | 3.16 | 0.27 | 3.16 | 0.26 | 3.16 | -0.07 | 6.33 | 0.00 | 3.16 | 0.06 | 6.33 | 0.26 |
| 3.24 | -0.14 | 3.25 | 4.58 | 6.50 | 0.18 | 3.25 | 0.40 | 3.25 | 0.26 | 3.25 | 0.09 | 6.50 | -0.14 | 3.24 | 0.07 | 6.50 | 0.32 |
| 3.32 | -0.13 | 3.33 | 4.68 | 6.67 | 0.03 | 3.33 | 0.43 | 3.33 | 0.23 | 3.33 | 0.04 | 6.67 | -0.03 | 3.33 | 0.10 | 6.67 | 0.27 |
| 3.40 | -0.12 | 3.42 | 4.71 | 6.83 | 0.02 | 3.41 | 0.48 | 3.41 | 0.22 | 3.41 | 0.01 | 6.83 | -0.02 | 3.41 | 0.01 | 6.83 | 0.32 |
| 3.49 | -0.13 | 3.50 | 4.63 | 7.00 | 0.17 | 3.50 | 0.47 | 3.50 | 0.24 | 3.50 | -0.13 | 7.00 | -0.15 | 3.49 | -0.01 | 7.00 | 0.33 |
| 3.57 | -0.14 | 3.58 | 4.74 | 7.17 | 0.02 | 3.58 | 0.47 | 3.58 | 0.25 | 3.58 | -0.12 | 7.17 | -0.03 | 3.58 | 0.12 | 7.17 | 0.29 |
| 3.65 | -0.13 | 3.67 | 4.83 | 7.33 | 0.20 | 3.66 | 0.34 | 3.66 | 0.27 | 3.66 | 0.06 | 7.33 | 0.02 | 3.66 | 0.13 | 7.33 | 0.32 |
| 3.74 | -0.13 | 3.75 | 4.67 | 7.50 | 0.16 | 3.75 | 0.32 | 3.75 | 0.28 | 3.75 | -0.10 | 7.50 | -0.14 | 3.74 | 0.03 | 7.50 | 0.35 |
| 3.82 | -0.13 | 3.83 | 4.80 | 7.67 | 0.18 | 3.83 | 0.58 | 3.83 | 0.26 | 3.83 | -0.01 | 7.67 | 0.05 | 3.83 | 0.13 | 7.67 | 0.35 |
| 3.90 | -0.12 | 3.92 | 4.65 | 7.83 | 0.23 | 3.91 | 0.29 | 3.91 | 0.28 | 3.91 | -0.06 | 7.83 | -0.01 | 3.91 | 0.02 | 7.83 | 0.37 |
| 3.99 | -0.11 | 4.00 | 4.83 | 8.00 | 0.14 | 4.00 | 0.33 | 4.00 | 0.29 | 4.00 | 0.07 | 8.00 | -0.15 | 3.99 | 0.03 | 8.00 | 0.38 |
| 4.07 | -0.10 | 4.08 | 4.62 | 8.17 | 0.14 | 4.08 | 0.34 | 4.08 | 0.39 | 4.08 | 0.07 | 8.17 | 0.00 | 4.08 | 0.09 | 8.17 | 0.42 |
| 4.15 | -0.11 | 4.17 | 4.83 | 8.33 | 0.26 | 4.16 | 0.64 | 4.16 | 0.44 | 4.16 | 0.05 | 8.33 | 0.01 | 4.16 | 0.05 | 8.33 | 0.47 |
| 4.24 | -0.13 | 4.25 | 4.63 | 8.50 | 0.07 | 4.25 | 0.58 | 4.25 | 0.45 | 4.25 | -0.08 | 8.50 | -0.14 | 4.24 | 0.13 | 8.50 | 0.44 |
| 4.32 | -0.08 | 4.33 | 4.67 | 8.67 | 0.05 | 4.33 | 0.28 | 4.33 | 0.39 | 4.33 | -0.02 | 8.67 | 0.00 | 4.33 | 0.03 | 8.67 | 0.41 |
| 4.40 | -0.07 | 4.42 | 4.83 | 8.83 | 0.23 | 4.41 | 0.58 | 4.41 | 0.48 | 4.41 | -0.04 | 8.83 | -0.07 | 4.41 | 0.03 | 8.83 | 0.47 |
| 4.49 | -0.06 | 4.50 | 4.63 | 9.00 | 0.20 | 4.50 | 0.28 | 4.50 | 0.34 | 4.50 | -0.05 | 9.00 | -0.15 | 4.49 | 0.04 | 9.00 | 0.46 |
| 4.57 | -0.04 | 4.58 | 4.83 | 9.17 | 0.23 | 4.58 | 0.33 | 4.58 | 0.49 | 4.58 | 0.06 | 9.17 | -0.01 | 4.58 | 0.07 | 9.17 | 0.44 |
| 4.65 | -0.06 | 4.67 | 4.63 | 9.33 | 0.21 | 4.66 | 0.60 | 4.66 | 0.40 | 4.66 | 0.05 | 9.33 | -0.08 | 4.66 | 0.07 | 9.33 | 0.47 |
| 4.74 | -0.06 | 4.75 | 4.81 | 9.50 | 0.22 | 4.75 | 0.28 | 4.75 | 0.48 | 4.75 | -0.06 | 9.50 | -0.14 | 4.74 | -0.02 | 9.50 | 0.46 |
| 4.82 | -0.05 | 4.83 | 4.65 | 9.67 | 0.30 | 4.83 | 0.60 | 4.83 | 0.45 | 4.83 | -0.06 | 9.67 | 0.00 | 4.83 | 0.03 | 9.67 | 0.44 |
| 4.90 | 0.00 | 4.92 | 4.82 | 9.83 | 0.26 | 4.91 | 0.31 | 4.91 | 0.45 | 4.91 | -0.07 | 9.83 | -0.09 | 4.91 | 0.01 | 9.83 | 0.47 |
| 4.99 | 0.00 | 5.00 | 4.62 | 10.00 | 0.28 | 5.00 | 0.59 | 5.00 | 0.39 | 5.00 | -0.07 | 10.00 | -0.14 | 4.99 | 0.07 | 10.00 | 0.53 |
| 5.07 | 0.00 | 5.08 | 4.82 | 10.17 | 0.26 | 5.08 | 0.27 | 5.08 | 0.41 | 5.08 | 0.05 | 10.17 | 0.02 | 5.08 | 0.09 | 10.17 | 0.47 |
| 5.15 | 0.00 | 5.17 | 4.65 | 10.33 | 0.33 | 5.16 | 0.51 | 5.16 | 0.42 | 5.16 | 0.05 | 10.33 | -0.01 | 5.16 | 0.10 | 10.33 | 0.48 |
| 5.24 | -0.03 | 5.25 | 4.66 | 10.50 | 0.32 | 5.25 | 0.31 | 5.25 | 0.43 | 5.25 | 0.09 | 10.50 | -0.14 | 5.24 | 0.06 | 10.50 | 0.54 |
| 5.32 | -0.04 | 5.33 | 4.83 | 10.67 | 0.43 | 5.33 | 0.55 | 5.33 | 0.54 | 5.33 | -0.10 | 10.67 | 0.01 | 5.33 | 0.05 | 10.67 | 0.51 |
| 5.40 | 0.01 | 5.42 | 4.62 | 10.83 | 0.44 | 5.41 | 0.31 | 5.41 | 0.51 | 5.41 | -0.10 | 10.83 | -0.16 | 5.41 | 0.04 | 10.83 | 0.53 |
| 5.49 | 0.01 | 5.50 | 4.81 | 11.00 | 0.30 | 5.50 | 0.57 | 5.50 | 0.48 | 5.50 | -0.06 | 11.00 | -0.15 | 5.49 | 0.05 | 11.00 | 0.59 |
| 5.57 | 0.00 | 5.58 | 4.67 | 11.17 | 0.47 | 5.58 | 0.27 | 5.58 | 0.46 | 5.58 | -0.04 | 11.17 | 0.02 | 5.58 | 0.10 | 11.17 | 0.57 |
| 5.65 | 0.00 | 5.67 | 4.65 | 11.33 | 0.27 | 5.66 | 0.50 | 5.66 | 0.48 | 5.66 | 0.04 | 11.33 | -0.14 | 5.66 | 0.06 | 11.33 | 0.57 |
| 5.74 | 0.03 | 5.75 | 4.86 | 11.50 | 0.45 | 5.75 | 0.30 | 5.75 | 0.56 | 5.75 | 0.09 | 11.50 | -0.14 | 5.74 | 0.04 | 11.50 | 0.66 |
| 5.82 | -0.01 | 5.83 | 4.62 | 11.67 | 0.36 | 5.83 | 0.32 | 5.83 | 0.57 | 5.83 | -0.10 | 11.67 | 0.00 | 5.83 | 0.04 | 11.67 | 0.65 |
| 5.90 | -0.01 | 5.92 | 4.63 | 11.83 | 0.28 | 5.91 | 0.57 | 5.91 | 0.58 | 5.91 | -0.08 | 11.83 | -0.15 | 5.91 | 0.03 | 11.83 | 0.64 |
| 5.99 | 1.14 | 6.00 | 4.87 | 12.00 | 0.45 | 6.00 | 0.31 | 6.00 | 0.58 | 6.00 | -0.05 | 12.00 | -0.11 | 5.99 | 0.07 | 12.00 | 0.70 |
| 6.07 | 0.60 | 6.08 | 4.65 | 12.17 | 0.33 | 6.08 | 0.58 | 6.08 | 0.57 | 6.08 | -0.03 | 12.17 | 0.00 | 6.08 | 0.06 | 12.17 | 0.73 |
| 6.15 | 0.03 | 6.17 | 4.83 | 12.33 | 0.30 | 6.16 | 0.29 | 6.16 | 0.53 | 6.16 | -0.05 | 12.33 | 0.02 | 6.16 | 0.09 | 12.33 | 0.71 |
| 6.24 | 0.03 | 6.25 | 4.64 | 12.50 | 0.46 | 6.25 | 0.31 | 6.25 | 0.55 | 6.25 | 0.09 | 12.50 | -0.13 | 6.24 | 0.02 | 12.50 | 0.72 |
| 6.32 | 0.01 | 6.33 | 4.81 | 12.67 | 0.30 | 6.33 | 0.61 | 6.33 | 0.54 | 6.33 | 0.12 | 12.67 | -0.04 | 6.33 | 0.01 | 12.67 | 0.73 |
| 6.40 | -0.03 | 6.42 | 4.61 | 12.83 | 0.49 | 6.41 | 0.29 | 6.41 | 0.46 | 6.41 | -0.08 | 12.83 | -0.16 | 6.41 | 0.02 | 12.83 | 0.71 |
| 6.49 | 0.00 | 6.50 | 4.63 | 13.00 | 0.26 | 6.50 | 0.61 | 6.50 | 0.32 | 6.50 | -0.04 | 13.00 | -0.13 | 6.49 | 0.11 | 13.00 | 0.73 |
| 6.57 | 0.05 | 6.58 | 4.70 | 13.17 | 0.30 | 6.58 | 0.31 | 6.58 | 0.29 | 6.58 | 0.06 | 13.17 | -0.11 | 6.58 | 0.10 | 13.17 | 0.73 |
| 6.65 | 0.03 | 6.67 | 4.85 | 13.33 | 0.50 | 6.66 | 0.60 | 6.66 | 0.43 | 6.66 | -0.10 | 13.33 | -0.16 | 6.66 | 0.06 | 13.33 | 0.72 |
| 6.74 | 0.01 | 6.75 | 4.65 | 13.50 | 0.27 | 6.75 | 0.27 | 6.75 | 0.42 | 6.75 | -0.09 | 13.50 | -0.02 | 6.74 | 0.04 | 13.50 | 0.72 |
| 6.82 | 0.01 | 6.83 | 4.83 | 13.67 | 0.30 | 6.83 | 0.34 | 6.83 | 0.42 | 6.83 | 0.07 | 13.67 | 0.00 | 6.83 | 0.03 | 13.67 | 0.72 |
| 6.90 | 0.00 | 6.92 | 4.71 | 13.83 | 0.47 | 6.91 | 0.56 | 6.91 | 0.47 | 6.91 | -0.09 | 13.83 | -0.15 | 6.91 | 0.10 | 13.83 | 0.81 |
| 6.99 | 0.52 | 7.00 | 4.69 | 14.00 | 0.27 | 7.00 | 0.30 | 7.00 | 0.51 | 7.00 | 0.07 | 14.00 | 0.01 | 6.99 | 0.11 | 14.00 | 0.83 |
| 7.07 | -0.01 | 7.08 | 4.69 | 14.17 | 0.30 | 7.08 | 0.59 | 7.08 | 0.44 | 7.08 | 0.03 | 14.17 | -0.01 | 7.08 | 0.04 | 14.17 | 0.81 |
| 7.15 | 1.58 | 7.17 | 4.66 | 14.33 | 0.47 | 7.16 | 0.30 | 7.16 | 0.55 | 7.16 | -0.05 | 14.33 | 0.00 | 7.16 | 0.07 | 14.33 | 0.83 |

| | | | | | | | | | | | | | | | | | |
|---|---|---|---|---|---|---|---|---|---|---|---|---|---|---|---|---|---|
| 7.24 | 1.29 | 7.25 | 4.86 | 14.50 | 0.31 | 7.25 | 0.58 | 7.25 | 0.53 | 7.25 | -0.07 | 14.50 | 0.01 | 7.24 | 0.03 | 14.50 | 0.94 |
| 7.32 | -0.25 | 7.33 | 4.61 | 14.67 | 0.29 | 7.33 | 0.27 | 7.33 | 0.45 | 7.33 | 0.07 | 14.67 | 0.02 | 7.33 | 0.03 | 14.67 | 0.93 |
| 7.40 | 1.15 | 7.42 | 4.88 | 14.83 | 0.46 | 7.41 | 0.35 | 7.41 | 0.55 | 7.41 | -0.08 | 14.83 | -0.15 | 7.41 | 0.08 | 14.83 | 0.95 |
| 7.49 | 1.12 | 7.50 | 4.65 | 15.00 | 0.32 | 7.50 | 0.56 | 7.50 | 0.64 | 7.50 | 0.00 | 15.00 | 0.02 | 7.49 | 0.08 | 15.00 | 0.98 |
| 7.57 | 1.22 | 7.58 | 4.63 | 15.17 | 0.31 | 7.58 | 0.31 | 7.58 | 0.67 | 7.58 | -0.05 | 15.17 | 0.01 | 7.58 | 0.11 | 15.17 | 0.97 |
| 7.65 | 1.08 | 7.67 | 4.66 | 15.33 | 0.49 | 7.66 | 0.58 | 7.66 | 0.81 | 7.66 | 0.08 | 15.33 | -0.14 | 7.66 | 0.12 | 15.33 | 0.96 |
| 7.74 | 1.08 | 7.75 | 4.72 | 15.50 | 0.31 | 7.75 | 0.31 | 7.75 | 0.87 | 7.75 | -0.08 | 15.50 | 0.00 | 7.74 | 0.03 | 15.50 | 1.10 |
| 7.82 | 1.28 | 7.83 | 4.70 | 15.67 | 0.32 | 7.83 | 0.58 | 7.83 | 0.86 | 7.83 | -0.07 | 15.67 | 0.00 | 7.83 | 0.03 | 15.67 | 1.09 |
| 7.90 | 1.04 | 7.92 | 4.67 | 15.83 | 0.45 | 7.91 | 0.39 | 7.91 | 0.88 | 7.91 | 0.04 | 15.83 | -0.13 | 7.91 | 0.09 | 15.83 | 1.08 |
| 7.99 | 1.22 | 8.00 | 4.84 | 16.00 | 0.32 | 8.00 | 0.31 | 8.00 | 0.89 | 8.00 | 0.00 | 16.00 | -0.15 | 7.99 | 0.08 | 16.00 | 1.11 |
| 8.07 | 1.09 | 8.08 | 4.65 | 16.17 | 0.30 | 8.08 | 0.60 | 8.08 | 0.90 | 8.08 | -0.01 | 16.17 | -0.03 | 8.08 | 0.05 | 16.17 | 1.13 |
| 8.15 | 1.27 | 8.17 | 4.66 | 16.33 | 0.49 | 8.16 | 0.28 | 8.16 | 0.90 | 8.16 | 0.06 | 16.33 | -0.06 | 8.16 | 0.07 | 16.33 | 1.13 |
| 8.24 | 1.06 | 8.25 | 4.74 | 16.50 | 0.31 | 8.25 | 0.59 | 8.25 | 0.90 | 8.25 | -0.07 | 16.50 | 0.04 | 8.24 | 0.02 | 16.50 | 1.22 |
| 8.32 | 1.22 | 8.33 | 4.90 | 16.67 | 0.38 | 8.33 | 0.28 | 8.33 | 0.91 | 8.33 | 0.04 | 16.67 | -0.08 | 8.33 | 0.04 | 16.67 | 1.24 |
| 8.40 | 1.08 | 8.42 | 4.96 | 16.83 | 0.43 | 8.41 | 0.31 | 8.41 | 0.89 | 8.41 | -0.04 | 16.83 | -0.14 | 8.41 | 0.11 | 16.83 | 1.23 |
| 8.49 | 1.27 | 8.50 | 4.97 | 17.00 | 0.36 | 8.50 | 0.35 | 8.50 | 0.89 | 8.50 | 0.02 | 17.00 | -0.01 | 8.49 | 0.09 | 17.00 | 1.25 |
| 8.57 | 1.04 | 8.58 | 4.81 | 17.17 | 0.44 | 8.58 | 0.60 | 8.58 | 0.91 | 8.58 | -0.01 | 17.17 | 0.00 | 8.58 | 0.08 | 17.17 | 1.26 |
| 8.65 | 1.22 | 8.67 | 4.77 | 17.33 | 0.47 | 8.66 | 0.31 | 8.66 | 0.92 | 8.66 | -0.05 | 17.33 | -0.14 | 8.66 | 0.08 | 17.33 | 1.26 |
| 8.74 | 1.08 | 8.75 | 4.77 | 17.50 | 0.50 | 8.75 | 0.61 | 8.75 | 0.92 | 8.75 | -0.06 | 17.50 | 0.02 | 8.74 | 0.11 | 17.50 | 1.26 |
| 8.82 | 1.26 | 8.83 | 4.89 | 17.67 | 0.48 | 8.83 | 0.29 | 8.83 | 0.93 | 8.83 | 0.01 | 17.67 | -0.07 | 8.83 | 0.10 | 17.67 | 1.27 |
| 8.90 | 1.03 | 8.92 | 4.80 | 17.83 | 0.55 | 8.91 | 0.61 | 8.91 | 0.92 | 8.91 | -0.07 | 17.83 | -0.13 | 8.91 | 0.16 | 17.83 | 1.29 |
| 8.99 | 1.22 | 9.00 | 4.78 | 18.00 | 0.57 | 9.00 | 0.26 | 9.00 | 1.19 | 9.00 | 0.05 | 18.00 | -0.13 | 8.99 | 0.13 | 18.00 | 1.28 |
| 9.07 | 1.23 | 9.08 | 5.07 | 18.17 | 0.49 | 9.08 | 0.35 | 9.66 | 1.86 | 9.08 | 0.03 | 18.17 | -0.15 | 9.08 | 0.10 | 18.17 | 1.36 |
| 9.15 | 1.09 | 9.17 | 4.86 | 18.33 | 0.52 | 9.16 | 0.56 | 9.75 | 2.32 | 9.16 | 0.02 | 18.33 | -0.06 | 9.16 | 0.09 | 18.33 | 1.38 |
| 9.24 | 1.25 | 9.25 | 4.86 | 18.50 | 0.52 | 9.25 | 0.32 | 9.83 | 2.83 | 9.25 | 0.01 | 18.50 | 0.02 | 9.24 | 0.12 | 18.50 | 1.41 |
| 9.32 | 1.04 | 9.33 | 4.91 | 18.67 | 0.52 | 9.33 | 0.59 | 9.91 | 3.20 | 9.33 | -0.04 | 18.67 | -0.10 | 9.33 | 0.09 | 18.67 | 1.40 |
| 9.40 | 1.12 | 9.42 | 4.88 | 18.83 | 0.56 | 9.41 | 0.29 | 10.00 | 3.06 | 9.41 | 0.00 | 18.83 | -0.13 | 9.41 | 0.13 | 18.83 | 1.42 |
| 9.49 | 1.25 | 9.50 | 4.86 | 19.00 | 0.63 | 9.50 | 0.58 | 10.08 | 3.40 | 9.50 | 0.05 | 19.00 | 0.00 | 9.49 | 0.18 | 19.00 | 1.50 |
| 9.57 | 1.08 | 9.58 | 4.86 | 19.17 | 0.65 | 9.58 | 0.28 | 10.16 | 3.57 | 9.58 | 0.09 | 19.17 | 0.02 | 9.58 | 0.12 | 19.17 | 1.51 |
| 9.65 | 1.26 | 9.67 | 4.88 | 19.33 | 0.71 | 9.66 | 0.41 | 10.25 | 4.07 | 9.66 | 0.11 | 19.33 | -0.14 | 9.66 | 0.10 | 19.33 | 1.52 |
| 9.74 | 1.07 | 9.75 | 5.05 | 19.50 | 0.64 | 9.75 | 0.34 | 10.33 | 4.81 | 9.75 | 0.08 | 19.50 | 0.00 | 9.74 | 0.14 | 19.50 | 1.55 |
| 9.82 | 1.22 | 9.83 | 4.84 | 19.67 | 0.71 | 9.83 | 0.61 | 10.41 | 5.66 | 9.83 | 0.15 | 19.67 | -0.13 | 9.83 | 0.15 | 19.67 | 1.55 |
| 9.90 | 1.24 | 9.92 | 4.87 | 19.83 | 0.67 | 9.91 | 0.31 | 10.50 | 5.74 | 9.91 | -0.02 | 19.83 | -0.11 | 9.91 | 0.16 | 19.83 | 1.57 |
| 9.99 | 1.09 | 10.00 | 4.87 | 20.00 | 0.67 | 10.00 | 0.61 | 10.58 | 5.74 | 10.00 | 0.06 | 20.00 | -0.12 | 9.99 | 0.20 | 20.00 | 1.64 |
| 10.07 | 1.27 | 10.08 | 4.88 | 20.17 | 0.72 | 10.08 | 0.29 | 10.66 | 5.74 | 10.08 | 0.13 | 20.17 | -0.15 | 10.08 | 0.14 | 20.17 | 1.67 |
| 10.15 | 1.06 | 10.17 | 4.96 | 20.33 | 0.64 | 10.16 | 0.37 | 10.75 | 5.23 | 10.16 | 0.16 | 20.33 | -0.17 | 10.16 | 0.12 | 20.33 | 1.68 |
| 10.24 | 1.20 | 10.25 | 4.91 | 20.50 | 0.63 | 10.25 | 0.40 | 10.83 | 4.81 | 10.25 | 0.08 | 20.50 | -0.01 | 10.24 | 0.16 | 20.50 | 1.69 |
| 10.32 | 1.21 | 10.33 | 4.87 | 20.67 | 0.64 | 10.33 | 0.58 | 10.91 | 5.40 | 10.33 | -0.01 | 20.67 | -0.12 | 10.33 | 0.11 | 20.67 | 1.77 |
| 10.40 | 1.24 | 10.42 | 4.86 | 20.83 | 0.71 | 10.41 | 0.34 | 11.00 | 5.74 | 10.41 | -0.04 | 20.83 | -0.11 | 10.41 | 0.12 | 20.83 | 1.76 |
| 10.49 | 1.10 | 10.50 | 4.90 | 21.00 | 0.63 | 10.50 | 0.61 | 11.08 | 5.74 | 10.50 | -0.04 | 21.00 | 0.00 | 10.49 | 0.12 | 21.00 | 1.81 |
| 10.57 | 1.23 | 10.58 | 4.90 | 21.17 | 0.63 | 10.58 | 0.56 | 11.16 | 4.64 | 10.58 | -0.04 | 21.17 | -0.01 | 10.58 | 0.13 | 21.17 | 1.91 |
| 10.65 | 1.09 | 10.67 | 4.95 | 21.33 | 0.68 | 10.66 | 0.38 | 11.25 | 4.64 | 10.66 | -0.03 | 21.33 | -0.05 | 10.66 | 0.14 | 21.33 | 1.90 |
| 10.74 | 1.26 | 10.75 | 5.00 | 21.50 | 0.63 | 10.75 | 0.48 | 11.33 | 4.64 | 10.75 | 0.02 | 21.50 | 0.01 | 10.74 | 0.19 | 21.50 | 1.91 |
| 10.82 | 1.06 | 10.83 | 5.00 | 21.67 | 0.65 | 10.83 | 0.31 | 12.33 | 5.57 | 10.83 | -0.05 | 21.67 | -0.03 | 10.83 | 0.19 | 21.67 | 2.04 |
| 10.90 | 1.22 | 10.92 | 5.08 | 21.83 | 0.66 | 10.91 | 0.31 | 12.41 | 5.46 | 10.91 | -0.06 | 21.83 | -0.05 | 10.91 | 0.16 | 21.83 | 2.05 |
| 10.99 | 1.24 | 11.00 | 5.12 | 22.00 | 0.74 | 11.00 | 0.36 | 12.50 | 5.45 | 11.00 | -0.04 | 22.00 | -0.05 | 10.99 | 0.18 | 22.00 | 2.06 |
| 11.07 | 1.11 | 11.08 | 5.06 | 22.17 | 0.65 | 11.08 | 0.61 | 12.58 | 5.42 | 11.08 | -0.04 | 22.17 | -0.15 | 11.08 | 0.15 | 22.17 | 2.11 |
| 11.15 | 1.26 | 11.17 | 5.17 | 22.33 | 0.69 | 11.16 | 0.32 | 12.66 | 5.51 | 11.16 | 0.02 | 22.33 | -0.14 | 11.16 | 0.16 | 22.33 | 2.17 |
| 11.24 | 1.09 | 11.25 | 5.02 | 22.50 | 0.63 | 11.25 | 0.59 | 12.75 | 5.35 | 11.25 | 0.03 | 22.50 | 0.02 | 11.24 | 0.21 | 22.50 | 2.18 |
| 11.32 | 1.25 | 11.33 | 5.02 | 22.67 | 0.68 | 11.33 | 0.46 | 12.83 | 5.36 | 11.33 | -0.04 | 22.67 | -0.15 | 11.33 | 0.18 | 22.67 | 2.20 |
| 11.40 | 1.08 | 11.42 | 5.18 | 22.83 | 0.78 | 11.41 | 0.40 | 12.91 | 5.62 | 11.41 | -0.04 | 22.83 | -0.14 | 11.41 | 0.13 | 22.83 | 2.24 |

| | | | | | | | | | | | | | | | | | |
|---|---|---|---|---|---|---|---|---|---|---|---|---|---|---|---|---|---|
| 11.49 | 1.25 | 11.50 | 5.07 | 23.00 | 0.72 | 11.50 | 0.37 | 13.00 | 5.32 | 11.50 | 0.05 | 23.00 | 0.01 | 11.49 | 0.08 | 23.00 | 2.24 |
| 11.57 | 1.08 | 11.58 | 5.06 | 23.17 | 0.82 | 11.58 | 0.34 | 13.08 | 5.45 | 11.58 | -0.06 | 23.17 | 0.01 | 11.58 | 0.03 | 23.17 | 2.24 |
| 11.65 | 1.11 | 11.67 | 5.17 | 23.33 | 0.91 | 11.66 | 0.38 | 13.16 | 5.49 | 11.66 | 0.04 | 23.33 | -0.07 | 11.66 | 0.09 | 23.33 | 2.33 |
| 11.74 | 1.25 | 11.75 | 5.01 | 23.50 | 0.79 | 11.75 | 0.62 | 13.25 | 5.53 | 11.75 | 0.09 | 23.50 | 0.00 | 11.74 | 0.16 | 23.50 | 2.33 |
| 11.82 | 1.09 | 11.83 | 5.17 | 23.67 | 0.83 | 11.83 | 0.35 | 13.33 | 5.37 | 11.83 | 0.08 | 23.67 | 0.02 | 11.83 | 0.10 | 23.67 | 2.37 |
| 11.90 | 1.27 | 11.92 | 5.01 | 23.83 | 0.72 | 11.91 | 0.41 | 13.41 | 5.46 | 11.91 | -0.01 | 23.83 | -0.03 | 11.91 | 0.08 | 23.83 | 2.39 |
| 11.99 | 1.10 | 12.00 | 5.03 | 24.00 | 0.81 | 12.00 | 0.40 | 13.50 | 5.45 | 12.00 | -0.05 | 24.00 | -0.02 | 11.99 | 0.08 | 24.00 | 2.46 |
| 12.07 | 1.26 | 12.08 | 5.17 | 24.17 | 0.85 | 12.08 | 0.56 | 13.58 | 5.49 | 12.08 | -0.05 | 24.17 | -0.14 | 12.08 | 0.13 | 24.17 | 2.61 |
| 12.15 | 1.08 | 12.17 | 5.02 | 24.33 | 0.88 | 12.16 | 0.37 | 13.66 | 5.58 | 12.16 | 0.05 | 24.33 | -0.14 | 12.16 | 0.13 | 24.33 | 2.62 |
| 12.24 | 1.26 | 12.25 | 5.16 | 24.50 | 0.94 | 12.25 | 0.60 | 13.75 | 5.62 | 12.25 | 0.08 | 24.50 | -0.06 | 12.24 | 0.15 | 24.50 | 2.62 |
| 12.32 | 1.80 | 12.33 | 5.06 | 24.67 | 0.99 | 12.33 | 0.32 | 13.83 | 5.62 | 12.33 | -0.01 | 24.67 | -0.13 | 12.33 | 0.19 | 24.67 | 2.62 |
| 12.40 | 1.95 | 12.42 | 5.04 | 24.83 | 1.02 | 12.41 | 0.37 | 13.91 | 5.54 | 12.41 | -0.04 | 24.83 | -0.14 | 12.41 | 0.16 | 24.83 | 2.63 |
| 12.49 | 2.02 | 12.50 | 5.16 | 25.00 | 0.92 | 12.50 | 0.43 | 14.00 | 5.65 | 12.50 | 0.06 | 25.00 | 0.01 | 12.49 | 0.12 | 25.00 | 2.65 |
| 12.57 | 2.02 | 12.58 | 5.03 | 25.17 | 1.02 | 12.58 | 0.43 | 14.08 | 5.71 | 12.58 | -0.03 | 25.17 | 0.03 | 12.58 | 0.12 | 25.17 | 2.66 |
| 12.65 | 2.02 | 12.67 | 5.17 | 25.33 | 0.98 | 12.66 | 0.58 | 14.16 | 5.73 | 12.66 | -0.03 | 25.33 | 0.02 | 12.66 | 0.13 | 25.33 | 2.91 |
| 12.74 | 2.21 | 12.75 | 5.04 | 25.50 | 1.05 | 12.75 | 0.34 | 14.25 | 5.66 | 12.75 | 0.04 | 25.50 | -0.02 | 12.74 | 0.18 | 25.50 | 2.87 |
| 12.82 | 2.35 | 12.83 | 5.10 | 25.67 | 1.14 | 12.83 | 0.61 | 14.33 | 5.71 | 12.83 | 0.00 | 25.67 | 0.03 | 12.83 | 0.21 | 25.67 | 2.89 |
| 12.90 | 2.20 | 12.92 | 5.02 | 25.83 | 1.14 | 12.91 | 0.38 | 14.41 | 5.64 | 12.91 | 0.04 | 25.83 | 0.03 | 12.91 | 0.18 | 25.83 | 2.86 |
| 12.99 | 2.03 | 13.00 | 5.01 | 26.00 | 1.11 | 13.00 | 0.40 | 14.50 | 5.61 | 13.00 | 0.05 | 26.00 | 0.02 | 12.99 | 0.16 | 26.00 | 2.73 |
| 13.07 | 2.24 | 13.08 | 5.01 | 26.17 | 1.09 | 13.08 | 0.45 | 14.58 | 5.60 | 13.08 | -0.01 | 26.17 | -0.16 | 13.08 | 0.11 | 26.17 | 3.13 |
| 13.15 | 2.20 | 13.17 | 5.04 | 26.33 | 1.11 | 13.16 | 0.54 | 14.66 | 5.75 | 13.16 | 0.10 | 26.33 | -0.13 | 13.16 | 0.10 | 26.33 | 3.13 |
| 13.24 | 1.98 | 13.25 | 5.07 | 26.50 | 1.17 | 13.25 | 0.43 | 14.75 | 5.66 | 13.25 | 0.12 | 26.50 | 0.03 | 13.24 | 0.19 | 26.50 | 3.04 |
| 13.32 | 2.23 | 13.33 | 5.14 | 26.67 | 1.10 | 13.33 | 0.57 | 14.83 | 5.73 | 13.33 | 0.09 | 26.67 | -0.16 | 13.33 | 0.19 | 26.67 | 3.11 |
| 13.40 | 2.19 | 13.42 | 5.12 | 26.83 | 1.10 | 13.41 | 0.37 | 14.91 | 5.83 | 13.41 | 0.10 | 26.83 | -0.15 | 13.41 | 0.21 | 26.83 | 3.13 |
| 13.49 | 1.97 | 13.50 | 5.08 | 27.00 | 1.12 | 13.50 | 0.38 | 15.00 | 5.87 | 13.50 | 0.10 | 27.00 | 0.01 | 13.49 | 0.14 | 27.00 | 3.12 |
| 13.57 | 2.13 | 13.58 | 5.09 | 27.17 | 1.17 | 13.58 | 0.51 | 15.08 | 5.86 | 13.58 | 0.09 | 27.17 | -0.01 | 13.58 | 0.20 | 27.17 | 3.16 |
| 13.65 | 2.19 | 13.67 | 5.31 | 27.33 | 1.11 | 13.66 | 0.42 | 15.16 | 5.80 | 13.66 | 0.16 | 27.33 | -0.04 | 13.66 | 0.17 | 27.33 | 3.21 |
| 13.74 | 2.18 | 13.75 | 5.34 | 27.50 | 1.12 | 13.75 | 0.39 | 15.25 | 5.80 | 13.75 | 0.15 | 27.50 | 0.00 | 13.74 | 0.16 | 27.50 | 3.21 |
| 13.82 | 1.93 | 13.83 | 5.21 | 27.67 | 1.13 | 13.83 | 0.56 | 15.33 | 5.84 | 13.83 | 0.18 | 27.67 | -0.01 | 13.83 | 0.19 | 27.67 | 3.21 |
| 13.90 | 2.13 | 13.92 | 5.28 | 27.83 | 1.22 | 13.91 | 0.36 | 15.41 | 5.99 | 13.91 | 0.21 | 27.83 | -0.03 | 13.91 | 0.15 | 27.83 | 3.31 |
| 13.99 | 2.00 | 14.00 | 5.20 | 28.00 | 1.33 | 14.00 | 0.57 | 15.50 | 5.97 | 14.00 | 0.19 | 28.00 | 0.06 | 13.99 | 0.15 | 28.00 | 3.32 |
| 14.07 | 2.17 | 14.08 | 5.32 | 28.17 | 1.30 | 14.08 | 0.39 | 15.58 | 6.04 | 14.08 | 0.13 | 28.17 | -0.15 | 14.08 | 0.22 | 28.17 | 3.30 |
| 14.15 | 2.17 | 14.17 | 5.33 | 28.33 | 1.22 | 14.16 | 0.47 | 15.66 | 5.90 | 14.16 | 0.18 | 28.33 | -0.16 | 14.16 | 0.24 | 28.33 | 3.34 |
| 14.24 | 2.17 | 14.25 | 5.48 | 28.50 | 1.24 | 14.25 | 0.51 | 15.75 | 6.10 | 14.25 | 0.18 | 28.50 | 0.04 | 14.24 | 0.19 | 28.50 | 3.42 |
| 14.32 | 2.16 | 14.33 | 5.57 | 28.67 | 1.22 | 14.33 | 0.53 | 15.83 | 5.97 | 14.33 | 0.18 | 28.67 | -0.14 | 14.33 | 0.25 | 28.67 | 3.41 |
| 14.40 | 1.94 | 14.42 | 5.27 | 28.83 | 1.35 | 14.41 | 0.42 | 15.91 | 6.03 | 14.41 | 0.18 | 28.83 | -0.15 | 14.41 | 0.18 | 28.83 | 3.44 |
| 14.49 | 2.11 | 14.50 | 5.32 | 29.00 | 1.25 | 14.50 | 0.39 | 16.00 | 6.13 | 14.50 | 0.18 | 29.00 | -0.01 | 14.49 | 0.18 | 29.00 | 3.46 |
| 14.57 | 2.14 | 14.58 | 5.31 | 29.17 | 1.23 | 14.58 | 0.43 | 16.08 | 6.15 | 14.58 | 0.25 | 29.17 | 0.01 | 14.58 | 0.24 | 29.17 | 3.47 |
| 14.65 | 1.98 | 14.67 | 5.55 | 29.33 | 1.28 | 14.66 | 0.40 | 16.16 | 6.20 | 14.66 | 0.23 | 29.33 | -0.14 | 14.66 | 0.24 | 29.33 | 3.47 |
| 14.74 | 2.16 | 14.75 | 5.30 | 29.50 | 1.28 | 14.75 | 0.55 | 16.25 | 6.24 | 14.75 | 0.32 | 29.50 | -0.02 | 14.74 | 0.20 | 29.50 | 3.56 |
| 14.82 | 2.16 | 14.83 | 5.33 | 29.67 | 1.41 | 14.83 | 0.50 | 16.33 | 6.10 | 14.83 | 0.29 | 29.67 | -0.02 | 14.83 | 0.18 | 29.67 | 3.57 |
| 14.90 | 2.17 | 14.92 | 5.33 | 29.83 | 1.42 | 14.91 | 0.54 | 16.41 | 6.22 | 14.91 | 0.31 | 29.83 | 0.05 | 14.91 | 0.16 | 29.83 | 3.60 |
| 14.99 | 1.95 | 15.00 | 5.37 | 30.00 | 1.42 | 15.00 | 0.31 | 16.50 | 6.17 | 15.00 | 0.32 | 30.00 | 0.02 | 14.99 | 0.20 | 30.00 | 3.62 |
| 15.07 | 2.21 | 15.08 | 5.42 | 30.17 | 1.46 | 15.08 | 0.36 | 16.58 | 6.21 | 15.08 | 0.27 | 30.17 | -0.15 | 15.08 | 0.21 | 30.17 | 3.63 |
| 15.15 | 1.97 | 15.17 | 5.36 | 30.33 | 1.53 | 15.16 | 0.43 | 16.66 | 6.22 | 15.16 | 0.36 | 30.33 | -0.14 | 15.16 | 0.21 | 30.33 | 3.63 |
| 15.24 | 2.23 | 15.25 | 5.52 | 30.50 | 1.57 | 15.25 | 0.43 | 16.75 | 6.27 | 15.25 | 0.36 | 30.50 | 0.04 | 15.24 | 0.19 | 30.50 | 3.63 |
| 15.32 | 2.18 | 15.33 | 5.31 | 30.67 | 1.61 | 15.33 | 0.46 | 16.83 | 6.28 | 15.33 | 0.29 | 30.67 | -0.16 | 15.33 | 0.17 | 30.67 | 3.70 |
| 15.40 | 2.17 | 15.42 | 5.32 | 30.83 | 1.57 | 15.41 | 0.46 | 16.91 | 6.31 | 15.41 | 0.36 | 30.83 | -0.15 | 15.41 | 0.21 | 30.83 | 3.72 |
| 15.49 | 2.18 | 15.50 | 5.45 | 31.00 | 1.63 | 15.50 | 0.49 | 17.00 | 6.33 | 15.50 | 0.28 | 31.00 | 0.00 | 15.49 | 0.14 | 31.00 | 4.40 |
| 15.57 | 1.97 | 15.58 | 5.51 | 31.17 | 1.69 | 15.58 | 0.50 | 17.08 | 6.39 | 15.58 | 0.37 | 31.17 | 0.01 | 15.58 | 0.20 | 31.17 | 5.23 |
| 15.65 | 2.01 | 15.67 | 5.57 | 31.33 | 1.76 | 15.66 | 0.38 | 17.16 | 6.30 | 15.66 | 0.31 | 31.33 | -0.13 | 15.66 | 0.22 | 31.33 | 5.23 |

| | | | | | | | | | | | | | | | | | |
|---|---|---|---|---|---|---|---|---|---|---|---|---|---|---|---|---|---|
| 15.74 | 2.22 | 15.75 | 5.57 | 31.50 | 1.76 | 15.75 | 0.42 | 17.25 | 6.41 | 15.75 | 0.29 | 31.50 | 0.03 | 15.74 | 0.17 | 31.50 | 5.29 |
| 15.82 | 2.21 | 15.83 | 5.56 | 31.67 | 1.64 | 15.83 | 0.46 | 17.33 | 6.39 | 15.83 | 0.38 | 31.67 | 0.00 | 15.83 | 0.19 | 31.67 | 5.23 |
| 15.90 | 2.20 | 15.92 | 5.66 | 31.83 | 1.66 | 15.91 | 0.57 | 17.41 | 6.28 | 15.91 | 0.27 | 31.83 | -0.01 | 15.91 | 0.16 | 31.83 | 5.33 |
| 15.99 | 1.97 | 16.00 | 5.60 | 32.00 | 1.66 | 16.00 | 0.57 | 17.50 | 6.28 | 16.00 | 0.36 | 32.00 | 0.02 | 15.99 | 0.16 | 32.00 | 5.33 |
| 16.07 | 2.21 | 16.08 | 5.58 | 32.17 | 1.78 | 16.08 | 0.58 | 17.58 | 6.34 | 16.08 | 0.30 | 32.17 | -0.03 | 16.08 | 0.19 | 32.17 | 5.32 |
| 16.15 | 2.00 | 16.17 | 5.59 | 32.33 | 1.67 | 16.16 | 0.57 | 17.66 | 6.47 | 16.16 | 0.30 | 32.33 | -0.15 | 16.16 | 0.19 | 32.33 | 5.40 |
| 16.24 | 2.21 | 16.25 | 5.68 | 32.50 | 1.64 | 16.25 | 0.63 | 17.75 | 6.52 | 16.25 | 0.30 | 32.50 | 0.05 | 16.24 | 0.20 | 32.50 | 5.43 |
| 16.32 | 2.00 | 16.33 | 5.58 | 32.67 | 1.67 | 16.33 | 0.65 | 17.83 | 6.53 | 16.33 | 0.36 | 32.67 | -0.14 | 16.33 | 0.15 | 32.67 | 5.41 |
| 16.40 | 2.21 | 16.42 | 5.56 | 32.83 | 1.69 | 16.41 | 0.71 | 17.91 | 6.43 | 16.41 | 0.32 | 32.83 | -0.16 | 16.41 | 0.18 | 32.83 | 5.48 |
| 16.49 | 2.21 | 16.50 | 5.68 | 33.00 | 1.69 | 16.50 | 0.61 | 18.00 | 6.42 | 16.50 | 0.35 | 33.00 | 0.01 | 16.49 | 0.15 | 33.00 | 5.53 |
| 16.57 | 1.98 | 16.58 | 5.56 | 33.17 | 1.69 | 16.58 | 0.71 | 18.08 | 6.49 | 16.58 | 0.27 | 33.17 | 0.01 | 16.58 | 0.19 | 33.17 | 5.54 |
| 16.65 | 2.01 | 16.67 | 5.57 | 33.33 | 1.72 | 16.66 | 0.59 | 18.16 | 6.54 | 16.66 | 0.37 | 33.33 | -0.15 | 16.66 | 0.20 | 33.33 | 5.61 |
| 16.74 | 2.22 | 16.75 | 5.65 | 33.50 | 1.71 | 16.75 | 0.73 | 18.25 | 6.58 | 16.75 | 0.32 | 33.50 | -0.01 | 16.74 | 0.16 | 33.50 | 5.57 |
| 16.82 | 2.20 | 16.83 | 5.60 | 33.67 | 1.81 | 16.83 | 0.59 | 18.33 | 6.58 | 16.83 | 0.34 | 33.67 | -0.02 | 16.83 | 0.17 | 33.67 | 5.57 |
| 16.90 | 1.99 | 16.92 | 5.57 | 33.83 | 1.74 | 16.91 | 0.73 | 18.41 | 6.60 | 16.91 | 0.37 | 33.83 | 0.06 | 16.91 | 0.20 | 33.83 | 5.65 |
| 16.99 | 2.22 | 17.00 | 5.53 | 34.00 | 1.83 | 17.00 | 0.65 | 18.50 | 6.64 | 17.00 | 0.39 | 34.00 | 0.01 | 16.99 | 0.20 | 34.00 | 5.55 |
| 17.07 | 2.20 | 17.08 | 5.76 | 34.17 | 1.87 | 17.08 | 0.67 | 18.58 | 6.73 | 17.08 | 0.34 | 34.17 | 0.04 | 17.08 | 0.16 | 34.17 | 5.59 |
| 17.15 | 1.96 | 17.17 | 5.97 | 34.33 | 1.90 | 17.16 | 0.71 | 18.66 | 6.71 | 17.16 | 0.39 | 34.33 | -0.14 | 17.16 | 0.19 | 34.33 | 5.61 |
| 17.24 | 2.00 | 17.25 | 6.11 | 34.50 | 1.94 | 17.25 | 0.67 | 18.75 | 6.65 | 17.25 | 0.45 | 34.50 | 0.02 | 17.24 | 0.14 | 34.50 | 5.70 |
| 17.32 | 2.04 | 17.33 | 5.96 | 34.67 | 2.01 | 17.33 | 0.78 | 18.83 | 6.68 | 17.33 | 0.43 | 34.67 | -0.15 | 17.33 | 0.20 | 34.67 | 5.74 |
| 17.40 | 2.19 | 17.42 | 5.77 | 34.83 | 2.01 | 17.41 | 0.77 | 18.91 | 6.67 | 17.41 | 0.46 | 34.83 | -0.13 | 17.41 | 0.20 | 34.83 | 5.74 |
| 17.49 | 2.19 | 17.50 | 6.03 | 35.00 | 2.00 | 17.50 | 0.83 | 19.00 | 6.71 | 17.50 | 0.45 | 35.00 | 0.00 | 17.49 | 0.23 | 35.00 | 5.76 |
| 17.57 | 2.00 | 17.58 | 5.85 | 35.17 | 2.01 | 17.58 | 0.90 | 19.08 | 6.74 | 17.58 | 0.33 | 35.17 | 0.01 | 17.58 | 0.24 | 35.17 | 5.89 |
| 17.65 | 2.22 | 17.67 | 6.13 | 35.33 | 2.05 | 17.66 | 0.77 | 19.16 | 6.75 | 17.66 | 0.40 | 35.33 | -0.13 | 17.66 | 0.21 | 35.33 | 5.90 |
| 17.74 | 2.01 | 17.75 | 6.08 | 35.50 | 2.07 | 17.75 | 0.91 | 19.25 | 6.80 | 17.75 | 0.39 | 35.50 | 0.02 | 17.74 | 0.21 | 35.50 | 5.90 |
| 17.82 | 2.04 | 17.83 | 5.90 | 35.67 | 2.15 | 17.83 | 0.74 | 19.33 | 6.86 | 17.83 | 0.47 | 35.67 | 0.01 | 17.83 | 0.22 | 35.67 | 5.94 |
| 17.90 | 2.20 | 17.92 | 6.10 | 35.83 | 2.19 | 17.91 | 0.91 | 19.41 | 6.90 | 17.91 | 0.48 | 35.83 | -0.05 | 17.91 | 0.25 | 35.83 | 6.04 |
| 17.99 | 2.01 | 18.00 | 6.15 | 36.00 | 2.23 | 18.00 | 0.75 | 19.50 | 6.80 | 18.00 | 0.44 | 36.00 | 0.02 | 17.99 | 0.21 | 36.00 | 5.93 |
| 18.07 | 2.24 | 18.08 | 5.98 | 36.17 | 2.08 | 18.08 | 0.76 | 19.58 | 6.93 | 18.08 | 0.44 | 36.17 | -0.01 | 18.08 | 0.23 | 36.17 | 6.05 |
| 18.15 | 2.04 | 18.17 | 5.97 | 36.33 | 2.09 | 18.16 | 0.92 | 19.66 | 6.76 | 18.16 | 0.48 | 36.33 | -0.15 | 18.16 | 0.20 | 36.33 | 5.99 |
| 18.24 | 2.19 | 18.25 | 5.97 | 36.50 | 2.14 | 18.25 | 0.77 | 19.75 | 6.82 | 18.25 | 0.49 | 36.50 | -0.02 | 18.24 | 0.24 | 36.50 | 6.03 |
| 18.32 | 2.06 | 18.33 | 6.10 | 36.67 | 2.12 | 18.33 | 0.77 | 19.83 | 6.86 | 18.33 | 0.48 | 36.67 | -0.14 | 18.33 | 0.20 | 36.67 | 6.04 |
| 18.40 | 2.10 | 18.42 | 6.07 | 36.83 | 2.13 | 18.41 | 0.92 | 19.91 | 6.90 | 18.41 | 0.48 | 36.83 | -0.16 | 18.41 | 0.21 | 36.83 | 6.13 |
| 18.49 | 2.10 | 18.50 | 5.89 | 37.00 | 2.24 | 18.50 | 0.90 | 20.00 | 7.02 | 18.50 | 0.46 | 37.00 | -0.02 | 18.49 | 0.20 | 37.00 | 6.13 |
| 18.57 | 2.08 | 18.58 | 5.81 | 37.17 | 2.26 | 18.58 | 0.75 | 20.08 | 6.97 | 18.58 | 0.47 | 37.17 | 0.01 | 18.58 | 0.25 | 37.17 | 6.11 |
| 18.65 | 2.09 | 18.67 | 5.88 | 37.33 | 2.28 | 18.66 | 0.83 | 20.16 | 7.08 | 18.66 | 0.42 | 37.33 | -0.15 | 18.66 | 0.18 | 37.33 | 6.17 |
| 18.74 | 2.24 | 18.75 | 5.87 | 37.50 | 2.33 | 18.75 | 0.87 | 20.25 | 6.91 | 18.75 | 0.45 | 37.50 | 0.01 | 18.74 | 0.17 | 37.50 | 6.23 |
| 18.82 | 2.07 | 18.83 | 6.13 | 37.67 | 2.39 | 18.83 | 0.81 | 20.33 | 6.99 | 18.83 | 0.45 | 37.67 | 0.02 | 18.83 | 0.16 | 37.67 | 6.24 |
| 18.90 | 2.19 | 18.92 | 6.03 | 37.83 | 2.41 | 18.91 | 0.74 | 20.41 | 7.11 | 18.91 | 0.47 | 37.83 | -0.14 | 18.91 | 0.23 | 37.83 | 6.26 |
| 18.99 | 2.13 | 19.00 | 6.07 | 38.00 | 2.47 | 19.00 | 0.76 | 20.50 | 6.98 | 19.00 | 0.48 | 38.00 | -0.06 | 18.99 | 0.23 | 38.00 | 6.32 |
| 19.07 | 2.17 | 19.08 | 6.08 | 38.17 | 2.45 | 19.08 | 0.91 | 20.58 | 6.95 | 19.08 | 0.46 | 38.17 | 0.03 | 19.08 | 0.22 | 38.17 | 6.34 |
| 19.15 | 2.16 | 19.17 | 6.16 | 38.33 | 2.45 | 19.16 | 0.76 | 20.66 | 6.99 | 19.16 | 0.46 | 38.33 | -0.15 | 19.16 | 0.18 | 38.33 | 6.31 |
| 19.24 | 2.26 | 19.25 | 6.19 | 38.50 | 2.47 | 19.25 | 0.79 | 20.75 | 6.95 | 19.25 | 0.46 | 38.50 | -0.07 | 19.24 | 0.18 | 38.50 | 6.42 |
| 19.32 | 2.31 | 19.33 | 6.13 | 38.67 | 2.49 | 19.33 | 0.78 | 20.83 | 6.99 | 19.33 | 0.46 | 38.67 | -0.11 | 19.33 | 0.19 | 38.67 | 6.41 |
| 19.40 | 2.30 | 19.42 | 6.17 | 38.83 | 2.51 | 19.41 | 0.92 | 20.91 | 7.00 | 19.41 | 0.46 | 38.83 | -0.12 | 19.41 | 0.18 | 38.83 | 6.33 |
| 19.49 | 2.19 | 19.50 | 6.36 | 39.00 | 2.61 | 19.50 | 0.77 | 21.00 | 7.02 | 19.50 | 0.48 | 39.00 | -0.07 | 19.49 | 0.23 | 39.00 | 6.38 |
| 19.57 | 2.21 | 19.58 | 6.20 | 39.17 | 2.67 | 19.58 | 0.90 | 21.08 | 7.06 | 19.58 | 0.45 | 39.17 | 0.02 | 19.58 | 0.21 | 39.17 | 6.42 |
| 19.65 | 2.22 | 19.67 | 6.39 | 39.33 | 2.54 | 19.66 | 0.76 | 21.16 | 7.07 | 19.66 | 0.45 | 39.33 | -0.12 | 19.66 | 0.23 | 39.33 | 6.45 |
| 19.74 | 2.31 | 19.75 | 6.28 | 39.50 | 2.54 | 19.75 | 0.78 | 21.25 | 7.08 | 19.75 | 0.45 | 39.50 | 0.02 | 19.74 | 0.22 | 39.50 | 6.44 |
| 19.82 | 2.20 | 19.83 | 6.32 | 39.67 | 2.59 | 19.83 | 0.78 | 21.33 | 7.13 | 19.83 | 0.45 | 39.67 | 0.00 | 19.83 | 0.21 | 39.67 | 6.55 |
| 19.90 | 2.19 | 19.92 | 6.14 | 39.83 | 2.68 | 19.91 | 0.90 | 21.41 | 7.20 | 19.91 | 0.46 | 39.83 | -0.13 | 19.91 | 0.19 | 39.83 | 6.55 |

| | | | | | | | | | | | | | | | | | |
|---|---|---|---|---|---|---|---|---|---|---|---|---|---|---|---|---|---|
| 19.99 | 2.32 | 20.00 | 6.19 | 40.00 | 2.70 | 20.00 | 0.84 | 21.50 | 7.22 | 20.00 | 0.46 | 40.00 | -0.03 | 19.99 | 0.26 | 40.00 | 6.56 |
| 20.07 | 2.22 | 20.08 | 6.43 | 40.17 | 2.69 | 20.08 | 0.78 | 21.58 | 7.14 | 20.08 | 0.47 | 40.17 | 0.00 | 20.08 | 0.21 | 40.17 | 6.66 |
| 20.15 | 2.24 | 20.17 | 6.13 | 40.33 | 2.77 | 20.16 | 0.89 | 21.66 | 7.15 | 20.16 | 0.46 | 40.33 | -0.13 | 20.16 | 0.21 | 40.33 | 6.68 |
| 20.24 | 2.26 | 20.25 | 6.14 | 40.50 | 2.80 | 20.25 | 0.81 | 21.75 | 7.25 | 20.25 | 0.47 | 40.50 | -0.04 | 20.24 | 0.26 | 40.50 | 6.70 |
| 20.32 | 2.32 | 20.33 | 6.20 | 40.67 | 2.81 | 20.33 | 0.81 | 21.83 | 7.13 | 20.33 | 0.46 | 40.67 | 0.02 | 20.33 | 0.21 | 40.67 | 6.71 |
| 20.40 | 2.29 | 20.42 | 6.42 | 40.83 | 2.93 | 20.41 | 0.80 | 21.91 | 7.19 | 20.41 | 0.46 | 40.83 | -0.11 | 20.41 | 0.24 | 40.83 | 6.83 |
| 20.49 | 2.41 | 20.50 | 6.21 | 41.00 | 2.91 | 20.50 | 0.81 | 22.00 | 7.26 | 20.50 | 0.45 | 41.00 | -0.03 | 20.49 | 0.26 | 41.00 | 6.77 |
| 20.57 | 2.47 | 20.58 | 6.22 | 41.17 | 2.93 | 20.58 | 0.80 | 22.08 | 7.29 | 20.58 | 0.48 | 41.17 | 0.02 | 20.58 | 0.22 | 41.17 | 6.88 |
| 20.65 | 2.29 | 20.67 | 6.41 | 41.33 | 3.06 | 20.66 | 0.90 | 22.16 | 7.34 | 20.66 | 0.48 | 41.33 | -0.14 | 20.66 | 0.27 | 41.33 | 6.80 |
| 20.74 | 2.30 | 20.75 | 6.14 | 41.50 | 3.08 | 20.75 | 0.89 | 22.25 | 7.38 | 20.75 | 0.45 | 41.50 | 0.02 | 20.74 | 0.26 | 41.50 | 6.79 |
| 20.82 | 2.49 | 20.83 | 6.41 | 41.67 | 2.99 | 20.83 | 0.80 | 22.33 | 7.37 | 20.83 | 0.47 | 41.67 | 0.01 | 20.83 | 0.29 | 41.67 | 6.75 |
| 20.90 | 2.27 | 20.92 | 6.11 | 41.83 | 3.00 | 20.91 | 0.84 | 22.41 | 7.33 | 20.91 | 0.46 | 41.83 | -0.14 | 20.91 | 0.33 | 41.83 | 6.86 |
| 20.99 | 2.49 | 21.00 | 6.14 | 42.00 | 3.01 | 21.00 | 0.94 | 22.50 | 7.33 | 21.00 | 0.46 | 42.00 | -0.11 | 20.99 | 0.32 | 42.00 | 6.83 |
| 21.07 | 2.25 | 21.08 | 6.43 | 42.17 | 3.14 | 21.08 | 0.90 | 22.58 | 7.38 | 21.08 | 0.46 | 42.17 | 0.02 | 21.08 | 0.34 | 42.17 | 6.88 |
| 21.15 | 2.27 | 21.17 | 6.12 | 42.33 | 3.08 | 21.16 | 0.90 | 22.66 | 7.44 | 21.16 | 0.46 | 42.33 | -0.14 | 21.16 | 0.28 | 42.33 | 6.93 |
| 21.24 | 2.29 | 21.25 | 6.42 | 42.50 | 3.12 | 21.25 | 0.90 | 22.75 | 7.49 | 21.25 | 0.45 | 42.50 | -0.10 | 21.24 | 0.33 | 42.50 | 6.96 |
| 21.32 | 2.31 | 21.33 | 6.43 | 42.67 | 3.22 | 21.33 | 0.93 | 22.83 | 7.52 | 21.33 | 0.47 | 42.67 | 0.02 | 21.33 | 0.33 | 42.67 | 6.99 |
| 21.40 | 2.51 | 21.42 | 6.41 | 42.83 | 3.20 | 21.41 | 1.00 | 22.91 | 7.56 | 21.41 | 0.47 | 42.83 | -0.12 | 21.41 | 0.33 | 42.83 | 6.95 |
| 21.49 | 2.27 | 21.50 | 7.00 | 43.00 | 3.21 | 21.50 | 1.05 | 23.00 | 7.43 | 21.50 | 0.48 | 43.00 | -0.11 | 21.49 | 0.35 | 43.00 | 6.97 |
| 21.57 | 2.29 | 21.58 | 6.61 | 43.17 | 3.32 | 21.58 | 0.99 | 23.08 | 7.42 | 21.58 | 0.45 | 43.17 | 0.00 | 21.58 | 0.29 | 43.17 | 7.05 |
| 21.65 | 2.30 | 21.67 | 6.63 | 43.33 | 3.33 | 21.66 | 1.06 | 23.16 | 7.44 | 21.66 | 0.41 | 43.33 | -0.12 | 21.66 | 0.29 | 43.33 | 6.99 |
| 21.74 | 2.38 | 21.75 | 6.33 | 43.50 | 3.38 | 21.75 | 0.94 | 23.25 | 7.48 | 21.75 | 0.45 | 43.50 | -0.11 | 21.74 | 0.36 | 43.50 | 7.09 |
| 21.82 | 2.48 | 21.83 | 6.49 | 43.67 | 3.38 | 21.83 | 1.07 | 23.33 | 7.52 | 21.83 | 0.45 | 43.67 | -0.01 | 21.83 | 0.33 | 43.67 | 7.14 |
| 21.90 | 2.52 | 21.92 | 6.86 | 43.83 | 3.40 | 21.91 | 0.93 | 23.41 | 7.55 | 21.91 | 0.46 | 43.83 | -0.14 | 21.91 | 0.39 | 43.83 | 7.10 |
| 21.99 | 2.58 | 22.00 | 6.37 | 44.00 | 3.46 | 22.00 | 0.99 | 23.50 | 7.57 | 22.00 | 0.45 | 44.00 | -0.11 | 21.99 | 0.35 | 44.00 | 7.08 |
| 22.07 | 2.61 | 22.08 | 5.74 | 44.17 | 3.55 | 22.08 | 1.00 | 23.58 | 7.54 | 22.08 | 0.46 | 44.17 | 0.00 | 22.08 | 0.32 | 44.17 | 7.20 |
| 22.15 | 2.49 | 22.17 | 6.37 | 44.33 | 3.44 | 22.16 | 1.00 | 23.66 | 7.62 | 22.16 | 0.43 | 44.33 | -0.11 | 22.16 | 0.33 | 44.33 | 7.23 |
| 22.24 | 2.54 | 22.25 | 6.63 | 44.50 | 3.52 | 22.25 | 1.04 | 23.75 | 7.71 | 22.25 | 0.45 | 44.50 | -0.05 | 22.24 | 0.31 | 44.50 | 7.22 |
| 22.32 | 2.60 | 22.33 | 6.72 | 44.67 | 3.47 | 22.33 | 0.98 | 23.83 | 7.74 | 22.33 | 0.46 | 44.67 | 0.00 | 22.33 | 0.36 | 44.67 | 7.25 |
| 22.40 | 2.62 | 22.42 | 6.63 | 44.83 | 3.48 | 22.41 | 1.07 | 23.91 | 7.68 | 22.41 | 0.47 | 44.83 | -0.11 | 22.41 | 0.35 | 44.83 | 7.23 |
| 22.49 | 2.68 | 22.50 | 6.59 | 45.00 | 3.49 | 22.50 | 0.99 | 24.00 | 7.68 | 22.50 | 0.48 | 45.00 | -0.11 | 22.49 | 0.33 | 45.00 | 7.22 |
| 22.57 | 2.71 | 22.58 | 6.34 | 45.17 | 3.55 | 22.58 | 1.01 | 24.08 | 7.76 | 22.58 | 0.45 | 45.17 | 0.01 | 22.58 | 0.31 | 45.17 | 7.20 |
| 22.65 | 2.73 | 22.67 | 6.37 | 45.33 | 3.57 | 22.66 | 0.99 | 24.16 | 7.76 | 22.66 | 0.47 | 45.33 | -0.15 | 22.66 | 0.30 | 45.33 | 7.23 |
| 22.74 | 2.62 | 22.75 | 6.39 | 45.50 | 3.64 | 22.75 | 1.04 | 24.25 | 7.63 | 22.75 | 0.42 | 45.50 | -0.11 | 22.74 | 0.32 | 45.50 | 7.23 |
| 22.82 | 2.66 | 22.83 | 6.59 | 45.67 | 3.66 | 22.83 | 1.07 | 24.33 | 7.63 | 22.83 | 0.46 | 45.67 | 0.01 | 22.83 | 0.38 | 45.67 | 7.24 |
| 22.90 | 2.67 | 22.92 | 6.75 | 45.83 | 3.70 | 22.91 | 1.01 | 24.41 | 7.67 | 22.91 | 0.47 | 45.83 | -0.14 | 22.91 | 0.37 | 45.83 | 7.34 |
| 22.99 | 3.37 | 23.00 | 6.98 | 46.00 | 3.78 | 23.00 | 1.02 | 24.50 | 7.69 | 23.00 | 0.47 | 46.00 | -0.13 | 22.99 | 0.40 | 46.00 | 7.37 |
| 23.07 | 3.55 | 23.08 | 6.83 | 46.17 | 3.80 | 23.08 | 1.12 | 24.58 | 7.72 | 23.08 | 0.47 | 46.17 | 0.02 | 23.08 | 0.39 | 46.17 | 7.37 |
| 23.15 | 3.55 | 23.17 | 6.64 | 46.33 | 3.81 | 23.16 | 1.14 | 24.66 | 7.78 | 23.16 | 0.46 | 46.33 | -0.13 | 23.16 | 0.35 | 46.33 | 7.49 |
| 23.24 | 3.50 | 23.25 | 6.62 | 46.50 | 3.93 | 23.25 | 1.19 | 24.75 | 7.83 | 23.25 | 0.43 | 46.50 | -0.14 | 23.24 | 0.42 | 46.50 | 7.48 |
| 23.32 | 3.35 | 23.33 | 6.65 | 46.67 | 3.96 | 23.33 | 1.22 | 24.83 | 7.86 | 23.33 | 0.46 | 46.67 | 0.01 | 23.33 | 0.40 | 46.67 | 7.44 |
| 23.40 | 3.23 | 23.42 | 6.75 | 46.83 | 3.96 | 23.41 | 1.17 | 24.91 | 7.83 | 23.41 | 0.46 | 46.83 | -0.13 | 23.41 | 0.42 | 46.83 | 7.52 |
| 23.49 | 3.41 | 23.50 | 6.83 | 47.00 | 3.87 | 23.50 | 1.23 | 25.00 | 7.89 | 23.50 | 0.47 | 47.00 | -0.16 | 23.49 | 0.42 | 47.00 | 7.54 |
| 23.57 | 3.41 | 23.58 | 6.85 | 47.17 | 3.87 | 23.58 | 1.24 | 25.08 | 7.89 | 23.58 | 0.47 | 47.17 | 0.01 | 23.58 | 0.40 | 47.17 | 7.59 |
| 23.65 | 3.40 | 23.67 | 6.82 | 47.33 | 3.92 | 23.66 | 1.18 | 25.16 | 7.89 | 23.66 | 0.43 | 47.33 | -0.08 | 23.66 | 0.39 | 47.33 | 7.59 |
| 23.74 | 3.39 | 23.75 | 6.85 | 47.50 | 3.92 | 23.75 | 1.22 | 25.25 | 7.96 | 23.75 | 0.40 | 47.50 | -0.16 | 23.74 | 0.47 | 47.50 | 7.68 |
| 23.82 | 3.40 | 23.83 | 6.91 | 47.67 | 3.95 | 23.83 | 1.23 | 25.33 | 7.97 | 23.83 | 0.42 | 47.67 | -0.01 | 23.83 | 0.46 | 47.67 | 7.64 |
| 23.90 | 3.61 | 23.92 | 6.84 | 47.83 | 3.93 | 23.91 | 1.24 | 25.41 | 8.08 | 23.91 | 0.46 | 47.83 | -0.10 | 23.91 | 0.46 | 47.83 | 7.59 |
| 23.99 | 3.47 | 24.00 | 6.91 | 48.00 | 3.95 | 24.00 | 1.28 | 25.50 | 7.98 | 24.00 | 0.47 | 48.00 | -0.14 | 23.99 | 0.41 | 48.00 | 7.71 |
| 24.07 | 3.47 | 24.08 | 6.91 | 48.17 | 4.06 | 24.08 | 1.23 | 25.58 | 7.99 | 24.08 | 0.46 | 48.17 | 0.01 | 24.08 | 0.42 | 48.17 | 7.68 |
| 24.15 | 3.45 | 24.17 | 6.86 | 48.33 | 4.08 | 24.16 | 1.28 | 25.66 | 8.02 | 24.16 | 0.42 | 48.33 | -0.08 | 24.16 | 0.41 | 48.33 | 7.58 |

| | | | | | | | | | | | | | | | | | |
|---|---|---|---|---|---|---|---|---|---|---|---|---|---|---|---|---|---|
| 24.24 | 3.46 | 24.25 | 6.87 | 48.50 | 4.12 | 24.25 | 1.36 | 25.75 | 8.00 | 24.25 | 0.42 | 48.50 | -0.16 | 24.24 | 0.42 | 48.50 | 7.56 |
| 24.32 | 3.59 | 24.33 | 6.96 | 48.67 | 4.18 | 24.33 | 1.39 | 25.83 | 8.07 | 24.33 | 0.43 | 48.67 | 0.00 | 24.33 | 0.46 | 48.67 | 7.65 |
| 24.40 | 3.38 | 24.42 | 7.02 | 48.83 | 4.24 | 24.41 | 1.34 | 25.91 | 8.02 | 24.41 | 0.46 | 48.83 | -0.08 | 24.41 | 0.35 | 48.83 | 7.70 |
| 24.49 | 3.41 | 24.50 | 7.06 | 49.00 | 4.25 | 24.50 | 1.44 | 26.00 | 8.07 | 24.50 | 0.45 | 49.00 | -0.14 | 24.49 | 0.36 | 49.00 | 7.65 |
| 24.57 | 3.42 | 24.58 | 6.86 | 49.17 | 4.25 | 24.58 | 1.32 | 26.08 | 8.14 | 24.58 | 0.52 | 49.17 | 0.00 | 24.58 | 0.41 | 49.17 | 7.73 |
| 24.65 | 3.44 | 24.67 | 6.86 | 49.33 | 4.36 | 24.66 | 1.40 | 26.16 | 8.17 | 24.66 | 0.55 | 49.33 | -0.07 | 24.66 | 0.47 | 49.33 | 7.78 |
| 24.74 | 3.47 | 24.75 | 6.85 | 49.50 | 4.40 | 24.75 | 1.32 | 26.25 | 8.20 | 24.75 | 0.50 | 49.50 | -0.12 | 24.74 | 0.39 | 49.50 | 7.81 |
| 24.82 | 3.56 | 24.83 | 6.94 | 49.67 | 4.41 | 24.83 | 1.44 | 26.33 | 8.22 | 24.83 | 0.55 | 49.67 | 0.01 | 24.83 | 0.46 | 49.67 | 7.80 |
| 24.90 | 3.43 | 24.92 | 6.98 | 49.83 | 4.37 | 24.91 | 1.40 | 26.41 | 8.28 | 24.91 | 0.57 | 49.83 | -0.08 | 24.91 | 0.38 | 49.83 | 7.88 |
| 24.99 | 3.47 | 25.00 | 7.09 | 50.00 | 4.36 | 25.00 | 1.39 | 26.50 | 8.15 | 25.00 | 0.59 | 50.00 | -0.15 | 24.99 | 0.38 | 50.00 | 7.88 |
| 25.07 | 3.46 | 25.08 | 7.06 | 50.17 | 4.37 | 25.08 | 1.42 | 26.58 | 8.16 | 25.08 | 0.60 | 50.17 | 0.00 | 25.08 | 0.44 | 50.17 | 7.91 |
| 25.15 | 3.51 | 25.17 | 6.90 | 50.33 | 4.38 | 25.16 | 1.50 | 26.66 | 8.18 | 25.16 | 0.60 | 50.33 | -0.14 | 25.16 | 0.48 | 50.33 | 7.97 |
| 25.24 | 3.56 | 25.25 | 7.01 | 50.50 | 4.42 | 25.25 | 1.44 | 26.75 | 8.22 | 25.25 | 0.57 | 50.50 | -0.15 | 25.24 | 0.46 | 50.50 | 7.99 |
| 25.32 | 3.61 | 25.33 | 6.91 | 50.67 | 4.45 | 25.33 | 1.48 | 26.83 | 8.22 | 25.33 | 0.61 | 50.67 | 0.01 | 25.33 | 0.48 | 50.67 | 7.97 |
| 25.40 | 3.62 | 25.42 | 7.03 | 50.83 | 4.54 | 25.41 | 1.55 | 26.91 | 8.20 | 25.41 | 0.60 | 50.83 | -0.09 | 25.41 | 0.47 | 50.83 | 8.04 |
| 25.49 | 3.68 | 25.50 | 6.89 | 51.00 | 4.56 | 25.50 | 1.62 | 27.00 | 8.22 | 25.50 | 0.60 | 51.00 | -0.15 | 25.49 | 0.38 | 51.00 | 8.06 |
| 25.57 | 3.65 | 25.58 | 7.03 | 51.17 | 4.56 | 25.58 | 1.48 | 27.08 | 8.28 | 25.58 | 0.61 | 51.17 | 0.01 | 25.58 | 0.41 | 51.17 | 8.10 |
| 25.65 | 3.73 | 25.67 | 6.94 | 51.33 | 4.67 | 25.66 | 1.48 | 27.16 | 8.28 | 25.66 | 0.56 | 51.33 | -0.06 | 25.66 | 0.44 | 51.33 | 8.00 |
| 25.74 | 3.80 | 25.75 | 6.93 | 51.50 | 4.70 | 25.75 | 1.61 | 27.25 | 8.08 | 25.75 | 0.58 | 51.50 | -0.14 | 25.74 | 0.48 | 51.50 | 8.08 |
| 25.82 | 3.76 | 25.83 | 6.91 | 51.67 | 4.72 | 25.83 | 1.58 | 27.33 | 7.77 | 25.83 | 0.59 | 51.67 | 0.02 | 25.83 | 0.49 | 51.67 | 8.05 |
| 25.90 | 3.75 | 25.92 | 7.08 | 51.83 | 4.79 | 25.91 | 1.47 | 27.41 | 7.91 | 25.91 | 0.62 | 51.83 | -0.04 | 25.91 | 0.48 | 51.83 | 8.00 |
| 25.99 | 3.84 | 26.00 | 6.97 | 52.00 | 4.85 | 26.00 | 1.47 | 27.50 | 8.21 | 26.00 | 0.62 | 52.00 | -0.15 | 25.99 | 0.38 | 52.00 | 8.03 |
| 26.07 | 3.72 | 26.08 | 6.91 | 52.17 | 4.81 | 26.08 | 1.51 | 27.58 | 8.33 | 26.08 | 0.61 | 52.17 | 0.02 | 26.08 | 0.44 | 52.17 | 8.05 |
| 26.15 | 3.72 | 26.17 | 6.93 | 52.33 | 4.79 | 26.16 | 1.51 | 27.66 | 8.39 | 26.16 | 0.57 | 52.33 | -0.03 | 26.16 | 0.48 | 52.33 | 8.04 |
| 26.24 | 3.75 | 26.25 | 6.94 | 52.50 | 4.90 | 26.25 | 1.63 | 27.75 | 8.34 | 26.25 | 0.60 | 52.50 | -0.14 | 26.24 | 0.50 | 52.50 | 8.13 |
| 26.32 | 3.75 | 26.33 | 7.02 | 52.67 | 4.83 | 26.33 | 1.62 | 27.83 | 8.41 | 26.33 | 0.59 | 52.67 | 0.00 | 26.33 | 0.49 | 52.67 | 8.09 |
| 26.40 | 3.84 | 26.42 | 6.89 | 52.83 | 4.84 | 26.41 | 1.45 | 27.91 | 8.53 | 26.41 | 0.61 | 52.83 | 0.00 | 26.41 | 0.43 | 52.83 | 8.14 |
| 26.49 | 3.76 | 26.50 | 7.00 | 53.00 | 4.85 | 26.50 | 1.51 | 28.00 | 8.60 | 26.50 | 0.60 | 53.00 | -0.13 | 26.49 | 0.47 | 53.00 | 8.06 |
| 26.57 | 3.76 | 26.58 | 6.91 | 53.17 | 4.99 | 26.58 | 1.57 | 28.08 | 8.61 | 26.58 | 0.61 | 53.17 | 0.01 | 26.58 | 0.48 | 53.17 | 8.19 |
| 26.65 | 3.87 | 26.67 | 6.94 | 53.33 | 5.00 | 26.66 | 1.61 | 28.16 | 8.58 | 26.66 | 0.60 | 53.33 | -0.03 | 26.66 | 0.49 | 53.33 | 8.18 |
| 26.74 | 3.78 | 26.75 | 6.96 | 53.50 | 5.01 | 26.75 | 2.60 | 28.25 | 8.74 | 26.75 | 0.58 | 53.50 | -0.14 | 26.74 | 0.48 | 53.50 | 8.20 |
| 26.82 | 3.86 | 26.83 | 7.02 | 53.67 | 5.07 | 26.83 | 2.60 | 28.33 | 8.74 | 26.83 | 0.60 | 53.67 | -0.01 | 26.83 | 0.48 | 53.67 | 8.27 |
| 26.90 | 3.98 | 26.92 | 7.05 | 53.83 | 5.14 | 26.91 | 2.34 | 28.41 | 8.67 | 26.91 | 0.62 | 53.83 | -0.04 | 26.91 | 0.43 | 53.83 | 8.31 |
| 26.99 | 4.00 | 27.00 | 7.12 | 54.00 | 5.16 | 27.00 | 2.35 | 28.50 | 8.65 | 27.00 | 0.61 | 54.00 | -0.13 | 26.99 | 0.46 | 54.00 | 8.33 |
| 27.07 | 3.80 | 27.08 | 7.14 | 54.17 | 5.25 | 27.08 | 2.32 | 28.58 | 8.83 | 27.08 | 0.60 | 54.17 | 0.02 | 27.08 | 0.45 | 54.17 | 8.33 |
| 27.15 | 3.82 | 27.17 | 7.18 | 54.33 | 5.29 | 27.16 | 2.44 | 28.66 | 8.80 | 27.16 | 0.60 | 54.33 | -0.01 | 27.16 | 0.48 | 54.33 | 8.36 |
| 27.24 | 4.03 | 27.25 | 7.11 | 54.50 | 5.27 | 27.25 | 2.42 | 28.75 | 8.63 | 27.25 | 0.58 | 54.50 | -0.12 | 27.24 | 0.48 | 54.50 | 8.37 |
| 27.32 | 3.78 | 27.33 | 7.13 | 54.67 | 5.25 | 27.33 | 2.65 | 28.83 | 8.68 | 27.33 | 0.60 | 54.67 | 0.02 | 27.33 | 0.48 | 54.67 | 8.34 |
| 27.40 | 3.85 | 27.42 | 7.17 | 54.83 | 5.36 | 27.41 | 2.35 | 28.91 | 8.70 | 27.41 | 0.63 | 54.83 | -0.05 | 27.41 | 0.49 | 54.83 | 8.44 |
| 27.49 | 3.89 | 27.50 | 7.25 | 55.00 | 5.27 | 27.50 | 2.68 | 29.00 | 8.70 | 27.50 | 0.62 | 55.00 | -0.13 | 27.49 | 0.47 | 55.00 | 8.50 |
| 27.57 | 3.90 | 27.58 | 7.25 | 55.17 | 5.26 | 27.58 | 2.35 | 29.08 | 8.69 | 27.58 | 0.61 | 55.17 | 0.01 | 27.58 | 0.47 | 55.17 | 8.53 |
| 27.65 | 3.85 | 27.67 | 7.30 | 55.33 | 5.26 | 27.66 | 2.38 | 29.16 | 8.66 | 27.66 | 0.60 | 55.33 | -0.04 | 27.66 | 0.48 | 55.33 | 8.45 |
| 27.74 | 3.87 | 27.75 | 7.41 | 55.50 | 5.32 | 27.75 | 2.43 | 29.25 | 8.78 | 27.75 | 0.60 | 55.50 | -0.13 | 27.74 | 0.45 | 55.50 | 8.45 |
| 27.82 | 4.04 | 27.83 | 7.25 | 55.67 | 5.34 | 27.83 | 2.46 | 29.33 | 8.68 | 27.83 | 0.60 | 55.67 | 0.02 | 27.83 | 0.46 | 55.67 | 8.40 |
| 27.90 | 3.88 | 27.92 | 7.24 | 55.83 | 5.37 | 27.91 | 2.47 | 29.41 | 8.68 | 27.91 | 0.62 | 55.83 | -0.01 | 27.91 | 0.49 | 55.83 | 8.42 |
| 27.99 | 3.92 | 28.00 | 7.28 | 56.00 | 5.43 | 28.00 | 2.68 | 29.50 | 8.68 | 28.00 | 0.61 | 56.00 | -0.13 | 27.99 | 0.46 | 56.00 | 8.47 |
| 28.07 | 4.00 | 28.08 | 7.38 | 56.17 | 5.47 | 28.08 | 2.39 | 29.58 | 8.73 | 28.08 | 0.61 | 56.17 | 0.03 | 28.08 | 0.48 | 56.17 | 8.45 |
| 28.15 | 4.07 | 28.17 | 7.36 | 56.33 | 5.48 | 28.16 | 2.66 | 29.66 | 8.70 | 28.16 | 0.61 | 56.33 | 0.00 | 28.16 | 0.48 | 56.33 | 8.47 |
| 28.24 | 4.03 | 28.25 | 7.22 | 56.50 | 5.58 | 28.25 | 2.47 | 29.75 | 8.70 | 28.25 | 0.59 | 56.50 | -0.14 | 28.24 | 0.46 | 56.50 | 8.55 |
| 28.32 | 4.12 | 28.33 | 7.20 | 56.67 | 5.59 | 28.33 | 2.52 | 29.83 | 8.72 | 28.33 | 0.60 | 56.67 | 0.01 | 28.33 | 0.42 | 56.67 | 8.54 |
| 28.40 | 4.09 | 28.42 | 7.22 | 56.83 | 5.60 | 28.41 | 2.53 | 29.91 | 8.79 | 28.41 | 0.59 | 56.83 | 0.00 | 28.41 | 0.45 | 56.83 | 8.56 |

| | | | | | | | | | | | | | | | | | |
|---|---|---|---|---|---|---|---|---|---|---|---|---|---|---|---|---|---|
| 28.49 | 4.17 | 28.50 | 7.27 | 57.00 | 5.64 | 28.50 | 2.39 | 30.00 | 8.86 | 28.50 | 0.60 | 57.00 | -0.13 | 28.49 | 0.47 | 57.00 | 8.61 |
| 28.57 | 4.25 | 28.58 | 7.26 | 57.17 | 5.75 | 28.58 | 2.52 | 30.08 | 8.88 | 28.58 | 0.61 | 57.17 | 0.01 | 28.58 | 0.49 | 57.17 | 8.62 |
| 28.65 | 4.16 | 28.67 | 7.32 | 57.33 | 5.74 | 28.66 | 2.30 | 30.16 | 8.86 | 28.66 | 0.62 | 57.33 | -0.02 | 28.66 | 0.48 | 57.33 | 8.63 |
| 28.74 | 4.16 | 28.75 | 7.25 | 57.50 | 5.67 | 28.75 | 2.41 | 30.25 | 8.90 | 28.75 | 0.61 | 57.50 | -0.03 | 28.74 | 0.49 | 57.50 | 8.68 |
| 28.82 | 4.31 | 28.83 | 7.22 | 57.67 | 5.68 | 28.83 | 2.47 | 30.33 | 8.94 | 28.83 | 0.60 | 57.67 | 0.03 | 28.83 | 0.46 | 57.67 | 8.75 |
| 28.90 | 4.17 | 28.92 | 7.40 | 57.83 | 5.73 | 28.91 | 2.50 | 30.41 | 8.84 | 28.91 | 0.60 | 57.83 | -0.02 | 28.91 | 0.40 | 57.83 | 8.77 |
| 28.99 | 4.14 | 29.00 | 7.27 | 58.00 | 5.72 | 29.00 | 2.59 | 30.50 | 8.83 | 29.00 | 0.60 | 58.00 | -0.12 | 28.99 | 0.49 | 58.00 | 8.73 |
| 29.07 | 4.23 | 29.08 | 7.24 | 58.17 | 5.70 | 29.08 | 2.46 | 30.58 | 8.84 | 29.08 | 0.60 | 58.17 | -0.01 | 29.08 | 0.49 | 58.17 | 8.78 |
| 29.15 | 4.27 | 29.17 | 7.28 | 58.33 | 5.75 | 29.16 | 2.43 | 30.66 | 8.94 | 29.16 | 0.61 | 58.33 | 0.00 | 29.16 | 0.53 | 58.33 | 8.83 |
| 29.24 | 4.36 | 29.25 | 7.36 | 58.50 | 5.77 | 29.25 | 2.63 | 30.75 | 8.97 | 29.25 | 0.60 | 58.50 | -0.03 | 29.24 | 0.53 | 58.50 | 8.83 |
| 29.32 | 4.43 | 29.33 | 7.27 | 58.67 | 5.83 | 29.33 | 2.46 | 30.83 | 8.90 | 29.33 | 0.60 | 58.67 | 0.04 | 29.33 | 0.47 | 58.67 | 8.83 |
| 29.40 | 4.23 | 29.42 | 7.25 | 58.83 | 5.87 | 29.41 | 2.52 | 30.91 | 9.03 | 29.41 | 0.59 | 58.83 | 0.01 | 29.41 | 0.51 | 58.83 | 8.89 |
| 29.49 | 4.46 | 29.50 | 7.26 | 59.00 | 5.92 | 29.50 | 2.51 | 31.00 | 9.06 | 29.50 | 0.60 | 59.00 | -0.04 | 29.49 | 0.52 | 59.00 | 8.84 |
| 29.57 | 4.23 | 29.58 | 7.27 | 59.17 | 5.94 | 29.58 | 2.60 | 31.08 | 8.97 | 29.58 | 0.60 | 59.17 | 0.02 | 29.58 | 0.53 | 59.17 | 8.84 |
| 29.65 | 4.24 | 29.67 | 7.29 | 59.33 | 6.01 | 29.66 | 2.73 | 31.16 | 8.98 | 29.66 | 0.61 | 59.33 | -0.02 | 29.66 | 0.61 | 59.33 | 8.89 |
| 29.74 | 4.26 | 29.75 | 7.27 | 59.50 | 6.04 | 29.75 | 2.80 | 31.25 | 9.01 | 29.75 | 0.60 | 59.50 | -0.04 | 29.74 | 0.57 | 59.50 | 8.88 |
| 29.82 | 4.26 | 29.83 | 7.39 | 59.67 | 6.03 | 29.83 | 2.79 | 31.33 | 9.02 | 29.83 | 0.58 | 59.67 | 0.05 | 29.83 | 0.54 | 59.67 | 8.95 |
| 29.90 | 4.28 | 29.92 | 7.26 | 59.83 | 6.07 | 29.91 | 2.69 | 31.41 | 8.99 | 29.91 | 0.60 | 59.83 | 0.02 | 29.91 | 0.56 | 59.83 | 8.88 |
| 29.99 | 4.50 | 30.00 | 7.29 | 60.00 | 6.19 | 30.00 | 2.82 | 31.50 | 9.15 | 30.00 | 0.59 | 60.00 | -0.04 | 29.99 | 0.53 | 60.00 | 8.94 |
| 30.07 | 4.32 | 30.08 | 7.33 | 60.17 | 6.18 | 30.08 | 2.97 | 31.58 | 8.95 | 30.08 | 0.60 | 60.17 | 0.04 | 30.08 | 0.59 | 60.17 | 8.93 |
| 30.15 | 4.30 | 30.17 | 7.37 | 60.33 | 6.12 | 30.16 | 2.99 | 31.66 | 8.99 | 30.16 | 0.60 | 60.33 | 0.04 | 30.16 | 0.61 | 60.33 | 8.91 |
| 30.24 | 4.32 | 30.25 | 7.46 | 60.50 | 6.16 | 30.25 | 2.77 | 31.75 | 8.99 | 30.25 | 0.61 | 60.50 | -0.03 | 30.24 | 0.62 | 60.50 | 9.03 |
| 30.32 | 4.35 | 30.33 | 7.49 | 60.67 | 6.15 | 30.33 | 3.00 | 31.83 | 9.05 | 30.33 | 0.61 | 60.67 | 0.04 | 30.33 | 0.60 | 60.67 | 9.05 |
| 30.40 | 4.31 | 30.42 | 7.54 | 60.83 | 6.15 | 30.41 | 2.75 | 31.91 | 9.06 | 30.41 | 0.61 | 60.83 | 0.04 | 30.41 | 0.59 | 60.83 | 9.02 |
| 30.49 | 4.49 | 30.50 | 7.46 | 61.00 | 6.17 | 30.50 | 2.86 | 32.00 | 9.07 | 30.50 | 0.61 | 61.00 | -0.04 | 30.49 | 0.60 | 61.00 | 9.10 |
| 30.57 | 4.28 | 30.58 | 7.48 | 61.17 | 6.20 | 30.58 | 2.86 | 32.08 | 9.13 | 30.58 | 0.61 | 61.17 | 0.05 | 30.58 | 0.61 | 61.17 | 9.13 |
| 30.65 | 4.28 | 30.67 | 7.60 | 61.33 | 6.23 | 30.66 | 2.82 | 32.16 | 9.11 | 30.66 | 0.60 | 61.33 | 0.02 | 30.66 | 0.63 | 61.33 | 9.06 |
| 30.74 | 4.30 | 30.75 | 7.66 | 61.50 | 6.28 | 30.75 | 2.80 | 32.25 | 9.19 | 30.75 | 0.61 | 61.50 | -0.03 | 30.74 | 0.64 | 61.50 | 9.15 |
| 30.82 | 4.30 | 30.83 | 7.66 | 61.67 | 6.30 | 30.83 | 2.82 | 32.33 | 9.21 | 30.83 | 0.63 | 61.67 | 0.04 | 30.83 | 0.59 | 61.67 | 9.17 |
| 30.90 | 4.35 | 30.92 | 7.69 | 61.83 | 6.35 | 30.91 | 3.08 | 32.41 | 9.24 | 30.91 | 0.63 | 61.83 | 0.04 | 30.91 | 0.66 | 61.83 | 9.15 |
| 30.99 | 4.32 | 31.00 | 7.74 | 62.00 | 6.35 | 31.00 | 2.85 | 32.50 | 9.17 | 31.00 | 0.61 | 62.00 | -0.04 | 30.99 | 0.67 | 62.00 | 9.14 |
| 31.07 | 4.35 | 31.08 | 7.73 | 62.17 | 6.42 | 31.08 | 2.89 | 32.58 | 9.29 | 31.08 | 0.61 | 62.17 | -0.04 | 31.08 | 0.62 | 62.17 | 9.20 |
| 31.15 | 4.46 | 31.17 | 7.80 | 62.33 | 6.48 | 31.16 | 3.09 | 32.66 | 9.20 | 31.16 | 0.61 | 62.33 | 0.03 | 31.16 | 0.66 | 62.33 | 9.19 |
| 31.24 | 4.35 | 31.25 | 7.91 | 62.50 | 6.43 | 31.25 | 2.98 | 32.75 | 9.18 | 31.25 | 0.61 | 62.50 | -0.03 | 31.24 | 0.64 | 62.50 | 9.25 |
| 31.32 | 4.49 | 31.33 | 7.84 | 62.67 | 6.51 | 31.33 | 3.15 | 32.83 | 9.26 | 31.33 | 0.61 | 62.67 | -0.02 | 31.33 | 0.65 | 62.67 | 9.31 |
| 31.40 | 4.52 | 31.42 | 7.79 | 62.83 | 6.49 | 31.41 | 2.90 | 32.91 | 9.22 | 31.41 | 0.63 | 62.83 | 0.02 | 31.41 | 0.61 | 62.83 | 9.29 |
| 31.49 | 4.50 | 31.50 | 7.73 | 63.00 | 6.49 | 31.50 | 2.92 | 33.00 | 9.24 | 31.50 | 0.64 | 63.00 | 0.01 | 31.49 | 0.67 | 63.00 | 9.35 |
| 31.57 | 4.55 | 31.58 | 7.94 | 63.17 | 6.57 | 31.58 | 3.19 | 33.08 | 9.27 | 31.58 | 0.62 | 63.17 | 0.03 | 31.58 | 0.68 | 63.17 | 9.24 |
| 31.65 | 4.46 | 31.67 | 7.68 | 63.33 | 6.65 | 31.66 | 3.01 | 33.16 | 9.25 | 31.66 | 0.61 | 63.33 | 0.02 | 31.66 | 0.67 | 63.33 | 9.29 |
| 31.74 | 4.44 | 31.75 | 7.75 | 63.50 | 6.62 | 31.75 | 3.10 | 33.25 | 9.30 | 31.75 | 0.60 | 63.50 | -0.06 | 31.74 | 0.63 | 63.50 | 9.24 |
| 31.82 | 4.47 | 31.83 | 7.80 | 63.67 | 6.70 | 31.83 | 2.99 | 33.33 | 9.41 | 31.83 | 0.63 | 63.67 | 0.06 | 31.83 | 0.60 | 63.67 | 9.35 |
| 31.90 | 4.47 | 31.92 | 7.80 | 63.83 | 6.56 | 31.91 | 2.93 | 33.41 | 9.44 | 31.91 | 0.62 | 63.83 | 0.03 | 31.91 | 0.62 | 63.83 | 9.33 |
| 31.99 | 4.54 | 32.00 | 7.89 | 64.00 | 6.61 | 32.00 | 2.98 | 33.50 | 9.38 | 32.00 | 0.63 | 64.00 | -0.01 | 31.99 | 0.68 | 64.00 | 9.31 |
| 32.07 | 4.60 | 32.08 | 7.74 | 64.17 | 6.71 | 32.08 | 3.18 | 33.58 | 9.45 | 32.08 | 0.61 | 64.17 | 0.07 | 32.08 | 0.72 | 64.17 | 9.27 |
| 32.15 | 4.62 | 32.17 | 7.75 | 64.33 | 6.54 | 32.16 | 3.07 | 33.66 | 9.36 | 32.16 | 0.61 | 64.33 | 0.03 | 32.16 | 0.73 | 64.33 | 9.28 |
| 32.24 | 4.66 | 32.25 | 7.90 | 64.50 | 6.61 | 32.25 | 3.09 | 33.75 | 9.31 | 32.25 | 0.60 | 64.50 | 0.01 | 32.24 | 0.70 | 64.50 | 9.33 |
| 32.32 | 4.71 | 32.33 | 7.79 | 64.67 | 6.57 | 32.33 | 3.22 | 33.83 | 9.34 | 32.33 | 0.62 | 64.67 | 0.05 | 32.33 | 0.65 | 64.67 | 9.33 |
| 32.40 | 4.60 | 32.42 | 7.77 | 64.83 | 6.73 | 32.41 | 3.27 | 33.91 | 9.39 | 32.41 | 0.63 | 64.83 | 0.13 | 32.41 | 0.73 | 64.83 | 9.31 |
| 32.49 | 4.64 | 32.50 | 7.81 | 65.00 | 6.59 | 32.50 | 3.34 | 34.00 | 9.51 | 32.50 | 0.62 | 65.00 | 0.03 | 32.49 | 0.77 | 65.00 | 9.41 |
| 32.57 | 4.64 | 32.58 | 7.77 | 65.17 | 6.61 | 32.58 | 3.15 | 34.08 | 9.56 | 32.58 | 0.70 | 65.17 | 0.04 | 32.58 | 0.78 | 65.17 | 9.43 |
| 32.65 | 4.60 | 32.67 | 7.85 | 65.33 | 6.69 | 32.66 | 3.34 | 34.16 | 9.60 | 32.66 | 0.71 | 65.33 | 0.12 | 32.66 | 0.78 | 65.33 | 9.39 |

| | | | | | | | | | | | | | | | | | |
|---|---|---|---|---|---|---|---|---|---|---|---|---|---|---|---|---|---|
| 32.74 | 4.74 | 32.75 | 7.81 | 65.50 | 6.63 | 32.75 | 3.18 | 34.25 | 9.44 | 32.75 | 0.68 | 65.50 | 0.07 | 32.74 | 0.81 | 65.50 | 9.44 |
| 32.82 | 4.60 | 32.83 | 7.81 | 65.67 | 6.68 | 32.83 | 3.17 | 34.33 | 9.57 | 32.83 | 0.68 | 65.67 | 0.12 | 32.83 | 0.80 | 65.67 | 9.46 |
| 32.90 | 4.60 | 32.92 | 7.82 | 65.83 | 6.65 | 32.91 | 3.19 | 34.41 | 9.51 | 32.91 | 0.73 | 65.83 | 0.15 | 32.91 | 0.81 | 65.83 | 9.44 |
| 32.99 | 4.60 | 33.00 | 7.82 | 66.00 | 6.70 | 33.00 | 3.38 | 34.50 | 9.56 | 33.00 | 0.74 | 66.00 | 0.07 | 32.99 | 0.81 | 66.00 | 9.50 |
| 33.07 | 4.75 | 33.08 | 7.88 | 66.17 | 6.79 | 33.08 | 3.15 | 34.58 | 9.59 | 33.08 | 0.75 | 66.17 | 0.08 | 33.08 | 0.89 | 66.17 | 9.54 |
| 33.15 | 4.66 | 33.17 | 7.90 | 66.33 | 6.77 | 33.16 | 3.40 | 34.66 | 9.69 | 33.16 | 0.76 | 66.33 | 0.25 | 33.16 | 0.83 | 66.33 | 9.53 |
| 33.24 | 4.72 | 33.25 | 7.95 | 66.50 | 6.74 | 33.25 | 3.13 | 34.75 | 9.63 | 33.25 | 0.76 | 66.50 | 0.20 | 33.24 | 0.83 | 66.50 | 9.54 |
| 33.32 | 4.68 | 33.33 | 8.00 | 66.67 | 6.78 | 33.33 | 3.22 | 34.83 | 9.56 | 33.33 | 0.76 | 66.67 | 0.15 | 33.33 | 0.75 | 66.67 | 9.59 |
| 33.40 | 4.79 | 33.42 | 8.01 | 66.83 | 6.81 | 33.41 | 3.21 | 34.91 | 9.61 | 33.41 | 0.75 | 66.83 | 0.25 | 33.41 | 0.75 | 66.83 | 9.57 |
| 33.49 | 4.90 | 33.50 | 8.04 | 67.00 | 6.84 | 33.50 | 3.26 | 35.00 | 9.76 | 33.50 | 0.77 | 67.00 | 0.22 | 33.49 | 0.83 | 67.00 | 9.55 |
| 33.57 | 4.67 | 33.58 | 8.08 | 67.17 | 6.92 | 33.58 | 3.25 | 35.08 | 9.76 | 33.58 | 0.76 | 67.17 | 0.17 | 33.58 | 0.88 | 67.17 | 9.62 |
| 33.65 | 4.68 | 33.67 | 8.17 | 67.33 | 6.90 | 33.66 | 3.13 | 35.16 | 9.76 | 33.66 | 0.77 | 67.33 | 0.25 | 33.66 | 0.87 | 67.33 | 9.66 |
| 33.74 | 4.73 | 33.75 | 8.21 | 67.50 | 6.95 | 33.75 | 3.15 | 35.25 | 9.77 | 33.75 | 0.76 | 67.50 | 0.22 | 33.74 | 0.82 | 67.50 | 9.62 |
| 33.82 | 4.91 | 33.83 | 8.14 | 67.67 | 6.91 | 33.83 | 3.18 | 35.33 | 9.73 | 33.83 | 0.78 | 67.67 | 0.24 | 33.83 | 0.84 | 67.67 | 9.72 |
| 33.90 | 4.66 | 33.92 | 8.14 | 67.83 | 6.94 | 33.91 | 3.28 | 35.41 | 9.72 | 33.91 | 0.74 | 67.83 | 0.28 | 33.91 | 0.78 | 67.83 | 9.70 |
| 33.99 | 4.68 | 34.00 | 8.17 | 68.00 | 6.96 | 34.00 | 3.42 | 35.50 | 9.76 | 34.00 | 0.74 | 68.00 | 0.29 | 33.99 | 0.89 | 68.00 | 9.76 |
| 34.07 | 4.71 | 34.08 | 8.21 | 68.17 | 6.99 | 34.08 | 3.41 | 35.58 | 9.85 | 34.08 | 0.76 | 68.17 | 0.27 | 34.08 | 0.90 | 68.17 | 9.67 |
| 34.15 | 4.73 | 34.17 | 8.21 | 68.33 | 7.08 | 34.16 | 3.49 | 35.66 | 9.87 | 34.16 | 0.78 | 68.33 | 0.28 | 34.16 | 0.91 | 68.33 | 9.72 |
| 34.24 | 4.78 | 34.25 | 8.26 | 68.50 | 7.05 | 34.25 | 3.34 | 35.75 | 9.87 | 34.25 | 0.79 | 68.50 | 0.23 | 34.24 | 0.90 | 68.50 | 9.75 |
| 34.32 | 4.79 | 34.33 | 8.34 | 68.67 | 7.11 | 34.33 | 3.52 | 35.83 | 9.90 | 34.33 | 0.79 | 68.67 | 0.24 | 34.33 | 0.88 | 68.67 | 9.75 |
| 34.40 | 4.78 | 34.42 | 8.42 | 68.83 | 7.04 | 34.41 | 3.42 | 35.91 | 9.95 | 34.41 | 0.79 | 68.83 | 0.31 | 34.41 | 0.87 | 68.83 | 9.75 |
| 34.49 | 4.71 | 34.50 | 8.38 | 69.00 | 7.03 | 34.50 | 3.67 | 36.00 | 9.93 | 34.50 | 0.79 | 69.00 | 0.28 | 34.49 | 0.90 | 69.00 | 9.82 |
| 34.57 | 4.90 | 34.58 | 8.31 | 69.17 | 7.09 | 34.58 | 3.61 | 36.08 | 9.83 | 34.58 | 0.77 | 69.17 | 0.25 | 34.58 | 0.87 | 69.17 | 9.83 |
| 34.65 | 4.78 | 34.67 | 8.31 | 69.33 | 7.05 | 34.66 | 3.44 | 36.16 | 9.85 | 34.66 | 0.78 | 69.33 | 0.33 | 34.66 | 0.91 | 69.33 | 9.82 |
| 34.74 | 4.86 | 34.75 | 8.31 | 69.50 | 7.08 | 34.75 | 3.47 | 36.25 | 9.86 | 34.75 | 0.80 | 69.50 | 0.29 | 34.74 | 0.93 | 69.50 | 9.84 |
| 34.82 | 4.93 | 34.83 | 8.45 | 69.67 | 7.13 | 34.83 | 3.75 | 36.33 | 9.90 | 34.83 | 0.80 | 69.67 | 0.28 | 34.83 | 0.94 | 69.67 | 9.85 |
| 34.90 | 4.97 | 34.92 | 8.27 | 69.83 | 7.13 | 34.91 | 3.42 | 36.41 | 9.78 | 34.91 | 0.79 | 69.83 | 0.34 | 34.91 | 0.97 | 69.83 | 9.86 |
| 34.99 | 4.99 | 35.00 | 8.44 | 70.00 | 7.20 | 35.00 | 3.63 | 36.50 | 9.79 | 35.00 | 0.80 | 70.00 | 0.32 | 34.99 | 0.96 | 70.00 | 9.88 |
| 35.07 | 4.90 | 35.08 | 8.27 | 70.17 | 7.19 | 35.08 | 3.68 | 36.58 | 9.88 | 35.08 | 0.78 | 70.17 | 0.26 | 35.08 | 0.95 | 70.17 | 9.96 |
| 35.15 | 5.02 | 35.17 | 8.33 | 70.33 | 7.23 | 35.16 | 3.77 | 36.66 | 9.94 | 35.16 | 0.79 | 70.33 | 0.35 | 35.16 | 0.92 | 70.33 | 9.89 |
| 35.24 | 4.86 | 35.25 | 8.30 | 70.50 | 7.27 | 35.25 | 3.83 | 36.75 | 9.93 | 35.25 | 0.79 | 70.50 | 0.36 | 35.24 | 0.93 | 70.50 | 9.97 |
| 35.32 | 4.94 | 35.33 | 8.38 | 70.67 | 7.35 | 35.33 | 3.90 | 36.83 | 9.95 | 35.33 | 0.80 | 70.67 | 0.31 | 35.33 | 0.97 | 70.67 | 10.02 |
| 35.40 | 5.03 | 35.42 | 8.32 | 70.83 | 7.39 | 35.41 | 3.70 | 36.91 | 10.05 | 35.41 | 0.80 | 70.83 | 0.38 | 35.41 | 0.96 | 70.83 | 9.98 |
| 35.49 | 5.11 | 35.50 | 8.30 | 71.00 | 7.39 | 35.50 | 3.89 | 37.00 | 10.08 | 35.50 | 0.80 | 71.00 | 0.39 | 35.49 | 1.03 | 71.00 | 9.99 |
| 35.57 | 5.14 | 35.58 | 8.30 | 71.17 | 7.46 | 35.58 | 3.71 | 37.08 | 9.99 | 35.58 | 0.78 | 71.17 | 0.33 | 35.58 | 1.01 | 71.17 | 10.12 |
| 35.65 | 5.01 | 35.67 | 8.46 | 71.33 | 7.55 | 35.66 | 3.72 | 37.16 | 10.00 | 35.66 | 0.77 | 71.33 | 0.35 | 35.66 | 0.97 | 71.33 | 10.10 |
| 35.74 | 5.17 | 35.75 | 8.31 | 71.50 | 7.51 | 35.75 | 3.87 | 37.25 | 10.13 | 35.75 | 0.78 | 71.50 | 0.42 | 35.74 | 0.94 | 71.50 | 10.12 |
| 35.82 | 5.01 | 35.83 | 8.36 | 71.67 | 7.48 | 35.83 | 3.72 | 37.33 | 10.11 | 35.83 | 0.81 | 71.67 | 0.39 | 35.83 | 1.02 | 71.67 | 10.11 |
| 35.90 | 5.05 | 35.92 | 8.43 | 71.83 | 7.49 | 35.91 | 3.72 | 37.41 | 10.16 | 35.91 | 0.80 | 71.83 | 0.34 | 35.91 | 1.06 | 71.83 | 10.15 |
| 35.99 | 5.04 | 36.00 | 8.47 | 72.00 | 7.53 | 36.00 | 3.92 | 37.50 | 10.03 | 36.00 | 0.80 | 72.00 | 0.44 | 35.99 | 1.05 | 72.00 | 10.07 |
| 36.07 | 5.04 | 36.08 | 8.53 | 72.17 | 7.51 | 36.08 | 3.68 | 37.58 | 9.93 | 36.08 | 0.78 | 72.17 | 0.41 | 36.08 | 1.05 | 72.17 | 10.10 |
| 36.15 | 5.19 | 36.17 | 8.56 | 72.33 | 7.51 | 36.16 | 3.75 | 37.66 | 10.00 | 36.16 | 0.78 | 72.33 | 0.44 | 36.16 | 1.01 | 72.33 | 10.11 |
| 36.24 | 5.01 | 36.25 | 8.53 | 72.50 | 7.53 | 36.25 | 3.75 | 37.75 | 10.01 | 36.25 | 0.79 | 72.50 | 0.45 | 36.24 | 1.00 | 72.50 | 10.16 |
| 36.32 | 5.03 | 36.33 | 8.62 | 72.67 | 7.53 | 36.33 | 3.74 | 37.83 | 10.08 | 36.33 | 0.80 | 72.67 | 0.45 | 36.33 | 1.04 | 72.67 | 10.10 |
| 36.40 | 5.05 | 36.42 | 8.68 | 72.83 | 7.62 | 36.41 | 3.93 | 37.91 | 10.19 | 36.41 | 0.80 | 72.83 | 0.47 | 36.41 | 1.07 | 72.83 | 10.12 |
| 36.49 | 5.10 | 36.50 | 8.70 | 73.00 | 7.64 | 36.50 | 3.70 | 38.00 | 10.20 | 36.50 | 0.81 | 73.00 | 0.45 | 36.49 | 1.12 | 73.00 | 10.12 |
| 36.57 | 5.15 | 36.58 | 8.76 | 73.17 | 7.67 | 36.58 | 3.95 | 38.08 | 10.18 | 36.58 | 0.78 | 73.17 | 0.43 | 36.58 | 1.07 | 73.17 | 10.13 |
| 36.65 | 5.19 | 36.67 | 8.66 | 73.33 | 7.69 | 36.66 | 3.81 | 38.16 | 10.04 | 36.66 | 0.78 | 73.33 | 0.43 | 36.66 | 1.08 | 73.33 | 10.18 |
| 36.74 | 5.26 | 36.75 | 8.70 | 73.50 | 7.77 | 36.75 | 3.96 | 38.25 | 10.01 | 36.75 | 0.79 | 73.50 | 0.45 | 36.74 | 1.04 | 73.50 | 10.22 |
| 36.82 | 5.32 | 36.83 | 8.76 | 73.67 | 7.76 | 36.83 | 4.02 | 38.33 | 10.05 | 36.83 | 0.80 | 73.67 | 0.47 | 36.83 | 1.05 | 73.67 | 10.22 |
| 36.90 | 5.18 | 36.92 | 8.89 | 73.83 | 7.76 | 36.91 | 4.01 | 38.41 | 10.10 | 36.91 | 0.80 | 73.83 | 0.42 | 36.91 | 1.11 | 73.83 | 10.22 |

| | | | | | | | | | | | | | | | | | |
|---|---|---|---|---|---|---|---|---|---|---|---|---|---|---|---|---|---|
| 36.99 | 5.20 | 37.00 | 8.95 | 74.00 | 7.84 | 37.00 | 3.90 | 38.50 | 10.19 | 37.00 | 0.80 | 74.00 | 0.53 | 36.99 | 1.13 | 74.00 | 10.24 |
| 37.07 | 5.28 | 37.08 | 8.79 | 74.17 | 7.83 | 37.08 | 3.90 | 38.58 | 10.24 | 37.08 | 0.78 | 74.17 | 0.50 | 37.08 | 1.16 | 74.17 | 10.26 |
| 37.15 | 5.38 | 37.17 | 8.90 | 74.33 | 7.86 | 37.16 | 4.09 | 38.66 | 10.27 | 37.16 | 0.78 | 74.33 | 0.51 | 37.16 | 1.11 | 74.33 | 10.27 |
| 37.24 | 5.43 | 37.25 | 8.82 | 74.50 | 7.98 | 37.25 | 4.08 | 38.75 | 10.27 | 37.25 | 0.79 | 74.50 | 0.54 | 37.24 | 1.12 | 74.50 | 10.33 |
| 37.32 | 5.52 | 37.33 | 8.79 | 74.67 | 7.92 | 37.33 | 4.20 | 38.83 | 10.16 | 37.33 | 1.46 | 74.67 | 0.53 | 37.33 | 1.16 | 74.67 | 10.37 |
| 37.40 | 5.48 | 37.42 | 8.81 | 74.83 | 7.95 | 37.41 | 4.11 | 38.91 | 10.19 | 37.41 | 1.62 | 74.83 | 0.53 | 37.41 | 1.18 | 74.83 | 10.30 |
| 37.49 | 5.48 | 37.50 | 8.85 | 75.00 | 8.04 | 37.50 | 4.23 | 39.00 | 10.21 | 37.50 | 2.35 | 75.00 | 0.57 | 37.49 | 1.19 | 75.00 | 10.38 |
| 37.57 | 5.63 | 37.58 | 8.92 | 75.17 | 7.91 | 37.58 | 4.00 | 39.08 | 10.26 | 37.58 | 2.76 | 75.17 | 0.54 | 37.58 | 1.19 | 75.17 | 10.41 |
| 37.65 | 5.69 | 37.67 | 8.81 | 75.33 | 8.02 | 37.66 | 4.02 | 39.16 | 10.20 | 37.66 | 3.24 | 75.33 | 0.54 | 37.66 | 1.20 | 75.33 | 10.42 |
| 37.74 | 5.60 | 37.75 | 8.82 | 75.50 | 7.92 | 37.75 | 4.03 | 39.25 | 10.30 | 37.75 | 3.08 | 75.50 | 0.61 | 37.74 | 1.18 | 75.50 | 10.44 |
| 37.82 | 5.75 | 37.83 | 8.81 | 75.67 | 7.92 | 37.83 | 4.10 | 39.33 | 10.16 | 37.83 | 3.14 | 75.67 | 0.59 | 37.83 | 1.12 | 75.67 | 10.52 |
| 37.90 | 5.58 | 37.92 | 9.01 | 75.83 | 7.95 | 37.91 | 4.14 | 39.41 | 10.29 | 37.91 | 3.37 | 75.83 | 0.56 | 37.91 | 1.18 | 75.83 | 10.53 |
| 37.99 | 5.58 | 38.00 | 8.88 | 76.00 | 7.93 | 38.00 | 4.13 | 39.50 | 10.21 | 38.00 | 3.27 | 76.00 | 0.65 | 37.99 | 1.18 | 76.00 | 10.49 |
| 38.07 | 5.59 | 38.08 | 8.93 | 76.17 | 7.97 | 38.08 | 4.15 | 39.58 | 10.19 | 38.08 | 3.19 | 76.17 | 0.63 | 38.08 | 1.19 | 76.17 | 10.56 |
| 38.15 | 5.64 | 38.17 | 8.93 | 76.33 | 7.97 | 38.16 | 4.22 | 39.66 | 10.29 | 38.16 | 3.22 | 76.33 | 0.59 | 38.16 | 1.18 | 76.33 | 10.50 |
| 38.24 | 5.66 | 38.25 | 8.97 | 76.50 | 7.91 | 38.25 | 4.04 | 39.75 | 10.19 | 38.25 | 3.15 | 76.50 | 0.64 | 38.24 | 1.17 | 76.50 | 10.55 |
| 38.32 | 5.62 | 38.33 | 8.97 | 76.67 | 7.98 | 38.33 | 4.26 | 39.83 | 10.21 | 38.33 | 3.14 | 76.67 | 0.68 | 38.33 | 1.19 | 76.67 | 10.51 |
| 38.40 | 5.79 | 38.42 | 9.00 | 76.83 | 8.08 | 38.41 | 4.23 | 39.91 | 10.26 | 38.41 | 3.13 | 76.83 | 0.65 | 38.41 | 1.18 | 76.83 | 10.50 |
| 38.49 | 5.57 | 38.50 | 9.01 | 77.00 | 8.09 | 38.50 | 4.30 | 40.00 | 10.19 | 38.50 | 3.13 | 77.00 | 0.72 | 38.49 | 1.18 | 77.00 | 10.55 |
| 38.57 | 5.59 | 38.58 | 9.06 | 77.17 | 8.05 | 38.58 | 4.33 | 40.08 | 10.19 | 38.58 | 3.20 | 77.17 | 0.72 | 38.58 | 1.19 | 77.17 | 10.50 |
| 38.65 | 5.63 | 38.67 | 9.06 | 77.33 | 8.13 | 38.66 | 4.39 | 40.16 | 10.27 | 38.66 | 3.25 | 77.33 | 0.67 | 38.66 | 1.20 | 77.33 | 10.52 |
| 38.74 | 5.68 | 38.75 | 9.02 | 77.50 | 8.17 | 38.75 | 4.29 | 40.25 | 10.18 | 38.75 | 3.19 | 77.50 | 0.74 | 38.74 | 1.20 | 77.50 | 10.55 |
| 38.82 | 5.76 | 38.83 | 9.10 | 77.67 | 8.21 | 38.83 | 4.45 | 40.33 | 10.27 | 38.83 | 3.25 | 77.67 | 0.78 | 38.83 | 1.20 | 77.67 | 10.54 |
| 38.90 | 5.79 | 38.92 | 9.04 | 77.83 | 8.28 | 38.91 | 4.25 | 40.41 | 10.18 | 38.91 | 3.35 | 77.83 | 0.72 | 38.91 | 1.19 | 77.83 | 10.56 |
| 38.99 | 5.79 | 39.00 | 9.05 | 78.00 | 8.32 | 39.00 | 4.27 | 40.50 | 10.18 | 39.00 | 3.38 | 78.00 | 0.74 | 38.99 | 1.17 | 78.00 | 10.60 |
| 39.07 | 5.82 | 39.08 | 9.04 | 78.17 | 8.39 | 39.08 | 4.31 | 40.58 | 10.19 | 39.08 | 3.28 | 78.17 | 0.79 | 39.08 | 1.18 | 78.17 | 10.61 |
| 39.15 | 5.89 | 39.17 | 9.06 | 78.33 | 8.45 | 39.16 | 4.30 | 40.66 | 10.25 | 39.16 | 3.28 | 78.33 | 0.73 | 39.16 | 1.18 | 78.33 | 10.64 |
| 39.24 | 5.81 | 39.25 | 9.10 | 78.50 | 8.41 | 39.25 | 4.48 | 40.75 | 10.27 | 39.25 | 3.41 | 78.50 | 0.76 | 39.24 | 1.19 | 78.50 | 10.64 |
| 39.32 | 5.91 | 39.33 | 9.12 | 78.67 | 8.40 | 39.33 | 4.28 | 40.83 | 10.32 | 39.33 | 3.30 | 78.67 | 0.83 | 39.33 | 1.19 | 78.67 | 10.70 |
| 39.40 | 5.99 | 39.42 | 9.11 | 78.83 | 8.34 | 39.41 | 4.45 | 40.91 | 10.22 | 39.41 | 3.27 | 78.83 | 0.81 | 39.41 | 1.20 | 78.83 | 10.70 |
| 39.49 | 6.02 | 39.50 | 9.14 | 79.00 | 8.42 | 39.50 | 4.24 | 41.00 | 10.36 | 39.50 | 3.28 | 79.00 | 0.79 | 39.49 | 1.18 | 79.00 | 10.72 |
| 39.57 | 5.89 | 39.58 | 9.21 | 79.17 | 8.46 | 39.58 | 4.30 | 41.08 | 10.35 | 39.58 | 3.40 | 79.17 | 0.84 | 39.58 | 1.18 | 79.17 | 10.77 |
| 39.65 | 5.88 | 39.67 | 9.25 | 79.33 | 8.47 | 39.66 | 4.38 | 41.16 | 10.37 | 39.66 | 3.31 | 79.33 | 0.84 | 39.66 | 1.15 | 79.33 | 10.75 |
| 39.74 | 5.90 | 39.75 | 9.25 | 79.50 | 8.51 | 39.75 | 4.45 | 41.25 | 10.43 | 39.75 | 3.28 | 79.50 | 0.85 | 39.74 | 1.19 | 79.50 | 10.79 |
| 39.82 | 5.91 | 39.83 | 9.28 | 79.67 | 8.57 | 39.83 | 4.43 | 41.33 | 10.42 | 39.83 | 3.28 | 79.67 | 0.86 | 39.83 | 1.19 | 79.67 | 10.86 |
| 39.90 | 5.94 | 39.92 | 9.33 | 79.83 | 8.61 | 39.91 | 4.39 | 41.41 | 10.43 | 39.91 | 3.29 | 79.83 | 0.86 | 39.91 | 1.19 | 79.83 | 10.79 |
| 39.99 | 6.05 | 40.00 | 9.36 | 80.00 | 8.65 | 40.00 | 4.54 | 41.50 | 10.39 | 40.00 | 3.43 | 80.00 | 0.87 | 39.99 | 1.18 | 80.00 | 10.80 |
| 40.07 | 5.91 | 40.08 | 9.45 | 80.17 | 8.71 | 40.08 | 4.48 | 41.58 | 10.42 | 40.08 | 3.30 | 80.17 | 0.86 | 40.08 | 1.19 | 80.17 | 10.88 |
| 40.15 | 5.91 | 40.17 | 9.43 | 80.33 | 8.74 | 40.16 | 4.46 | 41.66 | 10.51 | 40.16 | 3.29 | 80.33 | 0.86 | 40.16 | 1.15 | 80.33 | 10.88 |
| 40.24 | 5.91 | 40.25 | 9.41 | 80.50 | 8.87 | 40.25 | 4.51 | 41.75 | 10.57 | 40.25 | 3.43 | 80.50 | 0.89 | 40.24 | 1.18 | 80.50 | 10.87 |
| 40.32 | 5.92 | 40.33 | 9.35 | 80.67 | 8.86 | 40.33 | 4.64 | 41.83 | 10.59 | 40.33 | 3.31 | 80.67 | 0.86 | 40.33 | 1.18 | 80.67 | 10.95 |
| 40.40 | 5.95 | 40.42 | 9.31 | 80.83 | 8.88 | 40.41 | 4.45 | 41.91 | 10.63 | 40.41 | 3.35 | 80.83 | 0.80 | 40.41 | 1.18 | 80.83 | 10.90 |
| 40.49 | 5.96 | 40.50 | 9.30 | 81.00 | 8.97 | 40.50 | 4.65 | 42.00 | 10.58 | 40.50 | 3.43 | 81.00 | 0.84 | 40.49 | 1.18 | 81.00 | 10.91 |
| 40.57 | 5.97 | 40.58 | 9.55 | 81.17 | 9.02 | 40.58 | 4.52 | 42.08 | 10.63 | 40.58 | 3.49 | 81.17 | 0.86 | 40.58 | 1.16 | 81.17 | 10.99 |
| 40.65 | 6.02 | 40.67 | 9.30 | 81.33 | 9.01 | 40.66 | 4.73 | 42.16 | 10.61 | 40.66 | 3.57 | 81.33 | 0.87 | 40.66 | 1.17 | 81.33 | 10.95 |
| 40.74 | 6.07 | 40.75 | 9.41 | 81.50 | 9.05 | 40.75 | 4.66 | 42.25 | 10.76 | 40.75 | 3.54 | 81.50 | 0.87 | 40.74 | 1.17 | 81.50 | 10.96 |
| 40.82 | 6.11 | 40.83 | 9.45 | 81.67 | 9.02 | 40.83 | 4.63 | 42.33 | 10.77 | 40.83 | 3.38 | 81.67 | 0.93 | 40.83 | 1.17 | 81.67 | 10.95 |
| 40.90 | 6.19 | 40.92 | 9.51 | 81.83 | 9.11 | 40.91 | 4.81 | 42.41 | 10.75 | 40.91 | 3.45 | 81.83 | 0.91 | 40.91 | 1.25 | 81.83 | 10.95 |
| 40.99 | 5.99 | 41.00 | 9.51 | 82.00 | 9.20 | 41.00 | 4.83 | 42.50 | 10.73 | 41.00 | 3.53 | 82.00 | 0.92 | 40.99 | 1.25 | 82.00 | 11.00 |
| 41.07 | 6.01 | 41.08 | 9.50 | 82.17 | 9.18 | 41.08 | 4.67 | 42.58 | 10.73 | 41.08 | 3.60 | 82.17 | 1.07 | 41.08 | 1.26 | 82.17 | 11.04 |
| 41.15 | 6.03 | 41.17 | 9.46 | 82.33 | 9.26 | 41.16 | 4.54 | 42.66 | 10.77 | 41.16 | 3.67 | 82.33 | 0.96 | 41.16 | 1.22 | 82.33 | 11.08 |

| | | | | | | | | | | | | | | | | | |
|---|---|---|---|---|---|---|---|---|---|---|---|---|---|---|---|---|---|
| 41.24 | 6.26 | 41.25 | 9.53 | 82.50 | 9.29 | 41.25 | 4.64 | 42.75 | 10.76 | 41.25 | 3.66 | 82.50 | 0.96 | 41.24 | 1.23 | 82.50 | 11.04 |
| 41.32 | 6.05 | 41.33 | 9.63 | 82.67 | 9.30 | 41.33 | 4.67 | 42.83 | 10.82 | 41.33 | 3.71 | 82.67 | 1.06 | 41.33 | 1.30 | 82.67 | 11.09 |
| 41.40 | 6.04 | 41.42 | 9.68 | 82.83 | 9.35 | 41.41 | 4.74 | 42.91 | 10.85 | 41.41 | 3.59 | 82.83 | 1.05 | 41.41 | 1.31 | 82.83 | 11.11 |
| 41.49 | 6.23 | 41.50 | 9.59 | 83.00 | 9.29 | 41.50 | 4.69 | 43.00 | 10.90 | 41.50 | 3.62 | 83.00 | 1.00 | 41.49 | 1.33 | 83.00 | 11.14 |
| 41.57 | 6.03 | 41.58 | 9.58 | 83.17 | 9.30 | 41.58 | 4.66 | 43.08 | 10.95 | 41.58 | 3.61 | 83.17 | 1.09 | 41.58 | 1.32 | 83.17 | 11.15 |
| 41.65 | 6.04 | 41.67 | 9.53 | 83.33 | 9.29 | 41.66 | 4.84 | 43.16 | 10.86 | 41.66 | 3.62 | 83.33 | 1.09 | 41.66 | 1.31 | 83.33 | 11.14 |
| 41.74 | 6.03 | 41.75 | 9.54 | 83.50 | 9.37 | 41.75 | 4.74 | 43.25 | 10.93 | 41.75 | 3.72 | 83.50 | 1.07 | 41.74 | 1.30 | 83.50 | 11.18 |
| 41.82 | 6.25 | 41.83 | 9.67 | 83.67 | 9.32 | 41.83 | 4.79 | 43.33 | 10.83 | 41.83 | 3.62 | 83.67 | 1.12 | 41.83 | 1.32 | 83.67 | 11.22 |
| 41.90 | 6.08 | 41.92 | 9.53 | 83.83 | 9.36 | 41.91 | 4.86 | 43.41 | 10.97 | 41.91 | 3.63 | 83.83 | 1.16 | 41.91 | 1.32 | 83.83 | 11.21 |
| 41.99 | 6.05 | 42.00 | 9.60 | 84.00 | 9.37 | 42.00 | 4.90 | 43.50 | 10.85 | 42.00 | 3.64 | 84.00 | 1.11 | 41.99 | 1.34 | 84.00 | 11.20 |
| 42.07 | 6.08 | 42.08 | 9.60 | 84.17 | 9.42 | 42.08 | 4.97 | 43.58 | 10.95 | 42.08 | 3.63 | 84.17 | 1.12 | 42.08 | 1.34 | 84.17 | 11.30 |
| 42.15 | 6.08 | 42.17 | 9.65 | 84.33 | 9.46 | 42.16 | 4.99 | 43.66 | 10.86 | 42.16 | 3.64 | 84.33 | 1.16 | 42.16 | 1.31 | 84.33 | 11.23 |
| 42.24 | 6.10 | 42.25 | 9.65 | 84.50 | 9.47 | 42.25 | 4.76 | 43.75 | 10.86 | 42.25 | 3.70 | 84.50 | 1.13 | 42.24 | 1.33 | 84.50 | 11.27 |
| 42.32 | 6.06 | 42.33 | 9.62 | 84.67 | 9.53 | 42.33 | 4.80 | 43.83 | 10.97 | 42.33 | 3.65 | 84.67 | 1.13 | 42.33 | 1.33 | 84.67 | 11.37 |
| 42.40 | 6.03 | 42.42 | 9.76 | 84.83 | 9.60 | 42.41 | 4.87 | 43.91 | 10.85 | 42.41 | 3.68 | 84.83 | 1.14 | 42.41 | 1.32 | 84.83 | 11.32 |
| 42.49 | 6.26 | 42.50 | 9.78 | 85.00 | 9.56 | 42.50 | 4.85 | 44.00 | 10.89 | 42.50 | 3.77 | 85.00 | 1.11 | 42.49 | 1.33 | 85.00 | 11.31 |
| 42.57 | 6.12 | 42.58 | 9.79 | 85.17 | 9.61 | 42.58 | 5.00 | 44.08 | 10.88 | 42.58 | 3.84 | 85.17 | 1.14 | 42.58 | 1.35 | 85.17 | 11.34 |
| 42.65 | 6.17 | 42.67 | 9.84 | 85.33 | 9.70 | 42.66 | 4.83 | 44.16 | 10.96 | 42.66 | 3.87 | 85.33 | 1.12 | 42.66 | 1.41 | 85.33 | 11.41 |
| 42.74 | 6.28 | 42.75 | 9.84 | 85.50 | 9.65 | 42.75 | 4.83 | 44.25 | 10.86 | 42.75 | 3.79 | 85.50 | 1.11 | 42.74 | 1.40 | 85.50 | 11.36 |
| 42.82 | 6.33 | 42.83 | 9.83 | 85.67 | 9.76 | 42.83 | 5.02 | 44.33 | 10.88 | 42.83 | 3.78 | 85.67 | 1.20 | 42.83 | 1.43 | 85.67 | 11.32 |
| 42.90 | 6.17 | 42.92 | 9.83 | 85.83 | 9.71 | 42.91 | 4.81 | 44.41 | 10.95 | 42.91 | 3.77 | 85.83 | 1.17 | 42.91 | 1.43 | 85.83 | 11.34 |
| 42.99 | 6.35 | 43.00 | 9.88 | 86.00 | 9.76 | 43.00 | 4.86 | 44.50 | 10.88 | 43.00 | 3.91 | 86.00 | 1.13 | 42.99 | 1.43 | 86.00 | 11.37 |
| 43.07 | 6.15 | 43.08 | 10.01 | 86.17 | 9.69 | 43.08 | 4.86 | 44.58 | 10.93 | 43.08 | 3.76 | 86.17 | 1.23 | 43.08 | 1.45 | 86.17 | 11.38 |
| 43.15 | 6.21 | 43.17 | 10.04 | 86.33 | 9.72 | 43.16 | 4.86 | 44.66 | 10.88 | 43.16 | 3.79 | 86.33 | 1.16 | 43.16 | 1.46 | 86.33 | 11.36 |
| 43.24 | 6.26 | 43.25 | 9.83 | 86.50 | 9.78 | 43.25 | 5.02 | 44.75 | 10.91 | 43.25 | 3.83 | 86.50 | 1.14 | 43.24 | 1.48 | 86.50 | 11.41 |
| 43.32 | 6.32 | 43.33 | 9.93 | 86.67 | 9.72 | 43.33 | 4.79 | 44.83 | 10.93 | 43.33 | 3.87 | 86.67 | 1.25 | 43.33 | 1.46 | 86.67 | 11.44 |
| 43.40 | 6.34 | 43.42 | 9.96 | 86.83 | 9.82 | 43.41 | 4.81 | 44.91 | 10.99 | 43.41 | 3.92 | 86.83 | 2.05 | 43.41 | 1.46 | 86.83 | 11.47 |
| 43.49 | 6.22 | 43.50 | 9.92 | 87.00 | 9.77 | 43.50 | 4.91 | 45.00 | 11.08 | 43.50 | 4.00 | 87.00 | 3.01 | 43.49 | 1.46 | 87.00 | 11.45 |
| 43.57 | 6.20 | 43.58 | 9.97 | 87.17 | 9.89 | 43.58 | 4.98 | 45.08 | 11.03 | 43.58 | 4.07 | 87.17 | 3.69 | 43.58 | 1.46 | 87.17 | 11.49 |
| 43.65 | 6.18 | 43.67 | 9.89 | 87.33 | 9.83 | 43.66 | 5.08 | 45.16 | 11.05 | 43.66 | 3.88 | 87.33 | 3.69 | 43.66 | 1.47 | 87.33 | 11.51 |
| 43.74 | 6.25 | 43.75 | 9.87 | 87.50 | 9.91 | 43.75 | 5.13 | 45.25 | 11.05 | 43.75 | 3.95 | 87.50 | 3.65 | 43.74 | 1.47 | 87.50 | 11.48 |
| 43.82 | 6.27 | 43.83 | 9.89 | 87.67 | 9.89 | 43.83 | 4.98 | 45.33 | 11.12 | 43.83 | 3.94 | 87.67 | 3.86 | 43.83 | 1.46 | 87.67 | 11.51 |
| 43.90 | 6.30 | 43.92 | 10.02 | 87.83 | 9.88 | 43.91 | 5.16 | 45.41 | 11.02 | 43.91 | 3.96 | 87.83 | 3.89 | 43.91 | 1.45 | 87.83 | 11.47 |
| 43.99 | 6.39 | 44.00 | 9.88 | 88.00 | 9.92 | 44.00 | 4.97 | 45.50 | 11.00 | 44.00 | 3.93 | 88.00 | 3.76 | 43.99 | 1.45 | 88.00 | 11.52 |
| 44.07 | 6.40 | 44.08 | 9.83 | 88.17 | 9.95 | 44.08 | 5.09 | 45.58 | 11.15 | 44.08 | 4.14 | 88.17 | 3.71 | 44.08 | 1.44 | 88.17 | 11.56 |
| 44.15 | 6.46 | 44.17 | 9.86 | 88.33 | 9.96 | 44.16 | 5.28 | 45.66 | 10.98 | 44.16 | 4.04 | 88.33 | 3.75 | 44.16 | 1.45 | 88.33 | 11.52 |
| 44.24 | 6.32 | 44.25 | 9.94 | 88.50 | 10.04 | 44.25 | 5.14 | 45.75 | 11.06 | 44.25 | 4.04 | 88.50 | 3.75 | 44.24 | 1.47 | 88.50 | 11.58 |
| 44.32 | 6.33 | 44.33 | 9.91 | 88.67 | 10.06 | 44.33 | 5.37 | 45.83 | 11.01 | 44.33 | 4.12 | 88.67 | 3.81 | 44.33 | 1.46 | 88.67 | 11.64 |
| 44.40 | 6.49 | 44.42 | 9.89 | 88.83 | 10.06 | 44.41 | 5.10 | 45.91 | 11.05 | 44.41 | 4.18 | 88.83 | 3.77 | 44.41 | 1.46 | 88.83 | 11.61 |
| 44.49 | 6.34 | 44.50 | 9.86 | 89.00 | 10.06 | 44.50 | 5.40 | 46.00 | 11.01 | 44.50 | 4.12 | 89.00 | 3.75 | 44.49 | 1.47 | 89.00 | 11.58 |
| 44.57 | 6.49 | 44.58 | 10.05 | 89.17 | 10.12 | 44.58 | 5.05 | 46.08 | 11.03 | 44.58 | 4.21 | 89.17 | 3.72 | 44.58 | 1.46 | 89.17 | 11.66 |
| 44.65 | 6.32 | 44.67 | 9.88 | 89.33 | 10.19 | 44.66 | 5.19 | 46.16 | 11.06 | 44.66 | 4.08 | 89.33 | 3.75 | 44.66 | 1.47 | 89.33 | 11.71 |
| 44.74 | 6.32 | 44.75 | 9.93 | 89.50 | 10.27 | 44.75 | 5.26 | 46.25 | 11.08 | 44.75 | 4.18 | 89.50 | 3.78 | 44.74 | 1.48 | 89.50 | 11.65 |
| 44.82 | 6.50 | 44.83 | 9.97 | 89.67 | 10.22 | 44.83 | 5.28 | 46.33 | 11.13 | 44.83 | 4.11 | 89.67 | 3.81 | 44.83 | 1.53 | 89.67 | 11.78 |
| 44.90 | 6.37 | 44.92 | 10.03 | 89.83 | 10.21 | 44.91 | 5.09 | 46.41 | 11.18 | 44.91 | 4.16 | 89.83 | 3.81 | 44.91 | 1.57 | 89.83 | 11.73 |
| 44.99 | 6.38 | 45.00 | 10.05 | 90.00 | 10.22 | 45.00 | 5.13 | 46.50 | 11.19 | 45.00 | 4.25 | 90.00 | 3.85 | 44.99 | 1.56 | 90.00 | 11.73 |
| 45.07 | 6.36 | 45.08 | 10.02 | 90.17 | 10.19 | 45.08 | 5.16 | 46.58 | 11.18 | 45.08 | 4.03 | 90.17 | 3.88 | 45.08 | 1.56 | 90.17 | 11.83 |
| 45.15 | 6.35 | 45.17 | 10.07 | 90.33 | 10.26 | 45.16 | 5.17 | 46.66 | 11.25 | 45.16 | 4.04 | 90.33 | 3.82 | 45.16 | 1.56 | 90.33 | 11.73 |
| 45.24 | 6.52 | 45.25 | 10.11 | 90.50 | 10.27 | 45.25 | 5.20 | 46.75 | 11.32 | 45.25 | 4.20 | 90.50 | 3.86 | 45.24 | 1.60 | 90.50 | 11.78 |
| 45.32 | 6.32 | 45.33 | 10.18 | 90.67 | 10.25 | 45.33 | 5.26 | 46.83 | 11.20 | 45.33 | 4.18 | 90.67 | 3.88 | 45.33 | 1.60 | 90.67 | 11.73 |
| 45.40 | 6.34 | 45.42 | 10.13 | 90.83 | 10.37 | 45.41 | 5.26 | 46.91 | 11.21 | 45.41 | 4.30 | 90.83 | 3.88 | 45.41 | 1.60 | 90.83 | 11.79 |

| | | | | | | | | | | | | | | | | | |
|---|---|---|---|---|---|---|---|---|---|---|---|---|---|---|---|---|---|
| 45.49 | 6.39 | 45.50 | 10.16 | 91.00 | 10.38 | 45.50 | 5.13 | 47.00 | 11.26 | 45.50 | 4.30 | 91.00 | 3.88 | 45.49 | 1.61 | 91.00 | 11.79 |
| 45.57 | 6.39 | 45.58 | 10.16 | 91.17 | 10.39 | 45.58 | 5.12 | 47.08 | 11.20 | 45.58 | 4.27 | 91.17 | 3.89 | 45.58 | 1.60 | 91.17 | 11.79 |
| 45.65 | 6.38 | 45.67 | 10.25 | 91.33 | 10.35 | 45.66 | 5.16 | 47.16 | 11.21 | 45.66 | 4.27 | 91.33 | 3.88 | 45.66 | 1.60 | 91.33 | 11.85 |
| 45.74 | 6.49 | 45.75 | 10.28 | 91.50 | 10.38 | 45.75 | 5.20 | 47.25 | 11.31 | 45.75 | 4.28 | 91.50 | 3.88 | 45.74 | 1.60 | 91.50 | 11.83 |
| 45.82 | 6.39 | 45.83 | 10.34 | 91.67 | 10.38 | 45.83 | 5.21 | 47.33 | 11.17 | 45.83 | 4.36 | 91.67 | 3.88 | 45.83 | 1.59 | 91.67 | 11.90 |
| 45.90 | 6.44 | 45.92 | 10.35 | 91.83 | 10.41 | 45.91 | 5.35 | 47.41 | 11.26 | 45.91 | 4.33 | 91.83 | 3.89 | 45.91 | 1.59 | 91.83 | 11.89 |
| 45.99 | 6.52 | 46.00 | 10.32 | 92.00 | 10.45 | 46.00 | 5.23 | 47.50 | 11.17 | 46.00 | 4.46 | 92.00 | 3.87 | 45.99 | 1.60 | 92.00 | 11.87 |
| 46.07 | 6.60 | 46.08 | 10.34 | 92.17 | 10.52 | 46.08 | 5.21 | 47.58 | 11.19 | 46.08 | 4.42 | 92.17 | 3.88 | 46.08 | 1.60 | 92.17 | 11.93 |
| 46.15 | 6.47 | 46.17 | 10.33 | 92.33 | 10.52 | 46.16 | 5.36 | 47.66 | 11.32 | 46.16 | 4.49 | 92.33 | 3.90 | 46.16 | 1.60 | 92.33 | 11.97 |
| 46.24 | 6.64 | 46.25 | 10.40 | 92.50 | 10.54 | 46.25 | 5.22 | 47.75 | 11.21 | 46.25 | 4.51 | 92.50 | 3.91 | 46.24 | 1.59 | 92.50 | 11.92 |
| 46.32 | 6.43 | 46.33 | 10.48 | 92.67 | 10.65 | 46.33 | 5.21 | 47.83 | 11.23 | 46.33 | 4.38 | 92.67 | 3.91 | 46.33 | 1.58 | 92.67 | 11.94 |
| 46.40 | 6.44 | 46.42 | 10.47 | 92.83 | 10.66 | 46.41 | 5.23 | 47.91 | 11.24 | 46.41 | 4.41 | 92.83 | 3.94 | 46.41 | 1.59 | 92.83 | 11.89 |
| 46.49 | 6.67 | 46.50 | 10.42 | 93.00 | 10.61 | 46.50 | 5.25 | 48.00 | 11.21 | 46.50 | 4.39 | 93.00 | 3.91 | 46.49 | 1.60 | 93.00 | 11.91 |
| 46.57 | 6.48 | 46.58 | 10.41 | 93.17 | 10.67 | 46.58 | 5.29 | 48.08 | 11.24 | 46.58 | 4.44 | 93.17 | 3.97 | 46.58 | 1.60 | 93.17 | 11.97 |
| 46.65 | 6.48 | 46.67 | 10.38 | 93.33 | 10.62 | 46.66 | 5.19 | 48.16 | 11.25 | 46.66 | 4.38 | 93.33 | 4.02 | 46.66 | 1.61 | 93.33 | 11.93 |
| 46.74 | 6.48 | 46.75 | 10.57 | 93.50 | 10.60 | 46.75 | 5.21 | 48.25 | 11.30 | 46.75 | 4.54 | 93.50 | 4.03 | 46.74 | 1.60 | 93.50 | 11.91 |
| 46.82 | 6.49 | 46.83 | 10.44 | 93.67 | 10.60 | 46.83 | 5.36 | 48.33 | 11.34 | 46.83 | 4.39 | 93.67 | 4.02 | 46.83 | 1.59 | 93.67 | 12.04 |
| 46.90 | 6.67 | 46.92 | 10.49 | 93.83 | 10.68 | 46.91 | 5.32 | 48.41 | 11.38 | 46.91 | 4.41 | 93.83 | 4.07 | 46.91 | 1.68 | 93.83 | 12.05 |
| 46.99 | 6.43 | 47.00 | 10.46 | 94.00 | 10.63 | 47.00 | 5.44 | 48.50 | 11.43 | 47.00 | 4.43 | 94.00 | 4.09 | 46.99 | 1.68 | 94.00 | 12.02 |
| 47.07 | 6.46 | 47.08 | 10.58 | 94.17 | 10.60 | 47.08 | 5.53 | 48.58 | 11.44 | 47.08 | 4.57 | 94.17 | 4.07 | 47.08 | 1.72 | 94.17 | 12.05 |
| 47.15 | 6.46 | 47.17 | 10.54 | 94.33 | 10.60 | 47.16 | 5.49 | 48.66 | 11.37 | 47.16 | 4.42 | 94.33 | 4.13 | 47.16 | 1.73 | 94.33 | 12.08 |
| 47.24 | 6.49 | 47.25 | 10.54 | 94.50 | 10.73 | 47.25 | 5.56 | 48.75 | 11.37 | 47.25 | 4.44 | 94.50 | 4.16 | 47.24 | 1.73 | 94.50 | 12.05 |
| 47.32 | 6.65 | 47.33 | 10.55 | 94.67 | 10.61 | 47.33 | 5.38 | 48.83 | 11.36 | 47.33 | 4.43 | 94.67 | 4.16 | 47.33 | 1.72 | 94.67 | 12.14 |
| 47.40 | 6.51 | 47.42 | 10.57 | 94.83 | 10.66 | 47.41 | 5.49 | 48.91 | 11.51 | 47.41 | 4.43 | 94.83 | 4.16 | 47.41 | 1.72 | 94.83 | 12.21 |
| 47.49 | 6.66 | 47.50 | 10.60 | 95.00 | 10.69 | 47.50 | 5.61 | 49.00 | 11.37 | 47.50 | 4.44 | 95.00 | 4.17 | 47.49 | 1.72 | 95.00 | 12.16 |
| 47.57 | 6.43 | 47.58 | 10.66 | 95.17 | 10.69 | 47.58 | 5.60 | 49.08 | 11.37 | 47.58 | 4.44 | 95.17 | 4.21 | 47.58 | 1.73 | 95.17 | 12.24 |
| 47.65 | 6.44 | 47.67 | 10.75 | 95.33 | 10.76 | 47.66 | 5.60 | 49.16 | 11.50 | 47.66 | 4.44 | 95.33 | 4.22 | 47.66 | 1.73 | 95.33 | 12.21 |
| 47.74 | 6.46 | 47.75 | 10.73 | 95.50 | 10.80 | 47.75 | 5.57 | 49.25 | 11.35 | 47.75 | 4.49 | 95.50 | 4.22 | 47.74 | 1.73 | 95.50 | 12.18 |
| 47.82 | 6.49 | 47.83 | 10.59 | 95.67 | 10.82 | 47.83 | 5.76 | 49.33 | 11.34 | 47.83 | 4.53 | 95.67 | 4.29 | 47.83 | 1.72 | 95.67 | 12.11 |
| 47.90 | 6.53 | 47.92 | 10.74 | 95.83 | 10.83 | 47.91 | 5.64 | 49.41 | 11.45 | 47.91 | 4.55 | 95.83 | 4.31 | 47.91 | 3.81 | 95.83 | 12.17 |
| 47.99 | 6.65 | 48.00 | 10.58 | 96.00 | 10.90 | 48.00 | 5.71 | 49.50 | 11.41 | 48.00 | 4.59 | 96.00 | 4.31 | 47.99 | 3.73 | 96.00 | 12.18 |
| 48.07 | 6.54 | 48.08 | 10.75 | 96.17 | 10.89 | 48.08 | 5.85 | 49.58 | 11.45 | 48.08 | 4.63 | 96.17 | 4.34 | 48.08 | 3.32 | 96.17 | 12.27 |
| 48.15 | 6.54 | 48.17 | 10.56 | 96.33 | 10.91 | 48.16 | 5.82 | 49.66 | 11.60 | 48.16 | 4.60 | 96.33 | 4.39 | 48.16 | 3.68 | 96.33 | 12.11 |
| 48.24 | 6.52 | 48.25 | 10.65 | 96.50 | 10.97 | 48.25 | 5.85 | 49.75 | 11.53 | 48.25 | 4.61 | 96.50 | 4.39 | 48.24 | 3.62 | 96.50 | 12.21 |
| 48.32 | 6.58 | 48.33 | 10.63 | 96.67 | 11.00 | 48.33 | 5.68 | 49.83 | 11.57 | 48.33 | 4.68 | 96.67 | 4.43 | 48.33 | 3.66 | 96.67 | 12.19 |
| 48.40 | 6.65 | 48.42 | 10.73 | 96.83 | 11.02 | 48.41 | 5.75 | 49.91 | 11.60 | 48.41 | 4.65 | 96.83 | 4.48 | 48.41 | 3.74 | 96.83 | 12.09 |
| 48.49 | 6.69 | 48.50 | 10.54 | 97.00 | 11.07 | 48.50 | 5.71 | 50.00 | 11.61 | 48.50 | 4.71 | 97.00 | 4.49 | 48.49 | 3.69 | 97.00 | 12.20 |
| 48.57 | 6.73 | 48.58 | 10.76 | 97.17 | 11.12 | 48.58 | 5.73 | 50.08 | 11.59 | 48.58 | 4.70 | 97.17 | 4.51 | 48.58 | 3.75 | 97.17 | 12.14 |
| 48.65 | 6.65 | 48.67 | 10.56 | 97.33 | 11.07 | 48.66 | 5.79 | 50.16 | 11.76 | 48.66 | 4.63 | 97.33 | 4.56 | 48.66 | 3.85 | 97.33 | 12.12 |
| 48.74 | 6.65 | 48.75 | 10.64 | 97.50 | 11.14 | 48.75 | 5.81 | 50.25 | 11.77 | 48.75 | 4.75 | 97.50 | 4.57 | 48.74 | 3.90 | 97.50 | 12.18 |
| 48.82 | 6.71 | 48.83 | 10.65 | 97.67 | 11.06 | 48.83 | 5.83 | 50.33 | 11.73 | 48.83 | 4.83 | 97.67 | 4.55 | 48.83 | 3.98 | 97.67 | 12.23 |
| 48.90 | 6.76 | 48.92 | 10.65 | 97.83 | 11.07 | 48.91 | 5.87 | 50.41 | 11.73 | 48.91 | 4.86 | 97.83 | 4.59 | 48.91 | 4.01 | 97.83 | 12.23 |
| 48.99 | 6.82 | 49.00 | 10.64 | 98.00 | 11.12 | 49.00 | 5.90 | 50.50 | 11.79 | 49.00 | 4.74 | 98.00 | 4.61 | 48.99 | 4.02 | 98.00 | 12.18 |
| 49.07 | 6.85 | 49.08 | 10.63 | 98.17 | 11.10 | 49.08 | 5.99 | 50.58 | 11.73 | 49.08 | 4.75 | 98.17 | 4.60 | 49.08 | 4.06 | 98.17 | 12.25 |
| 49.15 | 6.88 | 49.17 | 10.64 | 98.33 | 11.16 | 49.16 | 6.08 | 50.66 | 11.74 | 49.16 | 4.73 | 98.33 | 4.65 | 49.16 | 3.98 | 98.33 | 12.32 |
| 49.24 | 6.78 | 49.25 | 10.72 | 98.50 | 11.14 | 49.25 | 5.92 | 50.75 | 11.76 | 49.25 | 4.78 | 98.50 | 4.71 | 49.24 | 4.01 | 98.50 | 12.28 |
| 49.32 | 6.80 | 49.33 | 10.79 | 98.67 | 11.22 | 49.33 | 5.93 | 50.83 | 11.75 | 49.33 | 4.88 | 98.67 | 4.71 | 49.33 | 4.05 | 98.67 | 12.34 |
| 49.40 | 6.84 | 49.42 | 10.82 | 98.83 | 11.24 | 49.41 | 6.09 | 50.91 | 11.80 | 49.41 | 4.98 | 98.83 | 4.73 | 49.41 | 4.10 | 98.83 | 12.33 |
| 49.49 | 6.93 | 49.50 | 10.87 | 99.00 | 11.24 | 49.50 | 5.90 | 51.00 | 11.80 | 49.50 | 5.00 | 99.00 | 4.76 | 49.49 | 4.12 | 99.00 | 12.36 |
| 49.57 | 6.99 | 49.58 | 10.77 | 99.17 | 11.27 | 49.58 | 6.10 | 51.08 | 11.80 | 49.58 | 4.88 | 99.17 | 4.80 | 49.58 | 4.18 | 99.17 | 12.37 |
| 49.65 | 7.03 | 49.67 | 10.88 | 99.33 | 11.22 | 49.66 | 5.87 | 51.16 | 11.84 | 49.66 | 5.03 | 99.33 | 4.81 | 49.66 | 4.09 | 99.33 | 12.38 |

| | | | | | | | | | | | | | | | | | |
|---|---|---|---|---|---|---|---|---|---|---|---|---|---|---|---|---|---|
| 49.74 | 6.87 | 49.75 | 10.75 | 99.50 | 11.28 | 49.75 | 5.97 | 51.25 | 11.87 | 49.75 | 4.83 | 99.50 | 4.85 | 49.74 | 4.46 | 99.50 | 12.38 |
| 49.82 | 6.88 | 49.83 | 10.76 | 99.67 | 11.32 | 49.83 | 5.96 | 51.33 | 11.91 | 49.83 | 4.86 | 99.67 | 4.86 | 49.83 | 4.21 | 99.67 | 12.44 |
| 49.90 | 7.09 | 49.92 | 10.88 | 99.83 | 11.28 | 49.91 | 5.94 | 51.41 | 11.96 | 49.91 | 4.87 | 99.83 | 4.86 | 49.91 | 4.23 | 99.83 | 12.48 |
| 49.99 | 6.89 | 50.00 | 10.85 | 100.00 | 11.31 | 50.00 | 6.08 | 51.50 | 11.90 | 50.00 | 4.91 | 100.00 | 4.89 | 49.99 | 4.16 | 100.00 | 12.44 |
| 50.07 | 6.87 | 50.08 | 10.81 | 100.17 | 11.41 | 50.08 | 5.93 | 51.58 | 11.95 | 50.08 | 4.91 | 100.17 | 4.98 | 50.08 | 4.15 | 100.17 | 12.49 |
| 50.15 | 6.91 | 50.17 | 10.85 | 100.33 | 11.38 | 50.16 | 5.93 | 51.66 | 11.86 | 50.16 | 4.90 | 100.33 | 4.98 | 50.16 | 4.13 | 100.33 | 12.57 |
| 50.24 | 7.09 | 50.25 | 10.77 | 100.50 | 11.45 | 50.25 | 6.12 | 51.75 | 11.95 | 50.25 | 5.05 | 100.50 | 4.98 | 50.24 | 4.15 | 100.50 | 12.57 |
| 50.32 | 6.88 | 50.33 | 10.76 | 100.67 | 11.42 | 50.33 | 6.06 | 51.83 | 11.85 | 50.33 | 4.89 | 100.67 | 4.99 | 50.33 | 4.18 | 100.67 | 12.59 |
| 50.40 | 6.98 | 50.42 | 10.81 | 100.83 | 11.41 | 50.41 | 6.17 | 51.91 | 11.86 | 50.41 | 4.92 | 100.83 | 5.03 | 50.41 | 4.26 | 100.83 | 12.64 |
| 50.49 | 7.11 | 50.50 | 10.88 | 101.00 | 11.57 | 50.50 | 6.18 | 52.00 | 11.95 | 50.50 | 4.91 | 101.00 | 5.03 | 50.49 | 4.33 | 101.00 | 12.60 |
| 50.57 | 7.14 | 50.58 | 10.81 | 101.17 | 11.51 | 50.58 | 6.21 | 52.08 | 11.87 | 50.58 | 4.93 | 101.17 | 5.08 | 50.58 | 4.39 | 101.17 | 12.56 |
| 50.65 | 7.15 | 50.67 | 10.75 | 101.33 | 11.58 | 50.66 | 6.06 | 52.16 | 11.99 | 50.66 | 4.94 | 101.33 | 5.10 | 50.66 | 4.35 | 101.33 | 12.67 |
| 50.74 | 7.25 | 50.75 | 10.92 | 101.50 | 11.53 | 50.75 | 6.09 | 52.25 | 11.87 | 50.75 | 4.94 | 101.50 | 5.13 | 50.74 | 4.43 | 101.50 | 12.60 |
| 50.82 | 7.29 | 50.83 | 10.82 | 101.67 | 11.52 | 50.83 | 6.27 | 52.33 | 11.85 | 50.83 | 4.96 | 101.67 | 5.15 | 50.83 | 4.45 | 101.67 | 12.60 |
| 50.90 | 7.32 | 50.92 | 10.86 | 101.83 | 11.62 | 50.91 | 6.10 | 52.41 | 11.89 | 50.91 | 4.96 | 101.83 | 5.21 | 50.91 | 4.37 | 101.83 | 12.59 |
| 50.99 | 7.31 | 51.00 | 10.89 | 102.00 | 11.47 | 51.00 | 6.19 | 52.50 | 11.89 | 51.00 | 5.08 | 102.00 | 5.21 | 50.99 | 4.54 | 102.00 | 12.60 |
| 51.07 | 7.44 | 51.08 | 10.98 | 102.17 | 11.54 | 51.08 | 6.32 | 52.58 | 11.98 | 51.08 | 5.11 | 102.17 | 5.26 | 51.08 | 4.40 | 102.17 | 12.67 |
| 51.15 | 7.33 | 51.17 | 11.03 | 102.33 | 11.51 | 51.16 | 6.41 | 52.66 | 11.89 | 51.16 | 5.13 | 102.33 | 5.32 | 51.16 | 4.38 | 102.33 | 12.56 |
| 51.24 | 7.35 | 51.25 | 11.05 | 102.50 | 11.51 | 51.25 | 6.41 | 52.75 | 11.86 | 51.25 | 5.17 | 102.50 | 5.35 | 51.24 | 4.38 | 102.50 | 12.59 |
| 51.32 | 7.52 | 51.33 | 10.98 | 102.67 | 11.51 | 51.33 | 6.18 | 52.83 | 11.90 | 51.33 | 5.09 | 102.67 | 5.34 | 51.33 | 4.55 | 102.67 | 12.61 |
| 51.40 | 7.32 | 51.42 | 11.03 | 102.83 | 11.52 | 51.41 | 6.35 | 52.91 | 11.90 | 51.41 | 5.07 | 102.83 | 5.39 | 51.41 | 4.40 | 102.83 | 12.59 |
| 51.49 | 7.32 | 51.50 | 10.97 | 103.00 | 11.62 | 51.50 | 6.48 | 53.00 | 11.96 | 51.50 | 5.10 | 103.00 | 5.40 | 51.49 | 4.39 | 103.00 | 12.58 |
| 51.57 | 7.55 | 51.58 | 10.93 | 103.17 | 11.47 | 51.58 | 6.35 | 53.08 | 11.98 | 51.58 | 5.17 | 103.17 | 5.41 | 51.58 | 4.45 | 103.17 | 12.62 |
| 51.65 | 7.35 | 51.67 | 11.03 | 103.33 | 11.55 | 51.66 | 6.57 | 53.16 | 12.04 | 51.66 | 5.15 | 103.33 | 5.42 | 51.66 | 4.45 | 103.33 | 12.63 |
| 51.74 | 7.39 | 51.75 | 10.92 | 103.50 | 11.57 | 51.75 | 6.66 | 53.25 | 12.05 | 51.75 | 5.19 | 103.50 | 5.48 | 51.74 | 4.57 | 103.50 | 12.59 |
| 51.82 | 7.37 | 51.83 | 11.01 | 103.67 | 11.59 | 51.83 | 6.65 | 53.33 | 12.05 | 51.83 | 5.27 | 103.67 | 5.47 | 51.83 | 4.60 | 103.67 | 12.62 |
| 51.90 | 7.50 | 51.92 | 10.87 | 103.83 | 11.69 | 51.91 | 6.40 | 53.41 | 12.10 | 51.91 | 5.30 | 103.83 | 5.48 | 51.91 | 4.64 | 103.83 | 12.68 |
| 51.99 | 7.34 | 52.00 | 10.88 | 104.00 | 11.71 | 52.00 | 6.49 | 53.50 | 12.02 | 52.00 | 5.24 | 104.00 | 5.54 | 51.99 | 4.66 | 104.00 | 12.64 |
| 52.07 | 7.34 | 52.08 | 10.89 | 104.17 | 11.75 | 52.08 | 6.48 | 53.58 | 12.08 | 52.08 | 5.22 | 104.17 | 5.54 | 52.08 | 4.66 | 104.17 | 12.66 |
| 52.15 | 7.37 | 52.17 | 11.10 | 104.33 | 11.78 | 52.16 | 6.61 | 53.66 | 12.09 | 52.16 | 5.26 | 104.33 | 5.60 | 52.16 | 4.75 | 104.33 | 12.73 |
| 52.24 | 7.38 | 52.25 | 11.00 | 104.50 | 11.79 | 52.25 | 6.46 | 53.75 | 12.11 | 52.25 | 5.27 | 104.50 | 5.59 | 52.24 | 4.73 | 104.50 | 12.71 |
| 52.32 | 7.39 | 52.33 | 11.01 | 104.67 | 11.89 | 52.33 | 6.67 | 53.83 | 12.10 | 52.33 | 5.27 | 104.67 | 5.72 | 52.33 | 4.81 | 104.67 | 12.70 |
| 52.40 | 7.42 | 52.42 | 10.95 | 104.83 | 11.82 | 52.41 | 6.45 | 53.91 | 11.99 | 52.41 | 5.39 | 104.83 | 5.73 | 52.41 | 4.81 | 104.83 | 12.74 |
| 52.49 | 7.44 | 52.50 | 10.88 | 105.00 | 11.85 | 52.50 | 6.44 | 54.00 | 12.03 | 52.50 | 5.43 | 105.00 | 5.70 | 52.49 | 4.75 | 105.00 | 12.79 |
| 52.57 | 7.47 | 52.58 | 10.91 | 105.17 | 11.85 | 52.58 | 6.62 | 54.08 | 12.02 | 52.58 | 5.48 | 105.17 | 5.80 | 52.58 | 4.96 | 105.17 | 12.79 |
| 52.65 | 7.57 | 52.67 | 10.90 | 105.33 | 11.87 | 52.66 | 6.47 | 54.16 | 12.08 | 52.66 | 5.52 | 105.33 | 5.81 | 52.66 | 4.80 | 105.33 | 12.82 |
| 52.74 | 7.55 | 52.75 | 11.05 | 105.50 | 11.96 | 52.75 | 6.49 | 54.25 | 12.15 | 52.75 | 5.37 | 105.50 | 5.81 | 52.74 | 4.82 | 105.50 | 12.86 |
| 52.82 | 7.57 | 52.83 | 10.92 | 105.67 | 12.02 | 52.83 | 6.67 | 54.33 | 12.14 | 52.83 | 5.37 | 105.67 | 5.82 | 52.83 | 4.83 | 105.67 | 12.89 |
| 52.90 | 7.63 | 52.92 | 10.91 | 105.83 | 12.04 | 52.91 | 6.43 | 54.41 | 12.19 | 52.91 | 5.44 | 105.83 | 5.85 | 52.91 | 4.96 | 105.83 | 12.90 |
| 52.99 | 7.67 | 53.00 | 11.13 | 106.00 | 11.90 | 53.00 | 6.64 | 54.50 | 12.22 | 53.00 | 5.41 | 106.00 | 5.85 | 52.99 | 4.77 | 106.00 | 12.94 |
| 53.07 | 7.68 | 53.08 | 11.01 | 106.17 | 11.98 | 53.08 | 6.43 | 54.58 | 12.23 | 53.08 | 5.39 | 106.17 | 5.89 | 53.08 | 4.78 | 106.17 | 12.98 |
| 53.15 | 7.73 | 53.17 | 10.98 | 106.33 | 11.96 | 53.16 | 6.46 | 54.66 | 12.25 | 53.16 | 5.52 | 106.33 | 5.93 | 53.16 | 4.81 | 106.33 | 12.99 |
| 53.24 | 7.77 | 53.25 | 10.99 | 106.50 | 12.07 | 53.25 | 6.48 | 54.75 | 12.24 | 53.25 | 5.39 | 106.50 | 5.93 | 53.24 | 4.87 | 106.50 | 13.00 |
| 53.32 | 7.67 | 53.33 | 10.97 | 106.67 | 11.92 | 53.33 | 6.63 | 54.83 | 12.23 | 53.33 | 5.47 | 106.67 | 5.94 | 53.33 | 4.86 | 106.67 | 13.01 |
| 53.40 | 7.73 | 53.42 | 10.89 | 106.83 | 11.93 | 53.41 | 6.44 | 54.91 | 12.23 | 53.41 | 5.54 | 106.83 | 6.00 | 53.41 | 4.86 | 106.83 | 13.08 |
| 53.49 | 7.85 | 53.50 | 10.90 | 107.00 | 12.07 | 53.50 | 6.66 | 55.00 | 12.30 | 53.50 | 5.60 | 107.00 | 6.01 | 53.49 | 4.84 | 107.00 | 12.99 |
| 53.57 | 7.91 | 53.58 | 10.99 | 107.17 | 11.96 | 53.58 | 6.41 | 55.08 | 12.26 | 53.58 | 5.64 | 107.17 | 5.97 | 53.58 | 4.98 | 107.17 | 13.02 |
| 53.65 | 7.73 | 53.67 | 10.89 | 107.33 | 11.97 | 53.66 | 6.45 | 55.16 | 12.21 | 53.66 | 5.53 | 107.33 | 6.05 | 53.66 | 4.89 | 107.33 | 12.95 |
| 53.74 | 7.94 | 53.75 | 10.93 | 107.50 | 11.96 | 53.75 | 6.50 | 55.25 | 12.21 | 53.75 | 5.57 | 107.50 | 6.08 | 53.74 | 5.00 | 107.50 | 12.96 |
| 53.82 | 7.72 | 53.83 | 10.94 | 107.67 | 11.96 | 53.83 | 6.51 | 55.33 | 12.22 | 53.83 | 5.59 | 107.67 | 6.08 | 53.83 | 5.03 | 107.67 | 13.01 |
| 53.90 | 7.73 | 53.92 | 11.06 | 107.83 | 12.03 | 53.91 | 6.64 | 55.41 | 12.27 | 53.91 | 5.64 | 107.83 | 6.09 | 53.91 | 5.06 | 107.83 | 12.99 |

| | | | | | | | | | | | | | | | | | |
|---|---|---|---|---|---|---|---|---|---|---|---|---|---|---|---|---|---|
| 53.99 | 7.97 | 54.00 | 10.92 | 108.00 | 12.00 | 54.00 | 6.43 | 55.50 | 12.24 | 54.00 | 5.68 | 108.00 | 6.18 | 53.99 | 5.10 | 108.00 | 12.99 |
| 54.07 | 7.82 | 54.08 | 11.03 | 108.17 | 12.02 | 54.08 | 6.46 | 55.58 | 12.24 | 54.08 | 5.68 | 108.17 | 6.18 | 54.08 | 5.11 | 108.17 | 13.07 |
| 54.15 | 7.80 | 54.17 | 10.94 | 108.33 | 12.19 | 54.16 | 6.46 | 55.66 | 12.26 | 54.16 | 5.69 | 108.33 | 6.20 | 54.16 | 5.18 | 108.33 | 13.00 |
| 54.24 | 7.77 | 54.25 | 10.97 | 108.50 | 12.22 | 54.25 | 6.49 | 55.75 | 12.29 | 54.25 | 5.78 | 108.50 | 6.22 | 54.24 | 5.11 | 108.50 | 12.99 |
| 54.32 | 7.98 | 54.33 | 10.95 | 108.67 | 12.11 | 54.33 | 6.57 | 55.83 | 12.38 | 54.33 | 5.76 | 108.67 | 6.22 | 54.33 | 5.16 | 108.67 | 13.04 |
| 54.40 | 7.72 | 54.42 | 11.01 | 108.83 | 12.07 | 54.41 | 6.66 | 55.91 | 12.37 | 54.41 | 5.75 | 108.83 | 6.23 | 54.41 | 5.29 | 108.83 | 13.03 |
| 54.49 | 7.77 | 54.50 | 10.94 | 109.00 | 12.14 | 54.50 | 6.64 | 56.00 | 12.46 | 54.50 | 5.75 | 109.00 | 6.24 | 54.49 | 5.30 | 109.00 | 13.05 |
| 54.57 | 7.79 | 54.58 | 10.94 | 109.17 | 12.20 | 54.58 | 6.74 | 56.08 | 12.48 | 54.58 | 5.72 | 109.17 | 6.26 | 54.58 | 5.36 | 109.17 | 13.01 |
| 54.65 | 7.80 | 54.67 | 10.93 | 109.33 | 12.28 | 54.66 | 6.77 | 56.16 | 12.35 | 54.66 | 5.84 | 109.33 | 6.26 | 54.66 | 5.18 | 109.33 | 13.08 |
| 54.74 | 7.94 | 54.75 | 11.12 | 109.50 | 12.47 | 54.75 | 6.78 | 56.25 | 12.47 | 54.75 | 5.73 | 109.50 | 6.30 | 54.74 | 5.21 | 109.50 | 13.10 |
| 54.82 | 7.76 | 54.83 | 11.00 | 109.67 | 12.55 | 54.83 | 6.80 | 56.33 | 12.36 | 54.83 | 5.82 | 109.67 | 6.37 | 54.83 | 5.25 | 109.67 | 13.14 |
| 54.90 | 7.96 | 54.92 | 11.07 | 109.83 | 12.55 | 54.91 | 6.98 | 56.41 | 12.39 | 54.91 | 5.75 | 109.83 | 6.43 | 54.91 | 5.26 | 109.83 | 13.15 |
| 54.99 | 7.74 | 55.00 | 11.06 | 110.00 | 12.63 | 55.00 | 6.79 | 56.50 | 12.43 | 55.00 | 5.77 | 110.00 | 6.39 | 54.99 | 5.25 | 110.00 | 13.14 |
| 55.07 | 7.81 | 55.08 | 11.06 | 110.17 | 12.60 | 55.08 | 6.79 | 56.58 | 12.43 | 55.08 | 5.87 | 110.17 | 6.48 | 55.08 | 5.26 | 110.17 | 13.19 |
| 55.15 | 7.86 | 55.17 | 11.08 | 110.33 | 12.63 | 55.16 | 7.09 | 56.66 | 12.53 | 55.16 | 5.90 | 110.33 | 6.54 | 55.16 | 5.29 | 110.33 | 13.13 |
| 55.24 | 7.93 | 55.25 | 10.95 | 110.50 | 12.57 | 55.25 | 6.84 | 56.75 | 12.53 | 55.25 | 5.82 | 110.50 | 6.53 | 55.24 | 5.26 | 110.50 | 13.20 |
| 55.32 | 7.97 | 55.33 | 11.01 | 110.67 | 12.66 | 55.33 | 6.80 | 56.83 | 12.56 | 55.33 | 5.86 | 110.67 | 6.55 | 55.33 | 5.40 | 110.67 | 13.17 |
| 55.40 | 8.01 | 55.42 | 11.07 | 110.83 | 12.69 | 55.41 | 6.83 | 56.91 | 12.60 | 55.41 | 5.95 | 110.83 | 6.62 | 55.41 | 5.34 | 110.83 | 13.14 |
| 55.49 | 8.07 | 55.50 | 11.08 | 111.00 | 12.66 | 55.50 | 6.87 | 57.00 | 12.66 | 55.50 | 5.87 | 111.00 | 6.63 | 55.49 | 5.42 | 111.00 | 13.21 |
| 55.57 | 7.94 | 55.58 | 11.17 | 111.17 | 12.69 | 55.58 | 6.89 | 57.08 | 12.56 | 55.58 | 6.02 | 111.17 | 6.63 | 55.58 | 5.45 | 111.17 | 13.27 |
| 55.65 | 7.95 | 55.67 | 11.18 | 111.33 | 12.74 | 55.66 | 6.80 | 57.16 | 12.54 | 55.66 | 5.85 | 111.33 | 6.63 | 55.66 | 5.51 | 111.33 | 13.25 |
| 55.74 | 7.99 | 55.75 | 11.20 | 111.50 | 12.78 | 55.75 | 6.78 | 57.25 | 12.67 | 55.75 | 5.87 | 111.50 | 6.64 | 55.74 | 5.53 | 111.50 | 13.30 |
| 55.82 | 8.08 | 55.83 | 11.13 | 111.67 | 12.83 | 55.83 | 7.04 | 57.33 | 12.49 | 55.83 | 5.99 | 111.67 | 6.64 | 55.83 | 5.59 | 111.67 | 13.32 |
| 55.90 | 7.95 | 55.92 | 11.12 | 111.83 | 12.87 | 55.91 | 6.78 | 57.41 | 12.52 | 55.91 | 5.83 | 111.83 | 6.67 | 55.91 | 5.54 | 111.83 | 13.27 |
| 55.99 | 8.08 | 56.00 | 11.26 | 112.00 | 12.87 | 56.00 | 6.85 | 57.50 | 12.61 | 56.00 | 5.89 | 112.00 | 6.74 | 55.99 | 5.63 | 112.00 | 13.28 |
| 56.07 | 7.95 | 56.08 | 11.11 | 112.17 | 12.90 | 56.08 | 6.88 | 57.58 | 12.60 | 56.08 | 5.87 | 112.17 | 6.73 | 56.08 | 5.67 | 112.17 | 13.29 |
| 56.15 | 7.95 | 56.17 | 11.11 | 112.33 | 12.85 | 56.16 | 6.92 | 57.66 | 12.64 | 56.16 | 5.90 | 112.33 | 6.71 | 56.16 | 5.74 | 112.33 | 13.35 |
| 56.24 | 8.01 | 56.25 | 11.15 | 112.50 | 12.93 | 56.25 | 6.84 | 57.75 | 12.64 | 56.25 | 5.88 | 112.50 | 6.78 | 56.24 | 5.65 | 112.50 | 13.29 |
| 56.32 | 8.09 | 56.33 | 11.18 | 112.67 | 12.82 | 56.33 | 6.77 | 57.83 | 12.67 | 56.33 | 5.90 | 112.67 | 6.78 | 56.33 | 5.79 | 112.67 | 13.43 |
| 56.40 | 8.14 | 56.42 | 11.17 | 112.83 | 12.96 | 56.41 | 6.78 | 57.91 | 12.69 | 56.41 | 5.87 | 112.83 | 6.79 | 56.41 | 5.62 | 112.83 | 13.41 |
| 56.49 | 8.18 | 56.50 | 11.15 | 113.00 | 12.80 | 56.50 | 6.82 | 58.00 | 12.74 | 56.50 | 5.87 | 113.00 | 6.89 | 56.49 | 5.63 | 113.00 | 13.45 |
| 56.57 | 8.11 | 56.58 | 11.16 | 113.17 | 12.85 | 56.58 | 6.88 | 58.08 | 12.79 | 56.58 | 5.93 | 113.17 | 6.95 | 56.58 | 5.65 | 113.17 | 13.37 |
| 56.65 | 8.09 | 56.67 | 11.20 | 113.33 | 12.82 | 56.66 | 6.89 | 58.16 | 12.79 | 56.66 | 6.01 | 113.33 | 6.93 | 56.66 | 5.69 | 113.33 | 13.42 |
| 56.74 | 8.11 | 56.75 | 11.25 | 113.50 | 12.84 | 56.75 | 6.95 | 58.25 | 12.80 | 56.75 | 5.93 | 113.50 | 6.95 | 56.74 | 5.71 | 113.50 | 13.48 |
| 56.82 | 8.11 | 56.83 | 11.25 | 113.67 | 12.96 | 56.83 | 6.93 | 58.33 | 12.80 | 56.83 | 5.98 | 113.67 | 7.03 | 56.83 | 5.80 | 113.67 | 13.36 |
| 56.90 | 8.12 | 56.92 | 11.28 | 113.83 | 12.86 | 56.91 | 6.99 | 58.41 | 12.77 | 56.91 | 6.11 | 113.83 | 7.01 | 56.91 | 5.65 | 113.83 | 13.41 |
| 56.99 | 8.13 | 57.00 | 11.29 | 114.00 | 12.86 | 57.00 | 7.05 | 58.50 | 12.75 | 57.00 | 6.06 | 114.00 | 7.04 | 56.99 | 5.65 | 114.00 | 13.46 |
| 57.07 | 8.13 | 57.08 | 11.23 | 114.17 | 12.85 | 57.08 | 7.16 | 58.58 | 12.74 | 57.08 | 6.08 | 114.17 | 7.05 | 57.08 | 5.72 | 114.17 | 13.36 |
| 57.15 | 8.15 | 57.17 | 11.28 | 114.33 | 12.87 | 57.16 | 7.02 | 58.66 | 12.76 | 57.16 | 6.14 | 114.33 | 7.08 | 57.16 | 5.82 | 114.33 | 13.39 |
| 57.24 | 8.24 | 57.25 | 11.38 | 114.50 | 12.86 | 57.25 | 7.20 | 58.75 | 12.81 | 57.25 | 6.12 | 114.50 | 7.06 | 57.24 | 5.87 | 114.50 | 13.40 |
| 57.32 | 8.07 | 57.33 | 11.41 | 114.67 | 12.90 | 57.33 | 7.00 | 58.83 | 12.83 | 57.33 | 6.20 | 114.67 | 7.10 | 57.33 | 5.89 | 114.67 | 13.49 |
| 57.40 | 8.15 | 57.42 | 11.29 | 114.83 | 12.93 | 57.41 | 7.19 | 58.91 | 12.85 | 57.41 | 6.22 | 114.83 | 7.14 | 57.41 | 5.90 | 114.83 | 13.43 |
| 57.49 | 8.21 | 57.50 | 11.42 | 115.00 | 12.99 | 57.50 | 7.01 | 59.00 | 12.86 | 57.50 | 6.28 | 115.00 | 7.15 | 57.49 | 5.88 | 115.00 | 13.44 |
| 57.57 | 8.32 | 57.58 | 11.36 | 115.17 | 13.03 | 57.58 | 6.99 | 59.08 | 12.84 | 57.58 | 6.29 | 115.17 | 7.17 | 57.58 | 5.91 | 115.17 | 13.42 |
| 57.65 | 8.37 | 57.67 | 11.43 | 115.33 | 13.00 | 57.66 | 7.01 | 59.16 | 12.87 | 57.66 | 6.23 | 115.33 | 7.16 | 57.66 | 6.03 | 115.33 | 13.43 |
| 57.74 | 8.17 | 57.75 | 11.51 | 115.50 | 13.08 | 57.75 | 7.03 | 59.25 | 12.90 | 57.75 | 6.29 | 115.50 | 7.18 | 57.74 | 6.13 | 115.50 | 13.50 |
| 57.82 | 8.18 | 57.83 | 11.55 | 115.67 | 13.03 | 57.83 | 7.04 | 59.33 | 12.93 | 57.83 | 6.23 | 115.67 | 7.19 | 57.83 | 6.16 | 115.67 | 13.49 |
| 57.90 | 8.20 | 57.92 | 11.56 | 115.83 | 13.12 | 57.91 | 7.23 | 59.41 | 12.97 | 57.91 | 6.24 | 115.83 | 7.21 | 57.91 | 6.09 | 115.83 | 13.52 |
| 57.99 | 8.40 | 58.00 | 11.48 | 116.00 | 13.09 | 58.00 | 7.00 | 59.50 | 12.89 | 58.00 | 6.24 | 116.00 | 7.25 | 57.99 | 6.20 | 116.00 | 13.57 |
| 58.07 | 8.18 | 58.08 | 11.54 | 116.17 | 13.15 | 58.08 | 7.01 | 59.58 | 12.99 | 58.08 | 6.25 | 116.17 | 7.23 | 58.08 | 6.05 | 116.17 | 13.58 |
| 58.15 | 8.19 | 58.17 | 11.49 | 116.33 | 13.20 | 58.16 | 7.27 | 59.66 | 12.89 | 58.16 | 6.24 | 116.33 | 7.27 | 58.16 | 6.06 | 116.33 | 13.55 |

| | | | | | | | | | | | | | | | | | |
|---|---|---|---|---|---|---|---|---|---|---|---|---|---|---|---|---|---|
| 58.24 | 8.43 | 58.25 | 11.49 | 116.50 | 13.22 | 58.25 | 7.17 | 59.75 | 12.88 | 58.25 | 6.26 | 116.50 | 7.31 | 58.24 | 6.08 | 116.50 | 13.62 |
| 58.32 | 8.25 | 58.33 | 11.47 | 116.67 | 13.16 | 58.33 | 7.26 | 59.83 | 12.99 | 58.33 | 6.31 | 116.67 | 7.29 | 58.33 | 6.10 | 116.67 | 13.57 |
| 58.40 | 8.22 | 58.42 | 11.53 | 116.83 | 13.18 | 58.41 | 7.20 | 59.91 | 12.89 | 58.41 | 6.41 | 116.83 | 7.31 | 58.41 | 6.22 | 116.83 | 13.62 |
| 58.49 | 8.24 | 58.50 | 11.48 | 117.00 | 13.17 | 58.50 | 7.34 | 60.00 | 12.90 | 58.50 | 6.47 | 117.00 | 7.39 | 58.49 | 6.08 | 117.00 | 13.57 |
| 58.57 | 8.26 | 58.58 | 11.46 | 117.17 | 13.17 | 58.58 | 7.16 | 60.08 | 12.98 | 58.58 | 6.33 | 117.17 | 7.39 | 58.58 | 6.11 | 117.17 | 13.66 |
| 58.65 | 8.39 | 58.67 | 11.44 | 117.33 | 13.20 | 58.66 | 7.16 | 60.16 | 12.87 | 58.66 | 6.34 | 117.33 | 7.38 | 58.66 | 6.10 | 117.33 | 13.71 |
| 58.74 | 8.22 | 58.75 | 11.64 | 117.50 | 13.18 | 58.75 | 7.17 | 60.25 | 13.01 | 58.75 | 6.49 | 117.50 | 7.40 | 58.74 | 6.11 | 117.50 | 13.73 |
| 58.82 | 8.21 | 58.83 | 11.47 | 117.67 | 13.18 | 58.83 | 7.22 | 60.33 | 12.87 | 58.83 | 6.34 | 117.67 | 7.45 | 58.83 | 6.12 | 117.67 | 13.69 |
| 58.90 | 8.42 | 58.92 | 11.53 | 117.83 | 13.17 | 58.91 | 7.23 | 60.41 | 12.89 | 58.91 | 6.35 | 117.83 | 7.43 | 58.91 | 6.16 | 117.83 | 13.78 |
| 58.99 | 8.24 | 59.00 | 11.52 | 118.00 | 13.18 | 59.00 | 7.30 | 60.50 | 12.89 | 59.00 | 6.36 | 118.00 | 7.45 | 58.99 | 6.21 | 118.00 | 13.71 |
| 59.07 | 8.26 | 59.08 | 11.54 | 118.17 | 13.18 | 59.08 | 7.31 | 60.58 | 12.93 | 59.08 | 6.37 | 118.17 | 7.45 | 59.08 | 6.27 | 118.17 | 13.68 |
| 59.15 | 8.25 | 59.17 | 11.43 | 118.33 | 13.27 | 59.16 | 7.15 | 60.66 | 12.93 | 59.16 | 6.40 | 118.33 | 7.44 | 59.16 | 6.28 | 118.33 | 13.77 |
| 59.24 | 8.40 | 59.25 | 11.47 | 118.50 | 13.27 | 59.25 | 7.14 | 60.75 | 12.89 | 59.25 | 6.51 | 118.50 | 7.45 | 59.24 | 6.30 | 118.50 | 13.84 |
| 59.32 | 8.19 | 59.33 | 11.51 | 118.67 | 13.38 | 59.33 | 7.16 | 60.83 | 12.90 | 59.33 | 6.34 | 118.67 | 7.45 | 59.33 | 6.28 | 118.67 | 13.84 |
| 59.40 | 8.43 | 59.42 | 11.49 | 118.83 | 13.40 | 59.41 | 7.34 | 60.91 | 12.94 | 59.41 | 6.37 | 118.83 | 7.46 | 59.41 | 6.32 | 118.83 | 13.79 |
| 59.49 | 8.16 | 59.50 | 11.49 | 119.00 | 13.29 | 59.50 | 7.40 | 61.00 | 12.99 | 59.50 | 6.39 | 119.00 | 7.47 | 59.49 | 6.38 | 119.00 | 13.90 |
| 59.57 | 8.18 | 59.58 | 11.50 | 119.17 | 13.32 | 59.58 | 7.46 | 61.08 | 12.99 | 59.58 | 6.44 | 119.17 | 7.53 | 59.58 | 6.43 | 119.17 | 13.83 |
| 59.65 | 8.25 | 59.67 | 11.60 | 119.33 | 13.28 | 59.66 | 7.48 | 61.16 | 13.07 | 59.66 | 6.51 | 119.33 | 7.94 | 59.66 | 6.42 | 119.33 | 13.83 |
| 59.74 | 8.23 | 59.75 | 11.51 | 119.50 | 13.28 | 59.75 | 7.29 | 61.25 | 13.05 | 59.75 | 6.47 | 119.50 | 8.12 | 59.74 | 6.41 | 119.50 | 13.87 |
| 59.82 | 8.25 | 59.83 | 11.53 | 119.67 | 13.33 | 59.83 | 7.31 | 61.33 | 13.07 | 59.83 | 6.49 | 119.67 | 8.11 | 59.83 | 6.41 | 119.67 | 13.83 |
| 59.90 | 8.41 | 59.92 | 11.55 | 119.83 | 13.33 | 59.91 | 7.56 | 61.41 | 13.09 | 59.91 | 6.51 | 119.83 | 8.02 | 59.91 | 6.49 | 119.83 | 13.80 |
| 59.99 | 8.22 | 60.00 | 11.57 | 120.00 | 13.35 | 60.00 | 7.48 | 61.50 | 13.03 | 60.00 | 6.56 | 120.00 | 8.04 | 59.99 | 6.41 | 120.00 | 13.83 |
| 60.07 | 8.21 | 60.08 | 11.62 | 120.17 | 13.49 | 60.08 | 7.53 | 61.58 | 13.14 | 60.08 | 6.60 | 120.17 | 8.01 | 60.08 | 6.50 | 120.17 | 13.87 |
| 60.15 | 8.40 | 60.17 | 11.58 | 120.33 | 13.51 | 60.16 | 7.60 | 61.66 | 13.08 | 60.16 | 6.64 | 120.33 | 8.11 | 60.16 | 6.56 | 120.33 | 13.78 |
| 60.24 | 8.27 | 60.25 | 11.60 | 120.50 | 13.45 | 60.25 | 7.58 | 61.75 | 13.08 | 60.25 | 6.62 | 120.50 | 8.12 | 60.24 | 6.58 | 120.50 | 13.79 |
| 60.32 | 8.23 | 60.33 | 11.60 | 120.67 | 13.47 | 60.33 | 7.70 | 61.83 | 13.12 | 60.33 | 6.56 | 120.67 | 8.12 | 60.33 | 6.53 | 120.67 | 13.83 |
| 60.40 | 8.24 | 60.42 | 11.68 | 120.83 | 13.47 | 60.41 | 7.78 | 61.91 | 13.01 | 60.41 | 6.58 | 120.83 | 8.11 | 60.41 | 6.63 | 120.83 | 13.89 |
| 60.49 | 8.38 | 60.50 | 11.71 | 121.00 | 13.50 | 60.50 | 7.54 | 62.00 | 13.05 | 60.50 | 6.59 | 121.00 | 8.13 | 60.49 | 6.50 | 121.00 | 13.77 |
| 60.57 | 8.25 | 60.58 | 11.75 | 121.17 | 13.50 | 60.58 | 7.73 | 62.08 | 13.05 | 60.58 | 6.65 | 121.17 | 8.14 | 60.58 | 6.52 | 121.17 | 13.80 |
| 60.65 | 8.25 | 60.67 | 11.80 | 121.33 | 13.52 | 60.66 | 7.59 | 62.16 | 13.07 | 60.66 | 6.74 | 121.33 | 8.12 | 60.66 | 6.54 | 121.33 | 13.83 |
| 60.74 | 8.28 | 60.75 | 11.83 | 121.50 | 13.62 | 60.75 | 7.56 | 62.25 | 13.09 | 60.75 | 6.77 | 121.50 | 8.17 | 60.74 | 6.56 | 121.50 | 13.89 |
| 60.82 | 8.31 | 60.83 | 11.85 | 121.67 | 13.63 | 60.83 | 7.81 | 62.33 | 13.05 | 60.83 | 6.70 | 121.67 | 8.24 | 60.83 | 6.54 | 121.67 | 13.81 |
| 60.90 | 8.35 | 60.92 | 11.87 | 121.83 | 13.64 | 60.91 | 7.64 | 62.41 | 13.06 | 60.91 | 6.75 | 121.83 | 8.24 | 60.91 | 6.66 | 121.83 | 13.83 |
| 60.99 | 8.39 | 61.00 | 11.88 | 122.00 | 13.66 | 61.00 | 7.65 | 62.50 | 13.15 | 61.00 | 6.71 | 122.00 | 8.11 | 60.99 | 6.53 | 122.00 | 13.91 |
| 61.07 | 8.41 | 61.08 | 11.91 | 122.17 | 13.66 | 61.08 | 7.68 | 62.58 | 13.10 | 61.08 | 6.71 | 122.17 | 8.10 | 61.08 | 6.63 | 122.17 | 13.82 |
| 61.15 | 8.34 | 61.17 | 11.94 | 122.33 | 13.69 | 61.16 | 7.73 | 62.66 | 13.14 | 61.16 | 6.77 | 122.33 | 8.26 | 61.16 | 6.69 | 122.33 | 13.88 |
| 61.24 | 8.50 | 61.25 | 12.06 | 122.50 | 13.79 | 61.25 | 7.82 | 62.75 | 13.18 | 61.25 | 6.77 | 122.50 | 8.28 | 61.24 | 6.72 | 122.50 | 13.88 |
| 61.32 | 8.41 | 61.33 | 12.10 | 122.67 | 13.81 | 61.33 | 7.87 | 62.83 | 13.21 | 61.33 | 6.78 | 122.67 | 8.33 | 61.33 | 6.72 | 122.67 | 13.83 |
| 61.40 | 8.44 | 61.42 | 12.10 | 122.83 | 13.69 | 61.41 | 7.74 | 62.91 | 13.23 | 61.41 | 6.81 | 122.83 | 8.30 | 61.41 | 6.75 | 122.83 | 13.85 |
| 61.49 | 8.47 | 61.50 | 11.95 | 123.00 | 13.71 | 61.50 | 7.90 | 63.00 | 13.25 | 61.50 | 6.82 | 123.00 | 8.25 | 61.49 | 6.77 | 123.00 | 13.89 |
| 61.57 | 8.52 | 61.58 | 12.02 | 123.17 | 13.82 | 61.58 | 7.71 | 63.08 | 13.24 | 61.58 | 6.86 | 123.17 | 8.46 | 61.58 | 6.81 | 123.17 | 13.87 |
| 61.65 | 8.56 | 61.67 | 12.04 | 123.33 | 13.70 | 61.66 | 7.92 | 63.16 | 13.31 | 61.66 | 6.92 | 123.33 | 8.38 | 61.66 | 6.89 | 123.33 | 13.96 |
| 61.74 | 8.51 | 61.75 | 11.98 | 123.50 | 13.83 | 61.75 | 7.69 | 63.25 | 13.32 | 61.75 | 6.97 | 123.50 | 8.50 | 61.74 | 6.92 | 123.50 | 13.98 |
| 61.82 | 8.60 | 61.83 | 12.13 | 123.67 | 13.71 | 61.83 | 7.74 | 63.33 | 13.25 | 61.83 | 6.84 | 123.67 | 8.45 | 61.83 | 6.98 | 123.67 | 14.02 |
| 61.90 | 8.66 | 61.92 | 11.98 | 123.83 | 13.75 | 61.91 | 7.95 | 63.41 | 13.29 | 61.91 | 6.83 | 123.83 | 8.48 | 61.91 | 6.92 | 123.83 | 13.96 |
| 61.99 | 8.52 | 62.00 | 12.08 | 124.00 | 13.70 | 62.00 | 7.90 | 63.50 | 13.24 | 62.00 | 6.94 | 124.00 | 8.44 | 61.99 | 7.04 | 124.00 | 14.08 |
| 62.07 | 8.66 | 62.08 | 12.00 | 124.17 | 13.85 | 62.08 | 7.65 | 63.58 | 13.32 | 62.08 | 6.82 | 124.17 | 8.14 | 62.08 | 7.07 | 124.17 | 14.05 |
| 62.15 | 8.52 | 62.17 | 12.01 | 124.33 | 13.77 | 62.16 | 7.68 | 63.66 | 13.26 | 62.16 | 6.82 | 124.33 | 8.26 | 62.16 | 6.94 | 124.33 | 14.03 |
| 62.24 | 8.53 | 62.25 | 11.98 | 124.50 | 13.70 | 62.25 | 7.75 | 63.75 | 13.25 | 62.25 | 6.83 | 124.50 | 8.41 | 62.24 | 6.96 | 124.50 | 14.10 |
| 62.32 | 8.53 | 62.33 | 11.98 | 124.67 | 13.71 | 62.33 | 7.73 | 63.83 | 13.26 | 62.33 | 6.83 | 124.67 | 8.45 | 62.33 | 6.95 | 124.67 | 14.13 |
| 62.40 | 8.67 | 62.42 | 12.05 | 124.83 | 13.75 | 62.41 | 7.74 | 63.91 | 13.30 | 62.41 | 6.93 | 124.83 | 8.63 | 62.41 | 7.08 | 124.83 | 14.13 |

| | | | | | | | | | | | | | | | | | |
|---|---|---|---|---|---|---|---|---|---|---|---|---|---|---|---|---|---|
| 62.49 | 8.63 | 62.50 | 12.05 | 125.00 | 13.87 | 62.50 | 8.00 | 64.00 | 13.29 | 62.50 | 6.84 | 125.00 | 8.66 | 62.49 | 6.90 | 125.00 | 14.07 |
| 62.57 | 8.66 | 62.58 | 11.94 | 125.17 | 13.73 | 62.58 | 7.75 | 64.08 | 13.33 | 62.58 | 6.84 | 125.17 | 8.70 | 62.58 | 7.10 | 125.17 | 14.15 |
| 62.65 | 8.59 | 62.67 | 11.94 | 125.33 | 13.81 | 62.66 | 7.75 | 64.16 | 13.36 | 62.66 | 6.83 | 125.33 | 8.74 | 62.66 | 6.94 | 125.33 | 14.14 |
| 62.74 | 8.76 | 62.75 | 11.96 | 125.50 | 13.83 | 62.75 | 7.78 | 64.25 | 13.30 | 62.75 | 7.00 | 125.50 | 8.68 | 62.74 | 6.97 | 125.50 | 14.17 |
| 62.82 | 8.84 | 62.83 | 12.00 | 125.67 | 13.84 | 62.83 | 7.80 | 64.33 | 13.35 | 62.83 | 6.91 | 125.67 | 8.78 | 62.83 | 7.01 | 125.67 | 14.28 |
| 62.90 | 8.62 | 62.92 | 12.03 | 125.83 | 13.95 | 62.91 | 7.80 | 64.41 | 13.42 | 62.91 | 6.89 | 125.83 | 8.77 | 62.91 | 7.08 | 125.83 | 14.30 |
| 62.99 | 8.63 | 63.00 | 12.06 | 126.00 | 13.94 | 63.00 | 7.78 | 64.50 | 13.44 | 63.00 | 6.90 | 126.00 | 8.76 | 62.99 | 7.05 | 126.00 | 14.18 |
| 63.07 | 8.65 | 63.08 | 11.98 | 126.17 | 13.91 | 63.08 | 7.96 | 64.58 | 13.46 | 63.08 | 6.93 | 126.17 | 8.68 | 63.08 | 7.11 | 126.17 | 14.28 |
| 63.15 | 8.83 | 63.17 | 12.14 | 126.33 | 13.96 | 63.16 | 7.73 | 64.66 | 13.38 | 63.16 | 6.99 | 126.33 | 8.71 | 63.16 | 7.16 | 126.33 | 14.27 |
| 63.24 | 8.63 | 63.25 | 11.97 | 126.50 | 13.96 | 63.25 | 7.74 | 64.75 | 13.44 | 63.25 | 7.05 | 126.50 | 8.80 | 63.24 | 7.13 | 126.50 | 14.27 |
| 63.32 | 8.84 | 63.33 | 12.04 | 126.67 | 14.00 | 63.33 | 7.84 | 64.83 | 13.38 | 63.33 | 7.09 | 126.67 | 8.81 | 63.33 | 7.13 | 126.67 | 14.29 |
| 63.40 | 8.59 | 63.42 | 12.02 | 126.83 | 14.01 | 63.41 | 7.88 | 64.91 | 13.39 | 63.41 | 7.05 | 126.83 | 8.82 | 63.41 | 7.20 | 126.83 | 14.35 |
| 63.49 | 8.60 | 63.50 | 12.02 | 127.00 | 14.08 | 63.50 | 7.88 | 65.00 | 13.48 | 63.50 | 7.08 | 127.00 | 8.88 | 63.49 | 7.23 | 127.00 | 14.38 |
| 63.57 | 8.63 | 63.58 | 12.12 | 127.17 | 14.05 | 63.58 | 8.04 | 65.08 | 13.46 | 63.58 | 7.03 | 127.17 | 8.93 | 63.58 | 7.27 | 127.17 | 14.35 |
| 63.65 | 8.65 | 63.67 | 11.99 | 127.33 | 14.09 | 63.66 | 8.09 | 65.16 | 13.41 | 63.66 | 7.04 | 127.33 | 8.95 | 63.66 | 7.31 | 127.33 | 14.39 |
| 63.74 | 8.83 | 63.75 | 12.06 | 127.50 | 14.10 | 63.75 | 7.81 | 65.25 | 13.36 | 63.75 | 7.12 | 127.50 | 8.94 | 63.74 | 7.26 | 127.50 | 14.42 |
| 63.82 | 8.64 | 63.83 | 12.05 | 127.67 | 14.16 | 63.83 | 7.83 | 65.33 | 13.51 | 63.83 | 7.04 | 127.67 | 8.96 | 63.83 | 7.32 | 127.67 | 14.47 |
| 63.90 | 8.64 | 63.92 | 12.07 | 127.83 | 14.14 | 63.91 | 7.90 | 65.41 | 13.41 | 63.91 | 7.06 | 127.83 | 8.97 | 63.91 | 7.40 | 127.83 | 14.35 |
| 63.99 | 8.85 | 64.00 | 12.15 | 128.00 | 14.24 | 64.00 | 7.90 | 65.50 | 13.42 | 64.00 | 7.11 | 128.00 | 9.01 | 63.99 | 7.46 | 128.00 | 14.37 |
| 64.07 | 8.62 | 64.08 | 12.20 | 128.17 | 14.28 | 64.08 | 7.92 | 65.58 | 13.48 | 64.08 | 7.17 | 128.17 | 9.04 | 64.08 | 7.37 | 128.17 | 14.42 |
| 64.15 | 8.63 | 64.17 | 12.21 | 128.33 | 14.17 | 64.16 | 7.94 | 65.66 | 13.46 | 64.16 | 7.19 | 128.33 | 9.10 | 64.16 | 7.49 | 128.33 | 14.44 |
| 64.24 | 8.67 | 64.25 | 12.13 | 128.50 | 14.16 | 64.25 | 8.15 | 65.75 | 13.38 | 64.25 | 7.18 | 128.50 | 9.10 | 64.24 | 7.33 | 128.50 | 14.37 |
| 64.32 | 8.66 | 64.33 | 12.16 | 128.67 | 14.31 | 64.33 | 7.87 | 65.83 | 13.36 | 64.33 | 7.23 | 128.67 | 9.14 | 64.33 | 7.36 | 128.67 | 14.45 |
| 64.40 | 8.86 | 64.42 | 12.24 | 128.83 | 14.14 | 64.41 | 8.16 | 65.91 | 13.40 | 64.41 | 7.23 | 128.83 | 9.27 | 64.41 | 7.35 | 128.83 | 14.35 |
| 64.49 | 8.62 | 64.50 | 12.29 | 129.00 | 14.16 | 64.50 | 8.11 | 66.00 | 13.44 | 64.50 | 7.19 | 129.00 | 9.27 | 64.49 | 7.38 | 129.00 | 14.37 |
| 64.57 | 8.85 | 64.58 | 12.19 | 129.17 | 14.31 | 64.58 | 8.18 | 66.08 | 13.52 | 64.58 | 7.20 | 129.17 | 9.25 | 64.58 | 7.38 | 129.17 | 14.40 |
| 64.65 | 8.60 | 64.67 | 12.25 | 129.33 | 14.15 | 64.66 | 8.15 | 66.16 | 13.53 | 64.66 | 7.28 | 129.33 | 9.37 | 64.66 | 7.49 | 129.33 | 14.35 |
| 64.74 | 8.62 | 64.75 | 12.21 | 129.50 | 14.15 | 64.75 | 8.26 | 66.25 | 13.51 | 64.75 | 7.19 | 129.50 | 9.36 | 64.74 | 7.37 | 129.50 | 14.38 |
| 64.82 | 8.66 | 64.83 | 12.26 | 129.67 | 14.30 | 64.83 | 8.28 | 66.33 | 13.49 | 64.83 | 7.21 | 129.67 | 9.35 | 64.83 | 7.52 | 129.67 | 14.42 |
| 64.90 | 8.82 | 64.92 | 12.21 | 129.83 | 14.18 | 64.91 | 8.04 | 66.41 | 13.56 | 64.91 | 7.28 | 129.83 | 9.36 | 64.91 | 7.34 | 129.83 | 14.41 |
| 64.99 | 8.63 | 65.00 | 12.22 | 130.00 | 14.14 | 65.00 | 8.08 | 66.50 | 13.59 | 65.00 | 7.17 | 130.00 | 9.37 | 64.99 | 7.42 | 130.00 | 14.43 |
| 65.07 | 8.63 | 65.08 | 12.26 | 130.17 | 14.30 | 65.08 | 8.13 | 66.58 | 13.66 | 65.08 | 7.23 | 130.17 | 9.43 | 65.08 | 7.48 | 130.17 | 14.47 |
| 65.15 | 8.84 | 65.17 | 12.26 | 130.33 | 14.25 | 65.16 | 8.16 | 66.66 | 13.70 | 65.16 | 7.23 | 130.33 | 9.31 | 65.16 | 7.55 | 130.33 | 14.50 |
| 65.24 | 8.65 | 65.25 | 12.34 | 130.50 | 14.27 | 65.25 | 8.26 | 66.75 | 13.60 | 65.25 | 7.33 | 130.50 | 9.35 | 65.24 | 7.57 | 130.50 | 14.50 |
| 65.32 | 8.65 | 65.33 | 12.30 | 130.67 | 14.22 | 65.33 | 8.11 | 66.83 | 13.59 | 65.33 | 7.33 | 130.67 | 9.43 | 65.33 | 7.61 | 130.67 | 14.53 |
| 65.40 | 8.67 | 65.42 | 12.38 | 130.83 | 14.21 | 65.41 | 8.31 | 66.91 | 13.57 | 65.41 | 7.36 | 130.83 | 9.34 | 65.41 | 7.50 | 130.83 | 14.54 |
| 65.49 | 8.68 | 65.50 | 12.42 | 131.00 | 14.23 | 65.50 | 8.07 | 67.00 | 13.51 | 65.50 | 7.33 | 131.00 | 9.40 | 65.49 | 7.62 | 131.00 | 14.49 |
| 65.57 | 8.84 | 65.58 | 12.36 | 131.17 | 14.30 | 65.58 | 8.28 | 67.08 | 13.61 | 65.58 | 7.44 | 131.17 | 9.45 | 65.58 | 7.64 | 131.17 | 14.58 |
| 65.65 | 8.66 | 65.67 | 12.39 | 131.33 | 14.32 | 65.66 | 8.17 | 67.16 | 13.73 | 65.66 | 7.42 | 131.33 | 9.41 | 65.66 | 7.70 | 131.33 | 14.59 |
| 65.74 | 8.66 | 65.75 | 12.43 | 131.50 | 14.35 | 65.75 | 8.14 | 67.25 | 13.72 | 65.75 | 7.37 | 131.50 | 9.48 | 65.74 | 7.73 | 131.50 | 14.61 |
| 65.82 | 8.70 | 65.83 | 12.45 | 131.67 | 14.39 | 65.83 | 8.11 | 67.33 | 13.75 | 65.83 | 7.33 | 131.67 | 9.49 | 65.83 | 7.71 | 131.67 | 14.59 |
| 65.90 | 8.69 | 65.92 | 12.41 | 131.83 | 14.43 | 65.91 | 8.29 | 67.41 | 13.57 | 65.91 | 7.33 | 131.83 | 9.51 | 65.91 | 7.70 | 131.83 | 14.69 |
| 65.99 | 8.69 | 66.00 | 12.46 | 132.00 | 14.38 | 66.00 | 8.08 | 67.50 | 13.52 | 66.00 | 7.46 | 132.00 | 9.54 | 65.99 | 7.84 | 132.00 | 14.63 |
| 66.07 | 8.70 | 66.08 | 12.52 | 132.17 | 14.47 | 66.08 | 8.10 | 67.58 | 13.63 | 66.08 | 7.34 | 132.17 | 9.60 | 66.08 | 7.77 | 132.17 | 14.70 |
| 66.15 | 8.70 | 66.17 | 12.56 | 132.33 | 14.44 | 66.16 | 8.27 | 67.66 | 13.70 | 66.16 | 7.34 | 132.33 | 9.62 | 66.16 | 7.79 | 132.33 | 14.63 |
| 66.24 | 8.80 | 66.25 | 12.63 | 132.50 | 14.45 | 66.25 | 8.09 | 67.75 | 13.73 | 66.25 | 7.35 | 132.50 | 9.65 | 66.24 | 7.80 | 132.50 | 14.68 |
| 66.32 | 8.71 | 66.33 | 12.65 | 132.67 | 14.54 | 66.33 | 8.11 | 67.83 | 13.75 | 66.33 | 7.35 | 132.67 | 9.67 | 66.33 | 7.80 | 132.67 | 14.58 |
| 66.40 | 8.72 | 66.42 | 12.57 | 132.83 | 14.52 | 66.41 | 8.13 | 67.91 | 13.77 | 66.41 | 7.35 | 132.83 | 9.71 | 66.41 | 7.95 | 132.83 | 14.63 |
| 66.49 | 8.75 | 66.50 | 12.58 | 133.00 | 14.53 | 66.50 | 8.33 | 68.00 | 13.80 | 66.50 | 7.49 | 133.00 | 9.74 | 66.49 | 7.75 | 133.00 | 14.66 |
| 66.57 | 8.80 | 66.58 | 12.59 | 133.17 | 14.59 | 66.58 | 8.09 | 68.08 | 13.84 | 66.58 | 7.32 | 133.17 | 9.78 | 66.58 | 7.76 | 133.17 | 14.73 |
| 66.65 | 8.85 | 66.67 | 12.73 | 133.33 | 14.60 | 66.66 | 8.30 | 68.16 | 13.71 | 66.66 | 7.42 | 133.33 | 9.77 | 66.66 | 7.81 | 133.33 | 14.61 |

| | | | | | | | | | | | | | | | | | |
|---|---|---|---|---|---|---|---|---|---|---|---|---|---|---|---|---|---|
| 66.74 | 8.87 | 66.75 | 12.78 | 133.50 | 14.67 | 66.75 | 8.10 | 68.25 | 13.77 | 66.75 | 7.40 | 133.50 | 9.85 | 66.74 | 7.82 | 133.50 | 14.66 |
| 66.82 | 8.90 | 66.83 | 12.83 | 133.67 | 14.72 | 66.83 | 8.21 | 68.33 | 13.78 | 66.83 | 7.47 | 133.67 | 9.73 | 66.83 | 7.90 | 133.67 | 14.64 |
| 66.90 | 8.90 | 66.92 | 12.72 | 133.83 | 14.63 | 66.91 | 8.20 | 68.41 | 13.80 | 66.91 | 7.54 | 133.83 | 9.81 | 66.91 | 7.81 | 133.83 | 14.73 |
| 66.99 | 8.93 | 67.00 | 12.74 | 134.00 | 14.61 | 67.00 | 8.21 | 68.50 | 13.81 | 67.00 | 7.59 | 134.00 | 9.85 | 66.99 | 7.80 | 134.00 | 14.63 |
| 67.07 | 8.94 | 67.08 | 12.82 | 134.17 | 14.76 | 67.08 | 8.29 | 68.58 | 13.83 | 67.08 | 7.60 | 134.17 | 9.82 | 67.08 | 7.92 | 134.17 | 14.65 |
| 67.15 | 9.01 | 67.17 | 12.70 | 134.33 | 14.61 | 67.16 | 8.34 | 68.66 | 13.86 | 67.16 | 7.55 | 134.33 | 9.90 | 67.16 | 7.79 | 134.33 | 14.70 |
| 67.24 | 9.02 | 67.25 | 12.79 | 134.50 | 14.62 | 67.25 | 8.12 | 68.75 | 13.89 | 67.25 | 7.59 | 134.50 | 9.93 | 67.24 | 7.82 | 134.50 | 14.64 |
| 67.32 | 9.07 | 67.33 | 12.75 | 134.67 | 14.75 | 67.33 | 8.35 | 68.83 | 13.91 | 67.33 | 7.57 | 134.67 | 9.94 | 67.33 | 7.79 | 134.67 | 14.64 |
| 67.40 | 8.95 | 67.42 | 12.78 | 134.83 | 14.57 | 67.41 | 8.43 | 68.91 | 13.92 | 67.41 | 7.71 | 134.83 | 9.90 | 67.41 | 7.82 | 134.83 | 14.70 |
| 67.49 | 8.97 | 67.50 | 12.81 | 135.00 | 14.59 | 67.50 | 8.29 | 69.00 | 13.93 | 67.50 | 7.75 | 135.00 | 9.93 | 67.49 | 7.85 | 135.00 | 14.65 |
| 67.57 | 9.00 | 67.58 | 12.84 | 135.17 | 14.74 | 67.58 | 8.27 | 69.08 | 13.95 | 67.58 | 7.66 | 135.17 | 9.96 | 67.58 | 7.83 | 135.17 | 14.62 |
| 67.65 | 9.03 | 67.67 | 12.86 | 135.33 | 14.60 | 67.66 | 8.48 | 69.16 | 13.99 | 67.66 | 7.67 | 135.33 | 9.95 | 67.66 | 7.81 | 135.33 | 14.70 |
| 67.74 | 9.12 | 67.75 | 12.85 | 135.50 | 14.60 | 67.75 | 8.24 | 69.25 | 13.85 | 67.75 | 7.77 | 135.50 | 10.04 | 67.74 | 7.84 | 135.50 | 14.64 |
| 67.82 | 9.14 | 67.83 | 12.87 | 135.67 | 14.75 | 67.83 | 8.25 | 69.33 | 13.87 | 67.83 | 7.66 | 135.67 | 10.04 | 67.83 | 7.94 | 135.67 | 14.61 |
| 67.90 | 9.17 | 67.92 | 12.94 | 135.83 | 14.60 | 67.91 | 8.30 | 69.41 | 13.96 | 67.91 | 7.66 | 135.83 | 10.06 | 67.91 | 7.96 | 135.83 | 14.66 |
| 67.99 | 9.22 | 68.00 | 12.96 | 136.00 | 14.59 | 68.00 | 8.30 | 69.50 | 13.93 | 68.00 | 7.76 | 136.00 | 10.05 | 67.99 | 7.99 | 136.00 | 14.64 |
| 68.07 | 9.06 | 68.08 | 13.01 | 136.17 | 14.75 | 68.08 | 8.32 | 69.58 | 13.87 | 68.08 | 7.74 | 136.17 | 10.08 | 68.08 | 8.02 | 136.17 | 14.62 |
| 68.15 | 9.10 | 68.17 | 13.02 | 136.33 | 14.60 | 68.16 | 8.53 | 69.66 | 14.00 | 68.16 | 7.73 | 136.33 | 10.12 | 68.16 | 8.01 | 136.33 | 14.69 |
| 68.24 | 9.10 | 68.25 | 13.02 | 136.50 | 14.76 | 68.25 | 8.35 | 69.75 | 13.88 | 68.25 | 7.73 | 136.50 | 10.02 | 68.24 | 8.08 | 136.50 | 14.72 |
| 68.32 | 9.25 | 68.33 | 13.06 | 136.67 | 14.61 | 68.33 | 8.32 | 69.83 | 14.01 | 68.33 | 7.81 | 136.67 | 10.16 | 68.33 | 8.12 | 136.67 | 14.60 |
| 68.40 | 9.08 | 68.42 | 13.10 | 136.83 | 14.63 | 68.41 | 8.41 | 69.91 | 13.95 | 68.41 | 7.87 | 136.83 | 10.21 | 68.41 | 8.10 | 136.83 | 14.67 |
| 68.49 | 9.09 | 68.50 | 13.16 | 137.00 | 14.73 | 68.50 | 8.53 | 70.00 | 13.93 | 68.50 | 7.91 | 137.00 | 10.24 | 68.49 | 8.15 | 137.00 | 14.78 |
| 68.57 | 9.28 | 68.58 | 13.13 | 137.17 | 14.74 | 68.58 | 8.46 | 70.08 | 13.94 | 68.58 | 7.80 | 137.17 | 10.16 | 68.58 | 8.20 | 137.17 | 14.76 |
| 68.65 | 9.09 | 68.67 | 13.17 | 137.33 | 14.62 | 68.66 | 8.46 | 70.16 | 13.94 | 68.66 | 7.92 | 137.33 | 10.26 | 68.66 | 8.18 | 137.33 | 14.72 |
| 68.74 | 9.10 | 68.75 | 13.19 | 137.50 | 14.63 | 68.75 | 8.67 | 70.25 | 13.98 | 68.75 | 7.80 | 137.50 | 10.20 | 68.74 | 8.16 | 137.50 | 14.80 |
| 68.82 | 9.14 | 68.83 | 13.25 | 137.67 | 14.73 | 68.83 | 8.64 | 70.33 | 14.02 | 68.83 | 7.93 | 137.67 | 10.19 | 68.83 | 8.08 | 137.67 | 14.80 |
| 68.90 | 9.14 | 68.92 | 13.31 | 137.83 | 14.71 | 68.91 | 8.45 | 70.41 | 14.11 | 68.91 | 7.81 | 137.83 | 10.23 | 68.91 | 8.22 | 137.83 | 14.79 |
| 68.99 | 9.13 | 69.00 | 13.33 | 138.00 | 14.71 | 69.00 | 8.47 | 70.50 | 14.09 | 69.00 | 7.83 | 138.00 | 10.17 | 68.99 | 8.30 | 138.00 | 14.87 |
| 69.07 | 9.23 | 69.08 | 13.32 | 138.17 | 14.82 | 69.08 | 8.57 | 70.58 | 14.16 | 69.08 | 7.83 | 138.17 | 10.12 | 69.08 | 8.24 | 138.17 | 14.87 |
| 69.15 | 9.19 | 69.17 | 13.32 | 138.33 | 14.84 | 69.16 | 8.56 | 70.66 | 14.08 | 69.16 | 7.92 | 138.33 | 10.21 | 69.16 | 8.24 | 138.33 | 14.81 |
| 69.24 | 9.28 | 69.25 | 13.32 | 138.50 | 14.78 | 69.25 | 8.63 | 70.75 | 14.14 | 69.25 | 7.81 | 138.50 | 10.24 | 69.24 | 8.32 | 138.50 | 14.89 |
| 69.32 | 9.34 | 69.33 | 13.29 | 138.67 | 14.83 | 69.33 | 8.48 | 70.83 | 14.07 | 69.33 | 7.92 | 138.67 | 10.18 | 69.33 | 8.19 | 138.67 | 14.96 |
| 69.40 | 9.37 | 69.42 | 13.28 | 138.83 | 14.87 | 69.41 | 8.42 | 70.91 | 14.06 | 69.41 | 7.82 | 138.83 | 10.22 | 69.41 | 8.21 | 138.83 | 14.94 |
| 69.49 | 9.43 | 69.50 | 13.33 | 139.00 | 14.93 | 69.50 | 8.48 | 71.00 | 14.09 | 69.50 | 7.85 | 139.00 | 10.30 | 69.49 | 8.23 | 139.00 | 14.94 |
| 69.57 | 9.45 | 69.58 | 13.35 | 139.17 | 14.94 | 69.58 | 8.51 | 71.08 | 14.10 | 69.58 | 7.83 | 139.17 | 10.26 | 69.58 | 8.26 | 139.17 | 15.05 |
| 69.65 | 9.49 | 69.67 | 13.36 | 139.33 | 14.95 | 69.66 | 8.49 | 71.16 | 14.15 | 69.66 | 7.94 | 139.33 | 10.32 | 69.66 | 8.26 | 139.33 | 15.03 |
| 69.74 | 9.42 | 69.75 | 13.41 | 139.50 | 14.99 | 69.75 | 8.51 | 71.25 | 14.09 | 69.75 | 7.83 | 139.50 | 10.33 | 69.74 | 8.23 | 139.50 | 15.04 |
| 69.82 | 9.41 | 69.83 | 13.42 | 139.67 | 15.02 | 69.83 | 8.63 | 71.33 | 14.05 | 69.83 | 7.92 | 139.67 | 10.34 | 69.83 | 8.35 | 139.67 | 15.13 |
| 69.90 | 9.43 | 69.92 | 13.37 | 139.83 | 14.95 | 69.91 | 8.48 | 71.41 | 14.03 | 69.91 | 7.80 | 139.83 | 10.32 | 69.91 | 8.19 | 139.83 | 15.07 |
| 69.99 | 9.44 | 70.00 | 13.37 | 140.00 | 14.98 | 70.00 | 8.67 | 71.50 | 14.05 | 70.00 | 7.86 | 140.00 | 10.36 | 69.99 | 8.22 | 140.00 | 15.13 |
| 70.07 | 9.44 | 70.08 | 13.41 | 140.17 | 14.97 | 70.08 | 8.51 | 71.58 | 14.08 | 70.08 | 7.84 | 140.17 | 10.38 | 70.08 | 8.29 | 140.17 | 15.01 |
| 70.15 | 9.56 | 70.17 | 13.44 | 140.33 | 14.99 | 70.16 | 8.52 | 71.66 | 14.10 | 70.16 | 7.85 | 140.33 | 10.42 | 70.16 | 8.34 | 140.33 | 15.07 |
| 70.24 | 9.41 | 70.25 | 13.47 | 140.50 | 15.09 | 70.25 | 8.52 | 71.75 | 14.09 | 70.25 | 7.85 | 140.50 | 10.43 | 70.24 | 8.39 | 140.50 | 15.14 |
| 70.32 | 9.42 | 70.33 | 13.50 | 140.67 | 15.05 | 70.33 | 8.51 | 71.83 | 14.05 | 70.33 | 7.83 | 140.67 | 10.46 | 70.33 | 8.41 | 140.67 | 15.00 |
| 70.40 | 9.44 | 70.42 | 13.47 | 140.83 | 15.14 | 70.41 | 8.66 | 71.91 | 14.19 | 70.41 | 7.91 | 140.83 | 10.49 | 70.41 | 8.45 | 140.83 | 15.07 |
| 70.49 | 9.45 | 70.50 | 13.48 | 141.00 | 15.19 | 70.50 | 8.47 | 72.00 | 14.02 | 70.50 | 7.85 | 141.00 | 10.46 | 70.49 | 8.37 | 141.00 | 15.13 |
| 70.57 | 9.45 | 70.58 | 13.48 | 141.17 | 15.05 | 70.58 | 8.47 | 72.08 | 14.09 | 70.58 | 7.86 | 141.17 | 10.52 | 70.58 | 8.42 | 141.17 | 15.07 |
| 70.65 | 9.51 | 70.67 | 13.60 | 141.33 | 15.07 | 70.66 | 8.63 | 72.16 | 14.10 | 70.66 | 7.83 | 141.33 | 10.56 | 70.66 | 8.54 | 141.33 | 15.05 |
| 70.74 | 9.47 | 70.75 | 13.64 | 141.50 | 15.23 | 70.75 | 8.50 | 72.25 | 14.07 | 70.75 | 7.83 | 141.50 | 10.56 | 70.74 | 8.57 | 141.50 | 15.06 |
| 70.82 | 9.53 | 70.83 | 13.68 | 141.67 | 15.04 | 70.83 | 8.53 | 72.33 | 14.08 | 70.83 | 7.89 | 141.67 | 10.60 | 70.83 | 8.53 | 141.67 | 15.04 |
| 70.90 | 9.63 | 70.92 | 13.58 | 141.83 | 15.07 | 70.91 | 8.51 | 72.41 | 14.08 | 70.91 | 7.88 | 141.83 | 10.65 | 70.91 | 8.59 | 141.83 | 15.11 |

| | | | | | | | | | | | | | | | | | |
|---|---|---|---|---|---|---|---|---|---|---|---|---|---|---|---|---|---|
| 70.99 | 9.67 | 71.00 | 13.60 | 142.00 | 15.21 | 71.00 | 8.62 | 72.50 | 14.16 | 71.00 | 7.87 | 142.00 | 10.67 | 70.99 | 8.58 | 142.00 | 15.07 |
| 71.07 | 9.52 | 71.08 | 13.69 | 142.17 | 15.06 | 71.08 | 8.44 | 72.58 | 14.09 | 71.08 | 7.93 | 142.17 | 10.63 | 71.08 | 8.50 | 142.17 | 15.04 |
| 71.15 | 9.51 | 71.17 | 13.57 | 142.33 | 15.05 | 71.16 | 8.48 | 72.66 | 14.12 | 71.16 | 7.97 | 142.33 | 10.71 | 71.16 | 8.57 | 142.33 | 15.10 |
| 71.24 | 9.71 | 71.25 | 13.67 | 142.50 | 15.19 | 71.25 | 8.49 | 72.75 | 14.11 | 71.25 | 8.07 | 142.50 | 10.74 | 71.24 | 8.71 | 142.50 | 15.07 |
| 71.32 | 9.49 | 71.33 | 13.59 | 142.67 | 15.03 | 71.33 | 8.52 | 72.83 | 14.18 | 71.33 | 8.03 | 142.67 | 10.70 | 71.33 | 8.64 | 142.67 | 15.11 |
| 71.40 | 9.52 | 71.42 | 13.61 | 142.83 | 15.04 | 71.41 | 8.51 | 72.91 | 14.22 | 71.41 | 8.03 | 142.83 | 10.79 | 71.41 | 8.75 | 142.83 | 15.06 |
| 71.49 | 9.70 | 71.50 | 13.67 | 143.00 | 15.21 | 71.50 | 8.64 | 73.00 | 14.19 | 71.50 | 8.00 | 143.00 | 10.80 | 71.49 | 8.60 | 143.00 | 15.10 |
| 71.57 | 9.55 | 71.58 | 13.56 | 143.17 | 15.05 | 71.58 | 8.63 | 73.08 | 14.22 | 71.58 | 8.02 | 143.17 | 10.76 | 71.58 | 8.63 | 143.17 | 15.18 |
| 71.65 | 9.56 | 71.67 | 13.53 | 143.33 | 15.05 | 71.66 | 8.45 | 73.16 | 14.24 | 71.66 | 8.01 | 143.33 | 10.84 | 71.66 | 8.79 | 143.33 | 15.09 |
| 71.74 | 9.58 | 71.75 | 13.62 | 143.50 | 15.08 | 71.75 | 8.49 | 73.25 | 14.26 | 71.75 | 8.06 | 143.50 | 10.93 | 71.74 | 8.63 | 143.50 | 15.17 |
| 71.82 | 9.69 | 71.83 | 13.63 | 143.67 | 15.09 | 71.83 | 8.53 | 73.33 | 14.27 | 71.83 | 8.02 | 143.67 | 10.90 | 71.83 | 8.69 | 143.67 | 15.21 |
| 71.90 | 9.53 | 71.92 | 13.68 | 143.83 | 15.18 | 71.91 | 8.56 | 73.41 | 14.32 | 71.91 | 8.03 | 143.83 | 10.94 | 71.91 | 8.73 | 143.83 | 15.21 |
| 71.99 | 9.56 | 72.00 | 13.63 | 144.00 | 15.19 | 72.00 | 8.71 | 73.50 | 14.23 | 72.00 | 8.11 | 144.00 | 11.02 | 71.99 | 8.73 | 144.00 | 15.26 |
| 72.07 | 9.55 | 72.08 | 13.55 | 144.17 | 15.11 | 72.08 | 8.78 | 73.58 | 14.30 | 72.08 | 8.10 | 144.17 | 11.00 | 72.08 | 8.80 | 144.17 | 15.22 |
| 72.15 | 9.60 | 72.17 | 13.52 | 144.33 | 15.16 | 72.16 | 8.78 | 73.66 | 14.23 | 72.16 | 8.14 | 144.33 | 11.08 | 72.16 | 8.78 | 144.33 | 15.28 |
| 72.24 | 9.60 | 72.25 | 13.66 | 144.50 | 15.28 | 72.25 | 8.65 | 73.75 | 14.27 | 72.25 | 8.16 | 144.50 | 11.04 | 72.24 | 8.82 | 144.50 | 15.17 |
| 72.32 | 9.62 | 72.33 | 13.68 | 144.67 | 15.33 | 72.33 | 8.65 | 73.83 | 14.29 | 72.33 | 8.14 | 144.67 | 11.04 | 72.33 | 8.87 | 144.67 | 15.25 |
| 72.40 | 9.61 | 72.42 | 13.74 | 144.83 | 15.24 | 72.41 | 8.65 | 73.91 | 14.28 | 72.41 | 8.22 | 144.83 | 10.89 | 72.41 | 8.91 | 144.83 | 15.25 |
| 72.49 | 9.64 | 72.50 | 13.79 | 145.00 | 15.26 | 72.50 | 8.71 | 74.00 | 14.31 | 72.50 | 8.23 | 145.00 | 11.06 | 72.49 | 8.85 | 145.00 | 15.24 |
| 72.57 | 9.68 | 72.58 | 13.79 | 145.17 | 15.31 | 72.58 | 8.80 | 74.08 | 14.35 | 72.58 | 8.15 | 145.17 | 11.10 | 72.58 | 8.85 | 145.17 | 15.26 |
| 72.65 | 9.77 | 72.67 | 13.68 | 145.33 | 15.21 | 72.66 | 8.68 | 74.16 | 14.38 | 72.66 | 8.17 | 145.33 | 11.06 | 72.66 | 8.84 | 145.33 | 15.30 |
| 72.74 | 9.81 | 72.75 | 13.72 | 145.50 | 15.25 | 72.75 | 8.63 | 74.25 | 14.38 | 72.75 | 8.25 | 145.50 | 11.10 | 72.74 | 8.88 | 145.50 | 15.38 |
| 72.82 | 9.70 | 72.83 | 13.74 | 145.67 | 15.29 | 72.83 | 8.66 | 74.33 | 14.40 | 72.83 | 8.16 | 145.67 | 11.12 | 72.83 | 8.96 | 145.67 | 15.38 |
| 72.90 | 9.83 | 72.92 | 13.85 | 145.83 | 15.30 | 72.91 | 8.73 | 74.41 | 14.43 | 72.91 | 8.18 | 145.83 | 11.15 | 72.91 | 8.99 | 145.83 | 15.34 |
| 72.99 | 9.70 | 73.00 | 13.88 | 146.00 | 15.33 | 73.00 | 8.80 | 74.50 | 14.48 | 73.00 | 8.23 | 146.00 | 11.17 | 72.99 | 8.99 | 146.00 | 15.37 |
| 73.07 | 9.84 | 73.08 | 13.80 | 146.17 | 14.76 | 73.08 | 8.90 | 74.58 | 14.48 | 73.08 | 8.21 | 146.17 | 11.14 | 73.08 | 9.06 | 146.17 | 15.39 |
| 73.15 | 9.74 | 73.17 | 13.81 | 146.33 | 15.14 | 73.16 | 8.86 | 74.66 | 14.39 | 73.16 | 8.27 | 146.33 | 11.15 | 73.16 | 8.98 | 146.33 | 15.38 |
| 73.24 | 9.70 | 73.25 | 13.81 | 146.50 | 15.36 | 73.25 | 9.00 | 74.75 | 14.45 | 73.25 | 8.27 | 146.50 | 11.18 | 73.24 | 9.01 | 146.50 | 15.52 |
| 73.32 | 9.76 | 73.33 | 13.79 | 146.67 | 15.35 | 73.33 | 8.99 | 74.83 | 14.43 | 73.33 | 8.26 | 146.67 | 11.15 | 73.33 | 9.00 | 146.67 | 15.49 |
| 73.40 | 9.82 | 73.42 | 13.75 | 146.83 | 15.44 | 73.41 | 8.79 | 74.91 | 14.50 | 73.41 | 8.35 | 146.83 | 11.25 | 73.41 | 9.14 | 146.83 | 15.43 |
| 73.49 | 9.86 | 73.50 | 13.76 | 147.00 | 15.39 | 73.50 | 8.84 | 75.00 | 14.39 | 73.50 | 8.31 | 147.00 | 11.25 | 73.49 | 9.07 | 147.00 | 15.43 |
| 73.57 | 9.84 | 73.58 | 13.78 | 147.17 | 15.40 | 73.58 | 8.83 | 75.08 | 14.44 | 73.58 | 8.36 | 147.17 | 11.19 | 73.58 | 9.06 | 147.17 | 15.49 |
| 73.65 | 9.87 | 73.67 | 13.80 | 147.33 | 15.42 | 73.66 | 8.90 | 75.16 | 14.46 | 73.66 | 8.31 | 147.33 | 11.29 | 73.66 | 9.10 | 147.33 | 15.41 |
| 73.74 | 9.96 | 73.75 | 13.87 | 147.50 | 15.44 | 73.75 | 9.03 | 75.25 | 14.48 | 73.75 | 8.42 | 147.50 | 11.32 | 73.74 | 9.18 | 147.50 | 15.46 |
| 73.82 | 9.86 | 73.83 | 13.87 | 147.67 | 15.50 | 73.83 | 9.00 | 75.33 | 14.53 | 73.83 | 8.30 | 147.67 | 11.33 | 73.83 | 9.04 | 147.67 | 15.51 |
| 73.90 | 9.91 | 73.92 | 13.77 | 147.83 | 15.52 | 73.91 | 8.84 | 75.41 | 14.50 | 73.91 | 8.44 | 147.83 | 11.29 | 73.91 | 9.07 | 147.83 | 15.41 |
| 73.99 | 9.98 | 74.00 | 13.78 | 148.00 | 15.56 | 74.00 | 8.79 | 75.50 | 14.57 | 74.00 | 8.31 | 148.00 | 11.42 | 73.99 | 9.08 | 148.00 | 15.56 |
| 74.07 | 9.94 | 74.08 | 13.83 | 148.17 | 15.59 | 74.08 | 8.99 | 75.58 | 14.60 | 74.08 | 8.32 | 148.17 | 11.39 | 74.08 | 9.10 | 148.17 | 15.54 |
| 74.15 | 10.02 | 74.17 | 13.81 | 148.33 | 15.63 | 74.16 | 9.11 | 75.66 | 14.62 | 74.16 | 8.30 | 148.33 | 11.38 | 74.16 | 9.19 | 148.33 | 15.53 |
| 74.24 | 10.07 | 74.25 | 13.83 | 148.50 | 15.48 | 74.25 | 9.02 | 75.75 | 14.64 | 74.25 | 8.45 | 148.50 | 11.45 | 74.24 | 9.07 | 148.50 | 15.47 |
| 74.32 | 10.16 | 74.33 | 13.83 | 148.67 | 15.51 | 74.33 | 9.17 | 75.83 | 14.60 | 74.33 | 8.31 | 148.67 | 11.51 | 74.33 | 9.04 | 148.67 | 15.53 |
| 74.40 | 9.92 | 74.42 | 13.82 | 148.83 | 15.64 | 74.41 | 8.95 | 75.91 | 14.65 | 74.41 | 8.29 | 148.83 | 11.37 | 74.41 | 9.20 | 148.83 | 15.50 |
| 74.49 | 9.99 | 74.50 | 13.86 | 149.00 | 15.46 | 74.50 | 9.21 | 76.00 | 14.57 | 74.50 | 8.39 | 149.00 | 11.41 | 74.49 | 9.10 | 149.00 | 15.44 |
| 74.57 | 9.99 | 74.58 | 13.89 | 149.17 | 15.51 | 74.58 | 8.94 | 76.08 | 14.66 | 74.58 | 8.35 | 149.17 | 11.42 | 74.58 | 9.11 | 149.17 | 15.50 |
| 74.65 | 10.13 | 74.67 | 13.98 | 149.33 | 15.64 | 74.66 | 8.95 | 76.16 | 14.63 | 74.66 | 8.33 | 149.33 | 11.47 | 74.66 | 9.16 | 149.33 | 15.53 |
| 74.74 | 9.92 | 74.75 | 14.02 | 149.50 | 15.48 | 74.75 | 9.12 | 76.25 | 14.75 | 74.75 | 8.34 | 149.50 | 11.45 | 74.74 | 9.17 | 149.50 | 15.46 |
| 74.82 | 9.94 | 74.83 | 13.95 | 149.67 | 15.48 | 74.83 | 9.19 | 76.33 | 14.76 | 74.83 | 8.33 | 149.67 | 11.38 | 74.83 | 9.18 | 149.67 | 15.50 |
| 74.90 | 9.95 | 74.92 | 13.93 | 149.83 | 15.66 | 74.91 | 9.20 | 76.41 | 14.76 | 74.91 | 8.32 | 149.83 | 11.46 | 74.91 | 9.18 | 149.83 | 15.48 |
| 74.99 | 10.13 | 75.00 | 13.94 | 150.00 | 15.51 | 75.00 | 8.77 | 76.50 | 14.77 | 75.00 | 8.40 | 150.00 | 11.44 | 74.99 | 9.26 | 150.00 | 15.47 |
| 75.07 | 9.97 | 75.08 | 13.97 | 150.17 | 15.48 | 75.08 | 8.97 | 76.58 | 14.84 | 75.08 | 8.30 | 150.17 | 11.49 | 75.08 | 9.32 | 150.17 | 15.46 |
| 75.15 | 9.96 | 75.17 | 13.99 | 150.33 | 15.49 | 75.16 | 8.99 | 76.66 | 14.76 | 75.16 | 8.35 | 150.33 | 11.47 | 75.16 | 9.24 | 150.33 | 15.44 |

| | | | | | | | | | | | | | | | | | |
|---|---|---|---|---|---|---|---|---|---|---|---|---|---|---|---|---|---|
| 75.24 | 10.14 | 75.25 | 13.98 | 150.50 | 15.65 | 75.25 | 9.02 | 76.75 | 14.86 | 75.25 | 8.44 | 150.50 | 11.51 | 75.24 | 9.26 | 150.50 | 15.50 |
| 75.32 | 9.92 | 75.33 | 14.01 | 150.67 | 15.52 | 75.33 | 9.03 | 76.83 | 14.74 | 75.33 | 8.48 | 150.67 | 11.54 | 75.33 | 9.29 | 150.67 | 15.50 |
| 75.40 | 9.96 | 75.42 | 13.99 | 150.83 | 15.54 | 75.41 | 9.16 | 76.91 | 14.77 | 75.41 | 8.50 | 150.83 | 11.49 | 75.41 | 9.35 | 150.83 | 15.42 |
| 75.49 | 9.97 | 75.50 | 14.01 | 151.00 | 15.66 | 75.50 | 9.00 | 77.00 | 14.77 | 75.50 | 8.51 | 151.00 | 11.56 | 75.49 | 9.34 | 151.00 | 15.49 |
| 75.57 | 10.15 | 75.58 | 14.08 | 151.17 | 15.53 | 75.58 | 9.20 | 77.08 | 14.86 | 75.58 | 8.51 | 151.17 | 11.57 | 75.58 | 9.36 | 151.17 | 15.52 |
| 75.65 | 9.97 | 75.67 | 14.15 | 151.33 | 15.59 | 75.66 | 9.01 | 77.16 | 14.75 | 75.66 | 8.52 | 151.33 | 11.58 | 75.66 | 9.42 | 151.33 | 15.50 |
| 75.74 | 9.97 | 75.75 | 14.06 | 151.50 | 15.48 | 75.75 | 9.00 | 77.25 | 14.77 | 75.75 | 8.51 | 151.50 | 11.54 | 75.74 | 9.41 | 151.50 | 15.55 |
| 75.82 | 10.15 | 75.83 | 14.08 | 151.67 | 15.56 | 75.83 | 9.05 | 77.33 | 14.83 | 75.83 | 8.49 | 151.67 | 11.63 | 75.83 | 9.43 | 151.67 | 15.61 |
| 75.90 | 9.94 | 75.92 | 14.20 | 151.83 | 15.69 | 75.91 | 9.04 | 77.41 | 14.77 | 75.91 | 8.58 | 151.83 | 11.54 | 75.91 | 9.50 | 151.83 | 15.59 |
| 75.99 | 10.00 | 76.00 | 14.22 | 152.00 | 15.66 | 76.00 | 9.17 | 77.50 | 14.84 | 76.00 | 8.50 | 152.00 | 11.53 | 75.99 | 9.53 | 152.00 | 15.57 |
| 76.07 | 9.98 | 76.08 | 14.06 | 152.17 | 15.73 | 76.08 | 8.98 | 77.58 | 14.73 | 76.08 | 8.51 | 152.17 | 11.62 | 76.08 | 9.57 | 152.17 | 15.66 |
| 76.15 | 10.00 | 76.17 | 14.24 | 152.33 | 15.75 | 76.16 | 9.03 | 77.66 | 14.85 | 76.16 | 8.53 | 152.33 | 11.60 | 76.16 | 9.59 | 152.33 | 15.65 |
| 76.24 | 10.12 | 76.25 | 14.18 | 152.50 | 15.73 | 76.25 | 9.18 | 77.75 | 14.73 | 76.25 | 8.51 | 152.50 | 11.64 | 76.24 | 9.42 | 152.50 | 15.64 |
| 76.32 | 9.98 | 76.33 | 14.17 | 152.67 | 15.69 | 76.33 | 9.07 | 77.83 | 14.74 | 76.33 | 8.59 | 152.67 | 11.65 | 76.33 | 9.47 | 152.67 | 15.72 |
| 76.40 | 9.99 | 76.42 | 14.18 | 152.83 | 15.72 | 76.41 | 9.03 | 77.91 | 14.82 | 76.41 | 8.61 | 152.83 | 11.64 | 76.41 | 9.56 | 152.83 | 15.76 |
| 76.49 | 10.13 | 76.50 | 14.17 | 153.00 | 15.79 | 76.50 | 9.05 | 78.00 | 14.76 | 76.50 | 8.62 | 153.00 | 11.72 | 76.49 | 9.55 | 153.00 | 15.72 |
| 76.57 | 10.05 | 76.58 | 14.15 | 153.17 | 15.84 | 76.58 | 9.18 | 78.08 | 14.84 | 76.58 | 8.54 | 153.17 | 11.71 | 76.58 | 9.61 | 153.17 | 15.79 |
| 76.65 | 10.06 | 76.67 | 14.11 | 153.33 | 15.81 | 76.66 | 9.05 | 78.16 | 14.76 | 76.66 | 8.63 | 153.33 | 11.69 | 76.66 | 9.63 | 153.33 | 15.79 |
| 76.74 | 10.07 | 76.75 | 14.13 | 153.50 | 15.89 | 76.75 | 9.04 | 78.25 | 14.74 | 76.75 | 8.68 | 153.50 | 11.79 | 76.74 | 9.63 | 153.50 | 15.76 |
| 76.82 | 10.19 | 76.83 | 14.14 | 153.67 | 15.90 | 76.83 | 9.16 | 78.33 | 14.82 | 76.83 | 8.73 | 153.67 | 11.84 | 76.83 | 9.71 | 153.67 | 15.74 |
| 76.90 | 10.25 | 76.92 | 14.14 | 153.83 | 15.84 | 76.91 | 9.05 | 78.41 | 14.80 | 76.91 | 8.66 | 153.83 | 11.86 | 76.91 | 9.73 | 153.83 | 15.83 |
| 76.99 | 10.13 | 77.00 | 14.12 | 154.00 | 15.88 | 77.00 | 9.05 | 78.50 | 14.79 | 77.00 | 8.66 | 154.00 | 11.86 | 76.99 | 9.66 | 154.00 | 15.82 |
| 77.07 | 10.27 | 77.08 | 14.14 | 154.17 | 15.89 | 77.08 | 9.03 | 78.58 | 14.82 | 77.08 | 8.73 | 154.17 | 11.97 | 77.08 | 9.73 | 154.17 | 15.73 |
| 77.15 | 10.12 | 77.17 | 14.13 | 154.33 | 15.88 | 77.16 | 9.03 | 78.66 | 14.81 | 77.16 | 8.69 | 154.33 | 11.98 | 77.16 | 9.61 | 154.33 | 15.84 |
| 77.24 | 10.21 | 77.25 | 14.23 | 154.50 | 15.98 | 77.25 | 9.18 | 78.75 | 14.84 | 77.25 | 8.70 | 154.50 | 12.03 | 77.24 | 9.77 | 154.50 | 15.89 |
| 77.32 | 10.29 | 77.33 | 14.14 | 154.67 | 16.02 | 77.33 | 9.03 | 78.83 | 14.89 | 77.33 | 8.67 | 154.67 | 11.99 | 77.33 | 9.60 | 154.67 | 15.93 |
| 77.40 | 10.31 | 77.42 | 14.17 | 154.83 | 16.06 | 77.41 | 9.04 | 78.91 | 14.93 | 77.41 | 8.66 | 154.83 | 11.98 | 77.41 | 9.73 | 154.83 | 15.93 |
| 77.49 | 10.34 | 77.50 | 14.14 | 155.00 | 15.95 | 77.50 | 9.03 | 79.00 | 14.92 | 77.50 | 8.69 | 155.00 | 11.94 | 77.49 | 9.65 | 155.00 | 15.88 |
| 77.57 | 10.45 | 77.58 | 14.17 | 155.17 | 16.08 | 77.58 | 9.04 | 79.08 | 14.90 | 77.58 | 8.72 | 155.17 | 12.03 | 77.58 | 9.64 | 155.17 | 15.83 |
| 77.65 | 10.50 | 77.67 | 14.16 | 155.33 | 15.92 | 77.66 | 9.07 | 79.16 | 14.90 | 77.66 | 8.74 | 155.33 | 11.99 | 77.66 | 9.65 | 155.33 | 15.95 |
| 77.74 | 10.57 | 77.75 | 14.15 | 155.50 | 15.95 | 77.75 | 9.19 | 79.25 | 14.91 | 77.75 | 8.79 | 155.50 | 12.03 | 77.74 | 9.76 | 155.50 | 15.84 |
| 77.82 | 10.66 | 77.83 | 14.20 | 155.67 | 16.08 | 77.83 | 9.06 | 79.33 | 14.91 | 77.83 | 8.75 | 155.67 | 12.09 | 77.83 | 9.62 | 155.67 | 15.86 |
| 77.90 | 10.61 | 77.92 | 14.25 | 155.83 | 15.92 | 77.91 | 9.02 | 79.41 | 14.94 | 77.91 | 8.83 | 155.83 | 12.11 | 77.91 | 9.65 | 155.83 | 15.96 |
| 77.99 | 10.62 | 78.00 | 14.26 | 156.00 | 16.10 | 78.00 | 9.03 | 79.50 | 15.03 | 78.00 | 8.86 | 156.00 | 12.14 | 77.99 | 9.69 | 156.00 | 15.83 |
| 78.07 | 10.61 | 78.08 | 14.28 | 156.17 | 15.92 | 78.08 | 9.08 | 79.58 | 14.96 | 78.08 | 8.89 | 156.17 | 12.18 | 78.08 | 9.74 | 156.17 | 15.90 |
| 78.15 | 10.71 | 78.17 | 14.28 | 156.33 | 15.96 | 78.16 | 9.18 | 79.66 | 14.98 | 78.16 | 8.79 | 156.33 | 12.25 | 78.16 | 9.72 | 156.33 | 15.88 |
| 78.24 | 10.68 | 78.25 | 14.29 | 156.50 | 16.11 | 78.25 | 9.19 | 79.75 | 14.92 | 78.25 | 8.76 | 156.50 | 12.25 | 78.24 | 9.77 | 156.50 | 15.97 |
| 78.32 | 10.76 | 78.33 | 14.33 | 156.67 | 15.91 | 78.33 | 9.28 | 79.83 | 14.88 | 78.33 | 8.87 | 156.67 | 12.21 | 78.33 | 9.76 | 156.67 | 15.91 |
| 78.40 | 10.80 | 78.42 | 14.32 | 156.83 | 15.95 | 78.41 | 9.32 | 79.91 | 14.99 | 78.41 | 8.79 | 156.83 | 12.19 | 78.41 | 9.81 | 156.83 | 15.91 |
| 78.49 | 10.88 | 78.50 | 14.31 | 157.00 | 16.11 | 78.50 | 9.18 | 80.00 | 14.89 | 78.50 | 8.81 | 157.00 | 12.25 | 78.49 | 9.84 | 157.00 | 15.83 |
| 78.57 | 10.94 | 78.58 | 14.32 | 157.17 | 15.91 | 78.58 | 9.18 | 80.08 | 14.91 | 78.58 | 8.83 | 157.17 | 12.25 | 78.58 | 9.92 | 157.17 | 15.88 |
| 78.65 | 10.85 | 78.67 | 14.37 | 157.33 | 15.92 | 78.66 | 9.32 | 80.16 | 14.97 | 78.66 | 8.80 | 157.33 | 12.17 | 78.66 | 9.97 | 157.33 | 15.90 |
| 78.74 | 10.85 | 78.75 | 14.40 | 157.50 | 16.08 | 78.75 | 9.37 | 80.25 | 15.01 | 78.75 | 8.92 | 157.50 | 12.30 | 78.74 | 9.96 | 157.50 | 15.81 |
| 78.82 | 11.00 | 78.83 | 14.26 | 157.67 | 15.98 | 78.83 | 9.50 | 80.33 | 15.04 | 78.83 | 8.85 | 157.67 | 12.31 | 78.83 | 9.99 | 157.67 | 15.90 |
| 78.90 | 10.86 | 78.92 | 14.32 | 157.83 | 16.12 | 78.91 | 9.50 | 80.41 | 14.96 | 78.91 | 8.79 | 157.83 | 12.28 | 78.91 | 9.86 | 157.83 | 15.93 |
| 78.99 | 10.87 | 79.00 | 14.34 | 158.00 | 15.94 | 79.00 | 9.52 | 80.50 | 14.99 | 79.00 | 8.83 | 158.00 | 12.31 | 78.99 | 9.88 | 158.00 | 15.88 |
| 79.07 | 10.86 | 79.08 | 14.32 | 158.17 | 15.98 | 79.08 | 9.35 | 80.58 | 15.11 | 79.08 | 8.78 | 158.17 | 12.38 | 79.08 | 9.93 | 158.17 | 15.88 |
| 79.15 | 10.87 | 79.17 | 14.33 | 158.33 | 16.11 | 79.16 | 9.39 | 80.66 | 15.12 | 79.16 | 8.28 | 158.33 | 12.44 | 79.16 | 9.94 | 158.33 | 15.90 |
| 79.24 | 11.00 | 79.25 | 14.32 | 158.50 | 16.02 | 79.25 | 9.45 | 80.75 | 15.09 | 79.25 | 8.72 | 158.50 | 12.43 | 79.24 | 9.92 | 158.50 | 15.89 |
| 79.32 | 10.87 | 79.33 | 14.37 | 158.67 | 16.07 | 79.33 | 9.63 | 80.83 | 15.08 | 79.33 | 8.83 | 158.67 | 12.41 | 79.33 | 9.91 | 158.67 | 15.95 |
| 79.40 | 10.88 | 79.42 | 14.31 | 158.83 | 15.93 | 79.41 | 9.69 | 80.91 | 15.14 | 79.41 | 8.89 | 158.83 | 12.37 | 79.41 | 10.04 | 158.83 | 15.91 |

| | | | | | | | | | | | | | | | | | |
|---|---|---|---|---|---|---|---|---|---|---|---|---|---|---|---|---|---|
| 79.49 | 10.91 | 79.50 | 14.31 | 159.00 | 16.09 | 79.50 | 9.50 | 81.00 | 15.04 | 79.50 | 8.91 | 159.00 | 12.36 | 79.49 | 9.89 | 159.00 | 15.99 |
| 79.57 | 10.91 | 79.58 | 14.39 | 159.17 | 16.15 | 79.58 | 9.75 | 81.08 | 15.09 | 79.58 | 8.89 | 159.17 | 12.39 | 79.58 | 9.91 | 159.17 | 15.89 |
| 79.65 | 10.90 | 79.67 | 14.33 | 159.33 | 16.04 | 79.66 | 9.44 | 81.16 | 15.12 | 79.66 | 8.80 | 159.33 | 12.37 | 79.66 | 9.94 | 159.33 | 15.99 |
| 79.74 | 11.01 | 79.75 | 14.34 | 159.50 | 16.18 | 79.75 | 9.47 | 81.25 | 15.20 | 79.75 | 8.88 | 159.50 | 12.43 | 79.74 | 10.01 | 159.50 | 16.00 |
| 79.82 | 10.85 | 79.83 | 14.32 | 159.67 | 16.14 | 79.83 | 9.55 | 81.33 | 15.20 | 79.83 | 8.89 | 159.67 | 12.41 | 79.83 | 9.92 | 159.67 | 15.99 |
| 79.90 | 10.89 | 79.92 | 14.30 | 159.83 | 16.20 | 79.91 | 9.56 | 81.41 | 15.06 | 79.91 | 8.94 | 159.83 | 12.47 | 79.91 | 9.92 | 159.83 | 16.03 |
| 79.99 | 10.92 | 80.00 | 14.41 | 160.00 | 16.14 | 80.00 | 9.80 | 81.50 | 15.02 | 80.00 | 9.02 | 160.00 | 12.38 | 79.99 | 10.04 | 160.00 | 16.04 |
| 80.07 | 10.99 | 80.08 | 14.30 | 160.17 | 16.14 | 80.08 | 9.53 | 81.58 | 15.06 | 80.08 | 9.00 | 160.17 | 12.45 | 80.08 | 9.89 | 160.17 | 16.04 |
| 80.15 | 11.05 | 80.17 | 14.29 | 160.33 | 16.19 | 80.16 | 9.54 | 81.66 | 15.04 | 80.16 | 9.04 | 160.33 | 12.57 | 80.16 | 9.90 | 160.33 | 16.06 |
| 80.24 | 11.07 | 80.25 | 14.39 | 160.50 | 16.12 | 80.25 | 9.58 | 81.75 | 15.11 | 80.25 | 9.05 | 160.50 | 12.51 | 80.24 | 10.03 | 160.50 | 16.13 |
| 80.32 | 11.09 | 80.33 | 14.35 | 160.67 | 16.21 | 80.33 | 9.64 | 81.83 | 15.16 | 80.33 | 9.12 | 160.67 | 12.54 | 80.33 | 9.95 | 160.67 | 16.16 |
| 80.40 | 11.06 | 80.42 | 14.33 | 160.83 | 16.23 | 80.41 | 9.65 | 81.91 | 15.10 | 80.41 | 9.10 | 160.83 | 12.60 | 80.41 | 9.96 | 160.83 | 16.09 |
| 80.49 | 11.11 | 80.50 | 14.34 | 161.00 | 16.25 | 80.50 | 9.74 | 82.00 | 15.06 | 80.50 | 9.10 | 161.00 | 12.63 | 80.49 | 9.97 | 161.00 | 16.17 |
| 80.57 | 11.15 | 80.58 | 14.37 | 161.17 | 16.26 | 80.58 | 9.76 | 82.08 | 15.19 | 80.58 | 9.14 | 161.17 | 12.65 | 80.58 | 9.96 | 161.17 | 16.20 |
| 80.65 | 11.20 | 80.67 | 14.38 | 161.33 | 16.31 | 80.66 | 9.76 | 82.16 | 15.10 | 80.66 | 9.15 | 161.33 | 12.66 | 80.66 | 10.08 | 161.33 | 16.19 |
| 80.74 | 11.21 | 80.75 | 14.42 | 161.50 | 16.31 | 80.75 | 9.81 | 82.25 | 15.17 | 80.75 | 9.18 | 161.50 | 12.70 | 80.74 | 10.10 | 161.50 | 16.16 |
| 80.82 | 11.22 | 80.83 | 14.49 | 161.67 | 16.26 | 80.83 | 9.95 | 82.33 | 15.19 | 80.83 | 9.21 | 161.67 | 12.63 | 80.83 | 10.14 | 161.67 | 16.15 |
| 80.90 | 11.25 | 80.92 | 14.53 | 161.83 | 16.33 | 80.91 | 9.77 | 82.41 | 15.15 | 80.91 | 9.13 | 161.83 | 12.62 | 80.91 | 10.16 | 161.83 | 16.19 |
| 80.99 | 11.29 | 81.00 | 14.56 | 162.00 | 16.36 | 81.00 | 9.76 | 82.50 | 15.20 | 81.00 | 9.17 | 162.00 | 12.59 | 80.99 | 10.14 | 162.00 | 16.18 |
| 81.07 | 11.39 | 81.08 | 14.48 | 162.17 | 16.40 | 81.08 | 9.92 | 82.58 | 15.15 | 81.08 | 9.17 | 162.17 | 12.60 | 81.08 | 10.13 | 162.17 | 16.25 |
| 81.15 | 11.31 | 81.17 | 14.48 | 162.33 | 16.40 | 81.16 | 9.72 | 82.66 | 15.15 | 81.16 | 9.17 | 162.33 | 12.69 | 81.16 | 10.13 | 162.33 | 16.27 |
| 81.24 | 11.31 | 81.25 | 14.50 | 162.50 | 16.44 | 81.25 | 9.95 | 82.75 | 15.28 | 81.25 | 9.21 | 162.50 | 12.66 | 81.24 | 10.22 | 162.50 | 16.33 |
| 81.32 | 11.41 | 81.33 | 14.54 | 162.67 | 16.50 | 81.33 | 9.70 | 82.83 | 15.27 | 81.33 | 9.24 | 162.67 | 12.65 | 81.33 | 10.27 | 162.67 | 16.37 |
| 81.40 | 11.29 | 81.42 | 14.61 | 162.83 | 16.54 | 81.41 | 9.93 | 82.91 | 15.28 | 81.41 | 9.28 | 162.83 | 12.63 | 81.41 | 10.27 | 162.83 | 16.30 |
| 81.49 | 11.28 | 81.50 | 14.64 | 163.00 | 16.37 | 81.50 | 9.75 | 83.00 | 15.31 | 81.50 | 9.28 | 163.00 | 12.61 | 81.49 | 10.34 | 163.00 | 16.29 |
| 81.57 | 11.45 | 81.58 | 14.60 | 163.17 | 16.42 | 81.58 | 9.74 | 83.08 | 15.26 | 81.58 | 9.31 | 163.17 | 12.63 | 81.58 | 10.34 | 163.17 | 16.28 |
| 81.65 | 11.29 | 81.67 | 14.62 | 163.33 | 16.55 | 81.66 | 9.93 | 83.16 | 15.33 | 81.66 | 9.35 | 163.33 | 12.66 | 81.66 | 10.38 | 163.33 | 16.34 |
| 81.74 | 11.30 | 81.75 | 14.69 | 163.50 | 16.36 | 81.75 | 9.72 | 83.25 | 15.23 | 81.75 | 9.39 | 163.50 | 12.62 | 81.74 | 10.43 | 163.50 | 16.25 |
| 81.82 | 11.32 | 81.83 | 14.62 | 163.67 | 16.39 | 81.83 | 9.91 | 83.33 | 15.32 | 81.83 | 9.26 | 163.67 | 12.65 | 81.83 | 10.31 | 163.67 | 16.28 |
| 81.90 | 11.32 | 81.92 | 14.73 | 163.83 | 16.53 | 81.91 | 9.68 | 83.41 | 15.29 | 81.91 | 9.27 | 163.83 | 12.66 | 81.91 | 10.33 | 163.83 | 16.36 |
| 81.99 | 10.76 | 82.00 | 14.66 | 164.00 | 16.37 | 82.00 | 9.73 | 83.50 | 15.32 | 82.00 | 9.34 | 164.00 | 12.59 | 81.99 | 10.32 | 164.00 | 16.30 |
| 82.07 | 9.45 | 82.08 | 14.66 | 164.17 | 16.55 | 82.08 | 9.90 | 83.58 | 15.27 | 82.08 | 9.36 | 164.17 | 12.71 | 82.08 | 10.33 | 164.17 | 16.29 |
| 82.15 | 6.83 | 82.17 | 14.73 | 164.33 | 16.35 | 82.16 | 9.76 | 83.66 | 15.22 | 82.16 | 9.25 | 164.33 | 12.73 | 82.16 | 10.46 | 164.33 | 16.39 |
| 82.24 | 3.84 | 82.25 | 14.66 | 164.50 | 16.39 | 82.25 | 9.92 | 83.75 | 15.23 | 82.25 | 9.25 | 164.50 | 12.65 | 82.24 | 10.29 | 164.50 | 16.32 |
| 82.32 | 2.49 | 82.33 | 14.65 | 164.67 | 16.56 | 82.33 | 9.71 | 83.83 | 15.35 | 82.33 | 9.38 | 164.67 | 12.69 | 82.33 | 10.48 | 164.67 | 16.36 |
| 82.40 | 2.54 | 82.42 | 14.70 | 164.83 | 16.43 | 82.41 | 9.73 | 83.91 | 15.28 | 82.41 | 9.27 | 164.83 | 12.76 | 82.41 | 10.39 | 164.83 | 16.30 |
| 82.49 | 2.54 | 82.50 | 14.70 | 165.00 | 16.40 | 82.50 | 9.96 | 84.00 | 15.30 | 82.50 | 9.37 | 165.00 | 12.77 | 82.49 | 10.46 | 165.00 | 16.28 |
| 82.57 | 2.55 | 82.58 | 14.71 | 165.17 | 16.54 | 82.58 | 9.73 | 84.08 | 15.31 | 82.58 | 9.29 | 165.17 | 12.70 | 82.58 | 10.47 | 165.17 | 16.30 |
| 82.65 | 2.57 | 82.67 | 14.81 | 165.33 | 16.41 | 82.66 | 9.75 | 84.16 | 15.31 | 82.66 | 9.26 | 165.33 | 12.84 | 82.66 | 10.49 | 165.33 | 16.38 |
| 82.74 | 2.55 | 82.75 | 14.81 | 165.50 | 16.37 | 82.75 | 9.76 | 84.25 | 15.36 | 82.75 | 9.40 | 165.50 | 12.81 | 82.74 | 10.42 | 165.50 | 16.28 |
| 82.82 | 2.61 | 82.83 | 14.84 | 165.67 | 16.55 | 82.83 | 9.94 | 84.33 | 15.36 | 82.83 | 9.30 | 165.67 | 12.82 | 82.83 | 10.49 | 165.67 | 16.36 |
| 82.90 | 2.67 | 82.92 | 14.78 | 165.83 | 16.43 | 82.91 | 9.92 | 84.41 | 15.38 | 82.91 | 9.27 | 165.83 | 12.89 | 82.91 | 10.57 | 165.83 | 16.34 |
| 82.99 | 2.54 | 83.00 | 14.82 | 166.00 | 16.43 | 83.00 | 9.70 | 84.50 | 15.42 | 83.00 | 9.40 | 166.00 | 12.94 | 82.99 | 10.58 | 166.00 | 16.31 |
| 83.07 | 2.53 | 83.08 | 14.83 | 166.17 | 16.55 | 83.08 | 9.73 | 84.58 | 15.45 | 83.08 | 9.29 | 166.17 | 12.94 | 83.08 | 10.50 | 166.17 | 16.36 |
| 83.15 | 2.69 | 83.17 | 14.86 | 166.33 | 16.38 | 83.16 | 9.75 | 84.66 | 15.47 | 83.16 | 9.29 | 166.33 | 12.93 | 83.16 | 10.52 | 166.33 | 16.42 |
| 83.24 | 2.53 | 83.25 | 14.93 | 166.50 | 16.43 | 83.25 | 9.91 | 84.75 | 15.47 | 83.25 | 9.27 | 166.50 | 13.02 | 83.24 | 10.49 | 166.50 | 16.37 |
| 83.32 | 2.68 | 83.33 | 14.82 | 166.67 | 16.38 | 83.33 | 9.82 | 84.83 | 15.49 | 83.33 | 9.29 | 166.67 | 13.04 | 83.33 | 10.48 | 166.67 | 16.44 |
| 83.40 | 2.51 | 83.42 | 14.93 | 166.83 | 16.41 | 83.41 | 9.81 | 84.91 | 15.38 | 83.41 | 9.29 | 166.83 | 13.05 | 83.41 | 10.60 | 166.83 | 16.46 |
| 83.49 | 2.53 | 83.50 | 14.84 | 167.00 | 16.51 | 83.50 | 9.69 | 85.00 | 15.48 | 83.50 | 9.39 | 167.00 | 13.14 | 83.49 | 10.48 | 167.00 | 16.45 |
| 83.57 | 2.69 | 83.58 | 14.85 | 167.17 | 16.45 | 83.58 | 9.70 | 85.08 | 15.47 | 83.58 | 9.27 | 167.17 | 13.12 | 83.58 | 10.52 | 167.17 | 16.46 |
| 83.65 | 2.52 | 83.67 | 14.84 | 167.33 | 16.40 | 83.66 | 9.72 | 85.16 | 15.52 | 83.66 | 9.38 | 167.33 | 13.03 | 83.66 | 10.58 | 167.33 | 16.56 |

| | | | | | | | | | | | | | | | | | |
|---|---|---|---|---|---|---|---|---|---|---|---|---|---|---|---|---|---|
| 83.74 | 2.68 | 83.75 | 14.87 | 167.50 | 16.44 | 83.75 | 9.76 | 85.25 | 15.56 | 83.75 | 9.30 | 167.50 | 13.01 | 83.74 | 10.57 | 167.50 | 16.57 |
| 83.82 | 2.52 | 83.83 | 14.87 | 167.67 | 16.50 | 83.83 | 9.79 | 85.33 | 15.53 | 83.83 | 9.35 | 167.67 | 13.07 | 83.83 | 10.61 | 167.67 | 16.53 |
| 83.90 | 2.68 | 83.92 | 14.89 | 167.83 | 16.39 | 83.91 | 9.83 | 85.41 | 15.58 | 83.91 | 9.34 | 167.83 | 13.03 | 83.91 | 10.64 | 167.83 | 16.60 |
| 83.99 | 2.52 | 84.00 | 14.88 | 168.00 | 16.45 | 84.00 | 9.71 | 85.50 | 15.58 | 84.00 | 9.27 | 168.00 | 13.01 | 83.99 | 10.65 | 168.00 | 16.60 |
| 84.07 | 2.56 | 84.08 | 14.96 | 168.17 | 16.52 | 84.08 | 9.94 | 85.58 | 15.65 | 84.08 | 9.30 | 168.17 | 13.05 | 84.08 | 10.69 | 168.17 | 16.56 |
| 84.15 | 2.55 | 84.17 | 14.99 | 168.33 | 16.52 | 84.16 | 9.74 | 85.66 | 15.66 | 84.16 | 9.32 | 168.33 | 13.07 | 84.16 | 10.65 | 168.33 | 16.58 |
| 84.24 | 2.69 | 84.25 | 15.01 | 168.50 | 16.53 | 84.25 | 9.74 | 85.75 | 15.64 | 84.25 | 9.35 | 168.50 | 13.01 | 84.24 | 10.65 | 168.50 | 16.61 |
| 84.32 | 2.52 | 84.33 | 14.95 | 168.67 | 16.61 | 84.33 | 9.78 | 85.83 | 15.57 | 84.33 | 9.45 | 168.67 | 13.04 | 84.33 | 10.65 | 168.67 | 16.67 |
| 84.40 | 2.69 | 84.42 | 15.04 | 168.83 | 16.63 | 84.41 | 9.80 | 85.91 | 15.65 | 84.41 | 9.47 | 168.83 | 13.06 | 84.41 | 10.72 | 168.83 | 16.66 |
| 84.49 | 2.51 | 84.50 | 15.08 | 169.00 | 16.65 | 84.50 | 9.79 | 86.00 | 15.65 | 84.50 | 9.42 | 169.00 | 13.16 | 84.49 | 10.62 | 169.00 | 16.71 |
| 84.57 | 2.53 | 84.58 | 14.99 | 169.17 | 16.63 | 84.58 | 9.93 | 86.08 | 15.65 | 84.58 | 9.46 | 169.17 | 13.13 | 84.58 | 10.62 | 169.17 | 16.75 |
| 84.65 | 2.69 | 84.67 | 15.06 | 169.33 | 16.72 | 84.66 | 9.73 | 86.16 | 15.70 | 84.66 | 9.40 | 169.33 | 13.11 | 84.66 | 10.68 | 169.33 | 16.75 |
| 84.74 | 2.51 | 84.75 | 15.04 | 169.50 | 16.75 | 84.75 | 9.75 | 86.25 | 15.68 | 84.75 | 9.45 | 169.50 | 13.13 | 84.74 | 10.70 | 169.50 | 16.67 |
| 84.82 | 2.52 | 84.83 | 15.07 | 169.67 | 16.70 | 84.83 | 9.83 | 86.33 | 15.76 | 84.83 | 9.55 | 169.67 | 13.01 | 84.83 | 10.81 | 169.67 | 16.71 |
| 84.90 | 2.70 | 84.92 | 15.17 | 169.83 | 16.80 | 84.91 | 9.83 | 86.41 | 15.83 | 84.91 | 9.56 | 169.83 | 13.08 | 84.91 | 10.77 | 169.83 | 16.72 |
| 84.99 | 2.51 | 85.00 | 15.18 | 170.00 | 16.73 | 85.00 | 10.00 | 86.50 | 15.87 | 85.00 | 9.45 | 170.00 | 13.09 | 84.99 | 10.86 | 170.00 | 16.79 |
| 85.07 | 2.69 | 85.08 | 15.21 | 170.17 | 16.81 | 85.08 | 10.06 | 86.58 | 15.83 | 85.08 | 9.48 | 170.17 | 13.07 | 85.08 | 10.73 | 170.17 | 16.69 |
| 85.15 | 2.51 | 85.17 | 15.19 | 170.33 | 16.75 | 85.16 | 10.06 | 86.66 | 15.75 | 85.16 | 9.54 | 170.33 | 13.13 | 85.16 | 10.75 | 170.33 | 16.73 |
| 85.24 | 2.52 | 85.25 | 15.19 | 170.50 | 16.73 | 85.25 | 9.91 | 86.75 | 15.75 | 85.25 | 9.47 | 170.50 | 13.20 | 85.24 | 10.88 | 170.50 | 16.63 |
| 85.32 | 2.66 | 85.33 | 15.26 | 170.67 | 16.82 | 85.33 | 9.92 | 86.83 | 15.85 | 85.33 | 9.47 | 170.67 | 13.14 | 85.33 | 10.80 | 170.67 | 16.68 |
| 85.40 | 2.51 | 85.42 | 15.25 | 170.83 | 16.76 | 85.41 | 9.91 | 86.91 | 15.75 | 85.41 | 9.52 | 170.83 | 13.24 | 85.41 | 10.80 | 170.83 | 16.75 |
| 85.49 | 2.68 | 85.50 | 15.24 | 171.00 | 16.80 | 85.50 | 9.98 | 87.00 | 15.77 | 85.50 | 9.59 | 171.00 | 13.22 | 85.49 | 10.82 | 171.00 | 16.68 |
| 85.57 | 2.49 | 85.58 | 15.26 | 171.17 | 16.81 | 85.58 | 9.95 | 87.08 | 15.82 | 85.58 | 9.59 | 171.17 | 13.26 | 85.58 | 10.86 | 171.17 | 16.69 |
| 85.65 | 2.69 | 85.67 | 15.26 | 171.33 | 16.85 | 85.66 | 10.13 | 87.16 | 15.76 | 85.66 | 9.55 | 171.33 | 13.17 | 85.66 | 10.87 | 171.33 | 16.72 |
| 85.74 | 2.51 | 85.75 | 15.39 | 171.50 | 16.87 | 85.75 | 9.94 | 87.25 | 15.79 | 85.75 | 9.60 | 171.50 | 13.23 | 85.74 | 10.93 | 171.50 | 16.78 |
| 85.82 | 2.53 | 85.83 | 15.42 | 171.67 | 16.96 | 85.83 | 10.01 | 87.33 | 15.74 | 85.83 | 9.61 | 171.67 | 13.21 | 85.83 | 10.98 | 171.67 | 16.69 |
| 85.90 | 2.69 | 85.92 | 15.37 | 171.83 | 16.97 | 85.91 | 9.96 | 87.41 | 15.78 | 85.91 | 9.66 | 171.83 | 13.18 | 85.91 | 11.01 | 171.83 | 16.72 |
| 85.99 | 2.52 | 86.00 | 15.43 | 172.00 | 16.82 | 86.00 | 9.94 | 87.50 | 15.78 | 86.00 | 9.70 | 172.00 | 13.22 | 85.99 | 10.91 | 172.00 | 16.70 |
| 86.07 | 2.68 | 86.08 | 15.41 | 172.17 | 16.86 | 86.08 | 10.05 | 87.58 | 15.80 | 86.08 | 9.70 | 172.17 | 13.26 | 86.08 | 11.00 | 172.17 | 16.76 |
| 86.15 | 2.50 | 86.17 | 15.37 | 172.33 | 17.02 | 86.16 | 10.16 | 87.66 | 15.80 | 86.16 | 9.64 | 172.33 | 13.18 | 86.16 | 10.89 | 172.33 | 16.72 |
| 86.24 | 2.52 | 86.25 | 15.37 | 172.50 | 16.97 | 86.25 | 10.11 | 87.75 | 15.80 | 86.25 | 9.61 | 172.50 | 13.25 | 86.24 | 11.03 | 172.50 | 16.73 |
| 86.32 | 2.69 | 86.33 | 15.38 | 172.67 | 16.86 | 86.33 | 10.25 | 87.83 | 15.77 | 86.33 | 9.64 | 172.67 | 13.29 | 86.33 | 10.91 | 172.67 | 16.71 |
| 86.40 | 2.51 | 86.42 | 15.41 | 172.83 | 16.80 | 86.41 | 10.19 | 87.91 | 15.77 | 86.41 | 9.65 | 172.83 | 13.35 | 86.41 | 10.90 | 172.83 | 16.72 |
| 86.49 | 2.53 | 86.50 | 15.39 | 173.00 | 17.00 | 86.50 | 10.03 | 88.00 | 15.84 | 86.50 | 9.66 | 173.00 | 13.35 | 86.49 | 10.96 | 173.00 | 16.71 |
| 86.57 | 2.70 | 86.58 | 15.39 | 173.17 | 16.90 | 86.58 | 10.07 | 88.08 | 15.75 | 86.58 | 9.73 | 173.17 | 13.47 | 86.58 | 10.94 | 173.17 | 16.75 |
| 86.65 | 2.52 | 86.67 | 15.39 | 173.33 | 16.82 | 86.66 | 10.08 | 88.16 | 15.81 | 86.66 | 9.73 | 173.33 | 13.50 | 86.66 | 10.91 | 173.33 | 16.75 |
| 86.74 | 2.68 | 86.75 | 15.45 | 173.50 | 17.02 | 86.75 | 10.11 | 88.25 | 15.77 | 86.75 | 9.73 | 173.50 | 13.46 | 86.74 | 11.02 | 173.50 | 16.81 |
| 86.82 | 2.52 | 86.83 | 15.47 | 173.67 | 16.85 | 86.83 | 10.14 | 88.33 | 15.76 | 86.83 | 9.71 | 173.67 | 13.43 | 86.83 | 10.90 | 173.67 | 16.81 |
| | | 86.92 | 15.46 | 173.83 | 16.85 | 86.91 | 10.28 | 88.41 | 15.86 | 86.91 | 9.78 | 173.83 | 13.48 | 86.91 | 10.94 | 173.83 | 16.82 |
| | | 87.00 | 15.50 | 174.00 | 16.85 | 87.00 | 10.32 | 88.50 | 15.74 | 87.00 | 9.83 | 174.00 | 13.54 | 86.99 | 10.97 | 174.00 | 16.87 |
| | | 87.08 | 15.51 | 174.17 | 16.85 | 87.08 | 10.09 | 88.58 | 15.80 | 87.08 | 9.88 | 174.17 | 13.43 | 87.08 | 11.03 | 174.17 | 16.87 |
| | | 87.17 | 15.56 | 174.33 | 16.86 | 87.16 | 10.18 | 88.66 | 15.80 | 87.16 | 9.76 | 174.33 | 13.53 | 87.16 | 11.03 | 174.33 | 16.88 |
| | | 87.25 | 15.58 | 174.50 | 16.97 | 87.25 | 10.26 | 88.75 | 15.82 | 87.25 | 9.75 | 174.50 | 13.57 | 87.24 | 11.00 | 174.50 | 16.94 |
| | | 87.33 | 15.58 | 174.67 | 16.82 | 87.33 | 10.25 | 88.83 | 15.84 | 87.33 | 9.87 | 174.67 | 13.57 | 87.33 | 11.03 | 174.67 | 16.98 |
| | | 87.42 | 15.59 | 174.83 | 16.86 | 87.41 | 10.16 | 88.91 | 15.83 | 87.41 | 9.73 | 174.83 | 13.61 | 87.41 | 11.12 | 174.83 | 17.01 |
| | | 87.50 | 15.67 | 175.00 | 16.89 | 87.50 | 10.10 | 89.00 | 15.85 | 87.50 | 9.90 | 175.00 | 13.61 | 87.49 | 11.09 | 175.00 | 17.02 |
| | | 87.58 | 15.74 | 175.17 | 16.98 | 87.58 | 10.02 | 89.08 | 15.89 | 87.58 | 9.78 | 175.17 | 13.62 | 87.58 | 11.20 | 175.17 | 17.01 |
| | | 87.67 | 15.75 | 175.33 | 16.88 | 87.66 | 10.06 | 89.16 | 15.93 | 87.66 | 9.77 | 175.33 | 13.63 | 87.66 | 11.24 | 175.33 | 17.12 |
| | | 87.75 | 15.74 | 175.50 | 16.90 | 87.75 | 10.17 | 89.25 | 15.91 | 87.75 | 9.77 | 175.50 | 13.72 | 87.74 | 11.18 | 175.50 | 17.05 |
| | | 87.83 | 15.70 | 175.67 | 16.97 | 87.83 | 10.19 | 89.33 | 15.92 | 87.83 | 9.76 | 175.67 | 13.73 | 87.83 | 11.28 | 175.67 | 17.06 |
| | | 87.92 | 15.75 | 175.83 | 16.92 | 87.91 | 10.25 | 89.41 | 15.88 | 87.91 | 9.89 | 175.83 | 13.75 | 87.91 | 11.28 | 175.83 | 17.20 |

| | | | | | | | | | | | | | | | | | |
|---|---|---|---|---|---|---|---|---|---|---|---|---|---|---|---|---|---|
| | | 88.00 | 15.73 | 176.00 | 17.02 | 88.00 | 10.12 | 89.50 | 15.93 | 88.00 | 9.77 | 176.00 | 13.77 | 87.99 | 11.12 | 176.00 | 17.12 |
| | | 88.08 | 15.69 | 176.17 | 16.97 | 88.08 | 10.03 | 89.58 | 15.97 | 88.08 | 9.78 | 176.17 | 13.83 | 88.08 | 11.19 | 176.17 | 17.19 |
| | | 88.17 | 15.71 | 176.33 | 17.05 | 88.16 | 10.08 | 89.66 | 16.02 | 88.16 | 9.76 | 176.33 | 13.84 | 88.16 | 11.20 | 176.33 | 17.11 |
| | | 88.25 | 15.71 | 176.50 | 17.12 | 88.25 | 10.15 | 89.75 | 15.90 | 88.25 | 9.77 | 176.50 | 13.91 | 88.24 | 11.20 | 176.50 | 17.17 |
| | | 88.33 | 15.68 | 176.67 | 17.04 | 88.33 | 10.14 | 89.83 | 15.97 | 88.33 | 9.91 | 176.67 | 13.87 | 88.33 | 11.32 | 176.67 | 17.09 |
| | | 88.42 | 15.80 | 176.83 | 17.09 | 88.41 | 10.24 | 89.91 | 15.90 | 88.41 | 9.74 | 176.83 | 13.85 | 88.41 | 11.16 | 176.83 | 17.06 |
| | | 88.50 | 15.70 | 177.00 | 16.99 | 88.50 | 10.10 | 90.00 | 16.03 | 88.50 | 9.85 | 177.00 | 13.93 | 88.49 | 11.15 | 177.00 | 17.14 |
| | | 88.58 | 15.71 | 177.17 | 17.02 | 88.58 | 10.09 | 90.08 | 15.88 | 88.58 | 9.78 | 177.17 | 13.92 | 88.58 | 11.18 | 177.17 | 17.07 |
| | | 88.67 | 15.73 | 177.33 | 17.08 | 88.66 | 10.31 | 90.16 | 16.02 | 88.66 | 9.80 | 177.33 | 13.96 | 88.66 | 11.30 | 177.33 | 17.09 |
| | | 88.75 | 15.70 | 177.50 | 17.03 | 88.75 | 10.08 | 90.25 | 15.99 | 88.75 | 9.79 | 177.50 | 13.95 | 88.74 | 11.19 | 177.50 | 17.16 |
| | | 88.83 | 15.71 | 177.67 | 17.13 | 88.83 | 10.13 | 90.33 | 16.03 | 88.83 | 9.78 | 177.67 | 13.96 | 88.83 | 11.17 | 177.67 | 17.11 |
| | | 88.92 | 15.81 | 177.83 | 17.12 | 88.91 | 10.15 | 90.41 | 15.98 | 88.91 | 9.80 | 177.83 | 13.98 | 88.91 | 11.20 | 177.83 | 17.07 |
| | | 89.00 | 15.69 | 178.00 | 17.05 | 89.00 | 10.25 | 90.50 | 15.94 | 89.00 | 9.88 | 178.00 | 13.93 | 88.99 | 11.32 | 178.00 | 17.13 |
| | | 89.08 | 15.69 | 178.17 | 17.20 | 89.08 | 10.09 | 90.58 | 15.91 | 89.08 | 9.84 | 178.17 | 13.97 | 89.08 | 11.20 | 178.17 | 17.12 |
| | | 89.17 | 15.71 | 178.33 | 17.21 | 89.16 | 10.12 | 90.66 | 15.91 | 89.16 | 9.76 | 178.33 | 14.02 | 89.16 | 11.20 | 178.33 | 17.20 |
| | | 89.25 | 15.76 | 178.50 | 17.16 | 89.25 | 10.27 | 90.75 | 16.01 | 89.25 | 9.75 | 178.50 | 14.02 | 89.24 | 11.18 | 178.50 | 17.15 |
| | | 89.33 | 15.77 | 178.67 | 17.18 | 89.33 | 10.11 | 90.83 | 16.08 | 89.33 | 9.86 | 178.67 | 14.06 | 89.33 | 11.20 | 178.67 | 17.14 |
| | | 89.42 | 15.78 | 178.83 | 17.25 | 89.41 | 10.15 | 90.91 | 16.07 | 89.41 | 9.92 | 178.83 | 14.05 | 89.41 | 11.29 | 178.83 | 17.12 |
| | | 89.50 | 15.82 | 179.00 | 17.19 | 89.50 | 10.13 | 91.00 | 15.96 | 89.50 | 9.87 | 179.00 | 14.06 | 89.49 | 11.20 | 179.00 | 17.13 |
| | | 89.58 | 15.77 | 179.17 | 17.16 | 89.58 | 10.13 | 91.08 | 15.97 | 89.58 | 9.88 | 179.17 | 14.00 | 89.58 | 11.22 | 179.17 | 17.20 |
| | | 89.67 | 15.81 | 179.33 | 17.26 | 89.66 | 10.28 | 91.16 | 16.02 | 89.66 | 9.92 | 179.33 | 14.05 | 89.66 | 11.21 | 179.33 | 17.15 |
| | | 89.75 | 15.87 | 179.50 | 17.23 | 89.75 | 10.09 | 91.25 | 16.14 | 89.75 | 9.98 | 179.50 | 14.11 | 89.74 | 11.24 | 179.50 | 17.19 |
| | | 89.83 | 15.91 | 179.67 | 17.21 | 89.83 | 10.27 | 91.33 | 16.15 | 89.83 | 10.02 | 179.67 | 14.12 | 89.83 | 11.30 | 179.67 | 17.19 |
| | | 89.92 | 15.96 | 179.83 | 17.20 | 89.91 | 10.16 | 91.41 | 16.12 | 89.91 | 10.01 | 179.83 | 14.12 | 89.91 | 11.27 | 179.83 | 17.24 |
| | | 90.00 | 15.88 | 180.00 | 17.36 | 90.00 | 10.10 | 91.50 | 16.08 | 90.00 | 9.98 | 180.00 | 14.15 | 89.99 | 11.33 | 180.00 | 17.27 |
| | | 90.08 | 15.90 | 180.17 | 17.42 | 90.08 | 10.12 | 91.58 | 16.18 | 90.08 | 10.01 | 180.17 | 14.16 | 90.08 | 11.36 | 180.17 | 17.24 |
| | | 90.17 | 15.91 | 180.33 | 17.25 | 90.16 | 10.24 | 91.66 | 16.06 | 90.16 | 10.04 | 180.33 | 14.18 | 90.16 | 11.37 | 180.33 | 17.25 |
| | | 90.25 | 15.91 | 180.50 | 17.31 | 90.25 | 10.11 | 91.75 | 16.19 | 90.25 | 10.09 | 180.50 | 14.28 | 90.24 | 11.35 | 180.50 | 17.29 |
| | | 90.33 | 15.94 | 180.67 | 17.44 | 90.33 | 10.16 | 91.83 | 16.23 | 90.33 | 10.13 | 180.67 | 14.30 | 90.33 | 11.35 | 180.67 | 17.28 |
| | | 90.42 | 15.92 | 180.83 | 17.26 | 90.41 | 10.27 | 91.91 | 16.21 | 90.41 | 10.12 | 180.83 | 14.27 | 90.41 | 11.37 | 180.83 | 17.30 |
| | | 90.50 | 15.91 | 181.00 | 17.43 | 90.50 | 10.13 | 92.00 | 16.25 | 90.50 | 10.19 | 181.00 | 14.25 | 90.49 | 11.42 | 181.00 | 17.38 |
| | | 90.58 | 15.87 | 181.17 | 17.26 | 90.58 | 10.12 | 92.08 | 16.28 | 90.58 | 10.11 | 181.17 | 14.26 | 90.58 | 11.45 | 181.17 | 17.33 |
| | | 90.67 | 15.92 | 181.33 | 17.28 | 90.66 | 10.15 | 92.16 | 16.19 | 90.66 | 10.20 | 181.33 | 14.31 | 90.66 | 11.50 | 181.33 | 17.39 |
| | | 90.75 | 15.92 | 181.50 | 17.46 | 90.75 | 10.12 | 92.25 | 16.29 | 90.75 | 10.13 | 181.50 | 14.23 | 90.74 | 11.50 | 181.50 | 17.43 |
| | | 90.83 | 16.00 | 181.67 | 17.26 | 90.83 | 10.18 | 92.33 | 16.31 | 90.83 | 10.11 | 181.67 | 14.29 | 90.83 | 11.54 | 181.67 | 17.44 |
| | | 90.92 | 15.97 | 181.83 | 17.30 | 90.91 | 10.20 | 92.41 | 16.35 | 90.91 | 10.12 | 181.83 | 14.27 | 90.91 | 11.51 | 181.83 | 17.40 |
| | | 91.00 | 16.01 | 182.00 | 17.44 | 91.00 | 10.10 | 92.50 | 16.27 | 91.00 | 10.11 | 182.00 | 14.36 | 90.99 | 11.59 | 182.00 | 17.46 |
| | | 91.08 | 16.01 | 182.17 | 17.27 | 91.08 | 10.14 | 92.58 | 16.37 | 91.08 | 10.20 | 182.17 | 14.30 | 91.08 | 11.61 | 182.17 | 17.47 |
| | | 91.17 | 16.01 | 182.33 | 17.26 | 91.16 | 10.22 | 92.66 | 16.35 | 91.16 | 10.13 | 182.33 | 14.33 | 91.16 | 11.68 | 182.33 | 17.42 |
| | | 91.25 | 16.08 | 182.50 | 17.44 | 91.25 | 10.32 | 92.75 | 16.29 | 91.25 | 10.18 | 182.50 | 14.33 | 91.24 | 11.70 | 182.50 | 17.56 |
| | | 91.33 | 16.11 | 182.67 | 17.29 | 91.33 | 10.28 | 92.83 | 16.27 | 91.33 | 10.14 | 182.67 | 14.35 | 91.33 | 11.61 | 182.67 | 17.51 |
| | | 91.42 | 16.14 | 182.83 | 17.30 | 91.41 | 10.35 | 92.91 | 16.27 | 91.41 | 10.21 | 182.83 | 14.41 | 91.41 | 11.61 | 182.83 | 17.61 |
| | | 91.50 | 16.08 | 183.00 | 17.44 | 91.50 | 10.36 | 93.00 | 16.33 | 91.50 | 10.25 | 183.00 | 14.42 | 91.49 | 11.72 | 183.00 | 17.48 |
| | | 91.58 | 16.08 | 183.17 | 17.25 | 91.58 | 10.46 | 93.08 | 16.31 | 91.58 | 10.29 | 183.17 | 14.44 | 91.58 | 11.70 | 183.17 | 17.56 |
| | | 91.67 | 16.13 | 183.33 | 17.32 | 91.66 | 10.29 | 93.16 | 16.33 | 91.66 | 10.29 | 183.33 | 14.45 | 91.66 | 11.74 | 183.33 | 17.55 |
| | | 91.75 | 16.05 | 183.50 | 17.28 | 91.75 | 10.45 | 93.25 | 16.32 | 91.75 | 10.28 | 183.50 | 14.41 | 91.74 | 11.68 | 183.50 | 17.48 |
| | | 91.83 | 16.16 | 183.67 | 17.34 | 91.83 | 10.54 | 93.33 | 16.30 | 91.83 | 10.29 | 183.67 | 14.51 | 91.83 | 11.67 | 183.67 | 17.54 |
| | | 91.92 | 16.07 | 183.83 | 17.44 | 91.91 | 10.56 | 93.41 | 16.39 | 91.91 | 10.37 | 183.83 | 14.39 | 91.91 | 11.68 | 183.83 | 17.53 |
| | | 92.00 | 16.06 | 184.00 | 17.29 | 92.00 | 10.56 | 93.50 | 16.41 | 92.00 | 10.25 | 184.00 | 14.45 | 91.99 | 11.78 | 184.00 | 17.61 |
| | | 92.08 | 16.07 | 184.17 | 17.30 | 92.08 | 10.45 | 93.58 | 16.42 | 92.08 | 10.36 | 184.17 | 14.46 | 92.08 | 11.82 | 184.17 | 17.56 |
| | | 92.17 | 16.06 | 184.33 | 17.31 | 92.16 | 10.44 | 93.66 | 16.45 | 92.16 | 10.24 | 184.33 | 14.47 | 92.16 | 11.85 | 184.33 | 17.53 |

| | | | | | | | | | | | | | | | | | |
|---|---|---|---|---|---|---|---|---|---|---|---|---|---|---|---|---|---|
| | | 92.25 | 16.07 | 184.50 | 17.37 | 92.25 | 10.54 | 93.75 | 16.44 | 92.25 | 10.24 | 184.50 | 14.52 | 92.24 | 11.76 | 184.50 | 17.49 |
| | | 92.33 | 16.13 | 184.67 | 17.34 | 92.33 | 10.73 | 93.83 | 16.45 | 92.33 | 10.36 | 184.67 | 14.55 | 92.33 | 11.87 | 184.67 | 17.61 |
| | | 92.42 | 16.06 | 184.83 | 17.29 | 92.41 | 10.77 | 93.91 | 16.42 | 92.41 | 10.24 | 184.83 | 14.56 | 92.41 | 11.74 | 184.83 | 17.54 |
| | | 92.50 | 16.07 | 185.00 | 17.36 | 92.50 | 10.57 | 94.00 | 16.44 | 92.50 | 10.26 | 185.00 | 14.59 | 92.49 | 11.86 | 185.00 | 17.51 |
| | | 92.58 | 16.12 | 185.17 | 17.39 | 92.58 | 10.84 | 94.08 | 16.43 | 92.58 | 10.38 | 185.17 | 14.56 | 92.58 | 11.76 | 185.17 | 17.53 |
| | | 92.67 | 16.18 | 185.33 | 17.32 | 92.66 | 10.80 | 94.16 | 16.47 | 92.66 | 10.26 | 185.33 | 14.55 | 92.66 | 11.76 | 185.33 | 17.62 |
| | | 92.75 | 16.22 | 185.50 | 17.40 | 92.75 | 10.49 | 94.25 | 16.51 | 92.75 | 10.24 | 185.50 | 14.58 | 92.74 | 11.87 | 185.50 | 17.57 |
| | | 92.83 | 16.17 | 185.67 | 17.45 | 92.83 | 10.60 | 94.33 | 16.54 | 92.83 | 10.34 | 185.67 | 14.61 | 92.83 | 11.78 | 185.67 | 17.55 |
| | | 92.92 | 16.17 | 185.83 | 17.39 | 92.91 | 10.65 | 94.41 | 16.45 | 92.91 | 10.26 | 185.83 | 14.60 | 92.91 | 11.87 | 185.83 | 17.51 |
| | | 93.00 | 16.19 | 186.00 | 17.52 | 93.00 | 10.61 | 94.50 | 16.50 | 93.00 | 10.24 | 186.00 | 14.59 | 92.99 | 11.74 | 186.00 | 17.56 |
| | | 93.08 | 16.27 | 186.17 | 17.52 | 93.08 | 10.88 | 94.58 | 16.44 | 93.08 | 10.37 | 186.17 | 14.67 | 93.08 | 11.78 | 186.17 | 17.57 |
| | | 93.17 | 16.24 | 186.33 | 17.51 | 93.16 | 10.57 | 94.66 | 16.51 | 93.16 | 10.28 | 186.33 | 14.70 | 93.16 | 11.82 | 186.33 | 17.51 |
| | | 93.25 | 16.27 | 186.50 | 17.48 | 93.25 | 10.61 | 94.75 | 16.42 | 93.25 | 10.28 | 186.50 | 14.64 | 93.24 | 11.82 | 186.50 | 17.55 |
| | | 93.33 | 16.25 | 186.67 | 17.50 | 93.33 | 10.63 | 94.83 | 16.51 | 93.33 | 10.36 | 186.67 | 14.74 | 93.33 | 11.91 | 186.67 | 17.56 |
| | | 93.42 | 16.28 | 186.83 | 17.57 | 93.41 | 10.64 | 94.91 | 16.40 | 93.41 | 10.25 | 186.83 | 14.74 | 93.41 | 11.92 | 186.83 | 17.57 |
| | | 93.50 | 16.20 | 187.00 | 17.51 | 93.50 | 10.67 | 95.00 | 16.49 | 93.50 | 10.23 | 187.00 | 14.66 | 93.49 | 11.81 | 187.00 | 17.55 |
| | | 93.58 | 16.21 | 187.17 | 17.54 | 93.58 | 10.87 | 95.08 | 16.44 | 93.58 | 10.39 | 187.17 | 14.65 | 93.58 | 11.94 | 187.17 | 17.64 |
| | | 93.67 | 16.21 | 187.33 | 17.67 | 93.66 | 10.65 | 95.16 | 16.49 | 93.66 | 10.25 | 187.33 | 14.68 | 93.66 | 11.91 | 187.33 | 17.65 |
| | | 93.75 | 16.26 | 187.50 | 17.70 | 93.75 | 10.90 | 95.25 | 16.41 | 93.75 | 10.29 | 187.50 | 14.71 | 93.74 | 11.99 | 187.50 | 17.65 |
| | | 93.83 | 16.27 | 187.67 | 17.59 | 93.83 | 10.70 | 95.33 | 16.39 | 93.83 | 10.33 | 187.67 | 14.68 | 93.83 | 11.97 | 187.67 | 17.68 |
| | | 93.92 | 16.28 | 187.83 | 17.66 | 93.91 | 10.72 | 95.41 | 16.54 | 93.91 | 10.32 | 187.83 | 14.72 | 93.91 | 11.93 | 187.83 | 17.69 |
| | | 94.00 | 16.26 | 188.00 | 17.65 | 94.00 | 10.77 | 95.50 | 16.42 | 94.00 | 10.32 | 188.00 | 14.74 | 93.99 | 11.93 | 188.00 | 17.68 |
| | | 94.08 | 16.20 | 188.17 | 17.67 | 94.08 | 10.86 | 95.58 | 16.47 | 94.08 | 10.34 | 188.17 | 14.67 | 94.08 | 11.94 | 188.17 | 17.73 |
| | | 94.17 | 16.20 | 188.33 | 17.80 | 94.16 | 10.92 | 95.66 | 16.46 | 94.16 | 10.40 | 188.33 | 14.70 | 94.16 | 12.02 | 188.33 | 17.73 |
| | | 94.25 | 16.22 | 188.50 | 17.85 | 94.25 | 11.00 | 95.75 | 16.45 | 94.25 | 10.46 | 188.50 | 14.69 | 94.24 | 12.09 | 188.50 | 17.67 |
| | | 94.33 | 16.27 | 188.67 | 17.72 | 94.33 | 10.81 | 95.83 | 16.52 | 94.33 | 10.49 | 188.67 | 14.68 | 94.33 | 12.05 | 188.67 | 17.73 |
| | | 94.42 | 16.28 | 188.83 | 17.87 | 94.41 | 11.07 | 95.91 | 16.50 | 94.41 | 10.45 | 188.83 | 14.69 | 94.41 | 12.13 | 188.83 | 17.79 |
| | | 94.50 | 16.34 | 189.00 | 17.76 | 94.50 | 11.00 | 96.00 | 16.50 | 94.50 | 10.50 | 189.00 | 14.71 | 94.49 | 12.13 | 189.00 | 17.78 |
| | | 94.58 | 16.35 | 189.17 | 17.71 | 94.58 | 10.81 | 96.08 | 16.48 | 94.58 | 10.41 | 189.17 | 14.71 | 94.58 | 12.00 | 189.17 | 17.79 |
| | | 94.67 | 16.31 | 189.33 | 17.88 | 94.66 | 10.84 | 96.16 | 16.54 | 94.66 | 10.50 | 189.33 | 14.65 | 94.66 | 12.14 | 189.33 | 17.83 |
| | | 94.75 | 16.38 | 189.50 | 17.73 | 94.75 | 11.02 | 96.25 | 16.59 | 94.75 | 10.55 | 189.50 | 14.67 | 94.74 | 11.97 | 189.50 | 17.85 |
| | | 94.83 | 16.42 | 189.67 | 17.72 | 94.83 | 10.78 | 96.33 | 16.58 | 94.83 | 10.54 | 189.67 | 14.69 | 94.83 | 12.02 | 189.67 | 17.85 |
| | | 94.92 | 16.45 | 189.83 | 17.86 | 94.91 | 11.10 | 96.41 | 16.52 | 94.91 | 10.59 | 189.83 | 14.77 | 94.91 | 12.05 | 189.83 | 17.88 |
| | | 95.00 | 16.50 | 190.00 | 17.71 | 95.00 | 10.87 | 96.50 | 16.62 | 95.00 | 10.63 | 190.00 | 14.66 | 94.99 | 12.05 | 190.00 | 17.92 |
| | | 95.08 | 16.38 | 190.17 | 17.88 | 95.08 | 10.87 | 96.58 | 16.67 | 95.08 | 10.59 | 190.17 | 14.69 | 95.08 | 12.04 | 190.17 | 17.93 |
| | | 95.17 | 16.43 | 190.33 | 17.69 | 95.16 | 10.87 | 96.66 | 16.62 | 95.16 | 10.64 | 190.33 | 14.68 | 95.16 | 12.13 | 190.33 | 17.98 |
| | | 95.25 | 16.49 | 190.50 | 17.72 | 95.25 | 11.01 | 96.75 | 16.67 | 95.25 | 10.58 | 190.50 | 14.79 | 95.24 | 12.01 | 190.50 | 17.92 |
| | | 95.33 | 16.41 | 190.67 | 17.90 | 95.33 | 10.83 | 96.83 | 16.59 | 95.33 | 10.67 | 190.67 | 14.72 | 95.33 | 12.03 | 190.67 | 18.02 |
| | | 95.42 | 16.42 | 190.83 | 17.69 | 95.41 | 11.01 | 96.91 | 16.62 | 95.41 | 10.63 | 190.83 | 14.75 | 95.41 | 12.03 | 190.83 | 17.99 |
| | | 95.50 | 16.52 | 191.00 | 17.76 | 95.50 | 10.79 | 97.00 | 16.68 | 95.50 | 10.66 | 191.00 | 14.79 | 95.49 | 12.13 | 191.00 | 17.90 |
| | | 95.58 | 16.52 | 191.17 | 17.75 | 95.58 | 10.84 | 97.08 | 16.70 | 95.58 | 10.70 | 191.17 | 14.84 | 95.58 | 12.08 | 191.17 | 17.92 |
| | | 95.67 | 16.45 | 191.33 | 17.91 | 95.66 | 10.98 | 97.16 | 16.60 | 95.66 | 10.77 | 191.33 | 14.84 | 95.66 | 12.05 | 191.33 | 17.94 |
| | | 95.75 | 16.39 | 191.50 | 17.71 | 95.75 | 10.84 | 97.25 | 16.59 | 95.75 | 10.72 | 191.50 | 14.86 | 95.74 | 12.01 | 191.50 | 18.02 |
| | | 95.83 | 16.38 | 191.67 | 17.89 | 95.83 | 11.02 | 97.33 | 16.70 | 95.83 | 10.73 | 191.67 | 14.91 | 95.83 | 12.03 | 191.67 | 17.95 |
| | | 95.92 | 16.43 | 191.83 | 17.79 | 95.91 | 10.80 | 97.41 | 16.60 | 95.91 | 10.79 | 191.83 | 14.87 | 95.91 | 12.04 | 191.83 | 17.92 |
| | | 96.00 | 16.48 | 192.00 | 17.75 | 96.00 | 11.04 | 97.50 | 16.68 | 96.00 | 10.78 | 192.00 | 14.97 | 95.99 | 12.05 | 192.00 | 18.05 |
| | | 96.08 | 16.48 | 192.17 | 17.72 | 96.08 | 10.78 | 97.58 | 16.68 | 96.08 | 10.84 | 192.17 | 14.99 | 96.08 | 12.07 | 192.17 | 17.95 |
| | | 96.17 | 16.47 | 192.33 | 17.72 | 96.16 | 10.82 | 97.66 | 16.71 | 96.16 | 10.70 | 192.33 | 15.01 | 96.16 | 12.13 | 192.33 | 17.93 |
| | | 96.25 | 16.46 | 192.50 | 17.75 | 96.25 | 10.67 | 97.75 | 16.71 | 96.25 | 10.87 | 192.50 | 15.11 | 96.24 | 12.03 | 192.50 | 18.03 |
| | | 96.33 | 16.52 | 192.67 | 17.92 | 96.33 | 9.20 | 97.83 | 16.78 | 96.33 | 10.73 | 192.67 | 15.08 | 96.33 | 12.04 | 192.67 | 17.97 |
| | | 96.42 | 16.59 | 192.83 | 17.71 | 96.41 | 10.67 | 97.91 | 16.72 | 96.41 | 10.86 | 192.83 | 15.18 | 96.41 | 12.16 | 192.83 | 18.01 |

| | | | | | | | | | | | | | | | | | |
|---|---|---|---|---|---|---|---|---|---|---|---|---|---|---|---|---|---|
| | | 96.50 | 16.54 | 193.00 | 17.74 | 96.50 | 10.36 | 98.00 | 16.77 | 96.50 | 10.70 | 193.00 | 15.17 | 96.49 | 12.05 | 193.00 | 17.95 |
| | | 96.58 | 16.58 | 193.17 | 17.90 | 96.58 | 10.45 | 98.08 | 16.84 | 96.58 | 10.71 | 193.17 | 15.17 | 96.58 | 12.14 | 193.17 | 17.92 |
| | | 96.67 | 16.57 | 193.33 | 17.77 | 96.66 | 10.66 | 98.16 | 16.89 | 96.66 | 10.87 | 193.33 | 15.01 | 96.66 | 12.19 | 193.33 | 17.95 |
| | | 96.75 | 16.58 | 193.50 | 17.72 | 96.75 | 10.41 | 98.25 | 16.89 | 96.75 | 10.70 | 193.50 | 15.17 | 96.74 | 12.20 | 193.50 | 17.92 |
| | | 96.83 | 16.63 | 193.67 | 17.90 | 96.83 | 10.42 | 98.33 | 16.79 | 96.83 | 10.75 | 193.67 | 15.18 | 96.83 | 12.22 | 193.67 | 18.00 |
| | | 96.92 | 16.61 | 193.83 | 17.73 | 96.91 | 10.44 | 98.41 | 16.90 | 96.91 | 10.85 | 193.83 | 15.11 | 96.91 | 12.25 | 193.83 | 17.95 |
| | | 97.00 | 16.62 | 194.00 | 17.82 | 97.00 | 10.77 | 98.50 | 16.74 | 97.00 | 10.74 | 194.00 | 15.05 | 96.99 | 12.22 | 194.00 | 17.95 |
| | | 97.08 | 16.66 | 194.17 | 17.83 | 97.08 | 10.50 | 98.58 | 16.75 | 97.08 | 10.86 | 194.17 | 15.16 | 97.08 | 12.28 | 194.17 | 18.01 |
| | | 97.17 | 16.74 | 194.33 | 17.78 | 97.16 | 10.48 | 98.66 | 16.87 | 97.16 | 10.73 | 194.33 | 15.05 | 97.16 | 12.37 | 194.33 | 17.93 |
| | | 97.25 | 16.78 | 194.50 | 17.81 | 97.25 | 10.74 | 98.75 | 16.86 | 97.25 | 10.73 | 194.50 | 15.07 | 97.24 | 12.35 | 194.50 | 17.96 |
| | | 97.33 | 16.77 | 194.67 | 17.87 | 97.33 | 10.43 | 98.83 | 16.80 | 97.33 | 10.75 | 194.67 | 15.06 | 97.33 | 12.36 | 194.67 | 18.01 |
| | | 97.42 | 16.79 | 194.83 | 17.80 | 97.41 | 10.46 | 98.91 | 16.79 | 97.41 | 10.87 | 194.83 | 15.19 | 97.41 | 12.31 | 194.83 | 17.93 |
| | | 97.50 | 16.77 | 195.00 | 17.97 | 97.50 | 10.49 | 99.00 | 16.75 | 97.50 | 10.72 | 195.00 | 15.06 | 97.49 | 12.32 | 195.00 | 17.99 |
| | | 97.58 | 16.79 | 195.17 | 17.94 | 97.58 | 10.75 | 99.08 | 16.79 | 97.58 | 10.86 | 195.17 | 15.13 | 97.58 | 12.41 | 195.17 | 17.94 |
| | | 97.67 | 16.80 | 195.33 | 18.03 | 97.66 | 10.42 | 99.16 | 16.81 | 97.66 | 10.73 | 195.33 | 15.11 | 97.66 | 12.43 | 195.33 | 17.92 |
| | | 97.75 | 16.84 | 195.50 | 17.94 | 97.75 | 10.74 | 99.25 | 16.83 | 97.75 | 10.73 | 195.50 | 15.12 | 97.74 | 12.51 | 195.50 | 17.93 |
| | | 97.83 | 16.81 | 195.67 | 17.99 | 97.83 | 10.41 | 99.33 | 16.89 | 97.83 | 10.84 | 195.67 | 15.20 | 97.83 | 12.45 | 195.67 | 17.98 |
| | | 97.92 | 16.82 | 195.83 | 17.95 | 97.91 | 10.41 | 99.41 | 16.90 | 97.91 | 10.76 | 195.83 | 15.23 | 97.91 | 12.54 | 195.83 | 17.96 |
| | | 98.00 | 16.85 | 196.00 | 17.90 | 98.00 | 10.45 | 99.50 | 16.91 | 98.00 | 10.72 | 196.00 | 15.25 | 97.99 | 12.44 | 196.00 | 17.94 |
| | | 98.08 | 16.95 | 196.17 | 17.97 | 98.08 | 10.47 | 99.58 | 16.91 | 98.08 | 10.85 | 196.17 | 15.29 | 98.08 | 12.44 | 196.17 | 17.94 |
| | | 98.17 | 16.98 | 196.33 | 18.02 | 98.16 | 10.72 | 99.66 | 16.92 | 98.16 | 10.73 | 196.33 | 15.33 | 98.16 | 12.57 | 196.33 | 18.03 |
| | | 98.25 | 17.01 | 196.50 | 18.03 | 98.25 | 10.42 | 99.75 | 16.93 | 98.25 | 10.85 | 196.50 | 15.30 | 98.24 | 12.44 | 196.50 | 17.98 |
| | | 98.33 | 16.96 | 196.67 | 18.02 | 98.33 | 10.75 | 99.83 | 16.94 | 98.33 | 10.72 | 196.67 | 15.34 | 98.33 | 12.51 | 196.67 | 18.06 |
| | | 98.42 | 16.97 | 196.83 | 18.11 | 98.41 | 10.39 | 99.91 | 16.92 | 98.41 | 10.73 | 196.83 | 15.40 | 98.41 | 12.45 | 196.83 | 18.04 |
| | | 98.50 | 16.99 | 197.00 | 18.14 | 98.50 | 10.46 | 100.00 | 16.93 | 98.50 | 10.77 | 197.00 | 15.40 | 98.49 | 12.43 | 197.00 | 18.05 |
| | | 98.58 | 17.01 | 197.17 | 18.04 | 98.58 | 10.71 | 100.08 | 16.95 | 98.58 | 10.85 | 197.17 | 15.43 | 98.58 | 12.46 | 197.17 | 18.09 |
| | | 98.67 | 17.06 | 197.33 | 18.15 | 98.66 | 10.49 | 100.16 | 16.92 | 98.66 | 10.76 | 197.33 | 15.42 | 98.66 | 12.57 | 197.33 | 18.09 |
| | | 98.75 | 17.09 | 197.50 | 18.12 | 98.75 | 10.46 | 100.25 | 16.99 | 98.75 | 10.85 | 197.50 | 15.53 | 98.74 | 12.45 | 197.50 | 18.10 |
| | | 98.83 | 17.06 | 197.67 | 18.04 | 98.83 | 10.73 | 100.33 | 17.01 | 98.83 | 10.76 | 197.67 | 15.55 | 98.83 | 12.50 | 197.67 | 18.12 |
| | | 98.92 | 17.10 | 197.83 | 18.12 | 98.91 | 10.69 | 100.41 | 16.96 | 98.91 | 10.75 | 197.83 | 15.59 | 98.91 | 12.48 | 197.83 | 18.12 |
| | | 99.00 | 17.11 | 198.00 | 18.20 | 99.00 | 10.34 | 100.50 | 16.99 | 99.00 | 10.74 | 198.00 | 15.54 | 98.99 | 12.46 | 198.00 | 18.10 |
| | | 99.08 | 17.11 | 198.17 | 18.14 | 99.08 | 10.46 | 100.58 | 16.95 | 99.08 | 10.76 | 198.17 | 15.60 | 99.08 | 12.45 | 198.17 | 18.16 |
| | | 99.17 | 17.13 | 198.33 | 18.20 | 99.16 | 10.62 | 100.66 | 17.04 | 99.16 | 10.85 | 198.33 | 15.48 | 99.16 | 12.56 | 198.33 | 18.16 |
| | | 99.25 | 17.14 | 198.50 | 18.30 | 99.25 | 10.62 | 100.75 | 16.91 | 99.25 | 10.74 | 198.50 | 15.49 | 99.24 | 12.48 | 198.50 | 18.21 |
| | | 99.33 | 17.10 | 198.67 | 18.32 | 99.33 | 10.40 | 100.83 | 17.05 | 99.33 | 10.79 | 198.67 | 15.53 | 99.33 | 12.48 | 198.67 | 18.20 |
| | | 99.42 | 17.12 | 198.83 | 18.15 | 99.41 | 10.42 | 100.91 | 16.90 | 99.41 | 10.80 | 198.83 | 15.49 | 99.41 | 12.51 | 198.83 | 18.21 |
| | | 99.50 | 17.17 | 199.00 | 18.35 | 99.50 | 10.46 | 101.00 | 16.97 | 99.50 | 10.87 | 199.00 | 15.54 | 99.49 | 12.53 | 199.00 | 18.26 |
| | | 99.58 | 17.19 | 199.17 | 18.15 | 99.58 | 10.46 | 101.08 | 16.99 | 99.58 | 10.89 | 199.17 | 15.56 | 99.58 | 12.56 | 199.17 | 18.28 |
| | | 99.67 | 17.09 | 199.33 | 18.14 | 99.66 | 10.50 | 101.16 | 16.95 | 99.66 | 10.81 | 199.33 | 15.51 | 99.66 | 12.52 | 199.33 | 18.24 |
| | | 99.75 | 17.16 | 199.50 | 18.04 | 99.75 | 10.60 | 101.25 | 16.91 | 99.75 | 10.80 | 199.50 | 15.53 | 99.74 | 12.61 | 199.50 | 18.33 |
| | | 99.83 | 17.16 | 199.67 | 18.11 | 99.83 | 10.49 | 101.33 | 17.04 | 99.83 | 10.90 | 199.67 | 15.53 | 99.83 | 12.66 | 199.67 | 18.38 |
| | | 99.92 | 17.22 | 199.83 | 18.22 | 99.91 | 10.47 | 101.41 | 16.96 | 99.91 | 10.89 | 199.83 | 15.52 | 99.91 | 12.63 | 199.83 | 18.36 |
| | | 100.00 | 17.24 | 200.00 | 18.21 | 100.00 | 10.49 | 101.50 | 17.00 | 100.00 | 10.96 | 200.00 | 15.49 | 99.99 | 12.68 | 200.00 | 18.40 |
| | | 100.08 | 17.23 | 200.17 | 18.15 | 100.08 | 10.77 | 101.58 | 16.99 | 100.08 | 11.00 | 200.17 | 15.53 | 100.08 | 12.70 | 200.17 | 18.39 |
| | | 100.17 | 17.26 | 200.33 | 18.21 | 100.16 | 10.69 | 101.66 | 17.02 | 100.16 | 10.89 | 200.33 | 15.53 | 100.16 | 12.71 | 200.33 | 18.33 |
| | | 100.25 | 17.24 | 200.50 | 18.24 | 100.25 | 10.77 | 101.75 | 17.01 | 100.25 | 11.02 | 200.50 | 15.57 | 100.24 | 12.68 | 200.50 | 18.31 |
| | | 100.33 | 17.28 | 200.67 | 18.16 | 100.33 | 10.82 | 101.83 | 16.96 | 100.33 | 10.88 | 200.67 | 15.59 | 100.33 | 12.73 | 200.67 | 18.33 |
| | | 100.42 | 17.33 | 200.83 | 18.33 | 100.41 | 10.68 | 101.91 | 17.01 | 100.41 | 10.98 | 200.83 | 15.64 | 100.41 | 12.70 | 200.83 | 18.43 |
| | | 100.50 | 17.33 | 201.00 | 18.22 | 100.50 | 10.86 | 102.00 | 17.07 | 100.50 | 10.96 | 201.00 | 15.67 | 100.49 | 12.81 | 201.00 | 18.29 |
| | | 100.58 | 17.29 | 201.17 | 18.18 | 100.58 | 10.66 | 102.08 | 17.12 | 100.58 | 11.05 | 201.17 | 15.68 | 100.58 | 12.80 | 201.17 | 18.37 |
| | | 100.67 | 17.32 | 201.33 | 18.33 | 100.66 | 10.89 | 102.16 | 17.11 | 100.66 | 11.07 | 201.33 | 15.66 | 100.66 | 12.88 | 201.33 | 18.36 |

| | | | | | | | | | | | | | | | | | |
|---|---|---|---|---|---|---|---|---|---|---|---|---|---|---|---|---|---|
| | | 100.75 | 17.31 | 201.50 | 18.23 | 100.75 | 10.66 | 102.25 | 17.08 | 100.75 | 11.08 | 201.50 | 15.73 | 100.74 | 12.91 | 201.50 | 18.45 |
| | | 100.83 | 17.26 | 201.67 | 18.34 | 100.83 | 10.74 | 102.33 | 17.09 | 100.83 | 10.98 | 201.67 | 15.67 | 100.83 | 12.91 | 201.67 | 18.38 |
| | | 100.92 | 17.29 | 201.83 | 18.16 | 100.91 | 10.79 | 102.41 | 17.14 | 100.91 | 10.96 | 201.83 | 15.73 | 100.91 | 12.94 | 201.83 | 18.47 |
| | | 101.00 | 17.29 | 202.00 | 18.18 | 101.00 | 10.70 | 102.50 | 17.23 | 101.00 | 11.03 | 202.00 | 15.74 | 100.99 | 12.86 | 202.00 | 18.49 |
| | | 101.08 | 17.31 | 202.17 | 18.35 | 101.08 | 10.89 | 102.58 | 17.12 | 101.08 | 11.13 | 202.17 | 15.77 | 101.08 | 12.88 | 202.17 | 18.51 |
| | | 101.17 | 17.31 | 202.33 | 18.15 | 101.16 | 10.69 | 102.66 | 17.13 | 101.16 | 11.17 | 202.33 | 15.72 | 101.16 | 12.89 | 202.33 | 18.55 |
| | | 101.25 | 17.31 | 202.50 | 18.19 | 101.25 | 10.71 | 102.75 | 17.24 | 101.25 | 11.09 | 202.50 | 15.82 | 101.24 | 12.90 | 202.50 | 18.54 |
| | | 101.33 | 17.37 | 202.67 | 18.35 | 101.33 | 10.91 | 102.83 | 17.07 | 101.33 | 11.16 | 202.67 | 15.81 | 101.33 | 13.02 | 202.67 | 18.52 |
| | | 101.42 | 17.28 | 202.83 | 18.14 | 101.41 | 10.76 | 102.91 | 17.08 | 101.41 | 11.06 | 202.83 | 15.76 | 101.41 | 12.86 | 202.83 | 18.61 |
| | | 101.50 | 17.27 | 203.00 | 18.22 | 101.50 | 10.94 | 103.00 | 17.10 | 101.50 | 11.08 | 203.00 | 15.79 | 101.49 | 12.87 | 203.00 | 18.60 |
| | | 101.58 | 17.30 | 203.17 | 18.35 | 101.58 | 10.97 | 103.08 | 17.15 | 101.58 | 11.10 | 203.17 | 15.88 | 101.58 | 13.00 | 203.17 | 18.59 |
| | | 101.67 | 17.35 | 203.33 | 18.12 | 101.66 | 10.97 | 103.16 | 17.18 | 101.66 | 11.10 | 203.33 | 15.81 | 101.66 | 12.90 | 203.33 | 18.64 |
| | | 101.75 | 17.33 | 203.50 | 18.16 | 101.75 | 10.99 | 103.25 | 17.15 | 101.75 | 11.16 | 203.50 | 15.82 | 101.74 | 12.92 | 203.50 | 18.68 |
| | | 101.83 | 17.33 | 203.67 | 18.31 | 101.83 | 10.82 | 103.33 | 17.10 | 101.83 | 11.14 | 203.67 | 15.94 | 101.83 | 12.90 | 203.67 | 18.65 |
| | | 101.92 | 17.38 | 203.83 | 18.16 | 101.91 | 10.84 | 103.41 | 17.17 | 101.91 | 11.16 | 203.83 | 15.93 | 101.91 | 12.97 | 203.83 | 18.68 |
| | | 102.00 | 17.37 | 204.00 | 18.17 | 102.00 | 10.88 | 103.50 | 17.11 | 102.00 | 11.16 | 204.00 | 15.80 | 101.99 | 12.85 | 204.00 | 18.65 |
| | | 102.08 | 17.45 | 204.17 | 18.35 | 102.08 | 10.89 | 103.58 | 17.20 | 102.08 | 11.19 | 204.17 | 15.89 | 102.08 | 12.86 | 204.17 | 18.65 |
| | | 102.17 | 17.46 | 204.33 | 18.20 | 102.16 | 10.89 | 103.66 | 17.25 | 102.16 | 11.26 | 204.33 | 15.98 | 102.16 | 12.91 | 204.33 | 18.70 |
| | | 102.25 | 17.46 | 204.50 | 18.16 | 102.25 | 11.12 | 103.75 | 17.21 | 102.25 | 11.30 | 204.50 | 16.01 | 102.24 | 12.97 | 204.50 | 18.66 |
| | | 102.33 | 17.49 | 204.67 | 18.33 | 102.33 | 11.07 | 103.83 | 17.24 | 102.33 | 11.33 | 204.67 | 15.93 | 102.33 | 12.93 | 204.67 | 18.69 |
| | | 102.42 | 17.51 | 204.83 | 18.19 | 102.41 | 10.81 | 103.91 | 17.28 | 102.41 | 11.21 | 204.83 | 15.88 | 102.41 | 12.95 | 204.83 | 18.64 |
| | | 102.50 | 17.52 | 205.00 | 18.35 | 102.50 | 10.85 | 104.00 | 17.18 | 102.50 | 11.30 | 205.00 | 16.04 | 102.49 | 12.92 | 205.00 | 18.63 |
| | | 102.58 | 17.53 | 205.17 | 18.14 | 102.58 | 10.96 | 104.08 | 17.29 | 102.58 | 11.21 | 205.17 | 15.90 | 102.58 | 12.86 | 205.17 | 18.72 |
| | | 102.67 | 17.51 | 205.33 | 18.19 | 102.66 | 10.90 | 104.16 | 17.31 | 102.66 | 11.22 | 205.33 | 15.87 | 102.66 | 12.95 | 205.33 | 18.71 |
| | | 102.75 | 17.53 | 205.50 | 18.33 | 102.75 | 10.85 | 104.25 | 17.33 | 102.75 | 11.25 | 205.50 | 16.02 | 102.74 | 13.00 | 205.50 | 18.67 |
| | | 102.83 | 17.55 | 205.67 | 18.15 | 102.83 | 11.10 | 104.33 | 17.36 | 102.83 | 11.33 | 205.67 | 15.93 | 102.83 | 13.05 | 205.67 | 18.73 |
| | | 102.92 | 17.55 | 205.83 | 18.17 | 102.91 | 10.83 | 104.41 | 17.30 | 102.91 | 11.23 | 205.83 | 15.86 | 102.91 | 13.06 | 205.83 | 18.74 |
| | | 103.00 | 17.54 | 206.00 | 18.34 | 103.00 | 10.90 | 104.50 | 17.27 | 103.00 | 11.23 | 206.00 | 15.92 | 102.99 | 13.04 | 206.00 | 18.71 |
| | | 103.08 | 17.56 | 206.17 | 18.18 | 103.08 | 10.90 | 104.58 | 17.38 | 103.08 | 11.30 | 206.17 | 15.97 | 103.08 | 13.02 | 206.17 | 18.83 |
| | | 103.17 | 17.62 | 206.33 | 18.18 | 103.16 | 10.90 | 104.66 | 17.27 | 103.16 | 11.32 | 206.33 | 15.88 | 103.16 | 13.07 | 206.33 | 18.75 |
| | | 103.25 | 17.66 | 206.50 | 18.33 | 103.25 | 11.09 | 104.75 | 17.32 | 103.25 | 11.27 | 206.50 | 15.95 | 103.24 | 13.10 | 206.50 | 18.83 |
| | | 103.33 | 17.65 | 206.67 | 18.20 | 103.33 | 10.89 | 104.83 | 17.29 | 103.33 | 11.30 | 206.67 | 16.00 | 103.33 | 13.16 | 206.67 | 18.85 |
| | | 103.42 | 17.67 | 206.83 | 18.17 | 103.41 | 11.09 | 104.91 | 17.34 | 103.41 | 11.38 | 206.83 | 15.95 | 103.41 | 13.19 | 206.83 | 18.75 |
| | | 103.50 | 17.61 | 207.00 | 18.33 | 103.50 | 10.85 | 105.00 | 17.24 | 103.50 | 11.41 | 207.00 | 16.01 | 103.49 | 13.19 | 207.00 | 18.72 |
| | | 103.58 | 17.61 | 207.17 | 18.19 | 103.58 | 10.90 | 105.08 | 17.39 | 103.58 | 11.30 | 207.17 | 16.05 | 103.58 | 13.17 | 207.17 | 18.88 |
| | | 103.67 | 17.67 | 207.33 | 18.34 | 103.66 | 10.90 | 105.16 | 17.30 | 103.66 | 11.36 | 207.33 | 16.05 | 103.66 | 13.19 | 207.33 | 18.81 |
| | | 103.75 | 17.70 | 207.50 | 18.20 | 103.75 | 10.90 | 105.25 | 17.28 | 103.75 | 11.42 | 207.50 | 16.07 | 103.74 | 13.22 | 207.50 | 18.79 |
| | | 103.83 | 17.66 | 207.67 | 18.20 | 103.83 | 11.09 | 105.33 | 17.31 | 103.83 | 11.47 | 207.67 | 16.07 | 103.83 | 13.23 | 207.67 | 18.67 |
| | | 103.92 | 17.67 | 207.83 | 18.35 | 103.91 | 10.85 | 105.41 | 17.32 | 103.91 | 11.49 | 207.83 | 16.09 | 103.91 | 13.33 | 207.83 | 18.81 |
| | | 104.00 | 17.69 | 208.00 | 18.22 | 104.00 | 10.89 | 105.50 | 17.29 | 104.00 | 11.40 | 208.00 | 16.13 | 103.99 | 13.31 | 208.00 | 18.80 |
| | | 104.08 | 17.67 | 208.17 | 18.23 | 104.08 | 11.07 | 105.58 | 17.26 | 104.08 | 11.49 | 208.17 | 16.08 | 104.08 | 13.33 | 208.17 | 18.78 |
| | | 104.17 | 17.71 | 208.33 | 18.18 | 104.16 | 10.83 | 105.66 | 17.36 | 104.16 | 11.38 | 208.33 | 16.11 | 104.16 | 13.34 | 208.33 | 18.75 |
| | | 104.25 | 17.77 | 208.50 | 18.21 | 104.25 | 10.90 | 105.75 | 17.28 | 104.25 | 11.51 | 208.50 | 16.03 | 104.24 | 13.35 | 208.50 | 18.79 |
| | | 104.33 | 17.76 | 208.67 | 18.26 | 104.33 | 10.92 | 105.83 | 17.32 | 104.33 | 11.35 | 208.67 | 16.13 | 104.33 | 13.31 | 208.67 | 18.83 |
| | | 104.42 | 17.81 | 208.83 | 18.19 | 104.41 | 10.90 | 105.91 | 17.32 | 104.41 | 11.43 | 208.83 | 16.18 | 104.41 | 13.42 | 208.83 | 18.73 |
| | | 104.50 | 17.83 | 209.00 | 18.32 | 104.50 | 11.10 | 106.00 | 17.32 | 104.50 | 11.39 | 209.00 | 16.14 | 104.49 | 13.33 | 209.00 | 18.74 |
| | | 104.58 | 17.83 | 209.17 | 18.27 | 104.58 | 10.86 | 106.08 | 17.34 | 104.58 | 11.40 | 209.17 | 16.21 | 104.58 | 13.31 | 209.17 | 18.74 |
| | | 104.67 | 17.86 | 209.33 | 18.33 | 104.66 | 10.87 | 106.16 | 17.29 | 104.66 | 11.50 | 209.33 | 16.24 | 104.66 | 13.30 | 209.33 | 18.81 |
| | | 104.75 | 17.90 | 209.50 | 18.37 | 104.75 | 10.89 | 106.25 | 17.26 | 104.75 | 11.38 | 209.50 | 16.24 | 104.74 | 13.44 | 209.50 | 18.78 |
| | | 104.83 | 17.84 | 209.67 | 18.43 | 104.83 | 11.06 | 106.33 | 17.30 | 104.83 | 11.44 | 209.67 | 16.23 | 104.83 | 13.32 | 209.67 | 18.80 |
| | | 104.92 | 17.79 | 209.83 | 18.45 | 104.91 | 10.87 | 106.41 | 17.34 | 104.91 | 11.44 | 209.83 | 16.26 | 104.91 | 13.28 | 209.83 | 18.74 |

| | | | | | | | | | | | | | | | | | |
|---|---|---|---|---|---|---|---|---|---|---|---|---|---|---|---|---|---|
| | | 105.00 | 17.85 | 210.00 | 18.36 | 105.00 | 10.90 | 106.50 | 17.30 | 105.00 | 11.49 | 210.00 | 16.21 | 104.99 | 13.32 | 210.00 | 18.72 |
| | | 105.08 | 17.99 | 210.17 | 18.46 | 105.08 | 11.07 | 106.58 | 17.30 | 105.08 | 11.40 | 210.17 | 16.21 | 105.08 | 13.33 | 210.17 | 18.84 |
| | | 105.17 | 17.98 | 210.33 | 18.37 | 105.16 | 10.84 | 106.66 | 17.41 | 105.16 | 11.43 | 210.33 | 16.18 | 105.16 | 13.39 | 210.33 | 18.75 |
| | | 105.25 | 17.84 | 210.50 | 18.38 | 105.25 | 11.07 | 106.75 | 17.26 | 105.25 | 11.43 | 210.50 | 16.19 | 105.24 | 13.33 | 210.50 | 18.75 |
| | | 105.33 | 17.82 | 210.67 | 18.36 | 105.33 | 10.95 | 106.83 | 17.37 | 105.33 | 11.46 | 210.67 | 16.22 | 105.33 | 13.31 | 210.67 | 18.80 |
| | | 105.42 | 17.57 | 210.83 | 18.42 | 105.41 | 11.12 | 106.91 | 17.30 | 105.41 | 11.49 | 210.83 | 16.18 | 105.41 | 13.41 | 210.83 | 18.77 |
| | | 105.50 | 17.76 | 211.00 | 18.45 | 105.50 | 11.20 | 107.00 | 17.34 | 105.50 | 11.50 | 211.00 | 16.22 | 105.49 | 13.30 | 211.00 | 18.77 |
| | | 105.58 | 17.92 | 211.17 | 18.40 | 105.58 | 10.98 | 107.08 | 17.37 | 105.58 | 11.42 | 211.17 | 16.27 | 105.58 | 13.33 | 211.17 | 18.78 |
| | | 105.67 | 17.92 | 211.33 | 18.45 | 105.66 | 11.25 | 107.16 | 17.30 | 105.66 | 11.45 | 211.33 | 16.20 | 105.66 | 13.31 | 211.33 | 18.84 |
| | | 105.75 | 17.89 | 211.50 | 18.53 | 105.75 | 10.94 | 107.25 | 17.27 | 105.75 | 11.49 | 211.50 | 16.17 | 105.74 | 13.34 | 211.50 | 18.74 |
| | | 105.83 | 17.87 | 211.67 | 18.40 | 105.83 | 10.98 | 107.33 | 17.36 | 105.83 | 11.48 | 211.67 | 16.27 | 105.83 | 13.39 | 211.67 | 18.77 |
| | | 105.92 | 17.95 | 211.83 | 18.61 | 105.91 | 11.27 | 107.41 | 17.28 | 105.91 | 11.59 | 211.83 | 16.24 | 105.91 | 13.36 | 211.83 | 18.84 |
| | | 106.00 | 18.00 | 212.00 | 18.61 | 106.00 | 10.98 | 107.50 | 17.34 | 106.00 | 11.62 | 212.00 | 16.30 | 105.99 | 13.37 | 212.00 | 18.75 |
| | | 106.08 | 18.04 | 212.17 | 18.51 | 106.08 | 10.98 | 107.58 | 17.33 | 106.08 | 11.57 | 212.17 | 16.35 | 106.08 | 13.45 | 212.17 | 18.76 |
| | | 106.17 | 18.02 | 212.33 | 18.52 | 106.16 | 10.98 | 107.66 | 17.36 | 106.16 | 11.60 | 212.33 | 16.38 | 106.16 | 13.47 | 212.33 | 18.86 |
| | | 106.25 | 18.05 | 212.50 | 18.51 | 106.25 | 11.29 | 107.75 | 17.43 | 106.25 | 11.56 | 212.50 | 16.40 | 106.24 | 13.50 | 212.50 | 18.77 |
| | | 106.33 | 18.05 | 212.67 | 18.52 | 106.33 | 10.96 | 107.83 | 17.32 | 106.33 | 11.65 | 212.67 | 16.38 | 106.33 | 13.52 | 212.67 | 18.79 |
| | | 106.42 | 18.03 | 212.83 | 18.61 | 106.41 | 11.00 | 107.91 | 17.33 | 106.41 | 11.59 | 212.83 | 16.36 | 106.41 | 13.49 | 212.83 | 18.88 |
| | | 106.50 | 18.10 | 213.00 | 18.53 | 106.50 | 11.04 | 108.00 | 17.43 | 106.50 | 11.59 | 213.00 | 16.26 | 106.49 | 13.48 | 213.00 | 18.83 |
| | | 106.58 | 18.12 | 213.17 | 18.56 | 106.58 | 11.03 | 108.08 | 17.47 | 106.58 | 11.58 | 213.17 | 16.32 | 106.58 | 13.46 | 213.17 | 18.83 |
| | | 106.67 | 18.08 | 213.33 | 18.58 | 106.66 | 11.26 | 108.16 | 17.44 | 106.66 | 11.58 | 213.33 | 16.36 | 106.66 | 13.54 | 213.33 | 18.76 |
| | | 106.75 | 18.11 | 213.50 | 18.49 | 106.75 | 10.98 | 108.25 | 17.48 | 106.75 | 11.65 | 213.50 | 16.34 | 106.74 | 13.58 | 213.50 | 18.79 |
| | | 106.83 | 18.16 | 213.67 | 18.53 | 106.83 | 11.30 | 108.33 | 17.50 | 106.83 | 11.58 | 213.67 | 16.36 | 106.83 | 13.64 | 213.67 | 18.85 |
| | | 106.92 | 18.15 | 213.83 | 18.57 | 106.91 | 10.94 | 108.41 | 17.50 | 106.91 | 11.60 | 213.83 | 16.40 | 106.91 | 13.61 | 213.83 | 18.80 |
| | | 107.00 | 18.14 | 214.00 | 18.49 | 107.00 | 11.21 | 108.50 | 17.51 | 107.00 | 11.58 | 214.00 | 16.34 | 106.99 | 13.67 | 214.00 | 18.79 |
| | | 107.08 | 18.16 | 214.17 | 18.51 | 107.08 | 10.98 | 108.58 | 17.50 | 107.08 | 11.59 | 214.17 | 16.34 | 107.08 | 13.59 | 214.17 | 18.92 |
| | | 107.17 | 18.10 | 214.33 | 18.55 | 107.16 | 10.99 | 108.66 | 17.54 | 107.16 | 11.62 | 214.33 | 16.34 | 107.16 | 13.64 | 214.33 | 18.87 |
| | | 107.25 | 18.12 | 214.50 | 18.49 | 107.25 | 11.26 | 108.75 | 17.60 | 107.25 | 11.61 | 214.50 | 16.35 | 107.24 | 13.73 | 214.50 | 18.82 |
| | | 107.33 | 18.19 | 214.67 | 18.51 | 107.33 | 10.97 | 108.83 | 17.62 | 107.33 | 11.68 | 214.67 | 16.37 | 107.33 | 13.79 | 214.67 | 18.93 |
| | | 107.42 | 18.17 | 214.83 | 18.56 | 107.41 | 10.99 | 108.91 | 17.58 | 107.41 | 11.73 | 214.83 | 16.34 | 107.41 | 13.72 | 214.83 | 18.98 |
| | | 107.50 | 18.17 | 215.00 | 18.49 | 107.50 | 11.30 | 109.00 | 17.63 | 107.50 | 11.74 | 215.00 | 16.35 | 107.49 | 13.84 | 215.00 | 18.96 |
| | | 107.58 | 18.20 | 215.17 | 18.49 | 107.58 | 10.96 | 109.08 | 17.63 | 107.58 | 11.78 | 215.17 | 16.43 | 107.58 | 13.72 | 215.17 | 18.98 |
| | | 107.67 | 18.16 | 215.33 | 18.54 | 107.66 | 10.99 | 109.16 | 17.62 | 107.66 | 11.83 | 215.33 | 16.43 | 107.66 | 13.84 | 215.33 | 19.04 |
| | | 107.75 | 18.18 | 215.50 | 18.50 | 107.75 | 11.00 | 109.25 | 17.63 | 107.75 | 11.68 | 215.50 | 16.43 | 107.74 | 13.79 | 215.50 | 18.98 |
| | | 107.83 | 18.21 | 215.67 | 18.49 | 107.83 | 11.04 | 109.33 | 17.65 | 107.83 | 11.82 | 215.67 | 16.48 | 107.83 | 13.84 | 215.67 | 19.07 |
| | | 107.92 | 18.22 | 215.83 | 18.55 | 107.91 | 11.29 | 109.41 | 17.68 | 107.91 | 11.71 | 215.83 | 16.50 | 107.91 | 13.95 | 215.83 | 19.08 |
| | | 108.00 | 18.19 | 216.00 | 18.54 | 108.00 | 10.98 | 109.50 | 17.64 | 108.00 | 11.69 | 216.00 | 16.52 | 107.99 | 13.93 | 216.00 | 19.05 |
| | | 108.08 | 18.17 | 216.17 | 18.57 | 108.08 | 11.00 | 109.58 | 17.59 | 108.08 | 11.73 | 216.17 | 16.55 | 108.08 | 13.94 | 216.17 | 19.05 |
| | | 108.17 | 18.21 | 216.33 | 18.61 | 108.16 | 11.03 | 109.66 | 17.67 | 108.16 | 11.82 | 216.33 | 16.57 | 108.16 | 13.94 | 216.33 | 19.08 |
| | | 108.25 | 18.23 | 216.50 | 18.51 | 108.25 | 11.07 | 109.75 | 17.71 | 108.25 | 11.68 | 216.50 | 16.60 | 108.24 | 13.86 | 216.50 | 19.03 |
| | | 108.33 | 18.33 | 216.67 | 18.50 | 108.33 | 11.06 | 109.83 | 17.68 | 108.33 | 11.83 | 216.67 | 16.63 | 108.33 | 13.94 | 216.67 | 19.08 |
| | | 108.42 | 18.34 | 216.83 | 18.54 | 108.41 | 11.27 | 109.91 | 17.74 | 108.41 | 11.70 | 216.83 | 16.67 | 108.41 | 13.99 | 216.83 | 19.17 |
| | | 108.50 | 18.33 | 217.00 | 18.62 | 108.50 | 11.27 | 110.00 | 17.77 | 108.50 | 11.71 | 217.00 | 16.62 | 108.49 | 14.01 | 217.00 | 19.14 |
| | | 108.58 | 18.32 | 217.17 | 18.70 | 108.58 | 10.95 | 110.08 | 17.76 | 108.58 | 11.72 | 217.17 | 16.73 | 108.58 | 14.00 | 217.17 | 19.11 |
| | | 108.67 | 18.39 | 217.33 | 18.73 | 108.66 | 10.98 | 110.16 | 17.77 | 108.66 | 11.84 | 217.33 | 16.72 | 108.66 | 14.08 | 217.33 | 19.14 |
| | | 108.75 | 18.32 | 217.50 | 18.61 | 108.75 | 11.07 | 110.25 | 17.76 | 108.75 | 11.71 | 217.50 | 16.65 | 108.74 | 14.10 | 217.50 | 19.20 |
| | | 108.83 | 18.36 | 217.67 | 18.67 | 108.83 | 11.11 | 110.33 | 17.83 | 108.83 | 11.70 | 217.67 | 16.78 | 108.83 | 14.05 | 217.67 | 19.24 |
| | | 108.92 | 18.35 | 217.83 | 18.63 | 108.91 | 11.06 | 110.41 | 17.86 | 108.91 | 11.81 | 217.83 | 16.73 | 108.91 | 14.14 | 217.83 | 19.22 |
| | | 109.00 | 18.34 | 218.00 | 18.78 | 109.00 | 11.28 | 110.50 | 17.80 | 109.00 | 11.69 | 218.00 | 16.84 | 108.99 | 14.23 | 218.00 | 19.26 |
| | | 109.08 | 18.34 | 218.17 | 18.64 | 109.08 | 11.00 | 110.58 | 17.85 | 109.08 | 11.71 | 218.17 | 16.72 | 109.08 | 14.14 | 218.17 | 19.12 |
| | | 109.17 | 18.42 | 218.33 | 18.63 | 109.16 | 11.05 | 110.66 | 17.83 | 109.16 | 11.83 | 218.33 | 16.77 | 109.16 | 14.25 | 218.33 | 19.18 |

| | | | | | | | | | | | | | | | | | |
|---|---|---|---|---|---|---|---|---|---|---|---|---|---|---|---|---|---|
| | | 109.25 | 18.34 | 218.50 | 18.79 | 109.25 | 11.26 | 110.75 | 17.84 | 109.25 | 11.74 | 218.50 | 16.80 | 109.24 | 14.11 | 218.50 | 19.26 |
| | | 109.33 | 18.33 | 218.67 | 18.70 | 109.33 | 11.08 | 110.83 | 17.83 | 109.33 | 11.83 | 218.67 | 16.84 | 109.33 | 14.12 | 218.67 | 19.19 |
| | | 109.42 | 18.43 | 218.83 | 18.67 | 109.41 | 11.08 | 110.91 | 17.87 | 109.41 | 11.67 | 218.83 | 16.84 | 109.41 | 14.27 | 218.83 | 19.20 |
| | | 109.50 | 18.40 | 219.00 | 18.62 | 109.50 | 11.06 | 111.00 | 17.89 | 109.50 | 11.71 | 219.00 | 16.89 | 109.49 | 14.20 | 219.00 | 19.13 |
| | | 109.58 | 18.43 | 219.17 | 18.72 | 109.58 | 11.27 | 111.08 | 17.87 | 109.58 | 11.70 | 219.17 | 16.89 | 109.58 | 14.31 | 219.17 | 19.19 |
| | | 109.67 | 18.50 | 219.33 | 18.66 | 109.66 | 11.04 | 111.16 | 17.90 | 109.66 | 11.72 | 219.33 | 16.91 | 109.66 | 14.27 | 219.33 | 19.23 |
| | | 109.75 | 18.51 | 219.50 | 18.63 | 109.75 | 11.10 | 111.25 | 17.88 | 109.75 | 11.76 | 219.50 | 16.93 | 109.74 | 14.33 | 219.50 | 19.13 |
| | | 109.83 | 18.48 | 219.67 | 18.67 | 109.83 | 11.27 | 111.33 | 17.92 | 109.83 | 11.74 | 219.67 | 16.91 | 109.83 | 14.38 | 219.67 | 19.21 |
| | | 109.92 | 18.50 | 219.83 | 18.66 | 109.91 | 11.25 | 111.41 | 17.91 | 109.91 | 11.70 | 219.83 | 16.85 | 109.91 | 14.40 | 219.83 | 19.26 |
| | | 110.00 | 18.53 | 220.00 | 18.69 | 110.00 | 11.28 | 111.50 | 17.89 | 110.00 | 11.83 | 220.00 | 16.88 | 109.99 | 14.29 | 220.00 | 19.19 |
| | | 110.08 | 18.55 | 220.17 | 18.69 | 110.08 | 11.29 | 111.58 | 17.93 | 110.08 | 11.74 | 220.17 | 16.92 | 110.08 | 14.41 | 220.17 | 19.17 |
| | | 110.17 | 18.59 | 220.33 | 18.64 | 110.16 | 11.27 | 111.66 | 17.99 | 110.16 | 11.76 | 220.33 | 16.86 | 110.16 | 14.30 | 220.33 | 19.24 |
| | | 110.25 | 18.52 | 220.50 | 18.77 | 110.25 | 11.37 | 111.75 | 18.01 | 110.25 | 11.72 | 220.50 | 16.89 | 110.24 | 14.39 | 220.50 | 19.22 |
| | | 110.33 | 18.56 | 220.67 | 18.81 | 110.33 | 11.43 | 111.83 | 18.00 | 110.33 | 11.71 | 220.67 | 16.96 | 110.33 | 14.27 | 220.67 | 19.17 |
| | | 110.42 | 18.57 | 220.83 | 18.79 | 110.41 | 11.22 | 111.91 | 17.97 | 110.41 | 11.70 | 220.83 | 16.88 | 110.41 | 14.40 | 220.83 | 19.18 |
| | | 110.50 | 18.54 | 221.00 | 18.88 | 110.50 | 11.26 | 112.00 | 18.03 | 110.50 | 11.78 | 221.00 | 16.93 | 110.49 | 14.33 | 221.00 | 19.16 |
| | | 110.58 | 18.53 | 221.17 | 18.85 | 110.58 | 11.42 | 112.08 | 17.92 | 110.58 | 11.77 | 221.17 | 16.99 | 110.58 | 14.29 | 221.17 | 19.26 |
| | | 110.67 | 18.54 | 221.33 | 18.89 | 110.66 | 11.29 | 112.16 | 18.05 | 110.66 | 11.82 | 221.33 | 16.94 | 110.66 | 14.43 | 221.33 | 19.19 |
| | | 110.75 | 18.62 | 221.50 | 18.95 | 110.75 | 11.45 | 112.25 | 17.92 | 110.75 | 11.86 | 221.50 | 16.92 | 110.74 | 14.28 | 221.50 | 19.18 |
| | | 110.83 | 18.56 | 221.67 | 18.98 | 110.83 | 11.21 | 112.33 | 18.01 | 110.83 | 11.90 | 221.67 | 17.04 | 110.83 | 14.41 | 221.67 | 19.26 |
| | | 110.92 | 18.55 | 221.83 | 18.98 | 110.91 | 11.44 | 112.41 | 17.97 | 110.91 | 11.90 | 221.83 | 17.09 | 110.91 | 14.33 | 221.83 | 19.17 |
| | | 111.00 | 18.60 | 222.00 | 19.04 | 111.00 | 11.22 | 112.50 | 18.04 | 111.00 | 11.92 | 222.00 | 17.01 | 110.99 | 14.30 | 222.00 | 19.18 |
| | | 111.08 | 18.63 | 222.17 | 18.99 | 111.08 | 11.38 | 112.58 | 17.94 | 111.08 | 11.91 | 222.17 | 17.04 | 111.08 | 14.40 | 222.17 | 19.26 |
| | | 111.17 | 18.66 | 222.33 | 18.92 | 111.16 | 11.38 | 112.66 | 18.04 | 111.16 | 11.95 | 222.33 | 17.15 | 111.16 | 14.38 | 222.33 | 19.12 |
| | | 111.25 | 18.66 | 222.50 | 18.94 | 111.25 | 11.39 | 112.75 | 17.92 | 111.25 | 11.91 | 222.50 | 17.20 | 111.24 | 14.44 | 222.50 | 19.18 |
| | | 111.33 | 18.64 | 222.67 | 18.97 | 111.33 | 11.40 | 112.83 | 17.97 | 111.33 | 11.93 | 222.67 | 17.18 | 111.33 | 14.46 | 222.67 | 19.19 |
| | | 111.42 | 18.65 | 222.83 | 19.06 | 111.41 | 11.37 | 112.91 | 18.03 | 111.41 | 11.97 | 222.83 | 17.09 | 111.41 | 14.46 | 222.83 | 19.29 |
| | | 111.50 | 18.70 | 223.00 | 18.98 | 111.50 | 11.46 | 113.00 | 17.94 | 111.50 | 11.98 | 223.00 | 17.16 | 111.49 | 14.44 | 223.00 | 19.19 |
| | | 111.58 | 18.71 | 223.17 | 19.09 | 111.58 | 11.62 | 113.08 | 18.04 | 111.58 | 11.92 | 223.17 | 17.18 | 111.58 | 14.39 | 223.17 | 19.17 |
| | | 111.67 | 18.69 | 223.33 | 19.01 | 111.66 | 11.59 | 113.16 | 18.03 | 111.66 | 11.92 | 223.33 | 17.07 | 111.66 | 14.47 | 223.33 | 19.28 |
| | | 111.75 | 18.68 | 223.50 | 19.13 | 111.75 | 11.46 | 113.25 | 17.94 | 111.75 | 12.03 | 223.50 | 17.08 | 111.74 | 14.49 | 223.50 | 19.18 |
| | | 111.83 | 18.71 | 223.67 | 19.21 | 111.83 | 11.41 | 113.33 | 17.99 | 111.83 | 12.05 | 223.67 | 17.14 | 111.83 | 14.57 | 223.67 | 19.27 |
| | | 111.92 | 18.72 | 223.83 | 19.20 | 111.91 | 11.62 | 113.41 | 18.00 | 111.91 | 12.10 | 223.83 | 17.29 | 111.91 | 14.49 | 223.83 | 19.23 |
| | | 112.00 | 18.71 | 224.00 | 19.09 | 112.00 | 11.40 | 113.50 | 18.04 | 112.00 | 12.06 | 224.00 | 17.17 | 111.99 | 14.44 | 224.00 | 19.22 |
| | | 112.08 | 18.72 | 224.17 | 19.23 | 112.08 | 11.61 | 113.58 | 18.04 | 112.08 | 12.12 | 224.17 | 17.22 | 112.08 | 14.52 | 224.17 | 19.24 |
| | | 112.17 | 18.75 | 224.33 | 19.04 | 112.16 | 11.46 | 113.66 | 17.97 | 112.16 | 12.06 | 224.33 | 17.14 | 112.16 | 14.53 | 224.33 | 19.29 |
| | | 112.25 | 18.70 | 224.50 | 19.10 | 112.25 | 11.52 | 113.75 | 17.96 | 112.25 | 12.05 | 224.50 | 17.13 | 112.24 | 14.63 | 224.50 | 19.30 |
| | | 112.33 | 18.73 | 224.67 | 19.26 | 112.33 | 11.46 | 113.83 | 17.98 | 112.33 | 12.12 | 224.67 | 17.17 | 112.33 | 14.58 | 224.67 | 19.31 |
| | | 112.42 | 18.74 | 224.83 | 19.06 | 112.41 | 11.40 | 113.91 | 18.01 | 112.41 | 12.11 | 224.83 | 17.12 | 112.41 | 14.57 | 224.83 | 19.33 |
| | | 112.50 | 18.73 | 225.00 | 19.09 | 112.50 | 11.59 | 114.00 | 18.03 | 112.50 | 12.12 | 225.00 | 17.15 | 112.49 | 14.59 | 225.00 | 19.34 |
| | | 112.58 | 18.75 | 225.17 | 19.25 | 112.58 | 11.48 | 114.08 | 18.04 | 112.58 | 12.14 | 225.17 | 17.18 | 112.58 | 14.69 | 225.17 | 19.40 |
| | | 112.67 | 18.77 | 225.33 | 19.03 | 112.66 | 11.46 | 114.16 | 17.91 | 112.66 | 12.20 | 225.33 | 17.08 | 112.66 | 14.55 | 225.33 | 19.38 |
| | | 112.75 | 18.80 | 225.50 | 19.09 | 112.75 | 11.44 | 114.25 | 17.96 | 112.75 | 12.19 | 225.50 | 17.12 | 112.74 | 14.55 | 225.50 | 19.33 |
| | | 112.83 | 18.92 | 225.67 | 19.23 | 112.83 | 11.61 | 114.33 | 17.93 | 112.83 | 12.20 | 225.67 | 17.19 | 112.83 | 14.70 | 225.67 | 19.38 |
| | | 112.92 | 18.89 | 225.83 | 19.03 | 112.91 | 11.41 | 114.41 | 18.00 | 112.91 | 12.26 | 225.83 | 17.15 | 112.91 | 14.59 | 225.83 | 19.34 |
| | | 113.00 | 18.93 | 226.00 | 19.08 | 113.00 | 11.42 | 114.50 | 18.05 | 113.00 | 12.30 | 226.00 | 17.12 | 112.99 | 14.61 | 226.00 | 19.42 |
| | | 113.08 | 18.93 | 226.17 | 19.25 | 113.08 | 11.62 | 114.58 | 18.09 | 113.08 | 12.30 | 226.17 | 17.21 | 113.08 | 14.65 | 226.17 | 19.35 |
| | | 113.17 | 18.88 | 226.33 | 19.04 | 113.16 | 11.41 | 114.66 | 18.03 | 113.16 | 12.15 | 226.33 | 17.19 | 113.16 | 14.66 | 226.33 | 19.38 |
| | | 113.25 | 18.87 | 226.50 | 19.10 | 113.25 | 11.44 | 114.75 | 17.96 | 113.25 | 12.19 | 226.50 | 17.12 | 113.24 | 14.64 | 226.50 | 19.48 |
| | | 113.33 | 18.90 | 226.67 | 19.25 | 113.33 | 11.48 | 114.83 | 17.97 | 113.33 | 12.32 | 226.67 | 17.16 | 113.33 | 14.61 | 226.67 | 19.47 |
| | | 113.42 | 18.91 | 226.83 | 19.04 | 113.41 | 11.59 | 114.91 | 18.04 | 113.41 | 12.20 | 226.83 | 17.15 | 113.41 | 14.70 | 226.83 | 19.38 |

| | | | | | | | | | | | | | | | | | |
|---|---|---|---|---|---|---|---|---|---|---|---|---|---|---|---|---|---|
| | | 113.50 | 18.92 | 227.00 | 19.09 | 113.50 | 11.50 | 115.00 | 18.12 | 113.50 | 12.20 | 227.00 | 17.23 | 113.49 | 14.72 | 227.00 | 19.50 |
| | | 113.58 | 18.91 | 227.17 | 19.24 | 113.58 | 11.39 | 115.08 | 18.13 | 113.58 | 12.32 | 227.17 | 17.14 | 113.58 | 14.78 | 227.17 | 19.53 |
| | | 113.67 | 18.94 | 227.33 | 19.06 | 113.66 | 11.42 | 115.16 | 18.03 | 113.66 | 12.20 | 227.33 | 17.12 | 113.66 | 14.83 | 227.33 | 19.46 |
| | | 113.75 | 18.96 | 227.50 | 19.09 | 113.75 | 11.44 | 115.25 | 18.13 | 113.75 | 12.29 | 227.50 | 17.17 | 113.74 | 14.84 | 227.50 | 19.52 |
| | | 113.83 | 19.04 | 227.67 | 19.24 | 113.83 | 11.61 | 115.33 | 18.14 | 113.83 | 12.17 | 227.67 | 17.22 | 113.83 | 14.67 | 227.67 | 19.50 |
| | | 113.92 | 19.11 | 227.83 | 19.09 | 113.91 | 11.41 | 115.41 | 18.14 | 113.91 | 12.18 | 227.83 | 17.12 | 113.91 | 14.83 | 227.83 | 19.49 |
| | | 114.00 | 19.03 | 228.00 | 19.07 | 114.00 | 11.46 | 115.50 | 18.21 | 114.00 | 12.19 | 228.00 | 17.17 | 113.99 | 14.73 | 228.00 | 19.46 |
| | | 114.08 | 19.05 | 228.17 | 19.24 | 114.08 | 11.45 | 115.58 | 18.19 | 114.08 | 12.29 | 228.17 | 17.14 | 114.08 | 14.73 | 228.17 | 19.55 |
| | | 114.17 | 19.01 | 228.33 | 19.11 | 114.16 | 11.61 | 115.66 | 18.19 | 114.16 | 12.17 | 228.33 | 17.24 | 114.16 | 14.79 | 228.33 | 19.55 |
| | | 114.25 | 19.09 | 228.50 | 19.11 | 114.25 | 11.41 | 115.75 | 18.21 | 114.25 | 12.33 | 228.50 | 17.19 | 114.24 | 14.73 | 228.50 | 19.47 |
| | | 114.33 | 19.16 | 228.67 | 19.24 | 114.33 | 11.44 | 115.83 | 18.14 | 114.33 | 12.15 | 228.67 | 17.23 | 114.33 | 14.81 | 228.67 | 19.53 |
| | | 114.42 | 19.06 | 228.83 | 19.06 | 114.41 | 11.47 | 115.91 | 18.07 | 114.41 | 12.19 | 228.83 | 17.26 | 114.41 | 14.69 | 228.83 | 19.58 |
| | | 114.50 | 19.10 | 229.00 | 19.09 | 114.50 | 11.59 | 116.00 | 18.19 | 114.50 | 12.32 | 229.00 | 17.30 | 114.49 | 14.73 | 229.00 | 19.54 |
| | | 114.58 | 19.08 | 229.17 | 19.24 | 114.58 | 11.42 | 116.08 | 18.22 | 114.58 | 12.21 | 229.17 | 17.31 | 114.58 | 14.78 | 229.17 | 19.62 |
| | | 114.67 | 19.13 | 229.33 | 19.12 | 114.66 | 11.44 | 116.16 | 18.28 | 114.66 | 12.22 | 229.33 | 17.29 | 114.66 | 14.78 | 229.33 | 19.66 |
| | | 114.75 | 19.11 | 229.50 | 19.16 | 114.75 | 11.61 | 116.25 | 18.25 | 114.75 | 12.32 | 229.50 | 17.34 | 114.74 | 14.75 | 229.50 | 19.65 |
| | | 114.83 | 19.09 | 229.67 | 19.09 | 114.83 | 11.40 | 116.33 | 18.24 | 114.83 | 12.17 | 229.67 | 17.30 | 114.83 | 14.69 | 229.67 | 19.64 |
| | | 114.92 | 19.11 | 229.83 | 19.13 | 114.91 | 11.61 | 116.41 | 18.24 | 114.91 | 12.21 | 229.83 | 17.35 | 114.91 | 14.83 | 229.83 | 19.61 |
| | | 115.00 | 19.15 | 230.00 | 19.21 | 115.00 | 11.53 | 116.50 | 18.30 | 115.00 | 12.31 | 230.00 | 17.29 | 114.99 | 14.70 | 230.00 | 19.59 |
| | | 115.08 | 19.20 | 230.17 | 19.11 | 115.08 | 11.70 | 116.58 | 18.33 | 115.08 | 12.22 | 230.17 | 17.33 | 115.08 | 14.72 | 230.17 | 19.66 |
| | | 115.17 | 19.22 | 230.33 | 19.14 | 115.16 | 11.71 | 116.66 | 18.35 | 115.16 | 12.19 | 230.33 | 17.31 | 115.16 | 14.76 | 230.33 | 19.56 |
| | | 115.25 | 19.22 | 230.50 | 19.23 | 115.25 | 11.73 | 116.75 | 18.36 | 115.25 | 12.21 | 230.50 | 17.31 | 115.24 | 14.79 | 230.50 | 19.60 |
| | | 115.33 | 19.13 | 230.67 | 19.17 | 115.33 | 11.52 | 116.83 | 18.33 | 115.33 | 12.29 | 230.67 | 17.44 | 115.33 | 14.79 | 230.67 | 19.58 |
| | | 115.42 | 19.15 | 230.83 | 19.26 | 115.41 | 11.54 | 116.91 | 18.34 | 115.41 | 12.19 | 230.83 | 17.44 | 115.41 | 14.80 | 230.83 | 19.69 |
| | | 115.50 | 19.23 | 231.00 | 19.35 | 115.50 | 11.80 | 117.00 | 18.33 | 115.50 | 12.31 | 231.00 | 17.43 | 115.49 | 14.78 | 231.00 | 19.60 |
| | | 115.58 | 19.24 | 231.17 | 19.27 | 115.58 | 11.51 | 117.08 | 18.39 | 115.58 | 12.21 | 231.17 | 17.46 | 115.58 | 14.85 | 231.17 | 19.57 |
| | | 115.67 | 19.24 | 231.33 | 19.33 | 115.66 | 11.52 | 117.16 | 18.42 | 115.66 | 12.21 | 231.33 | 17.45 | 115.66 | 14.91 | 231.33 | 19.66 |
| | | 115.75 | 19.24 | 231.50 | 19.25 | 115.75 | 11.56 | 117.25 | 18.46 | 115.75 | 12.21 | 231.50 | 17.49 | 115.74 | 14.88 | 231.50 | 19.57 |
| | | 115.83 | 19.16 | 231.67 | 19.29 | 115.83 | 11.85 | 117.33 | 18.48 | 115.83 | 12.21 | 231.67 | 17.46 | 115.83 | 14.89 | 231.67 | 19.61 |
| | | 115.92 | 19.15 | 231.83 | 19.39 | 115.91 | 11.58 | 117.41 | 18.46 | 115.91 | 12.32 | 231.83 | 17.51 | 115.91 | 14.95 | 231.83 | 19.67 |
| | | 116.00 | 19.22 | 232.00 | 19.20 | 116.00 | 11.59 | 117.50 | 18.42 | 116.00 | 12.18 | 232.00 | 17.59 | 115.99 | 14.85 | 232.00 | 19.56 |
| | | 116.08 | 19.23 | 232.17 | 19.29 | 116.08 | 11.59 | 117.58 | 18.46 | 116.08 | 12.19 | 232.17 | 17.53 | 116.08 | 14.97 | | |
| | | 116.17 | 19.24 | 232.33 | 19.43 | 116.16 | 11.60 | 117.66 | 18.47 | 116.16 | 12.30 | 232.33 | 17.54 | 116.16 | 14.90 | | |
| | | 116.25 | 19.22 | 232.50 | 19.42 | 116.25 | 11.86 | 117.75 | 18.50 | 116.25 | 12.21 | 232.50 | 17.62 | 116.24 | 14.96 | | |
| | | 116.33 | 19.18 | 232.67 | 19.47 | 116.33 | 11.67 | 117.83 | 18.53 | 116.33 | 12.25 | 232.67 | 17.64 | 116.33 | 15.03 | | |
| | | 116.42 | 19.19 | 232.83 | 19.49 | 116.41 | 11.62 | 117.91 | 18.54 | 116.41 | 12.22 | 232.83 | 17.50 | 116.41 | 15.08 | | |
| | | 116.50 | 19.19 | 233.00 | 19.37 | 116.50 | 11.83 | 118.00 | 18.43 | 116.50 | 12.20 | 233.00 | 17.57 | 116.49 | 15.10 | | |
| | | 116.58 | 19.23 | 233.17 | 19.37 | 116.58 | 11.56 | 118.08 | 18.57 | 116.58 | 12.19 | 233.17 | 17.67 | 116.58 | 14.97 | | |
| | | 116.67 | 19.19 | 233.33 | 19.48 | 116.66 | 11.57 | 118.16 | 18.45 | 116.66 | 12.32 | 233.33 | 17.65 | 116.66 | 15.10 | | |
| | | 116.75 | 19.21 | 233.50 | 19.40 | 116.75 | 11.63 | 118.25 | 18.47 | 116.75 | 12.22 | 233.50 | 17.62 | 116.74 | 14.97 | | |
| | | 116.83 | 19.25 | 233.67 | 19.40 | 116.83 | 11.82 | 118.33 | 18.51 | 116.83 | 12.19 | 233.67 | 17.60 | 116.83 | 14.95 | | |
| | | 116.92 | 19.29 | 233.83 | 19.42 | 116.91 | 11.53 | 118.41 | 18.50 | 116.91 | 12.19 | 233.83 | 17.48 | 116.91 | 14.98 | | |
| | | 117.00 | 19.21 | 234.00 | 19.54 | 117.00 | 11.86 | 118.50 | 18.43 | 117.00 | 12.32 | 234.00 | 17.57 | 116.99 | 15.00 | | |
| | | 117.08 | 19.30 | 234.17 | 19.40 | 117.08 | 11.66 | 118.58 | 18.43 | 117.08 | 12.24 | 234.17 | 17.60 | 117.08 | 15.14 | | |
| | | 117.17 | 19.24 | 234.33 | 19.43 | 117.16 | 11.58 | 118.66 | 18.58 | 117.16 | 12.30 | 234.33 | 17.48 | 117.16 | 14.96 | | |
| | | 117.25 | 19.27 | 234.50 | 19.40 | 117.25 | 11.80 | 118.75 | 18.42 | 117.25 | 12.27 | 234.50 | 17.55 | 117.24 | 15.11 | | |
| | | 117.33 | 19.33 | 234.67 | 19.43 | 117.33 | 11.59 | 118.83 | 18.50 | 117.33 | 12.30 | 234.67 | 17.57 | 117.33 | 14.96 | | |
| | | 117.42 | 19.33 | 234.83 | 19.48 | 117.41 | 11.64 | 118.91 | 18.51 | 117.41 | 12.30 | 234.83 | 17.54 | 117.41 | 14.97 | | |
| | | 117.50 | 19.35 | 235.00 | 19.43 | 117.50 | 11.59 | 119.00 | 18.45 | 117.50 | 12.38 | 235.00 | 17.63 | 117.49 | 15.00 | | |
| | | 117.58 | 19.29 | 235.17 | 19.57 | 117.58 | 11.82 | 119.08 | 18.44 | 117.58 | 12.36 | 235.17 | 17.54 | 117.58 | 15.11 | | |
| | | 117.67 | 19.33 | 235.33 | 19.59 | 117.66 | 11.54 | 119.16 | 18.57 | 117.66 | 12.35 | 235.33 | 17.56 | 117.66 | 15.06 | | |

| | | | | | | | | | | | | | | | | | |
|---|---|---|---|---|---|---|---|---|---|---|---|---|---|---|---|---|---|
| | | 117.75 | 19.34 | 235.50 | 19.62 | 117.75 | 11.56 | 119.25 | 18.43 | 117.75 | 12.42 | 235.50 | 17.62 | 117.74 | 14.98 | | |
| | | 117.83 | 19.41 | 235.67 | 19.55 | 117.83 | 11.82 | 119.33 | 18.49 | 117.83 | 12.48 | 235.67 | 17.50 | 117.83 | 14.97 | | |
| | | 117.92 | 19.42 | 235.83 | 19.65 | 117.91 | 11.68 | 119.41 | 18.52 | 117.91 | 12.37 | 235.83 | 17.56 | 117.91 | 14.99 | | |
| | | 118.00 | 19.44 | 236.00 | 19.47 | 118.00 | 11.70 | 119.50 | 18.43 | 118.00 | 12.48 | 236.00 | 17.58 | 117.99 | 15.00 | | |
| | | 118.08 | 19.49 | 236.17 | 19.53 | 118.08 | 11.64 | 119.58 | 18.54 | 118.08 | 12.38 | 236.17 | 17.71 | 118.08 | 15.11 | | |
| | | 118.17 | 19.37 | 236.33 | 19.67 | 118.16 | 11.49 | 119.66 | 18.45 | 118.16 | 12.40 | 236.33 | 17.52 | 118.16 | 15.00 | | |
| | | 118.25 | 19.44 | 236.50 | 19.48 | 118.25 | 11.74 | 119.75 | 18.55 | 118.25 | 12.34 | 236.50 | 17.57 | 118.24 | 14.99 | | |
| | | 118.33 | 19.49 | 236.67 | 19.52 | 118.33 | 11.52 | 119.83 | 18.46 | 118.33 | 12.36 | 236.67 | 17.67 | 118.33 | 15.14 | | |
| | | 118.42 | 19.52 | 236.83 | 19.67 | 118.41 | 11.60 | 119.91 | 18.45 | 118.41 | 12.40 | 236.83 | 17.52 | 118.41 | 14.98 | | |
| | | 118.50 | 19.49 | 237.00 | 19.49 | 118.50 | 11.54 | 120.00 | 18.54 | 118.50 | 12.45 | 237.00 | 17.60 | 118.49 | 15.00 | | |
| | | 118.58 | 19.54 | 237.17 | 19.53 | 118.58 | 11.58 | 120.08 | 18.47 | 118.58 | 12.40 | 237.17 | 17.49 | 118.58 | 15.02 | | |
| | | 118.67 | 19.57 | 237.33 | 19.67 | 118.66 | 11.61 | 120.16 | 18.57 | 118.66 | 12.43 | 237.33 | 17.50 | 118.66 | 15.09 | | |
| | | 118.75 | 19.47 | 237.50 | 19.49 | 118.75 | 11.86 | 120.25 | 18.42 | 118.75 | 12.41 | 237.50 | 17.58 | 118.74 | 15.01 | | |
| | | 118.83 | 19.55 | 237.67 | 19.54 | 118.83 | 11.61 | 120.33 | 18.54 | 118.83 | 12.40 | 237.67 | 17.66 | 118.83 | 14.99 | | |
| | | 118.92 | 19.57 | 237.83 | 19.68 | 118.91 | 11.69 | 120.41 | 18.41 | 118.91 | 12.43 | 237.83 | 17.52 | 118.91 | 15.10 | | |
| | | 119.00 | 19.63 | 238.00 | 19.49 | 119.00 | 11.82 | 120.50 | 18.47 | 119.00 | 12.41 | 238.00 | 17.59 | 118.99 | 15.01 | | |
| | | 119.08 | 19.58 | 238.17 | 19.54 | 119.08 | 11.85 | 120.58 | 18.54 | 119.08 | 12.37 | 238.17 | 17.53 | 119.08 | 15.01 | | |
| | | 119.17 | 19.65 | 238.33 | 19.69 | 119.16 | 11.81 | 120.66 | 18.45 | 119.16 | 12.38 | 238.33 | 17.51 | 119.16 | 15.06 | | |
| | | 119.25 | 19.67 | 238.50 | 19.49 | 119.25 | 11.57 | 120.75 | 18.44 | 119.25 | 12.38 | 238.50 | 17.57 | 119.24 | 15.08 | | |
| | | 119.33 | 19.67 | 238.67 | 19.54 | 119.33 | 11.48 | 120.83 | 18.57 | 119.33 | 12.39 | 238.67 | 17.65 | 119.33 | 15.01 | | |
| | | 119.42 | 19.54 | 238.83 | 19.70 | 119.41 | 11.59 | 120.91 | 18.42 | 119.41 | 12.44 | 238.83 | 17.52 | 119.41 | 14.99 | | |
| | | 119.50 | 19.60 | 239.00 | 19.51 | 119.50 | 11.76 | 121.00 | 18.51 | 119.50 | 12.42 | 239.00 | 17.62 | 119.49 | 15.01 | | |
| | | 119.58 | 19.61 | 239.17 | 19.55 | 119.58 | 11.67 | 121.08 | 18.52 | 119.58 | 12.46 | 239.17 | 17.51 | 119.58 | 15.02 | | |
| | | 119.67 | 19.63 | 239.33 | 19.71 | 119.66 | 11.62 | 121.16 | 18.53 | 119.66 | 12.56 | 239.33 | 17.56 | 119.66 | 15.05 | | |
| | | 119.75 | 19.59 | 239.50 | 19.51 | 119.75 | 11.60 | 121.25 | 18.54 | 119.75 | 12.51 | 239.50 | 17.61 | 119.74 | 15.09 | | |
| | | 119.83 | 19.55 | 239.67 | 19.60 | 119.83 | 11.80 | 121.33 | 18.47 | 119.83 | 12.52 | 239.67 | 17.51 | 119.83 | 15.08 | | |
| | | 119.92 | 19.69 | 239.83 | 19.48 | 119.91 | 11.58 | 121.41 | 18.44 | 119.91 | 12.58 | 239.83 | 17.57 | 119.91 | 15.06 | | |
| | | 120.00 | 19.61 | 240.00 | 19.51 | 120.00 | 11.80 | 121.50 | 18.47 | 120.00 | 12.55 | 240.00 | 17.58 | 119.99 | 15.05 | | |
| | | 120.08 | 19.62 | 240.17 | 19.64 | 120.08 | 11.65 | 121.58 | 18.49 | 120.08 | 12.61 | 240.17 | 17.52 | 120.08 | 15.15 | | |
| | | 120.17 | 19.65 | 240.33 | 19.53 | 120.16 | 11.74 | 121.66 | 18.61 | 120.16 | 12.54 | 240.33 | 17.58 | 120.16 | 15.20 | | |
| | | 120.25 | 19.68 | 240.50 | 19.51 | 120.25 | 11.73 | 121.75 | 18.62 | 120.25 | 12.53 | 240.50 | 17.62 | 120.24 | 15.20 | | |
| | | 120.33 | 19.65 | 240.67 | 19.60 | 120.33 | 11.84 | 121.83 | 18.53 | 120.33 | 12.61 | 240.67 | 17.65 | 120.33 | 15.21 | | |
| | | 120.42 | 19.64 | 240.83 | 19.57 | 120.41 | 11.81 | 121.91 | 18.51 | 120.41 | 12.55 | 240.83 | 17.76 | 120.41 | 15.20 | | |
| | | 120.50 | 19.65 | 241.00 | 19.51 | 120.50 | 11.91 | 122.00 | 18.68 | 120.50 | 12.53 | 241.00 | 17.91 | 120.49 | 15.19 | | |
| | | 120.58 | 19.68 | 241.17 | 19.59 | 120.58 | 12.01 | 122.08 | 18.70 | 120.58 | 12.62 | 241.17 | 17.87 | 120.58 | 15.20 | | |
| | | 120.67 | 19.71 | 241.33 | 19.58 | 120.66 | 11.77 | 122.16 | 18.70 | 120.66 | 12.57 | 241.33 | 18.03 | 120.66 | 15.32 | | |
| | | 120.75 | 19.72 | 241.50 | 19.60 | 120.75 | 11.84 | 122.25 | 18.72 | 120.75 | 12.54 | 241.50 | 18.01 | 120.74 | 15.31 | | |
| | | 120.83 | 19.69 | 241.67 | 19.73 | 120.83 | 11.84 | 122.33 | 18.63 | 120.83 | 12.55 | 241.67 | 17.93 | 120.83 | 15.37 | | |
| | | 120.92 | 19.66 | 241.83 | 19.72 | 120.91 | 11.97 | 122.41 | 18.56 | 120.91 | 12.54 | 241.83 | 17.96 | 120.91 | 15.32 | | |
| | | 121.00 | 19.67 | 242.00 | 19.78 | 121.00 | 12.02 | 122.50 | 18.74 | 121.00 | 12.54 | 242.00 | 18.00 | 120.99 | 15.32 | | |
| | | 121.08 | 19.74 | 242.17 | 19.74 | 121.08 | 11.78 | 122.58 | 18.55 | 121.08 | 12.61 | 242.17 | 17.96 | 121.08 | 15.28 | | |
| | | 121.17 | 19.75 | 242.33 | 19.77 | 121.16 | 12.00 | 122.66 | 18.71 | 121.16 | 12.53 | 242.33 | 17.96 | 121.16 | 15.27 | | |
| | | 121.25 | 19.77 | 242.50 | 19.82 | 121.25 | 11.87 | 122.75 | 18.74 | 121.25 | 12.53 | 242.50 | 17.98 | 121.24 | 15.30 | | |
| | | 121.33 | 19.70 | 242.67 | 19.88 | 121.33 | 11.93 | 122.83 | 18.71 | 121.33 | 12.63 | 242.67 | 17.99 | 121.33 | 15.31 | | |
| | | 121.42 | 19.72 | 242.83 | 19.94 | 121.41 | 12.09 | 122.91 | 18.82 | 121.41 | 12.54 | 242.83 | 17.99 | 121.41 | 15.29 | | |
| | | 121.50 | 19.75 | 243.00 | 19.90 | 121.50 | 12.11 | 123.00 | 18.78 | 121.50 | 12.55 | 243.00 | 18.06 | 121.49 | 15.38 | | |
| | | 121.58 | 19.76 | 243.17 | 19.92 | 121.58 | 12.07 | 123.08 | 18.84 | 121.58 | 12.57 | 243.17 | 18.09 | 121.58 | 15.29 | | |
| | | 121.67 | 19.72 | 243.33 | 19.81 | 121.66 | 11.94 | 123.16 | 18.85 | 121.66 | 12.55 | 243.33 | 18.12 | 121.66 | 15.29 | | |
| | | 121.75 | 19.76 | 243.50 | 19.82 | 121.75 | 11.94 | 123.25 | 18.86 | 121.75 | 12.52 | 243.50 | 18.13 | 121.74 | 15.31 | | |
| | | 121.83 | 19.75 | 243.67 | 19.85 | 121.83 | 11.99 | 123.33 | 18.92 | 121.83 | 12.64 | 243.67 | 18.15 | 121.83 | 15.33 | | |
| | | 121.92 | 19.72 | 243.83 | 19.83 | 121.91 | 12.04 | 123.41 | 18.95 | 121.91 | 12.55 | 243.83 | 18.21 | 121.91 | 15.37 | | |

| | | | | | | | | | | | | | | | | | |
|---|---|---|---|---|---|---|---|---|---|---|---|---|---|---|---|---|---|
| | | 122.00 | 19.77 | 244.00 | 19.88 | 122.00 | 12.17 | 123.50 | 18.87 | 122.00 | 12.54 | 244.00 | 18.12 | 121.99 | 15.39 | | |
| | | 122.08 | 19.78 | 244.17 | 19.94 | 122.08 | 12.15 | 123.58 | 18.91 | 122.08 | 12.56 | 244.17 | 18.11 | 122.08 | 15.42 | | |
| | | 122.17 | 19.82 | 244.33 | 19.85 | 122.16 | 11.92 | 123.66 | 18.94 | 122.16 | 12.61 | 244.33 | 18.18 | 122.16 | 15.50 | | |
| | | 122.25 | 19.80 | 244.50 | 19.83 | 122.25 | 11.95 | 123.75 | 18.99 | 122.25 | 12.54 | 244.50 | 18.13 | 122.24 | 15.52 | | |
| | | 122.33 | 19.77 | 244.67 | 19.93 | 122.33 | 11.97 | 123.83 | 18.99 | 122.33 | 12.53 | 244.67 | 18.10 | 122.33 | 15.41 | | |
| | | 122.42 | 19.81 | 244.83 | 19.84 | 122.41 | 11.98 | 123.91 | 18.98 | 122.41 | 12.61 | 244.83 | 18.20 | 122.41 | 15.41 | | |
| | | 122.50 | 19.77 | 245.00 | 19.84 | 122.50 | 12.18 | 124.00 | 18.98 | 122.50 | 12.54 | 245.00 | 18.19 | 122.49 | 15.42 | | |
| | | 122.58 | 19.82 | 245.17 | 19.92 | 122.58 | 12.00 | 124.08 | 18.99 | 122.58 | 12.53 | 245.17 | 18.18 | 122.58 | 15.53 | | |
| | | 122.67 | 19.72 | 245.33 | 19.85 | 122.66 | 12.17 | 124.16 | 19.00 | 122.66 | 12.64 | 245.33 | 18.23 | 122.66 | 15.40 | | |
| | | 122.75 | 19.78 | 245.50 | 19.83 | 122.75 | 11.93 | 124.25 | 19.03 | 122.75 | 12.55 | 245.50 | 18.29 | 122.74 | 15.39 | | |
| | | 122.83 | 19.73 | 245.67 | 19.88 | 122.83 | 11.95 | 124.33 | 18.99 | 122.83 | 12.54 | 245.67 | 18.31 | 122.83 | 15.55 | | |
| | | 122.92 | 19.76 | 245.83 | 19.88 | 122.91 | 11.98 | 124.41 | 19.02 | 122.91 | 12.65 | 245.83 | 18.28 | 122.91 | 15.42 | | |
| | | 123.00 | 19.83 | 246.00 | 19.86 | 123.00 | 12.02 | 124.50 | 18.98 | 123.00 | 12.52 | 246.00 | 18.29 | 122.99 | 15.39 | | |
| | | 123.08 | 19.74 | 246.17 | 19.89 | 123.08 | 12.16 | 124.58 | 19.06 | 123.08 | 12.56 | 246.17 | 18.38 | 123.08 | 15.55 | | |
| | | 123.17 | 19.77 | 246.33 | 19.94 | 123.16 | 11.95 | 124.66 | 18.93 | 123.16 | 12.57 | 246.33 | 18.38 | 123.16 | 15.41 | | |
| | | 123.25 | 19.76 | 246.50 | 19.95 | 123.25 | 12.18 | 124.75 | 19.07 | 123.25 | 12.61 | 246.50 | 18.44 | 123.24 | 15.41 | | |
| | | 123.33 | 19.80 | 246.67 | 20.00 | 123.33 | 11.93 | 124.83 | 18.99 | 123.33 | 12.66 | 246.67 | 18.49 | 123.33 | 15.54 | | |
| | | 123.42 | 19.77 | 246.83 | 20.07 | 123.41 | 11.96 | 124.91 | 19.03 | 123.41 | 12.64 | 246.83 | 18.43 | 123.41 | 15.42 | | |
| | | 123.50 | 19.79 | 247.00 | 20.09 | 123.50 | 11.98 | 125.00 | 18.95 | 123.50 | 12.62 | 247.00 | 18.31 | 123.49 | 15.43 | | |
| | | 123.58 | 19.76 | 247.17 | 19.96 | 123.58 | 12.02 | 125.08 | 19.05 | 123.58 | 12.65 | 247.17 | 18.30 | 123.58 | 15.53 | | |
| | | 123.67 | 19.78 | 247.33 | 19.88 | 123.66 | 12.01 | 125.16 | 18.94 | 123.66 | 12.73 | 247.33 | 18.42 | 123.66 | 15.42 | | |
| | | 123.75 | 19.78 | 247.50 | 19.97 | 123.75 | 12.20 | 125.25 | 19.07 | 123.75 | 12.72 | 247.50 | 18.29 | 123.74 | 15.44 | | |
| | | 123.83 | 19.80 | 247.67 | 19.80 | 123.83 | 11.95 | 125.33 | 18.94 | 123.83 | 12.78 | 247.67 | 18.44 | 123.83 | 15.52 | | |
| | | 123.92 | 19.86 | 247.83 | 20.12 | 123.91 | 12.02 | 125.41 | 18.97 | 123.91 | 12.67 | 247.83 | 18.45 | 123.91 | 15.42 | | |
| | | 124.00 | 19.80 | 248.00 | 20.05 | 124.00 | 12.16 | 125.50 | 19.02 | 124.00 | 12.80 | 248.00 | 18.27 | 123.99 | 15.41 | | |
| | | 124.08 | 19.91 | 248.17 | 20.03 | 124.08 | 12.00 | 125.58 | 19.00 | 124.08 | 12.67 | 248.17 | 18.39 | 124.08 | 15.53 | | |
| | | 124.17 | 19.92 | 248.33 | 19.88 | 124.16 | 12.01 | 125.66 | 19.07 | 124.16 | 12.80 | 248.33 | 18.41 | 124.16 | 15.44 | | |
| | | 124.25 | 19.90 | 248.50 | 19.93 | 124.25 | 12.04 | 125.75 | 18.93 | 124.25 | 12.72 | 248.50 | 18.28 | 124.24 | 15.47 | | |
| | | 124.33 | 19.93 | 248.67 | 19.80 | 124.33 | 12.13 | 125.83 | 18.98 | 124.33 | 12.69 | 248.67 | 18.33 | 124.33 | 15.47 | | |
| | | 124.42 | 19.95 | 248.83 | 20.01 | 124.41 | 11.98 | 125.91 | 18.95 | 124.41 | 12.81 | 248.83 | 18.43 | 124.41 | 15.50 | | |
| | | 124.50 | 19.93 | 249.00 | 19.87 | 124.50 | 11.98 | 126.00 | 19.01 | 124.50 | 12.65 | 249.00 | 18.33 | 124.49 | 15.47 | | |
| | | 124.58 | 19.93 | 249.17 | 19.85 | 124.58 | 12.01 | 126.08 | 18.99 | 124.58 | 12.82 | 249.17 | 18.33 | 124.58 | 15.54 | | |
| | | 124.67 | 19.96 | 249.33 | 20.07 | 124.66 | 12.03 | 126.16 | 19.06 | 124.66 | 12.68 | 249.33 | 18.38 | 124.66 | 15.59 | | |
| | | 124.75 | 19.93 | 249.50 | 19.86 | 124.75 | 12.13 | 126.25 | 18.94 | 124.75 | 12.68 | 249.50 | 18.48 | 124.74 | 15.64 | | |
| | | 124.83 | 20.00 | 249.67 | 19.91 | 124.83 | 12.03 | 126.33 | 18.95 | 124.83 | 12.69 | 249.67 | 18.37 | 124.83 | 15.65 | | |
| | | 124.92 | 19.91 | 249.83 | 19.87 | 124.91 | 12.00 | 126.41 | 19.06 | 124.91 | 12.70 | 249.83 | 18.40 | 124.91 | 15.62 | | |
| | | 125.00 | 20.01 | 250.00 | 20.05 | 125.00 | 12.17 | 126.50 | 18.93 | 125.00 | 12.82 | 250.00 | 18.37 | 124.99 | 15.56 | | |
| | | 125.08 | 19.96 | 250.17 | 19.90 | 125.08 | 11.98 | 126.58 | 19.00 | 125.08 | 12.78 | 250.17 | 18.39 | 125.08 | 15.56 | | |
| | | 125.17 | 20.03 | 250.33 | 19.91 | 125.16 | 12.01 | 126.66 | 18.98 | 125.16 | 12.64 | 250.33 | 18.39 | 125.16 | 15.57 | | |
| | | 125.25 | 20.06 | 250.50 | 20.04 | 125.25 | 12.03 | 126.75 | 19.00 | 125.25 | 12.67 | 250.50 | 18.45 | 125.24 | 15.59 | | |
| | | 125.33 | 20.03 | 250.67 | 19.85 | 125.33 | 12.02 | 126.83 | 18.98 | 125.33 | 12.67 | 250.67 | 18.41 | 125.33 | 15.63 | | |
| | | 125.42 | 20.04 | 250.83 | 19.92 | 125.41 | 12.03 | 126.91 | 18.95 | 125.41 | 12.68 | 250.83 | 18.40 | 125.41 | 15.68 | | |
| | | 125.50 | 20.06 | 251.00 | 20.03 | 125.50 | 12.16 | 127.00 | 19.02 | 125.50 | 12.72 | 251.00 | 18.42 | 125.49 | 15.60 | | |
| | | 125.58 | 20.05 | 251.17 | 19.85 | 125.58 | 11.99 | 127.08 | 19.04 | 125.58 | 12.80 | 251.17 | 18.50 | 125.58 | 15.56 | | |
| | | 125.67 | 20.12 | 251.33 | 19.92 | 125.66 | 11.98 | 127.16 | 19.03 | 125.66 | 12.81 | 251.33 | 18.50 | 125.66 | 15.67 | | |
| | | 125.75 | 20.15 | 251.50 | 20.04 | 125.75 | 12.04 | 127.25 | 19.12 | 125.75 | 12.66 | 251.50 | 18.54 | 125.74 | 15.58 | | |
| | | 125.83 | 20.17 | 251.67 | 19.88 | 125.83 | 12.09 | 127.33 | 19.15 | 125.83 | 12.66 | 251.67 | 18.60 | 125.83 | 15.63 | | |
| | | 125.92 | 20.16 | 251.83 | 19.95 | 125.91 | 12.17 | 127.41 | 19.13 | 125.91 | 12.73 | 251.83 | 18.56 | 125.91 | 15.74 | | |
| | | 126.00 | 20.11 | 252.00 | 19.89 | 126.00 | 12.08 | 127.50 | 19.17 | 126.00 | 12.75 | 252.00 | 18.59 | 125.99 | 15.73 | | |
| | | 126.08 | 20.16 | 252.17 | 19.86 | 126.08 | 12.31 | 127.58 | 19.24 | 126.08 | 12.70 | 252.17 | 18.73 | 126.08 | 15.73 | | |
| | | 126.17 | 20.14 | 252.33 | 19.88 | 126.16 | 12.35 | 127.66 | 19.09 | 126.16 | 12.66 | 252.33 | 18.65 | 126.16 | 15.74 | | |

| | | | | | | | | | | | | | | | | | |
|---|---|---|---|---|---|---|---|---|---|---|---|---|---|---|---|---|---|
| | | 126.25 | 20.18 | 252.50 | 19.92 | 126.25 | 12.09 | 127.75 | 19.13 | 126.25 | 12.79 | 252.50 | 18.63 | 126.24 | 15.76 | | |
| | | 126.33 | 20.21 | 252.67 | 19.91 | 126.33 | 12.11 | 127.83 | 19.25 | 126.33 | 12.67 | 252.67 | 18.69 | 126.33 | 15.79 | | |
| | | 126.42 | 20.19 | 252.83 | 20.01 | 126.41 | 12.15 | 127.91 | 19.12 | 126.41 | 12.69 | 252.83 | 18.76 | 126.41 | 15.82 | | |
| | | 126.50 | 20.23 | 253.00 | 19.92 | 126.50 | 12.15 | 128.00 | 19.15 | 126.50 | 12.71 | 253.00 | 18.66 | 126.49 | 15.79 | | |
| | | 126.58 | 20.20 | 253.17 | 19.94 | 126.58 | 12.41 | 128.08 | 19.25 | 126.58 | 12.69 | 253.17 | 18.74 | 126.58 | 15.87 | | |
| | | 126.67 | 20.20 | 253.33 | 19.96 | 126.66 | 12.14 | 128.16 | 19.07 | 126.66 | 12.80 | 253.33 | 18.69 | 126.66 | 15.88 | | |
| | | 126.75 | 20.23 | 253.50 | 19.98 | 126.75 | 12.18 | 128.25 | 19.18 | 126.75 | 12.77 | 253.50 | 18.67 | 126.74 | 15.92 | | |
| | | 126.83 | 20.19 | 253.67 | 20.07 | 126.83 | 12.16 | 128.33 | 19.08 | 126.83 | 12.68 | 253.67 | 18.76 | 126.83 | 15.87 | | |
| | | 126.92 | 20.19 | 253.83 | 20.08 | 126.91 | 12.35 | 128.41 | 19.11 | 126.91 | 12.69 | 253.83 | 18.83 | 126.91 | 15.85 | | |
| | | 127.00 | 20.23 | 254.00 | 20.07 | 127.00 | 12.14 | 128.50 | 19.13 | 127.00 | 12.80 | 254.00 | 18.87 | 126.99 | 15.98 | | |
| | | 127.08 | 20.28 | 254.17 | 20.12 | 127.08 | 12.23 | 128.58 | 19.23 | 127.08 | 12.73 | 254.17 | 18.83 | 127.08 | 15.84 | | |
| | | 127.17 | 20.26 | 254.33 | 20.16 | 127.16 | 12.12 | 128.66 | 19.19 | 127.16 | 12.72 | 254.33 | 18.88 | 127.16 | 15.96 | | |
| | | 127.25 | 20.24 | 254.50 | 20.18 | 127.25 | 12.12 | 128.75 | 19.19 | 127.25 | 12.68 | 254.50 | 18.85 | 127.24 | 15.94 | | |
| | | 127.33 | 20.24 | 254.67 | 20.11 | 127.33 | 12.12 | 128.83 | 19.19 | 127.33 | 12.79 | 254.67 | 18.89 | 127.33 | 15.87 | | |
| | | 127.42 | 20.27 | 254.83 | 20.23 | 127.41 | 12.14 | 128.91 | 19.28 | 127.41 | 12.67 | 254.83 | 18.86 | 127.41 | 15.85 | | |
| | | 127.50 | 20.26 | 255.00 | 20.13 | 127.50 | 12.26 | 129.00 | 19.13 | 127.50 | 12.72 | 255.00 | 18.97 | 127.49 | 15.81 | | |
| | | 127.58 | 20.28 | 255.17 | 20.16 | 127.58 | 12.18 | 129.08 | 19.09 | 127.58 | 12.72 | 255.17 | 19.02 | 127.58 | 15.85 | | |
| | | 127.67 | 20.24 | 255.33 | 20.29 | 127.66 | 12.19 | 129.16 | 19.19 | 127.66 | 12.74 | 255.33 | 19.05 | 127.66 | 15.96 | | |
| | | 127.75 | 20.25 | 255.50 | 20.32 | 127.75 | 12.16 | 129.25 | 19.13 | 127.75 | 12.74 | 255.50 | 18.96 | 127.74 | 15.81 | | |
| | | 127.83 | 20.23 | 255.67 | 20.19 | 127.83 | 12.39 | 129.33 | 19.10 | 127.83 | 12.74 | 255.67 | 18.99 | 127.83 | 15.84 | | |
| | | 127.92 | 20.29 | 255.83 | 20.27 | 127.91 | 12.10 | 129.41 | 19.18 | 127.91 | 12.73 | 255.83 | 18.82 | 127.91 | 15.98 | | |
| | | 128.00 | 20.29 | 256.00 | 20.18 | 128.00 | 12.19 | 129.50 | 19.10 | 128.00 | 12.77 | 256.00 | 18.92 | 127.99 | 15.85 | | |
| | | 128.08 | 20.30 | 256.17 | 20.31 | 128.08 | 12.25 | 129.58 | 19.14 | 128.08 | 12.87 | 256.17 | 18.96 | 128.08 | 15.83 | | |
| | | 128.17 | 20.30 | 256.33 | 20.26 | 128.16 | 12.23 | 129.66 | 19.14 | 128.16 | 12.91 | 256.33 | 18.95 | 128.16 | 15.85 | | |
| | | 128.25 | 20.20 | 256.50 | 20.37 | 128.25 | 12.32 | 129.75 | 19.23 | 128.25 | 12.95 | 256.50 | 18.87 | 128.24 | 15.95 | | |
| | | 128.33 | 20.23 | 256.67 | 20.25 | 128.33 | 12.26 | 129.83 | 19.24 | 128.33 | 12.85 | 256.67 | 18.95 | 128.33 | 15.85 | | |
| | | 128.42 | 20.30 | 256.83 | 20.44 | 128.41 | 12.31 | 129.91 | 19.25 | 128.41 | 12.92 | 256.83 | 18.92 | 128.41 | 15.83 | | |
| | | 128.50 | 20.31 | 257.00 | 20.50 | 128.50 | 12.38 | 130.00 | 19.24 | 128.50 | 12.94 | 257.00 | 18.90 | 128.49 | 15.97 | | |
| | | 128.58 | 20.36 | 257.17 | 20.27 | 128.58 | 12.51 | 130.08 | 19.27 | 128.58 | 12.84 | 257.17 | 18.94 | 128.58 | 15.82 | | |
| | | 128.67 | 20.37 | 257.33 | 20.49 | 128.66 | 12.51 | 130.16 | 19.32 | 128.66 | 12.92 | 257.33 | 19.04 | 128.66 | 15.86 | | |
| | | 128.75 | 20.30 | 257.50 | 20.28 | 128.75 | 12.40 | 130.25 | 19.33 | 128.75 | 12.85 | 257.50 | 18.93 | 128.74 | 15.95 | | |
| | | 128.83 | 20.28 | 257.67 | 20.31 | 128.83 | 12.38 | 130.33 | 19.34 | 128.83 | 12.91 | 257.67 | 18.95 | 128.83 | 15.88 | | |
| | | 128.92 | 20.35 | 257.83 | 20.51 | 128.91 | 12.57 | 130.41 | 19.34 | 128.91 | 12.88 | 257.83 | 18.91 | 128.91 | 15.86 | | |
| | | 129.00 | 20.30 | 258.00 | 20.27 | 129.00 | 12.54 | 130.50 | 19.33 | 129.00 | 12.86 | 258.00 | 19.04 | 128.99 | 15.98 | | |
| | | 129.08 | 20.35 | 258.17 | 20.48 | 129.08 | 12.31 | 130.58 | 19.36 | 129.08 | 12.94 | 258.17 | 18.94 | 129.08 | 15.81 | | |
| | | 129.17 | 20.31 | 258.33 | 20.28 | 129.16 | 12.43 | 130.66 | 19.33 | 129.16 | 12.89 | 258.33 | 18.96 | 129.16 | 15.82 | | |
| | | 129.25 | 20.32 | 258.50 | 20.28 | 129.25 | 12.48 | 130.75 | 19.34 | 129.25 | 12.89 | 258.50 | 18.95 | 129.24 | 15.85 | | |
| | | 129.33 | 20.37 | 258.67 | 20.32 | 129.33 | 12.54 | 130.83 | 19.41 | 129.33 | 12.87 | 258.67 | 18.94 | 129.33 | 15.88 | | |
| | | 129.42 | 20.34 | 258.83 | 20.33 | 129.41 | 12.47 | 130.91 | 19.42 | 129.41 | 12.88 | 258.83 | 18.92 | 129.41 | 15.95 | | |
| | | 129.50 | 20.36 | 259.00 | 20.56 | 129.50 | 12.32 | 131.00 | 19.42 | 129.50 | 12.95 | 259.00 | 18.97 | 129.49 | 15.83 | | |
| | | 129.58 | 20.37 | 259.17 | 20.36 | 129.58 | 12.32 | 131.08 | 19.44 | 129.58 | 12.83 | 259.17 | 18.90 | 129.58 | 15.96 | | |
| | | 129.67 | 20.41 | 259.33 | 20.36 | 129.66 | 12.33 | 131.16 | 19.37 | 129.66 | 12.98 | 259.33 | 18.92 | 129.66 | 15.83 | | |
| | | 129.75 | 20.48 | 259.50 | 20.34 | 129.75 | 12.39 | 131.25 | 19.51 | 129.75 | 12.88 | 259.50 | 19.04 | 129.74 | 15.83 | | |
| | | 129.83 | 20.52 | 259.67 | 20.47 | 129.83 | 12.40 | 131.33 | 19.57 | 129.83 | 12.88 | 259.67 | 18.95 | 129.83 | 15.90 | | |
| | | 129.92 | 20.46 | 259.83 | 20.37 | 129.91 | 12.40 | 131.41 | 19.48 | 129.91 | 12.95 | 259.83 | 18.91 | 129.91 | 15.92 | | |
| | | 130.00 | 20.44 | 260.00 | 20.30 | 130.00 | 12.40 | 131.50 | 19.53 | 130.00 | 12.89 | 260.00 | 18.96 | 129.99 | 15.93 | | |
| | | 130.08 | 20.46 | 260.17 | 20.49 | 130.08 | 12.51 | 131.58 | 19.48 | 130.08 | 12.88 | 260.17 | 19.06 | 130.08 | 15.93 | | |
| | | 130.17 | 20.46 | 260.33 | 20.36 | 130.16 | 12.34 | 131.66 | 19.48 | 130.16 | 12.85 | 260.33 | 18.97 | 130.16 | 15.96 | | |
| | | 130.25 | 20.52 | 260.50 | 20.36 | 130.25 | 12.40 | 131.75 | 19.58 | 130.25 | 12.97 | 260.50 | 19.07 | 130.24 | 16.02 | | |
| | | 130.33 | 20.47 | 260.67 | 20.49 | 130.33 | 12.45 | 131.83 | 19.45 | 130.33 | 12.92 | 260.67 | 19.09 | 130.33 | 16.05 | | |
| | | 130.42 | 20.54 | 260.83 | 20.44 | 130.41 | 12.37 | 131.91 | 19.57 | 130.41 | 12.89 | 260.83 | 19.12 | 130.41 | 16.07 | | |

| | | | | | | | | | | | | | | | | | |
|---|---|---|---|---|---|---|---|---|---|---|---|---|---|---|---|---|---|
| | | 130.50 | 20.48 | 261.00 | 20.50 | 130.50 | 12.35 | 132.00 | 19.49 | 130.50 | 12.90 | 261.00 | 19.05 | 130.49 | 16.07 | | |
| | | 130.58 | 20.48 | 261.17 | 20.44 | 130.58 | 12.39 | 132.08 | 19.49 | 130.58 | 12.93 | 261.17 | 19.15 | 130.58 | 16.06 | | |
| | | 130.67 | 20.45 | 261.33 | 20.51 | 130.66 | 12.51 | 132.16 | 19.56 | 130.66 | 12.96 | 261.33 | 19.16 | 130.66 | 16.09 | | |
| | | 130.75 | 20.48 | 261.50 | 20.59 | 130.75 | 12.33 | 132.25 | 19.44 | 130.75 | 12.92 | 261.50 | 19.09 | 130.74 | 16.05 | | |
| | | 130.83 | 20.46 | 261.67 | 20.58 | 130.83 | 12.36 | 132.33 | 19.57 | 130.83 | 12.98 | 261.67 | 19.24 | 130.83 | 16.16 | | |
| | | 130.92 | 20.44 | 261.83 | 20.65 | 130.91 | 12.40 | 132.41 | 19.45 | 130.91 | 13.03 | 261.83 | 19.28 | 130.91 | 16.19 | | |
| | | 131.00 | 20.55 | 262.00 | 20.58 | 131.00 | 12.49 | 132.50 | 19.58 | 131.00 | 13.08 | 262.00 | 19.21 | 130.99 | 16.22 | | |
| | | 131.08 | 20.44 | 262.17 | 20.56 | 131.08 | 12.44 | 132.58 | 19.51 | 131.08 | 13.07 | 262.17 | 19.19 | 131.08 | 16.13 | | |
| | | 131.17 | 20.45 | 262.33 | 20.48 | 131.16 | 12.34 | 132.66 | 19.50 | 131.16 | 13.01 | 262.33 | 19.25 | 131.16 | 16.19 | | |
| | | 131.25 | 20.48 | 262.50 | 20.55 | 131.25 | 12.34 | 132.75 | 19.47 | 131.25 | 13.11 | 262.50 | 19.17 | 131.24 | 16.11 | | |
| | | 131.33 | 20.45 | 262.67 | 20.62 | 131.33 | 12.51 | 132.83 | 19.58 | 131.33 | 13.00 | 262.67 | 19.21 | 131.33 | 16.18 | | |
| | | 131.42 | 20.46 | 262.83 | 20.62 | 131.41 | 12.44 | 132.91 | 19.43 | 131.41 | 13.11 | 262.83 | 19.20 | 131.41 | 16.25 | | |
| | | 131.50 | 20.52 | 263.00 | 20.70 | 131.50 | 12.52 | 133.00 | 19.44 | 131.50 | 13.09 | 263.00 | 19.31 | 131.49 | 16.30 | | |
| | | 131.58 | 20.44 | 263.17 | 20.68 | 131.58 | 12.61 | 133.08 | 19.55 | 131.58 | 13.04 | 263.17 | 19.15 | 131.58 | 16.33 | | |
| | | 131.67 | 20.48 | 263.33 | 20.78 | 131.66 | 12.65 | 133.16 | 19.51 | 131.66 | 13.01 | 263.33 | 19.30 | 131.66 | 16.35 | | |
| | | 131.75 | 20.51 | 263.50 | 20.65 | 131.75 | 12.66 | 133.25 | 19.51 | 131.75 | 13.01 | 263.50 | 19.22 | 131.74 | 16.35 | | |
| | | 131.83 | 20.56 | 263.67 | 20.74 | 131.83 | 12.48 | 133.33 | 19.56 | 131.83 | 13.12 | 263.67 | 19.15 | 131.83 | 16.22 | | |
| | | 131.92 | 20.54 | 263.83 | 20.69 | 131.91 | 12.49 | 133.41 | 19.47 | 131.91 | 13.08 | 263.83 | 19.32 | 131.91 | 16.23 | | |
| | | 132.00 | 20.64 | 264.00 | 20.63 | 132.00 | 12.51 | 133.50 | 19.47 | 132.00 | 13.09 | 264.00 | 19.15 | 131.99 | 16.28 | | |
| | | 132.08 | 20.60 | 264.17 | 20.66 | 132.08 | 12.69 | 133.58 | 19.45 | 132.08 | 13.02 | 264.17 | 19.21 | 132.08 | 16.29 | | |
| | | 132.17 | 20.60 | 264.33 | 20.65 | 132.16 | 12.54 | 133.66 | 19.59 | 132.16 | 13.02 | 264.33 | 19.20 | 132.16 | 16.39 | | |
| | | 132.25 | 20.59 | 264.50 | 20.72 | 132.25 | 12.50 | 133.75 | 19.53 | 132.25 | 13.02 | 264.50 | 19.33 | 132.24 | 16.25 | | |
| | | 132.33 | 20.58 | 264.67 | 20.65 | 132.33 | 12.72 | 133.83 | 19.47 | 132.33 | 13.09 | 264.67 | 19.27 | 132.33 | 16.39 | | |
| | | 132.42 | 20.62 | 264.83 | 20.67 | 132.41 | 12.69 | 133.91 | 19.44 | 132.41 | 13.06 | 264.83 | 19.20 | 132.41 | 16.37 | | |
| | | 132.50 | 20.67 | 265.00 | 20.71 | 132.50 | 12.46 | 134.00 | 19.59 | 132.50 | 13.05 | 265.00 | 19.31 | 132.49 | 16.23 | | |
| | | 132.58 | 20.70 | 265.17 | 20.72 | 132.58 | 12.64 | 134.08 | 19.47 | 132.58 | 13.05 | 265.17 | 19.16 | 132.58 | 16.27 | | |
| | | 132.67 | 20.63 | 265.33 | 20.69 | 132.66 | 12.75 | 134.16 | 19.45 | 132.66 | 13.03 | 265.33 | 19.17 | 132.66 | 16.26 | | |
| | | 132.75 | 20.68 | 265.50 | 20.84 | 132.75 | 12.53 | 134.25 | 19.53 | 132.75 | 13.08 | 265.50 | 19.18 | 132.74 | 16.26 | | |
| | | 132.83 | 20.64 | 265.67 | 20.77 | 132.83 | 12.51 | 134.33 | 19.47 | 132.83 | 13.01 | 265.67 | 19.23 | 132.83 | 16.27 | | |
| | | 132.92 | 20.70 | 265.83 | 20.72 | 132.91 | 12.51 | 134.41 | 19.56 | 132.91 | 13.04 | 265.83 | 19.18 | 132.91 | 16.39 | | |
| | | 133.00 | 20.64 | 266.00 | 20.91 | 133.00 | 12.51 | 134.50 | 19.49 | 133.00 | 13.12 | 266.00 | 19.32 | 132.99 | 16.25 | | |
| | | 133.08 | 20.64 | 266.17 | 20.95 | 133.08 | 12.74 | 134.58 | 19.46 | 133.08 | 13.02 | 266.17 | 19.26 | 133.08 | 16.26 | | |
| | | 133.17 | 20.71 | 266.33 | 20.73 | 133.16 | 12.55 | 134.66 | 19.53 | 133.16 | 13.03 | 266.33 | 19.28 | 133.16 | 16.38 | | |
| | | 133.25 | 20.67 | 266.50 | 20.77 | 133.25 | 12.58 | 134.75 | 19.53 | 133.25 | 13.11 | 266.50 | 19.18 | 133.24 | 16.26 | | |
| | | 133.33 | 20.60 | 266.67 | 20.95 | 133.33 | 12.71 | 134.83 | 19.57 | 133.33 | 13.04 | 266.67 | 19.27 | 133.33 | 16.30 | | |
| | | 133.42 | 20.60 | 266.83 | 20.71 | 133.41 | 12.70 | 134.91 | 19.55 | 133.41 | 13.02 | 266.83 | 19.29 | 133.41 | 16.39 | | |
| | | 133.50 | 20.72 | 267.00 | 20.93 | 133.50 | 12.48 | 135.00 | 19.57 | 133.50 | 13.12 | 267.00 | 19.19 | 133.49 | 16.24 | | |
| | | 133.58 | 20.65 | 267.17 | 20.74 | 133.58 | 12.53 | 135.08 | 19.58 | 133.58 | 13.04 | 267.17 | 19.17 | 133.58 | 16.38 | | |
| | | 133.67 | 20.66 | 267.33 | 20.75 | 133.66 | 12.51 | 135.16 | 19.74 | 133.66 | 13.03 | 267.33 | 19.25 | 133.66 | 16.22 | | |
| | | 133.75 | 20.65 | 267.50 | 20.77 | 133.75 | 12.54 | 135.25 | 19.73 | 133.75 | 13.12 | 267.50 | 19.29 | 133.74 | 16.25 | | |
| | | 133.83 | 20.69 | 267.67 | 20.81 | 133.83 | 12.70 | 135.33 | 19.59 | 133.83 | 13.04 | 267.67 | 19.23 | 133.83 | 16.27 | | |
| | | 133.92 | 20.66 | 267.83 | 20.93 | 133.91 | 12.56 | 135.41 | 19.66 | 133.91 | 13.06 | 267.83 | 19.32 | 133.91 | 16.36 | | |
| | | 134.00 | 20.68 | 268.00 | 20.75 | 134.00 | 12.71 | 135.50 | 19.77 | 134.00 | 13.05 | 268.00 | 19.30 | 133.99 | 16.28 | | |
| | | 134.08 | 20.67 | 268.17 | 20.97 | 134.08 | 12.50 | 135.58 | 19.61 | 134.08 | 13.15 | 268.17 | 19.30 | 134.08 | 16.28 | | |
| | | 134.17 | 20.73 | 268.33 | 20.85 | 134.16 | 12.50 | 135.66 | 19.61 | 134.16 | 13.18 | 268.33 | 19.32 | 134.16 | 16.39 | | |
| | | 134.25 | 20.74 | 268.50 | 20.77 | 134.25 | 12.57 | 135.75 | 19.69 | 134.25 | 13.24 | 268.50 | 19.37 | 134.24 | 16.27 | | |
| | | 134.33 | 20.76 | 268.67 | 20.71 | 134.33 | 12.55 | 135.83 | 19.71 | 134.33 | 13.26 | 268.67 | 19.38 | 134.33 | 16.28 | | |
| | | 134.42 | 20.79 | 268.83 | 20.78 | 134.41 | 12.69 | 135.91 | 19.75 | 134.41 | 13.18 | 268.83 | 19.35 | 134.41 | 16.30 | | |
| | | 134.50 | 20.73 | 269.00 | 20.83 | 134.50 | 12.52 | 136.00 | 19.68 | 134.50 | 13.17 | 269.00 | 19.42 | 134.49 | 16.35 | | |
| | | 134.58 | 20.71 | 269.17 | 20.80 | 134.58 | 12.52 | 136.08 | 19.76 | 134.58 | 13.28 | 269.17 | 19.33 | 134.58 | 16.29 | | |
| | | 134.67 | 20.79 | 269.33 | 20.93 | 134.66 | 12.73 | 136.16 | 19.81 | 134.66 | 13.21 | 269.33 | 19.36 | 134.66 | 16.27 | | |

| | | | | | | | | | | | | | | | | | |
|---|---|---|---|---|---|---|---|---|---|---|---|---|---|---|---|---|---|
| | | 134.75 | 20.81 | 269.50 | 20.86 | 134.75 | 12.50 | 136.25 | 19.82 | 134.75 | 13.18 | 269.50 | 19.42 | 134.74 | 16.36 | | |
| | | 134.83 | 20.80 | 269.67 | 20.89 | 134.83 | 12.54 | 136.33 | 19.85 | 134.83 | 13.20 | 269.67 | 19.39 | 134.83 | 16.28 | | |
| | | 134.92 | 20.79 | 269.83 | 20.87 | 134.91 | 12.58 | 136.41 | 19.89 | 134.91 | 13.19 | 269.83 | 19.36 | 134.91 | 16.31 | | |
| | | 135.00 | 20.77 | 270.00 | 20.99 | 135.00 | 12.56 | 136.50 | 19.90 | 135.00 | 13.20 | 270.00 | 19.33 | 134.99 | 16.29 | | |
| | | 135.08 | 20.72 | 270.17 | 20.96 | 135.08 | 12.71 | 136.58 | 19.77 | 135.08 | 13.29 | 270.17 | 19.42 | 135.08 | 16.26 | | |
| | | 135.17 | 20.76 | 270.33 | 21.06 | 135.16 | 12.51 | 136.66 | 19.92 | 135.16 | 13.21 | 270.33 | 19.47 | 135.16 | 16.38 | | |
| | | 135.25 | 20.83 | 270.50 | 21.01 | 135.25 | 12.71 | 136.75 | 19.77 | 135.25 | 13.26 | 270.50 | 19.31 | 135.24 | 16.25 | | |
| | | 135.33 | 20.83 | 270.67 | 21.04 | 135.33 | 12.56 | 136.83 | 19.83 | 135.33 | 13.29 | 270.67 | 19.45 | 135.33 | 16.25 | | |
| | | 135.42 | 20.88 | 270.83 | 20.98 | 135.41 | 12.59 | 136.91 | 19.84 | 135.41 | 13.34 | 270.83 | 19.46 | 135.41 | 16.31 | | |
| | | 135.50 | 20.79 | 271.00 | 21.04 | 135.50 | 12.61 | 137.00 | 19.90 | 135.50 | 13.32 | 271.00 | 19.34 | 135.49 | 16.36 | | |
| | | 135.58 | 20.81 | 271.17 | 21.11 | 135.58 | 12.67 | 137.08 | 19.88 | 135.58 | 13.40 | 271.17 | 19.35 | 135.58 | 16.37 | | |
| | | 135.67 | 20.87 | 271.33 | 21.01 | 135.66 | 12.81 | 137.16 | 19.78 | 135.66 | 13.42 | 271.33 | 19.46 | 135.66 | 16.27 | | |
| | | 135.75 | 20.82 | 271.50 | 21.13 | 135.75 | 12.89 | 137.25 | 19.78 | 135.75 | 13.38 | 271.50 | 19.35 | 135.74 | 16.26 | | |
| | | 135.83 | 20.82 | 271.67 | 21.19 | 135.83 | 12.63 | 137.33 | 19.92 | 135.83 | 13.43 | 271.67 | 19.38 | 135.83 | 16.25 | | |
| | | 135.92 | 20.87 | 271.83 | 21.17 | 135.91 | 12.65 | 137.41 | 19.81 | 135.91 | 13.44 | 271.83 | 19.49 | 135.91 | 16.26 | | |
| | | 136.00 | 20.79 | 272.00 | 21.13 | 136.00 | 12.69 | 137.50 | 19.89 | 136.00 | 13.41 | 272.00 | 19.48 | 135.99 | 16.35 | | |
| | | 136.08 | 20.84 | 272.17 | 21.10 | 136.08 | 12.90 | 137.58 | 19.88 | 136.08 | 13.47 | 272.17 | 19.51 | 136.08 | 16.40 | | |
| | | 136.17 | 20.86 | 272.33 | 21.13 | 136.16 | 12.62 | 137.66 | 19.91 | 136.16 | 13.51 | 272.33 | 19.52 | 136.16 | 16.31 | | |
| | | 136.25 | 20.85 | 272.50 | 21.21 | 136.25 | 12.92 | 137.75 | 19.74 | 136.25 | 13.47 | 272.50 | 19.48 | 136.24 | 16.32 | | |
| | | 136.33 | 20.87 | 272.67 | 21.07 | 136.33 | 12.61 | 137.83 | 19.79 | 136.33 | 13.54 | 272.67 | 19.51 | 136.33 | 16.35 | | |
| | | 136.42 | 20.86 | 272.83 | 21.13 | 136.41 | 12.63 | 137.91 | 19.79 | 136.41 | 13.60 | 272.83 | 19.55 | 136.41 | 16.45 | | |
| | | 136.50 | 20.91 | 273.00 | 21.13 | 136.50 | 12.92 | 138.00 | 19.86 | 136.50 | 13.52 | 273.00 | 19.57 | 136.49 | 16.48 | | |
| | | 136.58 | 20.94 | 273.17 | 21.12 | 136.58 | 12.62 | 138.08 | 19.88 | 136.58 | 13.50 | 273.17 | 19.54 | 136.58 | 16.53 | | |
| | | 136.67 | 21.00 | 273.33 | 21.14 | 136.66 | 12.65 | 138.16 | 19.89 | 136.66 | 13.57 | 273.33 | 19.63 | 136.66 | 16.54 | | |
| | | 136.75 | 20.95 | 273.50 | 21.17 | 136.75 | 12.67 | 138.25 | 19.86 | 136.75 | 13.54 | 273.50 | 19.71 | 136.74 | 16.54 | | |
| | | 136.83 | 20.99 | 273.67 | 21.18 | 136.83 | 12.96 | 138.33 | 19.86 | 136.83 | 13.57 | 273.67 | 19.71 | 136.83 | 16.49 | | |
| | | 136.92 | 20.95 | 273.83 | 21.20 | 136.91 | 12.74 | 138.41 | 19.90 | 136.91 | 13.52 | 273.83 | 19.55 | 136.91 | 16.51 | | |
| | | 137.00 | 20.98 | 274.00 | 21.29 | 137.00 | 12.69 | 138.50 | 19.98 | 137.00 | 13.52 | 274.00 | 19.58 | 136.99 | 16.59 | | |
| | | 137.08 | 21.03 | 274.17 | 21.33 | 137.08 | 12.90 | 138.58 | 19.99 | 137.08 | 13.52 | 274.17 | 19.62 | 137.08 | 16.57 | | |
| | | 137.17 | 21.05 | 274.33 | 21.41 | 137.16 | 12.69 | 138.66 | 20.00 | 137.16 | 13.51 | 274.33 | 19.65 | 137.16 | 16.57 | | |
| | | 137.25 | 21.05 | 274.50 | 21.19 | 137.25 | 12.74 | 138.75 | 19.99 | 137.25 | 13.52 | 274.50 | 19.55 | 137.24 | 16.65 | | |
| | | 137.33 | 20.98 | 274.67 | 21.18 | 137.33 | 12.68 | 138.83 | 19.99 | 137.33 | 13.58 | 274.67 | 19.67 | 137.33 | 16.59 | | |
| | | 137.42 | 21.03 | 274.83 | 21.43 | 137.41 | 12.95 | 138.91 | 19.96 | 137.41 | 13.55 | 274.83 | 19.66 | 137.41 | 16.58 | | |
| | | 137.50 | 20.99 | 275.00 | 21.24 | 137.50 | 12.65 | 139.00 | 19.96 | 137.50 | 13.53 | 275.00 | 19.49 | 137.49 | 16.55 | | |
| | | 137.58 | 21.03 | 275.17 | 21.26 | 137.58 | 12.65 | 139.08 | 20.02 | 137.58 | 13.61 | 275.17 | 19.60 | 137.58 | 16.58 | | |
| | | 137.67 | 20.98 | 275.33 | 21.41 | 137.66 | 12.93 | 139.16 | 20.04 | 137.66 | 13.55 | 275.33 | 19.66 | 137.66 | 16.66 | | |
| | | 137.75 | 20.95 | | | 137.7463 | 12.6735 | 139.25 | 19.99 | 137.7457 | 13.6301 | 275.50 | 19.53 | 137.74 | 16.59 | | |
| | | 137.83 | 21.05 | | | 137.8297 | 12.74205 | 139.33 | 20.06 | 137.829 | 13.70353 | 275.67 | 19.57 | 137.83 | 16.62 | | |
| | | 137.92 | 20.96 | | | 137.913 | 12.92495 | 139.41 | 19.93 | 137.9123 | 13.73326 | 275.83 | 19.62 | 137.91 | 16.63 | | |
| | | 138.00 | 20.96 | | | 137.9963 | 12.62525 | 139.50 | 20.11 | 137.9957 | 13.7369 | 276.00 | 19.69 | 137.99 | 16.75 | | |
| | | 138.08 | 21.00 | | | 138.0797 | 12.62917 | 139.58 | 19.92 | 138.079 | 13.62651 | 276.17 | 19.56 | 138.08 | 16.78 | | |
| | | 138.17 | 21.02 | | | 138.163 | 12.66142 | 139.66 | 19.97 | 138.1623 | 13.62951 | 276.33 | 19.66 | 138.16 | 16.65 | | |
| | | 138.25 | 21.03 | | | 138.2463 | 12.68537 | 139.75 | 20.03 | 138.2457 | 13.64358 | 276.50 | 19.60 | 138.24 | 16.67 | | |
| | | 138.33 | 21.00 | | | 138.3297 | 12.71544 | 139.83 | 20.01 | 138.329 | 13.75779 | 276.67 | 19.63 | 138.33 | 16.81 | | |
| | | 138.42 | 20.99 | | | 138.413 | 12.95037 | 139.91 | 19.95 | 138.4123 | 13.69122 | 276.83 | 19.63 | 138.41 | 16.65 | | |
| | | 138.50 | 20.97 | | | 138.4963 | 12.73395 | 140.00 | 19.93 | 138.4957 | 13.65984 | 277.00 | 19.62 | 138.49 | 16.67 | | |
| | | 138.58 | 20.99 | | | 138.5797 | 12.69466 | 140.08 | 20.09 | 138.579 | 13.78345 | 277.17 | 19.72 | 138.58 | 16.81 | | |
| | | 138.67 | 20.98 | | | 138.663 | 12.92867 | 140.16 | 19.93 | 138.6623 | 13.64516 | 277.33 | 19.64 | 138.66 | 16.68 | | |
| | | 138.75 | 21.01 | | | 138.7463 | 12.71854 | 140.25 | 20.00 | 138.7457 | 13.64329 | 277.50 | 19.63 | 138.74 | 16.66 | | |
| | | 138.83 | 21.01 | | | 138.8297 | 12.81023 | 140.33 | 20.07 | 138.829 | 13.76083 | 277.67 | 19.57 | 138.83 | 16.80 | | |
| | | 138.92 | 21.05 | | | 138.913 | 12.67897 | 140.41 | 19.98 | 138.9123 | 13.64581 | 277.83 | 19.64 | 138.91 | 16.66 | | |

| | | | | | | | | | | | | | | | | | |
|---|---|---|---|---|---|---|---|---|---|---|---|---|---|---|---|---|---|
| | | 139.00 | 21.05 | | | 138.9963 | 12.68359 | 140.50 | 20.07 | 138.9957 | 13.64541 | 278.00 | 19.67 | 138.99 | 16.73 | | |
| | | 139.08 | 21.06 | | | 139.0797 | 12.6565 | 140.58 | 19.92 | 139.079 | 13.78782 | 278.17 | 19.66 | 139.08 | 16.69 | | |
| | | 139.17 | 21.17 | | | 139.163 | 12.93745 | 140.66 | 20.11 | 139.1623 | 13.66841 | 278.33 | 19.72 | 139.16 | 16.69 | | |
| | | 139.25 | 21.11 | | | 139.2463 | 12.68225 | 140.75 | 20.01 | 139.2457 | 13.77389 | 278.50 | 19.77 | 139.24 | 16.81 | | |
| | | 139.33 | 21.18 | | | 139.3297 | 12.74545 | 140.83 | 20.05 | 139.329 | 13.62107 | 278.67 | 19.82 | 139.33 | 16.68 | | |
| | | 139.42 | 21.19 | | | 139.413 | 12.90239 | 140.91 | 20.05 | 139.4123 | 13.63619 | 278.83 | 19.79 | 139.41 | 16.68 | | |
| | | 139.50 | 21.24 | | | 139.4963 | 12.70555 | 141.00 | 20.05 | 139.4957 | 13.77749 | 279.00 | 19.88 | 139.49 | 16.69 | | |
| | | 139.58 | 21.21 | | | 139.5797 | 12.67549 | 141.08 | 19.95 | 139.579 | 13.69903 | 279.17 | 19.95 | 139.58 | 16.71 | | |
| | | 139.67 | 21.15 | | | 139.663 | 12.90988 | 141.16 | 19.94 | 139.6623 | 13.74084 | 279.33 | 20.00 | 139.66 | 16.81 | | |
| | | 139.75 | 21.17 | | | 139.7463 | 12.68089 | 141.25 | 19.95 | 139.7457 | 13.63699 | 279.50 | 19.96 | 139.74 | 16.67 | | |
| | | 139.83 | 21.17 | | | 139.8297 | 12.70294 | 141.33 | 20.11 | 139.829 | 13.62999 | 279.67 | 20.06 | 139.83 | 16.68 | | |
| | | 139.92 | 21.23 | | | 139.913 | 12.70164 | 141.41 | 20.02 | 139.9123 | 13.64167 | 279.83 | 20.07 | 139.91 | 16.81 | | |
| | | 140.00 | 21.25 | | | 139.9963 | 12.74411 | 141.50 | 20.02 | 139.9957 | 13.75125 | 280.00 | 20.01 | 139.99 | 16.73 | | |
| | | 140.08 | 21.20 | | | 140.0797 | 12.73002 | 141.58 | 19.94 | 140.079 | 13.70481 | 280.17 | 20.04 | 140.08 | 16.75 | | |
| | | 140.17 | 21.18 | | | 140.163 | 12.88393 | 141.66 | 19.94 | 140.1623 | 13.68227 | 280.33 | 20.08 | 140.16 | 16.75 | | |
| | | 140.25 | 21.19 | | | 140.2463 | 12.72311 | 141.75 | 20.10 | 140.2457 | 13.63633 | 280.50 | 20.03 | 140.24 | 16.77 | | |
| | | 140.33 | 21.23 | | | 140.3297 | 12.69198 | 141.83 | 19.93 | 140.329 | 13.76968 | 280.67 | 19.97 | 140.33 | 16.81 | | |
| | | 140.42 | 21.24 | | | 140.413 | 12.9149 | 141.91 | 20.02 | 140.4123 | 13.64546 | 280.83 | 20.06 | 140.41 | 16.79 | | |
| | | 140.50 | 21.22 | | | 140.4963 | 12.81694 | 142.00 | 20.01 | 140.4957 | 13.62954 | 281.00 | 20.07 | 140.49 | 16.87 | | |
| | | 140.58 | 21.22 | | | 140.5797 | 12.89069 | 142.08 | 20.03 | 140.579 | 13.66781 | 281.17 | 19.99 | 140.58 | 16.92 | | |
| | | 140.67 | 21.23 | | | 140.663 | 12.8977 | 142.16 | 20.01 | 140.6623 | 13.68014 | 281.33 | 20.08 | 140.66 | 16.85 | | |
| | | 140.75 | 21.29 | | | 140.7463 | 12.91813 | 142.25 | 20.07 | 140.7457 | 13.77303 | 281.50 | 20.06 | 140.74 | 16.93 | | |
| | | 140.83 | 21.33 | | | 140.8297 | 12.75461 | 142.33 | 20.14 | 140.829 | 13.67198 | 281.67 | 19.99 | 140.83 | 16.94 | | |
| | | 140.92 | 21.32 | | | 140.913 | 12.68699 | 142.41 | 20.17 | 140.9123 | 13.65039 | 281.83 | 20.04 | 140.91 | 16.80 | | |
| | | 141.00 | 21.34 | | | 140.9963 | 12.7497 | 142.50 | 20.19 | 140.9957 | 13.79927 | 282.00 | 20.07 | 140.99 | 16.83 | | |
| | | 141.08 | 21.34 | | | 141.0797 | 12.84018 | 142.58 | 20.24 | 141.079 | 13.64835 | 282.17 | 19.99 | 141.08 | 16.93 | | |
| | | 141.17 | 21.33 | | | 141.163 | 12.87992 | 142.66 | 20.23 | 141.1623 | 13.683 | 282.33 | 20.02 | 141.16 | 16.88 | | |
| | | 141.25 | 21.32 | | | 141.2463 | 12.95039 | 142.75 | 20.10 | 141.2457 | 13.74283 | 282.50 | 20.08 | 141.24 | 16.93 | | |
| | | 141.33 | 21.30 | | | 141.3297 | 13.00503 | 142.83 | 20.11 | 141.329 | 13.75397 | 282.67 | 20.06 | 141.33 | 16.84 | | |
| | | | | | | 141.413 | 13.03047 | 142.91 | 20.23 | 141.4123 | 13.7341 | 282.83 | 20.06 | 141.41 | 16.82 | | |
| | | | | | | 141.4963 | 12.85011 | 143.00 | 20.12 | 141.4957 | 13.63456 | 283.00 | 20.14 | 141.49 | 16.83 | | |
| | | | | | | 141.5797 | 12.87607 | 143.08 | 20.14 | 141.579 | 13.64503 | 283.17 | 20.16 | 141.58 | 16.93 | | |
| | | | | | | 141.663 | 12.89206 | 143.16 | 20.27 | 141.6623 | 13.67502 | 283.33 | 20.07 | 141.66 | 16.92 | | |
| | | | | | | 141.7463 | 12.9817 | 143.25 | 20.08 | 141.7457 | 13.74924 | 283.50 | 20.16 | 141.74 | 16.96 | | |
| | | | | | | 141.8297 | 12.92669 | 143.33 | 20.27 | 141.829 | 13.75194 | 283.67 | 20.22 | 141.83 | 16.96 | | |
| | | | | | | 141.913 | 13.07007 | 143.41 | 20.10 | 141.9123 | 13.68555 | 283.83 | 20.25 | 141.91 | 16.94 | | |
| | | | | | | 141.9963 | 12.84732 | 143.50 | 20.09 | 141.9957 | 13.66796 | 284.00 | 20.19 | 141.99 | 16.96 | | |
| | | | | | | 142.0797 | 12.90128 | 143.58 | 20.19 | 142.079 | 13.67761 | 284.17 | 20.19 | 142.08 | 17.00 | | |
| | | | | | | 142.163 | 12.9128 | 143.66 | 20.20 | 142.1623 | 13.6774 | 284.33 | 20.21 | 142.16 | 17.05 | | |
| | | | | | | 142.2463 | 12.94405 | 143.75 | 20.09 | 142.2457 | 13.68954 | 284.50 | 20.21 | 142.24 | 17.06 | | |
| | | | | | | 142.3297 | 12.93598 | 143.83 | 20.09 | 142.329 | 13.75696 | 284.67 | 20.32 | 142.33 | 16.98 | | |
| | | | | | | 142.413 | 13.0369 | 143.91 | 20.28 | 142.4123 | 13.76561 | 284.83 | 20.34 | 142.41 | 16.97 | | |
| | | | | | | 142.4963 | 12.87743 | 144.00 | 20.18 | 142.4957 | 13.7394 | 285.00 | 20.33 | 142.49 | 17.07 | | |
| | | | | | | 142.5797 | 13.06666 | 144.08 | 20.19 | 142.579 | 13.76627 | 285.17 | 20.37 | 142.58 | 17.07 | | |
| | | | | | | 142.663 | 12.92429 | 144.16 | 20.28 | 142.6623 | 13.80758 | 285.33 | 20.27 | 142.66 | 17.00 | | |
| | | | | | | 142.7463 | 12.95099 | 144.25 | 20.29 | 142.7457 | 13.86524 | 285.50 | 20.33 | 142.74 | 17.06 | | |
| | | | | | | 142.8297 | 13.03344 | 144.33 | 20.24 | 142.829 | 13.9146 | 285.67 | 20.28 | 142.83 | 17.01 | | |
| | | | | | | 142.913 | 13.1312 | 144.41 | 20.15 | 142.9123 | 13.90059 | 285.83 | 20.27 | 142.91 | 17.05 | | |
| | | | | | | 142.9963 | 13.17333 | 144.50 | 20.10 | 142.9957 | 13.91206 | 286.00 | 20.33 | 142.99 | 17.16 | | |
| | | | | | | 143.0797 | 13.05955 | 144.58 | 20.14 | 143.079 | 13.92976 | 286.17 | 20.30 | 143.08 | 17.19 | | |
| | | | | | | 143.163 | 13.20959 | 144.66 | 20.13 | 143.1623 | 13.87079 | 286.33 | 20.33 | 143.16 | 17.19 | | |

| | | | | | | | | | | | | | | | | | |
|---|---|---|---|---|---|---|---|---|---|---|---|---|---|---|---|---|---|
| | | | | | | 143.2463 | 13.0313 | 144.75 | 20.17 | 143.2457 | 13.96176 | 286.50 | 20.43 | 143.24 | 17.12 | | |
| | | | | | | 143.3297 | 13.23934 | 144.83 | 20.26 | 143.329 | 14.02232 | 286.67 | 20.29 | 143.33 | 17.08 | | |
| | | | | | | 143.413 | 13.05476 | 144.91 | 20.14 | 143.4123 | 14.06633 | 286.83 | 20.31 | 143.41 | 17.11 | | |
| | | | | | | 143.4963 | 13.08482 | 145.00 | 20.11 | 143.4957 | 14.05602 | 287.00 | 20.42 | 143.49 | 17.24 | | |
| | | | | | | 143.5797 | 13.08714 | 145.08 | 20.14 | 143.579 | 14.04427 | 287.17 | 20.39 | 143.58 | 17.08 | | |
| | | | | | | 143.663 | 13.2732 | 145.16 | 20.20 | 143.6623 | 13.97741 | 287.33 | 20.29 | 143.66 | 17.10 | | |
| | | | | | | 143.7463 | 13.03408 | 145.25 | 20.17 | 143.7457 | 14.04846 | 287.50 | 20.36 | 143.74 | 17.11 | | |
| | | | | | | 143.8297 | 13.05799 | 145.33 | 20.18 | 143.829 | 14.00412 | 287.67 | 20.45 | 143.83 | 17.11 | | |
| | | | | | | 143.913 | 13.07118 | 145.41 | 20.14 | 143.9123 | 13.9822 | 287.83 | 20.29 | 143.91 | 17.25 | | |
| | | | | | | 143.9963 | 13.25249 | 145.50 | 20.11 | 143.9957 | 14.04803 | 288.00 | 20.31 | 143.99 | 17.11 | | |
| | | | | | | 144.0797 | 13.04747 | 145.58 | 20.28 | 144.079 | 14.02498 | 288.17 | 20.40 | 144.08 | 17.09 | | |
| | | | | | | 144.163 | 13.06271 | 145.66 | 20.09 | 144.1623 | 14.04399 | 288.33 | 20.42 | 144.16 | 17.25 | | |
| | | | | | | 144.2463 | 13.28911 | 145.75 | 20.17 | 144.2457 | 14.04419 | 288.50 | 20.41 | 144.24 | 17.22 | | |
| | | | | | | 144.3297 | 13.02662 | 145.83 | 20.22 | 144.329 | 14.02823 | 288.67 | 20.40 | 144.33 | 17.07 | | |
| | | | | | | 144.413 | 13.05717 | 145.91 | 20.10 | 144.4123 | 14.12348 | 288.83 | 20.50 | 144.41 | 17.10 | | |
| | | | | | | 144.4963 | 13.10724 | 146.00 | 20.16 | 144.4957 | 14.22929 | 289.00 | 20.51 | 144.49 | 17.12 | | |
| | | | | | | 144.5797 | 13.09993 | 146.08 | 20.14 | 144.579 | 14.245 | 289.17 | 20.44 | 144.58 | 17.14 | | |
| | | | | | | 144.663 | 13.24367 | 146.16 | 20.28 | 144.6623 | 14.14876 | 289.33 | 20.54 | 144.66 | 17.23 | | |
| | | | | | | 144.7463 | 13.25732 | 146.25 | 20.19 | 144.7457 | 14.24521 | 289.50 | 20.39 | 144.74 | 17.11 | | |
| | | | | | | 144.8297 | 13.07416 | 146.33 | 20.26 | 144.829 | 14.23434 | 289.67 | 20.44 | 144.83 | 17.24 | | |
| | | | | | | 144.913 | 13.04253 | 146.41 | 20.17 | 144.9123 | 14.08815 | 289.83 | 20.40 | 144.91 | 17.08 | | |
| | | | | | | 144.9963 | 13.08129 | 146.50 | 20.24 | 144.9957 | 14.09363 | 290.00 | 20.56 | 144.99 | 17.10 | | |
| | | | | | | 145.0797 | 13.09148 | 146.58 | 20.24 | 145.079 | 14.12072 | 290.17 | 20.42 | 145.08 | 17.12 | | |
| | | | | | | 145.163 | 13.10113 | 146.66 | 20.24 | 145.1623 | 14.16699 | 290.33 | 20.43 | 145.16 | 17.22 | | |
| | | | | | | 145.2463 | 13.27971 | 146.75 | 20.28 | 145.2457 | 14.28426 | 290.50 | 20.44 | 145.24 | 17.16 | | |
| | | | | | | 145.3297 | 13.26241 | 146.83 | 20.34 | 145.329 | 14.10955 | 290.67 | 20.49 | 145.33 | 17.10 | | |
| | | | | | | 145.413 | 13.05043 | 146.91 | 20.36 | 145.4123 | 14.12256 | 290.83 | 20.55 | 145.41 | 17.09 | | |
| | | | | | | 145.4963 | 13.08104 | 147.00 | 20.39 | 145.4957 | 14.26409 | 291.00 | 20.50 | 145.49 | 17.11 | | |
| | | | | | | 145.5797 | 13.06261 | 147.08 | 20.32 | 145.579 | 14.125 | 291.17 | 20.49 | 145.58 | 17.21 | | |
| | | | | | | 145.663 | 13.25806 | 147.16 | 20.44 | 145.6623 | 14.16352 | 291.33 | 20.31 | 145.66 | 17.16 | | |
| | | | | | | 145.7463 | 13.10961 | 147.25 | 20.30 | 145.7457 | 14.27123 | 291.50 | 20.39 | 145.74 | 17.17 | | |
| | | | | | | 145.8297 | 13.05513 | 147.33 | 20.44 | 145.829 | 14.11467 | 291.67 | 20.47 | 145.83 | 17.19 | | |
| | | | | | | 145.913 | 13.2563 | 147.41 | 20.37 | 145.9123 | 14.27942 | 291.83 | 20.37 | 145.91 | 17.16 | | |
| | | | | | | 145.9963 | 13.11144 | 147.50 | 20.38 | 145.9957 | 14.11792 | 292.00 | 20.36 | 145.99 | 17.11 | | |
| | | | | | | 146.0797 | 13.14054 | 147.58 | 20.32 | 146.079 | 14.12664 | 292.17 | 20.49 | 146.08 | 17.10 | | |
| | | | | | | 146.163 | 13.11173 | 147.66 | 20.34 | 146.1623 | 14.26152 | 292.33 | 20.39 | 146.16 | 17.15 | | |
| | | | | | | 146.2463 | 13.07823 | 147.75 | 20.28 | 146.2457 | 14.14784 | 292.50 | 20.38 | 146.24 | 17.17 | | |
| | | | | | | 146.3297 | 13.06335 | 147.83 | 20.28 | 146.329 | 14.14212 | 292.67 | 20.46 | 146.33 | 17.17 | | |
| | | | | | | 146.413 | 13.24588 | 147.91 | 20.39 | 146.4123 | 14.2585 | 292.83 | 20.38 | 146.41 | 17.19 | | |
| | | | | | | 146.4963 | 13.06817 | 148.00 | 20.29 | 146.4957 | 14.11496 | 293.00 | 20.39 | 146.49 | 17.19 | | |
| | | | | | | 146.5797 | 13.05668 | 148.08 | 20.31 | 146.579 | 14.268 | 293.17 | 20.47 | 146.58 | 17.16 | | |
| | | | | | | 146.663 | 13.08477 | 148.16 | 20.36 | 146.6623 | 14.0967 | 293.33 | 20.42 | 146.66 | 17.29 | | |
| | | | | | | 146.7463 | 13.25119 | 148.25 | 20.33 | 146.7457 | 14.12986 | 293.50 | 20.55 | 146.74 | 17.30 | | |
| | | | | | | 146.8297 | 13.04045 | 148.33 | 20.31 | 146.829 | 14.16871 | 293.67 | 20.49 | 146.83 | 17.36 | | |
| | | | | | | 146.913 | 13.06037 | 148.41 | 20.39 | 146.9123 | 14.26634 | 293.83 | 20.50 | | | | |
| | | | | | | 146.9963 | 13.11814 | 148.50 | 20.34 | 146.9957 | 14.123 | 294.00 | 20.34 | | | | |
| | | | | | | 147.0797 | 13.27626 | 148.58 | 20.42 | 147.079 | 14.26101 | 294.17 | 20.47 | | | | |
| | | | | | | 147.163 | 13.04309 | 148.66 | 20.35 | 147.1623 | 14.11549 | 294.33 | 20.48 | | | | |
| | | | | | | 147.2463 | 13.08282 | 148.75 | 20.34 | 147.2457 | 14.16785 | 294.50 | 20.47 | | | | |
| | | | | | | 147.3297 | 13.13515 | 148.83 | 20.42 | 147.329 | 14.16563 | 294.67 | 20.53 | | | | |
| | | | | | | 147.413 | 13.15686 | 148.91 | 20.44 | 147.4123 | 14.1864 | 294.83 | 20.56 | | | | |

| | | | | | | | | | | | | | | | | | |
|---|---|---|---|---|---|---|---|---|---|---|---|---|---|---|---|---|---|
| | | | | | | 147.4963 | 13.13165 | 149.00 | 20.41 | 147.4957 | 14.12004 | 295.00 | 20.56 | | | | |
| | | | | | | 147.5797 | 13.10023 | 149.08 | 20.44 | 147.579 | 14.28649 | 295.17 | 18.64 | | | | |
| | | | | | | 147.663 | 13.12763 | 149.16 | 20.47 | 147.6623 | 14.13372 | 295.33 | 20.58 | | | | |
| | | | | | | 147.7463 | 13.07877 | 149.25 | 20.47 | 147.7457 | 14.14613 | 295.50 | 20.64 | | | | |
| | | | | | | 147.8297 | 13.06983 | 149.33 | 20.52 | 147.829 | 14.13987 | 295.67 | 20.63 | | | | |
| | | | | | | 147.913 | 13.11819 | 149.41 | 20.60 | 147.9123 | 14.27218 | 295.83 | 20.64 | | | | |
| | | | | | | 147.9963 | 13.14665 | 149.50 | 20.60 | 147.9957 | 14.1428 | 296.00 | 20.56 | | | | |
| | | | | | | 148.0797 | 13.15465 | 149.58 | 20.50 | 148.079 | 14.23739 | 296.17 | 20.57 | | | | |
| | | | | | | 148.163 | 13.05484 | 149.66 | 20.49 | 148.1623 | 14.10978 | 296.33 | 20.62 | | | | |
| | | | | | | 148.2463 | 13.05943 | 149.75 | 20.61 | 148.2457 | 14.265 | 296.50 | 20.59 | | | | |
| | | | | | | 148.3297 | 13.09308 | 149.83 | 20.45 | 148.329 | 14.12758 | 296.67 | 20.60 | | | | |
| | | | | | | 148.413 | 13.1471 | 149.91 | 20.60 | 148.4123 | 14.11284 | 296.83 | 20.58 | | | | |
| | | | | | | 148.4963 | 13.15588 | 150.00 | 20.43 | 148.4957 | 14.17623 | 297.00 | 20.68 | | | | |
| | | | | | | 148.5797 | 13.14773 | 150.08 | 20.53 | 148.579 | 14.25032 | 297.17 | 20.58 | | | | |
| | | | | | | 148.663 | 13.22081 | 150.16 | 20.49 | 148.6623 | 14.11591 | | | | | | |
| | | | | | | 148.7463 | 13.43598 | 150.25 | 20.60 | 148.7457 | 14.27386 | | | | | | |
| | | | | | | 148.8297 | 13.44877 | 150.33 | 20.49 | 148.829 | 14.11701 | | | | | | |
| | | | | | | 148.913 | 13.16513 | 150.41 | 20.58 | 148.9123 | 14.21693 | | | | | | |
| | | | | | | 148.9963 | 13.23172 | 150.50 | 20.46 | 148.9957 | 14.2573 | | | | | | |
| | | | | | | 149.0797 | 13.32153 | 150.58 | 20.51 | 149.079 | 14.25541 | | | | | | |
| | | | | | | 149.163 | 13.48377 | 150.66 | 20.56 | 149.1623 | 14.17968 | | | | | | |
| | | | | | | 149.2463 | 13.45773 | 150.75 | 20.48 | 149.2457 | 14.10798 | | | | | | |
| | | | | | | 149.3297 | 13.49609 | 150.83 | 20.59 | 149.329 | 14.21818 | | | | | | |
| | | | | | | 149.413 | 13.57876 | 150.91 | 20.46 | 149.4123 | 14.135 | | | | | | |
| | | | | | | 149.4963 | 13.4391 | 151.00 | 20.58 | 149.4957 | 14.19432 | | | | | | |
| | | | | | | 149.5797 | 13.4426 | 151.08 | 20.49 | 149.579 | 14.25868 | | | | | | |
| | | | | | | 149.663 | 13.4587 | 151.16 | 20.47 | 149.6623 | 14.27133 | | | | | | |
| | | | | | | 149.7463 | 13.59755 | 151.25 | 20.57 | 149.7457 | 14.22192 | | | | | | |
| | | | | | | 149.8297 | 13.45091 | 151.33 | 20.50 | 149.829 | 14.25339 | | | | | | |
| | | | | | | 149.913 | 13.43634 | 151.41 | 20.60 | 149.9123 | 14.31717 | | | | | | |
| | | | | | | 149.9963 | 13.63649 | 151.50 | 20.48 | 149.9957 | 14.37241 | | | | | | |
| | | | | | | 150.0797 | 13.50534 | 151.58 | 20.48 | 150.079 | 14.36513 | | | | | | |
| | | | | | | 150.163 | 13.60387 | 151.66 | 20.59 | 150.1623 | 14.39058 | | | | | | |
| | | | | | | 150.2463 | 13.68372 | 151.75 | 20.50 | 150.2457 | 14.38833 | | | | | | |
| | | | | | | 150.3297 | 13.7439 | 151.83 | 20.61 | 150.329 | 14.37111 | | | | | | |
| | | | | | | 150.413 | 13.72232 | 151.91 | 20.59 | 150.4123 | 14.32871 | | | | | | |
| | | | | | | 150.4963 | 13.58278 | 152.00 | 20.48 | 150.4957 | 14.2988 | | | | | | |
| | | | | | | 150.5797 | 13.59852 | 152.08 | 20.56 | 150.579 | 14.31412 | | | | | | |
| | | | | | | 150.663 | 13.60789 | 152.16 | 20.46 | 150.6623 | 14.35375 | | | | | | |
| | | | | | | 150.7463 | 13.78974 | 152.25 | 20.53 | 150.7457 | 14.37932 | | | | | | |
| | | | | | | 150.8297 | 13.60715 | 152.33 | 20.53 | 150.829 | 14.34235 | | | | | | |
| | | | | | | 150.913 | 13.67193 | 152.41 | 20.48 | 150.9123 | 14.30865 | | | | | | |
| | | | | | | 150.9963 | 13.82944 | 152.50 | 20.47 | 150.9957 | 14.40215 | | | | | | |
| | | | | | | 151.0797 | 13.57583 | 152.58 | 20.54 | 151.079 | 14.31729 | | | | | | |
| | | | | | | 151.163 | 13.60424 | 152.66 | 20.46 | 151.1623 | 14.38932 | | | | | | |
| | | | | | | 151.2463 | 13.79217 | 152.75 | 20.52 | 151.2457 | 14.43451 | | | | | | |
| | | | | | | 151.3297 | 13.62909 | 152.83 | 20.51 | 151.329 | 14.44214 | | | | | | |
| | | | | | | 151.413 | 13.62951 | 152.91 | 20.50 | 151.4123 | 14.46608 | | | | | | |
| | | | | | | 151.4963 | 13.79937 | 153.00 | 20.56 | 151.4957 | 14.38116 | | | | | | |
| | | | | | | 151.5797 | 13.78251 | 153.08 | 20.56 | 151.579 | 14.47005 | | | | | | |
| | | | | | | 151.663 | 13.60337 | 153.16 | 20.45 | 151.6623 | 14.52393 | | | | | | |

| | | | | | | | | | | | | | | | | | |
|---|---|---|---|---|---|---|---|---|---|---|---|---|---|---|---|---|---|
| | | | | | | 151.7463 | 13.62911 | 153.25 | 20.48 | 151.7457 | 14.55703 | | | | | | |
| | | | | | | 151.8297 | 13.65124 | 153.33 | 20.57 | 151.829 | 14.5408 | | | | | | |
| | | | | | | 151.913 | 13.63569 | 153.41 | 20.53 | 151.9123 | 14.55439 | | | | | | |
| | | | | | | 151.9963 | 13.65607 | 153.50 | 20.52 | 151.9957 | 14.52704 | | | | | | |
| | | | | | | 152.0797 | 13.82628 | 153.58 | 20.45 | 152.079 | 14.50714 | | | | | | |
| | | | | | | 152.163 | 13.59004 | 153.66 | 20.45 | 152.1623 | 14.50091 | | | | | | |
| | | | | | | 152.2463 | 13.80478 | 153.75 | 20.45 | 152.2457 | 14.47045 | | | | | | |
| | | | | | | 152.3297 | 13.70997 | 153.83 | 20.59 | 152.329 | 14.52225 | | | | | | |
| | | | | | | 152.413 | 13.69586 | 153.91 | 20.58 | 152.4123 | 14.55615 | | | | | | |
| | | | | | | 152.4963 | 13.90995 | 154.00 | 20.60 | 152.4957 | 14.55659 | | | | | | |
| | | | | | | 152.5797 | 13.96717 | 154.08 | 20.48 | 152.579 | 14.53641 | | | | | | |
| | | | | | | 152.663 | 13.73247 | 154.16 | 20.47 | 152.6623 | 14.52578 | | | | | | |
| | | | | | | 152.7463 | 13.98549 | 154.25 | 20.45 | 152.7457 | 14.54663 | | | | | | |
| | | | | | | 152.8297 | 13.69955 | 154.33 | 20.48 | 152.829 | 14.66645 | | | | | | |
| | | | | | | 152.913 | 13.72831 | 154.41 | 20.60 | 152.9123 | 14.72 | | | | | | |
| | | | | | | 152.9963 | 14.01458 | 154.50 | 20.57 | 152.9957 | 14.73829 | | | | | | |
| | | | | | | 153.0797 | 13.77928 | 154.58 | 20.52 | 153.079 | 14.62002 | | | | | | |
| | | | | | | 153.163 | 13.76496 | 154.66 | 20.57 | 153.1623 | 14.71808 | | | | | | |
| | | | | | | 153.2463 | 14.05345 | 154.75 | 20.53 | 153.2457 | 14.59822 | | | | | | |
| | | | | | | 153.3297 | 13.76047 | 154.83 | 20.63 | 153.329 | 14.75128 | | | | | | |
| | | | | | | 153.413 | 13.76698 | 154.91 | 20.69 | 153.4123 | 14.57732 | | | | | | |
| | | | | | | 153.4963 | 13.79172 | 155.00 | 20.74 | 153.4957 | 14.64678 | | | | | | |
| | | | | | | 153.5797 | 13.96481 | 155.08 | 20.71 | 153.579 | 14.63992 | | | | | | |
| | | | | | | 153.663 | 13.76351 | 155.16 | 20.75 | 153.6623 | 14.74271 | | | | | | |
| | | | | | | 153.7463 | 14.00104 | 155.25 | 20.69 | 153.7457 | 14.60332 | | | | | | |
| | | | | | | 153.8297 | 13.68867 | 155.33 | 20.80 | 153.829 | 14.76266 | | | | | | |
| | | | | | | 153.913 | 13.73483 | 155.41 | 20.86 | 153.9123 | 14.58399 | | | | | | |
| | | | | | | 153.9963 | 14.0177 | 155.50 | 20.90 | 153.9957 | 14.65908 | | | | | | |
| | | | | | | 154.0797 | 13.70677 | 155.58 | 20.91 | 154.079 | 14.6442 | | | | | | |
| | | | | | | 154.163 | 13.75989 | 155.66 | 20.93 | 154.1623 | 14.7552 | | | | | | |
| | | | | | | 154.2463 | 13.78462 | 155.75 | 20.90 | 154.2457 | 14.75997 | | | | | | |
| | | | | | | 154.3297 | 14.04282 | 155.83 | 20.91 | 154.329 | 14.6095 | | | | | | |
| | | | | | | 154.413 | 13.796 | 155.91 | 21.03 | 154.4123 | 14.6228 | | | | | | |
| | | | | | | 154.4963 | 13.7795 | 156.00 | 21.02 | 154.4957 | 14.65568 | | | | | | |
| | | | | | | 154.5797 | 13.80812 | 156.08 | 21.09 | 154.579 | 14.71901 | | | | | | |
| | | | | | | 154.663 | 13.85223 | 156.16 | 21.10 | 154.6623 | 14.60454 | | | | | | |
| | | | | | | 154.7463 | 14.02093 | 156.25 | 21.03 | 154.7457 | 14.61209 | | | | | | |
| | | | | | | 154.8297 | 13.74899 | 156.33 | 21.09 | 154.829 | 14.73512 | | | | | | |
| | | | | | | 154.913 | 14.02564 | 156.41 | 21.08 | 154.9123 | 14.5999 | | | | | | |
| | | | | | | 154.9963 | 13.72348 | 156.50 | 21.13 | 154.9957 | 14.63115 | | | | | | |
| | | | | | | 155.0797 | 13.74098 | 156.58 | 21.15 | 155.079 | 14.7278 | | | | | | |
| | | | | | | 155.163 | 14.01304 | 156.66 | 21.16 | 155.1623 | 14.62786 | | | | | | |
| | | | | | | 155.2463 | 13.77841 | 156.75 | 21.18 | 155.2457 | 14.63679 | | | | | | |
| | | | | | | 155.3297 | 13.77887 | 156.83 | 21.19 | 155.329 | 14.74275 | | | | | | |
| | | | | | | 155.413 | 13.75057 | 156.91 | 21.13 | 155.4123 | 14.60101 | | | | | | |
| | | | | | | 155.4963 | 14.02819 | 157.00 | 21.23 | 155.4957 | 14.75857 | | | | | | |
| | | | | | | 155.5797 | 13.74207 | 157.08 | 21.24 | 155.579 | 14.58273 | | | | | | |
| | | | | | | 155.663 | 13.99792 | 157.16 | 21.18 | 155.6623 | 14.62784 | | | | | | |
| | | | | | | 155.7463 | 13.71527 | 157.25 | 21.16 | 155.7457 | 14.76027 | | | | | | |
| | | | | | | 155.8297 | 13.73144 | 157.33 | 21.16 | 155.829 | 14.59742 | | | | | | |
| | | | | | | 155.913 | 13.76352 | 157.41 | 21.24 | 155.9123 | 14.61698 | | | | | | |

| | | | | | | | | | | | | | | | | | |
|---|---|---|---|---|---|---|---|---|---|---|---|---|---|---|---|---|---|
| | | | | | | 155.9963 | 13.77845 | 157.50 | 21.28 | 155.9957 | 14.62866 | | | | | | |
| | | | | | | 156.0797 | 14.01628 | 157.58 | 21.13 | 156.079 | 14.75717 | | | | | | |
| | | | | | | 156.163 | 13.98591 | 157.66 | 21.13 | 156.1623 | 14.66942 | | | | | | |
| | | | | | | 156.2463 | 13.69867 | 157.75 | 21.18 | 156.2457 | 14.65611 | | | | | | |
| | | | | | | 156.3297 | 13.84538 | 157.83 | 21.25 | 156.329 | 14.63885 | | | | | | |
| | | | | | | 156.413 | 13.77 | 157.91 | 21.12 | 156.4123 | 14.74838 | | | | | | |
| | | | | | | 156.4963 | 13.78815 | 158.00 | 21.27 | 156.4957 | 14.63594 | | | | | | |
| | | | | | | 156.5797 | 14.00335 | 158.08 | 21.11 | 156.579 | 14.62983 | | | | | | |
| | | | | | | 156.663 | 13.72043 | 158.16 | 21.17 | 156.6623 | 14.64633 | | | | | | |
| | | | | | | 156.7463 | 14.00221 | 158.25 | 21.26 | 156.7457 | 14.74116 | | | | | | |
| | | | | | | 156.8297 | 13.74216 | 158.33 | 21.24 | 156.829 | 14.65239 | | | | | | |
| | | | | | | 156.913 | 13.83428 | 158.41 | 21.14 | 156.9123 | 14.6282 | | | | | | |
| | | | | | | 156.9963 | 13.90271 | 158.50 | 21.12 | 156.9957 | 14.7307 | | | | | | |
| | | | | | | 157.0797 | 13.7701 | 158.58 | 21.12 | 157.079 | 14.63299 | | | | | | |
| | | | | | | 157.163 | 13.76834 | 158.66 | 21.09 | 157.1623 | 14.66163 | | | | | | |
| | | | | | | 157.2463 | 13.79411 | 158.75 | 21.20 | 157.2457 | 14.75213 | | | | | | |
| | | | | | | 157.3297 | 14.00331 | 158.83 | 21.20 | 157.329 | 14.72686 | | | | | | |
| | | | | | | 157.413 | 13.86399 | 158.91 | 21.24 | 157.4123 | 14.80069 | | | | | | |
| | | | | | | 157.4963 | 14.04536 | 159.00 | 21.16 | 157.4957 | 14.73555 | | | | | | |
| | | | | | | 157.5797 | 14.10341 | 159.08 | 21.12 | 157.579 | 14.79383 | | | | | | |
| | | | | | | 157.663 | 14.12907 | 159.16 | 21.16 | 157.6623 | 14.83322 | | | | | | |
| | | | | | | 157.7463 | 13.98387 | 159.25 | 21.21 | 157.7457 | 14.88307 | | | | | | |
| | | | | | | 157.8297 | 13.96498 | 159.33 | 21.31 | 157.829 | 14.83663 | | | | | | |
| | | | | | | 157.913 | 14.16006 | 159.41 | 21.33 | 157.9123 | 14.88037 | | | | | | |
| | | | | | | 157.9963 | 13.99448 | 159.50 | 21.33 | 157.9957 | 14.92865 | | | | | | |
| | | | | | | 158.0797 | 14.15706 | 159.58 | 21.32 | 158.079 | 14.9379 | | | | | | |
| | | | | | | 158.163 | 14.14778 | 159.66 | 21.33 | 158.1623 | 14.95388 | | | | | | |
| | | | | | | 158.2463 | 14.03166 | 159.75 | 21.36 | 158.2457 | 14.92907 | | | | | | |
| | | | | | | 158.3297 | 14.01676 | 159.83 | 21.38 | 158.329 | 14.97748 | | | | | | |
| | | | | | | 158.413 | 13.96537 | 159.91 | 21.43 | 158.4123 | 14.98763 | | | | | | |
| | | | | | | 158.4963 | 14.16759 | 160.00 | 21.46 | 158.4957 | 14.99226 | | | | | | |
| | | | | | | 158.5797 | 14.0488 | 160.08 | 21.46 | 158.579 | 15.01771 | | | | | | |
| | | | | | | 158.663 | 14.21506 | 160.16 | 21.40 | 158.6623 | 15.02962 | | | | | | |
| | | | | | | 158.7463 | 14.26244 | 160.25 | 21.39 | 158.7457 | 15.03864 | | | | | | |
| | | | | | | 158.8297 | 14.28059 | 160.33 | 21.40 | 158.829 | 15.02563 | | | | | | |
| | | | | | | 158.913 | 14.1589 | 160.41 | 21.50 | 158.9123 | 14.99589 | | | | | | |
| | | | | | | 158.9963 | 14.30429 | 160.50 | 21.51 | 158.9957 | 15.06157 | | | | | | |
| | | | | | | 159.0797 | 14.12606 | 160.58 | 21.53 | 159.079 | 15.14886 | | | | | | |
| | | | | | | 159.163 | 14.13767 | 160.66 | 21.59 | 159.1623 | 15.14046 | | | | | | |
| | | | | | | 159.2463 | 14.19082 | 160.75 | 21.58 | 159.2457 | 15.18034 | | | | | | |
| | | | | | | 159.3297 | 14.25373 | 160.83 | 21.49 | 159.329 | 15.21983 | | | | | | |
| | | | | | | 159.413 | 14.25302 | 160.91 | 21.53 | 159.4123 | 15.08102 | | | | | | |
| | | | | | | 159.4963 | 14.10508 | 161.00 | 21.54 | 159.4957 | 15.19918 | | | | | | |
| | | | | | | 159.5797 | 14.12954 | 161.08 | 21.53 | 159.579 | 15.09847 | | | | | | |
| | | | | | | 159.663 | 14.16209 | 161.16 | 21.54 | 159.6623 | 15.08664 | | | | | | |
| | | | | | | 159.7463 | 14.19747 | 161.25 | 21.58 | 159.7457 | 15.11259 | | | | | | |
| | | | | | | 159.8297 | 14.20218 | 161.33 | 21.53 | 159.829 | 15.21545 | | | | | | |
| | | | | | | 159.913 | 14.19317 | 161.41 | 21.61 | 159.9123 | 15.09009 | | | | | | |
| | | | | | | 159.9963 | 14.39546 | 161.50 | 21.65 | 159.9957 | 15.13007 | | | | | | |
| | | | | | | 160.0797 | 14.34661 | 161.58 | 21.63 | 160.079 | 15.22919 | | | | | | |
| | | | | | | 160.163 | 14.14239 | 161.66 | 21.65 | 160.1623 | 15.10371 | | | | | | |

| | | | | | | | | | | | | | | | | | |
|---|---|---|---|---|---|---|---|---|---|---|---|---|---|---|---|---|---|
| | | | | | | 160.2463 | 14.1779 | 161.75 | 21.58 | 160.2457 | 15.15292 | | | | | | |
| | | | | | | 160.3297 | 14.23001 | 161.83 | 21.61 | 160.329 | 15.20642 | | | | | | |
| | | | | | | 160.413 | 14.21593 | 161.91 | 21.67 | 160.4123 | 15.21986 | | | | | | |
| | | | | | | 160.4963 | 14.17939 | 162.00 | 21.68 | 160.4957 | 15.09929 | | | | | | |
| | | | | | | 160.5797 | 14.35046 | 162.08 | 21.71 | 160.579 | 15.08256 | | | | | | |
| | | | | | | 160.663 | 14.13707 | 162.16 | 21.72 | 160.6623 | 15.21244 | | | | | | |
| | | | | | | 160.7463 | 14.16002 | 162.25 | 21.78 | 160.7457 | 15.13904 | | | | | | |
| | | | | | | 160.8297 | 14.1797 | 162.33 | 21.60 | 160.829 | 15.10169 | | | | | | |
| | | | | | | 160.913 | 14.222 | 162.41 | 21.77 | 160.9123 | 15.23635 | | | | | | |
| | | | | | | 160.9963 | 14.32793 | 162.50 | 21.61 | 160.9957 | 15.10261 | | | | | | |
| | | | | | | 161.0797 | 14.20913 | 162.58 | 21.69 | 161.079 | 15.10679 | | | | | | |
| | | | | | | 161.163 | 14.34537 | 162.66 | 21.74 | 161.1623 | 15.19738 | | | | | | |
| | | | | | | 161.2463 | 14.15648 | 162.75 | 21.62 | 161.2457 | 15.12533 | | | | | | |
| | | | | | | 161.3297 | 14.15513 | 162.83 | 21.78 | 161.329 | 15.10359 | | | | | | |
| | | | | | | 161.413 | 14.18882 | 162.91 | 21.62 | 161.4123 | 15.24066 | | | | | | |
| | | | | | | 161.4963 | 14.18819 | 163.00 | 21.78 | 161.4957 | 15.13677 | | | | | | |
| | | | | | | 161.5797 | 14.20438 | 163.08 | 21.71 | 161.579 | 15.15752 | | | | | | |
| | | | | | | 161.663 | 14.32031 | 163.16 | 21.69 | 161.6623 | 15.19841 | | | | | | |
| | | | | | | 161.7463 | 14.15902 | 163.25 | 21.60 | 161.7457 | 15.21201 | | | | | | |
| | | | | | | 161.8297 | 14.35353 | 163.33 | 21.63 | 161.829 | 15.22519 | | | | | | |
| | | | | | | 161.913 | 14.14449 | 163.41 | 21.79 | 161.9123 | 15.28807 | | | | | | |
| | | | | | | 161.9963 | 14.1884 | 163.50 | 21.61 | 161.9957 | 15.23993 | | | | | | |
| | | | | | | 162.0797 | 14.20708 | 163.58 | 21.67 | 162.079 | 15.21071 | | | | | | |
| | | | | | | 162.163 | 14.1948 | 163.66 | 21.66 | 162.1623 | 15.27112 | | | | | | |
| | | | | | | 162.2463 | 14.33161 | 163.75 | 21.77 | 162.2457 | 15.30965 | | | | | | |
| | | | | | | 162.3297 | 14.14156 | 163.83 | 21.66 | 162.329 | 15.32732 | | | | | | |
| | | | | | | 162.413 | 14.16826 | 163.91 | 21.77 | 162.4123 | 15.38454 | | | | | | |
| | | | | | | 162.4963 | 14.34442 | 164.00 | 21.66 | 162.4957 | 15.39412 | | | | | | |
| | | | | | | 162.5797 | 14.2243 | 164.08 | 21.65 | 162.579 | 15.29481 | | | | | | |
| | | | | | | 162.663 | 14.29842 | 164.16 | 21.69 | 162.6623 | 15.28097 | | | | | | |
| | | | | | | 162.7463 | 14.2576 | 164.25 | 21.74 | 162.7457 | 15.40095 | | | | | | |
| | | | | | | 162.8297 | 14.41814 | 164.33 | 21.69 | 162.829 | 15.30314 | | | | | | |
| | | | | | | 162.913 | 14.47458 | 164.41 | 21.79 | 162.9123 | 15.29938 | | | | | | |
| | | | | | | 162.9963 | 14.49717 | 164.50 | 21.71 | 162.9957 | 15.41606 | | | | | | |
| | | | | | | 163.0797 | 14.25333 | 164.58 | 21.70 | 163.079 | 15.28143 | | | | | | |
| | | | | | | 163.163 | 14.34424 | 164.66 | 21.73 | 163.1623 | 15.30882 | | | | | | |
| | | | | | | 163.2463 | 14.54755 | 164.75 | 21.79 | 163.2457 | 15.38092 | | | | | | |
| | | | | | | 163.3297 | 14.2606 | 164.83 | 21.85 | 163.329 | 15.32024 | | | | | | |
| | | | | | | 163.413 | 14.54391 | 164.91 | 21.87 | 163.4123 | 15.33208 | | | | | | |
| | | | | | | 163.4963 | 14.24261 | 165.00 | 21.95 | 163.4957 | 15.34611 | | | | | | |
| | | | | | | 163.5797 | 14.53783 | 165.08 | 21.84 | 163.579 | 15.41834 | | | | | | |
| | | | | | | 163.663 | 14.258 | 165.16 | 21.92 | 163.6623 | 15.42462 | | | | | | |
| | | | | | | 163.7463 | 14.40023 | 165.25 | 21.94 | 163.7457 | 15.47155 | | | | | | |
| | | | | | | 163.8297 | 14.31694 | 165.33 | 21.95 | 163.829 | 15.51696 | | | | | | |
| | | | | | | 163.913 | 14.60042 | 165.41 | 21.84 | 163.9123 | 15.54628 | | | | | | |
| | | | | | | 163.9963 | 14.42491 | 165.50 | 21.85 | 163.9957 | 15.47464 | | | | | | |
| | | | | | | 164.0797 | 14.43521 | 165.58 | 21.92 | 164.079 | 15.47545 | | | | | | |
| | | | | | | 164.163 | 14.3835 | 165.66 | 21.85 | 164.1623 | 15.47037 | | | | | | |
| | | | | | | 164.2463 | 14.27253 | 165.75 | 21.95 | 164.2457 | 15.5298 | | | | | | |
| | | | | | | 164.3297 | 14.44939 | 165.83 | 21.91 | 164.329 | 15.45221 | | | | | | |
| | | | | | | 164.413 | 14.43765 | 165.91 | 21.89 | 164.4123 | 15.44793 | | | | | | |

| | | | | | | | | | | | | | | | | | |
|---|---|---|---|---|---|---|---|---|---|---|---|---|---|---|---|---|---|
| | | | | | | 164.4963 | 14.31431 | 166.00 | 21.94 | 164.4957 | 15.4924 | | | | | | |
| | | | | | | 164.5797 | 14.27933 | 166.08 | 22.00 | 164.579 | 15.47047 | | | | | | |
| | | | | | | 164.663 | 14.27192 | 166.16 | 21.85 | 164.6623 | 15.55551 | | | | | | |
| | | | | | | 164.7463 | 14.54821 | 166.25 | 21.94 | 164.7457 | 15.45205 | | | | | | |
| | | | | | | 164.8297 | 14.31767 | 166.33 | 21.93 | 164.829 | 15.47512 | | | | | | |
| | | | | | | 164.913 | 14.34614 | 166.41 | 22.00 | 164.9123 | 15.53936 | | | | | | |
| | | | | | | 164.9963 | 14.37158 | 166.50 | 22.00 | 164.9957 | 15.46693 | | | | | | |
| | | | | | | 165.0797 | 14.33064 | 166.58 | 21.90 | 165.079 | 15.49909 | | | | | | |
| | | | | | | 165.163 | 14.59665 | 166.66 | 22.04 | 165.1623 | 15.47913 | | | | | | |
| | | | | | | 165.2463 | 14.3538 | 166.75 | 22.10 | 165.2457 | 15.47873 | | | | | | |
| | | | | | | 165.3297 | 14.35034 | 166.83 | 22.00 | 165.329 | 15.52438 | | | | | | |
| | | | | | | 165.413 | 14.36188 | 166.91 | 22.10 | 165.4123 | 15.48041 | | | | | | |
| | | | | | | 165.4963 | 14.40351 | 167.00 | 22.07 | 165.4957 | 15.53067 | | | | | | |
| | | | | | | 165.5797 | 14.38781 | 167.08 | 21.98 | 165.579 | 15.48436 | | | | | | |
| | | | | | | 165.663 | 14.54072 | 167.16 | 21.98 | 165.6623 | 15.49987 | | | | | | |
| | | | | | | 165.7463 | 14.29081 | 167.25 | 22.10 | 165.7457 | 15.59046 | | | | | | |
| | | | | | | 165.8297 | 14.32097 | 167.33 | 22.00 | 165.829 | 15.59529 | | | | | | |
| | | | | | | 165.913 | 14.54609 | 167.41 | 22.04 | 165.9123 | 15.64769 | | | | | | |
| | | | | | | 165.9963 | 14.4287 | 167.50 | 22.01 | 165.9957 | 15.71382 | | | | | | |
| | | | | | | 166.0797 | 14.51514 | 167.58 | 21.99 | 166.079 | 15.57703 | | | | | | |
| | | | | | | 166.163 | 14.33065 | 167.66 | 22.11 | 166.1623 | 15.68957 | | | | | | |
| | | | | | | 166.2463 | 14.24991 | 167.75 | 21.99 | 166.2457 | 15.58528 | | | | | | |
| | | | | | | 166.3297 | 14.28078 | 167.83 | 22.09 | 166.329 | 15.71701 | | | | | | |
| | | | | | | 166.413 | 14.33868 | 167.91 | 22.01 | 166.4123 | 15.59897 | | | | | | |
| | | | | | | 166.4963 | 14.34844 | 168.00 | 22.00 | 166.4957 | 15.61093 | | | | | | |
| | | | | | | 166.5797 | 14.38899 | 168.08 | 22.09 | 166.579 | 15.69744 | | | | | | |
| | | | | | | 166.663 | 14.52195 | 168.16 | 22.02 | 166.6623 | 15.64077 | | | | | | |
| | | | | | | 166.7463 | 14.48138 | 168.25 | 22.11 | 166.7457 | 15.59425 | | | | | | |
| | | | | | | 166.8297 | 14.32883 | 168.33 | 21.97 | 166.829 | 15.73772 | | | | | | |
| | | | | | | 166.913 | 14.29131 | 168.41 | 21.99 | 166.9123 | 15.57582 | | | | | | |
| | | | | | | 166.9963 | 14.34451 | 168.50 | 22.11 | 166.9957 | 15.59658 | | | | | | |
| | | | | | | 167.0797 | 14.45054 | 168.58 | 22.04 | 167.079 | 15.68048 | | | | | | |
| | | | | | | 167.163 | 14.45013 | 168.66 | 22.04 | 167.1623 | 15.62012 | | | | | | |
| | | | | | | 167.2463 | 14.35101 | 168.75 | 21.97 | 167.2457 | 15.58294 | | | | | | |
| | | | | | | 167.3297 | 14.41597 | 168.83 | 21.99 | 167.329 | 15.71099 | | | | | | |
| | | | | | | 167.413 | 14.33158 | 168.91 | 22.10 | 167.4123 | 15.62415 | | | | | | |
| | | | | | | 167.4963 | 14.30458 | 169.00 | 21.98 | 167.4957 | 15.70325 | | | | | | |
| | | | | | | 167.5797 | 14.31335 | 169.08 | 22.04 | 167.579 | 15.61044 | | | | | | |
| | | | | | | 167.663 | 14.44735 | 169.16 | 22.03 | 167.6623 | 15.63148 | | | | | | |
| | | | | | | 167.7463 | 14.51888 | 169.25 | 22.00 | 167.7457 | 15.60052 | | | | | | |
| | | | | | | 167.8297 | 14.47247 | 169.33 | 22.10 | 167.829 | 15.72281 | | | | | | |
| | | | | | | 167.913 | 14.5703 | 169.41 | 22.09 | 167.9123 | 15.67527 | | | | | | |
| | | | | | | 167.9963 | 14.44347 | 169.50 | 21.97 | 167.9957 | 15.57633 | | | | | | |
| | | | | | | 168.0797 | 14.59937 | 169.58 | 22.04 | 168.079 | 15.61134 | | | | | | |
| | | | | | | 168.163 | 14.71172 | 169.66 | 22.05 | 168.1623 | 15.641 | | | | | | |
| | | | | | | 168.2463 | 14.62971 | 169.75 | 22.06 | 168.2457 | 15.6227 | | | | | | |
| | | | | | | 168.3297 | 14.71022 | 169.83 | 22.14 | 168.329 | 15.66407 | | | | | | |
| | | | | | | 168.413 | 14.67845 | 169.91 | 22.06 | 168.4123 | 15.66154 | | | | | | |
| | | | | | | 168.4963 | 14.63232 | 170.00 | 22.15 | 168.4957 | 15.57431 | | | | | | |
| | | | | | | 168.5797 | 14.46118 | 170.08 | 22.20 | 168.579 | 15.5978 | | | | | | |
| | | | | | | 168.663 | 14.56532 | 170.16 | 22.23 | 168.6623 | 15.62818 | | | | | | |

| | | | | | | | | | | | | | | | | | |
|---|---|---|---|---|---|---|---|---|---|---|---|---|---|---|---|---|---|
| | | | | | | 168.7463 | 14.65421 | 170.25 | 22.20 | 168.7457 | 15.61983 | | | | | | |
| | | | | | | 168.8297 | 14.77668 | 170.33 | 22.23 | 168.829 | 15.69884 | | | | | | |
| | | | | | | 168.913 | 14.81518 | 170.41 | 22.29 | 168.9123 | 15.71645 | | | | | | |
| | | | | | | 168.9963 | 14.82394 | 170.50 | 22.11 | 168.9957 | 15.66907 | | | | | | |
| | | | | | | 169.0797 | 14.81111 | 170.58 | 22.28 | 169.079 | 15.70329 | | | | | | |
| | | | | | | 169.163 | 14.67423 | 170.66 | 22.16 | 169.1623 | 15.7082 | | | | | | |
| | | | | | | 169.2463 | 14.70403 | 170.75 | 22.23 | 169.2457 | 15.78018 | | | | | | |
| | | | | | | 169.3297 | 14.69029 | 170.83 | 22.24 | 169.329 | 15.82987 | | | | | | |
| | | | | | | 169.413 | 14.75713 | 170.91 | 22.26 | 169.4123 | 15.86635 | | | | | | |
| | | | | | | 169.4963 | 14.71782 | 171.00 | 22.26 | 169.4957 | 15.79666 | | | | | | |
| | | | | | | 169.5797 | 14.91744 | 171.08 | 22.20 | 169.579 | 15.78898 | | | | | | |
| | | | | | | 169.663 | 14.68228 | 171.16 | 22.18 | 169.6623 | 15.87366 | | | | | | |
| | | | | | | 169.7463 | 14.89743 | 171.25 | 22.19 | 169.7457 | 15.84066 | | | | | | |
| | | | | | | 169.8297 | 14.69231 | 171.33 | 22.27 | 169.829 | 15.88732 | | | | | | |
| | | | | | | 169.913 | 14.72789 | 171.41 | 22.22 | 169.9123 | 15.86551 | | | | | | |
| | | | | | | 169.9963 | 14.77214 | 171.50 | 22.24 | 169.9957 | 15.94991 | | | | | | |
| | | | | | | 170.0797 | 14.71807 | 171.58 | 22.15 | 170.079 | 15.91773 | | | | | | |
| | | | | | | 170.163 | 14.69284 | 171.66 | 22.14 | 170.1623 | 15.77425 | | | | | | |
| | | | | | | 170.2463 | 14.88121 | 171.75 | 22.13 | 170.2457 | 15.75458 | | | | | | |
| | | | | | | 170.3297 | 14.71375 | 171.83 | 22.25 | 170.329 | 15.79496 | | | | | | |
| | | | | | | 170.413 | 14.68711 | 171.91 | 22.31 | 170.4123 | 15.79641 | | | | | | |
| | | | | | | 170.4963 | 14.71522 | 172.00 | 22.33 | 170.4957 | 15.84544 | | | | | | |
| | | | | | | 170.5797 | 14.75236 | 172.08 | 22.36 | 170.579 | 15.86821 | | | | | | |
| | | | | | | 170.663 | 14.74573 | 172.16 | 22.30 | 170.6623 | 15.88231 | | | | | | |
| | | | | | | 170.7463 | 14.88831 | 172.25 | 22.40 | 170.7457 | 15.88864 | | | | | | |
| | | | | | | 170.8297 | 14.88268 | 172.33 | 22.44 | 170.829 | 15.86881 | | | | | | |
| | | | | | | 170.913 | 14.68103 | 172.41 | 22.43 | 170.9123 | 15.9005 | | | | | | |
| | | | | | | 170.9963 | 14.75627 | 172.50 | 22.47 | 170.9957 | 15.97004 | | | | | | |
| | | | | | | 171.0797 | 14.75149 | 172.58 | 22.46 | 171.079 | 16.0191 | | | | | | |
| | | | | | | 171.163 | 14.74621 | 172.66 | 22.39 | 171.1623 | 15.98131 | | | | | | |
| | | | | | | 171.2463 | 14.87081 | 172.75 | 22.36 | 171.2457 | 15.9971 | | | | | | |
| | | | | | | 171.3297 | 14.70509 | 172.83 | 22.30 | 171.329 | 15.94616 | | | | | | |
| | | | | | | 171.413 | 14.90367 | 172.91 | 22.32 | 171.4123 | 15.9227 | | | | | | |
| | | | | | | 171.4963 | 14.69122 | 173.00 | 22.39 | 171.4957 | 15.92411 | | | | | | |
| | | | | | | 171.5797 | 14.68967 | 173.08 | 22.43 | 171.579 | 15.94074 | | | | | | |
| | | | | | | 171.663 | 14.7694 | 173.16 | 22.43 | 171.6623 | 16.00144 | | | | | | |
| | | | | | | 171.7463 | 14.78816 | 173.25 | 22.45 | 171.7457 | 15.99042 | | | | | | |
| | | | | | | 171.8297 | 14.83232 | 173.33 | 22.33 | 171.829 | 16.00416 | | | | | | |
| | | | | | | 171.913 | 14.76965 | 173.41 | 22.34 | 171.9123 | 15.93317 | | | | | | |
| | | | | | | 171.9963 | 14.66768 | 173.50 | 22.38 | 171.9957 | 15.97026 | | | | | | |
| | | | | | | 172.0797 | 14.70403 | 173.58 | 22.46 | 172.079 | 15.96523 | | | | | | |
| | | | | | | 172.163 | 14.77093 | 173.66 | 22.47 | 172.1623 | 15.99765 | | | | | | |
| | | | | | | 172.2463 | 14.85499 | 173.75 | 22.48 | 172.2457 | 16.064 | | | | | | |
| | | | | | | 172.3297 | 14.7813 | 173.83 | 22.34 | 172.329 | 15.99565 | | | | | | |
| | | | | | | 172.413 | 14.88039 | 173.91 | 22.31 | 172.4123 | 16.00684 | | | | | | |
| | | | | | | 172.4963 | 14.93189 | 174.00 | 22.31 | 172.4957 | 16.0268 | | | | | | |
| | | | | | | 172.5797 | 14.93588 | 174.08 | 22.34 | 172.579 | 16.11211 | | | | | | |
| | | | | | | 172.663 | 15.03336 | 174.16 | 22.41 | 172.6623 | 16.14054 | | | | | | |
| | | | | | | 172.7463 | 15.07752 | 174.25 | 22.36 | 172.7457 | 16.11503 | | | | | | |
| | | | | | | 172.8297 | 14.83894 | 174.33 | 22.34 | 172.829 | 16.18667 | | | | | | |
| | | | | | | 172.913 | 15.08293 | 174.41 | 22.47 | 172.9123 | 16.06868 | | | | | | |

| | | | | | | | | | | | | | | | | | |
|---|---|---|---|---|---|---|---|---|---|---|---|---|---|---|---|---|---|
| | | | | | | 172.9963 | 14.78996 | 174.50 | 22.32 | 172.9957 | 16.19697 | | | | | | |
| | | | | | | 173.0797 | 15.07382 | 174.58 | 22.34 | 173.079 | 16.17132 | | | | | | |
| | | | | | | 173.163 | 14.8319 | 174.66 | 22.40 | 173.1623 | 16.10797 | | | | | | |
| | | | | | | 173.2463 | 14.81094 | 174.75 | 22.38 | 173.2457 | 16.09902 | | | | | | |
| | | | | | | 173.3297 | 14.83972 | 174.83 | 22.41 | 173.329 | 16.08795 | | | | | | |
| | | | | | | 173.413 | 15.09652 | 174.91 | 22.42 | 173.4123 | 16.19932 | | | | | | |
| | | | | | | 173.4963 | 14.8536 | 175.00 | 22.40 | 173.4957 | 16.06472 | | | | | | |
| | | | | | | 173.5797 | 14.8684 | 175.08 | 22.48 | 173.579 | 16.20328 | | | | | | |
| | | | | | | 173.663 | 15.09868 | 175.16 | 22.52 | 173.6623 | 16.07856 | | | | | | |
| | | | | | | 173.7463 | 14.81729 | 175.25 | 22.53 | 173.7457 | 16.09249 | | | | | | |
| | | | | | | 173.8297 | 14.82713 | 175.33 | 22.52 | 173.829 | 16.0955 | | | | | | |
| | | | | | | 173.913 | 14.88325 | 175.41 | 22.53 | 173.9123 | 16.1675 | | | | | | |
| | | | | | | 173.9963 | 15.08167 | 175.50 | 22.57 | 173.9957 | 16.21316 | | | | | | |
| | | | | | | 174.0797 | 14.85275 | 175.58 | 22.64 | 174.079 | 16.03828 | | | | | | |
| | | | | | | 174.163 | 14.87861 | 175.66 | 22.48 | 174.1623 | 16.06902 | | | | | | |
| | | | | | | 174.2463 | 15.09646 | 175.75 | 22.62 | 174.2457 | 16.20674 | | | | | | |
| | | | | | | 174.3297 | 14.80278 | 175.83 | 22.56 | 174.329 | 16.09756 | | | | | | |
| | | | | | | 174.413 | 14.83209 | 175.91 | 22.51 | 174.4123 | 16.10873 | | | | | | |
| | | | | | | 174.4963 | 14.88056 | 176.00 | 22.49 | 174.4957 | 16.213 | | | | | | |
| | | | | | | 174.5797 | 15.0914 | 176.08 | 22.62 | 174.579 | 16.06346 | | | | | | |
| | | | | | | 174.663 | 14.85121 | 176.16 | 22.48 | 174.6623 | 16.07165 | | | | | | |
| | | | | | | 174.7463 | 14.8471 | 176.25 | 22.62 | 174.7457 | 16.19009 | | | | | | |
| | | | | | | 174.8297 | 15.13482 | 176.33 | 22.54 | 174.829 | 16.08635 | | | | | | |
| | | | | | | 174.913 | 14.94138 | 176.41 | 22.51 | 174.9123 | 16.09617 | | | | | | |
| | | | | | | 174.9963 | 14.90654 | 176.50 | 22.59 | 174.9957 | 16.21055 | | | | | | |
| | | | | | | 175.0797 | 14.98299 | 176.58 | 22.51 | 175.079 | 16.09938 | | | | | | |
| | | | | | | 175.163 | 15.04829 | 176.66 | 22.62 | 175.1623 | 16.17598 | | | | | | |
| | | | | | | 175.2463 | 14.99098 | 176.75 | 22.51 | 175.2457 | 16.05657 | | | | | | |
| | | | | | | 175.3297 | 14.78855 | 176.83 | 22.61 | 175.329 | 16.06001 | | | | | | |
| | | | | | | 175.413 | 14.8128 | 176.91 | 22.50 | 175.4123 | 16.22252 | | | | | | |
| | | | | | | 175.4963 | 14.91829 | 177.00 | 22.52 | 175.4957 | 16.13097 | | | | | | |
| | | | | | | 175.5797 | 14.89032 | 177.08 | 22.62 | 175.579 | 16.21154 | | | | | | |
| | | | | | | 175.663 | 14.89321 | 177.16 | 22.46 | 175.6623 | 16.17596 | | | | | | |
| | | | | | | 175.7463 | 15.08721 | 177.25 | 22.63 | 175.7457 | 16.14092 | | | | | | |
| | | | | | | 175.8297 | 14.88228 | 177.33 | 22.46 | 175.829 | 16.15371 | | | | | | |
| | | | | | | 175.913 | 15.09534 | 177.41 | 22.62 | 175.9123 | 16.16471 | | | | | | |
| | | | | | | 175.9963 | 14.81184 | 177.50 | 22.57 | 175.9957 | 16.29984 | | | | | | |
| | | | | | | 176.0797 | 14.86262 | 177.58 | 22.51 | 176.079 | 16.32899 | | | | | | |
| | | | | | | 176.163 | 15.00429 | 177.66 | 22.49 | 176.1623 | 16.35942 | | | | | | |
| | | | | | | 176.2463 | 15.01257 | 177.75 | 22.49 | 176.2457 | 16.27703 | | | | | | |
| | | | | | | 176.3297 | 14.91106 | 177.83 | 22.63 | 176.329 | 16.24724 | | | | | | |
| | | | | | | 176.413 | 14.85908 | 177.91 | 22.54 | 176.4123 | 16.32983 | | | | | | |
| | | | | | | 176.4963 | 14.87099 | 178.00 | 22.56 | 176.4957 | 16.35784 | | | | | | |
| | | | | | | 176.5797 | 14.86504 | 178.08 | 22.59 | 176.579 | 16.39551 | | | | | | |
| | | | | | | 176.663 | 15.09145 | 178.16 | 22.64 | 176.6623 | 16.43972 | | | | | | |
| | | | | | | 176.7463 | 14.92383 | 178.25 | 22.64 | 176.7457 | 16.38633 | | | | | | |
| | | | | | | 176.8297 | 14.94568 | 178.33 | 22.52 | 176.829 | 16.2949 | | | | | | |
| | | | | | | 176.913 | 14.92406 | 178.41 | 22.54 | 176.9123 | 16.28249 | | | | | | |
| | | | | | | 176.9963 | 15.07135 | 178.50 | 22.61 | 176.9957 | 16.26274 | | | | | | |
| | | | | | | 177.0797 | 14.85378 | 178.58 | 22.66 | 177.079 | 16.30438 | | | | | | |
| | | | | | | 177.163 | 14.87982 | 178.66 | 22.73 | 177.1623 | 16.34935 | | | | | | |

| | | | | | |
|---|---|---|---|---|---|
| 177.2463 | 14.93297 | 178.75 | 22.77 | 177.2457 | 16.36855 |
| 177.3297 | 14.96511 | 178.83 | 22.74 | 177.329 | 16.30099 |
| 177.413 | 14.95418 | 178.91 | 22.63 | 177.4123 | 16.29488 |
| 177.4963 | 15.02275 | 179.00 | 22.79 | 177.4957 | 16.39629 |
| 177.5797 | 14.96584 | 179.08 | 22.61 | 177.579 | 16.47402 |
| 177.663 | 14.92943 | 179.16 | 22.73 | 177.6623 | 16.48607 |
| 177.7463 | 14.94239 | 179.25 | 22.84 | 177.7457 | 16.47496 |
| 177.8297 | 14.92197 | 179.33 | 22.82 | 177.829 | 16.48505 |
| 177.913 | 14.9914 | 179.41 | 22.74 | 177.9123 | 16.47526 |
| 177.9963 | 14.99781 | 179.50 | 22.73 | 177.9957 | 16.53581 |
| 178.0797 | 15.09845 | 179.58 | 22.89 | 178.079 | 16.54857 |
| 178.163 | 15.02318 | 179.66 | 22.94 | 178.1623 | 16.6146 |
| 178.2463 | 15.08199 | 179.75 | 22.87 | 178.2457 | 16.61571 |
| 178.3297 | 15.19207 | 179.83 | 22.86 | 178.329 | 16.64202 |
| 178.413 | 15.21834 | 179.91 | 22.93 | 178.4123 | 16.70045 |
| 178.4963 | 15.21733 | 180.00 | 22.93 | 178.4957 | 16.64191 |
| 178.5797 | 15.14986 | 180.08 | 22.79 | 178.579 | 16.56312 |
| 178.663 | 15.10756 | 180.16 | 22.84 | 178.6623 | 16.65506 |
| 178.7463 | 15.03043 | 180.25 | 22.97 | 178.7457 | 16.57023 |
| 178.8297 | 15.12369 | 180.33 | 22.91 | 178.829 | 16.5563 |
| 178.913 | 15.08228 | 180.41 | 22.83 | 178.9123 | 16.5698 |
| 178.9963 | 15.13038 | 180.50 | 22.96 | 178.9957 | 16.6954 |
| 179.0797 | 15.18084 | 180.58 | 22.81 | 179.079 | 16.58023 |
| 179.163 | 15.1414 | 180.66 | 22.98 | 179.1623 | 16.70125 |
| 179.2463 | 15.12334 | 180.75 | 22.94 | 179.2457 | 16.52764 |
| 179.3297 | 15.10628 | 180.83 | 22.99 | 179.329 | 16.57329 |
| 179.413 | 15.09844 | 180.91 | 23.02 | 179.4123 | 16.67763 |
| 179.4963 | 15.10082 | 181.00 | 22.95 | 179.4957 | 16.56717 |
| 179.5797 | 15.14197 | 181.08 | 22.97 | 179.579 | 16.6794 |
| 179.663 | 15.21253 | 181.16 | 22.83 | 179.6623 | 16.65293 |
| 179.7463 | 15.22593 | 181.25 | 22.83 | 179.7457 | 16.5539 |
| 179.8297 | 15.28758 | 181.33 | 22.92 | 179.829 | 16.53937 |
| 179.913 | 15.17657 | 181.41 | 22.98 | 179.9123 | 16.61934 |
| 179.9963 | 15.35917 | 181.50 | 23.00 | 179.9957 | 16.5753 |
| 180.0797 | 15.39862 | 181.58 | 22.88 | 180.079 | 16.58318 |
| 180.163 | 15.29975 | 181.66 | 22.88 | 180.1623 | 16.68445 |
| 180.2463 | 15.26923 | 181.75 | 22.82 | 180.2457 | 16.54896 |
| 180.3297 | 15.4188 | 181.83 | 22.90 | 180.329 | 16.5624 |
| 180.413 | 15.23279 | 181.91 | 22.91 | 180.4123 | 16.63665 |
| 180.4963 | 15.25966 | 182.00 | 22.99 | 180.4957 | 16.56289 |
| 180.5797 | 15.30232 | 182.08 | 23.01 | 180.579 | 16.5731 |
| 180.663 | 15.43639 | 182.16 | 22.95 | 180.6623 | 16.69776 |
| 180.7463 | 15.28926 | 182.25 | 22.89 | 180.7457 | 16.56781 |
| 180.8297 | 15.3507 | 182.33 | 22.98 | 180.829 | 16.5956 |
| 180.913 | 15.36938 | 182.41 | 23.03 | 180.9123 | 16.66824 |
| 180.9963 | 15.29674 | 182.50 | 23.05 | 180.9957 | 16.56178 |
| 181.0797 | 15.23211 | 182.58 | 23.09 | 181.079 | 16.69937 |
| 181.163 | 15.25266 | 182.66 | 22.98 | 181.1623 | 16.57602 |
| 181.2463 | 15.31869 | 182.75 | 23.14 | 181.2457 | 16.66282 |
| 181.3297 | 15.30468 | 182.83 | 23.14 | 181.329 | 16.61714 |
| 181.413 | 15.45081 | 182.91 | 22.95 | 181.4123 | 16.60256 |

| | | | | | | | | | | | | | | | | | |
|---|---|---|---|---|---|---|---|---|---|---|---|---|---|---|---|---|---|
| | | | | | | 181.4963 | 15.27092 | 183.00 | 23.04 | 181.4957 | 16.57291 | | | | | | |
| | | | | | | 181.5797 | 15.26024 | 183.08 | 23.04 | 181.579 | 16.56276 | | | | | | |
| | | | | | | 181.663 | 15.44282 | 183.16 | 23.09 | 181.6623 | 16.66469 | | | | | | |
| | | | | | | 181.7463 | 15.2616 | 183.25 | 23.02 | 181.7457 | 16.54952 | | | | | | |
| | | | | | | 181.8297 | 15.32703 | 183.33 | 23.12 | 181.829 | 16.70169 | | | | | | |
| | | | | | | 181.913 | 15.38094 | 183.41 | 22.97 | 181.9123 | 16.5418 | | | | | | |
| | | | | | | 181.9963 | 15.34658 | 183.50 | 23.12 | 181.9957 | 16.58346 | | | | | | |
| | | | | | | 182.0797 | 15.27384 | 183.58 | 23.03 | 182.079 | 16.67149 | | | | | | |
| | | | | | | 182.163 | 15.26149 | 183.66 | 23.03 | 182.1623 | 16.55273 | | | | | | |
| | | | | | | 182.2463 | 15.3008 | 183.75 | 23.09 | 182.2457 | 16.56904 | | | | | | |
| | | | | | | 182.3297 | 15.29715 | 183.83 | 22.97 | 182.329 | 16.57257 | | | | | | |
| | | | | | | 182.413 | 15.29936 | 183.91 | 23.13 | 182.4123 | 16.67229 | | | | | | |
| | | | | | | 182.4963 | 15.30275 | 184.00 | 23.01 | 182.4957 | 16.6076 | | | | | | |
| | | | | | | 182.5797 | 15.41954 | 184.08 | 22.98 | 182.579 | 16.58829 | | | | | | |
| | | | | | | 182.663 | 15.42013 | 184.16 | 23.07 | 182.6623 | 16.60427 | | | | | | |
| | | | | | | 182.7463 | 15.221 | 184.25 | 23.07 | 182.7457 | 16.68844 | | | | | | |
| | | | | | | 182.8297 | 15.2925 | 184.33 | 22.99 | 182.829 | 16.6116 | | | | | | |
| | | | | | | 182.913 | 15.34616 | 184.41 | 23.13 | 182.9123 | 16.60216 | | | | | | |
| | | | | | | 182.9963 | 15.33081 | 184.50 | 22.98 | 182.9957 | 16.68301 | | | | | | |
| | | | | | | 183.0797 | 15.33214 | 184.58 | 23.13 | 183.079 | 16.58164 | | | | | | |
| | | | | | | 183.163 | 15.42605 | 184.66 | 23.07 | 183.1623 | 16.57465 | | | | | | |
| | | | | | | 183.2463 | 15.53907 | 184.75 | 23.05 | 183.2457 | 16.58759 | | | | | | |
| | | | | | | 183.3297 | 15.60169 | 184.83 | 23.10 | 183.329 | 16.65748 | | | | | | |
| | | | | | | 183.413 | 15.39308 | 184.91 | 23.12 | 183.4123 | 16.60578 | | | | | | |
| | | | | | | 183.4963 | 15.62921 | 185.00 | 23.03 | 183.4957 | 16.67811 | | | | | | |
| | | | | | | 183.5797 | 15.40653 | 185.08 | 22.99 | 183.579 | 16.60985 | | | | | | |
| | | | | | | 183.663 | 15.37551 | 185.16 | 23.04 | 183.6623 | 16.61406 | | | | | | |
| | | | | | | 183.7463 | 15.63166 | 185.25 | 23.02 | 183.7457 | 16.61063 | | | | | | |
| | | | | | | 183.8297 | 15.3707 | 185.33 | 23.09 | 183.829 | 16.61685 | | | | | | |
| | | | | | | 183.913 | 15.40892 | 185.41 | 23.04 | 183.9123 | 16.5764 | | | | | | |
| | | | | | | 183.9963 | 15.6546 | 185.50 | 23.11 | 183.9957 | 16.68322 | | | | | | |
| | | | | | | 184.0797 | 15.40731 | 185.58 | 23.01 | 184.079 | 16.58671 | | | | | | |
| | | | | | | 184.163 | 15.40242 | 185.66 | 23.14 | 184.1623 | 16.62375 | | | | | | |
| | | | | | | 184.2463 | 15.41487 | 185.75 | 22.93 | 184.2457 | 16.62966 | | | | | | |
| | | | | | | 184.3297 | 15.61848 | 185.83 | 23.03 | 184.329 | 16.72325 | | | | | | |
| | | | | | | 184.413 | 15.37918 | 185.91 | 23.09 | 184.4123 | 16.72536 | | | | | | |
| | | | | | | 184.4963 | 15.63036 | 186.00 | 23.00 | 184.4957 | 16.7477 | | | | | | |
| | | | | | | 184.5797 | 15.35344 | 186.08 | 23.13 | 184.579 | 16.77203 | | | | | | |
| | | | | | | 184.663 | 15.43279 | 186.16 | 22.99 | 184.6623 | 16.77594 | | | | | | |
| | | | | | | 184.7463 | 15.41149 | 186.25 | 23.13 | 184.7457 | 16.76742 | | | | | | |
| | | | | | | 184.8297 | 15.63434 | 186.33 | 23.03 | 184.829 | 16.77199 | | | | | | |
| | | | | | | 184.913 | 15.38518 | 186.41 | 23.01 | 184.9123 | 16.79842 | | | | | | |
| | | | | | | 184.9963 | 15.38168 | 186.50 | 23.11 | 184.9957 | 16.83847 | | | | | | |
| | | | | | | 185.0797 | 15.65946 | 186.58 | 23.04 | 185.079 | 16.79621 | | | | | | |
| | | | | | | 185.163 | 15.39213 | 186.66 | 23.14 | 185.1623 | 16.78136 | | | | | | |
| | | | | | | 185.2463 | 15.50618 | 186.75 | 22.98 | 185.2457 | 16.86096 | | | | | | |
| | | | | | | 185.3297 | 15.56646 | 186.83 | 23.02 | 185.329 | 16.87271 | | | | | | |
| | | | | | | 185.413 | 15.48225 | 186.91 | 23.11 | 185.4123 | 16.87436 | | | | | | |
| | | | | | | 185.4963 | 15.35249 | 187.00 | 23.03 | 185.4957 | 16.86403 | | | | | | |
| | | | | | | 185.5797 | 15.38158 | 187.08 | 23.02 | 185.579 | 16.85032 | | | | | | |
| | | | | | | 185.663 | 15.44458 | 187.16 | 23.13 | 185.6623 | 16.95359 | | | | | | |

| | | | | | | | | | | | | | | | | | |
|---|---|---|---|---|---|---|---|---|---|---|---|---|---|---|---|---|---|
| | | | | | | 185.7463 | 15.50136 | 187.25 | 22.98 | 185.7457 | 16.98311 | | | | | | |
| | | | | | | 185.8297 | 15.46074 | 187.33 | 23.12 | 185.829 | 16.98919 | | | | | | |
| | | | | | | 185.913 | 15.6221 | 187.41 | 22.98 | 185.9123 | 16.89015 | | | | | | |
| | | | | | | 185.9963 | 15.47825 | 187.50 | 23.06 | 185.9957 | 16.90673 | | | | | | |
| | | | | | | 186.0797 | 15.62585 | 187.58 | 22.98 | 186.079 | 16.95346 | | | | | | |
| | | | | | | 186.163 | 15.44002 | 187.66 | 22.99 | 186.1623 | 16.91095 | | | | | | |
| | | | | | | 186.2463 | 15.45294 | 187.75 | 23.02 | 186.2457 | 16.89922 | | | | | | |
| | | | | | | 186.3297 | 15.47484 | 187.83 | 23.14 | 186.329 | 16.90426 | | | | | | |
| | | | | | | 186.413 | 15.53783 | 187.91 | 22.99 | 186.4123 | 16.97729 | | | | | | |
| | | | | | | 186.4963 | 15.50205 | 188.00 | 23.05 | 186.4957 | 16.97167 | | | | | | |
| | | | | | | 186.5797 | 15.61449 | 188.08 | 23.09 | 186.579 | 16.87972 | | | | | | |
| | | | | | | 186.663 | 15.46683 | 188.16 | 23.03 | 186.6623 | 17.00955 | | | | | | |
| | | | | | | 186.7463 | 15.48185 | 188.25 | 23.04 | 186.7457 | 16.96171 | | | | | | |
| | | | | | | 186.8297 | 15.44965 | 188.33 | 23.12 | 186.829 | 16.95373 | | | | | | |
| | | | | | | 186.913 | 15.57119 | 188.41 | 23.10 | 186.9123 | 17.03037 | | | | | | |
| | | | | | | 186.9963 | 15.61857 | 188.50 | 23.06 | 186.9957 | 17.07277 | | | | | | |
| | | | | | | 187.0797 | 15.64484 | 188.58 | 23.13 | 187.079 | 16.9676 | | | | | | |
| | | | | | | 187.163 | 15.70306 | 188.66 | 23.18 | 187.1623 | 17.03358 | | | | | | |
| | | | | | | 187.2463 | 15.73765 | 188.75 | 23.20 | 187.2457 | 17.12348 | | | | | | |
| | | | | | | 187.3297 | 15.62729 | 188.83 | 23.21 | 187.329 | 17.14791 | | | | | | |
| | | | | | | 187.413 | 15.77729 | 188.91 | 23.25 | 187.4123 | 17.06097 | | | | | | |
| | | | | | | 187.4963 | 15.7647 | 189.00 | 23.30 | 187.4957 | 17.16957 | | | | | | |
| | | | | | | 187.5797 | 15.71211 | 189.08 | 23.14 | 187.579 | 17.15843 | | | | | | |
| | | | | | | 187.663 | 15.8107 | 189.16 | 23.20 | 187.6623 | 17.06647 | | | | | | |
| | | | | | | 187.7463 | 15.87659 | 189.25 | 23.26 | 187.7457 | 17.12306 | | | | | | |
| | | | | | | 187.8297 | 15.85518 | 189.33 | 23.14 | 187.829 | 17.03563 | | | | | | |
| | | | | | | 187.913 | 15.94463 | 189.41 | 23.16 | 187.9123 | 17.08908 | | | | | | |
| | | | | | | 187.9963 | 15.79987 | 189.50 | 23.33 | 187.9957 | 17.08705 | | | | | | |
| | | | | | | 188.0797 | 15.83398 | 189.58 | 23.14 | 188.079 | 17.16258 | | | | | | |
| | | | | | | 188.163 | 15.97236 | 189.66 | 23.22 | 188.1623 | 17.06171 | | | | | | |
| | | | | | | 188.2463 | 15.79327 | 189.75 | 23.28 | 188.2457 | 17.15068 | | | | | | |
| | | | | | | 188.3297 | 15.99204 | 189.83 | 23.33 | 188.329 | 17.03037 | | | | | | |
| | | | | | | 188.413 | 15.96932 | 189.91 | 23.31 | 188.4123 | 17.06297 | | | | | | |
| | | | | | | 188.4963 | 15.8341 | 190.00 | 23.32 | 188.4957 | 17.08289 | | | | | | |
| | | | | | | 188.5797 | 15.91485 | 190.08 | 23.33 | 188.579 | 17.1497 | | | | | | |
| | | | | | | 188.663 | 15.96635 | 190.16 | 23.36 | 188.6623 | 17.14693 | | | | | | |
| | | | | | | 188.7463 | 16.05797 | 190.25 | 23.39 | 188.7457 | 17.04011 | | | | | | |
| | | | | | | 188.8297 | 16.13411 | 190.33 | 23.43 | 188.829 | 17.17237 | | | | | | |
| | | | | | | 188.913 | 16.14356 | 190.41 | 23.42 | 188.9123 | 17.04582 | | | | | | |
| | | | | | | 188.9963 | 15.92537 | 190.50 | 23.36 | 188.9957 | 17.07163 | | | | | | |
| | | | | | | 189.0797 | 15.91712 | 190.58 | 23.38 | 189.079 | 17.10488 | | | | | | |
| | | | | | | 189.163 | 15.92568 | 190.66 | 23.38 | 189.1623 | 17.08483 | | | | | | |
| | | | | | | 189.2463 | 16.21352 | 190.75 | 23.46 | 189.2457 | 17.16392 | | | | | | |
| | | | | | | 189.3297 | 15.90356 | 190.83 | 23.39 | 189.329 | 17.02584 | | | | | | |
| | | | | | | 189.413 | 16.20307 | 190.91 | 23.40 | 189.4123 | 17.04414 | | | | | | |
| | | | | | | 189.4963 | 15.90834 | 191.00 | 23.31 | 189.4957 | 17.17534 | | | | | | |
| | | | | | | 189.5797 | 16.18067 | 191.08 | 23.30 | 189.579 | 17.14495 | | | | | | |
| | | | | | | 189.663 | 15.85997 | 191.16 | 23.32 | 189.6623 | 17.14868 | | | | | | |
| | | | | | | 189.7463 | 16.12989 | 191.25 | 23.42 | 189.7457 | 17.06318 | | | | | | |
| | | | | | | 189.8297 | 15.90307 | 191.33 | 23.37 | 189.829 | 17.00977 | | | | | | |
| | | | | | | 189.913 | 15.92558 | 191.41 | 23.39 | 189.9123 | 17.15716 | | | | | | |

| | | | | | | | | | | | | | | | | | |
|---|---|---|---|---|---|---|---|---|---|---|---|---|---|---|---|---|---|
| | | | | | | 189.9963 | 16.16568 | 191.50 | 23.35 | 189.9957 | 17.04665 | | | | | | |
| | | | | | | 190.0797 | 16.15549 | 191.58 | 23.33 | 190.079 | 17.07878 | | | | | | |
| | | | | | | 190.163 | 15.88238 | 191.66 | 23.36 | 190.1623 | 17.17539 | | | | | | |
| | | | | | | 190.2463 | 15.91411 | 191.75 | 23.48 | 190.2457 | 17.05703 | | | | | | |
| | | | | | | 190.3297 | 15.93881 | 191.83 | 23.41 | 190.329 | 17.03878 | | | | | | |
| | | | | | | 190.413 | 15.96792 | 191.91 | 23.41 | 190.4123 | 17.15004 | | | | | | |
| | | | | | | 190.4963 | 16.24375 | 192.00 | 23.36 | 190.4957 | 17.04154 | | | | | | |
| | | | | | | 190.5797 | 16.0151 | 192.08 | 23.34 | 190.579 | 17.0599 | | | | | | |
| | | | | | | 190.663 | 16.21336 | 192.16 | 23.36 | 190.6623 | 17.17981 | | | | | | |
| | | | | | | 190.7463 | 15.92484 | 192.25 | 23.47 | 190.7457 | 17.07756 | | | | | | |
| | | | | | | 190.8297 | 15.97561 | 192.33 | 23.43 | 190.829 | 17.17916 | | | | | | |
| | | | | | | 190.913 | 15.97627 | 192.41 | 23.44 | 190.9123 | 17.06112 | | | | | | |
| | | | | | | 190.9963 | 16.00034 | 192.50 | 23.45 | 190.9957 | 17.17285 | | | | | | |
| | | | | | | 191.0797 | 16.18575 | 192.58 | 23.51 | 191.079 | 17.02071 | | | | | | |
| | | | | | | 191.163 | 15.92522 | 192.66 | 23.44 | 191.1623 | 17.05228 | | | | | | |
| | | | | | | 191.2463 | 16.24305 | 192.75 | 23.55 | 191.2457 | 17.08345 | | | | | | |
| | | | | | | 191.3297 | 16.0299 | 192.83 | 23.60 | 191.329 | 17.18735 | | | | | | |
| | | | | | | 191.413 | 16.02532 | 192.91 | 23.50 | 191.4123 | 17.04554 | | | | | | |
| | | | | | | 191.4963 | 16.04758 | 193.00 | 23.58 | 191.4957 | 17.17527 | | | | | | |
| | | | | | | 191.5797 | 16.18058 | 193.08 | 23.54 | 191.579 | 17.01706 | | | | | | |
| | | | | | | 191.663 | 15.9295 | 193.16 | 23.64 | 191.6623 | 17.17403 | | | | | | |
| | | | | | | 191.7463 | 16.21345 | 193.25 | 23.50 | 191.7457 | 17.14062 | | | | | | |
| | | | | | | 191.8297 | 15.92803 | 193.33 | 23.63 | 191.829 | 17.19597 | | | | | | |
| | | | | | | 191.913 | 15.94638 | 193.41 | 23.51 | 191.9123 | 17.06082 | | | | | | |
| | | | | | | 191.9963 | 15.98023 | 193.50 | 23.56 | 191.9957 | 17.15123 | | | | | | |
| | | | | | | 192.0797 | 15.99561 | 193.58 | 23.54 | 192.079 | 17.07359 | | | | | | |
| | | | | | | 192.163 | 16.18109 | 193.66 | 23.50 | 192.1623 | 17.11201 | | | | | | |
| | | | | | | 192.2463 | 15.92462 | 193.75 | 23.61 | 192.2457 | 17.17363 | | | | | | |
| | | | | | | 192.3297 | 16.19097 | 193.83 | 23.51 | 192.329 | 17.23006 | | | | | | |
| | | | | | | 192.413 | 16.11954 | 193.91 | 23.61 | 192.4123 | 17.15779 | | | | | | |
| | | | | | | 192.4963 | 16.20258 | 194.00 | 23.57 | 192.4957 | 17.1959 | | | | | | |
| | | | | | | 192.5797 | 16.24324 | 194.08 | 23.57 | 192.579 | 17.11203 | | | | | | |
| | | | | | | 192.663 | 16.29797 | 194.16 | 23.56 | 192.6623 | 17.14472 | | | | | | |
| | | | | | | 192.7463 | 16.18331 | 194.25 | 23.60 | 192.7457 | 17.25171 | | | | | | |
| | | | | | | 192.8297 | 16.33858 | 194.33 | 23.56 | 192.829 | 17.30279 | | | | | | |
| | | | | | | 192.913 | 16.33775 | 194.41 | 23.61 | 192.9123 | 17.3177 | | | | | | |
| | | | | | | 192.9963 | 16.15939 | 194.50 | 23.69 | 192.9957 | 17.22558 | | | | | | |
| | | | | | | 193.0797 | 16.21833 | 194.58 | 23.73 | 193.079 | 17.28558 | | | | | | |
| | | | | | | 193.163 | 16.19154 | 194.66 | 23.74 | 193.1623 | 17.32324 | | | | | | |
| | | | | | | 193.2463 | 16.35737 | 194.75 | 23.70 | 193.2457 | 17.21993 | | | | | | |
| | | | | | | 193.3297 | 16.3646 | 194.83 | 23.80 | 193.329 | 17.28116 | | | | | | |
| | | | | | | 193.413 | 16.18588 | 194.91 | 23.67 | 193.4123 | 17.31562 | | | | | | |
| | | | | | | 193.4963 | 16.35458 | 195.00 | 23.69 | 193.4957 | 17.35726 | | | | | | |
| | | | | | | 193.5797 | 16.14779 | 195.08 | 23.77 | 193.579 | 17.23191 | | | | | | |
| | | | | | | 193.663 | 16.1628 | 195.16 | 23.95 | 193.6623 | 17.23123 | | | | | | |
| | | | | | | 193.7463 | 16.25734 | 195.25 | 24.03 | 193.7457 | 17.34828 | | | | | | |
| | | | | | | 193.8297 | 16.22526 | 195.33 | 24.06 | 193.829 | 17.19838 | | | | | | |
| | | | | | | 193.913 | 16.34366 | 195.41 | 24.05 | 193.9123 | 17.30139 | | | | | | |
| | | | | | | 193.9963 | 16.34975 | 195.50 | 24.09 | 193.9957 | 17.36083 | | | | | | |
| | | | | | | 194.0797 | 16.13532 | 195.58 | 24.06 | 194.079 | 17.28734 | | | | | | |
| | | | | | | 194.163 | 16.13266 | 195.66 | 24.10 | 194.1623 | 17.29641 | | | | | | |

| | | | | | | | | | | | | | | | | | |
|---|---|---|---|---|---|---|---|---|---|---|---|---|---|---|---|---|---|
| | | | | | | 194.2463 | 16.18623 | 195.75 | 24.11 | 194.2457 | 17.28915 | | | | | | |
| | | | | | | 194.3297 | 16.26557 | 195.83 | 24.11 | 194.329 | 17.30504 | | | | | | |
| | | | | | | 194.413 | 16.18135 | 195.91 | 24.12 | 194.4123 | 17.3777 | | | | | | |
| | | | | | | 194.4963 | 16.36367 | 196.00 | 24.11 | 194.4957 | 17.41873 | | | | | | |
| | | | | | | 194.5797 | 16.13589 | 196.08 | 24.19 | 194.579 | 17.43498 | | | | | | |
| | | | | | | 194.663 | 16.15966 | 196.16 | 24.22 | 194.6623 | 17.48615 | | | | | | |
| | | | | | | 194.7463 | 16.18552 | 196.25 | 24.24 | 194.7457 | 17.40616 | | | | | | |
| | | | | | | 194.8297 | 16.32703 | 196.33 | 24.27 | 194.829 | 17.42875 | | | | | | |
| | | | | | | 194.913 | 16.2274 | 196.41 | 24.19 | 194.9123 | 17.43333 | | | | | | |
| | | | | | | 194.9963 | 16.19364 | 196.50 | 24.32 | 194.9957 | 17.43527 | | | | | | |
| | | | | | | 195.0797 | 16.36855 | 196.58 | 24.20 | 195.079 | 17.46228 | | | | | | |
| | | | | | | 195.163 | 16.36643 | 196.66 | 24.25 | 195.1623 | 17.53253 | | | | | | |
| | | | | | | 195.2463 | 16.19497 | 196.75 | 24.34 | 195.2457 | 17.50929 | | | | | | |
| | | | | | | 195.3297 | 16.26868 | 196.83 | 24.36 | 195.329 | 17.51666 | | | | | | |
| | | | | | | 195.413 | 16.38379 | 196.91 | 24.34 | 195.4123 | 17.56301 | | | | | | |
| | | | | | | 195.4963 | 16.30373 | 197.00 | 24.39 | 195.4957 | 17.51826 | | | | | | |
| | | | | | | 195.5797 | 16.23386 | 197.08 | 24.40 | 195.579 | 17.58566 | | | | | | |
| | | | | | | 195.663 | 16.36718 | 197.16 | 24.42 | 195.6623 | 17.59092 | | | | | | |
| | | | | | | 195.7463 | 16.42478 | 197.25 | 24.44 | 195.7457 | 17.56237 | | | | | | |
| | | | | | | 195.8297 | 16.50919 | 197.33 | 24.44 | 195.829 | 17.61272 | | | | | | |
| | | | | | | 195.913 | 16.3309 | 197.41 | 24.45 | 195.9123 | 17.51483 | | | | | | |
| | | | | | | 195.9963 | 16.36029 | 197.50 | 24.34 | 195.9957 | 17.66557 | | | | | | |
| | | | | | | 196.0797 | 16.37903 | 197.58 | 24.39 | 196.079 | 17.56979 | | | | | | |
| | | | | | | 196.163 | 16.51291 | 197.66 | 24.40 | 196.1623 | 17.62068 | | | | | | |
| | | | | | | 196.2463 | 16.50204 | 197.75 | 24.46 | 196.2457 | 17.50272 | | | | | | |
| | | | | | | 196.3297 | 16.32761 | 197.83 | 24.54 | 196.329 | 17.66351 | | | | | | |
| | | | | | | 196.413 | 16.32927 | 197.91 | 24.55 | 196.4123 | 17.50911 | | | | | | |
| | | | | | | 196.4963 | 16.3609 | 198.00 | 24.46 | 196.4957 | 17.67209 | | | | | | |
| | | | | | | 196.5797 | 16.3785 | 198.08 | 24.48 | 196.579 | 17.57897 | | | | | | |
| | | | | | | 196.663 | 16.39089 | 198.16 | 24.42 | 196.6623 | 17.54793 | | | | | | |
| | | | | | | 196.7463 | 16.52831 | 198.25 | 24.54 | 196.7457 | 17.64937 | | | | | | |
| | | | | | | 196.8297 | 16.51046 | 198.33 | 24.59 | 196.829 | 17.5255 | | | | | | |
| | | | | | | 196.913 | 16.50559 | 198.41 | 24.62 | 196.9123 | 17.5452 | | | | | | |
| | | | | | | 196.9963 | 16.56873 | 198.50 | 24.57 | 196.9957 | 17.66123 | | | | | | |
| | | | | | | 197.0797 | 16.66926 | 198.58 | 24.58 | 197.079 | 17.61745 | | | | | | |
| | | | | | | 197.163 | 16.68114 | 198.66 | 24.51 | 197.1623 | 17.56529 | | | | | | |
| | | | | | | 197.2463 | 16.48845 | 198.75 | 24.50 | 197.2457 | 17.66875 | | | | | | |
| | | | | | | 197.3297 | 16.69566 | 198.83 | 24.53 | 197.329 | 17.49463 | | | | | | |
| | | | | | | 197.413 | 16.6949 | 198.91 | 24.61 | 197.4123 | 17.62963 | | | | | | |
| | | | | | | 197.4963 | 16.42605 | 199.00 | 24.61 | 197.4957 | 17.51767 | | | | | | |
| | | | | | | 197.5797 | 16.45714 | 199.08 | 24.62 | 197.579 | 17.53862 | | | | | | |
| | | | | | | 197.663 | 16.70663 | 199.16 | 24.53 | 197.6623 | 17.6674 | | | | | | |
| | | | | | | 197.7463 | 16.54658 | 199.25 | 24.49 | 197.7457 | 17.50291 | | | | | | |
| | | | | | | 197.8297 | 16.47733 | 199.33 | 24.65 | 197.829 | 17.51945 | | | | | | |
| | | | | | | 197.913 | 16.76844 | 199.41 | 24.53 | 197.9123 | 17.60633 | | | | | | |
| | | | | | | 197.9963 | 16.69832 | 199.50 | 24.59 | 197.9957 | 17.56001 | | | | | | |
| | | | | | | 198.0797 | 16.42387 | 199.58 | 24.58 | 198.079 | 17.5691 | | | | | | |
| | | | | | | 198.163 | 16.47689 | 199.66 | 24.57 | 198.1623 | 17.65187 | | | | | | |
| | | | | | | 198.2463 | 16.53025 | 199.75 | 24.57 | 198.2457 | 17.52696 | | | | | | |
| | | | | | | 198.3297 | 16.76386 | 199.83 | 24.53 | 198.329 | 17.66376 | | | | | | |
| | | | | | | 198.413 | 16.43862 | 199.91 | 24.47 | 198.4123 | 17.61922 | | | | | | |

| | | | | | | | | | | | | | | | | | |
|---|---|---|---|---|---|---|---|---|---|---|---|---|---|---|---|---|---|
| | | | | | | 198.4963 | 16.75112 | 200.00 | 24.56 | 198.4957 | 17.5511 | | | | | | |
| | | | | | | 198.5797 | 16.44005 | 200.08 | 24.54 | 198.579 | 17.56606 | | | | | | |
| | | | | | | 198.663 | 16.47413 | 200.16 | 24.61 | 198.6623 | 17.55959 | | | | | | |
| | | | | | | 198.7463 | 16.51217 | 200.25 | 24.53 | 198.7457 | 17.61979 | | | | | | |
| | | | | | | 198.8297 | 16.75392 | 200.33 | 24.66 | 198.829 | 17.54406 | | | | | | |
| | | | | | | 198.913 | 16.48831 | 200.41 | 24.51 | 198.9123 | 17.66245 | | | | | | |
| | | | | | | 198.9963 | 16.76365 | 200.50 | 24.54 | 198.9957 | 17.50791 | | | | | | |
| | | | | | | 199.0797 | 16.45282 | 200.58 | 24.62 | 199.079 | 17.67887 | | | | | | |
| | | | | | | 199.163 | 16.73212 | 200.66 | 24.56 | 199.1623 | 17.5354 | | | | | | |
| | | | | | | 199.2463 | 16.45427 | 200.75 | 24.60 | 199.2457 | 17.5853 | | | | | | |
| | | | | | | 199.3297 | 16.47902 | 200.83 | 24.50 | 199.329 | 17.57221 | | | | | | |
| | | | | | | 199.413 | 16.53773 | 200.91 | 24.64 | 199.4123 | 17.66461 | | | | | | |
| | | | | | | 199.4963 | 16.73546 | 201.00 | 24.52 | 199.4957 | 17.53893 | | | | | | |
| | | | | | | 199.5797 | 16.4674 | 201.08 | 24.52 | 199.579 | 17.66017 | | | | | | |
| | | | | | | 199.663 | 16.74992 | 201.16 | 24.59 | 199.6623 | 17.55278 | | | | | | |
| | | | | | | 199.7463 | 16.43831 | 201.25 | 24.56 | 199.7457 | 17.62164 | | | | | | |
| | | | | | | 199.8297 | 16.47318 | 201.33 | 24.62 | 199.829 | 17.59 | | | | | | |
| | | | | | | 199.913 | 16.52113 | 201.41 | 24.51 | 199.9123 | 17.51028 | | | | | | |
| | | | | | | 199.9963 | 16.5491 | 201.50 | 24.64 | 199.9957 | 17.49327 | | | | | | |
| | | | | | | 200.0797 | 16.74482 | 201.58 | 24.49 | 200.079 | 17.63852 | | | | | | |
| | | | | | | 200.163 | 16.47695 | 201.66 | 24.65 | 200.1623 | 17.59586 | | | | | | |
| | | | | | | 200.2463 | 16.76226 | 201.75 | 24.57 | 200.2457 | 17.70854 | | | | | | |
| | | | | | | 200.3297 | 16.457 | 201.83 | 24.59 | 200.329 | 17.72698 | | | | | | |
| | | | | | | 200.413 | 16.57189 | 201.91 | 24.61 | 200.4123 | 17.68659 | | | | | | |
| | | | | | | 200.4963 | 16.71145 | 202.00 | 24.54 | 200.4957 | 17.60295 | | | | | | |
| | | | | | | 200.5797 | 16.50077 | 202.08 | 24.51 | 200.579 | 17.59207 | | | | | | |
| | | | | | | 200.663 | 16.49402 | 202.16 | 24.53 | 200.6623 | 17.64169 | | | | | | |
| | | | | | | 200.7463 | 16.75282 | 202.25 | 24.53 | 200.7457 | 17.7645 | | | | | | |
| | | | | | | 200.8297 | 16.4649 | 202.33 | 24.58 | 200.829 | 17.81076 | | | | | | |
| | | | | | | 200.913 | 16.49954 | 202.41 | 24.57 | 200.9123 | 17.81649 | | | | | | |
| | | | | | | 200.9963 | 16.72862 | 202.50 | 24.57 | 200.9957 | 17.69642 | | | | | | |
| | | | | | | 201.0797 | 16.56723 | 202.58 | 24.51 | 201.079 | 17.81498 | | | | | | |
| | | | | | | 201.163 | 16.50275 | 202.66 | 24.51 | 201.1623 | 17.71113 | | | | | | |
| | | | | | | 201.2463 | 16.7711 | 202.75 | 24.63 | 201.2457 | 17.73041 | | | | | | |
| | | | | | | 201.3297 | 16.45102 | 202.83 | 24.64 | 201.329 | 17.83212 | | | | | | |
| | | | | | | 201.413 | 16.72139 | 202.91 | 24.64 | 201.4123 | 17.72626 | | | | | | |
| | | | | | | 201.4963 | 16.51752 | 203.00 | 24.58 | 201.4957 | 17.81006 | | | | | | |
| | | | | | | 201.5797 | 16.60049 | 203.08 | 24.66 | 201.579 | 17.69731 | | | | | | |
| | | | | | | 201.663 | 16.62427 | 203.16 | 24.57 | 201.6623 | 17.82792 | | | | | | |
| | | | | | | 201.7463 | 16.61994 | 203.25 | 24.60 | 201.7457 | 17.68381 | | | | | | |
| | | | | | | 201.8297 | 16.53375 | 203.33 | 24.71 | 201.829 | 17.82054 | | | | | | |
| | | | | | | 201.913 | 16.53569 | 203.41 | 24.77 | 201.9123 | 17.76952 | | | | | | |
| | | | | | | 201.9963 | 16.51989 | 203.50 | 24.80 | 201.9957 | 17.74919 | | | | | | |
| | | | | | | 202.0797 | 16.52349 | 203.58 | 24.69 | 202.079 | 17.69828 | | | | | | |
| | | | | | | 202.163 | 16.53591 | 203.66 | 24.78 | 202.1623 | 17.84142 | | | | | | |
| | | | | | | 202.2463 | 16.5486 | 203.75 | 24.78 | 202.2457 | 17.80889 | | | | | | |
| | | | | | | 202.3297 | 16.56109 | 203.83 | 24.65 | 202.329 | 17.70619 | | | | | | |
| | | | | | | 202.413 | 16.72679 | 203.91 | 24.66 | 202.4123 | 17.77889 | | | | | | |
| | | | | | | 202.4963 | 16.5092 | 204.00 | 24.79 | 202.4957 | 17.83275 | | | | | | |
| | | | | | | 202.5797 | 16.73358 | 204.08 | 24.69 | 202.579 | 17.85327 | | | | | | |
| | | | | | | 202.663 | 16.55436 | 204.16 | 24.83 | 202.6623 | 17.78695 | | | | | | |

| | | | | | | | | | | | | | | | | | |
|---|---|---|---|---|---|---|---|---|---|---|---|---|---|---|---|---|---|
| | | | | | | 202.7463 | 16.55974 | 204.25 | 24.64 | 202.7457 | 17.74857 | | | | | | |
| | | | | | | 202.8297 | 16.59145 | 204.33 | 24.81 | 202.829 | 17.70573 | | | | | | |
| | | | | | | 202.913 | 16.55329 | 204.41 | 24.65 | 202.9123 | 17.74104 | | | | | | |
| | | | | | | 202.9963 | 16.71526 | 204.50 | 24.71 | 202.9957 | 17.74759 | | | | | | |
| | | | | | | 203.0797 | 16.49507 | 204.58 | 24.75 | 203.079 | 17.80868 | | | | | | |
| | | | | | | 203.163 | 16.51603 | 204.66 | 24.68 | 203.1623 | 17.71806 | | | | | | |
| | | | | | | 203.2463 | 16.54524 | 204.75 | 24.80 | 203.2457 | 17.79319 | | | | | | |
| | | | | | | 203.3297 | 16.55628 | 204.83 | 24.64 | 203.329 | 17.73506 | | | | | | |
| | | | | | | 203.413 | 16.59175 | 204.91 | 24.65 | 203.4123 | 17.71461 | | | | | | |
| | | | | | | 203.4963 | 16.68278 | 205.00 | 24.77 | 203.4957 | 17.82391 | | | | | | |
| | | | | | | 203.5797 | 16.50222 | 205.08 | 24.83 | 203.579 | 17.80389 | | | | | | |
| | | | | | | 203.663 | 16.71747 | 205.16 | 24.82 | 203.6623 | 17.86621 | | | | | | |
| | | | | | | 203.7463 | 16.53762 | 205.25 | 24.85 | 203.7457 | 17.85838 | | | | | | |
| | | | | | | 203.8297 | 16.6302 | 205.33 | 24.84 | 203.829 | 17.81008 | | | | | | |
| | | | | | | 203.913 | 16.66281 | 205.41 | 24.71 | 203.9123 | 17.80604 | | | | | | |
| | | | | | | 203.9963 | 16.59163 | 205.50 | 24.71 | 203.9957 | 17.86433 | | | | | | |
| | | | | | | 204.0797 | 16.54042 | 205.58 | 24.71 | 204.079 | 17.88965 | | | | | | |
| | | | | | | 204.163 | 16.52634 | 205.66 | 24.75 | 204.1623 | 17.92494 | | | | | | |
| | | | | | | 204.2463 | 16.57273 | 205.75 | 24.81 | 204.2457 | 17.9643 | | | | | | |
| | | | | | | 204.3297 | 16.70275 | 205.83 | 24.67 | 204.329 | 17.8845 | | | | | | |
| | | | | | | 204.413 | 16.57903 | 205.91 | 24.65 | 204.4123 | 17.949 | | | | | | |
| | | | | | | 204.4963 | 16.62475 | 206.00 | 24.66 | 204.4957 | 17.92523 | | | | | | |
| | | | | | | 204.5797 | 16.72004 | 206.08 | 24.69 | 204.579 | 17.86955 | | | | | | |
| | | | | | | 204.663 | 16.48682 | 206.16 | 24.79 | 204.6623 | 17.92593 | | | | | | |
| | | | | | | 204.7463 | 16.53791 | 206.25 | 24.84 | 204.7457 | 17.94931 | | | | | | |
| | | | | | | 204.8297 | 16.53476 | 206.33 | 24.70 | 204.829 | 18.06345 | | | | | | |
| | | | | | | 204.913 | 16.59674 | 206.41 | 24.65 | 204.9123 | 17.93872 | | | | | | |
| | | | | | | 204.9963 | 16.56901 | 206.50 | 24.82 | 204.9957 | 17.94589 | | | | | | |
| | | | | | | 205.0797 | 16.61387 | 206.58 | 24.66 | 205.079 | 18.07627 | | | | | | |
| | | | | | | 205.163 | 16.70903 | 206.66 | 24.71 | 205.1623 | 18.1262 | | | | | | |
| | | | | | | 205.2463 | 16.65573 | 206.75 | 24.72 | 205.2457 | 18.01908 | | | | | | |
| | | | | | | 205.3297 | 16.58589 | 206.83 | 24.82 | 205.329 | 18.04204 | | | | | | |
| | | | | | | 205.413 | 16.52652 | 206.91 | 24.70 | 205.4123 | 18.14909 | | | | | | |
| | | | | | | 205.4963 | 16.61528 | 207.00 | 24.69 | 205.4957 | 18.06477 | | | | | | |
| | | | | | | 205.5797 | 16.75582 | 207.08 | 24.84 | 205.579 | 18.01371 | | | | | | |
| | | | | | | 205.663 | 16.78459 | 207.16 | 24.71 | 205.6623 | 18.15787 | | | | | | |
| | | | | | | 205.7463 | 16.82416 | 207.25 | 24.73 | 205.7457 | 18.01323 | | | | | | |
| | | | | | | 205.8297 | 16.82827 | 207.33 | 24.77 | 205.829 | 18.02166 | | | | | | |
| | | | | | | 205.913 | 16.75067 | 207.41 | 24.76 | 205.9123 | 18.13863 | | | | | | |
| | | | | | | 205.9963 | 16.72636 | 207.50 | 24.65 | 205.9957 | 18.14444 | | | | | | |
| | | | | | | 206.0797 | 16.80598 | 207.58 | 24.66 | 206.079 | 17.99488 | | | | | | |
| | | | | | | 206.163 | 16.89756 | 207.66 | 24.84 | 206.1623 | 18.01306 | | | | | | |
| | | | | | | 206.2463 | 16.95584 | 207.75 | 24.79 | 206.2457 | 18.0463 | | | | | | |
| | | | | | | 206.3297 | 16.86252 | 207.83 | 24.80 | 206.329 | 18.04889 | | | | | | |
| | | | | | | 206.413 | 16.75643 | 207.91 | 24.85 | 206.4123 | 18.15152 | | | | | | |
| | | | | | | 206.4963 | 16.69189 | 208.00 | 24.83 | 206.4957 | 18.1576 | | | | | | |
| | | | | | | 206.5797 | 16.83313 | 208.08 | 24.71 | 206.579 | 17.99071 | | | | | | |
| | | | | | | 206.663 | 16.95838 | 208.16 | 24.68 | 206.6623 | 18.13758 | | | | | | |
| | | | | | | 206.7463 | 16.96364 | 208.25 | 24.73 | 206.7457 | 18.0095 | | | | | | |
| | | | | | | 206.8297 | 16.92483 | 208.33 | 24.78 | 206.829 | 18.01977 | | | | | | |
| | | | | | | 206.913 | 17.03789 | 208.41 | 24.85 | 206.9123 | 18.16938 | | | | | | |

| | | | | | | | | | | | | | | | | | |
|---|---|---|---|---|---|---|---|---|---|---|---|---|---|---|---|---|---|
| | | | | | | 206.9963 | 17.02757 | 208.50 | 24.88 | 206.9957 | 18.02547 | | | | | | |
| | | | | | | 207.0797 | 16.89871 | 208.58 | 24.90 | 207.079 | 18.04314 | | | | | | |
| | | | | | | 207.163 | 16.88468 | 208.66 | 24.86 | 207.1623 | 18.14444 | | | | | | |
| | | | | | | 207.2463 | 16.97207 | 208.75 | 24.81 | 207.2457 | 18.03463 | | | | | | |
| | | | | | | 207.3297 | 16.96167 | 208.83 | 24.92 | 207.329 | 18.1418 | | | | | | |
| | | | | | | 207.413 | 16.99745 | 208.91 | 24.96 | 207.4123 | 18.02306 | | | | | | |
| | | | | | | 207.4963 | 16.89403 | 209.00 | 24.92 | 207.4957 | 18.0378 | | | | | | |
| | | | | | | 207.5797 | 16.96848 | 209.08 | 24.95 | 207.579 | 18.12996 | | | | | | |
| | | | | | | 207.663 | 16.85401 | 209.16 | 24.88 | 207.6623 | 18.02221 | | | | | | |
| | | | | | | 207.7463 | 16.98972 | 209.25 | 24.84 | 207.7457 | 18.15194 | | | | | | |
| | | | | | | 207.8297 | 17.16629 | 209.33 | 25.00 | 207.829 | 17.9819 | | | | | | |
| | | | | | | 207.913 | 17.21437 | 209.41 | 24.86 | 207.9123 | 18.04146 | | | | | | |
| | | | | | | 207.9963 | 17.04244 | 209.50 | 24.93 | 207.9957 | 18.12899 | | | | | | |
| | | | | | | 208.0797 | 17.24245 | 209.58 | 24.95 | 208.079 | 18.0235 | | | | | | |
| | | | | | | 208.163 | 17.02267 | 209.66 | 24.96 | 208.1623 | 18.03861 | | | | | | |
| | | | | | | 208.2463 | 17.26472 | 209.75 | 24.98 | 208.2457 | 18.15398 | | | | | | |
| | | | | | | 208.3297 | 16.99283 | 209.83 | 24.94 | 208.329 | 18.01671 | | | | | | |
| | | | | | | 208.413 | 17.03558 | 209.91 | 24.91 | 208.4123 | 18.07322 | | | | | | |
| | | | | | | 208.4963 | 17.05733 | 210.00 | 25.02 | 208.4957 | 18.11923 | | | | | | |
| | | | | | | 208.5797 | 17.28555 | 210.08 | 25.05 | 208.579 | 18.0504 | | | | | | |
| | | | | | | 208.663 | 17.24387 | 210.16 | 25.08 | 208.6623 | 18.15563 | | | | | | |
| | | | | | | 208.7463 | 16.94961 | 210.25 | 25.11 | 208.7457 | 18.03842 | | | | | | |
| | | | | | | 208.8297 | 17.04645 | 210.33 | 25.04 | 208.829 | 18.13138 | | | | | | |
| | | | | | | 208.913 | 17.22655 | 210.41 | 25.14 | 208.9123 | 18.05213 | | | | | | |
| | | | | | | 208.9963 | 17.04865 | 210.50 | 25.00 | 208.9957 | 18.03765 | | | | | | |
| | | | | | | 209.0797 | 17.06015 | 210.58 | 25.11 | 209.079 | 18.1478 | | | | | | |
| | | | | | | 209.163 | 17.28319 | 210.66 | 25.08 | 209.1623 | 18.00193 | | | | | | |
| | | | | | | 209.2463 | 17.25403 | 210.75 | 25.09 | 209.2457 | 18.03788 | | | | | | |
| | | | | | | 209.3297 | 17.0191 | 210.83 | 25.06 | 209.329 | 18.13104 | | | | | | |
| | | | | | | 209.413 | 17.09988 | 210.91 | 25.02 | 209.4123 | 18.0102 | | | | | | |
| | | | | | | 209.4963 | 17.03698 | 211.00 | 25.17 | 209.4957 | 18.08399 | | | | | | |
| | | | | | | 209.5797 | 17.2842 | 211.08 | 25.01 | 209.579 | 18.06591 | | | | | | |
| | | | | | | 209.663 | 17.23662 | 211.16 | 25.05 | 209.6623 | 18.04374 | | | | | | |
| | | | | | | 209.7463 | 17.31358 | 211.25 | 25.11 | 209.7457 | 18.14518 | | | | | | |
| | | | | | | 209.8297 | 17.00882 | 211.33 | 25.06 | 209.829 | 18.01831 | | | | | | |
| | | | | | | 209.913 | 17.01686 | 211.41 | 25.15 | 209.9123 | 18.05798 | | | | | | |
| | | | | | | 209.9963 | 17.2678 | 211.50 | 25.01 | 209.9957 | 18.14748 | | | | | | |
| | | | | | | 210.0797 | 17.08996 | 211.58 | 25.16 | 210.079 | 18.04725 | | | | | | |
| | | | | | | 210.163 | 17.08124 | 211.66 | 24.99 | 210.1623 | 18.14083 | | | | | | |
| | | | | | | 210.2463 | 17.09416 | 211.75 | 25.08 | 210.2457 | 18.03454 | | | | | | |
| | | | | | | 210.3297 | 17.28274 | 211.83 | 25.03 | 210.329 | 18.00895 | | | | | | |
| | | | | | | 210.413 | 17.0255 | 211.91 | 25.03 | 210.4123 | 18.04641 | | | | | | |
| | | | | | | 210.4963 | 17.27789 | 212.00 | 25.16 | 210.4957 | 18.14033 | | | | | | |
| | | | | | | 210.5797 | 17.00332 | 212.08 | 25.00 | 210.579 | 18.03685 | | | | | | |
| | | | | | | 210.663 | 17.03879 | 212.16 | 25.17 | 210.6623 | 18.0808 | | | | | | |
| | | | | | | 210.7463 | 17.06999 | 212.25 | 25.06 | 210.7457 | 18.04556 | | | | | | |
| | | | | | | 210.8297 | 17.28206 | 212.33 | 25.05 | 210.829 | 18.03599 | | | | | | |
| | | | | | | 210.913 | 17.02984 | 212.41 | 25.10 | 210.9123 | 18.05567 | | | | | | |
| | | | | | | 210.9963 | 17.27047 | 212.50 | 25.05 | 210.9957 | 18.13502 | | | | | | |
| | | | | | | 211.0797 | 16.98642 | 212.58 | 25.16 | 211.079 | 18.04398 | | | | | | |
| | | | | | | 211.163 | 17.03582 | 212.66 | 25.02 | 211.1623 | 18.03532 | | | | | | |

| | | | | | | | | | | | | | | | | | |
|---|---|---|---|---|---|---|---|---|---|---|---|---|---|---|---|---|---|
| | | | | | | 211.2463 | 17.0936 | 212.75 | 25.15 | 211.2457 | 18.12178 | | | | | | |
| | | | | | | 211.3297 | 17.0816 | 212.83 | 25.06 | 211.329 | 18.04454 | | | | | | |
| | | | | | | 211.413 | 17.29938 | 212.91 | 25.05 | 211.4123 | 18.04915 | | | | | | |
| | | | | | | 211.4963 | 17.27022 | 213.00 | 25.13 | 211.4957 | 18.12843 | | | | | | |
| | | | | | | 211.5797 | 16.9945 | 213.08 | 25.01 | 211.579 | 18.08614 | | | | | | |
| | | | | | | 211.663 | 17.03712 | 213.16 | 25.16 | 211.6623 | 18.08394 | | | | | | |
| | | | | | | 211.7463 | 17.09678 | 213.25 | 25.00 | 211.7457 | 18.16771 | | | | | | |
| | | | | | | 211.8297 | 17.08742 | 213.33 | 25.15 | 211.829 | 18.15441 | | | | | | |
| | | | | | | 211.913 | 17.26561 | 213.41 | 25.07 | 211.9123 | 18.12338 | | | | | | |
| | | | | | | 211.9963 | 17.0289 | 213.50 | 25.01 | 211.9957 | 18.1539 | | | | | | |
| | | | | | | 212.0797 | 17.26662 | 213.58 | 25.15 | 212.079 | 18.23459 | | | | | | |
| | | | | | | 212.163 | 17.01734 | 213.66 | 25.03 | 212.1623 | 18.26455 | | | | | | |
| | | | | | | 212.2463 | 17.05022 | 213.75 | 25.12 | 212.2457 | 18.29428 | | | | | | |
| | | | | | | 212.3297 | 17.12095 | 213.83 | 25.01 | 212.329 | 18.25921 | | | | | | |
| | | | | | | 212.413 | 17.27787 | 213.91 | 25.11 | 212.4123 | 18.1897 | | | | | | |
| | | | | | | 212.4963 | 17.03915 | 214.00 | 25.04 | 212.4957 | 18.31168 | | | | | | |
| | | | | | | 212.5797 | 17.04422 | 214.08 | 25.04 | 212.579 | 18.2292 | | | | | | |
| | | | | | | 212.663 | 17.29183 | 214.16 | 25.13 | 212.6623 | 18.24007 | | | | | | |
| | | | | | | 212.7463 | 17.04026 | 214.25 | 25.03 | 212.7457 | 18.29133 | | | | | | |
| | | | | | | 212.8297 | 17.02683 | 214.33 | 25.14 | 212.829 | 18.29034 | | | | | | |
| | | | | | | 212.913 | 17.10587 | 214.41 | 25.01 | 212.9123 | 18.3327 | | | | | | |
| | | | | | | 212.9963 | 17.07596 | 214.50 | 25.02 | 212.9957 | 18.35104 | | | | | | |
| | | | | | | 213.0797 | 17.24733 | 214.58 | 25.12 | 213.079 | 18.41019 | | | | | | |
| | | | | | | 213.163 | 17.04227 | 214.66 | 25.04 | 213.1623 | 18.41578 | | | | | | |
| | | | | | | 213.2463 | 17.26898 | 214.75 | 25.04 | 213.2457 | 18.36605 | | | | | | |
| | | | | | | 213.3297 | 17.03693 | 214.83 | 25.15 | 213.329 | 18.45828 | | | | | | |
| | | | | | | 213.413 | 17.03769 | 214.91 | 25.02 | 213.4123 | 18.36056 | | | | | | |
| | | | | | | 213.4963 | 17.13966 | 215.00 | 25.16 | 213.4957 | 18.39152 | | | | | | |
| | | | | | | 213.5797 | 17.13672 | 215.08 | 25.11 | 213.579 | 18.42808 | | | | | | |
| | | | | | | 213.663 | 17.09291 | 215.16 | 25.05 | 213.6623 | 18.40078 | | | | | | |
| | | | | | | 213.7463 | 17.27485 | 215.25 | 25.05 | 213.7457 | 18.39673 | | | | | | |
| | | | | | | 213.8297 | 17.0498 | 215.33 | 25.05 | 213.829 | 18.34963 | | | | | | |
| | | | | | | 213.913 | 17.27165 | 215.41 | 25.15 | 213.9123 | 18.40442 | | | | | | |
| | | | | | | 213.9963 | 17.15401 | 215.50 | 25.10 | 213.9957 | 18.38849 | | | | | | |
| | | | | | | 214.0797 | 17.29881 | 215.58 | 25.09 | 214.079 | 18.43097 | | | | | | |
| | | | | | | 214.163 | 17.32829 | | | 214.1623 | 18.35058 | | | | | | |
| | | | | | | 214.2463 | 17.3298 | | | 214.2457 | 18.35717 | | | | | | |
| | | | | | | 214.3297 | 17.41486 | | | 214.329 | 18.40283 | | | | | | |
| | | | | | | 214.413 | 17.23822 | | | 214.4123 | 18.46592 | | | | | | |
| | | | | | | 214.4963 | 17.43139 | | | 214.4957 | 18.47288 | | | | | | |
| | | | | | | 214.5797 | 17.29889 | | | 214.579 | 18.46073 | | | | | | |
| | | | | | | 214.663 | 17.3598 | | | 214.6623 | 18.37994 | | | | | | |
| | | | | | | 214.7463 | 17.289 | | | 214.7457 | 18.39221 | | | | | | |
| | | | | | | 214.8297 | 17.45683 | | | 214.829 | 18.38302 | | | | | | |
| | | | | | | 214.913 | 17.24789 | | | 214.9123 | 18.3945 | | | | | | |
| | | | | | | 214.9963 | 17.24683 | | | 214.9957 | 18.37607 | | | | | | |
| | | | | | | 215.0797 | 17.27541 | | | 215.079 | 18.38978 | | | | | | |
| | | | | | | 215.163 | 17.30649 | | | 215.1623 | 18.44075 | | | | | | |
| | | | | | | 215.2463 | 17.30854 | | | 215.2457 | 18.37101 | | | | | | |
| | | | | | | 215.3297 | 17.43558 | | | 215.329 | 18.37655 | | | | | | |
| | | | | | | 215.413 | 17.43043 | | | 215.4123 | 18.40317 | | | | | | |

| | | | | | | | | | | | | | | | | | |
|---|---|---|---|---|---|---|---|---|---|---|---|---|---|---|---|---|---|
| | | | | | | 215.4963 | 17.43519 | | | 215.4957 | 18.35428 | | | | | | |
| | | | | | | 215.5797 | 17.23688 | | | 215.579 | 18.40948 | | | | | | |
| | | | | | | 215.663 | 17.29715 | | | 215.6623 | 18.43351 | | | | | | |
| | | | | | | 215.7463 | 17.34883 | | | 215.7457 | 18.37545 | | | | | | |
| | | | | | | 215.8297 | 17.4156 | | | 215.829 | 18.38366 | | | | | | |
| | | | | | | 215.913 | 17.43272 | | | 215.9123 | 18.42357 | | | | | | |
| | | | | | | 215.9963 | 17.26152 | | | 215.9957 | 18.4468 | | | | | | |
| | | | | | | 216.0797 | 17.23115 | | | 216.079 | 18.51853 | | | | | | |
| | | | | | | 216.163 | 17.25807 | | | 216.1623 | 18.57037 | | | | | | |
| | | | | | | 216.2463 | 17.27295 | | | 216.2457 | 18.54876 | | | | | | |
| | | | | | | 216.3297 | 17.35073 | | | 216.329 | 18.61191 | | | | | | |
| | | | | | | 216.413 | 17.43965 | | | 216.4123 | 18.56369 | | | | | | |
| | | | | | | 216.4963 | 17.43087 | | | 216.4957 | 18.67873 | | | | | | |
| | | | | | | 216.5797 | 17.25764 | | | 216.579 | 18.69959 | | | | | | |
| | | | | | | 216.663 | 17.25976 | | | 216.6623 | 18.71699 | | | | | | |
| | | | | | | 216.7463 | 17.27875 | | | 216.7457 | 18.72145 | | | | | | |
| | | | | | | 216.8297 | 17.3336 | | | 216.829 | 18.67439 | | | | | | |
| | | | | | | 216.913 | 17.40443 | | | 216.9123 | 18.65039 | | | | | | |
| | | | | | | 216.9963 | 17.39475 | | | 216.9957 | 18.73237 | | | | | | |
| | | | | | | 217.0797 | 17.32884 | | | 217.079 | 18.77388 | | | | | | |
| | | | | | | 217.163 | 17.40764 | | | 217.1623 | 18.69063 | | | | | | |
| | | | | | | 217.2463 | 17.4741 | | | 217.2457 | 18.76475 | | | | | | |
| | | | | | | 217.3297 | 17.50132 | | | 217.329 | 18.72543 | | | | | | |
| | | | | | | 217.413 | 17.59893 | | | 217.4123 | 18.70951 | | | | | | |
| | | | | | | 217.4963 | 17.48906 | | | 217.4957 | 18.67409 | | | | | | |
| | | | | | | 217.5797 | 17.61413 | | | 217.579 | 18.80105 | | | | | | |
| | | | | | | 217.663 | 17.60344 | | | 217.6623 | 18.69218 | | | | | | |
| | | | | | | 217.7463 | 17.4358 | | | 217.7457 | 18.7584 | | | | | | |
| | | | | | | 217.8297 | 17.43193 | | | 217.829 | 18.77142 | | | | | | |
| | | | | | | 217.913 | 17.44343 | | | 217.9123 | 18.7535 | | | | | | |
| | | | | | | 217.9963 | 17.51541 | | | 217.9957 | 18.66078 | | | | | | |
| | | | | | | 218.0797 | 17.51379 | | | 218.079 | 18.68978 | | | | | | |
| | | | | | | 218.163 | 17.47959 | | | 218.1623 | 18.70328 | | | | | | |
| | | | | | | 218.2463 | 17.42861 | | | 218.2457 | 18.71107 | | | | | | |
| | | | | | | 218.3297 | 17.43994 | | | 218.329 | 18.75004 | | | | | | |
| | | | | | | 218.413 | 17.63598 | | | 218.4123 | 18.75604 | | | | | | |
| | | | | | | 218.4963 | 17.42724 | | | 218.4957 | 18.72098 | | | | | | |
| | | | | | | 218.5797 | 17.54182 | | | 218.579 | 18.71619 | | | | | | |
| | | | | | | 218.663 | 17.59111 | | | 218.6623 | 18.7783 | | | | | | |
| | | | | | | 218.7463 | 17.46582 | | | 218.7457 | 18.72514 | | | | | | |
| | | | | | | 218.8297 | 17.42513 | | | 218.829 | 18.83405 | | | | | | |
| | | | | | | 218.913 | 17.62947 | | | 218.9123 | 18.83764 | | | | | | |
| | | | | | | 218.9963 | 17.44222 | | | 218.9957 | 18.85035 | | | | | | |
| | | | | | | 219.0797 | 17.47432 | | | 219.079 | 18.81875 | | | | | | |
| | | | | | | 219.163 | 17.49219 | | | 219.1623 | 18.73044 | | | | | | |
| | | | | | | 219.2463 | 17.48894 | | | 219.2457 | 18.87 | | | | | | |
| | | | | | | 219.3297 | 17.61162 | | | 219.329 | 18.8885 | | | | | | |
| | | | | | | 219.413 | 17.61903 | | | 219.4123 | 18.93063 | | | | | | |
| | | | | | | 219.4963 | 17.42119 | | | 219.4957 | 18.90801 | | | | | | |
| | | | | | | 219.5797 | 17.44143 | | | 219.579 | 18.88448 | | | | | | |
| | | | | | | 219.663 | 17.5227 | | | 219.6623 | 18.82858 | | | | | | |

| | | | | | | | | | | | | | | | | | |
|---|---|---|---|---|---|---|---|---|---|---|---|---|---|---|---|---|---|
| | | | | | | 219.7463 | 17.52211 | | | 219.7457 | 18.92093 | | | | | | |
| | | | | | | 219.8297 | 17.60532 | | | 219.829 | 18.83712 | | | | | | |
| | | | | | | 219.913 | 17.6431 | | | 219.9123 | 18.85634 | | | | | | |
| | | | | | | 219.9963 | 17.44295 | | | 219.9957 | 18.93182 | | | | | | |
| | | | | | | 220.0797 | 17.49645 | | | 220.079 | 18.90162 | | | | | | |
| | | | | | | 220.163 | 17.53158 | | | 220.1623 | 18.91635 | | | | | | |
| | | | | | | 220.2463 | 17.61943 | | | 220.2457 | 18.92834 | | | | | | |
| | | | | | | 220.3297 | 17.68635 | | | 220.329 | 18.8835 | | | | | | |
| | | | | | | 220.413 | 17.74089 | | | 220.4123 | 18.92384 | | | | | | |
| | | | | | | 220.4963 | 17.79978 | | | 220.4957 | 18.99896 | | | | | | |
| | | | | | | 220.5797 | 17.57885 | | | 220.579 | 19.04518 | | | | | | |
| | | | | | | 220.663 | 17.80047 | | | 220.6623 | 19.00297 | | | | | | |
| | | | | | | 220.7463 | 17.53475 | | | 220.7457 | 19.04755 | | | | | | |
| | | | | | | 220.8297 | 17.59142 | | | 220.829 | 19.07443 | | | | | | |
| | | | | | | 220.913 | 17.60739 | | | 220.9123 | 19.11582 | | | | | | |
| | | | | | | 220.9963 | 17.82323 | | | 220.9957 | 18.98241 | | | | | | |
| | | | | | | 221.0797 | 17.56783 | | | 221.079 | 18.97138 | | | | | | |
| | | | | | | 221.163 | 17.8211 | | | 221.1623 | 19.09884 | | | | | | |
| | | | | | | 221.2463 | 17.53339 | | | 221.2457 | 18.9628 | | | | | | |
| | | | | | | 221.3297 | 17.59166 | | | 221.329 | 19.12784 | | | | | | |
| | | | | | | 221.413 | 17.62891 | | | 221.4123 | 18.94689 | | | | | | |
| | | | | | | 221.4963 | 17.81625 | | | 221.4957 | 18.98991 | | | | | | |
| | | | | | | 221.5797 | 17.57861 | | | 221.579 | 19.00983 | | | | | | |
| | | | | | | 221.663 | 17.83866 | | | 221.6623 | 19.08581 | | | | | | |
| | | | | | | 221.7463 | 17.54046 | | | 221.7457 | 18.99055 | | | | | | |
| | | | | | | 221.8297 | 17.82186 | | | 221.829 | 19.14056 | | | | | | |
| | | | | | | 221.913 | 17.5937 | | | 221.9123 | 18.96974 | | | | | | |
| | | | | | | 221.9963 | 17.63011 | | | 221.9957 | 19.06445 | | | | | | |
| | | | | | | 222.0797 | 17.81471 | | | 222.079 | 18.97749 | | | | | | |
| | | | | | | 222.163 | 17.54693 | | | 222.1623 | 19.0128 | | | | | | |
| | | | | | | 222.2463 | 17.82142 | | | 222.2457 | 19.0051 | | | | | | |
| | | | | | | 222.3297 | 17.53256 | | | 222.329 | 19.11759 | | | | | | |
| | | | | | | 222.413 | 17.58989 | | | 222.4123 | 18.99761 | | | | | | |
| | | | | | | 222.4963 | 17.62186 | | | 222.4957 | 19.12773 | | | | | | |
| | | | | | | 222.5797 | 17.86248 | | | 222.579 | 18.969 | | | | | | |
| | | | | | | 222.663 | 17.5528 | | | 222.6623 | 19.01029 | | | | | | |
| | | | | | | 222.7463 | 17.60113 | | | 222.7457 | 19.02274 | | | | | | |
| | | | | | | 222.8297 | 17.8246 | | | 222.829 | 19.05305 | | | | | | |
| | | | | | | 222.913 | 17.5623 | | | 222.9123 | 18.97506 | | | | | | |
| | | | | | | 222.9963 | 17.81369 | | | 222.9957 | 19.14453 | | | | | | |
| | | | | | | 223.0797 | 17.6032 | | | 223.079 | 18.96388 | | | | | | |
| | | | | | | 223.163 | 17.62143 | | | 223.1623 | 18.98972 | | | | | | |
| | | | | | | 223.2463 | 17.60843 | | | 223.2457 | 19.03897 | | | | | | |
| | | | | | | 223.3297 | 17.82393 | | | 223.329 | 19.08768 | | | | | | |
| | | | | | | 223.413 | 17.56487 | | | 223.4123 | 19.01318 | | | | | | |
| | | | | | | 223.4963 | 17.5849 | | | 223.4957 | 19.13309 | | | | | | |
| | | | | | | 223.5797 | 17.83468 | | | 223.579 | 19.12277 | | | | | | |
| | | | | | | 223.663 | 17.58427 | | | 223.6623 | 19.00271 | | | | | | |
| | | | | | | 223.7463 | 17.64335 | | | 223.7457 | 19.01127 | | | | | | |
| | | | | | | 223.8297 | 17.61597 | | | 223.829 | 19.01857 | | | | | | |
| | | | | | | 223.913 | 17.84091 | | | 223.9123 | 19.01635 | | | | | | |

| | | | | | | | | | | | | | | | | |
|---|---|---|---|---|---|---|---|---|---|---|---|---|---|---|---|---|
| | | | | | | 223.9963 | 17.56375 | | | 223.9957 | 19.11183 | | | | | |
| | | | | | | 224.0797 | 17.81692 | | | 224.079 | 19.00184 | | | | | |
| | | | | | | 224.163 | 17.61331 | | | 224.1623 | 19.11393 | | | | | |
| | | | | | | 224.2463 | 17.65482 | | | 224.2457 | 18.98027 | | | | | |
| | | | | | | 224.3297 | 17.6026 | | | 224.329 | 19.01468 | | | | | |
| | | | | | | 224.413 | 17.84307 | | | 224.4123 | 19.0075 | | | | | |
| | | | | | | 224.4963 | 17.55989 | | | 224.4957 | 19.09822 | | | | | |
| | | | | | | 224.5797 | 17.80681 | | | 224.579 | 19.00821 | | | | | |
| | | | | | | 224.663 | 17.57204 | | | 224.6623 | 19.11233 | | | | | |
| | | | | | | 224.7463 | 17.66079 | | | 224.7457 | 18.97966 | | | | | |
| | | | | | | 224.8297 | 17.65836 | | | 224.829 | 18.9925 | | | | | |
| | | | | | | 224.913 | 17.6189 | | | 224.9123 | 19.03282 | | | | | |
| | | | | | | 224.9963 | 17.82906 | | | 224.9957 | 19.10255 | | | | | |
| | | | | | | 225.0797 | 17.58537 | | | 225.079 | 19.02757 | | | | | |
| | | | | | | 225.163 | 17.60729 | | | 225.1623 | 19.13611 | | | | | |
| | | | | | | 225.2463 | 17.83062 | | | 225.2457 | 18.99639 | | | | | |
| | | | | | | 225.3297 | 17.65846 | | | 225.329 | 19.11516 | | | | | |
| | | | | | | 225.413 | 17.70555 | | | 225.4123 | 19.00201 | | | | | |
| | | | | | | 225.4963 | 17.7544 | | | 225.4957 | 19.06052 | | | | | |
| | | | | | | 225.5797 | 17.63222 | | | 225.579 | 19.1023 | | | | | |
| | | | | | | 225.663 | 17.59978 | | | 225.6623 | 19.10554 | | | | | |
| | | | | | | 225.7463 | 17.58938 | | | 225.7457 | 19.0296 | | | | | |
| | | | | | | 225.8297 | 17.63236 | | | 225.829 | 19.00359 | | | | | |
| | | | | | | 225.913 | 17.69612 | | | 225.9123 | 18.98287 | | | | | |
| | | | | | | 225.9963 | 17.71656 | | | 225.9957 | 19.04728 | | | | | |
| | | | | | | 226.0797 | 17.69889 | | | 226.079 | 19.06989 | | | | | |
| | | | | | | 226.163 | 17.7892 | | | 226.1623 | 19.09539 | | | | | |
| | | | | | | 226.2463 | 17.76477 | | | 226.2457 | 19.12364 | | | | | |
| | | | | | | 226.3297 | 17.74204 | | | 226.329 | 19.04601 | | | | | |
| | | | | | | 226.413 | 17.8149 | | | 226.4123 | 19.0089 | | | | | |
| | | | | | | 226.4963 | 17.91706 | | | 226.4957 | 19.00072 | | | | | |
| | | | | | | 226.5797 | 17.86946 | | | 226.579 | 19.01375 | | | | | |
| | | | | | | 226.663 | 17.96669 | | | 226.6623 | 19.0702 | | | | | |
| | | | | | | 226.7463 | 18.00109 | | | 226.7457 | 19.08695 | | | | | |
| | | | | | | 226.8297 | 17.82177 | | | 226.829 | 19.03461 | | | | | |
| | | | | | | 226.913 | 17.88772 | | | 226.9123 | 19.02801 | | | | | |
| | | | | | | 226.9963 | 17.97197 | | | 226.9957 | 19.1234 | | | | | |
| | | | | | | 227.0797 | 18.03159 | | | 227.079 | 18.99589 | | | | | |
| | | | | | | 227.163 | 18.03719 | | | 227.1623 | 19.08607 | | | | | |
| | | | | | | 227.2463 | 18.03157 | | | 227.2457 | 19.03391 | | | | | |
| | | | | | | 227.3297 | 17.96478 | | | 227.329 | 19.02845 | | | | | |
| | | | | | | 227.413 | 18.11785 | | | 227.4123 | 19.02226 | | | | | |
| | | | | | | 227.4963 | 18.1352 | | | 227.4957 | 19.10144 | | | | | |
| | | | | | | 227.5797 | 17.9797 | | | 227.579 | 19.00104 | | | | | |
| | | | | | | 227.663 | 18.01828 | | | 227.6623 | 19.07777 | | | | | |
| | | | | | | 227.7463 | 17.99838 | | | 227.7457 | 19.03801 | | | | | |
| | | | | | | 227.8297 | 18.1678 | | | 227.829 | 19.04304 | | | | | |
| | | | | | | 227.913 | 17.97932 | | | 227.9123 | 19.06266 | | | | | |
| | | | | | | 227.9963 | 18.1483 | | | 227.9957 | 19.06538 | | | | | |
| | | | | | | 228.0797 | 18.09521 | | | 228.079 | 19.12726 | | | | | |
| | | | | | | 228.163 | 18.13733 | | | 228.1623 | 19.11307 | | | | | |

| | | | | | | | | | | | | | | | | | |
|---|---|---|---|---|---|---|---|---|---|---|---|---|---|---|---|---|---|
| | | | | | | 228.2463 | 18.26431 | | | 228.2457 | 19.1765 | | | | | | |
| | | | | | | 228.3297 | 18.30447 | | | 228.329 | 19.22783 | | | | | | |
| | | | | | | 228.413 | 18.30434 | | | 228.4123 | 19.25678 | | | | | | |
| | | | | | | 228.4963 | 18.11841 | | | 228.4957 | 19.23967 | | | | | | |
| | | | | | | 228.5797 | 18.33052 | | | 228.579 | 19.19985 | | | | | | |
| | | | | | | 228.663 | 18.0632 | | | 228.6623 | 19.20588 | | | | | | |
| | | | | | | 228.7463 | 18.15124 | | | 228.7457 | 19.27435 | | | | | | |
| | | | | | | 228.8297 | 18.16118 | | | 228.829 | 19.23416 | | | | | | |
| | | | | | | 228.913 | 18.36768 | | | 228.9123 | 19.22568 | | | | | | |
| | | | | | | 228.9963 | 18.10739 | | | 228.9957 | 19.26403 | | | | | | |
| | | | | | | 229.0797 | 18.37126 | | | 229.079 | 19.28967 | | | | | | |
| | | | | | | 229.163 | 18.08898 | | | 229.1623 | 19.3366 | | | | | | |
| | | | | | | 229.2463 | 18.11828 | | | 229.2457 | 19.33452 | | | | | | |
| | | | | | | 229.3297 | 18.1875 | | | 229.329 | 19.36906 | | | | | | |
| | | | | | | 229.413 | 18.35384 | | | 229.4123 | 19.39217 | | | | | | |
| | | | | | | 229.4963 | 18.10705 | | | 229.4957 | 19.42348 | | | | | | |
| | | | | | | 229.5797 | 18.37804 | | | 229.579 | 19.32504 | | | | | | |
| | | | | | | 229.663 | 18.09303 | | | | | | | | | | |
| | | | | | | 229.7463 | 18.3518 | | | | | | | | | | |
| | | | | | | 229.8297 | 18.12217 | | | | | | | | | | |
| | | | | | | 229.913 | 18.14604 | | | | | | | | | | |
| | | | | | | 229.9963 | 18.37156 | | | | | | | | | | |
| | | | | | | 230.0797 | 18.08368 | | | | | | | | | | |
| | | | | | | 230.163 | 18.35392 | | | | | | | | | | |
| | | | | | | 230.2463 | 18.09235 | | | | | | | | | | |
| | | | | | | 230.3297 | 18.12576 | | | | | | | | | | |
| | | | | | | 230.413 | 18.14994 | | | | | | | | | | |
| | | | | | | 230.4963 | 18.36114 | | | | | | | | | | |
| | | | | | | 230.5797 | 18.11376 | | | | | | | | | | |
| | | | | | | 230.663 | 18.35374 | | | | | | | | | | |
| | | | | | | 230.7463 | 18.09616 | | | | | | | | | | |
| | | | | | | 230.8297 | 18.122 | | | | | | | | | | |
| | | | | | | 230.913 | 18.36508 | | | | | | | | | | |
| | | | | | | 230.9963 | 18.14465 | | | | | | | | | | |
| | | | | | | 231.0797 | 18.21103 | | | | | | | | | | |
| | | | | | | 231.163 | 18.33882 | | | | | | | | | | |
| | | | | | | 231.2463 | 18.09685 | | | | | | | | | | |
| | | | | | | 231.3297 | 18.37885 | | | | | | | | | | |
| | | | | | | 231.413 | 18.09612 | | | | | | | | | | |
| | | | | | | 231.4963 | 18.15542 | | | | | | | | | | |
| | | | | | | 231.5797 | 18.16684 | | | | | | | | | | |
| | | | | | | 231.663 | 18.34992 | | | | | | | | | | |
| | | | | | | 231.7463 | 18.12842 | | | | | | | | | | |
| | | | | | | 231.8297 | 18.36642 | | | | | | | | | | |
| | | | | | | 231.913 | 18.09555 | | | | | | | | | | |
| | | | | | | 231.9963 | 18.13026 | | | | | | | | | | |
| | | | | | | 232.0797 | 18.35539 | | | | | | | | | | |
| | | | | | | 232.163 | 18.19623 | | | | | | | | | | |
| | | | | | | 232.2463 | 18.15522 | | | | | | | | | | |
| | | | | | | 232.3297 | 18.38697 | | | | | | | | | | |
| | | | | | | 232.413 | 18.11401 | | | | | | | | | | |

| | | | | | | | | | | | | | | | | | |
|---|---|---|---|---|---|---|---|---|---|---|---|---|---|---|---|---|---|
| | | | | | | 232.4963 | 18.13323 | | | | | | | | | | |
| | | | | | | 232.5797 | 18.15294 | | | | | | | | | | |
| | | | | | | 232.663 | 18.37229 | | | | | | | | | | |
| | | | | | | 232.7463 | 18.15734 | | | | | | | | | | |
| | | | | | | 232.8297 | 18.17992 | | | | | | | | | | |
| | | | | | | 232.913 | 18.35151 | | | | | | | | | | |
| | | | | | | 232.9963 | 18.12129 | | | | | | | | | | |
| | | | | | | 233.0797 | 18.37033 | | | | | | | | | | |
| | | | | | | 233.163 | 18.16756 | | | | | | | | | | |
| | | | | | | 233.2463 | 18.17347 | | | | | | | | | | |
| | | | | | | 233.3297 | 18.20982 | | | | | | | | | | |
| | | | | | | 233.413 | 18.21413 | | | | | | | | | | |
| | | | | | | 233.4963 | 18.34947 | | | | | | | | | | |
| | | | | | | 233.5797 | 18.3451 | | | | | | | | | | |
| | | | | | | 233.663 | 18.10164 | | | | | | | | | | |
| | | | | | | 233.7463 | 18.1756 | | | | | | | | | | |
| | | | | | | 233.8297 | 18.18418 | | | | | | | | | | |
| | | | | | | 233.913 | 18.27614 | | | | | | | | | | |
| | | | | | | 233.9963 | 18.28885 | | | | | | | | | | |
| | | | | | | 234.0797 | 18.12874 | | | | | | | | | | |
| | | | | | | 234.163 | 18.3294 | | | | | | | | | | |
| | | | | | | 234.2463 | 18.23005 | | | | | | | | | | |
| | | | | | | 234.3297 | 18.24138 | | | | | | | | | | |
| | | | | | | 234.413 | 18.19411 | | | | | | | | | | |
| | | | | | | 234.4963 | 18.27538 | | | | | | | | | | |
| | | | | | | 234.5797 | 18.14203 | | | | | | | | | | |
| | | | | | | 234.663 | 18.20092 | | | | | | | | | | |
| | | | | | | 234.7463 | 18.32517 | | | | | | | | | | |
| | | | | | | 234.8297 | 18.25385 | | | | | | | | | | |
| | | | | | | 234.913 | 18.39628 | | | | | | | | | | |
| | | | | | | 234.9963 | 18.40791 | | | | | | | | | | |
| | | | | | | 235.0797 | 18.28943 | | | | | | | | | | |
| | | | | | | 235.163 | 18.40241 | | | | | | | | | | |
| | | | | | | 235.2463 | 18.46445 | | | | | | | | | | |
| | | | | | | 235.3297 | 18.50444 | | | | | | | | | | |
| | | | | | | 235.413 | 18.38198 | | | | | | | | | | |
| | | | | | | 235.4963 | 18.40401 | | | | | | | | | | |
| | | | | | | 235.5797 | 18.4003 | | | | | | | | | | |
| | | | | | | 235.663 | 18.52916 | | | | | | | | | | |
| | | | | | | 235.7463 | 18.52211 | | | | | | | | | | |
| | | | | | | 235.8297 | 18.35877 | | | | | | | | | | |
| | | | | | | 235.913 | 18.35119 | | | | | | | | | | |
| | | | | | | 235.9963 | 18.42122 | | | | | | | | | | |
| | | | | | | 236.0797 | 18.51491 | | | | | | | | | | |
| | | | | | | 236.163 | 18.58377 | | | | | | | | | | |
| | | | | | | 236.2463 | 18.53011 | | | | | | | | | | |
| | | | | | | 236.3297 | 18.54916 | | | | | | | | | | |
| | | | | | | 236.413 | 18.31651 | | | | | | | | | | |
| | | | | | | 236.4963 | 18.39161 | | | | | | | | | | |
| | | | | | | 236.5797 | 18.46386 | | | | | | | | | | |
| | | | | | | 236.663 | 18.60487 | | | | | | | | | | |

| | | | | | | | | | | | | | | | | | |
|---|---|---|---|---|---|---|---|---|---|---|---|---|---|---|---|---|---|
| | | | | | | 236.7463 | 18.60934 | | | | | | | | | | |
| | | | | | | 236.8297 | 18.54389 | | | | | | | | | | |
| | | | | | | 236.913 | 18.6696 | | | | | | | | | | |
| | | | | | | 236.9963 | 18.62848 | | | | | | | | | | |
| | | | | | | 237.0797 | 18.52978 | | | | | | | | | | |
| | | | | | | 237.163 | 18.62892 | | | | | | | | | | |
| | | | | | | 237.2463 | 18.72989 | | | | | | | | | | |
| | | | | | | 237.3297 | 18.56768 | | | | | | | | | | |
| | | | | | | 237.413 | 18.51742 | | | | | | | | | | |
| | | | | | | 237.4963 | 18.60951 | | | | | | | | | | |
| | | | | | | 237.5797 | 18.52027 | | | | | | | | | | |
| | | | | | | 237.663 | 18.54101 | | | | | | | | | | |
| | | | | | | 237.7463 | 18.62969 | | | | | | | | | | |
| | | | | | | 237.8297 | 18.70324 | | | | | | | | | | |
| | | | | | | 237.913 | 18.7671 | | | | | | | | | | |
| | | | | | | 237.9963 | 18.77138 | | | | | | | | | | |
| | | | | | | 238.0797 | 18.63721 | | | | | | | | | | |
| | | | | | | 238.163 | 18.62723 | | | | | | | | | | |
| | | | | | | 238.2463 | 18.8988 | | | | | | | | | | |
| | | | | | | 238.3297 | 18.91441 | | | | | | | | | | |
| | | | | | | 238.413 | 18.71394 | | | | | | | | | | |
| | | | | | | 238.4963 | 18.693 | | | | | | | | | | |
| | | | | | | 238.5797 | 18.8906 | | | | | | | | | | |
| | | | | | | 238.663 | 18.87242 | | | | | | | | | | |
| | | | | | | 238.7463 | 18.61547 | | | | | | | | | | |
| | | | | | | 238.8297 | 18.66679 | | | | | | | | | | |
| | | | | | | 238.913 | 18.69726 | | | | | | | | | | |
| | | | | | | 238.9963 | 18.74548 | | | | | | | | | | |
| | | | | | | 239.0797 | 18.96225 | | | | | | | | | | |
| | | | | | | 239.163 | 18.65741 | | | | | | | | | | |
| | | | | | | 239.2463 | 18.91744 | | | | | | | | | | |
| | | | | | | 239.3297 | 18.65384 | | | | | | | | | | |
| | | | | | | 239.413 | 18.69831 | | | | | | | | | | |
| | | | | | | 239.4963 | 18.72706 | | | | | | | | | | |
| | | | | | | 239.5797 | 18.95179 | | | | | | | | | | |
| | | | | | | 239.663 | 18.6518 | | | | | | | | | | |
| | | | | | | 239.7463 | 18.93134 | | | | | | | | | | |
| | | | | | | 239.8297 | 18.65207 | | | | | | | | | | |
| | | | | | | 239.913 | 18.68028 | | | | | | | | | | |
| | | | | | | 239.9963 | 18.7094 | | | | | | | | | | |
| | | | | | | 240.0797 | 18.88268 | | | | | | | | | | |
| | | | | | | 240.163 | 18.69247 | | | | | | | | | | |
| | | | | | | 240.2463 | 18.91783 | | | | | | | | | | |
| | | | | | | 240.3297 | 18.65377 | | | | | | | | | | |
| | | | | | | 240.413 | 18.90803 | | | | | | | | | | |
| | | | | | | 240.4963 | 18.64967 | | | | | | | | | | |
| | | | | | | 240.5797 | 18.72121 | | | | | | | | | | |
| | | | | | | 240.663 | 18.72514 | | | | | | | | | | |
| | | | | | | 240.7463 | 18.89469 | | | | | | | | | | |
| | | | | | | 240.8297 | 18.66835 | | | | | | | | | | |
| | | | | | | 240.913 | 18.90176 | | | | | | | | | | |

| | | | | | | | | | | | | | | | | | |
|---|---|---|---|---|---|---|---|---|---|---|---|---|---|---|---|---|---|
| | | | | | | 240.9963 | 18.63224 | | | | | | | | | | |
| | | | | | | 241.0797 | 18.69636 | | | | | | | | | | |
| | | | | | | 241.163 | 18.88836 | | | | | | | | | | |
| | | | | | | 241.2463 | 18.72359 | | | | | | | | | | |
| | | | | | | 241.3297 | 18.69896 | | | | | | | | | | |
| | | | | | | 241.413 | 18.91785 | | | | | | | | | | |
| | | | | | | 241.4963 | 18.90539 | | | | | | | | | | |
| | | | | | | 241.5797 | 18.64211 | | | | | | | | | | |
| | | | | | | 241.663 | 18.7123 | | | | | | | | | | |
| | | | | | | 241.7463 | 18.72948 | | | | | | | | | | |
| | | | | | | 241.8297 | 18.71522 | | | | | | | | | | |
| | | | | | | 241.913 | 18.93925 | | | | | | | | | | |
| | | | | | | 241.9963 | 18.90715 | | | | | | | | | | |
| | | | | | | 242.0797 | 18.63553 | | | | | | | | | | |
| | | | | | | 242.163 | 18.67456 | | | | | | | | | | |
| | | | | | | 242.2463 | 18.70249 | | | | | | | | | | |
| | | | | | | 242.3297 | 18.71767 | | | | | | | | | | |
| | | | | | | 242.413 | 18.90331 | | | | | | | | | | |
| | | | | | | 242.4963 | 18.67491 | | | | | | | | | | |
| | | | | | | 242.5797 | 18.92336 | | | | | | | | | | |
| | | | | | | 242.663 | 18.67808 | | | | | | | | | | |
| | | | | | | 242.7463 | 18.69351 | | | | | | | | | | |
| | | | | | | 242.8297 | 18.74961 | | | | | | | | | | |
| | | | | | | 242.913 | 18.72341 | | | | | | | | | | |
| | | | | | | 242.9963 | 18.88257 | | | | | | | | | | |
| | | | | | | 243.0797 | 18.89349 | | | | | | | | | | |
| | | | | | | 243.163 | 18.69131 | | | | | | | | | | |
| | | | | | | 243.2463 | 18.66677 | | | | | | | | | | |
| | | | | | | 243.3297 | 18.72205 | | | | | | | | | | |
| | | | | | | 243.413 | 18.73112 | | | | | | | | | | |
| | | | | | | 243.4963 | 18.77128 | | | | | | | | | | |
| | | | | | | 243.5797 | 18.74571 | | | | | | | | | | |
| | | | | | | 243.663 | 18.88726 | | | | | | | | | | |
| | | | | | | 243.7463 | 18.88318 | | | | | | | | | | |
| | | | | | | 243.8297 | 18.64344 | | | | | | | | | | |
| | | | | | | 243.913 | 18.68564 | | | | | | | | | | |
| | | | | | | 243.9963 | 18.80427 | | | | | | | | | | |
| | | | | | | 244.0797 | 18.8172 | | | | | | | | | | |
| | | | | | | 244.163 | 18.87496 | | | | | | | | | | |
| | | | | | | 244.2463 | 18.75908 | | | | | | | | | | |
| | | | | | | 244.3297 | 18.65214 | | | | | | | | | | |
| | | | | | | 244.413 | 18.69885 | | | | | | | | | | |
| | | | | | | 244.4963 | 18.78612 | | | | | | | | | | |
| | | | | | | 244.5797 | 18.81239 | | | | | | | | | | |
| | | | | | | 244.663 | 18.94827 | | | | | | | | | | |
| | | | | | | 244.7463 | 18.76645 | | | | | | | | | | |
| | | | | | | 244.8297 | 18.72378 | | | | | | | | | | |
| | | | | | | 244.913 | 18.87166 | | | | | | | | | | |
| | | | | | | 244.9963 | 18.84938 | | | | | | | | | | |
| | | | | | | 245.0797 | 18.9823 | | | | | | | | | | |
| | | | | | | 245.163 | 19.01603 | | | | | | | | | | |

| | | | | | | | | | | | | | | | | | |
|---|---|---|---|---|---|---|---|---|---|---|---|---|---|---|---|---|---|
| | | | | | | 245.2463 | 19.03028 | | | | | | | | | | |
| | | | | | | 245.3297 | 18.92342 | | | | | | | | | | |
| | | | | | | 245.413 | 19.04921 | | | | | | | | | | |
| | | | | | | 245.4963 | 18.8825 | | | | | | | | | | |
| | | | | | | 245.5797 | 19.07245 | | | | | | | | | | |
| | | | | | | 245.663 | 19.07145 | | | | | | | | | | |
| | | | | | | 245.7463 | 19.1389 | | | | | | | | | | |
| | | | | | | 245.8297 | 19.17656 | | | | | | | | | | |
| | | | | | | 245.913 | 19.15281 | | | | | | | | | | |
| | | | | | | 245.9963 | 19.19279 | | | | | | | | | | |
| | | | | | | 246.0797 | 19.09791 | | | | | | | | | | |
| | | | | | | 246.163 | 19.13386 | | | | | | | | | | |
| | | | | | | 246.2463 | 19.13511 | | | | | | | | | | |
| | | | | | | 246.3297 | 19.19106 | | | | | | | | | | |
| | | | | | | 246.413 | 19.12437 | | | | | | | | | | |
| | | | | | | 246.4963 | 19.10263 | | | | | | | | | | |
| | | | | | | 246.5797 | 19.23318 | | | | | | | | | | |
| | | | | | | 246.663 | 19.07461 | | | | | | | | | | |
| | | | | | | 246.7463 | 19.18493 | | | | | | | | | | |
| | | | | | | 246.8297 | 19.30188 | | | | | | | | | | |
| | | | | | | 246.913 | 19.35565 | | | | | | | | | | |
| | | | | | | 246.9963 | 19.38804 | | | | | | | | | | |
| | | | | | | 247.0797 | 19.39613 | | | | | | | | | | |
| | | | | | | 247.163 | 19.17932 | | | | | | | | | | |
| | | | | | | 247.2463 | 19.40956 | | | | | | | | | | |
| | | | | | | 247.3297 | 19.27987 | | | | | | | | | | |
| | | | | | | 247.413 | 19.30384 | | | | | | | | | | |
| | | | | | | 247.4963 | 19.27227 | | | | | | | | | | |
| | | | | | | 247.5797 | 19.45644 | | | | | | | | | | |
| | | | | | | 247.663 | 19.20746 | | | | | | | | | | |
| | | | | | | 247.7463 | 19.44906 | | | | | | | | | | |
| | | | | | | 247.8297 | 19.31902 | | | | | | | | | | |
| | | | | | | 247.913 | 19.34599 | | | | | | | | | | |
| | | | | | | 247.9963 | 19.26379 | | | | | | | | | | |
| | | | | | | 248.0797 | 19.27725 | | | | | | | | | | |
| | | | | | | 248.163 | 19.25612 | | | | | | | | | | |
| | | | | | | 248.2463 | 19.43964 | | | | | | | | | | |
| | | | | | | 248.3297 | 19.26013 | | | | | | | | | | |
| | | | | | | 248.413 | 19.22993 | | | | | | | | | | |
| | | | | | | 248.4963 | 19.27633 | | | | | | | | | | |
| | | | | | | 248.5797 | 19.44892 | | | | | | | | | | |
| | | | | | | 248.663 | 19.29366 | | | | | | | | | | |
| | | | | | | 248.7463 | 19.44306 | | | | | | | | | | |
| | | | | | | 248.8297 | 19.25074 | | | | | | | | | | |
| | | | | | | 248.913 | 19.24756 | | | | | | | | | | |
| | | | | | | 248.9963 | 19.26073 | | | | | | | | | | |
| | | | | | | 249.0797 | 19.44329 | | | | | | | | | | |
| | | | | | | 249.163 | 19.36421 | | | | | | | | | | |
| | | | | | | 249.2463 | 19.3175 | | | | | | | | | | |
| | | | | | | 249.3297 | 19.22603 | | | | | | | | | | |
| | | | | | | 249.413 | 19.24626 | | | | | | | | | | |

| | | | | | | | | | | | | | | | | | |
|---|---|---|---|---|---|---|---|---|---|---|---|---|---|---|---|---|---|
| | | | | | | 249.4963 | 19.45825 | | | | | | | | | | |
| | | | | | | 249.5797 | 19.28338 | | | | | | | | | | |
| | | | | | | 249.663 | 19.30032 | | | | | | | | | | |
| | | | | | | 249.7463 | 19.33402 | | | | | | | | | | |
| | | | | | | 249.8297 | 19.44569 | | | | | | | | | | |
| | | | | | | 249.913 | 19.24077 | | | | | | | | | | |
| | | | | | | 249.9963 | 19.24935 | | | | | | | | | | |
| | | | | | | 250.0797 | 19.44057 | | | | | | | | | | |
| | | | | | | 250.163 | 19.38532 | | | | | | | | | | |
| | | | | | | 250.2463 | 19.46222 | | | | | | | | | | |
| | | | | | | 250.3297 | 19.5027 | | | | | | | | | | |
| | | | | | | 250.413 | 19.4138 | | | | | | | | | | |
| | | | | | | 250.4963 | 19.4675 | | | | | | | | | | |
| | | | | | | 250.5797 | 19.5011 | | | | | | | | | | |
| | | | | | | 250.663 | 19.59008 | | | | | | | | | | |
| | | | | | | 250.7463 | 19.50141 | | | | | | | | | | |
| | | | | | | 250.8297 | 19.63393 | | | | | | | | | | |
| | | | | | | 250.913 | 19.46269 | | | | | | | | | | |
| | | | | | | 250.9963 | 19.50233 | | | | | | | | | | |
| | | | | | | 251.0797 | 19.58716 | | | | | | | | | | |
| | | | | | | 251.163 | 19.44074 | | | | | | | | | | |
| | | | | | | 251.2463 | 19.4874 | | | | | | | | | | |
| | | | | | | 251.3297 | 19.54119 | | | | | | | | | | |
| | | | | | | 251.413 | 19.57643 | | | | | | | | | | |
| | | | | | | 251.4963 | 19.61933 | | | | | | | | | | |
| | | | | | | 251.5797 | 19.5182 | | | | | | | | | | |
| | | | | | | 251.663 | 19.64661 | | | | | | | | | | |
| | | | | | | 251.7463 | 19.56042 | | | | | | | | | | |
| | | | | | | 251.8297 | 19.73689 | | | | | | | | | | |
| | | | | | | 251.913 | 19.68026 | | | | | | | | | | |
| | | | | | | 251.9963 | 19.67729 | | | | | | | | | | |
| | | | | | | 252.0797 | 19.78146 | | | | | | | | | | |
| | | | | | | 252.163 | 19.63447 | | | | | | | | | | |
| | | | | | | 252.2463 | 19.67959 | | | | | | | | | | |
| | | | | | | 252.3297 | 19.79831 | | | | | | | | | | |
| | | | | | | 252.413 | 19.73011 | | | | | | | | | | |
| | | | | | | 252.4963 | 19.82153 | | | | | | | | | | |
| | | | | | | 252.5797 | 19.80285 | | | | | | | | | | |
| | | | | | | 252.663 | 19.6692 | | | | | | | | | | |
| | | | | | | 252.7463 | 19.61227 | | | | | | | | | | |
| | | | | | | 252.8297 | 19.62608 | | | | | | | | | | |
| | | | | | | 252.913 | 19.63532 | | | | | | | | | | |
| | | | | | | 252.9963 | 19.67351 | | | | | | | | | | |
| | | | | | | 253.0797 | 19.74477 | | | | | | | | | | |
| | | | | | | 253.163 | 19.69104 | | | | | | | | | | |
| | | | | | | 253.2463 | 19.79393 | | | | | | | | | | |
| | | | | | | 253.3297 | 19.64555 | | | | | | | | | | |
| | | | | | | 253.413 | 19.6305 | | | | | | | | | | |
| | | | | | | 253.4963 | 19.80807 | | | | | | | | | | |
| | | | | | | 253.5797 | 19.75968 | | | | | | | | | | |
| | | | | | | 253.663 | 19.88515 | | | | | | | | | | |

| | | | | | | | | | | | | | | | | | |
|---|---|---|---|---|---|---|---|---|---|---|---|---|---|---|---|---|---|
| | | | | | | 253.7463 | 19.93741 | | | | | | | | | | |
| | | | | | | 253.8297 | 19.92015 | | | | | | | | | | |
| | | | | | | 253.913 | 19.769 | | | | | | | | | | |
| | | | | | | 253.9963 | 19.76033 | | | | | | | | | | |
| | | | | | | 254.0797 | 19.98906 | | | | | | | | | | |
| | | | | | | 254.163 | 19.73977 | | | | | | | | | | |
| | | | | | | 254.2463 | 19.76959 | | | | | | | | | | |
| | | | | | | 254.3297 | 19.79439 | | | | | | | | | | |
| | | | | | | 254.413 | 20.03288 | | | | | | | | | | |
| | | | | | | 254.4963 | 20.00912 | | | | | | | | | | |
| | | | | | | 254.5797 | 19.71373 | | | | | | | | | | |
| | | | | | | 254.663 | 19.78446 | | | | | | | | | | |
| | | | | | | 254.7463 | 19.8436 | | | | | | | | | | |
| | | | | | | 254.8297 | 19.81797 | | | | | | | | | | |
| | | | | | | 254.913 | 20.03915 | | | | | | | | | | |
| | | | | | | 254.9963 | 20.01466 | | | | | | | | | | |
| | | | | | | 255.0797 | 19.71996 | | | | | | | | | | |
| | | | | | | 255.163 | 19.76509 | | | | | | | | | | |
| | | | | | | 255.2463 | 19.79387 | | | | | | | | | | |
| | | | | | | 255.3297 | 19.97997 | | | | | | | | | | |
| | | | | | | 255.413 | 19.80215 | | | | | | | | | | |
| | | | | | | 255.4963 | 19.78964 | | | | | | | | | | |
| | | | | | | 255.5797 | 20.03588 | | | | | | | | | | |
| | | | | | | 255.663 | 19.78248 | | | | | | | | | | |
| | | | | | | 255.7463 | 19.78965 | | | | | | | | | | |
| | | | | | | 255.8297 | 19.8143 | | | | | | | | | | |
| | | | | | | 255.913 | 19.99741 | | | | | | | | | | |
| | | | | | | 255.9963 | 19.78636 | | | | | | | | | | |
| | | | | | | 256.0797 | 20.00721 | | | | | | | | | | |
| | | | | | | 256.163 | 19.76299 | | | | | | | | | | |
| | | | | | | 256.2463 | 19.79346 | | | | | | | | | | |
| | | | | | | 256.3297 | 20.02938 | | | | | | | | | | |
| | | | | | | 256.413 | 19.77015 | | | | | | | | | | |
| | | | | | | 256.4963 | 19.79896 | | | | | | | | | | |
| | | | | | | 256.5797 | 20.00073 | | | | | | | | | | |
| | | | | | | 256.663 | 19.78724 | | | | | | | | | | |
| | | | | | | 256.7463 | 20.01044 | | | | | | | | | | |
| | | | | | | 256.8297 | 19.72885 | | | | | | | | | | |
| | | | | | | 256.913 | 19.78563 | | | | | | | | | | |
| | | | | | | 256.9963 | 19.81697 | | | | | | | | | | |
| | | | | | | 257.0797 | 19.83246 | | | | | | | | | | |
| | | | | | | 257.163 | 20.00076 | | | | | | | | | | |
| | | | | | | 257.2463 | 19.77619 | | | | | | | | | | |
| | | | | | | 257.3297 | 20.03198 | | | | | | | | | | |
| | | | | | | 257.413 | 19.77414 | | | | | | | | | | |
| | | | | | | 257.4963 | 19.79901 | | | | | | | | | | |
| | | | | | | 257.5797 | 19.83252 | | | | | | | | | | |
| | | | | | | 257.663 | 19.81942 | | | | | | | | | | |
| | | | | | | 257.7463 | 20.0247 | | | | | | | | | | |
| | | | | | | 257.8297 | 19.78032 | | | | | | | | | | |
| | | | | | | 257.913 | 20.01505 | | | | | | | | | | |

| | | | | | | | | | | | | | | | | | |
|---|---|---|---|---|---|---|---|---|---|---|---|---|---|---|---|---|---|
| | | | | | | 257.9963 | 19.78529 | | | | | | | | | | |
| | | | | | | 258.0797 | 19.79664 | | | | | | | | | | |
| | | | | | | 258.163 | 19.81515 | | | | | | | | | | |
| | | | | | | 258.2463 | 19.81602 | | | | | | | | | | |
| | | | | | | 258.3297 | 20.01349 | | | | | | | | | | |
| | | | | | | 258.413 | 19.78094 | | | | | | | | | | |
| | | | | | | 258.4963 | 19.98753 | | | | | | | | | | |
| | | | | | | 258.5797 | 19.85869 | | | | | | | | | | |
| | | | | | | 258.663 | 19.79318 | | | | | | | | | | |
| | | | | | | 258.7463 | 19.83851 | | | | | | | | | | |
| | | | | | | 258.8297 | 19.85841 | | | | | | | | | | |
| | | | | | | 258.913 | 19.9887 | | | | | | | | | | |
| | | | | | | 258.9963 | 19.7877 | | | | | | | | | | |
| | | | | | | 259.0797 | 20.0004 | | | | | | | | | | |
| | | | | | | 259.163 | 19.76757 | | | | | | | | | | |
| | | | | | | 259.2463 | 19.82373 | | | | | | | | | | |
| | | | | | | 259.3297 | 19.88375 | | | | | | | | | | |
| | | | | | | 259.413 | 19.84883 | | | | | | | | | | |
| | | | | | | 259.4963 | 19.79642 | | | | | | | | | | |
| | | | | | | 259.5797 | 20.0078 | | | | | | | | | | |
| | | | | | | 259.663 | 19.77651 | | | | | | | | | | |
| | | | | | | 259.7463 | 19.84883 | | | | | | | | | | |
| | | | | | | 259.8297 | 19.90585 | | | | | | | | | | |
| | | | | | | 259.913 | 19.88238 | | | | | | | | | | |
| | | | | | | 259.9963 | 19.86845 | | | | | | | | | | |
| | | | | | | 260.0797 | 19.78597 | | | | | | | | | | |
| | | | | | | 260.163 | 19.79561 | | | | | | | | | | |
| | | | | | | 260.2463 | 19.81046 | | | | | | | | | | |
| | | | | | | 260.3297 | 19.87264 | | | | | | | | | | |
| | | | | | | 260.413 | 19.94203 | | | | | | | | | | |
| | | | | | | 260.4963 | 19.90683 | | | | | | | | | | |
| | | | | | | 260.5797 | 19.84767 | | | | | | | | | | |
| | | | | | | 260.663 | 19.85243 | | | | | | | | | | |
| | | | | | | 260.7463 | 19.81593 | | | | | | | | | | |
| | | | | | | 260.8297 | 19.99332 | | | | | | | | | | |
| | | | | | | 260.913 | 19.8998 | | | | | | | | | | |
| | | | | | | 260.9963 | 20.06347 | | | | | | | | | | |
| | | | | | | 261.0797 | 20.07769 | | | | | | | | | | |
| | | | | | | 261.163 | 20.12103 | | | | | | | | | | |
| | | | | | | 261.2463 | 20.11907 | | | | | | | | | | |
| | | | | | | 261.3297 | 19.98421 | | | | | | | | | | |
| | | | | | | 261.413 | 20.05289 | | | | | | | | | | |
| | | | | | | 261.4963 | 20.09189 | | | | | | | | | | |
| | | | | | | 261.5797 | 20.07108 | | | | | | | | | | |
| | | | | | | 261.663 | 20.24195 | | | | | | | | | | |
| | | | | | | 261.7463 | 20.20411 | | | | | | | | | | |
| | | | | | | 261.8297 | 19.99321 | | | | | | | | | | |
| | | | | | | 261.913 | 20.00655 | | | | | | | | | | |
| | | | | | | 261.9963 | 20.04865 | | | | | | | | | | |
| | | | | | | 262.0797 | 20.1413 | | | | | | | | | | |
| | | | | | | 262.163 | 20.19972 | | | | | | | | | | |

| | | | | | | | | | | | | | | | | | |
|---|---|---|---|---|---|---|---|---|---|---|---|---|---|---|---|---|---|
| | | | | | | 262.2463 | 20.09319 | | | | | | | | | | |
| | | | | | | 262.3297 | 20.13225 | | | | | | | | | | |
| | | | | | | 262.413 | 20.14337 | | | | | | | | | | |
| | | | | | | 262.4963 | 20.26212 | | | | | | | | | | |
| | | | | | | 262.5797 | 20.31127 | | | | | | | | | | |
| | | | | | | 262.663 | 20.23822 | | | | | | | | | | |
| | | | | | | 262.7463 | 20.22538 | | | | | | | | | | |
| | | | | | | 262.8297 | 20.34449 | | | | | | | | | | |
| | | | | | | 262.913 | 20.16262 | | | | | | | | | | |
| | | | | | | 262.9963 | 20.32471 | | | | | | | | | | |
| | | | | | | 263.0797 | 20.26334 | | | | | | | | | | |
| | | | | | | 263.163 | 20.1504 | | | | | | | | | | |
| | | | | | | 263.2463 | 20.25261 | | | | | | | | | | |
| | | | | | | 263.3297 | 20.42131 | | | | | | | | | | |
| | | | | | | 263.413 | 20.33143 | | | | | | | | | | |
| | | | | | | 263.4963 | 20.19185 | | | | | | | | | | |
| | | | | | | 263.5797 | 20.2351 | | | | | | | | | | |
| | | | | | | 263.663 | 20.17603 | | | | | | | | | | |
| | | | | | | 263.7463 | 20.19524 | | | | | | | | | | |
| | | | | | | 263.8297 | 20.25028 | | | | | | | | | | |
| | | | | | | 263.913 | 20.27921 | | | | | | | | | | |
| | | | | | | 263.9963 | 20.19305 | | | | | | | | | | |
| | | | | | | 264.0797 | 20.16744 | | | | | | | | | | |
| | | | | | | 264.163 | 20.19852 | | | | | | | | | | |
| | | | | | | 264.2463 | 20.20087 | | | | | | | | | | |
| | | | | | | 264.3297 | 20.21356 | | | | | | | | | | |
| | | | | | | 264.413 | 20.25961 | | | | | | | | | | |
| | | | | | | 264.4963 | 20.22938 | | | | | | | | | | |
| | | | | | | 264.5797 | 20.3575 | | | | | | | | | | |
| | | | | | | 264.663 | 20.17151 | | | | | | | | | | |
| | | | | | | 264.7463 | 20.20236 | | | | | | | | | | |
| | | | | | | 264.8297 | 20.20056 | | | | | | | | | | |
| | | | | | | 264.913 | 20.23795 | | | | | | | | | | |
| | | | | | | 264.9963 | 20.21957 | | | | | | | | | | |
| | | | | | | 265.0797 | 20.36205 | | | | | | | | | | |
| | | | | | | 265.163 | 20.19373 | | | | | | | | | | |
| | | | | | | 265.2463 | 20.34652 | | | | | | | | | | |
| | | | | | | 265.3297 | 20.19391 | | | | | | | | | | |
| | | | | | | 265.413 | 20.19693 | | | | | | | | | | |
| | | | | | | 265.4963 | 20.24984 | | | | | | | | | | |
| | | | | | | 265.5797 | 20.27682 | | | | | | | | | | |
| | | | | | | 265.663 | 20.28584 | | | | | | | | | | |
| | | | | | | 265.7463 | 20.29164 | | | | | | | | | | |
| | | | | | | 265.8297 | 20.35768 | | | | | | | | | | |
| | | | | | | 265.913 | 20.47939 | | | | | | | | | | |
| | | | | | | 265.9963 | 20.54195 | | | | | | | | | | |
| | | | | | | 266.0797 | 20.35699 | | | | | | | | | | |
| | | | | | | 266.163 | 20.38027 | | | | | | | | | | |
| | | | | | | 266.2463 | 20.5398 | | | | | | | | | | |
| | | | | | | 266.3297 | 20.53511 | | | | | | | | | | |
| | | | | | | 266.413 | 20.27772 | | | | | | | | | | |

| | | | | | | | | | | | | | | | | | |
|---|---|---|---|---|---|---|---|---|---|---|---|---|---|---|---|---|---|
| | | | | | | 266.4963 | 20.31203 | | | | | | | | | | |
| | | | | | | 266.5797 | 20.34051 | | | | | | | | | | |
| | | | | | | 266.663 | 20.36375 | | | | | | | | | | |
| | | | | | | 266.7463 | 20.54374 | | | | | | | | | | |
| | | | | | | 266.8297 | 20.3153 | | | | | | | | | | |
| | | | | | | 266.913 | 20.56273 | | | | | | | | | | |
| | | | | | | 266.9963 | 20.31859 | | | | | | | | | | |
| | | | | | | 267.0797 | 20.32228 | | | | | | | | | | |
| | | | | | | 267.163 | 20.54232 | | | | | | | | | | |
| | | | | | | 267.2463 | 20.32884 | | | | | | | | | | |
| | | | | | | 267.3297 | 20.32575 | | | | | | | | | | |
| | | | | | | 267.413 | 20.5639 | | | | | | | | | | |
| | | | | | | 267.4963 | 20.32424 | | | | | | | | | | |
| | | | | | | 267.5797 | 20.5432 | | | | | | | | | | |
| | | | | | | 267.663 | 20.2784 | | | | | | | | | | |
| | | | | | | 267.7463 | 20.32381 | | | | | | | | | | |
| | | | | | | 267.8297 | 20.40967 | | | | | | | | | | |
| | | | | | | 267.913 | 20.54934 | | | | | | | | | | |
| | | | | | | 267.9963 | 20.31766 | | | | | | | | | | |
| | | | | | | 268.0797 | 20.5572 | | | | | | | | | | |
| | | | | | | 268.163 | 20.28615 | | | | | | | | | | |
| | | | | | | 268.2463 | 20.32697 | | | | | | | | | | |
| | | | | | | 268.3297 | 20.38133 | | | | | | | | | | |
| | | | | | | 268.413 | 20.39479 | | | | | | | | | | |
| | | | | | | 268.4963 | 20.55441 | | | | | | | | | | |
| | | | | | | 268.5797 | 20.31745 | | | | | | | | | | |
| | | | | | | 268.663 | 20.56852 | | | | | | | | | | |
| | | | | | | 268.7463 | 20.32313 | | | | | | | | | | |
| | | | | | | 268.8297 | 20.36307 | | | | | | | | | | |
| | | | | | | 268.913 | 20.40887 | | | | | | | | | | |
| | | | | | | 268.9963 | 20.5597 | | | | | | | | | | |
| | | | | | | 269.0797 | 20.32691 | | | | | | | | | | |
| | | | | | | 269.163 | 20.54706 | | | | | | | | | | |
| | | | | | | 269.2463 | 20.33888 | | | | | | | | | | |
| | | | | | | 269.3297 | 20.37621 | | | | | | | | | | |
| | | | | | | 269.413 | 20.35445 | | | | | | | | | | |
| | | | | | | 269.4963 | 20.38862 | | | | | | | | | | |
| | | | | | | 269.5797 | 20.53515 | | | | | | | | | | |
| | | | | | | 269.663 | 20.34988 | | | | | | | | | | |
| | | | | | | 269.7463 | 20.52086 | | | | | | | | | | |
| | | | | | | 269.8297 | 20.32041 | | | | | | | | | | |
| | | | | | | 269.913 | 20.35757 | | | | | | | | | | |
| | | | | | | 269.9963 | 20.34569 | | | | | | | | | | |
| | | | | | | 270.0797 | 20.54257 | | | | | | | | | | |
| | | | | | | 270.163 | 20.38216 | | | | | | | | | | |
| | | | | | | 270.2463 | 20.36626 | | | | | | | | | | |
| | | | | | | 270.3297 | 20.5667 | | | | | | | | | | |
| | | | | | | 270.413 | 20.32416 | | | | | | | | | | |
| | | | | | | 270.4963 | 20.37926 | | | | | | | | | | |
| | | | | | | 270.5797 | 20.41502 | | | | | | | | | | |
| | | | | | | 270.663 | 20.45105 | | | | | | | | | | |

| | | | | | | | | | | | | | | | | | |
|---|---|---|---|---|---|---|---|---|---|---|---|---|---|---|---|---|---|
| | | | | | | 270.7463 | 20.36198 | | | | | | | | | | |
| | | | | | | 270.8297 | 20.3371 | | | | | | | | | | |
| | | | | | | 270.913 | 20.56054 | | | | | | | | | | |
| | | | | | | 270.9963 | 20.37958 | | | | | | | | | | |
| | | | | | | 271.0797 | 20.38908 | | | | | | | | | | |
| | | | | | | 271.163 | 20.37303 | | | | | | | | | | |
| | | | | | | 271.2463 | 20.42697 | | | | | | | | | | |
| | | | | | | 271.3297 | 20.53391 | | | | | | | | | | |
| | | | | | | 271.413 | 20.35096 | | | | | | | | | | |
| | | | | | | 271.4963 | 20.51099 | | | | | | | | | | |
| | | | | | | 271.5797 | 20.4735 | | | | | | | | | | |
| | | | | | | 271.663 | 20.36684 | | | | | | | | | | |
| | | | | | | 271.7463 | 20.42358 | | | | | | | | | | |
| | | | | | | 271.8297 | 20.44465 | | | | | | | | | | |
| | | | | | | 271.913 | 20.5201 | | | | | | | | | | |
| | | | | | | 271.9963 | 20.40022 | | | | | | | | | | |
| | | | | | | 272.0797 | 20.35769 | | | | | | | | | | |

## Exp3_Output Force of inflatable membrane:

| Sugar Concentration 21% | | | | | | Sugar Concentration 12% | | | | | | Sugar Concentration 3% | | | | | |
|---|---|---|---|---|---|---|---|---|---|---|---|---|---|---|---|---|---|
| T1 | | T2 | | T3 | | T1 | | T2 | | T3 | | T1 | | T2 | | T3 | |
| t (s) | F (N) | t (s) | F (N) | t (s) | F (N) | t (s) | F (N) | t (s) | F (N) | t (s) | F (N) | t (s) | F (N) | t (s) | F (N) | t (s) | F (N) |
| 0 | -0.12748648 | 0 | -0.11767982 | 0 | -0.11767982 | 0 | 0.19613304 | 0 | 0 | 0 | 0.01574017 | 0.00 | 0.04903326 | 0 | 0.0196133 | 0 | 0.04903326 |
| 0.1 | -0.13729313 | 0.1 | -0.10787317 | 0.1 | -0.10787317 | 0.1 | 0.225553 | 0.1 | -0.0196133 | 2.760736 | 0.12626584 | 0.10 | 0.04903326 | 0.1 | 0.00980665 | 0.1 | 0.04903326 |
| 0.2 | -0.12748648 | 0.2 | -0.08825987 | 0.2 | -0.08825987 | 0.2 | 0.14709978 | 0.2 | -0.02941996 | 5.521472 | 2.9144847 | 0.20 | 0.07845322 | 0.2 | 0.00980665 | 0.2 | 0.07845322 |
| 0.3 | -0.12748648 | 0.3 | -0.07845322 | 0.3 | -0.07845322 | 0.3 | 0.05883991 | 0.3 | 0 | 8.282208 | 2.93325375 | 0.30 | 0.04903326 | 0.3 | 0 | 0.3 | 0.04903326 |
| 0.4 | -0.12748648 | 0.4 | -0.08825987 | 0.4 | -0.08825987 | 0.4 | -0.0196133 | 0.4 | -0.02941996 | 11.042944 | 1.91128745 | 0.40 | 0.03922661 | 0.4 | 0.03922661 | 0.4 | 0.03922661 |
| 0.5 | -0.08825987 | 0.5 | -0.09806652 | 0.5 | -0.09806652 | 0.5 | -0.0196133 | 0.5 | -0.02941996 | 13.80368 | 1.86147417 | 0.50 | 0.0196133 | 0.5 | 0.00980665 | 0.5 | 0.0196133 |
| 0.6 | -0.09806652 | 0.6 | -0.11767982 | 0.6 | -0.11767982 | 0.6 | 0.00980665 | 0.6 | -0.03922661 | 16.564416 | 1.84750067 | 0.60 | -0.00980665 | 0.6 | 0.00980665 | 0.6 | -0.00980665 |
| 0.7 | -0.07845322 | 0.7 | -0.10787317 | 0.7 | -0.10787317 | 0.7 | 0.05883991 | 0.7 | -0.03922661 | 19.325152 | 1.86888545 | 0.70 | -0.03922661 | 0.7 | 0.00980665 | 0.7 | -0.03922661 |
| 0.8 | -0.05883991 | 0.8 | -0.10787317 | 0.8 | -0.10787317 | 0.8 | 0.10787317 | 0.8 | 0 | 22.085888 | 1.83504161 | 0.80 | -0.0196133 | 0.8 | 0.05883991 | 0.8 | -0.0196133 |
| 0.9 | -0.08825987 | 0.9 | -0.09806652 | 0.9 | -0.09806652 | 0.9 | 0.12748648 | 0.9 | -0.00980665 | 24.846624 | 1.7329827 | 0.90 | 0.0196133 | 0.9 | 0.03922661 | 0.9 | 0.0196133 |
| 1 | -0.09806652 | 1 | -0.08825987 | 1 | -0.08825987 | 1 | 0.10787317 | 1 | -0.0196133 | 27.60736 | 3.21425974 | 1.00 | 0.02941996 | 1 | 0.03922661 | 1 | 0.02941996 |
| 1.1 | -0.12748648 | 1.1 | -0.06864656 | 1.1 | -0.06864656 | 1.1 | 0.12748648 | 1.1 | -0.02941996 | 30.368096 | 3.21425974 | 1.10 | 0.04903326 | 1.1 | 0.03922661 | 1.1 | 0.04903326 |
| 1.2 | -0.08825987 | 1.2 | -0.07845322 | 1.2 | -0.07845322 | 1.2 | 0.12748648 | 1.2 | -0.03922661 | 33.128832 | 3.21100148 | 1.20 | 0.04903326 | 1.2 | 0.03922661 | 1.2 | 0.04903326 |
| 1.3 | -0.07845322 | 1.3 | -0.07845322 | 1.3 | -0.07845322 | 1.3 | 0.10787317 | 1.3 | -0.04903326 | 35.889568 | 3.1398955 | 1.30 | 0.02941996 | 1.3 | 0.02941996 | 1.3 | 0.02941996 |
| 1.4 | -0.09806652 | 1.4 | -0.08825987 | 1.4 | -0.08825987 | 1.4 | 0.11767982 | 1.4 | -0.02941996 | 38.650304 | 3.21283728 | 1.40 | 0.04903326 | 1.4 | 0.02941996 | 1.4 | 0.04903326 |
| 1.5 | -0.08825987 | 1.5 | -0.08825987 | 1.5 | -0.08825987 | 1.5 | 0.11767982 | 1.5 | -0.02941996 | 41.41104 | 3.21297497 | 1.50 | 0.04903326 | 1.5 | -0.00980665 | 1.5 | 0.04903326 |
| 1.6 | -0.10787317 | 1.6 | -0.08825987 | 1.6 | -0.08825987 | 1.6 | 0.12748648 | 1.6 | -0.0196133 | 44.171776 | 3.21364025 | 1.60 | 0.04903326 | 1.6 | -0.00980665 | 1.6 | 0.04903326 |
| 1.7 | -0.08825987 | 1.7 | -0.07845322 | 1.7 | -0.07845322 | 1.7 | 0.07845322 | 1.7 | -0.03922661 | 46.932512 | 3.21136874 | 1.70 | 0.07845322 | 1.7 | 0.0196133 | 1.7 | 0.07845322 |
| 1.8 | -0.09806652 | 1.8 | -0.07845322 | 1.8 | -0.07845322 | 1.8 | 0.02941996 | 1.8 | -0.03922661 | 49.693248 | 3.21263076 | 1.80 | 0.12748648 | 1.8 | 0.0196133 | 1.8 | 0.12748648 |
| 1.9 | -0.10787317 | 1.9 | -0.06864656 | 1.9 | -0.06864656 | 1.9 | 0.0196133 | 1.9 | -0.03922661 | 52.453984 | 3.21123105 | 1.90 | 0.18632639 | 1.9 | 0.04903326 | 1.9 | 0.18632639 |
| 2 | -0.10787317 | 2 | -0.08825987 | 2 | -0.08825987 | 2 | 0.0196133 | 2 | -0.0196133 | 55.21472 | 3.21166715 | 2.00 | 0.2451663 | 2 | 0.02941996 | 2 | 0.2451663 |
| 2.1 | -0.11767982 | 2.1 | -0.11767982 | 2.1 | -0.11767982 | 2.1 | 0.0196133 | 2.1 | -0.0196133 | 57.975456 | 3.20918911 | 2.10 | 0.2647796 | 2.1 | 0.03922661 | 2.1 | 0.2647796 |
| 2.2 | -0.09806652 | 2.2 | -0.11767982 | 2.2 | -0.11767982 | 2.2 | 0.0196133 | 2.2 | -0.03922661 | 60.736192 | 3.21210316 | 2.20 | 0.17651974 | 2.2 | 0.07845322 | 2.2 | 0.17651974 |
| 2.3 | -0.13729313 | 2.3 | 0.0196133 | 2.3 | 0.0196133 | 2.3 | -0.02941996 | 2.3 | -0.04903326 | 63.496928 | 3.21338793 | 2.30 | 0.08825987 | 2.3 | 0.07845322 | 2.3 | 0.08825987 |
| 2.4 | -0.13729313 | 2.4 | 0.10787317 | 2.4 | 0.10787317 | 2.4 | -0.08825987 | 2.4 | -0.05883991 | 66.257664 | 3.2141909 | 2.40 | 0.04903326 | 2.4 | 0.05883991 | 2.4 | 0.04903326 |
| 2.5 | -0.11767982 | 2.5 | 0.30400621 | 2.5 | 0.30400621 | 2.5 | -0.09806652 | 2.5 | -0.06864656 | 69.0184 | -0.02861228 | 2.50 | 0.06864656 | 2.5 | 0.03922661 | 2.5 | 0.06864656 |
| 2.6 | -0.11767982 | 2.6 | 0.48052595 | 2.6 | 0.48052595 | 2.6 | -0.11767982 | 2.6 | -0.04903326 | 71.779136 | -0.08542388 | 2.60 | 0.03922661 | 2.6 | 0.03922661 | 2.6 | 0.03922661 |
| 2.7 | -0.10787317 | 2.7 | 0.68646564 | 2.7 | 0.68646564 | 2.7 | -0.09806652 | 2.7 | -0.02941996 | 74.539872 | -0.07893051 | 2.70 | 0.02941996 | 2.7 | 0.04903326 | 2.7 | 0.02941996 |
| 2.8 | -0.11767982 | 2.8 | 0.88259868 | 2.8 | 0.88259868 | 2.8 | -0.07845322 | 2.8 | -0.04903326 | 77.300608 | -0.03281119 | 2.80 | 0.03922661 | 2.8 | 0.04903326 | 2.8 | 0.03922661 |
| 2.9 | -0.11767982 | 2.9 | 1.15718494 | 2.9 | 1.15718494 | 2.9 | -0.06864656 | 2.9 | -0.04903326 | 80.061344 | 2.92676008 | 2.90 | 0.02941996 | 2.9 | 0.0196133 | 2.9 | 0.02941996 |
| 3 | -0.11767982 | 3 | 1.32389802 | 3 | 1.32389802 | 3 | -0.02941996 | 3 | -0.06864656 | 82.82208 | 0.38604798 | 3.00 | 0.04903326 | 3 | 0.03922661 | 3 | 0.04903326 |
| 3.1 | -0.11767982 | 3.1 | 1.34351132 | 3.1 | 1.34351132 | 3.1 | 0.04903326 | 3.1 | -0.09806652 | 85.582816 | 1.38006719 | 3.10 | 0.04903326 | 3.1 | 0.06864656 | 3.1 | 0.04903326 |
| 3.2 | -0.11767982 | 3.2 | 0.81395212 | 3.2 | 0.81395212 | 3.2 | 0.02941996 | 3.2 | -0.06864656 | 88.343552 | -0.04024532 | 3.20 | 0.02941996 | 3.2 | 0.07845322 | 3.2 | 0.02941996 |
| 3.3 | -0.10787317 | 3.3 | 0.32361952 | 3.3 | 0.32361952 | 3.3 | 0.00980665 | 3.3 | -0.05883991 | 91.104288 | 0.03586293 | 3.30 | 0.0196133 | 3.3 | 0.06864656 | 3.3 | 0.0196133 |

| | | | | | | | | | | | | | | | | | |
|---|---|---|---|---|---|---|---|---|---|---|---|---|---|---|---|---|---|
| 3.4 | -0.08825987 | 3.4 | 0.10787317 | 3.4 | 0.10787317 | 3.4 | 0.04903326 | 3.4 | -0.06864656 | 93.865024 | 0.0881773 | 3.40 | 0.03922661 | 3.4 | 0.05883991 | 3.4 | 0.03922661 |
| 3.5 | -0.13729313 | 3.5 | 0.00980665 | 3.5 | 0.00980665 | 3.5 | 0.05883991 | 3.5 | -0.06864656 | 96.62576 | 0.15226239 | 3.50 | 0.04903326 | 3.5 | 0.05883991 | 3.5 | 0.04903326 |
| 3.6 | -0.09806652 | 3.6 | -0.03922661 | 3.6 | -0.03922661 | 3.6 | 0.03922661 | 3.6 | -0.08825987 | 99.386496 | 0.43576966 | 3.60 | 0.03922661 | 3.6 | 0.04903326 | 3.6 | 0.03922661 |
| 3.7 | -0.10787317 | 3.7 | -0.00980665 | 3.7 | -0.00980665 | 3.7 | 0.05883991 | 3.7 | -0.08825987 | 102.147232 | 0.48608779 | 3.70 | 0.03922661 | 3.7 | 0.07845322 | 3.7 | 0.03922661 |
| 3.8 | -0.08825987 | 3.8 | 0.00980665 | 3.8 | 0.00980665 | 3.8 | 0.04903326 | 3.8 | -0.07845322 | 104.907968 | 0.56903363 | 3.80 | 0.03922661 | 3.8 | 0.06864656 | 3.8 | 0.03922661 |
| 3.9 | -0.07845322 | 3.9 | 0.00980665 | 3.9 | 0.00980665 | 3.9 | 0.03922661 | 3.9 | -0.08825987 | 107.668704 | 0.65815168 | 3.90 | 0.08825987 | 3.9 | 0.08825987 | 3.9 | 0.08825987 |
| 4 | -0.09806652 | 4 | -0.00980665 | 4 | -0.00980665 | 4 | 0.06864656 | 4 | -0.07845322 | 110.42944 | 0.76661227 | 4.00 | 0.08825987 | 4 | 0.09806652 | 4 | 0.08825987 |
| 4.1 | -0.08825987 | 4.1 | 0.00980665 | 4.1 | 0.00980665 | 4.1 | 0.03922661 | 4.1 | -0.06864656 | 113.190176 | 0.85492715 | 4.10 | 0.08825987 | 4.1 | 0.06864656 | 4.1 | 0.08825987 |
| 4.2 | -0.08825987 | 4.2 | 0.0196133 | 4.2 | 0.0196133 | 4.2 | -0.0196133 | 4.2 | -0.05883991 | 115.950912 | 0.97024828 | 4.20 | 0.12748648 | 4.2 | 0.05883991 | 4.2 | 0.12748648 |
| 4.3 | -0.10787317 | 4.3 | 0.0196133 | 4.3 | 0.0196133 | 4.3 | 0 | 4.3 | -0.05883991 | 118.711648 | 1.08933232 | 4.30 | 0.11767982 | 4.3 | 0.06864656 | 4.3 | 0.11767982 |
| 4.4 | -0.09806652 | 4.4 | 0.02941996 | 4.4 | 0.02941996 | 4.4 | 0.00980665 | 4.4 | -0.06864656 | 121.472384 | 1.2127759 | 4.40 | 0.08825987 | 4.4 | 0.05883991 | 4.4 | 0.08825987 |
| 4.5 | -0.07845322 | 4.5 | 0 | 4.5 | 0 | 4.5 | 0.0196133 | 4.5 | -0.05883991 | 124.23312 | 1.34273572 | 4.50 | 0.10787317 | 4.5 | 0.08825987 | 4.5 | 0.10787317 |
| 4.6 | -0.08825987 | 4.6 | -0.02941996 | 4.6 | -0.02941996 | 4.6 | 0.02941996 | 4.6 | -0.04903326 | 126.993856 | 1.47368237 | 4.60 | 0.08825987 | 4.6 | 0.09806652 | 4.6 | 0.08825987 |
| 4.7 | -0.07845322 | 4.7 | 0 | 4.7 | 0 | 4.7 | 0.02941996 | 4.7 | -0.04903326 | 129.754592 | 1.59480855 | 4.70 | 0.05883991 | 4.7 | 0.07845322 | 4.7 | 0.05883991 |
| 4.8 | -0.07845322 | 4.8 | 0.00980665 | 4.8 | 0.00980665 | 4.8 | 0.05883991 | 4.8 | -0.04903326 | 132.515328 | 1.73642435 | 4.80 | 0.06864656 | 4.8 | 0.08825987 | 4.8 | 0.06864656 |
| 4.9 | -0.09806652 | 4.9 | 0 | 4.9 | 0 | 4.9 | 0.06864656 | 4.9 | -0.0196133 | 135.276064 | 1.8284334 | 4.90 | 0.07845322 | 4.9 | 0.08825987 | 4.9 | 0.07845322 |
| 5 | -0.08825987 | 5 | 0.0196133 | 5 | 0.0196133 | 5 | 0.0196133 | 5 | -0.03922661 | 138.0368 | 1.98170548 | 5.00 | 0.08825987 | 5 | 0.07845322 | 5 | 0.08825987 |
| 5.1 | -0.09806652 | 5.1 | 0.04903326 | 5.1 | 0.04903326 | 5.1 | -0.00980665 | 5.1 | -0.04903326 | 140.797536 | 2.09493848 | 5.10 | 0.08825987 | 5.1 | 0.09806652 | 5.1 | 0.08825987 |
| 5.2 | -0.13729313 | 5.2 | 0.15690643 | 5.2 | 0.15690643 | 5.2 | -0.00980665 | 5.2 | -0.05883991 | 143.558272 | 2.21718899 | 5.20 | 0.05883991 | 5.2 | 0.10787317 | 5.2 | 0.05883991 |
| 5.3 | -0.10787317 | 5.3 | 0.11767982 | 5.3 | 0.11767982 | 5.3 | 0 | 5.3 | -0.04903326 | 146.319008 | 2.3457493 | 5.30 | 0.04903326 | 5.3 | 0.10787317 | 5.3 | 0.04903326 |
| 5.4 | -0.09806652 | 5.4 | 0.14709978 | 5.4 | 0.14709978 | 5.4 | -0.0196133 | 5.4 | -0.02941996 | 149.079744 | 2.48800773 | 5.40 | 0.07845322 | 5.4 | 0.08825987 | 5.4 | 0.07845322 |
| 5.5 | -0.08825987 | 5.5 | 0.25497295 | 5.5 | 0.25497295 | 5.5 | -0.02941996 | 5.5 | 0.04903326 | 151.84048 | 2.63010553 | 5.50 | 0.06864656 | 5.5 | 0.06864656 | 5.5 | 0.06864656 |
| 5.6 | -0.07845322 | 5.6 | 0.38245943 | 5.6 | 0.38245943 | 5.6 | -0.00980665 | 5.6 | 0.03922661 | 154.601216 | 2.78938909 | 5.60 | 0.06864656 | 5.6 | 0.08825987 | 5.6 | 0.06864656 |
| 5.7 | -0.08825987 | 5.7 | 0.58839912 | 5.7 | 0.58839912 | 5.7 | -0.03922661 | 5.7 | 0 | 157.361952 | 2.94142181 | 5.70 | 0.07845322 | 5.7 | 0.11767982 | 5.7 | 0.07845322 |
| 5.8 | -0.12748648 | 5.8 | 0.91201864 | 5.8 | 0.91201864 | 5.8 | -0.05883991 | 5.8 | 0.02941996 | 160.122688 | 3.09008203 | 5.80 | 0.07845322 | 5.8 | 0.10787317 | 5.8 | 0.07845322 |
| 5.9 | -0.08825987 | 5.9 | 0.65704568 | 5.9 | 0.65704568 | 5.9 | -0.04903326 | 5.9 | 0.05883991 | 162.883424 | 3.25677687 | 5.90 | 0.10787317 | 5.9 | 0.07845322 | 5.9 | 0.10787317 |
| 6 | -0.10787317 | 6 | 0.30400621 | 6 | 0.30400621 | 6 | -0.03922661 | 6 | 0.00980665 | 165.64416 | 3.41390356 | 6.00 | 0.10787317 | 6 | 0.09806652 | 6 | 0.10787317 |
| 6.1 | -0.10787317 | 6.1 | 0.09806652 | 6.1 | 0.09806652 | 6.1 | -0.03922661 | 6.1 | -0.02941996 | 168.404896 | 3.55207772 | 6.10 | 0.09806652 | 6.1 | 0.10787317 | 6.1 | 0.09806652 |
| 6.2 | -0.09806652 | 6.2 | 0.03922661 | 6.2 | 0.03922661 | 6.2 | -0.0196133 | 6.2 | -0.00980665 | 171.165632 | 3.71448156 | 6.20 | 0.06864656 | 6.2 | 0.13729313 | 6.2 | 0.06864656 |
| 6.3 | -0.10787317 | 6.3 | 0.05883991 | 6.3 | 0.05883991 | 6.3 | -0.0196133 | 6.3 | 0 | 173.926368 | 3.87275573 | 6.30 | 0.03922661 | 6.3 | 0.11767982 | 6.3 | 0.03922661 |
| 6.4 | -0.08825987 | 6.4 | 0.04903326 | 6.4 | 0.04903326 | 6.4 | -0.02941996 | 6.4 | 0.00980665 | 176.687104 | 4.00003087 | 6.40 | 0.06864656 | 6.4 | 0.11767982 | 6.4 | 0.06864656 |
| 6.5 | -0.10787317 | 6.5 | 0.04903326 | 6.5 | 0.04903326 | 6.5 | -0.02941996 | 6.5 | 0.0196133 | 179.44784 | 4.16009465 | 6.50 | 0.09806652 | 6.5 | 0.12748648 | 6.5 | 0.09806652 |
| 6.6 | -0.08825987 | 6.6 | 0.04903326 | 6.6 | 0.04903326 | 6.6 | -0.00980665 | 6.6 | 0.02941996 | 182.208576 | 4.30501481 | 6.60 | 0.07845322 | 6.6 | 0.13729313 | 6.6 | 0.07845322 |
| 6.7 | -0.08825987 | 6.7 | 0.04903326 | 6.7 | 0.04903326 | 6.7 | -0.0196133 | 6.7 | 0.07845322 | 184.969312 | 4.43137214 | 6.70 | 0.09806652 | 6.7 | 0.12748648 | 6.7 | 0.09806652 |
| 6.8 | -0.06864656 | 6.8 | 0.04903326 | 6.8 | 0.04903326 | 6.8 | 0 | 6.8 | 0.10787317 | 187.730048 | 4.56649496 | 6.80 | 0.09806652 | 6.8 | 0.12748648 | 6.8 | 0.09806652 |
| 6.9 | -0.08825987 | 6.9 | 0.05883991 | 6.9 | 0.05883991 | 6.9 | 0 | 6.9 | 0.11767982 | 190.490784 | 4.69969009 | 6.90 | 0.10787317 | 6.9 | 0.13729313 | 6.9 | 0.10787317 |
| 7 | -0.10787317 | 7 | 0.06864656 | 7 | 0.06864656 | 7 | -0.00980665 | 7 | 0.16671308 | 193.25152 | 4.8416501 | 7.00 | 0.10787317 | 7 | 0.13729313 | 7 | 0.10787317 |
| 7.1 | -0.09806652 | 7.1 | 0.06864656 | 7.1 | 0.06864656 | 7.1 | -0.0196133 | 7.1 | 0.20593969 | 196.012256 | 4.9876486 | 7.10 | 0.09806652 | 7.1 | 0.13729313 | 7.1 | 0.09806652 |
| 7.2 | -0.10787317 | 7.2 | 0.06864656 | 7.2 | 0.06864656 | 7.2 | -0.02941996 | 7.2 | 0.20593969 | 198.772992 | 5.11974204 | 7.20 | 0.08825987 | 7.2 | 0.13729313 | 7.2 | 0.08825987 |
| 7.3 | -0.06864656 | 7.3 | 0.08825987 | 7.3 | 0.08825987 | 7.3 | -0.03922661 | 7.3 | 0.19613304 | 201.533728 | 5.24564121 | 7.30 | 0.07845322 | 7.3 | 0.12748648 | 7.3 | 0.07845322 |
| 7.4 | -0.07845322 | 7.4 | 0.06864656 | 7.4 | 0.06864656 | 7.4 | -0.05883991 | 7.4 | 0.27458626 | 204.294464 | 5.40210232 | 7.40 | 0.07845322 | 7.4 | 0.17651974 | 7.4 | 0.07845322 |
| 7.5 | -0.11767982 | 7.5 | 0.06864656 | 7.5 | 0.06864656 | 7.5 | -0.05883991 | 7.5 | 0.21574634 | 207.0552 | 5.52841386 | 7.50 | 0.10787317 | 7.5 | 0.15690643 | 7.5 | 0.10787317 |
| 7.6 | -0.10787317 | 7.6 | 0.10787317 | 7.6 | 0.10787317 | 7.6 | -0.04903326 | 7.6 | 0.11767982 | 209.815936 | 5.63735665 | 7.60 | 0.11767982 | 7.6 | 0.16671308 | 7.6 | 0.11767982 |
| 7.7 | -0.09806652 | 7.7 | 0.08825987 | 7.7 | 0.08825987 | 7.7 | -0.0196133 | 7.7 | 0.03922661 | 212.576672 | 5.77353466 | 7.70 | 0.10787317 | 7.7 | 0.13729313 | 7.7 | 0.10787317 |
| 7.8 | -0.10787317 | 7.8 | 0.06864656 | 7.8 | 0.06864656 | 7.8 | -0.02941996 | 7.8 | 0.0196133 | 215.337408 | 5.90030525 | 7.80 | 0.10787317 | 7.8 | 0.11767982 | 7.8 | 0.10787317 |
| 7.9 | -0.09806652 | 7.9 | 0.07845322 | 7.9 | 0.07845322 | 7.9 | -0.02941996 | 7.9 | 0.07845322 | 218.098144 | 6.03331689 | 7.90 | 0.10787317 | 7.9 | 0.12748648 | 7.9 | 0.10787317 |
| 8 | -0.07845322 | 8 | 0.08825987 | 8 | 0.08825987 | 8 | -0.03922661 | 8 | 0.12748648 | 220.85888 | 6.15818293 | 8.00 | 0.10787317 | 8 | 0.17651974 | 8 | 0.10787317 |
| 8.1 | -0.06864656 | 8.1 | 0.08825987 | 8.1 | 0.08825987 | 8.1 | -0.02941996 | 8.1 | 0.30400621 | 223.619616 | 6.29615066 | 8.10 | 0.10787317 | 8.1 | 0.17651974 | 8.1 | 0.10787317 |
| 8.2 | -0.07845322 | 8.2 | 0.09806652 | 8.2 | 0.09806652 | 8.2 | -0.02941996 | 8.2 | 0.7354989 | 226.380352 | 6.3978426 | 8.20 | 0.12748648 | 8.2 | 0.17651974 | 8.2 | 0.12748648 |
| 8.3 | -0.07845322 | 8.3 | 0.06864656 | 8.3 | 0.06864656 | 8.3 | -0.02941996 | 8.3 | 1.16699159 | 229.141088 | 6.52881201 | 8.30 | 0.14709978 | 8.3 | 0.16671308 | 8.3 | 0.14709978 |
| 8.4 | -0.08825987 | 8.4 | 0.08825987 | 8.4 | 0.08825987 | 8.4 | -0.04903326 | 8.4 | 1.13757163 | 231.901824 | 6.63188061 | 8.40 | 0.10787317 | 8.4 | 0.15690643 | 8.4 | 0.10787317 |
| 8.5 | -0.10787317 | 8.5 | 0.10787317 | 8.5 | 0.10787317 | 8.5 | -0.04903326 | 8.5 | 0.99047185 | 234.66256 | 6.73572913 | 8.50 | 0.09806652 | 8.5 | 0.14709978 | 8.5 | 0.09806652 |
| 8.6 | -0.10787317 | 8.6 | 0.07845322 | 8.6 | 0.07845322 | 8.6 | -0.02941996 | 8.6 | 0.67665899 | 237.423296 | 6.82342463 | 8.60 | 0.10787317 | 8.6 | 0.13729313 | 8.6 | 0.10787317 |
| 8.7 | -0.13729313 | 8.7 | 0.09806652 | 8.7 | 0.09806652 | 8.7 | -0.02941996 | 8.7 | 0.53936586 | 240.184032 | 6.93725397 | 8.70 | 0.08825987 | 8.7 | 0.13729313 | 8.7 | 0.08825987 |
| 8.8 | -0.11767982 | 8.8 | 0.07845322 | 8.8 | 0.07845322 | 8.8 | -0.03922661 | 8.8 | 0.58839912 | 242.944768 | 7.04837608 | 8.80 | 0.10787317 | 8.8 | 0.15690643 | 8.8 | 0.10787317 |
| 8.9 | -0.12748648 | 8.9 | 0.06864656 | 8.9 | 0.06864656 | 8.9 | -0.0196133 | 8.9 | 0.80414546 | 245.705504 | 7.13737929 | 8.90 | 0.12748648 | 8.9 | 0.17651974 | 8.9 | 0.12748648 |
| 9 | -0.12748648 | 9 | 0.09806652 | 9 | 0.09806652 | 9 | -0.0196133 | 9 | 1.30428472 | 248.46624 | 7.26043297 | 9.00 | 0.15690643 | 9 | 0.16671308 | 9 | 0.15690643 |
| 9.1 | -0.11767982 | 9.1 | 0.09806652 | 9.1 | 0.09806652 | 9.1 | -0.0196133 | 9.1 | 1.80442397 | 251.226976 | 7.3439068 | 9.10 | 0.15690643 | 9.1 | 0.16671308 | 9.1 | 0.15690643 |
| 9.2 | -0.10787317 | 9.2 | 0.09806652 | 9.2 | 0.09806652 | 9.2 | -0.00980665 | 9.2 | 2.13785014 | 253.987712 | 7.43894463 | 9.20 | 0.14709978 | 9.2 | 0.15690643 | 9.2 | 0.14709978 |
| 9.3 | -0.10787317 | 9.3 | 0.09806652 | 9.3 | 0.09806652 | 9.3 | -0.04903326 | 9.3 | 1.89268384 | 256.748448 | 7.50514071 | 9.30 | 0.11767982 | 9.3 | 0.17651974 | 9.3 | 0.11767982 |
| 9.4 | -0.10787317 | 9.4 | 0.16671308 | 9.4 | 0.16671308 | 9.4 | -0.03922661 | 9.4 | 1.30428472 | 259.509184 | 7.60146312 | 9.40 | 0.13729313 | 9.4 | 0.17651974 | 9.4 | 0.13729313 |

| | | | | | | | | | | | | | | | | | |
|---|---|---|---|---|---|---|---|---|---|---|---|---|---|---|---|---|---|
| 9.5 | -0.08825987 | 9.5 | 0.13729313 | 9.5 | 0.13729313 | 9.5 | -0.02941996 | 9.5 | 1.13757163 | 262.26992 | 7.66217522 | 9.50 | 0.12748648 | 9.5 | 0.18632639 | 9.5 | 0.12748648 |
| 9.6 | -0.07845322 | 9.6 | 0.11767982 | 9.6 | 0.11767982 | 9.6 | -0.02941996 | 9.6 | 1.14737828 | 265.030656 | 7.75354222 | 9.60 | 0.12748648 | 9.6 | 0.17651974 | 9.6 | 0.12748648 |
| 9.7 | -0.06864656 | 9.7 | 0.11767982 | 9.7 | 0.11767982 | 9.7 | -0.03922661 | 9.7 | 1.12776498 | 267.791392 | 7.82790675 | 9.70 | 0.13729313 | 9.7 | 0.15690643 | 9.7 | 0.13729313 |
| 9.8 | -0.09806652 | 9.8 | 0.10787317 | 9.8 | 0.10787317 | 9.8 | -0.04903326 | 9.8 | 1.06892507 | 270.552128 | 7.90094003 | 9.80 | 0.12748648 | 9.8 | 0.17651974 | 9.8 | 0.12748648 |
| 9.9 | -0.09806652 | 9.9 | 0.09806652 | 9.9 | 0.09806652 | 9.9 | -0.04903326 | 9.9 | 1.03950511 | 273.312864 | 7.96789337 | 9.90 | 0.13729313 | 9.9 | 0.19613304 | 9.9 | 0.13729313 |
| 10 | -0.08825987 | 10 | 0.10787317 | 10 | 0.10787317 | 10 | -0.04903326 | 10 | 0.9610519 | 276.0736 | 8.06515663 | 10.00 | 0.14709978 | 10 | 0.19613304 | 10 | 0.14709978 |
| 10.1 | -0.09806652 | 10.1 | 0.11767982 | 10.1 | 0.11767982 | 10.1 | -0.03922661 | 10.1 | 0.94143859 | 278.834336 | 8.14514273 | 10.10 | 0.11767982 | 10.1 | 0.18632639 | 10.1 | 0.11767982 |
| 10.2 | -0.08825987 | 10.2 | 0.11767982 | 10.2 | 0.11767982 | 10.2 | -0.03922661 | 10.2 | 0.87279203 | 281.595072 | 8.20695621 | 10.20 | 0.14709978 | 10.2 | 0.19613304 | 10.2 | 0.14709978 |
| 10.3 | -0.06864656 | 10.3 | 0.13729313 | 10.3 | 0.13729313 | 10.3 | -0.04903326 | 10.3 | 0.7551122 | 284.355808 | 8.27737444 | 10.30 | 0.16671308 | 10.3 | 0.19613304 | 10.3 | 0.16671308 |
| 10.4 | -0.05883991 | 10.4 | 0.10787317 | 10.4 | 0.10787317 | 10.4 | -0.03922661 | 10.4 | 0.65704568 | 287.116544 | 8.33232739 | 10.40 | 0.15690643 | 10.4 | 0.18632639 | 10.4 | 0.15690643 |
| 10.5 | -0.07845322 | 10.5 | 0.11767982 | 10.5 | 0.11767982 | 10.5 | 0.00980665 | 10.5 | 0.56878582 | 289.87728 | 8.39739884 | 10.50 | 0.12748648 | 10.5 | 0.20593969 | 10.5 | 0.12748648 |
| 10.6 | -0.09806652 | 10.6 | 0.11767982 | 10.6 | 0.11767982 | 10.6 | -0.00980665 | 10.6 | 0.52955921 | 292.638016 | 8.10033162 | 10.60 | 0.13729313 | 10.6 | 0.20593969 | 10.6 | 0.13729313 |
| 10.7 | -0.06864656 | 10.7 | 0.12748648 | 10.7 | 0.12748648 | 10.7 | -0.03922661 | 10.7 | 0.5099459 | 295.398752 | 8.22540409 | 10.70 | 0.14709978 | 10.7 | 0.21574634 | 10.7 | 0.14709978 |
| 10.8 | -0.05883991 | 10.8 | 0.13729313 | 10.8 | 0.13729313 | 10.8 | -0.02941996 | 10.8 | 0.5099459 | 298.159488 | 8.1781833 | 10.80 | 0.14709978 | 10.8 | 0.225553 | 10.8 | 0.14709978 |
| 10.9 | -0.07845322 | 10.9 | 0.12748648 | 10.9 | 0.12748648 | 10.9 | -0.00980665 | 10.9 | 0.62762573 | 300.920224 | 8.31314509 | 10.90 | 0.14709978 | 10.9 | 0.18632639 | 10.9 | 0.14709978 |
| 11 | -0.08825987 | 11 | 0.10787317 | 11 | 0.10787317 | 11 | -0.03922661 | 11 | 0.61781908 | 303.68096 | 8.28421174 | 11.00 | 0.14709978 | 11 | 0.20593969 | 11 | 0.14709978 |
| 11.1 | -0.07845322 | 11.1 | 0.08825987 | 11.1 | 0.08825987 | 11.1 | -0.04903326 | 11.1 | 0.63743238 | 306.441696 | 8.3664466 | 11.10 | 0.13729313 | 11.1 | 0.25497295 | 11.1 | 0.13729313 |
| 11.2 | -0.08825987 | 11.2 | 0.11767982 | 11.2 | 0.11767982 | 11.2 | -0.0196133 | 11.2 | 0.64723903 | 309.202432 | 8.53431128 | 11.20 | 0.14709978 | 11.2 | 0.2451663 | 11.2 | 0.14709978 |
| 11.3 | -0.05883991 | 11.3 | 0.11767982 | 11.3 | 0.11767982 | 11.3 | 0.04903326 | 11.3 | 0.66685234 | 311.963168 | 8.59814366 | 11.30 | 0.17651974 | 11.3 | 0.21574634 | 11.3 | 0.17651974 |
| 11.4 | -0.05883991 | 11.4 | 0.12748648 | 11.4 | 0.12748648 | 11.4 | 0 | 11.4 | 0.69627229 | 314.723904 | 8.64974714 | 11.40 | 0.14709978 | 11.4 | 0.21574634 | 11.4 | 0.14709978 |
| 11.5 | -0.08825987 | 11.5 | 0.13729313 | 11.5 | 0.13729313 | 11.5 | 0.05883991 | 11.5 | 0.68646564 | 317.48464 | 8.76047895 | 11.50 | 0.16671308 | 11.5 | 0.225553 | 11.5 | 0.16671308 |
| 11.6 | -0.10787317 | 11.6 | 0.13729313 | 11.6 | 0.13729313 | 11.6 | 0.13729313 | 11.6 | 0.66685234 | 320.245376 | 8.82548215 | 11.60 | 0.18632639 | 11.6 | 0.2451663 | 11.6 | 0.18632639 |
| 11.7 | -0.11767982 | 11.7 | 0.11767982 | 11.7 | 0.11767982 | 11.7 | 0.13729313 | 11.7 | 0.61781908 | 323.006112 | 8.89254974 | 11.70 | 0.15690643 | 11.7 | 0.225553 | 11.7 | 0.15690643 |
| 11.8 | -0.09806652 | 11.8 | 0.11767982 | 11.8 | 0.11767982 | 11.8 | 0.08825987 | 11.8 | 0.54917251 | 325.766848 | 8.9405739 | 11.80 | 0.14709978 | 11.8 | 0.19613304 | 11.8 | 0.14709978 |
| 11.9 | -0.09806652 | 11.9 | 0.11767982 | 11.9 | 0.11767982 | 11.9 | 0.03922661 | 11.9 | 0.51975256 | 328.527584 | 8.99623783 | 11.90 | 0.14709978 | 11.9 | 0.21574634 | 11.9 | 0.14709978 |
| 12 | -0.08825987 | 12 | 0.13729313 | 12 | 0.13729313 | 12 | 0.03922661 | 12 | 0.54917251 | 331.28832 | 9.03707999 | 12.00 | 0.14709978 | 12 | 0.21574634 | 12 | 0.14709978 |
| 12.1 | -0.07845322 | 12.1 | 0.14709978 | 12.1 | 0.14709978 | 12.1 | 0.05883991 | 12.1 | 0.54917251 | 334.049056 | 9.07948189 | 12.10 | 0.18632639 | 12.1 | 0.21574634 | 12.1 | 0.18632639 |
| 12.2 | -0.06864656 | 12.2 | 0.12748648 | 12.2 | 0.12748648 | 12.2 | 0.05883991 | 12.2 | 0.54917251 | 336.809792 | 9.14056154 | 12.20 | 0.17651974 | 12.2 | 0.21574634 | 12.2 | 0.17651974 |
| 12.3 | -0.07845322 | 12.3 | 0.14709978 | 12.3 | 0.14709978 | 12.3 | 0.03922661 | 12.3 | 0.53936586 | 339.570528 | 9.18007245 | 12.30 | 0.16671308 | 12.3 | 0.23535965 | 12.3 | 0.16671308 |
| 12.4 | -0.06864656 | 12.4 | 0.13729313 | 12.4 | 0.13729313 | 12.4 | 0 | 12.4 | 0.43149269 | 342.331264 | 9.22766021 | 12.40 | 0.17651974 | 12.4 | 0.2451663 | 12.4 | 0.17651974 |
| 12.5 | -0.05883991 | 12.5 | 0.14709978 | 12.5 | 0.14709978 | 12.5 | -0.00980665 | 12.5 | 0.23535965 | 345.092 | 9.12048497 | 12.50 | 0.20593969 | 12.5 | 0.2451663 | 12.5 | 0.20593969 |
| 12.6 | -0.02941996 | 12.6 | 0.14709978 | 12.6 | 0.14709978 | 12.6 | -0.0196133 | 12.6 | 0.21574634 | 347.852736 | 9.20739996 | 12.60 | 0.16671308 | 12.6 | 0.25497295 | 12.6 | 0.16671308 |
| 12.7 | -0.00980665 | 12.7 | 0.13729313 | 12.7 | 0.13729313 | 12.7 | -0.02941996 | 12.7 | 0.18632639 | 350.613472 | 9.27862086 | 12.70 | 0.18632639 | 12.7 | 0.2451663 | 12.7 | 0.18632639 |
| 12.8 | 0.00980665 | 12.8 | 0.15690643 | 12.8 | 0.15690643 | 12.8 | -0.00980665 | 12.8 | 0.13729313 | 353.374208 | 9.33288548 | 12.80 | 0.17651974 | 12.8 | 0.2451663 | 12.8 | 0.17651974 |
| 12.9 | -0.00980665 | 12.9 | 0.15690643 | 12.9 | 0.15690643 | 12.9 | 0 | 12.9 | 0.11767982 | 356.134944 | 9.27758832 | 12.90 | 0.13729313 | 12.9 | 0.25497295 | 12.9 | 0.13729313 |
| 13 | 0.02941996 | 13 | 0.16671308 | 13 | 0.16671308 | 13 | 0.04903326 | 13 | 0.05883991 | 358.89568 | 9.34027391 | 13.00 | 0.16671308 | 13 | 0.2647796 | 13 | 0.16671308 |
| 13.1 | 0.54917251 | 13.1 | 0.13729313 | 13.1 | 0.13729313 | 13.1 | 0.07845322 | 13.1 | 0.10787317 | 361.656416 | 9.54932486 | 13.10 | 0.17651974 | 13.1 | 0.25497295 | 13.1 | 0.17651974 |
| 13.2 | 0.62762573 | 13.2 | 0.13729313 | 13.2 | 0.13729313 | 13.2 | 0.03922661 | 13.2 | 0.13729313 | 364.417152 | 9.6225881 | 13.20 | 0.20593969 | 13.2 | 0.25497295 | 13.2 | 0.20593969 |
| 13.3 | 0.64723903 | 13.3 | 0.11767982 | 13.3 | 0.11767982 | 13.3 | 0.03922661 | 13.3 | 0.225553 | 367.177888 | 9.68433275 | 13.30 | 0.225553 | 13.3 | 0.2647796 | 13.3 | 0.225553 |
| 13.4 | 0.61781908 | 13.4 | 0.12748648 | 13.4 | 0.12748648 | 13.4 | 0.02941996 | 13.4 | 0.21574634 | 369.938624 | 9.71707529 | 13.40 | 0.225553 | 13.4 | 0.29419956 | 13.4 | 0.225553 |
| 13.5 | 0.59820577 | 13.5 | 0.14709978 | 13.5 | 0.14709978 | 13.5 | 0.06864656 | 13.5 | 0.17651974 | 372.69936 | 9.7854968 | 13.50 | 0.18632639 | 13.5 | 0.29419956 | 13.5 | 0.18632639 |
| 13.6 | 0.60801242 | 13.6 | 0.14709978 | 13.6 | 0.14709978 | 13.6 | 0.16671308 | 13.6 | 0.18632639 | 375.460096 | 9.8205796 | 13.60 | 0.19613304 | 13.6 | 0.27458626 | 13.6 | 0.19613304 |
| 13.7 | 0.62762573 | 13.7 | 0.12748648 | 13.7 | 0.12748648 | 13.7 | 0.29419956 | 13.7 | 0.19613304 | 378.220832 | 9.88601851 | 13.70 | 0.21574634 | 13.7 | 0.27458626 | 13.7 | 0.21574634 |
| 13.8 | 0.60801242 | 13.8 | 0.14709978 | 13.8 | 0.14709978 | 13.8 | 0.46091264 | 13.8 | 0.18632639 | 380.981568 | 9.84432769 | 13.80 | 0.225553 | 13.8 | 0.27458626 | 13.8 | 0.225553 |
| 13.9 | 0.55897916 | 13.9 | 0.15690643 | 13.9 | 0.15690643 | 13.9 | 0.59820577 | 13.9 | 0.18632639 | 383.742304 | 9.78556564 | 13.90 | 0.19613304 | 13.9 | 0.29419956 | 13.9 | 0.19613304 |
| 14 | 0.56878582 | 14 | 0.16671308 | 14 | 0.16671308 | 14 | 0.63743238 | 14 | 0.17651974 | 386.50304 | 9.82275952 | 14.00 | 0.17651974 | 14 | 0.32361952 | 14 | 0.17651974 |
| 14.1 | 0.56878582 | 14.1 | 0.12748648 | 14.1 | 0.12748648 | 14.1 | 0.76491886 | 14.1 | 0.17651974 | 389.263776 | 9.86249971 | 14.10 | 0.20593969 | 14.1 | 0.29419956 | 14.1 | 0.20593969 |
| 14.2 | 0.53936586 | 14.2 | 0.11767982 | 14.2 | 0.11767982 | 14.2 | 0.92182529 | 14.2 | 0.21574634 | 392.024512 | 9.90366323 | 14.20 | 0.23535965 | 14.2 | 0.27458626 | 14.2 | 0.23535965 |
| 14.3 | 0.55897916 | 14.3 | 0.12748648 | 14.3 | 0.12748648 | 14.3 | 0.91201864 | 14.3 | 0.225553 | 394.785248 | 9.94569826 | 14.30 | 0.20593969 | 14.3 | 0.28439291 | 14.3 | 0.20593969 |
| 14.4 | 0.55897916 | 14.4 | 0.14709978 | 14.4 | 0.14709978 | 14.4 | 0.70607894 | 14.4 | 0.20593969 | 397.545984 | 9.97596277 | 14.40 | 0.19613304 | 14.4 | 0.30400621 | 14.4 | 0.19613304 |
| 14.5 | 0.55897916 | 14.5 | 0.13729313 | 14.5 | 0.13729313 | 14.5 | 0.52955921 | 14.5 | 0.19613304 | 400.30672 | 10.0319943 | 14.50 | 0.18632639 | 14.5 | 0.30400621 | 14.5 | 0.18632639 |
| 14.6 | 0.53936586 | 14.6 | 0.13729313 | 14.6 | 0.13729313 | 14.6 | 0.35303947 | 14.6 | 0.2647796 | 403.067456 | 10.0878881 | 14.60 | 0.18632639 | 14.6 | 0.29419956 | 14.6 | 0.18632639 |
| 14.7 | 0.53936586 | 14.7 | 0.16671308 | 14.7 | 0.16671308 | 14.7 | 0.17651974 | 14.7 | 0.42168604 | 405.828192 | 10.116087 | 14.70 | 0.15690643 | 14.7 | 0.30400621 | 14.7 | 0.15690643 |
| 14.8 | 0.54917251 | 14.8 | 0.15690643 | 14.8 | 0.15690643 | 14.8 | 0.05883991 | 14.8 | 0.58839912 | 408.588928 | 10.0525293 | 14.80 | 0.18632639 | 14.8 | 0.29419956 | 14.8 | 0.18632639 |
| 14.9 | 0.52955921 | 14.9 | 0.15690643 | 14.9 | 0.15690643 | 14.9 | 0.05883991 | 14.9 | 0.76491886 | 411.349664 | 10.1478892 | 14.90 | 0.20593969 | 14.9 | 0.31381286 | 14.9 | 0.20593969 |
| 15 | 0.51975256 | 15 | 0.17651974 | 15 | 0.17651974 | 15 | 0.08825987 | 15 | 0.92182529 | 414.1104 | 10.2195225 | 15.00 | 0.20593969 | 15 | 0.31381286 | 15 | 0.20593969 |
| 15.1 | 0.51975256 | 15.1 | 0.17651974 | 15.1 | 0.17651974 | 15.1 | 0.10787317 | 15.1 | 1.09834502 | 416.871136 | 10.174551 | 15.10 | 0.18632639 | 15.1 | 0.30400621 | 15.1 | 0.18632639 |
| 15.2 | 0.51975256 | 15.2 | 0.17651974 | 15.2 | 0.17651974 | 15.2 | 0.09806652 | 15.2 | 1.16699159 | 419.631872 | 10.3906912 | 15.20 | 0.19613304 | 15.2 | 0.34323282 | 15.2 | 0.19613304 |
| 15.3 | 0.51975256 | 15.3 | 0.17651974 | 15.3 | 0.17651974 | 15.3 | 0.11767982 | 15.3 | 1.03950511 | 422.392608 | 10.4530554 | 15.30 | 0.225553 | 15.3 | 0.33342617 | 15.3 | 0.225553 |
| 15.4 | 0.54917251 | 15.4 | 0.18632639 | 15.4 | 0.18632639 | 15.4 | 0.08825987 | 15.4 | 0.78453216 | 425.153344 | 10.5155117 | 15.40 | 0.2451663 | 15.4 | 0.29419956 | 15.4 | 0.2451663 |
| 15.5 | 0.55897916 | 15.5 | 0.18632639 | 15.5 | 0.18632639 | 15.5 | 0.06864656 | 15.5 | 0.61781908 | 427.91408 | 10.5829924 | 15.50 | 0.2451663 | 15.5 | 0.33342617 | 15.5 | 0.2451663 |

| | | | | | | | | | | | | | | | | | |
|---|---|---|---|---|---|---|---|---|---|---|---|---|---|---|---|---|---|
| 15.6 | 0.54917251 | 15.6 | 0.19613304 | 15.6 | 0.19613304 | 15.6 | 0.05883991 | 15.6 | 0.46091264 | 430.674816 | 10.6255098 | 15.60 | 0.21574634 | 15.6 | 0.34323282 | 15.6 | 0.21574634 |
| 15.7 | 0.50013925 | 15.7 | 0.20593969 | 15.7 | 0.20593969 | 15.7 | -0.07845322 | 15.7 | 0.33342617 | 433.435552 | 10.6734417 | 15.70 | 0.19613304 | 15.7 | 0.33342617 | 15.7 | 0.19613304 |
| 15.8 | 0.5099459 | 15.8 | 0.21574634 | 15.8 | 0.21574634 | 15.8 | -0.15690643 | 15.8 | 0.44129934 | 436.196288 | 10.6724553 | 15.80 | 0.21574634 | 15.8 | 0.35303947 | 15.8 | 0.21574634 |
| 15.9 | 0.53936586 | 15.9 | 0.2451663 | 15.9 | 0.2451663 | 15.9 | -0.15690643 | 15.9 | 0.65704568 | 438.957024 | 10.7663224 | 15.90 | 0.225553 | 15.9 | 0.36284612 | 15.9 | 0.225553 |
| 16 | 0.54917251 | 16 | 0.2451663 | 16 | 0.2451663 | 16 | -0.15690643 | 16 | 0.76491886 | 441.71776 | 10.7824988 | 16.00 | 0.225553 | 16 | 0.35303947 | 16 | 0.225553 |
| 16.1 | 0.55897916 | 16.1 | 0.225553 | 16.1 | 0.225553 | 16.1 | -0.17651974 | 16.1 | 0.78453216 | | | 16.10 | 0.225553 | 16.1 | 0.31381286 | 16.1 | 0.225553 |
| 16.2 | 0.54917251 | 16.2 | 0.225553 | 16.2 | 0.225553 | 16.2 | -0.23535965 | 16.2 | 0.80414546 | | | 16.20 | 0.225553 | 16.2 | 0.31381286 | 16.2 | 0.225553 |
| 16.3 | 0.53936586 | 16.3 | 0.19613304 | 16.3 | 0.19613304 | 16.3 | -0.29419956 | 16.3 | 0.79433881 | | | 16.30 | 0.23535965 | 16.3 | 0.34323282 | 16.3 | 0.23535965 |
| 16.4 | 0.52955921 | 16.4 | 0.20593969 | 16.4 | 0.20593969 | 16.4 | -0.28439291 | 16.4 | 0.82375877 | | | 16.40 | 0.225553 | 16.4 | 0.33342617 | 16.4 | 0.225553 |
| 16.5 | 0.53936586 | 16.5 | 0.23535965 | 16.5 | 0.23535965 | 16.5 | -0.29419956 | 16.5 | 0.79433881 | | | 16.50 | 0.2451663 | 16.5 | 0.38245943 | 16.5 | 0.2451663 |
| 16.6 | 0.52955921 | 16.6 | 0.225553 | 16.6 | 0.225553 | 16.6 | -0.30400621 | 16.6 | 0.76491886 | | | 16.60 | 0.225553 | 16.6 | 0.35303947 | 16.6 | 0.225553 |
| 16.7 | 0.53936586 | 16.7 | 0.20593969 | 16.7 | 0.20593969 | 16.7 | -0.33342617 | 16.7 | 0.74530555 | | | 16.70 | 0.23535965 | 16.7 | 0.32361952 | 16.7 | 0.23535965 |
| 16.8 | 0.54917251 | 16.8 | 0.20593969 | 16.8 | 0.20593969 | 16.8 | -0.34323282 | 16.8 | 0.83356542 | | | 16.80 | 0.2451663 | 16.8 | 0.34323282 | 16.8 | 0.2451663 |
| 16.9 | 0.55897916 | 16.9 | 0.20593969 | 16.9 | 0.20593969 | 16.9 | -0.35303947 | 16.9 | 0.99047185 | | | 16.90 | 0.23535965 | 16.9 | 0.37265278 | 16.9 | 0.23535965 |
| 17 | 0.52955921 | 17 | 0.21574634 | 17 | 0.21574634 | 17 | -0.35303947 | 17 | 1.2062182 | | | 17.00 | 0.225553 | 17 | 0.37265278 | 17 | 0.225553 |
| 17.1 | 0.50013925 | 17.1 | 0.2451663 | 17.1 | 0.2451663 | 17.1 | -0.32361952 | 17.1 | 1.39254458 | | | 17.10 | 0.25497295 | 17.1 | 0.36284612 | 17.1 | 0.25497295 |
| 17.2 | 0.5099459 | 17.2 | 0.2451663 | 17.2 | 0.2451663 | 17.2 | -0.32361952 | 17.2 | 1.55925767 | | | 17.20 | 0.25497295 | 17.2 | 0.35303947 | 17.2 | 0.25497295 |
| 17.3 | 0.53936586 | 17.3 | 0.23535965 | 17.3 | 0.23535965 | 17.3 | -0.31381286 | 17.3 | 1.54945102 | | | 17.30 | 0.2451663 | 17.3 | 0.34323282 | 17.3 | 0.2451663 |
| 17.4 | 0.54917251 | 17.4 | 0.25497295 | 17.4 | 0.25497295 | 17.4 | -0.28439291 | 17.4 | 1.21602485 | | | 17.40 | 0.2451663 | 17.4 | 0.38245943 | 17.4 | 0.2451663 |
| 17.5 | 0.54917251 | 17.5 | 0.2451663 | 17.5 | 0.2451663 | 17.5 | -0.28439291 | 17.5 | 1.02969846 | | | 17.50 | 0.23535965 | 17.5 | 0.39226608 | 17.5 | 0.23535965 |
| 17.6 | 0.54917251 | 17.6 | 0.225553 | 17.6 | 0.225553 | 17.6 | -0.29419956 | 17.6 | 1.03950511 | | | 17.60 | 0.23535965 | 17.6 | 0.36284612 | 17.6 | 0.23535965 |
| 17.7 | 0.52955921 | 17.7 | 0.2451663 | 17.7 | 0.2451663 | 17.7 | -0.29419956 | 17.7 | 1.08853837 | | | 17.70 | 0.2451663 | 17.7 | 0.40207273 | 17.7 | 0.2451663 |
| 17.8 | 0.52955921 | 17.8 | 0.23535965 | 17.8 | 0.23535965 | 17.8 | -0.30400621 | 17.8 | 1.19641154 | | | 17.80 | 0.25497295 | 17.8 | 0.42168604 | 17.8 | 0.25497295 |
| 17.9 | 0.50013925 | 17.9 | 0.2451663 | 17.9 | 0.2451663 | 17.9 | -0.30400621 | 17.9 | 1.43177119 | | | 17.90 | 0.225553 | 17.9 | 0.38245943 | 17.9 | 0.225553 |
| 18 | 0.52955921 | 18 | 0.2451663 | 18 | 0.2451663 | 18 | -0.31381286 | 18 | 1.65732419 | | | 18.00 | 0.23535965 | 18 | 0.39226608 | 18 | 0.23535965 |
| 18.1 | 0.51975256 | 18.1 | 0.20593969 | 18.1 | 0.20593969 | 18.1 | -0.31381286 | 18.1 | 1.54945102 | | | 18.10 | 0.2647796 | 18.1 | 0.39226608 | 18.1 | 0.2647796 |
| 18.2 | 0.50013925 | 18.2 | 0.20593969 | 18.2 | 0.20593969 | 18.2 | -0.25497295 | 18.2 | 1.29447806 | | | 18.20 | 0.25497295 | 18.2 | 0.40207273 | 18.2 | 0.25497295 |
| 18.3 | 0.5099459 | 18.3 | 0.25497295 | 18.3 | 0.25497295 | 18.3 | -0.14709978 | 18.3 | 1.23563815 | | | 18.30 | 0.2647796 | 18.3 | 0.40207273 | 18.3 | 0.2647796 |
| 18.4 | 0.5099459 | 18.4 | 0.2647796 | 18.4 | 0.2647796 | 18.4 | -0.10787317 | 18.4 | 1.41215789 | | | 18.40 | 0.27458626 | 18.4 | 0.37265278 | 18.4 | 0.27458626 |
| 18.5 | 0.50013925 | 18.5 | 0.23535965 | 18.5 | 0.23535965 | 18.5 | -0.09806652 | 18.5 | 1.4906111 | | | 18.50 | 0.29419956 | 18.5 | 0.36284612 | 18.5 | 0.29419956 |
| 18.6 | 0.5099459 | 18.6 | 0.2451663 | 18.6 | 0.2451663 | 18.6 | -0.14709978 | 18.6 | 1.4906111 | | | 18.60 | 0.30400621 | 18.6 | 0.36284612 | 18.6 | 0.30400621 |
| 18.7 | 0.50013925 | 18.7 | 0.2451663 | 18.7 | 0.2451663 | 18.7 | -0.15690643 | 18.7 | 1.55925767 | | | 18.70 | 0.31381286 | 18.7 | 0.38245943 | 18.7 | 0.31381286 |
| 18.8 | 0.5099459 | 18.8 | 0.25497295 | 18.8 | 0.25497295 | 18.8 | -0.15690643 | 18.8 | 1.4906111 | | | 18.80 | 0.27458626 | 18.8 | 0.40207273 | 18.8 | 0.27458626 |
| 18.9 | 0.50013925 | 18.9 | 0.23535965 | 18.9 | 0.23535965 | 18.9 | -0.16671308 | 18.9 | 1.38273793 | | | 18.90 | 0.25497295 | 18.9 | 0.39226608 | 18.9 | 0.25497295 |
| 19 | 0.50013925 | 19 | 0.27458626 | 19 | 0.27458626 | 19 | -0.18632639 | 19 | 1.28467141 | | | 19.00 | 0.27458626 | 19 | 0.38245943 | 19 | 0.27458626 |
| 19.1 | 0.5099459 | 19.1 | 0.2451663 | 19.1 | 0.2451663 | 19.1 | -0.225553 | 19.1 | 1.21602485 | | | 19.10 | 0.30400621 | 19.1 | 0.39226608 | 19.1 | 0.30400621 |
| 19.2 | 0.50013925 | 19.2 | 0.2451663 | 19.2 | 0.2451663 | 19.2 | -0.2647796 | 19.2 | 1.2258315 | | | 19.20 | 0.28439291 | 19.2 | 0.40207273 | 19.2 | 0.28439291 |
| 19.3 | 0.51975256 | 19.3 | 0.2451663 | 19.3 | 0.2451663 | 19.3 | -0.225553 | 19.3 | 1.27486476 | | | 19.30 | 0.25497295 | 19.3 | 0.38245943 | 19.3 | 0.25497295 |
| 19.4 | 0.4903326 | 19.4 | 0.2451663 | 19.4 | 0.2451663 | 19.4 | -0.15690643 | 19.4 | 1.26505811 | | | 19.40 | 0.27458626 | 19.4 | 0.43149269 | 19.4 | 0.27458626 |
| 19.5 | 0.50013925 | 19.5 | 0.25497295 | 19.5 | 0.25497295 | 19.5 | -0.16671308 | 19.5 | 1.21602485 | | | 19.50 | 0.31381286 | 19.5 | 0.43149269 | 19.5 | 0.31381286 |
| 19.6 | 0.50013925 | 19.6 | 0.25497295 | 19.6 | 0.25497295 | 19.6 | -0.21574634 | 19.6 | 1.18660489 | | | 19.60 | 0.28439291 | 19.6 | 0.41187938 | 19.6 | 0.28439291 |
| 19.7 | 0.50013925 | 19.7 | 0.2647796 | 19.7 | 0.2647796 | 19.7 | -0.23535965 | 19.7 | 1.16699159 | | | 19.70 | 0.27458626 | 19.7 | 0.42168604 | 19.7 | 0.27458626 |
| 19.8 | 0.52955921 | 19.8 | 0.2647796 | 19.8 | 0.2647796 | 19.8 | -0.2647796 | 19.8 | 1.17679824 | | | 19.80 | 0.27458626 | 19.8 | 0.42168604 | 19.8 | 0.27458626 |
| 19.9 | 0.5099459 | 19.9 | 0.25497295 | 19.9 | 0.25497295 | 19.9 | -0.27458626 | 19.9 | 1.16699159 | | | 19.90 | 0.28439291 | 19.9 | 0.41187938 | 19.9 | 0.28439291 |
| 20 | 0.48052595 | 20 | 0.2451663 | 20 | 0.2451663 | 20 | -0.25497295 | 20 | 1.15718494 | | | 20.00 | 0.28439291 | 20 | 0.41187938 | 20 | 0.28439291 |
| 20.1 | 0.48052595 | 20.1 | 0.21574634 | 20.1 | 0.21574634 | 20.1 | -0.18632639 | 20.1 | 1.16699159 | | | 20.10 | 0.30400621 | 20.1 | 0.41187938 | 20.1 | 0.30400621 |
| 20.2 | 0.50013925 | 20.2 | 0.21574634 | 20.2 | 0.21574634 | 20.2 | -0.19613304 | 20.2 | 1.13757163 | | | 20.20 | 0.27458626 | 20.2 | 0.44129934 | 20.2 | 0.27458626 |
| 20.3 | 0.5099459 | 20.3 | 0.21574634 | 20.3 | 0.21574634 | 20.3 | -0.225553 | 20.3 | 1.18660489 | | | 20.30 | 0.32361952 | 20.3 | 0.44129934 | 20.3 | 0.32361952 |
| 20.4 | 0.52955921 | 20.4 | 0.2451663 | 20.4 | 0.2451663 | 20.4 | -0.19613304 | 20.4 | 1.25525146 | | | 20.40 | 0.32361952 | 20.4 | 0.43149269 | 20.4 | 0.32361952 |
| 20.5 | 0.51975256 | 20.5 | 0.2451663 | 20.5 | 0.2451663 | 20.5 | -0.19613304 | 20.5 | 1.38273793 | | | 20.50 | 0.29419956 | 20.5 | 0.43149269 | 20.5 | 0.29419956 |
| 20.6 | 0.5099459 | 20.6 | 0.23535965 | 20.6 | 0.23535965 | 20.6 | -0.21574634 | 20.6 | 1.4709978 | | | 20.60 | 0.29419956 | 20.6 | 0.43149269 | 20.6 | 0.29419956 |
| 20.7 | 0.5099459 | 20.7 | 0.27458626 | 20.7 | 0.27458626 | 20.7 | -0.225553 | 20.7 | 1.44157784 | | | 20.70 | 0.28439291 | 20.7 | 0.45110599 | 20.7 | 0.28439291 |
| 20.8 | 0.52955921 | 20.8 | 0.27458626 | 20.8 | 0.27458626 | 20.8 | -0.19613304 | 20.8 | 1.18660489 | | | 20.80 | 0.27458626 | 20.8 | 0.4707193 | 20.8 | 0.27458626 |
| 20.9 | 0.5099459 | 20.9 | 0.20593969 | 20.9 | 0.20593969 | 20.9 | -0.17651974 | 20.9 | 0.84337207 | | | 20.90 | 0.30400621 | 20.9 | 0.45110599 | 20.9 | 0.30400621 |
| 21 | 0.5099459 | 21 | 0.23535965 | 21 | 0.23535965 | 21 | -0.20593969 | 21 | 0.60801242 | | | 21.00 | 0.33342617 | 21 | 0.45110599 | 21 | 0.33342617 |
| 21.1 | 0.54917251 | 21.1 | 0.27458626 | 21.1 | 0.27458626 | 21.1 | -0.21574634 | 21.1 | 0.57859247 | | | 21.10 | 0.31381286 | 21.1 | 0.46091264 | 21.1 | 0.31381286 |
| 21.2 | 0.53936586 | 21.2 | 0.25497295 | 21.2 | 0.25497295 | 21.2 | -0.18632639 | 21.2 | 0.63743238 | | | 21.20 | 0.32361952 | 21.2 | 0.48052595 | 21.2 | 0.32361952 |
| 21.3 | 0.51975256 | 21.3 | 0.29419956 | 21.3 | 0.29419956 | 21.3 | -0.10787317 | 21.3 | 0.87279203 | | | 21.30 | 0.30400621 | 21.3 | 0.46091264 | 21.3 | 0.30400621 |
| 21.4 | 0.5099459 | 21.4 | 0.29419956 | 21.4 | 0.29419956 | 21.4 | -0.03922661 | 21.4 | 1.0002785 | | | 21.40 | 0.33342617 | 21.4 | 0.42168604 | 21.4 | 0.33342617 |
| 21.5 | 0.51975256 | 21.5 | 0.27458626 | 21.5 | 0.27458626 | 21.5 | -0.13729313 | 21.5 | 1.13757163 | | | 21.50 | 0.35303947 | 21.5 | 0.44129934 | 21.5 | 0.35303947 |
| 21.6 | 0.5099459 | 21.6 | 0.25497295 | 21.6 | 0.25497295 | 21.6 | -0.10787317 | 21.6 | 1.17679824 | | | 21.60 | 0.32361952 | 21.6 | 0.4707193 | 21.6 | 0.32361952 |

| | | | | | | | | | | | | | | | | | |
|---|---|---|---|---|---|---|---|---|---|---|---|---|---|---|---|---|---|
| 21.7 | 0.51975256 | 21.7 | 0.28439291 | 21.7 | 0.28439291 | 21.7 | -0.07845322 | 21.7 | 1.19641154 | | | 21.70 | 0.31381286 | 21.7 | 0.4903326 | 21.7 | 0.31381286 |
| 21.8 | 0.51975256 | 21.8 | 0.30400621 | 21.8 | 0.30400621 | 21.8 | 0.2451663 | 21.8 | 1.01989181 | | | 21.80 | 0.33342617 | 21.8 | 0.45110599 | 21.8 | 0.33342617 |
| 21.9 | 0.53936586 | 21.9 | 0.28439291 | 21.9 | 0.28439291 | 21.9 | 0.45110599 | 21.9 | 0.69627229 | | | 21.90 | 0.33342617 | 21.9 | 0.45110599 | 21.9 | 0.33342617 |
| 22 | 0.55897916 | 22 | 0.25497295 | 22 | 0.25497295 | 22 | 0.42168604 | 22 | 0.4903326 | | | 22.00 | 0.31381286 | 22 | 0.44129934 | 22 | 0.31381286 |
| 22.1 | 0.52955921 | 22.1 | 0.28439291 | 22.1 | 0.28439291 | 22.1 | 0.27458626 | 22.1 | 0.38245943 | | | 22.10 | 0.34323282 | 22.1 | 0.43149269 | 22.1 | 0.34323282 |
| 22.2 | 0.5099459 | 22.2 | 0.27458626 | 22.2 | 0.27458626 | 22.2 | 0.20593969 | 22.2 | 0.35303947 | | | 22.20 | 0.33342617 | 22.2 | 0.46091264 | 22.2 | 0.33342617 |
| 22.3 | 0.5099459 | 22.3 | 0.27458626 | 22.3 | 0.27458626 | 22.3 | 0.29419956 | 22.3 | 0.31381286 | | | 22.30 | 0.34323282 | 22.3 | 0.4903326 | 22.3 | 0.34323282 |
| 22.4 | 0.50013925 | 22.4 | 0.25497295 | 22.4 | 0.25497295 | 22.4 | 0.44129934 | 22.4 | 0.2647796 | | | 22.40 | 0.35303947 | 22.4 | 0.5099459 | 22.4 | 0.35303947 |
| 22.5 | 0.4903326 | 22.5 | 0.25497295 | 22.5 | 0.25497295 | 22.5 | 0.51975256 | 22.5 | 0.2451663 | | | 22.50 | 0.33342617 | 22.5 | 0.4903326 | 22.5 | 0.33342617 |
| 22.6 | 0.50013925 | 22.6 | 0.23535965 | 22.6 | 0.23535965 | 22.6 | 0.4903326 | 22.6 | 0.29419956 | | | 22.60 | 0.33342617 | 22.6 | 0.50013925 | 22.6 | 0.33342617 |
| 22.7 | 0.51975256 | 22.7 | 0.25497295 | 22.7 | 0.25497295 | 22.7 | 0.39226608 | 22.7 | 0.42168604 | | | 22.70 | 0.30400621 | 22.7 | 0.50013925 | 22.7 | 0.30400621 |
| 22.8 | 0.53936586 | 22.8 | 0.31381286 | 22.8 | 0.31381286 | 22.8 | 0.4707193 | 22.8 | 0.43149269 | | | 22.80 | 0.31381286 | 22.8 | 0.4903326 | 22.8 | 0.31381286 |
| 22.9 | 0.53936586 | 22.9 | 0.33342617 | 22.9 | 0.33342617 | 22.9 | 0.56878582 | 22.9 | 0.44129934 | | | 22.90 | 0.30400621 | 22.9 | 0.52955921 | 22.9 | 0.30400621 |
| 23 | 0.53936586 | 23 | 0.27458626 | 23 | 0.27458626 | 23 | 0.80414546 | 23 | 0.4903326 | | | 23.00 | 0.32361952 | 23 | 0.52955921 | 23 | 0.32361952 |
| 23.1 | 0.52955921 | 23.1 | 0.27458626 | 23.1 | 0.27458626 | 23.1 | 0.94143859 | 23.1 | 0.46091264 | | | 23.10 | 0.35303947 | 23.1 | 0.5099459 | 23.1 | 0.35303947 |
| 23.2 | 0.51975256 | 23.2 | 0.29419956 | 23.2 | 0.29419956 | 23.2 | 1.08853837 | 23.2 | 0.39226608 | | | 23.20 | 0.34323282 | 23.2 | 0.52955921 | 23.2 | 0.34323282 |
| 23.3 | 0.52955921 | 23.3 | 0.29419956 | 23.3 | 0.29419956 | 23.3 | 1.27486476 | 23.3 | 0.38245943 | | | 23.30 | 0.33342617 | 23.3 | 0.51975256 | 23.3 | 0.33342617 |
| 23.4 | 0.53936586 | 23.4 | 0.27458626 | 23.4 | 0.27458626 | 23.4 | 1.44157784 | 23.4 | 0.40207273 | | | 23.40 | 0.34323282 | 23.4 | 0.51975256 | 23.4 | 0.34323282 |
| 23.5 | 0.52955921 | 23.5 | 0.2647796 | 23.5 | 0.2647796 | 23.5 | 1.54945102 | 23.5 | 0.42168604 | | | 23.50 | 0.34323282 | 23.5 | 0.52955921 | 23.5 | 0.34323282 |
| 23.6 | 0.51975256 | 23.6 | 0.28439291 | 23.6 | 0.28439291 | 23.6 | 1.68674414 | 23.6 | 0.43149269 | | | 23.60 | 0.33342617 | 23.6 | 0.51975256 | 23.6 | 0.33342617 |
| 23.7 | 0.52955921 | 23.7 | 0.29419956 | 23.7 | 0.29419956 | 23.7 | 1.81423062 | 23.7 | 0.44129934 | | | 23.70 | 0.37265278 | 23.7 | 0.51975256 | 23.7 | 0.37265278 |
| 23.8 | 0.53936586 | 23.8 | 0.29419956 | 23.8 | 0.29419956 | 23.8 | 1.6965508 | 23.8 | 0.50013925 | | | 23.80 | 0.38245943 | 23.8 | 0.52955921 | 23.8 | 0.38245943 |
| 23.9 | 0.50013925 | 23.9 | 0.2451663 | 23.9 | 0.2451663 | 23.9 | 1.59848428 | 23.9 | 0.62762573 | | | 23.90 | 0.35303947 | 23.9 | 0.52955921 | 23.9 | 0.35303947 |
| 24 | 0.52955921 | 24 | 0.25497295 | 24 | 0.25497295 | 24 | 1.60829093 | 24 | 0.72569225 | | | 24.00 | 0.34323282 | 24 | 0.53936586 | 24 | 0.34323282 |
| 24.1 | 0.53936586 | 24.1 | 0.28439291 | 24.1 | 0.28439291 | 24.1 | 1.56906432 | 24.1 | 0.70607894 | | | 24.10 | 0.35303947 | 24.1 | 0.55897916 | 24.1 | 0.35303947 |
| 24.2 | 0.51975256 | 24.2 | 0.28439291 | 24.2 | 0.28439291 | 24.2 | 2.05939692 | 24.2 | 0.53936586 | | | 24.20 | 0.36284612 | 24.2 | 0.51975256 | 24.2 | 0.36284612 |
| 24.3 | 0.5099459 | 24.3 | 0.29419956 | 24.3 | 0.29419956 | 24.3 | 2.60856943 | 24.3 | 0.27458626 | | | 24.30 | 0.34323282 | 24.3 | 0.55897916 | 24.3 | 0.34323282 |
| 24.4 | 0.50013925 | 24.4 | 0.28439291 | 24.4 | 0.28439291 | 24.4 | 2.9616089 | 24.4 | 0.10787317 | | | 24.40 | 0.34323282 | 24.4 | 0.54917251 | 24.4 | 0.34323282 |
| 24.5 | 0.5099459 | 24.5 | 0.27458626 | 24.5 | 0.27458626 | 24.5 | 3.11851534 | 24.5 | -0.03922661 | | | 24.50 | 0.37265278 | 24.5 | 0.54917251 | 24.5 | 0.37265278 |
| 24.6 | 0.5099459 | 24.6 | 0.2647796 | 24.6 | 0.2647796 | 24.6 | 3.08909538 | 24.6 | -0.06864656 | | | 24.60 | 0.38245943 | 24.6 | 0.56878582 | 24.6 | 0.38245943 |
| 24.7 | 0.5099459 | 24.7 | 0.27458626 | 24.7 | 0.27458626 | 24.7 | 2.88315569 | 24.7 | -0.08825987 | | | 24.70 | 0.35303947 | 24.7 | 0.55897916 | 24.7 | 0.35303947 |
| 24.8 | 0.5099459 | 24.8 | 0.2647796 | 24.8 | 0.2647796 | 24.8 | 2.61837608 | 24.8 | -0.10787317 | | | 24.80 | 0.36284612 | 24.8 | 0.55897916 | 24.8 | 0.36284612 |
| 24.9 | 0.51975256 | 24.9 | 0.27458626 | 24.9 | 0.27458626 | 24.9 | 2.44185635 | 24.9 | -0.04903326 | | | 24.90 | 0.38245943 | 24.9 | 0.54917251 | 24.9 | 0.38245943 |
| 25 | 0.50013925 | 25 | 0.2647796 | 25 | 0.2647796 | 25 | 2.42224304 | 25 | 0.34323282 | | | 25.00 | 0.39226608 | 25 | 0.54917251 | 25 | 0.39226608 |
| 25.1 | 0.4903326 | 25.1 | 0.2451663 | 25.1 | 0.2451663 | 25.1 | 2.79489582 | 25.1 | 0.37265278 | | | 25.10 | 0.39226608 | 25.1 | 0.56878582 | 25.1 | 0.39226608 |
| 25.2 | 0.4903326 | 25.2 | 0.2451663 | 25.2 | 0.2451663 | 25.2 | 3.44213485 | 25.2 | 0.37265278 | | | 25.20 | 0.39226608 | 25.2 | 0.59820577 | 25.2 | 0.39226608 |
| 25.3 | 0.50013925 | 25.3 | 0.27458626 | 25.3 | 0.27458626 | 25.3 | 3.79517432 | 25.3 | 0.35303947 | | | 25.30 | 0.38245943 | 25.3 | 0.58839912 | 25.3 | 0.38245943 |
| 25.4 | 0.51975256 | 25.4 | 0.2451663 | 25.4 | 0.2451663 | 25.4 | 3.84420758 | 25.4 | 0.38245943 | | | 25.40 | 0.39226608 | 25.4 | 0.59820577 | 25.4 | 0.39226608 |
| 25.5 | 0.51975256 | 25.5 | 0.25497295 | 25.5 | 0.25497295 | 25.5 | 3.27542177 | 25.5 | 0.40207273 | | | 25.50 | 0.41187938 | 25.5 | 0.57859247 | 25.5 | 0.41187938 |
| 25.6 | 0.51975256 | 25.6 | 0.28439291 | 25.6 | 0.28439291 | 25.6 | 2.70663595 | 25.6 | 0.46091264 | | | 25.60 | 0.39226608 | 25.6 | 0.55897916 | 25.6 | 0.39226608 |
| 25.7 | 0.52955921 | 25.7 | 0.28439291 | 25.7 | 0.28439291 | 25.7 | 2.51050291 | 25.7 | 0.48052595 | | | 25.70 | 0.39226608 | 25.7 | 0.58839912 | 25.7 | 0.39226608 |
| 25.8 | 0.52955921 | 25.8 | 0.30400621 | 25.8 | 0.30400621 | 25.8 | 2.15746344 | 25.8 | 0.45110599 | | | 25.80 | 0.41187938 | 25.8 | 0.61781908 | 25.8 | 0.41187938 |
| 25.9 | 0.53936586 | 25.9 | 0.28439291 | 25.9 | 0.28439291 | 25.9 | 1.77500401 | 25.9 | 0.46091264 | | | 25.90 | 0.40207273 | 25.9 | 0.59820577 | 25.9 | 0.40207273 |
| 26 | 0.51975256 | 26 | 0.33342617 | 26 | 0.33342617 | 26 | 1.63771088 | 26 | 0.50013925 | | | 26.00 | 0.39226608 | 26 | 0.57859247 | 26 | 0.39226608 |
| 26.1 | 0.5099459 | 26.1 | 0.30400621 | 26.1 | 0.30400621 | 26.1 | 1.51022441 | 26.1 | 0.5099459 | | | 26.10 | 0.42168604 | 26.1 | 0.58839912 | 26.1 | 0.42168604 |
| 26.2 | 0.52955921 | 26.2 | 0.29419956 | 26.2 | 0.29419956 | 26.2 | 1.50041776 | 26.2 | 0.4903326 | | | 26.20 | 0.41187938 | 26.2 | 0.59820577 | 26.2 | 0.41187938 |
| 26.3 | 0.52955921 | 26.3 | 0.31381286 | 26.3 | 0.31381286 | 26.3 | 1.51022441 | 26.3 | 0.5099459 | | | 26.30 | 0.39226608 | 26.3 | 0.60801242 | 26.3 | 0.39226608 |
| 26.4 | 0.51975256 | 26.4 | 0.31381286 | 26.4 | 0.31381286 | 26.4 | 1.31409137 | 26.4 | 0.50013925 | | | 26.40 | 0.42168604 | 26.4 | 0.58839912 | 26.4 | 0.42168604 |
| 26.5 | 0.52955921 | 26.5 | 0.29419956 | 26.5 | 0.29419956 | 26.5 | 1.02969846 | 26.5 | 0.51975256 | | | 26.50 | 0.39226608 | 26.5 | 0.62762573 | 26.5 | 0.39226608 |
| 26.6 | 0.5099459 | 26.6 | 0.30400621 | 26.6 | 0.30400621 | 26.6 | 1.09834502 | 26.6 | 0.52955921 | | | 26.60 | 0.36284612 | 26.6 | 0.62762573 | 26.6 | 0.36284612 |
| 26.7 | 0.50013925 | 26.7 | 0.31381286 | 26.7 | 0.31381286 | 26.7 | 1.29447806 | 26.7 | 0.56878582 | | | 26.70 | 0.39226608 | 26.7 | 0.63743238 | 26.7 | 0.39226608 |
| 26.8 | 0.53936586 | 26.8 | 0.30400621 | 26.8 | 0.30400621 | 26.8 | 1.50041776 | 26.8 | 0.58839912 | | | 26.80 | 0.41187938 | 26.8 | 0.62762573 | 26.8 | 0.41187938 |
| 26.9 | 0.54917251 | 26.9 | 0.32361952 | 26.9 | 0.32361952 | 26.9 | 1.78481066 | 26.9 | 0.59820577 | | | 26.90 | 0.40207273 | 26.9 | 0.61781908 | 26.9 | 0.40207273 |
| 27 | 0.52955921 | 27 | 0.32361952 | 27 | 0.32361952 | 27 | 2.27514326 | 27 | 0.62762573 | | | 27.00 | 0.43149269 | 27 | 0.63743238 | 27 | 0.43149269 |
| 27.1 | 0.50013925 | 27.1 | 0.31381286 | 27.1 | 0.31381286 | 27.1 | 3.03025547 | 27.1 | 0.65704568 | | | 27.10 | 0.44129934 | 27.1 | 0.64723903 | 27.1 | 0.44129934 |
| 27.2 | 0.5099459 | 27.2 | 0.30400621 | 27.2 | 0.30400621 | 27.2 | 3.9226608 | 27.2 | 0.66685234 | | | 27.20 | 0.42168604 | 27.2 | 0.65704568 | 27.2 | 0.42168604 |
| 27.3 | 0.50013925 | 27.3 | 0.31381286 | 27.3 | 0.31381286 | 27.3 | 4.40318675 | 27.3 | 0.69627229 | | | 27.30 | 0.43149269 | 27.3 | 0.66685234 | 27.3 | 0.43149269 |
| 27.4 | 0.54917251 | 27.4 | 0.30400621 | 27.4 | 0.30400621 | 27.4 | 4.55028653 | 27.4 | 0.68646564 | | | 27.40 | 0.45110599 | 27.4 | 0.63743238 | 27.4 | 0.45110599 |
| 27.5 | 0.54917251 | 27.5 | 0.31381286 | 27.5 | 0.31381286 | 27.5 | 4.8837127 | 27.5 | 0.65704568 | | | 27.50 | 0.42168604 | 27.5 | 0.62762573 | 27.5 | 0.42168604 |
| 27.6 | 0.51975256 | 27.6 | 0.31381286 | 27.6 | 0.31381286 | 27.6 | 5.34462534 | 27.6 | 0.63743238 | | | 27.60 | 0.44129934 | 27.6 | 0.63743238 | 27.6 | 0.44129934 |
| 27.7 | 0.50013925 | 27.7 | 0.36284612 | 27.7 | 0.36284612 | 27.7 | 4.75622622 | 27.7 | 0.60801242 | | | 27.70 | 0.46091264 | 27.7 | 0.62762573 | 27.7 | 0.46091264 |

| | | | | | | | | | | | | | | | | | |
|---|---|---|---|---|---|---|---|---|---|---|---|---|---|---|---|---|---|
| 27.8 | 0.50013925 | 27.8 | 0.33342617 | 27.8 | 0.33342617 | 27.8 | 3.9030475 | 27.8 | 0.56878582 | | | 27.80 | 0.42168604 | 27.8 | 0.65704568 | 27.8 | 0.42168604 |
| 27.9 | 0.5099459 | 27.9 | 0.30400621 | 27.9 | 0.30400621 | 27.9 | 2.77528252 | 27.9 | 0.56878582 | | | 27.90 | 0.44129934 | 27.9 | 0.66685234 | 27.9 | 0.44129934 |
| 28 | 0.5099459 | 28 | 0.31381286 | 28 | 0.31381286 | 28 | 2.44185635 | 28 | 0.55897916 | | | 28.00 | 0.44129934 | 28 | 0.66685234 | 28 | 0.44129934 |
| 28.1 | 0.50013925 | 28.1 | 0.31381286 | 28.1 | 0.31381286 | 28.1 | 3.00083551 | 28.1 | 0.59820577 | | | 28.10 | 0.43149269 | 28.1 | 0.65704568 | 28.1 | 0.43149269 |
| 28.2 | 0.51975256 | 28.2 | 0.35303947 | 28.2 | 0.35303947 | 28.2 | 4.04034062 | 28.2 | 0.59820577 | | | 28.20 | 0.46091264 | 28.2 | 0.64723903 | 28.2 | 0.46091264 |
| 28.3 | 0.52955921 | 28.3 | 0.36284612 | 28.3 | 0.36284612 | 28.3 | 5.23675217 | 28.3 | 0.56878582 | | | 28.30 | 0.4707193 | 28.3 | 0.66685234 | 28.3 | 0.4707193 |
| 28.4 | 0.51975256 | 28.4 | 0.39226608 | 28.4 | 0.39226608 | 28.4 | 5.6192116 | 28.4 | 0.57859247 | | | 28.40 | 0.4707193 | 28.4 | 0.69627229 | 28.4 | 0.4707193 |
| 28.5 | 0.51975256 | 28.5 | 0.37265278 | 28.5 | 0.37265278 | 28.5 | 5.57017834 | 28.5 | 0.54917251 | | | 28.50 | 0.48052595 | 28.5 | 0.7158856 | 28.5 | 0.48052595 |
| 28.6 | 0.52955921 | 28.6 | 0.33342617 | 28.6 | 0.33342617 | 28.6 | 5.67805151 | 28.6 | 0.55897916 | | | 28.60 | 0.45110599 | 28.6 | 0.70607894 | 28.6 | 0.45110599 |
| 28.7 | 0.51975256 | 28.7 | 0.36284612 | 28.7 | 0.36284612 | 28.7 | 6.00167102 | 28.7 | 0.5099459 | | | 28.70 | 0.45110599 | 28.7 | 0.68646564 | 28.7 | 0.45110599 |
| 28.8 | 0.51975256 | 28.8 | 0.35303947 | 28.8 | 0.35303947 | 28.8 | 4.64835305 | 28.8 | 0.5099459 | | | 28.80 | 0.46091264 | 28.8 | 0.67665899 | 28.8 | 0.46091264 |
| 28.9 | 0.4903326 | 28.9 | 0.33342617 | 28.9 | 0.33342617 | 28.9 | 1.62790423 | 28.9 | 0.4903326 | | | 28.90 | 0.4707193 | 28.9 | 0.68646564 | 28.9 | 0.4707193 |
| 29 | 0.51975256 | 29 | 0.31381286 | 29 | 0.31381286 | 29 | 1.51022441 | 29 | 0.46091264 | | | 29.00 | 0.48052595 | 29 | 0.69627229 | 29 | 0.48052595 |
| 29.1 | 0.52955921 | 29.1 | 0.32361952 | 29.1 | 0.32361952 | 29.1 | 1.41215789 | 29.1 | 0.45110599 | | | 29.10 | 0.46091264 | 29.1 | 0.68646564 | 29.1 | 0.46091264 |
| 29.2 | 0.5099459 | 29.2 | 0.34323282 | 29.2 | 0.34323282 | 29.2 | 1.30428472 | 29.2 | 0.5099459 | | | 29.20 | 0.46091264 | 29.2 | 0.67665899 | 29.2 | 0.46091264 |
| 29.3 | 0.5099459 | 29.3 | 0.36284612 | 29.3 | 0.36284612 | 29.3 | 1.2258315 | 29.3 | 0.52955921 | | | 29.30 | 0.50013925 | 29.3 | 0.67665899 | 29.3 | 0.50013925 |
| 29.4 | 0.51975256 | 29.4 | 0.37265278 | 29.4 | 0.37265278 | 29.4 | 1.18660489 | 29.4 | 0.51975256 | | | 29.40 | 0.48052595 | 29.4 | 0.68646564 | 29.4 | 0.48052595 |
| 29.5 | 0.53936586 | 29.5 | 0.37265278 | 29.5 | 0.37265278 | 29.5 | 1.19641154 | 29.5 | 0.51975256 | | | 29.50 | 0.4707193 | 29.5 | 0.7158856 | 29.5 | 0.4707193 |
| 29.6 | 0.5099459 | 29.6 | 0.35303947 | 29.6 | 0.35303947 | 29.6 | 1.17679824 | 29.6 | 0.64723903 | | | 29.60 | 0.45110599 | 29.6 | 0.72569225 | 29.6 | 0.45110599 |
| 29.7 | 0.50013925 | 29.7 | 0.35303947 | 29.7 | 0.35303947 | 29.7 | 1.15718494 | 29.7 | 0.72569225 | | | 29.70 | 0.4707193 | 29.7 | 0.72569225 | 29.7 | 0.4707193 |
| 29.8 | 0.4903326 | 29.8 | 0.34323282 | 29.8 | 0.34323282 | 29.8 | 1.17679824 | 29.8 | 0.69627229 | | | 29.80 | 0.50013925 | 29.8 | 0.72569225 | 29.8 | 0.50013925 |
| 29.9 | 0.4903326 | 29.9 | 0.34323282 | 29.9 | 0.34323282 | 29.9 | 1.14737828 | 29.9 | 0.7158856 | | | 29.90 | 0.48052595 | 29.9 | 0.74530555 | 29.9 | 0.48052595 |
| 30 | 0.50013925 | 30 | 0.37265278 | 30 | 0.37265278 | 30 | 1.13757163 | 30 | 0.7354989 | | | 30.00 | 0.44129934 | 30 | 0.72569225 | 30 | 0.44129934 |
| 30.1 | 0.51975256 | 30.1 | 0.37265278 | 30.1 | 0.37265278 | 30.1 | 1.05911842 | 30.1 | 0.70607894 | | | 30.10 | 0.45110599 | 30.1 | 0.72569225 | 30.1 | 0.45110599 |
| 30.2 | 0.50013925 | 30.2 | 0.38245943 | 30.2 | 0.38245943 | 30.2 | 1.03950511 | 30.2 | 0.69627229 | | | 30.20 | 0.4903326 | 30.2 | 0.72569225 | 30.2 | 0.4903326 |
| 30.3 | 0.48052595 | 30.3 | 0.36284612 | 30.3 | 0.36284612 | 30.3 | 1.05911842 | 30.3 | 0.7158856 | | | 30.30 | 0.50013925 | 30.3 | 0.7158856 | 30.3 | 0.50013925 |
| 30.4 | 0.52955921 | 30.4 | 0.35303947 | 30.4 | 0.35303947 | 30.4 | 1.07873172 | 30.4 | 0.7158856 | | | 30.40 | 0.5099459 | 30.4 | 0.72569225 | 30.4 | 0.5099459 |
| 30.5 | 0.52955921 | 30.5 | 0.34323282 | 30.5 | 0.34323282 | 30.5 | 1.09834502 | 30.5 | 0.7158856 | | | 30.50 | 0.51975256 | 30.5 | 0.7354989 | 30.5 | 0.51975256 |
| 30.6 | 0.4903326 | 30.6 | 0.38245943 | 30.6 | 0.38245943 | 30.6 | 1.08853837 | 30.6 | 0.69627229 | | | 30.60 | 0.46091264 | 30.6 | 0.74530555 | 30.6 | 0.46091264 |
| 30.7 | 0.5099459 | 30.7 | 0.39226608 | 30.7 | 0.39226608 | 30.7 | 1.04931176 | 30.7 | 0.7158856 | | | 30.70 | 0.4707193 | 30.7 | 0.7354989 | 30.7 | 0.4707193 |
| 30.8 | 0.50013925 | 30.8 | 0.34323282 | 30.8 | 0.34323282 | 30.8 | 1.03950511 | 30.8 | 0.70607894 | | | 30.80 | 0.4903326 | 30.8 | 0.7551122 | 30.8 | 0.4903326 |
| 30.9 | 0.5099459 | 30.9 | 0.35303947 | 30.9 | 0.35303947 | 30.9 | 1.07873172 | 30.9 | 0.67665899 | | | 30.90 | 0.50013925 | 30.9 | 0.78453216 | 30.9 | 0.50013925 |
| 31 | 0.5099459 | 31 | 0.36284612 | 31 | 0.36284612 | 31 | 1.03950511 | 31 | 0.63743238 | | | 31.00 | 0.48052595 | 31 | 0.77472551 | 31 | 0.48052595 |
| 31.1 | 0.5099459 | 31.1 | 0.35303947 | 31.1 | 0.35303947 | 31.1 | 1.02969846 | 31.1 | 0.62762573 | | | 31.10 | 0.4903326 | 31.1 | 0.79433881 | 31.1 | 0.4903326 |
| 31.2 | 0.53936586 | 31.2 | 0.36284612 | 31.2 | 0.36284612 | 31.2 | 1.02969846 | 31.2 | 0.60801242 | | | 31.20 | 0.50013925 | 31.2 | 0.77472551 | 31.2 | 0.50013925 |
| 31.3 | 0.5099459 | 31.3 | 0.39226608 | 31.3 | 0.39226608 | 31.3 | 1.05911842 | 31.3 | 0.63743238 | | | 31.30 | 0.52955921 | 31.3 | 0.79433881 | 31.3 | 0.52955921 |
| 31.4 | 0.4707193 | 31.4 | 0.40207273 | 31.4 | 0.40207273 | 31.4 | 1.05911842 | 31.4 | 0.63743238 | | | 31.40 | 0.55897916 | 31.4 | 0.79433881 | 31.4 | 0.55897916 |
| 31.5 | 0.4903326 | 31.5 | 0.40207273 | 31.5 | 0.40207273 | 31.5 | 1.01989181 | 31.5 | 0.63743238 | | | 31.50 | 0.52955921 | 31.5 | 0.76491886 | 31.5 | 0.52955921 |
| 31.6 | 0.51975256 | 31.6 | 0.41187938 | 31.6 | 0.41187938 | 31.6 | 1.02969846 | 31.6 | 0.67665899 | | | 31.60 | 0.53936586 | 31.6 | 0.7551122 | 31.6 | 0.53936586 |
| 31.7 | 0.4903326 | 31.7 | 0.38245943 | 31.7 | 0.38245943 | 31.7 | 1.03950511 | 31.7 | 0.65704568 | | | 31.70 | 0.53936586 | 31.7 | 0.77472551 | 31.7 | 0.53936586 |
| 31.8 | 0.50013925 | 31.8 | 0.37265278 | 31.8 | 0.37265278 | 31.8 | 1.04931176 | 31.8 | 0.67665899 | | | 31.80 | 0.4903326 | 31.8 | 0.81395212 | 31.8 | 0.4903326 |
| 31.9 | 0.5099459 | 31.9 | 0.39226608 | 31.9 | 0.39226608 | 31.9 | 1.04931176 | 31.9 | 0.67665899 | | | 31.90 | 0.51975256 | 31.9 | 0.79433881 | 31.9 | 0.51975256 |
| 32 | 0.4903326 | 32 | 0.41187938 | 32 | 0.41187938 | 32 | 1.03950511 | 32 | 0.7354989 | | | 32.00 | 0.53936586 | 32 | 0.79433881 | 32 | 0.53936586 |
| 32.1 | 0.50013925 | 32.1 | 0.38245943 | 32.1 | 0.38245943 | 32.1 | 1.02969846 | 32.1 | 0.69627229 | | | 32.10 | 0.5099459 | 32.1 | 0.82375877 | 32.1 | 0.5099459 |
| 32.2 | 0.52955921 | 32.2 | 0.38245943 | 32.2 | 0.38245943 | 32.2 | 1.02969846 | 32.2 | 0.67665899 | | | 32.20 | 0.52955921 | 32.2 | 0.81395212 | 32.2 | 0.52955921 |
| 32.3 | 0.5099459 | 32.3 | 0.37265278 | 32.3 | 0.37265278 | 32.3 | 1.02969846 | 32.3 | 0.70607894 | | | 32.30 | 0.54917251 | 32.3 | 0.81395212 | 32.3 | 0.54917251 |
| 32.4 | 0.50013925 | 32.4 | 0.37265278 | 32.4 | 0.37265278 | 32.4 | 1.01989181 | 32.4 | 0.68646564 | | | 32.40 | 0.54917251 | 32.4 | 0.81395212 | 32.4 | 0.54917251 |
| 32.5 | 0.5099459 | 32.5 | 0.40207273 | 32.5 | 0.40207273 | 32.5 | 1.01989181 | 32.5 | 0.67665899 | | | 32.50 | 0.52955921 | 32.5 | 0.82375877 | 32.5 | 0.52955921 |
| 32.6 | 0.50013925 | 32.6 | 0.41187938 | 32.6 | 0.41187938 | 32.6 | 1.02969846 | 32.6 | 0.66685234 | | | 32.60 | 0.53936586 | 32.6 | 0.83356542 | 32.6 | 0.53936586 |
| 32.7 | 0.4903326 | 32.7 | 0.39226608 | 32.7 | 0.39226608 | 32.7 | 1.01008516 | 32.7 | 0.68646564 | | | 32.70 | 0.54917251 | 32.7 | 0.82375877 | 32.7 | 0.54917251 |
| 32.8 | 0.52955921 | 32.8 | 0.38245943 | 32.8 | 0.38245943 | 32.8 | 0.99047185 | 32.8 | 0.70607894 | | | 32.80 | 0.55897916 | 32.8 | 0.81395212 | 32.8 | 0.55897916 |
| 32.9 | 0.51975256 | 32.9 | 0.37265278 | 32.9 | 0.37265278 | 32.9 | 1.0002785 | 32.9 | 0.74530555 | | | 32.90 | 0.54917251 | 32.9 | 0.81395212 | 32.9 | 0.54917251 |
| 33 | 0.50013925 | 33 | 0.39226608 | 33 | 0.39226608 | 33 | 1.01008516 | 33 | 0.76491886 | | | 33.00 | 0.54917251 | 33 | 0.83356542 | 33 | 0.54917251 |
| 33.1 | 0.52955921 | 33.1 | 0.39226608 | 33.1 | 0.39226608 | 33.1 | 1.0002785 | 33.1 | 0.7158856 | | | 33.10 | 0.56878582 | 33.1 | 0.83356542 | 33.1 | 0.56878582 |
| 33.2 | 0.50013925 | 33.2 | 0.41187938 | 33.2 | 0.41187938 | 33.2 | 1.01008516 | 33.2 | 0.72569225 | | | 33.20 | 0.54917251 | 33.2 | 0.82375877 | 33.2 | 0.54917251 |
| 33.3 | 0.5099459 | 33.3 | 0.42168604 | 33.3 | 0.42168604 | 33.3 | 1.0002785 | 33.3 | 0.69627229 | | | 33.30 | 0.53936586 | 33.3 | 0.83356542 | 33.3 | 0.53936586 |
| 33.4 | 0.4707193 | 33.4 | 0.43149269 | 33.4 | 0.43149269 | 33.4 | 1.0002785 | 33.4 | 0.68646564 | | | 33.40 | 0.55897916 | 33.4 | 0.83356542 | 33.4 | 0.55897916 |
| 33.5 | 0.4903326 | 33.5 | 0.43149269 | 33.5 | 0.43149269 | 33.5 | 1.01008516 | 33.5 | 0.67665899 | | | 33.50 | 0.58839912 | 33.5 | 0.83356542 | 33.5 | 0.58839912 |
| 33.6 | 0.5099459 | 33.6 | 0.43149269 | 33.6 | 0.43149269 | 33.6 | 0.9610519 | 33.6 | 0.66685234 | | | 33.60 | 0.54917251 | 33.6 | 0.82375877 | 33.6 | 0.54917251 |
| 33.7 | 0.48052595 | 33.7 | 0.44129934 | 33.7 | 0.44129934 | 33.7 | 0.97085855 | 33.7 | 0.64723903 | | | 33.70 | 0.53936586 | 33.7 | 0.83356542 | 33.7 | 0.53936586 |
| 33.8 | 0.51975256 | 33.8 | 0.42168604 | 33.8 | 0.42168604 | 33.8 | 0.99047185 | 33.8 | 0.62762573 | | | 33.80 | 0.53936586 | 33.8 | 0.87279203 | 33.8 | 0.53936586 |

| | | | | | | | | | | | | | | | | | |
|---|---|---|---|---|---|---|---|---|---|---|---|---|---|---|---|---|---|
| 33.9 | 0.5099459 | 33.9 | 0.40207273 | 33.9 | 0.40207273 | 33.9 | 1.0002785 | 33.9 | 0.63743238 | | | 33.90 | 0.54917251 | 33.9 | 0.87279203 | 33.9 | 0.54917251 |
| 34 | 0.52955921 | 34 | 0.43149269 | 34 | 0.43149269 | 34 | 0.97085855 | 34 | 0.62762573 | | | 34.00 | 0.57859247 | 34 | 0.84337207 | 34 | 0.57859247 |
| 34.1 | 0.56878582 | 34.1 | 0.44129934 | 34.1 | 0.44129934 | 34.1 | 0.97085855 | 34.1 | 0.61781908 | | | 34.10 | 0.56878582 | 34.1 | 0.84337207 | 34.1 | 0.56878582 |
| 34.2 | 0.53936586 | 34.2 | 0.43149269 | 34.2 | 0.43149269 | 34.2 | 0.9806652 | 34.2 | 0.57859247 | | | 34.20 | 0.58839912 | 34.2 | 0.88259868 | 34.2 | 0.58839912 |
| 34.3 | 0.50013925 | 34.3 | 0.42168604 | 34.3 | 0.42168604 | 34.3 | 0.97085855 | 34.3 | 0.61781908 | | | 34.30 | 0.56878582 | 34.3 | 0.86298538 | 34.3 | 0.56878582 |
| 34.4 | 0.50013925 | 34.4 | 0.42168604 | 34.4 | 0.42168604 | 34.4 | 0.9806652 | 34.4 | 0.63743238 | | | 34.40 | 0.55897916 | 34.4 | 0.88259868 | 34.4 | 0.55897916 |
| 34.5 | 0.50013925 | 34.5 | 0.44129934 | 34.5 | 0.44129934 | 34.5 | 1.02969846 | 34.5 | 0.66685234 | | | 34.50 | 0.57859247 | 34.5 | 0.89240533 | 34.5 | 0.57859247 |
| 34.6 | 0.5099459 | 34.6 | 0.43149269 | 34.6 | 0.43149269 | 34.6 | 1.01008516 | 34.6 | 0.65704568 | | | 34.60 | 0.57859247 | 34.6 | 0.85317872 | 34.6 | 0.57859247 |
| 34.7 | 0.5099459 | 34.7 | 0.41187938 | 34.7 | 0.41187938 | 34.7 | 1.01989181 | 34.7 | 0.66685234 | | | 34.70 | 0.59820577 | 34.7 | 0.85317872 | 34.7 | 0.59820577 |
| 34.8 | 0.5099459 | 34.8 | 0.42168604 | 34.8 | 0.42168604 | 34.8 | 1.01989181 | 34.8 | 0.67665899 | | | 34.80 | 0.61781908 | 34.8 | 0.86298538 | 34.8 | 0.61781908 |
| 34.9 | 0.4903326 | 34.9 | 0.43149269 | 34.9 | 0.43149269 | 34.9 | 0.99047185 | 34.9 | 0.67665899 | | | 34.90 | 0.59820577 | 34.9 | 0.88259868 | 34.9 | 0.59820577 |
| 35 | 0.4707193 | 35 | 0.44129934 | 35 | 0.44129934 | 35 | 0.9806652 | 35 | 0.66685234 | | | 35.00 | 0.57859247 | 35 | 0.86298538 | 35 | 0.57859247 |
| 35.1 | 0.4903326 | 35.1 | 0.43149269 | 35.1 | 0.43149269 | 35.1 | 0.97085855 | 35.1 | 0.66685234 | | | 35.10 | 0.60801242 | 35.1 | 0.86298538 | 35.1 | 0.60801242 |
| 35.2 | 0.48052595 | 35.2 | 0.42168604 | 35.2 | 0.42168604 | 35.2 | 0.9806652 | 35.2 | 0.67665899 | | | 35.20 | 0.60801242 | 35.2 | 0.88259868 | 35.2 | 0.60801242 |
| 35.3 | 0.4903326 | 35.3 | 0.46091264 | 35.3 | 0.46091264 | 35.3 | 0.9610519 | 35.3 | 0.65704568 | | | 35.30 | 0.58839912 | 35.3 | 0.92182529 | 35.3 | 0.58839912 |
| 35.4 | 0.51975256 | 35.4 | 0.48052595 | 35.4 | 0.48052595 | 35.4 | 0.97085855 | 35.4 | 0.66685234 | | | 35.40 | 0.57859247 | 35.4 | 0.94143859 | 35.4 | 0.57859247 |
| 35.5 | 0.50013925 | 35.5 | 0.43149269 | 35.5 | 0.43149269 | 35.5 | 0.97085855 | 35.5 | 0.68646564 | | | 35.50 | 0.56878582 | 35.5 | 0.91201864 | 35.5 | 0.56878582 |
| 35.6 | 0.4707193 | 35.6 | 0.43149269 | 35.6 | 0.43149269 | 35.6 | 1.0002785 | 35.6 | 0.68646564 | | | 35.60 | 0.57859247 | 35.6 | 0.91201864 | 35.6 | 0.57859247 |
| 35.7 | 0.4707193 | 35.7 | 0.44129934 | 35.7 | 0.44129934 | 35.7 | 1.01008516 | 35.7 | 0.70607894 | | | 35.70 | 0.57859247 | 35.7 | 0.92182529 | 35.7 | 0.57859247 |
| 35.8 | 0.5099459 | 35.8 | 0.44129934 | 35.8 | 0.44129934 | 35.8 | 1.01989181 | 35.8 | 0.72569225 | | | 35.80 | 0.61781908 | 35.8 | 0.92182529 | 35.8 | 0.61781908 |
| 35.9 | 0.5099459 | 35.9 | 0.46091264 | 35.9 | 0.46091264 | 35.9 | 0.99047185 | 35.9 | 0.70607894 | | | 35.90 | 0.61781908 | 35.9 | 0.90221198 | 35.9 | 0.61781908 |
| 36 | 0.5099459 | 36 | 0.48052595 | 36 | 0.48052595 | 36 | 0.9806652 | 36 | 0.66685234 | | | 36.00 | 0.58839912 | 36 | 0.92182529 | 36 | 0.58839912 |
| 36.1 | 0.50013925 | 36.1 | 0.4903326 | 36.1 | 0.4903326 | 36.1 | 0.9610519 | 36.1 | 0.67665899 | | | 36.10 | 0.58839912 | 36.1 | 0.95124524 | 36.1 | 0.58839912 |
| 36.2 | 0.4903326 | 36.2 | 0.4707193 | 36.2 | 0.4707193 | 36.2 | 0.9610519 | 36.2 | 0.67665899 | | | 36.20 | 0.60801242 | 36.2 | 0.88259868 | 36.2 | 0.60801242 |
| 36.3 | 0.50013925 | 36.3 | 0.45110599 | 36.3 | 0.45110599 | 36.3 | 0.9610519 | 36.3 | 0.68646564 | | | 36.30 | 0.59820577 | 36.3 | 0.91201864 | 36.3 | 0.59820577 |
| 36.4 | 0.50013925 | 36.4 | 0.4707193 | 36.4 | 0.4707193 | 36.4 | 0.9806652 | 36.4 | 0.70607894 | | | 36.40 | 0.59820577 | 36.4 | 0.93163194 | 36.4 | 0.59820577 |
| 36.5 | 0.4903326 | 36.5 | 0.4707193 | 36.5 | 0.4707193 | 36.5 | 0.9806652 | 36.5 | 0.70607894 | | | 36.50 | 0.60801242 | 36.5 | 0.93163194 | 36.5 | 0.60801242 |
| 36.6 | 0.4903326 | 36.6 | 0.45110599 | 36.6 | 0.45110599 | 36.6 | 0.95124524 | 36.6 | 0.69627229 | | | 36.60 | 0.62762573 | 36.6 | 0.92182529 | 36.6 | 0.62762573 |
| 36.7 | 0.48052595 | 36.7 | 0.4707193 | 36.7 | 0.4707193 | 36.7 | 0.9610519 | 36.7 | 0.7158856 | | | 36.70 | 0.63743238 | 36.7 | 0.95124524 | 36.7 | 0.63743238 |
| 36.8 | 0.4903326 | 36.8 | 0.48052595 | 36.8 | 0.48052595 | 36.8 | 0.9806652 | 36.8 | 0.70607894 | | | 36.80 | 0.65704568 | 36.8 | 0.94143859 | 36.8 | 0.65704568 |
| 36.9 | 0.48052595 | 36.9 | 0.4903326 | 36.9 | 0.4903326 | 36.9 | 0.99047185 | 36.9 | 0.7158856 | | | 36.90 | 0.64723903 | 36.9 | 0.95124524 | 36.9 | 0.64723903 |
| 37 | 0.5099459 | 37 | 0.4903326 | 37 | 0.4903326 | 37 | 0.9806652 | 37 | 0.70607894 | | | 37.00 | 0.61781908 | 37 | 0.9806652 | 37 | 0.61781908 |
| 37.1 | 0.5099459 | 37.1 | 0.4903326 | 37.1 | 0.4903326 | 37.1 | 0.97085855 | 37.1 | 0.68646564 | | | 37.10 | 0.60801242 | 37.1 | 1.01008516 | 37.1 | 0.60801242 |
| 37.2 | 0.52955921 | 37.2 | 0.48052595 | 37.2 | 0.48052595 | 37.2 | 0.9610519 | 37.2 | 0.67665899 | | | 37.20 | 0.63743238 | 37.2 | 0.93163194 | 37.2 | 0.63743238 |
| 37.3 | 0.54917251 | 37.3 | 0.48052595 | 37.3 | 0.48052595 | 37.3 | 0.97085855 | 37.3 | 0.67665899 | | | 37.30 | 0.62762573 | 37.3 | 0.94143859 | 37.3 | 0.62762573 |
| 37.4 | 0.53936586 | 37.4 | 0.4707193 | 37.4 | 0.4707193 | 37.4 | 0.97085855 | 37.4 | 0.72569225 | | | 37.40 | 0.64723903 | 37.4 | 0.97085855 | 37.4 | 0.64723903 |
| 37.5 | 0.52955921 | 37.5 | 0.48052595 | 37.5 | 0.48052595 | 37.5 | 0.99047185 | 37.5 | 0.74530555 | | | 37.50 | 0.60801242 | 37.5 | 0.9610519 | 37.5 | 0.60801242 |
| 37.6 | 0.51975256 | 37.6 | 0.46091264 | 37.6 | 0.46091264 | 37.6 | 0.99047185 | 37.6 | 0.74530555 | | | 37.60 | 0.59820577 | 37.6 | 1.0002785 | 37.6 | 0.59820577 |
| 37.7 | 0.48052595 | 37.7 | 0.43149269 | 37.7 | 0.43149269 | 37.7 | 1.01989181 | 37.7 | 0.74530555 | | | 37.70 | 0.61781908 | 37.7 | 1.01989181 | 37.7 | 0.61781908 |
| 37.8 | 0.51975256 | 37.8 | 0.43149269 | 37.8 | 0.43149269 | 37.8 | 1.01989181 | 37.8 | 0.74530555 | | | 37.80 | 0.66685234 | 37.8 | 1.01989181 | 37.8 | 0.66685234 |
| 37.9 | 0.5099459 | 37.9 | 0.45110599 | 37.9 | 0.45110599 | 37.9 | 1.01008516 | 37.9 | 0.77472551 | | | 37.90 | 0.64723903 | 37.9 | 1.01989181 | 37.9 | 0.64723903 |
| 38 | 0.4903326 | 38 | 0.45110599 | 38 | 0.45110599 | 38 | 0.99047185 | 38 | 0.7551122 | | | 38.00 | 0.64723903 | 38 | 0.99047185 | 38 | 0.64723903 |
| 38.1 | 0.48052595 | 38.1 | 0.46091264 | 38.1 | 0.46091264 | 38.1 | 0.95124524 | 38.1 | 0.74530555 | | | 38.10 | 0.64723903 | 38.1 | 1.01008516 | 38.1 | 0.64723903 |
| 38.2 | 0.5099459 | 38.2 | 0.50013925 | 38.2 | 0.50013925 | 38.2 | 0.97085855 | 38.2 | 0.74530555 | | | 38.20 | 0.64723903 | 38.2 | 1.03950511 | 38.2 | 0.64723903 |
| 38.3 | 0.48052595 | 38.3 | 0.5099459 | 38.3 | 0.5099459 | 38.3 | 0.95124524 | 38.3 | 0.74530555 | | | 38.30 | 0.70607894 | 38.3 | 1.01008516 | 38.3 | 0.70607894 |
| 38.4 | 0.45110599 | 38.4 | 0.4903326 | 38.4 | 0.4903326 | 38.4 | 0.9610519 | 38.4 | 0.76491886 | | | 38.40 | 0.67665899 | 38.4 | 1.02969846 | 38.4 | 0.67665899 |
| 38.5 | 0.4707193 | 38.5 | 0.5099459 | 38.5 | 0.5099459 | 38.5 | 0.93163194 | 38.5 | 0.7551122 | | | 38.50 | 0.70607894 | 38.5 | 1.01989181 | 38.5 | 0.70607894 |
| 38.6 | 0.48052595 | 38.6 | 0.51975256 | 38.6 | 0.51975256 | 38.6 | 0.94143859 | 38.6 | 0.74530555 | | | 38.60 | 0.7158856 | 38.6 | 1.01989181 | 38.6 | 0.7158856 |
| 38.7 | 0.4903326 | 38.7 | 0.51975256 | 38.7 | 0.51975256 | 38.7 | 0.93163194 | 38.7 | 0.74530555 | | | 38.70 | 0.69627229 | 38.7 | 1.02969846 | 38.7 | 0.69627229 |
| 38.8 | 0.5099459 | 38.8 | 0.51975256 | 38.8 | 0.51975256 | 38.8 | 0.92182529 | 38.8 | 0.7551122 | | | 38.80 | 0.64723903 | 38.8 | 1.03950511 | 38.8 | 0.64723903 |
| 38.9 | 0.51975256 | 38.9 | 0.51975256 | 38.9 | 0.51975256 | 38.9 | 0.93163194 | 38.9 | 0.7158856 | | | 38.90 | 0.65704568 | 38.9 | 1.02969846 | 38.9 | 0.65704568 |
| 39 | 0.51975256 | 39 | 0.51975256 | 39 | 0.51975256 | 39 | 0.97085855 | 39 | 0.76491886 | | | 39.00 | 0.69627229 | 39 | 1.04931176 | 39 | 0.69627229 |
| 39.1 | 0.4903326 | 39.1 | 0.51975256 | 39.1 | 0.51975256 | 39.1 | 0.94143859 | 39.1 | 0.74530555 | | | 39.10 | 0.70607894 | 39.1 | 1.04931176 | 39.1 | 0.70607894 |
| 39.2 | 0.5099459 | 39.2 | 0.50013925 | 39.2 | 0.50013925 | 39.2 | 0.93163194 | 39.2 | 0.70607894 | | | 39.20 | 0.7158856 | 39.2 | 1.04931176 | 39.2 | 0.7158856 |
| 39.3 | 0.52955921 | 39.3 | 0.4903326 | 39.3 | 0.4903326 | 39.3 | 0.95124524 | 39.3 | 0.69627229 | | | 39.30 | 0.68646564 | 39.3 | 1.02969846 | 39.3 | 0.68646564 |
| 39.4 | 0.50013925 | 39.4 | 0.48052595 | 39.4 | 0.48052595 | 39.4 | 0.9610519 | 39.4 | 0.66685234 | | | 39.40 | 0.67665899 | 39.4 | 1.03950511 | 39.4 | 0.67665899 |
| 39.5 | 0.50013925 | 39.5 | 0.50013925 | 39.5 | 0.50013925 | 39.5 | 0.94143859 | 39.5 | 0.67665899 | | | 39.50 | 0.67665899 | 39.5 | 1.08853837 | 39.5 | 0.67665899 |
| 39.6 | 0.4903326 | 39.6 | 0.50013925 | 39.6 | 0.50013925 | 39.6 | 0.95124524 | 39.6 | 0.68646564 | | | 39.60 | 0.70607894 | 39.6 | 1.05911842 | 39.6 | 0.70607894 |
| 39.7 | 0.50013925 | 39.7 | 0.5099459 | 39.7 | 0.5099459 | 39.7 | 0.9610519 | 39.7 | 0.70607894 | | | 39.70 | 0.69627229 | 39.7 | 1.04931176 | 39.7 | 0.69627229 |
| 39.8 | 0.51975256 | 39.8 | 0.52955921 | 39.8 | 0.52955921 | 39.8 | 0.9610519 | 39.8 | 0.70607894 | | | 39.80 | 0.68646564 | 39.8 | 1.03950511 | 39.8 | 0.68646564 |
| 39.9 | 0.51975256 | 39.9 | 0.52955921 | 39.9 | 0.52955921 | 39.9 | 0.95124524 | 39.9 | 0.70607894 | | | 39.90 | 0.68646564 | 39.9 | 1.06892507 | 39.9 | 0.68646564 |

| | | | | | | | | | | | | | | | | | |
|---|---|---|---|---|---|---|---|---|---|---|---|---|---|---|---|---|---|
| 40 | 0.52955921 | 40 | 0.5099459 | 40 | 0.5099459 | 40 | 0.92182529 | 40 | 0.7354989 | | | 40.00 | 0.67665899 | 40 | 1.06892507 | 40 | 0.67665899 |
| 40.1 | 0.53936586 | 40.1 | 0.52955921 | 40.1 | 0.52955921 | 40.1 | 0.93163194 | 40.1 | 0.7551122 | | | 40.10 | 0.70607894 | 40.1 | 1.07873172 | 40.1 | 0.70607894 |
| 40.2 | 0.51975256 | 40.2 | 0.51975256 | 40.2 | 0.51975256 | 40.2 | 0.91201864 | 40.2 | 0.7354989 | | | 40.20 | 0.74530555 | 40.2 | 1.06892507 | 40.2 | 0.74530555 |
| 40.3 | 0.52955921 | 40.3 | 0.52955921 | 40.3 | 0.52955921 | 40.3 | 0.94143859 | 40.3 | 0.72569225 | | | 40.30 | 0.72569225 | 40.3 | 1.05911842 | 40.3 | 0.72569225 |
| 40.4 | 0.51975256 | 40.4 | 0.51975256 | 40.4 | 0.51975256 | 40.4 | 1.0002785 | 40.4 | 0.65704568 | | | 40.40 | 0.70607894 | 40.4 | 1.08853837 | 40.4 | 0.70607894 |
| 40.5 | 0.51975256 | 40.5 | 0.51975256 | 40.5 | 0.51975256 | 40.5 | 0.97085855 | 40.5 | 0.67665899 | | | 40.50 | 0.72569225 | 40.5 | 1.09834502 | 40.5 | 0.72569225 |
| 40.6 | 0.52955921 | 40.6 | 0.54917251 | 40.6 | 0.54917251 | 40.6 | 0.95124524 | 40.6 | 0.72569225 | | | 40.60 | 0.78453216 | 40.6 | 1.09834502 | 40.6 | 0.78453216 |
| 40.7 | 0.51975256 | 40.7 | 0.55897916 | 40.7 | 0.55897916 | 40.7 | 0.9610519 | 40.7 | 0.72569225 | | | 40.70 | 0.7354989 | 40.7 | 1.10815168 | 40.7 | 0.7354989 |
| 40.8 | 0.50013925 | 40.8 | 0.52955921 | 40.8 | 0.52955921 | 40.8 | 0.93163194 | 40.8 | 0.72569225 | | | 40.80 | 0.67665899 | 40.8 | 1.10815168 | 40.8 | 0.67665899 |
| 40.9 | 0.51975256 | 40.9 | 0.52955921 | 40.9 | 0.52955921 | 40.9 | 0.89240533 | 40.9 | 0.7158856 | | | 40.90 | 0.69627229 | 40.9 | 1.09834502 | 40.9 | 0.69627229 |
| 41 | 0.51975256 | 41 | 0.52955921 | 41 | 0.52955921 | 41 | 0.91201864 | 41 | 0.7354989 | | | 41.00 | 0.69627229 | 41 | 1.08853837 | 41 | 0.69627229 |
| 41.1 | 0.53936586 | 41.1 | 0.54917251 | 41.1 | 0.54917251 | 41.1 | 0.99047185 | 41.1 | 0.74530555 | | | 41.10 | 0.7158856 | 41.1 | 1.10815168 | 41.1 | 0.7158856 |
| 41.2 | 0.52955921 | 41.2 | 0.52955921 | 41.2 | 0.52955921 | 41.2 | 0.99047185 | 41.2 | 0.74530555 | | | 41.20 | 0.7158856 | 41.2 | 1.14737828 | 41.2 | 0.7158856 |
| 41.3 | 0.52955921 | 41.3 | 0.51975256 | 41.3 | 0.51975256 | 41.3 | 0.9610519 | 41.3 | 0.74530555 | | | 41.30 | 0.72569225 | 41.3 | 1.18660489 | 41.3 | 0.72569225 |
| 41.4 | 0.54917251 | 41.4 | 0.52955921 | 41.4 | 0.52955921 | 41.4 | 0.9806652 | 41.4 | 0.70607894 | | | 41.40 | 0.7158856 | 41.4 | 1.18660489 | 41.4 | 0.7158856 |
| 41.5 | 0.51975256 | 41.5 | 0.51975256 | 41.5 | 0.51975256 | 41.5 | 1.0002785 | 41.5 | 0.70607894 | | | 41.50 | 0.72569225 | 41.5 | 1.17679824 | 41.5 | 0.72569225 |
| 41.6 | 0.50013925 | 41.6 | 0.52955921 | 41.6 | 0.52955921 | 41.6 | 1.0002785 | 41.6 | 0.72569225 | | | 41.60 | 0.7354989 | 41.6 | 1.16699159 | 41.6 | 0.7354989 |
| 41.7 | 0.5099459 | 41.7 | 0.54917251 | 41.7 | 0.54917251 | 41.7 | 0.9610519 | 41.7 | 0.7354989 | | | 41.70 | 0.72569225 | 41.7 | 1.15718494 | 41.7 | 0.72569225 |
| 41.8 | 0.5099459 | 41.8 | 0.54917251 | 41.8 | 0.54917251 | 41.8 | 0.97085855 | 41.8 | 0.7354989 | | | 41.80 | 0.72569225 | 41.8 | 1.14737828 | 41.8 | 0.72569225 |
| 41.9 | 0.51975256 | 41.9 | 0.53936586 | 41.9 | 0.53936586 | 41.9 | 0.9610519 | 41.9 | 0.7158856 | | | 41.90 | 0.7354989 | 41.9 | 1.18660489 | 41.9 | 0.7354989 |
| 42 | 0.51975256 | 42 | 0.53936586 | 42 | 0.53936586 | 42 | 0.93163194 | 42 | 0.70607894 | | | 42.00 | 0.7354989 | 42 | 1.17679824 | 42 | 0.7354989 |
| 42.1 | 0.4903326 | 42.1 | 0.53936586 | 42.1 | 0.53936586 | 42.1 | 0.92182529 | 42.1 | 0.72569225 | | | 42.10 | 0.74530555 | 42.1 | 1.17679824 | 42.1 | 0.74530555 |
| 42.2 | 0.5099459 | 42.2 | 0.53936586 | 42.2 | 0.53936586 | 42.2 | 0.95124524 | 42.2 | 0.7354989 | | | 42.20 | 0.74530555 | 42.2 | 1.15718494 | 42.2 | 0.74530555 |
| 42.3 | 0.5099459 | 42.3 | 0.54917251 | 42.3 | 0.54917251 | 42.3 | 0.97085855 | 42.3 | 0.76491886 | | | 42.30 | 0.77472551 | 42.3 | 1.16699159 | 42.3 | 0.77472551 |
| 42.4 | 0.50013925 | 42.4 | 0.54917251 | 42.4 | 0.54917251 | 42.4 | 0.93163194 | 42.4 | 0.7354989 | | | 42.40 | 0.78453216 | 42.4 | 1.17679824 | 42.4 | 0.78453216 |
| 42.5 | 0.53936586 | 42.5 | 0.5099459 | 42.5 | 0.5099459 | 42.5 | 0.94143859 | 42.5 | 0.7158856 | | | 42.50 | 0.77472551 | 42.5 | 1.2258315 | 42.5 | 0.77472551 |
| 42.6 | 0.55897916 | 42.6 | 0.55897916 | 42.6 | 0.55897916 | 42.6 | 0.92182529 | 42.6 | 0.7354989 | | | 42.60 | 0.76491886 | 42.6 | 1.26505811 | 42.6 | 0.76491886 |
| 42.7 | 0.55897916 | 42.7 | 0.56878582 | 42.7 | 0.56878582 | 42.7 | 0.95124524 | 42.7 | 0.7158856 | | | 42.70 | 0.77472551 | 42.7 | 1.2258315 | 42.7 | 0.77472551 |
| 42.8 | 0.53936586 | 42.8 | 0.57859247 | 42.8 | 0.57859247 | 42.8 | 0.9610519 | 42.8 | 0.7158856 | | | 42.80 | 0.7551122 | 42.8 | 1.2258315 | 42.8 | 0.7551122 |
| 42.9 | 0.50013925 | 42.9 | 0.55897916 | 42.9 | 0.55897916 | 42.9 | 0.9610519 | 42.9 | 0.7158856 | | | 42.90 | 0.78453216 | 42.9 | 1.21602485 | 42.9 | 0.78453216 |
| 43 | 0.52955921 | 43 | 0.55897916 | 43 | 0.55897916 | 43 | 0.9610519 | 43 | 0.7158856 | | | 43.00 | 0.79433881 | 43 | 1.21602485 | 43 | 0.79433881 |
| 43.1 | 0.51975256 | 43.1 | 0.58839912 | 43.1 | 0.58839912 | 43.1 | 0.91201864 | 43.1 | 0.7158856 | | | 43.10 | 0.76491886 | 43.1 | 1.25525146 | 43.1 | 0.76491886 |
| 43.2 | 0.50013925 | 43.2 | 0.57859247 | 43.2 | 0.57859247 | 43.2 | 0.89240533 | 43.2 | 0.7158856 | | | 43.20 | 0.7551122 | 43.2 | 1.23563815 | 43.2 | 0.7551122 |
| 43.3 | 0.5099459 | 43.3 | 0.54917251 | 43.3 | 0.54917251 | 43.3 | 0.92182529 | 43.3 | 0.7158856 | | | 43.30 | 0.78453216 | 43.3 | 1.23563815 | 43.3 | 0.78453216 |
| 43.4 | 0.52955921 | 43.4 | 0.57859247 | 43.4 | 0.57859247 | 43.4 | 0.90221198 | 43.4 | 0.7158856 | | | 43.40 | 0.79433881 | 43.4 | 1.21602485 | 43.4 | 0.79433881 |
| 43.5 | 0.51975256 | 43.5 | 0.60801242 | 43.5 | 0.60801242 | 43.5 | 0.92182529 | 43.5 | 0.72569225 | | | 43.50 | 0.76491886 | 43.5 | 1.18660489 | 43.5 | 0.76491886 |
| 43.6 | 0.50013925 | 43.6 | 0.58839912 | 43.6 | 0.58839912 | 43.6 | 0.91201864 | 43.6 | 0.7354989 | | | 43.60 | 0.78453216 | 43.6 | 1.21602485 | 43.6 | 0.78453216 |
| 43.7 | 0.5099459 | 43.7 | 0.57859247 | 43.7 | 0.57859247 | 43.7 | 0.94143859 | 43.7 | 0.69627229 | | | 43.70 | 0.78453216 | 43.7 | 1.2258315 | 43.7 | 0.78453216 |
| 43.8 | 0.51975256 | 43.8 | 0.54917251 | 43.8 | 0.54917251 | 43.8 | 0.9610519 | 43.8 | 0.70607894 | | | 43.80 | 0.79433881 | 43.8 | 1.21602485 | 43.8 | 0.79433881 |
| 43.9 | 0.5099459 | 43.9 | 0.56878582 | 43.9 | 0.56878582 | 43.9 | 0.9806652 | 43.9 | 0.69627229 | | | 43.90 | 0.79433881 | 43.9 | 1.2258315 | 43.9 | 0.79433881 |
| 44 | 0.4707193 | 44 | 0.59820577 | 44 | 0.59820577 | 44 | 1.01989181 | 44 | 0.70607894 | | | 44.00 | 0.79433881 | 44 | 1.23563815 | 44 | 0.79433881 |
| 44.1 | 0.50013925 | 44.1 | 0.58839912 | 44.1 | 0.58839912 | 44.1 | 0.9806652 | 44.1 | 0.7158856 | | | 44.10 | 0.77472551 | 44.1 | 1.23563815 | 44.1 | 0.77472551 |
| 44.2 | 0.51975256 | 44.2 | 0.57859247 | 44.2 | 0.57859247 | 44.2 | 0.93163194 | 44.2 | 0.70607894 | | | 44.20 | 0.79433881 | 44.2 | 1.25525146 | 44.2 | 0.79433881 |
| 44.3 | 0.51975256 | 44.3 | 0.60801242 | 44.3 | 0.60801242 | 44.3 | 0.95124524 | 44.3 | 0.70607894 | | | 44.30 | 0.81395212 | 44.3 | 1.2454448 | 44.3 | 0.81395212 |
| 44.4 | 0.51975256 | 44.4 | 0.59820577 | 44.4 | 0.59820577 | 44.4 | 0.9610519 | 44.4 | 0.69627229 | | | 44.40 | 0.81395212 | 44.4 | 1.27486476 | 44.4 | 0.81395212 |
| 44.5 | 0.50013925 | 44.5 | 0.58839912 | 44.5 | 0.58839912 | 44.5 | 0.95124524 | 44.5 | 0.63743238 | | | 44.50 | 0.82375877 | 44.5 | 1.26505811 | 44.5 | 0.82375877 |
| 44.6 | 0.50013925 | 44.6 | 0.61781908 | 44.6 | 0.61781908 | 44.6 | 0.94143859 | 44.6 | 0.63743238 | | | 44.60 | 0.78453216 | 44.6 | 1.27486476 | 44.6 | 0.78453216 |
| 44.7 | 0.53936586 | 44.7 | 0.60801242 | 44.7 | 0.60801242 | 44.7 | 0.95124524 | 44.7 | 0.72569225 | | | 44.70 | 0.80414546 | 44.7 | 1.27486476 | 44.7 | 0.80414546 |
| 44.8 | 0.53936586 | 44.8 | 0.56878582 | 44.8 | 0.56878582 | 44.8 | 0.97085855 | 44.8 | 0.77472551 | | | 44.80 | 0.80414546 | 44.8 | 1.27486476 | 44.8 | 0.80414546 |
| 44.9 | 0.5099459 | 44.9 | 0.57859247 | 44.9 | 0.57859247 | 44.9 | 0.9806652 | 44.9 | 0.77472551 | | | 44.90 | 0.84337207 | 44.9 | 1.31409137 | 44.9 | 0.84337207 |
| 45 | 0.5099459 | 45 | 0.59820577 | 45 | 0.59820577 | 45 | 0.97085855 | 45 | 0.7551122 | | | 45.00 | 0.82375877 | 45 | 1.31409137 | 45 | 0.82375877 |
| 45.1 | 0.5099459 | 45.1 | 0.63743238 | 45.1 | 0.63743238 | 45.1 | 0.93163194 | 45.1 | 0.76491886 | | | 45.10 | 0.83356542 | 45.1 | 1.28467141 | 45.1 | 0.83356542 |
| 45.2 | 0.5099459 | 45.2 | 0.62762573 | 45.2 | 0.62762573 | 45.2 | 0.95124524 | 45.2 | 0.77472551 | | | 45.20 | 0.82375877 | 45.2 | 1.28467141 | 45.2 | 0.82375877 |
| 45.3 | 0.5099459 | 45.3 | 0.60801242 | 45.3 | 0.60801242 | 45.3 | 0.9610519 | 45.3 | 0.77472551 | | | 45.30 | 0.83356542 | 45.3 | 1.31409137 | 45.3 | 0.83356542 |
| 45.4 | 0.51975256 | 45.4 | 0.58839912 | 45.4 | 0.58839912 | 45.4 | 0.93163194 | 45.4 | 0.77472551 | | | 45.40 | 0.81395212 | 45.4 | 1.32389802 | 45.4 | 0.81395212 |
| 45.5 | 0.52955921 | 45.5 | 0.59820577 | 45.5 | 0.59820577 | 45.5 | 0.9610519 | 45.5 | 0.78453216 | | | 45.50 | 0.82375877 | 45.5 | 1.29447806 | 45.5 | 0.82375877 |
| 45.6 | 0.54917251 | 45.6 | 0.58839912 | 45.6 | 0.58839912 | 45.6 | 0.9610519 | 45.6 | 0.81395212 | | | 45.60 | 0.86298538 | 45.6 | 1.28467141 | 45.6 | 0.86298538 |
| 45.7 | 0.5099459 | 45.7 | 0.58839912 | 45.7 | 0.58839912 | 45.7 | 0.94143859 | 45.7 | 0.80414546 | | | 45.70 | 0.84337207 | 45.7 | 1.29447806 | 45.7 | 0.84337207 |
| 45.8 | 0.4707193 | 45.8 | 0.60801242 | 45.8 | 0.60801242 | 45.8 | 0.97085855 | 45.8 | 0.79433881 | | | 45.80 | 0.84337207 | 45.8 | 1.33370467 | 45.8 | 0.84337207 |
| 45.9 | 0.5099459 | 45.9 | 0.62762573 | 45.9 | 0.62762573 | 45.9 | 0.95124524 | 45.9 | 0.81395212 | | | 45.90 | 0.84337207 | 45.9 | 1.33370467 | 45.9 | 0.84337207 |
| 46 | 0.54917251 | 46 | 0.61781908 | 46 | 0.61781908 | 46 | 0.88259868 | 46 | 0.81395212 | | | 46.00 | 0.85317872 | 46 | 1.33370467 | 46 | 0.85317872 |

| | | | | | | | | | | | | | | | | | |
|---|---|---|---|---|---|---|---|---|---|---|---|---|---|---|---|---|---|
| 46.1 | 0.56878582 | 46.1 | 0.61781908 | 46.1 | 0.61781908 | 46.1 | 0.88259868 | 46.1 | 0.80414546 | | | 46.10 | 0.85317872 | 46.1 | 1.35331798 | 46.1 | 0.85317872 |
| 46.2 | 0.53936586 | 46.2 | 0.60801242 | 46.2 | 0.60801242 | 46.2 | 0.93163194 | 46.2 | 0.79433881 | | | 46.20 | 0.84337207 | 46.2 | 1.35331798 | 46.2 | 0.84337207 |
| 46.3 | 0.51975256 | 46.3 | 0.61781908 | 46.3 | 0.61781908 | 46.3 | 0.93163194 | 46.3 | 0.83356542 | | | 46.30 | 0.86298538 | 46.3 | 1.33370467 | 46.3 | 0.86298538 |
| 46.4 | 0.52955921 | 46.4 | 0.59820577 | 46.4 | 0.59820577 | 46.4 | 0.93163194 | 46.4 | 0.84337207 | | | 46.40 | 0.89240533 | 46.4 | 1.34351132 | 46.4 | 0.89240533 |
| 46.5 | 0.52955921 | 46.5 | 0.62762573 | 46.5 | 0.62762573 | 46.5 | 0.93163194 | 46.5 | 0.82375877 | | | 46.50 | 0.88259868 | 46.5 | 1.35331798 | 46.5 | 0.88259868 |
| 46.6 | 0.5099459 | 46.6 | 0.61781908 | 46.6 | 0.61781908 | 46.6 | 0.94143859 | 46.6 | 0.82375877 | | | 46.60 | 0.88259868 | 46.6 | 1.36312463 | 46.6 | 0.88259868 |
| 46.7 | 0.51975256 | 46.7 | 0.62762573 | 46.7 | 0.62762573 | 46.7 | 0.92182529 | 46.7 | 0.80414546 | | | 46.70 | 0.87279203 | 46.7 | 1.41215789 | 46.7 | 0.87279203 |
| 46.8 | 0.5099459 | 46.8 | 0.63743238 | 46.8 | 0.63743238 | 46.8 | 0.91201864 | 46.8 | 0.79433881 | | | 46.80 | 0.88259868 | 46.8 | 1.39254458 | 46.8 | 0.88259868 |
| 46.9 | 0.5099459 | 46.9 | 0.63743238 | 46.9 | 0.63743238 | 46.9 | 0.90221198 | 46.9 | 0.84337207 | | | 46.90 | 0.88259868 | 46.9 | 1.38273793 | 46.9 | 0.88259868 |
| 47 | 0.53936586 | 47 | 0.62762573 | 47 | 0.62762573 | 47 | 0.94143859 | 47 | 0.85317872 | | | 47.00 | 0.88259868 | 47 | 1.40235124 | 47 | 0.88259868 |
| 47.1 | 0.53936586 | 47.1 | 0.61781908 | 47.1 | 0.61781908 | 47.1 | 0.9610519 | 47.1 | 0.83356542 | | | 47.10 | 0.88259868 | 47.1 | 1.44157784 | 47.1 | 0.88259868 |
| 47.2 | 0.51975256 | 47.2 | 0.63743238 | 47.2 | 0.63743238 | 47.2 | 0.95124524 | 47.2 | 0.85317872 | | | 47.20 | 0.89240533 | 47.2 | 1.43177119 | 47.2 | 0.89240533 |
| 47.3 | 0.53936586 | 47.3 | 0.63743238 | 47.3 | 0.63743238 | 47.3 | 1.01008516 | 47.3 | 0.86298538 | | | 47.30 | 0.87279203 | 47.3 | 1.42196454 | 47.3 | 0.87279203 |
| 47.4 | 0.54917251 | 47.4 | 0.62762573 | 47.4 | 0.62762573 | 47.4 | 1.03950511 | 47.4 | 0.83356542 | | | 47.40 | 0.85317872 | 47.4 | 1.42196454 | 47.4 | 0.85317872 |
| 47.5 | 0.53936586 | 47.5 | 0.63743238 | 47.5 | 0.63743238 | 47.5 | 1.08853837 | 47.5 | 0.85317872 | | | 47.50 | 0.85317872 | 47.5 | 1.40235124 | 47.5 | 0.85317872 |
| 47.6 | 0.57859247 | 47.6 | 0.60801242 | 47.6 | 0.60801242 | 47.6 | 1.18660489 | 47.6 | 0.87279203 | | | 47.60 | 0.89240533 | 47.6 | 1.41215789 | 47.6 | 0.89240533 |
| 47.7 | 0.55897916 | 47.7 | 0.58839912 | 47.7 | 0.58839912 | 47.7 | 1.64751754 | 47.7 | 0.86298538 | | | 47.70 | 0.93163194 | 47.7 | 1.41215789 | 47.7 | 0.93163194 |
| 47.8 | 0.53936586 | 47.8 | 0.59820577 | 47.8 | 0.59820577 | 47.8 | 1.99075036 | 47.8 | 0.85317872 | | | 47.80 | 0.93163194 | 47.8 | 1.40235124 | 47.8 | 0.93163194 |
| 47.9 | 0.53936586 | 47.9 | 0.60801242 | 47.9 | 0.60801242 | 47.9 | 2.27514326 | 47.9 | 0.86298538 | | | 47.90 | 0.91201864 | 47.9 | 1.44157784 | 47.9 | 0.91201864 |
| 48 | 0.51975256 | 48 | 0.62762573 | 48 | 0.62762573 | 48 | 2.56934282 | 48 | 0.86298538 | | | 48.00 | 0.91201864 | 48 | 1.44157784 | 48 | 0.91201864 |
| 48.1 | 0.54917251 | 48.1 | 0.63743238 | 48.1 | 0.63743238 | 48.1 | 2.89296234 | 48.1 | 0.86298538 | | | 48.10 | 0.91201864 | 48.1 | 1.40235124 | 48.1 | 0.91201864 |
| 48.2 | 0.54917251 | 48.2 | 0.62762573 | 48.2 | 0.62762573 | 48.2 | 3.09890203 | 48.2 | 0.88259868 | | | 48.20 | 0.88259868 | 48.2 | 1.39254458 | 48.2 | 0.88259868 |
| 48.3 | 0.51975256 | 48.3 | 0.60801242 | 48.3 | 0.60801242 | 48.3 | 3.1871619 | 48.3 | 0.89240533 | | | 48.30 | 0.88259868 | 48.3 | 1.43177119 | 48.3 | 0.88259868 |
| 48.4 | 0.5099459 | 48.4 | 0.62762573 | 48.4 | 0.62762573 | 48.4 | 2.87334904 | 48.4 | 0.91201864 | | | 48.40 | 0.88259868 | 48.4 | 1.4709978 | 48.4 | 0.88259868 |
| 48.5 | 0.5099459 | 48.5 | 0.61781908 | 48.5 | 0.61781908 | 48.5 | 1.97113705 | 48.5 | 0.91201864 | | | 48.50 | 0.90221198 | 48.5 | 1.4709978 | 48.5 | 0.90221198 |
| 48.6 | 0.5099459 | 48.6 | 0.63743238 | 48.6 | 0.63743238 | 48.6 | 1.37293128 | 48.6 | 0.91201864 | | | 48.60 | 0.90221198 | 48.6 | 1.46119115 | 48.6 | 0.90221198 |
| 48.7 | 0.53936586 | 48.7 | 0.66685234 | 48.7 | 0.66685234 | 48.7 | 1.16699159 | 48.7 | 0.93163194 | | | 48.70 | 0.91201864 | 48.7 | 1.4513845 | 48.7 | 0.91201864 |
| 48.8 | 0.52955921 | 48.8 | 0.66685234 | 48.8 | 0.66685234 | 48.8 | 1.12776498 | 48.8 | 0.90221198 | | | 48.80 | 0.88259868 | 48.8 | 1.44157784 | 48.8 | 0.88259868 |
| 48.9 | 0.52955921 | 48.9 | 0.64723903 | 48.9 | 0.64723903 | 48.9 | 1.11795833 | 48.9 | 0.92182529 | | | 48.90 | 0.92182529 | 48.9 | 1.44157784 | 48.9 | 0.92182529 |
| 49 | 0.54917251 | 49 | 0.63743238 | 49 | 0.63743238 | 49 | 1.13757163 | 49 | 0.9806652 | | | 49.00 | 0.9610519 | 49 | 1.4709978 | 49 | 0.9610519 |
| 49.1 | 0.54917251 | 49.1 | 0.63743238 | 49.1 | 0.63743238 | 49.1 | 1.10815168 | 49.1 | 0.97085855 | | | 49.10 | 0.94143859 | 49.1 | 1.51022441 | 49.1 | 0.94143859 |
| 49.2 | 0.55897916 | 49.2 | 0.64723903 | 49.2 | 0.64723903 | 49.2 | 1.10815168 | 49.2 | 0.95124524 | | | 49.20 | 0.93163194 | 49.2 | 1.52983771 | 49.2 | 0.93163194 |
| 49.3 | 0.57859247 | 49.3 | 0.65704568 | 49.3 | 0.65704568 | 49.3 | 1.11795833 | 49.3 | 0.94143859 | | | 49.30 | 0.93163194 | 49.3 | 1.51022441 | 49.3 | 0.93163194 |
| 49.4 | 0.58839912 | 49.4 | 0.64723903 | 49.4 | 0.64723903 | 49.4 | 1.12776498 | 49.4 | 0.93163194 | | | 49.40 | 0.93163194 | 49.4 | 1.4906111 | 49.4 | 0.93163194 |
| 49.5 | 0.56878582 | 49.5 | 0.67665899 | 49.5 | 0.67665899 | 49.5 | 1.10815168 | 49.5 | 0.92182529 | | | 49.50 | 0.95124524 | 49.5 | 1.4709978 | 49.5 | 0.95124524 |
| 49.6 | 0.59820577 | 49.6 | 0.66685234 | 49.6 | 0.66685234 | 49.6 | 1.09834502 | 49.6 | 0.94143859 | | | 49.60 | 0.95124524 | 49.6 | 1.52003106 | 49.6 | 0.95124524 |
| 49.7 | 0.60801242 | 49.7 | 0.62762573 | 49.7 | 0.62762573 | 49.7 | 1.10815168 | 49.7 | 0.97085855 | | | 49.70 | 0.95124524 | 49.7 | 1.50041776 | 49.7 | 0.95124524 |
| 49.8 | 0.56878582 | 49.8 | 0.67665899 | 49.8 | 0.67665899 | 49.8 | 1.10815168 | 49.8 | 0.97085855 | | | 49.80 | 1.0002785 | 49.8 | 1.54945102 | 49.8 | 1.0002785 |
| 49.9 | 0.53936586 | 49.9 | 0.69627229 | 49.9 | 0.69627229 | 49.9 | 1.08853837 | 49.9 | 0.9610519 | | | 49.90 | 0.97085855 | 49.9 | 1.56906432 | 49.9 | 0.97085855 |
| 50 | 0.51975256 | 50 | 0.64723903 | 50 | 0.64723903 | 50 | 1.06892507 | 50 | 0.99047185 | | | 50.00 | 0.93163194 | 50 | 1.56906432 | 50 | 0.93163194 |
| 50.1 | 0.56878582 | 50.1 | 0.62762573 | 50.1 | 0.62762573 | 50.1 | 1.05911842 | 50.1 | 1.0002785 | | | 50.10 | 0.9610519 | 50.1 | 1.54945102 | 50.1 | 0.9610519 |
| 50.2 | 0.54917251 | 50.2 | 0.63743238 | 50.2 | 0.63743238 | 50.2 | 1.09834502 | 50.2 | 0.99047185 | | | 50.20 | 0.9806652 | 50.2 | 1.53964436 | 50.2 | 0.9806652 |
| 50.3 | 0.53936586 | 50.3 | 0.67665899 | 50.3 | 0.67665899 | 50.3 | 1.07873172 | 50.3 | 0.9610519 | | | 50.30 | 0.94143859 | 50.3 | 1.52983771 | 50.3 | 0.94143859 |
| 50.4 | 0.54917251 | 50.4 | 0.66685234 | 50.4 | 0.66685234 | 50.4 | 1.07873172 | 50.4 | 0.9610519 | | | 50.40 | 0.92182529 | 50.4 | 1.52983771 | 50.4 | 0.92182529 |
| 50.5 | 0.51975256 | 50.5 | 0.68646564 | 50.5 | 0.68646564 | 50.5 | 1.07873172 | 50.5 | 0.95124524 | | | 50.50 | 0.93163194 | 50.5 | 1.57887097 | 50.5 | 0.93163194 |
| 50.6 | 0.54917251 | 50.6 | 0.69627229 | 50.6 | 0.69627229 | 50.6 | 1.03950511 | 50.6 | 0.91201864 | | | 50.60 | 0.9806652 | 50.6 | 1.54945102 | 50.6 | 0.9806652 |
| 50.7 | 0.56878582 | 50.7 | 0.66685234 | 50.7 | 0.66685234 | 50.7 | 1.01008516 | 50.7 | 1.0002785 | | | 50.70 | 0.9610519 | 50.7 | 1.55925767 | 50.7 | 0.9610519 |
| 50.8 | 0.53936586 | 50.8 | 0.65704568 | 50.8 | 0.65704568 | 50.8 | 1.01989181 | 50.8 | 0.95124524 | | | 50.80 | 0.97085855 | 50.8 | 1.56906432 | 50.8 | 0.97085855 |
| 50.9 | 0.55897916 | 50.9 | 0.69627229 | 50.9 | 0.69627229 | 50.9 | 1.03950511 | 50.9 | 0.91201864 | | | 50.90 | 0.97085855 | 50.9 | 1.59848428 | 50.9 | 0.97085855 |
| 51 | 0.55897916 | 51 | 0.68646564 | 51 | 0.68646564 | 51 | 1.02969846 | 51 | 0.93163194 | | | 51.00 | 0.99047185 | 51 | 1.60829093 | 51 | 0.99047185 |
| 51.1 | 0.53936586 | 51.1 | 0.66685234 | 51.1 | 0.66685234 | 51.1 | 1.02969846 | 51.1 | 0.9806652 | | | 51.10 | 0.99047185 | 51.1 | 1.55925767 | 51.1 | 0.99047185 |
| 51.2 | 0.54917251 | 51.2 | 0.67665899 | 51.2 | 0.67665899 | 51.2 | 1.02969846 | 51.2 | 1.01008516 | | | 51.20 | 1.0002785 | 51.2 | 1.57887097 | 51.2 | 1.0002785 |
| 51.3 | 0.55897916 | 51.3 | 0.67665899 | 51.3 | 0.67665899 | 51.3 | 1.02969846 | 51.3 | 0.97085855 | | | 51.30 | 1.01008516 | 51.3 | 1.57887097 | 51.3 | 1.01008516 |
| 51.4 | 0.52955921 | 51.4 | 0.69627229 | 51.4 | 0.69627229 | 51.4 | 1.03950511 | 51.4 | 1.0002785 | | | 51.40 | 1.0002785 | 51.4 | 1.59848428 | 51.4 | 1.0002785 |
| 51.5 | 0.54917251 | 51.5 | 0.68646564 | 51.5 | 0.68646564 | 51.5 | 1.01008516 | 51.5 | 0.99047185 | | | 51.50 | 0.9806652 | 51.5 | 1.58867762 | 51.5 | 0.9806652 |
| 51.6 | 0.55897916 | 51.6 | 0.69627229 | 51.6 | 0.69627229 | 51.6 | 1.01008516 | 51.6 | 1.01989181 | | | 51.60 | 1.01008516 | 51.6 | 1.61809758 | 51.6 | 1.01008516 |
| 51.7 | 0.53936586 | 51.7 | 0.70607894 | 51.7 | 0.70607894 | 51.7 | 1.02969846 | 51.7 | 1.01989181 | | | 51.70 | 1.01008516 | 51.7 | 1.62790423 | 51.7 | 1.01008516 |
| 51.8 | 0.51975256 | 51.8 | 0.67665899 | 51.8 | 0.67665899 | 51.8 | 1.01989181 | 51.8 | 1.0002785 | | | 51.80 | 1.01008516 | 51.8 | 1.60829093 | 51.8 | 1.01008516 |
| 51.9 | 0.53936586 | 51.9 | 0.70607894 | 51.9 | 0.70607894 | 51.9 | 1.01008516 | 51.9 | 1.0002785 | | | 51.90 | 1.0002785 | 51.9 | 1.64751754 | 51.9 | 1.0002785 |
| 52 | 0.52955921 | 52 | 0.70607894 | 52 | 0.70607894 | 52 | 1.06892507 | 52 | 1.01008516 | | | 52.00 | 1.01989181 | 52 | 1.66713084 | 52 | 1.01989181 |
| 52.1 | 0.52955921 | 52.1 | 0.69627229 | 52.1 | 0.69627229 | 52.1 | 1.04931176 | 52.1 | 0.9806652 | | | 52.10 | 1.04931176 | 52.1 | 1.63771088 | 52.1 | 1.04931176 |

| | | | | | | | | | | | | | | | | | |
|---|---|---|---|---|---|---|---|---|---|---|---|---|---|---|---|---|---|
| 52.2 | 0.52955921 | 52.2 | 0.68646564 | 52.2 | 0.68646564 | 52.2 | 1.02969846 | 52.2 | 0.99047185 | | | 52.20 | 1.01008516 | 52.2 | 1.63771088 | 52.2 | 1.01008516 |
| 52.3 | 0.54917251 | 52.3 | 0.70607894 | 52.3 | 0.70607894 | 52.3 | 1.01008516 | 52.3 | 1.01008516 | | | 52.30 | 1.02969846 | 52.3 | 1.6965508 | 52.3 | 1.02969846 |
| 52.4 | 0.53936586 | 52.4 | 0.69627229 | 52.4 | 0.69627229 | 52.4 | 1.03950511 | 52.4 | 1.01989181 | | | 52.40 | 1.03950511 | 52.4 | 1.65732419 | 52.4 | 1.03950511 |
| 52.5 | 0.52955921 | 52.5 | 0.70607894 | 52.5 | 0.70607894 | 52.5 | 1.03950511 | 52.5 | 1.02969846 | | | 52.50 | 1.01008516 | 52.5 | 1.64751754 | 52.5 | 1.01008516 |
| 52.6 | 0.55897916 | 52.6 | 0.69627229 | 52.6 | 0.69627229 | 52.6 | 1.04931176 | 52.6 | 1.01008516 | | | 52.60 | 1.03950511 | 52.6 | 1.63771088 | 52.6 | 1.03950511 |
| 52.7 | 0.53936586 | 52.7 | 0.69627229 | 52.7 | 0.69627229 | 52.7 | 1.02969846 | 52.7 | 1.01008516 | | | 52.70 | 1.04931176 | 52.7 | 1.67693749 | 52.7 | 1.04931176 |
| 52.8 | 0.51975256 | 52.8 | 0.74530555 | 52.8 | 0.74530555 | 52.8 | 1.03950511 | 52.8 | 1.01989181 | | | 52.80 | 1.01008516 | 52.8 | 1.63771088 | 52.8 | 1.01008516 |
| 52.9 | 0.54917251 | 52.9 | 0.7551122 | 52.9 | 0.7551122 | 52.9 | 1.02969846 | 52.9 | 1.01989181 | | | 52.90 | 1.02969846 | 52.9 | 1.64751754 | 52.9 | 1.02969846 |
| 53 | 0.59820577 | 53 | 0.7551122 | 53 | 0.7551122 | 53 | 1.01989181 | 53 | 1.03950511 | | | 53.00 | 0.99047185 | 53 | 1.67693749 | 53 | 0.99047185 |
| 53.1 | 0.55897916 | 53.1 | 0.72569225 | 53.1 | 0.72569225 | 53.1 | 0.99047185 | 53.1 | 1.08853837 | | | 53.10 | 1.04931176 | 53.1 | 1.66713084 | 53.1 | 1.04931176 |
| 53.2 | 0.53936586 | 53.2 | 0.7158856 | 53.2 | 0.7158856 | 53.2 | 1.0002785 | 53.2 | 1.07873172 | | | 53.20 | 1.05911842 | 53.2 | 1.65732419 | 53.2 | 1.05911842 |
| 53.3 | 0.5099459 | 53.3 | 0.70607894 | 53.3 | 0.70607894 | 53.3 | 1.02969846 | 53.3 | 1.05911842 | | | 53.30 | 1.07873172 | 53.3 | 1.68674414 | 53.3 | 1.07873172 |
| 53.4 | 0.53936586 | 53.4 | 0.70607894 | 53.4 | 0.70607894 | 53.4 | 1.03950511 | 53.4 | 1.07873172 | | | 53.40 | 1.07873172 | 53.4 | 1.70635745 | 53.4 | 1.07873172 |
| 53.5 | 0.55897916 | 53.5 | 0.7354989 | 53.5 | 0.7354989 | 53.5 | 1.04931176 | 53.5 | 1.05911842 | | | 53.50 | 1.03950511 | 53.5 | 1.6965508 | 53.5 | 1.03950511 |
| 53.6 | 0.54917251 | 53.6 | 0.76491886 | 53.6 | 0.76491886 | 53.6 | 1.03950511 | 53.6 | 1.04931176 | | | 53.60 | 1.02969846 | 53.6 | 1.7161641 | 53.6 | 1.02969846 |
| 53.7 | 0.56878582 | 53.7 | 0.72569225 | 53.7 | 0.72569225 | 53.7 | 1.03950511 | 53.7 | 1.07873172 | | | 53.70 | 1.02969846 | 53.7 | 1.7161641 | 53.7 | 1.02969846 |
| 53.8 | 0.56878582 | 53.8 | 0.74530555 | 53.8 | 0.74530555 | 53.8 | 1.01989181 | 53.8 | 1.10815168 | | | 53.80 | 1.05911842 | 53.8 | 1.7357774 | 53.8 | 1.05911842 |
| 53.9 | 0.53936586 | 53.9 | 0.78453216 | 53.9 | 0.78453216 | 53.9 | 1.02969846 | 53.9 | 1.09834502 | | | 53.90 | 1.06892507 | 53.9 | 1.74558406 | 53.9 | 1.06892507 |
| 54 | 0.56878582 | 54 | 0.76491886 | 54 | 0.76491886 | 54 | 1.03950511 | 54 | 1.08853837 | | | 54.00 | 1.07873172 | 54 | 1.7357774 | 54 | 1.07873172 |
| 54.1 | 0.56878582 | 54.1 | 0.7354989 | 54.1 | 0.7354989 | 54.1 | 1.04931176 | 54.1 | 1.08853837 | | | 54.10 | 1.06892507 | 54.1 | 1.75539071 | 54.1 | 1.06892507 |
| 54.2 | 0.5099459 | 54.2 | 0.7354989 | 54.2 | 0.7354989 | 54.2 | 1.03950511 | 54.2 | 1.09834502 | | | 54.20 | 1.08853837 | 54.2 | 1.74558406 | 54.2 | 1.08853837 |
| 54.3 | 0.50013925 | 54.3 | 0.74530555 | 54.3 | 0.74530555 | 54.3 | 1.03950511 | 54.3 | 1.05911842 | | | 54.30 | 1.09834502 | 54.3 | 1.74558406 | 54.3 | 1.09834502 |
| 54.4 | 0.5099459 | 54.4 | 0.77472551 | 54.4 | 0.77472551 | 54.4 | 1.05911842 | 54.4 | 1.11795833 | | | 54.40 | 1.10815168 | 54.4 | 1.74558406 | 54.4 | 1.10815168 |
| 54.5 | 0.55897916 | 54.5 | 0.7551122 | 54.5 | 0.7551122 | 54.5 | 1.04931176 | 54.5 | 1.11795833 | | | 54.50 | 1.11795833 | 54.5 | 1.75539071 | 54.5 | 1.11795833 |
| 54.6 | 0.54917251 | 54.6 | 0.7354989 | 54.6 | 0.7354989 | 54.6 | 1.04931176 | 54.6 | 1.09834502 | | | 54.60 | 1.11795833 | 54.6 | 1.77500401 | 54.6 | 1.11795833 |
| 54.7 | 0.54917251 | 54.7 | 0.7354989 | 54.7 | 0.7354989 | 54.7 | 1.03950511 | 54.7 | 1.11795833 | | | 54.70 | 1.06892507 | 54.7 | 1.76519736 | 54.7 | 1.06892507 |
| 54.8 | 0.54917251 | 54.8 | 0.7354989 | 54.8 | 0.7354989 | 54.8 | 1.05911842 | 54.8 | 1.11795833 | | | 54.80 | 1.05911842 | 54.8 | 1.76519736 | 54.8 | 1.05911842 |
| 54.9 | 0.58839912 | 54.9 | 0.74530555 | 54.9 | 0.74530555 | 54.9 | 1.07873172 | 54.9 | 1.12776498 | | | 54.90 | 1.11795833 | 54.9 | 1.75539071 | 54.9 | 1.11795833 |
| 55 | 0.57859247 | 55 | 0.72569225 | 55 | 0.72569225 | 55 | 1.07873172 | 55 | 1.12776498 | | | 55.00 | 1.14737828 | 55 | 1.76519736 | 55 | 1.14737828 |
| 55.1 | 0.57859247 | 55.1 | 0.7158856 | 55.1 | 0.7158856 | 55.1 | 1.07873172 | 55.1 | 1.12776498 | | | 55.10 | 1.12776498 | 55.1 | 1.79461732 | 55.1 | 1.12776498 |
| 55.2 | 0.56878582 | 55.2 | 0.76491886 | 55.2 | 0.76491886 | 55.2 | 1.07873172 | 55.2 | 1.13757163 | | | 55.20 | 1.10815168 | 55.2 | 1.80442397 | 55.2 | 1.10815168 |
| 55.3 | 0.54917251 | 55.3 | 0.76491886 | 55.3 | 0.76491886 | 55.3 | 1.07873172 | 55.3 | 1.11795833 | | | 55.30 | 1.11795833 | 55.3 | 1.78481066 | 55.3 | 1.11795833 |
| 55.4 | 0.54917251 | 55.4 | 0.7551122 | 55.4 | 0.7551122 | 55.4 | 1.05911842 | 55.4 | 1.16699159 | | | 55.40 | 1.10815168 | 55.4 | 1.76519736 | 55.4 | 1.10815168 |
| 55.5 | 0.55897916 | 55.5 | 0.77472551 | 55.5 | 0.77472551 | 55.5 | 1.09834502 | 55.5 | 1.16699159 | | | 55.50 | 1.09834502 | 55.5 | 1.80442397 | 55.5 | 1.09834502 |
| 55.6 | 0.52955921 | 55.6 | 0.7551122 | 55.6 | 0.7551122 | 55.6 | 1.10815168 | 55.6 | 1.16699159 | | | 55.60 | 1.11795833 | 55.6 | 1.81423062 | 55.6 | 1.11795833 |
| 55.7 | 0.53936586 | 55.7 | 0.74530555 | 55.7 | 0.74530555 | 55.7 | 1.06892507 | 55.7 | 1.14737828 | | | 55.70 | 1.10815168 | 55.7 | 1.82403727 | 55.7 | 1.10815168 |
| 55.8 | 0.57859247 | 55.8 | 0.76491886 | 55.8 | 0.76491886 | 55.8 | 1.06892507 | 55.8 | 1.15718494 | | | 55.80 | 1.09834502 | 55.8 | 1.80442397 | 55.8 | 1.09834502 |
| 55.9 | 0.56878582 | 55.9 | 0.79433881 | 55.9 | 0.79433881 | 55.9 | 1.09834502 | 55.9 | 1.16699159 | | | 55.90 | 1.10815168 | 55.9 | 1.79461732 | 55.9 | 1.10815168 |
| 56 | 0.53936586 | 56 | 0.7551122 | 56 | 0.7551122 | 56 | 1.07873172 | 56 | 1.18660489 | | | 56.00 | 1.09834502 | 56 | 1.80442397 | 56 | 1.09834502 |
| 56.1 | 0.55897916 | 56.1 | 0.74530555 | 56.1 | 0.74530555 | 56.1 | 1.08853837 | 56.1 | 1.2062182 | | | 56.10 | 1.08853837 | 56.1 | 1.84365058 | 56.1 | 1.08853837 |
| 56.2 | 0.53936586 | 56.2 | 0.76491886 | 56.2 | 0.76491886 | 56.2 | 1.09834502 | 56.2 | 1.18660489 | | | 56.20 | 1.13757163 | 56.2 | 1.87307053 | 56.2 | 1.13757163 |
| 56.3 | 0.53936586 | 56.3 | 0.78453216 | 56.3 | 0.78453216 | 56.3 | 1.09834502 | 56.3 | 1.15718494 | | | 56.30 | 1.17679824 | 56.3 | 1.84365058 | 56.3 | 1.17679824 |
| 56.4 | 0.55897916 | 56.4 | 0.77472551 | 56.4 | 0.77472551 | 56.4 | 1.10815168 | 56.4 | 1.17679824 | | | 56.40 | 1.17679824 | 56.4 | 1.82403727 | 56.4 | 1.17679824 |
| 56.5 | 0.56878582 | 56.5 | 0.79433881 | 56.5 | 0.79433881 | 56.5 | 1.11795833 | 56.5 | 1.17679824 | | | 56.50 | 1.10815168 | 56.5 | 1.82403727 | 56.5 | 1.10815168 |
| 56.6 | 0.56878582 | 56.6 | 0.78453216 | 56.6 | 0.78453216 | 56.6 | 1.11795833 | 56.6 | 1.15718494 | | | 56.60 | 1.14737828 | 56.6 | 1.83384392 | 56.6 | 1.14737828 |
| 56.7 | 0.58839912 | 56.7 | 0.77472551 | 56.7 | 0.77472551 | 56.7 | 1.12776498 | 56.7 | 1.19641154 | | | 56.70 | 1.14737828 | 56.7 | 1.87307053 | 56.7 | 1.14737828 |
| 56.8 | 0.59820577 | 56.8 | 0.78453216 | 56.8 | 0.78453216 | 56.8 | 1.18660489 | 56.8 | 1.18660489 | | | 56.80 | 1.15718494 | 56.8 | 1.89268384 | 56.8 | 1.15718494 |
| 56.9 | 0.53936586 | 56.9 | 0.77472551 | 56.9 | 0.77472551 | 56.9 | 1.11795833 | 56.9 | 1.17679824 | | | 56.90 | 1.16699159 | 56.9 | 1.86326388 | 56.9 | 1.16699159 |
| 57 | 0.53936586 | 57 | 0.79433881 | 57 | 0.79433881 | 57 | 1.05911842 | 57 | 1.18660489 | | | 57.00 | 1.15718494 | 57 | 1.86326388 | 57 | 1.15718494 |
| 57.1 | 0.55897916 | 57.1 | 0.81395212 | 57.1 | 0.81395212 | 57.1 | 1.13757163 | 57.1 | 1.18660489 | | | 57.10 | 1.15718494 | 57.1 | 1.89268384 | 57.1 | 1.15718494 |
| 57.2 | 0.57859247 | 57.2 | 0.79433881 | 57.2 | 0.79433881 | 57.2 | 1.13757163 | 57.2 | 1.17679824 | | | 57.20 | 1.16699159 | 57.2 | 1.88287718 | 57.2 | 1.16699159 |
| 57.3 | 0.56878582 | 57.3 | 0.79433881 | 57.3 | 0.79433881 | 57.3 | 1.13757163 | 57.3 | 1.18660489 | | | 57.30 | 1.14737828 | 57.3 | 1.86326388 | 57.3 | 1.14737828 |
| 57.4 | 0.57859247 | 57.4 | 0.79433881 | 57.4 | 0.79433881 | 57.4 | 1.16699159 | 57.4 | 1.17679824 | | | 57.40 | 1.15718494 | 57.4 | 1.88287718 | 57.4 | 1.15718494 |
| 57.5 | 0.58839912 | 57.5 | 0.78453216 | 57.5 | 0.78453216 | 57.5 | 1.19641154 | 57.5 | 1.17679824 | | | 57.50 | 1.17679824 | 57.5 | 1.89268384 | 57.5 | 1.17679824 |
| 57.6 | 0.56878582 | 57.6 | 0.76491886 | 57.6 | 0.76491886 | 57.6 | 1.16699159 | 57.6 | 1.21602485 | | | 57.60 | 1.16699159 | 57.6 | 1.90249049 | 57.6 | 1.16699159 |
| 57.7 | 0.56878582 | 57.7 | 0.80414546 | 57.7 | 0.80414546 | 57.7 | 1.18660489 | 57.7 | 1.2062182 | | | 57.70 | 1.18660489 | 57.7 | 1.92210379 | 57.7 | 1.18660489 |
| 57.8 | 0.57859247 | 57.8 | 0.77472551 | 57.8 | 0.77472551 | 57.8 | 1.19641154 | 57.8 | 1.14737828 | | | 57.80 | 1.18660489 | 57.8 | 1.93191044 | 57.8 | 1.18660489 |
| 57.9 | 0.59820577 | 57.9 | 0.80414546 | 57.9 | 0.80414546 | 57.9 | 1.16699159 | 57.9 | 1.15718494 | | | 57.90 | 1.18660489 | 57.9 | 1.9417171 | 57.9 | 1.18660489 |
| 58 | 0.58839912 | 58 | 0.78453216 | 58 | 0.78453216 | 58 | 1.21602485 | 58 | 1.14737828 | | | 58.00 | 1.18660489 | 58 | 1.93191044 | 58 | 1.18660489 |
| 58.1 | 0.57859247 | 58.1 | 0.81395212 | 58.1 | 0.81395212 | 58.1 | 1.21602485 | 58.1 | 1.16699159 | | | 58.10 | 1.17679824 | 58.1 | 1.95152375 | 58.1 | 1.17679824 |
| 58.2 | 0.56878582 | 58.2 | 0.85317872 | 58.2 | 0.85317872 | 58.2 | 1.2062182 | 58.2 | 1.21602485 | | | 58.20 | 1.17679824 | 58.2 | 1.9613304 | 58.2 | 1.17679824 |

| | | | | | | | | | | | | | | | | | |
|---|---|---|---|---|---|---|---|---|---|---|---|---|---|---|---|---|---|
| 58.3 | 0.55897916 | 58.3 | 0.81395212 | 58.3 | 0.81395212 | 58.3 | 1.19641154 | 58.3 | 1.21602485 | | | 58.30 | 1.17679824 | 58.3 | 1.9809437 | 58.3 | 1.17679824 |
| 58.4 | 0.55897916 | 58.4 | 0.81395212 | 58.4 | 0.81395212 | 58.4 | 1.23563815 | 58.4 | 1.19641154 | | | 58.40 | 1.2062182 | 58.4 | 1.9809437 | 58.4 | 1.2062182 |
| 58.5 | 0.55897916 | 58.5 | 0.81395212 | 58.5 | 0.81395212 | 58.5 | 1.19641154 | 58.5 | 1.19641154 | | | 58.50 | 1.2062182 | 58.5 | 1.9613304 | 58.5 | 1.2062182 |
| 58.6 | 0.56878582 | 58.6 | 0.83356542 | 58.6 | 0.83356542 | 58.6 | 1.17679824 | 58.6 | 1.21602485 | | | 58.60 | 1.2258315 | 58.6 | 1.9613304 | 58.6 | 1.2258315 |
| 58.7 | 0.55897916 | 58.7 | 0.83356542 | 58.7 | 0.83356542 | 58.7 | 1.19641154 | 58.7 | 1.2258315 | | | 58.70 | 1.19641154 | 58.7 | 1.97113705 | 58.7 | 1.19641154 |
| 58.8 | 0.54917251 | 58.8 | 0.83356542 | 58.8 | 0.83356542 | 58.8 | 1.19641154 | 58.8 | 1.17679824 | | | 58.80 | 1.19641154 | 58.8 | 1.9809437 | 58.8 | 1.19641154 |
| 58.9 | 0.55897916 | 58.9 | 0.82375877 | 58.9 | 0.82375877 | 58.9 | 1.19641154 | 58.9 | 1.2258315 | | | 58.90 | 1.2062182 | 58.9 | 2.01036366 | 58.9 | 1.2062182 |
| 59 | 0.56878582 | 59 | 0.80414546 | 59 | 0.80414546 | 59 | 1.2258315 | 59 | 1.25525146 | | | 59.00 | 1.17679824 | 59 | 2.01036366 | 59 | 1.17679824 |
| 59.1 | 0.57859247 | 59.1 | 0.81395212 | 59.1 | 0.81395212 | 59.1 | 1.19641154 | 59.1 | 1.25525146 | | | 59.10 | 1.19641154 | 59.1 | 2.01036366 | 59.1 | 1.19641154 |
| 59.2 | 0.56878582 | 59.2 | 0.83356542 | 59.2 | 0.83356542 | 59.2 | 1.18660489 | 59.2 | 1.2454448 | | | 59.20 | 1.21602485 | 59.2 | 1.9809437 | 59.2 | 1.21602485 |
| 59.3 | 0.56878582 | 59.3 | 0.83356542 | 59.3 | 0.83356542 | 59.3 | 1.23563815 | 59.3 | 1.2454448 | | | 59.30 | 1.23563815 | 59.3 | 1.99075036 | 59.3 | 1.23563815 |
| 59.4 | 0.54917251 | 59.4 | 0.86298538 | 59.4 | 0.86298538 | 59.4 | 1.19641154 | 59.4 | 1.27486476 | | | 59.40 | 1.2258315 | 59.4 | 2.02997696 | 59.4 | 1.2258315 |
| 59.5 | 0.54917251 | 59.5 | 0.85317872 | 59.5 | 0.85317872 | 59.5 | 1.2062182 | 59.5 | 1.26505811 | | | 59.50 | 1.2454448 | 59.5 | 2.02997696 | 59.5 | 1.2454448 |
| 59.6 | 0.55897916 | 59.6 | 0.84337207 | 59.6 | 0.84337207 | 59.6 | 1.21602485 | 59.6 | 1.27486476 | | | 59.60 | 1.23563815 | 59.6 | 2.02997696 | 59.6 | 1.23563815 |
| 59.7 | 0.56878582 | 59.7 | 0.82375877 | 59.7 | 0.82375877 | 59.7 | 1.2258315 | 59.7 | 1.29447806 | | | 59.70 | 1.2258315 | 59.7 | 2.04959027 | 59.7 | 1.2258315 |
| 59.8 | 0.58839912 | 59.8 | 0.81395212 | 59.8 | 0.81395212 | 59.8 | 1.19641154 | 59.8 | 1.26505811 | | | 59.80 | 1.21602485 | 59.8 | 2.01036366 | 59.8 | 1.21602485 |
| 59.9 | 0.59820577 | 59.9 | 0.84337207 | 59.9 | 0.84337207 | 59.9 | 1.2258315 | 59.9 | 1.2454448 | | | 59.90 | 1.2258315 | 59.9 | 2.01036366 | 59.9 | 1.2258315 |
| 60 | 0.60801242 | 60 | 0.83356542 | 60 | 0.83356542 | 60 | 1.2454448 | 60 | 1.23563815 | | | 60.00 | 1.26505811 | 60 | 2.02997696 | 60 | 1.26505811 |
| 60.1 | 0.59820577 | 60.1 | 0.82375877 | 60.1 | 0.82375877 | 60.1 | 1.26505811 | 60.1 | 1.2454448 | | | 60.10 | 1.23563815 | 60.1 | 2.06920357 | 60.1 | 1.23563815 |
| 60.2 | 0.56878582 | 60.2 | 0.83356542 | 60.2 | 0.83356542 | 60.2 | 1.2062182 | 60.2 | 1.30428472 | | | 60.20 | 1.26505811 | 60.2 | 2.08881688 | 60.2 | 1.26505811 |
| 60.3 | 0.54917251 | 60.3 | 0.86298538 | 60.3 | 0.86298538 | 60.3 | 1.2454448 | 60.3 | 1.33370467 | | | 60.30 | 1.2454448 | 60.3 | 2.06920357 | 60.3 | 1.2454448 |
| 60.4 | 0.56878582 | 60.4 | 0.85317872 | 60.4 | 0.85317872 | 60.4 | 1.26505811 | 60.4 | 1.29447806 | | | 60.40 | 1.23563815 | 60.4 | 2.06920357 | 60.4 | 1.23563815 |
| 60.5 | 0.57859247 | 60.5 | 0.86298538 | 60.5 | 0.86298538 | 60.5 | 1.23563815 | 60.5 | 1.31409137 | | | 60.50 | 1.23563815 | 60.5 | 2.07901022 | 60.5 | 1.23563815 |
| 60.6 | 0.58839912 | 60.6 | 0.87279203 | 60.6 | 0.87279203 | 60.6 | 1.21602485 | 60.6 | 1.34351132 | | | 60.60 | 1.23563815 | 60.6 | 2.08881688 | 60.6 | 1.23563815 |
| 60.7 | 0.58839912 | 60.7 | 0.90221198 | 60.7 | 0.90221198 | 60.7 | 1.2258315 | 60.7 | 1.34351132 | | | 60.70 | 1.23563815 | 60.7 | 2.11823683 | 60.7 | 1.23563815 |
| 60.8 | 0.54917251 | 60.8 | 0.89240533 | 60.8 | 0.89240533 | 60.8 | 1.2062182 | 60.8 | 1.31409137 | | | 60.80 | 1.2454448 | 60.8 | 2.12804348 | 60.8 | 1.2454448 |
| 60.9 | 0.57859247 | 60.9 | 0.87279203 | 60.9 | 0.87279203 | 60.9 | 1.2258315 | 60.9 | 1.29447806 | | | 60.90 | 1.23563815 | 60.9 | 2.09862353 | 60.9 | 1.23563815 |
| 61 | 0.57859247 | 61 | 0.87279203 | 61 | 0.87279203 | 61 | 1.2454448 | 61 | 1.32389802 | | | 61.00 | 1.2258315 | 61 | 2.08881688 | 61 | 1.2258315 |
| 61.1 | 0.55897916 | 61.1 | 0.85317872 | 61.1 | 0.85317872 | 61.1 | 1.2258315 | 61.1 | 1.34351132 | | | 61.10 | 1.2454448 | 61.1 | 2.11823683 | 61.1 | 1.2454448 |
| 61.2 | 0.57859247 | 61.2 | 0.84337207 | 61.2 | 0.84337207 | 61.2 | 1.23563815 | 61.2 | 1.31409137 | | | 61.20 | 1.2454448 | 61.2 | 2.09862353 | 61.2 | 1.2454448 |
| 61.3 | 0.59820577 | 61.3 | 0.85317872 | 61.3 | 0.85317872 | 61.3 | 1.27486476 | 61.3 | 1.31409137 | | | 61.30 | 1.23563815 | 61.3 | 2.08881688 | 61.3 | 1.23563815 |
| 61.4 | 0.59820577 | 61.4 | 0.87279203 | 61.4 | 0.87279203 | 61.4 | 1.27486476 | 61.4 | 1.32389802 | | | 61.40 | 1.2258315 | 61.4 | 2.07901022 | 61.4 | 1.2258315 |
| 61.5 | 0.56878582 | 61.5 | 0.87279203 | 61.5 | 0.87279203 | 61.5 | 1.25525146 | 61.5 | 1.33370467 | | | 61.50 | 1.23563815 | 61.5 | 2.07901022 | 61.5 | 1.23563815 |
| 61.6 | 0.53936586 | 61.6 | 0.87279203 | 61.6 | 0.87279203 | 61.6 | 1.27486476 | 61.6 | 1.31409137 | | | 61.60 | 1.2454448 | 61.6 | 2.11823683 | 61.6 | 1.2454448 |
| 61.7 | 0.57859247 | 61.7 | 0.86298538 | 61.7 | 0.86298538 | 61.7 | 1.2454448 | 61.7 | 1.31409137 | | | 61.70 | 1.2454448 | 61.7 | 2.14765679 | 61.7 | 1.2454448 |
| 61.8 | 0.56878582 | 61.8 | 0.90221198 | 61.8 | 0.90221198 | 61.8 | 1.26505811 | 61.8 | 1.33370467 | | | 61.80 | 1.30428472 | 61.8 | 2.15746344 | 61.8 | 1.30428472 |
| 61.9 | 0.54917251 | 61.9 | 0.87279203 | 61.9 | 0.87279203 | 61.9 | 1.2454448 | 61.9 | 1.36312463 | | | 61.90 | 1.31409137 | 61.9 | 2.17707674 | 61.9 | 1.31409137 |
| 62 | 0.55897916 | 62 | 0.85317872 | 62 | 0.85317872 | 62 | 1.25525146 | 62 | 1.36312463 | | | 62.00 | 1.2454448 | 62 | 2.15746344 | 62 | 1.2454448 |
| 62.1 | 0.55897916 | 62.1 | 0.86298538 | 62.1 | 0.86298538 | 62.1 | 1.25525146 | 62.1 | 1.34351132 | | | 62.10 | 1.23563815 | 62.1 | 2.14765679 | 62.1 | 1.23563815 |
| 62.2 | 0.54917251 | 62.2 | 0.86298538 | 62.2 | 0.86298538 | 62.2 | 1.28467141 | 62.2 | 1.36312463 | | | 62.20 | 1.27486476 | 62.2 | 2.16727009 | 62.2 | 1.27486476 |
| 62.3 | 0.57859247 | 62.3 | 0.88259868 | 62.3 | 0.88259868 | 62.3 | 1.27486476 | 62.3 | 1.33370467 | | | 62.30 | 1.28467141 | 62.3 | 2.1868834 | 62.3 | 1.28467141 |
| 62.4 | 0.58839912 | 62.4 | 0.88259868 | 62.4 | 0.88259868 | 62.4 | 1.26505811 | 62.4 | 1.35331798 | | | 62.40 | 1.27486476 | 62.4 | 2.19669005 | 62.4 | 1.27486476 |
| 62.5 | 0.56878582 | 62.5 | 0.88259868 | 62.5 | 0.88259868 | 62.5 | 1.30428472 | 62.5 | 1.37293128 | | | 62.50 | 1.28467141 | 62.5 | 2.1868834 | 62.5 | 1.28467141 |
| 62.6 | 0.57859247 | 62.6 | 0.90221198 | 62.6 | 0.90221198 | 62.6 | 1.26505811 | 62.6 | 1.38273793 | | | 62.60 | 1.26505811 | 62.6 | 2.19669005 | 62.6 | 1.26505811 |
| 62.7 | 0.55897916 | 62.7 | 0.92182529 | 62.7 | 0.92182529 | 62.7 | 1.2454448 | 62.7 | 1.36312463 | | | 62.70 | 1.28467141 | 62.7 | 2.17707674 | 62.7 | 1.28467141 |
| 62.8 | 0.57859247 | 62.8 | 0.90221198 | 62.8 | 0.90221198 | 62.8 | 1.26505811 | 62.8 | 1.38273793 | | | 62.80 | 1.30428472 | 62.8 | 2.17707674 | 62.8 | 1.30428472 |
| 62.9 | 0.59820577 | 62.9 | 0.91201864 | 62.9 | 0.91201864 | 62.9 | 1.29447806 | 62.9 | 1.37293128 | | | 62.90 | 1.29447806 | 62.9 | 2.1868834 | 62.9 | 1.29447806 |
| 63 | 0.58839912 | 63 | 0.88259868 | 63 | 0.88259868 | 63 | 1.29447806 | 63 | 1.41215789 | | | 63.00 | 1.25525146 | 63 | 2.19669005 | 63 | 1.25525146 |
| 63.1 | 0.60801242 | 63.1 | 0.91201864 | 63.1 | 0.91201864 | 63.1 | 1.31409137 | 63.1 | 1.38273793 | | | 63.10 | 1.27486476 | 63.1 | 2.2064967 | 63.1 | 1.27486476 |
| 63.2 | 0.57859247 | 63.2 | 0.88259868 | 63.2 | 0.88259868 | 63.2 | 1.27486476 | 63.2 | 1.38273793 | | | 63.20 | 1.30428472 | 63.2 | 2.21630335 | 63.2 | 1.30428472 |
| 63.3 | 0.59820577 | 63.3 | 0.88259868 | 63.3 | 0.88259868 | 63.3 | 1.2454448 | 63.3 | 1.38273793 | | | 63.30 | 1.33370467 | 63.3 | 2.2064967 | 63.3 | 1.33370467 |
| 63.4 | 0.59820577 | 63.4 | 0.92182529 | 63.4 | 0.92182529 | 63.4 | 1.2454448 | 63.4 | 1.40235124 | | | 63.40 | 1.34351132 | 63.4 | 2.22611 | 63.4 | 1.34351132 |
| 63.5 | 0.61781908 | 63.5 | 0.92182529 | 63.5 | 0.92182529 | 63.5 | 1.27486476 | 63.5 | 1.41215789 | | | 63.50 | 1.31409137 | 63.5 | 2.21630335 | 63.5 | 1.31409137 |
| 63.6 | 0.59820577 | 63.6 | 0.91201864 | 63.6 | 0.91201864 | 63.6 | 1.25525146 | 63.6 | 1.39254458 | | | 63.60 | 1.30428472 | 63.6 | 2.23591666 | 63.6 | 1.30428472 |
| 63.7 | 0.59820577 | 63.7 | 0.90221198 | 63.7 | 0.90221198 | 63.7 | 1.27486476 | 63.7 | 1.38273793 | | | 63.70 | 1.31409137 | 63.7 | 2.25552996 | 63.7 | 1.31409137 |
| 63.8 | 0.59820577 | 63.8 | 0.91201864 | 63.8 | 0.91201864 | 63.8 | 1.31409137 | 63.8 | 1.40235124 | | | 63.80 | 1.31409137 | 63.8 | 2.26533661 | 63.8 | 1.31409137 |
| 63.9 | 0.59820577 | 63.9 | 0.92182529 | 63.9 | 0.92182529 | 63.9 | 1.31409137 | 63.9 | 1.39254458 | | | 63.90 | 1.31409137 | 63.9 | 2.25552996 | 63.9 | 1.31409137 |
| 64 | 0.58839912 | 64 | 0.90221198 | 64 | 0.90221198 | 64 | 1.29447806 | 64 | 1.43177119 | | | 64.00 | 1.30428472 | 64 | 2.27514326 | 64 | 1.30428472 |
| 64.1 | 0.57859247 | 64.1 | 0.91201864 | 64.1 | 0.91201864 | 64.1 | 1.34351132 | 64.1 | 1.42196454 | | | 64.10 | 1.30428472 | 64.1 | 2.29475657 | 64.1 | 1.30428472 |
| 64.2 | 0.55897916 | 64.2 | 0.95124524 | 64.2 | 0.95124524 | 64.2 | 1.32389802 | 64.2 | 1.41215789 | | | 64.20 | 1.31409137 | 64.2 | 2.30456322 | 64.2 | 1.31409137 |
| 64.3 | 0.56878582 | 64.3 | 0.95124524 | 64.3 | 0.95124524 | 64.3 | 1.31409137 | 64.3 | 1.40235124 | | | 64.30 | 1.34351132 | 64.3 | 2.28494992 | 64.3 | 1.34351132 |

| | | | | | | | | | | | | | | | | | |
|---|---|---|---|---|---|---|---|---|---|---|---|---|---|---|---|---|---|
| 64.4 | 0.56878582 | 64.4 | 0.94143859 | 64.4 | 0.94143859 | 64.4 | 1.28467141 | 64.4 | 1.41215789 | | | 64.40 | 1.34351132 | 64.4 | 2.29475657 | 64.4 | 1.34351132 |
| 64.5 | 0.54917251 | 64.5 | 0.9610519 | 64.5 | 0.9610519 | 64.5 | 1.29447806 | 64.5 | 1.46119115 | | | 64.50 | 1.32389802 | 64.5 | 2.29475657 | 64.5 | 1.32389802 |
| 64.6 | 0.55897916 | 64.6 | 0.9806652 | 64.6 | 0.9806652 | 64.6 | 1.30428472 | 64.6 | 1.4709978 | | | 64.60 | 1.31409137 | 64.6 | 2.29475657 | 64.6 | 1.31409137 |
| 64.7 | 0.57859247 | 64.7 | 0.95124524 | 64.7 | 0.95124524 | 64.7 | 1.32389802 | 64.7 | 1.4513845 | | | 64.70 | 1.33370467 | 64.7 | 2.30456322 | 64.7 | 1.33370467 |
| 64.8 | 0.55897916 | 64.8 | 0.93163194 | 64.8 | 0.93163194 | 64.8 | 1.34351132 | 64.8 | 1.44157784 | | | 64.80 | 1.35331798 | 64.8 | 2.29475657 | 64.8 | 1.35331798 |
| 64.9 | 0.55897916 | 64.9 | 0.94143859 | 64.9 | 0.94143859 | 64.9 | 1.36312463 | 64.9 | 1.46119115 | | | 64.90 | 1.33370467 | 64.9 | 2.29475657 | 64.9 | 1.33370467 |
| 65 | 0.55897916 | 65 | 0.91201864 | 65 | 0.91201864 | 65 | 1.36312463 | 65 | 1.46119115 | | | 65.00 | 1.34351132 | 65 | 2.31436987 | 65 | 1.34351132 |
| 65.1 | 0.58839912 | 65.1 | 0.90221198 | 65.1 | 0.90221198 | 65.1 | 1.29447806 | 65.1 | 1.46119115 | | | 65.10 | 1.37293128 | 65.1 | 2.31436987 | 65.1 | 1.37293128 |
| 65.2 | 0.58839912 | 65.2 | 0.97085855 | 65.2 | 0.97085855 | 65.2 | 1.29447806 | 65.2 | 1.4709978 | | | 65.20 | 1.35331798 | 65.2 | 2.31436987 | 65.2 | 1.35331798 |
| 65.3 | 0.60801242 | 65.3 | 0.99047185 | 65.3 | 0.99047185 | 65.3 | 1.32389802 | 65.3 | 1.51022441 | | | 65.30 | 1.36312463 | 65.3 | 2.30456322 | 65.3 | 1.36312463 |
| 65.4 | 0.58839912 | 65.4 | 0.99047185 | 65.4 | 0.99047185 | 65.4 | 1.34351132 | 65.4 | 1.50041776 | | | 65.40 | 1.36312463 | 65.4 | 2.29475657 | 65.4 | 1.36312463 |
| 65.5 | 0.57859247 | 65.5 | 0.97085855 | 65.5 | 0.97085855 | 65.5 | 1.33370467 | 65.5 | 1.4906111 | | | 65.50 | 1.36312463 | 65.5 | 2.32417652 | 65.5 | 1.36312463 |
| 65.6 | 0.59820577 | 65.6 | 0.9806652 | 65.6 | 0.9806652 | 65.6 | 1.33370467 | 65.6 | 1.51022441 | | | 65.60 | 1.33370467 | 65.6 | 2.32417652 | 65.6 | 1.33370467 |
| 65.7 | 0.58839912 | 65.7 | 0.99047185 | 65.7 | 0.99047185 | 65.7 | 1.32389802 | 65.7 | 1.53964436 | | | 65.70 | 1.38273793 | 65.7 | 2.34378983 | 65.7 | 1.38273793 |
| 65.8 | 0.55897916 | 65.8 | 0.9610519 | 65.8 | 0.9610519 | 65.8 | 1.35331798 | 65.8 | 1.54945102 | | | 65.80 | 1.36312463 | 65.8 | 2.32417652 | 65.8 | 1.36312463 |
| 65.9 | 0.56878582 | 65.9 | 0.95124524 | 65.9 | 0.95124524 | 65.9 | 1.37293128 | 65.9 | 1.52003106 | | | 65.90 | 1.39254458 | 65.9 | 2.31436987 | 65.9 | 1.39254458 |
| 66 | 0.59820577 | 66 | 0.97085855 | 66 | 0.97085855 | 66 | 1.37293128 | 66 | 1.4906111 | | | 66.00 | 1.35331798 | 66 | 2.31436987 | 66 | 1.35331798 |
| 66.1 | 0.58839912 | 66.1 | 0.97085855 | 66.1 | 0.97085855 | 66.1 | 1.36312463 | 66.1 | 1.52003106 | | | 66.10 | 1.37293128 | 66.1 | 2.34378983 | 66.1 | 1.37293128 |
| 66.2 | 0.57859247 | 66.2 | 0.9806652 | 66.2 | 0.9806652 | 66.2 | 1.39254458 | 66.2 | 1.53964436 | | | 66.20 | 1.38273793 | 66.2 | 2.35359648 | 66.2 | 1.38273793 |
| 66.3 | 0.58839912 | 66.3 | 0.99047185 | 66.3 | 0.99047185 | 66.3 | 1.38273793 | 66.3 | 1.52983771 | | | 66.30 | 1.36312463 | 66.3 | 2.35359648 | 66.3 | 1.36312463 |
| 66.4 | 0.61781908 | 66.4 | 0.9610519 | 66.4 | 0.9610519 | 66.4 | 1.36312463 | 66.4 | 1.53964436 | | | 66.40 | 1.34351132 | 66.4 | 2.36340313 | 66.4 | 1.34351132 |
| 66.5 | 0.61781908 | 66.5 | 0.9610519 | 66.5 | 0.9610519 | 66.5 | 1.37293128 | 66.5 | 1.55925767 | | | 66.50 | 1.39254458 | 66.5 | 2.36340313 | 66.5 | 1.39254458 |
| 66.6 | 0.60801242 | 66.6 | 0.99047185 | 66.6 | 0.99047185 | 66.6 | 1.36312463 | 66.6 | 1.57887097 | | | 66.60 | 1.37293128 | 66.6 | 2.36340313 | 66.6 | 1.37293128 |
| 66.7 | 0.61781908 | 66.7 | 0.99047185 | 66.7 | 0.99047185 | 66.7 | 1.38273793 | 66.7 | 1.56906432 | | | 66.70 | 1.38273793 | 66.7 | 2.36340313 | 66.7 | 1.38273793 |
| 66.8 | 0.59820577 | 66.8 | 0.9806652 | 66.8 | 0.9806652 | 66.8 | 1.37293128 | 66.8 | 1.58867762 | | | 66.80 | 1.41215789 | 66.8 | 2.36340313 | 66.8 | 1.41215789 |
| 66.9 | 0.59820577 | 66.9 | 0.97085855 | 66.9 | 0.97085855 | 66.9 | 1.40235124 | 66.9 | 1.58867762 | | | 66.90 | 1.38273793 | 66.9 | 2.38301644 | 66.9 | 1.38273793 |
| 67 | 0.57859247 | 67 | 0.9806652 | 67 | 0.9806652 | 67 | 1.41215789 | 67 | 1.59848428 | | | 67.00 | 1.38273793 | 67 | 2.36340313 | 67 | 1.38273793 |
| 67.1 | 0.57859247 | 67.1 | 1.01008516 | 67.1 | 1.01008516 | 67.1 | 1.36312463 | 67.1 | 1.57887097 | | | 67.10 | 1.41215789 | 67.1 | 2.36340313 | 67.1 | 1.41215789 |
| 67.2 | 0.57859247 | 67.2 | 1.01989181 | 67.2 | 1.01989181 | 67.2 | 1.38273793 | 67.2 | 1.58867762 | | | 67.20 | 1.37293128 | 67.2 | 2.37320978 | 67.2 | 1.37293128 |
| 67.3 | 0.58839912 | 67.3 | 1.01989181 | 67.3 | 1.01989181 | 67.3 | 1.39254458 | 67.3 | 1.56906432 | | | 67.30 | 1.37293128 | 67.3 | 2.41243639 | 67.3 | 1.37293128 |
| 67.4 | 0.59820577 | 67.4 | 1.01989181 | 67.4 | 1.01989181 | 67.4 | 1.40235124 | 67.4 | 1.56906432 | | | 67.40 | 1.41215789 | 67.4 | 2.42224304 | 67.4 | 1.41215789 |
| 67.5 | 0.61781908 | 67.5 | 1.01989181 | 67.5 | 1.01989181 | 67.5 | 1.39254458 | 67.5 | 1.59848428 | | | 67.50 | 1.40235124 | 67.5 | 2.41243639 | 67.5 | 1.40235124 |
| 67.6 | 0.61781908 | 67.6 | 0.99047185 | 67.6 | 0.99047185 | 67.6 | 1.39254458 | 67.6 | 1.60829093 | | | 67.60 | 1.41215789 | 67.6 | 2.42224304 | 67.6 | 1.41215789 |
| 67.7 | 0.57859247 | 67.7 | 1.0002785 | 67.7 | 1.0002785 | 67.7 | 1.40235124 | 67.7 | 1.59848428 | | | 67.70 | 1.40235124 | 67.7 | 2.44185635 | 67.7 | 1.40235124 |
| 67.8 | 0.59820577 | 67.8 | 1.0002785 | 67.8 | 1.0002785 | 67.8 | 1.39254458 | 67.8 | 1.61809758 | | | 67.80 | 1.42196454 | 67.8 | 2.44185635 | 67.8 | 1.42196454 |
| 67.9 | 0.59820577 | 67.9 | 0.99047185 | 67.9 | 0.99047185 | 67.9 | 1.39254458 | 67.9 | 1.65732419 | | | 67.90 | 1.43177119 | 67.9 | 2.42224304 | 67.9 | 1.43177119 |
| 68 | 0.56878582 | 68 | 1.01008516 | 68 | 1.01008516 | 68 | 1.40235124 | 68 | 1.63771088 | | | 68.00 | 1.42196454 | 68 | 2.4320497 | 68 | 1.42196454 |
| 68.1 | 0.58839912 | 68.1 | 1.02969846 | 68.1 | 1.02969846 | 68.1 | 1.40235124 | 68.1 | 1.60829093 | | | 68.10 | 1.42196454 | 68.1 | 2.41243639 | 68.1 | 1.42196454 |
| 68.2 | 0.61781908 | 68.2 | 1.03950511 | 68.2 | 1.03950511 | 68.2 | 1.37293128 | 68.2 | 1.60829093 | | | 68.20 | 1.4513845 | 68.2 | 2.42224304 | 68.2 | 1.4513845 |
| 68.3 | 0.60801242 | 68.3 | 1.01989181 | 68.3 | 1.01989181 | 68.3 | 1.39254458 | 68.3 | 1.67693749 | | | 68.30 | 1.42196454 | 68.3 | 2.42224304 | 68.3 | 1.42196454 |
| 68.4 | 0.61781908 | 68.4 | 1.01008516 | 68.4 | 1.01008516 | 68.4 | 1.40235124 | 68.4 | 1.66713084 | | | 68.40 | 1.39254458 | 68.4 | 2.44185635 | 68.4 | 1.39254458 |
| 68.5 | 0.60801242 | 68.5 | 1.01989181 | 68.5 | 1.01989181 | 68.5 | 1.39254458 | 68.5 | 1.65732419 | | | 68.50 | 1.42196454 | 68.5 | 2.44185635 | 68.5 | 1.42196454 |
| 68.6 | 0.58839912 | 68.6 | 1.03950511 | 68.6 | 1.03950511 | 68.6 | 1.37293128 | 68.6 | 1.65732419 | | | 68.60 | 1.46119115 | 68.6 | 2.44185635 | 68.6 | 1.46119115 |
| 68.7 | 0.57859247 | 68.7 | 1.02969846 | 68.7 | 1.02969846 | 68.7 | 1.41215789 | 68.7 | 1.63771088 | | | 68.70 | 1.4709978 | 68.7 | 2.42224304 | 68.7 | 1.4709978 |
| 68.8 | 0.57859247 | 68.8 | 1.03950511 | 68.8 | 1.03950511 | 68.8 | 1.4513845 | 68.8 | 1.66713084 | | | 68.80 | 1.46119115 | 68.8 | 2.44185635 | 68.8 | 1.46119115 |
| 68.9 | 0.58839912 | 68.9 | 1.04931176 | 68.9 | 1.04931176 | 68.9 | 1.46119115 | 68.9 | 1.68674414 | | | 68.90 | 1.42196454 | 68.9 | 2.46146965 | 68.9 | 1.42196454 |
| 69 | 0.61781908 | 69 | 1.03950511 | 69 | 1.03950511 | 69 | 1.44157784 | 69 | 1.68674414 | | | 69.00 | 1.40235124 | 69 | 2.46146965 | 69 | 1.40235124 |
| 69.1 | 0.62762573 | 69.1 | 1.06892507 | 69.1 | 1.06892507 | 69.1 | 1.41215789 | 69.1 | 1.65732419 | | | 69.10 | 1.39254458 | 69.1 | 2.46146965 | 69.1 | 1.39254458 |
| 69.2 | 0.59820577 | 69.2 | 1.07873172 | 69.2 | 1.07873172 | 69.2 | 1.44157784 | 69.2 | 1.66713084 | | | 69.20 | 1.43177119 | 69.2 | 2.4712763 | 69.2 | 1.43177119 |
| 69.3 | 0.58839912 | 69.3 | 1.05911842 | 69.3 | 1.05911842 | 69.3 | 1.43177119 | 69.3 | 1.70635745 | | | 69.30 | 1.46119115 | 69.3 | 2.44185635 | 69.3 | 1.46119115 |
| 69.4 | 0.59820577 | 69.4 | 1.04931176 | 69.4 | 1.04931176 | 69.4 | 1.4513845 | 69.4 | 1.70635745 | | | 69.40 | 1.4513845 | 69.4 | 2.4712763 | 69.4 | 1.4513845 |
| 69.5 | 0.60801242 | 69.5 | 1.06892507 | 69.5 | 1.06892507 | 69.5 | 1.41215789 | 69.5 | 1.7161641 | | | 69.50 | 1.4709978 | 69.5 | 2.4712763 | 69.5 | 1.4709978 |
| 69.6 | 0.55897916 | 69.6 | 1.02969846 | 69.6 | 1.02969846 | 69.6 | 1.41215789 | 69.6 | 1.72597075 | | | 69.60 | 1.4513845 | 69.6 | 2.49088961 | 69.6 | 1.4513845 |
| 69.7 | 0.56878582 | 69.7 | 1.04931176 | 69.7 | 1.04931176 | 69.7 | 1.43177119 | 69.7 | 1.70635745 | | | 69.70 | 1.4906111 | 69.7 | 2.48108296 | 69.7 | 1.4906111 |
| 69.8 | 0.57859247 | 69.8 | 1.04931176 | 69.8 | 1.04931176 | 69.8 | 1.4709978 | 69.8 | 1.75539071 | | | 69.80 | 1.48080445 | 69.8 | 2.49088961 | 69.8 | 1.48080445 |
| 69.9 | 0.60801242 | 69.9 | 1.07873172 | 69.9 | 1.07873172 | 69.9 | 1.46119115 | 69.9 | 1.7357774 | | | 69.90 | 1.4513845 | 69.9 | 2.50069626 | 69.9 | 1.4513845 |
| 70 | 0.63743238 | 70 | 1.09834502 | 70 | 1.09834502 | 70 | 1.46119115 | 70 | 1.70635745 | | | 70.00 | 1.4513845 | 70 | 2.48108296 | 70 | 1.4513845 |
| 70.1 | 0.62762573 | 70.1 | 1.08853837 | 70.1 | 1.08853837 | 70.1 | 1.44157784 | 70.1 | 1.7161641 | | | 70.10 | 1.4709978 | 70.1 | 2.4712763 | 70.1 | 1.4709978 |
| 70.2 | 0.60801242 | 70.2 | 1.07873172 | 70.2 | 1.07873172 | 70.2 | 1.4709978 | 70.2 | 1.7161641 | | | 70.20 | 1.50041776 | 70.2 | 2.48108296 | 70.2 | 1.50041776 |
| 70.3 | 0.56878582 | 70.3 | 1.07873172 | 70.3 | 1.07873172 | 70.3 | 1.4513845 | 70.3 | 1.72597075 | | | 70.30 | 1.46119115 | 70.3 | 2.51050291 | 70.3 | 1.46119115 |
| 70.4 | 0.62762573 | 70.4 | 1.06892507 | 70.4 | 1.06892507 | 70.4 | 1.4513845 | 70.4 | 1.75539071 | | | 70.40 | 1.46119115 | 70.4 | 2.51050291 | 70.4 | 1.46119115 |

| | | | | | | | | | | | | | | | | | |
|---|---|---|---|---|---|---|---|---|---|---|---|---|---|---|---|---|---|
| 70.5 | 0.62762573 | 70.5 | 1.07873172 | 70.5 | 1.07873172 | 70.5 | 1.4513845 | 70.5 | 1.74558406 | | | 70.50 | 1.4709978 | 70.5 | 2.49088961 | 70.5 | 1.4709978 |
| 70.6 | 0.60801242 | 70.6 | 1.04931176 | 70.6 | 1.04931176 | 70.6 | 1.4513845 | 70.6 | 1.81423062 | | | 70.60 | 1.4513845 | 70.6 | 2.48108296 | 70.6 | 1.4513845 |
| 70.7 | 0.60801242 | 70.7 | 1.07873172 | 70.7 | 1.07873172 | 70.7 | 1.44157784 | 70.7 | 1.79461732 | | | 70.70 | 1.50041776 | 70.7 | 2.51050291 | 70.7 | 1.50041776 |
| 70.8 | 0.62762573 | 70.8 | 1.05911842 | 70.8 | 1.05911842 | 70.8 | 1.4709978 | 70.8 | 1.77500401 | | | 70.80 | 1.50041776 | 70.8 | 2.51050291 | 70.8 | 1.50041776 |
| 70.9 | 0.62762573 | 70.9 | 1.04931176 | 70.9 | 1.04931176 | 70.9 | 1.4513845 | 70.9 | 1.75539071 | | | 70.90 | 1.51022441 | 70.9 | 2.52030956 | 70.9 | 1.51022441 |
| 71 | 0.62762573 | 71 | 1.07873172 | 71 | 1.07873172 | 71 | 1.4709978 | 71 | 1.78481066 | | | 71.00 | 1.52003106 | 71 | 2.52030956 | 71 | 1.52003106 |
| 71.1 | 0.60801242 | 71.1 | 1.09834502 | 71.1 | 1.09834502 | 71.1 | 1.4513845 | 71.1 | 1.82403727 | | | 71.10 | 1.48080445 | 71.1 | 2.53011622 | 71.1 | 1.48080445 |
| 71.2 | 0.61781908 | 71.2 | 1.13757163 | 71.2 | 1.13757163 | 71.2 | 1.42196454 | 71.2 | 1.83384392 | | | 71.20 | 1.51022441 | 71.2 | 2.53011622 | 71.2 | 1.51022441 |
| 71.3 | 0.60801242 | 71.3 | 1.11795833 | 71.3 | 1.11795833 | 71.3 | 1.44157784 | 71.3 | 1.81423062 | | | 71.30 | 1.53964436 | 71.3 | 2.54972952 | 71.3 | 1.53964436 |
| 71.4 | 0.59820577 | 71.4 | 1.10815168 | 71.4 | 1.10815168 | 71.4 | 1.46119115 | 71.4 | 1.79461732 | | | 71.40 | 1.52983771 | 71.4 | 2.54972952 | 71.4 | 1.52983771 |
| 71.5 | 0.60801242 | 71.5 | 1.08853837 | 71.5 | 1.08853837 | 71.5 | 1.4709978 | 71.5 | 1.79461732 | | | 71.50 | 1.50041776 | 71.5 | 2.53992287 | 71.5 | 1.50041776 |
| 71.6 | 0.62762573 | 71.6 | 1.10815168 | 71.6 | 1.10815168 | 71.6 | 1.4513845 | 71.6 | 1.81423062 | | | 71.60 | 1.50041776 | 71.6 | 2.52030956 | 71.6 | 1.50041776 |
| 71.7 | 0.65704568 | 71.7 | 1.11795833 | 71.7 | 1.11795833 | 71.7 | 1.4906111 | 71.7 | 1.82403727 | | | 71.70 | 1.50041776 | 71.7 | 2.55953617 | 71.7 | 1.50041776 |
| 71.8 | 0.62762573 | 71.8 | 1.09834502 | 71.8 | 1.09834502 | 71.8 | 1.50041776 | 71.8 | 1.88287718 | | | 71.80 | 1.50041776 | 71.8 | 2.54972952 | 71.8 | 1.50041776 |
| 71.9 | 0.63743238 | 71.9 | 1.09834502 | 71.9 | 1.09834502 | 71.9 | 1.4906111 | 71.9 | 1.85345723 | | | 71.90 | 1.50041776 | 71.9 | 2.53992287 | 71.9 | 1.50041776 |
| 72 | 0.62762573 | 72 | 1.09834502 | 72 | 1.09834502 | 72 | 1.52983771 | 72 | 1.84365058 | | | 72.00 | 1.52003106 | 72 | 2.53011622 | 72 | 1.52003106 |
| 72.1 | 0.61781908 | 72.1 | 1.08853837 | 72.1 | 1.08853837 | 72.1 | 1.51022441 | 72.1 | 1.86326388 | | | 72.10 | 1.54945102 | 72.1 | 2.52030956 | 72.1 | 1.54945102 |
| 72.2 | 0.63743238 | 72.2 | 1.10815168 | 72.2 | 1.10815168 | 72.2 | 1.48080445 | 72.2 | 1.85345723 | | | 72.20 | 1.54945102 | 72.2 | 2.54972952 | 72.2 | 1.54945102 |
| 72.3 | 0.63743238 | 72.3 | 1.17679824 | 72.3 | 1.17679824 | 72.3 | 1.4709978 | 72.3 | 1.86326388 | | | 72.30 | 1.52983771 | 72.3 | 2.55953617 | 72.3 | 1.52983771 |
| 72.4 | 0.61781908 | 72.4 | 1.13757163 | 72.4 | 1.13757163 | 72.4 | 1.4906111 | 72.4 | 1.89268384 | | | 72.40 | 1.52003106 | 72.4 | 2.56934282 | 72.4 | 1.52003106 |
| 72.5 | 0.62762573 | 72.5 | 1.11795833 | 72.5 | 1.11795833 | 72.5 | 1.4906111 | 72.5 | 1.89268384 | | | 72.50 | 1.52983771 | 72.5 | 2.56934282 | 72.5 | 1.52983771 |
| 72.6 | 0.59820577 | 72.6 | 1.12776498 | 72.6 | 1.12776498 | 72.6 | 1.48080445 | 72.6 | 1.86326388 | | | 72.60 | 1.53964436 | 72.6 | 2.55953617 | 72.6 | 1.53964436 |
| 72.7 | 0.60801242 | 72.7 | 1.13757163 | 72.7 | 1.13757163 | 72.7 | 1.50041776 | 72.7 | 1.85345723 | | | 72.70 | 1.53964436 | 72.7 | 2.56934282 | 72.7 | 1.53964436 |
| 72.8 | 0.59820577 | 72.8 | 1.14737828 | 72.8 | 1.14737828 | 72.8 | 1.4906111 | 72.8 | 1.87307053 | | | 72.80 | 1.52003106 | 72.8 | 2.57914948 | 72.8 | 1.52003106 |
| 72.9 | 0.62762573 | 72.9 | 1.21602485 | 72.9 | 1.21602485 | 72.9 | 1.52983771 | 72.9 | 1.91229714 | | | 72.90 | 1.52983771 | 72.9 | 2.58895613 | 72.9 | 1.52983771 |
| 73 | 0.61781908 | 73 | 1.18660489 | 73 | 1.18660489 | 73 | 1.53964436 | 73 | 1.87307053 | | | 73.00 | 1.54945102 | 73 | 2.58895613 | 73 | 1.54945102 |
| 73.1 | 0.61781908 | 73.1 | 1.14737828 | 73.1 | 1.14737828 | 73.1 | 1.52983771 | 73.1 | 1.89268384 | | | 73.10 | 1.55925767 | 73.1 | 2.60856943 | 73.1 | 1.55925767 |
| 73.2 | 0.62762573 | 73.2 | 1.17679824 | 73.2 | 1.17679824 | 73.2 | 1.52983771 | 73.2 | 1.92210379 | | | 73.20 | 1.56906432 | 73.2 | 2.58895613 | 73.2 | 1.56906432 |
| 73.3 | 0.62762573 | 73.3 | 1.17679824 | 73.3 | 1.17679824 | 73.3 | 1.52983771 | 73.3 | 1.90249049 | | | 73.30 | 1.55925767 | 73.3 | 2.56934282 | 73.3 | 1.55925767 |
| 73.4 | 0.62762573 | 73.4 | 1.17679824 | 73.4 | 1.17679824 | 73.4 | 1.52983771 | 73.4 | 1.89268384 | | | 73.40 | 1.54945102 | 73.4 | 2.60856943 | 73.4 | 1.54945102 |
| 73.5 | 0.60801242 | 73.5 | 1.13757163 | 73.5 | 1.13757163 | 73.5 | 1.52003106 | 73.5 | 1.90249049 | | | 73.50 | 1.57887097 | 73.5 | 2.60856943 | 73.5 | 1.57887097 |
| 73.6 | 0.60801242 | 73.6 | 1.18660489 | 73.6 | 1.18660489 | 73.6 | 1.52003106 | 73.6 | 1.92210379 | | | 73.60 | 1.56906432 | 73.6 | 2.58895613 | 73.6 | 1.56906432 |
| 73.7 | 0.60801242 | 73.7 | 1.17679824 | 73.7 | 1.17679824 | 73.7 | 1.52983771 | 73.7 | 1.9417171 | | | 73.70 | 1.55925767 | 73.7 | 2.59876278 | 73.7 | 1.55925767 |
| 73.8 | 0.62762573 | 73.8 | 1.16699159 | 73.8 | 1.16699159 | 73.8 | 1.52983771 | 73.8 | 1.97113705 | | | 73.80 | 1.55925767 | 73.8 | 2.59876278 | 73.8 | 1.55925767 |
| 73.9 | 0.62762573 | 73.9 | 1.16699159 | 73.9 | 1.16699159 | 73.9 | 1.52003106 | 73.9 | 1.9417171 | | | 73.90 | 1.57887097 | 73.9 | 2.61837608 | 73.9 | 1.57887097 |
| 74 | 0.61781908 | 74 | 1.15718494 | 74 | 1.15718494 | 74 | 1.52983771 | 74 | 1.92210379 | | | 74.00 | 1.58867762 | 74 | 2.61837608 | 74 | 1.58867762 |
| 74.1 | 0.63743238 | 74.1 | 1.16699159 | 74.1 | 1.16699159 | 74.1 | 1.55925767 | 74.1 | 1.93191044 | | | 74.10 | 1.57887097 | 74.1 | 2.62818274 | 74.1 | 1.57887097 |
| 74.2 | 0.61781908 | 74.2 | 1.16699159 | 74.2 | 1.16699159 | 74.2 | 1.55925767 | 74.2 | 1.9809437 | | | 74.20 | 1.58867762 | 74.2 | 2.62818274 | 74.2 | 1.58867762 |
| 74.3 | 0.60801242 | 74.3 | 1.17679824 | 74.3 | 1.17679824 | 74.3 | 1.53964436 | 74.3 | 2.00055701 | | | 74.30 | 1.57887097 | 74.3 | 2.62818274 | 74.3 | 1.57887097 |
| 74.4 | 0.62762573 | 74.4 | 1.21602485 | 74.4 | 1.21602485 | 74.4 | 1.48080445 | 74.4 | 1.9809437 | | | 74.40 | 1.56906432 | 74.4 | 2.63798939 | 74.4 | 1.56906432 |
| 74.5 | 0.62762573 | 74.5 | 1.16699159 | 74.5 | 1.16699159 | 74.5 | 1.52003106 | 74.5 | 1.9613304 | | | 74.50 | 1.56906432 | 74.5 | 2.63798939 | 74.5 | 1.56906432 |
| 74.6 | 0.62762573 | 74.6 | 1.16699159 | 74.6 | 1.16699159 | 74.6 | 1.52003106 | 74.6 | 1.97113705 | | | 74.60 | 1.53964436 | 74.6 | 2.65760269 | 74.6 | 1.53964436 |
| 74.7 | 0.61781908 | 74.7 | 1.21602485 | 74.7 | 1.21602485 | 74.7 | 1.53964436 | 74.7 | 2.00055701 | | | 74.70 | 1.59848428 | 74.7 | 2.65760269 | 74.7 | 1.59848428 |
| 74.8 | 0.58839912 | 74.8 | 1.2258315 | 74.8 | 1.2258315 | 74.8 | 1.51022441 | 74.8 | 1.99075036 | | | 74.80 | 1.59848428 | 74.8 | 2.63798939 | 74.8 | 1.59848428 |
| 74.9 | 0.59820577 | 74.9 | 1.21602485 | 74.9 | 1.21602485 | 74.9 | 1.51022441 | 74.9 | 1.99075036 | | | 74.90 | 1.57887097 | 74.9 | 2.63798939 | 74.9 | 1.57887097 |
| 75 | 0.61781908 | 75 | 1.18660489 | 75 | 1.18660489 | 75 | 1.51022441 | 75 | 2.01036366 | | | 75.00 | 1.59848428 | 75 | 2.64779604 | 75 | 1.59848428 |
| 75.1 | 0.66685234 | 75.1 | 1.2062182 | 75.1 | 1.2062182 | 75.1 | 1.4709978 | 75.1 | 2.00055701 | | | 75.10 | 1.60829093 | 75.1 | 2.66740934 | 75.1 | 1.60829093 |
| 75.2 | 0.58839912 | 75.2 | 1.2062182 | 75.2 | 1.2062182 | 75.2 | 1.48080445 | 75.2 | 2.01036366 | | | 75.20 | 1.59848428 | 75.2 | 2.6968293 | 75.2 | 1.59848428 |
| 75.3 | 0.61781908 | 75.3 | 1.2062182 | 75.3 | 1.2062182 | 75.3 | 1.52983771 | 75.3 | 2.01036366 | | | 75.30 | 1.58867762 | 75.3 | 2.65760269 | 75.3 | 1.58867762 |
| 75.4 | 0.62762573 | 75.4 | 1.18660489 | 75.4 | 1.18660489 | 75.4 | 1.54945102 | 75.4 | 2.03978362 | | | 75.40 | 1.59848428 | 75.4 | 2.66740934 | 75.4 | 1.59848428 |
| 75.5 | 0.63743238 | 75.5 | 1.19641154 | 75.5 | 1.19641154 | 75.5 | 1.54945102 | 75.5 | 2.02997696 | | | 75.50 | 1.58867762 | 75.5 | 2.677216 | 75.5 | 1.58867762 |
| 75.6 | 0.64723903 | 75.6 | 1.21602485 | 75.6 | 1.21602485 | 75.6 | 1.53964436 | 75.6 | 2.02997696 | | | 75.60 | 1.60829093 | 75.6 | 2.70663595 | 75.6 | 1.60829093 |
| 75.7 | 0.63743238 | 75.7 | 1.2258315 | 75.7 | 1.2258315 | 75.7 | 1.54945102 | 75.7 | 2.05939692 | | | 75.70 | 1.61809758 | 75.7 | 2.6968293 | 75.7 | 1.61809758 |
| 75.8 | 0.62762573 | 75.8 | 1.23563815 | 75.8 | 1.23563815 | 75.8 | 1.55925767 | 75.8 | 2.07901022 | | | 75.80 | 1.61809758 | 75.8 | 2.68702265 | 75.8 | 1.61809758 |
| 75.9 | 0.61781908 | 75.9 | 1.2258315 | 75.9 | 1.2258315 | 75.9 | 1.52003106 | 75.9 | 2.07901022 | | | 75.90 | 1.62790423 | 75.9 | 2.70663595 | 75.9 | 1.62790423 |
| 76 | 0.63743238 | 76 | 1.21602485 | 76 | 1.21602485 | 76 | 1.52003106 | 76 | 2.06920357 | | | 76.00 | 1.64751754 | 76 | 2.70663595 | 76 | 1.64751754 |
| 76.1 | 0.63743238 | 76.1 | 1.21602485 | 76.1 | 1.21602485 | 76.1 | 1.52983771 | 76.1 | 2.06920357 | | | 76.10 | 1.63771088 | 76.1 | 2.6968293 | 76.1 | 1.63771088 |
| 76.2 | 0.63743238 | 76.2 | 1.21602485 | 76.2 | 1.21602485 | 76.2 | 1.52983771 | 76.2 | 2.05939692 | | | 76.20 | 1.60829093 | 76.2 | 2.6968293 | 76.2 | 1.60829093 |
| 76.3 | 0.65704568 | 76.3 | 1.21602485 | 76.3 | 1.21602485 | 76.3 | 1.53964436 | 76.3 | 2.03978362 | | | 76.30 | 1.59848428 | 76.3 | 2.70663595 | 76.3 | 1.59848428 |
| 76.4 | 0.63743238 | 76.4 | 1.2258315 | 76.4 | 1.2258315 | 76.4 | 1.56906432 | 76.4 | 2.05939692 | | | 76.40 | 1.60829093 | 76.4 | 2.7164426 | 76.4 | 1.60829093 |
| 76.5 | 0.63743238 | 76.5 | 1.25525146 | 76.5 | 1.25525146 | 76.5 | 1.56906432 | 76.5 | 2.10843018 | | | 76.50 | 1.63771088 | 76.5 | 2.7164426 | 76.5 | 1.63771088 |

| | | | | | | | | | | | | | | | | | |
|---|---|---|---|---|---|---|---|---|---|---|---|---|---|---|---|---|---|
| 76.6 | 0.63743238 | 76.6 | 1.2454448 | 76.6 | 1.2454448 | 76.6 | 1.53964436 | 76.6 | 2.09862353 | | | 76.60 | 1.62790423 | 76.6 | 2.72624926 | 76.6 | 1.62790423 |
| 76.7 | 0.61781908 | 76.7 | 1.2062182 | 76.7 | 1.2062182 | 76.7 | 1.55925767 | 76.7 | 2.07901022 | | | 76.70 | 1.60829093 | 76.7 | 2.73605591 | 76.7 | 1.60829093 |
| 76.8 | 0.61781908 | 76.8 | 1.18660489 | 76.8 | 1.18660489 | 76.8 | 1.60829093 | 76.8 | 2.10843018 | | | 76.80 | 1.60829093 | 76.8 | 2.74586256 | 76.8 | 1.60829093 |
| 76.9 | 0.62762573 | 76.9 | 1.19641154 | 76.9 | 1.19641154 | 76.9 | 1.60829093 | 76.9 | 2.13785014 | | | 76.90 | 1.62790423 | 76.9 | 2.73605591 | 76.9 | 1.62790423 |
| 77 | 0.66685234 | 77 | 1.2454448 | 77 | 1.2454448 | 77 | 1.56906432 | 77 | 2.14765679 | | | 77.00 | 1.65732419 | 77 | 2.75566921 | 77 | 1.65732419 |
| 77.1 | 0.65704568 | 77.1 | 1.2258315 | 77.1 | 1.2258315 | 77.1 | 1.58867762 | 77.1 | 2.12804348 | | | 77.10 | 1.65732419 | 77.1 | 2.74586256 | 77.1 | 1.65732419 |
| 77.2 | 0.62762573 | 77.2 | 1.2454448 | 77.2 | 1.2454448 | 77.2 | 1.56906432 | 77.2 | 2.10843018 | | | 77.20 | 1.62790423 | 77.2 | 2.74586256 | 77.2 | 1.62790423 |
| 77.3 | 0.62762573 | 77.3 | 1.23563815 | 77.3 | 1.23563815 | 77.3 | 1.54945102 | 77.3 | 2.15746344 | | | 77.30 | 1.63771088 | 77.3 | 2.73605591 | 77.3 | 1.63771088 |
| 77.4 | 0.64723903 | 77.4 | 1.2258315 | 77.4 | 1.2258315 | 77.4 | 1.58867762 | 77.4 | 2.17707674 | | | 77.40 | 1.64751754 | 77.4 | 2.74586256 | 77.4 | 1.64751754 |
| 77.5 | 0.64723903 | 77.5 | 1.23563815 | 77.5 | 1.23563815 | 77.5 | 1.60829093 | 77.5 | 2.1868834 | | | 77.50 | 1.64751754 | 77.5 | 2.75566921 | 77.5 | 1.64751754 |
| 77.6 | 0.66685234 | 77.6 | 1.25525146 | 77.6 | 1.25525146 | 77.6 | 1.58867762 | 77.6 | 2.16727009 | | | 77.60 | 1.67693749 | 77.6 | 2.77528252 | 77.6 | 1.67693749 |
| 77.7 | 0.65704568 | 77.7 | 1.23563815 | 77.7 | 1.23563815 | 77.7 | 1.58867762 | 77.7 | 2.1868834 | | | 77.70 | 1.66713084 | 77.7 | 2.79489582 | 77.7 | 1.66713084 |
| 77.8 | 0.61781908 | 77.8 | 1.2258315 | 77.8 | 1.2258315 | 77.8 | 1.61809758 | 77.8 | 2.17707674 | | | 77.80 | 1.66713084 | 77.8 | 2.79489582 | 77.8 | 1.66713084 |
| 77.9 | 0.63743238 | 77.9 | 1.2454448 | 77.9 | 1.2454448 | 77.9 | 1.63771088 | 77.9 | 2.19669005 | | | 77.90 | 1.67693749 | 77.9 | 2.79489582 | 77.9 | 1.67693749 |
| 78 | 0.64723903 | 78 | 1.28467141 | 78 | 1.28467141 | 78 | 1.62790423 | 78 | 2.19669005 | | | 78.00 | 1.68674414 | 78 | 2.77528252 | 78 | 1.68674414 |
| 78.1 | 0.61781908 | 78.1 | 1.25525146 | 78.1 | 1.25525146 | 78.1 | 1.63771088 | 78.1 | 2.19669005 | | | 78.10 | 1.65732419 | 78.1 | 2.77528252 | 78.1 | 1.65732419 |
| 78.2 | 0.65704568 | 78.2 | 1.25525146 | 78.2 | 1.25525146 | 78.2 | 1.59848428 | 78.2 | 2.22611 | | | 78.20 | 1.65732419 | 78.2 | 2.80470247 | 78.2 | 1.65732419 |
| 78.3 | 0.64723903 | 78.3 | 1.27486476 | 78.3 | 1.27486476 | 78.3 | 1.59848428 | 78.3 | 2.24572331 | | | 78.30 | 1.64751754 | 78.3 | 2.82431578 | 78.3 | 1.64751754 |
| 78.4 | 0.61781908 | 78.4 | 1.2454448 | 78.4 | 1.2454448 | 78.4 | 1.61809758 | 78.4 | 2.23591666 | | | 78.40 | 1.66713084 | 78.4 | 2.84392908 | 78.4 | 1.66713084 |
| 78.5 | 0.64723903 | 78.5 | 1.2454448 | 78.5 | 1.2454448 | 78.5 | 1.60829093 | 78.5 | 2.22611 | | | 78.50 | 1.66713084 | 78.5 | 2.82431578 | 78.5 | 1.66713084 |
| 78.6 | 0.63743238 | 78.6 | 1.28467141 | 78.6 | 1.28467141 | 78.6 | 1.58867762 | 78.6 | 2.22611 | | | 78.60 | 1.65732419 | 78.6 | 2.81450912 | 78.6 | 1.65732419 |
| 78.7 | 0.64723903 | 78.7 | 1.30428472 | 78.7 | 1.30428472 | 78.7 | 1.61809758 | 78.7 | 2.26533661 | | | 78.70 | 1.66713084 | 78.7 | 2.81450912 | 78.7 | 1.66713084 |
| 78.8 | 0.65704568 | 78.8 | 1.29447806 | 78.8 | 1.29447806 | 78.8 | 1.61809758 | 78.8 | 2.25552996 | | | 78.80 | 1.6965508 | 78.8 | 2.82431578 | 78.8 | 1.6965508 |
| 78.9 | 0.63743238 | 78.9 | 1.31409137 | 78.9 | 1.31409137 | 78.9 | 1.58867762 | 78.9 | 2.25552996 | | | 78.90 | 1.67693749 | 78.9 | 2.82431578 | 78.9 | 1.67693749 |
| 79 | 0.63743238 | 79 | 1.31409137 | 79 | 1.31409137 | 79 | 1.63771088 | 79 | 2.25552996 | | | 79.00 | 1.68674414 | 79 | 2.83412243 | 79 | 1.68674414 |
| 79.1 | 0.63743238 | 79.1 | 1.28467141 | 79.1 | 1.28467141 | 79.1 | 1.60829093 | 79.1 | 2.27514326 | | | 79.10 | 1.7161641 | 79.1 | 2.85373573 | 79.1 | 1.7161641 |
| 79.2 | 0.65704568 | 79.2 | 1.28467141 | 79.2 | 1.28467141 | 79.2 | 1.58867762 | 79.2 | 2.28494992 | | | 79.20 | 1.65732419 | 79.2 | 2.85373573 | 79.2 | 1.65732419 |
| 79.3 | 0.60801242 | 79.3 | 1.29447806 | 79.3 | 1.29447806 | 79.3 | 1.60829093 | 79.3 | 2.29475657 | | | 79.30 | 1.67693749 | 79.3 | 2.85373573 | 79.3 | 1.67693749 |
| 79.4 | 0.59820577 | 79.4 | 1.28467141 | 79.4 | 1.28467141 | 79.4 | 1.61809758 | 79.4 | 2.30456322 | | | 79.40 | 1.68674414 | 79.4 | 2.86354238 | 79.4 | 1.68674414 |
| 79.5 | 0.60801242 | 79.5 | 1.27486476 | 79.5 | 1.27486476 | 79.5 | 1.63771088 | 79.5 | 2.31436987 | | | 79.50 | 1.6965508 | 79.5 | 2.87334904 | 79.5 | 1.6965508 |
| 79.6 | 0.62762573 | 79.6 | 1.29447806 | 79.6 | 1.29447806 | 79.6 | 1.65732419 | 79.6 | 2.28494992 | | | 79.60 | 1.6965508 | 79.6 | 2.86354238 | 79.6 | 1.6965508 |
| 79.7 | 0.62762573 | 79.7 | 1.29447806 | 79.7 | 1.29447806 | 79.7 | 1.66713084 | 79.7 | 2.30456322 | | | 79.70 | 1.67693749 | 79.7 | 2.86354238 | 79.7 | 1.67693749 |
| 79.8 | 0.62762573 | 79.8 | 1.28467141 | 79.8 | 1.28467141 | 79.8 | 1.63771088 | 79.8 | 2.30456322 | | | 79.80 | 1.66713084 | 79.8 | 2.87334904 | 79.8 | 1.66713084 |
| 79.9 | 0.64723903 | 79.9 | 1.28467141 | 79.9 | 1.28467141 | 79.9 | 1.64751754 | 79.9 | 2.30456322 | | | 79.90 | 1.66713084 | 79.9 | 2.88315569 | 79.9 | 1.66713084 |
| 80 | 0.64723903 | 80 | 1.29447806 | 80 | 1.29447806 | 80 | 1.65732419 | 80 | 2.30456322 | | | 80.00 | 1.67693749 | 80 | 2.88315569 | 80 | 1.67693749 |
| 80.1 | 0.65704568 | 80.1 | 1.32389802 | 80.1 | 1.32389802 | 80.1 | 1.65732419 | 80.1 | 2.28494992 | | | 80.10 | 1.68674414 | 80.1 | 2.88315569 | 80.1 | 1.68674414 |
| 80.2 | 0.68646564 | 80.2 | 1.35331798 | 80.2 | 1.35331798 | 80.2 | 1.64751754 | 80.2 | 2.30456322 | | | 80.20 | 1.70635745 | 80.2 | 2.88315569 | 80.2 | 1.70635745 |
| 80.3 | 0.67665899 | 80.3 | 1.34351132 | 80.3 | 1.34351132 | 80.3 | 1.65732419 | 80.3 | 2.30456322 | | | 80.30 | 1.70635745 | 80.3 | 2.89296234 | 80.3 | 1.70635745 |
| 80.4 | 0.64723903 | 80.4 | 1.32389802 | 80.4 | 1.32389802 | 80.4 | 1.67693749 | 80.4 | 2.31436987 | | | 80.40 | 1.7161641 | 80.4 | 2.89296234 | 80.4 | 1.7161641 |
| 80.5 | 0.63743238 | 80.5 | 1.31409137 | 80.5 | 1.31409137 | 80.5 | 1.66713084 | 80.5 | 2.33398318 | | | 80.50 | 1.6965508 | 80.5 | 2.91257564 | 80.5 | 1.6965508 |
| 80.6 | 0.66685234 | 80.6 | 1.34351132 | 80.6 | 1.34351132 | 80.6 | 1.63771088 | 80.6 | 2.34378983 | | | 80.60 | 1.70635745 | 80.6 | 2.93218895 | 80.6 | 1.70635745 |
| 80.7 | 0.67665899 | 80.7 | 1.35331798 | 80.7 | 1.35331798 | 80.7 | 1.65732419 | 80.7 | 2.33398318 | | | 80.70 | 1.6965508 | 80.7 | 2.9419956 | 80.7 | 1.6965508 |
| 80.8 | 0.68646564 | 80.8 | 1.36312463 | 80.8 | 1.36312463 | 80.8 | 1.66713084 | 80.8 | 2.34378983 | | | 80.80 | 1.7161641 | 80.8 | 2.9419956 | 80.8 | 1.7161641 |
| 80.9 | 0.67665899 | 80.9 | 1.37293128 | 80.9 | 1.37293128 | 80.9 | 1.62790423 | 80.9 | 2.38301644 | | | 80.90 | 1.7357774 | 80.9 | 2.93218895 | 80.9 | 1.7357774 |
| 81 | 0.64723903 | 81 | 1.36312463 | 81 | 1.36312463 | 81 | 1.62790423 | 81 | 2.38301644 | | | 81.00 | 1.7357774 | 81 | 2.91257564 | 81 | 1.7357774 |
| 81.1 | 0.65704568 | 81.1 | 1.39254458 | 81.1 | 1.39254458 | 81.1 | 1.60829093 | 81.1 | 2.38301644 | | | 81.10 | 1.7357774 | 81.1 | 2.91257564 | 81.1 | 1.7357774 |
| 81.2 | 0.65704568 | 81.2 | 1.40235124 | 81.2 | 1.40235124 | 81.2 | 1.61809758 | 81.2 | 2.37320978 | | | 81.20 | 1.72597075 | 81.2 | 2.93218895 | 81.2 | 1.72597075 |
| 81.3 | 0.65704568 | 81.3 | 1.36312463 | 81.3 | 1.36312463 | 81.3 | 1.64751754 | 81.3 | 2.38301644 | | | 81.30 | 1.72597075 | 81.3 | 2.95180225 | 81.3 | 1.72597075 |
| 81.4 | 0.64723903 | 81.4 | 1.36312463 | 81.4 | 1.36312463 | 81.4 | 1.66713084 | 81.4 | 2.40262974 | | | 81.40 | 1.7357774 | 81.4 | 2.9616089 | 81.4 | 1.7357774 |
| 81.5 | 0.65704568 | 81.5 | 1.36312463 | 81.5 | 1.36312463 | 81.5 | 1.65732419 | 81.5 | 2.39282309 | | | 81.50 | 1.72597075 | 81.5 | 2.9616089 | 81.5 | 1.72597075 |
| 81.6 | 0.64723903 | 81.6 | 1.36312463 | 81.6 | 1.36312463 | 81.6 | 1.62790423 | 81.6 | 2.40262974 | | | 81.60 | 1.7357774 | 81.6 | 2.98122221 | 81.6 | 1.7357774 |
| 81.7 | 0.64723903 | 81.7 | 1.37293128 | 81.7 | 1.37293128 | 81.7 | 1.65732419 | 81.7 | 2.41243639 | | | 81.70 | 1.7357774 | 81.7 | 2.98122221 | 81.7 | 1.7357774 |
| 81.8 | 0.64723903 | 81.8 | 1.39254458 | 81.8 | 1.39254458 | 81.8 | 1.68674414 | 81.8 | 2.41243639 | | | 81.80 | 1.72597075 | 81.8 | 2.9616089 | 81.8 | 1.72597075 |
| 81.9 | 0.66685234 | 81.9 | 1.38273793 | 81.9 | 1.38273793 | 81.9 | 1.67693749 | 81.9 | 2.42224304 | | | 81.90 | 1.7161641 | 81.9 | 2.97141556 | 81.9 | 1.7161641 |
| 82 | 0.65704568 | 82 | 1.37293128 | 82 | 1.37293128 | 82 | 1.6965508 | 82 | 2.4320497 | | | 82.00 | 1.6965508 | 82 | 2.98122221 | 82 | 1.6965508 |
| 82.1 | 0.64723903 | 82.1 | 1.37293128 | 82.1 | 1.37293128 | 82.1 | 1.7161641 | 82.1 | 2.4320497 | | | 82.10 | 1.70635745 | 82.1 | 2.98122221 | 82.1 | 1.70635745 |
| 82.2 | 0.64723903 | 82.2 | 1.37293128 | 82.2 | 1.37293128 | 82.2 | 1.70635745 | 82.2 | 2.44185635 | | | 82.20 | 1.7357774 | 82.2 | 2.99102886 | 82.2 | 1.7357774 |
| 82.3 | 0.64723903 | 82.3 | 1.40235124 | 82.3 | 1.40235124 | 82.3 | 1.70635745 | 82.3 | 2.451663 | | | 82.30 | 1.7357774 | 82.3 | 2.98122221 | 82.3 | 1.7357774 |
| 82.4 | 0.64723903 | 82.4 | 1.40235124 | 82.4 | 1.40235124 | 82.4 | 1.70635745 | 82.4 | 2.451663 | | | 82.40 | 1.7357774 | 82.4 | 2.99102886 | 82.4 | 1.7357774 |
| 82.5 | 0.66685234 | 82.5 | 1.42196454 | 82.5 | 1.42196454 | 82.5 | 1.6965508 | 82.5 | 2.451663 | | | 82.50 | 1.77500401 | 82.5 | 3.01064216 | 82.5 | 1.77500401 |
| 82.6 | 0.67665899 | 82.6 | 1.43177119 | 82.6 | 1.43177119 | 82.6 | 1.7161641 | 82.6 | 2.46146965 | | | 82.60 | 1.75539071 | 82.6 | 3.00083551 | 82.6 | 1.75539071 |

| | | | | | | | | | | | | | | | | | |
|---|---|---|---|---|---|---|---|---|---|---|---|---|---|---|---|---|---|
| 82.7 | 0.65704568 | 82.7 | 1.42196454 | 82.7 | 1.42196454 | 82.7 | 1.7161641 | 82.7 | 2.4712763 | | | 82.70 | 1.74558406 | 82.7 | 3.00083551 | 82.7 | 1.74558406 |
| 82.8 | 0.64723903 | 82.8 | 1.41215789 | 82.8 | 1.41215789 | 82.8 | 1.72597075 | 82.8 | 2.46146965 | | | 82.80 | 1.74558406 | 82.8 | 3.00083551 | 82.8 | 1.74558406 |
| 82.9 | 0.64723903 | 82.9 | 1.44157784 | 82.9 | 1.44157784 | 82.9 | 1.7357774 | 82.9 | 2.50069626 | | | 82.90 | 1.75539071 | 82.9 | 3.00083551 | 82.9 | 1.75539071 |
| 83 | 0.67665899 | 83 | 1.41215789 | 83 | 1.41215789 | 83 | 1.7161641 | 83 | 2.51050291 | | | 83.00 | 1.7357774 | 83 | 3.01064216 | 83 | 1.7357774 |
| 83.1 | 0.67665899 | 83.1 | 1.44157784 | 83.1 | 1.44157784 | 83.1 | 1.72597075 | 83.1 | 2.50069626 | | | 83.10 | 1.74558406 | 83.1 | 3.01064216 | 83.1 | 1.74558406 |
| 83.2 | 0.67665899 | 83.2 | 1.43177119 | 83.2 | 1.43177119 | 83.2 | 1.74558406 | 83.2 | 2.52030956 | | | 83.20 | 1.78481066 | 83.2 | 3.01064216 | 83.2 | 1.78481066 |
| 83.3 | 0.66685234 | 83.3 | 1.42196454 | 83.3 | 1.42196454 | 83.3 | 1.7357774 | 83.3 | 2.51050291 | | | 83.30 | 1.77500401 | 83.3 | 3.02044882 | 83.3 | 1.77500401 |
| 83.4 | 0.64723903 | 83.4 | 1.43177119 | 83.4 | 1.43177119 | 83.4 | 1.70635745 | 83.4 | 2.50069626 | | | 83.40 | 1.76519736 | 83.4 | 3.03025547 | 83.4 | 1.76519736 |
| 83.5 | 0.64723903 | 83.5 | 1.41215789 | 83.5 | 1.41215789 | 83.5 | 1.7161641 | 83.5 | 2.52030956 | | | 83.50 | 1.75539071 | 83.5 | 3.06948208 | 83.5 | 1.75539071 |
| 83.6 | 0.68646564 | 83.6 | 1.41215789 | 83.6 | 1.41215789 | 83.6 | 1.7357774 | 83.6 | 2.53011622 | | | 83.60 | 1.7357774 | 83.6 | 3.04006212 | 83.6 | 1.7357774 |
| 83.7 | 0.67665899 | 83.7 | 1.4513845 | 83.7 | 1.4513845 | 83.7 | 1.74558406 | 83.7 | 2.54972952 | | | 83.70 | 1.72597075 | 83.7 | 3.06948208 | 83.7 | 1.72597075 |
| 83.8 | 0.64723903 | 83.8 | 1.46119115 | 83.8 | 1.46119115 | 83.8 | 1.7357774 | 83.8 | 2.53992287 | | | 83.80 | 1.7161641 | 83.8 | 3.05967542 | 83.8 | 1.7161641 |
| 83.9 | 0.67665899 | 83.9 | 1.46119115 | 83.9 | 1.46119115 | 83.9 | 1.72597075 | 83.9 | 2.53992287 | | | 83.90 | 1.75539071 | 83.9 | 3.06948208 | 83.9 | 1.75539071 |
| 84 | 0.68646564 | 84 | 1.4709978 | 84 | 1.4709978 | 84 | 1.75539071 | 84 | 2.57914948 | | | 84.00 | 1.74558406 | 84 | 3.06948208 | 84 | 1.74558406 |
| 84.1 | 0.65704568 | 84.1 | 1.4513845 | 84.1 | 1.4513845 | 84.1 | 1.75539071 | 84.1 | 2.58895613 | | | 84.10 | 1.74558406 | 84.1 | 3.04986877 | 84.1 | 1.74558406 |
| 84.2 | 0.63743238 | 84.2 | 1.4709978 | 84.2 | 1.4709978 | 84.2 | 1.7357774 | 84.2 | 2.57914948 | | | 84.20 | 1.7357774 | 84.2 | 3.05967542 | 84.2 | 1.7357774 |
| 84.3 | 0.64723903 | 84.3 | 1.4513845 | 84.3 | 1.4513845 | 84.3 | 1.75539071 | 84.3 | 2.58895613 | | | 84.30 | 1.72597075 | 84.3 | 3.07928873 | 84.3 | 1.72597075 |
| 84.4 | 0.67665899 | 84.4 | 1.4513845 | 84.4 | 1.4513845 | 84.4 | 1.72597075 | 84.4 | 2.60856943 | | | 84.40 | 1.74558406 | 84.4 | 3.07928873 | 84.4 | 1.74558406 |
| 84.5 | 0.67665899 | 84.5 | 1.4709978 | 84.5 | 1.4709978 | 84.5 | 1.74558406 | 84.5 | 2.60856943 | | | 84.50 | 1.75539071 | 84.5 | 3.10870868 | 84.5 | 1.75539071 |
| 84.6 | 0.68646564 | 84.6 | 1.46119115 | 84.6 | 1.46119115 | 84.6 | 1.78481066 | 84.6 | 2.57914948 | | | 84.60 | 1.74558406 | 84.6 | 3.09890203 | 84.6 | 1.74558406 |
| 84.7 | 0.69627229 | 84.7 | 1.4906111 | 84.7 | 1.4906111 | 84.7 | 1.72597075 | 84.7 | 2.63798939 | | | 84.70 | 1.75539071 | 84.7 | 3.09890203 | 84.7 | 1.75539071 |
| 84.8 | 0.67665899 | 84.8 | 1.52983771 | 84.8 | 1.52983771 | 84.8 | 1.7357774 | 84.8 | 2.62818274 | | | 84.80 | 1.74558406 | 84.8 | 3.09890203 | 84.8 | 1.74558406 |
| 84.9 | 0.7158856 | 84.9 | 1.50041776 | 84.9 | 1.50041776 | 84.9 | 1.74558406 | 84.9 | 2.62818274 | | | 84.90 | 1.76519736 | 84.9 | 3.11851534 | 84.9 | 1.76519736 |
| 85 | 0.72569225 | 85 | 1.48080445 | 85 | 1.48080445 | 85 | 1.76519736 | 85 | 2.62818274 | | | 85.00 | 1.78481066 | 85 | 3.12832199 | 85 | 1.78481066 |
| 85.1 | 0.70607894 | 85.1 | 1.4906111 | 85.1 | 1.4906111 | 85.1 | 1.78481066 | 85.1 | 2.65760269 | | | 85.10 | 1.77500401 | 85.1 | 3.14793529 | 85.1 | 1.77500401 |
| 85.2 | 0.70607894 | 85.2 | 1.48080445 | 85.2 | 1.48080445 | 85.2 | 1.79461732 | 85.2 | 2.66740934 | | | 85.20 | 1.75539071 | 85.2 | 3.14793529 | 85.2 | 1.75539071 |
| 85.3 | 0.66685234 | 85.3 | 1.4906111 | 85.3 | 1.4906111 | 85.3 | 1.78481066 | 85.3 | 2.64779604 | | | 85.30 | 1.75539071 | 85.3 | 3.12832199 | 85.3 | 1.75539071 |
| 85.4 | 0.65704568 | 85.4 | 1.52983771 | 85.4 | 1.52983771 | 85.4 | 1.76519736 | 85.4 | 2.63798939 | | | 85.40 | 1.72597075 | 85.4 | 3.11851534 | 85.4 | 1.72597075 |
| 85.5 | 0.66685234 | 85.5 | 1.52983771 | 85.5 | 1.52983771 | 85.5 | 1.76519736 | 85.5 | 2.65760269 | | | 85.50 | 1.7357774 | 85.5 | 3.12832199 | 85.5 | 1.7357774 |
| 85.6 | 0.68646564 | 85.6 | 1.48080445 | 85.6 | 1.48080445 | 85.6 | 1.77500401 | 85.6 | 2.65760269 | | | 85.60 | 1.72597075 | 85.6 | 3.13812864 | 85.6 | 1.72597075 |
| 85.7 | 0.67665899 | 85.7 | 1.48080445 | 85.7 | 1.48080445 | 85.7 | 1.77500401 | 85.7 | 2.65760269 | | | 85.70 | 1.72597075 | 85.7 | 3.14793529 | 85.7 | 1.72597075 |
| 85.8 | 0.69627229 | 85.8 | 1.48080445 | 85.8 | 1.48080445 | 85.8 | 1.77500401 | 85.8 | 2.65760269 | | | 85.80 | 1.76519736 | 85.8 | 3.1871619 | 85.8 | 1.76519736 |
| 85.9 | 0.69627229 | 85.9 | 1.4906111 | 85.9 | 1.4906111 | 85.9 | 1.79461732 | 85.9 | 2.6968293 | | | 85.90 | 1.77500401 | 85.9 | 3.19696855 | 85.9 | 1.77500401 |
| 86 | 0.65704568 | 86 | 1.51022441 | 86 | 1.51022441 | 86 | 1.78481066 | 86 | 2.68702265 | | | 86.00 | 1.75539071 | 86 | 3.19696855 | 86 | 1.75539071 |
| 86.1 | 0.66685234 | 86.1 | 1.52983771 | 86.1 | 1.52983771 | 86.1 | 1.76519736 | 86.1 | 2.6968293 | | | 86.10 | 1.7357774 | 86.1 | 3.2067752 | 86.1 | 1.7357774 |
| 86.2 | 0.67665899 | 86.2 | 1.54945102 | 86.2 | 1.54945102 | 86.2 | 1.75539071 | 86.2 | 2.7164426 | | | 86.20 | 1.80442397 | 86.2 | 3.22638851 | 86.2 | 1.80442397 |
| 86.3 | 0.68646564 | 86.3 | 1.52983771 | 86.3 | 1.52983771 | 86.3 | 1.79461732 | 86.3 | 2.72624926 | | | 86.30 | 1.79461732 | 86.3 | 3.24600181 | 86.3 | 1.79461732 |
| 86.4 | 0.68646564 | 86.4 | 1.52983771 | 86.4 | 1.52983771 | 86.4 | 1.81423062 | 86.4 | 2.72624926 | | | 86.40 | 1.76519736 | 86.4 | 3.25580846 | 86.4 | 1.76519736 |
| 86.5 | 0.66685234 | 86.5 | 1.52983771 | 86.5 | 1.52983771 | 86.5 | 1.80442397 | 86.5 | 2.73605591 | | | 86.50 | 1.76519736 | 86.5 | 3.25580846 | 86.5 | 1.76519736 |
| 86.6 | 0.69627229 | 86.6 | 1.56906432 | 86.6 | 1.56906432 | 86.6 | 1.79461732 | 86.6 | 2.76547586 | | | 86.60 | 1.79461732 | 86.6 | 3.24600181 | 86.6 | 1.79461732 |
| 86.7 | 0.7158856 | 86.7 | 1.58867762 | 86.7 | 1.58867762 | 86.7 | 1.79461732 | 86.7 | 2.75566921 | | | 86.70 | 1.77500401 | 86.7 | 3.25580846 | 86.7 | 1.77500401 |
| 86.8 | 0.70607894 | 86.8 | 1.58867762 | 86.8 | 1.58867762 | 86.8 | 1.80442397 | 86.8 | 2.75566921 | | | 86.80 | 1.75539071 | 86.8 | 3.25580846 | 86.8 | 1.75539071 |
| 86.9 | 0.69627229 | 86.9 | 1.57887097 | 86.9 | 1.57887097 | 86.9 | 1.82403727 | 86.9 | 2.77528252 | | | 86.90 | 1.75539071 | 86.9 | 3.27542177 | 86.9 | 1.75539071 |
| 87 | 0.68646564 | 87 | 1.55925767 | 87 | 1.55925767 | 87 | 1.82403727 | 87 | 2.75566921 | | | 87.00 | 1.76519736 | 87 | 3.28522842 | 87 | 1.76519736 |
| 87.1 | 0.68646564 | 87.1 | 1.56906432 | 87.1 | 1.56906432 | 87.1 | 1.80442397 | 87.1 | 2.78508917 | | | 87.10 | 1.75539071 | 87.1 | 3.29503507 | 87.1 | 1.75539071 |
| 87.2 | 0.68646564 | 87.2 | 1.54945102 | 87.2 | 1.54945102 | 87.2 | 1.81423062 | 87.2 | 2.77528252 | | | 87.20 | 1.75539071 | 87.2 | 3.29503507 | 87.2 | 1.75539071 |
| 87.3 | 0.67665899 | 87.3 | 1.52983771 | 87.3 | 1.52983771 | 87.3 | 1.82403727 | 87.3 | 2.79489582 | | | 87.30 | 1.74558406 | 87.3 | 3.30484172 | 87.3 | 1.74558406 |
| 87.4 | 0.69627229 | 87.4 | 1.55925767 | 87.4 | 1.55925767 | 87.4 | 1.83384392 | 87.4 | 2.80470247 | | | 87.40 | 1.75539071 | 87.4 | 3.30484172 | 87.4 | 1.75539071 |
| 87.5 | 0.68646564 | 87.5 | 1.58867762 | 87.5 | 1.58867762 | 87.5 | 1.84365058 | 87.5 | 2.79489582 | | | 87.50 | 1.76519736 | 87.5 | 3.30484172 | 87.5 | 1.76519736 |
| 87.6 | 0.70607894 | 87.6 | 1.58867762 | 87.6 | 1.58867762 | 87.6 | 1.83384392 | 87.6 | 2.78508917 | | | 87.60 | 1.75539071 | 87.6 | 3.34406833 | 87.6 | 1.75539071 |
| 87.7 | 0.7158856 | 87.7 | 1.60829093 | 87.7 | 1.60829093 | 87.7 | 1.81423062 | 87.7 | 2.81450912 | | | 87.70 | 1.77500401 | 87.7 | 3.35387498 | 87.7 | 1.77500401 |
| 87.8 | 0.67665899 | 87.8 | 1.61809758 | 87.8 | 1.61809758 | 87.8 | 1.81423062 | 87.8 | 2.81450912 | | | 87.80 | 1.79461732 | 87.8 | 3.37348829 | 87.8 | 1.79461732 |
| 87.9 | 0.68646564 | 87.9 | 1.59848428 | 87.9 | 1.59848428 | 87.9 | 1.80442397 | 87.9 | 2.82431578 | | | 87.90 | 1.80442397 | 87.9 | 3.38329494 | 87.9 | 1.80442397 |
| 88 | 0.67665899 | 88 | 1.58867762 | 88 | 1.58867762 | 88 | 1.83384392 | 88 | 2.83412243 | | | 88.00 | 1.84365058 | 88 | 3.38329494 | 88 | 1.84365058 |
| 88.1 | 0.69627229 | 88.1 | 1.57887097 | 88.1 | 1.57887097 | 88.1 | 1.85345723 | 88.1 | 2.84392908 | | | 88.10 | 1.82403727 | 88.1 | 3.37348829 | 88.1 | 1.82403727 |
| 88.2 | 0.7158856 | 88.2 | 1.58867762 | 88.2 | 1.58867762 | 88.2 | 1.86326388 | 88.2 | 2.85373573 | | | 88.20 | 1.79461732 | 88.2 | 3.38329494 | 88.2 | 1.79461732 |
| 88.3 | 0.70607894 | 88.3 | 1.58867762 | 88.3 | 1.58867762 | 88.3 | 1.86326388 | 88.3 | 2.86354238 | | | 88.30 | 1.75539071 | 88.3 | 3.4127149 | 88.3 | 1.75539071 |
| 88.4 | 0.67665899 | 88.4 | 1.60829093 | 88.4 | 1.60829093 | 88.4 | 1.84365058 | 88.4 | 2.88315569 | | | 88.40 | 1.75539071 | 88.4 | 3.40290824 | 88.4 | 1.75539071 |
| 88.5 | 0.68646564 | 88.5 | 1.61809758 | 88.5 | 1.61809758 | 88.5 | 1.84365058 | 88.5 | 2.89296234 | | | 88.50 | 1.77500401 | 88.5 | 3.4323282 | 88.5 | 1.77500401 |
| 88.6 | 0.68646564 | 88.6 | 1.62790423 | 88.6 | 1.62790423 | 88.6 | 1.81423062 | 88.6 | 2.89296234 | | | 88.60 | 1.78481066 | 88.6 | 3.4323282 | 88.6 | 1.78481066 |
| 88.7 | 0.7158856 | 88.7 | 1.66713084 | 88.7 | 1.66713084 | 88.7 | 1.84365058 | 88.7 | 2.90276899 | | | 88.70 | 1.76519736 | 88.7 | 3.4127149 | 88.7 | 1.76519736 |

| | | | | | | | | | | | | | | | | | |
|---|---|---|---|---|---|---|---|---|---|---|---|---|---|---|---|---|---|
| 88.8 | 0.7158856 | 88.8 | 1.64751754 | 88.8 | 1.64751754 | 88.8 | 1.88287718 | 88.8 | 2.9223823 | | | 88.80 | 1.79461732 | 88.8 | 3.4127149 | 88.8 | 1.79461732 |
| 88.9 | 0.69627229 | 88.9 | 1.62790423 | 88.9 | 1.62790423 | 88.9 | 1.89268384 | 88.9 | 2.93218895 | | | 88.90 | 1.79461732 | 88.9 | 3.44213485 | 88.9 | 1.79461732 |
| 89 | 0.68646564 | 89 | 1.62790423 | 89 | 1.62790423 | 89 | 1.86326388 | 89 | 2.9223823 | | | 89.00 | 1.81423062 | 89 | 3.42252155 | 89 | 1.81423062 |
| 89.1 | 0.69627229 | 89.1 | 1.62790423 | 89.1 | 1.62790423 | 89.1 | 1.85345723 | 89.1 | 2.9419956 | | | 89.10 | 1.81423062 | 89.1 | 3.4323282 | 89.1 | 1.81423062 |
| 89.2 | 0.7158856 | 89.2 | 1.60829093 | 89.2 | 1.60829093 | 89.2 | 1.84365058 | 89.2 | 2.9419956 | | | 89.20 | 1.79461732 | 89.2 | 3.47155481 | 89.2 | 1.79461732 |
| 89.3 | 0.68646564 | 89.3 | 1.58867762 | 89.3 | 1.58867762 | 89.3 | 1.83384392 | 89.3 | 2.9419956 | | | 89.30 | 1.79461732 | 89.3 | 3.44213485 | 89.3 | 1.79461732 |
| 89.4 | 0.68646564 | 89.4 | 1.59848428 | 89.4 | 1.59848428 | 89.4 | 1.84365058 | 89.4 | 2.93218895 | | | 89.40 | 1.80442397 | 89.4 | 3.4519415 | 89.4 | 1.80442397 |
| 89.5 | 0.70607894 | 89.5 | 1.58867762 | 89.5 | 1.58867762 | 89.5 | 1.84365058 | 89.5 | 2.9419956 | | | 89.50 | 1.79461732 | 89.5 | 3.48136146 | 89.5 | 1.79461732 |
| 89.6 | 0.69627229 | 89.6 | 1.59848428 | 89.6 | 1.59848428 | 89.6 | 1.83384392 | 89.6 | 2.97141556 | | | 89.60 | 1.82403727 | 89.6 | 3.48136146 | 89.6 | 1.82403727 |
| 89.7 | 0.7158856 | 89.7 | 1.63771088 | 89.7 | 1.63771088 | 89.7 | 1.83384392 | 89.7 | 2.97141556 | | | 89.70 | 1.82403727 | 89.7 | 3.48136146 | 89.7 | 1.82403727 |
| 89.8 | 0.68646564 | 89.8 | 1.63771088 | 89.8 | 1.63771088 | 89.8 | 1.84365058 | 89.8 | 2.97141556 | | | 89.80 | 1.80442397 | 89.8 | 3.50097476 | 89.8 | 1.80442397 |
| 89.9 | 0.68646564 | 89.9 | 1.63771088 | 89.9 | 1.63771088 | 89.9 | 1.88287718 | 89.9 | 2.97141556 | | | 89.90 | 1.80442397 | 89.9 | 3.49116811 | 89.9 | 1.80442397 |
| 90 | 0.69627229 | 90 | 1.67693749 | 90 | 1.67693749 | 90 | 1.85345723 | 90 | 2.98122221 | | | 90.00 | 1.81423062 | 90 | 3.50097476 | 90 | 1.81423062 |
| 90.1 | 0.69627229 | 90.1 | 1.66713084 | 90.1 | 1.66713084 | 90.1 | 1.84365058 | 90.1 | 2.98122221 | | | 90.10 | 1.80442397 | 90.1 | 3.52058807 | 90.1 | 1.80442397 |
| 90.2 | 0.68646564 | 90.2 | 1.65732419 | 90.2 | 1.65732419 | 90.2 | 1.87307053 | 90.2 | 3.00083551 | | | 90.20 | 1.80442397 | 90.2 | 3.50097476 | 90.2 | 1.80442397 |
| 90.3 | 0.70607894 | 90.3 | 1.63771088 | 90.3 | 1.63771088 | 90.3 | 1.87307053 | 90.3 | 3.01064216 | | | 90.30 | 1.87307053 | 90.3 | 3.50097476 | 90.3 | 1.87307053 |
| 90.4 | 0.70607894 | 90.4 | 1.65732419 | 90.4 | 1.65732419 | 90.4 | 1.85345723 | 90.4 | 3.01064216 | | | 90.40 | 1.85345723 | 90.4 | 3.51078142 | 90.4 | 1.85345723 |
| 90.5 | 0.69627229 | 90.5 | 1.68674414 | 90.5 | 1.68674414 | 90.5 | 1.85345723 | 90.5 | 3.03025547 | | | 90.50 | 1.82403727 | 90.5 | 3.51078142 | 90.5 | 1.82403727 |
| 90.6 | 0.70607894 | 90.6 | 1.67693749 | 90.6 | 1.67693749 | 90.6 | 1.86326388 | 90.6 | 3.03025547 | | | 90.60 | 1.83384392 | 90.6 | 3.53039472 | 90.6 | 1.83384392 |
| 90.7 | 0.68646564 | 90.7 | 1.7161641 | 90.7 | 1.7161641 | 90.7 | 1.87307053 | 90.7 | 3.04006212 | | | 90.70 | 1.85345723 | 90.7 | 3.51078142 | 90.7 | 1.85345723 |
| 90.8 | 0.69627229 | 90.8 | 1.7161641 | 90.8 | 1.7161641 | 90.8 | 1.85345723 | 90.8 | 3.05967542 | | | 90.80 | 1.81423062 | 90.8 | 3.51078142 | 90.8 | 1.81423062 |
| 90.9 | 0.70607894 | 90.9 | 1.7161641 | 90.9 | 1.7161641 | 90.9 | 1.81423062 | 90.9 | 3.05967542 | | | 90.90 | 1.81423062 | 90.9 | 3.51078142 | 90.9 | 1.81423062 |
| 91 | 0.67665899 | 91 | 1.66713084 | 91 | 1.66713084 | 91 | 1.82403727 | 91 | 3.05967542 | | | 91.00 | 1.85345723 | 91 | 3.52058807 | 91 | 1.85345723 |
| 91.1 | 0.69627229 | 91.1 | 1.68674414 | 91.1 | 1.68674414 | 91.1 | 1.89268384 | 91.1 | 3.06948208 | | | 91.10 | 1.85345723 | 91.1 | 3.54020137 | 91.1 | 1.85345723 |
| 91.2 | 0.72569225 | 91.2 | 1.67693749 | 91.2 | 1.67693749 | 91.2 | 1.89268384 | 91.2 | 3.09890203 | | | 91.20 | 1.79461732 | 91.2 | 3.56962133 | 91.2 | 1.79461732 |
| 91.3 | 0.72569225 | 91.3 | 1.66713084 | 91.3 | 1.66713084 | 91.3 | 1.88287718 | 91.3 | 3.10870868 | | | 91.30 | 1.80442397 | 91.3 | 3.54020137 | 91.3 | 1.80442397 |
| 91.4 | 0.70607894 | 91.4 | 1.70635745 | 91.4 | 1.70635745 | 91.4 | 1.88287718 | 91.4 | 3.10870868 | | | 91.40 | 1.82403727 | 91.4 | 3.55981468 | 91.4 | 1.82403727 |
| 91.5 | 0.70607894 | 91.5 | 1.72597075 | 91.5 | 1.72597075 | 91.5 | 1.89268384 | 91.5 | 3.09890203 | | | 91.50 | 1.82403727 | 91.5 | 3.55000802 | 91.5 | 1.82403727 |
| 91.6 | 0.70607894 | 91.6 | 1.7161641 | 91.6 | 1.7161641 | 91.6 | 1.90249049 | 91.6 | 3.10870868 | | | 91.60 | 1.80442397 | 91.6 | 3.55981468 | 91.6 | 1.80442397 |
| 91.7 | 0.7354989 | 91.7 | 1.7357774 | 91.7 | 1.7357774 | 91.7 | 1.88287718 | 91.7 | 3.12832199 | | | 91.70 | 1.80442397 | 91.7 | 3.55981468 | 91.7 | 1.80442397 |
| 91.8 | 0.7354989 | 91.8 | 1.76519736 | 91.8 | 1.76519736 | 91.8 | 1.88287718 | 91.8 | 3.12832199 | | | 91.80 | 1.84365058 | 91.8 | 3.57942798 | 91.8 | 1.84365058 |
| 91.9 | 0.69627229 | 91.9 | 1.74558406 | 91.9 | 1.74558406 | 91.9 | 1.89268384 | 91.9 | 3.13812864 | | | 91.90 | 1.84365058 | 91.9 | 3.55981468 | 91.9 | 1.84365058 |
| 92 | 0.69627229 | 92 | 1.7357774 | 92 | 1.7357774 | 92 | 1.89268384 | 92 | 3.14793529 | | | 92.00 | 1.84365058 | 92 | 3.59904128 | 92 | 1.84365058 |
| 92.1 | 0.70607894 | 92.1 | 1.77500401 | 92.1 | 1.77500401 | 92.1 | 1.93191044 | 92.1 | 3.14793529 | | | 92.10 | 1.84365058 | 92.1 | 3.59904128 | 92.1 | 1.84365058 |
| 92.2 | 0.69627229 | 92.2 | 1.76519736 | 92.2 | 1.76519736 | 92.2 | 1.93191044 | 92.2 | 3.15774194 | | | 92.20 | 1.84365058 | 92.2 | 3.58923463 | 92.2 | 1.84365058 |
| 92.3 | 0.69627229 | 92.3 | 1.75539071 | 92.3 | 1.75539071 | 92.3 | 1.9613304 | 92.3 | 3.15774194 | | | 92.30 | 1.81423062 | 92.3 | 3.58923463 | 92.3 | 1.81423062 |
| 92.4 | 0.70607894 | 92.4 | 1.78481066 | 92.4 | 1.78481066 | 92.4 | 2.00055701 | 92.4 | 3.15774194 | | | 92.40 | 1.82403727 | 92.4 | 3.60884794 | 92.4 | 1.82403727 |
| 92.5 | 0.72569225 | 92.5 | 1.75539071 | 92.5 | 1.75539071 | 92.5 | 1.99075036 | 92.5 | 3.17735525 | | | 92.50 | 1.85345723 | 92.5 | 3.61865459 | 92.5 | 1.85345723 |
| 92.6 | 0.7158856 | 92.6 | 1.75539071 | 92.6 | 1.75539071 | 92.6 | 1.97113705 | 92.6 | 3.1871619 | | | 92.60 | 1.86326388 | 92.6 | 3.62846124 | 92.6 | 1.86326388 |
| 92.7 | 0.69627229 | 92.7 | 1.75539071 | 92.7 | 1.75539071 | 92.7 | 2.00055701 | 92.7 | 3.2067752 | | | 92.70 | 1.84365058 | 92.7 | 3.64807454 | 92.7 | 1.84365058 |
| 92.8 | 0.68646564 | 92.8 | 1.77500401 | 92.8 | 1.77500401 | 92.8 | 2.02017031 | 92.8 | 3.22638851 | | | 92.80 | 1.84365058 | 92.8 | 3.63826789 | 92.8 | 1.84365058 |
| 92.9 | 0.66685234 | 92.9 | 1.78481066 | 92.9 | 1.78481066 | 92.9 | 2.02017031 | 92.9 | 3.22638851 | | | 92.90 | 1.85345723 | 92.9 | 3.63826789 | 92.9 | 1.85345723 |
| 93 | 0.7158856 | 93 | 1.77500401 | 93 | 1.77500401 | 93 | 2.02017031 | 93 | 3.22638851 | | | 93.00 | 1.82403727 | 93 | 3.62846124 | 93 | 1.82403727 |
| 93.1 | 0.69627229 | 93.1 | 1.76519736 | 93.1 | 1.76519736 | 93.1 | 2.02017031 | 93.1 | 3.24600181 | | | 93.10 | 1.82403727 | 93.1 | 3.62846124 | 93.1 | 1.82403727 |
| 93.2 | 0.68646564 | 93.2 | 1.78481066 | 93.2 | 1.78481066 | 93.2 | 2.02997696 | 93.2 | 3.23619516 | | | 93.20 | 1.83384392 | 93.2 | 3.63826789 | 93.2 | 1.83384392 |
| 93.3 | 0.7158856 | 93.3 | 1.79461732 | 93.3 | 1.79461732 | 93.3 | 2.02017031 | 93.3 | 3.22638851 | | | 93.30 | 1.86326388 | 93.3 | 3.63826789 | 93.3 | 1.86326388 |
| 93.4 | 0.72569225 | 93.4 | 1.76519736 | 93.4 | 1.76519736 | 93.4 | 2.03978362 | 93.4 | 3.25580846 | | | 93.40 | 1.87307053 | 93.4 | 3.6578812 | 93.4 | 1.87307053 |
| 93.5 | 0.74530555 | 93.5 | 1.75539071 | 93.5 | 1.75539071 | 93.5 | 2.03978362 | 93.5 | 3.26561512 | | | 93.50 | 1.87307053 | 93.5 | 3.64807454 | 93.5 | 1.87307053 |
| 93.6 | 0.7551122 | 93.6 | 1.79461732 | 93.6 | 1.79461732 | 93.6 | 2.03978362 | 93.6 | 3.26561512 | | | 93.60 | 1.85345723 | 93.6 | 3.64807454 | 93.6 | 1.85345723 |
| 93.7 | 0.72569225 | 93.7 | 1.79461732 | 93.7 | 1.79461732 | 93.7 | 2.02997696 | 93.7 | 3.26561512 | | | 93.70 | 1.86326388 | 93.7 | 3.64807454 | 93.7 | 1.86326388 |
| 93.8 | 0.69627229 | 93.8 | 1.80442397 | 93.8 | 1.80442397 | 93.8 | 2.02997696 | 93.8 | 3.28522842 | | | 93.80 | 1.83384392 | 93.8 | 3.62846124 | 93.8 | 1.83384392 |
| 93.9 | 0.7158856 | 93.9 | 1.83384392 | 93.9 | 1.83384392 | 93.9 | 2.04959027 | 93.9 | 3.28522842 | | | 93.90 | 1.87307053 | 93.9 | 3.64807454 | 93.9 | 1.87307053 |
| 94 | 0.72569225 | 94 | 1.81423062 | 94 | 1.81423062 | 94 | 2.04959027 | 94 | 3.27542177 | | | 94.00 | 1.89268384 | 94 | 3.62846124 | 94 | 1.89268384 |
| 94.1 | 0.72569225 | 94.1 | 1.82403727 | 94.1 | 1.82403727 | 94.1 | 2.06920357 | 94.1 | 3.30484172 | | | 94.10 | 1.87307053 | 94.1 | 3.61865459 | 94.1 | 1.87307053 |
| 94.2 | 0.7551122 | 94.2 | 1.83384392 | 94.2 | 1.83384392 | 94.2 | 2.06920357 | 94.2 | 3.31464838 | | | 94.20 | 1.85345723 | 94.2 | 3.6774945 | 94.2 | 1.85345723 |
| 94.3 | 0.7354989 | 94.3 | 1.80442397 | 94.3 | 1.80442397 | 94.3 | 2.04959027 | 94.3 | 3.32445503 | | | 94.30 | 1.85345723 | 94.3 | 3.68730115 | 94.3 | 1.85345723 |
| 94.4 | 0.7551122 | 94.4 | 1.81423062 | 94.4 | 1.81423062 | 94.4 | 2.06920357 | 94.4 | 3.32445503 | | | 94.40 | 1.83384392 | 94.4 | 3.66768785 | 94.4 | 1.83384392 |
| 94.5 | 0.76491886 | 94.5 | 1.82403727 | 94.5 | 1.82403727 | 94.5 | 2.06920357 | 94.5 | 3.32445503 | | | 94.50 | 1.87307053 | 94.5 | 3.66768785 | 94.5 | 1.87307053 |
| 94.6 | 0.72569225 | 94.6 | 1.81423062 | 94.6 | 1.81423062 | 94.6 | 2.05939692 | 94.6 | 3.34406833 | | | 94.60 | 1.86326388 | 94.6 | 3.66768785 | 94.6 | 1.86326388 |
| 94.7 | 0.72569225 | 94.7 | 1.82403727 | 94.7 | 1.82403727 | 94.7 | 2.09862353 | 94.7 | 3.37348829 | | | 94.70 | 1.88287718 | 94.7 | 3.6774945 | 94.7 | 1.88287718 |
| 94.8 | 0.72569225 | 94.8 | 1.83384392 | 94.8 | 1.83384392 | 94.8 | 2.08881688 | 94.8 | 3.37348829 | | | 94.80 | 1.84365058 | 94.8 | 3.70691446 | 94.8 | 1.84365058 |

| | | | | | | | | | | | | | | | | | |
|---|---|---|---|---|---|---|---|---|---|---|---|---|---|---|---|---|---|
| 94.9 | 0.72569225 | 94.9 | 1.84365058 | 94.9 | 1.84365058 | 94.9 | 2.07901022 | 94.9 | 3.37348829 | | | 94.90 | 1.85345723 | 94.9 | 3.6774945 | 94.9 | 1.85345723 |
| 95 | 0.72569225 | 95 | 1.84365058 | 95 | 1.84365058 | 95 | 2.08881688 | 95 | 3.37348829 | | | 95.00 | 1.84365058 | 95 | 3.68730115 | 95 | 1.84365058 |
| 95.1 | 0.72569225 | 95.1 | 1.82403727 | 95.1 | 1.82403727 | 95.1 | 2.07901022 | 95.1 | 3.36368164 | | | 95.10 | 1.86326388 | 95.1 | 3.6774945 | 95.1 | 1.86326388 |
| 95.2 | 0.70607894 | 95.2 | 1.83384392 | 95.2 | 1.83384392 | 95.2 | 2.10843018 | 95.2 | 3.42252155 | | | 95.20 | 1.86326388 | 95.2 | 3.6971078 | 95.2 | 1.86326388 |
| 95.3 | 0.74530555 | 95.3 | 1.85345723 | 95.3 | 1.85345723 | 95.3 | 2.12804348 | 95.3 | 3.40290824 | | | 95.30 | 1.83384392 | 95.3 | 3.72652776 | 95.3 | 1.83384392 |
| 95.4 | 0.7551122 | 95.4 | 1.87307053 | 95.4 | 1.87307053 | 95.4 | 2.13785014 | 95.4 | 3.40290824 | | | 95.40 | 1.84365058 | 95.4 | 3.6774945 | 95.4 | 1.84365058 |
| 95.5 | 0.7354989 | 95.5 | 1.87307053 | 95.5 | 1.87307053 | 95.5 | 2.11823683 | 95.5 | 3.38329494 | | | 95.50 | 1.86326388 | 95.5 | 3.6774945 | 95.5 | 1.86326388 |
| 95.6 | 0.74530555 | 95.6 | 1.86326388 | 95.6 | 1.86326388 | 95.6 | 2.11823683 | 95.6 | 3.42252155 | | | 95.60 | 1.85345723 | 95.6 | 3.72652776 | 95.6 | 1.85345723 |
| 95.7 | 0.7551122 | 95.7 | 1.86326388 | 95.7 | 1.86326388 | 95.7 | 2.15746344 | 95.7 | 3.4519415 | | | 95.70 | 1.83384392 | 95.7 | 3.71672111 | 95.7 | 1.83384392 |
| 95.8 | 0.74530555 | 95.8 | 1.89268384 | 95.8 | 1.89268384 | 95.8 | 2.15746344 | 95.8 | 3.4519415 | | | 95.80 | 1.84365058 | 95.8 | 3.72652776 | 95.8 | 1.84365058 |
| 95.9 | 0.7354989 | 95.9 | 1.86326388 | 95.9 | 1.86326388 | 95.9 | 2.15746344 | 95.9 | 3.46174816 | | | 95.90 | 1.83384392 | 95.9 | 3.74614106 | 95.9 | 1.83384392 |
| 96 | 0.74530555 | 96 | 1.88287718 | 96 | 1.88287718 | 96 | 2.1868834 | 96 | 3.47155481 | | | 96.00 | 1.89268384 | 96 | 3.75594772 | 96 | 1.89268384 |
| 96.1 | 0.74530555 | 96.1 | 1.87307053 | 96.1 | 1.87307053 | 96.1 | 2.1868834 | 96.1 | 3.49116811 | | | 96.10 | 1.88287718 | 96.1 | 3.75594772 | 96.1 | 1.88287718 |
| 96.2 | 0.74530555 | 96.2 | 1.87307053 | 96.2 | 1.87307053 | 96.2 | 2.15746344 | 96.2 | 3.49116811 | | | 96.20 | 1.87307053 | 96.2 | 3.76575437 | 96.2 | 1.87307053 |
| 96.3 | 0.74530555 | 96.3 | 1.89268384 | 96.3 | 1.89268384 | 96.3 | 2.15746344 | 96.3 | 3.51078142 | | | 96.30 | 1.84365058 | 96.3 | 3.78536767 | 96.3 | 1.84365058 |
| 96.4 | 0.7551122 | 96.4 | 1.89268384 | 96.4 | 1.89268384 | 96.4 | 2.13785014 | 96.4 | 3.50097476 | | | 96.40 | 1.81423062 | 96.4 | 3.77556102 | 96.4 | 1.81423062 |
| 96.5 | 0.74530555 | 96.5 | 1.88287718 | 96.5 | 1.88287718 | 96.5 | 2.16727009 | 96.5 | 3.52058807 | | | 96.50 | 1.82403727 | 96.5 | 3.77556102 | 96.5 | 1.82403727 |
| 96.6 | 0.7354989 | 96.6 | 1.89268384 | 96.6 | 1.89268384 | 96.6 | 2.16727009 | 96.6 | 3.52058807 | | | 96.60 | 1.85345723 | 96.6 | 3.76575437 | 96.6 | 1.85345723 |
| 96.7 | 0.76491886 | 96.7 | 1.88287718 | 96.7 | 1.88287718 | 96.7 | 2.14765679 | 96.7 | 3.53039472 | | | 96.70 | 1.84365058 | 96.7 | 3.74614106 | 96.7 | 1.84365058 |
| 96.8 | 0.7551122 | 96.8 | 1.86326388 | 96.8 | 1.86326388 | 96.8 | 2.17707674 | 96.8 | 3.52058807 | | | 96.80 | 1.83384392 | 96.8 | 3.78536767 | 96.8 | 1.83384392 |
| 96.9 | 0.74530555 | 96.9 | 1.87307053 | 96.9 | 1.87307053 | 96.9 | 2.1868834 | 96.9 | 3.53039472 | | | 96.90 | 1.85345723 | 96.9 | 3.76575437 | 96.9 | 1.85345723 |
| 97 | 0.7551122 | 97 | 1.87307053 | 97 | 1.87307053 | 97 | 2.17707674 | 97 | 3.52058807 | | | 97.00 | 1.80442397 | 97 | 3.75594772 | 97 | 1.80442397 |
| 97.1 | 0.74530555 | 97.1 | 1.86326388 | 97.1 | 1.86326388 | 97.1 | 2.19669005 | 97.1 | 3.54020137 | | | 97.10 | 1.76519736 | 97.1 | 3.76575437 | 97.1 | 1.76519736 |
| 97.2 | 0.72569225 | 97.2 | 1.87307053 | 97.2 | 1.87307053 | 97.2 | 2.16727009 | 97.2 | 3.54020137 | | | 97.20 | 1.78481066 | 97.2 | 3.79517432 | 97.2 | 1.78481066 |
| 97.3 | 0.7354989 | 97.3 | 1.89268384 | 97.3 | 1.89268384 | 97.3 | 2.14765679 | 97.3 | 3.54020137 | | | 97.30 | 1.81423062 | 97.3 | 3.78536767 | 97.3 | 1.81423062 |
| 97.4 | 0.79433881 | 97.4 | 1.91229714 | 97.4 | 1.91229714 | 97.4 | 2.17707674 | 97.4 | 3.55000802 | | | 97.40 | 1.80442397 | 97.4 | 3.76575437 | 97.4 | 1.80442397 |
| 97.5 | 0.77472551 | 97.5 | 1.90249049 | 97.5 | 1.90249049 | 97.5 | 2.19669005 | 97.5 | 3.55000802 | | | 97.50 | 1.77500401 | 97.5 | 3.78536767 | 97.5 | 1.77500401 |
| 97.6 | 0.74530555 | 97.6 | 1.91229714 | 97.6 | 1.91229714 | 97.6 | 2.2064967 | 97.6 | 3.59904128 | | | 97.60 | 1.77500401 | 97.6 | 3.81478763 | 97.6 | 1.77500401 |
| 97.7 | 0.74530555 | 97.7 | 1.91229714 | 97.7 | 1.91229714 | 97.7 | 2.2064967 | 97.7 | 3.60884794 | | | 97.70 | 1.77500401 | 97.7 | 3.83440093 | 97.7 | 1.77500401 |
| 97.8 | 0.74530555 | 97.8 | 1.91229714 | 97.8 | 1.91229714 | 97.8 | 2.19669005 | 97.8 | 3.60884794 | | | 97.80 | 1.81423062 | 97.8 | 3.86382089 | 97.8 | 1.81423062 |
| 97.9 | 0.74530555 | 97.9 | 1.93191044 | 97.9 | 1.93191044 | 97.9 | 2.1868834 | 97.9 | 3.62846124 | | | 97.90 | 1.80442397 | 97.9 | 3.85401424 | 97.9 | 1.80442397 |
| 98 | 0.7551122 | 98 | 1.9613304 | 98 | 1.9613304 | 98 | 2.19669005 | 98 | 3.62846124 | | | 98.00 | 1.76519736 | 98 | 3.83440093 | 98 | 1.76519736 |
| 98.1 | 0.7354989 | 98.1 | 1.95152375 | 98.1 | 1.95152375 | 98.1 | 2.2064967 | 98.1 | 3.64807454 | | | 98.10 | 1.79461732 | 98.1 | 3.85401424 | 98.1 | 1.79461732 |
| 98.2 | 0.72569225 | 98.2 | 1.9613304 | 98.2 | 1.9613304 | 98.2 | 2.2064967 | 98.2 | 3.63826789 | | | 98.20 | 1.83384392 | 98.2 | 3.87362754 | 98.2 | 1.83384392 |
| 98.3 | 0.7354989 | 98.3 | 1.95152375 | 98.3 | 1.95152375 | 98.3 | 2.21630335 | 98.3 | 3.63826789 | | | 98.30 | 1.82403727 | 98.3 | 3.87362754 | 98.3 | 1.82403727 |
| 98.4 | 0.74530555 | 98.4 | 1.95152375 | 98.4 | 1.95152375 | 98.4 | 2.22611 | 98.4 | 3.63826789 | | | 98.40 | 1.80442397 | 98.4 | 3.83440093 | 98.4 | 1.80442397 |
| 98.5 | 0.7354989 | 98.5 | 1.9613304 | 98.5 | 1.9613304 | 98.5 | 2.22611 | 98.5 | 3.62846124 | | | 98.50 | 1.79461732 | 98.5 | 3.83440093 | 98.5 | 1.79461732 |
| 98.6 | 0.7551122 | 98.6 | 1.93191044 | 98.6 | 1.93191044 | 98.6 | 2.23591666 | 98.6 | 3.64807454 | | | 98.60 | 1.79461732 | 98.6 | 3.85401424 | 98.6 | 1.79461732 |
| 98.7 | 0.76491886 | 98.7 | 1.9417171 | 98.7 | 1.9417171 | 98.7 | 2.23591666 | 98.7 | 3.68730115 | | | 98.70 | 1.78481066 | 98.7 | 3.86382089 | 98.7 | 1.78481066 |
| 98.8 | 0.76491886 | 98.8 | 1.99075036 | 98.8 | 1.99075036 | 98.8 | 2.21630335 | 98.8 | 3.71672111 | | | 98.80 | 1.79461732 | 98.8 | 3.86382089 | 98.8 | 1.79461732 |
| 98.9 | 0.76491886 | 98.9 | 1.9809437 | 98.9 | 1.9809437 | 98.9 | 2.23591666 | 98.9 | 3.72652776 | | | 98.90 | 1.78481066 | 98.9 | 3.9030475 | 98.9 | 1.78481066 |
| 99 | 0.77472551 | 99 | 1.97113705 | 99 | 1.97113705 | 99 | 2.26533661 | 99 | 3.74614106 | | | 99.00 | 1.79461732 | 99 | 3.97169406 | 99 | 1.79461732 |
| 99.1 | 0.7158856 | 99.1 | 1.9809437 | 99.1 | 1.9809437 | 99.1 | 2.23591666 | 99.1 | 3.72652776 | | | 99.10 | 1.80442397 | 99.1 | 3.99130736 | 99.1 | 1.80442397 |
| 99.2 | 0.74530555 | 99.2 | 1.95152375 | 99.2 | 1.95152375 | 99.2 | 2.22611 | 99.2 | 3.70691446 | | | 99.20 | 1.79461732 | 99.2 | 3.98150071 | 99.2 | 1.79461732 |
| 99.3 | 0.76491886 | 99.3 | 1.97113705 | 99.3 | 1.97113705 | 99.3 | 2.24572331 | 99.3 | 3.6971078 | | | 99.30 | 1.79461732 | 99.3 | 3.97169406 | 99.3 | 1.79461732 |
| 99.4 | 0.77472551 | 99.4 | 2.00055701 | 99.4 | 2.00055701 | 99.4 | 2.24572331 | 99.4 | 3.74614106 | | | 99.40 | 1.82403727 | 99.4 | 3.98150071 | 99.4 | 1.82403727 |
| 99.5 | 0.77472551 | 99.5 | 1.99075036 | 99.5 | 1.99075036 | 99.5 | 2.26533661 | 99.5 | 3.75594772 | | | 99.50 | 1.82403727 | 99.5 | 3.98150071 | 99.5 | 1.82403727 |
| 99.6 | 0.80414546 | 99.6 | 2.00055701 | 99.6 | 2.00055701 | 99.6 | 2.28494992 | 99.6 | 3.74614106 | | | 99.60 | 1.77500401 | 99.6 | 4.00111402 | 99.6 | 1.77500401 |
| 99.7 | 0.79433881 | 99.7 | 1.9809437 | 99.7 | 1.9809437 | 99.7 | 2.27514326 | 99.7 | 3.74614106 | | | 99.70 | 1.80442397 | 99.7 | 3.99130736 | 99.7 | 1.80442397 |
| 99.8 | 0.76491886 | 99.8 | 1.97113705 | 99.8 | 1.97113705 | 99.8 | 2.25552996 | 99.8 | 3.74614106 | | | 99.80 | 1.81423062 | 99.8 | 3.99130736 | 99.8 | 1.81423062 |
| 99.9 | 0.7354989 | 99.9 | 2.00055701 | 99.9 | 2.00055701 | 99.9 | 2.27514326 | 99.9 | 3.76575437 | | | 99.90 | 1.79461732 | 99.9 | 4.02072732 | 99.9 | 1.79461732 |
| 100 | 0.77472551 | 100 | 2.00055701 | 100 | 2.00055701 | 100 | 2.29475657 | 100 | 3.79517432 | | | 100.00 | 1.79461732 | 100 | 4.01092067 | 100 | 1.79461732 |
| 100.1 | 0.7551122 | 100.1 | 2.00055701 | 100.1 | 2.00055701 | 100.1 | 2.28494992 | 100.1 | 3.81478763 | | | 100.10 | 1.80442397 | 100.1 | 4.00111402 | 100.1 | 1.80442397 |
| 100.2 | 0.76491886 | 100.2 | 2.01036366 | 100.2 | 2.01036366 | 100.2 | 2.29475657 | 100.2 | 3.81478763 | | | 100.20 | 1.78481066 | 100.2 | 4.01092067 | 100.2 | 1.78481066 |
| 100.3 | 0.72569225 | 100.3 | 2.02017031 | 100.3 | 2.02017031 | 100.3 | 2.31436987 | 100.3 | 3.81478763 | | | 100.30 | 1.78481066 | 100.3 | 4.02072732 | 100.3 | 1.78481066 |
| 100.4 | 0.72569225 | 100.4 | 2.03978362 | 100.4 | 2.03978362 | 100.4 | 2.31436987 | 100.4 | 3.81478763 | | | 100.40 | 1.79461732 | 100.4 | 4.04034062 | 100.4 | 1.79461732 |
| 100.5 | 0.74530555 | 100.5 | 2.02017031 | 100.5 | 2.02017031 | 100.5 | 2.27514326 | 100.5 | 3.81478763 | | | 100.50 | 1.79461732 | 100.5 | 4.01092067 | 100.5 | 1.79461732 |
| 100.6 | 0.7354989 | 100.6 | 2.01036366 | 100.6 | 2.01036366 | 100.6 | 2.28494992 | 100.6 | 3.82459428 | | | 100.60 | 1.77500401 | 100.6 | 4.04034062 | 100.6 | 1.77500401 |
| 100.7 | 0.7354989 | 100.7 | 2.02017031 | 100.7 | 2.02017031 | 100.7 | 2.29475657 | 100.7 | 3.83440093 | | | 100.70 | 1.78481066 | 100.7 | 4.05995393 | 100.7 | 1.78481066 |
| 100.8 | 0.7354989 | 100.8 | 2.03978362 | 100.8 | 2.03978362 | 100.8 | 2.30456322 | 100.8 | 3.86382089 | | | 100.80 | 1.78481066 | 100.8 | 4.02072732 | 100.8 | 1.78481066 |
| 100.9 | 0.74530555 | 100.9 | 2.05939692 | 100.9 | 2.05939692 | 100.9 | 2.31436987 | 100.9 | 3.87362754 | | | 100.90 | 1.78481066 | 100.9 | 4.03053397 | 100.9 | 1.78481066 |

| | | | | | | | | | | | | | | | | | |
|---|---|---|---|---|---|---|---|---|---|---|---|---|---|---|---|---|---|
| 101 | 0.76491886 | 101 | 2.03978362 | 101 | 2.03978362 | 101 | 2.31436987 | 101 | 3.86382089 | | | 101.00 | 1.79461732 | 101 | 4.06976058 | 101 | 1.79461732 |
| 101.1 | 0.77472551 | 101.1 | 2.03978362 | 101.1 | 2.03978362 | 101.1 | 2.32417652 | 101.1 | 3.88343419 | | | 101.10 | 1.79461732 | 101.1 | 4.06976058 | 101.1 | 1.79461732 |
| 101.2 | 0.76491886 | 101.2 | 2.05939692 | 101.2 | 2.05939692 | 101.2 | 2.31436987 | 101.2 | 3.91285415 | | | 101.20 | 1.76519736 | 101.2 | 4.06976058 | 101.2 | 1.76519736 |
| 101.3 | 0.72569225 | 101.3 | 2.06920357 | 101.3 | 2.06920357 | 101.3 | 2.31436987 | 101.3 | 3.89324084 | | | 101.30 | 1.77500401 | 101.3 | 4.09918054 | 101.3 | 1.77500401 |
| 101.4 | 0.74530555 | 101.4 | 2.05939692 | 101.4 | 2.05939692 | 101.4 | 2.34378983 | 101.4 | 3.87362754 | | | 101.40 | 1.80442397 | 101.4 | 4.09918054 | 101.4 | 1.80442397 |
| 101.5 | 0.7551122 | 101.5 | 2.09862353 | 101.5 | 2.09862353 | 101.5 | 2.34378983 | 101.5 | 3.87362754 | | | 101.50 | 1.81423062 | 101.5 | 4.06976058 | 101.5 | 1.81423062 |
| 101.6 | 0.74530555 | 101.6 | 2.10843018 | 101.6 | 2.10843018 | 101.6 | 2.32417652 | 101.6 | 3.89324084 | | | 101.60 | 1.80442397 | 101.6 | 4.07956723 | 101.6 | 1.80442397 |
| 101.7 | 0.76491886 | 101.7 | 2.08881688 | 101.7 | 2.08881688 | 101.7 | 2.32417652 | 101.7 | 3.93246745 | | | 101.70 | 1.82403727 | 101.7 | 4.11879384 | 101.7 | 1.82403727 |
| 101.8 | 0.77472551 | 101.8 | 2.06920357 | 101.8 | 2.06920357 | 101.8 | 2.33398318 | 101.8 | 3.91285415 | | | 101.80 | 1.81423062 | 101.8 | 4.09918054 | 101.8 | 1.81423062 |
| 101.9 | 0.7551122 | 101.9 | 2.08881688 | 101.9 | 2.08881688 | 101.9 | 2.33398318 | 101.9 | 3.89324084 | | | 101.90 | 1.83384392 | 101.9 | 4.10898719 | 101.9 | 1.83384392 |
| 102 | 0.7551122 | 102 | 2.08881688 | 102 | 2.08881688 | 102 | 2.34378983 | 102 | 3.9226608 | | | 102.00 | 1.83384392 | 102 | 4.13840714 | 102 | 1.83384392 |
| 102.1 | 0.74530555 | 102.1 | 2.06920357 | 102.1 | 2.06920357 | 102.1 | 2.36340313 | 102.1 | 3.93246745 | | | 102.10 | 1.82403727 | 102.1 | 4.15802045 | 102.1 | 1.82403727 |
| 102.2 | 0.76491886 | 102.2 | 2.06920357 | 102.2 | 2.06920357 | 102.2 | 2.37320978 | 102.2 | 3.9422741 | | | 102.20 | 1.79461732 | 102.2 | 4.12860049 | 102.2 | 1.79461732 |
| 102.3 | 0.80414546 | 102.3 | 2.06920357 | 102.3 | 2.06920357 | 102.3 | 2.36340313 | 102.3 | 3.96188741 | | | 102.30 | 1.79461732 | 102.3 | 4.08937388 | 102.3 | 1.79461732 |
| 102.4 | 0.77472551 | 102.4 | 2.08881688 | 102.4 | 2.08881688 | 102.4 | 2.36340313 | 102.4 | 3.97169406 | | | 102.40 | 1.84365058 | 102.4 | 4.12860049 | 102.4 | 1.84365058 |
| 102.5 | 0.74530555 | 102.5 | 2.09862353 | 102.5 | 2.09862353 | 102.5 | 2.35359648 | 102.5 | 3.97169406 | | | 102.50 | 1.86326388 | 102.5 | 4.11879384 | 102.5 | 1.86326388 |
| 102.6 | 0.76491886 | 102.6 | 2.09862353 | 102.6 | 2.09862353 | 102.6 | 2.35359648 | 102.6 | 3.97169406 | | | 102.60 | 1.84365058 | 102.6 | 4.13840714 | 102.6 | 1.84365058 |
| 102.7 | 0.74530555 | 102.7 | 2.11823683 | 102.7 | 2.11823683 | 102.7 | 2.35359648 | 102.7 | 3.95208076 | | | 102.70 | 1.85345723 | 102.7 | 4.1482138 | 102.7 | 1.85345723 |
| 102.8 | 0.7158856 | 102.8 | 2.15746344 | 102.8 | 2.15746344 | 102.8 | 2.40262974 | 102.8 | 3.99130736 | | | 102.80 | 1.84365058 | 102.8 | 4.15802045 | 102.8 | 1.84365058 |
| 102.9 | 0.7354989 | 102.9 | 2.14765679 | 102.9 | 2.14765679 | 102.9 | 2.39282309 | 102.9 | 4.00111402 | | | 102.90 | 1.82403727 | 102.9 | 4.1874404 | 102.9 | 1.82403727 |
| 103 | 0.76491886 | 103 | 2.12804348 | 103 | 2.12804348 | 103 | 2.38301644 | 103 | 3.98150071 | | | 103.00 | 1.83384392 | 103 | 4.1678271 | 103 | 1.83384392 |
| 103.1 | 0.76491886 | 103.1 | 2.12804348 | 103.1 | 2.12804348 | 103.1 | 2.38301644 | 103.1 | 3.98150071 | | | 103.10 | 1.86326388 | 103.1 | 4.13840714 | 103.1 | 1.86326388 |
| 103.2 | 0.7551122 | 103.2 | 2.12804348 | 103.2 | 2.12804348 | 103.2 | 2.38301644 | 103.2 | 3.96188741 | | | 103.20 | 1.83384392 | 103.2 | 4.1482138 | 103.2 | 1.83384392 |
| 103.3 | 0.78453216 | 103.3 | 2.13785014 | 103.3 | 2.13785014 | 103.3 | 2.40262974 | 103.3 | 4.00111402 | | | 103.30 | 1.83384392 | 103.3 | 4.1482138 | 103.3 | 1.83384392 |
| 103.4 | 0.76491886 | 103.4 | 2.12804348 | 103.4 | 2.12804348 | 103.4 | 2.42224304 | 103.4 | 3.97169406 | | | 103.40 | 1.84365058 | 103.4 | 4.13840714 | 103.4 | 1.84365058 |
| 103.5 | 0.77472551 | 103.5 | 2.11823683 | 103.5 | 2.11823683 | 103.5 | 2.39282309 | 103.5 | 3.97169406 | | | 103.50 | 1.87307053 | 103.5 | 4.17763375 | 103.5 | 1.87307053 |
| 103.6 | 0.79433881 | 103.6 | 2.12804348 | 103.6 | 2.12804348 | 103.6 | 2.41243639 | 103.6 | 4.00111402 | | | 103.60 | 1.84365058 | 103.6 | 4.1874404 | 103.6 | 1.84365058 |
| 103.7 | 0.83356542 | 103.7 | 2.14765679 | 103.7 | 2.14765679 | 103.7 | 2.40262974 | 103.7 | 4.01092067 | | | 103.70 | 1.85345723 | 103.7 | 4.20705371 | 103.7 | 1.85345723 |
| 103.8 | 0.80414546 | 103.8 | 2.17707674 | 103.8 | 2.17707674 | 103.8 | 2.40262974 | 103.8 | 4.01092067 | | | 103.80 | 1.83384392 | 103.8 | 4.1874404 | 103.8 | 1.83384392 |
| 103.9 | 0.77472551 | 103.9 | 2.17707674 | 103.9 | 2.17707674 | 103.9 | 2.41243639 | 103.9 | 4.01092067 | | | 103.90 | 1.83384392 | 103.9 | 4.1678271 | 103.9 | 1.83384392 |
| 104 | 0.80414546 | 104 | 2.19669005 | 104 | 2.19669005 | 104 | 2.4320497 | 104 | 4.04034062 | | | 104.00 | 1.85345723 | 104 | 4.20705371 | 104 | 1.85345723 |
| 104.1 | 0.81395212 | 104.1 | 2.16727009 | 104.1 | 2.16727009 | 104.1 | 2.4712763 | 104.1 | 4.01092067 | | | 104.10 | 1.82403727 | 104.1 | 4.22666701 | 104.1 | 1.82403727 |
| 104.2 | 0.79433881 | 104.2 | 2.1868834 | 104.2 | 2.1868834 | 104.2 | 2.4712763 | 104.2 | 3.99130736 | | | 104.20 | 1.85345723 | 104.2 | 4.20705371 | 104.2 | 1.85345723 |
| 104.3 | 0.79433881 | 104.3 | 2.1868834 | 104.3 | 2.1868834 | 104.3 | 2.46146965 | 104.3 | 3.99130736 | | | 104.30 | 1.86326388 | 104.3 | 4.19724706 | 104.3 | 1.86326388 |
| 104.4 | 0.77472551 | 104.4 | 2.16727009 | 104.4 | 2.16727009 | 104.4 | 2.4712763 | 104.4 | 3.99130736 | | | 104.40 | 1.86326388 | 104.4 | 4.17763375 | 104.4 | 1.86326388 |
| 104.5 | 0.7551122 | 104.5 | 2.1868834 | 104.5 | 2.1868834 | 104.5 | 2.4712763 | 104.5 | 4.04034062 | | | 104.50 | 1.87307053 | 104.5 | 4.15802045 | 104.5 | 1.87307053 |
| 104.6 | 0.7551122 | 104.6 | 2.17707674 | 104.6 | 2.17707674 | 104.6 | 2.49088961 | 104.6 | 4.04034062 | | | 104.60 | 1.86326388 | 104.6 | 4.19724706 | 104.6 | 1.86326388 |
| 104.7 | 0.76491886 | 104.7 | 2.17707674 | 104.7 | 2.17707674 | 104.7 | 2.49088961 | 104.7 | 4.03053397 | | | 104.70 | 1.85345723 | 104.7 | 4.23647366 | 104.7 | 1.85345723 |
| 104.8 | 0.80414546 | 104.8 | 2.1868834 | 104.8 | 2.1868834 | 104.8 | 2.50069626 | 104.8 | 4.06976058 | | | 104.80 | 1.90249049 | 104.8 | 4.20705371 | 104.8 | 1.90249049 |
| 104.9 | 0.80414546 | 104.9 | 2.19669005 | 104.9 | 2.19669005 | 104.9 | 2.53992287 | 104.9 | 4.07956723 | | | 104.90 | 1.88287718 | 104.9 | 4.26589362 | 104.9 | 1.88287718 |
| 105 | 0.78453216 | 105 | 2.22611 | 105 | 2.22611 | 105 | 2.53011622 | 105 | 4.03053397 | | | 105.00 | 1.87307053 | 105 | 4.28550692 | 105 | 1.87307053 |
| 105.1 | 0.78453216 | 105.1 | 2.19669005 | 105.1 | 2.19669005 | 105.1 | 2.53992287 | 105.1 | 4.04034062 | | | 105.10 | 1.88287718 | 105.1 | 4.26589362 | 105.1 | 1.88287718 |
| 105.2 | 0.77472551 | 105.2 | 2.2064967 | 105.2 | 2.2064967 | 105.2 | 2.51050291 | 105.2 | 4.08937388 | | | 105.20 | 1.89268384 | 105.2 | 4.23647366 | 105.2 | 1.89268384 |
| 105.3 | 0.80414546 | 105.3 | 2.21630335 | 105.3 | 2.21630335 | 105.3 | 2.53011622 | 105.3 | 4.05995393 | | | 105.30 | 1.88287718 | 105.3 | 4.20705371 | 105.3 | 1.88287718 |
| 105.4 | 0.78453216 | 105.4 | 2.2064967 | 105.4 | 2.2064967 | 105.4 | 2.53992287 | 105.4 | 4.05995393 | | | 105.40 | 1.92210379 | 105.4 | 4.24628032 | 105.4 | 1.92210379 |
| 105.5 | 0.76491886 | 105.5 | 2.2064967 | 105.5 | 2.2064967 | 105.5 | 2.51050291 | 105.5 | 4.08937388 | | | 105.50 | 1.91229714 | 105.5 | 4.21686036 | 105.5 | 1.91229714 |
| 105.6 | 0.77472551 | 105.6 | 2.21630335 | 105.6 | 2.21630335 | 105.6 | 2.53992287 | 105.6 | 4.08937388 | | | 105.60 | 1.91229714 | 105.6 | 4.25608697 | 105.6 | 1.91229714 |
| 105.7 | 0.79433881 | 105.7 | 2.22611 | 105.7 | 2.22611 | 105.7 | 2.53992287 | 105.7 | 4.10898719 | | | 105.70 | 1.92210379 | 105.7 | 4.29531358 | 105.7 | 1.92210379 |
| 105.8 | 0.78453216 | 105.8 | 2.24572331 | 105.8 | 2.24572331 | 105.8 | 2.54972952 | 105.8 | 4.10898719 | | | 105.80 | 1.90249049 | 105.8 | 4.28550692 | 105.8 | 1.90249049 |
| 105.9 | 0.81395212 | 105.9 | 2.26533661 | 105.9 | 2.26533661 | 105.9 | 2.54972952 | 105.9 | 4.10898719 | | | 105.90 | 1.92210379 | 105.9 | 4.28550692 | 105.9 | 1.92210379 |
| 106 | 0.77472551 | 106 | 2.27514326 | 106 | 2.27514326 | 106 | 2.54972952 | 106 | 4.13840714 | | | 106.00 | 1.92210379 | 106 | 4.26589362 | 106 | 1.92210379 |
| 106.1 | 0.74530555 | 106.1 | 2.26533661 | 106.1 | 2.26533661 | 106.1 | 2.57914948 | 106.1 | 4.1482138 | | | 106.10 | 1.9417171 | 106.1 | 4.30512023 | 106.1 | 1.9417171 |
| 106.2 | 0.7551122 | 106.2 | 2.27514326 | 106.2 | 2.27514326 | 106.2 | 2.55953617 | 106.2 | 4.13840714 | | | 106.20 | 1.95152375 | 106.2 | 4.32473353 | 106.2 | 1.95152375 |
| 106.3 | 0.82375877 | 106.3 | 2.27514326 | 106.3 | 2.27514326 | 106.3 | 2.56934282 | 106.3 | 4.1482138 | | | 106.30 | 1.95152375 | 106.3 | 4.31492688 | 106.3 | 1.95152375 |
| 106.4 | 0.83356542 | 106.4 | 2.29475657 | 106.4 | 2.29475657 | 106.4 | 2.58895613 | 106.4 | 4.13840714 | | | 106.40 | 1.93191044 | 106.4 | 4.29531358 | 106.4 | 1.93191044 |
| 106.5 | 0.81395212 | 106.5 | 2.28494992 | 106.5 | 2.28494992 | 106.5 | 2.60856943 | 106.5 | 4.1678271 | | | 106.50 | 1.93191044 | 106.5 | 4.30512023 | 106.5 | 1.93191044 |
| 106.6 | 0.85317872 | 106.6 | 2.28494992 | 106.6 | 2.28494992 | 106.6 | 2.59876278 | 106.6 | 4.1874404 | | | 106.60 | 1.95152375 | 106.6 | 4.30512023 | 106.6 | 1.95152375 |
| 106.7 | 0.85317872 | 106.7 | 2.30456322 | 106.7 | 2.30456322 | 106.7 | 2.58895613 | 106.7 | 4.1874404 | | | 106.70 | 1.95152375 | 106.7 | 4.29531358 | 106.7 | 1.95152375 |
| 106.8 | 0.82375877 | 106.8 | 2.29475657 | 106.8 | 2.29475657 | 106.8 | 2.58895613 | 106.8 | 4.19724706 | | | 106.80 | 1.97113705 | 106.8 | 4.30512023 | 106.8 | 1.97113705 |
| 106.9 | 0.81395212 | 106.9 | 2.31436987 | 106.9 | 2.31436987 | 106.9 | 2.59876278 | 106.9 | 4.1874404 | | | 106.90 | 1.9613304 | 106.9 | 4.32473353 | 106.9 | 1.9613304 |
| 107 | 0.78453216 | 107 | 2.31436987 | 107 | 2.31436987 | 107 | 2.58895613 | 107 | 4.24628032 | | | 107.00 | 1.9809437 | 107 | 4.35415349 | 107 | 1.9809437 |

| | | | | | | | | | | | | | | | | | |
|---|---|---|---|---|---|---|---|---|---|---|---|---|---|---|---|---|---|
| 107.1 | 0.81395212 | 107.1 | 2.31436987 | 107.1 | 2.31436987 | 107.1 | 2.57914948 | 107.1 | 4.23647366 | | | 107.10 | 2.00055701 | 107.1 | 4.30512023 | 107.1 | 2.00055701 |
| 107.2 | 0.79433881 | 107.2 | 2.33398318 | 107.2 | 2.33398318 | 107.2 | 2.57914948 | 107.2 | 4.24628032 | | | 107.20 | 1.9809437 | 107.2 | 4.31492688 | 107.2 | 1.9809437 |
| 107.3 | 0.7551122 | 107.3 | 2.34378983 | 107.3 | 2.34378983 | 107.3 | 2.59876278 | 107.3 | 4.25608697 | | | 107.30 | 1.99075036 | 107.3 | 4.33454018 | 107.3 | 1.99075036 |
| 107.4 | 0.79433881 | 107.4 | 2.32417652 | 107.4 | 2.32417652 | 107.4 | 2.61837608 | 107.4 | 4.22666701 | | | 107.40 | 2.00055701 | 107.4 | 4.34434684 | 107.4 | 2.00055701 |
| 107.5 | 0.83356542 | 107.5 | 2.34378983 | 107.5 | 2.34378983 | 107.5 | 2.59876278 | 107.5 | 4.19724706 | | | 107.50 | 1.97113705 | 107.5 | 4.33454018 | 107.5 | 1.97113705 |
| 107.6 | 0.80414546 | 107.6 | 2.34378983 | 107.6 | 2.34378983 | 107.6 | 2.59876278 | 107.6 | 4.22666701 | | | 107.60 | 1.97113705 | 107.6 | 4.34434684 | 107.6 | 1.97113705 |
| 107.7 | 0.83356542 | 107.7 | 2.33398318 | 107.7 | 2.33398318 | 107.7 | 2.61837608 | 107.7 | 4.25608697 | | | 107.70 | 1.99075036 | 107.7 | 4.31492688 | 107.7 | 1.99075036 |
| 107.8 | 0.80414546 | 107.8 | 2.31436987 | 107.8 | 2.31436987 | 107.8 | 2.63798939 | 107.8 | 4.30512023 | | | 107.80 | 2.00055701 | 107.8 | 4.31492688 | 107.8 | 2.00055701 |
| 107.9 | 0.81395212 | 107.9 | 2.31436987 | 107.9 | 2.31436987 | 107.9 | 2.61837608 | 107.9 | 4.31492688 | | | 107.90 | 2.02997696 | 107.9 | 4.35415349 | 107.9 | 2.02997696 |
| 108 | 0.78453216 | 108 | 2.31436987 | 108 | 2.31436987 | 108 | 2.63798939 | 108 | 4.30512023 | | | 108.00 | 2.01036366 | 108 | 4.37376679 | 108 | 2.01036366 |
| 108.1 | 0.79433881 | 108.1 | 2.33398318 | 108.1 | 2.33398318 | 108.1 | 2.64779604 | 108.1 | 4.29531358 | | | 108.10 | 1.9809437 | 108.1 | 4.3933801 | 108.1 | 1.9809437 |
| 108.2 | 0.79433881 | 108.2 | 2.32417652 | 108.2 | 2.32417652 | 108.2 | 2.64779604 | 108.2 | 4.32473353 | | | 108.20 | 2.02017031 | 108.2 | 4.4129934 | 108.2 | 2.02017031 |
| 108.3 | 0.80414546 | 108.3 | 2.32417652 | 108.3 | 2.32417652 | 108.3 | 2.63798939 | 108.3 | 4.36396014 | | | 108.30 | 2.02017031 | 108.3 | 4.42280005 | 108.3 | 2.02017031 |
| 108.4 | 0.80414546 | 108.4 | 2.32417652 | 108.4 | 2.32417652 | 108.4 | 2.62818274 | 108.4 | 4.36396014 | | | 108.40 | 2.02017031 | 108.4 | 4.4129934 | 108.4 | 2.02017031 |
| 108.5 | 0.82375877 | 108.5 | 2.34378983 | 108.5 | 2.34378983 | 108.5 | 2.63798939 | 108.5 | 4.34434684 | | | 108.50 | 2.02017031 | 108.5 | 4.38357344 | 108.5 | 2.02017031 |
| 108.6 | 0.85317872 | 108.6 | 2.34378983 | 108.6 | 2.34378983 | 108.6 | 2.65760269 | 108.6 | 4.38357344 | | | 108.60 | 2.01036366 | 108.6 | 4.37376679 | 108.6 | 2.01036366 |
| 108.7 | 0.83356542 | 108.7 | 2.33398318 | 108.7 | 2.33398318 | 108.7 | 2.677216 | 108.7 | 4.40318675 | | | 108.70 | 2.00055701 | 108.7 | 4.38357344 | 108.7 | 2.00055701 |
| 108.8 | 0.83356542 | 108.8 | 2.35359648 | 108.8 | 2.35359648 | 108.8 | 2.66740934 | 108.8 | 4.3933801 | | | 108.80 | 2.01036366 | 108.8 | 4.37376679 | 108.8 | 2.01036366 |
| 108.9 | 0.83356542 | 108.9 | 2.35359648 | 108.9 | 2.35359648 | 108.9 | 2.677216 | 108.9 | 4.4129934 | | | 108.90 | 2.02017031 | 108.9 | 4.36396014 | 108.9 | 2.02017031 |
| 109 | 0.81395212 | 109 | 2.36340313 | 109 | 2.36340313 | 109 | 2.677216 | 109 | 4.44241336 | | | 109.00 | 2.05939692 | 109 | 4.3933801 | 109 | 2.05939692 |
| 109.1 | 0.78453216 | 109.1 | 2.35359648 | 109.1 | 2.35359648 | 109.1 | 2.68702265 | 109.1 | 4.44241336 | | | 109.10 | 2.05939692 | 109.1 | 4.4129934 | 109.1 | 2.05939692 |
| 109.2 | 0.79433881 | 109.2 | 2.34378983 | 109.2 | 2.34378983 | 109.2 | 2.6968293 | 109.2 | 4.44241336 | | | 109.20 | 2.04959027 | 109.2 | 4.4129934 | 109.2 | 2.04959027 |
| 109.3 | 0.78453216 | 109.3 | 2.34378983 | 109.3 | 2.34378983 | 109.3 | 2.6968293 | 109.3 | 4.4326067 | | | 109.30 | 2.06920357 | 109.3 | 4.42280005 | 109.3 | 2.06920357 |
| 109.4 | 0.79433881 | 109.4 | 2.35359648 | 109.4 | 2.35359648 | 109.4 | 2.6968293 | 109.4 | 4.4326067 | | | 109.40 | 2.04959027 | 109.4 | 4.4129934 | 109.4 | 2.04959027 |
| 109.5 | 0.81395212 | 109.5 | 2.33398318 | 109.5 | 2.33398318 | 109.5 | 2.6968293 | 109.5 | 4.44241336 | | | 109.50 | 2.02997696 | 109.5 | 4.40318675 | 109.5 | 2.02997696 |
| 109.6 | 0.78453216 | 109.6 | 2.34378983 | 109.6 | 2.34378983 | 109.6 | 2.6968293 | 109.6 | 4.46202666 | | | 109.60 | 2.06920357 | 109.6 | 4.4129934 | 109.6 | 2.06920357 |
| 109.7 | 0.79433881 | 109.7 | 2.35359648 | 109.7 | 2.35359648 | 109.7 | 2.70663595 | 109.7 | 4.48163996 | | | 109.70 | 2.11823683 | 109.7 | 4.47183331 | 109.7 | 2.11823683 |
| 109.8 | 0.83356542 | 109.8 | 2.36340313 | 109.8 | 2.36340313 | 109.8 | 2.70663595 | 109.8 | 4.46202666 | | | 109.80 | 2.07901022 | 109.8 | 4.46202666 | 109.8 | 2.07901022 |
| 109.9 | 0.88259868 | 109.9 | 2.34378983 | 109.9 | 2.34378983 | 109.9 | 2.72624926 | 109.9 | 4.47183331 | | | 109.90 | 2.04959027 | 109.9 | 4.46202666 | 109.9 | 2.04959027 |
| 110 | 0.85317872 | 110 | 2.35359648 | 110 | 2.35359648 | 110 | 2.73605591 | 110 | 4.52086657 | | | 110.00 | 2.06920357 | 110 | 4.48163996 | 110 | 2.06920357 |
| 110.1 | 0.84337207 | 110.1 | 2.35359648 | 110.1 | 2.35359648 | 110.1 | 2.74586256 | 110.1 | 4.52086657 | | | 110.10 | 2.08881688 | 110.1 | 4.47183331 | 110.1 | 2.08881688 |
| 110.2 | 0.82375877 | 110.2 | 2.36340313 | 110.2 | 2.36340313 | 110.2 | 2.75566921 | 110.2 | 4.50125327 | | | 110.20 | 2.11823683 | 110.2 | 4.47183331 | 110.2 | 2.11823683 |
| 110.3 | 0.83356542 | 110.3 | 2.39282309 | 110.3 | 2.39282309 | 110.3 | 2.76547586 | 110.3 | 4.51105992 | | | 110.30 | 2.09862353 | 110.3 | 4.46202666 | 110.3 | 2.09862353 |
| 110.4 | 0.83356542 | 110.4 | 2.40262974 | 110.4 | 2.40262974 | 110.4 | 2.76547586 | 110.4 | 4.51105992 | | | 110.40 | 2.06920357 | 110.4 | 4.45222001 | 110.4 | 2.06920357 |
| 110.5 | 0.80414546 | 110.5 | 2.41243639 | 110.5 | 2.41243639 | 110.5 | 2.75566921 | 110.5 | 4.55028653 | | | 110.50 | 2.07901022 | 110.5 | 4.50125327 | 110.5 | 2.07901022 |
| 110.6 | 0.81395212 | 110.6 | 2.44185635 | 110.6 | 2.44185635 | 110.6 | 2.74586256 | 110.6 | 4.55028653 | | | 110.60 | 2.09862353 | 110.6 | 4.51105992 | 110.6 | 2.09862353 |
| 110.7 | 0.83356542 | 110.7 | 2.44185635 | 110.7 | 2.44185635 | 110.7 | 2.75566921 | 110.7 | 4.56009318 | | | 110.70 | 2.09862353 | 110.7 | 4.49144662 | 110.7 | 2.09862353 |
| 110.8 | 0.80414546 | 110.8 | 2.46146965 | 110.8 | 2.46146965 | 110.8 | 2.78508917 | 110.8 | 4.56989983 | | | 110.80 | 2.10843018 | 110.8 | 4.50125327 | 110.8 | 2.10843018 |
| 110.9 | 0.80414546 | 110.9 | 2.451663 | 110.9 | 2.451663 | 110.9 | 2.77528252 | 110.9 | 4.56989983 | | | 110.90 | 2.10843018 | 110.9 | 4.50125327 | 110.9 | 2.10843018 |
| 111 | 0.84337207 | 111 | 2.451663 | 111 | 2.451663 | 111 | 2.75566921 | 111 | 4.57970648 | | | 111.00 | 2.08881688 | 111 | 4.48163996 | 111 | 2.08881688 |
| 111.1 | 0.84337207 | 111.1 | 2.44185635 | 111.1 | 2.44185635 | 111.1 | 2.77528252 | 111.1 | 4.59931979 | | | 111.10 | 2.12804348 | 111.1 | 4.49144662 | 111.1 | 2.12804348 |
| 111.2 | 0.82375877 | 111.2 | 2.48108296 | 111.2 | 2.48108296 | 111.2 | 2.77528252 | 111.2 | 4.58951314 | | | 111.20 | 2.14765679 | 111.2 | 4.48163996 | 111.2 | 2.14765679 |
| 111.3 | 0.81395212 | 111.3 | 2.49088961 | 111.3 | 2.49088961 | 111.3 | 2.77528252 | 111.3 | 4.60912644 | | | 111.30 | 2.15746344 | 111.3 | 4.48163996 | 111.3 | 2.15746344 |
| 111.4 | 0.82375877 | 111.4 | 2.51050291 | 111.4 | 2.51050291 | 111.4 | 2.78508917 | 111.4 | 4.58951314 | | | 111.40 | 2.15746344 | 111.4 | 4.49144662 | 111.4 | 2.15746344 |
| 111.5 | 0.83356542 | 111.5 | 2.52030956 | 111.5 | 2.52030956 | 111.5 | 2.79489582 | 111.5 | 4.59931979 | | | 111.50 | 2.11823683 | 111.5 | 4.50125327 | 111.5 | 2.11823683 |
| 111.6 | 0.80414546 | 111.6 | 2.50069626 | 111.6 | 2.50069626 | 111.6 | 2.80470247 | 111.6 | 4.62873974 | | | 111.60 | 2.11823683 | 111.6 | 4.53067322 | 111.6 | 2.11823683 |
| 111.7 | 0.80414546 | 111.7 | 2.51050291 | 111.7 | 2.51050291 | 111.7 | 2.80470247 | 111.7 | 4.64835305 | | | 111.70 | 2.14765679 | 111.7 | 4.52086657 | 111.7 | 2.14765679 |
| 111.8 | 0.82375877 | 111.8 | 2.52030956 | 111.8 | 2.52030956 | 111.8 | 2.80470247 | 111.8 | 4.64835305 | | | 111.80 | 2.14765679 | 111.8 | 4.54047988 | 111.8 | 2.14765679 |
| 111.9 | 0.81395212 | 111.9 | 2.49088961 | 111.9 | 2.49088961 | 111.9 | 2.80470247 | 111.9 | 4.6385464 | | | 111.90 | 2.17707674 | 111.9 | 4.54047988 | 111.9 | 2.17707674 |
| 112 | 0.83356542 | 112 | 2.48108296 | 112 | 2.48108296 | 112 | 2.82431578 | 112 | 4.62873974 | | | 112.00 | 2.16727009 | 112 | 4.55028653 | 112 | 2.16727009 |
| 112.1 | 0.85317872 | 112.1 | 2.50069626 | 112.1 | 2.50069626 | 112.1 | 2.82431578 | 112.1 | 4.677773 | | | 112.10 | 2.14765679 | 112.1 | 4.55028653 | 112.1 | 2.14765679 |
| 112.2 | 0.81395212 | 112.2 | 2.52030956 | 112.2 | 2.52030956 | 112.2 | 2.79489582 | 112.2 | 4.677773 | | | 112.20 | 2.16727009 | 112.2 | 4.56009318 | 112.2 | 2.16727009 |
| 112.3 | 0.82375877 | 112.3 | 2.52030956 | 112.3 | 2.52030956 | 112.3 | 2.80470247 | 112.3 | 4.68757966 | | | 112.30 | 2.15746344 | 112.3 | 4.56009318 | 112.3 | 2.15746344 |
| 112.4 | 0.81395212 | 112.4 | 2.51050291 | 112.4 | 2.51050291 | 112.4 | 2.82431578 | 112.4 | 4.69738631 | | | 112.40 | 2.16727009 | 112.4 | 4.56989983 | 112.4 | 2.16727009 |
| 112.5 | 0.78453216 | 112.5 | 2.51050291 | 112.5 | 2.51050291 | 112.5 | 2.84392908 | 112.5 | 4.72680626 | | | 112.50 | 2.16727009 | 112.5 | 4.53067322 | 112.5 | 2.16727009 |
| 112.6 | 0.81395212 | 112.6 | 2.53992287 | 112.6 | 2.53992287 | 112.6 | 2.84392908 | 112.6 | 4.73661292 | | | 112.60 | 2.17707674 | 112.6 | 4.53067322 | 112.6 | 2.17707674 |
| 112.7 | 0.84337207 | 112.7 | 2.53011622 | 112.7 | 2.53011622 | 112.7 | 2.84392908 | 112.7 | 4.74641957 | | | 112.70 | 2.2064967 | 112.7 | 4.60912644 | 112.7 | 2.2064967 |
| 112.8 | 0.84337207 | 112.8 | 2.53011622 | 112.8 | 2.53011622 | 112.8 | 2.85373573 | 112.8 | 4.69738631 | | | 112.80 | 2.21630335 | 112.8 | 4.57970648 | 112.8 | 2.21630335 |
| 112.9 | 0.83356542 | 112.9 | 2.52030956 | 112.9 | 2.52030956 | 112.9 | 2.83412243 | 112.9 | 4.68757966 | | | 112.90 | 2.21630335 | 112.9 | 4.58951314 | 112.9 | 2.21630335 |
| 113 | 0.84337207 | 113 | 2.52030956 | 113 | 2.52030956 | 113 | 2.83412243 | 113 | 4.73661292 | | | 113.00 | 2.2064967 | 113 | 4.57970648 | 113 | 2.2064967 |
| 113.1 | 0.84337207 | 113.1 | 2.53992287 | 113.1 | 2.53992287 | 113.1 | 2.84392908 | 113.1 | 4.73661292 | | | 113.10 | 2.2064967 | 113.1 | 4.58951314 | 113.1 | 2.2064967 |

| | | | | | | | | | | | | | | | | | |
|---|---|---|---|---|---|---|---|---|---|---|---|---|---|---|---|---|---|
| 113.2 | 0.82375877 | 113.2 | 2.53011622 | 113.2 | 2.53011622 | 113.2 | 2.83412243 | 113.2 | 4.72680626 | | | 113.20 | 2.19669005 | 113.2 | 4.58951314 | 113.2 | 2.19669005 |
| 113.3 | 0.82375877 | 113.3 | 2.53011622 | 113.3 | 2.53011622 | 113.3 | 2.84392908 | 113.3 | 4.72680626 | | | 113.30 | 2.1868834 | 113.3 | 4.58951314 | 113.3 | 2.1868834 |
| 113.4 | 0.79433881 | 113.4 | 2.53992287 | 113.4 | 2.53992287 | 113.4 | 2.87334904 | 113.4 | 4.72680626 | | | 113.40 | 2.2064967 | 113.4 | 4.58951314 | 113.4 | 2.2064967 |
| 113.5 | 0.82375877 | 113.5 | 2.53992287 | 113.5 | 2.53992287 | 113.5 | 2.86354238 | 113.5 | 4.75622622 | | | 113.50 | 2.21630335 | 113.5 | 4.55028653 | 113.5 | 2.21630335 |
| 113.6 | 0.82375877 | 113.6 | 2.55953617 | 113.6 | 2.55953617 | 113.6 | 2.86354238 | 113.6 | 4.76603287 | | | 113.60 | 2.21630335 | 113.6 | 4.54047988 | 113.6 | 2.21630335 |
| 113.7 | 0.81395212 | 113.7 | 2.55953617 | 113.7 | 2.55953617 | 113.7 | 2.85373573 | 113.7 | 4.80525948 | | | 113.70 | 2.21630335 | 113.7 | 4.60912644 | 113.7 | 2.21630335 |
| 113.8 | 0.81395212 | 113.8 | 2.56934282 | 113.8 | 2.56934282 | 113.8 | 2.87334904 | 113.8 | 4.82487278 | | | 113.80 | 2.22611 | 113.8 | 4.64835305 | 113.8 | 2.22611 |
| 113.9 | 0.84337207 | 113.9 | 2.56934282 | 113.9 | 2.56934282 | 113.9 | 2.87334904 | 113.9 | 4.77583952 | | | 113.90 | 2.22611 | 113.9 | 4.61893309 | 113.9 | 2.22611 |
| 114 | 0.84337207 | 114 | 2.58895613 | 114 | 2.58895613 | 114 | 2.89296234 | 114 | 4.84448609 | | | 114.00 | 2.23591666 | 114 | 4.59931979 | 114 | 2.23591666 |
| 114.1 | 0.83356542 | 114.1 | 2.56934282 | 114.1 | 2.56934282 | 114.1 | 2.91257564 | 114.1 | 4.82487278 | | | 114.10 | 2.25552996 | 114.1 | 4.59931979 | 114.1 | 2.25552996 |
| 114.2 | 0.83356542 | 114.2 | 2.56934282 | 114.2 | 2.56934282 | 114.2 | 2.90276899 | 114.2 | 4.83467944 | | | 114.20 | 2.23591666 | 114.2 | 4.6581597 | 114.2 | 2.23591666 |
| 114.3 | 0.83356542 | 114.3 | 2.58895613 | 114.3 | 2.58895613 | 114.3 | 2.89296234 | 114.3 | 4.8837127 | | | 114.30 | 2.25552996 | 114.3 | 4.64835305 | 114.3 | 2.25552996 |
| 114.4 | 0.82375877 | 114.4 | 2.58895613 | 114.4 | 2.58895613 | 114.4 | 2.89296234 | 114.4 | 4.903326 | | | 114.40 | 2.26533661 | 114.4 | 4.70719296 | 114.4 | 2.26533661 |
| 114.5 | 0.82375877 | 114.5 | 2.58895613 | 114.5 | 2.58895613 | 114.5 | 2.89296234 | 114.5 | 4.903326 | | | 114.50 | 2.28494992 | 114.5 | 4.68757966 | 114.5 | 2.28494992 |
| 114.6 | 0.82375877 | 114.6 | 2.58895613 | 114.6 | 2.58895613 | 114.6 | 2.90276899 | 114.6 | 4.903326 | | | 114.60 | 2.27514326 | 114.6 | 4.68757966 | 114.6 | 2.27514326 |
| 114.7 | 0.83356542 | 114.7 | 2.62818274 | 114.7 | 2.62818274 | 114.7 | 2.9223823 | 114.7 | 4.8837127 | | | 114.70 | 2.31436987 | 114.7 | 4.677773 | 114.7 | 2.31436987 |
| 114.8 | 0.82375877 | 114.8 | 2.62818274 | 114.8 | 2.62818274 | 114.8 | 2.93218895 | 114.8 | 4.86409939 | | | 114.80 | 2.27514326 | 114.8 | 4.66796635 | 114.8 | 2.27514326 |
| 114.9 | 0.81395212 | 114.9 | 2.62818274 | 114.9 | 2.62818274 | 114.9 | 2.9419956 | 114.9 | 4.93274596 | | | 114.90 | 2.28494992 | 114.9 | 4.6581597 | 114.9 | 2.28494992 |
| 115 | 0.82375877 | 115 | 2.61837608 | 115 | 2.61837608 | 115 | 2.9419956 | 115 | 4.96216591 | | | 115.00 | 2.29475657 | 115 | 4.66796635 | 115 | 2.29475657 |
| 115.1 | 0.82375877 | 115.1 | 2.62818274 | 115.1 | 2.62818274 | 115.1 | 2.9419956 | 115.1 | 4.93274596 | | | 115.10 | 2.29475657 | 115.1 | 4.66796635 | 115.1 | 2.29475657 |
| 115.2 | 0.83356542 | 115.2 | 2.61837608 | 115.2 | 2.61837608 | 115.2 | 2.93218895 | 115.2 | 4.94255261 | | | 115.20 | 2.29475657 | 115.2 | 4.68757966 | 115.2 | 2.29475657 |
| 115.3 | 0.87279203 | 115.3 | 2.59876278 | 115.3 | 2.59876278 | 115.3 | 2.9419956 | 115.3 | 4.95235926 | | | 115.30 | 2.28494992 | 115.3 | 4.69738631 | 115.3 | 2.28494992 |
| 115.4 | 0.90221198 | 115.4 | 2.60856943 | 115.4 | 2.60856943 | 115.4 | 2.9616089 | 115.4 | 4.94255261 | | | 115.40 | 2.27514326 | 115.4 | 4.70719296 | 115.4 | 2.27514326 |
| 115.5 | 0.89240533 | 115.5 | 2.61837608 | 115.5 | 2.61837608 | 115.5 | 2.9616089 | 115.5 | 4.97197256 | | | 115.50 | 2.29475657 | 115.5 | 4.73661292 | 115.5 | 2.29475657 |
| 115.6 | 0.88259868 | 115.6 | 2.63798939 | 115.6 | 2.63798939 | 115.6 | 2.97141556 | 115.6 | 4.95235926 | | | 115.60 | 2.31436987 | 115.6 | 4.70719296 | 115.6 | 2.31436987 |
| 115.7 | 0.87279203 | 115.7 | 2.63798939 | 115.7 | 2.63798939 | 115.7 | 2.95180225 | 115.7 | 4.95235926 | | | 115.70 | 2.30456322 | 115.7 | 4.69738631 | 115.7 | 2.30456322 |
| 115.8 | 0.81395212 | 115.8 | 2.64779604 | 115.8 | 2.64779604 | 115.8 | 2.9616089 | 115.8 | 4.97197256 | | | 115.80 | 2.31436987 | 115.8 | 4.74641957 | 115.8 | 2.31436987 |
| 115.9 | 0.84337207 | 115.9 | 2.677216 | 115.9 | 2.677216 | 115.9 | 2.9616089 | 115.9 | 4.98177922 | | | 115.90 | 2.33398318 | 115.9 | 4.75622622 | 115.9 | 2.33398318 |
| 116 | 0.89240533 | 116 | 2.68702265 | 116 | 2.68702265 | 116 | 2.97141556 | 116 | 4.99158587 | | | 116.00 | 2.34378983 | 116 | 4.71699961 | 116 | 2.34378983 |
| 116.1 | 0.87279203 | 116.1 | 2.65760269 | 116.1 | 2.65760269 | 116.1 | 2.97141556 | 116.1 | 4.98177922 | | | 116.10 | 2.33398318 | 116.1 | 4.72680626 | 116.1 | 2.33398318 |
| 116.2 | 0.84337207 | 116.2 | 2.677216 | 116.2 | 2.677216 | 116.2 | 2.98122221 | 116.2 | 5.01119917 | | | 116.20 | 2.34378983 | 116.2 | 4.71699961 | 116.2 | 2.34378983 |
| 116.3 | 0.84337207 | 116.3 | 2.6968293 | 116.3 | 2.6968293 | 116.3 | 2.99102886 | 116.3 | 5.02100582 | | | 116.30 | 2.33398318 | 116.3 | 4.72680626 | 116.3 | 2.33398318 |
| 116.4 | 0.86298538 | 116.4 | 2.70663595 | 116.4 | 2.70663595 | 116.4 | 2.98122221 | 116.4 | 5.07003908 | | | 116.40 | 2.35359648 | 116.4 | 4.70719296 | 116.4 | 2.35359648 |
| 116.5 | 0.87279203 | 116.5 | 2.70663595 | 116.5 | 2.70663595 | 116.5 | 2.98122221 | 116.5 | 5.09945904 | | | 116.50 | 2.35359648 | 116.5 | 4.76603287 | 116.5 | 2.35359648 |
| 116.6 | 0.85317872 | 116.6 | 2.6968293 | 116.6 | 2.6968293 | 116.6 | 2.98122221 | 116.6 | 5.09945904 | | | 116.60 | 2.35359648 | 116.6 | 4.76603287 | 116.6 | 2.35359648 |
| 116.7 | 0.88259868 | 116.7 | 2.6968293 | 116.7 | 2.6968293 | 116.7 | 2.99102886 | 116.7 | 5.09945904 | | | 116.70 | 2.34378983 | 116.7 | 4.78564618 | 116.7 | 2.34378983 |
| 116.8 | 0.86298538 | 116.8 | 2.70663595 | 116.8 | 2.70663595 | 116.8 | 3.00083551 | 116.8 | 5.10926569 | | | 116.80 | 2.35359648 | 116.8 | 4.77583952 | 116.8 | 2.35359648 |
| 116.9 | 0.86298538 | 116.9 | 2.70663595 | 116.9 | 2.70663595 | 116.9 | 2.99102886 | 116.9 | 5.09945904 | | | 116.90 | 2.36340313 | 116.9 | 4.81506613 | 116.9 | 2.36340313 |
| 117 | 0.87279203 | 117 | 2.6968293 | 117 | 2.6968293 | 117 | 2.99102886 | 117 | 5.11907234 | | | 117.00 | 2.39282309 | 117 | 4.80525948 | 117 | 2.39282309 |
| 117.1 | 0.86298538 | 117.1 | 2.72624926 | 117.1 | 2.72624926 | 117.1 | 2.99102886 | 117.1 | 5.10926569 | | | 117.10 | 2.39282309 | 117.1 | 4.79545283 | 117.1 | 2.39282309 |
| 117.2 | 0.85317872 | 117.2 | 2.7164426 | 117.2 | 2.7164426 | 117.2 | 2.99102886 | 117.2 | 5.08965239 | | | 117.20 | 2.40262974 | 117.2 | 4.82487278 | 117.2 | 2.40262974 |
| 117.3 | 0.82375877 | 117.3 | 2.6968293 | 117.3 | 2.6968293 | 117.3 | 3.00083551 | 117.3 | 5.10926569 | | | 117.30 | 2.41243639 | 117.3 | 4.81506613 | 117.3 | 2.41243639 |
| 117.4 | 0.81395212 | 117.4 | 2.677216 | 117.4 | 2.677216 | 117.4 | 3.01064216 | 117.4 | 5.10926569 | | | 117.40 | 2.40262974 | 117.4 | 4.77583952 | 117.4 | 2.40262974 |
| 117.5 | 0.84337207 | 117.5 | 2.65760269 | 117.5 | 2.65760269 | 117.5 | 3.00083551 | 117.5 | 5.1484923 | | | 117.50 | 2.39282309 | 117.5 | 4.77583952 | 117.5 | 2.39282309 |
| 117.6 | 0.88259868 | 117.6 | 2.62818274 | 117.6 | 2.62818274 | 117.6 | 3.00083551 | 117.6 | 5.15829895 | | | 117.60 | 2.39282309 | 117.6 | 4.80525948 | 117.6 | 2.39282309 |
| 117.7 | 0.86298538 | 117.7 | 2.62818274 | 117.7 | 2.62818274 | 117.7 | 3.03025547 | 117.7 | 5.1681056 | | | 117.70 | 2.38301644 | 117.7 | 4.84448609 | 117.7 | 2.38301644 |
| 117.8 | 0.84337207 | 117.8 | 2.64779604 | 117.8 | 2.64779604 | 117.8 | 3.02044882 | 117.8 | 5.1681056 | | | 117.80 | 2.37320978 | 117.8 | 4.83467944 | 117.8 | 2.37320978 |
| 117.9 | 0.88259868 | 117.9 | 2.6968293 | 117.9 | 2.6968293 | 117.9 | 3.03025547 | 117.9 | 5.17791226 | | | 117.90 | 2.40262974 | 117.9 | 4.80525948 | 117.9 | 2.40262974 |
| 118 | 0.88259868 | 118 | 2.70663595 | 118 | 2.70663595 | 118 | 3.04986877 | 118 | 5.20733221 | | | 118.00 | 2.40262974 | 118 | 4.80525948 | 118 | 2.40262974 |
| 118.1 | 0.87279203 | 118.1 | 2.68702265 | 118.1 | 2.68702265 | 118.1 | 3.04986877 | 118.1 | 5.20733221 | | | 118.10 | 2.42224304 | 118.1 | 4.81506613 | 118.1 | 2.42224304 |
| 118.2 | 0.88259868 | 118.2 | 2.64779604 | 118.2 | 2.64779604 | 118.2 | 3.04006212 | 118.2 | 5.21713886 | | | 118.20 | 2.4320497 | 118.2 | 4.83467944 | 118.2 | 2.4320497 |
| 118.3 | 0.82375877 | 118.3 | 2.68702265 | 118.3 | 2.68702265 | 118.3 | 3.06948208 | 118.3 | 5.21713886 | | | 118.30 | 2.4320497 | 118.3 | 4.82487278 | 118.3 | 2.4320497 |
| 118.4 | 0.81395212 | 118.4 | 2.7164426 | 118.4 | 2.7164426 | 118.4 | 3.07928873 | 118.4 | 5.20733221 | | | 118.40 | 2.42224304 | 118.4 | 4.83467944 | 118.4 | 2.42224304 |
| 118.5 | 0.87279203 | 118.5 | 2.58895613 | 118.5 | 2.58895613 | 118.5 | 3.06948208 | 118.5 | 5.19752556 | | | 118.50 | 2.451663 | 118.5 | 4.83467944 | 118.5 | 2.451663 |
| 118.6 | 0.86298538 | 118.6 | 2.51050291 | 118.6 | 2.51050291 | 118.6 | 3.05967542 | 118.6 | 5.20733221 | | | 118.60 | 2.451663 | 118.6 | 4.85429274 | 118.6 | 2.451663 |
| 118.7 | 0.88259868 | 118.7 | 2.51050291 | 118.7 | 2.51050291 | 118.7 | 3.05967542 | 118.7 | 5.23675217 | | | 118.70 | 2.44185635 | 118.7 | 4.8837127 | 118.7 | 2.44185635 |
| 118.8 | 0.87279203 | 118.8 | 2.53011622 | 118.8 | 2.53011622 | 118.8 | 3.07928873 | 118.8 | 5.27597878 | | | 118.80 | 2.451663 | 118.8 | 4.89351935 | 118.8 | 2.451663 |
| 118.9 | 0.89240533 | 118.9 | 2.54972952 | 118.9 | 2.54972952 | 118.9 | 3.08909538 | 118.9 | 5.28578543 | | | 118.90 | 2.48108296 | 118.9 | 4.89351935 | 118.9 | 2.48108296 |
| 119 | 0.89240533 | 119 | 2.58895613 | 119 | 2.58895613 | 119 | 3.09890203 | 119 | 5.29559208 | | | 119.00 | 2.46146965 | 119 | 4.87390604 | 119 | 2.46146965 |
| 119.1 | 0.86298538 | 119.1 | 2.60856943 | 119.1 | 2.60856943 | 119.1 | 3.07928873 | 119.1 | 5.29559208 | | | 119.10 | 2.451663 | 119.1 | 4.86409939 | 119.1 | 2.451663 |
| 119.2 | 0.86298538 | 119.2 | 2.60856943 | 119.2 | 2.60856943 | 119.2 | 3.09890203 | 119.2 | 5.26617212 | | | 119.20 | 2.46146965 | 119.2 | 4.903326 | 119.2 | 2.46146965 |

| | | | | | | | | | | | | | | | | | |
|---|---|---|---|---|---|---|---|---|---|---|---|---|---|---|---|---|---|
| 119.3 | 0.84337207 | 119.3 | 2.59876278 | 119.3 | 2.59876278 | 119.3 | 3.08909538 | 119.3 | 5.27597878 | | | 119.30 | 2.4712763 | 119.3 | 4.903326 | 119.3 | 2.4712763 |
| 119.4 | 0.88259868 | 119.4 | 2.57914948 | 119.4 | 2.57914948 | 119.4 | 3.07928873 | 119.4 | 5.27597878 | | | 119.40 | 2.50069626 | 119.4 | 4.91313265 | 119.4 | 2.50069626 |
| 119.5 | 0.87279203 | 119.5 | 2.58895613 | 119.5 | 2.58895613 | 119.5 | 3.10870868 | 119.5 | 5.27597878 | | | 119.50 | 2.50069626 | 119.5 | 4.93274596 | 119.5 | 2.50069626 |
| 119.6 | 0.89240533 | 119.6 | 2.58895613 | 119.6 | 2.58895613 | 119.6 | 3.10870868 | 119.6 | 5.27597878 | | | 119.60 | 2.48108296 | 119.6 | 4.95235926 | 119.6 | 2.48108296 |
| 119.7 | 0.87279203 | 119.7 | 2.57914948 | 119.7 | 2.57914948 | 119.7 | 3.10870868 | 119.7 | 5.28578543 | | | 119.70 | 2.48108296 | 119.7 | 4.95235926 | 119.7 | 2.48108296 |
| 119.8 | 0.88259868 | 119.8 | 2.58895613 | 119.8 | 2.58895613 | 119.8 | 3.12832199 | 119.8 | 5.32501204 | | | 119.80 | 2.51050291 | 119.8 | 4.93274596 | 119.8 | 2.51050291 |
| 119.9 | 0.89240533 | 119.9 | 2.58895613 | 119.9 | 2.58895613 | 119.9 | 3.10870868 | 119.9 | 5.29559208 | | | 119.90 | 2.50069626 | 119.9 | 4.9229393 | 119.9 | 2.50069626 |
| 120 | 0.91201864 | 120 | 2.56934282 | 120 | 2.56934282 | 120 | 3.10870868 | 120 | 5.32501204 | | | 120.00 | 2.50069626 | 120 | 4.94255261 | 120 | 2.50069626 |
| 120.1 | 0.90221198 | 120.1 | 2.58895613 | 120.1 | 2.58895613 | 120.1 | 3.12832199 | 120.1 | 5.35443199 | | | 120.10 | 2.51050291 | 120.1 | 4.9229393 | 120.1 | 2.51050291 |
| 120.2 | 0.88259868 | 120.2 | 2.63798939 | 120.2 | 2.63798939 | 120.2 | 3.12832199 | 120.2 | 5.35443199 | | | 120.20 | 2.53011622 | 120.2 | 4.95235926 | 120.2 | 2.53011622 |
| 120.3 | 0.85317872 | 120.3 | 2.64779604 | 120.3 | 2.64779604 | 120.3 | 3.12832199 | 120.3 | 5.36423864 | | | 120.30 | 2.53011622 | 120.3 | 4.95235926 | 120.3 | 2.53011622 |
| 120.4 | 0.87279203 | 120.4 | 2.66740934 | 120.4 | 2.66740934 | 120.4 | 3.1675486 | 120.4 | 5.36423864 | | | 120.40 | 2.53992287 | 120.4 | 4.94255261 | 120.4 | 2.53992287 |
| 120.5 | 0.88259868 | 120.5 | 2.6968293 | 120.5 | 2.6968293 | 120.5 | 3.1675486 | 120.5 | 5.3936586 | | | 120.50 | 2.53011622 | 120.5 | 4.98177922 | 120.5 | 2.53011622 |
| 120.6 | 0.86298538 | 120.6 | 2.68702265 | 120.6 | 2.68702265 | 120.6 | 3.1675486 | 120.6 | 5.40346525 | | | 120.60 | 2.52030956 | 120.6 | 5.00139252 | 120.6 | 2.52030956 |
| 120.7 | 0.85317872 | 120.7 | 2.68702265 | 120.7 | 2.68702265 | 120.7 | 3.15774194 | 120.7 | 5.43288521 | | | 120.70 | 2.53011622 | 120.7 | 4.99158587 | 120.7 | 2.53011622 |
| 120.8 | 0.87279203 | 120.8 | 2.7164426 | 120.8 | 2.7164426 | 120.8 | 3.1675486 | 120.8 | 5.40346525 | | | 120.80 | 2.53992287 | 120.8 | 4.99158587 | 120.8 | 2.53992287 |
| 120.9 | 0.89240533 | 120.9 | 2.73605591 | 120.9 | 2.73605591 | 120.9 | 3.1675486 | 120.9 | 5.42307856 | | | 120.90 | 2.54972952 | 120.9 | 4.97197256 | 120.9 | 2.54972952 |
| 121 | 0.88259868 | 121 | 2.7164426 | 121 | 2.7164426 | 121 | 3.14793529 | 121 | 5.46230516 | | | 121.00 | 2.53992287 | 121 | 4.95235926 | 121 | 2.53992287 |
| 121.1 | 0.86298538 | 121.1 | 2.7164426 | 121.1 | 2.7164426 | 121.1 | 3.13812864 | 121.1 | 5.45249851 | | | 121.10 | 2.54972952 | 121.1 | 4.97197256 | 121.1 | 2.54972952 |
| 121.2 | 0.88259868 | 121.2 | 2.73605591 | 121.2 | 2.73605591 | 121.2 | 3.15774194 | 121.2 | 5.47211182 | | | 121.20 | 2.55953617 | 121.2 | 5.01119917 | 121.2 | 2.55953617 |
| 121.3 | 0.88259868 | 121.3 | 2.75566921 | 121.3 | 2.75566921 | 121.3 | 3.1675486 | 121.3 | 5.47211182 | | | 121.30 | 2.56934282 | 121.3 | 5.05042578 | 121.3 | 2.56934282 |
| 121.4 | 0.90221198 | 121.4 | 2.74586256 | 121.4 | 2.74586256 | 121.4 | 3.1871619 | 121.4 | 5.46230516 | | | 121.40 | 2.56934282 | 121.4 | 5.07003908 | 121.4 | 2.56934282 |
| 121.5 | 0.90221198 | 121.5 | 2.72624926 | 121.5 | 2.72624926 | 121.5 | 3.19696855 | 121.5 | 5.49172512 | | | 121.50 | 2.58895613 | 121.5 | 5.03081248 | 121.5 | 2.58895613 |
| 121.6 | 0.88259868 | 121.6 | 2.72624926 | 121.6 | 2.72624926 | 121.6 | 3.19696855 | 121.6 | 5.51133842 | | | 121.60 | 2.60856943 | 121.6 | 5.01119917 | 121.6 | 2.60856943 |
| 121.7 | 0.89240533 | 121.7 | 2.74586256 | 121.7 | 2.74586256 | 121.7 | 3.2067752 | 121.7 | 5.51133842 | | | 121.70 | 2.59876278 | 121.7 | 5.02100582 | 121.7 | 2.59876278 |
| 121.8 | 0.92182529 | 121.8 | 2.73605591 | 121.8 | 2.73605591 | 121.8 | 3.19696855 | 121.8 | 5.55056503 | | | 121.80 | 2.56934282 | 121.8 | 5.02100582 | 121.8 | 2.56934282 |
| 121.9 | 0.90221198 | 121.9 | 2.75566921 | 121.9 | 2.75566921 | 121.9 | 3.22638851 | 121.9 | 5.56037168 | | | 121.90 | 2.56934282 | 121.9 | 5.06023243 | 121.9 | 2.56934282 |
| 122 | 0.88259868 | 122 | 2.77528252 | 122 | 2.77528252 | 122 | 3.21658186 | 122 | 5.55056503 | | | 122.00 | 2.57914948 | 122 | 5.06023243 | 122 | 2.57914948 |
| 122.1 | 0.90221198 | 122.1 | 2.78508917 | 122.1 | 2.78508917 | 122.1 | 3.21658186 | 122.1 | 5.57017834 | | | 122.10 | 2.59876278 | 122.1 | 5.06023243 | 122.1 | 2.59876278 |
| 122.2 | 0.89240533 | 122.2 | 2.78508917 | 122.2 | 2.78508917 | 122.2 | 3.19696855 | 122.2 | 5.62901825 | | | 122.20 | 2.60856943 | 122.2 | 5.06023243 | 122.2 | 2.60856943 |
| 122.3 | 0.91201864 | 122.3 | 2.78508917 | 122.3 | 2.78508917 | 122.3 | 3.19696855 | 122.3 | 5.62901825 | | | 122.30 | 2.61837608 | 122.3 | 5.07984574 | 122.3 | 2.61837608 |
| 122.4 | 0.90221198 | 122.4 | 2.79489582 | 122.4 | 2.79489582 | 122.4 | 3.2067752 | 122.4 | 5.6388249 | | | 122.40 | 2.61837608 | 122.4 | 5.06023243 | 122.4 | 2.61837608 |
| 122.5 | 0.87279203 | 122.5 | 2.79489582 | 122.5 | 2.79489582 | 122.5 | 3.19696855 | 122.5 | 5.6388249 | | | 122.50 | 2.63798939 | 122.5 | 5.08965239 | 122.5 | 2.63798939 |
| 122.6 | 0.87279203 | 122.6 | 2.78508917 | 122.6 | 2.78508917 | 122.6 | 3.22638851 | 122.6 | 5.64863155 | | | 122.60 | 2.65760269 | 122.6 | 5.07003908 | 122.6 | 2.65760269 |
| 122.7 | 0.87279203 | 122.7 | 2.79489582 | 122.7 | 2.79489582 | 122.7 | 3.25580846 | 122.7 | 5.67805151 | | | 122.70 | 2.64779604 | 122.7 | 5.128879 | 122.7 | 2.64779604 |
| 122.8 | 0.87279203 | 122.8 | 2.80470247 | 122.8 | 2.80470247 | 122.8 | 3.22638851 | 122.8 | 5.67805151 | | | 122.80 | 2.66740934 | 122.8 | 5.13868565 | 122.8 | 2.66740934 |
| 122.9 | 0.88259868 | 122.9 | 2.80470247 | 122.9 | 2.80470247 | 122.9 | 3.22638851 | 122.9 | 5.68785816 | | | 122.90 | 2.66740934 | 122.9 | 5.10926569 | 122.9 | 2.66740934 |
| 123 | 0.89240533 | 123 | 2.82431578 | 123 | 2.82431578 | 123 | 3.23619516 | 123 | 5.66824486 | | | 123.00 | 2.64779604 | 123 | 5.08965239 | 123 | 2.64779604 |
| 123.1 | 0.86298538 | 123.1 | 2.83412243 | 123.1 | 2.83412243 | 123.1 | 3.22638851 | 123.1 | 5.68785816 | | | 123.10 | 2.64779604 | 123.1 | 5.11907234 | 123.1 | 2.64779604 |
| 123.2 | 0.91201864 | 123.2 | 2.83412243 | 123.2 | 2.83412243 | 123.2 | 3.22638851 | 123.2 | 5.71727812 | | | 123.20 | 2.63798939 | 123.2 | 5.11907234 | 123.2 | 2.63798939 |
| 123.3 | 0.93163194 | 123.3 | 2.86354238 | 123.3 | 2.86354238 | 123.3 | 3.24600181 | 123.3 | 5.74669807 | | | 123.30 | 2.63798939 | 123.3 | 5.128879 | 123.3 | 2.63798939 |
| 123.4 | 0.91201864 | 123.4 | 2.86354238 | 123.4 | 2.86354238 | 123.4 | 3.25580846 | 123.4 | 5.75650472 | | | 123.40 | 2.63798939 | 123.4 | 5.128879 | 123.4 | 2.63798939 |
| 123.5 | 0.94143859 | 123.5 | 2.86354238 | 123.5 | 2.86354238 | 123.5 | 3.25580846 | 123.5 | 5.75650472 | | | 123.50 | 2.65760269 | 123.5 | 5.1484923 | 123.5 | 2.65760269 |
| 123.6 | 0.89240533 | 123.6 | 2.87334904 | 123.6 | 2.87334904 | 123.6 | 3.24600181 | 123.6 | 5.79573133 | | | 123.60 | 2.66740934 | 123.6 | 5.128879 | 123.6 | 2.66740934 |
| 123.7 | 0.91201864 | 123.7 | 2.88315569 | 123.7 | 2.88315569 | 123.7 | 3.23619516 | 123.7 | 5.82515129 | | | 123.70 | 2.677216 | 123.7 | 5.128879 | 123.7 | 2.677216 |
| 123.8 | 0.93163194 | 123.8 | 2.89296234 | 123.8 | 2.89296234 | 123.8 | 3.24600181 | 123.8 | 5.84476459 | | | 123.80 | 2.6968293 | 123.8 | 5.15829895 | 123.8 | 2.6968293 |
| 123.9 | 0.94143859 | 123.9 | 2.90276899 | 123.9 | 2.90276899 | 123.9 | 3.26561512 | 123.9 | 5.83495794 | | | 123.90 | 2.6968293 | 123.9 | 5.17791226 | 123.9 | 2.6968293 |
| 124 | 0.91201864 | 124 | 2.90276899 | 124 | 2.90276899 | 124 | 3.27542177 | 124 | 5.80553798 | | | 124.00 | 2.68702265 | 124 | 5.19752556 | 124 | 2.68702265 |
| 124.1 | 0.90221198 | 124.1 | 2.89296234 | 124.1 | 2.89296234 | 124.1 | 3.28522842 | 124.1 | 5.82515129 | | | 124.10 | 2.698533 | 124.1 | 5.19752556 | 124.1 | #VALUE! |
| 124.2 | 0.93163194 | 124.2 | 2.90276899 | 124.2 | 2.90276899 | 124.2 | 3.28522842 | 124.2 | 5.8643779 | | | 124.20 | 2.70663595 | 124.2 | 5.20733221 | 124.2 | 2.70663595 |
| 124.3 | 0.92182529 | 124.3 | 2.90276899 | 124.3 | 2.90276899 | 124.3 | 3.29503507 | 124.3 | 5.87418455 | | | 124.30 | 2.7164426 | 124.3 | 5.19752556 | 124.3 | 2.7164426 |
| 124.4 | 0.91201864 | 124.4 | 2.91257564 | 124.4 | 2.91257564 | 124.4 | 3.26561512 | 124.4 | 5.89379785 | | | 124.40 | 2.70663595 | 124.4 | 5.21713886 | 124.4 | 2.70663595 |
| 124.5 | 0.91201864 | 124.5 | 2.9419956 | 124.5 | 2.9419956 | 124.5 | 3.26561512 | 124.5 | 5.94283111 | | | 124.50 | 2.6968293 | 124.5 | 5.18771891 | 124.5 | 2.6968293 |
| 124.6 | 0.94143859 | 124.6 | 2.93218895 | 124.6 | 2.93218895 | 124.6 | 3.29503507 | 124.6 | 5.94283111 | | | 124.60 | 2.7164426 | 124.6 | 5.18771891 | 124.6 | 2.7164426 |
| 124.7 | 0.93163194 | 124.7 | 2.95180225 | 124.7 | 2.95180225 | 124.7 | 3.29503507 | 124.7 | 5.94283111 | | | 124.70 | 2.72624926 | 124.7 | 5.19752556 | 124.7 | 2.72624926 |
| 124.8 | 0.92182529 | 124.8 | 2.9419956 | 124.8 | 2.9419956 | 124.8 | 3.29503507 | 124.8 | 5.96244442 | | | 124.80 | 2.7164426 | 124.8 | 5.19752556 | 124.8 | 2.7164426 |
| 124.9 | 0.92182529 | 124.9 | 2.95180225 | 124.9 | 2.95180225 | 124.9 | 3.30484172 | 124.9 | 5.98205772 | | | 124.90 | 2.72624926 | 124.9 | 5.20733221 | 124.9 | 2.72624926 |
| 125 | 0.89240533 | 125 | 2.9616089 | 125 | 2.9616089 | 125 | 3.32445503 | 125 | 6.01147768 | | | 125.00 | 2.72624926 | 125 | 5.22694552 | 125 | 2.72624926 |
| 125.1 | 0.88259868 | 125.1 | 2.9419956 | 125.1 | 2.9419956 | 125.1 | 3.31464838 | 125.1 | 6.04089763 | | | 125.10 | 2.73605591 | 125.1 | 5.24655882 | 125.1 | 2.73605591 |
| 125.2 | 0.91201864 | 125.2 | 2.9419956 | 125.2 | 2.9419956 | 125.2 | 3.31464838 | 125.2 | 6.02128433 | | | 125.20 | 2.74586256 | 125.2 | 5.25636547 | 125.2 | 2.74586256 |
| 125.3 | 0.93163194 | 125.3 | 2.9419956 | 125.3 | 2.9419956 | 125.3 | 3.31464838 | 125.3 | 6.03109098 | | | 125.30 | 2.74586256 | 125.3 | 5.24655882 | 125.3 | 2.74586256 |

| | | | | | | | | | | | | | | | | | |
|---|---|---|---|---|---|---|---|---|---|---|---|---|---|---|---|---|---|
| 125.4 | 0.95124524 | 125.4 | 2.98122221 | 125.4 | 2.98122221 | 125.4 | 3.32445503 | 125.4 | 6.05070428 | | | 125.40 | 2.75566921 | 125.4 | 5.22694552 | 125.4 | 2.75566921 |
| 125.5 | 0.93163194 | 125.5 | 3.01064216 | 125.5 | 3.01064216 | 125.5 | 3.34406833 | 125.5 | 6.07031759 | | | 125.50 | 2.77528252 | 125.5 | 5.23675217 | 125.5 | 2.77528252 |
| 125.6 | 0.91201864 | 125.6 | 2.99102886 | 125.6 | 2.99102886 | 125.6 | 3.33426168 | 125.6 | 6.07031759 | | | 125.60 | 2.78508917 | 125.6 | 5.25636547 | 125.6 | 2.78508917 |
| 125.7 | 0.91201864 | 125.7 | 3.00083551 | 125.7 | 3.00083551 | 125.7 | 3.33426168 | 125.7 | 6.08993089 | | | 125.70 | 2.78508917 | 125.7 | 5.26617212 | 125.7 | 2.78508917 |
| 125.8 | 0.93163194 | 125.8 | 3.02044882 | 125.8 | 3.02044882 | 125.8 | 3.35387498 | 125.8 | 6.11935085 | | | 125.80 | 2.78508917 | 125.8 | 5.28578543 | 125.8 | 2.78508917 |
| 125.9 | 0.93163194 | 125.9 | 3.02044882 | 125.9 | 3.02044882 | 125.9 | 3.36368164 | 125.9 | 6.13896415 | | | 125.90 | 2.75566921 | 125.9 | 5.31520538 | 125.9 | 2.75566921 |
| 126 | 0.92182529 | 126 | 3.04006212 | 126 | 3.04006212 | 126 | 3.35387498 | 126 | 6.1487708 | | | 126.00 | 2.77528252 | 126 | 5.29559208 | 126 | 2.77528252 |
| 126.1 | 0.90221198 | 126.1 | 3.05967542 | 126.1 | 3.05967542 | 126.1 | 3.35387498 | 126.1 | 6.15857746 | | | 126.10 | 2.77528252 | 126.1 | 5.30539873 | 126.1 | 2.77528252 |
| 126.2 | 0.87279203 | 126.2 | 3.07928873 | 126.2 | 3.07928873 | 126.2 | 3.36368164 | 126.2 | 6.15857746 | | | 126.20 | 2.78508917 | 126.2 | 5.30539873 | 126.2 | 2.78508917 |
| 126.3 | 0.90221198 | 126.3 | 3.05967542 | 126.3 | 3.05967542 | 126.3 | 3.37348829 | 126.3 | 6.13896415 | | | 126.30 | 2.79489582 | 126.3 | 5.28578543 | 126.3 | 2.79489582 |
| 126.4 | 0.93163194 | 126.4 | 3.05967542 | 126.4 | 3.05967542 | 126.4 | 3.34406833 | 126.4 | 6.1291575 | | | 126.40 | 2.80470247 | 126.4 | 5.30539873 | 126.4 | 2.80470247 |
| 126.5 | 0.93163194 | 126.5 | 3.07928873 | 126.5 | 3.07928873 | 126.5 | 3.35387498 | 126.5 | 6.1487708 | | | 126.50 | 2.81450912 | 126.5 | 5.32501204 | 126.5 | 2.81450912 |
| 126.6 | 0.91201864 | 126.6 | 3.08909538 | 126.6 | 3.08909538 | 126.6 | 3.37348829 | 126.6 | 6.1487708 | | | 126.60 | 2.82431578 | 126.6 | 5.29559208 | 126.6 | 2.82431578 |
| 126.7 | 0.92182529 | 126.7 | 3.09890203 | 126.7 | 3.09890203 | 126.7 | 3.38329494 | 126.7 | 6.15857746 | | | 126.70 | 2.81450912 | 126.7 | 5.28578543 | 126.7 | 2.81450912 |
| 126.8 | 0.92182529 | 126.8 | 3.11851534 | 126.8 | 3.11851534 | 126.8 | 3.38329494 | 126.8 | 6.19780406 | | | 126.80 | 2.81450912 | 126.8 | 5.30539873 | 126.8 | 2.81450912 |
| 126.9 | 0.92182529 | 126.9 | 3.12832199 | 126.9 | 3.12832199 | 126.9 | 3.35387498 | 126.9 | 6.20761072 | | | 126.90 | 2.81450912 | 126.9 | 5.32501204 | 126.9 | 2.81450912 |
| 127 | 0.94143859 | 127 | 3.13812864 | 127 | 3.13812864 | 127 | 3.36368164 | 127 | 6.23703067 | | | 127.00 | 2.83412243 | 127 | 5.32501204 | 127 | 2.83412243 |
| 127.1 | 0.97085855 | 127.1 | 3.11851534 | 127.1 | 3.11851534 | 127.1 | 3.40290824 | 127.1 | 6.22722402 | | | 127.10 | 2.83412243 | 127.1 | 5.33481869 | 127.1 | 2.83412243 |
| 127.2 | 0.94143859 | 127.2 | 3.13812864 | 127.2 | 3.13812864 | 127.2 | 3.40290824 | 127.2 | 6.25664398 | | | 127.20 | 2.82431578 | 127.2 | 5.3740453 | 127.2 | 2.82431578 |
| 127.3 | 0.94143859 | 127.3 | 3.15774194 | 127.3 | 3.15774194 | 127.3 | 3.39310159 | 127.3 | 6.27625728 | | | 127.30 | 2.83412243 | 127.3 | 5.38385195 | 127.3 | 2.83412243 |
| 127.4 | 0.94143859 | 127.4 | 3.14793529 | 127.4 | 3.14793529 | 127.4 | 3.40290824 | 127.4 | 6.27625728 | | | 127.40 | 2.84392908 | 127.4 | 5.40346525 | 127.4 | 2.84392908 |
| 127.5 | 0.94143859 | 127.5 | 3.14793529 | 127.5 | 3.14793529 | 127.5 | 3.4127149 | 127.5 | 6.29587058 | | | 127.50 | 2.84392908 | 127.5 | 5.36423864 | 127.5 | 2.84392908 |
| 127.6 | 0.94143859 | 127.6 | 3.15774194 | 127.6 | 3.15774194 | 127.6 | 3.39310159 | 127.6 | 6.27625728 | | | 127.60 | 2.85373573 | 127.6 | 5.40346525 | 127.6 | 2.85373573 |
| 127.7 | 0.9610519 | 127.7 | 3.15774194 | 127.7 | 3.15774194 | 127.7 | 3.39310159 | 127.7 | 6.30567724 | | | 127.70 | 2.87334904 | 127.7 | 5.40346525 | 127.7 | 2.87334904 |
| 127.8 | 0.9806652 | 127.8 | 3.14793529 | 127.8 | 3.14793529 | 127.8 | 3.4127149 | 127.8 | 6.32529054 | | | 127.80 | 2.87334904 | 127.8 | 5.40346525 | 127.8 | 2.87334904 |
| 127.9 | 0.9610519 | 127.9 | 3.13812864 | 127.9 | 3.13812864 | 127.9 | 3.4127149 | 127.9 | 6.33509719 | | | 127.90 | 2.87334904 | 127.9 | 5.4132719 | 127.9 | 2.87334904 |
| 128 | 0.9610519 | 128 | 3.17735525 | 128 | 3.17735525 | 128 | 3.42252155 | 128 | 6.34490384 | | | 128.00 | 2.87334904 | 128 | 5.44269186 | 128 | 2.87334904 |
| 128.1 | 0.95124524 | 128.1 | 3.1675486 | 128.1 | 3.1675486 | 128.1 | 3.42252155 | 128.1 | 6.38413045 | | | 128.10 | 2.87334904 | 128.1 | 5.44269186 | 128.1 | 2.87334904 |
| 128.2 | 0.9610519 | 128.2 | 3.14793529 | 128.2 | 3.14793529 | 128.2 | 3.42252155 | 128.2 | 6.40374376 | | | 128.20 | 2.88315569 | 128.2 | 5.42307856 | 128.2 | 2.88315569 |
| 128.3 | 0.9610519 | 128.3 | 3.13812864 | 128.3 | 3.13812864 | 128.3 | 3.4323282 | 128.3 | 6.41355041 | | | 128.30 | 2.88315569 | 128.3 | 5.4132719 | 128.3 | 2.88315569 |
| 128.4 | 0.91201864 | 128.4 | 3.1675486 | 128.4 | 3.1675486 | 128.4 | 3.42252155 | 128.4 | 6.42335706 | | | 128.40 | 2.89296234 | 128.4 | 5.42307856 | 128.4 | 2.89296234 |
| 128.5 | 0.88259868 | 128.5 | 3.2067752 | 128.5 | 3.2067752 | 128.5 | 3.42252155 | 128.5 | 6.42335706 | | | 128.50 | 2.89296234 | 128.5 | 5.42307856 | 128.5 | 2.89296234 |
| 128.6 | 0.89240533 | 128.6 | 3.1871619 | 128.6 | 3.1871619 | 128.6 | 3.4323282 | 128.6 | 6.40374376 | | | 128.60 | 2.90276899 | 128.6 | 5.42307856 | 128.6 | 2.90276899 |
| 128.7 | 0.93163194 | 128.7 | 3.17735525 | 128.7 | 3.17735525 | 128.7 | 3.4323282 | 128.7 | 6.41355041 | | | 128.70 | 2.90276899 | 128.7 | 5.47211182 | 128.7 | 2.90276899 |
| 128.8 | 0.9610519 | 128.8 | 3.21658186 | 128.8 | 3.21658186 | 128.8 | 3.4519415 | 128.8 | 6.42335706 | | | 128.80 | 2.90276899 | 128.8 | 5.47211182 | 128.8 | 2.90276899 |
| 128.9 | 0.93163194 | 128.9 | 3.21658186 | 128.9 | 3.21658186 | 128.9 | 3.46174816 | 128.9 | 6.44297036 | | | 128.90 | 2.9223823 | 128.9 | 5.46230516 | 128.9 | 2.9223823 |
| 129 | 0.94143859 | 129 | 3.2067752 | 129 | 3.2067752 | 129 | 3.46174816 | 129 | 6.46258367 | | | 129.00 | 2.91257564 | 129 | 5.47211182 | 129 | 2.91257564 |
| 129.1 | 0.93163194 | 129.1 | 3.2067752 | 129.1 | 3.2067752 | 129.1 | 3.48136146 | 129.1 | 6.45277702 | | | 129.10 | 2.9223823 | 129.1 | 5.50153177 | 129.1 | 2.9223823 |
| 129.2 | 0.92182529 | 129.2 | 3.2067752 | 129.2 | 3.2067752 | 129.2 | 3.46174816 | 129.2 | 6.48219697 | | | 129.20 | 2.9419956 | 129.2 | 5.49172512 | 129.2 | 2.9419956 |
| 129.3 | 0.97085855 | 129.3 | 3.22638851 | 129.3 | 3.22638851 | 129.3 | 3.4519415 | 129.3 | 6.50181028 | | | 129.30 | 2.9616089 | 129.3 | 5.50153177 | 129.3 | 2.9616089 |
| 129.4 | 0.93163194 | 129.4 | 3.2067752 | 129.4 | 3.2067752 | 129.4 | 3.47155481 | 129.4 | 6.53123023 | | | 129.40 | 2.9419956 | 129.4 | 5.52114508 | 129.4 | 2.9419956 |
| 129.5 | 0.92182529 | 129.5 | 3.22638851 | 129.5 | 3.22638851 | 129.5 | 3.47155481 | 129.5 | 6.55084354 | | | 129.50 | 2.95180225 | 129.5 | 5.51133842 | 129.5 | 2.95180225 |
| 129.6 | 0.97085855 | 129.6 | 3.23619516 | 129.6 | 3.23619516 | 129.6 | 3.46174816 | 129.6 | 6.54103688 | | | 129.60 | 2.97141556 | 129.6 | 5.51133842 | 129.6 | 2.97141556 |
| 129.7 | 0.92182529 | 129.7 | 3.23619516 | 129.7 | 3.23619516 | 129.7 | 3.48136146 | 129.7 | 6.52142358 | | | 129.70 | 2.9616089 | 129.7 | 5.50153177 | 129.7 | 2.9616089 |
| 129.8 | 0.91201864 | 129.8 | 3.22638851 | 129.8 | 3.22638851 | 129.8 | 3.48136146 | 129.8 | 6.54103688 | | | 129.80 | 2.97141556 | 129.8 | 5.56037168 | 129.8 | 2.97141556 |
| 129.9 | 0.9806652 | 129.9 | 3.24600181 | 129.9 | 3.24600181 | 129.9 | 3.50097476 | 129.9 | 6.5998768 | | | 129.90 | 2.9616089 | 129.9 | 5.55056503 | 129.9 | 2.9616089 |
| 130 | 0.9806652 | 130 | 3.24600181 | 130 | 3.24600181 | 130 | 3.51078142 | 130 | 6.60968345 | | | 130.00 | 2.97141556 | 130 | 5.52114508 | 130 | 2.97141556 |
| 130.1 | 0.97085855 | 130.1 | 3.25580846 | 130.1 | 3.25580846 | 130.1 | 3.49116811 | 130.1 | 6.6194901 | | | 130.10 | 2.97141556 | 130.1 | 5.52114508 | 130.1 | 2.97141556 |
| 130.2 | 0.9806652 | 130.2 | 3.26561512 | 130.2 | 3.26561512 | 130.2 | 3.51078142 | 130.2 | 6.60968345 | | | 130.20 | 2.98122221 | 130.2 | 5.56037168 | 130.2 | 2.98122221 |
| 130.3 | 0.97085855 | 130.3 | 3.28522842 | 130.3 | 3.28522842 | 130.3 | 3.51078142 | 130.3 | 6.62929675 | | | 130.30 | 2.97141556 | 130.3 | 5.57998499 | 130.3 | 2.97141556 |
| 130.4 | 0.95124524 | 130.4 | 3.26561512 | 130.4 | 3.26561512 | 130.4 | 3.52058807 | 130.4 | 6.65871671 | | | 130.40 | 2.98122221 | 130.4 | 5.57998499 | 130.4 | 2.98122221 |
| 130.5 | 0.9610519 | 130.5 | 3.27542177 | 130.5 | 3.27542177 | 130.5 | 3.53039472 | 130.5 | 6.65871671 | | | 130.50 | 2.99102886 | 130.5 | 5.56037168 | 130.5 | 2.99102886 |
| 130.6 | 0.97085855 | 130.6 | 3.28522842 | 130.6 | 3.28522842 | 130.6 | 3.55981468 | 130.6 | 6.66852336 | | | 130.60 | 2.98122221 | 130.6 | 5.57998499 | 130.6 | 2.98122221 |
| 130.7 | 0.97085855 | 130.7 | 3.27542177 | 130.7 | 3.27542177 | 130.7 | 3.54020137 | 130.7 | 6.66852336 | | | 130.70 | 3.01064216 | 130.7 | 5.62901825 | 130.7 | 3.01064216 |
| 130.8 | 0.9610519 | 130.8 | 3.27542177 | 130.8 | 3.27542177 | 130.8 | 3.49116811 | 130.8 | 6.68813666 | | | 130.80 | 3.01064216 | 130.8 | 5.62901825 | 130.8 | 3.01064216 |
| 130.9 | 0.94143859 | 130.9 | 3.28522842 | 130.9 | 3.28522842 | 130.9 | 3.51078142 | 130.9 | 6.71755662 | | | 130.90 | 3.00083551 | 130.9 | 5.59959829 | 130.9 | 3.00083551 |
| 131 | 0.93163194 | 131 | 3.31464838 | 131 | 3.31464838 | 131 | 3.51078142 | 131 | 6.69794332 | | | 131.00 | 3.02044882 | 131 | 5.60940494 | 131 | 3.02044882 |
| 131.1 | 0.9610519 | 131.1 | 3.33426168 | 131.1 | 3.33426168 | 131.1 | 3.51078142 | 131.1 | 6.68813666 | | | 131.10 | 3.02044882 | 131.1 | 5.60940494 | 131.1 | 3.02044882 |
| 131.2 | 0.94143859 | 131.2 | 3.34406833 | 131.2 | 3.34406833 | 131.2 | 3.51078142 | 131.2 | 6.72736327 | | | 131.20 | 3.02044882 | 131.2 | 5.60940494 | 131.2 | 3.02044882 |
| 131.3 | 0.94143859 | 131.3 | 3.34406833 | 131.3 | 3.34406833 | 131.3 | 3.54020137 | 131.3 | 6.75678323 | | | 131.30 | 3.02044882 | 131.3 | 5.59959829 | 131.3 | 3.02044882 |
| 131.4 | 0.94143859 | 131.4 | 3.32445503 | 131.4 | 3.32445503 | 131.4 | 3.55000802 | 131.4 | 6.77639653 | | | 131.40 | 3.03025547 | 131.4 | 5.62901825 | 131.4 | 3.03025547 |

| | | | | | | | | | | | | | | | | | |
|---|---|---|---|---|---|---|---|---|---|---|---|---|---|---|---|---|---|
| 131.5 | 0.94143859 | 131.5 | 3.32445503 | 131.5 | 3.32445503 | 131.5 | 3.53039472 | 131.5 | 6.75678323 | | | 131.50 | 3.04986877 | 131.5 | 5.64863155 | 131.5 | 3.04986877 |
| 131.6 | 0.9610519 | 131.6 | 3.35387498 | 131.6 | 3.35387498 | 131.6 | 3.55000802 | 131.6 | 6.79600984 | | | 131.60 | 3.04986877 | 131.6 | 5.64863155 | 131.6 | 3.04986877 |
| 131.7 | 0.95124524 | 131.7 | 3.37348829 | 131.7 | 3.37348829 | 131.7 | 3.55981468 | 131.7 | 6.78620318 | | | 131.70 | 3.05967542 | 131.7 | 5.60940494 | 131.7 | 3.05967542 |
| 131.8 | 0.9610519 | 131.8 | 3.37348829 | 131.8 | 3.37348829 | 131.8 | 3.54020137 | 131.8 | 6.79600984 | | | 131.80 | 3.05967542 | 131.8 | 5.6192116 | 131.8 | 3.05967542 |
| 131.9 | 0.90221198 | 131.9 | 3.35387498 | 131.9 | 3.35387498 | 131.9 | 3.55000802 | 131.9 | 6.80581649 | | | 131.90 | 3.05967542 | 131.9 | 5.6388249 | 131.9 | 3.05967542 |
| 132 | 0.87279203 | 132 | 3.39310159 | 132 | 3.39310159 | 132 | 3.57942798 | 132 | 6.80581649 | | | 132.00 | 3.04986877 | 132 | 5.68785816 | 132 | 3.04986877 |
| 132.1 | 0.87279203 | 132.1 | 3.38329494 | 132.1 | 3.38329494 | 132.1 | 3.55981468 | 132.1 | 6.81562314 | | | 132.10 | 3.04986877 | 132.1 | 5.70747146 | 132.1 | 3.04986877 |
| 132.2 | 0.82375877 | 132.2 | 3.37348829 | 132.2 | 3.37348829 | 132.2 | 3.55981468 | 132.2 | 6.83523644 | | | 132.20 | 3.05967542 | 132.2 | 5.68785816 | 132.2 | 3.05967542 |
| 132.3 | 0.83356542 | 132.3 | 3.38329494 | 132.3 | 3.38329494 | 132.3 | 3.55981468 | 132.3 | 6.85484975 | | | 132.30 | 3.06948208 | 132.3 | 5.72708477 | 132.3 | 3.06948208 |
| 132.4 | 0.86298538 | 132.4 | 3.39310159 | 132.4 | 3.39310159 | 132.4 | 3.57942798 | 132.4 | 6.8646564 | | | 132.40 | 3.08909538 | 132.4 | 5.71727812 | 132.4 | 3.08909538 |
| 132.5 | 0.83356542 | 132.5 | 3.40290824 | 132.5 | 3.40290824 | 132.5 | 3.57942798 | 132.5 | 6.85484975 | | | 132.50 | 3.09890203 | 132.5 | 5.72708477 | 132.5 | 3.09890203 |
| 132.6 | 0.83356542 | 132.6 | 3.4127149 | 132.6 | 3.4127149 | 132.6 | 3.58923463 | 132.6 | 6.85484975 | | | 132.60 | 3.09890203 | 132.6 | 5.73689142 | 132.6 | 3.09890203 |
| 132.7 | 0.84337207 | 132.7 | 3.44213485 | 132.7 | 3.44213485 | 132.7 | 3.59904128 | 132.7 | 6.87446305 | | | 132.70 | 3.08909538 | 132.7 | 5.69766481 | 132.7 | 3.08909538 |
| 132.8 | 0.86298538 | 132.8 | 3.4519415 | 132.8 | 3.4519415 | 132.8 | 3.61865459 | 132.8 | 6.90388301 | | | 132.80 | 3.08909538 | 132.8 | 5.70747146 | 132.8 | 3.08909538 |
| 132.9 | 0.85317872 | 132.9 | 3.46174816 | 132.9 | 3.46174816 | 132.9 | 3.58923463 | 132.9 | 6.91368966 | | | 132.90 | 3.09890203 | 132.9 | 5.73689142 | 132.9 | 3.09890203 |
| 133 | 0.84337207 | 133 | 3.44213485 | 133 | 3.44213485 | 133 | 3.56962133 | 133 | 6.93330296 | | | 133.00 | 3.09890203 | 133 | 5.72708477 | 133 | 3.09890203 |
| 133.1 | 0.84337207 | 133.1 | 3.4519415 | 133.1 | 3.4519415 | 133.1 | 3.59904128 | 133.1 | 6.98233622 | | | 133.10 | 3.10870868 | 133.1 | 5.75650472 | 133.1 | 3.10870868 |
| 133.2 | 0.87279203 | 133.2 | 3.46174816 | 133.2 | 3.46174816 | 133.2 | 3.60884794 | 133.2 | 7.00194953 | | | 133.20 | 3.11851534 | 133.2 | 5.76631138 | 133.2 | 3.11851534 |
| 133.3 | 0.85317872 | 133.3 | 3.47155481 | 133.3 | 3.47155481 | 133.3 | 3.59904128 | 133.3 | 6.98233622 | | | 133.30 | 3.12832199 | 133.3 | 5.77611803 | 133.3 | 3.12832199 |
| 133.4 | 0.85317872 | 133.4 | 3.48136146 | 133.4 | 3.48136146 | 133.4 | 3.58923463 | 133.4 | 6.98233622 | | | 133.40 | 3.11851534 | 133.4 | 5.79573133 | 133.4 | 3.11851534 |
| 133.5 | 0.85317872 | 133.5 | 3.49116811 | 133.5 | 3.49116811 | 133.5 | 3.60884794 | 133.5 | 7.00194953 | | | 133.50 | 3.12832199 | 133.5 | 5.79573133 | 133.5 | 3.12832199 |
| 133.6 | 0.88259868 | 133.6 | 3.48136146 | 133.6 | 3.48136146 | 133.6 | 3.60884794 | 133.6 | 7.03136948 | | | 133.60 | 3.12832199 | 133.6 | 5.81534464 | 133.6 | 3.12832199 |
| 133.7 | 0.85317872 | 133.7 | 3.48136146 | 133.7 | 3.48136146 | 133.7 | 3.59904128 | 133.7 | 7.05098279 | | | 133.70 | 3.13812864 | 133.7 | 5.80553798 | 133.7 | 3.13812864 |
| 133.8 | 0.85317872 | 133.8 | 3.47155481 | 133.8 | 3.47155481 | 133.8 | 3.59904128 | 133.8 | 7.04117614 | | | 133.80 | 3.12832199 | 133.8 | 5.78592468 | 133.8 | 3.12832199 |
| 133.9 | 0.83356542 | 133.9 | 3.51078142 | 133.9 | 3.51078142 | 133.9 | 3.60884794 | 133.9 | 7.06078944 | | | 133.90 | 3.14793529 | 133.9 | 5.79573133 | 133.9 | 3.14793529 |
| 134 | 0.82375877 | 134 | 3.51078142 | 134 | 3.51078142 | 134 | 3.61865459 | 134 | 7.07059609 | | | 134.00 | 3.1675486 | 134 | 5.79573133 | 134 | 3.1675486 |
| 134.1 | 0.84337207 | 134.1 | 3.54020137 | 134.1 | 3.54020137 | 134.1 | 3.59904128 | 134.1 | 7.08040274 | | | 134.10 | 3.1675486 | 134.1 | 5.80553798 | 134.1 | 3.1675486 |
| 134.2 | 0.86298538 | 134.2 | 3.55981468 | 134.2 | 3.55981468 | 134.2 | 3.60884794 | 134.2 | 7.08040274 | | | 134.20 | 3.15774194 | 134.2 | 5.83495794 | 134.2 | 3.15774194 |
| 134.3 | 0.85317872 | 134.3 | 3.53039472 | 134.3 | 3.53039472 | 134.3 | 3.63826789 | 134.3 | 7.0902094 | | | 134.30 | 3.14793529 | 134.3 | 5.85457124 | 134.3 | 3.14793529 |
| 134.4 | 0.86298538 | 134.4 | 3.50097476 | 134.4 | 3.50097476 | 134.4 | 3.63826789 | 134.4 | 7.10001605 | | | 134.40 | 3.1871619 | 134.4 | 5.84476459 | 134.4 | 3.1871619 |
| 134.5 | 0.84337207 | 134.5 | 3.53039472 | 134.5 | 3.53039472 | 134.5 | 3.61865459 | 134.5 | 7.1098227 | | | 134.50 | 3.1871619 | 134.5 | 5.82515129 | 134.5 | 3.1871619 |
| 134.6 | 0.82375877 | 134.6 | 3.55000802 | 134.6 | 3.55000802 | 134.6 | 3.6578812 | 134.6 | 7.11962935 | | | 134.60 | 3.17735525 | 134.6 | 5.82515129 | 134.6 | 3.17735525 |
| 134.7 | 0.81395212 | 134.7 | 3.55000802 | 134.7 | 3.55000802 | 134.7 | 3.6578812 | 134.7 | 7.13924266 | | | 134.70 | 3.1871619 | 134.7 | 5.80553798 | 134.7 | 3.1871619 |
| 134.8 | 0.81395212 | 134.8 | 3.53039472 | 134.8 | 3.53039472 | 134.8 | 3.64807454 | 134.8 | 7.14904931 | | | 134.80 | 3.19696855 | 134.8 | 5.83495794 | 134.8 | 3.19696855 |
| 134.9 | 0.83356542 | 134.9 | 3.55000802 | 134.9 | 3.55000802 | 134.9 | 3.66768785 | 134.9 | 7.13924266 | | | 134.90 | 3.1871619 | 134.9 | 5.85457124 | 134.9 | 3.1871619 |
| 135 | 0.83356542 | 135 | 3.58923463 | 135 | 3.58923463 | 135 | 3.66768785 | 135 | 7.1098227 | | | 135.00 | 3.19696855 | 135 | 5.84476459 | 135 | 3.19696855 |
| 135.1 | 0.81395212 | 135.1 | 3.56962133 | 135.1 | 3.56962133 | 135.1 | 3.63826789 | 135.1 | 7.19808257 | | | 135.10 | 3.1871619 | 135.1 | 5.84476459 | 135.1 | 3.1871619 |
| 135.2 | 0.82375877 | 135.2 | 3.58923463 | 135.2 | 3.58923463 | 135.2 | 3.6578812 | 135.2 | 7.22750252 | | | 135.20 | 3.1871619 | 135.2 | 5.8643779 | 135.2 | 3.1871619 |
| 135.3 | 0.82375877 | 135.3 | 3.59904128 | 135.3 | 3.59904128 | 135.3 | 3.64807454 | 135.3 | 7.24711583 | | | 135.30 | 3.19696855 | 135.3 | 5.91341116 | 135.3 | 3.19696855 |
| 135.4 | 0.78453216 | 135.4 | 3.61865459 | 135.4 | 3.61865459 | 135.4 | 3.6774945 | 135.4 | 7.21769587 | | | 135.40 | 3.22638851 | 135.4 | 5.9036045 | 135.4 | 3.22638851 |
| 135.5 | 0.80414546 | 135.5 | 3.61865459 | 135.5 | 3.61865459 | 135.5 | 3.68730115 | 135.5 | 7.23730918 | | | 135.50 | 3.21658186 | 135.5 | 5.87418455 | 135.5 | 3.21658186 |
| 135.6 | 0.80414546 | 135.6 | 3.61865459 | 135.6 | 3.61865459 | 135.6 | 3.66768785 | 135.6 | 7.23730918 | | | 135.60 | 3.22638851 | 135.6 | 5.91341116 | 135.6 | 3.22638851 |
| 135.7 | 0.82375877 | 135.7 | 3.61865459 | 135.7 | 3.61865459 | 135.7 | 3.62846124 | 135.7 | 7.22750252 | | | 135.70 | 3.23619516 | 135.7 | 5.91341116 | 135.7 | 3.23619516 |
| 135.8 | 0.81395212 | 135.8 | 3.61865459 | 135.8 | 3.61865459 | 135.8 | 3.62846124 | 135.8 | 7.27653578 | | | 135.80 | 3.21658186 | 135.8 | 5.92321781 | 135.8 | 3.21658186 |
| 135.9 | 0.81395212 | 135.9 | 3.61865459 | 135.9 | 3.61865459 | 135.9 | 3.63826789 | 135.9 | 7.29614909 | | | 135.90 | 3.22638851 | 135.9 | 5.94283111 | 135.9 | 3.22638851 |
| 136 | 0.83356542 | 136 | 3.62846124 | 136 | 3.62846124 | 136 | 3.6774945 | 136 | 7.29614909 | | | 136.00 | 3.23619516 | 136 | 5.91341116 | 136 | 3.23619516 |
| 136.1 | 0.84337207 | 136.1 | 3.64807454 | 136.1 | 3.64807454 | 136.1 | 3.68730115 | 136.1 | 7.29614909 | | | 136.10 | 3.23619516 | 136.1 | 5.93302446 | 136.1 | 3.23619516 |
| 136.2 | 0.85317872 | 136.2 | 3.61865459 | 136.2 | 3.61865459 | 136.2 | 3.66768785 | 136.2 | 7.31576239 | | | 136.20 | 3.25580846 | 136.2 | 5.96244442 | 136.2 | 3.25580846 |
| 136.3 | 0.82375877 | 136.3 | 3.61865459 | 136.3 | 3.61865459 | 136.3 | 3.6578812 | 136.3 | 7.31576239 | | | 136.30 | 3.25580846 | 136.3 | 5.94283111 | 136.3 | 3.25580846 |
| 136.4 | 0.83356542 | 136.4 | 3.64807454 | 136.4 | 3.64807454 | 136.4 | 3.70691446 | 136.4 | 7.34518235 | | | 136.40 | 3.27542177 | 136.4 | 5.95263776 | 136.4 | 3.27542177 |
| 136.5 | 0.82375877 | 136.5 | 3.66768785 | 136.5 | 3.66768785 | 136.5 | 3.66768785 | 136.5 | 7.36479565 | | | 136.50 | 3.26561512 | 136.5 | 5.96244442 | 136.5 | 3.26561512 |
| 136.6 | 0.83356542 | 136.6 | 3.68730115 | 136.6 | 3.68730115 | 136.6 | 3.64807454 | 136.6 | 7.354989 | | | 136.60 | 3.27542177 | 136.6 | 5.94283111 | 136.6 | 3.27542177 |
| 136.7 | 0.85317872 | 136.7 | 3.68730115 | 136.7 | 3.68730115 | 136.7 | 3.68730115 | 136.7 | 7.36479565 | | | 136.70 | 3.26561512 | 136.7 | 5.96244442 | 136.7 | 3.26561512 |
| 136.8 | 0.91201864 | 136.8 | 3.6578812 | 136.8 | 3.6578812 | 136.8 | 3.6774945 | 136.8 | 7.36479565 | | | 136.80 | 3.29503507 | 136.8 | 5.98205772 | 136.8 | 3.29503507 |
| 136.9 | 0.88259868 | 136.9 | 3.64807454 | 136.9 | 3.64807454 | 136.9 | 3.66768785 | 136.9 | 7.40402226 | | | 136.90 | 3.29503507 | 136.9 | 6.00167102 | 136.9 | 3.29503507 |
| 137 | 0.90221198 | 137 | 3.66768785 | 137 | 3.66768785 | 137 | 3.6774945 | 137 | 7.41382891 | | | 137.00 | 3.29503507 | 137 | 6.02128433 | 137 | 3.29503507 |
| 137.1 | 0.91201864 | 137.1 | 3.6971078 | 137.1 | 3.6971078 | 137.1 | 3.68730115 | 137.1 | 7.40402226 | | | 137.10 | 3.29503507 | 137.1 | 6.00167102 | 137.1 | 3.29503507 |
| 137.2 | 0.92182529 | 137.2 | 3.6971078 | 137.2 | 3.6971078 | 137.2 | 3.70691446 | 137.2 | 7.43344222 | | | 137.20 | 3.30484172 | 137.2 | 6.00167102 | 137.2 | 3.30484172 |
| 137.3 | 0.91201864 | 137.3 | 3.68730115 | 137.3 | 3.68730115 | 137.3 | 3.71672111 | 137.3 | 7.44324887 | | | 137.30 | 3.31464838 | 137.3 | 6.00167102 | 137.3 | 3.31464838 |
| 137.4 | 0.89240533 | 137.4 | 3.6971078 | 137.4 | 3.6971078 | 137.4 | 3.71672111 | 137.4 | 7.46286217 | | | 137.40 | 3.31464838 | 137.4 | 6.02128433 | 137.4 | 3.31464838 |
| 137.5 | 0.92182529 | 137.5 | 3.71672111 | 137.5 | 3.71672111 | 137.5 | 3.70691446 | 137.5 | 7.46286217 | | | 137.50 | 3.31464838 | 137.5 | 6.03109098 | 137.5 | 3.31464838 |

| | | | | | | | | | | | | | | | | | |
|---|---|---|---|---|---|---|---|---|---|---|---|---|---|---|---|---|---|
| 137.6 | 0.90221198 | 137.6 | 3.73633441 | 137.6 | 3.73633441 | 137.6 | 3.6971078 | 137.6 | 7.46286217 | | | 137.60 | 3.31464838 | 137.6 | 6.02128433 | 137.6 | 3.31464838 |
| 137.7 | 0.88259868 | 137.7 | 3.71672111 | 137.7 | 3.71672111 | 137.7 | 3.6971078 | 137.7 | 7.46286217 | | | 137.70 | 3.30484172 | 137.7 | 6.01147768 | 137.7 | 3.30484172 |
| 137.8 | 0.89240533 | 137.8 | 3.72652776 | 137.8 | 3.72652776 | 137.8 | 3.70691446 | 137.8 | 7.49228213 | | | 137.80 | 3.31464838 | 137.8 | 6.01147768 | 137.8 | 3.31464838 |
| 137.9 | 0.89240533 | 137.9 | 3.74614106 | 137.9 | 3.74614106 | 137.9 | 3.70691446 | 137.9 | 7.51189543 | | | 137.90 | 3.33426168 | 137.9 | 6.04089763 | 137.9 | 3.33426168 |
| 138 | 0.89240533 | 138 | 3.73633441 | 138 | 3.73633441 | 138 | 3.74614106 | 138 | 7.53150874 | | | 138.00 | 3.35387498 | 138 | 6.06051094 | 138 | 3.35387498 |
| 138.1 | 0.93163194 | 138.1 | 3.74614106 | 138.1 | 3.74614106 | 138.1 | 3.73633441 | 138.1 | 7.55112204 | | | 138.10 | 3.35387498 | 138.1 | 6.04089763 | 138.1 | 3.35387498 |
| 138.2 | 0.89240533 | 138.2 | 3.72652776 | 138.2 | 3.72652776 | 138.2 | 3.72652776 | 138.2 | 7.580542 | | | 138.20 | 3.34406833 | 138.2 | 6.06051094 | 138.2 | 3.34406833 |
| 138.3 | 0.90221198 | 138.3 | 3.72652776 | 138.3 | 3.72652776 | 138.3 | 3.72652776 | 138.3 | 7.57073534 | | | 138.30 | 3.34406833 | 138.3 | 6.06051094 | 138.3 | 3.34406833 |
| 138.4 | 0.92182529 | 138.4 | 3.72652776 | 138.4 | 3.72652776 | 138.4 | 3.76575437 | 138.4 | 7.56092869 | | | 138.40 | 3.35387498 | 138.4 | 6.09973754 | 138.4 | 3.35387498 |
| 138.5 | 0.88259868 | 138.5 | 3.73633441 | 138.5 | 3.73633441 | 138.5 | 3.75594772 | 138.5 | 7.55112204 | | | 138.50 | 3.34406833 | 138.5 | 6.1095442 | 138.5 | 3.34406833 |
| 138.6 | 0.88259868 | 138.6 | 3.75594772 | 138.6 | 3.75594772 | 138.6 | 3.73633441 | 138.6 | 7.57073534 | | | 138.60 | 3.36368164 | 138.6 | 6.1095442 | 138.6 | 3.36368164 |
| 138.7 | 0.90221198 | 138.7 | 3.79517432 | 138.7 | 3.79517432 | 138.7 | 3.70691446 | 138.7 | 7.6001553 | | | 138.70 | 3.37348829 | 138.7 | 6.08012424 | 138.7 | 3.37348829 |
| 138.8 | 0.90221198 | 138.8 | 3.78536767 | 138.8 | 3.78536767 | 138.8 | 3.70691446 | 138.8 | 7.6001553 | | | 138.80 | 3.37348829 | 138.8 | 6.08993089 | 138.8 | 3.37348829 |
| 138.9 | 0.91201864 | 138.9 | 3.78536767 | 138.9 | 3.78536767 | 138.9 | 3.6774945 | 138.9 | 7.6197686 | | | 138.90 | 3.37348829 | 138.9 | 6.09973754 | 138.9 | 3.37348829 |
| 139 | 0.93163194 | 139 | 3.80498098 | 139 | 3.80498098 | 139 | 3.71672111 | 139 | 7.62957526 | | | 139.00 | 3.39310159 | 139 | 6.08012424 | 139 | 3.39310159 |
| 139.1 | 0.97085855 | 139.1 | 3.83440093 | 139.1 | 3.83440093 | 139.1 | 3.76575437 | 139.1 | 7.63938191 | | | 139.10 | 3.39310159 | 139.1 | 6.1095442 | 139.1 | 3.39310159 |
| 139.2 | 0.92182529 | 139.2 | 3.80498098 | 139.2 | 3.80498098 | 139.2 | 3.78536767 | 139.2 | 7.66880186 | | | 139.20 | 3.38329494 | 139.2 | 6.11935085 | 139.2 | 3.38329494 |
| 139.3 | 0.86298538 | 139.3 | 3.81478763 | 139.3 | 3.81478763 | 139.3 | 3.77556102 | 139.3 | 7.69822182 | | | 139.30 | 3.38329494 | 139.3 | 6.13896415 | 139.3 | 3.38329494 |
| 139.4 | 0.91201864 | 139.4 | 3.80498098 | 139.4 | 3.80498098 | 139.4 | 3.76575437 | 139.4 | 7.68841517 | | | 139.40 | 3.39310159 | 139.4 | 6.1291575 | 139.4 | 3.39310159 |
| 139.5 | 0.90221198 | 139.5 | 3.80498098 | 139.5 | 3.80498098 | 139.5 | 3.76575437 | 139.5 | 7.72764178 | | | 139.50 | 3.4127149 | 139.5 | 6.13896415 | 139.5 | 3.4127149 |
| 139.6 | 0.90221198 | 139.6 | 3.80498098 | 139.6 | 3.80498098 | 139.6 | 3.76575437 | 139.6 | 7.72764178 | | | 139.60 | 3.40290824 | 139.6 | 6.13896415 | 139.6 | 3.40290824 |
| 139.7 | 0.90221198 | 139.7 | 3.80498098 | 139.7 | 3.80498098 | 139.7 | 3.74614106 | 139.7 | 7.73744843 | | | 139.70 | 3.40290824 | 139.7 | 6.1291575 | 139.7 | 3.40290824 |
| 139.8 | 0.91201864 | 139.8 | 3.82459428 | 139.8 | 3.82459428 | 139.8 | 3.75594772 | 139.8 | 7.76686838 | | | 139.80 | 3.44213485 | 139.8 | 6.16838411 | 139.8 | 3.44213485 |
| 139.9 | 0.91201864 | 139.9 | 3.88343419 | 139.9 | 3.88343419 | 139.9 | 3.78536767 | 139.9 | 7.75706173 | | | 139.90 | 3.4323282 | 139.9 | 6.1487708 | 139.9 | 3.4323282 |
| 140 | 0.90221198 | 140 | 3.87362754 | 140 | 3.87362754 | 140 | 3.75594772 | 140 | 7.74725508 | | | 140.00 | 3.4127149 | 140 | 6.1487708 | 140 | 3.4127149 |
| 140.1 | 0.88259868 | 140.1 | 3.85401424 | 140.1 | 3.85401424 | 140.1 | 3.74614106 | 140.1 | 7.75706173 | | | 140.10 | 3.4323282 | 140.1 | 6.1487708 | 140.1 | 3.4323282 |
| 140.2 | 0.91201864 | 140.2 | 3.85401424 | 140.2 | 3.85401424 | 140.2 | 3.73633441 | 140.2 | 7.77667504 | | | 140.20 | 3.44213485 | 140.2 | 6.18799741 | 140.2 | 3.44213485 |
| 140.3 | 0.92182529 | 140.3 | 3.88343419 | 140.3 | 3.88343419 | 140.3 | 3.74614106 | 140.3 | 7.77667504 | | | 140.30 | 3.4323282 | 140.3 | 6.21741737 | 140.3 | 3.4323282 |
| 140.4 | 0.92182529 | 140.4 | 3.88343419 | 140.4 | 3.88343419 | 140.4 | 3.75594772 | 140.4 | 7.77667504 | | | 140.40 | 3.4323282 | 140.4 | 6.20761072 | 140.4 | 3.4323282 |
| 140.5 | 0.92182529 | 140.5 | 3.89324084 | 140.5 | 3.89324084 | 140.5 | 3.76575437 | 140.5 | 7.79628834 | | | 140.50 | 3.4323282 | 140.5 | 6.19780406 | 140.5 | 3.4323282 |
| 140.6 | 0.91201864 | 140.6 | 3.9226608 | 140.6 | 3.9226608 | 140.6 | 3.77556102 | 140.6 | 7.8453216 | | | 140.60 | 3.42252155 | 140.6 | 6.18799741 | 140.6 | 3.42252155 |
| 140.7 | 0.94143859 | 140.7 | 3.9030475 | 140.7 | 3.9030475 | 140.7 | 3.79517432 | 140.7 | 7.87474156 | | | 140.70 | 3.42252155 | 140.7 | 6.16838411 | 140.7 | 3.42252155 |
| 140.8 | 0.95124524 | 140.8 | 3.9226608 | 140.8 | 3.9226608 | 140.8 | 3.81478763 | 140.8 | 7.88454821 | | | 140.80 | 3.47155481 | 140.8 | 6.16838411 | 140.8 | 3.47155481 |
| 140.9 | 0.93163194 | 140.9 | 3.9226608 | 140.9 | 3.9226608 | 140.9 | 3.80498098 | 140.9 | 7.87474156 | | | 140.90 | 3.44213485 | 140.9 | 6.21741737 | 140.9 | 3.44213485 |
| 141 | 0.94143859 | 141 | 3.9030475 | 141 | 3.9030475 | 141 | 3.77556102 | 141 | 7.89435486 | | | 141.00 | 3.4519415 | 141 | 6.21741737 | 141 | 3.4519415 |
| 141.1 | 0.94143859 | 141.1 | 3.9030475 | 141.1 | 3.9030475 | 141.1 | 3.77556102 | 141.1 | 7.88454821 | | | 141.10 | 3.47155481 | 141.1 | 6.22722402 | 141.1 | 3.47155481 |
| 141.2 | 0.95124524 | 141.2 | 3.91285415 | 141.2 | 3.91285415 | 141.2 | 3.79517432 | 141.2 | 7.89435486 | | | 141.20 | 3.46174816 | 141.2 | 6.25664398 | 141.2 | 3.46174816 |
| 141.3 | 0.99047185 | 141.3 | 3.9226608 | 141.3 | 3.9226608 | 141.3 | 3.80498098 | 141.3 | 7.90416151 | | | 141.30 | 3.46174816 | 141.3 | 6.24683732 | 141.3 | 3.46174816 |
| 141.4 | 0.97085855 | 141.4 | 3.9226608 | 141.4 | 3.9226608 | 141.4 | 3.82459428 | 141.4 | 7.91396816 | | | 141.40 | 3.48136146 | 141.4 | 6.23703067 | 141.4 | 3.48136146 |
| 141.5 | 0.94143859 | 141.5 | 3.91285415 | 141.5 | 3.91285415 | 141.5 | 3.82459428 | 141.5 | 7.93358147 | | | 141.50 | 3.49116811 | 141.5 | 6.25664398 | 141.5 | 3.49116811 |
| 141.6 | 0.94143859 | 141.6 | 3.9226608 | 141.6 | 3.9226608 | 141.6 | 3.80498098 | 141.6 | 7.94338812 | | | 141.60 | 3.47155481 | 141.6 | 6.27625728 | 141.6 | 3.47155481 |
| 141.7 | 0.92182529 | 141.7 | 3.96188741 | 141.7 | 3.96188741 | 141.7 | 3.82459428 | 141.7 | 7.94338812 | | | 141.70 | 3.49116811 | 141.7 | 6.27625728 | 141.7 | 3.49116811 |
| 141.8 | 0.92182529 | 141.8 | 3.95208076 | 141.8 | 3.95208076 | 141.8 | 3.84420758 | 141.8 | 7.93358147 | | | 141.80 | 3.52058807 | 141.8 | 6.26645063 | 141.8 | 3.52058807 |
| 141.9 | 0.92182529 | 141.9 | 3.9422741 | 141.9 | 3.9422741 | 141.9 | 3.83440093 | 141.9 | 7.95319477 | | | 141.90 | 3.50097476 | 141.9 | 6.28606393 | 141.9 | 3.50097476 |
| 142 | 0.92182529 | 142 | 3.96188741 | 142 | 3.96188741 | 142 | 3.85401424 | 142 | 7.97280808 | | | 142.00 | 3.50097476 | 142 | 6.29587058 | 142 | 3.50097476 |
| 142.1 | 0.95124524 | 142.1 | 3.96188741 | 142.1 | 3.96188741 | 142.1 | 3.87362754 | 142.1 | 7.99242138 | | | 142.10 | 3.49116811 | 142.1 | 6.29587058 | 142.1 | 3.49116811 |
| 142.2 | 0.9610519 | 142.2 | 3.98150071 | 142.2 | 3.98150071 | 142.2 | 3.85401424 | 142.2 | 8.00222803 | | | 142.20 | 3.50097476 | 142.2 | 6.30567724 | 142.2 | 3.50097476 |
| 142.3 | 0.94143859 | 142.3 | 4.00111402 | 142.3 | 4.00111402 | 142.3 | 3.85401424 | 142.3 | 8.00222803 | | | 142.30 | 3.51078142 | 142.3 | 6.32529054 | 142.3 | 3.51078142 |
| 142.4 | 0.92182529 | 142.4 | 3.99130736 | 142.4 | 3.99130736 | 142.4 | 3.85401424 | 142.4 | 8.04145464 | | | 142.40 | 3.50097476 | 142.4 | 6.32529054 | 142.4 | 3.50097476 |
| 142.5 | 0.95124524 | 142.5 | 3.97169406 | 142.5 | 3.97169406 | 142.5 | 3.85401424 | 142.5 | 8.05126129 | | | 142.50 | 3.54020137 | 142.5 | 6.30567724 | 142.5 | 3.54020137 |
| 142.6 | 0.95124524 | 142.6 | 3.98150071 | 142.6 | 3.98150071 | 142.6 | 3.82459428 | 142.6 | 8.05126129 | | | 142.60 | 3.53039472 | 142.6 | 6.31548389 | 142.6 | 3.53039472 |
| 142.7 | 0.95124524 | 142.7 | 3.99130736 | 142.7 | 3.99130736 | 142.7 | 3.84420758 | 142.7 | 8.03164799 | | | 142.70 | 3.52058807 | 142.7 | 6.31548389 | 142.7 | 3.52058807 |
| 142.8 | 0.95124524 | 142.8 | 4.02072732 | 142.8 | 4.02072732 | 142.8 | 3.81478763 | 142.8 | 8.0708746 | | | 142.80 | 3.53039472 | 142.8 | 6.33509719 | 142.8 | 3.53039472 |
| 142.9 | 0.95124524 | 142.9 | 4.00111402 | 142.9 | 4.00111402 | 142.9 | 3.81478763 | 142.9 | 8.06106794 | | | 142.90 | 3.54020137 | 142.9 | 6.34490384 | 142.9 | 3.54020137 |
| 143 | 0.9610519 | 143 | 3.99130736 | 143 | 3.99130736 | 143 | 3.84420758 | 143 | 8.0708746 | | | 143.00 | 3.53039472 | 143 | 6.34490384 | 143 | 3.53039472 |
| 143.1 | 0.97085855 | 143.1 | 4.03053397 | 143.1 | 4.03053397 | 143.1 | 3.84420758 | 143.1 | 8.08068125 | | | 143.10 | 3.55000802 | 143.1 | 6.34490384 | 143.1 | 3.55000802 |
| 143.2 | 1.0002785 | 143.2 | 4.04034062 | 143.2 | 4.04034062 | 143.2 | 3.85401424 | 143.2 | 8.0904879 | | | 143.20 | 3.56962133 | 143.2 | 6.3547105 | 143.2 | 3.56962133 |
| 143.3 | 1.0002785 | 143.3 | 4.03053397 | 143.3 | 4.03053397 | 143.3 | 3.87362754 | 143.3 | 8.13952116 | | | 143.30 | 3.54020137 | 143.3 | 6.36451715 | 143.3 | 3.54020137 |
| 143.4 | 0.97085855 | 143.4 | 4.02072732 | 143.4 | 4.02072732 | 143.4 | 3.89324084 | 143.4 | 8.15913446 | | | 143.40 | 3.53039472 | 143.4 | 6.3547105 | 143.4 | 3.53039472 |
| 143.5 | 0.93163194 | 143.5 | 4.03053397 | 143.5 | 4.03053397 | 143.5 | 3.89324084 | 143.5 | 8.14932781 | | | 143.50 | 3.55000802 | 143.5 | 6.3743238 | 143.5 | 3.55000802 |
| 143.6 | 0.9806652 | 143.6 | 4.04034062 | 143.6 | 4.04034062 | 143.6 | 3.87362754 | 143.6 | 8.15913446 | | | 143.60 | 3.56962133 | 143.6 | 6.40374376 | 143.6 | 3.56962133 |

| | | | | | | | | | | | | | | | | | |
|---|---|---|---|---|---|---|---|---|---|---|---|---|---|---|---|---|---|
| 143.7 | 0.9610519 | 143.7 | 4.04034062 | 143.7 | 4.04034062 | 143.7 | 3.86382089 | 143.7 | 8.17874777 | | | 143.70 | 3.58923463 | 143.7 | 6.40374376 | 143.7 | 3.58923463 |
| 143.8 | 0.90221198 | 143.8 | 4.04034062 | 143.8 | 4.04034062 | 143.8 | 3.88343419 | 143.8 | 8.20816772 | | | 143.80 | 3.58923463 | 143.8 | 6.3939371 | 143.8 | 3.58923463 |
| 143.9 | 0.90221198 | 143.9 | 4.05014728 | 143.9 | 4.05014728 | 143.9 | 3.9030475 | 143.9 | 8.18855442 | | | 143.90 | 3.58923463 | 143.9 | 6.42335706 | 143.9 | 3.58923463 |
| 144 | 0.94143859 | 144 | 4.05995393 | 144 | 4.05995393 | 144 | 3.9030475 | 144 | 8.21797438 | | | 144.00 | 3.57942798 | 144 | 6.41355041 | 144 | 3.57942798 |
| 144.1 | 0.9806652 | 144.1 | 4.05995393 | 144.1 | 4.05995393 | 144.1 | 3.89324084 | 144.1 | 8.25720098 | | | 144.10 | 3.59904128 | 144.1 | 6.43316371 | 144.1 | 3.59904128 |
| 144.2 | 0.97085855 | 144.2 | 4.05995393 | 144.2 | 4.05995393 | 144.2 | 3.89324084 | 144.2 | 8.25720098 | | | 144.20 | 3.60884794 | 144.2 | 6.42335706 | 144.2 | 3.60884794 |
| 144.3 | 0.9610519 | 144.3 | 4.09918054 | 144.3 | 4.09918054 | 144.3 | 3.89324084 | 144.3 | 8.23758768 | | | 144.30 | 3.59904128 | 144.3 | 6.42335706 | 144.3 | 3.59904128 |
| 144.4 | 0.99047185 | 144.4 | 4.09918054 | 144.4 | 4.09918054 | 144.4 | 3.89324084 | 144.4 | 8.22778103 | | | 144.40 | 3.61865459 | 144.4 | 6.45277702 | 144.4 | 3.61865459 |
| 144.5 | 0.94143859 | 144.5 | 4.10898719 | 144.5 | 4.10898719 | 144.5 | 3.9030475 | 144.5 | 8.24739433 | | | 144.50 | 3.59904128 | 144.5 | 6.49200362 | 144.5 | 3.59904128 |
| 144.6 | 0.95124524 | 144.6 | 4.08937388 | 144.6 | 4.08937388 | 144.6 | 3.91285415 | 144.6 | 8.27681429 | | | 144.60 | 3.56962133 | 144.6 | 6.46258367 | 144.6 | 3.56962133 |
| 144.7 | 1.0002785 | 144.7 | 4.10898719 | 144.7 | 4.10898719 | 144.7 | 3.9030475 | 144.7 | 8.28662094 | | | 144.70 | 3.59904128 | 144.7 | 6.48219697 | 144.7 | 3.59904128 |
| 144.8 | 0.97085855 | 144.8 | 4.12860049 | 144.8 | 4.12860049 | 144.8 | 3.9030475 | 144.8 | 8.28662094 | | | 144.80 | 3.61865459 | 144.8 | 6.49200362 | 144.8 | 3.61865459 |
| 144.9 | 0.9610519 | 144.9 | 4.11879384 | 144.9 | 4.11879384 | 144.9 | 3.95208076 | 144.9 | 8.27681429 | | | 144.90 | 3.59904128 | 144.9 | 6.49200362 | 144.9 | 3.59904128 |
| 145 | 0.9806652 | 145 | 4.10898719 | 145 | 4.10898719 | 145 | 3.95208076 | 145 | 8.29642759 | | | 145.00 | 3.60884794 | 145 | 6.49200362 | 145 | 3.60884794 |
| 145.1 | 0.95124524 | 145.1 | 4.12860049 | 145.1 | 4.12860049 | 145.1 | 3.95208076 | 145.1 | 8.34546085 | | | 145.10 | 3.63826789 | 145.1 | 6.50181028 | 145.1 | 3.63826789 |
| 145.2 | 0.93163194 | 145.2 | 4.13840714 | 145.2 | 4.13840714 | 145.2 | 3.95208076 | 145.2 | 8.34546085 | | | 145.20 | 3.64807454 | 145.2 | 6.51161693 | 145.2 | 3.64807454 |
| 145.3 | 0.95124524 | 145.3 | 4.12860049 | 145.3 | 4.12860049 | 145.3 | 3.9422741 | 145.3 | 8.3552675 | | | 145.30 | 3.6578812 | 145.3 | 6.55084354 | 145.3 | 3.6578812 |
| 145.4 | 0.9610519 | 145.4 | 4.13840714 | 145.4 | 4.13840714 | 145.4 | 3.9226608 | 145.4 | 8.36507416 | | | 145.40 | 3.64807454 | 145.4 | 6.54103688 | 145.4 | 3.64807454 |
| 145.5 | 0.93163194 | 145.5 | 4.1482138 | 145.5 | 4.1482138 | 145.5 | 3.9226608 | 145.5 | 8.3552675 | | | 145.50 | 3.63826789 | 145.5 | 6.53123023 | 145.5 | 3.63826789 |
| 145.6 | 0.95124524 | 145.6 | 4.13840714 | 145.6 | 4.13840714 | 145.6 | 3.88343419 | 145.6 | 8.36507416 | | | 145.60 | 3.64807454 | 145.6 | 6.54103688 | 145.6 | 3.64807454 |
| 145.7 | 0.95124524 | 145.7 | 4.1678271 | 145.7 | 4.1678271 | 145.7 | 3.93246745 | 145.7 | 8.39449411 | | | 145.70 | 3.6578812 | 145.7 | 6.54103688 | 145.7 | 3.6578812 |
| 145.8 | 0.94143859 | 145.8 | 4.15802045 | 145.8 | 4.15802045 | 145.8 | 3.96188741 | 145.8 | 8.41410742 | | | 145.80 | 3.64807454 | 145.8 | 6.54103688 | 145.8 | 3.64807454 |
| 145.9 | 0.9610519 | 145.9 | 4.1678271 | 145.9 | 4.1678271 | 145.9 | 3.96188741 | 145.9 | 8.42391407 | | | 145.90 | 3.60884794 | 145.9 | 6.53123023 | 145.9 | 3.60884794 |
| 146 | 0.93163194 | 146 | 4.1678271 | 146 | 4.1678271 | 146 | 3.95208076 | 146 | 8.41410742 | | | 146.00 | 3.63826789 | 146 | 6.54103688 | 146 | 3.63826789 |
| 146.1 | 0.95124524 | 146.1 | 4.1678271 | 146.1 | 4.1678271 | 146.1 | 3.98150071 | 146.1 | 8.44352737 | | | 146.10 | 3.66768785 | 146.1 | 6.55084354 | 146.1 | 3.66768785 |
| 146.2 | 0.99047185 | 146.2 | 4.1678271 | 146.2 | 4.1678271 | 146.2 | 3.9422741 | 146.2 | 8.46314068 | | | 146.20 | 3.6578812 | 146.2 | 6.58026349 | 146.2 | 3.6578812 |
| 146.3 | 0.97085855 | 146.3 | 4.17763375 | 146.3 | 4.17763375 | 146.3 | 3.91285415 | 146.3 | 8.44352737 | | | 146.30 | 3.6774945 | 146.3 | 6.5998768 | 146.3 | 3.6774945 |
| 146.4 | 0.9610519 | 146.4 | 4.20705371 | 146.4 | 4.20705371 | 146.4 | 3.9226608 | 146.4 | 8.46314068 | | | 146.40 | 3.68730115 | 146.4 | 6.59007014 | 146.4 | 3.68730115 |
| 146.5 | 0.9806652 | 146.5 | 4.19724706 | 146.5 | 4.19724706 | 146.5 | 3.93246745 | 146.5 | 8.50236728 | | | 146.50 | 3.68730115 | 146.5 | 6.57045684 | 146.5 | 3.68730115 |
| 146.6 | 1.01989181 | 146.6 | 4.17763375 | 146.6 | 4.17763375 | 146.6 | 3.91285415 | 146.6 | 8.51217394 | | | 146.60 | 3.6774945 | 146.6 | 6.5998768 | 146.6 | 3.6774945 |
| 146.7 | 0.97085855 | 146.7 | 4.1874404 | 146.7 | 4.1874404 | 146.7 | 3.9422741 | 146.7 | 8.50236728 | | | 146.70 | 3.6971078 | 146.7 | 6.59007014 | 146.7 | 3.6971078 |
| 146.8 | 0.94143859 | 146.8 | 4.19724706 | 146.8 | 4.19724706 | 146.8 | 3.99130736 | 146.8 | 8.5808205 | | | 146.80 | 3.6971078 | 146.8 | 6.60968345 | 146.8 | 3.6971078 |
| 146.9 | 0.95124524 | 146.9 | 4.22666701 | 146.9 | 4.22666701 | 146.9 | 3.97169406 | 146.9 | 8.57101385 | | | 146.90 | 3.68730115 | 146.9 | 6.59007014 | 146.9 | 3.68730115 |
| 147 | 0.9610519 | 147 | 4.23647366 | 147 | 4.23647366 | 147 | 3.96188741 | 147 | 8.5612072 | | | 147.00 | 3.70691446 | 147 | 6.59007014 | 147 | 3.70691446 |
| 147.1 | 1.0002785 | 147.1 | 4.21686036 | 147.1 | 4.21686036 | 147.1 | 4.01092067 | 147.1 | 8.5808205 | | | 147.10 | 3.71672111 | 147.1 | 6.6194901 | 147.1 | 3.71672111 |
| 147.2 | 0.97085855 | 147.2 | 4.22666701 | 147.2 | 4.22666701 | 147.2 | 3.99130736 | 147.2 | 8.5808205 | | | 147.20 | 3.71672111 | 147.2 | 6.62929675 | 147.2 | 3.71672111 |
| 147.3 | 0.97085855 | 147.3 | 4.21686036 | 147.3 | 4.21686036 | 147.3 | 4.01092067 | 147.3 | 8.59062715 | | | 147.30 | 3.6971078 | 147.3 | 6.65871671 | 147.3 | 3.6971078 |
| 147.4 | 0.9610519 | 147.4 | 4.20705371 | 147.4 | 4.20705371 | 147.4 | 3.98150071 | 147.4 | 8.61024046 | | | 147.40 | 3.71672111 | 147.4 | 6.6391034 | 147.4 | 3.71672111 |
| 147.5 | 0.97085855 | 147.5 | 4.21686036 | 147.5 | 4.21686036 | 147.5 | 3.99130736 | 147.5 | 8.61024046 | | | 147.50 | 3.72652776 | 147.5 | 6.6391034 | 147.5 | 3.72652776 |
| 147.6 | 0.97085855 | 147.6 | 4.22666701 | 147.6 | 4.22666701 | 147.6 | 3.99130736 | 147.6 | 8.62985376 | | | 147.60 | 3.71672111 | 147.6 | 6.64891006 | 147.6 | 3.71672111 |
| 147.7 | 0.9610519 | 147.7 | 4.24628032 | 147.7 | 4.24628032 | 147.7 | 3.98150071 | 147.7 | 8.62985376 | | | 147.70 | 3.6971078 | 147.7 | 6.65871671 | 147.7 | 3.6971078 |
| 147.8 | 0.99047185 | 147.8 | 4.22666701 | 147.8 | 4.22666701 | 147.8 | 3.99130736 | 147.8 | 8.64946706 | | | 147.80 | 3.70691446 | 147.8 | 6.67833001 | 147.8 | 3.70691446 |
| 147.9 | 1.0002785 | 147.9 | 4.23647366 | 147.9 | 4.23647366 | 147.9 | 4.01092067 | 147.9 | 8.63966041 | | | 147.90 | 3.78536767 | 147.9 | 6.70774997 | 147.9 | 3.78536767 |
| 148 | 0.97085855 | 148 | 4.28550692 | 148 | 4.28550692 | 148 | 3.98150071 | 148 | 8.66908037 | | | 148.00 | 3.74614106 | 148 | 6.71755662 | 148 | 3.74614106 |
| 148.1 | 0.99047185 | 148.1 | 4.26589362 | 148.1 | 4.26589362 | 148.1 | 3.97169406 | 148.1 | 8.66908037 | | | 148.10 | 3.74614106 | 148.1 | 6.70774997 | 148.1 | 3.74614106 |
| 148.2 | 0.97085855 | 148.2 | 4.24628032 | 148.2 | 4.24628032 | 148.2 | 4.01092067 | 148.2 | 8.66908037 | | | 148.20 | 3.75594772 | 148.2 | 6.74697658 | 148.2 | 3.75594772 |
| 148.3 | 1.0002785 | 148.3 | 4.26589362 | 148.3 | 4.26589362 | 148.3 | 4.02072732 | 148.3 | 8.66908037 | | | 148.30 | 3.77556102 | 148.3 | 6.73716992 | 148.3 | 3.77556102 |
| 148.4 | 0.99047185 | 148.4 | 4.26589362 | 148.4 | 4.26589362 | 148.4 | 4.00111402 | 148.4 | 8.66908037 | | | 148.40 | 3.77556102 | 148.4 | 6.73716992 | 148.4 | 3.77556102 |
| 148.5 | 0.9806652 | 148.5 | 4.26589362 | 148.5 | 4.26589362 | 148.5 | 4.02072732 | 148.5 | 8.68869367 | | | 148.50 | 3.78536767 | 148.5 | 6.74697658 | 148.5 | 3.78536767 |
| 148.6 | 0.97085855 | 148.6 | 4.26589362 | 148.6 | 4.26589362 | 148.6 | 4.02072732 | 148.6 | 8.71811363 | | | 148.60 | 3.77556102 | 148.6 | 6.75678323 | 148.6 | 3.77556102 |
| 148.7 | 0.97085855 | 148.7 | 4.29531358 | 148.7 | 4.29531358 | 148.7 | 4.04034062 | 148.7 | 8.72792028 | | | 148.70 | 3.77556102 | 148.7 | 6.76658988 | 148.7 | 3.77556102 |
| 148.8 | 0.99047185 | 148.8 | 4.35415349 | 148.8 | 4.35415349 | 148.8 | 3.98150071 | 148.8 | 8.72792028 | | | 148.80 | 3.78536767 | 148.8 | 6.78620318 | 148.8 | 3.78536767 |
| 148.9 | 0.97085855 | 148.9 | 4.36396014 | 148.9 | 4.36396014 | 148.9 | 3.95208076 | 148.9 | 8.74753358 | | | 148.90 | 3.78536767 | 148.9 | 6.79600984 | 148.9 | 3.78536767 |
| 149 | 0.97085855 | 149 | 4.31492688 | 149 | 4.31492688 | 149 | 4.00111402 | 149 | 8.76714689 | | | 149.00 | 3.76575437 | 149 | 6.77639653 | 149 | 3.76575437 |
| 149.1 | 0.97085855 | 149.1 | 4.30512023 | 149.1 | 4.30512023 | 149.1 | 4.03053397 | 149.1 | 8.79656684 | | | 149.10 | 3.77556102 | 149.1 | 6.77639653 | 149.1 | 3.77556102 |
| 149.2 | 1.0002785 | 149.2 | 4.28550692 | 149.2 | 4.28550692 | 149.2 | 4.02072732 | 149.2 | 8.81618015 | | | 149.20 | 3.73633441 | 149.2 | 6.80581649 | 149.2 | 3.73633441 |
| 149.3 | 1.01008516 | 149.3 | 4.30512023 | 149.3 | 4.30512023 | 149.3 | 4.03053397 | 149.3 | 8.79656684 | | | 149.30 | 3.76575437 | 149.3 | 6.82542979 | 149.3 | 3.76575437 |
| 149.4 | 1.01008516 | 149.4 | 4.35415349 | 149.4 | 4.35415349 | 149.4 | 4.02072732 | 149.4 | 8.78676019 | | | 149.40 | 3.79517432 | 149.4 | 6.85484975 | 149.4 | 3.79517432 |
| 149.5 | 0.9806652 | 149.5 | 4.37376679 | 149.5 | 4.37376679 | 149.5 | 4.04034062 | 149.5 | 8.78676019 | | | 149.50 | 3.79517432 | 149.5 | 6.85484975 | 149.5 | 3.79517432 |
| 149.6 | 0.9610519 | 149.6 | 4.37376679 | 149.6 | 4.37376679 | 149.6 | 4.05014728 | 149.6 | 8.81618015 | | | 149.60 | 3.81478763 | 149.6 | 6.8646564 | 149.6 | 3.81478763 |
| 149.7 | 1.0002785 | 149.7 | 4.37376679 | 149.7 | 4.37376679 | 149.7 | 4.00111402 | 149.7 | 8.83579345 | | | 149.70 | 3.83440093 | 149.7 | 6.89407636 | 149.7 | 3.83440093 |

| | | | | | | | | | | | | | | | | | |
|---|---|---|---|---|---|---|---|---|---|---|---|---|---|---|---|---|---|
| 149.8 | 1.01008516 | 149.8 | 4.36396014 | 149.8 | 4.36396014 | 149.8 | 4.05014728 | 149.8 | 8.81618015 | | | 149.80 | 3.80498098 | 149.8 | 6.87446305 | 149.8 | 3.80498098 |
| 149.9 | 1.01989181 | 149.9 | 4.37376679 | 149.9 | 4.37376679 | 149.9 | 4.05014728 | 149.9 | 8.8259868 | | | 149.90 | 3.82459428 | 149.9 | 6.8842697 | 149.9 | 3.82459428 |
| 150 | 1.01008516 | 150 | 4.38357344 | 150 | 4.38357344 | 150 | 4.04034062 | 150 | 8.85540676 | | | 150.00 | 3.83440093 | 150 | 6.8842697 | 150 | 3.83440093 |
| 150.1 | 1.01989181 | 150.1 | 4.36396014 | 150.1 | 4.36396014 | 150.1 | 4.04034062 | 150.1 | 8.87502006 | | | 150.10 | 3.83440093 | 150.1 | 6.8646564 | 150.1 | 3.83440093 |
| 150.2 | 1.0002785 | 150.2 | 4.34434684 | 150.2 | 4.34434684 | 150.2 | 4.04034062 | 150.2 | 8.87502006 | | | 150.20 | 3.81478763 | 150.2 | 6.89407636 | 150.2 | 3.81478763 |
| 150.3 | 0.9806652 | 150.3 | 4.38357344 | 150.3 | 4.38357344 | 150.3 | 4.03053397 | 150.3 | 8.90444002 | | | 150.30 | 3.79517432 | 150.3 | 6.90388301 | 150.3 | 3.79517432 |
| 150.4 | 1.01008516 | 150.4 | 4.42280005 | 150.4 | 4.42280005 | 150.4 | 4.02072732 | 150.4 | 8.91424667 | | | 150.40 | 3.80498098 | 150.4 | 6.91368966 | 150.4 | 3.80498098 |
| 150.5 | 1.02969846 | 150.5 | 4.37376679 | 150.5 | 4.37376679 | 150.5 | 4.03053397 | 150.5 | 8.92405332 | | | 150.50 | 3.82459428 | 150.5 | 6.92349631 | 150.5 | 3.82459428 |
| 150.6 | 1.01008516 | 150.6 | 4.37376679 | 150.6 | 4.37376679 | 150.6 | 4.04034062 | 150.6 | 8.95347328 | | | 150.60 | 3.83440093 | 150.6 | 6.90388301 | 150.6 | 3.83440093 |
| 150.7 | 1.02969846 | 150.7 | 4.4129934 | 150.7 | 4.4129934 | 150.7 | 4.05995393 | 150.7 | 8.95347328 | | | 150.70 | 3.83440093 | 150.7 | 6.8842697 | 150.7 | 3.83440093 |
| 150.8 | 1.01989181 | 150.8 | 4.44241336 | 150.8 | 4.44241336 | 150.8 | 4.02072732 | 150.8 | 8.93385997 | | | 150.80 | 3.84420758 | 150.8 | 6.90388301 | 150.8 | 3.84420758 |
| 150.9 | 0.97085855 | 150.9 | 4.44241336 | 150.9 | 4.44241336 | 150.9 | 4.06976058 | 150.9 | 8.96327993 | | | 150.90 | 3.84420758 | 150.9 | 6.95291627 | 150.9 | 3.84420758 |
| 151 | 0.9610519 | 151 | 4.45222001 | 151 | 4.45222001 | 151 | 4.08937388 | 151 | 8.97308658 | | | 151.00 | 3.86382089 | 151 | 6.96272292 | 151 | 3.86382089 |
| 151.1 | 1.0002785 | 151.1 | 4.45222001 | 151.1 | 4.45222001 | 151.1 | 4.08937388 | 151.1 | 9.00250654 | | | 151.10 | 3.87362754 | 151.1 | 6.94310962 | 151.1 | 3.87362754 |
| 151.2 | 1.0002785 | 151.2 | 4.48163996 | 151.2 | 4.48163996 | 151.2 | 4.09918054 | 151.2 | 9.00250654 | | | 151.20 | 3.84420758 | 151.2 | 6.93330296 | 151.2 | 3.84420758 |
| 151.3 | 0.9806652 | 151.3 | 4.44241336 | 151.3 | 4.44241336 | 151.3 | 4.09918054 | 151.3 | 9.04173314 | | | 151.30 | 3.86382089 | 151.3 | 6.94310962 | 151.3 | 3.86382089 |
| 151.4 | 1.0002785 | 151.4 | 4.42280005 | 151.4 | 4.42280005 | 151.4 | 4.08937388 | 151.4 | 9.02211984 | | | 151.40 | 3.85401424 | 151.4 | 6.97252957 | 151.4 | 3.85401424 |
| 151.5 | 1.02969846 | 151.5 | 4.44241336 | 151.5 | 4.44241336 | 151.5 | 4.09918054 | 151.5 | 9.03192649 | | | 151.50 | 3.85401424 | 151.5 | 7.00194953 | 151.5 | 3.85401424 |
| 151.6 | 1.01008516 | 151.6 | 4.45222001 | 151.6 | 4.45222001 | 151.6 | 4.08937388 | 151.6 | 9.0711531 | | | 151.60 | 3.89324084 | 151.6 | 7.00194953 | 151.6 | 3.89324084 |
| 151.7 | 1.03950511 | 151.7 | 4.52086657 | 151.7 | 4.52086657 | 151.7 | 4.09918054 | 151.7 | 9.0907664 | | | 151.70 | 3.9030475 | 151.7 | 7.01175618 | 151.7 | 3.9030475 |
| 151.8 | 1.03950511 | 151.8 | 4.49144662 | 151.8 | 4.49144662 | 151.8 | 4.08937388 | 151.8 | 9.11037971 | | | 151.80 | 3.88343419 | 151.8 | 7.01175618 | 151.8 | 3.88343419 |
| 151.9 | 1.03950511 | 151.9 | 4.46202666 | 151.9 | 4.46202666 | 151.9 | 4.07956723 | 151.9 | 9.12999301 | | | 151.90 | 3.93246745 | 151.9 | 6.99214288 | 151.9 | 3.93246745 |
| 152 | 1.02969846 | 152 | 4.46202666 | 152 | 4.46202666 | 152 | 4.06976058 | 152 | 9.12018636 | | | 152.00 | 3.91285415 | 152 | 7.00194953 | 152 | 3.91285415 |
| 152.1 | 1.02969846 | 152.1 | 4.45222001 | 152.1 | 4.45222001 | 152.1 | 4.06976058 | 152.1 | 9.12999301 | | | 152.10 | 3.9030475 | 152.1 | 7.00194953 | 152.1 | 3.9030475 |
| 152.2 | 1.06892507 | 152.2 | 4.46202666 | 152.2 | 4.46202666 | 152.2 | 4.08937388 | 152.2 | 9.14960632 | | | 152.20 | 3.9030475 | 152.2 | 7.01175618 | 152.2 | 3.9030475 |
| 152.3 | 1.02969846 | 152.3 | 4.47183331 | 152.3 | 4.47183331 | 152.3 | 4.10898719 | 152.3 | 9.14960632 | | | 152.30 | 3.91285415 | 152.3 | 7.05098279 | 152.3 | 3.91285415 |
| 152.4 | 1.01008516 | 152.4 | 4.50125327 | 152.4 | 4.50125327 | 152.4 | 4.08937388 | 152.4 | 9.16921962 | | | 152.40 | 3.9226608 | 152.4 | 7.06078944 | 152.4 | 3.9226608 |
| 152.5 | 1.02969846 | 152.5 | 4.51105992 | 152.5 | 4.51105992 | 152.5 | 4.07956723 | 152.5 | 9.18883292 | | | 152.50 | 3.9422741 | 152.5 | 7.05098279 | 152.5 | 3.9422741 |
| 152.6 | 1.03950511 | 152.6 | 4.52086657 | 152.6 | 4.52086657 | 152.6 | 4.05995393 | 152.6 | 9.14960632 | | | 152.60 | 3.88343419 | 152.6 | 7.06078944 | 152.6 | 3.88343419 |
| 152.7 | 1.05911842 | 152.7 | 4.51105992 | 152.7 | 4.51105992 | 152.7 | 4.05995393 | 152.7 | 9.19863958 | | | 152.70 | 3.9030475 | 152.7 | 7.10001605 | 152.7 | 3.9030475 |
| 152.8 | 1.05911842 | 152.8 | 4.48163996 | 152.8 | 4.48163996 | 152.8 | 4.10898719 | 152.8 | 9.18883292 | | | 152.80 | 3.9226608 | 152.8 | 7.1098227 | 152.8 | 3.9226608 |
| 152.9 | 1.09834502 | 152.9 | 4.50125327 | 152.9 | 4.50125327 | 152.9 | 4.11879384 | 152.9 | 9.15941297 | | | 152.90 | 3.9226608 | 152.9 | 7.129436 | 152.9 | 3.9226608 |
| 153 | 1.04931176 | 153 | 4.51105992 | 153 | 4.51105992 | 153 | 4.06976058 | 153 | 9.15941297 | | | 153.00 | 3.93246745 | 153 | 7.1098227 | 153 | 3.93246745 |
| 153.1 | 1.05911842 | 153.1 | 4.50125327 | 153.1 | 4.50125327 | 153.1 | 4.07956723 | 153.1 | 9.16921962 | | | 153.10 | 3.93246745 | 153.1 | 7.10001605 | 153.1 | 3.93246745 |
| 153.2 | 1.07873172 | 153.2 | 4.49144662 | 153.2 | 4.49144662 | 153.2 | 4.09918054 | 153.2 | 9.22805953 | | | 153.20 | 3.91285415 | 153.2 | 7.11962935 | 153.2 | 3.91285415 |
| 153.3 | 1.05911842 | 153.3 | 4.53067322 | 153.3 | 4.53067322 | 153.3 | 4.10898719 | 153.3 | 9.22805953 | | | 153.30 | 3.93246745 | 153.3 | 7.1098227 | 153.3 | 3.93246745 |
| 153.4 | 1.04931176 | 153.4 | 4.55028653 | 153.4 | 4.55028653 | 153.4 | 4.09918054 | 153.4 | 9.23786618 | | | 153.40 | 3.95208076 | 153.4 | 7.07059609 | 153.4 | 3.95208076 |
| 153.5 | 1.03950511 | 153.5 | 4.54047988 | 153.5 | 4.54047988 | 153.5 | 4.05995393 | 153.5 | 9.24767284 | | | 153.50 | 3.93246745 | 153.5 | 7.0902094 | 153.5 | 3.93246745 |
| 153.6 | 1.04931176 | 153.6 | 4.59931979 | 153.6 | 4.59931979 | 153.6 | 4.11879384 | 153.6 | 9.27709279 | | | 153.60 | 3.9226608 | 153.6 | 7.11962935 | 153.6 | 3.9226608 |
| 153.7 | 1.03950511 | 153.7 | 4.59931979 | 153.7 | 4.59931979 | 153.7 | 4.11879384 | 153.7 | 9.27709279 | | | 153.70 | 3.95208076 | 153.7 | 7.11962935 | 153.7 | 3.95208076 |
| 153.8 | 1.04931176 | 153.8 | 4.57970648 | 153.8 | 4.57970648 | 153.8 | 4.11879384 | 153.8 | 9.28689944 | | | 153.80 | 3.97169406 | 153.8 | 7.129436 | 153.8 | 3.97169406 |
| 153.9 | 1.06892507 | 153.9 | 4.56989983 | 153.9 | 4.56989983 | 153.9 | 4.11879384 | 153.9 | 9.2967061 | | | 153.90 | 3.97169406 | 153.9 | 7.13924266 | 153.9 | 3.97169406 |
| 154 | 1.05911842 | 154 | 4.56989983 | 154 | 4.56989983 | 154 | 4.13840714 | 154 | 9.30651275 | | | 154.00 | 3.96188741 | 154 | 7.17846926 | 154 | 3.96188741 |
| 154.1 | 1.08853837 | 154.1 | 4.59931979 | 154.1 | 4.59931979 | 154.1 | 4.13840714 | 154.1 | 9.3163194 | | | 154.10 | 3.95208076 | 154.1 | 7.18827592 | 154.1 | 3.95208076 |
| 154.2 | 1.10815168 | 154.2 | 4.60912644 | 154.2 | 4.60912644 | 154.2 | 4.12860049 | 154.2 | 9.35554601 | | | 154.20 | 3.96188741 | 154.2 | 7.17846926 | 154.2 | 3.96188741 |
| 154.3 | 1.08853837 | 154.3 | 4.62873974 | 154.3 | 4.62873974 | 154.3 | 4.11879384 | 154.3 | 9.37515931 | | | 154.30 | 3.97169406 | 154.3 | 7.16866261 | 154.3 | 3.97169406 |
| 154.4 | 1.03950511 | 154.4 | 4.64835305 | 154.4 | 4.64835305 | 154.4 | 4.15802045 | 154.4 | 9.40457927 | | | 154.40 | 3.99130736 | 154.4 | 7.16866261 | 154.4 | 3.99130736 |
| 154.5 | 1.03950511 | 154.5 | 4.6385464 | 154.5 | 4.6385464 | 154.5 | 4.1678271 | 154.5 | 9.39477262 | | | 154.50 | 3.99130736 | 154.5 | 7.17846926 | 154.5 | 3.99130736 |
| 154.6 | 1.06892507 | 154.6 | 4.6385464 | 154.6 | 4.6385464 | 154.6 | 4.17763375 | 154.6 | 9.38496596 | | | 154.60 | 4.00111402 | 154.6 | 7.18827592 | 154.6 | 4.00111402 |
| 154.7 | 1.06892507 | 154.7 | 4.60912644 | 154.7 | 4.60912644 | 154.7 | 4.1874404 | 154.7 | 9.38496596 | | | 154.70 | 4.00111402 | 154.7 | 7.20788922 | 154.7 | 4.00111402 |
| 154.8 | 1.04931176 | 154.8 | 4.61893309 | 154.8 | 4.61893309 | 154.8 | 4.15802045 | 154.8 | 9.39477262 | | | 154.80 | 4.00111402 | 154.8 | 7.20788922 | 154.8 | 4.00111402 |
| 154.9 | 1.02969846 | 154.9 | 4.64835305 | 154.9 | 4.64835305 | 154.9 | 4.17763375 | 154.9 | 9.43399922 | | | 154.90 | 4.01092067 | 154.9 | 7.22750252 | 154.9 | 4.01092067 |
| 155 | 1.07873172 | 155 | 4.61893309 | 155 | 4.61893309 | 155 | 4.1874404 | 155 | 9.47322583 | | | 155.00 | 3.99130736 | 155 | 7.21769587 | 155 | 3.99130736 |
| 155.1 | 1.07873172 | 155.1 | 4.62873974 | 155.1 | 4.62873974 | 155.1 | 4.1678271 | 155.1 | 9.47322583 | | | 155.10 | 4.02072732 | 155.1 | 7.22750252 | 155.1 | 4.02072732 |
| 155.2 | 1.06892507 | 155.2 | 4.64835305 | 155.2 | 4.64835305 | 155.2 | 4.15802045 | 155.2 | 9.48303248 | | | 155.20 | 3.99130736 | 155.2 | 7.27653578 | 155.2 | 3.99130736 |
| 155.3 | 1.06892507 | 155.3 | 4.64835305 | 155.3 | 4.64835305 | 155.3 | 4.1678271 | 155.3 | 9.51245244 | | | 155.30 | 4.00111402 | 155.3 | 7.26672913 | 155.3 | 4.00111402 |
| 155.4 | 1.09834502 | 155.4 | 4.6581597 | 155.4 | 4.6581597 | 155.4 | 4.1482138 | 155.4 | 9.52225909 | | | 155.40 | 4.04034062 | 155.4 | 7.28634244 | 155.4 | 4.04034062 |
| 155.5 | 1.09834502 | 155.5 | 4.66796635 | 155.5 | 4.66796635 | 155.5 | 4.19724706 | 155.5 | 9.5418724 | | | 155.50 | 4.03053397 | 155.5 | 7.27653578 | 155.5 | 4.03053397 |
| 155.6 | 1.06892507 | 155.6 | 4.677773 | 155.6 | 4.677773 | 155.6 | 4.1678271 | 155.6 | 9.55167905 | | | 155.60 | 4.01092067 | 155.6 | 7.29614909 | 155.6 | 4.01092067 |
| 155.7 | 1.10815168 | 155.7 | 4.64835305 | 155.7 | 4.64835305 | 155.7 | 4.15802045 | 155.7 | 9.53206574 | | | 155.70 | 4.04034062 | 155.7 | 7.25692248 | 155.7 | 4.04034062 |
| 155.8 | 1.06892507 | 155.8 | 4.66796635 | 155.8 | 4.66796635 | 155.8 | 4.1678271 | 155.8 | 9.53206574 | | | 155.80 | 4.05995393 | 155.8 | 7.26672913 | 155.8 | 4.05995393 |

| | | | | | | | | | | | | | | | | | |
|---|---|---|---|---|---|---|---|---|---|---|---|---|---|---|---|---|---|
| 155.9 | 1.05911842 | 155.9 | 4.70719296 | 155.9 | 4.70719296 | 155.9 | 4.17763375 | 155.9 | 9.55167905 | | | 155.90 | 4.05014728 | 155.9 | 7.32556904 | 155.9 | 4.05014728 |
| 156 | 1.05911842 | 156 | 4.73661292 | 156 | 4.73661292 | 156 | 4.19724706 | 156 | 9.59090566 | | | 156.00 | 4.05014728 | 156 | 7.31576239 | 156 | 4.05014728 |
| 156.1 | 1.03950511 | 156.1 | 4.74641957 | 156.1 | 4.74641957 | 156.1 | 4.21686036 | 156.1 | 9.60071231 | | | 156.10 | 4.05014728 | 156.1 | 7.31576239 | 156.1 | 4.05014728 |
| 156.2 | 1.07873172 | 156.2 | 4.70719296 | 156.2 | 4.70719296 | 156.2 | 4.24628032 | 156.2 | 9.60071231 | | | 156.20 | 4.04034062 | 156.2 | 7.31576239 | 156.2 | 4.04034062 |
| 156.3 | 1.09834502 | 156.3 | 4.73661292 | 156.3 | 4.73661292 | 156.3 | 4.19724706 | 156.3 | 9.62032561 | | | 156.30 | 4.05995393 | 156.3 | 7.31576239 | 156.3 | 4.05995393 |
| 156.4 | 1.07873172 | 156.4 | 4.77583952 | 156.4 | 4.77583952 | 156.4 | 4.15802045 | 156.4 | 9.63013226 | | | 156.40 | 4.05995393 | 156.4 | 7.32556904 | 156.4 | 4.05995393 |
| 156.5 | 1.08853837 | 156.5 | 4.75622622 | 156.5 | 4.75622622 | 156.5 | 4.1482138 | 156.5 | 9.63993892 | | | 156.50 | 4.05995393 | 156.5 | 7.354989 | 156.5 | 4.05995393 |
| 156.6 | 1.09834502 | 156.6 | 4.75622622 | 156.6 | 4.75622622 | 156.6 | 4.19724706 | 156.6 | 9.66935887 | | | 156.60 | 4.05014728 | 156.6 | 7.3746023 | 156.6 | 4.05014728 |
| 156.7 | 1.09834502 | 156.7 | 4.69738631 | 156.7 | 4.69738631 | 156.7 | 4.21686036 | 156.7 | 9.67916552 | | | 156.70 | 4.06976058 | 156.7 | 7.38440896 | 156.7 | 4.06976058 |
| 156.8 | 1.12776498 | 156.8 | 4.74641957 | 156.8 | 4.74641957 | 156.8 | 4.19724706 | 156.8 | 9.70858548 | | | 156.80 | 4.05014728 | 156.8 | 7.40402226 | 156.8 | 4.05014728 |
| 156.9 | 1.14737828 | 156.9 | 4.76603287 | 156.9 | 4.76603287 | 156.9 | 4.19724706 | 156.9 | 9.72819878 | | | 156.90 | 4.06976058 | 156.9 | 7.3746023 | 156.9 | 4.06976058 |
| 157 | 1.09834502 | 157 | 4.73661292 | 157 | 4.73661292 | 157 | 4.1874404 | 157 | 9.69877883 | | | 157.00 | 4.06976058 | 157 | 7.3353757 | 157 | 4.06976058 |
| 157.1 | 1.10815168 | 157.1 | 4.74641957 | 157.1 | 4.74641957 | 157.1 | 4.17763375 | 157.1 | 9.72819878 | | | 157.10 | 4.05014728 | 157.1 | 7.38440896 | 157.1 | 4.05014728 |
| 157.2 | 1.10815168 | 157.2 | 4.77583952 | 157.2 | 4.77583952 | 157.2 | 4.19724706 | 157.2 | 9.74781209 | | | 157.20 | 4.07956723 | 157.2 | 7.40402226 | 157.2 | 4.07956723 |
| 157.3 | 1.11795833 | 157.3 | 4.77583952 | 157.3 | 4.77583952 | 157.3 | 4.20705371 | 157.3 | 9.7870387 | | | 157.30 | 4.09918054 | 157.3 | 7.40402226 | 157.3 | 4.09918054 |
| 157.4 | 1.10815168 | 157.4 | 4.76603287 | 157.4 | 4.76603287 | 157.4 | 4.1874404 | 157.4 | 9.7870387 | | | 157.40 | 4.05995393 | 157.4 | 7.39421561 | 157.4 | 4.05995393 |
| 157.5 | 1.09834502 | 157.5 | 4.80525948 | 157.5 | 4.80525948 | 157.5 | 4.21686036 | 157.5 | 9.7870387 | | | 157.50 | 4.08937388 | 157.5 | 7.3746023 | 157.5 | 4.08937388 |
| 157.6 | 1.12776498 | 157.6 | 4.80525948 | 157.6 | 4.80525948 | 157.6 | 4.25608697 | 157.6 | 9.79684535 | | | 157.60 | 4.07956723 | 157.6 | 7.38440896 | 157.6 | 4.07956723 |
| 157.7 | 1.11795833 | 157.7 | 4.79545283 | 157.7 | 4.79545283 | 157.7 | 4.22666701 | 157.7 | 9.8262653 | | | 157.70 | 4.05995393 | 157.7 | 7.41382891 | 157.7 | 4.05995393 |
| 157.8 | 1.12776498 | 157.8 | 4.79545283 | 157.8 | 4.79545283 | 157.8 | 4.23647366 | 157.8 | 9.83607196 | | | 157.80 | 4.05995393 | 157.8 | 7.42363556 | 157.8 | 4.05995393 |
| 157.9 | 1.10815168 | 157.9 | 4.80525948 | 157.9 | 4.80525948 | 157.9 | 4.24628032 | 157.9 | 9.83607196 | | | 157.90 | 4.06976058 | 157.9 | 7.46286217 | 157.9 | 4.06976058 |
| 158 | 1.09834502 | 158 | 4.80525948 | 158 | 4.80525948 | 158 | 4.23647366 | 158 | 9.85568526 | | | 158.00 | 4.07956723 | 158 | 7.47266882 | 158 | 4.07956723 |
| 158.1 | 1.08853837 | 158.1 | 4.82487278 | 158.1 | 4.82487278 | 158.1 | 4.22666701 | 158.1 | 9.86549191 | | | 158.10 | 4.09918054 | 158.1 | 7.45305552 | 158.1 | 4.09918054 |
| 158.2 | 1.07873172 | 158.2 | 4.81506613 | 158.2 | 4.81506613 | 158.2 | 4.23647366 | 158.2 | 9.86549191 | | | 158.20 | 4.11879384 | 158.2 | 7.45305552 | 158.2 | 4.11879384 |
| 158.3 | 1.07873172 | 158.3 | 4.84448609 | 158.3 | 4.84448609 | 158.3 | 4.21686036 | 158.3 | 9.88510522 | | | 158.30 | 4.13840714 | 158.3 | 7.50208878 | 158.3 | 4.13840714 |
| 158.4 | 1.07873172 | 158.4 | 4.83467944 | 158.4 | 4.83467944 | 158.4 | 4.22666701 | 158.4 | 9.89491187 | | | 158.40 | 4.10898719 | 158.4 | 7.49228213 | 158.4 | 4.10898719 |
| 158.5 | 1.06892507 | 158.5 | 4.81506613 | 158.5 | 4.81506613 | 158.5 | 4.23647366 | 158.5 | 9.90471852 | | | 158.50 | 4.12860049 | 158.5 | 7.47266882 | 158.5 | 4.12860049 |
| 158.6 | 1.13757163 | 158.6 | 4.82487278 | 158.6 | 4.82487278 | 158.6 | 4.24628032 | 158.6 | 9.92433182 | | | 158.60 | 4.11879384 | 158.6 | 7.50208878 | 158.6 | 4.11879384 |
| 158.7 | 1.14737828 | 158.7 | 4.82487278 | 158.7 | 4.82487278 | 158.7 | 4.22666701 | 158.7 | 9.96355843 | | | 158.70 | 4.10898719 | 158.7 | 7.54131539 | 158.7 | 4.10898719 |
| 158.8 | 1.09834502 | 158.8 | 4.8837127 | 158.8 | 4.8837127 | 158.8 | 4.22666701 | 158.8 | 9.96355843 | | | 158.80 | 4.09918054 | 158.8 | 7.52170208 | 158.8 | 4.09918054 |
| 158.9 | 1.10815168 | 158.9 | 4.903326 | 158.9 | 4.903326 | 158.9 | 4.22666701 | 158.9 | 9.98317174 | | | 158.90 | 4.08937388 | 158.9 | 7.51189543 | 158.9 | 4.08937388 |
| 159 | 1.09834502 | 159 | 4.903326 | 159 | 4.903326 | 159 | 4.24628032 | 159 | 10.002785 | | | 159.00 | 4.08937388 | 159 | 7.51189543 | 159 | 4.08937388 |
| 159.1 | 1.10815168 | 159.1 | 4.91313265 | 159.1 | 4.91313265 | 159.1 | 4.24628032 | 159.1 | 10.0223983 | | | 159.10 | 4.11879384 | 159.1 | 7.51189543 | 159.1 | 4.11879384 |
| 159.2 | 1.07873172 | 159.2 | 4.9229393 | 159.2 | 4.9229393 | 159.2 | 4.24628032 | 159.2 | 10.0125917 | | | 159.20 | 4.15802045 | 159.2 | 7.54131539 | 159.2 | 4.15802045 |
| 159.3 | 1.05911842 | 159.3 | 4.903326 | 159.3 | 4.903326 | 159.3 | 4.27570027 | 159.3 | 10.0223983 | | | 159.30 | 4.13840714 | 159.3 | 7.54131539 | 159.3 | 4.13840714 |
| 159.4 | 1.06892507 | 159.4 | 4.89351935 | 159.4 | 4.89351935 | 159.4 | 4.28550692 | 159.4 | 10.061625 | | | 159.40 | 4.15802045 | 159.4 | 7.52170208 | 159.4 | 4.15802045 |
| 159.5 | 1.07873172 | 159.5 | 4.903326 | 159.5 | 4.903326 | 159.5 | 4.26589362 | 159.5 | 10.0420116 | | | 159.50 | 4.13840714 | 159.5 | 7.56092869 | 159.5 | 4.13840714 |
| 159.6 | 1.08853837 | 159.6 | 4.9229393 | 159.6 | 4.9229393 | 159.6 | 4.24628032 | 159.6 | 10.0420116 | | | 159.60 | 4.11879384 | 159.6 | 7.6001553 | 159.6 | 4.11879384 |
| 159.7 | 1.12776498 | 159.7 | 4.94255261 | 159.7 | 4.94255261 | 159.7 | 4.23647366 | 159.7 | 10.0812383 | | | 159.70 | 4.12860049 | 159.7 | 7.60996195 | 159.7 | 4.12860049 |
| 159.8 | 1.10815168 | 159.8 | 4.95235926 | 159.8 | 4.95235926 | 159.8 | 4.27570027 | 159.8 | 10.0812383 | | | 159.80 | 4.11879384 | 159.8 | 7.6197686 | 159.8 | 4.11879384 |
| 159.9 | 1.06892507 | 159.9 | 4.98177922 | 159.9 | 4.98177922 | 159.9 | 4.24628032 | 159.9 | 10.1008516 | | | 159.90 | 4.15802045 | 159.9 | 7.59034865 | 159.9 | 4.15802045 |
| 160 | 1.07873172 | 160 | 4.903326 | 160 | 4.903326 | 160 | 4.20705371 | 160 | 10.1204649 | | | 160.00 | 4.1874404 | 160 | 7.6001553 | 160 | 4.1874404 |
| 160.1 | 1.09834502 | 160.1 | 4.91313265 | 160.1 | 4.91313265 | 160.1 | 4.21686036 | 160.1 | 10.1400782 | | | 160.10 | 4.1678271 | 160.1 | 7.6197686 | 160.1 | 4.1678271 |
| 160.2 | 1.14737828 | 160.2 | 4.96216591 | 160.2 | 4.96216591 | 160.2 | 4.22666701 | 160.2 | 10.1204649 | | | 160.20 | 4.20705371 | 160.2 | 7.6197686 | 160.2 | 4.20705371 |
| 160.3 | 1.13757163 | 160.3 | 4.95235926 | 160.3 | 4.95235926 | 160.3 | 4.24628032 | 160.3 | 10.1302715 | | | 160.30 | 4.21686036 | 160.3 | 7.60996195 | 160.3 | 4.21686036 |
| 160.4 | 1.11795833 | 160.4 | 5.00139252 | 160.4 | 5.00139252 | 160.4 | 4.28550692 | 160.4 | 10.1596915 | | | 160.40 | 4.17763375 | 160.4 | 7.6197686 | 160.4 | 4.17763375 |
| 160.5 | 1.13757163 | 160.5 | 5.04061913 | 160.5 | 5.04061913 | 160.5 | 4.29531358 | 160.5 | 10.1793048 | | | 160.50 | 4.15802045 | 160.5 | 7.59034865 | 160.5 | 4.15802045 |
| 160.6 | 1.13757163 | 160.6 | 4.99158587 | 160.6 | 4.99158587 | 160.6 | 4.25608697 | 160.6 | 10.1891114 | | | 160.60 | 4.17763375 | 160.6 | 7.6001553 | 160.6 | 4.17763375 |
| 160.7 | 1.12776498 | 160.7 | 5.01119917 | 160.7 | 5.01119917 | 160.7 | 4.21686036 | 160.7 | 10.228338 | | | 160.70 | 4.1874404 | 160.7 | 7.64918856 | 160.7 | 4.1874404 |
| 160.8 | 1.13757163 | 160.8 | 5.02100582 | 160.8 | 5.02100582 | 160.8 | 4.20705371 | 160.8 | 10.2381447 | | | 160.80 | 4.15802045 | 160.8 | 7.65899521 | 160.8 | 4.15802045 |
| 160.9 | 1.14737828 | 160.9 | 5.00139252 | 160.9 | 5.00139252 | 160.9 | 4.22666701 | 160.9 | 10.2381447 | | | 160.90 | 4.15802045 | 160.9 | 7.66880186 | 160.9 | 4.15802045 |
| 161 | 1.16699159 | 161 | 4.99158587 | 161 | 4.99158587 | 161 | 4.25608697 | 161 | 10.2479513 | | | 161.00 | 4.1482138 | 161 | 7.65899521 | 161 | 4.1482138 |
| 161.1 | 1.18660489 | 161.1 | 5.02100582 | 161.1 | 5.02100582 | 161.1 | 4.25608697 | 161.1 | 10.2871779 | | | 161.10 | 4.1874404 | 161.1 | 7.66880186 | 161.1 | 4.1874404 |
| 161.2 | 1.10815168 | 161.2 | 5.03081248 | 161.2 | 5.03081248 | 161.2 | 4.25608697 | 161.2 | 10.2871779 | | | 161.20 | 4.21686036 | 161.2 | 7.65899521 | 161.2 | 4.21686036 |
| 161.3 | 1.11795833 | 161.3 | 5.05042578 | 161.3 | 5.05042578 | 161.3 | 4.24628032 | 161.3 | 10.2773713 | | | 161.30 | 4.21686036 | 161.3 | 7.65899521 | 161.3 | 4.21686036 |
| 161.4 | 1.14737828 | 161.4 | 5.04061913 | 161.4 | 5.04061913 | 161.4 | 4.23647366 | 161.4 | 10.2871779 | | | 161.40 | 4.22666701 | 161.4 | 7.67860852 | 161.4 | 4.22666701 |
| 161.5 | 1.13757163 | 161.5 | 5.06023243 | 161.5 | 5.06023243 | 161.5 | 4.27570027 | 161.5 | 10.2773713 | | | 161.50 | 4.22666701 | 161.5 | 7.68841517 | 161.5 | 4.22666701 |
| 161.6 | 1.10815168 | 161.6 | 5.04061913 | 161.6 | 5.04061913 | 161.6 | 4.26589362 | 161.6 | 10.3067913 | | | 161.60 | 4.19724706 | 161.6 | 7.70802847 | 161.6 | 4.19724706 |
| 161.7 | 1.12776498 | 161.7 | 5.05042578 | 161.7 | 5.05042578 | 161.7 | 4.26589362 | 161.7 | 10.3165979 | | | 161.70 | 4.17763375 | 161.7 | 7.70802847 | 161.7 | 4.17763375 |
| 161.8 | 1.14737828 | 161.8 | 5.05042578 | 161.8 | 5.05042578 | 161.8 | 4.28550692 | 161.8 | 10.3264046 | | | 161.80 | 4.1874404 | 161.8 | 7.71783512 | 161.8 | 4.1874404 |
| 161.9 | 1.14737828 | 161.9 | 5.04061913 | 161.9 | 5.04061913 | 161.9 | 4.28550692 | 161.9 | 10.3460179 | | | 161.90 | 4.22666701 | 161.9 | 7.73744843 | 161.9 | 4.22666701 |

| | | | | | | | | | | | | | | | | | |
|---|---|---|---|---|---|---|---|---|---|---|---|---|---|---|---|---|---|
| 162 | 1.15718494 | 162 | 5.02100582 | 162 | 5.02100582 | 162 | 4.28550692 | 162 | 10.3362112 | | | 162.00 | 4.24628032 | 162 | 7.73744843 | 162 | 4.24628032 |
| 162.1 | 1.13757163 | 162.1 | 5.04061913 | 162.1 | 5.04061913 | 162.1 | 4.24628032 | 162.1 | 10.3754378 | | | 162.10 | 4.23647366 | 162.1 | 7.72764178 | 162.1 | 4.23647366 |
| 162.2 | 1.10815168 | 162.2 | 5.07003908 | 162.2 | 5.07003908 | 162.2 | 4.27570027 | 162.2 | 10.3852445 | | | 162.20 | 4.22666701 | 162.2 | 7.75706173 | 162.2 | 4.22666701 |
| 162.3 | 1.12776498 | 162.3 | 5.06023243 | 162.3 | 5.06023243 | 162.3 | 4.28550692 | 162.3 | 10.3656312 | | | 162.30 | 4.22666701 | 162.3 | 7.78648169 | 162.3 | 4.22666701 |
| 162.4 | 1.11795833 | 162.4 | 5.07003908 | 162.4 | 5.07003908 | 162.4 | 4.22666701 | 162.4 | 10.4048578 | | | 162.40 | 4.23647366 | 162.4 | 7.79628834 | 162.4 | 4.23647366 |
| 162.5 | 1.10815168 | 162.5 | 5.08965239 | 162.5 | 5.08965239 | 162.5 | 4.21686036 | 162.5 | 10.4342777 | | | 162.50 | 4.23647366 | 162.5 | 7.78648169 | 162.5 | 4.23647366 |
| 162.6 | 1.12776498 | 162.6 | 5.08965239 | 162.6 | 5.08965239 | 162.6 | 4.24628032 | 162.6 | 10.4440844 | | | 162.60 | 4.23647366 | 162.6 | 7.76686838 | 162.6 | 4.23647366 |
| 162.7 | 1.12776498 | 162.7 | 5.07984574 | 162.7 | 5.07984574 | 162.7 | 4.21686036 | 162.7 | 10.4636977 | | | 162.70 | 4.20705371 | 162.7 | 7.77667504 | 162.7 | 4.20705371 |
| 162.8 | 1.13757163 | 162.8 | 5.09945904 | 162.8 | 5.09945904 | 162.8 | 4.20705371 | 162.8 | 10.4931176 | | | 162.80 | 4.23647366 | 162.8 | 7.81590164 | 162.8 | 4.23647366 |
| 162.9 | 1.13757163 | 162.9 | 5.09945904 | 162.9 | 5.09945904 | 162.9 | 4.22666701 | 162.9 | 10.4931176 | | | 162.90 | 4.26589362 | 162.9 | 7.83551495 | 162.9 | 4.26589362 |
| 163 | 1.12776498 | 163 | 5.10926569 | 163 | 5.10926569 | 163 | 4.22666701 | 163 | 10.4931176 | | | 163.00 | 4.25608697 | 163 | 7.81590164 | 163 | 4.25608697 |
| 163.1 | 1.13757163 | 163.1 | 5.09945904 | 163.1 | 5.09945904 | 163.1 | 4.22666701 | 163.1 | 10.5421509 | | | 163.10 | 4.25608697 | 163.1 | 7.80609499 | 163.1 | 4.25608697 |
| 163.2 | 1.13757163 | 163.2 | 5.10926569 | 163.2 | 5.10926569 | 163.2 | 4.21686036 | 163.2 | 10.5519576 | | | 163.20 | 4.26589362 | 163.2 | 7.79628834 | 163.2 | 4.26589362 |
| 163.3 | 1.13757163 | 163.3 | 5.11907234 | 163.3 | 5.11907234 | 163.3 | 4.24628032 | 163.3 | 10.5617642 | | | 163.30 | 4.26589362 | 163.3 | 7.8257083 | 163.3 | 4.26589362 |
| 163.4 | 1.14737828 | 163.4 | 5.11907234 | 163.4 | 5.11907234 | 163.4 | 4.25608697 | 163.4 | 10.5519576 | | | 163.40 | 4.26589362 | 163.4 | 7.87474156 | 163.4 | 4.26589362 |
| 163.5 | 1.16699159 | 163.5 | 5.13868565 | 163.5 | 5.13868565 | 163.5 | 4.27570027 | 163.5 | 10.5519576 | | | 163.50 | 4.27570027 | 163.5 | 7.87474156 | 163.5 | 4.27570027 |
| 163.6 | 1.17679824 | 163.6 | 5.15829895 | 163.6 | 5.15829895 | 163.6 | 4.31492688 | 163.6 | 10.5421509 | | | 163.60 | 4.28550692 | 163.6 | 7.87474156 | 163.6 | 4.28550692 |
| 163.7 | 1.17679824 | 163.7 | 5.18771891 | 163.7 | 5.18771891 | 163.7 | 4.33454018 | 163.7 | 10.5421509 | | | 163.70 | 4.25608697 | 163.7 | 7.88454821 | 163.7 | 4.25608697 |
| 163.8 | 1.17679824 | 163.8 | 5.18771891 | 163.8 | 5.18771891 | 163.8 | 4.26589362 | 163.8 | 10.5715709 | | | 163.80 | 4.27570027 | 163.8 | 7.88454821 | 163.8 | 4.27570027 |
| 163.9 | 1.19641154 | 163.9 | 5.19752556 | 163.9 | 5.19752556 | 163.9 | 4.29531358 | 163.9 | 10.5813775 | | | 163.90 | 4.26589362 | 163.9 | 7.88454821 | 163.9 | 4.26589362 |
| 164 | 1.18660489 | 164 | 5.19752556 | 164 | 5.19752556 | 164 | 4.32473353 | 164 | 10.5813775 | | | 164.00 | 4.28550692 | 164 | 7.90416151 | 164 | 4.28550692 |
| 164.1 | 1.18660489 | 164.1 | 5.19752556 | 164.1 | 5.19752556 | 164.1 | 4.30512023 | 164.1 | 10.5813775 | | | 164.10 | 4.29531358 | 164.1 | 7.90416151 | 164.1 | 4.29531358 |
| 164.2 | 1.16699159 | 164.2 | 5.19752556 | 164.2 | 5.19752556 | 164.2 | 4.30512023 | 164.2 | 10.6009908 | | | 164.20 | 4.29531358 | 164.2 | 7.90416151 | 164.2 | 4.29531358 |
| 164.3 | 1.16699159 | 164.3 | 5.19752556 | 164.3 | 5.19752556 | 164.3 | 4.31492688 | 164.3 | 10.6107975 | | | 164.30 | 4.29531358 | 164.3 | 7.89435486 | 164.3 | 4.29531358 |
| 164.4 | 1.16699159 | 164.4 | 5.24655882 | 164.4 | 5.24655882 | 164.4 | 4.34434684 | 164.4 | 10.6598307 | | | 164.40 | 4.27570027 | 164.4 | 7.91396816 | 164.4 | 4.27570027 |
| 164.5 | 1.13757163 | 164.5 | 5.28578543 | 164.5 | 5.28578543 | 164.5 | 4.35415349 | 164.5 | 10.679444 | | | 164.50 | 4.28550692 | 164.5 | 7.93358147 | 164.5 | 4.28550692 |
| 164.6 | 1.12776498 | 164.6 | 5.30539873 | 164.6 | 5.30539873 | 164.6 | 4.32473353 | 164.6 | 10.6892507 | | | 164.60 | 4.31492688 | 164.6 | 7.93358147 | 164.6 | 4.31492688 |
| 164.7 | 1.14737828 | 164.7 | 5.28578543 | 164.7 | 5.28578543 | 164.7 | 4.30512023 | 164.7 | 10.679444 | | | 164.70 | 4.31492688 | 164.7 | 7.94338812 | 164.7 | 4.31492688 |
| 164.8 | 1.15718494 | 164.8 | 5.26617212 | 164.8 | 5.26617212 | 164.8 | 4.28550692 | 164.8 | 10.7186706 | | | 164.80 | 4.29531358 | 164.8 | 7.92377482 | 164.8 | 4.29531358 |
| 164.9 | 1.15718494 | 164.9 | 5.30539873 | 164.9 | 5.30539873 | 164.9 | 4.27570027 | 164.9 | 10.7677039 | | | 164.90 | 4.31492688 | 164.9 | 7.91396816 | 164.9 | 4.31492688 |
| 165 | 1.16699159 | 165 | 5.29559208 | 165 | 5.29559208 | 165 | 4.34434684 | 165 | 10.7578972 | | | 165.00 | 4.32473353 | 165 | 7.92377482 | 165 | 4.32473353 |
| 165.1 | 1.18660489 | 165.1 | 5.29559208 | 165.1 | 5.29559208 | 165.1 | 4.31492688 | 165.1 | 10.7578972 | | | 165.10 | 4.32473353 | 165.1 | 7.98261473 | 165.1 | 4.32473353 |
| 165.2 | 1.19641154 | 165.2 | 5.31520538 | 165.2 | 5.31520538 | 165.2 | 4.32473353 | 165.2 | 10.7578972 | | | 165.20 | 4.30512023 | 165.2 | 7.99242138 | 165.2 | 4.30512023 |
| 165.3 | 1.18660489 | 165.3 | 5.31520538 | 165.3 | 5.31520538 | 165.3 | 4.31492688 | 165.3 | 10.7677039 | | | 165.30 | 4.30512023 | 165.3 | 7.99242138 | 165.3 | 4.30512023 |
| 165.4 | 1.14737828 | 165.4 | 5.30539873 | 165.4 | 5.30539873 | 165.4 | 4.32473353 | 165.4 | 10.7775105 | | | 165.40 | 4.32473353 | 165.4 | 7.98261473 | 165.4 | 4.32473353 |
| 165.5 | 1.16699159 | 165.5 | 5.30539873 | 165.5 | 5.30539873 | 165.5 | 4.31492688 | 165.5 | 10.7578972 | | | 165.50 | 4.33454018 | 165.5 | 7.99242138 | 165.5 | 4.33454018 |
| 165.6 | 1.17679824 | 165.6 | 5.35443199 | 165.6 | 5.35443199 | 165.6 | 4.29531358 | 165.6 | 10.7480906 | | | 165.60 | 4.33454018 | 165.6 | 8.02184134 | 165.6 | 4.33454018 |
| 165.7 | 1.17679824 | 165.7 | 5.36423864 | 165.7 | 5.36423864 | 165.7 | 4.31492688 | 165.7 | 10.7480906 | | | 165.70 | 4.34434684 | 165.7 | 8.03164799 | 165.7 | 4.34434684 |
| 165.8 | 1.18660489 | 165.8 | 5.34462534 | 165.8 | 5.34462534 | 165.8 | 4.35415349 | 165.8 | 10.7873172 | | | 165.80 | 4.34434684 | 165.8 | 7.98261473 | 165.8 | 4.34434684 |
| 165.9 | 1.18660489 | 165.9 | 5.35443199 | 165.9 | 5.35443199 | 165.9 | 4.32473353 | 165.9 | 10.8167372 | | | 165.90 | 4.32473353 | 165.9 | 7.98261473 | 165.9 | 4.32473353 |
| 166 | 1.19641154 | 166 | 5.36423864 | 166 | 5.36423864 | 166 | 4.31492688 | 166 | 10.8167372 | | | 166.00 | 4.35415349 | 166 | 8.00222803 | 166 | 4.35415349 |
| 166.1 | 1.17679824 | 166.1 | 5.34462534 | 166.1 | 5.34462534 | 166.1 | 4.36396014 | 166.1 | 10.8461571 | | | 166.10 | 4.34434684 | 166.1 | 8.02184134 | 166.1 | 4.34434684 |
| 166.2 | 1.17679824 | 166.2 | 5.34462534 | 166.2 | 5.34462534 | 166.2 | 4.29531358 | 166.2 | 10.8559638 | | | 166.20 | 4.35415349 | 166.2 | 8.03164799 | 166.2 | 4.35415349 |
| 166.3 | 1.18660489 | 166.3 | 5.36423864 | 166.3 | 5.36423864 | 166.3 | 4.37376679 | 166.3 | 10.8657704 | | | 166.30 | 4.37376679 | 166.3 | 8.05126129 | 166.3 | 4.37376679 |
| 166.4 | 1.19641154 | 166.4 | 5.3740453 | 166.4 | 5.3740453 | 166.4 | 4.4129934 | 166.4 | 10.8559638 | | | 166.40 | 4.32473353 | 166.4 | 8.05126129 | 166.4 | 4.32473353 |
| 166.5 | 1.18660489 | 166.5 | 5.38385195 | 166.5 | 5.38385195 | 166.5 | 4.38357344 | 166.5 | 10.8657704 | | | 166.50 | 4.31492688 | 166.5 | 8.06106794 | 166.5 | 4.31492688 |
| 166.6 | 1.18660489 | 166.6 | 5.38385195 | 166.6 | 5.38385195 | 166.6 | 4.38357344 | 166.6 | 10.8755771 | | | 166.60 | 4.36396014 | 166.6 | 8.05126129 | 166.6 | 4.36396014 |
| 166.7 | 1.14737828 | 166.7 | 5.38385195 | 166.7 | 5.38385195 | 166.7 | 4.38357344 | 166.7 | 10.904997 | | | 166.70 | 4.36396014 | 166.7 | 8.06106794 | 166.7 | 4.36396014 |
| 166.8 | 1.15718494 | 166.8 | 5.40346525 | 166.8 | 5.40346525 | 166.8 | 4.40318675 | 166.8 | 10.9148037 | | | 166.80 | 4.36396014 | 166.8 | 8.06106794 | 166.8 | 4.36396014 |
| 166.9 | 1.18660489 | 166.9 | 5.4132719 | 166.9 | 5.4132719 | 166.9 | 4.45222001 | 166.9 | 10.9246103 | | | 166.90 | 4.38357344 | 166.9 | 8.0708746 | 166.9 | 4.38357344 |
| 167 | 1.19641154 | 167 | 5.43288521 | 167 | 5.43288521 | 167 | 4.45222001 | 167 | 10.904997 | | | 167.00 | 4.37376679 | 167 | 8.06106794 | 167 | 4.37376679 |
| 167.1 | 1.17679824 | 167.1 | 5.43288521 | 167.1 | 5.43288521 | 167.1 | 4.45222001 | 167.1 | 10.9148037 | | | 167.10 | 4.34434684 | 167.1 | 8.11990786 | 167.1 | 4.34434684 |
| 167.2 | 1.2062182 | 167.2 | 5.45249851 | 167.2 | 5.45249851 | 167.2 | 4.45222001 | 167.2 | 10.934417 | | | 167.20 | 4.34434684 | 167.2 | 8.15913446 | 167.2 | 4.34434684 |
| 167.3 | 1.2062182 | 167.3 | 5.45249851 | 167.3 | 5.45249851 | 167.3 | 4.4129934 | 167.3 | 10.9540303 | | | 167.30 | 4.38357344 | 167.3 | 8.13952116 | 167.3 | 4.38357344 |
| 167.4 | 1.2258315 | 167.4 | 5.43288521 | 167.4 | 5.43288521 | 167.4 | 4.37376679 | 167.4 | 10.9736436 | | | 167.40 | 4.4129934 | 167.4 | 8.12971451 | 167.4 | 4.4129934 |
| 167.5 | 1.2454448 | 167.5 | 5.45249851 | 167.5 | 5.45249851 | 167.5 | 4.30512023 | 167.5 | 10.9932569 | | | 167.50 | 4.37376679 | 167.5 | 8.11990786 | 167.5 | 4.37376679 |
| 167.6 | 1.21602485 | 167.6 | 5.45249851 | 167.6 | 5.45249851 | 167.6 | 4.27570027 | 167.6 | 10.9736436 | | | 167.60 | 4.38357344 | 167.6 | 8.10029455 | 167.6 | 4.38357344 |
| 167.7 | 1.2258315 | 167.7 | 5.46230516 | 167.7 | 5.46230516 | 167.7 | 4.32473353 | 167.7 | 11.0030635 | | | 167.70 | 4.4129934 | 167.7 | 8.10029455 | 167.7 | 4.4129934 |
| 167.8 | 1.2062182 | 167.8 | 5.48191847 | 167.8 | 5.48191847 | 167.8 | 4.35415349 | 167.8 | 11.0422902 | | | 167.80 | 4.4326067 | 167.8 | 8.15913446 | 167.8 | 4.4326067 |
| 167.9 | 1.2062182 | 167.9 | 5.50153177 | 167.9 | 5.50153177 | 167.9 | 4.40318675 | 167.9 | 11.0422902 | | | 167.90 | 4.37376679 | 167.9 | 8.14932781 | 167.9 | 4.37376679 |
| 168 | 1.2258315 | 168 | 5.51133842 | 168 | 5.51133842 | 168 | 4.38357344 | 168 | 11.0324835 | | | 168.00 | 4.42280005 | 168 | 8.17874777 | 168 | 4.42280005 |

| | | | | | | | | | | | | | | | | | |
|---|---|---|---|---|---|---|---|---|---|---|---|---|---|---|---|---|---|
| 168.1 | 1.21602485 | 168.1 | 5.48191847 | 168.1 | 5.48191847 | 168.1 | 4.36396014 | 168.1 | 11.0324835 | | | 168.10 | 4.42280005 | 168.1 | 8.18855442 | 168.1 | 4.42280005 |
| 168.2 | 1.2062182 | 168.2 | 5.50153177 | 168.2 | 5.50153177 | 168.2 | 4.35415349 | 168.2 | 11.0619035 | | | 168.20 | 4.42280005 | 168.2 | 8.17874777 | 168.2 | 4.42280005 |
| 168.3 | 1.19641154 | 168.3 | 5.53095173 | 168.3 | 5.53095173 | 168.3 | 4.38357344 | 168.3 | 11.0815168 | | | 168.30 | 4.4129934 | 168.3 | 8.19836107 | 168.3 | 4.4129934 |
| 168.4 | 1.2062182 | 168.4 | 5.54075838 | 168.4 | 5.54075838 | 168.4 | 4.36396014 | 168.4 | 11.0815168 | | | 168.40 | 4.40318675 | 168.4 | 8.18855442 | 168.4 | 4.40318675 |
| 168.5 | 1.2258315 | 168.5 | 5.54075838 | 168.5 | 5.54075838 | 168.5 | 4.38357344 | 168.5 | 11.1011301 | | | 168.50 | 4.42280005 | 168.5 | 8.17874777 | 168.5 | 4.42280005 |
| 168.6 | 1.21602485 | 168.6 | 5.56037168 | 168.6 | 5.56037168 | 168.6 | 4.36396014 | 168.6 | 11.1109367 | | | 168.60 | 4.3933801 | 168.6 | 8.20816772 | 168.6 | 4.3933801 |
| 168.7 | 1.18660489 | 168.7 | 5.58979164 | 168.7 | 5.58979164 | 168.7 | 4.37376679 | 168.7 | 11.1011301 | | | 168.70 | 4.40318675 | 168.7 | 8.21797438 | 168.7 | 4.40318675 |
| 168.8 | 1.2258315 | 168.8 | 5.58979164 | 168.8 | 5.58979164 | 168.8 | 4.40318675 | 168.8 | 11.1501633 | | | 168.80 | 4.44241336 | 168.8 | 8.20816772 | 168.8 | 4.44241336 |
| 168.9 | 1.21602485 | 168.9 | 5.57998499 | 168.9 | 5.57998499 | 168.9 | 4.40318675 | 168.9 | 11.1697766 | | | 168.90 | 4.45222001 | 168.9 | 8.23758768 | 168.9 | 4.45222001 |
| 169 | 1.19641154 | 169 | 5.56037168 | 169 | 5.56037168 | 169 | 4.3933801 | 169 | 11.1893899 | | | 169.00 | 4.44241336 | 169 | 8.25720098 | 169 | 4.44241336 |
| 169.1 | 1.21602485 | 169.1 | 5.57017834 | 169.1 | 5.57017834 | 169.1 | 4.3933801 | 169.1 | 11.1991966 | | | 169.10 | 4.45222001 | 169.1 | 8.26700764 | 169.1 | 4.45222001 |
| 169.2 | 1.2062182 | 169.2 | 5.59959829 | 169.2 | 5.59959829 | 169.2 | 4.3933801 | 169.2 | 11.1893899 | | | 169.20 | 4.47183331 | 169.2 | 8.25720098 | 169.2 | 4.47183331 |
| 169.3 | 1.2062182 | 169.3 | 5.59959829 | 169.3 | 5.59959829 | 169.3 | 4.40318675 | 169.3 | 11.1991966 | | | 169.30 | 4.48163996 | 169.3 | 8.25720098 | 169.3 | 4.48163996 |
| 169.4 | 1.21602485 | 169.4 | 5.60940494 | 169.4 | 5.60940494 | 169.4 | 4.4326067 | 169.4 | 11.1795833 | | | 169.40 | 4.48163996 | 169.4 | 8.24739433 | 169.4 | 4.48163996 |
| 169.5 | 1.17679824 | 169.5 | 5.60940494 | 169.5 | 5.60940494 | 169.5 | 4.4129934 | 169.5 | 11.1991966 | | | 169.50 | 4.4326067 | 169.5 | 8.24739433 | 169.5 | 4.4326067 |
| 169.6 | 1.2258315 | 169.6 | 5.6388249 | 169.6 | 5.6388249 | 169.6 | 4.38357344 | 169.6 | 11.2188099 | | | 169.60 | 4.4326067 | 169.6 | 8.26700764 | 169.6 | 4.4326067 |
| 169.7 | 1.2258315 | 169.7 | 5.64863155 | 169.7 | 5.64863155 | 169.7 | 4.37376679 | 169.7 | 11.2482298 | | | 169.70 | 4.47183331 | 169.7 | 8.24739433 | 169.7 | 4.47183331 |
| 169.8 | 1.2258315 | 169.8 | 5.64863155 | 169.8 | 5.64863155 | 169.8 | 4.37376679 | 169.8 | 11.2580365 | | | 169.80 | 4.47183331 | 169.8 | 8.26700764 | 169.8 | 4.47183331 |
| 169.9 | 1.23563815 | 169.9 | 5.62901825 | 169.9 | 5.62901825 | 169.9 | 4.34434684 | 169.9 | 11.2580365 | | | 169.90 | 4.49144662 | 169.9 | 8.24739433 | 169.9 | 4.49144662 |
| 170 | 1.21602485 | 170 | 5.64863155 | 170 | 5.64863155 | 170 | 4.28550692 | 170 | 11.2776498 | | | 170.00 | 4.47183331 | 170 | 8.27681429 | 170 | 4.47183331 |
| 170.1 | 1.2258315 | 170.1 | 5.64863155 | 170.1 | 5.64863155 | 170.1 | 4.31492688 | 170.1 | 11.2580365 | | | 170.10 | 4.47183331 | 170.1 | 8.3160409 | 170.1 | 4.47183331 |
| 170.2 | 1.2258315 | 170.2 | 5.6388249 | 170.2 | 5.6388249 | 170.2 | 4.36396014 | 170.2 | 11.2874565 | | | 170.20 | 4.48163996 | 170.2 | 8.3160409 | 170.2 | 4.48163996 |
| 170.3 | 1.2062182 | 170.3 | 5.67805151 | 170.3 | 5.67805151 | 170.3 | 4.35415349 | 170.3 | 11.3266831 | | | 170.30 | 4.49144662 | 170.3 | 8.34546085 | 170.3 | 4.49144662 |
| 170.4 | 1.2062182 | 170.4 | 5.66824486 | 170.4 | 5.66824486 | 170.4 | 4.33454018 | 170.4 | 11.3462964 | | | 170.40 | 4.49144662 | 170.4 | 8.32584755 | 170.4 | 4.49144662 |
| 170.5 | 1.18660489 | 170.5 | 5.67805151 | 170.5 | 5.67805151 | 170.5 | 4.34434684 | 170.5 | 11.3364897 | | | 170.50 | 4.48163996 | 170.5 | 8.30623424 | 170.5 | 4.48163996 |
| 170.6 | 1.19641154 | 170.6 | 5.66824486 | 170.6 | 5.66824486 | 170.6 | 4.35415349 | 170.6 | 11.3364897 | | | 170.60 | 4.51105992 | 170.6 | 8.3160409 | 170.6 | 4.51105992 |
| 170.7 | 1.23563815 | 170.7 | 5.67805151 | 170.7 | 5.67805151 | 170.7 | 4.37376679 | 170.7 | 11.3364897 | | | 170.70 | 4.51105992 | 170.7 | 8.3356542 | 170.7 | 4.51105992 |
| 170.8 | 1.21602485 | 170.8 | 5.69766481 | 170.8 | 5.69766481 | 170.8 | 4.34434684 | 170.8 | 11.356103 | | | 170.80 | 4.48163996 | 170.8 | 8.34546085 | 170.8 | 4.48163996 |
| 170.9 | 1.17679824 | 170.9 | 5.71727812 | 170.9 | 5.71727812 | 170.9 | 4.35415349 | 170.9 | 11.3757163 | | | 170.90 | 4.50125327 | 170.9 | 8.3552675 | 170.9 | 4.50125327 |
| 171 | 1.18660489 | 171 | 5.69766481 | 171 | 5.69766481 | 171 | 4.38357344 | 171 | 11.3757163 | | | 171.00 | 4.53067322 | 171 | 8.3552675 | 171 | 4.53067322 |
| 171.1 | 1.2258315 | 171.1 | 5.69766481 | 171.1 | 5.69766481 | 171.1 | 4.40318675 | 171.1 | 11.3757163 | | | 171.10 | 4.49144662 | 171.1 | 8.34546085 | 171.1 | 4.49144662 |
| 171.2 | 1.23563815 | 171.2 | 5.69766481 | 171.2 | 5.69766481 | 171.2 | 4.40318675 | 171.2 | 11.385523 | | | 171.20 | 4.51105992 | 171.2 | 8.3552675 | 171.2 | 4.51105992 |
| 171.3 | 1.25525146 | 171.3 | 5.70747146 | 171.3 | 5.70747146 | 171.3 | 4.3933801 | 171.3 | 11.4443629 | | | 171.30 | 4.52086657 | 171.3 | 8.38468746 | 171.3 | 4.52086657 |
| 171.4 | 1.25525146 | 171.4 | 5.70747146 | 171.4 | 5.70747146 | 171.4 | 4.40318675 | 171.4 | 11.4541695 | | | 171.40 | 4.55028653 | 171.4 | 8.37488081 | 171.4 | 4.55028653 |
| 171.5 | 1.2454448 | 171.5 | 5.72708477 | 171.5 | 5.72708477 | 171.5 | 4.4129934 | 171.5 | 11.4541695 | | | 171.50 | 4.51105992 | 171.5 | 8.40430076 | 171.5 | 4.51105992 |
| 171.6 | 1.30428472 | 171.6 | 5.73689142 | 171.6 | 5.73689142 | 171.6 | 4.42280005 | 171.6 | 11.4639762 | | | 171.60 | 4.55028653 | 171.6 | 8.43372072 | 171.6 | 4.55028653 |
| 171.7 | 1.29447806 | 171.7 | 5.72708477 | 171.7 | 5.72708477 | 171.7 | 4.3933801 | 171.7 | 11.4835895 | | | 171.70 | 4.54047988 | 171.7 | 8.44352737 | 171.7 | 4.54047988 |
| 171.8 | 1.2258315 | 171.8 | 5.70747146 | 171.8 | 5.70747146 | 171.8 | 4.34434684 | 171.8 | 11.4835895 | | | 171.80 | 4.53067322 | 171.8 | 8.41410742 | 171.8 | 4.53067322 |
| 171.9 | 1.23563815 | 171.9 | 5.74669807 | 171.9 | 5.74669807 | 171.9 | 4.42280005 | 171.9 | 11.5032028 | | | 171.90 | 4.53067322 | 171.9 | 8.42391407 | 171.9 | 4.53067322 |
| 172 | 1.23563815 | 172 | 5.75650472 | 172 | 5.75650472 | 172 | 4.37376679 | 172 | 11.5130094 | | | 172.00 | 4.56009318 | 172 | 8.43372072 | 172 | 4.56009318 |
| 172.1 | 1.25525146 | 172.1 | 5.75650472 | 172.1 | 5.75650472 | 172.1 | 4.42280005 | 172.1 | 11.5424294 | | | 172.10 | 4.56989983 | 172.1 | 8.44352737 | 172.1 | 4.56989983 |
| 172.2 | 1.2258315 | 172.2 | 5.74669807 | 172.2 | 5.74669807 | 172.2 | 4.44241336 | 172.2 | 11.5522361 | | | 172.20 | 4.58951314 | 172.2 | 8.45333402 | 172.2 | 4.58951314 |
| 172.3 | 1.21602485 | 172.3 | 5.76631138 | 172.3 | 5.76631138 | 172.3 | 4.4129934 | 172.3 | 11.5424294 | | | 172.30 | 4.57970648 | 172.3 | 8.45333402 | 172.3 | 4.57970648 |
| 172.4 | 1.2258315 | 172.4 | 5.78592468 | 172.4 | 5.78592468 | 172.4 | 4.42280005 | 172.4 | 11.5620427 | | | 172.40 | 4.56009318 | 172.4 | 8.45333402 | 172.4 | 4.56009318 |
| 172.5 | 1.19641154 | 172.5 | 5.80553798 | 172.5 | 5.80553798 | 172.5 | 4.47183331 | 172.5 | 11.581656 | | | 172.50 | 4.57970648 | 172.5 | 8.46314068 | 172.5 | 4.57970648 |
| 172.6 | 1.2062182 | 172.6 | 5.77611803 | 172.6 | 5.77611803 | 172.6 | 4.48163996 | 172.6 | 11.6012693 | | | 172.60 | 4.58951314 | 172.6 | 8.46314068 | 172.6 | 4.58951314 |
| 172.7 | 1.25525146 | 172.7 | 5.75650472 | 172.7 | 5.75650472 | 172.7 | 4.49144662 | 172.7 | 11.6404959 | | | 172.70 | 4.59931979 | 172.7 | 8.46314068 | 172.7 | 4.59931979 |
| 172.8 | 1.2454448 | 172.8 | 5.76631138 | 172.8 | 5.76631138 | 172.8 | 4.51105992 | 172.8 | 11.6503026 | | | 172.80 | 4.60912644 | 172.8 | 8.51217394 | 172.8 | 4.60912644 |
| 172.9 | 1.21602485 | 172.9 | 5.78592468 | 172.9 | 5.78592468 | 172.9 | 4.48163996 | 172.9 | 11.6306893 | | | 172.90 | 4.58951314 | 172.9 | 8.50236728 | 172.9 | 4.58951314 |
| 173 | 1.17679824 | 173 | 5.79573133 | 173 | 5.79573133 | 173 | 4.52086657 | 173 | 11.6208826 | | | 173.00 | 4.58951314 | 173 | 8.51217394 | 173 | 4.58951314 |
| 173.1 | 1.2258315 | 173.1 | 5.83495794 | 173.1 | 5.83495794 | 173.1 | 4.53067322 | 173.1 | 11.6404959 | | | 173.10 | 4.59931979 | 173.1 | 8.52198059 | 173.1 | 4.59931979 |
| 173.2 | 1.21602485 | 173.2 | 5.83495794 | 173.2 | 5.83495794 | 173.2 | 4.52086657 | 173.2 | 11.6503026 | | | 173.20 | 4.56009318 | 173.2 | 8.50236728 | 173.2 | 4.56009318 |
| 173.3 | 1.2258315 | 173.3 | 5.85457124 | 173.3 | 5.85457124 | 173.3 | 4.45222001 | 173.3 | 11.6404959 | | | 173.30 | 4.57970648 | 173.3 | 8.50236728 | 173.3 | 4.57970648 |
| 173.4 | 1.26505811 | 173.4 | 5.8839912 | 173.4 | 5.8839912 | 173.4 | 4.45222001 | 173.4 | 11.6503026 | | | 173.40 | 4.58951314 | 173.4 | 8.50236728 | 173.4 | 4.58951314 |
| 173.5 | 1.28467141 | 173.5 | 5.8643779 | 173.5 | 5.8643779 | 173.5 | 4.49144662 | 173.5 | 11.6601092 | | | 173.50 | 4.58951314 | 173.5 | 8.51217394 | 173.5 | 4.58951314 |
| 173.6 | 1.25525146 | 173.6 | 5.8839912 | 173.6 | 5.8839912 | 173.6 | 4.49144662 | 173.6 | 11.6993358 | | | 173.60 | 4.61893309 | 173.6 | 8.53178724 | 173.6 | 4.61893309 |
| 173.7 | 1.28467141 | 173.7 | 5.8839912 | 173.7 | 5.8839912 | 173.7 | 4.54047988 | 173.7 | 11.7287558 | | | 173.70 | 4.59931979 | 173.7 | 8.54159389 | 173.7 | 4.59931979 |
| 173.8 | 1.26505811 | 173.8 | 5.87418455 | 173.8 | 5.87418455 | 173.8 | 4.50125327 | 173.8 | 11.7483691 | | | 173.80 | 4.62873974 | 173.8 | 8.5612072 | 173.8 | 4.62873974 |
| 173.9 | 1.23563815 | 173.9 | 5.89379785 | 173.9 | 5.89379785 | 173.9 | 4.50125327 | 173.9 | 11.7581757 | | | 173.90 | 4.64835305 | 173.9 | 8.57101385 | 173.9 | 4.64835305 |
| 174 | 1.26505811 | 174 | 5.9036045 | 174 | 5.9036045 | 174 | 4.53067322 | 174 | 11.7875957 | | | 174.00 | 4.6581597 | 174 | 8.5808205 | 174 | 4.6581597 |
| 174.1 | 1.26505811 | 174.1 | 5.93302446 | 174.1 | 5.93302446 | 174.1 | 4.53067322 | 174.1 | 11.807209 | | | 174.10 | 4.64835305 | 174.1 | 8.59062715 | 174.1 | 4.64835305 |

| | | | | | | | | | | | | | | | | | |
|---|---|---|---|---|---|---|---|---|---|---|---|---|---|---|---|---|---|
| 174.2 | 1.2258315 | 174.2 | 5.94283111 | 174.2 | 5.94283111 | 174.2 | 4.55028653 | 174.2 | 11.7974024 | | | 174.20 | 4.61893309 | 174.2 | 8.59062715 | 174.2 | 4.61893309 |
| 174.3 | 1.23563815 | 174.3 | 5.91341116 | 174.3 | 5.91341116 | 174.3 | 4.53067322 | 174.3 | 11.7875957 | | | 174.30 | 4.64835305 | 174.3 | 8.57101385 | 174.3 | 4.64835305 |
| 174.4 | 1.19641154 | 174.4 | 5.9036045 | 174.4 | 5.9036045 | 174.4 | 4.54047988 | 174.4 | 11.7777891 | | | 174.40 | 4.6385464 | 174.4 | 8.59062715 | 174.4 | 4.6385464 |
| 174.5 | 1.2062182 | 174.5 | 5.89379785 | 174.5 | 5.89379785 | 174.5 | 4.55028653 | 174.5 | 11.7974024 | | | 174.50 | 4.60912644 | 174.5 | 8.61024046 | 174.5 | 4.60912644 |
| 174.6 | 1.25525146 | 174.6 | 5.94283111 | 174.6 | 5.94283111 | 174.6 | 4.55028653 | 174.6 | 11.807209 | | | 174.60 | 4.6385464 | 174.6 | 8.61024046 | 174.6 | 4.6385464 |
| 174.7 | 1.2454448 | 174.7 | 5.95263776 | 174.7 | 5.95263776 | 174.7 | 4.56009318 | 174.7 | 11.8268223 | | | 174.70 | 4.6581597 | 174.7 | 8.61024046 | 174.7 | 4.6581597 |
| 174.8 | 1.2258315 | 174.8 | 5.93302446 | 174.8 | 5.93302446 | 174.8 | 4.56009318 | 174.8 | 11.8464356 | | | 174.80 | 4.60912644 | 174.8 | 8.65927372 | 174.8 | 4.60912644 |
| 174.9 | 1.23563815 | 174.9 | 5.94283111 | 174.9 | 5.94283111 | 174.9 | 4.57970648 | 174.9 | 11.8660489 | | | 174.90 | 4.61893309 | 174.9 | 8.66908037 | 174.9 | 4.61893309 |
| 175 | 1.2258315 | 175 | 5.98205772 | 175 | 5.98205772 | 175 | 4.58951314 | 175 | 11.8660489 | | | 175.00 | 4.6581597 | 175 | 8.65927372 | 175 | 4.6581597 |
| 175.1 | 1.2454448 | 175.1 | 5.99186437 | 175.1 | 5.99186437 | 175.1 | 4.56989983 | 175.1 | 11.8856622 | | | 175.10 | 4.64835305 | 175.1 | 8.62985376 | 175.1 | 4.64835305 |
| 175.2 | 1.25525146 | 175.2 | 5.97225107 | 175.2 | 5.97225107 | 175.2 | 4.58951314 | 175.2 | 11.9150822 | | | 175.20 | 4.677773 | 175.2 | 8.64946706 | 175.2 | 4.677773 |
| 175.3 | 1.2454448 | 175.3 | 5.97225107 | 175.3 | 5.97225107 | 175.3 | 4.58951314 | 175.3 | 11.9346955 | | | 175.30 | 4.69738631 | 175.3 | 8.67888702 | 175.3 | 4.69738631 |
| 175.4 | 1.2258315 | 175.4 | 5.99186437 | 175.4 | 5.99186437 | 175.4 | 4.56989983 | 175.4 | 11.9543088 | | | 175.40 | 4.69738631 | 175.4 | 8.68869367 | 175.4 | 4.69738631 |
| 175.5 | 1.2062182 | 175.5 | 6.00167102 | 175.5 | 6.00167102 | 175.5 | 4.56009318 | 175.5 | 11.9543088 | | | 175.50 | 4.66796635 | 175.5 | 8.68869367 | 175.5 | 4.66796635 |
| 175.6 | 1.2258315 | 175.6 | 6.03109098 | 175.6 | 6.03109098 | 175.6 | 4.57970648 | 175.6 | 11.9543088 | | | 175.60 | 4.6581597 | 175.6 | 8.71811363 | 175.6 | 4.6581597 |
| 175.7 | 1.21602485 | 175.7 | 6.03109098 | 175.7 | 6.03109098 | 175.7 | 4.57970648 | 175.7 | 11.9739221 | | | 175.70 | 4.68757966 | 175.7 | 8.73772693 | 175.7 | 4.68757966 |
| 175.8 | 1.2454448 | 175.8 | 6.06051094 | 175.8 | 6.06051094 | 175.8 | 4.57970648 | 175.8 | 11.9641154 | | | 175.80 | 4.68757966 | 175.8 | 8.71811363 | 175.8 | 4.68757966 |
| 175.9 | 1.27486476 | 175.9 | 6.05070428 | 175.9 | 6.05070428 | 175.9 | 4.58951314 | 175.9 | 11.9641154 | | | 175.90 | 4.68757966 | 175.9 | 8.72792028 | 175.9 | 4.68757966 |
| 176 | 1.25525146 | 176 | 6.03109098 | 176 | 6.03109098 | 176 | 4.59931979 | 176 | 12.0131487 | | | 176.00 | 4.66796635 | 176 | 8.72792028 | 176 | 4.66796635 |
| 176.1 | 1.23563815 | 176.1 | 6.02128433 | 176.1 | 6.02128433 | 176.1 | 4.62873974 | 176.1 | 12.0131487 | | | 176.10 | 4.677773 | 176.1 | 8.74753358 | 176.1 | 4.677773 |
| 176.2 | 1.2258315 | 176.2 | 6.03109098 | 176.2 | 6.03109098 | 176.2 | 4.60912644 | 176.2 | 12.0131487 | | | 176.20 | 4.6581597 | 176.2 | 8.76714689 | 176.2 | 4.6581597 |
| 176.3 | 1.2454448 | 176.3 | 6.06051094 | 176.3 | 6.06051094 | 176.3 | 4.60912644 | 176.3 | 12.0523753 | | | 176.30 | 4.677773 | 176.3 | 8.74753358 | 176.3 | 4.677773 |
| 176.4 | 1.2258315 | 176.4 | 6.05070428 | 176.4 | 6.05070428 | 176.4 | 4.62873974 | 176.4 | 12.0523753 | | | 176.40 | 4.66796635 | 176.4 | 8.75734024 | 176.4 | 4.66796635 |
| 176.5 | 1.21602485 | 176.5 | 6.05070428 | 176.5 | 6.05070428 | 176.5 | 4.62873974 | 176.5 | 12.0425687 | | | 176.50 | 4.677773 | 176.5 | 8.77695354 | 176.5 | 4.677773 |
| 176.6 | 1.26505811 | 176.6 | 6.07031759 | 176.6 | 6.07031759 | 176.6 | 4.62873974 | 176.6 | 12.0425687 | | | 176.60 | 4.70719296 | 176.6 | 8.79656684 | 176.6 | 4.70719296 |
| 176.7 | 1.27486476 | 176.7 | 6.07031759 | 176.7 | 6.07031759 | 176.7 | 4.6581597 | 176.7 | 12.062182 | | | 176.70 | 4.69738631 | 176.7 | 8.79656684 | 176.7 | 4.69738631 |
| 176.8 | 1.27486476 | 176.8 | 6.1095442 | 176.8 | 6.1095442 | 176.8 | 4.6581597 | 176.8 | 12.0817953 | | | 176.80 | 4.69738631 | 176.8 | 8.8063735 | 176.8 | 4.69738631 |
| 176.9 | 1.28467141 | 176.9 | 6.1095442 | 176.9 | 6.1095442 | 176.9 | 4.68757966 | 176.9 | 12.0916019 | | | 176.90 | 4.69738631 | 176.9 | 8.81618015 | 176.9 | 4.69738631 |
| 177 | 1.29447806 | 177 | 6.11935085 | 177 | 6.11935085 | 177 | 4.677773 | 177 | 12.0817953 | | | 177.00 | 4.68757966 | 177 | 8.81618015 | 177 | 4.68757966 |
| 177.1 | 1.27486476 | 177.1 | 6.08993089 | 177.1 | 6.08993089 | 177.1 | 4.68757966 | 177.1 | 12.062182 | | | 177.10 | 4.71699961 | 177.1 | 8.83579345 | 177.1 | 4.71699961 |
| 177.2 | 1.2454448 | 177.2 | 6.08993089 | 177.2 | 6.08993089 | 177.2 | 4.68757966 | 177.2 | 12.0817953 | | | 177.20 | 4.74641957 | 177.2 | 8.8259868 | 177.2 | 4.74641957 |
| 177.3 | 1.23563815 | 177.3 | 6.13896415 | 177.3 | 6.13896415 | 177.3 | 4.68757966 | 177.3 | 12.1112152 | | | 177.30 | 4.75622622 | 177.3 | 8.8456001 | 177.3 | 4.75622622 |
| 177.4 | 1.25525146 | 177.4 | 6.1291575 | 177.4 | 6.1291575 | 177.4 | 4.72680626 | 177.4 | 12.1406352 | | | 177.40 | 4.69738631 | 177.4 | 8.83579345 | 177.4 | 4.69738631 |
| 177.5 | 1.2454448 | 177.5 | 6.08993089 | 177.5 | 6.08993089 | 177.5 | 4.73661292 | 177.5 | 12.1406352 | | | 177.50 | 4.71699961 | 177.5 | 8.8456001 | 177.5 | 4.71699961 |
| 177.6 | 1.2454448 | 177.6 | 6.09973754 | 177.6 | 6.09973754 | 177.6 | 4.69738631 | 177.6 | 12.1602485 | | | 177.60 | 4.70719296 | 177.6 | 8.8259868 | 177.6 | 4.70719296 |
| 177.7 | 1.27486476 | 177.7 | 6.13896415 | 177.7 | 6.13896415 | 177.7 | 4.71699961 | 177.7 | 12.1798618 | | | 177.70 | 4.71699961 | 177.7 | 8.81618015 | 177.7 | 4.71699961 |
| 177.8 | 1.29447806 | 177.8 | 6.16838411 | 177.8 | 6.16838411 | 177.8 | 4.72680626 | 177.8 | 12.2092817 | | | 177.80 | 4.77583952 | 177.8 | 8.8456001 | 177.8 | 4.77583952 |
| 177.9 | 1.2454448 | 177.9 | 6.15857746 | 177.9 | 6.15857746 | 177.9 | 4.70719296 | 177.9 | 12.1994751 | | | 177.90 | 4.77583952 | 177.9 | 8.85540676 | 177.9 | 4.77583952 |
| 178 | 1.26505811 | 178 | 6.1487708 | 178 | 6.1487708 | 178 | 4.72680626 | 178 | 12.1896684 | | | 178.00 | 4.78564618 | 178 | 8.87502006 | 178 | 4.78564618 |
| 178.1 | 1.29447806 | 178.1 | 6.1487708 | 178.1 | 6.1487708 | 178.1 | 4.75622622 | 178.1 | 12.1896684 | | | 178.10 | 4.78564618 | 178.1 | 8.90444002 | 178.1 | 4.78564618 |
| 178.2 | 1.30428472 | 178.2 | 6.15857746 | 178.2 | 6.15857746 | 178.2 | 4.73661292 | 178.2 | 12.1994751 | | | 178.20 | 4.79545283 | 178.2 | 8.89463336 | 178.2 | 4.79545283 |
| 178.3 | 1.27486476 | 178.3 | 6.15857746 | 178.3 | 6.15857746 | 178.3 | 4.73661292 | 178.3 | 12.2681217 | | | 178.30 | 4.78564618 | 178.3 | 8.89463336 | 178.3 | 4.78564618 |
| 178.4 | 1.23563815 | 178.4 | 6.18799741 | 178.4 | 6.18799741 | 178.4 | 4.72680626 | 178.4 | 12.2387017 | | | 178.40 | 4.76603287 | 178.4 | 8.92405332 | 178.4 | 4.76603287 |
| 178.5 | 1.25525146 | 178.5 | 6.22722402 | 178.5 | 6.22722402 | 178.5 | 4.72680626 | 178.5 | 12.258315 | | | 178.50 | 4.78564618 | 178.5 | 8.96327993 | 178.5 | 4.78564618 |
| 178.6 | 1.26505811 | 178.6 | 6.22722402 | 178.6 | 6.22722402 | 178.6 | 4.73661292 | 178.6 | 12.2975416 | | | 178.60 | 4.76603287 | 178.6 | 8.94366662 | 178.6 | 4.76603287 |
| 178.7 | 1.27486476 | 178.7 | 6.20761072 | 178.7 | 6.20761072 | 178.7 | 4.71699961 | 178.7 | 12.287735 | | | 178.70 | 4.77583952 | 178.7 | 8.94366662 | 178.7 | 4.77583952 |
| 178.8 | 1.28467141 | 178.8 | 6.22722402 | 178.8 | 6.22722402 | 178.8 | 4.69738631 | 178.8 | 12.287735 | | | 178.80 | 4.74641957 | 178.8 | 8.94366662 | 178.8 | 4.74641957 |
| 178.9 | 1.29447806 | 178.9 | 6.22722402 | 178.9 | 6.22722402 | 178.9 | 4.69738631 | 178.9 | 12.3269616 | | | 178.90 | 4.76603287 | 178.9 | 8.92405332 | 178.9 | 4.76603287 |
| 179 | 1.30428472 | 179 | 6.22722402 | 179 | 6.22722402 | 179 | 4.68757966 | 179 | 12.3269616 | | | 179.00 | 4.79545283 | 179 | 8.94366662 | 179 | 4.79545283 |
| 179.1 | 1.29447806 | 179.1 | 6.24683732 | 179.1 | 6.24683732 | 179.1 | 4.72680626 | 179.1 | 12.3269616 | | | 179.10 | 4.78564618 | 179.1 | 8.98289323 | 179.1 | 4.78564618 |
| 179.2 | 1.28467141 | 179.2 | 6.25664398 | 179.2 | 6.25664398 | 179.2 | 4.71699961 | 179.2 | 12.3367682 | | | 179.20 | 4.77583952 | 179.2 | 8.98289323 | 179.2 | 4.77583952 |
| 179.3 | 1.28467141 | 179.3 | 6.21741737 | 179.3 | 6.21741737 | 179.3 | 4.72680626 | 179.3 | 12.3367682 | | | 179.30 | 4.76603287 | 179.3 | 8.99269988 | 179.3 | 4.76603287 |
| 179.4 | 1.28467141 | 179.4 | 6.24683732 | 179.4 | 6.24683732 | 179.4 | 4.70719296 | 179.4 | 12.3563815 | | | 179.40 | 4.80525948 | 179.4 | 8.99269988 | 179.4 | 4.80525948 |
| 179.5 | 1.25525146 | 179.5 | 6.29587058 | 179.5 | 6.29587058 | 179.5 | 4.75622622 | 179.5 | 12.3858015 | | | 179.50 | 4.83467944 | 179.5 | 9.00250654 | 179.5 | 4.83467944 |
| 179.6 | 1.23563815 | 179.6 | 6.29587058 | 179.6 | 6.29587058 | 179.6 | 4.75622622 | 179.6 | 12.4054148 | | | 179.60 | 4.84448609 | 179.6 | 9.03192649 | 179.6 | 4.84448609 |
| 179.7 | 1.25525146 | 179.7 | 6.27625728 | 179.7 | 6.27625728 | 179.7 | 4.74641957 | 179.7 | 12.4054148 | | | 179.70 | 4.84448609 | 179.7 | 9.01231319 | 179.7 | 4.84448609 |
| 179.8 | 1.26505811 | 179.8 | 6.28606393 | 179.8 | 6.28606393 | 179.8 | 4.73661292 | 179.8 | 12.3858015 | | | 179.80 | 4.79545283 | 179.8 | 9.01231319 | 179.8 | 4.79545283 |
| 179.9 | 1.2454448 | 179.9 | 6.30567724 | 179.9 | 6.30567724 | 179.9 | 4.72680626 | 179.9 | 12.3858015 | | | 179.90 | 4.80525948 | 179.9 | 9.02211984 | 179.9 | 4.80525948 |
| 180 | 1.23563815 | 180 | 6.31548389 | 180 | 6.31548389 | 180 | 4.75622622 | 180 | 12.4446414 | | | 180.00 | 4.79545283 | 180 | 9.02211984 | 180 | 4.79545283 |
| 180.1 | 1.25525146 | 180.1 | 6.31548389 | 180.1 | 6.31548389 | 180.1 | 4.79545283 | 180.1 | 12.4740613 | | | 180.10 | 4.80525948 | 180.1 | 9.03192649 | 180.1 | 4.80525948 |
| 180.2 | 1.27486476 | 180.2 | 6.31548389 | 180.2 | 6.31548389 | 180.2 | 4.76603287 | 180.2 | 12.4642547 | | | 180.20 | 4.84448609 | 180.2 | 9.0515398 | 180.2 | 4.84448609 |

| | | | | | | | | | | | | | | | | | |
|---|---|---|---|---|---|---|---|---|---|---|---|---|---|---|---|---|---|
| 180.3 | 1.26505811 | 180.3 | 6.30567724 | 180.3 | 6.30567724 | 180.3 | 4.76603287 | 180.3 | 12.454448 | | | 180.30 | 4.85429274 | 180.3 | 9.06134645 | 180.3 | 4.85429274 |
| 180.4 | 1.27486476 | 180.4 | 6.30567724 | 180.4 | 6.30567724 | 180.4 | 4.80525948 | 180.4 | 12.4446414 | | | 180.40 | 4.82487278 | 180.4 | 9.06134645 | 180.4 | 4.82487278 |
| 180.5 | 1.25525146 | 180.5 | 6.31548389 | 180.5 | 6.31548389 | 180.5 | 4.78564618 | 180.5 | 12.4642547 | | | 180.50 | 4.81506613 | 180.5 | 9.06134645 | 180.5 | 4.81506613 |
| 180.6 | 1.25525146 | 180.6 | 6.30567724 | 180.6 | 6.30567724 | 180.6 | 4.77583952 | 180.6 | 12.513288 | | | 180.60 | 4.84448609 | 180.6 | 9.0711531 | 180.6 | 4.84448609 |
| 180.7 | 1.26505811 | 180.7 | 6.31548389 | 180.7 | 6.31548389 | 180.7 | 4.79545283 | 180.7 | 12.5230946 | | | 180.70 | 4.85429274 | 180.7 | 9.08095975 | 180.7 | 4.85429274 |
| 180.8 | 1.26505811 | 180.8 | 6.30567724 | 180.8 | 6.30567724 | 180.8 | 4.83467944 | 180.8 | 12.5329013 | | | 180.80 | 4.86409939 | 180.8 | 9.0907664 | 180.8 | 4.86409939 |
| 180.9 | 1.27486476 | 180.9 | 6.30567724 | 180.9 | 6.30567724 | 180.9 | 4.83467944 | 180.9 | 12.5427079 | | | 180.90 | 4.84448609 | 180.9 | 9.06134645 | 180.9 | 4.84448609 |
| 181 | 1.27486476 | 181 | 6.32529054 | 181 | 6.32529054 | 181 | 4.83467944 | 181 | 12.513288 | | | 181.00 | 4.8837127 | 181 | 9.08095975 | 181 | 4.8837127 |
| 181.1 | 1.25525146 | 181.1 | 6.31548389 | 181.1 | 6.31548389 | 181.1 | 4.83467944 | 181.1 | 12.5525146 | | | 181.10 | 4.903326 | 181.1 | 9.0711531 | 181.1 | 4.903326 |
| 181.2 | 1.30428472 | 181.2 | 6.33509719 | 181.2 | 6.33509719 | 181.2 | 4.82487278 | 181.2 | 12.5623212 | | | 181.20 | 4.86409939 | 181.2 | 9.08095975 | 181.2 | 4.86409939 |
| 181.3 | 1.30428472 | 181.3 | 6.3743238 | 181.3 | 6.3743238 | 181.3 | 4.82487278 | 181.3 | 12.5525146 | | | 181.30 | 4.85429274 | 181.3 | 9.10057306 | 181.3 | 4.85429274 |
| 181.4 | 1.27486476 | 181.4 | 6.3547105 | 181.4 | 6.3547105 | 181.4 | 4.85429274 | 181.4 | 12.5623212 | | | 181.40 | 4.8837127 | 181.4 | 9.13979966 | 181.4 | 4.8837127 |
| 181.5 | 1.28467141 | 181.5 | 6.36451715 | 181.5 | 6.36451715 | 181.5 | 4.83467944 | 181.5 | 12.5623212 | | | 181.50 | 4.8837127 | 181.5 | 9.15941297 | 181.5 | 4.8837127 |
| 181.6 | 1.27486476 | 181.6 | 6.36451715 | 181.6 | 6.36451715 | 181.6 | 4.85429274 | 181.6 | 12.5721279 | | | 181.60 | 4.86409939 | 181.6 | 9.13979966 | 181.6 | 4.86409939 |
| 181.7 | 1.27486476 | 181.7 | 6.3547105 | 181.7 | 6.3547105 | 181.7 | 4.84448609 | 181.7 | 12.6015478 | | | 181.70 | 4.85429274 | 181.7 | 9.11037971 | 181.7 | 4.85429274 |
| 181.8 | 1.30428472 | 181.8 | 6.3743238 | 181.8 | 6.3743238 | 181.8 | 4.75622622 | 181.8 | 12.6309678 | | | 181.80 | 4.89351935 | 181.8 | 9.14960632 | 181.8 | 4.89351935 |
| 181.9 | 1.29447806 | 181.9 | 6.38413045 | 181.9 | 6.38413045 | 181.9 | 4.80525948 | 181.9 | 12.6015478 | | | 181.90 | 4.89351935 | 181.9 | 9.16921962 | 181.9 | 4.89351935 |
| 182 | 1.29447806 | 182 | 6.3743238 | 182 | 6.3743238 | 182 | 4.83467944 | 182 | 12.6015478 | | | 182.00 | 4.89351935 | 182 | 9.17902627 | 182 | 4.89351935 |
| 182.1 | 1.28467141 | 182.1 | 6.3939371 | 182.1 | 6.3939371 | 182.1 | 4.81506613 | 182.1 | 12.6505811 | | | 182.10 | 4.89351935 | 182.1 | 9.18883292 | 182.1 | 4.89351935 |
| 182.2 | 1.30428472 | 182.2 | 6.41355041 | 182.2 | 6.41355041 | 182.2 | 4.81506613 | 182.2 | 12.6996143 | | | 182.20 | 4.9229393 | 182.2 | 9.17902627 | 182.2 | 4.9229393 |
| 182.3 | 1.29447806 | 182.3 | 6.42335706 | 182.3 | 6.42335706 | 182.3 | 4.84448609 | 182.3 | 12.6898077 | | | 182.30 | 4.903326 | 182.3 | 9.19863958 | 182.3 | 4.903326 |
| 182.4 | 1.25525146 | 182.4 | 6.41355041 | 182.4 | 6.41355041 | 182.4 | 4.86409939 | 182.4 | 12.709421 | | | 182.40 | 4.8837127 | 182.4 | 9.22805953 | 182.4 | 4.8837127 |
| 182.5 | 1.25525146 | 182.5 | 6.45277702 | 182.5 | 6.45277702 | 182.5 | 4.86409939 | 182.5 | 12.6996143 | | | 182.50 | 4.91313265 | 182.5 | 9.25747949 | 182.5 | 4.91313265 |
| 182.6 | 1.27486476 | 182.6 | 6.46258367 | 182.6 | 6.46258367 | 182.6 | 4.84448609 | 182.6 | 12.6996143 | | | 182.60 | 4.903326 | 182.6 | 9.23786618 | 182.6 | 4.903326 |
| 182.7 | 1.26505811 | 182.7 | 6.45277702 | 182.7 | 6.45277702 | 182.7 | 4.89351935 | 182.7 | 12.7192276 | | | 182.70 | 4.89351935 | 182.7 | 9.24767284 | 182.7 | 4.89351935 |
| 182.8 | 1.23563815 | 182.8 | 6.47239032 | 182.8 | 6.47239032 | 182.8 | 4.87390604 | 182.8 | 12.7192276 | | | 182.80 | 4.87390604 | 182.8 | 9.27709279 | 182.8 | 4.87390604 |
| 182.9 | 1.27486476 | 182.9 | 6.49200362 | 182.9 | 6.49200362 | 182.9 | 4.84448609 | 182.9 | 12.7192276 | | | 182.90 | 4.8837127 | 182.9 | 9.26728614 | 182.9 | 4.8837127 |
| 183 | 1.26505811 | 183 | 6.46258367 | 183 | 6.46258367 | 183 | 4.85429274 | 183 | 12.7192276 | | | 183.00 | 4.91313265 | 183 | 9.25747949 | 183 | 4.91313265 |
| 183.1 | 1.29447806 | 183.1 | 6.48219697 | 183.1 | 6.48219697 | 183.1 | 4.86409939 | 183.1 | 12.7290343 | | | 183.10 | 4.91313265 | 183.1 | 9.24767284 | 183.1 | 4.91313265 |
| 183.2 | 1.30428472 | 183.2 | 6.52142358 | 183.2 | 6.52142358 | 183.2 | 4.903326 | 183.2 | 12.7584543 | | | 183.20 | 4.91313265 | 183.2 | 9.3163194 | 183.2 | 4.91313265 |
| 183.3 | 1.27486476 | 183.3 | 6.52142358 | 183.3 | 6.52142358 | 183.3 | 4.903326 | 183.3 | 12.7584543 | | | 183.30 | 4.97197256 | 183.3 | 9.2967061 | 183.3 | 4.97197256 |
| 183.4 | 1.28467141 | 183.4 | 6.50181028 | 183.4 | 6.50181028 | 183.4 | 4.903326 | 183.4 | 12.7584543 | | | 183.40 | 4.97197256 | 183.4 | 9.27709279 | 183.4 | 4.97197256 |
| 183.5 | 1.28467141 | 183.5 | 6.49200362 | 183.5 | 6.49200362 | 183.5 | 4.903326 | 183.5 | 12.7584543 | | | 183.50 | 4.97197256 | 183.5 | 9.2967061 | 183.5 | 4.97197256 |
| 183.6 | 1.28467141 | 183.6 | 6.51161693 | 183.6 | 6.51161693 | 183.6 | 4.93274596 | 183.6 | 12.7878742 | | | 183.60 | 4.95235926 | 183.6 | 9.3163194 | 183.6 | 4.95235926 |
| 183.7 | 1.26505811 | 183.7 | 6.51161693 | 183.7 | 6.51161693 | 183.7 | 4.94255261 | 183.7 | 12.8172942 | | | 183.70 | 4.95235926 | 183.7 | 9.32612605 | 183.7 | 4.95235926 |
| 183.8 | 1.32389802 | 183.8 | 6.51161693 | 183.8 | 6.51161693 | 183.8 | 4.8837127 | 183.8 | 12.8172942 | | | 183.80 | 4.93274596 | 183.8 | 9.32612605 | 183.8 | 4.93274596 |
| 183.9 | 1.31409137 | 183.9 | 6.51161693 | 183.9 | 6.51161693 | 183.9 | 4.91313265 | 183.9 | 12.8172942 | | | 183.90 | 4.94255261 | 183.9 | 9.3359327 | 183.9 | 4.94255261 |
| 184 | 1.28467141 | 184 | 6.54103688 | 184 | 6.54103688 | 184 | 4.96216591 | 184 | 12.8074875 | | | 184.00 | 4.97197256 | 184 | 9.32612605 | 184 | 4.97197256 |
| 184.1 | 1.29447806 | 184.1 | 6.56065019 | 184.1 | 6.56065019 | 184.1 | 4.97197256 | 184.1 | 12.8467141 | | | 184.10 | 4.97197256 | 184.1 | 9.32612605 | 184.1 | 4.97197256 |
| 184.2 | 1.28467141 | 184.2 | 6.53123023 | 184.2 | 6.53123023 | 184.2 | 4.97197256 | 184.2 | 12.8565208 | | | 184.20 | 4.95235926 | 184.2 | 9.3359327 | 184.2 | 4.95235926 |
| 184.3 | 1.26505811 | 184.3 | 6.52142358 | 184.3 | 6.52142358 | 184.3 | 4.96216591 | 184.3 | 12.8761341 | | | 184.30 | 4.97197256 | 184.3 | 9.34573936 | 184.3 | 4.97197256 |
| 184.4 | 1.28467141 | 184.4 | 6.54103688 | 184.4 | 6.54103688 | 184.4 | 4.98177922 | 184.4 | 12.8957474 | | | 184.40 | 4.94255261 | 184.4 | 9.37515931 | 184.4 | 4.94255261 |
| 184.5 | 1.26505811 | 184.5 | 6.55084354 | 184.5 | 6.55084354 | 184.5 | 4.96216591 | 184.5 | 12.8859407 | | | 184.50 | 4.93274596 | 184.5 | 9.37515931 | 184.5 | 4.93274596 |
| 184.6 | 1.26505811 | 184.6 | 6.56065019 | 184.6 | 6.56065019 | 184.6 | 4.93274596 | 184.6 | 12.905554 | | | 184.60 | 4.96216591 | 184.6 | 9.35554601 | 184.6 | 4.96216591 |
| 184.7 | 1.28467141 | 184.7 | 6.59007014 | 184.7 | 6.59007014 | 184.7 | 4.95235926 | 184.7 | 12.9153607 | | | 184.70 | 4.97197256 | 184.7 | 9.36535266 | 184.7 | 4.97197256 |
| 184.8 | 1.32389802 | 184.8 | 6.59007014 | 184.8 | 6.59007014 | 184.8 | 4.98177922 | 184.8 | 12.934974 | | | 184.80 | 4.98177922 | 184.8 | 9.35554601 | 184.8 | 4.98177922 |
| 184.9 | 1.31409137 | 184.9 | 6.57045684 | 184.9 | 6.57045684 | 184.9 | 4.96216591 | 184.9 | 12.9447806 | | | 184.90 | 4.98177922 | 184.9 | 9.35554601 | 184.9 | 4.98177922 |
| 185 | 1.26505811 | 185 | 6.57045684 | 185 | 6.57045684 | 185 | 4.94255261 | 185 | 12.9742006 | | | 185.00 | 5.01119917 | 185 | 9.37515931 | 185 | 5.01119917 |
| 185.1 | 1.26505811 | 185.1 | 6.58026349 | 185.1 | 6.58026349 | 185.1 | 5.00139252 | 185.1 | 13.0134272 | | | 185.10 | 5.02100582 | 185.1 | 9.38496596 | 185.1 | 5.02100582 |
| 185.2 | 1.28467141 | 185.2 | 6.58026349 | 185.2 | 6.58026349 | 185.2 | 5.01119917 | 185.2 | 13.0330405 | | | 185.20 | 4.98177922 | 185.2 | 9.36535266 | 185.2 | 4.98177922 |
| 185.3 | 1.30428472 | 185.3 | 6.59007014 | 185.3 | 6.59007014 | 185.3 | 5.01119917 | 185.3 | 13.0526538 | | | 185.30 | 4.99158587 | 185.3 | 9.37515931 | 185.3 | 4.99158587 |
| 185.4 | 1.30428472 | 185.4 | 6.5998768 | 185.4 | 6.5998768 | 185.4 | 5.02100582 | 185.4 | 13.0624605 | | | 185.40 | 4.99158587 | 185.4 | 9.36535266 | 185.4 | 4.99158587 |
| 185.5 | 1.26505811 | 185.5 | 6.5998768 | 185.5 | 6.5998768 | 185.5 | 4.97197256 | 185.5 | 13.0526538 | | | 185.50 | 4.98177922 | 185.5 | 9.36535266 | 185.5 | 4.98177922 |
| 185.6 | 1.27486476 | 185.6 | 6.59007014 | 185.6 | 6.59007014 | 185.6 | 4.97197256 | 185.6 | 13.0722671 | | | 185.60 | 5.02100582 | 185.6 | 9.38496596 | 185.6 | 5.02100582 |
| 185.7 | 1.27486476 | 185.7 | 6.58026349 | 185.7 | 6.58026349 | 185.7 | 4.98177922 | 185.7 | 13.1114937 | | | 185.70 | 5.04061913 | 185.7 | 9.38496596 | 185.7 | 5.04061913 |
| 185.8 | 1.29447806 | 185.8 | 6.58026349 | 185.8 | 6.58026349 | 185.8 | 5.00139252 | 185.8 | 13.1114937 | | | 185.80 | 5.07003908 | 185.8 | 9.39477262 | 185.8 | 5.07003908 |
| 185.9 | 1.29447806 | 185.9 | 6.62929675 | 185.9 | 6.62929675 | 185.9 | 5.00139252 | 185.9 | 13.0918804 | | | 185.90 | 5.07003908 | 185.9 | 9.42419257 | 185.9 | 5.07003908 |
| 186 | 1.29447806 | 186 | 6.65871671 | 186 | 6.65871671 | 186 | 4.98177922 | 186 | 13.1114937 | | | 186.00 | 5.03081248 | 186 | 9.43399922 | 186 | 5.03081248 |
| 186.1 | 1.29447806 | 186.1 | 6.62929675 | 186.1 | 6.62929675 | 186.1 | 5.01119917 | 186.1 | 13.1409137 | | | 186.10 | 5.04061913 | 186.1 | 9.41438592 | 186.1 | 5.04061913 |
| 186.2 | 1.26505811 | 186.2 | 6.65871671 | 186.2 | 6.65871671 | 186.2 | 5.01119917 | 186.2 | 13.160527 | | | 186.20 | 5.05042578 | 186.2 | 9.40457927 | 186.2 | 5.05042578 |
| 186.3 | 1.29447806 | 186.3 | 6.64891006 | 186.3 | 6.64891006 | 186.3 | 5.02100582 | 186.3 | 13.1703336 | | | 186.30 | 5.06023243 | 186.3 | 9.41438592 | 186.3 | 5.06023243 |

| | | | | | | | | | | | | | | | | | |
|---|---|---|---|---|---|---|---|---|---|---|---|---|---|---|---|---|---|
| 186.4 | 1.27486476 | 186.4 | 6.6391034 | 186.4 | 6.6391034 | 186.4 | 5.04061913 | 186.4 | 13.1997536 | | | 186.40 | 5.05042578 | 186.4 | 9.42419257 | 186.4 | 5.05042578 |
| 186.5 | 1.2454448 | 186.5 | 6.64891006 | 186.5 | 6.64891006 | 186.5 | 5.05042578 | 186.5 | 13.1899469 | | | 186.50 | 5.05042578 | 186.5 | 9.42419257 | 186.5 | 5.05042578 |
| 186.6 | 1.2454448 | 186.6 | 6.6391034 | 186.6 | 6.6391034 | 186.6 | 5.07003908 | 186.6 | 13.2487869 | | | 186.60 | 5.09945904 | 186.6 | 9.44380588 | 186.6 | 5.09945904 |
| 186.7 | 1.25525146 | 186.7 | 6.6391034 | 186.7 | 6.6391034 | 186.7 | 5.07984574 | 186.7 | 13.2585935 | | | 186.70 | 5.07984574 | 186.7 | 9.46341918 | 186.7 | 5.07984574 |
| 186.8 | 1.23563815 | 186.8 | 6.67833001 | 186.8 | 6.67833001 | 186.8 | 5.08965239 | 186.8 | 13.2487869 | | | 186.80 | 5.05042578 | 186.8 | 9.48303248 | 186.8 | 5.05042578 |
| 186.9 | 1.2454448 | 186.9 | 6.66852336 | 186.9 | 6.66852336 | 186.9 | 5.05042578 | 186.9 | 13.2389802 | | | 186.90 | 5.06023243 | 186.9 | 9.50264579 | 186.9 | 5.06023243 |
| 187 | 1.27486476 | 187 | 6.64891006 | 187 | 6.64891006 | 187 | 5.04061913 | 187 | 13.2095602 | | | 187.00 | 5.07984574 | 187 | 9.48303248 | 187 | 5.07984574 |
| 187.1 | 1.2454448 | 187.1 | 6.67833001 | 187.1 | 6.67833001 | 187.1 | 5.04061913 | 187.1 | 13.2487869 | | | 187.10 | 5.08965239 | 187.1 | 9.45361253 | 187.1 | 5.08965239 |
| 187.2 | 1.23563815 | 187.2 | 6.69794332 | 187.2 | 6.69794332 | 187.2 | 5.06023243 | 187.2 | 13.2684002 | | | 187.20 | 5.08965239 | 187.2 | 9.48303248 | 187.2 | 5.08965239 |
| 187.3 | 1.2454448 | 187.3 | 6.70774997 | 187.3 | 6.70774997 | 187.3 | 5.07003908 | 187.3 | 13.2880135 | | | 187.30 | 5.08965239 | 187.3 | 9.52225909 | 187.3 | 5.08965239 |
| 187.4 | 1.26505811 | 187.4 | 6.71755662 | 187.4 | 6.71755662 | 187.4 | 5.10926569 | 187.4 | 13.3076268 | | | 187.40 | 5.11907234 | 187.4 | 9.52225909 | 187.4 | 5.11907234 |
| 187.5 | 1.28467141 | 187.5 | 6.67833001 | 187.5 | 6.67833001 | 187.5 | 5.11907234 | 187.5 | 13.3076268 | | | 187.50 | 5.10926569 | 187.5 | 9.52225909 | 187.5 | 5.10926569 |
| 187.6 | 1.27486476 | 187.6 | 6.69794332 | 187.6 | 6.69794332 | 187.6 | 5.09945904 | 187.6 | 13.2880135 | | | 187.60 | 5.09945904 | 187.6 | 9.49283914 | 187.6 | 5.09945904 |
| 187.7 | 1.26505811 | 187.7 | 6.70774997 | 187.7 | 6.70774997 | 187.7 | 5.08965239 | 187.7 | 13.3076268 | | | 187.70 | 5.11907234 | 187.7 | 9.49283914 | 187.7 | 5.11907234 |
| 187.8 | 1.27486476 | 187.8 | 6.72736327 | 187.8 | 6.72736327 | 187.8 | 5.10926569 | 187.8 | 13.35666 | | | 187.80 | 5.13868565 | 187.8 | 9.51245244 | 187.8 | 5.13868565 |
| 187.9 | 1.28467141 | 187.9 | 6.72736327 | 187.9 | 6.72736327 | 187.9 | 5.10926569 | 187.9 | 13.38608 | | | 187.90 | 5.11907234 | 187.9 | 9.53206574 | 187.9 | 5.11907234 |
| 188 | 1.25525146 | 188 | 6.71755662 | 188 | 6.71755662 | 188 | 5.11907234 | 188 | 13.4056933 | | | 188.00 | 5.11907234 | 188 | 9.5614857 | 188 | 5.11907234 |
| 188.1 | 1.26505811 | 188.1 | 6.73716992 | 188.1 | 6.73716992 | 188.1 | 5.15829895 | 188.1 | 13.4056933 | | | 188.10 | 5.11907234 | 188.1 | 9.59090566 | 188.1 | 5.11907234 |
| 188.2 | 1.23563815 | 188.2 | 6.74697658 | 188.2 | 6.74697658 | 188.2 | 5.17791226 | 188.2 | 13.4056933 | | | 188.20 | 5.11907234 | 188.2 | 9.57129235 | 188.2 | 5.11907234 |
| 188.3 | 1.26505811 | 188.3 | 6.74697658 | 188.3 | 6.74697658 | 188.3 | 5.15829895 | 188.3 | 13.4154999 | | | 188.30 | 5.10926569 | 188.3 | 9.581099 | 188.3 | 5.10926569 |
| 188.4 | 1.28467141 | 188.4 | 6.78620318 | 188.4 | 6.78620318 | 188.4 | 5.15829895 | 188.4 | 13.4056933 | | | 188.40 | 5.10926569 | 188.4 | 9.57129235 | 188.4 | 5.10926569 |
| 188.5 | 1.28467141 | 188.5 | 6.74697658 | 188.5 | 6.74697658 | 188.5 | 5.15829895 | 188.5 | 13.4253066 | | | 188.50 | 5.1484923 | 188.5 | 9.60071231 | 188.5 | 5.1484923 |
| 188.6 | 1.25525146 | 188.6 | 6.76658988 | 188.6 | 6.76658988 | 188.6 | 5.15829895 | 188.6 | 13.4351132 | | | 188.60 | 5.15829895 | 188.6 | 9.60071231 | 188.6 | 5.15829895 |
| 188.7 | 1.27486476 | 188.7 | 6.78620318 | 188.7 | 6.78620318 | 188.7 | 5.15829895 | 188.7 | 13.4351132 | | | 188.70 | 5.15829895 | 188.7 | 9.61051896 | 188.7 | 5.15829895 |
| 188.8 | 1.26505811 | 188.8 | 6.77639653 | 188.8 | 6.77639653 | 188.8 | 5.18771891 | 188.8 | 13.4939532 | | | 188.80 | 5.1681056 | 188.8 | 9.60071231 | 188.8 | 5.1681056 |
| 188.9 | 1.28467141 | 188.9 | 6.77639653 | 188.9 | 6.77639653 | 188.9 | 5.15829895 | 188.9 | 13.5037598 | | | 188.90 | 5.19752556 | 188.9 | 9.62032561 | 188.9 | 5.19752556 |
| 189 | 1.28467141 | 189 | 6.78620318 | 189 | 6.78620318 | 189 | 5.1484923 | 189 | 13.5037598 | | | 189.00 | 5.1681056 | 189 | 9.63013226 | 189 | 5.1681056 |
| 189.1 | 1.26505811 | 189.1 | 6.79600984 | 189.1 | 6.79600984 | 189.1 | 5.20733221 | 189.1 | 13.5135665 | | | 189.10 | 5.1681056 | 189.1 | 9.61051896 | 189.1 | 5.1681056 |
| 189.2 | 1.26505811 | 189.2 | 6.80581649 | 189.2 | 6.80581649 | 189.2 | 5.22694552 | 189.2 | 13.5135665 | | | 189.20 | 5.15829895 | 189.2 | 9.64974557 | 189.2 | 5.15829895 |
| 189.3 | 1.26505811 | 189.3 | 6.80581649 | 189.3 | 6.80581649 | 189.3 | 5.21713886 | 189.3 | 13.5037598 | | | 189.30 | 5.1681056 | 189.3 | 9.64974557 | 189.3 | 5.1681056 |
| 189.4 | 1.25525146 | 189.4 | 6.79600984 | 189.4 | 6.79600984 | 189.4 | 5.1681056 | 189.4 | 13.5331798 | | | 189.40 | 5.1681056 | 189.4 | 9.62032561 | 189.4 | 5.1681056 |
| 189.5 | 1.29447806 | 189.5 | 6.81562314 | 189.5 | 6.81562314 | 189.5 | 5.1681056 | 189.5 | 13.5429864 | | | 189.50 | 5.1681056 | 189.5 | 9.62032561 | 189.5 | 5.1681056 |
| 189.6 | 1.26505811 | 189.6 | 6.81562314 | 189.6 | 6.81562314 | 189.6 | 5.19752556 | 189.6 | 13.5625997 | | | 189.60 | 5.15829895 | 189.6 | 9.66935887 | 189.6 | 5.15829895 |
| 189.7 | 1.25525146 | 189.7 | 6.83523644 | 189.7 | 6.83523644 | 189.7 | 5.18771891 | 189.7 | 13.5920197 | | | 189.70 | 5.20733221 | 189.7 | 9.70858548 | 189.7 | 5.20733221 |
| 189.8 | 1.2258315 | 189.8 | 6.82542979 | 189.8 | 6.82542979 | 189.8 | 5.22694552 | 189.8 | 13.5625997 | | | 189.80 | 5.19752556 | 189.8 | 9.68897218 | 189.8 | 5.19752556 |
| 189.9 | 1.2258315 | 189.9 | 6.82542979 | 189.9 | 6.82542979 | 189.9 | 5.20733221 | 189.9 | 13.6018263 | | | 189.90 | 5.1681056 | 189.9 | 9.65955222 | 189.9 | 5.1681056 |
| 190 | 1.21602485 | 190 | 6.83523644 | 190 | 6.83523644 | 190 | 5.23675217 | 190 | 13.6018263 | | | 190.00 | 5.1681056 | 190 | 9.65955222 | 190 | 5.1681056 |
| 190.1 | 1.21602485 | 190.1 | 6.85484975 | 190.1 | 6.85484975 | 190.1 | 5.23675217 | 190.1 | 13.5920197 | | | 190.10 | 5.1681056 | 190.1 | 9.67916552 | 190.1 | 5.1681056 |
| 190.2 | 1.2454448 | 190.2 | 6.85484975 | 190.2 | 6.85484975 | 190.2 | 5.19752556 | 190.2 | 13.6214396 | | | 190.20 | 5.18771891 | 190.2 | 9.70858548 | 190.2 | 5.18771891 |
| 190.3 | 1.2454448 | 190.3 | 6.85484975 | 190.3 | 6.85484975 | 190.3 | 5.17791226 | 190.3 | 13.6312463 | | | 190.30 | 5.20733221 | 190.3 | 9.71839213 | 190.3 | 5.20733221 |
| 190.4 | 1.2258315 | 190.4 | 6.8450431 | 190.4 | 6.8450431 | 190.4 | 5.19752556 | 190.4 | 13.5920197 | | | 190.40 | 5.17791226 | 190.4 | 9.70858548 | 190.4 | 5.17791226 |
| 190.5 | 1.21602485 | 190.5 | 6.82542979 | 190.5 | 6.82542979 | 190.5 | 5.20733221 | 190.5 | 13.6018263 | | | 190.50 | 5.19752556 | 190.5 | 9.71839213 | 190.5 | 5.19752556 |
| 190.6 | 1.2258315 | 190.6 | 6.83523644 | 190.6 | 6.83523644 | 190.6 | 5.22694552 | 190.6 | 13.6606662 | | | 190.60 | 5.19752556 | 190.6 | 9.73800544 | 190.6 | 5.19752556 |
| 190.7 | 1.25525146 | 190.7 | 6.8646564 | 190.7 | 6.8646564 | 190.7 | 5.22694552 | 190.7 | 13.6606662 | | | 190.70 | 5.21713886 | 190.7 | 9.73800544 | 190.7 | 5.21713886 |
| 190.8 | 1.23563815 | 190.8 | 6.89407636 | 190.8 | 6.89407636 | 190.8 | 5.21713886 | 190.8 | 13.6606662 | | | 190.80 | 5.20733221 | 190.8 | 9.72819878 | 190.8 | 5.20733221 |
| 190.9 | 1.2062182 | 190.9 | 6.87446305 | 190.9 | 6.87446305 | 190.9 | 5.25636547 | 190.9 | 13.6704729 | | | 190.90 | 5.21713886 | 190.9 | 9.74781209 | 190.9 | 5.21713886 |
| 191 | 1.23563815 | 191 | 6.91368966 | 191 | 6.91368966 | 191 | 5.27597878 | 191 | 13.6900862 | | | 191.00 | 5.22694552 | 191 | 9.74781209 | 191 | 5.22694552 |
| 191.1 | 1.21602485 | 191.1 | 6.90388301 | 191.1 | 6.90388301 | 191.1 | 5.26617212 | 191.1 | 13.7195061 | | | 191.10 | 5.23675217 | 191.1 | 9.75761874 | 191.1 | 5.23675217 |
| 191.2 | 1.18660489 | 191.2 | 6.91368966 | 191.2 | 6.91368966 | 191.2 | 5.27597878 | 191.2 | 13.7096995 | | | 191.20 | 5.23675217 | 191.2 | 9.79684535 | 191.2 | 5.23675217 |
| 191.3 | 1.15718494 | 191.3 | 6.91368966 | 191.3 | 6.91368966 | 191.3 | 5.27597878 | 191.3 | 13.7096995 | | | 191.30 | 5.24655882 | 191.3 | 9.75761874 | 191.3 | 5.24655882 |
| 191.4 | 1.18660489 | 191.4 | 6.91368966 | 191.4 | 6.91368966 | 191.4 | 5.29559208 | 191.4 | 13.7587328 | | | 191.40 | 5.24655882 | 191.4 | 9.75761874 | 191.4 | 5.24655882 |
| 191.5 | 1.18660489 | 191.5 | 6.89407636 | 191.5 | 6.89407636 | 191.5 | 5.28578543 | 191.5 | 13.7587328 | | | 191.50 | 5.23675217 | 191.5 | 9.806652 | 191.5 | 5.23675217 |
| 191.6 | 1.17679824 | 191.6 | 6.87446305 | 191.6 | 6.87446305 | 191.6 | 5.31520538 | 191.6 | 13.7489261 | | | 191.60 | 5.25636547 | 191.6 | 9.8262653 | 191.6 | 5.25636547 |
| 191.7 | 1.2062182 | 191.7 | 6.90388301 | 191.7 | 6.90388301 | 191.7 | 5.27597878 | 191.7 | 13.7587328 | | | 191.70 | 5.25636547 | 191.7 | 9.7870387 | 191.7 | 5.25636547 |
| 191.8 | 1.2062182 | 191.8 | 6.92349631 | 191.8 | 6.92349631 | 191.8 | 5.25636547 | 191.8 | 13.8175727 | | | 191.80 | 5.25636547 | 191.8 | 9.7870387 | 191.8 | 5.25636547 |
| 191.9 | 1.2062182 | 191.9 | 6.90388301 | 191.9 | 6.90388301 | 191.9 | 5.29559208 | 191.9 | 13.8273793 | | | 191.90 | 5.23675217 | 191.9 | 9.7870387 | 191.9 | 5.23675217 |
| 192 | 1.19641154 | 192 | 6.94310962 | 192 | 6.94310962 | 192 | 5.28578543 | 192 | 13.837186 | | | 192.00 | 5.25636547 | 192 | 9.806652 | 192 | 5.25636547 |
| 192.1 | 1.14737828 | 192.1 | 6.92349631 | 192.1 | 6.92349631 | 192.1 | 5.29559208 | 192.1 | 13.8469926 | | | 192.10 | 5.29559208 | 192.1 | 9.8262653 | 192.1 | 5.29559208 |
| 192.2 | 1.13757163 | 192.2 | 6.94310962 | 192.2 | 6.94310962 | 192.2 | 5.25636547 | 192.2 | 13.8567993 | | | 192.20 | 5.26617212 | 192.2 | 9.81645865 | 192.2 | 5.26617212 |
| 192.3 | 1.15718494 | 192.3 | 6.95291627 | 192.3 | 6.95291627 | 192.3 | 5.26617212 | 192.3 | 13.8764126 | | | 192.30 | 5.29559208 | 192.3 | 9.806652 | 192.3 | 5.29559208 |
| 192.4 | 1.15718494 | 192.4 | 6.96272292 | 192.4 | 6.96272292 | 192.4 | 5.31520538 | 192.4 | 13.9058325 | | | 192.40 | 5.31520538 | 192.4 | 9.8262653 | 192.4 | 5.31520538 |

| | | | | | | | | | | | | | | | | | |
|---|---|---|---|---|---|---|---|---|---|---|---|---|---|---|---|---|---|
| 192.5 | 1.11795833 | 192.5 | 6.97252957 | 192.5 | 6.97252957 | 192.5 | 5.30539873 | 192.5 | 13.9254458 | | | 192.50 | 5.28578543 | 192.5 | 9.83607196 | 192.5 | 5.28578543 |
| 192.6 | 1.10815168 | 192.6 | 6.97252957 | 192.6 | 6.97252957 | 192.6 | 5.29559208 | 192.6 | 13.9450591 | | | 192.60 | 5.29559208 | 192.6 | 9.83607196 | 192.6 | 5.29559208 |
| 192.7 | 1.13757163 | 192.7 | 6.98233622 | 192.7 | 6.98233622 | 192.7 | 5.28578543 | 192.7 | 13.9548658 | | | 192.70 | 5.32501204 | 192.7 | 9.8262653 | 192.7 | 5.32501204 |
| 192.8 | 1.12776498 | 192.8 | 6.98233622 | 192.8 | 6.98233622 | 192.8 | 5.29559208 | 192.8 | 13.9450591 | | | 192.80 | 5.28578543 | 192.8 | 9.84587861 | 192.8 | 5.28578543 |
| 192.9 | 1.11795833 | 192.9 | 6.96272292 | 192.9 | 6.96272292 | 192.9 | 5.34462534 | 192.9 | 13.9548658 | | | 192.90 | 5.28578543 | 192.9 | 9.84587861 | 192.9 | 5.28578543 |
| 193 | 1.12776498 | 193 | 6.97252957 | 193 | 6.97252957 | 193 | 5.32501204 | 193 | 13.9548658 | | | 193.00 | 5.30539873 | 193 | 9.88510522 | 193 | 5.30539873 |
| 193.1 | 1.13757163 | 193.1 | 6.98233622 | 193.1 | 6.98233622 | 193.1 | 5.28578543 | 193.1 | 13.9744791 | | | 193.10 | 5.32501204 | 193.1 | 9.88510522 | 193.1 | 5.32501204 |
| 193.2 | 1.11795833 | 193.2 | 6.98233622 | 193.2 | 6.98233622 | 193.2 | 5.27597878 | 193.2 | 13.9744791 | | | 193.20 | 5.34462534 | 193.2 | 9.86549191 | 193.2 | 5.34462534 |
| 193.3 | 1.10815168 | 193.3 | 7.00194953 | 193.3 | 7.00194953 | 193.3 | 5.29559208 | 193.3 | 13.9842858 | | | 193.30 | 5.32501204 | 193.3 | 9.83607196 | 193.3 | 5.32501204 |
| 193.4 | 1.09834502 | 193.4 | 7.03136948 | 193.4 | 7.03136948 | 193.4 | 5.29559208 | 193.4 | 13.9842858 | | | 193.40 | 5.34462534 | 193.4 | 9.85568526 | 193.4 | 5.34462534 |
| 193.5 | 1.08853837 | 193.5 | 7.03136948 | 193.5 | 7.03136948 | 193.5 | 5.29559208 | 193.5 | 13.9940924 | | | 193.50 | 5.33481869 | 193.5 | 9.88510522 | 193.5 | 5.33481869 |
| 193.6 | 1.05911842 | 193.6 | 7.01175618 | 193.6 | 7.01175618 | 193.6 | 5.27597878 | 193.6 | 14.0137057 | | | 193.60 | 5.30539873 | 193.6 | 9.90471852 | 193.6 | 5.30539873 |
| 193.7 | 1.08853837 | 193.7 | 7.04117614 | 193.7 | 7.04117614 | 193.7 | 5.26617212 | 193.7 | 14.0431257 | | | 193.70 | 5.33481869 | 193.7 | 9.89491187 | 193.7 | 5.33481869 |
| 193.8 | 1.07873172 | 193.8 | 7.04117614 | 193.8 | 7.04117614 | 193.8 | 5.25636547 | 193.8 | 14.062739 | | | 193.80 | 5.32501204 | 193.8 | 9.88510522 | 193.8 | 5.32501204 |
| 193.9 | 1.08853837 | 193.9 | 7.02156283 | 193.9 | 7.02156283 | 193.9 | 5.29559208 | 193.9 | 14.0823523 | | | 193.90 | 5.28578543 | 193.9 | 9.89491187 | 193.9 | 5.28578543 |
| 194 | 1.10815168 | 194 | 7.00194953 | 194 | 7.00194953 | 194 | 5.28578543 | 194 | 14.0725456 | | | 194.00 | 5.30539873 | 194 | 9.90471852 | 194 | 5.30539873 |
| 194.1 | 1.06892507 | 194.1 | 7.03136948 | 194.1 | 7.03136948 | 194.1 | 5.27597878 | 194.1 | 14.062739 | | | 194.10 | 5.32501204 | 194.1 | 9.91452517 | 194.1 | 5.32501204 |
| 194.2 | 1.04931176 | 194.2 | 7.05098279 | 194.2 | 7.05098279 | 194.2 | 5.30539873 | 194.2 | 14.1019656 | | | 194.20 | 5.36423864 | 194.2 | 9.92433182 | 194.2 | 5.36423864 |
| 194.3 | 1.07873172 | 194.3 | 7.02156283 | 194.3 | 7.02156283 | 194.3 | 5.36423864 | 194.3 | 14.0725456 | | | 194.30 | 5.36423864 | 194.3 | 9.93413848 | 194.3 | 5.36423864 |
| 194.4 | 1.09834502 | 194.4 | 7.05098279 | 194.4 | 7.05098279 | 194.4 | 5.36423864 | 194.4 | 14.0725456 | | | 194.40 | 5.35443199 | 194.4 | 9.93413848 | 194.4 | 5.35443199 |
| 194.5 | 1.08853837 | 194.5 | 7.07059609 | 194.5 | 7.07059609 | 194.5 | 5.34462534 | 194.5 | 14.1313855 | | | 194.50 | 5.34462534 | 194.5 | 9.93413848 | 194.5 | 5.34462534 |
| 194.6 | 1.06892507 | 194.6 | 7.04117614 | 194.6 | 7.04117614 | 194.6 | 5.34462534 | 194.6 | 14.1313855 | | | 194.60 | 5.34462534 | 194.6 | 9.95375178 | 194.6 | 5.34462534 |
| 194.7 | 1.03950511 | 194.7 | 7.06078944 | 194.7 | 7.06078944 | 194.7 | 5.35443199 | 194.7 | 14.1706121 | | | 194.70 | 5.36423864 | 194.7 | 9.95375178 | 194.7 | 5.36423864 |
| 194.8 | 1.06892507 | 194.8 | 7.07059609 | 194.8 | 7.07059609 | 194.8 | 5.3740453 | 194.8 | 14.2000321 | | | 194.80 | 5.3740453 | 194.8 | 9.94394513 | 194.8 | 5.3740453 |
| 194.9 | 1.09834502 | 194.9 | 7.08040274 | 194.9 | 7.08040274 | 194.9 | 5.38385195 | 194.9 | 14.1902254 | | | 194.90 | 5.35443199 | 194.9 | 9.98317174 | 194.9 | 5.35443199 |
| 195 | 1.07873172 | 195 | 7.06078944 | 195 | 7.06078944 | 195 | 5.36423864 | 195 | 14.1902254 | | | 195.00 | 5.38385195 | 195 | 9.98317174 | 195 | 5.38385195 |
| 195.1 | 1.10815168 | 195.1 | 7.06078944 | 195.1 | 7.06078944 | 195.1 | 5.3740453 | 195.1 | 14.2098387 | | | 195.10 | 5.38385195 | 195.1 | 9.95375178 | 195.1 | 5.38385195 |
| 195.2 | 1.09834502 | 195.2 | 7.129436 | 195.2 | 7.129436 | 195.2 | 5.33481869 | 195.2 | 14.2000321 | | | 195.20 | 5.36423864 | 195.2 | 9.95375178 | 195.2 | 5.36423864 |
| 195.3 | 1.07873172 | 195.3 | 7.1098227 | 195.3 | 7.1098227 | 195.3 | 5.35443199 | 195.3 | 14.2196454 | | | 195.30 | 5.3740453 | 195.3 | 9.96355843 | 195.3 | 5.3740453 |
| 195.4 | 1.04931176 | 195.4 | 7.10001605 | 195.4 | 7.10001605 | 195.4 | 5.35443199 | 195.4 | 14.2392587 | | | 195.40 | 5.40346525 | 195.4 | 9.99297839 | 195.4 | 5.40346525 |
| 195.5 | 1.03950511 | 195.5 | 7.11962935 | 195.5 | 7.11962935 | 195.5 | 5.34462534 | 195.5 | 14.258872 | | | 195.50 | 5.3936586 | 195.5 | 9.99297839 | 195.5 | 5.3936586 |
| 195.6 | 1.03950511 | 195.6 | 7.14904931 | 195.6 | 7.14904931 | 195.6 | 5.33481869 | 195.6 | 14.258872 | | | 195.60 | 5.3936586 | 195.6 | 9.99297839 | 195.6 | 5.3936586 |
| 195.7 | 1.01989181 | 195.7 | 7.15885596 | 195.7 | 7.15885596 | 195.7 | 5.28578543 | 195.7 | 14.288292 | | | 195.70 | 5.3936586 | 195.7 | 9.98317174 | 195.7 | 5.3936586 |
| 195.8 | 1.06892507 | 195.8 | 7.13924266 | 195.8 | 7.13924266 | 195.8 | 5.26617212 | 195.8 | 14.2392587 | | | 195.80 | 5.35443199 | 195.8 | 9.97336508 | 195.8 | 5.35443199 |
| 195.9 | 1.06892507 | 195.9 | 7.11962935 | 195.9 | 7.11962935 | 195.9 | 5.28578543 | 195.9 | 14.258872 | | | 195.90 | 5.40346525 | 195.9 | 10.002785 | 195.9 | 5.40346525 |
| 196 | 1.05911842 | 196 | 7.17846926 | 196 | 7.17846926 | 196 | 5.31520538 | 196 | 14.288292 | | | 196.00 | 5.4132719 | 196 | 10.0125917 | 196 | 5.4132719 |
| 196.1 | 1.06892507 | 196.1 | 7.17846926 | 196.1 | 7.17846926 | 196.1 | 5.33481869 | 196.1 | 14.3373252 | | | 196.10 | 5.40346525 | 196.1 | 10.0125917 | 196.1 | 5.40346525 |
| 196.2 | 1.10815168 | 196.2 | 7.15885596 | 196.2 | 7.15885596 | 196.2 | 5.35443199 | 196.2 | 14.3471319 | | | 196.20 | 5.42307856 | 196.2 | 10.0223983 | 196.2 | 5.42307856 |
| 196.3 | 1.06892507 | 196.3 | 7.16866261 | 196.3 | 7.16866261 | 196.3 | 5.36423864 | 196.3 | 14.3471319 | | | 196.30 | 5.40346525 | 196.3 | 10.061625 | 196.3 | 5.40346525 |
| 196.4 | 1.06892507 | 196.4 | 7.16866261 | 196.4 | 7.16866261 | 196.4 | 5.3740453 | 196.4 | 14.3373252 | | | 196.40 | 5.4132719 | 196.4 | 10.032205 | 196.4 | 5.4132719 |
| 196.5 | 1.07873172 | 196.5 | 7.16866261 | 196.5 | 7.16866261 | 196.5 | 5.36423864 | 196.5 | 14.3569385 | | | 196.50 | 5.45249851 | 196.5 | 10.0223983 | 196.5 | 5.45249851 |
| 196.6 | 1.04931176 | 196.6 | 7.18827592 | 196.6 | 7.18827592 | 196.6 | 5.35443199 | 196.6 | 14.3667452 | | | 196.60 | 5.43288521 | 196.6 | 10.061625 | 196.6 | 5.43288521 |
| 196.7 | 1.07873172 | 196.7 | 7.20788922 | 196.7 | 7.20788922 | 196.7 | 5.3740453 | 196.7 | 14.3765518 | | | 196.70 | 5.43288521 | 196.7 | 10.061625 | 196.7 | 5.43288521 |
| 196.8 | 1.09834502 | 196.8 | 7.21769587 | 196.8 | 7.21769587 | 196.8 | 5.3740453 | 196.8 | 14.3961651 | | | 196.80 | 5.44269186 | 196.8 | 10.061625 | 196.8 | 5.44269186 |
| 196.9 | 1.07873172 | 196.9 | 7.19808257 | 196.9 | 7.19808257 | 196.9 | 5.38385195 | 196.9 | 14.4353917 | | | 196.90 | 5.44269186 | 196.9 | 10.0910449 | 196.9 | 5.44269186 |
| 197 | 1.06892507 | 197 | 7.23730918 | 197 | 7.23730918 | 197 | 5.36423864 | 197 | 14.4255851 | | | 197.00 | 5.44269186 | 197 | 10.1204649 | 197 | 5.44269186 |
| 197.1 | 1.08853837 | 197.1 | 7.24711583 | 197.1 | 7.24711583 | 197.1 | 5.38385195 | 197.1 | 14.4157784 | | | 197.10 | 5.47211182 | 197.1 | 10.1106582 | 197.1 | 5.47211182 |
| 197.2 | 1.07873172 | 197.2 | 7.23730918 | 197.2 | 7.23730918 | 197.2 | 5.3740453 | 197.2 | 14.455005 | | | 197.20 | 5.48191847 | 197.2 | 10.0910449 | 197.2 | 5.48191847 |
| 197.3 | 1.05911842 | 197.3 | 7.21769587 | 197.3 | 7.21769587 | 197.3 | 5.38385195 | 197.3 | 14.4746184 | | | 197.30 | 5.44269186 | 197.3 | 10.0910449 | 197.3 | 5.44269186 |
| 197.4 | 1.07873172 | 197.4 | 7.21769587 | 197.4 | 7.21769587 | 197.4 | 5.3740453 | 197.4 | 14.4648117 | | | 197.40 | 5.45249851 | 197.4 | 10.0910449 | 197.4 | 5.45249851 |
| 197.5 | 1.04931176 | 197.5 | 7.21769587 | 197.5 | 7.21769587 | 197.5 | 5.38385195 | 197.5 | 14.4746184 | | | 197.50 | 5.45249851 | 197.5 | 10.0910449 | 197.5 | 5.45249851 |
| 197.6 | 1.04931176 | 197.6 | 7.23730918 | 197.6 | 7.23730918 | 197.6 | 5.40346525 | 197.6 | 14.4746184 | | | 197.60 | 5.46230516 | 197.6 | 10.1008516 | 197.6 | 5.46230516 |
| 197.7 | 1.05911842 | 197.7 | 7.26672913 | 197.7 | 7.26672913 | 197.7 | 5.43288521 | 197.7 | 14.484425 | | | 197.70 | 5.46230516 | 197.7 | 10.1302715 | 197.7 | 5.46230516 |
| 197.8 | 1.03950511 | 197.8 | 7.27653578 | 197.8 | 7.27653578 | 197.8 | 5.42307856 | 197.8 | 14.513845 | | | 197.80 | 5.47211182 | 197.8 | 10.1694981 | 197.8 | 5.47211182 |
| 197.9 | 1.05911842 | 197.9 | 7.27653578 | 197.9 | 7.27653578 | 197.9 | 5.47211182 | 197.9 | 14.513845 | | | 197.90 | 5.47211182 | 197.9 | 10.1596915 | 197.9 | 5.47211182 |
| 198 | 1.06892507 | 198 | 7.27653578 | 198 | 7.27653578 | 198 | 5.45249851 | 198 | 14.5040383 | | | 198.00 | 5.47211182 | 198 | 10.1596915 | 198 | 5.47211182 |
| 198.1 | 1.07873172 | 198.1 | 7.27653578 | 198.1 | 7.27653578 | 198.1 | 5.42307856 | 198.1 | 14.5236516 | | | 198.10 | 5.48191847 | 198.1 | 10.1596915 | 198.1 | 5.48191847 |
| 198.2 | 1.06892507 | 198.2 | 7.29614909 | 198.2 | 7.29614909 | 198.2 | 5.44269186 | 198.2 | 14.5334583 | | | 198.20 | 5.48191847 | 198.2 | 10.1694981 | 198.2 | 5.48191847 |
| 198.3 | 1.05911842 | 198.3 | 7.29614909 | 198.3 | 7.29614909 | 198.3 | 5.45249851 | 198.3 | 14.5530716 | | | 198.30 | 5.48191847 | 198.3 | 10.1793048 | 198.3 | 5.48191847 |
| 198.4 | 1.06892507 | 198.4 | 7.30595574 | 198.4 | 7.30595574 | 198.4 | 5.47211182 | 198.4 | 14.5824915 | | | 198.40 | 5.50153177 | 198.4 | 10.1596915 | 198.4 | 5.50153177 |
| 198.5 | 1.06892507 | 198.5 | 7.29614909 | 198.5 | 7.29614909 | 198.5 | 5.49172512 | 198.5 | 14.5922982 | | | 198.50 | 5.51133842 | 198.5 | 10.1596915 | 198.5 | 5.51133842 |

| | | | | | | | | | | | | | | | | | |
|---|---|---|---|---|---|---|---|---|---|---|---|---|---|---|---|---|---|
| 198.6 | 1.06892507 | 198.6 | 7.29614909 | 198.6 | 7.29614909 | 198.6 | 5.51133842 | 198.6 | 14.5922982 | | | 198.60 | 5.49172512 | 198.6 | 10.1596915 | 198.6 | 5.49172512 |
| 198.7 | 1.05911842 | 198.7 | 7.31576239 | 198.7 | 7.31576239 | 198.7 | 5.53095173 | 198.7 | 14.5922982 | | | 198.70 | 5.48191847 | 198.7 | 10.1498848 | 198.7 | 5.48191847 |
| 198.8 | 1.04931176 | 198.8 | 7.3353757 | 198.8 | 7.3353757 | 198.8 | 5.53095173 | 198.8 | 14.6021048 | | | 198.80 | 5.52114508 | 198.8 | 10.1596915 | 198.8 | 5.52114508 |
| 198.9 | 1.06892507 | 198.9 | 7.30595574 | 198.9 | 7.30595574 | 198.9 | 5.49172512 | 198.9 | 14.6217181 | | | 198.90 | 5.53095173 | 198.9 | 10.1891114 | 198.9 | 5.53095173 |
| 199 | 1.02969846 | 199 | 7.3353757 | 199 | 7.3353757 | 199 | 5.48191847 | 199 | 14.6315248 | | | 199.00 | 5.49172512 | 199 | 10.1989181 | 199 | 5.49172512 |
| 199.1 | 1.05911842 | 199.1 | 7.36479565 | 199.1 | 7.36479565 | 199.1 | 5.43288521 | 199.1 | 14.6609447 | | | 199.10 | 5.50153177 | 199.1 | 10.2087247 | 199.1 | 5.50153177 |
| 199.2 | 1.07873172 | 199.2 | 7.354989 | 199.2 | 7.354989 | 199.2 | 5.48191847 | 199.2 | 14.6707514 | | | 199.20 | 5.52114508 | 199.2 | 10.228338 | 199.2 | 5.52114508 |
| 199.3 | 1.04931176 | 199.3 | 7.354989 | 199.3 | 7.354989 | 199.3 | 5.52114508 | 199.3 | 14.680558 | | | 199.30 | 5.55056503 | 199.3 | 10.228338 | 199.3 | 5.55056503 |
| 199.4 | 1.02969846 | 199.4 | 7.34518235 | 199.4 | 7.34518235 | 199.4 | 5.55056503 | 199.4 | 14.6903647 | | | 199.40 | 5.55056503 | 199.4 | 10.2479513 | 199.4 | 5.55056503 |
| 199.5 | 1.04931176 | 199.5 | 7.36479565 | 199.5 | 7.36479565 | 199.5 | 5.50153177 | 199.5 | 14.709978 | | | 199.50 | 5.56037168 | 199.5 | 10.228338 | 199.5 | 5.56037168 |
| 199.6 | 1.06892507 | 199.6 | 7.36479565 | 199.6 | 7.36479565 | 199.6 | 5.49172512 | 199.6 | 14.709978 | | | 199.60 | 5.54075838 | 199.6 | 10.228338 | 199.6 | 5.54075838 |
| 199.7 | 1.07873172 | 199.7 | 7.354989 | 199.7 | 7.354989 | 199.7 | 5.51133842 | 199.7 | 14.709978 | | | 199.70 | 5.54075838 | 199.7 | 10.2675646 | 199.7 | 5.54075838 |
| 199.8 | 1.07873172 | 199.8 | 7.354989 | 199.8 | 7.354989 | 199.8 | 5.51133842 | 199.8 | 14.7295913 | | | 199.80 | 5.57998499 | 199.8 | 10.2675646 | 199.8 | 5.57998499 |
| 199.9 | 1.06892507 | 199.9 | 7.3746023 | 199.9 | 7.3746023 | 199.9 | 5.52114508 | 199.9 | 14.7197847 | | | 199.90 | 5.57017834 | 199.9 | 10.257758 | 199.9 | 5.57017834 |
| 200 | 1.06892507 | 200 | 7.39421561 | 200 | 7.39421561 | 200 | 5.53095173 | 200 | 14.709978 | | | 200.00 | 5.56037168 | 200 | 10.2381447 | 200 | 5.56037168 |
| 200.1 | 1.06892507 | 200.1 | 7.43344222 | 200.1 | 7.43344222 | 200.1 | 5.54075838 | 200.1 | 14.739398 | | | 200.10 | 5.57017834 | 200.1 | 10.2675646 | 200.1 | 5.57017834 |
| 200.2 | 1.07873172 | 200.2 | 7.43344222 | 200.2 | 7.43344222 | 200.2 | 5.51133842 | 200.2 | 14.7688179 | | | 200.20 | 5.57017834 | 200.2 | 10.2773713 | 200.2 | 5.57017834 |
| 200.3 | 1.09834502 | 200.3 | 7.44324887 | 200.3 | 7.44324887 | 200.3 | 5.52114508 | 200.3 | 14.7786246 | | | 200.30 | 5.57017834 | 200.3 | 10.2773713 | 200.3 | 5.57017834 |
| 200.4 | 1.09834502 | 200.4 | 7.44324887 | 200.4 | 7.44324887 | 200.4 | 5.55056503 | 200.4 | 14.7884312 | | | 200.40 | 5.56037168 | 200.4 | 10.257758 | 200.4 | 5.56037168 |
| 200.5 | 1.06892507 | 200.5 | 7.45305552 | 200.5 | 7.45305552 | 200.5 | 5.56037168 | 200.5 | 14.8276578 | | | 200.50 | 5.57998499 | 200.5 | 10.2773713 | 200.5 | 5.57998499 |
| 200.6 | 1.07873172 | 200.6 | 7.45305552 | 200.6 | 7.45305552 | 200.6 | 5.56037168 | 200.6 | 14.8374645 | | | 200.60 | 5.56037168 | 200.6 | 10.2969846 | 200.6 | 5.56037168 |
| 200.7 | 1.05911842 | 200.7 | 7.45305552 | 200.7 | 7.45305552 | 200.7 | 5.57998499 | 200.7 | 14.8276578 | | | 200.70 | 5.55056503 | 200.7 | 10.3165979 | 200.7 | 5.55056503 |
| 200.8 | 1.06892507 | 200.8 | 7.43344222 | 200.8 | 7.43344222 | 200.8 | 5.57017834 | 200.8 | 14.8276578 | | | 200.80 | 5.54075838 | 200.8 | 10.3165979 | 200.8 | 5.54075838 |
| 200.9 | 1.05911842 | 200.9 | 7.43344222 | 200.9 | 7.43344222 | 200.9 | 5.56037168 | 200.9 | 14.8374645 | | | 200.90 | 5.57017834 | 200.9 | 10.3264046 | 200.9 | 5.57017834 |
| 201 | 1.07873172 | 201 | 7.47266882 | 201 | 7.47266882 | 201 | 5.57998499 | 201 | 14.8374645 | | | 201.00 | 5.57017834 | 201 | 10.3165979 | 201 | 5.57017834 |
| 201.1 | 1.07873172 | 201.1 | 7.45305552 | 201.1 | 7.45305552 | 201.1 | 5.56037168 | 201.1 | 14.8472711 | | | 201.10 | 5.55056503 | 201.1 | 10.3264046 | 201.1 | 5.55056503 |
| 201.2 | 1.13757163 | 201.2 | 7.45305552 | 201.2 | 7.45305552 | 201.2 | 5.56037168 | 201.2 | 14.906111 | | | 201.20 | 5.57998499 | 201.2 | 10.3558245 | 201.2 | 5.57998499 |
| 201.3 | 1.11795833 | 201.3 | 7.49228213 | 201.3 | 7.49228213 | 201.3 | 5.59959829 | 201.3 | 14.9257243 | | | 201.30 | 5.57998499 | 201.3 | 10.3656312 | 201.3 | 5.57998499 |
| 201.4 | 1.07873172 | 201.4 | 7.48247548 | 201.4 | 7.48247548 | 201.4 | 5.62901825 | 201.4 | 14.8963044 | | | 201.40 | 5.59959829 | 201.4 | 10.3460179 | 201.4 | 5.59959829 |
| 201.5 | 1.04931176 | 201.5 | 7.52170208 | 201.5 | 7.52170208 | 201.5 | 5.6192116 | 201.5 | 14.8963044 | | | 201.50 | 5.6192116 | 201.5 | 10.3558245 | 201.5 | 5.6192116 |
| 201.6 | 1.08853837 | 201.6 | 7.53150874 | 201.6 | 7.53150874 | 201.6 | 5.6192116 | 201.6 | 14.935531 | | | 201.60 | 5.60940494 | 201.6 | 10.3558245 | 201.6 | 5.60940494 |
| 201.7 | 1.08853837 | 201.7 | 7.53150874 | 201.7 | 7.53150874 | 201.7 | 5.60940494 | 201.7 | 14.9257243 | | | 201.70 | 5.62901825 | 201.7 | 10.3754378 | 201.7 | 5.62901825 |
| 201.8 | 1.07873172 | 201.8 | 7.53150874 | 201.8 | 7.53150874 | 201.8 | 5.6192116 | 201.8 | 14.935531 | | | 201.80 | 5.6192116 | 201.8 | 10.3852445 | 201.8 | 5.6192116 |
| 201.9 | 1.08853837 | 201.9 | 7.50208878 | 201.9 | 7.50208878 | 201.9 | 5.59959829 | 201.9 | 14.9257243 | | | 201.90 | 5.62901825 | 201.9 | 10.3754378 | 201.9 | 5.62901825 |
| 202 | 1.06892507 | 202 | 7.51189543 | 202 | 7.51189543 | 202 | 5.60940494 | 202 | 14.9453376 | | | 202.00 | 5.6192116 | 202 | 10.3754378 | 202 | 5.6192116 |
| 202.1 | 1.04931176 | 202.1 | 7.52170208 | 202.1 | 7.52170208 | 202.1 | 5.6388249 | 202.1 | 14.9845643 | | | 202.10 | 5.60940494 | 202.1 | 10.3558245 | 202.1 | 5.60940494 |
| 202.2 | 1.07873172 | 202.2 | 7.52170208 | 202.2 | 7.52170208 | 202.2 | 5.6192116 | 202.2 | 15.0041776 | | | 202.20 | 5.64863155 | 202.2 | 10.3264046 | 202.2 | 5.64863155 |
| 202.3 | 1.06892507 | 202.3 | 7.51189543 | 202.3 | 7.51189543 | 202.3 | 5.6388249 | 202.3 | 15.0335975 | | | 202.30 | 5.66824486 | 202.3 | 10.3362112 | 202.3 | 5.66824486 |
| 202.4 | 1.07873172 | 202.4 | 7.53150874 | 202.4 | 7.53150874 | 202.4 | 5.6584382 | 202.4 | 15.0532108 | | | 202.40 | 5.62901825 | 202.4 | 10.3460179 | 202.4 | 5.62901825 |
| 202.5 | 1.09834502 | 202.5 | 7.55112204 | 202.5 | 7.55112204 | 202.5 | 5.6584382 | 202.5 | 15.0237909 | | | 202.50 | 5.62901825 | 202.5 | 10.3656312 | 202.5 | 5.62901825 |
| 202.6 | 1.05911842 | 202.6 | 7.54131539 | 202.6 | 7.54131539 | 202.6 | 5.66824486 | 202.6 | 15.0335975 | | | 202.60 | 5.6388249 | 202.6 | 10.3852445 | 202.6 | 5.6388249 |
| 202.7 | 1.04931176 | 202.7 | 7.56092869 | 202.7 | 7.56092869 | 202.7 | 5.6584382 | 202.7 | 15.0924374 | | | 202.70 | 5.66824486 | 202.7 | 10.3852445 | 202.7 | 5.66824486 |
| 202.8 | 1.05911842 | 202.8 | 7.57073534 | 202.8 | 7.57073534 | 202.8 | 5.67805151 | 202.8 | 15.0924374 | | | 202.80 | 5.6584382 | 202.8 | 10.4146644 | 202.8 | 5.6584382 |
| 202.9 | 1.10815168 | 202.9 | 7.580542 | 202.9 | 7.580542 | 202.9 | 5.66824486 | 202.9 | 15.0924374 | | | 202.90 | 5.6584382 | 202.9 | 10.3950511 | 202.9 | 5.6584382 |
| 203 | 1.10815168 | 203 | 7.59034865 | 203 | 7.59034865 | 203 | 5.66824486 | 203 | 15.1022441 | | | 203.00 | 5.64863155 | 203 | 10.3754378 | 203 | 5.64863155 |
| 203.1 | 1.12776498 | 203.1 | 7.56092869 | 203.1 | 7.56092869 | 203.1 | 5.69766481 | 203.1 | 15.1218574 | | | 203.10 | 5.6584382 | 203.1 | 10.3950511 | 203.1 | 5.6584382 |
| 203.2 | 1.13757163 | 203.2 | 7.59034865 | 203.2 | 7.59034865 | 203.2 | 5.73689142 | 203.2 | 15.131664 | | | 203.20 | 5.66824486 | 203.2 | 10.4342777 | 203.2 | 5.66824486 |
| 203.3 | 1.12776498 | 203.3 | 7.60996195 | 203.3 | 7.60996195 | 203.3 | 5.74669807 | 203.3 | 15.161084 | | | 203.30 | 5.69766481 | 203.3 | 10.4636977 | 203.3 | 5.69766481 |
| 203.4 | 1.12776498 | 203.4 | 7.59034865 | 203.4 | 7.59034865 | 203.4 | 5.70747146 | 203.4 | 15.161084 | | | 203.40 | 5.70747146 | 203.4 | 10.483311 | 203.4 | 5.70747146 |
| 203.5 | 1.10815168 | 203.5 | 7.580542 | 203.5 | 7.580542 | 203.5 | 5.70747146 | 203.5 | 15.1905039 | | | 203.50 | 5.67805151 | 203.5 | 10.483311 | 203.5 | 5.67805151 |
| 203.6 | 1.08853837 | 203.6 | 7.6001553 | 203.6 | 7.6001553 | 203.6 | 5.71727812 | 203.6 | 15.1806973 | | | 203.60 | 5.67805151 | 203.6 | 10.4735043 | 203.6 | 5.67805151 |
| 203.7 | 1.10815168 | 203.7 | 7.60996195 | 203.7 | 7.60996195 | 203.7 | 5.68785816 | 203.7 | 15.2003106 | | | 203.70 | 5.67805151 | 203.7 | 10.4931176 | 203.7 | 5.67805151 |
| 203.8 | 1.11795833 | 203.8 | 7.63938191 | 203.8 | 7.63938191 | 203.8 | 5.68785816 | 203.8 | 15.2199239 | | | 203.80 | 5.67805151 | 203.8 | 10.5225376 | 203.8 | 5.67805151 |
| 203.9 | 1.09834502 | 203.9 | 7.63938191 | 203.9 | 7.63938191 | 203.9 | 5.68785816 | 203.9 | 15.2199239 | | | 203.90 | 5.67805151 | 203.9 | 10.5323442 | 203.9 | 5.67805151 |
| 204 | 1.10815168 | 204 | 7.63938191 | 204 | 7.63938191 | 204 | 5.68785816 | 204 | 15.2297306 | | | 204.00 | 5.69766481 | 204 | 10.5127309 | 204 | 5.69766481 |
| 204.1 | 1.07873172 | 204.1 | 7.64918856 | 204.1 | 7.64918856 | 204.1 | 5.69766481 | 204.1 | 15.2297306 | | | 204.10 | 5.70747146 | 204.1 | 10.5029243 | 204.1 | 5.70747146 |
| 204.2 | 1.11795833 | 204.2 | 7.63938191 | 204.2 | 7.63938191 | 204.2 | 5.71727812 | 204.2 | 15.2297306 | | | 204.20 | 5.68785816 | 204.2 | 10.5323442 | 204.2 | 5.68785816 |
| 204.3 | 1.15718494 | 204.3 | 7.65899521 | 204.3 | 7.65899521 | 204.3 | 5.71727812 | 204.3 | 15.2689572 | | | 204.30 | 5.69766481 | 204.3 | 10.5715709 | 204.3 | 5.69766481 |
| 204.4 | 1.11795833 | 204.4 | 7.64918856 | 204.4 | 7.64918856 | 204.4 | 5.70747146 | 204.4 | 15.2983771 | | | 204.40 | 5.71727812 | 204.4 | 10.5617642 | 204.4 | 5.71727812 |
| 204.5 | 1.10815168 | 204.5 | 7.66880186 | 204.5 | 7.66880186 | 204.5 | 5.68785816 | 204.5 | 15.3179904 | | | 204.50 | 5.71727812 | 204.5 | 10.5323442 | 204.5 | 5.71727812 |
| 204.6 | 1.11795833 | 204.6 | 7.66880186 | 204.6 | 7.66880186 | 204.6 | 5.68785816 | 204.6 | 15.3081838 | | | 204.60 | 5.68785816 | 204.6 | 10.5617642 | 204.6 | 5.68785816 |

| | | | | | | | | | | | | | | | | | |
|---|---|---|---|---|---|---|---|---|---|---|---|---|---|---|---|---|---|
| 204.7 | 1.12776498 | 204.7 | 7.63938191 | 204.7 | 7.63938191 | 204.7 | 5.69766481 | 204.7 | 15.3081838 | | | 204.70 | 5.70747146 | 204.7 | 10.5813775 | 204.7 | 5.70747146 |
| 204.8 | 1.11795833 | 204.8 | 7.65899521 | 204.8 | 7.65899521 | 204.8 | 5.69766481 | 204.8 | 15.3179904 | | | 204.80 | 5.71727812 | 204.8 | 10.5911842 | 204.8 | 5.71727812 |
| 204.9 | 1.10815168 | 204.9 | 7.63938191 | 204.9 | 7.63938191 | 204.9 | 5.72708477 | 204.9 | 15.3277971 | | | 204.90 | 5.73689142 | 204.9 | 10.6304108 | 204.9 | 5.73689142 |
| 205 | 1.09834502 | 205 | 7.6197686 | 205 | 7.6197686 | 205 | 5.71727812 | 205 | 15.3474104 | | | 205.00 | 5.70747146 | 205 | 10.5911842 | 205 | 5.70747146 |
| 205.1 | 1.11795833 | 205.1 | 7.70802847 | 205.1 | 7.70802847 | 205.1 | 5.70747146 | 205.1 | 15.3768303 | | | 205.10 | 5.69766481 | 205.1 | 10.5519576 | 205.1 | 5.69766481 |
| 205.2 | 1.11795833 | 205.2 | 7.70802847 | 205.2 | 7.70802847 | 205.2 | 5.73689142 | 205.2 | 15.3964436 | | | 205.20 | 5.70747146 | 205.2 | 10.5715709 | 205.2 | 5.70747146 |
| 205.3 | 1.10815168 | 205.3 | 7.71783512 | 205.3 | 7.71783512 | 205.3 | 5.76631138 | 205.3 | 15.4062503 | | | 205.30 | 5.72708477 | 205.3 | 10.6107975 | 205.3 | 5.72708477 |
| 205.4 | 1.12776498 | 205.4 | 7.71783512 | 205.4 | 7.71783512 | 205.4 | 5.76631138 | 205.4 | 15.3964436 | | | 205.40 | 5.73689142 | 205.4 | 10.6206041 | 205.4 | 5.73689142 |
| 205.5 | 1.09834502 | 205.5 | 7.72764178 | 205.5 | 7.72764178 | 205.5 | 5.79573133 | 205.5 | 15.386637 | | | 205.50 | 5.73689142 | 205.5 | 10.6206041 | 205.5 | 5.73689142 |
| 205.6 | 1.09834502 | 205.6 | 7.71783512 | 205.6 | 7.71783512 | 205.6 | 5.80553798 | 205.6 | 15.4258636 | | | 205.60 | 5.71727812 | 205.6 | 10.6206041 | 205.6 | 5.71727812 |
| 205.7 | 1.14737828 | 205.7 | 7.73744843 | 205.7 | 7.73744843 | 205.7 | 5.78592468 | 205.7 | 15.4552836 | | | 205.70 | 5.72708477 | 205.7 | 10.6500241 | 205.7 | 5.72708477 |
| 205.8 | 1.15718494 | 205.8 | 7.72764178 | 205.8 | 7.72764178 | 205.8 | 5.77611803 | 205.8 | 15.4650902 | | | 205.80 | 5.73689142 | 205.8 | 10.6304108 | 205.8 | 5.73689142 |
| 205.9 | 1.11795833 | 205.9 | 7.72764178 | 205.9 | 7.72764178 | 205.9 | 5.75650472 | 205.9 | 15.4552836 | | | 205.90 | 5.73689142 | 205.9 | 10.6206041 | 205.9 | 5.73689142 |
| 206 | 1.15718494 | 206 | 7.72764178 | 206 | 7.72764178 | 206 | 5.75650472 | 206 | 15.4650902 | | | 206.00 | 5.72708477 | 206 | 10.6500241 | 206 | 5.72708477 |
| 206.1 | 1.15718494 | 206.1 | 7.72764178 | 206.1 | 7.72764178 | 206.1 | 5.78592468 | 206.1 | 15.4748969 | | | 206.10 | 5.74669807 | 206.1 | 10.6598307 | 206.1 | 5.74669807 |
| 206.2 | 1.13757163 | 206.2 | 7.73744843 | 206.2 | 7.73744843 | 206.2 | 5.79573133 | 206.2 | 15.5043168 | | | 206.20 | 5.76631138 | 206.2 | 10.6598307 | 206.2 | 5.76631138 |
| 206.3 | 1.09834502 | 206.3 | 7.73744843 | 206.3 | 7.73744843 | 206.3 | 5.77611803 | 206.3 | 15.5337368 | | | 206.30 | 5.74669807 | 206.3 | 10.6696374 | 206.3 | 5.74669807 |
| 206.4 | 1.11795833 | 206.4 | 7.71783512 | 206.4 | 7.71783512 | 206.4 | 5.80553798 | 206.4 | 15.5043168 | | | 206.40 | 5.75650472 | 206.4 | 10.6598307 | 206.4 | 5.75650472 |
| 206.5 | 1.11795833 | 206.5 | 7.73744843 | 206.5 | 7.73744843 | 206.5 | 5.79573133 | 206.5 | 15.5337368 | | | 206.50 | 5.78592468 | 206.5 | 10.6892507 | 206.5 | 5.78592468 |
| 206.6 | 1.13757163 | 206.6 | 7.74725508 | 206.6 | 7.74725508 | 206.6 | 5.77611803 | 206.6 | 15.5533501 | | | 206.60 | 5.77611803 | 206.6 | 10.708864 | 206.6 | 5.77611803 |
| 206.7 | 1.13757163 | 206.7 | 7.75706173 | 206.7 | 7.75706173 | 206.7 | 5.75650472 | 206.7 | 15.5239301 | | | 206.70 | 5.78592468 | 206.7 | 10.679444 | 206.7 | 5.78592468 |
| 206.8 | 1.12776498 | 206.8 | 7.76686838 | 206.8 | 7.76686838 | 206.8 | 5.76631138 | 206.8 | 15.5239301 | | | 206.80 | 5.79573133 | 206.8 | 10.6696374 | 206.8 | 5.79573133 |
| 206.9 | 1.13757163 | 206.9 | 7.78648169 | 206.9 | 7.78648169 | 206.9 | 5.79573133 | 206.9 | 15.5239301 | | | 206.90 | 5.78592468 | 206.9 | 10.708864 | 206.9 | 5.78592468 |
| 207 | 1.11795833 | 207 | 7.78648169 | 207 | 7.78648169 | 207 | 5.79573133 | 207 | 15.5533501 | | | 207.00 | 5.78592468 | 207 | 9.98317174 | 207 | 5.78592468 |
| 207.1 | 1.12776498 | 207.1 | 7.79628834 | 207.1 | 7.79628834 | 207.1 | 5.81534464 | 207.1 | 15.5729634 | | | 207.10 | 5.83495794 | 207.1 | 5.20733221 | 207.1 | 5.83495794 |
| 207.2 | 1.12776498 | 207.2 | 7.78648169 | 207.2 | 7.78648169 | 207.2 | 5.83495794 | 207.2 | 15.5729634 | | | 207.20 | 5.80553798 | 207.2 | 1.06892507 | 207.2 | 5.80553798 |
| 207.3 | 1.12776498 | 207.3 | 7.81590164 | 207.3 | 7.81590164 | 207.3 | 5.81534464 | 207.3 | 15.61219 | | | 207.30 | 5.75650472 | 207.3 | 0.07845322 | 207.3 | 5.75650472 |
| 207.4 | 1.15718494 | 207.4 | 7.8257083 | 207.4 | 7.8257083 | 207.4 | 5.83495794 | 207.4 | 15.6318033 | | | 207.40 | 5.79573133 | 207.4 | -0.15690643 | 207.4 | 5.79573133 |
| 207.5 | 1.13757163 | 207.5 | 7.8257083 | 207.5 | 7.8257083 | 207.5 | 5.8643779 | 207.5 | 15.6514166 | | | 207.50 | 5.78592468 | 207.5 | -0.21574634 | 207.5 | 5.78592468 |
| 207.6 | 1.13757163 | 207.6 | 7.8453216 | 207.6 | 7.8453216 | 207.6 | 5.83495794 | 207.6 | 15.6710299 | | | 207.60 | 5.79573133 | 207.6 | -0.2451663 | 207.6 | 5.79573133 |
| 207.7 | 1.15718494 | 207.7 | 7.81590164 | 207.7 | 7.81590164 | 207.7 | 5.82515129 | 207.7 | 15.6710299 | | | 207.70 | 5.82515129 | 207.7 | -0.27458626 | 207.7 | 5.82515129 |
| 207.8 | 1.17679824 | 207.8 | 7.85512825 | 207.8 | 7.85512825 | 207.8 | 5.84476459 | 207.8 | 15.6808365 | | | 207.80 | 5.83495794 | | | 207.8 | 5.83495794 |
| 207.9 | 1.14737828 | 207.9 | 7.8649349 | 207.9 | 7.8649349 | 207.9 | 5.8643779 | 207.9 | 15.6906432 | | | 207.90 | 5.83495794 | | | 207.9 | 5.83495794 |
| 208 | 1.12776498 | 208 | 7.87474156 | 208 | 7.87474156 | 208 | 5.87418455 | 208 | 15.7102565 | | | 208.00 | 5.82515129 | | | 208 | 5.82515129 |
| 208.1 | 1.15718494 | 208.1 | 7.8649349 | 208.1 | 7.8649349 | 208.1 | 5.87418455 | 208.1 | 15.7200632 | | | 208.10 | 5.81534464 | | | 208.1 | 5.81534464 |
| 208.2 | 1.17679824 | 208.2 | 7.88454821 | 208.2 | 7.88454821 | 208.2 | 5.85457124 | 208.2 | 15.7789031 | | | 208.20 | 5.82515129 | | | 208.2 | 5.82515129 |
| 208.3 | 1.12776498 | 208.3 | 7.88454821 | 208.3 | 7.88454821 | 208.3 | 5.87418455 | 208.3 | 15.7985164 | | | 208.30 | 5.80553798 | | | 208.3 | 5.80553798 |
| 208.4 | 1.12776498 | 208.4 | 7.87474156 | 208.4 | 7.87474156 | 208.4 | 5.9036045 | 208.4 | 15.7789031 | | | 208.40 | 5.79573133 | | | 208.4 | 5.79573133 |
| 208.5 | 1.14737828 | 208.5 | 7.88454821 | 208.5 | 7.88454821 | 208.5 | 5.8839912 | 208.5 | 15.7494831 | | | 208.50 | 5.80553798 | | | 208.5 | 5.80553798 |
| 208.6 | 1.16699159 | 208.6 | 7.89435486 | 208.6 | 7.89435486 | 208.6 | 5.9036045 | 208.6 | 15.7789031 | | | 208.60 | 5.83495794 | | | 208.6 | 5.83495794 |
| 208.7 | 1.15718494 | 208.7 | 7.92377482 | 208.7 | 7.92377482 | 208.7 | 5.93302446 | 208.7 | 15.808323 | | | 208.70 | 5.84476459 | | | 208.7 | 5.84476459 |
| 208.8 | 1.15718494 | 208.8 | 7.92377482 | 208.8 | 7.92377482 | 208.8 | 5.94283111 | 208.8 | 15.837743 | | | 208.80 | 5.83495794 | | | 208.8 | 5.83495794 |
| 208.9 | 1.16699159 | 208.9 | 7.94338812 | 208.9 | 7.94338812 | 208.9 | 5.95263776 | 208.9 | 15.8475496 | | | 208.90 | 5.84476459 | | | 208.9 | 5.84476459 |
| 209 | 1.12776498 | 209 | 7.96300142 | 209 | 7.96300142 | 209 | 5.94283111 | 209 | 15.8671629 | | | 209.00 | 5.8643779 | | | 209 | 5.8643779 |
| 209.1 | 1.13757163 | 209.1 | 7.94338812 | 209.1 | 7.94338812 | 209.1 | 5.93302446 | 209.1 | 15.8573563 | | | 209.10 | 5.84476459 | | | 209.1 | 5.84476459 |
| 209.2 | 1.16699159 | 209.2 | 7.96300142 | 209.2 | 7.96300142 | 209.2 | 5.95263776 | 209.2 | 15.8671629 | | | 209.20 | 5.82515129 | | | 209.2 | 5.82515129 |
| 209.3 | 1.2062182 | 209.3 | 7.97280808 | 209.3 | 7.97280808 | 209.3 | 5.93302446 | 209.3 | 15.8867762 | | | 209.30 | 5.83495794 | | | 209.3 | 5.83495794 |
| 209.4 | 1.17679824 | 209.4 | 7.96300142 | 209.4 | 7.96300142 | 209.4 | 5.9036045 | 209.4 | 15.8965829 | | | 209.40 | 5.83495794 | | | 209.4 | 5.83495794 |
| 209.5 | 1.17679824 | 209.5 | 7.96300142 | 209.5 | 7.96300142 | 209.5 | 5.91341116 | 209.5 | 15.8769696 | | | 209.50 | 5.8643779 | | | 209.5 | 5.8643779 |
| 209.6 | 1.16699159 | 209.6 | 7.97280808 | 209.6 | 7.97280808 | 209.6 | 5.95263776 | 209.6 | 15.8965829 | | | 209.60 | 5.8839912 | | | 209.6 | 5.8839912 |
| 209.7 | 1.13757163 | 209.7 | 7.97280808 | 209.7 | 7.97280808 | 209.7 | 5.96244442 | 209.7 | 15.9848428 | | | 209.70 | 5.89379785 | | | 209.7 | 5.89379785 |
| 209.8 | 1.12776498 | 209.8 | 7.99242138 | 209.8 | 7.99242138 | 209.8 | 5.95263776 | 209.8 | 15.9652295 | | | 209.80 | 5.87418455 | | | 209.8 | 5.87418455 |
| 209.9 | 1.14737828 | 209.9 | 7.95319477 | 209.9 | 7.95319477 | 209.9 | 5.95263776 | 209.9 | 15.9848428 | | | 209.90 | 5.89379785 | | | 209.9 | 5.89379785 |
| 210 | 1.14737828 | 210 | 7.96300142 | 210 | 7.96300142 | 210 | 5.93302446 | 210 | 15.9848428 | | | 210.00 | 5.91341116 | | | 210 | 5.91341116 |
| 210.1 | 1.16699159 | 210.1 | 7.98261473 | 210.1 | 7.98261473 | 210.1 | 5.94283111 | 210.1 | 15.9848428 | | | 210.10 | 5.87418455 | | | 210.1 | 5.87418455 |
| 210.2 | 1.18660489 | 210.2 | 8.00222803 | 210.2 | 8.00222803 | 210.2 | 5.96244442 | 210.2 | 15.9848428 | | | 210.20 | 5.89379785 | | | 210.2 | 5.89379785 |
| 210.3 | 1.17679824 | 210.3 | 8.00222803 | 210.3 | 8.00222803 | 210.3 | 5.99186437 | 210.3 | 15.9652295 | | | 210.30 | 5.87418455 | | | 210.3 | 5.87418455 |
| 210.4 | 1.16699159 | 210.4 | 8.03164799 | 210.4 | 8.03164799 | 210.4 | 5.96244442 | 210.4 | 15.9750361 | | | 210.40 | 5.87418455 | | | 210.4 | 5.87418455 |
| 210.5 | 1.18660489 | 210.5 | 8.05126129 | 210.5 | 8.05126129 | 210.5 | 5.98205772 | 210.5 | 16.0240694 | | | 210.50 | 5.89379785 | | | 210.5 | 5.89379785 |
| 210.6 | 1.16699159 | 210.6 | 8.03164799 | 210.6 | 8.03164799 | 210.6 | 6.00167102 | 210.6 | 16.0534893 | | | 210.60 | 5.87418455 | | | 210.6 | 5.87418455 |
| 210.7 | 1.17679824 | 210.7 | 8.04145464 | 210.7 | 8.04145464 | 210.7 | 5.97225107 | 210.7 | 16.0731026 | | | 210.70 | 5.8839912 | | | 210.7 | 5.8839912 |

| | | | | | | | | | | | | | | | | | |
|---|---|---|---|---|---|---|---|---|---|---|---|---|---|---|---|---|---|
| 210.8 | 1.19641154 | 210.8 | 8.03164799 | 210.8 | 8.03164799 | 210.8 | 5.97225107 | 210.8 | 16.0534893 | | | 210.80 | 5.9036045 | | | 210.8 | 5.9036045 |
| 210.9 | 1.21602485 | 210.9 | 8.04145464 | 210.9 | 8.04145464 | 210.9 | 6.01147768 | 210.9 | 16.0534893 | | | 210.90 | 5.92321781 | | | 210.9 | 5.92321781 |
| 211 | 1.23563815 | 211 | 8.0708746 | 211 | 8.0708746 | 211 | 6.02128433 | 211 | 16.0534893 | | | 211.00 | 5.91341116 | | | 211 | 5.91341116 |
| 211.1 | 1.21602485 | 211.1 | 8.0904879 | 211.1 | 8.0904879 | 211.1 | 6.00167102 | 211.1 | 16.0731026 | | | 211.10 | 5.89379785 | | | 211.1 | 5.89379785 |
| 211.2 | 1.18660489 | 211.2 | 8.06106794 | 211.2 | 8.06106794 | 211.2 | 5.96244442 | 211.2 | 16.063296 | | | 211.20 | 5.89379785 | | | 211.2 | 5.89379785 |
| 211.3 | 1.15718494 | 211.3 | 8.0904879 | 211.3 | 8.0904879 | 211.3 | 5.99186437 | 211.3 | 16.063296 | | | 211.30 | 5.9036045 | | | 211.3 | 5.9036045 |
| 211.4 | 1.19641154 | 211.4 | 8.10029455 | 211.4 | 8.10029455 | 211.4 | 6.01147768 | 211.4 | 16.0829093 | | | 211.40 | 5.87418455 | | | 211.4 | 5.87418455 |
| 211.5 | 1.17679824 | 211.5 | 8.0708746 | 211.5 | 8.0708746 | 211.5 | 6.03109098 | 211.5 | 16.1025226 | | | 211.50 | 5.89379785 | | | 211.5 | 5.89379785 |
| 211.6 | 1.17679824 | 211.6 | 8.0904879 | 211.6 | 8.0904879 | 211.6 | 6.02128433 | 211.6 | 16.1221359 | | | 211.60 | 5.94283111 | | | 211.6 | 5.94283111 |
| 211.7 | 1.21602485 | 211.7 | 8.1101012 | 211.7 | 8.1101012 | 211.7 | 6.01147768 | 211.7 | 16.1123292 | | | 211.70 | 5.96244442 | | | 211.7 | 5.96244442 |
| 211.8 | 1.2454448 | 211.8 | 8.1101012 | 211.8 | 8.1101012 | 211.8 | 6.04089763 | 211.8 | 16.1221359 | | | 211.80 | 5.92321781 | | | 211.8 | 5.92321781 |
| 211.9 | 1.19641154 | 211.9 | 8.0904879 | 211.9 | 8.0904879 | 211.9 | 6.07031759 | 211.9 | 16.1515558 | | | 211.90 | 5.91341116 | | | 211.9 | 5.91341116 |
| 212 | 1.19641154 | 212 | 8.08068125 | 212 | 8.08068125 | 212 | 6.07031759 | 212 | 16.1711691 | | | 212.00 | 5.92321781 | | | 212 | 5.92321781 |
| 212.1 | 1.18660489 | 212.1 | 8.1101012 | 212.1 | 8.1101012 | 212.1 | 6.08012424 | 212.1 | 16.2005891 | | | 212.10 | 5.95263776 | | | 212.1 | 5.95263776 |
| 212.2 | 1.17679824 | 212.2 | 8.13952116 | 212.2 | 8.13952116 | 212.2 | 6.07031759 | 212.2 | 16.2005891 | | | 212.20 | 5.92321781 | | | 212.2 | 5.92321781 |
| 212.3 | 1.16699159 | 212.3 | 8.15913446 | 212.3 | 8.15913446 | 212.3 | 6.09973754 | 212.3 | 16.2103958 | | | 212.30 | 5.92321781 | | | 212.3 | 5.92321781 |
| 212.4 | 1.2062182 | 212.4 | 8.14932781 | 212.4 | 8.14932781 | 212.4 | 6.09973754 | 212.4 | 16.2398157 | | | 212.40 | 5.93302446 | | | 212.4 | 5.93302446 |
| 212.5 | 1.21602485 | 212.5 | 8.14932781 | 212.5 | 8.14932781 | 212.5 | 6.07031759 | 212.5 | 16.2398157 | | | 212.50 | 5.98205772 | | | 212.5 | 5.98205772 |
| 212.6 | 1.17679824 | 212.6 | 8.14932781 | 212.6 | 8.14932781 | 212.6 | 6.08993089 | 212.6 | 16.259429 | | | 212.60 | 5.99186437 | | | 212.6 | 5.99186437 |
| 212.7 | 1.18660489 | 212.7 | 8.15913446 | 212.7 | 8.15913446 | 212.7 | 6.08012424 | 212.7 | 16.2496224 | | | 212.70 | 5.95263776 | | | 212.7 | 5.95263776 |
| 212.8 | 1.2062182 | 212.8 | 8.15913446 | 212.8 | 8.15913446 | 212.8 | 6.09973754 | 212.8 | 16.2692357 | | | 212.80 | 5.93302446 | | | 212.8 | 5.93302446 |
| 212.9 | 1.19641154 | 212.9 | 8.15913446 | 212.9 | 8.15913446 | 212.9 | 6.08993089 | 212.9 | 16.2986556 | | | 212.90 | 5.94283111 | | | 212.9 | 5.94283111 |
| 213 | 1.2062182 | 213 | 8.17874777 | 213 | 8.17874777 | 213 | 6.08012424 | 213 | 16.3280756 | | | 213.00 | 5.96244442 | | | 213 | 5.96244442 |
| 213.1 | 1.23563815 | 213.1 | 8.18855442 | 213.1 | 8.18855442 | 213.1 | 6.11935085 | 213.1 | 16.3378822 | | | 213.10 | 5.94283111 | | | 213.1 | 5.94283111 |
| 213.2 | 1.2062182 | 213.2 | 8.17874777 | 213.2 | 8.17874777 | 213.2 | 6.1095442 | 213.2 | 16.3280756 | | | 213.20 | 5.96244442 | | | 213.2 | 5.96244442 |
| 213.3 | 1.21602485 | 213.3 | 8.15913446 | 213.3 | 8.15913446 | 213.3 | 6.11935085 | 213.3 | 16.3378822 | | | 213.30 | 5.99186437 | | | 213.3 | 5.99186437 |
| 213.4 | 1.2062182 | 213.4 | 8.17874777 | 213.4 | 8.17874777 | 213.4 | 6.11935085 | 213.4 | 16.3476889 | | | 213.40 | 5.95263776 | | | 213.4 | 5.95263776 |
| 213.5 | 1.16699159 | 213.5 | 8.17874777 | 213.5 | 8.17874777 | 213.5 | 6.08993089 | 213.5 | 16.3673022 | | | 213.50 | 5.95263776 | | | 213.5 | 5.95263776 |
| 213.6 | 1.2062182 | 213.6 | 8.19836107 | 213.6 | 8.19836107 | 213.6 | 6.1291575 | 213.6 | 16.3771088 | | | 213.60 | 5.97225107 | | | 213.6 | 5.97225107 |
| 213.7 | 1.2258315 | 213.7 | 8.22778103 | 213.7 | 8.22778103 | 213.7 | 6.13896415 | 213.7 | 16.3771088 | | | 213.70 | 5.96244442 | | | 213.7 | 5.96244442 |
| 213.8 | 1.2454448 | 213.8 | 8.23758768 | 213.8 | 8.23758768 | 213.8 | 6.13896415 | 213.8 | 16.3673022 | | | 213.80 | 5.99186437 | | | 213.8 | 5.99186437 |
| 213.9 | 1.2258315 | 213.9 | 8.24739433 | 213.9 | 8.24739433 | 213.9 | 6.1291575 | 213.9 | 16.3574955 | | | 213.90 | 5.98205772 | | | 213.9 | 5.98205772 |
| 214 | 1.19641154 | 214 | 8.23758768 | 214 | 8.23758768 | 214 | 6.1487708 | 214 | 16.3771088 | | | 214.00 | 6.00167102 | | | 214 | 6.00167102 |
| 214.1 | 1.2062182 | 214.1 | 8.21797438 | 214.1 | 8.21797438 | 214.1 | 6.16838411 | 214.1 | 16.3967221 | | | 214.10 | 5.98205772 | | | 214.1 | 5.98205772 |
| 214.2 | 1.21602485 | 214.2 | 8.22778103 | 214.2 | 8.22778103 | 214.2 | 6.15857746 | 214.2 | 16.4163354 | | | 214.20 | 6.03109098 | | | 214.2 | 6.03109098 |
| 214.3 | 1.2258315 | 214.3 | 8.22778103 | 214.3 | 8.22778103 | 214.3 | 6.1487708 | 214.3 | 16.4065288 | | | 214.30 | 6.02128433 | | | 214.3 | 6.02128433 |
| 214.4 | 1.21602485 | 214.4 | 8.24739433 | 214.4 | 8.24739433 | 214.4 | 6.15857746 | 214.4 | 16.4163354 | | | 214.40 | 6.01147768 | | | 214.4 | 6.01147768 |
| 214.5 | 1.21602485 | 214.5 | 8.27681429 | 214.5 | 8.27681429 | 214.5 | 6.18799741 | 214.5 | 16.4457554 | | | 214.50 | 5.99186437 | | | 214.5 | 5.99186437 |
| 214.6 | 1.2258315 | 214.6 | 8.28662094 | 214.6 | 8.28662094 | 214.6 | 6.17819076 | 214.6 | 16.4555621 | | | 214.60 | 6.00167102 | | | 214.6 | 6.00167102 |
| 214.7 | 1.2258315 | 214.7 | 8.27681429 | 214.7 | 8.27681429 | 214.7 | 6.16838411 | 214.7 | 16.4359488 | | | 214.70 | 6.01147768 | | | 214.7 | 6.01147768 |
| 214.8 | 1.2258315 | 214.8 | 8.27681429 | 214.8 | 8.27681429 | 214.8 | 6.15857746 | 214.8 | 16.4653687 | | | 214.80 | 6.00167102 | | | 214.8 | 6.00167102 |
| 214.9 | 1.2062182 | 214.9 | 8.27681429 | 214.9 | 8.27681429 | 214.9 | 6.1487708 | 214.9 | 16.4653687 | | | 214.90 | 6.02128433 | | | 214.9 | 6.02128433 |
| 215 | 1.2258315 | 215 | 8.25720098 | 215 | 8.25720098 | 215 | 6.1487708 | 215 | 16.4751754 | | | 215.00 | 6.03109098 | | | 215 | 6.03109098 |
| 215.1 | 1.25525146 | 215.1 | 8.25720098 | 215.1 | 8.25720098 | 215.1 | 6.15857746 | 215.1 | 16.5045953 | | | 215.10 | 6.02128433 | | | 215.1 | 6.02128433 |
| 215.2 | 1.2454448 | 215.2 | 8.26700764 | 215.2 | 8.26700764 | 215.2 | 6.15857746 | 215.2 | 16.5242086 | | | 215.20 | 6.03109098 | | | 215.2 | 6.03109098 |
| 215.3 | 1.2062182 | 215.3 | 8.28662094 | 215.3 | 8.28662094 | 215.3 | 6.16838411 | 215.3 | 16.5536286 | | | 215.30 | 6.04089763 | | | 215.3 | 6.04089763 |
| 215.4 | 1.21602485 | 215.4 | 8.30623424 | 215.4 | 8.30623424 | 215.4 | 6.21741737 | 215.4 | 16.5536286 | | | 215.40 | 6.01147768 | | | 215.4 | 6.01147768 |
| 215.5 | 1.23563815 | 215.5 | 8.3160409 | 215.5 | 8.3160409 | 215.5 | 6.17819076 | 215.5 | 16.5732419 | | | 215.50 | 6.04089763 | | | 215.5 | 6.04089763 |
| 215.6 | 1.2258315 | 215.6 | 8.27681429 | 215.6 | 8.27681429 | 215.6 | 6.16838411 | 215.6 | 16.5830485 | | | 215.60 | 6.01147768 | | | 215.6 | 6.01147768 |
| 215.7 | 1.21602485 | 215.7 | 8.28662094 | 215.7 | 8.28662094 | 215.7 | 6.18799741 | 215.7 | 16.5928552 | | | 215.70 | 5.98205772 | | | 215.7 | 5.98205772 |
| 215.8 | 1.23563815 | 215.8 | 8.29642759 | 215.8 | 8.29642759 | 215.8 | 6.17819076 | 215.8 | 16.5928552 | | | 215.80 | 5.99186437 | | | 215.8 | 5.99186437 |
| 215.9 | 1.28467141 | 215.9 | 8.32584755 | 215.9 | 8.32584755 | 215.9 | 6.18799741 | 215.9 | 16.6026618 | | | 215.90 | 6.01147768 | | | 215.9 | 6.01147768 |
| 216 | 1.23563815 | 216 | 8.32584755 | 216 | 8.32584755 | 216 | 6.23703067 | 216 | 16.6026618 | | | 216.00 | 6.04089763 | | | 216 | 6.04089763 |
| 216.1 | 1.2454448 | 216.1 | 8.3552675 | 216.1 | 8.3552675 | 216.1 | 6.24683732 | 216.1 | 16.6124685 | | | 216.10 | 6.05070428 | | | 216.1 | 6.05070428 |
| 216.2 | 1.27486476 | 216.2 | 8.3356542 | 216.2 | 8.3356542 | 216.2 | 6.23703067 | 216.2 | 16.6418884 | | | 216.20 | 6.05070428 | | | 216.2 | 6.05070428 |
| 216.3 | 1.25525146 | 216.3 | 8.3356542 | 216.3 | 8.3356542 | 216.3 | 6.23703067 | 216.3 | 16.6713084 | | | 216.30 | 6.05070428 | | | 216.3 | 6.05070428 |
| 216.4 | 1.27486476 | 216.4 | 8.32584755 | 216.4 | 8.32584755 | 216.4 | 6.26645063 | 216.4 | 16.6811151 | | | 216.40 | 6.03109098 | | | 216.4 | 6.03109098 |
| 216.5 | 1.26505811 | 216.5 | 8.34546085 | 216.5 | 8.34546085 | 216.5 | 6.23703067 | 216.5 | 16.6811151 | | | 216.50 | 6.06051094 | | | 216.5 | 6.06051094 |
| 216.6 | 1.26505811 | 216.6 | 8.3552675 | 216.6 | 8.3552675 | 216.6 | 6.23703067 | 216.6 | 16.7007284 | | | 216.60 | 6.04089763 | | | 216.6 | 6.04089763 |
| 216.7 | 1.26505811 | 216.7 | 8.39449411 | 216.7 | 8.39449411 | 216.7 | 6.25664398 | 216.7 | 16.7301483 | | | 216.70 | 6.05070428 | | | 216.7 | 6.05070428 |
| 216.8 | 1.28467141 | 216.8 | 8.38468746 | 216.8 | 8.38468746 | 216.8 | 6.25664398 | 216.8 | 16.7301483 | | | 216.80 | 6.08012424 | | | 216.8 | 6.08012424 |

| | | | | | | | | | | | | | | | | | |
|---|---|---|---|---|---|---|---|---|---|---|---|---|---|---|---|---|---|
| 216.9 | 1.27486476 | 216.9 | 8.38468746 | 216.9 | 8.38468746 | 216.9 | 6.26645063 | 216.9 | 16.7595683 | | | 216.90 | 6.08993089 | | | 216.9 | 6.08993089 |
| 217 | 1.28467141 | 217 | 8.38468746 | 217 | 8.38468746 | 217 | 6.27625728 | 217 | 16.7791816 | | | 217.00 | 6.1095442 | | | 217 | 6.1095442 |
| 217.1 | 1.32389802 | 217.1 | 8.39449411 | 217.1 | 8.39449411 | 217.1 | 6.24683732 | 217.1 | 16.7595683 | | | 217.10 | 6.09973754 | | | 217.1 | 6.09973754 |
| 217.2 | 1.28467141 | 217.2 | 8.39449411 | 217.2 | 8.39449411 | 217.2 | 6.26645063 | 217.2 | 16.7693749 | | | 217.20 | 6.08993089 | | | 217.2 | 6.08993089 |
| 217.3 | 1.26505811 | 217.3 | 8.39449411 | 217.3 | 8.39449411 | 217.3 | 6.27625728 | 217.3 | 16.7791816 | | | 217.30 | 6.1095442 | | | 217.3 | 6.1095442 |
| 217.4 | 1.26505811 | 217.4 | 8.40430076 | 217.4 | 8.40430076 | 217.4 | 6.30567724 | 217.4 | 16.7889882 | | | 217.40 | 6.09973754 | | | 217.4 | 6.09973754 |
| 217.5 | 1.25525146 | 217.5 | 8.39449411 | 217.5 | 8.39449411 | 217.5 | 6.30567724 | 217.5 | 16.8086015 | | | 217.50 | 6.07031759 | | | 217.5 | 6.07031759 |
| 217.6 | 1.25525146 | 217.6 | 8.41410742 | 217.6 | 8.41410742 | 217.6 | 6.26645063 | 217.6 | 16.8086015 | | | 217.60 | 6.08993089 | | | 217.6 | 6.08993089 |
| 217.7 | 1.28467141 | 217.7 | 8.46314068 | 217.7 | 8.46314068 | 217.7 | 6.27625728 | 217.7 | 16.8380215 | | | 217.70 | 6.09973754 | | | 217.7 | 6.09973754 |
| 217.8 | 1.29447806 | 217.8 | 8.47294733 | 217.8 | 8.47294733 | 217.8 | 6.26645063 | 217.8 | 16.8282148 | | | 217.80 | 6.1291575 | | | 217.8 | 6.1291575 |
| 217.9 | 1.29447806 | 217.9 | 8.48275398 | 217.9 | 8.48275398 | 217.9 | 6.25664398 | 217.9 | 16.8086015 | | | 217.90 | 6.11935085 | | | 217.9 | 6.11935085 |
| 218 | 1.29447806 | 218 | 8.49256063 | 218 | 8.49256063 | 218 | 6.22722402 | 218 | 16.8282148 | | | 218.00 | 6.09973754 | | | 218 | 6.09973754 |
| 218.1 | 1.28467141 | 218.1 | 8.48275398 | 218.1 | 8.48275398 | 218.1 | 6.24683732 | 218.1 | 16.8184082 | | | 218.10 | 6.1095442 | | | 218.1 | 6.1095442 |
| 218.2 | 1.30428472 | 218.2 | 8.47294733 | 218.2 | 8.47294733 | 218.2 | 6.23703067 | 218.2 | 16.8478281 | | | 218.20 | 6.07031759 | | | 218.2 | 6.07031759 |
| 218.3 | 1.31409137 | 218.3 | 8.49256063 | 218.3 | 8.49256063 | 218.3 | 6.22722402 | 218.3 | 16.8576348 | | | 218.30 | 6.09973754 | | | 218.3 | 6.09973754 |
| 218.4 | 1.29447806 | 218.4 | 8.50236728 | 218.4 | 8.50236728 | 218.4 | 6.23703067 | 218.4 | 16.8674414 | | | 218.40 | 6.13896415 | | | 218.4 | 6.13896415 |
| 218.5 | 1.30428472 | 218.5 | 8.52198059 | 218.5 | 8.52198059 | 218.5 | 6.24683732 | 218.5 | 16.8674414 | | | 218.50 | 6.1291575 | | | 218.5 | 6.1291575 |
| 218.6 | 1.30428472 | 218.6 | 8.52198059 | 218.6 | 8.52198059 | 218.6 | 6.24683732 | 218.6 | 16.8772481 | | | 218.60 | 6.1291575 | | | 218.6 | 6.1291575 |
| 218.7 | 1.29447806 | 218.7 | 8.51217394 | 218.7 | 8.51217394 | 218.7 | 6.24683732 | 218.7 | 16.9164747 | | | 218.70 | 6.1095442 | | | 218.7 | 6.1095442 |
| 218.8 | 1.30428472 | 218.8 | 8.50236728 | 218.8 | 8.50236728 | 218.8 | 6.25664398 | 218.8 | 16.9458947 | | | 218.80 | 6.11935085 | | | 218.8 | 6.11935085 |
| 218.9 | 1.32389802 | 218.9 | 8.50236728 | 218.9 | 8.50236728 | 218.9 | 6.25664398 | 218.9 | 16.9458947 | | | 218.90 | 6.1291575 | | | 218.9 | 6.1291575 |
| 219 | 1.32389802 | 219 | 8.48275398 | 219 | 8.48275398 | 219 | 6.26645063 | 219 | 16.9851213 | | | 219.00 | 6.1095442 | | | 219 | 6.1095442 |
| 219.1 | 1.30428472 | 219.1 | 8.50236728 | 219.1 | 8.50236728 | 219.1 | 6.24683732 | 219.1 | 16.9949279 | | | 219.10 | 6.1095442 | | | 219.1 | 6.1095442 |
| 219.2 | 1.30428472 | 219.2 | 8.51217394 | 219.2 | 8.51217394 | 219.2 | 6.25664398 | 219.2 | 16.9753146 | | | 219.20 | 6.13896415 | | | 219.2 | 6.13896415 |
| 219.3 | 1.30428472 | 219.3 | 8.54159389 | 219.3 | 8.54159389 | 219.3 | 6.25664398 | 219.3 | 17.0145412 | | | 219.30 | 6.15857746 | | | 219.3 | 6.15857746 |
| 219.4 | 1.28467141 | 219.4 | 8.5612072 | 219.4 | 8.5612072 | 219.4 | 6.23703067 | 219.4 | 17.0341545 | | | 219.40 | 6.1291575 | | | 219.4 | 6.1291575 |
| 219.5 | 1.30428472 | 219.5 | 8.57101385 | 219.5 | 8.57101385 | 219.5 | 6.28606393 | 219.5 | 17.0439612 | | | 219.50 | 6.1291575 | | | 219.5 | 6.1291575 |
| 219.6 | 1.30428472 | 219.6 | 8.5612072 | 219.6 | 8.5612072 | 219.6 | 6.26645063 | 219.6 | 17.0439612 | | | 219.60 | 6.13896415 | | | 219.6 | 6.13896415 |
| 219.7 | 1.30428472 | 219.7 | 8.5612072 | 219.7 | 8.5612072 | 219.7 | 6.25664398 | 219.7 | 17.0635745 | | | 219.70 | 6.13896415 | | | 219.7 | 6.13896415 |
| 219.8 | 1.28467141 | 219.8 | 8.57101385 | 219.8 | 8.57101385 | 219.8 | 6.27625728 | 219.8 | 17.0929944 | | | 219.80 | 6.13896415 | | | 219.8 | 6.13896415 |
| 219.9 | 1.33370467 | 219.9 | 8.57101385 | 219.9 | 8.57101385 | 219.9 | 6.27625728 | 219.9 | 17.0831878 | | | 219.90 | 6.16838411 | | | 219.9 | 6.16838411 |
| 220 | 1.34351132 | 220 | 8.6004338 | 220 | 8.6004338 | 220 | 6.27625728 | 220 | 17.0537678 | | | 220.00 | 6.16838411 | | | 220 | 6.16838411 |
| 220.1 | 1.33370467 | 220.1 | 8.61024046 | 220.1 | 8.61024046 | 220.1 | 6.27625728 | 220.1 | 17.0831878 | | | 220.10 | 6.15857746 | | | 220.1 | 6.15857746 |
| 220.2 | 1.35331798 | 220.2 | 8.6004338 | 220.2 | 8.6004338 | 220.2 | 6.28606393 | 220.2 | 17.1126077 | | | 220.20 | 6.17819076 | | | 220.2 | 6.17819076 |
| 220.3 | 1.36312463 | 220.3 | 8.5808205 | 220.3 | 8.5808205 | 220.3 | 6.28606393 | 220.3 | 17.1028011 | | | 220.30 | 6.16838411 | | | 220.3 | 6.16838411 |
| 220.4 | 1.36312463 | 220.4 | 8.59062715 | 220.4 | 8.59062715 | 220.4 | 6.31548389 | 220.4 | 17.1224144 | | | 220.40 | 6.18799741 | | | 220.4 | 6.18799741 |
| 220.5 | 1.38273793 | 220.5 | 8.57101385 | 220.5 | 8.57101385 | 220.5 | 6.36451715 | 220.5 | 17.1518343 | | | 220.50 | 6.18799741 | | | 220.5 | 6.18799741 |
| 220.6 | 1.36312463 | 220.6 | 8.5808205 | 220.6 | 8.5808205 | 220.6 | 6.3547105 | 220.6 | 17.161641 | | | 220.60 | 6.17819076 | | | 220.6 | 6.17819076 |
| 220.7 | 1.37293128 | 220.7 | 8.6004338 | 220.7 | 8.6004338 | 220.7 | 6.32529054 | 220.7 | 17.1714477 | | | 220.70 | 6.17819076 | | | 220.7 | 6.17819076 |
| 220.8 | 1.36312463 | 220.8 | 8.61024046 | 220.8 | 8.61024046 | 220.8 | 6.34490384 | 220.8 | 17.1812543 | | | 220.80 | 6.16838411 | | | 220.8 | 6.16838411 |
| 220.9 | 1.38273793 | 220.9 | 8.59062715 | 220.9 | 8.59062715 | 220.9 | 6.3547105 | 220.9 | 17.2204809 | | | 220.90 | 6.17819076 | | | 220.9 | 6.17819076 |
| 221 | 1.35331798 | 221 | 8.62004711 | 221 | 8.62004711 | 221 | 6.34490384 | 221 | 17.2302876 | | | 221.00 | 6.16838411 | | | 221 | 6.16838411 |
| 221.1 | 1.35331798 | 221.1 | 8.63966041 | 221.1 | 8.63966041 | 221.1 | 6.33509719 | 221.1 | 17.2302876 | | | 221.10 | 6.1487708 | | | 221.1 | 6.1487708 |
| 221.2 | 1.32389802 | 221.2 | 8.62985376 | 221.2 | 8.62985376 | 221.2 | 6.33509719 | 221.2 | 17.2400942 | | | 221.20 | 6.13896415 | | | 221.2 | 6.13896415 |
| 221.3 | 1.32389802 | 221.3 | 8.63966041 | 221.3 | 8.63966041 | 221.3 | 6.3743238 | 221.3 | 17.2400942 | | | 221.30 | 6.16838411 | | | 221.3 | 6.16838411 |
| 221.4 | 1.33370467 | 221.4 | 8.65927372 | 221.4 | 8.65927372 | 221.4 | 6.3743238 | 221.4 | 17.2793208 | | | 221.40 | 6.17819076 | | | 221.4 | 6.17819076 |
| 221.5 | 1.34351132 | 221.5 | 8.68869367 | 221.5 | 8.68869367 | 221.5 | 6.38413045 | 221.5 | 17.2793208 | | | 221.50 | 6.18799741 | | | 221.5 | 6.18799741 |
| 221.6 | 1.35331798 | 221.6 | 8.67888702 | 221.6 | 8.67888702 | 221.6 | 6.3743238 | 221.6 | 17.2695142 | | | 221.60 | 6.18799741 | | | 221.6 | 6.18799741 |
| 221.7 | 1.38273793 | 221.7 | 8.68869367 | 221.7 | 8.68869367 | 221.7 | 6.36451715 | 221.7 | 17.2695142 | | | 221.70 | 6.18799741 | | | 221.7 | 6.18799741 |
| 221.8 | 1.38273793 | 221.8 | 8.65927372 | 221.8 | 8.65927372 | 221.8 | 6.36451715 | 221.8 | 17.2989341 | | | 221.80 | 6.19780406 | | | 221.8 | 6.19780406 |
| 221.9 | 1.37293128 | 221.9 | 8.66908037 | 221.9 | 8.66908037 | 221.9 | 6.3939371 | 221.9 | 17.3185474 | | | 221.90 | 6.19780406 | | | 221.9 | 6.19780406 |
| 222 | 1.34351132 | 222 | 8.68869367 | 222 | 8.68869367 | 222 | 6.36451715 | 222 | 17.3185474 | | | 222.00 | 6.18799741 | | | 222 | 6.18799741 |
| 222.1 | 1.34351132 | 222.1 | 8.71811363 | 222.1 | 8.71811363 | 222.1 | 6.3743238 | 222.1 | 17.3381607 | | | 222.10 | 6.17819076 | | | 222.1 | 6.17819076 |
| 222.2 | 1.39254458 | 222.2 | 8.71811363 | 222.2 | 8.71811363 | 222.2 | 6.40374376 | 222.2 | 17.3381607 | | | 222.20 | 6.21741737 | | | 222.2 | 6.21741737 |
| 222.3 | 1.40235124 | 222.3 | 8.72792028 | 222.3 | 8.72792028 | 222.3 | 6.3939371 | 222.3 | 17.3479674 | | | 222.30 | 6.24683732 | | | 222.3 | 6.24683732 |
| 222.4 | 1.36312463 | 222.4 | 8.71811363 | 222.4 | 8.71811363 | 222.4 | 6.40374376 | 222.4 | 17.3773873 | | | 222.40 | 6.25664398 | | | 222.4 | 6.25664398 |
| 222.5 | 1.35331798 | 222.5 | 8.71811363 | 222.5 | 8.71811363 | 222.5 | 6.42335706 | 222.5 | 17.3970006 | | | 222.50 | 6.22722402 | | | 222.5 | 6.22722402 |
| 222.6 | 1.36312463 | 222.6 | 8.71811363 | 222.6 | 8.71811363 | 222.6 | 6.40374376 | 222.6 | 17.416614 | | | 222.60 | 6.24683732 | | | 222.6 | 6.24683732 |
| 222.7 | 1.39254458 | 222.7 | 8.72792028 | 222.7 | 8.72792028 | 222.7 | 6.43316371 | 222.7 | 17.4264206 | | | 222.70 | 6.25664398 | | | 222.7 | 6.25664398 |
| 222.8 | 1.38273793 | 222.8 | 8.73772693 | 222.8 | 8.73772693 | 222.8 | 6.44297036 | 222.8 | 17.416614 | | | 222.80 | 6.22722402 | | | 222.8 | 6.22722402 |
| 222.9 | 1.39254458 | 222.9 | 8.69850032 | 222.9 | 8.69850032 | 222.9 | 6.47239032 | 222.9 | 17.4362273 | | | 222.90 | 6.22722402 | | | 222.9 | 6.22722402 |

| | | | | | | | | | | | | | | | | | |
|---|---|---|---|---|---|---|---|---|---|---|---|---|---|---|---|---|---|
| 223 | 1.36312463 | 223 | 8.72792028 | 223 | 8.72792028 | 223 | 6.47239032 | 223 | 17.4264206 | | | 223.00 | 6.20761072 | | | 223 | 6.20761072 |
| 223.1 | 1.34351132 | 223.1 | 8.73772693 | 223.1 | 8.73772693 | 223.1 | 6.46258367 | 223.1 | 17.416614 | | | 223.10 | 6.24683732 | | | 223.1 | 6.24683732 |
| 223.2 | 1.38273793 | 223.2 | 8.77695354 | 223.2 | 8.77695354 | 223.2 | 6.46258367 | 223.2 | 17.4362273 | | | 223.20 | 6.26645063 | | | 223.2 | 6.26645063 |
| 223.3 | 1.40235124 | 223.3 | 8.76714689 | 223.3 | 8.76714689 | 223.3 | 6.49200362 | 223.3 | 17.4754539 | | | 223.30 | 6.26645063 | | | 223.3 | 6.26645063 |
| 223.4 | 1.37293128 | 223.4 | 8.75734024 | 223.4 | 8.75734024 | 223.4 | 6.49200362 | 223.4 | 17.4656472 | | | 223.40 | 6.22722402 | | | 223.4 | 6.22722402 |
| 223.5 | 1.38273793 | 223.5 | 8.77695354 | 223.5 | 8.77695354 | 223.5 | 6.49200362 | 223.5 | 17.4656472 | | | 223.50 | 6.24683732 | | | 223.5 | 6.24683732 |
| 223.6 | 1.42196454 | 223.6 | 8.77695354 | 223.6 | 8.77695354 | 223.6 | 6.52142358 | 223.6 | 17.4950672 | | | 223.60 | 6.23703067 | | | 223.6 | 6.23703067 |
| 223.7 | 1.41215789 | 223.7 | 8.76714689 | 223.7 | 8.76714689 | 223.7 | 6.53123023 | 223.7 | 17.5342938 | | | 223.70 | 6.26645063 | | | 223.7 | 6.26645063 |
| 223.8 | 1.42196454 | 223.8 | 8.78676019 | 223.8 | 8.78676019 | 223.8 | 6.50181028 | 223.8 | 17.5244871 | | | 223.80 | 6.26645063 | | | 223.8 | 6.26645063 |
| 223.9 | 1.41215789 | 223.9 | 8.78676019 | 223.9 | 8.78676019 | 223.9 | 6.52142358 | 223.9 | 17.5146805 | | | 223.90 | 6.26645063 | | | 223.9 | 6.26645063 |
| 224 | 1.42196454 | 224 | 8.79656684 | 224 | 8.79656684 | 224 | 6.50181028 | 224 | 17.5441004 | | | 224.00 | 6.23703067 | | | 224 | 6.23703067 |
| 224.1 | 1.42196454 | 224.1 | 8.79656684 | 224.1 | 8.79656684 | 224.1 | 6.51161693 | 224.1 | 17.5637137 | | | 224.10 | 6.24683732 | | | 224.1 | 6.24683732 |
| 224.2 | 1.43177119 | 224.2 | 8.79656684 | 224.2 | 8.79656684 | 224.2 | 6.51161693 | 224.2 | 17.583327 | | | 224.20 | 6.24683732 | | | 224.2 | 6.24683732 |
| 224.3 | 1.42196454 | 224.3 | 8.81618015 | 224.3 | 8.81618015 | 224.3 | 6.50181028 | 224.3 | 17.5931337 | | | 224.30 | 6.26645063 | | | 224.3 | 6.26645063 |
| 224.4 | 1.43177119 | 224.4 | 8.8063735 | 224.4 | 8.8063735 | 224.4 | 6.48219697 | 224.4 | 17.612747 | | | 224.40 | 6.26645063 | | | 224.4 | 6.26645063 |
| 224.5 | 1.40235124 | 224.5 | 8.81618015 | 224.5 | 8.81618015 | 224.5 | 6.50181028 | 224.5 | 17.6225536 | | | 224.50 | 6.27625728 | | | 224.5 | 6.27625728 |
| 224.6 | 1.42196454 | 224.6 | 8.85540676 | 224.6 | 8.85540676 | 224.6 | 6.53123023 | 224.6 | 17.6323603 | | | 224.60 | 6.30567724 | | | 224.6 | 6.30567724 |
| 224.7 | 1.43177119 | 224.7 | 8.8259868 | 224.7 | 8.8259868 | 224.7 | 6.53123023 | 224.7 | 17.6519736 | | | 224.70 | 6.31548389 | | | 224.7 | 6.31548389 |
| 224.8 | 1.38273793 | 224.8 | 8.8063735 | 224.8 | 8.8063735 | 224.8 | 6.56065019 | 224.8 | 17.6617803 | | | 224.80 | 6.30567724 | | | 224.8 | 6.30567724 |
| 224.9 | 1.42196454 | 224.9 | 8.8063735 | 224.9 | 8.8063735 | 224.9 | 6.55084354 | 224.9 | 17.6813936 | | | 224.90 | 6.30567724 | | | 224.9 | 6.30567724 |
| 225 | 1.4709978 | 225 | 8.8456001 | 225 | 8.8456001 | 225 | 6.55084354 | 225 | 17.6912002 | | | 225.00 | 6.31548389 | | | 225 | 6.31548389 |
| 225.1 | 1.42196454 | 225.1 | 8.87502006 | 225.1 | 8.87502006 | 225.1 | 6.56065019 | 225.1 | 17.7206202 | | | 225.10 | 6.30567724 | | | 225.1 | 6.30567724 |
| 225.2 | 1.43177119 | 225.2 | 8.86521341 | 225.2 | 8.86521341 | 225.2 | 6.59007014 | 225.2 | 17.7304268 | | | 225.20 | 6.29587058 | | | 225.2 | 6.29587058 |
| 225.3 | 1.4513845 | 225.3 | 8.8456001 | 225.3 | 8.8456001 | 225.3 | 6.56065019 | 225.3 | 17.7304268 | | | 225.30 | 6.33509719 | | | 225.3 | 6.33509719 |
| 225.4 | 1.4513845 | 225.4 | 8.85540676 | 225.4 | 8.85540676 | 225.4 | 6.55084354 | 225.4 | 17.7500401 | | | 225.40 | 6.31548389 | | | 225.4 | 6.31548389 |
| 225.5 | 1.46119115 | 225.5 | 8.86521341 | 225.5 | 8.86521341 | 225.5 | 6.57045684 | 225.5 | 17.7696534 | | | 225.50 | 6.31548389 | | | 225.5 | 6.31548389 |
| 225.6 | 1.4709978 | 225.6 | 8.85540676 | 225.6 | 8.85540676 | 225.6 | 6.56065019 | 225.6 | 17.7598468 | | | 225.60 | 6.33509719 | | | 225.6 | 6.33509719 |
| 225.7 | 1.46119115 | 225.7 | 8.85540676 | 225.7 | 8.85540676 | 225.7 | 6.56065019 | 225.7 | 17.7598468 | | | 225.70 | 6.33509719 | | | 225.7 | 6.33509719 |
| 225.8 | 1.4513845 | 225.8 | 8.86521341 | 225.8 | 8.86521341 | 225.8 | 6.54103688 | 225.8 | 17.7794601 | | | 225.80 | 6.3547105 | | | 225.8 | 6.3547105 |
| 225.9 | 1.4513845 | 225.9 | 8.88482671 | 225.9 | 8.88482671 | 225.9 | 6.55084354 | 225.9 | 17.7892667 | | | 225.90 | 6.33509719 | | | 225.9 | 6.33509719 |
| 226 | 1.46119115 | 226 | 8.87502006 | 226 | 8.87502006 | 226 | 6.56065019 | 226 | 17.8284933 | | | 226.00 | 6.32529054 | | | 226 | 6.32529054 |
| 226.1 | 1.4709978 | 226.1 | 8.90444002 | 226.1 | 8.90444002 | 226.1 | 6.59007014 | 226.1 | 17.8383 | | | 226.10 | 6.31548389 | | | 226.1 | 6.31548389 |
| 226.2 | 1.4513845 | 226.2 | 8.92405332 | 226.2 | 8.92405332 | 226.2 | 6.56065019 | 226.2 | 17.8579133 | | | 226.20 | 6.34490384 | | | 226.2 | 6.34490384 |
| 226.3 | 1.43177119 | 226.3 | 8.93385997 | 226.3 | 8.93385997 | 226.3 | 6.55084354 | 226.3 | 17.8677199 | | | 226.30 | 6.33509719 | | | 226.3 | 6.33509719 |
| 226.4 | 1.4513845 | 226.4 | 8.93385997 | 226.4 | 8.93385997 | 226.4 | 6.58026349 | 226.4 | 17.8579133 | | | 226.40 | 6.30567724 | | | 226.4 | 6.30567724 |
| 226.5 | 1.4709978 | 226.5 | 8.98289323 | 226.5 | 8.98289323 | 226.5 | 6.58026349 | 226.5 | 17.8873332 | | | 226.50 | 6.30567724 | | | 226.5 | 6.30567724 |
| 226.6 | 1.50041776 | 226.6 | 8.97308658 | 226.6 | 8.97308658 | 226.6 | 6.59007014 | 226.6 | 17.8873332 | | | 226.60 | 6.30567724 | | | 226.6 | 6.30567724 |
| 226.7 | 1.48080445 | 226.7 | 8.95347328 | 226.7 | 8.95347328 | 226.7 | 6.58026349 | 226.7 | 17.8873332 | | | 226.70 | 6.32529054 | | | 226.7 | 6.32529054 |
| 226.8 | 1.50041776 | 226.8 | 8.94366662 | 226.8 | 8.94366662 | 226.8 | 6.56065019 | 226.8 | 17.9167532 | | | 226.80 | 6.3743238 | | | 226.8 | 6.3743238 |
| 226.9 | 1.52003106 | 226.9 | 8.98289323 | 226.9 | 8.98289323 | 226.9 | 6.5998768 | 226.9 | 17.9069466 | | | 226.90 | 6.34490384 | | | 226.9 | 6.34490384 |
| 227 | 1.4709978 | 227 | 9.02211984 | 227 | 9.02211984 | 227 | 6.6194901 | 227 | 17.9069466 | | | 227.00 | 6.34490384 | | | 227 | 6.34490384 |
| 227.1 | 1.4906111 | 227.1 | 8.97308658 | 227.1 | 8.97308658 | 227.1 | 6.6194901 | 227.1 | 17.9461732 | | | 227.10 | 6.33509719 | | | 227.1 | 6.33509719 |
| 227.2 | 1.48080445 | 227.2 | 8.96327993 | 227.2 | 8.96327993 | 227.2 | 6.6194901 | 227.2 | 17.9657865 | | | 227.20 | 6.3547105 | | | 227.2 | 6.3547105 |
| 227.3 | 1.4513845 | 227.3 | 8.99269988 | 227.3 | 8.99269988 | 227.3 | 6.6391034 | 227.3 | 17.9657865 | | | 227.30 | 6.36451715 | | | 227.3 | 6.36451715 |
| 227.4 | 1.48080445 | 227.4 | 9.00250654 | 227.4 | 9.00250654 | 227.4 | 6.6194901 | 227.4 | 17.9657865 | | | 227.40 | 6.33509719 | | | 227.4 | 6.33509719 |
| 227.5 | 1.48080445 | 227.5 | 8.98289323 | 227.5 | 8.98289323 | 227.5 | 6.62929675 | 227.5 | 17.9755931 | | | 227.50 | 6.3547105 | | | 227.5 | 6.3547105 |
| 227.6 | 1.52983771 | 227.6 | 8.98289323 | 227.6 | 8.98289323 | 227.6 | 6.6391034 | 227.6 | 17.9853998 | | | 227.60 | 6.38413045 | | | 227.6 | 6.38413045 |
| 227.7 | 1.52983771 | 227.7 | 8.98289323 | 227.7 | 8.98289323 | 227.7 | 6.66852336 | 227.7 | 17.9952064 | | | 227.70 | 6.38413045 | | | 227.7 | 6.38413045 |
| 227.8 | 1.4906111 | 227.8 | 8.96327993 | 227.8 | 8.96327993 | 227.8 | 6.67833001 | 227.8 | 18.0148197 | | | 227.80 | 6.38413045 | | | 227.8 | 6.38413045 |
| 227.9 | 1.51022441 | 227.9 | 8.97308658 | 227.9 | 8.97308658 | 227.9 | 6.6391034 | 227.9 | 18.0050131 | | | 227.90 | 6.3939371 | | | 227.9 | 6.3939371 |
| 228 | 1.53964436 | 228 | 9.03192649 | 228 | 9.03192649 | 228 | 6.62929675 | 228 | 18.0050131 | | | 228.00 | 6.41355041 | | | 228 | 6.41355041 |
| 228.1 | 1.52003106 | 228.1 | 9.04173314 | 228.1 | 9.04173314 | 228.1 | 6.64891006 | 228.1 | 18.0050131 | | | 228.10 | 6.41355041 | | | 228.1 | 6.41355041 |
| 228.2 | 1.55925767 | 228.2 | 9.03192649 | 228.2 | 9.03192649 | 228.2 | 6.64891006 | 228.2 | 18.034433 | | | 228.20 | 6.41355041 | | | 228.2 | 6.41355041 |
| 228.3 | 1.54945102 | 228.3 | 9.00250654 | 228.3 | 9.00250654 | 228.3 | 6.60968345 | 228.3 | -18.2011461 | | | 228.30 | 6.3939371 | | | 228.3 | 6.3939371 |
| 228.4 | 1.54945102 | 228.4 | 9.01231319 | 228.4 | 9.01231319 | 228.4 | 6.6194901 | 228.4 | 2.88315569 | | | 228.40 | 6.38413045 | | | 228.4 | 6.38413045 |
| 228.5 | 1.57887097 | 228.5 | 9.0515398 | 228.5 | 9.0515398 | 228.5 | 6.62929675 | 228.5 | 18.0932729 | | | 228.50 | 6.40374376 | | | 228.5 | 6.40374376 |
| 228.6 | 1.59848428 | 228.6 | 9.04173314 | 228.6 | 9.04173314 | 228.6 | 6.6391034 | 228.6 | 18.1226929 | | | 228.60 | 6.40374376 | | | 228.6 | 6.40374376 |
| 228.7 | 1.57887097 | 228.7 | 9.03192649 | 228.7 | 9.03192649 | 228.7 | 6.64891006 | 228.7 | 18.1521129 | | | 228.70 | 6.38413045 | | | 228.7 | 6.38413045 |
| 228.8 | 1.56906432 | 228.8 | 9.04173314 | 228.8 | 9.04173314 | 228.8 | 6.64891006 | 228.8 | 18.1324995 | | | 228.80 | 6.38413045 | | | 228.8 | 6.38413045 |
| 228.9 | 1.59848428 | 228.9 | 9.06134645 | 228.9 | 9.06134645 | 228.9 | 6.64891006 | 228.9 | 18.1423062 | | | 228.90 | 6.3939371 | | | 228.9 | 6.3939371 |
| 229 | 1.60829093 | 229 | 9.0907664 | 229 | 9.0907664 | 229 | 6.65871671 | 229 | 18.1423062 | | | 229.00 | 6.3939371 | | | 229 | 6.3939371 |

| | | | | | | | | | | | | | | | | | |
|---|---|---|---|---|---|---|---|---|---|---|---|---|---|---|---|---|---|
| 229.1 | 1.59848428 | 229.1 | 9.0711531 | 229.1 | 9.0711531 | 229.1 | 6.67833001 | 229.1 | 18.1717262 | | | 229.10 | 6.3939371 | | | 229.1 | 6.3939371 |
| 229.2 | 1.58867762 | 229.2 | 9.06134645 | 229.2 | 9.06134645 | 229.2 | 6.67833001 | 229.2 | 18.1717262 | | | 229.20 | 6.41355041 | | | 229.2 | 6.41355041 |
| 229.3 | 1.61809758 | 229.3 | 9.0711531 | 229.3 | 9.0711531 | 229.3 | 6.67833001 | 229.3 | 18.1521129 | | | 229.30 | 6.42335706 | | | 229.3 | 6.42335706 |
| 229.4 | 1.59848428 | 229.4 | 9.0711531 | 229.4 | 9.0711531 | 229.4 | 6.68813666 | 229.4 | 18.1717262 | | | 229.40 | 6.41355041 | | | 229.4 | 6.41355041 |
| 229.5 | 1.62790423 | 229.5 | 9.08095975 | 229.5 | 9.08095975 | 229.5 | 6.70774997 | 229.5 | 18.2109528 | | | 229.50 | 6.3939371 | | | 229.5 | 6.3939371 |
| 229.6 | 1.64751754 | 229.6 | 9.10057306 | 229.6 | 9.10057306 | 229.6 | 6.69794332 | 229.6 | 18.2403727 | | | 229.60 | 6.40374376 | | | 229.6 | 6.40374376 |
| 229.7 | 1.59848428 | 229.7 | 9.10057306 | 229.7 | 9.10057306 | 229.7 | 6.67833001 | 229.7 | 18.259986 | | | 229.70 | 6.43316371 | | | 229.7 | 6.43316371 |
| 229.8 | 1.63771088 | 229.8 | 9.11037971 | 229.8 | 9.11037971 | 229.8 | 6.71755662 | 229.8 | 18.259986 | | | 229.80 | 6.42335706 | | | 229.8 | 6.42335706 |
| 229.9 | 1.62790423 | 229.9 | 9.13979966 | 229.9 | 9.13979966 | 229.9 | 6.74697658 | 229.9 | 18.2501794 | | | 229.90 | 6.3939371 | | | 229.9 | 6.3939371 |
| 230 | 1.62790423 | 230 | 9.10057306 | 230 | 9.10057306 | 230 | 6.71755662 | 230 | 18.2501794 | | | 230.00 | 6.44297036 | | | 230 | 6.44297036 |
| 230.1 | 1.61809758 | 230.1 | 9.0907664 | 230.1 | 9.0907664 | 230.1 | 6.72736327 | 230.1 | 18.2403727 | | | 230.10 | 6.44297036 | | | 230.1 | 6.44297036 |
| 230.2 | 1.59848428 | 230.2 | 9.10057306 | 230.2 | 9.10057306 | 230.2 | 6.70774997 | 230.2 | 18.2403727 | | | 230.20 | 6.41355041 | | | 230.2 | 6.41355041 |
| 230.3 | 1.59848428 | 230.3 | 9.12999301 | 230.3 | 9.12999301 | 230.3 | 6.71755662 | 230.3 | 18.259986 | | | 230.30 | 6.40374376 | | | 230.3 | 6.40374376 |
| 230.4 | 1.60829093 | 230.4 | 9.13979966 | 230.4 | 9.13979966 | 230.4 | 6.72736327 | 230.4 | 18.2992126 | | | 230.40 | 6.3939371 | | | 230.4 | 6.3939371 |
| 230.5 | 1.60829093 | 230.5 | 9.12999301 | 230.5 | 9.12999301 | 230.5 | 6.72736327 | 230.5 | 18.3482459 | | | 230.50 | 6.43316371 | | | 230.5 | 6.43316371 |
| 230.6 | 1.65732419 | 230.6 | 9.15941297 | 230.6 | 9.15941297 | 230.6 | 6.72736327 | 230.6 | 18.3580525 | | | 230.60 | 6.44297036 | | | 230.6 | 6.44297036 |
| 230.7 | 1.63771088 | 230.7 | 9.13979966 | 230.7 | 9.13979966 | 230.7 | 6.73716992 | 230.7 | 18.3580525 | | | 230.70 | 6.44297036 | | | 230.7 | 6.44297036 |
| 230.8 | 1.67693749 | 230.8 | 9.17902627 | 230.8 | 9.17902627 | 230.8 | 6.73716992 | 230.8 | 18.3286326 | | | 230.80 | 6.46258367 | | | 230.8 | 6.46258367 |
| 230.9 | 1.66713084 | 230.9 | 9.18883292 | 230.9 | 9.18883292 | 230.9 | 6.75678323 | 230.9 | 18.3580525 | | | 230.90 | 6.43316371 | | | 230.9 | 6.43316371 |
| 231 | 1.64751754 | 231 | 9.17902627 | 231 | 9.17902627 | 231 | 6.72736327 | 231 | 18.3678592 | | | 231.00 | 6.42335706 | | | 231 | 6.42335706 |
| 231.1 | 1.63771088 | 231.1 | 9.16921962 | 231.1 | 9.16921962 | 231.1 | 6.73716992 | 231.1 | 18.3482459 | | | 231.10 | 6.45277702 | | | 231.1 | 6.45277702 |
| 231.2 | 1.64751754 | 231.2 | 9.18883292 | 231.2 | 9.18883292 | 231.2 | 6.72736327 | 231.2 | 18.3090193 | | | 231.20 | 6.47239032 | | | 231.2 | 6.47239032 |
| 231.3 | 1.64751754 | 231.3 | 9.18883292 | 231.3 | 9.18883292 | 231.3 | 6.74697658 | 231.3 | 18.2305661 | | | 231.30 | 6.46258367 | | | 231.3 | 6.46258367 |
| 231.4 | 1.64751754 | 231.4 | 9.20844623 | 231.4 | 9.20844623 | 231.4 | 6.73716992 | 231.4 | 18.1815328 | | | 231.40 | 6.47239032 | | | 231.4 | 6.47239032 |
| 231.5 | 1.58867762 | 231.5 | 9.19863958 | 231.5 | 9.19863958 | 231.5 | 6.75678323 | 231.5 | 18.0442397 | | | 231.50 | 6.46258367 | | | 231.5 | 6.46258367 |
| 231.6 | 1.61809758 | 231.6 | 9.20844623 | 231.6 | 9.20844623 | 231.6 | 6.75678323 | 231.6 | 18.0148197 | | | 231.60 | 6.45277702 | | | 231.6 | 6.45277702 |
| 231.7 | 1.59848428 | 231.7 | 9.22805953 | 231.7 | 9.22805953 | 231.7 | 6.76658988 | 231.7 | 18.034433 | | | 231.70 | 6.44297036 | | | 231.7 | 6.44297036 |
| 231.8 | 1.60829093 | 231.8 | 9.22805953 | 231.8 | 9.22805953 | 231.8 | 6.79600984 | 231.8 | 18.034433 | | | 231.80 | 6.46258367 | | | 231.8 | 6.46258367 |
| 231.9 | 1.61809758 | 231.9 | 9.20844623 | 231.9 | 9.20844623 | 231.9 | 6.81562314 | 231.9 | 18.0932729 | | | 231.90 | 6.48219697 | | | 231.9 | 6.48219697 |
| 232 | 1.61809758 | 232 | 9.20844623 | 232 | 9.20844623 | 232 | 6.77639653 | 232 | 18.0834663 | | | 232.00 | 6.46258367 | | | 232 | 6.46258367 |
| 232.1 | 1.65732419 | 232.1 | 9.21825288 | 232.1 | 9.21825288 | 232.1 | 6.77639653 | 232.1 | 18.0736596 | | | 232.10 | 6.43316371 | | | 232.1 | 6.43316371 |
| 232.2 | 1.67693749 | 232.2 | 9.22805953 | 232.2 | 9.22805953 | 232.2 | 6.75678323 | 232.2 | 18.1030796 | | | 232.20 | 6.46258367 | | | 232.2 | 6.46258367 |
| 232.3 | 1.65732419 | 232.3 | 9.23786618 | 232.3 | 9.23786618 | 232.3 | 6.77639653 | 232.3 | 18.0932729 | | | 232.30 | 6.45277702 | | | 232.3 | 6.45277702 |
| 232.4 | 1.63771088 | 232.4 | 9.21825288 | 232.4 | 9.21825288 | 232.4 | 6.78620318 | 232.4 | 18.1030796 | | | 232.40 | 6.45277702 | | | 232.4 | 6.45277702 |
| 232.5 | 1.63771088 | 232.5 | 9.23786618 | 232.5 | 9.23786618 | 232.5 | 6.77639653 | 232.5 | 18.1423062 | | | 232.50 | 6.48219697 | | | 232.5 | 6.48219697 |
| 232.6 | 1.64751754 | 232.6 | 9.24767284 | 232.6 | 9.24767284 | 232.6 | 6.75678323 | 232.6 | 18.1717262 | | | 232.60 | 6.49200362 | | | 232.6 | 6.49200362 |
| 232.7 | 1.60829093 | 232.7 | 9.21825288 | 232.7 | 9.21825288 | 232.7 | 6.78620318 | 232.7 | 18.1521129 | | | 232.70 | 6.48219697 | | | 232.7 | 6.48219697 |
| 232.8 | 1.64751754 | 232.8 | 9.22805953 | 232.8 | 9.22805953 | 232.8 | 6.78620318 | 232.8 | 18.1717262 | | | 232.80 | 6.46258367 | | | 232.8 | 6.46258367 |
| 232.9 | 1.67693749 | 232.9 | 9.21825288 | 232.9 | 9.21825288 | 232.9 | 6.76658988 | 232.9 | 18.2011461 | | | 232.90 | 6.51161693 | | | 232.9 | 6.51161693 |
| 233 | 1.65732419 | 233 | 9.22805953 | 233 | 9.22805953 | 233 | 6.79600984 | 233 | 18.2011461 | | | 233.00 | 6.51161693 | | | 233 | 6.51161693 |
| 233.1 | 1.67693749 | 233.1 | 9.23786618 | 233.1 | 9.23786618 | 233.1 | 6.77639653 | 233.1 | 18.1815328 | | | 233.10 | 6.52142358 | | | 233.1 | 6.52142358 |
| 233.2 | 1.67693749 | 233.2 | 9.25747949 | 233.2 | 9.25747949 | 233.2 | 6.77639653 | 233.2 | 18.1815328 | | | 233.20 | 6.49200362 | | | 233.2 | 6.49200362 |
| 233.3 | 1.64751754 | 233.3 | 9.27709279 | 233.3 | 9.27709279 | 233.3 | 6.79600984 | 233.3 | 18.1815328 | | | 233.30 | 6.49200362 | | | 233.3 | 6.49200362 |
| 233.4 | 1.65732419 | 233.4 | 9.27709279 | 233.4 | 9.27709279 | 233.4 | 6.81562314 | 233.4 | 18.1717262 | | | 233.40 | 6.50181028 | | | 233.4 | 6.50181028 |
| 233.5 | 1.65732419 | 233.5 | 9.23786618 | 233.5 | 9.23786618 | 233.5 | 6.81562314 | 233.5 | 18.1717262 | | | 233.50 | 6.49200362 | | | 233.5 | 6.49200362 |
| 233.6 | 1.63771088 | 233.6 | 9.21825288 | 233.6 | 9.21825288 | 233.6 | 6.79600984 | 233.6 | 18.1815328 | | | 233.60 | 6.50181028 | | | 233.6 | 6.50181028 |
| 233.7 | 1.63771088 | 233.7 | 9.23786618 | 233.7 | 9.23786618 | 233.7 | 6.78620318 | 233.7 | 18.2011461 | | | 233.70 | 6.53123023 | | | 233.7 | 6.53123023 |
| 233.8 | 1.65732419 | 233.8 | 9.24767284 | 233.8 | 9.24767284 | 233.8 | 6.78620318 | 233.8 | 18.2207594 | | | 233.80 | 6.52142358 | | | 233.8 | 6.52142358 |
| 233.9 | 1.68674414 | 233.9 | 9.27709279 | 233.9 | 9.27709279 | 233.9 | 6.81562314 | 233.9 | 18.2697927 | | | 233.90 | 6.50181028 | | | 233.9 | 6.50181028 |
| 234 | 1.6965508 | 234 | 9.25747949 | 234 | 9.25747949 | 234 | 6.83523644 | 234 | 18.289406 | | | 234.00 | 6.49200362 | | | 234 | 6.49200362 |
| 234.1 | 1.68674414 | 234.1 | 9.28689944 | 234.1 | 9.28689944 | 234.1 | 6.82542979 | 234.1 | 18.3188259 | | | 234.10 | 6.52142358 | | | 234.1 | 6.52142358 |
| 234.2 | 1.6965508 | 234.2 | 9.28689944 | 234.2 | 9.28689944 | 234.2 | 6.8450431 | 234.2 | 18.3090193 | | | 234.20 | 6.53123023 | | | 234.2 | 6.53123023 |
| 234.3 | 1.6965508 | 234.3 | 9.26728614 | 234.3 | 9.26728614 | 234.3 | 6.85484975 | 234.3 | 18.3286326 | | | 234.30 | 6.51161693 | | | 234.3 | 6.51161693 |
| 234.4 | 1.70635745 | 234.4 | 9.2967061 | 234.4 | 9.2967061 | 234.4 | 6.8450431 | 234.4 | 18.3482459 | | | 234.40 | 6.54103688 | | | 234.4 | 6.54103688 |
| 234.5 | 1.70635745 | 234.5 | 9.28689944 | 234.5 | 9.28689944 | 234.5 | 6.83523644 | 234.5 | 18.3580525 | | | 234.50 | 6.53123023 | | | 234.5 | 6.53123023 |
| 234.6 | 1.6965508 | 234.6 | 9.26728614 | 234.6 | 9.26728614 | 234.6 | 6.8450431 | 234.6 | 18.3776658 | | | 234.60 | 6.52142358 | | | 234.6 | 6.52142358 |
| 234.7 | 1.7161641 | 234.7 | 9.26728614 | 234.7 | 9.26728614 | 234.7 | 6.8646564 | 234.7 | 18.3874725 | | | 234.70 | 6.54103688 | | | 234.7 | 6.54103688 |
| 234.8 | 1.6965508 | 234.8 | 9.26728614 | 234.8 | 9.26728614 | 234.8 | 6.8842697 | 234.8 | 18.4070858 | | | 234.80 | 6.54103688 | | | 234.8 | 6.54103688 |
| 234.9 | 1.70635745 | 234.9 | 9.24767284 | 234.9 | 9.24767284 | 234.9 | 6.8450431 | 234.9 | 18.4365058 | | | 234.90 | 6.53123023 | | | 234.9 | 6.53123023 |
| 235 | 1.6965508 | 235 | 9.23786618 | 235 | 9.23786618 | 235 | 6.82542979 | 235 | 18.4561191 | | | 235.00 | 6.52142358 | | | 235 | 6.52142358 |
| 235.1 | 1.68674414 | 235.1 | 9.23786618 | 235.1 | 9.23786618 | 235.1 | 6.8450431 | 235.1 | 18.4463124 | | | 235.10 | 6.54103688 | | | 235.1 | 6.54103688 |

| | | | | | | | | | | | | | | | | | |
|---|---|---|---|---|---|---|---|---|---|---|---|---|---|---|---|---|---|
| 235.2 | 1.68674414 | 235.2 | 9.27709279 | 235.2 | 9.27709279 | 235.2 | 6.83523644 | 235.2 | 18.4757324 | | | 235.20 | 6.54103688 | | | 235.2 | 6.54103688 |
| 235.3 | 1.68674414 | 235.3 | 9.23786618 | 235.3 | 9.23786618 | 235.3 | 6.82542979 | 235.3 | 18.4659257 | | | 235.30 | 6.54103688 | | | 235.3 | 6.54103688 |
| 235.4 | 1.6965508 | 235.4 | 9.24767284 | 235.4 | 9.24767284 | 235.4 | 6.82542979 | 235.4 | 18.4953457 | | | 235.40 | 6.57045684 | | | 235.4 | 6.57045684 |
| 235.5 | 1.70635745 | 235.5 | 9.26728614 | 235.5 | 9.26728614 | 235.5 | 6.85484975 | 235.5 | 18.5247656 | | | 235.50 | 6.53123023 | | | 235.5 | 6.53123023 |
| 235.6 | 1.74558406 | 235.6 | 9.23786618 | 235.6 | 9.23786618 | 235.6 | 6.87446305 | 235.6 | 18.5247656 | | | 235.60 | 6.53123023 | | | 235.6 | 6.53123023 |
| 235.7 | 1.7161641 | 235.7 | 9.24767284 | 235.7 | 9.24767284 | 235.7 | 6.85484975 | 235.7 | 18.5443789 | | | 235.70 | 6.53123023 | | | 235.7 | 6.53123023 |
| 235.8 | 1.6965508 | 235.8 | 9.25747949 | 235.8 | 9.25747949 | 235.8 | 6.83523644 | 235.8 | 18.5639922 | | | 235.80 | 6.55084354 | | | 235.8 | 6.55084354 |
| 235.9 | 1.6965508 | 235.9 | 9.26728614 | 235.9 | 9.26728614 | 235.9 | 6.81562314 | 235.9 | 18.5934122 | | | 235.90 | 6.59007014 | | | 235.9 | 6.59007014 |
| 236 | 1.6965508 | 236 | 9.24767284 | 236 | 9.24767284 | 236 | 6.8646564 | 236 | 18.5737989 | | | 236.00 | 6.59007014 | | | 236 | 6.59007014 |
| 236.1 | 1.68674414 | 236.1 | 9.24767284 | 236.1 | 9.24767284 | 236.1 | 6.85484975 | 236.1 | 18.5934122 | | | 236.10 | 6.55084354 | | | 236.1 | 6.55084354 |
| 236.2 | 1.6965508 | 236.2 | 9.26728614 | 236.2 | 9.26728614 | 236.2 | 6.82542979 | 236.2 | 18.6130255 | | | 236.20 | 6.54103688 | | | 236.2 | 6.54103688 |
| 236.3 | 1.7357774 | 236.3 | 9.2967061 | 236.3 | 9.2967061 | 236.3 | 6.83523644 | 236.3 | 18.6032188 | | | 236.30 | 6.56065019 | | | 236.3 | 6.56065019 |
| 236.4 | 1.75539071 | 236.4 | 9.28689944 | 236.4 | 9.28689944 | 236.4 | 6.8450431 | 236.4 | 18.6130255 | | | 236.40 | 6.57045684 | | | 236.4 | 6.57045684 |
| 236.5 | 1.76519736 | 236.5 | 9.27709279 | 236.5 | 9.27709279 | 236.5 | 6.8842697 | 236.5 | 18.6326388 | | | 236.50 | 6.55084354 | | | 236.5 | 6.55084354 |
| 236.6 | 1.75539071 | 236.6 | 9.27709279 | 236.6 | 9.27709279 | 236.6 | 6.8842697 | 236.6 | 18.6620588 | | | 236.60 | 6.54103688 | | | 236.6 | 6.54103688 |
| 236.7 | 1.76519736 | 236.7 | 9.25747949 | 236.7 | 9.25747949 | 236.7 | 6.87446305 | 236.7 | 18.6620588 | | | 236.70 | 6.58026349 | | | 236.7 | 6.58026349 |
| 236.8 | 1.75539071 | 236.8 | 9.22805953 | 236.8 | 9.22805953 | 236.8 | 6.8842697 | 236.8 | 18.6816721 | | | 236.80 | 6.56065019 | | | 236.8 | 6.56065019 |
| 236.9 | 1.72597075 | 236.9 | 9.22805953 | 236.9 | 9.22805953 | 236.9 | 6.87446305 | 236.9 | 18.6816721 | | | 236.90 | 6.57045684 | | | 236.9 | 6.57045684 |
| 237 | 1.74558406 | 237 | 9.22805953 | 237 | 9.22805953 | 237 | 6.8646564 | 237 | 18.7012854 | | | 237.00 | 6.57045684 | | | 237 | 6.57045684 |
| 237.1 | 1.7357774 | 237.1 | 9.24767284 | 237.1 | 9.24767284 | 237.1 | 6.85484975 | 237.1 | 18.711092 | | | 237.10 | 6.56065019 | | | 237.1 | 6.56065019 |
| 237.2 | 1.74558406 | 237.2 | 9.23786618 | 237.2 | 9.23786618 | 237.2 | 6.87446305 | 237.2 | 18.7307053 | | | 237.20 | 6.57045684 | | | 237.2 | 6.57045684 |
| 237.3 | 1.78481066 | 237.3 | 9.22805953 | 237.3 | 9.22805953 | 237.3 | 6.85484975 | 237.3 | 18.7208987 | | | 237.30 | 6.57045684 | | | 237.3 | 6.57045684 |
| 237.4 | 1.75539071 | 237.4 | 9.22805953 | 237.4 | 9.22805953 | 237.4 | 6.8646564 | 237.4 | 18.711092 | | | 237.40 | 6.56065019 | | | 237.4 | 6.56065019 |
| 237.5 | 1.76519736 | 237.5 | 9.22805953 | 237.5 | 9.22805953 | 237.5 | 6.85484975 | 237.5 | 18.7012854 | | | 237.50 | 6.55084354 | | | 237.5 | 6.55084354 |
| 237.6 | 1.76519736 | 237.6 | 9.22805953 | 237.6 | 9.22805953 | 237.6 | 6.87446305 | 237.6 | 18.711092 | | | 237.60 | 6.55084354 | | | 237.6 | 6.55084354 |
| 237.7 | 1.77500401 | 237.7 | 9.25747949 | 237.7 | 9.25747949 | 237.7 | 6.87446305 | 237.7 | 18.7601253 | | | 237.70 | 6.55084354 | | | 237.7 | 6.55084354 |
| 237.8 | 1.77500401 | 237.8 | 9.27709279 | 237.8 | 9.27709279 | 237.8 | 6.87446305 | 237.8 | 18.7601253 | | | 237.80 | 6.56065019 | | | 237.8 | 6.56065019 |
| 237.9 | 1.76519736 | 237.9 | 9.27709279 | 237.9 | 9.27709279 | 237.9 | 6.90388301 | 237.9 | 18.7895452 | | | 237.90 | 6.56065019 | | | 237.9 | 6.56065019 |
| 238 | 1.75539071 | 238 | 9.26728614 | 238 | 9.26728614 | 238 | 6.91368966 | 238 | 18.8091585 | | | 238.00 | 6.56065019 | | | 238 | 6.56065019 |
| 238.1 | 1.76519736 | 238.1 | 9.24767284 | 238.1 | 9.24767284 | 238.1 | 6.93330296 | 238.1 | 18.8189652 | | | 238.10 | 6.59007014 | | | 238.1 | 6.59007014 |
| 238.2 | 1.77500401 | 238.2 | 9.25747949 | 238.2 | 9.25747949 | 238.2 | 6.95291627 | 238.2 | 18.7993519 | | | 238.20 | 6.5998768 | | | 238.2 | 6.5998768 |
| 238.3 | 1.75539071 | 238.3 | 9.24767284 | 238.3 | 9.24767284 | 238.3 | 6.95291627 | 238.3 | 18.7797386 | | | 238.30 | 6.5998768 | | | 238.3 | 6.5998768 |
| 238.4 | 1.76519736 | 238.4 | 9.24767284 | 238.4 | 9.24767284 | 238.4 | 6.95291627 | 238.4 | 18.7895452 | | | 238.40 | 6.58026349 | | | 238.4 | 6.58026349 |
| 238.5 | 1.77500401 | 238.5 | 9.27709279 | 238.5 | 9.27709279 | 238.5 | 6.95291627 | 238.5 | 18.8091585 | | | 238.50 | 6.58026349 | | | 238.5 | 6.58026349 |
| 238.6 | 1.77500401 | 238.6 | 9.32612605 | 238.6 | 9.32612605 | 238.6 | 6.93330296 | 238.6 | 18.8189652 | | | 238.60 | 6.55084354 | | | 238.6 | 6.55084354 |
| 238.7 | 1.78481066 | 238.7 | 9.30651275 | 238.7 | 9.30651275 | 238.7 | 6.93330296 | 238.7 | 18.8385785 | | | 238.70 | 6.57045684 | | | 238.7 | 6.57045684 |
| 238.8 | 1.79461732 | 238.8 | 9.30651275 | 238.8 | 9.30651275 | 238.8 | 6.92349631 | 238.8 | 18.8483851 | | | 238.80 | 6.55084354 | | | 238.8 | 6.55084354 |
| 238.9 | 1.80442397 | 238.9 | 9.32612605 | 238.9 | 9.32612605 | 238.9 | 6.93330296 | 238.9 | 18.8679984 | | | 238.90 | 6.55084354 | | | 238.9 | 6.55084354 |
| 239 | 1.80442397 | 239 | 9.3359327 | 239 | 9.3359327 | 239 | 6.93330296 | 239 | 18.8385785 | | | 239.00 | 6.54103688 | | | 239 | 6.54103688 |
| 239.1 | 1.79461732 | 239.1 | 9.3163194 | 239.1 | 9.3163194 | 239.1 | 6.94310962 | 239.1 | 18.9072251 | | | 239.10 | 6.53123023 | | | 239.1 | 6.53123023 |
| 239.2 | 1.77500401 | 239.2 | 9.30651275 | 239.2 | 9.30651275 | 239.2 | 6.92349631 | 239.2 | 18.9268384 | | | 239.20 | 6.55084354 | | | 239.2 | 6.55084354 |
| 239.3 | 1.79461732 | 239.3 | 9.30651275 | 239.3 | 9.30651275 | 239.3 | 6.94310962 | 239.3 | 18.9170317 | | | 239.30 | 6.56065019 | | | 239.3 | 6.56065019 |
| 239.4 | 1.80442397 | 239.4 | 9.32612605 | 239.4 | 9.32612605 | 239.4 | 6.96272292 | 239.4 | 18.9268384 | | | 239.40 | 6.58026349 | | | 239.4 | 6.58026349 |
| 239.5 | 1.79461732 | 239.5 | 9.35554601 | 239.5 | 9.35554601 | 239.5 | 6.95291627 | 239.5 | 18.936645 | | | 239.50 | 6.5998768 | | | 239.5 | 6.5998768 |
| 239.6 | 1.79461732 | 239.6 | 9.37515931 | 239.6 | 9.37515931 | 239.6 | 6.92349631 | 239.6 | 18.936645 | | | 239.60 | 6.58026349 | | | 239.6 | 6.58026349 |
| 239.7 | 1.81423062 | 239.7 | 9.36535266 | 239.7 | 9.36535266 | 239.7 | 6.93330296 | 239.7 | 18.9758716 | | | 239.70 | 6.56065019 | | | 239.7 | 6.56065019 |
| 239.8 | 1.81423062 | 239.8 | 9.36535266 | 239.8 | 9.36535266 | 239.8 | 6.96272292 | 239.8 | 18.966065 | | | 239.80 | 6.54103688 | | | 239.8 | 6.54103688 |
| 239.9 | 1.83384392 | 239.9 | 9.37515931 | 239.9 | 9.37515931 | 239.9 | 6.94310962 | 239.9 | 18.966065 | | | 239.90 | 6.57045684 | | | 239.9 | 6.57045684 |
| 240 | 1.82403727 | 240 | 9.37515931 | 240 | 9.37515931 | 240 | 6.94310962 | 240 | 19.0150982 | | | 240.00 | 6.59007014 | | | 240 | 6.59007014 |
| 240.1 | 1.82403727 | 240.1 | 9.35554601 | 240.1 | 9.35554601 | 240.1 | 6.93330296 | 240.1 | 19.0543248 | | | 240.10 | 6.58026349 | | | 240.1 | 6.58026349 |
| 240.2 | 1.80442397 | 240.2 | 9.35554601 | 240.2 | 9.35554601 | 240.2 | 6.97252957 | 240.2 | 19.0445182 | | | 240.20 | 6.5998768 | | | 240.2 | 6.5998768 |
| 240.3 | 1.80442397 | 240.3 | 9.35554601 | 240.3 | 9.35554601 | 240.3 | 6.97252957 | 240.3 | 19.0445182 | | | 240.30 | 6.59007014 | | | 240.3 | 6.59007014 |
| 240.4 | 1.83384392 | 240.4 | 9.36535266 | 240.4 | 9.36535266 | 240.4 | 7.00194953 | 240.4 | 19.0543248 | | | 240.40 | 6.59007014 | | | 240.4 | 6.59007014 |
| 240.5 | 1.82403727 | 240.5 | 9.37515931 | 240.5 | 9.37515931 | 240.5 | 6.98233622 | 240.5 | 19.0347115 | | | 240.50 | 6.57045684 | | | 240.5 | 6.57045684 |
| 240.6 | 1.84365058 | 240.6 | 9.38496596 | 240.6 | 9.38496596 | 240.6 | 6.97252957 | 240.6 | 19.0445182 | | | 240.60 | 6.57045684 | | | 240.6 | 6.57045684 |
| 240.7 | 1.81423062 | 240.7 | 9.37515931 | 240.7 | 9.37515931 | 240.7 | 6.95291627 | 240.7 | 19.0641315 | | | 240.70 | 6.5998768 | | | 240.7 | 6.5998768 |
| 240.8 | 1.78481066 | 240.8 | 9.36535266 | 240.8 | 9.36535266 | 240.8 | 6.95291627 | 240.8 | 19.0837448 | | | 240.80 | 6.59007014 | | | 240.8 | 6.59007014 |
| 240.9 | 1.81423062 | 240.9 | 9.37515931 | 240.9 | 9.37515931 | 240.9 | 6.98233622 | 240.9 | 19.162198 | | | 240.90 | 6.5998768 | | | 240.9 | 6.5998768 |
| 241 | 1.83384392 | 241 | 9.37515931 | 241 | 9.37515931 | 241 | 7.01175618 | 241 | 19.1818113 | | | 241.00 | 6.6194901 | | | 241 | 6.6194901 |
| 241.1 | 1.84365058 | 241.1 | 9.36535266 | 241.1 | 9.36535266 | 241.1 | 7.00194953 | 241.1 | 19.1818113 | | | 241.10 | 6.5998768 | | | 241.1 | 6.5998768 |
| 241.2 | 1.83384392 | 241.2 | 9.34573936 | 241.2 | 9.34573936 | 241.2 | 7.01175618 | 241.2 | 19.1818113 | | | 241.20 | 6.59007014 | | | 241.2 | 6.59007014 |

| | | | | | | | | | | | | | | | | | |
|---|---|---|---|---|---|---|---|---|---|---|---|---|---|---|---|---|---|
| 241.3 | 1.83384392 | 241.3 | 9.35554601 | 241.3 | 9.35554601 | 241.3 | 6.99214288 | 241.3 | 19.1720047 | | | 241.30 | 6.58026349 | | | 241.3 | 6.58026349 |
| 241.4 | 1.85345723 | 241.4 | 9.38496596 | 241.4 | 9.38496596 | 241.4 | 7.02156283 | 241.4 | 19.191618 | | | 241.40 | 6.60968345 | | | 241.4 | 6.60968345 |
| 241.5 | 1.82403727 | 241.5 | 9.38496596 | 241.5 | 9.38496596 | 241.5 | 7.04117614 | 241.5 | 19.2210379 | | | 241.50 | 6.6194901 | | | 241.5 | 6.6194901 |
| 241.6 | 1.82403727 | 241.6 | 9.38496596 | 241.6 | 9.38496596 | 241.6 | 7.04117614 | 241.6 | 19.2112313 | | | 241.60 | 6.6194901 | | | 241.6 | 6.6194901 |
| 241.7 | 1.82403727 | 241.7 | 9.41438592 | 241.7 | 9.41438592 | 241.7 | 7.05098279 | 241.7 | 19.2014246 | | | 241.70 | 6.62929675 | | | 241.7 | 6.62929675 |
| 241.8 | 1.84365058 | 241.8 | 9.39477262 | 241.8 | 9.39477262 | 241.8 | 7.06078944 | 241.8 | 19.2014246 | | | 241.80 | 6.62929675 | | | 241.8 | 6.62929675 |
| 241.9 | 1.85345723 | 241.9 | 9.40457927 | 241.9 | 9.40457927 | 241.9 | 7.04117614 | 241.9 | 19.2210379 | | | 241.90 | 6.6391034 | | | 241.9 | 6.6391034 |
| 242 | 1.84365058 | 242 | 9.42419257 | 242 | 9.42419257 | 242 | 7.03136948 | 242 | 19.2210379 | | | 242.00 | 6.62929675 | | | 242 | 6.62929675 |
| 242.1 | 1.83384392 | 242.1 | 9.43399922 | 242.1 | 9.43399922 | 242.1 | 7.02156283 | 242.1 | 19.2406512 | | | 242.10 | 6.62929675 | | | 242.1 | 6.62929675 |
| 242.2 | 1.84365058 | 242.2 | 9.41438592 | 242.2 | 9.41438592 | 242.2 | 6.99214288 | 242.2 | 19.2896845 | | | 242.20 | 6.60968345 | | | 242.2 | 6.60968345 |
| 242.3 | 1.86326388 | 242.3 | 9.40457927 | 242.3 | 9.40457927 | 242.3 | 7.01175618 | 242.3 | 19.2994911 | | | 242.30 | 6.6194901 | | | 242.3 | 6.6194901 |
| 242.4 | 1.87307053 | 242.4 | 9.40457927 | 242.4 | 9.40457927 | 242.4 | 7.03136948 | 242.4 | 19.2896845 | | | 242.40 | 6.64891006 | | | 242.4 | 6.64891006 |
| 242.5 | 1.87307053 | 242.5 | 9.37515931 | 242.5 | 9.37515931 | 242.5 | 7.03136948 | 242.5 | 19.2602645 | | | 242.50 | 6.6391034 | | | 242.5 | 6.6391034 |
| 242.6 | 1.85345723 | 242.6 | 9.39477262 | 242.6 | 9.39477262 | 242.6 | 7.03136948 | 242.6 | 19.2700712 | | | 242.60 | 6.64891006 | | | 242.6 | 6.64891006 |
| 242.7 | 1.86326388 | 242.7 | 9.41438592 | 242.7 | 9.41438592 | 242.7 | 7.04117614 | 242.7 | 19.3191044 | | | 242.70 | 6.6194901 | | | 242.7 | 6.6194901 |
| 242.8 | 1.86326388 | 242.8 | 9.41438592 | 242.8 | 9.41438592 | 242.8 | 7.07059609 | 242.8 | 19.358331 | | | 242.80 | 6.64891006 | | | 242.8 | 6.64891006 |
| 242.9 | 1.83384392 | 242.9 | 9.40457927 | 242.9 | 9.40457927 | 242.9 | 7.05098279 | 242.9 | 19.358331 | | | 242.90 | 6.64891006 | | | 242.9 | 6.64891006 |
| 243 | 1.87307053 | 243 | 9.41438592 | 243 | 9.41438592 | 243 | 7.05098279 | 243 | 19.417171 | | | 243.00 | 6.64891006 | | | 243 | 6.64891006 |
| 243.1 | 1.92210379 | 243.1 | 9.43399922 | 243.1 | 9.43399922 | 243.1 | 7.04117614 | 243.1 | 19.4269776 | | | 243.10 | 6.66852336 | | | 243.1 | 6.66852336 |
| 243.2 | 1.90249049 | 243.2 | 9.43399922 | 243.2 | 9.43399922 | 243.2 | 7.06078944 | 243.2 | 19.4367843 | | | 243.20 | 6.64891006 | | | 243.2 | 6.64891006 |
| 243.3 | 1.87307053 | 243.3 | 9.41438592 | 243.3 | 9.41438592 | 243.3 | 7.08040274 | 243.3 | 19.4367843 | | | 243.30 | 6.64891006 | | | 243.3 | 6.64891006 |
| 243.4 | 1.89268384 | 243.4 | 9.43399922 | 243.4 | 9.43399922 | 243.4 | 7.1098227 | 243.4 | 19.4563976 | | | 243.40 | 6.64891006 | | | 243.4 | 6.64891006 |
| 243.5 | 1.89268384 | 243.5 | 9.43399922 | 243.5 | 9.43399922 | 243.5 | 7.07059609 | 243.5 | 19.4563976 | | | 243.50 | 6.67833001 | | | 243.5 | 6.67833001 |
| 243.6 | 1.90249049 | 243.6 | 9.43399922 | 243.6 | 9.43399922 | 243.6 | 7.1098227 | 243.6 | 19.4563976 | | | 243.60 | 6.69794332 | | | 243.6 | 6.69794332 |
| 243.7 | 1.89268384 | 243.7 | 9.43399922 | 243.7 | 9.43399922 | 243.7 | 7.11962935 | 243.7 | 19.4073643 | | | 243.70 | 6.67833001 | | | 243.7 | 6.67833001 |
| 243.8 | 1.88287718 | 243.8 | 9.45361253 | 243.8 | 9.45361253 | 243.8 | 7.0902094 | 243.8 | 19.4073643 | | | 243.80 | 6.65871671 | | | 243.8 | 6.65871671 |
| 243.9 | 1.91229714 | 243.9 | 9.44380588 | 243.9 | 9.44380588 | 243.9 | 7.11962935 | 243.9 | 19.4073643 | | | 243.90 | 6.64891006 | | | 243.9 | 6.64891006 |
| 244 | 1.92210379 | 244 | 9.47322583 | 244 | 9.47322583 | 244 | 7.13924266 | 244 | 19.3975577 | | | 244.00 | 6.67833001 | | | 244 | 6.67833001 |
| 244.1 | 1.89268384 | 244.1 | 9.46341918 | 244.1 | 9.46341918 | 244.1 | 7.13924266 | 244.1 | 19.4269776 | | | 244.10 | 6.70774997 | | | 244.1 | 6.70774997 |
| 244.2 | 1.93191044 | 244.2 | 9.45361253 | 244.2 | 9.45361253 | 244.2 | 7.11962935 | 244.2 | 19.4662042 | | | 244.20 | 6.68813666 | | | 244.2 | 6.68813666 |
| 244.3 | 1.88287718 | 244.3 | 9.47322583 | 244.3 | 9.47322583 | 244.3 | 7.10001605 | 244.3 | 19.4858175 | | | 244.30 | 6.70774997 | | | 244.3 | 6.70774997 |
| 244.4 | 1.87307053 | 244.4 | 9.46341918 | 244.4 | 9.46341918 | 244.4 | 7.1098227 | 244.4 | 19.5250441 | | | 244.40 | 6.68813666 | | | 244.4 | 6.68813666 |
| 244.5 | 1.88287718 | 244.5 | 9.47322583 | 244.5 | 9.47322583 | 244.5 | 7.11962935 | 244.5 | 19.5250441 | | | 244.50 | 6.71755662 | | | 244.5 | 6.71755662 |
| 244.6 | 1.90249049 | 244.6 | 9.49283914 | 244.6 | 9.49283914 | 244.6 | 7.129436 | 244.6 | 19.5642707 | | | 244.60 | 6.72736327 | | | 244.6 | 6.72736327 |
| 244.7 | 1.89268384 | 244.7 | 9.50264579 | 244.7 | 9.50264579 | 244.7 | 7.16866261 | 244.7 | 19.583884 | | | 244.70 | 6.72736327 | | | 244.7 | 6.72736327 |
| 244.8 | 1.92210379 | 244.8 | 9.48303248 | 244.8 | 9.48303248 | 244.8 | 7.18827592 | 244.8 | 19.5642707 | | | 244.80 | 6.72736327 | | | 244.8 | 6.72736327 |
| 244.9 | 1.92210379 | 244.9 | 9.48303248 | 244.9 | 9.48303248 | 244.9 | 7.16866261 | 244.9 | 19.6034973 | | | 244.90 | 6.72736327 | | | 244.9 | 6.72736327 |
| 245 | 1.93191044 | 245 | 9.47322583 | 245 | 9.47322583 | 245 | 7.16866261 | 245 | 19.6231107 | | | 245.00 | 6.73716992 | | | 245 | 6.73716992 |
| 245.1 | 1.95152375 | 245.1 | 9.49283914 | 245.1 | 9.49283914 | 245.1 | 7.18827592 | 245.1 | 19.613304 | | | 245.10 | 6.74697658 | | | 245.1 | 6.74697658 |
| 245.2 | 1.9417171 | 245.2 | 9.50264579 | 245.2 | 9.50264579 | 245.2 | 7.20788922 | 245.2 | 19.583884 | | | 245.20 | 6.71755662 | | | 245.2 | 6.71755662 |
| 245.3 | 1.92210379 | 245.3 | 9.49283914 | 245.3 | 9.49283914 | 245.3 | 7.21769587 | 245.3 | 19.5152375 | | | 245.30 | 6.71755662 | | | 245.3 | 6.71755662 |
| 245.4 | 1.92210379 | 245.4 | 9.48303248 | 245.4 | 9.48303248 | 245.4 | 7.20788922 | 245.4 | 19.4760109 | | | 245.40 | 6.72736327 | | | 245.4 | 6.72736327 |
| 245.5 | 1.90249049 | 245.5 | 9.50264579 | 245.5 | 9.50264579 | 245.5 | 7.19808257 | 245.5 | 19.4465909 | | | 245.50 | 6.75678323 | | | 245.5 | 6.75678323 |
| 245.6 | 1.91229714 | 245.6 | 9.51245244 | 245.6 | 9.51245244 | 245.6 | 7.19808257 | 245.6 | 19.4073643 | | | 245.60 | 6.73716992 | | | 245.6 | 6.73716992 |
| 245.7 | 1.91229714 | 245.7 | 9.52225909 | 245.7 | 9.52225909 | 245.7 | 7.18827592 | 245.7 | 19.4269776 | | | 245.70 | 6.73716992 | | | 245.7 | 6.73716992 |
| 245.8 | 1.9613304 | 245.8 | 9.53206574 | 245.8 | 9.53206574 | 245.8 | 7.21769587 | 245.8 | 19.4662042 | | | 245.80 | 6.74697658 | | | 245.8 | 6.74697658 |
| 245.9 | 1.9809437 | 245.9 | 9.51245244 | 245.9 | 9.51245244 | 245.9 | 7.19808257 | 245.9 | 19.4760109 | | | 245.90 | 6.74697658 | | | 245.9 | 6.74697658 |
| 246 | 1.9809437 | 246 | 9.52225909 | 246 | 9.52225909 | 246 | 7.19808257 | 246 | 19.5054308 | | | 246.00 | 6.74697658 | | | 246 | 6.74697658 |
| 246.1 | 1.99075036 | 246.1 | 9.55167905 | 246.1 | 9.55167905 | 246.1 | 7.22750252 | 246.1 | 19.5446574 | | | 246.10 | 6.75678323 | | | 246.1 | 6.75678323 |
| 246.2 | 1.9417171 | 246.2 | 9.5418724 | 246.2 | 9.5418724 | 246.2 | 7.20788922 | 246.2 | 19.5348508 | | | 246.20 | 6.78620318 | | | 246.2 | 6.78620318 |
| 246.3 | 1.9417171 | 246.3 | 9.55167905 | 246.3 | 9.55167905 | 246.3 | 7.21769587 | 246.3 | 19.5348508 | | | 246.30 | 6.78620318 | | | 246.3 | 6.78620318 |
| 246.4 | 1.93191044 | 246.4 | 9.57129235 | 246.4 | 9.57129235 | 246.4 | 7.21769587 | 246.4 | 19.583884 | | | 246.40 | 6.78620318 | | | 246.4 | 6.78620318 |
| 246.5 | 1.93191044 | 246.5 | 9.5418724 | 246.5 | 9.5418724 | 246.5 | 7.22750252 | 246.5 | 19.6034973 | | | 246.50 | 6.78620318 | | | 246.5 | 6.78620318 |
| 246.6 | 1.92210379 | 246.6 | 9.55167905 | 246.6 | 9.55167905 | 246.6 | 7.23730918 | 246.6 | 19.5642707 | | | 246.60 | 6.80581649 | | | 246.6 | 6.80581649 |
| 246.7 | 1.9417171 | 246.7 | 9.53206574 | 246.7 | 9.53206574 | 246.7 | 7.21769587 | 246.7 | 19.5740774 | | | 246.70 | 6.83523644 | | | 246.7 | 6.83523644 |
| 246.8 | 1.92210379 | 246.8 | 9.5418724 | 246.8 | 9.5418724 | 246.8 | 7.23730918 | 246.8 | 19.5936907 | | | 246.80 | 6.79600984 | | | 246.8 | 6.79600984 |
| 246.9 | 1.95152375 | 246.9 | 9.581099 | 246.9 | 9.581099 | 246.9 | 7.24711583 | 246.9 | 19.6034973 | | | 246.90 | 6.78620318 | | | 246.9 | 6.78620318 |
| 247 | 1.97113705 | 247 | 9.5614857 | 247 | 9.5614857 | 247 | 7.26672913 | 247 | 19.6329173 | | | 247.00 | 6.78620318 | | | 247 | 6.78620318 |
| 247.1 | 1.99075036 | 247.1 | 9.5614857 | 247.1 | 9.5614857 | 247.1 | 7.26672913 | 247.1 | 19.642724 | | | 247.10 | 6.82542979 | | | 247.1 | 6.82542979 |
| 247.2 | 1.97113705 | 247.2 | 9.57129235 | 247.2 | 9.57129235 | 247.2 | 7.25692248 | 247.2 | 19.6623373 | | | 247.20 | 6.82542979 | | | 247.2 | 6.82542979 |
| 247.3 | 1.97113705 | 247.3 | 9.581099 | 247.3 | 9.581099 | 247.3 | 7.26672913 | 247.3 | 19.6525306 | | | 247.30 | 6.80581649 | | | 247.3 | 6.80581649 |

| | | | | | | | | | | | | | | | | | |
|---|---|---|---|---|---|---|---|---|---|---|---|---|---|---|---|---|---|
| 247.4 | 1.95152375 | 247.4 | 9.581099 | 247.4 | 9.581099 | 247.4 | 7.28634244 | 247.4 | 19.6329173 | | | 247.40 | 6.83523644 | | | 247.4 | 6.83523644 |
| 247.5 | 1.95152375 | 247.5 | 9.581099 | 247.5 | 9.581099 | 247.5 | 7.31576239 | 247.5 | 19.6623373 | | | 247.50 | 6.85484975 | | | 247.5 | 6.85484975 |
| 247.6 | 1.99075036 | 247.6 | 9.581099 | 247.6 | 9.581099 | 247.6 | 7.28634244 | 247.6 | 19.6819506 | | | 247.60 | 6.8450431 | | | 247.6 | 6.8450431 |
| 247.7 | 2.01036366 | 247.7 | 9.5614857 | 247.7 | 9.5614857 | 247.7 | 7.30595574 | 247.7 | 19.6819506 | | | 247.70 | 6.85484975 | | | 247.7 | 6.85484975 |
| 247.8 | 2.00055701 | 247.8 | 9.59090566 | 247.8 | 9.59090566 | 247.8 | 7.31576239 | 247.8 | 19.7211772 | | | 247.80 | 6.8646564 | | | 247.8 | 6.8646564 |
| 247.9 | 1.99075036 | 247.9 | 9.581099 | 247.9 | 9.581099 | 247.9 | 7.30595574 | 247.9 | 19.7309838 | | | 247.90 | 6.8450431 | | | 247.9 | 6.8450431 |
| 248 | 2.00055701 | 248 | 9.59090566 | 248 | 9.59090566 | 248 | 7.29614909 | 248 | 19.7113705 | | | 248.00 | 6.83523644 | | | 248 | 6.83523644 |
| 248.1 | 1.99075036 | 248.1 | 9.59090566 | 248.1 | 9.59090566 | 248.1 | 7.27653578 | 248.1 | 19.7309838 | | | 248.10 | 6.83523644 | | | 248.1 | 6.83523644 |
| 248.2 | 2.00055701 | 248.2 | 9.60071231 | 248.2 | 9.60071231 | 248.2 | 7.28634244 | 248.2 | 19.7604038 | | | 248.20 | 6.8450431 | | | 248.2 | 6.8450431 |
| 248.3 | 2.00055701 | 248.3 | 9.60071231 | 248.3 | 9.60071231 | 248.3 | 7.29614909 | 248.3 | 19.7800171 | | | 248.30 | 6.85484975 | | | 248.3 | 6.85484975 |
| 248.4 | 2.00055701 | 248.4 | 9.61051896 | 248.4 | 9.61051896 | 248.4 | 7.29614909 | 248.4 | 19.7898237 | | | 248.40 | 6.85484975 | | | 248.4 | 6.85484975 |
| 248.5 | 1.99075036 | 248.5 | 9.61051896 | 248.5 | 9.61051896 | 248.5 | 7.28634244 | 248.5 | 19.7898237 | | | 248.50 | 6.85484975 | | | 248.5 | 6.85484975 |
| 248.6 | 2.02017031 | 248.6 | 9.60071231 | 248.6 | 9.60071231 | 248.6 | 7.29614909 | 248.6 | 19.7604038 | | | 248.60 | 6.8646564 | | | 248.6 | 6.8646564 |
| 248.7 | 2.01036366 | 248.7 | 9.60071231 | 248.7 | 9.60071231 | 248.7 | 7.31576239 | 248.7 | 19.7211772 | | | 248.70 | 6.85484975 | | | 248.7 | 6.85484975 |
| 248.8 | 2.01036366 | 248.8 | 9.62032561 | 248.8 | 9.62032561 | 248.8 | 7.30595574 | 248.8 | 19.6721439 | | | 248.80 | 6.85484975 | | | 248.8 | 6.85484975 |
| 248.9 | 2.02997696 | 248.9 | 9.62032561 | 248.9 | 9.62032561 | 248.9 | 7.30595574 | 248.9 | 19.642724 | | | 248.90 | 6.8646564 | | | 248.9 | 6.8646564 |
| 249 | 1.99075036 | 249 | 9.60071231 | 249 | 9.60071231 | 249 | 7.29614909 | 249 | 19.6329173 | | | 249.00 | 6.8646564 | | | 249 | 6.8646564 |
| 249.1 | 1.99075036 | 249.1 | 9.63013226 | 249.1 | 9.63013226 | 249.1 | 7.31576239 | 249.1 | 19.642724 | | | 249.10 | 6.8842697 | | | 249.1 | 6.8842697 |
| 249.2 | 2.01036366 | 249.2 | 9.64974557 | 249.2 | 9.64974557 | 249.2 | 7.31576239 | 249.2 | 19.6525306 | | | 249.20 | 6.87446305 | | | 249.2 | 6.87446305 |
| 249.3 | 2.02017031 | 249.3 | 9.63013226 | 249.3 | 9.63013226 | 249.3 | 7.32556904 | 249.3 | 19.6525306 | | | 249.30 | 6.85484975 | | | 249.3 | 6.85484975 |
| 249.4 | 2.02997696 | 249.4 | 9.63013226 | 249.4 | 9.63013226 | 249.4 | 7.32556904 | 249.4 | 19.6819506 | | | 249.40 | 6.87446305 | | | 249.4 | 6.87446305 |
| 249.5 | 2.04959027 | 249.5 | 9.62032561 | 249.5 | 9.62032561 | 249.5 | 7.31576239 | 249.5 | 19.7113705 | | | 249.50 | 6.90388301 | | | 249.5 | 6.90388301 |
| 249.6 | 2.08881688 | 249.6 | 9.63013226 | 249.6 | 9.63013226 | 249.6 | 7.34518235 | 249.6 | 19.7604038 | | | 249.60 | 6.90388301 | | | 249.6 | 6.90388301 |
| 249.7 | 2.09862353 | 249.7 | 9.62032561 | 249.7 | 9.62032561 | 249.7 | 7.3353757 | 249.7 | 19.7800171 | | | 249.70 | 6.91368966 | | | 249.7 | 6.91368966 |
| 249.8 | 2.06920357 | 249.8 | 9.64974557 | 249.8 | 9.64974557 | 249.8 | 7.3353757 | 249.8 | 19.7898237 | | | 249.80 | 6.92349631 | | | 249.8 | 6.92349631 |
| 249.9 | 2.04959027 | 249.9 | 9.67916552 | 249.9 | 9.67916552 | 249.9 | 7.3353757 | 249.9 | 19.7996304 | | | 249.90 | 6.8842697 | | | 249.9 | 6.8842697 |
| 250 | 2.07901022 | 250 | 9.68897218 | 250 | 9.68897218 | 250 | 7.3353757 | 250 | 19.7996304 | | | 250.00 | 6.89407636 | | | 250 | 6.89407636 |
| 250.1 | 2.06920357 | 250.1 | 9.68897218 | 250.1 | 9.68897218 | 250.1 | 7.3353757 | 250.1 | 19.8486636 | | | 250.10 | 6.93330296 | | | 250.1 | 6.93330296 |
| 250.2 | 2.07901022 | 250.2 | 9.66935887 | 250.2 | 9.66935887 | 250.2 | 7.354989 | 250.2 | 19.8584703 | | | 250.20 | 6.92349631 | | | 250.2 | 6.92349631 |
| 250.3 | 2.05939692 | 250.3 | 9.65955222 | 250.3 | 9.65955222 | 250.3 | 7.3353757 | 250.3 | 19.8486636 | | | 250.30 | 6.89407636 | | | 250.3 | 6.89407636 |
| 250.4 | 2.03978362 | 250.4 | 9.65955222 | 250.4 | 9.65955222 | 250.4 | 7.3353757 | 250.4 | 19.868277 | | | 250.40 | 6.89407636 | | | 250.4 | 6.89407636 |
| 250.5 | 2.03978362 | 250.5 | 9.66935887 | 250.5 | 9.66935887 | 250.5 | 7.3746023 | 250.5 | 19.8878903 | | | 250.50 | 6.94310962 | | | 250.5 | 6.94310962 |
| 250.6 | 2.04959027 | 250.6 | 9.71839213 | 250.6 | 9.71839213 | 250.6 | 7.36479565 | 250.6 | 19.8584703 | | | 250.60 | 6.97252957 | | | 250.6 | 6.97252957 |
| 250.7 | 2.03978362 | 250.7 | 9.72819878 | 250.7 | 9.72819878 | 250.7 | 7.3746023 | 250.7 | 19.9173102 | | | 250.70 | 6.93330296 | | | 250.7 | 6.93330296 |
| 250.8 | 2.08881688 | 250.8 | 9.71839213 | 250.8 | 9.71839213 | 250.8 | 7.354989 | 250.8 | 19.9565368 | | | 250.80 | 6.95291627 | | | 250.8 | 6.95291627 |
| 250.9 | 2.08881688 | 250.9 | 9.70858548 | 250.9 | 9.70858548 | 250.9 | 7.3746023 | 250.9 | 19.9271169 | | | 250.90 | 6.94310962 | | | 250.9 | 6.94310962 |
| 251 | 2.05939692 | 251 | 9.71839213 | 251 | 9.71839213 | 251 | 7.39421561 | 251 | 19.9467302 | | | 251.00 | 6.94310962 | | | 251 | 6.94310962 |
| 251.1 | 2.04959027 | 251.1 | 9.72819878 | 251.1 | 9.72819878 | 251.1 | 7.40402226 | 251.1 | 19.9565368 | | | 251.10 | 6.95291627 | | | 251.1 | 6.95291627 |
| 251.2 | 2.09862353 | 251.2 | 9.73800544 | 251.2 | 9.73800544 | 251.2 | 7.38440896 | 251.2 | 19.9173102 | | | 251.20 | 6.95291627 | | | 251.2 | 6.95291627 |
| 251.3 | 2.09862353 | 251.3 | 9.69877883 | 251.3 | 9.69877883 | 251.3 | 7.36479565 | 251.3 | 19.8780836 | | | 251.30 | 6.96272292 | | | 251.3 | 6.96272292 |
| 251.4 | 2.06920357 | 251.4 | 9.70858548 | 251.4 | 9.70858548 | 251.4 | 7.38440896 | 251.4 | 19.8486636 | | | 251.40 | 6.96272292 | | | 251.4 | 6.96272292 |
| 251.5 | 2.06920357 | 251.5 | 9.73800544 | 251.5 | 9.73800544 | 251.5 | 7.39421561 | 251.5 | 19.8584703 | | | 251.50 | 6.98233622 | | | 251.5 | 6.98233622 |
| 251.6 | 2.05939692 | 251.6 | 9.75761874 | 251.6 | 9.75761874 | 251.6 | 7.36479565 | 251.6 | 19.7407905 | | | 251.60 | 7.00194953 | | | 251.6 | 7.00194953 |
| 251.7 | 2.05939692 | 251.7 | 9.75761874 | 251.7 | 9.75761874 | 251.7 | 7.3746023 | 251.7 | 19.7604038 | | | 251.70 | 6.98233622 | | | 251.7 | 6.98233622 |
| 251.8 | 2.09862353 | 251.8 | 9.71839213 | 251.8 | 9.71839213 | 251.8 | 7.41382891 | 251.8 | 19.8780836 | | | 251.80 | 6.95291627 | | | 251.8 | 6.95291627 |
| 251.9 | 2.09862353 | 251.9 | 9.70858548 | 251.9 | 9.70858548 | 251.9 | 7.41382891 | 251.9 | 19.9565368 | | | 251.90 | 6.94310962 | | | 251.9 | 6.94310962 |
| 252 | 2.08881688 | 252 | 9.72819878 | 252 | 9.72819878 | 252 | 7.42363556 | 252 | 19.8976969 | | | 252.00 | 6.95291627 | | | 252 | 6.95291627 |
| 252.1 | 2.11823683 | 252.1 | 9.70858548 | 252.1 | 9.70858548 | 252.1 | 7.40402226 | 252.1 | 19.7996304 | | | 252.10 | 6.97252957 | | | 252.1 | 6.97252957 |
| 252.2 | 2.09862353 | 252.2 | 9.72819878 | 252.2 | 9.72819878 | 252.2 | 7.40402226 | 252.2 | 19.809437 | | | 252.20 | 6.96272292 | | | 252.2 | 6.96272292 |
| 252.3 | 2.09862353 | 252.3 | 9.74781209 | 252.3 | 9.74781209 | 252.3 | 7.45305552 | 252.3 | 19.7996304 | | | 252.30 | 6.95291627 | | | 252.3 | 6.95291627 |
| 252.4 | 2.09862353 | 252.4 | 9.75761874 | 252.4 | 9.75761874 | 252.4 | 7.44324887 | 252.4 | 19.7996304 | | | 252.40 | 6.96272292 | | | 252.4 | 6.96272292 |
| 252.5 | 2.10843018 | 252.5 | 9.74781209 | 252.5 | 9.74781209 | 252.5 | 7.42363556 | 252.5 | 19.7996304 | | | 252.50 | 6.97252957 | | | 252.5 | 6.97252957 |
| 252.6 | 2.12804348 | 252.6 | 9.75761874 | 252.6 | 9.75761874 | 252.6 | 7.43344222 | 252.6 | 19.8192437 | | | 252.60 | 6.98233622 | | | 252.6 | 6.98233622 |
| 252.7 | 2.12804348 | 252.7 | 9.75761874 | 252.7 | 9.75761874 | 252.7 | 7.44324887 | 252.7 | 19.7702104 | | | 252.70 | 6.97252957 | | | 252.7 | 6.97252957 |
| 252.8 | 2.12804348 | 252.8 | 9.76742539 | 252.8 | 9.76742539 | 252.8 | 7.45305552 | 252.8 | 19.838857 | | | 252.80 | 6.97252957 | | | 252.8 | 6.97252957 |
| 252.9 | 2.15746344 | 252.9 | 9.75761874 | 252.9 | 9.75761874 | 252.9 | 7.45305552 | 252.9 | 19.9075036 | | | 252.90 | 6.99214288 | | | 252.9 | 6.99214288 |
| 253 | 2.14765679 | 253 | 9.77723204 | 253 | 9.77723204 | 253 | 7.41382891 | 253 | 19.9075036 | | | 253.00 | 6.98233622 | | | 253 | 6.98233622 |
| 253.1 | 2.10843018 | 253.1 | 9.79684535 | 253.1 | 9.79684535 | 253.1 | 7.41382891 | 253.1 | 19.9173102 | | | 253.10 | 6.96272292 | | | 253.1 | 6.96272292 |
| 253.2 | 2.12804348 | 253.2 | 9.76742539 | 253.2 | 9.76742539 | 253.2 | 7.44324887 | 253.2 | 19.9173102 | | | 253.20 | 6.95291627 | | | 253.2 | 6.95291627 |
| 253.3 | 2.12804348 | 253.3 | 9.77723204 | 253.3 | 9.77723204 | 253.3 | 7.47266882 | 253.3 | 19.868277 | | | 253.30 | 7.00194953 | | | 253.3 | 7.00194953 |
| 253.4 | 2.13785014 | 253.4 | 9.806652 | 253.4 | 9.806652 | 253.4 | 7.45305552 | 253.4 | 19.838857 | | | 253.40 | 6.98233622 | | | 253.4 | 6.98233622 |

| | | | | | | | | | | | | | | | | | |
|---|---|---|---|---|---|---|---|---|---|---|---|---|---|---|---|---|---|
| 253.5 | 2.14765679 | 253.5 | 9.77723204 | 253.5 | 9.77723204 | 253.5 | 7.44324887 | 253.5 | 19.8290503 | | | 253.50 | 6.97252957 | | | 253.5 | 6.97252957 |
| 253.6 | 2.12804348 | 253.6 | 9.75761874 | 253.6 | 9.75761874 | 253.6 | 7.47266882 | 253.6 | 19.8486636 | | | 253.60 | 7.01175618 | | | 253.6 | 7.01175618 |
| 253.7 | 2.15746344 | 253.7 | 9.79684535 | 253.7 | 9.79684535 | 253.7 | 7.48247548 | 253.7 | 19.838857 | | | 253.70 | 7.02156283 | | | 253.7 | 7.02156283 |
| 253.8 | 2.15746344 | 253.8 | 9.7870387 | 253.8 | 9.7870387 | 253.8 | 7.49228213 | 253.8 | 19.8192437 | | | 253.80 | 7.03136948 | | | 253.8 | 7.03136948 |
| 253.9 | 2.13785014 | 253.9 | 9.806652 | 253.9 | 9.806652 | 253.9 | 7.47266882 | 253.9 | 19.8486636 | | | 253.90 | 7.03136948 | | | 253.9 | 7.03136948 |
| 254 | 2.17707674 | 254 | 9.806652 | 254 | 9.806652 | 254 | 7.46286217 | 254 | 19.809437 | | | 254.00 | 7.01175618 | | | 254 | 7.01175618 |
| 254.1 | 2.17707674 | 254.1 | 9.806652 | 254.1 | 9.806652 | 254.1 | 7.47266882 | 254.1 | 19.8192437 | | | 254.10 | 7.01175618 | | | 254.1 | 7.01175618 |
| 254.2 | 2.15746344 | 254.2 | 9.806652 | 254.2 | 9.806652 | 254.2 | 7.43344222 | 254.2 | 19.8486636 | | | 254.20 | 7.00194953 | | | 254.2 | 7.00194953 |
| 254.3 | 2.15746344 | 254.3 | 9.7870387 | 254.3 | 9.7870387 | 254.3 | 7.41382891 | 254.3 | 19.8584703 | | | 254.30 | 6.99214288 | | | 254.3 | 6.99214288 |
| 254.4 | 2.17707674 | 254.4 | 9.79684535 | 254.4 | 9.79684535 | 254.4 | 7.43344222 | 254.4 | 19.8290503 | | | 254.40 | 6.96272292 | | | 254.4 | 6.96272292 |
| 254.5 | 2.17707674 | 254.5 | 9.806652 | 254.5 | 9.806652 | 254.5 | 7.46286217 | 254.5 | 19.7898237 | | | 254.50 | 7.00194953 | | | 254.5 | 7.00194953 |
| 254.6 | 2.16727009 | 254.6 | 9.84587861 | 254.6 | 9.84587861 | 254.6 | 7.45305552 | 254.6 | 19.7996304 | | | 254.60 | 7.01175618 | | | 254.6 | 7.01175618 |
| 254.7 | 2.16727009 | 254.7 | 9.85568526 | 254.7 | 9.85568526 | 254.7 | 7.45305552 | 254.7 | 19.809437 | | | 254.70 | 7.01175618 | | | 254.7 | 7.01175618 |
| 254.8 | 2.17707674 | 254.8 | 9.83607196 | 254.8 | 9.83607196 | 254.8 | 7.42363556 | 254.8 | 19.8192437 | | | 254.80 | 6.99214288 | | | 254.8 | 6.99214288 |
| 254.9 | 2.16727009 | 254.9 | 9.83607196 | 254.9 | 9.83607196 | 254.9 | 7.44324887 | 254.9 | 19.838857 | | | 254.90 | 7.02156283 | | | 254.9 | 7.02156283 |
| 255 | 2.14765679 | 255 | 9.83607196 | 255 | 9.83607196 | 255 | 7.43344222 | 255 | 19.809437 | | | 255.00 | 7.01175618 | | | 255 | 7.01175618 |
| 255.1 | 2.15746344 | 255.1 | 9.83607196 | 255.1 | 9.83607196 | 255.1 | 7.43344222 | 255.1 | 19.8486636 | | | 255.10 | 7.01175618 | | | 255.1 | 7.01175618 |
| 255.2 | 2.17707674 | 255.2 | 9.806652 | 255.2 | 9.806652 | 255.2 | 7.42363556 | 255.2 | 19.8584703 | | | 255.20 | 7.02156283 | | | 255.2 | 7.02156283 |
| 255.3 | 2.2064967 | 255.3 | 9.81645865 | 255.3 | 9.81645865 | 255.3 | 7.42363556 | 255.3 | 19.868277 | | | 255.30 | 7.01175618 | | | 255.3 | 7.01175618 |
| 255.4 | 2.17707674 | 255.4 | 9.85568526 | 255.4 | 9.85568526 | 255.4 | 7.45305552 | 255.4 | 19.868277 | | | 255.40 | 7.02156283 | | | 255.4 | 7.02156283 |
| 255.5 | 2.1868834 | 255.5 | 9.85568526 | 255.5 | 9.85568526 | 255.5 | 7.45305552 | 255.5 | 19.8878903 | | | 255.50 | 7.02156283 | | | 255.5 | 7.02156283 |
| 255.6 | 2.16727009 | 255.6 | 9.83607196 | 255.6 | 9.83607196 | 255.6 | 7.44324887 | 255.6 | 19.9369235 | | | 255.60 | 7.02156283 | | | 255.6 | 7.02156283 |
| 255.7 | 2.17707674 | 255.7 | 9.83607196 | 255.7 | 9.83607196 | 255.7 | 7.44324887 | 255.7 | 19.9859568 | | | 255.70 | 7.03136948 | | | 255.7 | 7.03136948 |
| 255.8 | 2.1868834 | 255.8 | 9.85568526 | 255.8 | 9.85568526 | 255.8 | 7.44324887 | 255.8 | 19.7898237 | | | 255.80 | 7.04117614 | | | 255.8 | 7.04117614 |
| 255.9 | 2.1868834 | 255.9 | 9.87529856 | 255.9 | 9.87529856 | 255.9 | 7.45305552 | 255.9 | 19.6623373 | | | 255.90 | 7.02156283 | | | 255.9 | 7.02156283 |
| 256 | 2.19669005 | 256 | 9.87529856 | 256 | 9.87529856 | 256 | 7.46286217 | 256 | 19.868277 | | | 256.00 | 7.02156283 | | | 256 | 7.02156283 |
| 256.1 | 2.19669005 | 256.1 | 9.87529856 | 256.1 | 9.87529856 | 256.1 | 7.45305552 | 256.1 | 19.9369235 | | | 256.10 | 7.03136948 | | | 256.1 | 7.03136948 |
| 256.2 | 2.19669005 | 256.2 | 9.88510522 | 256.2 | 9.88510522 | 256.2 | 7.46286217 | 256.2 | 19.9271169 | | | 256.20 | 7.05098279 | | | 256.2 | 7.05098279 |
| 256.3 | 2.24572331 | 256.3 | 9.88510522 | 256.3 | 9.88510522 | 256.3 | 7.47266882 | 256.3 | 19.9271169 | | | 256.30 | 7.06078944 | | | 256.3 | 7.06078944 |
| 256.4 | 2.21630335 | 256.4 | 9.88510522 | 256.4 | 9.88510522 | 256.4 | 7.49228213 | 256.4 | 19.9957634 | | | 256.40 | 7.07059609 | | | 256.4 | 7.07059609 |
| 256.5 | 2.19669005 | 256.5 | 9.90471852 | 256.5 | 9.90471852 | 256.5 | 7.51189543 | 256.5 | 20.1036366 | | | 256.50 | 7.05098279 | | | 256.5 | 7.05098279 |
| 256.6 | 2.22611 | 256.6 | 9.90471852 | 256.6 | 9.90471852 | 256.6 | 7.49228213 | 256.6 | 20.1036366 | | | 256.60 | 7.05098279 | | | 256.6 | 7.05098279 |
| 256.7 | 2.24572331 | 256.7 | 9.90471852 | 256.7 | 9.90471852 | 256.7 | 7.49228213 | 256.7 | 19.9663435 | | | 256.70 | 7.06078944 | | | 256.7 | 7.06078944 |
| 256.8 | 2.2064967 | 256.8 | 9.92433182 | 256.8 | 9.92433182 | 256.8 | 7.50208878 | 256.8 | 19.7800171 | | | 256.80 | 7.04117614 | | | 256.8 | 7.04117614 |
| 256.9 | 2.19669005 | 256.9 | 9.93413848 | 256.9 | 9.93413848 | 256.9 | 7.49228213 | 256.9 | 19.6329173 | | | 256.90 | 7.05098279 | | | 256.9 | 7.05098279 |
| 257 | 2.23591666 | 257 | 9.93413848 | 257 | 9.93413848 | 257 | 7.47266882 | 257 | 19.7309838 | | | 257.00 | 7.06078944 | | | 257 | 7.06078944 |
| 257.1 | 2.23591666 | 257.1 | 9.91452517 | 257.1 | 9.91452517 | 257.1 | 7.51189543 | 257.1 | 19.7800171 | | | 257.10 | 7.08040274 | | | 257.1 | 7.08040274 |
| 257.2 | 2.24572331 | 257.2 | 9.93413848 | 257.2 | 9.93413848 | 257.2 | 7.52170208 | 257.2 | 19.6525306 | | | 257.20 | 7.05098279 | | | 257.2 | 7.05098279 |
| 257.3 | 2.27514326 | 257.3 | 9.92433182 | 257.3 | 9.92433182 | 257.3 | 7.52170208 | 257.3 | 19.6819506 | | | 257.30 | 7.05098279 | | | 257.3 | 7.05098279 |
| 257.4 | 2.26533661 | 257.4 | 9.93413848 | 257.4 | 9.93413848 | 257.4 | 7.51189543 | 257.4 | 19.809437 | | | 257.40 | 7.0902094 | | | 257.4 | 7.0902094 |
| 257.5 | 2.27514326 | 257.5 | 9.95375178 | 257.5 | 9.95375178 | 257.5 | 7.47266882 | 257.5 | 19.7505971 | | | 257.50 | 7.07059609 | | | 257.5 | 7.07059609 |
| 257.6 | 2.25552996 | 257.6 | 9.96355843 | 257.6 | 9.96355843 | 257.6 | 7.48247548 | 257.6 | 19.838857 | | | 257.60 | 7.06078944 | | | 257.6 | 7.06078944 |
| 257.7 | 2.23591666 | 257.7 | 9.96355843 | 257.7 | 9.96355843 | 257.7 | 7.47266882 | 257.7 | 19.7505971 | | | 257.70 | 7.10001605 | | | 257.7 | 7.10001605 |
| 257.8 | 2.26533661 | 257.8 | 9.96355843 | 257.8 | 9.96355843 | 257.8 | 7.52170208 | 257.8 | 19.7015639 | | | 257.80 | 7.1098227 | | | 257.8 | 7.1098227 |
| 257.9 | 2.28494992 | 257.9 | 9.93413848 | 257.9 | 9.93413848 | 257.9 | 7.49228213 | 257.9 | 19.7113705 | | | 257.90 | 7.08040274 | | | 257.9 | 7.08040274 |
| 258 | 2.26533661 | 258 | 9.95375178 | 258 | 9.95375178 | 258 | 7.51189543 | 258 | 19.7309838 | | | 258.00 | 7.0902094 | | | 258 | 7.0902094 |
| 258.1 | 2.27514326 | 258.1 | 9.99297839 | 258.1 | 9.99297839 | 258.1 | 7.55112204 | 258.1 | 19.868277 | | | 258.10 | 7.07059609 | | | 258.1 | 7.07059609 |
| 258.2 | 2.27514326 | 258.2 | 9.97336508 | 258.2 | 9.97336508 | 258.2 | 7.55112204 | 258.2 | 20.0153767 | | | 258.20 | 7.10001605 | | | 258.2 | 7.10001605 |
| 258.3 | 2.24572331 | 258.3 | 9.97336508 | 258.3 | 9.97336508 | 258.3 | 7.54131539 | 258.3 | 20.0546033 | | | 258.30 | 7.10001605 | | | 258.3 | 7.10001605 |
| 258.4 | 2.26533661 | 258.4 | 9.98317174 | 258.4 | 9.98317174 | 258.4 | 7.54131539 | 258.4 | 20.06441 | | | 258.40 | 7.1098227 | | | 258.4 | 7.1098227 |
| 258.5 | 2.25552996 | 258.5 | 9.99297839 | 258.5 | 9.99297839 | 258.5 | 7.56092869 | 258.5 | 20.0153767 | | | 258.50 | 7.1098227 | | | 258.5 | 7.1098227 |
| 258.6 | 2.24572331 | 258.6 | 10.0125917 | 258.6 | 10.0125917 | 258.6 | 7.53150874 | 258.6 | 19.7996304 | | | 258.60 | 7.11962935 | | | 258.6 | 7.11962935 |
| 258.7 | 2.27514326 | 258.7 | 9.99297839 | 258.7 | 9.99297839 | 258.7 | 7.54131539 | 258.7 | 19.6034973 | | | 258.70 | 7.129436 | | | 258.7 | 7.129436 |
| 258.8 | 2.28494992 | 258.8 | 9.99297839 | 258.8 | 9.99297839 | 258.8 | 7.580542 | 258.8 | 19.6034973 | | | 258.80 | 7.10001605 | | | 258.8 | 7.10001605 |
| 258.9 | 2.29475657 | 258.9 | 10.0125917 | 258.9 | 10.0125917 | 258.9 | 7.580542 | 258.9 | 19.5936907 | | | 258.90 | 7.11962935 | | | 258.9 | 7.11962935 |
| 259 | 2.27514326 | 259 | 10.0223983 | 259 | 10.0223983 | 259 | 7.56092869 | 259 | 19.5446574 | | | 259.00 | 7.14904931 | | | 259 | 7.14904931 |
| 259.1 | 2.28494992 | 259.1 | 10.0125917 | 259.1 | 10.0125917 | 259.1 | 7.6001553 | 259.1 | 19.4858175 | | | 259.10 | 7.129436 | | | 259.1 | 7.129436 |
| 259.2 | 2.32417652 | 259.2 | 10.002785 | 259.2 | 10.002785 | 259.2 | 7.60996195 | 259.2 | 19.4956242 | | | 259.20 | 7.10001605 | | | 259.2 | 7.10001605 |
| 259.3 | 2.30456322 | 259.3 | 9.99297839 | 259.3 | 9.99297839 | 259.3 | 7.6001553 | 259.3 | 19.5250441 | | | 259.30 | 7.1098227 | | | 259.3 | 7.1098227 |
| 259.4 | 2.29475657 | 259.4 | 10.0420116 | 259.4 | 10.0420116 | 259.4 | 7.60996195 | 259.4 | 19.5054308 | | | 259.40 | 7.11962935 | | | 259.4 | 7.11962935 |
| 259.5 | 2.31436987 | 259.5 | 10.061625 | 259.5 | 10.061625 | 259.5 | 7.59034865 | 259.5 | 19.4367843 | | | 259.50 | 7.13924266 | | | 259.5 | 7.13924266 |

| | | | | | | | | | | | | | | | | | |
|---|---|---|---|---|---|---|---|---|---|---|---|---|---|---|---|---|---|
| 259.6 | 2.34378983 | 259.6 | 10.0714316 | 259.6 | 10.0714316 | 259.6 | 7.60996195 | 259.6 | 19.417171 | | | 259.60 | 7.14904931 | | | 259.6 | 7.14904931 |
| 259.7 | 2.30456322 | 259.7 | 10.0518183 | 259.7 | 10.0518183 | 259.7 | 7.6197686 | 259.7 | 19.4563976 | | | 259.70 | 7.11962935 | | | 259.7 | 7.11962935 |
| 259.8 | 2.29475657 | 259.8 | 10.032205 | 259.8 | 10.032205 | 259.8 | 7.6197686 | 259.8 | 19.6231107 | | | 259.80 | 7.11962935 | | | 259.8 | 7.11962935 |
| 259.9 | 2.30456322 | 259.9 | 10.032205 | 259.9 | 10.032205 | 259.9 | 7.64918856 | 259.9 | 19.5936907 | | | 259.90 | 7.1098227 | | | 259.9 | 7.1098227 |
| 260 | 2.30456322 | 260 | 10.0518183 | 260 | 10.0518183 | 260 | 7.60996195 | 260 | 19.5348508 | | | 260.00 | 7.13924266 | | | 260 | 7.13924266 |
| 260.1 | 2.32417652 | 260.1 | 10.061625 | 260.1 | 10.061625 | 260.1 | 7.6197686 | 260.1 | 19.4563976 | | | 260.10 | 7.14904931 | | | 260.1 | 7.14904931 |
| 260.2 | 2.33398318 | 260.2 | 10.0518183 | 260.2 | 10.0518183 | 260.2 | 7.63938191 | 260.2 | 19.5348508 | | | 260.20 | 7.15885596 | | | 260.2 | 7.15885596 |
| 260.3 | 2.30456322 | 260.3 | 10.0518183 | 260.3 | 10.0518183 | 260.3 | 7.6001553 | 260.3 | 19.7015639 | | | 260.30 | 7.16866261 | | | 260.3 | 7.16866261 |
| 260.4 | 2.29475657 | 260.4 | 10.0714316 | 260.4 | 10.0714316 | 260.4 | 7.6197686 | 260.4 | 19.7800171 | | | 260.40 | 7.15885596 | | | 260.4 | 7.15885596 |
| 260.5 | 2.31436987 | 260.5 | 10.0910449 | 260.5 | 10.0910449 | 260.5 | 7.60996195 | 260.5 | 19.8486636 | | | 260.50 | 7.15885596 | | | 260.5 | 7.15885596 |
| 260.6 | 2.34378983 | 260.6 | 10.0714316 | 260.6 | 10.0714316 | 260.6 | 7.60996195 | 260.6 | 19.7800171 | | | 260.60 | 7.16866261 | | | 260.6 | 7.16866261 |
| 260.7 | 2.32417652 | 260.7 | 10.061625 | 260.7 | 10.061625 | 260.7 | 7.6197686 | 260.7 | 19.7309838 | | | 260.70 | 7.17846926 | | | 260.7 | 7.17846926 |
| 260.8 | 2.31436987 | 260.8 | 10.061625 | 260.8 | 10.061625 | 260.8 | 7.63938191 | 260.8 | 19.7702104 | | | 260.80 | 7.18827592 | | | 260.8 | 7.18827592 |
| 260.9 | 2.33398318 | 260.9 | 10.0812383 | 260.9 | 10.0812383 | 260.9 | 7.65899521 | 260.9 | 19.6917572 | | | 260.90 | 7.18827592 | | | 260.9 | 7.18827592 |
| 261 | 2.36340313 | 261 | 10.0812383 | 261 | 10.0812383 | 261 | 7.68841517 | 261 | 19.4956242 | | | 261.00 | 7.21769587 | | | 261 | 7.21769587 |
| 261.1 | 2.34378983 | 261.1 | 10.0714316 | 261.1 | 10.0714316 | 261.1 | 7.70802847 | 261.1 | 19.4956242 | | | 261.10 | 7.18827592 | | | 261.1 | 7.18827592 |
| 261.2 | 2.33398318 | 261.2 | 10.1106582 | 261.2 | 10.1106582 | 261.2 | 7.67860852 | 261.2 | 19.417171 | | | 261.20 | 7.14904931 | | | 261.2 | 7.14904931 |
| 261.3 | 2.34378983 | 261.3 | 10.1302715 | 261.3 | 10.1302715 | 261.3 | 7.68841517 | 261.3 | 19.5152375 | | | 261.30 | 7.17846926 | | | 261.3 | 7.17846926 |
| 261.4 | 2.35359648 | 261.4 | 10.1302715 | 261.4 | 10.1302715 | 261.4 | 7.70802847 | 261.4 | 19.7702104 | | | 261.40 | 7.21769587 | | | 261.4 | 7.21769587 |
| 261.5 | 2.34378983 | 261.5 | 10.1400782 | 261.5 | 10.1400782 | 261.5 | 7.71783512 | 261.5 | 19.8976969 | | | 261.50 | 7.19808257 | | | 261.5 | 7.19808257 |
| 261.6 | 2.35359648 | 261.6 | 10.1400782 | 261.6 | 10.1400782 | 261.6 | 7.71783512 | 261.6 | 19.8584703 | | | 261.60 | 7.21769587 | | | 261.6 | 7.21769587 |
| 261.7 | 2.32417652 | 261.7 | 10.1596915 | 261.7 | 10.1596915 | 261.7 | 7.71783512 | 261.7 | 19.838857 | | | 261.70 | 7.23730918 | | | 261.7 | 7.23730918 |
| 261.8 | 2.35359648 | 261.8 | 10.1498848 | 261.8 | 10.1498848 | 261.8 | 7.74725508 | 261.8 | 19.7996304 | | | 261.80 | 7.23730918 | | | 261.8 | 7.23730918 |
| 261.9 | 2.35359648 | 261.9 | 10.1400782 | 261.9 | 10.1400782 | 261.9 | 7.75706173 | 261.9 | 19.6917572 | | | 261.90 | 7.22750252 | | | 261.9 | 7.22750252 |
| 262 | 2.33398318 | 262 | 10.1302715 | 262 | 10.1302715 | 262 | 7.72764178 | 262 | 19.5740774 | | | 262.00 | 7.22750252 | | | 262 | 7.22750252 |
| 262.1 | 2.34378983 | 262.1 | 10.1498848 | 262.1 | 10.1498848 | 262.1 | 7.73744843 | 262.1 | 19.642724 | | | 262.10 | 7.21769587 | | | 262.1 | 7.21769587 |
| 262.2 | 2.37320978 | 262.2 | 10.1400782 | 262.2 | 10.1400782 | 262.2 | 7.74725508 | 262.2 | 19.5152375 | | | 262.20 | 7.21769587 | | | 262.2 | 7.21769587 |
| 262.3 | 2.37320978 | 262.3 | 10.1596915 | 262.3 | 10.1596915 | 262.3 | 7.81590164 | 262.3 | 19.3975577 | | | 262.30 | 7.22750252 | | | 262.3 | 7.22750252 |
| 262.4 | 2.34378983 | 262.4 | 10.1596915 | 262.4 | 10.1596915 | 262.4 | 7.79628834 | 262.4 | 19.3779444 | | | 262.40 | 7.25692248 | | | 262.4 | 7.25692248 |
| 262.5 | 2.35359648 | 262.5 | 10.1694981 | 262.5 | 10.1694981 | 262.5 | 7.77667504 | 262.5 | 19.358331 | | | 262.50 | 7.24711583 | | | 262.5 | 7.24711583 |
| 262.6 | 2.37320978 | 262.6 | 10.1793048 | 262.6 | 10.1793048 | 262.6 | 7.78648169 | 262.6 | 19.4269776 | | | 262.60 | 7.25692248 | | | 262.6 | 7.25692248 |
| 262.7 | 2.37320978 | 262.7 | 10.1694981 | 262.7 | 10.1694981 | 262.7 | 7.81590164 | 262.7 | 19.5250441 | | | 262.70 | 7.26672913 | | | 262.7 | 7.26672913 |
| 262.8 | 2.36340313 | 262.8 | 10.1989181 | 262.8 | 10.1989181 | 262.8 | 7.79628834 | 262.8 | 19.613304 | | | 262.80 | 7.24711583 | | | 262.8 | 7.24711583 |
| 262.9 | 2.39282309 | 262.9 | 10.2087247 | 262.9 | 10.2087247 | 262.9 | 7.77667504 | 262.9 | 19.7211772 | | | 262.90 | 7.22750252 | | | 262.9 | 7.22750252 |
| 263 | 2.41243639 | 263 | 10.1891114 | 263 | 10.1891114 | 263 | 7.81590164 | 263 | 19.7113705 | | | 263.00 | 7.21769587 | | | 263 | 7.21769587 |
| 263.1 | 2.42224304 | 263.1 | 10.2087247 | 263.1 | 10.2087247 | 263.1 | 7.8257083 | 263.1 | 19.6721439 | | | 263.10 | 7.22750252 | | | 263.1 | 7.22750252 |
| 263.2 | 2.40262974 | 263.2 | 10.2087247 | 263.2 | 10.2087247 | 263.2 | 7.81590164 | 263.2 | 19.6034973 | | | 263.20 | 7.22750252 | | | 263.2 | 7.22750252 |
| 263.3 | 2.40262974 | 263.3 | 10.1989181 | 263.3 | 10.1989181 | 263.3 | 7.8453216 | 263.3 | 19.5642707 | | | 263.30 | 7.22750252 | | | 263.3 | 7.22750252 |
| 263.4 | 2.37320978 | 263.4 | 10.2087247 | 263.4 | 10.2087247 | 263.4 | 7.83551495 | 263.4 | 19.5544641 | | | 263.40 | 7.22750252 | | | 263.4 | 7.22750252 |
| 263.5 | 2.36340313 | 263.5 | 10.2185314 | 263.5 | 10.2185314 | 263.5 | 7.81590164 | 263.5 | 19.5642707 | | | 263.50 | 7.23730918 | | | 263.5 | 7.23730918 |
| 263.6 | 2.37320978 | 263.6 | 10.2087247 | 263.6 | 10.2087247 | 263.6 | 7.8257083 | 263.6 | 19.4760109 | | | 263.60 | 7.23730918 | | | 263.6 | 7.23730918 |
| 263.7 | 2.41243639 | 263.7 | 10.2185314 | 263.7 | 10.2185314 | 263.7 | 7.85512825 | 263.7 | 19.4073643 | | | 263.70 | 7.24711583 | | | 263.7 | 7.24711583 |
| 263.8 | 2.40262974 | 263.8 | 10.2479513 | 263.8 | 10.2479513 | 263.8 | 7.8453216 | 263.8 | 19.3975577 | | | 263.80 | 7.24711583 | | | 263.8 | 7.24711583 |
| 263.9 | 2.40262974 | 263.9 | 10.2381447 | 263.9 | 10.2381447 | 263.9 | 7.8649349 | 263.9 | 19.4269776 | | | 263.90 | 7.25692248 | | | 263.9 | 7.25692248 |
| 264 | 2.42224304 | 264 | 10.2381447 | 264 | 10.2381447 | 264 | 7.87474156 | 264 | 19.3975577 | | | 264.00 | 7.28634244 | | | 264 | 7.28634244 |
| 264.1 | 2.4320497 | 264.1 | 10.257758 | 264.1 | 10.257758 | 264.1 | 7.87474156 | 264.1 | 19.4269776 | | | 264.10 | 7.29614909 | | | 264.1 | 7.29614909 |
| 264.2 | 2.4320497 | 264.2 | 10.2773713 | 264.2 | 10.2773713 | 264.2 | 7.89435486 | 264.2 | 19.4563976 | | | 264.20 | 7.29614909 | | | 264.2 | 7.29614909 |
| 264.3 | 2.42224304 | 264.3 | 10.2773713 | 264.3 | 10.2773713 | 264.3 | 7.88454821 | 264.3 | 19.4367843 | | | 264.30 | 7.27653578 | | | 264.3 | 7.27653578 |
| 264.4 | 2.42224304 | 264.4 | 10.2773713 | 264.4 | 10.2773713 | 264.4 | 7.85512825 | 264.4 | 19.3485244 | | | 264.40 | 7.25692248 | | | 264.4 | 7.25692248 |
| 264.5 | 2.4320497 | 264.5 | 10.2773713 | 264.5 | 10.2773713 | 264.5 | 7.85512825 | 264.5 | 18.7307053 | | | 264.50 | 7.29614909 | | | 264.5 | 7.29614909 |
| 264.6 | 2.40262974 | 264.6 | 10.2969846 | 264.6 | 10.2969846 | 264.6 | 7.83551495 | 264.6 | 17.7598468 | | | 264.60 | 7.28634244 | | | 264.6 | 7.28634244 |
| 264.7 | 2.41243639 | 264.7 | 10.2871779 | 264.7 | 10.2871779 | 264.7 | 7.87474156 | 264.7 | 16.5438219 | | | 264.70 | 7.23730918 | | | 264.7 | 7.23730918 |
| 264.8 | 2.42224304 | 264.8 | 10.2871779 | 264.8 | 10.2871779 | 264.8 | 7.90416151 | 264.8 | 15.3376037 | | | 264.80 | 7.25692248 | | | 264.8 | 7.25692248 |
| 264.9 | 2.42224304 | 264.9 | 10.2871779 | 264.9 | 10.2871779 | 264.9 | 7.90416151 | 264.9 | 14.258872 | | | 264.90 | 7.30595574 | | | 264.9 | 7.30595574 |
| 265 | 2.42224304 | 265 | 10.2871779 | 265 | 10.2871779 | 265 | 7.90416151 | 265 | 13.1213004 | | | 265.00 | 7.30595574 | | | 265 | 7.30595574 |
| 265.1 | 2.4320497 | 265.1 | 10.3165979 | 265.1 | 10.3165979 | 265.1 | 7.90416151 | 265.1 | 11.9543088 | | | 265.10 | 7.27653578 | | | 265.1 | 7.27653578 |
| 265.2 | 2.44185635 | 265.2 | 10.3165979 | 265.2 | 10.3165979 | 265.2 | 7.87474156 | 265.2 | 10.7775105 | | | 265.20 | 7.27653578 | | | 265.2 | 7.27653578 |
| 265.3 | 2.4320497 | 265.3 | 10.3067913 | 265.3 | 10.3067913 | 265.3 | 7.87474156 | 265.3 | 9.53206574 | | | 265.30 | 7.28634244 | | | 265.3 | 7.28634244 |
| 265.4 | 2.42224304 | 265.4 | 10.3362112 | 265.4 | 10.3362112 | 265.4 | 7.92377482 | 265.4 | 8.36507416 | | | 265.40 | 7.30595574 | | | 265.4 | 7.30595574 |
| 265.5 | 2.40262974 | 265.5 | 10.3165979 | 265.5 | 10.3165979 | 265.5 | 7.95319477 | 265.5 | 7.40402226 | | | 265.50 | 7.28634244 | | | 265.5 | 7.28634244 |
| 265.6 | 2.42224304 | 265.6 | 10.3165979 | 265.6 | 10.3165979 | 265.6 | 7.93358147 | 265.6 | 5.87418455 | | | 265.60 | 7.26672913 | | | 265.6 | 7.26672913 |

| | | | | | | | | | | | | | | | | | |
|---|---|---|---|---|---|---|---|---|---|---|---|---|---|---|---|---|---|
| 265.7 | 2.42224304 | 265.7 | 10.3264046 | 265.7 | 10.3264046 | 265.7 | 7.93358147 | 265.7 | 5.26617212 | | | 265.70 | 7.30595574 | | | 265.7 | 7.30595574 |
| 265.8 | 2.4320497 | 265.8 | 10.3264046 | 265.8 | 10.3264046 | 265.8 | 7.94338812 | 265.8 | 4.76603287 | | | 265.80 | 7.29614909 | | | 265.8 | 7.29614909 |
| 265.9 | 2.44185635 | 265.9 | 10.3165979 | 265.9 | 10.3165979 | 265.9 | 7.92377482 | 265.9 | 4.36396014 | | | 265.90 | 7.31576239 | | | 265.9 | 7.31576239 |
| 266 | 2.4320497 | 266 | 10.3165979 | 266 | 10.3165979 | 266 | 7.91396816 | 266 | 4.01092067 | | | 266.00 | 7.32556904 | | | 266 | 7.32556904 |
| 266.1 | 2.44185635 | 266.1 | 10.3362112 | 266.1 | 10.3362112 | 266.1 | 7.90416151 | 266.1 | 3.80498098 | | | 266.10 | 7.3353757 | | | 266.1 | 7.3353757 |
| 266.2 | 2.451663 | 266.2 | 10.3460179 | 266.2 | 10.3460179 | 266.2 | 7.92377482 | 266.2 | 3.59904128 | | | 266.20 | 7.3353757 | | | 266.2 | 7.3353757 |
| 266.3 | 2.42224304 | 266.3 | 10.3558245 | 266.3 | 10.3558245 | 266.3 | 7.94338812 | 266.3 | 3.4323282 | | | 266.30 | 7.31576239 | | | 266.3 | 7.31576239 |
| 266.4 | 2.451663 | 266.4 | 10.3656312 | 266.4 | 10.3656312 | 266.4 | 7.96300142 | 266.4 | 3.30484172 | | | 266.40 | 7.3353757 | | | 266.4 | 7.3353757 |
| 266.5 | 2.48108296 | 266.5 | 10.3558245 | 266.5 | 10.3558245 | 266.5 | 7.96300142 | 266.5 | 3.24600181 | | | 266.50 | 7.354989 | | | 266.5 | 7.354989 |
| 266.6 | 2.4712763 | 266.6 | 10.3558245 | 266.6 | 10.3558245 | 266.6 | 7.98261473 | 266.6 | 3.17735525 | | | 266.60 | 7.31576239 | | | 266.6 | 7.31576239 |
| 266.7 | 2.4712763 | 266.7 | 10.3852445 | 266.7 | 10.3852445 | 266.7 | 7.99242138 | 266.7 | 3.11851534 | | | 266.70 | 7.32556904 | | | 266.7 | 7.32556904 |
| 266.8 | 2.50069626 | 266.8 | 10.3852445 | 266.8 | 10.3852445 | 266.8 | 7.99242138 | 266.8 | 2.97141556 | | | 266.80 | 7.354989 | | | 266.8 | 7.354989 |
| 266.9 | 2.49088961 | 266.9 | 10.3754378 | 266.9 | 10.3754378 | 266.9 | 8.00222803 | 266.9 | 2.77528252 | | | 266.90 | 7.354989 | | | 266.9 | 7.354989 |
| 267 | 2.4712763 | 267 | 10.3852445 | 267 | 10.3852445 | 267 | 7.99242138 | 267 | 2.52030956 | | | 267.00 | 7.34518235 | | | 267 | 7.34518235 |
| 267.1 | 2.49088961 | 267.1 | 10.3852445 | 267.1 | 10.3852445 | 267.1 | 7.99242138 | 267.1 | 2.33398318 | | | 267.10 | 7.354989 | | | 267.1 | 7.354989 |
| 267.2 | 2.48108296 | 267.2 | 10.4048578 | 267.2 | 10.4048578 | 267.2 | 7.96300142 | 267.2 | 2.09862353 | | | 267.20 | 7.34518235 | | | 267.2 | 7.34518235 |
| 267.3 | 2.48108296 | 267.3 | 10.4146644 | 267.3 | 10.4146644 | 267.3 | 7.97280808 | 267.3 | 1.88287718 | | | 267.30 | 7.354989 | | | 267.3 | 7.354989 |
| 267.4 | 2.49088961 | 267.4 | 10.4342777 | 267.4 | 10.4342777 | 267.4 | 7.97280808 | 267.4 | 1.72597075 | | | 267.40 | 7.34518235 | | | 267.4 | 7.34518235 |
| 267.5 | 2.49088961 | 267.5 | 10.4636977 | 267.5 | 10.4636977 | 267.5 | 7.95319477 | 267.5 | 1.56906432 | | | 267.50 | 7.34518235 | | | 267.5 | 7.34518235 |
| 267.6 | 2.49088961 | 267.6 | 10.4048578 | 267.6 | 10.4048578 | 267.6 | 7.95319477 | 267.6 | 1.38273793 | | | 267.60 | 7.34518235 | | | 267.6 | 7.34518235 |
| 267.7 | 2.4712763 | 267.7 | 10.4146644 | 267.7 | 10.4146644 | 267.7 | 8.00222803 | 267.7 | 1.16699159 | | | 267.70 | 7.32556904 | | | 267.7 | 7.32556904 |
| 267.8 | 2.48108296 | 267.8 | 10.4342777 | 267.8 | 10.4342777 | 267.8 | 7.99242138 | 267.8 | 1.01989181 | | | 267.80 | 7.3353757 | | | 267.8 | 7.3353757 |
| 267.9 | 2.49088961 | 267.9 | 10.4244711 | 267.9 | 10.4244711 | 267.9 | 8.01203468 | 267.9 | 0.89240533 | | | 267.90 | 7.36479565 | | | 267.9 | 7.36479565 |
| 268 | 2.49088961 | 268 | 10.4244711 | 268 | 10.4244711 | 268 | 8.01203468 | 268 | 0.60801242 | | | 268.00 | 7.36479565 | | | 268 | 7.36479565 |
| 268.1 | 2.50069626 | 268.1 | 10.4636977 | 268.1 | 10.4636977 | 268.1 | 8.00222803 | 268.1 | 0.50013925 | | | 268.10 | 7.38440896 | | | 268.1 | 7.38440896 |
| 268.2 | 2.50069626 | 268.2 | 10.4931176 | 268.2 | 10.4931176 | 268.2 | 8.00222803 | 268.2 | 0.38245943 | | | 268.20 | 7.3746023 | | | 268.2 | 7.3746023 |
| 268.3 | 2.50069626 | 268.3 | 10.483311 | 268.3 | 10.483311 | 268.3 | 7.99242138 | 268.3 | 0.31381286 | | | 268.30 | 7.36479565 | | | 268.3 | 7.36479565 |
| 268.4 | 2.52030956 | 268.4 | 10.4735043 | 268.4 | 10.4735043 | 268.4 | 8.01203468 | 268.4 | 0.25497295 | | | 268.40 | 7.354989 | | | 268.4 | 7.354989 |
| 268.5 | 2.51050291 | 268.5 | 10.4735043 | 268.5 | 10.4735043 | 268.5 | 8.02184134 | 268.5 | 0.16671308 | | | 268.50 | 7.36479565 | | | 268.5 | 7.36479565 |
| 268.6 | 2.50069626 | 268.6 | 10.4931176 | 268.6 | 10.4931176 | 268.6 | 8.03164799 | 268.6 | 0.12748648 | | | 268.60 | 7.3746023 | | | 268.6 | 7.3746023 |
| 268.7 | 2.50069626 | 268.7 | 10.5029243 | 268.7 | 10.5029243 | 268.7 | 8.01203468 | 268.7 | 0.06864656 | | | 268.70 | 7.38440896 | | | 268.7 | 7.38440896 |
| 268.8 | 2.50069626 | 268.8 | 10.483311 | 268.8 | 10.483311 | 268.8 | 8.00222803 | 268.8 | 0.02941996 | | | 268.80 | 7.38440896 | | | 268.8 | 7.38440896 |
| 268.9 | 2.53011622 | 268.9 | 10.4931176 | 268.9 | 10.4931176 | 268.9 | 8.04145464 | 268.9 | 0.00980665 | | | 268.90 | 7.40402226 | | | 268.9 | 7.40402226 |
| 269 | 2.52030956 | 269 | 10.5323442 | 269 | 10.5323442 | 269 | 8.04145464 | 269 | -0.00980665 | | | 269.00 | 7.38440896 | | | 269 | 7.38440896 |
| 269.1 | 2.53011622 | 269.1 | 10.5323442 | 269.1 | 10.5323442 | 269.1 | 8.05126129 | 269.1 | -0.12748648 | | | 269.10 | 7.3746023 | | | 269.1 | 7.3746023 |
| 269.2 | 2.52030956 | 269.2 | 10.5225376 | 269.2 | 10.5225376 | 269.2 | 8.0708746 | 269.2 | -0.17651974 | | | 269.20 | 7.354989 | | | 269.2 | 7.354989 |
| 269.3 | 2.53011622 | 269.3 | 10.5421509 | 269.3 | 10.5421509 | 269.3 | 8.0904879 | 269.3 | -0.18632639 | | | 269.30 | 7.36479565 | | | 269.3 | 7.36479565 |
| 269.4 | 2.53011622 | 269.4 | 10.5421509 | 269.4 | 10.5421509 | 269.4 | 8.1101012 | 269.4 | -0.18632639 | | | 269.40 | 7.39421561 | | | 269.4 | 7.39421561 |
| 269.5 | 2.52030956 | 269.5 | 10.5225376 | 269.5 | 10.5225376 | 269.5 | 8.0904879 | 269.5 | -0.15690643 | | | 269.50 | 7.39421561 | | | 269.5 | 7.39421561 |
| 269.6 | 2.52030956 | 269.6 | 10.5421509 | 269.6 | 10.5421509 | 269.6 | 8.0708746 | 269.6 | -0.18632639 | | | 269.60 | 7.38440896 | | | 269.6 | 7.38440896 |
| 269.7 | 2.53992287 | 269.7 | 10.5323442 | 269.7 | 10.5323442 | 269.7 | 8.0708746 | 269.7 | -0.23535965 | | | 269.70 | 7.41382891 | | | 269.7 | 7.41382891 |
| 269.8 | 2.55953617 | 269.8 | 10.5421509 | 269.8 | 10.5421509 | 269.8 | 8.0904879 | 269.8 | -0.2647796 | | | 269.80 | 7.40402226 | | | 269.8 | 7.40402226 |
| 269.9 | 2.53992287 | 269.9 | 10.5519576 | 269.9 | 10.5519576 | 269.9 | 8.08068125 | 269.9 | -0.2647796 | | | 269.90 | 7.39421561 | | | 269.9 | 7.39421561 |
| 270 | 2.53992287 | 270 | 10.5911842 | 270 | 10.5911842 | 270 | 8.06106794 | 270 | -0.29419956 | | | 270.00 | 7.40402226 | | | 270 | 7.40402226 |
| 270.1 | 2.53011622 | 270.1 | 10.5813775 | 270.1 | 10.5813775 | 270.1 | 8.08068125 | 270.1 | -0.30400621 | | | 270.10 | 7.42363556 | | | 270.1 | 7.42363556 |
| 270.2 | 2.53011622 | 270.2 | 10.6304108 | 270.2 | 10.6304108 | 270.2 | 8.10029455 | 270.2 | -0.28439291 | | | 270.20 | 7.40402226 | | | 270.2 | 7.40402226 |
| 270.3 | 2.54972952 | 270.3 | 10.6206041 | 270.3 | 10.6206041 | 270.3 | 8.08068125 | 270.3 | -0.29419956 | | | 270.30 | 7.39421561 | | | 270.3 | 7.39421561 |
| 270.4 | 2.55953617 | 270.4 | 10.6107975 | 270.4 | 10.6107975 | 270.4 | 8.08068125 | 270.4 | -0.33342617 | | | 270.40 | 7.39421561 | | | 270.4 | 7.39421561 |
| 270.5 | 2.55953617 | 270.5 | 10.5911842 | 270.5 | 10.5911842 | 270.5 | 8.13952116 | 270.5 | -0.31381286 | | | 270.50 | 7.39421561 | | | 270.5 | 7.39421561 |
| 270.6 | 2.56934282 | 270.6 | 10.5813775 | 270.6 | 10.5813775 | 270.6 | 8.13952116 | | | | | 270.60 | 7.38440896 | | | 270.6 | 7.38440896 |
| 270.7 | 2.55953617 | 270.7 | 10.6304108 | 270.7 | 10.6304108 | 270.7 | 8.13952116 | | | | | 270.70 | 7.39421561 | | | 270.7 | 7.39421561 |
| 270.8 | 2.55953617 | 270.8 | 10.6304108 | 270.8 | 10.6304108 | 270.8 | 8.16894112 | | | | | 270.80 | 7.42363556 | | | 270.8 | 7.42363556 |
| 270.9 | 2.59876278 | 270.9 | 10.6009908 | 270.9 | 10.6009908 | 270.9 | 8.19836107 | | | | | 270.90 | 7.41382891 | | | 270.9 | 7.41382891 |
| 271 | 2.58895613 | 271 | 10.6206041 | 271 | 10.6206041 | 271 | 8.17874777 | | | | | 271.00 | 7.38440896 | | | 271 | 7.38440896 |
| 271.1 | 2.58895613 | 271.1 | 10.6402174 | 271.1 | 10.6402174 | 271.1 | 8.18855442 | | | | | 271.10 | 7.39421561 | | | 271.1 | 7.39421561 |
| 271.2 | 2.58895613 | 271.2 | 10.6500241 | 271.2 | 10.6500241 | 271.2 | 8.19836107 | | | | | 271.20 | 7.43344222 | | | 271.2 | 7.43344222 |
| 271.3 | 2.59876278 | 271.3 | 10.6402174 | 271.3 | 10.6402174 | 271.3 | 8.21797438 | | | | | 271.30 | 7.42363556 | | | 271.3 | 7.42363556 |
| 271.4 | 2.59876278 | 271.4 | 10.6500241 | 271.4 | 10.6500241 | 271.4 | 8.22778103 | | | | | 271.40 | 7.43344222 | | | 271.4 | 7.43344222 |
| 271.5 | 2.58895613 | 271.5 | 10.6696374 | 271.5 | 10.6696374 | 271.5 | 8.22778103 | | | | | 271.50 | 7.43344222 | | | 271.5 | 7.43344222 |
| 271.6 | 2.59876278 | 271.6 | 10.6598307 | 271.6 | 10.6598307 | 271.6 | 8.21797438 | | | | | 271.60 | 7.44324887 | | | 271.6 | 7.44324887 |
| 271.7 | 2.61837608 | 271.7 | 10.6304108 | 271.7 | 10.6304108 | 271.7 | 8.25720098 | | | | | 271.70 | 7.43344222 | | | 271.7 | 7.43344222 |

| | | | | | | | | | | | | | | | | | |
|---|---|---|---|---|---|---|---|---|---|---|---|---|---|---|---|---|---|
| 271.8 | 2.61837608 | 271.8 | 10.6598307 | 271.8 | 10.6598307 | 271.8 | 8.25720098 | | | | | 271.80 | 7.40402226 | | | 271.8 | 7.40402226 |
| 271.9 | 2.59876278 | 271.9 | 10.6696374 | 271.9 | 10.6696374 | 271.9 | 8.21797438 | | | | | 271.90 | 7.42363556 | | | 271.9 | 7.42363556 |
| 272 | 2.61837608 | 272 | 10.6598307 | 272 | 10.6598307 | 272 | 8.22778103 | | | | | 272.00 | 7.43344222 | | | 272 | 7.43344222 |
| 272.1 | 2.60856943 | 272.1 | 10.6696374 | 272.1 | 10.6696374 | 272.1 | 8.24739433 | | | | | 272.10 | 7.44324887 | | | 272.1 | 7.44324887 |
| 272.2 | 2.61837608 | 272.2 | 10.6892507 | 272.2 | 10.6892507 | 272.2 | 8.23758768 | | | | | 272.20 | 7.43344222 | | | 272.2 | 7.43344222 |
| 272.3 | 2.60856943 | 272.3 | 10.679444 | 272.3 | 10.679444 | 272.3 | 8.23758768 | | | | | 272.30 | 7.41382891 | | | 272.3 | 7.41382891 |
| 272.4 | 2.60856943 | 272.4 | 10.708864 | 272.4 | 10.708864 | 272.4 | 8.25720098 | | | | | 272.40 | 7.43344222 | | | 272.4 | 7.43344222 |
| 272.5 | 2.60856943 | 272.5 | 10.7186706 | 272.5 | 10.7186706 | 272.5 | 8.29642759 | | | | | 272.50 | 7.45305552 | | | 272.5 | 7.45305552 |
| 272.6 | 2.62818274 | 272.6 | 10.708864 | 272.6 | 10.708864 | 272.6 | 8.27681429 | | | | | 272.60 | 7.42363556 | | | 272.6 | 7.42363556 |
| 272.7 | 2.63798939 | 272.7 | 10.6892507 | 272.7 | 10.6892507 | 272.7 | 8.26700764 | | | | | 272.70 | 7.41382891 | | | 272.7 | 7.41382891 |
| 272.8 | 2.62818274 | 272.8 | 10.6892507 | 272.8 | 10.6892507 | 272.8 | 8.28662094 | | | | | 272.80 | 7.44324887 | | | 272.8 | 7.44324887 |
| 272.9 | 2.64779604 | 272.9 | 10.708864 | 272.9 | 10.708864 | 272.9 | 8.29642759 | | | | | 272.90 | 7.46286217 | | | 272.9 | 7.46286217 |
| 273 | 2.64779604 | 273 | 10.7186706 | 273 | 10.7186706 | 273 | 8.27681429 | | | | | 273.00 | 7.46286217 | | | 273 | 7.46286217 |
| 273.1 | 2.64779604 | 273.1 | 10.7186706 | 273.1 | 10.7186706 | 273.1 | 8.27681429 | | | | | 273.10 | 7.45305552 | | | 273.1 | 7.45305552 |
| 273.2 | 2.63798939 | 273.2 | 10.7284773 | 273.2 | 10.7284773 | 273.2 | 8.3160409 | | | | | 273.20 | 7.44324887 | | | 273.2 | 7.44324887 |
| 273.3 | 2.65760269 | 273.3 | 10.7382839 | 273.3 | 10.7382839 | 273.3 | 8.3552675 | | | | | 273.30 | 7.43344222 | | | 273.3 | 7.43344222 |
| 273.4 | 2.64779604 | 273.4 | 10.7677039 | 273.4 | 10.7677039 | 273.4 | 8.36507416 | | | | | 273.40 | 7.45305552 | | | 273.4 | 7.45305552 |
| 273.5 | 2.65760269 | 273.5 | 10.7578972 | 273.5 | 10.7578972 | 273.5 | 8.3552675 | | | | | 273.50 | 7.46286217 | | | 273.5 | 7.46286217 |
| 273.6 | 2.66740934 | 273.6 | 10.7480906 | 273.6 | 10.7480906 | 273.6 | 8.34546085 | | | | | 273.60 | 7.47266882 | | | 273.6 | 7.47266882 |
| 273.7 | 2.65760269 | 273.7 | 10.7578972 | 273.7 | 10.7578972 | 273.7 | 8.32584755 | | | | | 273.70 | 7.46286217 | | | 273.7 | 7.46286217 |
| 273.8 | 2.66740934 | 273.8 | 10.7873172 | 273.8 | 10.7873172 | 273.8 | 8.29642759 | | | | | 273.80 | 7.46286217 | | | 273.8 | 7.46286217 |
| 273.9 | 2.6968293 | 273.9 | 10.7775105 | 273.9 | 10.7775105 | 273.9 | 8.3356542 | | | | | 273.90 | 7.46286217 | | | 273.9 | 7.46286217 |
| 274 | 2.6968293 | 274 | 10.8069305 | 274 | 10.8069305 | 274 | 8.3552675 | | | | | 274.00 | 7.50208878 | | | 274 | 7.50208878 |
| 274.1 | 2.68702265 | 274.1 | 10.8167372 | 274.1 | 10.8167372 | 274.1 | 8.3552675 | | | | | 274.10 | 7.50208878 | | | 274.1 | 7.50208878 |
| 274.2 | 2.677216 | 274.2 | 10.8069305 | 274.2 | 10.8069305 | 274.2 | 8.38468746 | | | | | 274.20 | 7.48247548 | | | 274.2 | 7.48247548 |
| 274.3 | 2.68702265 | 274.3 | 10.7971239 | 274.3 | 10.7971239 | 274.3 | 8.38468746 | | | | | 274.30 | 7.48247548 | | | 274.3 | 7.48247548 |
| 274.4 | 2.6968293 | 274.4 | 10.7971239 | 274.4 | 10.7971239 | 274.4 | 8.38468746 | | | | | 274.40 | 7.48247548 | | | 274.4 | 7.48247548 |
| 274.5 | 2.677216 | 274.5 | 10.8069305 | 274.5 | 10.8069305 | 274.5 | 8.36507416 | | | | | 274.50 | 7.48247548 | | | 274.5 | 7.48247548 |
| 274.6 | 2.6968293 | 274.6 | 10.7971239 | 274.6 | 10.7971239 | 274.6 | 8.3552675 | | | | | 274.60 | 7.48247548 | | | 274.6 | 7.48247548 |
| 274.7 | 2.70663595 | 274.7 | 10.7873172 | 274.7 | 10.7873172 | 274.7 | 8.37488081 | | | | | 274.70 | 7.49228213 | | | 274.7 | 7.49228213 |
| 274.8 | 2.677216 | 274.8 | 10.8069305 | 274.8 | 10.8069305 | 274.8 | 8.3552675 | | | | | 274.80 | 7.49228213 | | | 274.8 | 7.49228213 |
| 274.9 | 2.65760269 | 274.9 | 10.8265438 | 274.9 | 10.8265438 | 274.9 | 8.40430076 | | | | | 274.90 | 7.48247548 | | | 274.9 | 7.48247548 |
| 275 | 2.66740934 | 275 | 10.8363505 | 275 | 10.8363505 | 275 | 8.39449411 | | | | | 275.00 | 7.48247548 | | | 275 | 7.48247548 |
| 275.1 | 2.68702265 | 275.1 | 10.8363505 | 275.1 | 10.8363505 | 275.1 | 8.37488081 | | | | | 275.10 | 7.50208878 | | | 275.1 | 7.50208878 |
| 275.2 | 2.7164426 | 275.2 | 10.8461571 | 275.2 | 10.8461571 | 275.2 | 8.42391407 | | | | | 275.20 | 7.49228213 | | | 275.2 | 7.49228213 |
| 275.3 | 2.6968293 | 275.3 | 10.8657704 | 275.3 | 10.8657704 | 275.3 | 8.42391407 | | | | | 275.30 | 7.48247548 | | | 275.3 | 7.48247548 |
| 275.4 | 2.6968293 | 275.4 | 10.8559638 | 275.4 | 10.8559638 | 275.4 | 8.41410742 | | | | | 275.40 | 7.48247548 | | | 275.4 | 7.48247548 |
| 275.5 | 2.7164426 | 275.5 | 10.8461571 | 275.5 | 10.8461571 | 275.5 | 8.40430076 | | | | | 275.50 | 7.49228213 | | | 275.5 | 7.49228213 |
| 275.6 | 2.72624926 | 275.6 | 10.8363505 | 275.6 | 10.8363505 | 275.6 | 8.41410742 | | | | | 275.60 | 7.48247548 | | | 275.6 | 7.48247548 |
| 275.7 | 2.73605591 | 275.7 | 10.8461571 | 275.7 | 10.8461571 | 275.7 | 8.40430076 | | | | | 275.70 | 7.46286217 | | | 275.7 | 7.46286217 |
| 275.8 | 2.72624926 | 275.8 | 10.8755771 | 275.8 | 10.8755771 | 275.8 | 8.39449411 | | | | | 275.80 | 7.48247548 | | | 275.8 | 7.48247548 |
| 275.9 | 2.72624926 | 275.9 | 10.8853837 | 275.9 | 10.8853837 | 275.9 | 8.43372072 | | | | | 275.90 | 7.49228213 | | | 275.9 | 7.49228213 |
| 276 | 2.73605591 | 276 | 10.904997 | 276 | 10.904997 | 276 | 8.46314068 | | | | | 276.00 | 7.48247548 | | | 276 | 7.48247548 |
| 276.1 | 2.73605591 | 276.1 | 10.904997 | 276.1 | 10.904997 | 276.1 | 8.47294733 | | | | | 276.10 | 7.47266882 | | | 276.1 | 7.47266882 |
| 276.2 | 2.72624926 | 276.2 | 10.8951904 | 276.2 | 10.8951904 | 276.2 | 8.45333402 | | | | | 276.20 | 7.47266882 | | | 276.2 | 7.47266882 |
| 276.3 | 2.72624926 | 276.3 | 10.8951904 | 276.3 | 10.8951904 | 276.3 | 8.45333402 | | | | | 276.30 | 7.50208878 | | | 276.3 | 7.50208878 |
| 276.4 | 2.75566921 | 276.4 | 10.9148037 | 276.4 | 10.9148037 | 276.4 | 8.45333402 | | | | | 276.40 | 7.50208878 | | | 276.4 | 7.50208878 |
| 276.5 | 2.76547586 | 276.5 | 10.9148037 | 276.5 | 10.9148037 | 276.5 | 8.45333402 | | | | | 276.50 | 7.49228213 | | | 276.5 | 7.49228213 |
| 276.6 | 2.74586256 | 276.6 | 10.9246103 | 276.6 | 10.9246103 | 276.6 | 8.45333402 | | | | | 276.60 | 7.53150874 | | | 276.6 | 7.53150874 |
| 276.7 | 2.73605591 | 276.7 | 10.934417 | 276.7 | 10.934417 | 276.7 | 8.40430076 | | | | | 276.70 | 7.51189543 | | | 276.7 | 7.51189543 |
| 276.8 | 2.75566921 | 276.8 | 10.9246103 | 276.8 | 10.9246103 | 276.8 | 8.40430076 | | | | | 276.80 | 7.49228213 | | | 276.8 | 7.49228213 |
| 276.9 | 2.75566921 | 276.9 | 10.9246103 | 276.9 | 10.9246103 | 276.9 | 8.38468746 | | | | | 276.90 | 7.51189543 | | | 276.9 | 7.51189543 |
| 277 | 2.75566921 | 277 | 10.9246103 | 277 | 10.9246103 | 277 | 8.38468746 | | | | | 277.00 | 7.49228213 | | | 277 | 7.49228213 |
| 277.1 | 2.74586256 | 277.1 | 10.934417 | 277.1 | 10.934417 | 277.1 | 8.39449411 | | | | | 277.10 | 7.50208878 | | | 277.1 | 7.50208878 |
| 277.2 | 2.74586256 | 277.2 | 10.9442236 | 277.2 | 10.9442236 | 277.2 | 8.42391407 | | | | | 277.20 | 7.50208878 | | | 277.2 | 7.50208878 |
| 277.3 | 2.76547586 | 277.3 | 10.9442236 | 277.3 | 10.9442236 | 277.3 | 8.41410742 | | | | | 277.30 | 7.51189543 | | | 277.3 | 7.51189543 |
| 277.4 | 2.78508917 | 277.4 | 10.9442236 | 277.4 | 10.9442236 | 277.4 | 8.39449411 | | | | | 277.40 | 7.53150874 | | | 277.4 | 7.53150874 |
| 277.5 | 2.79489582 | 277.5 | 10.9638369 | 277.5 | 10.9638369 | 277.5 | 8.42391407 | | | | | 277.50 | 7.54131539 | | | 277.5 | 7.54131539 |
| 277.6 | 2.79489582 | 277.6 | 10.9638369 | 277.6 | 10.9638369 | 277.6 | 8.43372072 | | | | | 277.60 | 7.57073534 | | | 277.6 | 7.57073534 |
| 277.7 | 2.79489582 | 277.7 | 10.9638369 | 277.7 | 10.9638369 | 277.7 | 8.38468746 | | | | | 277.70 | 7.56092869 | | | 277.7 | 7.56092869 |
| 277.8 | 2.79489582 | 277.8 | 10.9834502 | 277.8 | 10.9834502 | 277.8 | 8.41410742 | | | | | 277.80 | 7.55112204 | | | 277.8 | 7.55112204 |

| | | | | | | | | | | | | | | | | | |
|---|---|---|---|---|---|---|---|---|---|---|---|---|---|---|---|---|---|
| 277.9 | 2.79489582 | 277.9 | 10.9932569 | 277.9 | 10.9932569 | 277.9 | 8.43372072 | | | | | 277.90 | 7.53150874 | | | 277.9 | 7.53150874 |
| 278 | 2.78508917 | 278 | 11.0030635 | 278 | 11.0030635 | 278 | 8.43372072 | | | | | 278.00 | 7.53150874 | | | 278 | 7.53150874 |
| 278.1 | 2.78508917 | 278.1 | 11.0030635 | 278.1 | 11.0030635 | 278.1 | 8.45333402 | | | | | 278.10 | 7.56092869 | | | 278.1 | 7.56092869 |
| 278.2 | 2.78508917 | 278.2 | 11.0128702 | 278.2 | 11.0128702 | 278.2 | 8.42391407 | | | | | 278.20 | 7.55112204 | | | 278.2 | 7.55112204 |
| 278.3 | 2.79489582 | 278.3 | 11.0128702 | 278.3 | 11.0128702 | 278.3 | 8.41410742 | | | | | 278.30 | 7.53150874 | | | 278.3 | 7.53150874 |
| 278.4 | 2.79489582 | 278.4 | 10.9932569 | 278.4 | 10.9932569 | 278.4 | 8.43372072 | | | | | 278.40 | 7.52170208 | | | 278.4 | 7.52170208 |
| 278.5 | 2.80470247 | 278.5 | 11.0324835 | 278.5 | 11.0324835 | 278.5 | 8.46314068 | | | | | 278.50 | 7.53150874 | | | 278.5 | 7.53150874 |
| 278.6 | 2.82431578 | 278.6 | 11.0422902 | 278.6 | 11.0422902 | 278.6 | 8.48275398 | | | | | 278.60 | 7.55112204 | | | 278.6 | 7.55112204 |
| 278.7 | 2.82431578 | 278.7 | 11.0422902 | 278.7 | 11.0422902 | 278.7 | 8.47294733 | | | | | 278.70 | 7.55112204 | | | 278.7 | 7.55112204 |
| 278.8 | 2.81450912 | 278.8 | 11.0422902 | 278.8 | 11.0422902 | 278.8 | 8.46314068 | | | | | 278.80 | 7.55112204 | | | 278.8 | 7.55112204 |
| 278.9 | 2.81450912 | 278.9 | 11.0324835 | 278.9 | 11.0324835 | 278.9 | 8.45333402 | | | | | 278.90 | 7.54131539 | | | 278.9 | 7.54131539 |
| 279 | 2.82431578 | 279 | 11.0324835 | 279 | 11.0324835 | 279 | 8.47294733 | | | | | 279.00 | 7.56092869 | | | 279 | 7.56092869 |
| 279.1 | 2.84392908 | 279.1 | 11.0226768 | 279.1 | 11.0226768 | 279.1 | 8.48275398 | | | | | 279.10 | 7.56092869 | | | 279.1 | 7.56092869 |
| 279.2 | 2.84392908 | 279.2 | 11.0520968 | 279.2 | 11.0520968 | 279.2 | 8.45333402 | | | | | 279.20 | 7.56092869 | | | 279.2 | 7.56092869 |
| 279.3 | 2.85373573 | 279.3 | 11.0717101 | 279.3 | 11.0717101 | 279.3 | 8.47294733 | | | | | 279.30 | 7.56092869 | | | 279.3 | 7.56092869 |
| 279.4 | 2.86354238 | 279.4 | 11.0520968 | 279.4 | 11.0520968 | 279.4 | 8.50236728 | | | | | 279.40 | 7.59034865 | | | 279.4 | 7.59034865 |
| 279.5 | 2.86354238 | 279.5 | 11.0717101 | 279.5 | 11.0717101 | 279.5 | 8.51217394 | | | | | 279.50 | 7.59034865 | | | 279.5 | 7.59034865 |
| 279.6 | 2.84392908 | 279.6 | 11.1011301 | 279.6 | 11.1011301 | 279.6 | 8.50236728 | | | | | 279.60 | 7.6001553 | | | 279.6 | 7.6001553 |
| 279.7 | 2.84392908 | 279.7 | 11.1109367 | 279.7 | 11.1109367 | 279.7 | 8.47294733 | | | | | 279.70 | 7.580542 | | | 279.7 | 7.580542 |
| 279.8 | 2.87334904 | 279.8 | 11.1011301 | 279.8 | 11.1011301 | 279.8 | 8.47294733 | | | | | 279.80 | 7.580542 | | | 279.8 | 7.580542 |
| 279.9 | 2.87334904 | 279.9 | 11.0913234 | 279.9 | 11.0913234 | 279.9 | 8.49256063 | | | | | 279.90 | 7.6001553 | | | 279.9 | 7.6001553 |
| 280 | 2.86354238 | 280 | 11.1109367 | 280 | 11.1109367 | 280 | 8.51217394 | | | | | 280.00 | 7.59034865 | | | 280 | 7.59034865 |
| 280.1 | 2.87334904 | 280.1 | 11.1011301 | 280.1 | 11.1011301 | 280.1 | 8.50236728 | | | | | 280.10 | 7.60996195 | | | 280.1 | 7.60996195 |
| 280.2 | 2.86354238 | 280.2 | 11.1011301 | 280.2 | 11.1011301 | 280.2 | 8.54159389 | | | | | 280.20 | 7.6197686 | | | 280.2 | 7.6197686 |
| 280.3 | 2.86354238 | 280.3 | 11.13055 | 280.3 | 11.13055 | 280.3 | 8.5612072 | | | | | 280.30 | 7.60996195 | | | 280.3 | 7.60996195 |
| 280.4 | 2.88315569 | 280.4 | 11.1207434 | 280.4 | 11.1207434 | 280.4 | 8.52198059 | | | | | 280.40 | 7.6001553 | | | 280.4 | 7.6001553 |
| 280.5 | 2.87334904 | 280.5 | 11.1011301 | 280.5 | 11.1011301 | 280.5 | 8.53178724 | | | | | 280.50 | 7.60996195 | | | 280.5 | 7.60996195 |
| 280.6 | 2.88315569 | 280.6 | 11.1207434 | 280.6 | 11.1207434 | 280.6 | 8.57101385 | | | | | 280.60 | 7.59034865 | | | 280.6 | 7.59034865 |
| 280.7 | 2.88315569 | 280.7 | 11.13055 | 280.7 | 11.13055 | 280.7 | 8.5612072 | | | | | 280.70 | 7.6001553 | | | 280.7 | 7.6001553 |
| 280.8 | 2.89296234 | 280.8 | 11.15997 | 280.8 | 11.15997 | 280.8 | 8.53178724 | | | | | 280.80 | 7.60996195 | | | 280.8 | 7.60996195 |
| 280.9 | 2.89296234 | 280.9 | 11.1109367 | 280.9 | 11.1109367 | 280.9 | 8.53178724 | | | | | 280.90 | 7.57073534 | | | 280.9 | 7.57073534 |
| 281 | 2.90276899 | 281 | 11.1109367 | 281 | 11.1109367 | 281 | 8.57101385 | | | | | 281.00 | 7.57073534 | | | 281 | 7.57073534 |
| 281.1 | 2.9223823 | 281.1 | 11.1403567 | 281.1 | 11.1403567 | 281.1 | 8.5808205 | | | | | 281.10 | 7.59034865 | | | 281.1 | 7.59034865 |
| 281.2 | 2.90276899 | 281.2 | 11.1501633 | 281.2 | 11.1501633 | 281.2 | 8.59062715 | | | | | 281.20 | 7.59034865 | | | 281.2 | 7.59034865 |
| 281.3 | 2.91257564 | 281.3 | 11.1697766 | 281.3 | 11.1697766 | 281.3 | 8.61024046 | | | | | 281.30 | 7.59034865 | | | 281.3 | 7.59034865 |
| 281.4 | 2.91257564 | 281.4 | 11.1697766 | 281.4 | 11.1697766 | 281.4 | 8.63966041 | | | | | 281.40 | 7.59034865 | | | 281.4 | 7.59034865 |
| 281.5 | 2.90276899 | 281.5 | 11.1795833 | 281.5 | 11.1795833 | 281.5 | 8.65927372 | | | | | 281.50 | 7.57073534 | | | 281.5 | 7.57073534 |
| 281.6 | 2.90276899 | 281.6 | 11.15997 | 281.6 | 11.15997 | 281.6 | 8.62004711 | | | | | 281.60 | 7.59034865 | | | 281.6 | 7.59034865 |
| 281.7 | 2.89296234 | 281.7 | 11.2090032 | 281.7 | 11.2090032 | 281.7 | 8.6004338 | | | | | 281.70 | 7.60996195 | | | 281.7 | 7.60996195 |
| 281.8 | 2.91257564 | 281.8 | 11.2090032 | 281.8 | 11.2090032 | 281.8 | 8.64946706 | | | | | 281.80 | 7.6197686 | | | 281.8 | 7.6197686 |
| 281.9 | 2.9223823 | 281.9 | 11.1893899 | 281.9 | 11.1893899 | 281.9 | 8.65927372 | | | | | 281.90 | 7.6197686 | | | 281.9 | 7.6197686 |
| 282 | 2.90276899 | 282 | 11.1991966 | 282 | 11.1991966 | 282 | 8.67888702 | | | | | 282.00 | 7.60996195 | | | 282 | 7.60996195 |
| 282.1 | 2.93218895 | 282.1 | 11.1991966 | 282.1 | 11.1991966 | 282.1 | 8.66908037 | | | | | 282.10 | 7.59034865 | | | 282.1 | 7.59034865 |
| 282.2 | 2.9419956 | 282.2 | 11.2286165 | 282.2 | 11.2286165 | 282.2 | 8.65927372 | | | | | 282.20 | 7.60996195 | | | 282.2 | 7.60996195 |
| 282.3 | 2.9419956 | 282.3 | 11.2482298 | 282.3 | 11.2482298 | 282.3 | 8.67888702 | | | | | 282.30 | 7.63938191 | | | 282.3 | 7.63938191 |
| 282.4 | 2.93218895 | 282.4 | 11.2580365 | 282.4 | 11.2580365 | 282.4 | 8.69850032 | | | | | 282.40 | 7.63938191 | | | 282.4 | 7.63938191 |
| 282.5 | 2.93218895 | 282.5 | 11.2384232 | 282.5 | 11.2384232 | 282.5 | 8.71811363 | | | | | 282.50 | 7.6197686 | | | 282.5 | 7.6197686 |
| 282.6 | 2.9419956 | 282.6 | 11.2482298 | 282.6 | 11.2482298 | 282.6 | 8.68869367 | | | | | 282.60 | 7.64918856 | | | 282.6 | 7.64918856 |
| 282.7 | 2.9223823 | 282.7 | 11.2482298 | 282.7 | 11.2482298 | 282.7 | 8.67888702 | | | | | 282.70 | 7.66880186 | | | 282.7 | 7.66880186 |
| 282.8 | 2.93218895 | 282.8 | 11.2580365 | 282.8 | 11.2580365 | 282.8 | 8.69850032 | | | | | 282.80 | 7.64918856 | | | 282.8 | 7.64918856 |
| 282.9 | 2.97141556 | 282.9 | 11.2580365 | 282.9 | 11.2580365 | 282.9 | 8.71811363 | | | | | 282.90 | 7.62957526 | | | 282.9 | 7.62957526 |
| 283 | 2.97141556 | 283 | 11.2286165 | 283 | 11.2286165 | 283 | 8.69850032 | | | | | 283.00 | 7.6197686 | | | 283 | 7.6197686 |
| 283.1 | 2.95180225 | 283.1 | 11.2188099 | 283.1 | 11.2188099 | 283.1 | 8.71811363 | | | | | 283.10 | 7.62957526 | | | 283.1 | 7.62957526 |
| 283.2 | 2.9616089 | 283.2 | 11.2874565 | 283.2 | 11.2874565 | 283.2 | 8.72792028 | | | | | 283.20 | 7.64918856 | | | 283.2 | 7.64918856 |
| 283.3 | 2.9616089 | 283.3 | 11.2776498 | 283.3 | 11.2776498 | 283.3 | 8.73772693 | | | | | 283.30 | 7.62957526 | | | 283.3 | 7.62957526 |
| 283.4 | 2.9616089 | 283.4 | 11.2874565 | 283.4 | 11.2874565 | 283.4 | 8.72792028 | | | | | 283.40 | 7.64918856 | | | 283.4 | 7.64918856 |
| 283.5 | 2.9616089 | 283.5 | 11.3070698 | 283.5 | 11.3070698 | 283.5 | 8.75734024 | | | | | 283.50 | 7.66880186 | | | 283.5 | 7.66880186 |
| 283.6 | 2.98122221 | 283.6 | 11.2972631 | 283.6 | 11.2972631 | 283.6 | 8.77695354 | | | | | 283.60 | 7.64918856 | | | 283.6 | 7.64918856 |
| 283.7 | 2.9616089 | 283.7 | 11.3070698 | 283.7 | 11.3070698 | 283.7 | 8.76714689 | | | | | 283.70 | 7.64918856 | | | 283.7 | 7.64918856 |
| 283.8 | 2.97141556 | 283.8 | 11.2678431 | 283.8 | 11.2678431 | 283.8 | 8.75734024 | | | | | 283.80 | 7.62957526 | | | 283.8 | 7.62957526 |
| 283.9 | 3.00083551 | 283.9 | 11.2776498 | 283.9 | 11.2776498 | 283.9 | 8.73772693 | | | | | 283.90 | 7.65899521 | | | 283.9 | 7.65899521 |

| | | | | | | | | | | | | | | | | | |
|---|---|---|---|---|---|---|---|---|---|---|---|---|---|---|---|---|---|
| 284 | 2.99102886 | 284 | 11.2972631 | 284 | 11.2972631 | 284 | 8.77695354 | | | | | 284.00 | 7.67860852 | | | 284 | 7.67860852 |
| 284.1 | 2.99102886 | 284.1 | 11.2972631 | 284.1 | 11.2972631 | 284.1 | 8.79656684 | | | | | 284.10 | 7.68841517 | | | 284.1 | 7.68841517 |
| 284.2 | 2.99102886 | 284.2 | 11.2972631 | 284.2 | 11.2972631 | 284.2 | 8.74753358 | | | | | 284.20 | 7.65899521 | | | 284.2 | 7.65899521 |
| 284.3 | 2.99102886 | 284.3 | 11.2972631 | 284.3 | 11.2972631 | 284.3 | 8.75734024 | | | | | 284.30 | 7.66880186 | | | 284.3 | 7.66880186 |
| 284.4 | 2.99102886 | 284.4 | 11.2874565 | 284.4 | 11.2874565 | 284.4 | 8.76714689 | | | | | 284.40 | 7.65899521 | | | 284.4 | 7.65899521 |
| 284.5 | 2.98122221 | 284.5 | 11.2874565 | 284.5 | 11.2874565 | 284.5 | 8.77695354 | | | | | 284.50 | 7.68841517 | | | 284.5 | 7.68841517 |
| 284.6 | 3.00083551 | 284.6 | 11.3266831 | 284.6 | 11.3266831 | 284.6 | 8.8063735 | | | | | 284.60 | 7.67860852 | | | 284.6 | 7.67860852 |
| 284.7 | 3.00083551 | 284.7 | 11.3364897 | 284.7 | 11.3364897 | 284.7 | 8.81618015 | | | | | 284.70 | 7.65899521 | | | 284.7 | 7.65899521 |
| 284.8 | 3.01064216 | 284.8 | 11.3364897 | 284.8 | 11.3364897 | 284.8 | 8.8259868 | | | | | 284.80 | 7.66880186 | | | 284.8 | 7.66880186 |
| 284.9 | 3.04006212 | 284.9 | 11.3462964 | 284.9 | 11.3462964 | 284.9 | 8.79656684 | | | | | 284.90 | 7.67860852 | | | 284.9 | 7.67860852 |
| 285 | 3.02044882 | 285 | 11.356103 | 285 | 11.356103 | 285 | 8.78676019 | | | | | 285.00 | 7.70802847 | | | 285 | 7.70802847 |
| 285.1 | 3.02044882 | 285.1 | 11.3364897 | 285.1 | 11.3364897 | 285.1 | 8.8063735 | | | | | 285.10 | 7.73744843 | | | 285.1 | 7.73744843 |
| 285.2 | 3.03025547 | 285.2 | 11.3659097 | 285.2 | 11.3659097 | 285.2 | 8.8456001 | | | | | 285.20 | 7.72764178 | | | 285.2 | 7.72764178 |
| 285.3 | 3.02044882 | 285.3 | 11.3757163 | 285.3 | 11.3757163 | 285.3 | 8.85540676 | | | | | 285.30 | 7.70802847 | | | 285.3 | 7.70802847 |
| 285.4 | 3.04006212 | 285.4 | 11.356103 | 285.4 | 11.356103 | 285.4 | 8.83579345 | | | | | 285.40 | 7.69822182 | | | 285.4 | 7.69822182 |
| 285.5 | 3.04006212 | 285.5 | 11.385523 | 285.5 | 11.385523 | 285.5 | 8.83579345 | | | | | 285.50 | 7.71783512 | | | 285.5 | 7.71783512 |
| 285.6 | 3.03025547 | 285.6 | 11.4051363 | 285.6 | 11.4051363 | 285.6 | 8.8259868 | | | | | 285.60 | 7.72764178 | | | 285.6 | 7.72764178 |
| 285.7 | 3.03025547 | 285.7 | 11.4051363 | 285.7 | 11.4051363 | 285.7 | 8.86521341 | | | | | 285.70 | 7.71783512 | | | 285.7 | 7.71783512 |
| 285.8 | 3.04986877 | 285.8 | 11.4051363 | 285.8 | 11.4051363 | 285.8 | 8.83579345 | | | | | 285.80 | 7.70802847 | | | 285.8 | 7.70802847 |
| 285.9 | 3.04986877 | 285.9 | 11.385523 | 285.9 | 11.385523 | 285.9 | 8.88482671 | | | | | 285.90 | 7.68841517 | | | 285.9 | 7.68841517 |
| 286 | 3.04006212 | 286 | 11.4051363 | 286 | 11.4051363 | 286 | 8.90444002 | | | | | 286.00 | 7.69822182 | | | 286 | 7.69822182 |
| 286.1 | 3.04006212 | 286.1 | 11.4149429 | 286.1 | 11.4149429 | 286.1 | 8.86521341 | | | | | 286.10 | 7.74725508 | | | 286.1 | 7.74725508 |
| 286.2 | 3.03025547 | 286.2 | 11.3953296 | 286.2 | 11.3953296 | 286.2 | 8.86521341 | | | | | 286.20 | 7.73744843 | | | 286.2 | 7.73744843 |
| 286.3 | 3.03025547 | 286.3 | 11.385523 | 286.3 | 11.385523 | 286.3 | 8.86521341 | | | | | 286.30 | 7.70802847 | | | 286.3 | 7.70802847 |
| 286.4 | 3.04986877 | 286.4 | 11.4051363 | 286.4 | 11.4051363 | 286.4 | 8.85540676 | | | | | 286.40 | 7.72764178 | | | 286.4 | 7.72764178 |
| 286.5 | 3.05967542 | 286.5 | 11.4247496 | 286.5 | 11.4247496 | 286.5 | 8.8456001 | | | | | 286.50 | 7.74725508 | | | 286.5 | 7.74725508 |
| 286.6 | 3.04986877 | 286.6 | 11.4345562 | 286.6 | 11.4345562 | 286.6 | 8.86521341 | | | | | 286.60 | 7.74725508 | | | 286.6 | 7.74725508 |
| 286.7 | 3.04986877 | 286.7 | 11.4541695 | 286.7 | 11.4541695 | 286.7 | 8.88482671 | | | | | 286.70 | 7.73744843 | | | 286.7 | 7.73744843 |
| 286.8 | 3.05967542 | 286.8 | 11.4443629 | 286.8 | 11.4443629 | 286.8 | 8.85540676 | | | | | 286.80 | 7.73744843 | | | 286.8 | 7.73744843 |
| 286.9 | 3.07928873 | 286.9 | 11.4541695 | 286.9 | 11.4541695 | 286.9 | 8.87502006 | | | | | 286.90 | 7.71783512 | | | 286.9 | 7.71783512 |
| 287 | 3.08909538 | 287 | 11.4541695 | 287 | 11.4541695 | 287 | 8.86521341 | | | | | 287.00 | 7.73744843 | | | 287 | 7.73744843 |
| 287.1 | 3.07928873 | 287.1 | 11.4541695 | 287.1 | 11.4541695 | 287.1 | 8.86521341 | | | | | 287.10 | 7.73744843 | | | 287.1 | 7.73744843 |
| 287.2 | 3.06948208 | 287.2 | 11.4345562 | 287.2 | 11.4345562 | 287.2 | 8.89463336 | | | | | 287.20 | 7.73744843 | | | 287.2 | 7.73744843 |
| 287.3 | 3.08909538 | 287.3 | 11.4541695 | 287.3 | 11.4541695 | 287.3 | 8.90444002 | | | | | 287.30 | 7.74725508 | | | 287.3 | 7.74725508 |
| 287.4 | 3.08909538 | 287.4 | 11.4737828 | 287.4 | 11.4737828 | 287.4 | 8.87502006 | | | | | 287.40 | 7.74725508 | | | 287.4 | 7.74725508 |
| 287.5 | 3.07928873 | 287.5 | 11.4737828 | 287.5 | 11.4737828 | 287.5 | 8.83579345 | | | | | 287.50 | 7.72764178 | | | 287.5 | 7.72764178 |
| 287.6 | 3.10870868 | 287.6 | 11.4737828 | 287.6 | 11.4737828 | 287.6 | 8.85540676 | | | | | 287.60 | 7.74725508 | | | 287.6 | 7.74725508 |
| 287.7 | 3.08909538 | 287.7 | 11.4835895 | 287.7 | 11.4835895 | 287.7 | 8.86521341 | | | | | 287.70 | 7.73744843 | | | 287.7 | 7.73744843 |
| 287.8 | 3.07928873 | 287.8 | 11.5032028 | 287.8 | 11.5032028 | 287.8 | 8.87502006 | | | | | 287.80 | 7.75706173 | | | 287.8 | 7.75706173 |
| 287.9 | 3.10870868 | 287.9 | 11.5228161 | 287.9 | 11.5228161 | 287.9 | 8.92405332 | | | | | 287.90 | 7.77667504 | | | 287.9 | 7.77667504 |
| 288 | 3.11851534 | 288 | 11.4835895 | 288 | 11.4835895 | 288 | 8.91424667 | | | | | 288.00 | 7.76686838 | | | 288 | 7.76686838 |
| 288.1 | 3.10870868 | 288.1 | 11.4835895 | 288.1 | 11.4835895 | 288.1 | 8.88482671 | | | | | 288.10 | 7.74725508 | | | 288.1 | 7.74725508 |
| 288.2 | 3.11851534 | 288.2 | 11.5130094 | 288.2 | 11.5130094 | 288.2 | 8.88482671 | | | | | 288.20 | 7.74725508 | | | 288.2 | 7.74725508 |
| 288.3 | 3.09890203 | 288.3 | 11.5130094 | 288.3 | 11.5130094 | 288.3 | 8.90444002 | | | | | 288.30 | 7.76686838 | | | 288.3 | 7.76686838 |
| 288.4 | 3.10870868 | 288.4 | 11.5228161 | 288.4 | 11.5228161 | 288.4 | 8.90444002 | | | | | 288.40 | 7.77667504 | | | 288.4 | 7.77667504 |
| 288.5 | 3.14793529 | 288.5 | 11.5424294 | 288.5 | 11.5424294 | 288.5 | 8.91424667 | | | | | 288.50 | 7.79628834 | | | 288.5 | 7.79628834 |
| 288.6 | 3.13812864 | 288.6 | 11.5424294 | 288.6 | 11.5424294 | 288.6 | 8.88482671 | | | | | 288.60 | 7.81590164 | | | 288.6 | 7.81590164 |
| 288.7 | 3.11851534 | 288.7 | 11.5522361 | 288.7 | 11.5522361 | 288.7 | 8.89463336 | | | | | 288.70 | 7.77667504 | | | 288.7 | 7.77667504 |
| 288.8 | 3.12832199 | 288.8 | 11.5326228 | 288.8 | 11.5326228 | 288.8 | 8.90444002 | | | | | 288.80 | 7.74725508 | | | 288.8 | 7.74725508 |
| 288.9 | 3.12832199 | 288.9 | 11.5326228 | 288.9 | 11.5326228 | 288.9 | 8.91424667 | | | | | 288.90 | 7.74725508 | | | 288.9 | 7.74725508 |
| 289 | 3.14793529 | 289 | 11.5424294 | 289 | 11.5424294 | 289 | 8.94366662 | | | | | 289.00 | 7.76686838 | | | 289 | 7.76686838 |
| 289.1 | 3.12832199 | 289.1 | 11.5718494 | 289.1 | 11.5718494 | 289.1 | 8.95347328 | | | | | 289.10 | 7.80609499 | | | 289.1 | 7.80609499 |
| 289.2 | 3.10870868 | 289.2 | 11.581656 | 289.2 | 11.581656 | 289.2 | 8.93385997 | | | | | 289.20 | 7.79628834 | | | 289.2 | 7.79628834 |
| 289.3 | 3.11851534 | 289.3 | 11.5914627 | 289.3 | 11.5914627 | 289.3 | 8.95347328 | | | | | 289.30 | 7.79628834 | | | 289.3 | 7.79628834 |
| 289.4 | 3.14793529 | 289.4 | 11.5718494 | 289.4 | 11.5718494 | 289.4 | 8.96327993 | | | | | 289.40 | 7.79628834 | | | 289.4 | 7.79628834 |
| 289.5 | 3.14793529 | 289.5 | 11.5522361 | 289.5 | 11.5522361 | 289.5 | 8.97308658 | | | | | 289.50 | 7.78648169 | | | 289.5 | 7.78648169 |
| 289.6 | 3.14793529 | 289.6 | 11.5522361 | 289.6 | 11.5522361 | 289.6 | 8.95347328 | | | | | 289.60 | 7.80609499 | | | 289.6 | 7.80609499 |
| 289.7 | 3.13812864 | 289.7 | 11.581656 | 289.7 | 11.581656 | 289.7 | 8.96327993 | | | | | 289.70 | 7.80609499 | | | 289.7 | 7.80609499 |
| 289.8 | 3.12832199 | 289.8 | 11.5914627 | 289.8 | 11.5914627 | 289.8 | 9.00250654 | | | | | 289.80 | 7.80609499 | | | 289.8 | 7.80609499 |
| 289.9 | 3.11851534 | 289.9 | 11.5718494 | 289.9 | 11.5718494 | 289.9 | 9.00250654 | | | | | 289.90 | 7.79628834 | | | 289.9 | 7.79628834 |
| 290 | 3.08909538 | 290 | 11.5522361 | 290 | 11.5522361 | 290 | 8.99269988 | | | | | 290.00 | 7.76686838 | | | 290 | 7.76686838 |

| | | | | | | | | | | | | | | | | | |
|---|---|---|---|---|---|---|---|---|---|---|---|---|---|---|---|---|---|
| 290.1 | 3.09890203 | 290.1 | 11.5718494 | 290.1 | 11.5718494 | 290.1 | 9.00250654 | | | | | 290.10 | 7.81590164 | | | 290.1 | 7.81590164 |
| 290.2 | 3.11851534 | 290.2 | 11.6306893 | 290.2 | 11.6306893 | 290.2 | 8.99269988 | | | | | 290.20 | 7.8257083 | | | 290.2 | 7.8257083 |
| 290.3 | 3.13812864 | 290.3 | 11.6503026 | 290.3 | 11.6503026 | 290.3 | 8.98289323 | | | | | 290.30 | 7.8257083 | | | 290.3 | 7.8257083 |
| 290.4 | 3.13812864 | 290.4 | 11.6404959 | 290.4 | 11.6404959 | 290.4 | 9.03192649 | | | | | 290.40 | 7.79628834 | | | 290.4 | 7.79628834 |
| 290.5 | 3.13812864 | 290.5 | 11.6306893 | 290.5 | 11.6306893 | 290.5 | 9.03192649 | | | | | 290.50 | 7.78648169 | | | 290.5 | 7.78648169 |
| 290.6 | 3.15774194 | 290.6 | 11.611076 | 290.6 | 11.611076 | 290.6 | 9.01231319 | | | | | 290.60 | 7.80609499 | | | 290.6 | 7.80609499 |
| 290.7 | 3.13812864 | 290.7 | 11.6306893 | 290.7 | 11.6306893 | 290.7 | 9.00250654 | | | | | 290.70 | 7.8257083 | | | 290.7 | 7.8257083 |
| 290.8 | 3.11851534 | 290.8 | 11.6306893 | 290.8 | 11.6306893 | 290.8 | 9.02211984 | | | | | 290.80 | 7.8257083 | | | 290.8 | 7.8257083 |
| 290.9 | 3.13812864 | 290.9 | 11.6306893 | 290.9 | 11.6306893 | 290.9 | 9.0515398 | | | | | 290.90 | 7.8257083 | | | 290.9 | 7.8257083 |
| 291 | 3.12832199 | 291 | 11.6503026 | 291 | 11.6503026 | 291 | 9.03192649 | | | | | 291.00 | 7.8257083 | | | 291 | 7.8257083 |
| 291.1 | 3.14793529 | 291.1 | 11.6503026 | 291.1 | 11.6503026 | 291.1 | 9.02211984 | | | | | 291.10 | 7.81590164 | | | 291.1 | 7.81590164 |
| 291.2 | 3.14793529 | 291.2 | 11.6503026 | 291.2 | 11.6503026 | 291.2 | 9.03192649 | | | | | 291.20 | 7.83551495 | | | 291.2 | 7.83551495 |
| 291.3 | 3.15774194 | 291.3 | 11.6404959 | 291.3 | 11.6404959 | 291.3 | 9.02211984 | | | | | 291.30 | 7.8649349 | | | 291.3 | 7.8649349 |
| 291.4 | 3.14793529 | 291.4 | 11.6503026 | 291.4 | 11.6503026 | 291.4 | 9.02211984 | | | | | 291.40 | 7.87474156 | | | 291.4 | 7.87474156 |
| 291.5 | 3.13812864 | 291.5 | 11.6601092 | 291.5 | 11.6601092 | 291.5 | 9.04173314 | | | | | 291.50 | 7.85512825 | | | 291.5 | 7.85512825 |
| 291.6 | 3.13812864 | 291.6 | 11.6699159 | 291.6 | 11.6699159 | 291.6 | 9.03192649 | | | | | 291.60 | 7.87474156 | | | 291.6 | 7.87474156 |
| 291.7 | 3.15774194 | 291.7 | 11.6601092 | 291.7 | 11.6601092 | 291.7 | 9.04173314 | | | | | 291.70 | 7.8649349 | | | 291.7 | 7.8649349 |
| 291.8 | 3.1675486 | 291.8 | 11.6601092 | 291.8 | 11.6601092 | 291.8 | 9.0515398 | | | | | 291.80 | 7.8649349 | | | 291.8 | 7.8649349 |
| 291.9 | 3.1871619 | 291.9 | 11.6797225 | 291.9 | 11.6797225 | 291.9 | 9.08095975 | | | | | 291.90 | 7.8649349 | | | 291.9 | 7.8649349 |
| 292 | 3.1675486 | 292 | 11.6797225 | 292 | 11.6797225 | 292 | 9.0711531 | | | | | 292.00 | 7.8257083 | | | 292 | 7.8257083 |
| 292.1 | 3.15774194 | 292.1 | 11.6601092 | 292.1 | 11.6601092 | 292.1 | 9.06134645 | | | | | 292.10 | 7.8257083 | | | 292.1 | 7.8257083 |
| 292.2 | 3.15774194 | 292.2 | 11.6895292 | 292.2 | 11.6895292 | 292.2 | 9.08095975 | | | | | 292.20 | 7.83551495 | | | 292.2 | 7.83551495 |
| 292.3 | 3.1675486 | 292.3 | 11.6993358 | 292.3 | 11.6993358 | 292.3 | 9.11037971 | | | | | 292.30 | 7.8453216 | | | 292.3 | 7.8453216 |
| 292.4 | 3.17735525 | 292.4 | 11.6895292 | 292.4 | 11.6895292 | 292.4 | 9.0907664 | | | | | 292.40 | 7.89435486 | | | 292.4 | 7.89435486 |
| 292.5 | 3.1675486 | 292.5 | 11.7091425 | 292.5 | 11.7091425 | 292.5 | 9.10057306 | | | | | 292.50 | 7.90416151 | | | 292.5 | 7.90416151 |
| 292.6 | 3.1675486 | 292.6 | 11.8268223 | 292.6 | 11.8268223 | 292.6 | 9.08095975 | | | | | 292.60 | 7.89435486 | | | 292.6 | 7.89435486 |
| 292.7 | 3.1871619 | 292.7 | 11.8268223 | 292.7 | 11.8268223 | 292.7 | 9.0907664 | | | | | 292.70 | 7.88454821 | | | 292.7 | 7.88454821 |
| 292.8 | 3.1871619 | 292.8 | 11.7875957 | 292.8 | 11.7875957 | 292.8 | 9.08095975 | | | | | 292.80 | 7.87474156 | | | 292.8 | 7.87474156 |
| 292.9 | 3.1871619 | 292.9 | 11.7875957 | 292.9 | 11.7875957 | 292.9 | 9.10057306 | | | | | 292.90 | 7.89435486 | | | 292.9 | 7.89435486 |
| 293 | 3.1871619 | 293 | 11.8170157 | 293 | 11.8170157 | 293 | 9.0907664 | | | | | 293.00 | 7.90416151 | | | 293 | 7.90416151 |
| 293.1 | 3.21658186 | 293.1 | 11.8268223 | 293.1 | 11.8268223 | 293.1 | 9.08095975 | | | | | 293.10 | 7.87474156 | | | 293.1 | 7.87474156 |
| 293.2 | 3.21658186 | 293.2 | 11.8464356 | 293.2 | 11.8464356 | 293.2 | 9.0907664 | | | | | 293.20 | 7.87474156 | | | 293.2 | 7.87474156 |
| 293.3 | 3.21658186 | 293.3 | 11.836629 | 293.3 | 11.836629 | 293.3 | 9.0907664 | | | | | 293.30 | 7.89435486 | | | 293.3 | 7.89435486 |
| 293.4 | 3.19696855 | 293.4 | 11.8464356 | 293.4 | 11.8464356 | 293.4 | 9.0907664 | | | | | 293.40 | 7.91396816 | | | 293.4 | 7.91396816 |
| 293.5 | 3.19696855 | 293.5 | 11.836629 | 293.5 | 11.836629 | 293.5 | 9.10057306 | | | | | 293.50 | 7.92377482 | | | 293.5 | 7.92377482 |
| 293.6 | 3.2067752 | 293.6 | 11.8464356 | 293.6 | 11.8464356 | 293.6 | 9.10057306 | | | | | 293.60 | 7.91396816 | | | 293.6 | 7.91396816 |
| 293.7 | 3.19696855 | 293.7 | 11.8758556 | 293.7 | 11.8758556 | 293.7 | 9.11037971 | | | | | 293.70 | 7.91396816 | | | 293.7 | 7.91396816 |
| 293.8 | 3.2067752 | 293.8 | 11.836629 | 293.8 | 11.836629 | 293.8 | 9.12999301 | | | | | 293.80 | 7.92377482 | | | 293.8 | 7.92377482 |
| 293.9 | 3.1871619 | 293.9 | 11.9052755 | 293.9 | 11.9052755 | 293.9 | 9.13979966 | | | | | 293.90 | 7.92377482 | | | 293.9 | 7.92377482 |
| 294 | 3.19696855 | 294 | 11.9052755 | 294 | 11.9052755 | 294 | 9.13979966 | | | | | 294.00 | 7.94338812 | | | 294 | 7.94338812 |
| 294.1 | 3.1871619 | 294.1 | 11.8954689 | 294.1 | 11.8954689 | 294.1 | 9.13979966 | | | | | 294.10 | 7.93358147 | | | 294.1 | 7.93358147 |
| 294.2 | 3.21658186 | 294.2 | 11.8954689 | 294.2 | 11.8954689 | 294.2 | 9.12999301 | | | | | 294.20 | 7.91396816 | | | 294.2 | 7.91396816 |
| 294.3 | 3.24600181 | 294.3 | 11.8758556 | 294.3 | 11.8758556 | 294.3 | 9.12999301 | | | | | 294.30 | 7.91396816 | | | 294.3 | 7.91396816 |
| 294.4 | 3.21658186 | 294.4 | 11.8856622 | 294.4 | 11.8856622 | 294.4 | 9.14960632 | | | | | 294.40 | 7.92377482 | | | 294.4 | 7.92377482 |
| 294.5 | 3.2067752 | 294.5 | 11.9052755 | 294.5 | 11.9052755 | 294.5 | 9.12999301 | | | | | 294.50 | 7.92377482 | | | 294.5 | 7.92377482 |
| 294.6 | 3.23619516 | 294.6 | 11.9445021 | 294.6 | 11.9445021 | 294.6 | 9.12999301 | | | | | 294.60 | 7.91396816 | | | 294.6 | 7.91396816 |
| 294.7 | 3.21658186 | 294.7 | 11.9150822 | 294.7 | 11.9150822 | 294.7 | 9.13979966 | | | | | 294.70 | 7.92377482 | | | 294.7 | 7.92377482 |
| 294.8 | 3.24600181 | 294.8 | 11.8856622 | 294.8 | 11.8856622 | 294.8 | 9.13979966 | | | | | 294.80 | 7.92377482 | | | 294.8 | 7.92377482 |
| 294.9 | 3.23619516 | 294.9 | 11.9150822 | 294.9 | 11.9150822 | 294.9 | 9.13979966 | | | | | 294.90 | 7.92377482 | | | 294.9 | 7.92377482 |
| 295 | 3.22638851 | 295 | 11.9248888 | 295 | 11.9248888 | 295 | 9.15941297 | | | | | 295.00 | 7.92377482 | | | 295 | 7.92377482 |
| 295.1 | 3.22638851 | 295.1 | 11.9641154 | 295.1 | 11.9641154 | 295.1 | 9.14960632 | | | | | 295.10 | 7.94338812 | | | 295.1 | 7.94338812 |
| 295.2 | 3.24600181 | 295.2 | 11.9641154 | 295.2 | 11.9641154 | 295.2 | 9.17902627 | | | | | 295.20 | 7.95319477 | | | 295.2 | 7.95319477 |
| 295.3 | 3.26561512 | 295.3 | 11.9739221 | 295.3 | 11.9739221 | 295.3 | 9.15941297 | | | | | 295.30 | 7.96300142 | | | 295.3 | 7.96300142 |
| 295.4 | 3.27542177 | 295.4 | 11.9543088 | 295.4 | 11.9543088 | 295.4 | 9.16921962 | | | | | 295.40 | 7.95319477 | | | 295.4 | 7.95319477 |
| 295.5 | 3.27542177 | 295.5 | 11.9739221 | 295.5 | 11.9739221 | 295.5 | 9.15941297 | | | | | 295.50 | 7.97280808 | | | 295.5 | 7.97280808 |
| 295.6 | 3.26561512 | 295.6 | 11.9837287 | 295.6 | 11.9837287 | 295.6 | 9.15941297 | | | | | 295.60 | 7.95319477 | | | 295.6 | 7.95319477 |
| 295.7 | 3.23619516 | 295.7 | 11.9739221 | 295.7 | 11.9739221 | 295.7 | 9.14960632 | | | | | 295.70 | 7.96300142 | | | 295.7 | 7.96300142 |
| 295.8 | 3.23619516 | 295.8 | 11.9739221 | 295.8 | 11.9739221 | 295.8 | 9.16921962 | | | | | 295.80 | 7.97280808 | | | 295.8 | 7.97280808 |
| 295.9 | 3.26561512 | 295.9 | 11.9837287 | 295.9 | 11.9837287 | 295.9 | 9.15941297 | | | | | 295.90 | 7.98261473 | | | 295.9 | 7.98261473 |
| 296 | 3.26561512 | 296 | 12.003342 | 296 | 12.003342 | 296 | 9.17902627 | | | | | 296.00 | 7.95319477 | | | 296 | 7.95319477 |
| 296.1 | 3.27542177 | 296.1 | 12.0131487 | 296.1 | 12.0131487 | 296.1 | 9.17902627 | | | | | 296.10 | 7.95319477 | | | 296.1 | 7.95319477 |

| | | | | | | | | | | | | | | | | | |
|---|---|---|---|---|---|---|---|---|---|---|---|---|---|---|---|---|---|
| 296.2 | 3.27542177 | 296.2 | 12.0229554 | 296.2 | 12.0229554 | 296.2 | 9.16921962 | | | | | 296.20 | 7.98261473 | | | 296.2 | 7.98261473 |
| 296.3 | 3.27542177 | 296.3 | 12.032762 | 296.3 | 12.032762 | 296.3 | 9.16921962 | | | | | 296.30 | 7.98261473 | | | 296.3 | 7.98261473 |
| 296.4 | 3.28522842 | 296.4 | 12.062182 | 296.4 | 12.062182 | 296.4 | 9.16921962 | | | | | 296.40 | 7.98261473 | | | 296.4 | 7.98261473 |
| 296.5 | 3.29503507 | 296.5 | 12.0425687 | 296.5 | 12.0425687 | 296.5 | 9.16921962 | | | | | 296.50 | 7.99242138 | | | 296.5 | 7.99242138 |
| 296.6 | 3.26561512 | 296.6 | 12.0425687 | 296.6 | 12.0425687 | 296.6 | 9.17902627 | | | | | 296.60 | 7.99242138 | | | 296.6 | 7.99242138 |
| 296.7 | 3.26561512 | 296.7 | 12.0229554 | 296.7 | 12.0229554 | 296.7 | 9.18883292 | | | | | 296.70 | 8.00222803 | | | 296.7 | 8.00222803 |
| 296.8 | 3.26561512 | 296.8 | 12.0131487 | 296.8 | 12.0131487 | 296.8 | 9.17902627 | | | | | 296.80 | 7.99242138 | | | 296.8 | 7.99242138 |
| 296.9 | 3.25580846 | 296.9 | 12.032762 | 296.9 | 12.032762 | 296.9 | 9.17902627 | | | | | 296.90 | 8.01203468 | | | 296.9 | 8.01203468 |
| 297 | 3.26561512 | 297 | 12.0425687 | 297 | 12.0425687 | 297 | 9.16921962 | | | | | 297.00 | 8.02184134 | | | 297 | 8.02184134 |
| 297.1 | 3.30484172 | 297.1 | 12.0817953 | 297.1 | 12.0817953 | 297.1 | 9.18883292 | | | | | 297.10 | 7.99242138 | | | 297.1 | 7.99242138 |
| 297.2 | 3.28522842 | 297.2 | 12.0817953 | 297.2 | 12.0817953 | 297.2 | 9.19863958 | | | | | 297.20 | 8.02184134 | | | 297.2 | 8.02184134 |
| 297.3 | 3.30484172 | 297.3 | 12.1014086 | 297.3 | 12.1014086 | 297.3 | 9.19863958 | | | | | 297.30 | 8.01203468 | | | 297.3 | 8.01203468 |
| 297.4 | 3.31464838 | 297.4 | 12.0916019 | 297.4 | 12.0916019 | 297.4 | 9.17902627 | | | | | 297.40 | 8.01203468 | | | 297.4 | 8.01203468 |
| 297.5 | 3.31464838 | 297.5 | 12.062182 | 297.5 | 12.062182 | 297.5 | 9.21825288 | | | | | 297.50 | 8.00222803 | | | 297.5 | 8.00222803 |
| 297.6 | 3.31464838 | 297.6 | 12.0817953 | 297.6 | 12.0817953 | 297.6 | 9.21825288 | | | | | 297.60 | 7.99242138 | | | 297.6 | 7.99242138 |
| 297.7 | 3.30484172 | 297.7 | 12.0817953 | 297.7 | 12.0817953 | 297.7 | 9.22805953 | | | | | 297.70 | 8.00222803 | | | 297.7 | 8.00222803 |
| 297.8 | 3.27542177 | 297.8 | 12.0916019 | 297.8 | 12.0916019 | 297.8 | 9.19863958 | | | | | 297.80 | 8.01203468 | | | 297.8 | 8.01203468 |
| 297.9 | 3.29503507 | 297.9 | 12.1112152 | 297.9 | 12.1112152 | 297.9 | 9.17902627 | | | | | 297.90 | 8.01203468 | | | 297.9 | 8.01203468 |
| 298 | 3.32445503 | 298 | 12.1014086 | 298 | 12.1014086 | 298 | 9.17902627 | | | | | 298.00 | 8.01203468 | | | 298 | 8.01203468 |
| 298.1 | 3.32445503 | 298.1 | 12.1210219 | 298.1 | 12.1210219 | 298.1 | 9.19863958 | | | | | 298.10 | 8.00222803 | | | 298.1 | 8.00222803 |
| 298.2 | 3.30484172 | 298.2 | 12.1504418 | 298.2 | 12.1504418 | 298.2 | 9.20844623 | | | | | 298.20 | 8.01203468 | | | 298.2 | 8.01203468 |
| 298.3 | 3.32445503 | 298.3 | 12.1700551 | 298.3 | 12.1700551 | 298.3 | 9.20844623 | | | | | 298.30 | 8.03164799 | | | 298.3 | 8.03164799 |
| 298.4 | 3.31464838 | 298.4 | 12.1504418 | 298.4 | 12.1504418 | 298.4 | 9.22805953 | | | | | 298.40 | 8.03164799 | | | 298.4 | 8.03164799 |
| 298.5 | 3.29503507 | 298.5 | 12.1700551 | 298.5 | 12.1700551 | 298.5 | 9.21825288 | | | | | 298.50 | 8.04145464 | | | 298.5 | 8.04145464 |
| 298.6 | 3.30484172 | 298.6 | 12.1798618 | 298.6 | 12.1798618 | 298.6 | 9.21825288 | | | | | 298.60 | 8.02184134 | | | 298.6 | 8.02184134 |
| 298.7 | 3.31464838 | 298.7 | 12.1700551 | 298.7 | 12.1700551 | 298.7 | 9.24767284 | | | | | 298.70 | 8.02184134 | | | 298.7 | 8.02184134 |
| 298.8 | 3.33426168 | 298.8 | 12.1798618 | 298.8 | 12.1798618 | 298.8 | 9.20844623 | | | | | 298.80 | 8.05126129 | | | 298.8 | 8.05126129 |
| 298.9 | 3.35387498 | 298.9 | 12.1700551 | 298.9 | 12.1700551 | 298.9 | 9.22805953 | | | | | 298.90 | 8.04145464 | | | 298.9 | 8.04145464 |
| 299 | 3.31464838 | 299 | 12.1504418 | 299 | 12.1504418 | 299 | 9.23786618 | | | | | 299.00 | 8.04145464 | | | 299 | 8.04145464 |
| 299.1 | 3.30484172 | 299.1 | 12.1602485 | 299.1 | 12.1602485 | 299.1 | 9.23786618 | | | | | 299.10 | 8.02184134 | | | 299.1 | 8.02184134 |
| 299.2 | 3.31464838 | 299.2 | 12.1896684 | 299.2 | 12.1896684 | 299.2 | 9.25747949 | | | | | 299.20 | 8.04145464 | | | 299.2 | 8.04145464 |
| 299.3 | 3.30484172 | 299.3 | 12.1798618 | 299.3 | 12.1798618 | 299.3 | 9.17902627 | | | | | 299.30 | 8.0708746 | | | 299.3 | 8.0708746 |
| 299.4 | 3.31464838 | 299.4 | 12.1994751 | 299.4 | 12.1994751 | 299.4 | 9.17902627 | | | | | 299.40 | 8.04145464 | | | 299.4 | 8.04145464 |
| 299.5 | 3.30484172 | 299.5 | 12.1994751 | 299.5 | 12.1994751 | 299.5 | 9.14960632 | | | | | 299.50 | 8.0904879 | | | 299.5 | 8.0904879 |
| 299.6 | 3.31464838 | 299.6 | 12.2092817 | 299.6 | 12.2092817 | 299.6 | 9.13979966 | | | | | 299.60 | 8.08068125 | | | 299.6 | 8.08068125 |
| 299.7 | 3.33426168 | 299.7 | 12.228895 | 299.7 | 12.228895 | 299.7 | 9.16921962 | | | | | 299.70 | 8.0904879 | | | 299.7 | 8.0904879 |
| 299.8 | 3.35387498 | 299.8 | 12.2190884 | 299.8 | 12.2190884 | 299.8 | 9.13979966 | | | | | 299.80 | 8.1101012 | | | 299.8 | 8.1101012 |
| 299.9 | 3.35387498 | 299.9 | 12.2092817 | 299.9 | 12.2092817 | 299.9 | 9.15941297 | | | | | 299.90 | 8.10029455 | | | 299.9 | 8.10029455 |
| 300 | 3.35387498 | 300 | 12.1994751 | 300 | 12.1994751 | 300 | 9.18883292 | | | | | 300.00 | 8.1101012 | | | 300 | 8.1101012 |
| 300.1 | 3.34406833 | 300.1 | 12.228895 | 300.1 | 12.228895 | 300.1 | 9.16921962 | | | | | 300.10 | 8.0904879 | | | 300.1 | 8.0904879 |
| 300.2 | 3.34406833 | 300.2 | 12.2387017 | 300.2 | 12.2387017 | 300.2 | 9.15941297 | | | | | 300.20 | 8.08068125 | | | 300.2 | 8.08068125 |
| 300.3 | 3.36368164 | 300.3 | 12.228895 | 300.3 | 12.228895 | 300.3 | 9.18883292 | | | | | 300.30 | 8.10029455 | | | 300.3 | 8.10029455 |
| 300.4 | 3.36368164 | 300.4 | 12.2190884 | 300.4 | 12.2190884 | 300.4 | 9.17902627 | | | | | 300.40 | 8.10029455 | | | 300.4 | 8.10029455 |
| 300.5 | 3.38329494 | 300.5 | 12.2387017 | 300.5 | 12.2387017 | 300.5 | 9.15941297 | | | | | 300.50 | 8.10029455 | | | 300.5 | 8.10029455 |
| 300.6 | 3.36368164 | 300.6 | 12.2485083 | 300.6 | 12.2485083 | 300.6 | 9.19863958 | | | | | 300.60 | 8.0904879 | | | 300.6 | 8.0904879 |
| 300.7 | 3.35387498 | 300.7 | 12.228895 | 300.7 | 12.228895 | 300.7 | 9.20844623 | | | | | 300.70 | 8.11990786 | | | 300.7 | 8.11990786 |
| 300.8 | 3.35387498 | 300.8 | 12.2681217 | 300.8 | 12.2681217 | 300.8 | 9.17902627 | | | | | 300.80 | 8.12971451 | | | 300.8 | 8.12971451 |
| 300.9 | 3.35387498 | 300.9 | 12.2681217 | 300.9 | 12.2681217 | 300.9 | 9.16921962 | | | | | 300.90 | 8.13952116 | | | 300.9 | 8.13952116 |
| 301 | 3.35387498 | 301 | 12.2779283 | 301 | 12.2779283 | 301 | 9.15941297 | | | | | 301.00 | 8.12971451 | | | 301 | 8.12971451 |
| 301.1 | 3.34406833 | 301.1 | 12.2387017 | 301.1 | 12.2387017 | 301.1 | 9.12999301 | | | | | 301.10 | 8.11990786 | | | 301.1 | 8.11990786 |
| 301.2 | 3.35387498 | 301.2 | 12.2681217 | 301.2 | 12.2681217 | 301.2 | 9.11037971 | | | | | 301.20 | 8.1101012 | | | 301.2 | 8.1101012 |
| 301.3 | 3.35387498 | 301.3 | 12.2779283 | 301.3 | 12.2779283 | 301.3 | 9.0907664 | | | | | 301.30 | 8.11990786 | | | 301.3 | 8.11990786 |
| 301.4 | 3.34406833 | 301.4 | 12.2681217 | 301.4 | 12.2681217 | 301.4 | 9.0711531 | | | | | 301.40 | 8.12971451 | | | 301.4 | 8.12971451 |
| 301.5 | 3.34406833 | 301.5 | 12.2975416 | 301.5 | 12.2975416 | 301.5 | 9.08095975 | | | | | 301.50 | 8.15913446 | | | 301.5 | 8.15913446 |
| 301.6 | 3.39310159 | 301.6 | 12.3367682 | 301.6 | 12.3367682 | 301.6 | 9.0907664 | | | | | 301.60 | 8.12971451 | | | 301.6 | 8.12971451 |
| 301.7 | 3.39310159 | 301.7 | 12.3171549 | 301.7 | 12.3171549 | 301.7 | 9.0711531 | | | | | 301.70 | 8.1101012 | | | 301.7 | 8.1101012 |
| 301.8 | 3.35387498 | 301.8 | 12.3073483 | 301.8 | 12.3073483 | 301.8 | 9.0711531 | | | | | 301.80 | 8.12971451 | | | 301.8 | 8.12971451 |
| 301.9 | 3.39310159 | 301.9 | 12.3367682 | 301.9 | 12.3367682 | 301.9 | 9.0907664 | | | | | 301.90 | 8.10029455 | | | 301.9 | 8.10029455 |
| 302 | 3.39310159 | 302 | 12.3661882 | 302 | 12.3661882 | 302 | 9.08095975 | | | | | 302.00 | 8.1101012 | | | 302 | 8.1101012 |
| 302.1 | 3.38329494 | 302.1 | 12.3858015 | 302.1 | 12.3858015 | 302.1 | 9.0711531 | | | | | 302.10 | 8.13952116 | | | 302.1 | 8.13952116 |
| 302.2 | 3.38329494 | 302.2 | 12.4152214 | 302.2 | 12.4152214 | 302.2 | 9.10057306 | | | | | 302.20 | 8.16894112 | | | 302.2 | 8.16894112 |

| | | | | | | | | | | | | | | | | | |
|---|---|---|---|---|---|---|---|---|---|---|---|---|---|---|---|---|---|
| 302.3 | 3.4127149 | 302.3 | 12.3956081 | 302.3 | 12.3956081 | 302.3 | 9.11037971 | | | | | 302.30 | 8.13952116 | | | 302.3 | 8.13952116 |
| 302.4 | 3.4127149 | 302.4 | 12.4054148 | 302.4 | 12.4054148 | 302.4 | 9.15941297 | | | | | 302.40 | 8.11990786 | | | 302.4 | 8.11990786 |
| 302.5 | 3.38329494 | 302.5 | 12.4054148 | 302.5 | 12.4054148 | 302.5 | 9.13979966 | | | | | 302.50 | 8.15913446 | | | 302.5 | 8.15913446 |
| 302.6 | 3.39310159 | 302.6 | 12.3858015 | 302.6 | 12.3858015 | 302.6 | 9.12018636 | | | | | 302.60 | 8.16894112 | | | 302.6 | 8.16894112 |
| 302.7 | 3.38329494 | 302.7 | 12.4054148 | 302.7 | 12.4054148 | 302.7 | 9.12018636 | | | | | 302.70 | 8.14932781 | | | 302.7 | 8.14932781 |
| 302.8 | 3.38329494 | 302.8 | 12.4152214 | 302.8 | 12.4152214 | 302.8 | 9.11037971 | | | | | 302.80 | 8.18855442 | | | 302.8 | 8.18855442 |
| 302.9 | 3.38329494 | 302.9 | 12.4152214 | 302.9 | 12.4152214 | 302.9 | 9.10057306 | | | | | 302.90 | 8.18855442 | | | 302.9 | 8.18855442 |
| 303 | 3.42252155 | 303 | 12.3759948 | 303 | 12.3759948 | 303 | 9.12018636 | | | | | 303.00 | 8.16894112 | | | 303 | 8.16894112 |
| 303.1 | 3.40290824 | 303.1 | 12.3956081 | 303.1 | 12.3956081 | 303.1 | 9.13979966 | | | | | 303.10 | 8.16894112 | | | 303.1 | 8.16894112 |
| 303.2 | 3.39310159 | 303.2 | 12.4250281 | 303.2 | 12.4250281 | 303.2 | 9.13979966 | | | | | 303.20 | 8.16894112 | | | 303.2 | 8.16894112 |
| 303.3 | 3.39310159 | 303.3 | 12.4642547 | 303.3 | 12.4642547 | 303.3 | 9.12018636 | | | | | 303.30 | 8.16894112 | | | 303.3 | 8.16894112 |
| 303.4 | 3.40290824 | 303.4 | 12.454448 | 303.4 | 12.454448 | 303.4 | 9.10057306 | | | | | 303.40 | 8.14932781 | | | 303.4 | 8.14932781 |
| 303.5 | 3.42252155 | 303.5 | 12.4348347 | 303.5 | 12.4348347 | 303.5 | 9.12018636 | | | | | 303.50 | 8.15913446 | | | 303.5 | 8.15913446 |
| 303.6 | 3.42252155 | 303.6 | 12.454448 | 303.6 | 12.454448 | 303.6 | 9.12018636 | | | | | 303.60 | 8.18855442 | | | 303.6 | 8.18855442 |
| 303.7 | 3.42252155 | 303.7 | 12.4642547 | 303.7 | 12.4642547 | 303.7 | 9.08095975 | | | | | 303.70 | 8.16894112 | | | 303.7 | 8.16894112 |
| 303.8 | 3.42252155 | 303.8 | 12.483868 | 303.8 | 12.483868 | 303.8 | 9.10057306 | | | | | 303.80 | 8.17874777 | | | 303.8 | 8.17874777 |
| 303.9 | 3.4127149 | 303.9 | 12.513288 | 303.9 | 12.513288 | 303.9 | 9.11037971 | | | | | 303.90 | 8.18855442 | | | 303.9 | 8.18855442 |
| 304 | 3.4323282 | 304 | 12.513288 | 304 | 12.513288 | 304 | 9.12999301 | | | | | 304.00 | 8.16894112 | | | 304 | 8.16894112 |
| 304.1 | 3.4519415 | 304.1 | 12.5230946 | 304.1 | 12.5230946 | 304.1 | 9.14960632 | | | | | 304.10 | 8.19836107 | | | 304.1 | 8.19836107 |
| 304.2 | 3.4323282 | 304.2 | 12.5329013 | 304.2 | 12.5329013 | 304.2 | 9.17902627 | | | | | 304.20 | 8.21797438 | | | 304.2 | 8.21797438 |
| 304.3 | 3.42252155 | 304.3 | 12.513288 | 304.3 | 12.513288 | 304.3 | 9.14960632 | | | | | 304.30 | 8.21797438 | | | 304.3 | 8.21797438 |
| 304.4 | 3.4323282 | 304.4 | 12.5525146 | 304.4 | 12.5525146 | 304.4 | 9.14960632 | | | | | 304.40 | 8.20816772 | | | 304.4 | 8.20816772 |
| 304.5 | 3.42252155 | 304.5 | 12.5525146 | 304.5 | 12.5525146 | 304.5 | 9.14960632 | | | | | 304.50 | 8.21797438 | | | 304.5 | 8.21797438 |
| 304.6 | 3.4127149 | 304.6 | 12.5525146 | 304.6 | 12.5525146 | 304.6 | 9.14960632 | | | | | 304.60 | 8.22778103 | | | 304.6 | 8.22778103 |
| 304.7 | 3.44213485 | 304.7 | 12.5427079 | 304.7 | 12.5427079 | 304.7 | 9.12999301 | | | | | 304.70 | 8.23758768 | | | 304.7 | 8.23758768 |
| 304.8 | 3.4519415 | 304.8 | 12.5329013 | 304.8 | 12.5329013 | 304.8 | 9.12999301 | | | | | 304.80 | 8.21797438 | | | 304.8 | 8.21797438 |
| 304.9 | 3.46174816 | 304.9 | 12.5525146 | 304.9 | 12.5525146 | 304.9 | 9.15941297 | | | | | 304.90 | 8.18855442 | | | 304.9 | 8.18855442 |
| 305 | 3.4519415 | 305 | 12.5623212 | 305 | 12.5623212 | 305 | 9.15941297 | | | | | 305.00 | 8.19836107 | | | 305 | 8.19836107 |
| 305.1 | 3.46174816 | 305.1 | 12.5427079 | 305.1 | 12.5427079 | 305.1 | 9.15941297 | | | | | 305.10 | 8.22778103 | | | 305.1 | 8.22778103 |
| 305.2 | 3.4519415 | 305.2 | 12.5721279 | 305.2 | 12.5721279 | 305.2 | 9.18883292 | | | | | 305.20 | 8.24739433 | | | 305.2 | 8.24739433 |
| 305.3 | 3.46174816 | 305.3 | 12.6309678 | 305.3 | 12.6309678 | 305.3 | 9.17902627 | | | | | 305.30 | 8.26700764 | | | 305.3 | 8.26700764 |
| 305.4 | 3.46174816 | 305.4 | 12.6015478 | 305.4 | 12.6015478 | 305.4 | 9.14960632 | | | | | 305.40 | 8.22778103 | | | 305.4 | 8.22778103 |
| 305.5 | 3.46174816 | 305.5 | 12.5917412 | 305.5 | 12.5917412 | 305.5 | 9.15941297 | | | | | 305.50 | 8.24739433 | | | 305.5 | 8.24739433 |
| 305.6 | 3.46174816 | 305.6 | 12.6015478 | 305.6 | 12.6015478 | 305.6 | 9.18883292 | | | | | 305.60 | 8.24739433 | | | 305.6 | 8.24739433 |
| 305.7 | 3.46174816 | 305.7 | 12.6211611 | 305.7 | 12.6211611 | 305.7 | 9.20844623 | | | | | 305.70 | 8.23758768 | | | 305.7 | 8.23758768 |
| 305.8 | 3.47155481 | 305.8 | 12.6309678 | 305.8 | 12.6309678 | 305.8 | 9.18883292 | | | | | 305.80 | 8.23758768 | | | 305.8 | 8.23758768 |
| 305.9 | 3.46174816 | 305.9 | 12.6113545 | 305.9 | 12.6113545 | 305.9 | 9.18883292 | | | | | 305.90 | 8.25720098 | | | 305.9 | 8.25720098 |
| 306 | 3.46174816 | 306 | 12.6309678 | 306 | 12.6309678 | 306 | 9.16921962 | | | | | 306.00 | 8.26700764 | | | 306 | 8.26700764 |
| 306.1 | 3.46174816 | 306.1 | 12.6505811 | 306.1 | 12.6505811 | 306.1 | 9.16921962 | | | | | 306.10 | 8.25720098 | | | 306.1 | 8.25720098 |
| 306.2 | 3.47155481 | 306.2 | 12.6701944 | 306.2 | 12.6701944 | 306.2 | 9.18883292 | | | | | 306.20 | 8.25720098 | | | 306.2 | 8.25720098 |
| 306.3 | 3.48136146 | 306.3 | 12.6603877 | 306.3 | 12.6603877 | 306.3 | 9.17902627 | | | | | 306.30 | 8.25720098 | | | 306.3 | 8.25720098 |
| 306.4 | 3.46174816 | 306.4 | 12.6898077 | 306.4 | 12.6898077 | 306.4 | 9.16921962 | | | | | 306.40 | 8.27681429 | | | 306.4 | 8.27681429 |
| 306.5 | 3.47155481 | 306.5 | 12.6898077 | 306.5 | 12.6898077 | 306.5 | 9.16921962 | | | | | 306.50 | 8.28662094 | | | 306.5 | 8.28662094 |
| 306.6 | 3.48136146 | 306.6 | 12.6701944 | 306.6 | 12.6701944 | 306.6 | 9.19863958 | | | | | 306.60 | 8.26700764 | | | 306.6 | 8.26700764 |
| 306.7 | 3.48136146 | 306.7 | 12.709421 | 306.7 | 12.709421 | 306.7 | 9.19863958 | | | | | 306.70 | 8.26700764 | | | 306.7 | 8.26700764 |
| 306.8 | 3.49116811 | 306.8 | 12.7290343 | 306.8 | 12.7290343 | 306.8 | 9.18883292 | | | | | 306.80 | 8.28662094 | | | 306.8 | 8.28662094 |
| 306.9 | 3.49116811 | 306.9 | 12.6996143 | 306.9 | 12.6996143 | 306.9 | 9.20844623 | | | | | 306.90 | 8.3160409 | | | 306.9 | 8.3160409 |
| 307 | 3.49116811 | 307 | 12.680001 | 307 | 12.680001 | 307 | 9.20844623 | | | | | 307.00 | 8.32584755 | | | 307 | 8.32584755 |
| 307.1 | 3.47155481 | 307.1 | 12.6898077 | 307.1 | 12.6898077 | 307.1 | 9.19863958 | | | | | 307.10 | 8.3160409 | | | 307.1 | 8.3160409 |
| 307.2 | 3.49116811 | 307.2 | 12.6898077 | 307.2 | 12.6898077 | 307.2 | 9.21825288 | | | | | 307.20 | 8.3356542 | | | 307.2 | 8.3356542 |
| 307.3 | 3.51078142 | 307.3 | 12.7290343 | 307.3 | 12.7290343 | 307.3 | 9.19863958 | | | | | 307.30 | 8.3356542 | | | 307.3 | 8.3356542 |
| 307.4 | 3.50097476 | 307.4 | 12.7388409 | 307.4 | 12.7388409 | 307.4 | 9.19863958 | | | | | 307.40 | 8.3356542 | | | 307.4 | 8.3356542 |
| 307.5 | 3.48136146 | 307.5 | 12.6996143 | 307.5 | 12.6996143 | 307.5 | 9.22805953 | | | | | 307.50 | 8.3552675 | | | 307.5 | 8.3552675 |
| 307.6 | 3.50097476 | 307.6 | 12.709421 | 307.6 | 12.709421 | 307.6 | 9.22805953 | | | | | 307.60 | 8.36507416 | | | 307.6 | 8.36507416 |
| 307.7 | 3.49116811 | 307.7 | 12.7192276 | 307.7 | 12.7192276 | 307.7 | 9.19863958 | | | | | 307.70 | 8.37488081 | | | 307.7 | 8.37488081 |
| 307.8 | 3.49116811 | 307.8 | 12.7388409 | 307.8 | 12.7388409 | 307.8 | 9.21825288 | | | | | 307.80 | 8.34546085 | | | 307.8 | 8.34546085 |
| 307.9 | 3.50097476 | 307.9 | 12.8074875 | 307.9 | 12.8074875 | 307.9 | 9.22805953 | | | | | 307.90 | 8.34546085 | | | 307.9 | 8.34546085 |
| 308 | 3.51078142 | 308 | 12.7976809 | 308 | 12.7976809 | 308 | 9.22805953 | | | | | 308.00 | 8.3552675 | | | 308 | 8.3552675 |
| 308.1 | 3.52058807 | 308.1 | 12.8074875 | 308.1 | 12.8074875 | 308.1 | 9.20844623 | | | | | 308.10 | 8.34546085 | | | 308.1 | 8.34546085 |
| 308.2 | 3.55000802 | 308.2 | 12.7976809 | 308.2 | 12.7976809 | 308.2 | 9.21825288 | | | | | 308.20 | 8.3552675 | | | 308.2 | 8.3552675 |
| 308.3 | 3.55981468 | 308.3 | 12.7976809 | 308.3 | 12.7976809 | 308.3 | 9.23786618 | | | | | 308.30 | 8.38468746 | | | 308.3 | 8.38468746 |

| | | | | | | | | | | | | | | | | | |
|---|---|---|---|---|---|---|---|---|---|---|---|---|---|---|---|---|---|
| 308.4 | 3.53039472 | 308.4 | 12.8369075 | 308.4 | 12.8369075 | 308.4 | 9.23786618 | | | | | 308.40 | 8.38468746 | | | 308.4 | 8.38468746 |
| 308.5 | 3.52058807 | 308.5 | 12.8074875 | 308.5 | 12.8074875 | 308.5 | 9.23786618 | | | | | 308.50 | 8.36507416 | | | 308.5 | 8.36507416 |
| 308.6 | 3.53039472 | 308.6 | 12.7682609 | 308.6 | 12.7682609 | 308.6 | 9.24767284 | | | | | 308.60 | 8.3552675 | | | 308.6 | 8.3552675 |
| 308.7 | 3.54020137 | 308.7 | 12.7682609 | 308.7 | 12.7682609 | 308.7 | 9.22805953 | | | | | 308.70 | 8.37488081 | | | 308.7 | 8.37488081 |
| 308.8 | 3.52058807 | 308.8 | 12.7976809 | 308.8 | 12.7976809 | 308.8 | 9.22805953 | | | | | 308.80 | 8.38468746 | | | 308.8 | 8.38468746 |
| 308.9 | 3.53039472 | 308.9 | 12.8074875 | 308.9 | 12.8074875 | 308.9 | 9.21825288 | | | | | 308.90 | 8.41410742 | | | 308.9 | 8.41410742 |
| 309 | 3.51078142 | 309 | 12.8172942 | 309 | 12.8172942 | 309 | 9.20844623 | | | | | 309.00 | 8.40430076 | | | 309 | 8.40430076 |
| 309.1 | 3.55000802 | 309.1 | 12.8271008 | 309.1 | 12.8271008 | 309.1 | 9.21825288 | | | | | 309.10 | 8.40430076 | | | 309.1 | 8.40430076 |
| 309.2 | 3.53039472 | 309.2 | 12.8369075 | 309.2 | 12.8369075 | 309.2 | 9.23786618 | | | | | 309.20 | 8.39449411 | | | 309.2 | 8.39449411 |
| 309.3 | 3.52058807 | 309.3 | 12.8565208 | 309.3 | 12.8565208 | 309.3 | 9.24767284 | | | | | 309.30 | 8.40430076 | | | 309.3 | 8.40430076 |
| 309.4 | 3.52058807 | 309.4 | 12.8565208 | 309.4 | 12.8565208 | 309.4 | 9.22805953 | | | | | 309.40 | 8.42391407 | | | 309.4 | 8.42391407 |
| 309.5 | 3.53039472 | 309.5 | 12.8467141 | 309.5 | 12.8467141 | 309.5 | 9.24767284 | | | | | 309.50 | 8.39449411 | | | 309.5 | 8.39449411 |
| 309.6 | 3.53039472 | 309.6 | 12.8663274 | 309.6 | 12.8663274 | 309.6 | 9.26728614 | | | | | 309.60 | 8.40430076 | | | 309.6 | 8.40430076 |
| 309.7 | 3.55000802 | 309.7 | 12.8565208 | 309.7 | 12.8565208 | 309.7 | 9.26728614 | | | | | 309.70 | 8.41410742 | | | 309.7 | 8.41410742 |
| 309.8 | 3.55000802 | 309.8 | 12.8761341 | 309.8 | 12.8761341 | 309.8 | 9.26728614 | | | | | 309.80 | 8.38468746 | | | 309.8 | 8.38468746 |
| 309.9 | 3.55981468 | 309.9 | 12.8761341 | 309.9 | 12.8761341 | 309.9 | 9.26728614 | | | | | 309.90 | 8.39449411 | | | 309.9 | 8.39449411 |
| 310 | 3.55000802 | 310 | 12.8957474 | 310 | 12.8957474 | 310 | 9.24767284 | | | | | 310.00 | 8.42391407 | | | 310 | 8.42391407 |
| 310.1 | 3.56962133 | 310.1 | 12.9153607 | 310.1 | 12.9153607 | 310.1 | 9.25747949 | | | | | 310.10 | 8.42391407 | | | 310.1 | 8.42391407 |
| 310.2 | 3.55981468 | 310.2 | 12.9251673 | 310.2 | 12.9251673 | 310.2 | 9.26728614 | | | | | 310.20 | 8.41410742 | | | 310.2 | 8.41410742 |
| 310.3 | 3.57942798 | 310.3 | 12.905554 | 310.3 | 12.905554 | 310.3 | 9.28689944 | | | | | 310.30 | 8.42391407 | | | 310.3 | 8.42391407 |
| 310.4 | 3.59904128 | 310.4 | 12.8761341 | 310.4 | 12.8761341 | 310.4 | 9.3163194 | | | | | 310.40 | 8.43372072 | | | 310.4 | 8.43372072 |
| 310.5 | 3.57942798 | 310.5 | 12.8761341 | 310.5 | 12.8761341 | 310.5 | 9.32612605 | | | | | 310.50 | 8.43372072 | | | 310.5 | 8.43372072 |
| 310.6 | 3.55000802 | 310.6 | 12.9251673 | 310.6 | 12.9251673 | 310.6 | 9.3359327 | | | | | 310.60 | 8.43372072 | | | 310.6 | 8.43372072 |
| 310.7 | 3.55981468 | 310.7 | 12.9251673 | 310.7 | 12.9251673 | 310.7 | 9.3359327 | | | | | 310.70 | 8.42391407 | | | 310.7 | 8.42391407 |
| 310.8 | 3.56962133 | 310.8 | 12.9251673 | 310.8 | 12.9251673 | 310.8 | 9.32612605 | | | | | 310.80 | 8.40430076 | | | 310.8 | 8.40430076 |
| 310.9 | 3.58923463 | 310.9 | 12.9742006 | 310.9 | 12.9742006 | 310.9 | 9.2967061 | | | | | 310.90 | 8.40430076 | | | 310.9 | 8.40430076 |
| 311 | 3.57942798 | 311 | 12.934974 | 311 | 12.934974 | 311 | 9.30651275 | | | | | 311.00 | 8.39449411 | | | 311 | 8.39449411 |
| 311.1 | 3.55981468 | 311.1 | 12.9251673 | 311.1 | 12.9251673 | 311.1 | 9.2967061 | | | | | 311.10 | 8.38468746 | | | 311.1 | 8.38468746 |
| 311.2 | 3.57942798 | 311.2 | 12.9545873 | 311.2 | 12.9545873 | 311.2 | 9.30651275 | | | | | 311.20 | 8.39449411 | | | 311.2 | 8.39449411 |
| 311.3 | 3.56962133 | 311.3 | 12.9447806 | 311.3 | 12.9447806 | 311.3 | 9.30651275 | | | | | 311.30 | 8.37488081 | | | 311.3 | 8.37488081 |
| 311.4 | 3.60884794 | 311.4 | 13.0036206 | 311.4 | 13.0036206 | 311.4 | 9.3163194 | | | | | 311.40 | 8.38468746 | | | 311.4 | 8.38468746 |
| 311.5 | 3.58923463 | 311.5 | 12.9840072 | 311.5 | 12.9840072 | 311.5 | 9.3359327 | | | | | 311.50 | 8.38468746 | | | 311.5 | 8.38468746 |
| 311.6 | 3.59904128 | 311.6 | 13.0036206 | 311.6 | 13.0036206 | 311.6 | 9.3359327 | | | | | 311.60 | 8.38468746 | | | 311.6 | 8.38468746 |
| 311.7 | 3.61865459 | 311.7 | 12.9938139 | 311.7 | 12.9938139 | 311.7 | 9.32612605 | | | | | 311.70 | 8.40430076 | | | 311.7 | 8.40430076 |
| 311.8 | 3.60884794 | 311.8 | 12.9840072 | 311.8 | 12.9840072 | 311.8 | 9.32612605 | | | | | 311.80 | 8.39449411 | | | 311.8 | 8.39449411 |
| 311.9 | 3.60884794 | 311.9 | 13.0134272 | 311.9 | 13.0134272 | 311.9 | 9.34573936 | | | | | 311.90 | 8.38468746 | | | 311.9 | 8.38468746 |
| 312 | 3.63826789 | 312 | 12.9938139 | 312 | 12.9938139 | 312 | 9.37515931 | | | | | 312.00 | 8.34546085 | | | 312 | 8.34546085 |
| 312.1 | 3.60884794 | 312.1 | 12.9938139 | 312.1 | 12.9938139 | 312.1 | 9.35554601 | | | | | 312.10 | 8.32584755 | | | 312.1 | 8.32584755 |
| 312.2 | 3.58923463 | 312.2 | 13.0134272 | 312.2 | 13.0134272 | 312.2 | 9.34573936 | | | | | 312.20 | 8.3356542 | | | 312.2 | 8.3356542 |
| 312.3 | 3.59904128 | 312.3 | 13.0036206 | 312.3 | 13.0036206 | 312.3 | 9.36535266 | | | | | 312.30 | 8.34546085 | | | 312.3 | 8.34546085 |
| 312.4 | 3.57942798 | 312.4 | 13.0330405 | 312.4 | 13.0330405 | 312.4 | 9.36535266 | | | | | 312.40 | 8.3160409 | | | 312.4 | 8.3160409 |
| 312.5 | 3.57942798 | 312.5 | 13.0330405 | 312.5 | 13.0330405 | 312.5 | 9.36535266 | | | | | 312.50 | 8.26700764 | | | 312.5 | 8.26700764 |
| 312.6 | 3.58923463 | 312.6 | 13.0624605 | 312.6 | 13.0624605 | 312.6 | 9.36535266 | | | | | 312.60 | 8.20816772 | | | 312.6 | 8.20816772 |
| 312.7 | 3.57942798 | 312.7 | 13.0820738 | 312.7 | 13.0820738 | 312.7 | 9.38496596 | | | | | 312.70 | 8.22778103 | | | 312.7 | 8.22778103 |
| 312.8 | 3.59904128 | 312.8 | 13.1016871 | 312.8 | 13.1016871 | 312.8 | 9.38496596 | | | | | 312.80 | 8.22778103 | | | 312.8 | 8.22778103 |
| 312.9 | 3.60884794 | 312.9 | 13.1114937 | 312.9 | 13.1114937 | 312.9 | 9.36535266 | | | | | 312.90 | 8.25720098 | | | 312.9 | 8.25720098 |
| 313 | 3.60884794 | 313 | 13.1016871 | 313 | 13.1016871 | 313 | 9.38496596 | | | | | 313.00 | 8.28662094 | | | 313 | 8.28662094 |
| 313.1 | 3.60884794 | 313.1 | 13.1016871 | 313.1 | 13.1016871 | 313.1 | 9.36535266 | | | | | 313.10 | 8.28662094 | | | 313.1 | 8.28662094 |
| 313.2 | 3.62846124 | 313.2 | 13.1016871 | 313.2 | 13.1016871 | 313.2 | 9.35554601 | | | | | 313.20 | 8.27681429 | | | 313.2 | 8.27681429 |
| 313.3 | 3.63826789 | 313.3 | 13.1114937 | 313.3 | 13.1114937 | 313.3 | 9.36535266 | | | | | 313.30 | 8.24739433 | | | 313.3 | 8.24739433 |
| 313.4 | 3.60884794 | 313.4 | 13.131107 | 313.4 | 13.131107 | 313.4 | 9.37515931 | | | | | 313.40 | 8.21797438 | | | 313.4 | 8.21797438 |
| 313.5 | 3.61865459 | 313.5 | 13.1213004 | 313.5 | 13.1213004 | 313.5 | 9.37515931 | | | | | 313.50 | 8.22778103 | | | 313.5 | 8.22778103 |
| 313.6 | 3.59904128 | 313.6 | 13.131107 | 313.6 | 13.131107 | 313.6 | 9.37515931 | | | | | 313.60 | 8.25720098 | | | 313.6 | 8.25720098 |
| 313.7 | 3.60884794 | 313.7 | 13.160527 | 313.7 | 13.160527 | 313.7 | 9.37515931 | | | | | 313.70 | 8.25720098 | | | 313.7 | 8.25720098 |
| 313.8 | 3.63826789 | 313.8 | 13.131107 | 313.8 | 13.131107 | 313.8 | 9.37515931 | | | | | 313.80 | 8.24739433 | | | 313.8 | 8.24739433 |
| 313.9 | 3.64807454 | 313.9 | 13.160527 | 313.9 | 13.160527 | 313.9 | 9.37515931 | | | | | 313.90 | 8.25720098 | | | 313.9 | 8.25720098 |
| 314 | 3.66768785 | 314 | 13.1507203 | 314 | 13.1507203 | 314 | 9.38496596 | | | | | 314.00 | 8.24739433 | | | 314 | 8.24739433 |
| 314.1 | 3.66768785 | 314.1 | 13.1899469 | 314.1 | 13.1899469 | 314.1 | 9.40457927 | | | | | 314.10 | 8.25720098 | | | 314.1 | 8.25720098 |
| 314.2 | 3.64807454 | 314.2 | 13.2095602 | 314.2 | 13.2095602 | 314.2 | 9.38496596 | | | | | 314.20 | 8.26700764 | | | 314.2 | 8.26700764 |
| 314.3 | 3.63826789 | 314.3 | 13.1899469 | 314.3 | 13.1899469 | 314.3 | 9.37515931 | | | | | 314.30 | 8.29642759 | | | 314.3 | 8.29642759 |
| 314.4 | 3.61865459 | 314.4 | 13.1997536 | 314.4 | 13.1997536 | 314.4 | 9.35554601 | | | | | 314.40 | 8.32584755 | | | 314.4 | 8.32584755 |

| | | | | | | | | | | | | | | | | | |
|---|---|---|---|---|---|---|---|---|---|---|---|---|---|---|---|---|---|
| 314.5 | 3.63826789 | 314.5 | 13.2193669 | 314.5 | 13.2193669 | 314.5 | 9.36535266 | | | | | 314.50 | 8.29642759 | | | 314.5 | 8.29642759 |
| 314.6 | 3.66768785 | 314.6 | 13.2389802 | 314.6 | 13.2389802 | 314.6 | 9.38496596 | | | | | 314.60 | 8.26700764 | | | 314.6 | 8.26700764 |
| 314.7 | 3.64807454 | 314.7 | 13.2585935 | 314.7 | 13.2585935 | 314.7 | 9.40457927 | | | | | 314.70 | 8.26700764 | | | 314.7 | 8.26700764 |
| 314.8 | 3.61865459 | 314.8 | 13.2487869 | 314.8 | 13.2487869 | 314.8 | 9.40457927 | | | | | 314.80 | 8.30623424 | | | 314.8 | 8.30623424 |
| 314.9 | 3.63826789 | 314.9 | 13.2291735 | 314.9 | 13.2291735 | 314.9 | 9.41438592 | | | | | 314.90 | 8.32584755 | | | 314.9 | 8.32584755 |
| 315 | 3.6774945 | 315 | 13.2684002 | 315 | 13.2684002 | 315 | 9.42419257 | | | | | 315.00 | 8.32584755 | | | 315 | 8.32584755 |
| 315.1 | 3.6578812 | 315.1 | 13.2684002 | 315.1 | 13.2684002 | 315.1 | 9.39477262 | | | | | 315.10 | 8.3160409 | | | 315.1 | 8.3160409 |
| 315.2 | 3.6578812 | 315.2 | 13.3076268 | 315.2 | 13.3076268 | 315.2 | 9.40457927 | | | | | 315.20 | 8.28662094 | | | 315.2 | 8.28662094 |
| 315.3 | 3.66768785 | 315.3 | 13.2978201 | 315.3 | 13.2978201 | 315.3 | 9.41438592 | | | | | 315.30 | 8.26700764 | | | 315.3 | 8.26700764 |
| 315.4 | 3.66768785 | 315.4 | 13.2880135 | 315.4 | 13.2880135 | 315.4 | 9.43399922 | | | | | 315.40 | 8.28662094 | | | 315.4 | 8.28662094 |
| 315.5 | 3.6578812 | 315.5 | 13.3174334 | 315.5 | 13.3174334 | 315.5 | 9.43399922 | | | | | 315.50 | 8.3356542 | | | 315.5 | 8.3356542 |
| 315.6 | 3.66768785 | 315.6 | 13.3174334 | 315.6 | 13.3174334 | 315.6 | 9.41438592 | | | | | 315.60 | 8.34546085 | | | 315.6 | 8.34546085 |
| 315.7 | 3.6774945 | 315.7 | 13.3272401 | 315.7 | 13.3272401 | 315.7 | 9.45361253 | | | | | 315.70 | 8.34546085 | | | 315.7 | 8.34546085 |
| 315.8 | 3.66768785 | 315.8 | 13.3370467 | 315.8 | 13.3370467 | 315.8 | 9.47322583 | | | | | 315.80 | 8.3160409 | | | 315.8 | 8.3160409 |
| 315.9 | 3.66768785 | 315.9 | 13.3468534 | 315.9 | 13.3468534 | 315.9 | 9.44380588 | | | | | 315.90 | 8.32584755 | | | 315.9 | 8.32584755 |
| 316 | 3.66768785 | 316 | 13.3664667 | 316 | 13.3664667 | 316 | 9.47322583 | | | | | 316.00 | 8.3160409 | | | 316 | 8.3160409 |
| 316.1 | 3.6774945 | 316.1 | 13.3664667 | 316.1 | 13.3664667 | 316.1 | 9.46341918 | | | | | 316.10 | 8.34546085 | | | 316.1 | 8.34546085 |
| 316.2 | 3.66768785 | 316.2 | 13.35666 | 316.2 | 13.35666 | 316.2 | 9.45361253 | | | | | 316.20 | 8.37488081 | | | 316.2 | 8.37488081 |
| 316.3 | 3.66768785 | 316.3 | 13.38608 | 316.3 | 13.38608 | 316.3 | 9.46341918 | | | | | 316.30 | 8.34546085 | | | 316.3 | 8.34546085 |
| 316.4 | 3.71672111 | 316.4 | 13.3664667 | 316.4 | 13.3664667 | 316.4 | 9.43399922 | | | | | 316.40 | 8.34546085 | | | 316.4 | 8.34546085 |
| 316.5 | 3.70691446 | 316.5 | 13.35666 | 316.5 | 13.35666 | 316.5 | 9.43399922 | | | | | 316.50 | 8.34546085 | | | 316.5 | 8.34546085 |
| 316.6 | 3.6971078 | 316.6 | 13.3958866 | 316.6 | 13.3958866 | 316.6 | 9.46341918 | | | | | 316.60 | 8.37488081 | | | 316.6 | 8.37488081 |
| 316.7 | 3.6971078 | 316.7 | 13.4056933 | 316.7 | 13.4056933 | 316.7 | 9.46341918 | | | | | 316.70 | 8.37488081 | | | 316.7 | 8.37488081 |
| 316.8 | 3.66768785 | 316.8 | 13.4253066 | 316.8 | 13.4253066 | 316.8 | 9.45361253 | | | | | 316.80 | 8.36507416 | | | 316.8 | 8.36507416 |
| 316.9 | 3.6971078 | 316.9 | 13.4253066 | 316.9 | 13.4253066 | 316.9 | 9.43399922 | | | | | 316.90 | 8.38468746 | | | 316.9 | 8.38468746 |
| 317 | 3.73633441 | 317 | 13.4449199 | 317 | 13.4449199 | 317 | 9.45361253 | | | | | 317.00 | 8.39449411 | | | 317 | 8.39449411 |
| 317.1 | 3.72652776 | 317.1 | 13.3958866 | 317.1 | 13.3958866 | 317.1 | 9.46341918 | | | | | 317.10 | 8.37488081 | | | 317.1 | 8.37488081 |
| 317.2 | 3.73633441 | 317.2 | 13.4351132 | 317.2 | 13.4351132 | 317.2 | 9.46341918 | | | | | 317.20 | 8.3552675 | | | 317.2 | 8.3552675 |
| 317.3 | 3.72652776 | 317.3 | 13.4253066 | 317.3 | 13.4253066 | 317.3 | 9.47322583 | | | | | 317.30 | 8.36507416 | | | 317.3 | 8.36507416 |
| 317.4 | 3.77556102 | 317.4 | 13.4351132 | 317.4 | 13.4351132 | 317.4 | 9.50264579 | | | | | 317.40 | 8.40430076 | | | 317.4 | 8.40430076 |
| 317.5 | 3.75594772 | 317.5 | 13.4645332 | 317.5 | 13.4645332 | 317.5 | 9.47322583 | | | | | 317.50 | 8.39449411 | | | 317.5 | 8.39449411 |
| 317.6 | 3.74614106 | 317.6 | 13.4449199 | 317.6 | 13.4449199 | 317.6 | 9.46341918 | | | | | 317.60 | 8.3552675 | | | 317.6 | 8.3552675 |
| 317.7 | 3.76575437 | 317.7 | 13.4253066 | 317.7 | 13.4253066 | 317.7 | 9.48303248 | | | | | 317.70 | 8.34546085 | | | 317.7 | 8.34546085 |
| 317.8 | 3.76575437 | 317.8 | 13.4547265 | 317.8 | 13.4547265 | 317.8 | 9.49283914 | | | | | 317.80 | 8.37488081 | | | 317.8 | 8.37488081 |
| 317.9 | 3.73633441 | 317.9 | 13.4547265 | 317.9 | 13.4547265 | 317.9 | 9.48303248 | | | | | 317.90 | 8.38468746 | | | 317.9 | 8.38468746 |
| 318 | 3.74614106 | 318 | 13.4841465 | 318 | 13.4841465 | 318 | 9.47322583 | | | | | 318.00 | 8.36507416 | | | 318 | 8.36507416 |
| 318.1 | 3.73633441 | 318.1 | 13.5037598 | 318.1 | 13.5037598 | 318.1 | 9.46341918 | | | | | 318.10 | 8.38468746 | | | 318.1 | 8.38468746 |
| 318.2 | 3.73633441 | 318.2 | 13.4939532 | 318.2 | 13.4939532 | 318.2 | 9.47322583 | | | | | 318.20 | 8.40430076 | | | 318.2 | 8.40430076 |
| 318.3 | 3.73633441 | 318.3 | 13.4743398 | 318.3 | 13.4743398 | 318.3 | 9.46341918 | | | | | 318.30 | 8.41410742 | | | 318.3 | 8.41410742 |
| 318.4 | 3.74614106 | 318.4 | 13.5037598 | 318.4 | 13.5037598 | 318.4 | 9.47322583 | | | | | 318.40 | 8.3552675 | | | 318.4 | 8.3552675 |
| 318.5 | 3.77556102 | 318.5 | 13.5135665 | 318.5 | 13.5135665 | 318.5 | 9.47322583 | | | | | 318.50 | 8.40430076 | | | 318.5 | 8.40430076 |
| 318.6 | 3.76575437 | 318.6 | 13.5625997 | 318.6 | 13.5625997 | 318.6 | 9.50264579 | | | | | 318.60 | 8.40430076 | | | 318.6 | 8.40430076 |
| 318.7 | 3.76575437 | 318.7 | 13.5625997 | 318.7 | 13.5625997 | 318.7 | 9.50264579 | | | | | 318.70 | 8.42391407 | | | 318.7 | 8.42391407 |
| 318.8 | 3.78536767 | 318.8 | 13.5527931 | 318.8 | 13.5527931 | 318.8 | 9.50264579 | | | | | 318.80 | 8.42391407 | | | 318.8 | 8.42391407 |
| 318.9 | 3.76575437 | 318.9 | 13.5625997 | 318.9 | 13.5625997 | 318.9 | 9.50264579 | | | | | 318.90 | 8.41410742 | | | 318.9 | 8.41410742 |
| 319 | 3.75594772 | 319 | 13.582213 | 319 | 13.582213 | 319 | 9.50264579 | | | | | 319.00 | 8.45333402 | | | 319 | 8.45333402 |
| 319.1 | 3.78536767 | 319.1 | 13.582213 | 319.1 | 13.582213 | 319.1 | 9.50264579 | | | | | 319.10 | 8.45333402 | | | 319.1 | 8.45333402 |
| 319.2 | 3.80498098 | 319.2 | 13.5920197 | 319.2 | 13.5920197 | 319.2 | 9.52225909 | | | | | 319.20 | 8.44352737 | | | 319.2 | 8.44352737 |
| 319.3 | 3.80498098 | 319.3 | 13.6018263 | 319.3 | 13.6018263 | 319.3 | 9.53206574 | | | | | 319.30 | 8.45333402 | | | 319.3 | 8.45333402 |
| 319.4 | 3.78536767 | 319.4 | 13.582213 | 319.4 | 13.582213 | 319.4 | 9.52225909 | | | | | 319.40 | 8.44352737 | | | 319.4 | 8.44352737 |
| 319.5 | 3.78536767 | 319.5 | 13.5724064 | 319.5 | 13.5724064 | 319.5 | 9.50264579 | | | | | 319.50 | 8.47294733 | | | 319.5 | 8.47294733 |
| 319.6 | 3.77556102 | 319.6 | 13.611633 | 319.6 | 13.611633 | 319.6 | 9.49283914 | | | | | 319.60 | 8.48275398 | | | 319.6 | 8.48275398 |
| 319.7 | 3.76575437 | 319.7 | 13.6214396 | 319.7 | 13.6214396 | 319.7 | 9.49283914 | | | | | 319.70 | 8.46314068 | | | 319.7 | 8.46314068 |
| 319.8 | 3.81478763 | 319.8 | 13.6508596 | 319.8 | 13.6508596 | 319.8 | 9.52225909 | | | | | 319.80 | 8.48275398 | | | 319.8 | 8.48275398 |
| 319.9 | 3.77556102 | 319.9 | 13.6410529 | 319.9 | 13.6410529 | 319.9 | 9.47322583 | | | | | 319.90 | 8.49256063 | | | 319.9 | 8.49256063 |
| 320 | 3.79517432 | 320 | 13.6410529 | 320 | 13.6410529 | 320 | 9.50264579 | | | | | 320.00 | 8.46314068 | | | 320 | 8.46314068 |
| 320.1 | 3.81478763 | 320.1 | 13.6508596 | 320.1 | 13.6508596 | 320.1 | 9.5418724 | | | | | 320.10 | 8.48275398 | | | 320.1 | 8.48275398 |
| 320.2 | 3.78536767 | 320.2 | 13.6802795 | 320.2 | 13.6802795 | 320.2 | 9.51245244 | | | | | 320.20 | 8.49256063 | | | 320.2 | 8.49256063 |
| 320.3 | 3.77556102 | 320.3 | 13.6802795 | 320.3 | 13.6802795 | 320.3 | 9.50264579 | | | | | 320.30 | 8.49256063 | | | 320.3 | 8.49256063 |
| 320.4 | 3.78536767 | 320.4 | 13.6704729 | 320.4 | 13.6704729 | 320.4 | 9.51245244 | | | | | 320.40 | 8.49256063 | | | 320.4 | 8.49256063 |
| 320.5 | 3.79517432 | 320.5 | 13.6606662 | 320.5 | 13.6606662 | 320.5 | 9.51245244 | | | | | 320.50 | 8.48275398 | | | 320.5 | 8.48275398 |

| | | | | | | | | | | | | | | | | | |
|---|---|---|---|---|---|---|---|---|---|---|---|---|---|---|---|---|---|
| 320.6 | 3.82459428 | 320.6 | 13.6998928 | 320.6 | 13.6998928 | 320.6 | 9.5418724 | | | | | 320.60 | 8.48275398 | | | 320.6 | 8.48275398 |
| 320.7 | 3.80498098 | 320.7 | 13.6802795 | 320.7 | 13.6802795 | 320.7 | 9.5418724 | | | | | 320.70 | 8.47294733 | | | 320.7 | 8.47294733 |
| 320.8 | 3.80498098 | 320.8 | 13.6802795 | 320.8 | 13.6802795 | 320.8 | 9.5418724 | | | | | 320.80 | 8.51217394 | | | 320.8 | 8.51217394 |
| 320.9 | 3.79517432 | 320.9 | 13.7293128 | 320.9 | 13.7293128 | 320.9 | 9.5418724 | | | | | 320.90 | 8.52198059 | | | 320.9 | 8.52198059 |
| 321 | 3.82459428 | 321 | 13.7195061 | 321 | 13.7195061 | 321 | 9.5418724 | | | | | 321.00 | 8.48275398 | | | 321 | 8.48275398 |
| 321.1 | 3.79517432 | 321.1 | 13.7293128 | 321.1 | 13.7293128 | 321.1 | 9.52225909 | | | | | 321.10 | 8.47294733 | | | 321.1 | 8.47294733 |
| 321.2 | 3.79517432 | 321.2 | 13.7489261 | 321.2 | 13.7489261 | 321.2 | 9.5418724 | | | | | 321.20 | 8.50236728 | | | 321.2 | 8.50236728 |
| 321.3 | 3.81478763 | 321.3 | 13.7489261 | 321.3 | 13.7489261 | 321.3 | 9.55167905 | | | | | 321.30 | 8.51217394 | | | 321.3 | 8.51217394 |
| 321.4 | 3.82459428 | 321.4 | 13.7685394 | 321.4 | 13.7685394 | 321.4 | 9.55167905 | | | | | 321.40 | 8.53178724 | | | 321.4 | 8.53178724 |
| 321.5 | 3.84420758 | 321.5 | 13.7293128 | 321.5 | 13.7293128 | 321.5 | 9.5418724 | | | | | 321.50 | 8.54159389 | | | 321.5 | 8.54159389 |
| 321.6 | 3.84420758 | 321.6 | 13.7096995 | 321.6 | 13.7096995 | 321.6 | 9.55167905 | | | | | 321.60 | 8.53178724 | | | 321.6 | 8.53178724 |
| 321.7 | 3.85401424 | 321.7 | 13.7096995 | 321.7 | 13.7096995 | 321.7 | 9.55167905 | | | | | 321.70 | 8.51217394 | | | 321.7 | 8.51217394 |
| 321.8 | 3.84420758 | 321.8 | 13.7195061 | 321.8 | 13.7195061 | 321.8 | 9.5418724 | | | | | 321.80 | 8.54159389 | | | 321.8 | 8.54159389 |
| 321.9 | 3.81478763 | 321.9 | 13.7685394 | 321.9 | 13.7685394 | 321.9 | 9.52225909 | | | | | 321.90 | 8.52198059 | | | 321.9 | 8.52198059 |
| 322 | 3.83440093 | 322 | 13.7685394 | 322 | 13.7685394 | 322 | 9.51245244 | | | | | 322.00 | 8.5612072 | | | 322 | 8.5612072 |
| 322.1 | 3.85401424 | 322.1 | 13.7783461 | 322.1 | 13.7783461 | 322.1 | 9.47322583 | | | | | 322.10 | 8.5612072 | | | 322.1 | 8.5612072 |
| 322.2 | 3.81478763 | 322.2 | 13.7979594 | 322.2 | 13.7979594 | 322.2 | 9.46341918 | | | | | 322.20 | 8.51217394 | | | 322.2 | 8.51217394 |
| 322.3 | 3.83440093 | 322.3 | 13.8567993 | 322.3 | 13.8567993 | 322.3 | 9.47322583 | | | | | 322.30 | 8.52198059 | | | 322.3 | 8.52198059 |
| 322.4 | 3.85401424 | 322.4 | 13.8764126 | 322.4 | 13.8764126 | 322.4 | 9.49283914 | | | | | 322.40 | 8.54159389 | | | 322.4 | 8.54159389 |
| 322.5 | 3.86382089 | 322.5 | 13.8469926 | 322.5 | 13.8469926 | 322.5 | 9.47322583 | | | | | 322.50 | 8.52198059 | | | 322.5 | 8.52198059 |
| 322.6 | 3.85401424 | 322.6 | 13.837186 | 322.6 | 13.837186 | 322.6 | 9.49283914 | | | | | 322.60 | 8.54159389 | | | 322.6 | 8.54159389 |
| 322.7 | 3.82459428 | 322.7 | 13.8469926 | 322.7 | 13.8469926 | 322.7 | 9.50264579 | | | | | 322.70 | 8.5612072 | | | 322.7 | 8.5612072 |
| 322.8 | 3.82459428 | 322.8 | 13.8469926 | 322.8 | 13.8469926 | 322.8 | 9.48303248 | | | | | 322.80 | 8.55140054 | | | 322.8 | 8.55140054 |
| 322.9 | 3.84420758 | 322.9 | 13.837186 | 322.9 | 13.837186 | 322.9 | 9.49283914 | | | | | 322.90 | 8.53178724 | | | 322.9 | 8.53178724 |
| 323 | 3.85401424 | 323 | 13.8469926 | 323 | 13.8469926 | 323 | 9.52225909 | | | | | 323.00 | 8.52198059 | | | 323 | 8.52198059 |
| 323.1 | 3.86382089 | 323.1 | 13.8469926 | 323.1 | 13.8469926 | 323.1 | 9.51245244 | | | | | 323.10 | 8.59062715 | | | 323.1 | 8.59062715 |
| 323.2 | 3.86382089 | 323.2 | 13.8469926 | 323.2 | 13.8469926 | 323.2 | 9.53206574 | | | | | 323.20 | 8.6004338 | | | 323.2 | 8.6004338 |
| 323.3 | 3.84420758 | 323.3 | 13.8862192 | 323.3 | 13.8862192 | 323.3 | 9.5418724 | | | | | 323.30 | 8.59062715 | | | 323.3 | 8.59062715 |
| 323.4 | 3.86382089 | 323.4 | 13.8764126 | 323.4 | 13.8764126 | 323.4 | 9.5418724 | | | | | 323.40 | 8.6004338 | | | 323.4 | 8.6004338 |
| 323.5 | 3.87362754 | 323.5 | 13.8862192 | 323.5 | 13.8862192 | 323.5 | 9.5418724 | | | | | 323.50 | 8.62004711 | | | 323.5 | 8.62004711 |
| 323.6 | 3.86382089 | 323.6 | 13.9156392 | 323.6 | 13.9156392 | 323.6 | 9.5418724 | | | | | 323.60 | 8.6004338 | | | 323.6 | 8.6004338 |
| 323.7 | 3.85401424 | 323.7 | 13.9058325 | 323.7 | 13.9058325 | 323.7 | 9.51245244 | | | | | 323.70 | 8.61024046 | | | 323.7 | 8.61024046 |
| 323.8 | 3.86382089 | 323.8 | 13.8960259 | 323.8 | 13.8960259 | 323.8 | 9.53206574 | | | | | 323.80 | 8.62004711 | | | 323.8 | 8.62004711 |
| 323.9 | 3.85401424 | 323.9 | 13.8862192 | 323.9 | 13.8862192 | 323.9 | 9.51245244 | | | | | 323.90 | 8.62004711 | | | 323.9 | 8.62004711 |
| 324 | 3.88343419 | 324 | 13.9254458 | 324 | 13.9254458 | 324 | 9.49283914 | | | | | 324.00 | 8.59062715 | | | 324 | 8.59062715 |
| 324.1 | 3.88343419 | 324.1 | 13.9156392 | 324.1 | 13.9156392 | 324.1 | 9.51245244 | | | | | 324.10 | 8.62004711 | | | 324.1 | 8.62004711 |
| 324.2 | 3.87362754 | 324.2 | 13.9058325 | 324.2 | 13.9058325 | 324.2 | 9.53206574 | | | | | 324.20 | 8.63966041 | | | 324.2 | 8.63966041 |
| 324.3 | 3.9030475 | 324.3 | 13.9156392 | 324.3 | 13.9156392 | 324.3 | 9.53206574 | | | | | 324.30 | 8.62985376 | | | 324.3 | 8.62985376 |
| 324.4 | 3.88343419 | 324.4 | 13.9450591 | 324.4 | 13.9450591 | 324.4 | 9.51245244 | | | | | 324.40 | 8.61024046 | | | 324.4 | 8.61024046 |
| 324.5 | 3.89324084 | 324.5 | 13.9450591 | 324.5 | 13.9450591 | 324.5 | 9.53206574 | | | | | 324.50 | 8.62004711 | | | 324.5 | 8.62004711 |
| 324.6 | 3.89324084 | 324.6 | 13.9352525 | 324.6 | 13.9352525 | 324.6 | 9.5614857 | | | | | 324.60 | 8.62004711 | | | 324.6 | 8.62004711 |
| 324.7 | 3.9030475 | 324.7 | 13.9352525 | 324.7 | 13.9352525 | 324.7 | 9.55167905 | | | | | 324.70 | 8.63966041 | | | 324.7 | 8.63966041 |
| 324.8 | 3.93246745 | 324.8 | 13.9646724 | 324.8 | 13.9646724 | 324.8 | 9.5614857 | | | | | 324.80 | 8.65927372 | | | 324.8 | 8.65927372 |
| 324.9 | 3.9226608 | 324.9 | 13.9744791 | 324.9 | 13.9744791 | 324.9 | 9.55167905 | | | | | 324.90 | 8.65927372 | | | 324.9 | 8.65927372 |
| 325 | 3.9030475 | 325 | 13.9744791 | 325 | 13.9744791 | 325 | 9.5418724 | | | | | 325.00 | 8.63966041 | | | 325 | 8.63966041 |
| 325.1 | 3.9030475 | 325.1 | 13.9940924 | 325.1 | 13.9940924 | 325.1 | 9.55167905 | | | | | 325.10 | 8.62004711 | | | 325.1 | 8.62004711 |
| 325.2 | 3.91285415 | 325.2 | 13.9842858 | 325.2 | 13.9842858 | 325.2 | 9.55167905 | | | | | 325.20 | 8.64946706 | | | 325.2 | 8.64946706 |
| 325.3 | 3.93246745 | 325.3 | 14.0137057 | 325.3 | 14.0137057 | 325.3 | 9.5614857 | | | | | 325.30 | 8.65927372 | | | 325.3 | 8.65927372 |
| 325.4 | 3.93246745 | 325.4 | 14.0235124 | 325.4 | 14.0235124 | 325.4 | 9.57129235 | | | | | 325.40 | 8.67888702 | | | 325.4 | 8.67888702 |
| 325.5 | 3.9422741 | 325.5 | 14.0529323 | 325.5 | 14.0529323 | 325.5 | 9.57129235 | | | | | 325.50 | 8.65927372 | | | 325.5 | 8.65927372 |
| 325.6 | 3.9226608 | 325.6 | 14.0431257 | 325.6 | 14.0431257 | 325.6 | 9.53206574 | | | | | 325.60 | 8.64946706 | | | 325.6 | 8.64946706 |
| 325.7 | 3.9422741 | 325.7 | 14.033319 | 325.7 | 14.033319 | 325.7 | 9.53206574 | | | | | 325.70 | 8.66908037 | | | 325.7 | 8.66908037 |
| 325.8 | 3.9422741 | 325.8 | 14.033319 | 325.8 | 14.033319 | 325.8 | 9.5418724 | | | | | 325.80 | 8.69850032 | | | 325.8 | 8.69850032 |
| 325.9 | 3.95208076 | 325.9 | 14.033319 | 325.9 | 14.033319 | 325.9 | 9.5614857 | | | | | 325.90 | 8.68869367 | | | 325.9 | 8.68869367 |
| 326 | 3.97169406 | 326 | 14.0235124 | 326 | 14.0235124 | 326 | 9.5614857 | | | | | 326.00 | 8.67888702 | | | 326 | 8.67888702 |
| 326.1 | 3.97169406 | 326.1 | 14.0235124 | 326.1 | 14.0235124 | 326.1 | 9.57129235 | | | | | 326.10 | 8.67888702 | | | 326.1 | 8.67888702 |
| 326.2 | 3.9422741 | 326.2 | 14.0431257 | 326.2 | 14.0431257 | 326.2 | 9.581099 | | | | | 326.20 | 8.67888702 | | | 326.2 | 8.67888702 |
| 326.3 | 3.9226608 | 326.3 | 14.0725456 | 326.3 | 14.0725456 | 326.3 | 9.5614857 | | | | | 326.30 | 8.70830698 | | | 326.3 | 8.70830698 |
| 326.4 | 3.9226608 | 326.4 | 14.062739 | 326.4 | 14.062739 | 326.4 | 9.55167905 | | | | | 326.40 | 8.69850032 | | | 326.4 | 8.69850032 |
| 326.5 | 3.97169406 | 326.5 | 14.0725456 | 326.5 | 14.0725456 | 326.5 | 9.581099 | | | | | 326.50 | 8.69850032 | | | 326.5 | 8.69850032 |
| 326.6 | 3.97169406 | 326.6 | 14.1117722 | 326.6 | 14.1117722 | 326.6 | 9.581099 | | | | | 326.60 | 8.69850032 | | | 326.6 | 8.69850032 |

| | | | | | | | | | | | | | | | | | |
|---|---|---|---|---|---|---|---|---|---|---|---|---|---|---|---|---|---|
| 326.7 | 3.97169406 | 326.7 | 14.1117722 | 326.7 | 14.1117722 | 326.7 | 9.57129235 | | | | | 326.70 | 8.73772693 | | | 326.7 | 8.73772693 |
| 326.8 | 3.96188741 | 326.8 | 14.1509988 | 326.8 | 14.1509988 | 326.8 | 9.59090566 | | | | | 326.80 | 8.72792028 | | | 326.8 | 8.72792028 |
| 326.9 | 3.99130736 | 326.9 | 14.1706121 | 326.9 | 14.1706121 | 326.9 | 9.59090566 | | | | | 326.90 | 8.68869367 | | | 326.9 | 8.68869367 |
| 327 | 3.96188741 | 327 | 14.1706121 | 327 | 14.1706121 | 327 | 9.61051896 | | | | | 327.00 | 8.68869367 | | | 327 | 8.68869367 |
| 327.1 | 3.95208076 | 327.1 | 14.1313855 | 327.1 | 14.1313855 | 327.1 | 9.62032561 | | | | | 327.10 | 8.67888702 | | | 327.1 | 8.67888702 |
| 327.2 | 3.95208076 | 327.2 | 14.1411922 | 327.2 | 14.1411922 | 327.2 | 9.59090566 | | | | | 327.20 | 8.67888702 | | | 327.2 | 8.67888702 |
| 327.3 | 3.96188741 | 327.3 | 14.1608055 | 327.3 | 14.1608055 | 327.3 | 9.59090566 | | | | | 327.30 | 8.69850032 | | | 327.3 | 8.69850032 |
| 327.4 | 3.99130736 | 327.4 | 14.1608055 | 327.4 | 14.1608055 | 327.4 | 9.59090566 | | | | | 327.40 | 8.72792028 | | | 327.4 | 8.72792028 |
| 327.5 | 4.00111402 | 327.5 | 14.2000321 | 327.5 | 14.2000321 | 327.5 | 9.60071231 | | | | | 327.50 | 8.72792028 | | | 327.5 | 8.72792028 |
| 327.6 | 3.98150071 | 327.6 | 14.2098387 | 327.6 | 14.2098387 | 327.6 | 9.59090566 | | | | | 327.60 | 8.73772693 | | | 327.6 | 8.73772693 |
| 327.7 | 3.98150071 | 327.7 | 14.2294521 | 327.7 | 14.2294521 | 327.7 | 9.581099 | | | | | 327.70 | 8.71811363 | | | 327.7 | 8.71811363 |
| 327.8 | 4.00111402 | 327.8 | 14.2196454 | 327.8 | 14.2196454 | 327.8 | 9.581099 | | | | | 327.80 | 8.71811363 | | | 327.8 | 8.71811363 |
| 327.9 | 3.99130736 | 327.9 | 14.2000321 | 327.9 | 14.2000321 | 327.9 | 9.59090566 | | | | | 327.90 | 8.72792028 | | | 327.9 | 8.72792028 |
| 328 | 3.97169406 | 328 | 14.2000321 | 328 | 14.2000321 | 328 | 9.60071231 | | | | | 328.00 | 8.73772693 | | | 328 | 8.73772693 |
| 328.1 | 4.00111402 | 328.1 | 14.2196454 | 328.1 | 14.2196454 | 328.1 | 9.60071231 | | | | | 328.10 | 8.72792028 | | | 328.1 | 8.72792028 |
| 328.2 | 3.97169406 | 328.2 | 14.2490654 | 328.2 | 14.2490654 | 328.2 | 9.60071231 | | | | | 328.20 | 8.74753358 | | | 328.2 | 8.74753358 |
| 328.3 | 4.00111402 | 328.3 | 14.2490654 | 328.3 | 14.2490654 | 328.3 | 9.61051896 | | | | | 328.30 | 8.73772693 | | | 328.3 | 8.73772693 |
| 328.4 | 4.00111402 | 328.4 | 14.2686787 | 328.4 | 14.2686787 | 328.4 | 9.581099 | | | | | 328.40 | 8.73772693 | | | 328.4 | 8.73772693 |
| 328.5 | 3.99130736 | 328.5 | 14.2686787 | 328.5 | 14.2686787 | 328.5 | 9.57129235 | | | | | 328.50 | 8.74753358 | | | 328.5 | 8.74753358 |
| 328.6 | 4.02072732 | 328.6 | 14.258872 | 328.6 | 14.258872 | 328.6 | 9.59090566 | | | | | 328.60 | 8.74753358 | | | 328.6 | 8.74753358 |
| 328.7 | 4.01092067 | 328.7 | 14.258872 | 328.7 | 14.258872 | 328.7 | 9.60071231 | | | | | 328.70 | 8.76714689 | | | 328.7 | 8.76714689 |
| 328.8 | 4.02072732 | 328.8 | 14.2686787 | 328.8 | 14.2686787 | 328.8 | 9.60071231 | | | | | 328.80 | 8.76714689 | | | 328.8 | 8.76714689 |
| 328.9 | 4.01092067 | 328.9 | 14.2784853 | 328.9 | 14.2784853 | 328.9 | 9.62032561 | | | | | 328.90 | 8.74753358 | | | 328.9 | 8.74753358 |
| 329 | 3.98150071 | 329 | 14.3079053 | 329 | 14.3079053 | 329 | 9.60071231 | | | | | 329.00 | 8.74753358 | | | 329 | 8.74753358 |
| 329.1 | 3.99130736 | 329.1 | 14.2980986 | 329.1 | 14.2980986 | 329.1 | 9.60071231 | | | | | 329.10 | 8.77695354 | | | 329.1 | 8.77695354 |
| 329.2 | 4.01092067 | 329.2 | 14.3079053 | 329.2 | 14.3079053 | 329.2 | 9.61051896 | | | | | 329.20 | 8.78676019 | | | 329.2 | 8.78676019 |
| 329.3 | 4.01092067 | 329.3 | 14.3373252 | 329.3 | 14.3373252 | 329.3 | 9.61051896 | | | | | 329.30 | 8.77695354 | | | 329.3 | 8.77695354 |
| 329.4 | 4.00111402 | 329.4 | 14.3373252 | 329.4 | 14.3373252 | 329.4 | 9.581099 | | | | | 329.40 | 8.79656684 | | | 329.4 | 8.79656684 |
| 329.5 | 4.01092067 | 329.5 | 14.3177119 | 329.5 | 14.3177119 | 329.5 | 9.60071231 | | | | | 329.50 | 8.79656684 | | | 329.5 | 8.79656684 |
| 329.6 | 4.02072732 | 329.6 | 14.3177119 | 329.6 | 14.3177119 | 329.6 | 9.62032561 | | | | | 329.60 | 8.77695354 | | | 329.6 | 8.77695354 |
| 329.7 | 4.02072732 | 329.7 | 14.3471319 | 329.7 | 14.3471319 | 329.7 | 9.63013226 | | | | | 329.70 | 8.77695354 | | | 329.7 | 8.77695354 |
| 329.8 | 4.01092067 | 329.8 | 14.3569385 | 329.8 | 14.3569385 | 329.8 | 9.61051896 | | | | | 329.80 | 8.81618015 | | | 329.8 | 8.81618015 |
| 329.9 | 4.01092067 | 329.9 | 14.3569385 | 329.9 | 14.3569385 | 329.9 | 9.61051896 | | | | | 329.90 | 8.8063735 | | | 329.9 | 8.8063735 |
| 330 | 4.05995393 | 330 | 14.3667452 | 330 | 14.3667452 | 330 | 9.62032561 | | | | | 330.00 | 8.76714689 | | | 330 | 8.76714689 |
| 330.1 | 4.07956723 | 330.1 | 14.3373252 | 330.1 | 14.3373252 | 330.1 | 9.61051896 | | | | | 330.10 | 8.76714689 | | | 330.1 | 8.76714689 |
| 330.2 | 4.06976058 | 330.2 | 14.3569385 | 330.2 | 14.3569385 | 330.2 | 9.61051896 | | | | | 330.20 | 8.77695354 | | | 330.2 | 8.77695354 |
| 330.3 | 4.02072732 | 330.3 | 14.3765518 | 330.3 | 14.3765518 | 330.3 | 9.61051896 | | | | | 330.30 | 8.76714689 | | | 330.3 | 8.76714689 |
| 330.4 | 4.00111402 | 330.4 | 14.3863585 | 330.4 | 14.3863585 | 330.4 | 9.63013226 | | | | | 330.40 | 8.75734024 | | | 330.4 | 8.75734024 |
| 330.5 | 4.01092067 | 330.5 | 14.3863585 | 330.5 | 14.3863585 | 330.5 | 9.63013226 | | | | | 330.50 | 8.76714689 | | | 330.5 | 8.76714689 |
| 330.6 | 4.01092067 | 330.6 | 14.4255851 | 330.6 | 14.4255851 | 330.6 | 9.60071231 | | | | | 330.60 | 8.78676019 | | | 330.6 | 8.78676019 |
| 330.7 | 4.01092067 | 330.7 | 14.4353917 | 330.7 | 14.4353917 | 330.7 | 9.59090566 | | | | | 330.70 | 8.81618015 | | | 330.7 | 8.81618015 |
| 330.8 | 4.04034062 | 330.8 | 14.4157784 | 330.8 | 14.4157784 | 330.8 | 9.60071231 | | | | | 330.80 | 8.81618015 | | | 330.8 | 8.81618015 |
| 330.9 | 4.05014728 | 330.9 | 14.455005 | 330.9 | 14.455005 | 330.9 | 9.63013226 | | | | | 330.90 | 8.81618015 | | | 330.9 | 8.81618015 |
| 331 | 4.04034062 | 331 | 14.4746184 | 331 | 14.4746184 | 331 | 9.62032561 | | | | | 331.00 | 8.8063735 | | | 331 | 8.8063735 |
| 331.1 | 4.02072732 | 331.1 | 14.4451984 | 331.1 | 14.4451984 | 331.1 | 9.581099 | | | | | 331.10 | 8.79656684 | | | 331.1 | 8.79656684 |
| 331.2 | 4.05014728 | 331.2 | 14.4451984 | 331.2 | 14.4451984 | 331.2 | 9.59090566 | | | | | 331.20 | 8.81618015 | | | 331.2 | 8.81618015 |
| 331.3 | 4.06976058 | 331.3 | 14.455005 | 331.3 | 14.455005 | 331.3 | 9.61051896 | | | | | 331.30 | 8.8456001 | | | 331.3 | 8.8456001 |
| 331.4 | 4.07956723 | 331.4 | 14.5040383 | 331.4 | 14.5040383 | 331.4 | 9.60071231 | | | | | 331.40 | 8.8259868 | | | 331.4 | 8.8259868 |
| 331.5 | 4.05014728 | 331.5 | 14.5236516 | 331.5 | 14.5236516 | 331.5 | 9.63013226 | | | | | 331.50 | 8.81618015 | | | 331.5 | 8.81618015 |
| 331.6 | 4.04034062 | 331.6 | 14.484425 | 331.6 | 14.484425 | 331.6 | 9.62032561 | | | | | 331.60 | 8.86521341 | | | 331.6 | 8.86521341 |
| 331.7 | 4.05995393 | 331.7 | 14.4942317 | 331.7 | 14.4942317 | 331.7 | 9.60071231 | | | | | 331.70 | 8.83579345 | | | 331.7 | 8.83579345 |
| 331.8 | 4.05014728 | 331.8 | 14.513845 | 331.8 | 14.513845 | 331.8 | 9.59090566 | | | | | 331.80 | 8.8259868 | | | 331.8 | 8.8259868 |
| 331.9 | 4.05995393 | 331.9 | 14.5628782 | 331.9 | 14.5628782 | 331.9 | 9.62032561 | | | | | 331.90 | 8.83579345 | | | 331.9 | 8.83579345 |
| 332 | 4.05014728 | 332 | 14.5628782 | 332 | 14.5628782 | 332 | 9.63993892 | | | | | 332.00 | 8.8456001 | | | 332 | 8.8456001 |
| 332.1 | 4.06976058 | 332.1 | 14.5530716 | 332.1 | 14.5530716 | 332.1 | 9.62032561 | | | | | 332.10 | 8.8259868 | | | 332.1 | 8.8259868 |
| 332.2 | 4.06976058 | 332.2 | 14.5726849 | 332.2 | 14.5726849 | 332.2 | 9.61051896 | | | | | 332.20 | 8.8259868 | | | 332.2 | 8.8259868 |
| 332.3 | 4.04034062 | 332.3 | 14.5824915 | 332.3 | 14.5824915 | 332.3 | 9.60071231 | | | | | 332.30 | 8.8456001 | | | 332.3 | 8.8456001 |
| 332.4 | 4.07956723 | 332.4 | 14.5726849 | 332.4 | 14.5726849 | 332.4 | 9.62032561 | | | | | 332.40 | 8.8456001 | | | 332.4 | 8.8456001 |
| 332.5 | 4.10898719 | 332.5 | 14.5824915 | 332.5 | 14.5824915 | 332.5 | 9.63013226 | | | | | 332.50 | 8.87502006 | | | 332.5 | 8.87502006 |
| 332.6 | 4.09918054 | 332.6 | 14.5726849 | 332.6 | 14.5726849 | 332.6 | 9.63013226 | | | | | 332.60 | 8.89463336 | | | 332.6 | 8.89463336 |
| 332.7 | 4.07956723 | 332.7 | 14.5824915 | 332.7 | 14.5824915 | 332.7 | 9.63993892 | | | | | 332.70 | 8.89463336 | | | 332.7 | 8.89463336 |

| | | | | | | | | | | | | | | | | |
|---|---|---|---|---|---|---|---|---|---|---|---|---|---|---|---|---|
| 332.8 | 4.06976058 | 332.8 | 14.6021048 | 332.8 | 14.6021048 | 332.8 | 9.63993892 | | | | | 332.80 | 8.87502006 | | | 332.8 | 8.87502006 |
| 332.9 | 4.09918054 | 332.9 | 14.6119115 | 332.9 | 14.6119115 | 332.9 | 9.63993892 | | | | | 332.90 | 8.89463336 | | | 332.9 | 8.89463336 |
| 333 | 4.06976058 | 333 | 14.6315248 | 333 | 14.6315248 | 333 | 9.64974557 | | | | | 333.00 | 8.91424667 | | | 333 | 8.91424667 |
| 333.1 | 4.08937388 | 333.1 | 14.6119115 | 333.1 | 14.6119115 | 333.1 | 9.63993892 | | | | | 333.10 | 8.92405332 | | | 333.1 | 8.92405332 |
| 333.2 | 4.09918054 | 333.2 | 14.6413314 | 333.2 | 14.6413314 | 333.2 | 9.63013226 | | | | | 333.20 | 8.94366662 | | | 333.2 | 8.94366662 |
| 333.3 | 4.06976058 | 333.3 | 14.6609447 | 333.3 | 14.6609447 | 333.3 | 9.63013226 | | | | | 333.30 | 8.94366662 | | | 333.3 | 8.94366662 |
| 333.4 | 4.06976058 | 333.4 | 14.6315248 | 333.4 | 14.6315248 | 333.4 | 9.63013226 | | | | | 333.40 | 8.91424667 | | | 333.4 | 8.91424667 |
| 333.5 | 4.08937388 | 333.5 | 14.6511381 | 333.5 | 14.6511381 | 333.5 | 9.62032561 | | | | | 333.50 | 8.90444002 | | | 333.5 | 8.90444002 |
| 333.6 | 4.06976058 | 333.6 | 14.6707514 | 333.6 | 14.6707514 | 333.6 | 9.63993892 | | | | | 333.60 | 8.91424667 | | | 333.6 | 8.91424667 |
| 333.7 | 4.07956723 | 333.7 | 14.680558 | 333.7 | 14.680558 | 333.7 | 9.64974557 | | | | | 333.70 | 8.92405332 | | | 333.7 | 8.92405332 |
| 333.8 | 4.11879384 | 333.8 | 14.6903647 | 333.8 | 14.6903647 | 333.8 | 9.62032561 | | | | | 333.80 | 8.93385997 | | | 333.8 | 8.93385997 |
| 333.9 | 4.13840714 | 333.9 | 14.6707514 | 333.9 | 14.6707514 | 333.9 | 9.63013226 | | | | | 333.90 | 8.91424667 | | | 333.9 | 8.91424667 |
| 334 | 4.10898719 | 334 | 14.6707514 | 334 | 14.6707514 | 334 | 9.65955222 | | | | | 334.00 | 8.91424667 | | | 334 | 8.91424667 |
| 334.1 | 4.09918054 | 334.1 | 14.7001713 | 334.1 | 14.7001713 | 334.1 | 9.64974557 | | | | | 334.10 | 8.92405332 | | | 334.1 | 8.92405332 |
| 334.2 | 4.07956723 | 334.2 | 14.709978 | 334.2 | 14.709978 | 334.2 | 9.63013226 | | | | | 334.20 | 8.91424667 | | | 334.2 | 8.91424667 |
| 334.3 | 4.09918054 | 334.3 | 14.6903647 | 334.3 | 14.6903647 | 334.3 | 9.62032561 | | | | | 334.30 | 8.93385997 | | | 334.3 | 8.93385997 |
| 334.4 | 4.09918054 | 334.4 | 14.7001713 | 334.4 | 14.7001713 | 334.4 | 9.61051896 | | | | | 334.40 | 8.92405332 | | | 334.4 | 8.92405332 |
| 334.5 | 4.08937388 | 334.5 | 14.7197847 | 334.5 | 14.7197847 | 334.5 | 9.63013226 | | | | | 334.50 | 8.93385997 | | | 334.5 | 8.93385997 |
| 334.6 | 4.09918054 | 334.6 | 14.7295913 | 334.6 | 14.7295913 | 334.6 | 9.63993892 | | | | | 334.60 | 8.94366662 | | | 334.6 | 8.94366662 |
| 334.7 | 4.11879384 | 334.7 | 14.7492046 | 334.7 | 14.7492046 | 334.7 | 9.63993892 | | | | | 334.70 | 8.95347328 | | | 334.7 | 8.95347328 |
| 334.8 | 4.10898719 | 334.8 | 14.7590113 | 334.8 | 14.7590113 | 334.8 | 9.62032561 | | | | | 334.80 | 8.96327993 | | | 334.8 | 8.96327993 |
| 334.9 | 4.10898719 | 334.9 | 14.739398 | 334.9 | 14.739398 | 334.9 | 9.63013226 | | | | | 334.90 | 8.97308658 | | | 334.9 | 8.97308658 |
| 335 | 4.10898719 | 335 | 14.7786246 | 335 | 14.7786246 | 335 | 9.64974557 | | | | | 335.00 | 9.00250654 | | | 335 | 9.00250654 |
| 335.1 | 4.09918054 | 335.1 | 14.7786246 | 335.1 | 14.7786246 | 335.1 | 9.62032561 | | | | | 335.10 | 8.99269988 | | | 335.1 | 8.99269988 |
| 335.2 | 4.11879384 | 335.2 | 14.7786246 | 335.2 | 14.7786246 | 335.2 | 9.63013226 | | | | | 335.20 | 8.98289323 | | | 335.2 | 8.98289323 |
| 335.3 | 4.11879384 | 335.3 | 14.7492046 | 335.3 | 14.7492046 | 335.3 | 9.64974557 | | | | | 335.30 | 9.00250654 | | | 335.3 | 9.00250654 |
| 335.4 | 4.1482138 | 335.4 | 14.7688179 | 335.4 | 14.7688179 | 335.4 | 9.65955222 | | | | | 335.40 | 9.00250654 | | | 335.4 | 9.00250654 |
| 335.5 | 4.15802045 | 335.5 | 14.8080445 | 335.5 | 14.8080445 | 335.5 | 9.65955222 | | | | | 335.50 | 9.00250654 | | | 335.5 | 9.00250654 |
| 335.6 | 4.1874404 | 335.6 | 14.8178512 | 335.6 | 14.8178512 | 335.6 | 9.63993892 | | | | | 335.60 | 9.01231319 | | | 335.6 | 9.01231319 |
| 335.7 | 4.1874404 | 335.7 | 14.8472711 | 335.7 | 14.8472711 | 335.7 | 9.65955222 | | | | | 335.70 | 9.03192649 | | | 335.7 | 9.03192649 |
| 335.8 | 4.1678271 | 335.8 | 14.8668844 | 335.8 | 14.8668844 | 335.8 | 9.65955222 | | | | | 335.80 | 9.0515398 | | | 335.8 | 9.0515398 |
| 335.9 | 4.1482138 | 335.9 | 14.8668844 | 335.9 | 14.8668844 | 335.9 | 9.64974557 | | | | | 335.90 | 9.0515398 | | | 335.9 | 9.0515398 |
| 336 | 4.1482138 | 336 | 14.8766911 | 336 | 14.8766911 | 336 | 9.65955222 | | | | | 336.00 | 9.06134645 | | | 336 | 9.06134645 |
| 336.1 | 4.15802045 | 336.1 | 14.8963044 | 336.1 | 14.8963044 | 336.1 | 9.65955222 | | | | | 336.10 | 9.06134645 | | | 336.1 | 9.06134645 |
| 336.2 | 4.1678271 | 336.2 | 14.8766911 | 336.2 | 14.8766911 | 336.2 | 9.65955222 | | | | | 336.20 | 9.0515398 | | | 336.2 | 9.0515398 |
| 336.3 | 4.15802045 | 336.3 | 14.8766911 | 336.3 | 14.8766911 | 336.3 | 9.65955222 | | | | | 336.30 | 9.04173314 | | | 336.3 | 9.04173314 |
| 336.4 | 4.19724706 | 336.4 | 14.906111 | 336.4 | 14.906111 | 336.4 | 9.66935887 | | | | | 336.40 | 9.04173314 | | | 336.4 | 9.04173314 |
| 336.5 | 4.17763375 | 336.5 | 14.9257243 | 336.5 | 14.9257243 | 336.5 | 9.68897218 | | | | | 336.50 | 9.0515398 | | | 336.5 | 9.0515398 |
| 336.6 | 4.1482138 | 336.6 | 14.935531 | 336.6 | 14.935531 | 336.6 | 9.67916552 | | | | | 336.60 | 9.06134645 | | | 336.6 | 9.06134645 |
| 336.7 | 4.1678271 | 336.7 | 14.9257243 | 336.7 | 14.9257243 | 336.7 | 9.67916552 | | | | | 336.70 | 9.06134645 | | | 336.7 | 9.06134645 |
| 336.8 | 4.1874404 | 336.8 | 14.9159177 | 336.8 | 14.9159177 | 336.8 | 9.67916552 | | | | | 336.80 | 9.0711531 | | | 336.8 | 9.0711531 |
| 336.9 | 4.1482138 | 336.9 | 14.906111 | 336.9 | 14.906111 | 336.9 | 9.66935887 | | | | | 336.90 | 9.06134645 | | | 336.9 | 9.06134645 |
| 337 | 4.1678271 | 337 | 14.906111 | 337 | 14.906111 | 337 | 9.63993892 | | | | | 337.00 | 9.06134645 | | | 337 | 9.06134645 |
| 337.1 | 4.20705371 | 337.1 | 14.9257243 | 337.1 | 14.9257243 | 337.1 | 9.65955222 | | | | | 337.10 | 9.08095975 | | | 337.1 | 9.08095975 |
| 337.2 | 4.19724706 | 337.2 | 14.9551443 | 337.2 | 14.9551443 | 337.2 | 9.66935887 | | | | | 337.20 | 9.11037971 | | | 337.2 | 9.11037971 |
| 337.3 | 4.1874404 | 337.3 | 14.9747576 | 337.3 | 14.9747576 | 337.3 | 9.68897218 | | | | | 337.30 | 9.11037971 | | | 337.3 | 9.11037971 |
| 337.4 | 4.15802045 | 337.4 | 14.9453376 | 337.4 | 14.9453376 | 337.4 | 9.69877883 | | | | | 337.40 | 9.0907664 | | | 337.4 | 9.0907664 |
| 337.5 | 4.1874404 | 337.5 | 14.9551443 | 337.5 | 14.9551443 | 337.5 | 9.66935887 | | | | | 337.50 | 9.0907664 | | | 337.5 | 9.0907664 |
| 337.6 | 4.20705371 | 337.6 | 14.9747576 | 337.6 | 14.9747576 | 337.6 | 9.65955222 | | | | | 337.60 | 9.0907664 | | | 337.6 | 9.0907664 |
| 337.7 | 4.21686036 | 337.7 | 14.9551443 | 337.7 | 14.9551443 | 337.7 | 9.68897218 | | | | | 337.70 | 9.0907664 | | | 337.7 | 9.0907664 |
| 337.8 | 4.20705371 | 337.8 | 14.964951 | 337.8 | 14.964951 | 337.8 | 9.69877883 | | | | | 337.80 | 9.0907664 | | | 337.8 | 9.0907664 |
| 337.9 | 4.21686036 | 337.9 | 14.9943709 | 337.9 | 14.9943709 | 337.9 | 9.69877883 | | | | | 337.90 | 9.0907664 | | | 337.9 | 9.0907664 |
| 338 | 4.19724706 | 338 | 14.9943709 | 338 | 14.9943709 | 338 | 9.65955222 | | | | | 338.00 | 9.11037971 | | | 338 | 9.11037971 |
| 338.1 | 4.19724706 | 338.1 | 15.0041776 | 338.1 | 15.0041776 | 338.1 | 9.66935887 | | | | | 338.10 | 9.12018636 | | | 338.1 | 9.12018636 |
| 338.2 | 4.1874404 | 338.2 | 14.9845643 | 338.2 | 14.9845643 | 338.2 | 9.67916552 | | | | | 338.20 | 9.12999301 | | | 338.2 | 9.12999301 |
| 338.3 | 4.17763375 | 338.3 | 15.0041776 | 338.3 | 15.0041776 | 338.3 | 9.67916552 | | | | | 338.30 | 9.15941297 | | | 338.3 | 9.15941297 |
| 338.4 | 4.23647366 | 338.4 | 15.0237909 | 338.4 | 15.0237909 | 338.4 | 9.68897218 | | | | | 338.40 | 9.14960632 | | | 338.4 | 9.14960632 |
| 338.5 | 4.24628032 | 338.5 | 15.0335975 | 338.5 | 15.0335975 | 338.5 | 9.67916552 | | | | | 338.50 | 9.12018636 | | | 338.5 | 9.12018636 |
| 338.6 | 4.24628032 | 338.6 | 15.0532108 | 338.6 | 15.0532108 | 338.6 | 9.67916552 | | | | | 338.60 | 9.10057306 | | | 338.6 | 9.10057306 |
| 338.7 | 4.26589362 | 338.7 | 15.0532108 | 338.7 | 15.0532108 | 338.7 | 9.68897218 | | | | | 338.70 | 9.11037971 | | | 338.7 | 9.11037971 |
| 338.8 | 4.25608697 | 338.8 | 15.0434042 | 338.8 | 15.0434042 | 338.8 | 9.69877883 | | | | | 338.80 | 9.12999301 | | | 338.8 | 9.12999301 |

| | | | | | | | | | | | | | | | | | |
|---|---|---|---|---|---|---|---|---|---|---|---|---|---|---|---|---|---|
| 338.9 | 4.24628032 | 338.9 | 15.0434042 | 338.9 | 15.0434042 | 338.9 | 9.69877883 | | | | | 338.90 | 9.12999301 | | | 338.9 | 9.12999301 |
| 339 | 4.24628032 | 339 | 15.0630175 | 339 | 15.0630175 | 339 | 9.72819878 | | | | | 339.00 | 9.13979966 | | | 339 | 9.13979966 |
| 339.1 | 4.23647366 | 339.1 | 15.0826308 | 339.1 | 15.0826308 | 339.1 | 9.70858548 | | | | | 339.10 | 9.12999301 | | | 339.1 | 9.12999301 |
| 339.2 | 4.25608697 | 339.2 | 15.0532108 | 339.2 | 15.0532108 | 339.2 | 9.70858548 | | | | | 339.20 | 9.13979966 | | | 339.2 | 9.13979966 |
| 339.3 | 4.24628032 | 339.3 | 15.0924374 | 339.3 | 15.0924374 | 339.3 | 9.72819878 | | | | | 339.30 | 9.12999301 | | | 339.3 | 9.12999301 |
| 339.4 | 4.28550692 | 339.4 | 15.0826308 | 339.4 | 15.0826308 | 339.4 | 9.72819878 | | | | | 339.40 | 9.12999301 | | | 339.4 | 9.12999301 |
| 339.5 | 4.32473353 | 339.5 | 15.0924374 | 339.5 | 15.0924374 | 339.5 | 9.73800544 | | | | | 339.50 | 9.12018636 | | | 339.5 | 9.12018636 |
| 339.6 | 4.29531358 | 339.6 | 15.1022441 | 339.6 | 15.1022441 | 339.6 | 9.73800544 | | | | | 339.60 | 9.12018636 | | | 339.6 | 9.12018636 |
| 339.7 | 4.27570027 | 339.7 | 15.1218574 | 339.7 | 15.1218574 | 339.7 | 9.72819878 | | | | | 339.70 | 9.12999301 | | | 339.7 | 9.12999301 |
| 339.8 | 4.31492688 | 339.8 | 15.131664 | 339.8 | 15.131664 | 339.8 | 9.74781209 | | | | | 339.80 | 9.14960632 | | | 339.8 | 9.14960632 |
| 339.9 | 4.25608697 | 339.9 | 15.1218574 | 339.9 | 15.1218574 | 339.9 | 9.72819878 | | | | | 339.90 | 9.12999301 | | | 339.9 | 9.12999301 |
| 340 | 4.27570027 | 340 | 15.1022441 | 340 | 15.1022441 | 340 | 9.72819878 | | | | | 340.00 | 9.13979966 | | | 340 | 9.13979966 |
| 340.1 | 4.29531358 | 340.1 | 15.0826308 | 340.1 | 15.0826308 | 340.1 | 9.74781209 | | | | | 340.10 | 9.15941297 | | | 340.1 | 9.15941297 |
| 340.2 | 4.31492688 | 340.2 | 15.1022441 | 340.2 | 15.1022441 | 340.2 | 9.75761874 | | | | | 340.20 | 9.18883292 | | | 340.2 | 9.18883292 |
| 340.3 | 4.29531358 | 340.3 | 15.1414707 | 340.3 | 15.1414707 | 340.3 | 9.76742539 | | | | | 340.30 | 9.18883292 | | | 340.3 | 9.18883292 |
| 340.4 | 4.27570027 | 340.4 | 15.1414707 | 340.4 | 15.1414707 | 340.4 | 9.75761874 | | | | | 340.40 | 9.17902627 | | | 340.4 | 9.17902627 |
| 340.5 | 4.27570027 | 340.5 | 15.1512773 | 340.5 | 15.1512773 | 340.5 | 9.76742539 | | | | | 340.50 | 9.15941297 | | | 340.5 | 9.15941297 |
| 340.6 | 4.28550692 | 340.6 | 15.1708906 | 340.6 | 15.1708906 | 340.6 | 9.7870387 | | | | | 340.60 | 9.14960632 | | | 340.6 | 9.14960632 |
| 340.7 | 4.27570027 | 340.7 | 15.1806973 | 340.7 | 15.1806973 | 340.7 | 9.77723204 | | | | | 340.70 | 9.16921962 | | | 340.7 | 9.16921962 |
| 340.8 | 4.25608697 | 340.8 | 15.1905039 | 340.8 | 15.1905039 | 340.8 | 9.74781209 | | | | | 340.80 | 9.20844623 | | | 340.8 | 9.20844623 |
| 340.9 | 4.27570027 | 340.9 | 15.2199239 | 340.9 | 15.2199239 | 340.9 | 9.75761874 | | | | | 340.90 | 9.18883292 | | | 340.9 | 9.18883292 |
| 341 | 4.29531358 | 341 | 15.2297306 | 341 | 15.2297306 | 341 | 9.75761874 | | | | | 341.00 | 9.17902627 | | | 341 | 9.17902627 |
| 341.1 | 4.28550692 | 341.1 | 15.2003106 | 341.1 | 15.2003106 | 341.1 | 9.76742539 | | | | | 341.10 | 9.19863958 | | | 341.1 | 9.19863958 |
| 341.2 | 4.28550692 | 341.2 | 15.2199239 | 341.2 | 15.2199239 | 341.2 | 9.76742539 | | | | | 341.20 | 9.21825288 | | | 341.2 | 9.21825288 |
| 341.3 | 4.28550692 | 341.3 | 15.2493439 | 341.3 | 15.2493439 | 341.3 | 9.72819878 | | | | | 341.30 | 9.22805953 | | | 341.3 | 9.22805953 |
| 341.4 | 4.28550692 | 341.4 | 15.2199239 | 341.4 | 15.2199239 | 341.4 | 9.72819878 | | | | | 341.40 | 9.22805953 | | | 341.4 | 9.22805953 |
| 341.5 | 4.28550692 | 341.5 | 15.2395372 | 341.5 | 15.2395372 | 341.5 | 9.76742539 | | | | | 341.50 | 9.21825288 | | | 341.5 | 9.21825288 |
| 341.6 | 4.30512023 | 341.6 | 15.2689572 | 341.6 | 15.2689572 | 341.6 | 9.7870387 | | | | | 341.60 | 9.20844623 | | | 341.6 | 9.20844623 |
| 341.7 | 4.32473353 | 341.7 | 15.2983771 | 341.7 | 15.2983771 | 341.7 | 9.7870387 | | | | | 341.70 | 9.19863958 | | | 341.7 | 9.19863958 |
| 341.8 | 4.31492688 | 341.8 | 15.2983771 | 341.8 | 15.2983771 | 341.8 | 9.75761874 | | | | | 341.80 | 9.19863958 | | | 341.8 | 9.19863958 |
| 341.9 | 4.30512023 | 341.9 | 15.2787638 | 341.9 | 15.2787638 | 341.9 | 9.76742539 | | | | | 341.90 | 9.19863958 | | | 341.9 | 9.19863958 |
| 342 | 4.28550692 | 342 | 15.3081838 | 342 | 15.3081838 | 342 | 9.76742539 | | | | | 342.00 | 9.24767284 | | | 342 | 9.24767284 |
| 342.1 | 4.31492688 | 342.1 | 15.2983771 | 342.1 | 15.2983771 | 342.1 | 9.7870387 | | | | | 342.10 | 9.23786618 | | | 342.1 | 9.23786618 |
| 342.2 | 4.36396014 | 342.2 | 15.3081838 | 342.2 | 15.3081838 | 342.2 | 9.76742539 | | | | | 342.20 | 9.24767284 | | | 342.2 | 9.24767284 |
| 342.3 | 4.35415349 | 342.3 | 15.3081838 | 342.3 | 15.3081838 | 342.3 | 9.74781209 | | | | | 342.30 | 9.21825288 | | | 342.3 | 9.21825288 |
| 342.4 | 4.34434684 | 342.4 | 15.2983771 | 342.4 | 15.2983771 | 342.4 | 9.77723204 | | | | | 342.40 | 9.22805953 | | | 342.4 | 9.22805953 |
| 342.5 | 4.36396014 | 342.5 | 15.3081838 | 342.5 | 15.3081838 | 342.5 | 9.79684535 | | | | | 342.50 | 9.24767284 | | | 342.5 | 9.24767284 |
| 342.6 | 4.35415349 | 342.6 | 15.357217 | 342.6 | 15.357217 | 342.6 | 9.79684535 | | | | | 342.60 | 9.22805953 | | | 342.6 | 9.22805953 |
| 342.7 | 4.35415349 | 342.7 | 15.386637 | 342.7 | 15.386637 | 342.7 | 9.76742539 | | | | | 342.70 | 9.21825288 | | | 342.7 | 9.21825288 |
| 342.8 | 4.33454018 | 342.8 | 15.386637 | 342.8 | 15.386637 | 342.8 | 9.77723204 | | | | | 342.80 | 9.21825288 | | | 342.8 | 9.21825288 |
| 342.9 | 4.34434684 | 342.9 | 15.3768303 | 342.9 | 15.3768303 | 342.9 | 9.7870387 | | | | | 342.90 | 9.24767284 | | | 342.9 | 9.24767284 |
| 343 | 4.34434684 | 343 | 15.3670237 | 343 | 15.3670237 | 343 | 9.77723204 | | | | | 343.00 | 9.25747949 | | | 343 | 9.25747949 |
| 343.1 | 4.32473353 | 343.1 | 15.3768303 | 343.1 | 15.3768303 | 343.1 | 9.77723204 | | | | | 343.10 | 9.24767284 | | | 343.1 | 9.24767284 |
| 343.2 | 4.35415349 | 343.2 | 15.4062503 | 343.2 | 15.4062503 | 343.2 | 9.77723204 | | | | | 343.20 | 9.24767284 | | | 343.2 | 9.24767284 |
| 343.3 | 4.35415349 | 343.3 | 15.3964436 | 343.3 | 15.3964436 | 343.3 | 9.75761874 | | | | | 343.30 | 9.27709279 | | | 343.3 | 9.27709279 |
| 343.4 | 4.36396014 | 343.4 | 15.4062503 | 343.4 | 15.4062503 | 343.4 | 9.77723204 | | | | | 343.40 | 9.2967061 | | | 343.4 | 9.2967061 |
| 343.5 | 4.34434684 | 343.5 | 15.4160569 | 343.5 | 15.4160569 | 343.5 | 9.7870387 | | | | | 343.50 | 9.28689944 | | | 343.5 | 9.28689944 |
| 343.6 | 4.33454018 | 343.6 | 15.3964436 | 343.6 | 15.3964436 | 343.6 | 9.7870387 | | | | | 343.60 | 9.26728614 | | | 343.6 | 9.26728614 |
| 343.7 | 4.37376679 | 343.7 | 15.386637 | 343.7 | 15.386637 | 343.7 | 9.77723204 | | | | | 343.70 | 9.26728614 | | | 343.7 | 9.26728614 |
| 343.8 | 4.37376679 | 343.8 | 15.4356702 | 343.8 | 15.4356702 | 343.8 | 9.79684535 | | | | | 343.80 | 9.2967061 | | | 343.8 | 9.2967061 |
| 343.9 | 4.33454018 | 343.9 | 15.4258636 | 343.9 | 15.4258636 | 343.9 | 9.7870387 | | | | | 343.90 | 9.2967061 | | | 343.9 | 9.2967061 |
| 344 | 4.32473353 | 344 | 15.4356702 | 344 | 15.4356702 | 344 | 9.79684535 | | | | | 344.00 | 9.2967061 | | | 344 | 9.2967061 |
| 344.1 | 4.32473353 | 344.1 | 15.4847035 | 344.1 | 15.4847035 | 344.1 | 9.806652 | | | | | 344.10 | 9.2967061 | | | 344.1 | 9.2967061 |
| 344.2 | 4.34434684 | 344.2 | 15.4847035 | 344.2 | 15.4847035 | 344.2 | 9.806652 | | | | | 344.20 | 9.2967061 | | | 344.2 | 9.2967061 |
| 344.3 | 4.37376679 | 344.3 | 15.4847035 | 344.3 | 15.4847035 | 344.3 | 9.806652 | | | | | 344.30 | 9.30651275 | | | 344.3 | 9.30651275 |
| 344.4 | 4.35415349 | 344.4 | 15.5141235 | 344.4 | 15.5141235 | 344.4 | 9.79684535 | | | | | 344.40 | 9.32612605 | | | 344.4 | 9.32612605 |
| 344.5 | 4.35415349 | 344.5 | 15.4748969 | 344.5 | 15.4748969 | 344.5 | 9.806652 | | | | | 344.50 | 9.3163194 | | | 344.5 | 9.3163194 |
| 344.6 | 4.35415349 | 344.6 | 15.4945102 | 344.6 | 15.4945102 | 344.6 | 9.806652 | | | | | 344.60 | 9.30651275 | | | 344.6 | 9.30651275 |
| 344.7 | 4.36396014 | 344.7 | 15.5337368 | 344.7 | 15.5337368 | 344.7 | 9.7870387 | | | | | 344.70 | 9.32612605 | | | 344.7 | 9.32612605 |
| 344.8 | 4.40318675 | 344.8 | 15.5239301 | 344.8 | 15.5239301 | 344.8 | 9.79684535 | | | | | 344.80 | 9.3359327 | | | 344.8 | 9.3359327 |
| 344.9 | 4.37376679 | 344.9 | 15.5043168 | 344.9 | 15.5043168 | 344.9 | 9.7870387 | | | | | 344.90 | 9.3359327 | | | 344.9 | 9.3359327 |

| | | | | | | | | | | | | | | | | | |
|---|---|---|---|---|---|---|---|---|---|---|---|---|---|---|---|---|---|
| 345 | 4.37376679 | 345 | 15.5141235 | 345 | 15.5141235 | 345 | 9.77723204 | | | | | 345.00 | 9.3359327 | | | 345 | 9.3359327 |
| 345.1 | 4.40318675 | 345.1 | 15.5043168 | 345.1 | 15.5043168 | 345.1 | 9.7870387 | | | | | 345.10 | 9.3163194 | | | 345.1 | 9.3163194 |
| 345.2 | 4.4129934 | 345.2 | 15.4945102 | 345.2 | 15.4945102 | 345.2 | 9.79684535 | | | | | 345.20 | 9.3359327 | | | 345.2 | 9.3359327 |
| 345.3 | 4.3933801 | 345.3 | 15.5435434 | 345.3 | 15.5435434 | 345.3 | 9.79684535 | | | | | 345.30 | 9.34573936 | | | 345.3 | 9.34573936 |
| 345.4 | 4.3933801 | 345.4 | 15.5925767 | 345.4 | 15.5925767 | 345.4 | 9.79684535 | | | | | 345.40 | 9.35554601 | | | 345.4 | 9.35554601 |
| 345.5 | 4.36396014 | 345.5 | 15.58277 | 345.5 | 15.58277 | 345.5 | 9.79684535 | | | | | 345.50 | 9.34573936 | | | 345.5 | 9.34573936 |
| 345.6 | 4.37376679 | 345.6 | 15.58277 | 345.6 | 15.58277 | 345.6 | 9.79684535 | | | | | 345.60 | 9.38496596 | | | 345.6 | 9.38496596 |
| 345.7 | 4.3933801 | 345.7 | 15.61219 | 345.7 | 15.61219 | 345.7 | 9.8262653 | | | | | 345.70 | 9.37515931 | | | 345.7 | 9.37515931 |
| 345.8 | 4.37376679 | 345.8 | 15.6219966 | 345.8 | 15.6219966 | 345.8 | 9.806652 | | | | | 345.80 | 9.35554601 | | | 345.8 | 9.35554601 |
| 345.9 | 4.4129934 | 345.9 | 15.6416099 | 345.9 | 15.6416099 | 345.9 | 9.77723204 | | | | | 345.90 | 9.36535266 | | | 345.9 | 9.36535266 |
| 346 | 4.3933801 | 346 | 15.6514166 | 346 | 15.6514166 | 346 | 9.77723204 | | | | | 346.00 | 9.37515931 | | | 346 | 9.37515931 |
| 346.1 | 4.38357344 | 346.1 | 15.6514166 | 346.1 | 15.6514166 | 346.1 | 9.79684535 | | | | | 346.10 | 9.36535266 | | | 346.1 | 9.36535266 |
| 346.2 | 4.37376679 | 346.2 | 15.6416099 | 346.2 | 15.6416099 | 346.2 | 9.79684535 | | | | | 346.20 | 9.38496596 | | | 346.2 | 9.38496596 |
| 346.3 | 4.34434684 | 346.3 | 15.6416099 | 346.3 | 15.6416099 | 346.3 | 9.8262653 | | | | | 346.30 | 9.38496596 | | | 346.3 | 9.38496596 |
| 346.4 | 4.37376679 | 346.4 | 15.6514166 | 346.4 | 15.6514166 | 346.4 | 9.806652 | | | | | 346.40 | 9.37515931 | | | 346.4 | 9.37515931 |
| 346.5 | 4.40318675 | 346.5 | 15.6514166 | 346.5 | 15.6514166 | 346.5 | 9.79684535 | | | | | 346.50 | 9.40457927 | | | 346.5 | 9.40457927 |
| 346.6 | 4.42280005 | 346.6 | 15.6416099 | 346.6 | 15.6416099 | 346.6 | 9.7870387 | | | | | 346.60 | 9.41438592 | | | 346.6 | 9.41438592 |
| 346.7 | 4.4326067 | 346.7 | 15.6612232 | 346.7 | 15.6612232 | 346.7 | 9.806652 | | | | | 346.70 | 9.41438592 | | | 346.7 | 9.41438592 |
| 346.8 | 4.4326067 | 346.8 | 15.6808365 | 346.8 | 15.6808365 | 346.8 | 9.806652 | | | | | 346.80 | 9.38496596 | | | 346.8 | 9.38496596 |
| 346.9 | 4.44241336 | 346.9 | 15.6906432 | 346.9 | 15.6906432 | 346.9 | 9.79684535 | | | | | 346.90 | 9.39477262 | | | 346.9 | 9.39477262 |
| 347 | 4.45222001 | 347 | 15.6906432 | 347 | 15.6906432 | 347 | 9.81645865 | | | | | 347.00 | 9.41438592 | | | 347 | 9.41438592 |
| 347.1 | 4.4326067 | 347.1 | 15.7102565 | 347.1 | 15.7102565 | 347.1 | 9.8262653 | | | | | 347.10 | 9.40457927 | | | 347.1 | 9.40457927 |
| 347.2 | 4.3933801 | 347.2 | 15.7396765 | 347.2 | 15.7396765 | 347.2 | 9.81645865 | | | | | 347.20 | 9.39477262 | | | 347.2 | 9.39477262 |
| 347.3 | 4.3933801 | 347.3 | 15.7298698 | 347.3 | 15.7298698 | 347.3 | 9.7870387 | | | | | 347.30 | 9.41438592 | | | 347.3 | 9.41438592 |
| 347.4 | 4.40318675 | 347.4 | 15.7396765 | 347.4 | 15.7396765 | 347.4 | 9.806652 | | | | | 347.40 | 9.42419257 | | | 347.4 | 9.42419257 |
| 347.5 | 4.40318675 | 347.5 | 15.7102565 | 347.5 | 15.7102565 | 347.5 | 9.79684535 | | | | | 347.50 | 9.41438592 | | | 347.5 | 9.41438592 |
| 347.6 | 4.38357344 | 347.6 | 15.7200632 | 347.6 | 15.7200632 | 347.6 | 9.806652 | | | | | 347.60 | 9.41438592 | | | 347.6 | 9.41438592 |
| 347.7 | 4.42280005 | 347.7 | 15.7592898 | 347.7 | 15.7592898 | 347.7 | 9.806652 | | | | | 347.70 | 9.42419257 | | | 347.7 | 9.42419257 |
| 347.8 | 4.44241336 | 347.8 | 15.7789031 | 347.8 | 15.7789031 | 347.8 | 9.76742539 | | | | | 347.80 | 9.41438592 | | | 347.8 | 9.41438592 |
| 347.9 | 4.48163996 | 347.9 | 15.7494831 | 347.9 | 15.7494831 | 347.9 | 9.7870387 | | | | | 347.90 | 9.42419257 | | | 347.9 | 9.42419257 |
| 348 | 4.46202666 | 348 | 15.7396765 | 348 | 15.7396765 | 348 | 9.806652 | | | | | 348.00 | 9.44380588 | | | 348 | 9.44380588 |
| 348.1 | 4.40318675 | 348.1 | 15.7396765 | 348.1 | 15.7396765 | 348.1 | 9.79684535 | | | | | 348.10 | 9.45361253 | | | 348.1 | 9.45361253 |
| 348.2 | 4.40318675 | 348.2 | 15.7690964 | 348.2 | 15.7690964 | 348.2 | 9.79684535 | | | | | 348.20 | 9.44380588 | | | 348.2 | 9.44380588 |
| 348.3 | 4.4326067 | 348.3 | 15.7690964 | 348.3 | 15.7690964 | 348.3 | 9.81645865 | | | | | 348.30 | 9.46341918 | | | 348.3 | 9.46341918 |
| 348.4 | 4.4129934 | 348.4 | 15.7494831 | 348.4 | 15.7494831 | 348.4 | 9.806652 | | | | | 348.40 | 9.46341918 | | | 348.4 | 9.46341918 |
| 348.5 | 4.40318675 | 348.5 | 15.7789031 | 348.5 | 15.7789031 | 348.5 | 9.806652 | | | | | 348.50 | 9.46341918 | | | 348.5 | 9.46341918 |
| 348.6 | 4.44241336 | 348.6 | 15.7887097 | 348.6 | 15.7887097 | 348.6 | 9.7870387 | | | | | 348.60 | 9.47322583 | | | 348.6 | 9.47322583 |
| 348.7 | 4.44241336 | 348.7 | 15.8279363 | 348.7 | 15.8279363 | 348.7 | 9.806652 | | | | | 348.70 | 9.47322583 | | | 348.7 | 9.47322583 |
| 348.8 | 4.44241336 | 348.8 | 15.8181297 | 348.8 | 15.8181297 | 348.8 | 9.81645865 | | | | | 348.80 | 9.46341918 | | | 348.8 | 9.46341918 |
| 348.9 | 4.46202666 | 348.9 | 15.7985164 | 348.9 | 15.7985164 | 348.9 | 9.8262653 | | | | | 348.90 | 9.45361253 | | | 348.9 | 9.45361253 |
| 349 | 4.44241336 | 349 | 15.8181297 | 349 | 15.8181297 | 349 | 9.7870387 | | | | | 349.00 | 9.49283914 | | | 349 | 9.49283914 |
| 349.1 | 4.47183331 | 349.1 | 15.8671629 | 349.1 | 15.8671629 | 349.1 | 9.806652 | | | | | 349.10 | 9.47322583 | | | 349.1 | 9.47322583 |
| 349.2 | 4.46202666 | 349.2 | 15.837743 | 349.2 | 15.837743 | 349.2 | 9.81645865 | | | | | 349.20 | 9.48303248 | | | 349.2 | 9.48303248 |
| 349.3 | 4.46202666 | 349.3 | 15.8769696 | 349.3 | 15.8769696 | 349.3 | 9.806652 | | | | | 349.30 | 9.47322583 | | | 349.3 | 9.47322583 |
| 349.4 | 4.48163996 | 349.4 | 15.8573563 | 349.4 | 15.8573563 | 349.4 | 9.79684535 | | | | | 349.40 | 9.52225909 | | | 349.4 | 9.52225909 |
| 349.5 | 4.49144662 | 349.5 | 15.8279363 | 349.5 | 15.8279363 | 349.5 | 9.7870387 | | | | | 349.50 | 9.55167905 | | | 349.5 | 9.55167905 |
| 349.6 | 4.51105992 | 349.6 | 15.8475496 | 349.6 | 15.8475496 | 349.6 | 9.77723204 | | | | | 349.60 | 9.52225909 | | | 349.6 | 9.52225909 |
| 349.7 | 4.50125327 | 349.7 | 15.8867762 | 349.7 | 15.8867762 | 349.7 | 9.79684535 | | | | | 349.70 | 9.51245244 | | | 349.7 | 9.51245244 |
| 349.8 | 4.45222001 | 349.8 | 15.8965829 | 349.8 | 15.8965829 | 349.8 | 9.8262653 | | | | | 349.80 | 9.51245244 | | | 349.8 | 9.51245244 |
| 349.9 | 4.49144662 | 349.9 | 15.8867762 | 349.9 | 15.8867762 | 349.9 | 9.81645865 | | | | | 349.90 | 9.53206574 | | | 349.9 | 9.53206574 |
| 350 | 4.54047988 | 350 | 15.8965829 | 350 | 15.8965829 | 350 | 9.79684535 | | | | | 350.00 | 9.5418724 | | | 350 | 9.5418724 |
| 350.1 | 4.53067322 | 350.1 | 15.9161962 | 350.1 | 15.9161962 | 350.1 | 9.806652 | | | | | 350.10 | 9.5418724 | | | 350.1 | 9.5418724 |
| 350.2 | 4.49144662 | 350.2 | 15.9456162 | 350.2 | 15.9456162 | 350.2 | 9.7870387 | | | | | 350.20 | 9.51245244 | | | 350.2 | 9.51245244 |
| 350.3 | 4.47183331 | 350.3 | 15.9260028 | 350.3 | 15.9260028 | 350.3 | 9.81645865 | | | | | 350.30 | 9.52225909 | | | 350.3 | 9.52225909 |
| 350.4 | 4.49144662 | 350.4 | 15.8965829 | 350.4 | 15.8965829 | 350.4 | 9.81645865 | | | | | 350.40 | 9.53206574 | | | 350.4 | 9.53206574 |
| 350.5 | 4.49144662 | 350.5 | 15.9456162 | 350.5 | 15.9456162 | 350.5 | 9.81645865 | | | | | 350.50 | 9.5418724 | | | 350.5 | 9.5418724 |
| 350.6 | 4.48163996 | 350.6 | 15.9554228 | 350.6 | 15.9554228 | 350.6 | 9.81645865 | | | | | 350.60 | 9.5418724 | | | 350.6 | 9.5418724 |
| 350.7 | 4.50125327 | 350.7 | 15.9358095 | 350.7 | 15.9358095 | 350.7 | 9.8262653 | | | | | 350.70 | 9.5418724 | | | 350.7 | 9.5418724 |
| 350.8 | 4.54047988 | 350.8 | 15.9358095 | 350.8 | 15.9358095 | 350.8 | 9.806652 | | | | | 350.80 | 9.5418724 | | | 350.8 | 9.5418724 |
| 350.9 | 4.50125327 | 350.9 | 15.9554228 | 350.9 | 15.9554228 | 350.9 | 9.806652 | | | | | 350.90 | 9.5418724 | | | 350.9 | 9.5418724 |
| 351 | 4.53067322 | 351 | 15.9848428 | 351 | 15.9848428 | 351 | 9.806652 | | | | | 351.00 | 9.5418724 | | | 351 | 9.5418724 |

| | | | | | | | | | | | | | | | | | |
|---|---|---|---|---|---|---|---|---|---|---|---|---|---|---|---|---|---|
| 351.1 | 4.53067322 | 351.1 | 15.9946494 | 351.1 | 15.9946494 | 351.1 | 9.83607196 | | | | | 351.10 | 9.5418724 | | | 351.1 | 9.5418724 |
| 351.2 | 4.51105992 | 351.2 | 15.9848428 | 351.2 | 15.9848428 | 351.2 | 9.84587861 | | | | | 351.20 | 9.52225909 | | | 351.2 | 9.52225909 |
| 351.3 | 4.53067322 | 351.3 | 16.0142627 | 351.3 | 16.0142627 | 351.3 | 9.85568526 | | | | | 351.30 | 9.53206574 | | | 351.3 | 9.53206574 |
| 351.4 | 4.55028653 | 351.4 | 16.0240694 | 351.4 | 16.0240694 | 351.4 | 9.806652 | | | | | 351.40 | 9.53206574 | | | 351.4 | 9.53206574 |
| 351.5 | 4.57970648 | 351.5 | 16.0436827 | 351.5 | 16.0436827 | 351.5 | 9.8262653 | | | | | 351.50 | 9.55167905 | | | 351.5 | 9.55167905 |
| 351.6 | 4.54047988 | 351.6 | 16.0436827 | 351.6 | 16.0436827 | 351.6 | 9.8262653 | | | | | 351.60 | 9.5418724 | | | 351.6 | 9.5418724 |
| 351.7 | 4.52086657 | 351.7 | 16.033876 | 351.7 | 16.033876 | 351.7 | 9.806652 | | | | | 351.70 | 9.581099 | | | 351.7 | 9.581099 |
| 351.8 | 4.49144662 | 351.8 | 16.0436827 | 351.8 | 16.0436827 | 351.8 | 9.8262653 | | | | | 351.80 | 9.581099 | | | 351.8 | 9.581099 |
| 351.9 | 4.50125327 | 351.9 | 16.0436827 | 351.9 | 16.0436827 | 351.9 | 9.84587861 | | | | | 351.90 | 9.581099 | | | 351.9 | 9.581099 |
| 352 | 4.56989983 | 352 | 16.063296 | 352 | 16.063296 | 352 | 9.8262653 | | | | | 352.00 | 9.57129235 | | | 352 | 9.57129235 |
| 352.1 | 4.59931979 | 352.1 | 16.0534893 | 352.1 | 16.0534893 | 352.1 | 9.85568526 | | | | | 352.10 | 9.57129235 | | | 352.1 | 9.57129235 |
| 352.2 | 4.60912644 | 352.2 | 16.0534893 | 352.2 | 16.0534893 | 352.2 | 9.88510522 | | | | | 352.20 | 9.581099 | | | 352.2 | 9.581099 |
| 352.3 | 4.56009318 | 352.3 | 16.063296 | 352.3 | 16.063296 | 352.3 | 9.84587861 | | | | | 352.30 | 9.57129235 | | | 352.3 | 9.57129235 |
| 352.4 | 4.55028653 | 352.4 | 16.1025226 | 352.4 | 16.1025226 | 352.4 | 9.85568526 | | | | | 352.40 | 9.57129235 | | | 352.4 | 9.57129235 |
| 352.5 | 4.56989983 | 352.5 | 16.1025226 | 352.5 | 16.1025226 | 352.5 | 9.806652 | | | | | 352.50 | 9.59090566 | | | 352.5 | 9.59090566 |
| 352.6 | 4.58951314 | 352.6 | 16.1123292 | 352.6 | 16.1123292 | 352.6 | 9.8262653 | | | | | 352.60 | 9.581099 | | | 352.6 | 9.581099 |
| 352.7 | 4.56989983 | 352.7 | 16.1319425 | 352.7 | 16.1319425 | 352.7 | 9.83607196 | | | | | 352.70 | 9.59090566 | | | 352.7 | 9.59090566 |
| 352.8 | 4.56989983 | 352.8 | 16.1515558 | 352.8 | 16.1515558 | 352.8 | 9.84587861 | | | | | 352.80 | 9.57129235 | | | 352.8 | 9.57129235 |
| 352.9 | 4.56009318 | 352.9 | 16.1515558 | 352.9 | 16.1515558 | 352.9 | 9.85568526 | | | | | 352.90 | 9.61051896 | | | 352.9 | 9.61051896 |
| 353 | 4.55028653 | 353 | 16.1319425 | 353 | 16.1319425 | 353 | 9.85568526 | | | | | 353.00 | 9.63013226 | | | 353 | 9.63013226 |
| 353.1 | 4.56009318 | 353.1 | 16.1221359 | 353.1 | 16.1221359 | 353.1 | 9.806652 | | | | | 353.10 | 9.59090566 | | | 353.1 | 9.59090566 |
| 353.2 | 4.56989983 | 353.2 | 16.1417492 | 353.2 | 16.1417492 | 353.2 | 9.8262653 | | | | | 353.20 | 9.59090566 | | | 353.2 | 9.59090566 |
| 353.3 | 4.56009318 | 353.3 | 16.1711691 | 353.3 | 16.1711691 | 353.3 | 9.83607196 | | | | | 353.30 | 9.62032561 | | | 353.3 | 9.62032561 |
| 353.4 | 4.55028653 | 353.4 | 16.1809758 | 353.4 | 16.1809758 | 353.4 | 9.86549191 | | | | | 353.40 | 9.63993892 | | | 353.4 | 9.63993892 |
| 353.5 | 4.54047988 | 353.5 | 16.1809758 | 353.5 | 16.1809758 | 353.5 | 9.87529856 | | | | | 353.50 | 9.63013226 | | | 353.5 | 9.63013226 |
| 353.6 | 4.55028653 | 353.6 | 16.1809758 | 353.6 | 16.1809758 | 353.6 | 9.86549191 | | | | | 353.60 | 9.64974557 | | | 353.6 | 9.64974557 |
| 353.7 | 4.56989983 | 353.7 | 16.1809758 | 353.7 | 16.1809758 | 353.7 | 9.84587861 | | | | | 353.70 | 9.63013226 | | | 353.7 | 9.63013226 |
| 353.8 | 4.56989983 | 353.8 | 16.1809758 | 353.8 | 16.1809758 | 353.8 | 9.86549191 | | | | | 353.80 | 9.63993892 | | | 353.8 | 9.63993892 |
| 353.9 | 4.57970648 | 353.9 | 16.1907825 | 353.9 | 16.1907825 | 353.9 | 9.86549191 | | | | | 353.90 | 9.64974557 | | | 353.9 | 9.64974557 |
| 354 | 4.58951314 | 354 | 16.2005891 | 354 | 16.2005891 | 354 | 9.84587861 | | | | | 354.00 | 9.63993892 | | | 354 | 9.63993892 |
| 354.1 | 4.59931979 | 354.1 | 16.2005891 | 354.1 | 16.2005891 | 354.1 | 9.85568526 | | | | | 354.10 | 9.66935887 | | | 354.1 | 9.66935887 |
| 354.2 | 4.57970648 | 354.2 | 16.2398157 | 354.2 | 16.2398157 | 354.2 | 9.85568526 | | | | | 354.20 | 9.65955222 | | | 354.2 | 9.65955222 |
| 354.3 | 4.55028653 | 354.3 | 16.259429 | 354.3 | 16.259429 | 354.3 | 9.88510522 | | | | | 354.30 | 9.67916552 | | | 354.3 | 9.67916552 |
| 354.4 | 4.61893309 | 354.4 | 16.2300091 | 354.4 | 16.2300091 | 354.4 | 9.87529856 | | | | | 354.40 | 9.69877883 | | | 354.4 | 9.69877883 |
| 354.5 | 4.60912644 | 354.5 | 16.2496224 | 354.5 | 16.2496224 | 354.5 | 9.87529856 | | | | | 354.50 | 9.71839213 | | | 354.5 | 9.71839213 |
| 354.6 | 4.59931979 | 354.6 | 16.2496224 | 354.6 | 16.2496224 | 354.6 | 9.85568526 | | | | | 354.60 | 9.73800544 | | | 354.6 | 9.73800544 |
| 354.7 | 4.59931979 | 354.7 | 16.2398157 | 354.7 | 16.2398157 | 354.7 | 9.85568526 | | | | | 354.70 | 9.75761874 | | | 354.7 | 9.75761874 |
| 354.8 | 4.60912644 | 354.8 | 16.2496224 | 354.8 | 16.2496224 | 354.8 | 9.87529856 | | | | | 354.80 | 9.75761874 | | | 354.8 | 9.75761874 |
| 354.9 | 4.62873974 | 354.9 | 16.2496224 | 354.9 | 16.2496224 | 354.9 | 9.86549191 | | | | | 354.90 | 9.72819878 | | | 354.9 | 9.72819878 |
| 355 | 4.61893309 | 355 | 16.2300091 | 355 | 16.2300091 | 355 | 9.86549191 | | | | | 355.00 | 9.71839213 | | | 355 | 9.71839213 |
| 355.1 | 4.57970648 | 355.1 | 16.259429 | 355.1 | 16.259429 | 355.1 | 9.89491187 | | | | | 355.10 | 9.71839213 | | | 355.1 | 9.71839213 |
| 355.2 | 4.58951314 | 355.2 | 16.2790423 | 355.2 | 16.2790423 | 355.2 | 9.90471852 | | | | | 355.20 | 9.74781209 | | | 355.2 | 9.74781209 |
| 355.3 | 4.58951314 | 355.3 | 16.2790423 | 355.3 | 16.2790423 | 355.3 | 9.88510522 | | | | | 355.30 | 9.76742539 | | | 355.3 | 9.76742539 |
| 355.4 | 4.56989983 | 355.4 | 16.2790423 | 355.4 | 16.2790423 | 355.4 | 9.88510522 | | | | | 355.40 | 9.74781209 | | | 355.4 | 9.74781209 |
| 355.5 | 4.58951314 | 355.5 | 16.3084623 | 355.5 | 16.3084623 | 355.5 | 9.88510522 | | | | | 355.50 | 9.74781209 | | | 355.5 | 9.74781209 |
| 355.6 | 4.61893309 | 355.6 | 16.2986556 | 355.6 | 16.2986556 | 355.6 | 9.90471852 | | | | | 355.60 | 9.75761874 | | | 355.6 | 9.75761874 |
| 355.7 | 4.59931979 | 355.7 | 16.2790423 | 355.7 | 16.2790423 | 355.7 | 9.91452517 | | | | | 355.70 | 9.76742539 | | | 355.7 | 9.76742539 |
| 355.8 | 4.58951314 | 355.8 | 16.288849 | 355.8 | 16.288849 | 355.8 | 9.90471852 | | | | | 355.80 | 9.75761874 | | | 355.8 | 9.75761874 |
| 355.9 | 4.62873974 | 355.9 | 16.3280756 | 355.9 | 16.3280756 | 355.9 | 9.89491187 | | | | | 355.90 | 9.806652 | | | 355.9 | 9.806652 |
| 356 | 4.6385464 | 356 | 16.3378822 | 356 | 16.3378822 | 356 | 9.91452517 | | | | | 356.00 | 9.81645865 | | | 356 | 9.81645865 |
| 356.1 | 4.62873974 | 356.1 | 16.3182689 | 356.1 | 16.3182689 | 356.1 | 9.87529856 | | | | | 356.10 | 9.81645865 | | | 356.1 | 9.81645865 |
| 356.2 | 4.62873974 | 356.2 | 16.3574955 | 356.2 | 16.3574955 | 356.2 | 9.87529856 | | | | | 356.20 | 9.79684535 | | | 356.2 | 9.79684535 |
| 356.3 | 4.64835305 | 356.3 | 16.3476889 | 356.3 | 16.3476889 | 356.3 | 9.88510522 | | | | | 356.30 | 9.77723204 | | | 356.3 | 9.77723204 |
| 356.4 | 4.6385464 | 356.4 | 16.3574955 | 356.4 | 16.3574955 | 356.4 | 9.91452517 | | | | | 356.40 | 9.7870387 | | | 356.4 | 9.7870387 |
| 356.5 | 4.59931979 | 356.5 | 16.3967221 | 356.5 | 16.3967221 | 356.5 | 9.92433182 | | | | | 356.50 | 9.79684535 | | | 356.5 | 9.79684535 |
| 356.6 | 4.60912644 | 356.6 | 16.3967221 | 356.6 | 16.3967221 | 356.6 | 9.92433182 | | | | | 356.60 | 9.7870387 | | | 356.6 | 9.7870387 |
| 356.7 | 4.61893309 | 356.7 | 16.3673022 | 356.7 | 16.3673022 | 356.7 | 9.91452517 | | | | | 356.70 | 9.79684535 | | | 356.7 | 9.79684535 |
| 356.8 | 4.6385464 | 356.8 | 16.3771088 | 356.8 | 16.3771088 | 356.8 | 9.91452517 | | | | | 356.80 | 9.7870387 | | | 356.8 | 9.7870387 |
| 356.9 | 4.66796635 | 356.9 | 16.3967221 | 356.9 | 16.3967221 | 356.9 | 9.91452517 | | | | | 356.90 | 9.79684535 | | | 356.9 | 9.79684535 |
| 357 | 4.6385464 | 357 | 16.4163354 | 357 | 16.4163354 | 357 | 9.92433182 | | | | | 357.00 | 9.79684535 | | | 357 | 9.79684535 |
| 357.1 | 4.64835305 | 357.1 | 16.4065288 | 357.1 | 16.4065288 | 357.1 | 9.91452517 | | | | | 357.10 | 9.79684535 | | | 357.1 | 9.79684535 |

| | | | | | | | | | | | | | | | | | |
|---|---|---|---|---|---|---|---|---|---|---|---|---|---|---|---|---|---|
| 357.2 | 4.66796635 | 357.2 | 16.3869155 | 357.2 | 16.3869155 | 357.2 | 9.91452517 | | | | | 357.20 | 9.77723204 | | | 357.2 | 9.77723204 |
| 357.3 | 4.66796635 | 357.3 | 16.4065288 | 357.3 | 16.4065288 | 357.3 | 9.92433182 | | | | | 357.30 | 9.7870387 | | | 357.3 | 9.7870387 |
| 357.4 | 4.66796635 | 357.4 | 16.4359488 | 357.4 | 16.4359488 | 357.4 | 9.94394513 | | | | | 357.40 | 9.75761874 | | | 357.4 | 9.75761874 |
| 357.5 | 4.677773 | 357.5 | 16.4751754 | 357.5 | 16.4751754 | 357.5 | 9.94394513 | | | | | 357.50 | 9.7870387 | | | 357.5 | 9.7870387 |
| 357.6 | 4.66796635 | 357.6 | 16.484982 | 357.6 | 16.484982 | 357.6 | 9.93413848 | | | | | 357.60 | 9.77723204 | | | 357.6 | 9.77723204 |
| 357.7 | 4.66796635 | 357.7 | 16.484982 | 357.7 | 16.484982 | 357.7 | 9.92433182 | | | | | 357.70 | 9.77723204 | | | 357.7 | 9.77723204 |
| 357.8 | 4.68757966 | 357.8 | 16.4947887 | 357.8 | 16.4947887 | 357.8 | 9.94394513 | | | | | 357.80 | 9.75761874 | | | 357.8 | 9.75761874 |
| 357.9 | 4.677773 | 357.9 | 16.5340153 | 357.9 | 16.5340153 | 357.9 | 9.96355843 | | | | | 357.90 | 9.77723204 | | | 357.9 | 9.77723204 |
| 358 | 4.64835305 | 358 | 16.5242086 | 358 | 16.5242086 | 358 | 9.96355843 | | | | | 358.00 | 9.7870387 | | | 358 | 9.7870387 |
| 358.1 | 4.64835305 | 358.1 | 16.4947887 | 358.1 | 16.4947887 | 358.1 | 9.95375178 | | | | | 358.10 | 9.77723204 | | | 358.1 | 9.77723204 |
| 358.2 | 4.6581597 | 358.2 | 16.4947887 | 358.2 | 16.4947887 | 358.2 | 9.98317174 | | | | | 358.20 | 9.79684535 | | | 358.2 | 9.79684535 |
| 358.3 | 4.6385464 | 358.3 | 16.514402 | 358.3 | 16.514402 | 358.3 | 10.002785 | | | | | 358.30 | 9.81645865 | | | 358.3 | 9.81645865 |
| 358.4 | 4.66796635 | 358.4 | 16.5340153 | 358.4 | 16.5340153 | 358.4 | 9.99297839 | | | | | 358.40 | 9.81645865 | | | 358.4 | 9.81645865 |
| 358.5 | 4.677773 | 358.5 | 16.5634352 | 358.5 | 16.5634352 | 358.5 | 10.0223983 | | | | | 358.50 | 9.77723204 | | | 358.5 | 9.77723204 |
| 358.6 | 4.72680626 | 358.6 | 16.5928552 | 358.6 | 16.5928552 | 358.6 | 10.0125917 | | | | | 358.60 | 9.77723204 | | | 358.6 | 9.77723204 |
| 358.7 | 4.70719296 | 358.7 | 16.5830485 | 358.7 | 16.5830485 | 358.7 | 9.97336508 | | | | | 358.70 | 9.79684535 | | | 358.7 | 9.79684535 |
| 358.8 | 4.6581597 | 358.8 | 16.5928552 | 358.8 | 16.5928552 | 358.8 | 9.98317174 | | | | | 358.80 | 9.79684535 | | | 358.8 | 9.79684535 |
| 358.9 | 4.64835305 | 358.9 | 16.5634352 | 358.9 | 16.5634352 | 358.9 | 10.002785 | | | | | 358.90 | 9.77723204 | | | 358.9 | 9.77723204 |
| 359 | 4.68757966 | 359 | 16.5830485 | 359 | 16.5830485 | 359 | 9.99297839 | | | | | 359.00 | 9.75761874 | | | 359 | 9.75761874 |
| 359.1 | 4.6581597 | 359.1 | 16.6124685 | 359.1 | 16.6124685 | 359.1 | 9.97336508 | | | | | 359.10 | 9.76742539 | | | 359.1 | 9.76742539 |
| 359.2 | 4.6581597 | 359.2 | 16.6222751 | 359.2 | 16.6222751 | 359.2 | 9.99297839 | | | | | 359.20 | 9.79684535 | | | 359.2 | 9.79684535 |
| 359.3 | 4.70719296 | 359.3 | 16.6222751 | 359.3 | 16.6222751 | 359.3 | 10.0223983 | | | | | 359.30 | 9.7870387 | | | 359.3 | 9.7870387 |
| 359.4 | 4.71699961 | 359.4 | 16.6222751 | 359.4 | 16.6222751 | 359.4 | 10.0223983 | | | | | 359.40 | 9.806652 | | | 359.4 | 9.806652 |
| 359.5 | 4.69738631 | 359.5 | 16.6124685 | 359.5 | 16.6124685 | 359.5 | 10.002785 | | | | | 359.50 | 9.77723204 | | | 359.5 | 9.77723204 |
| 359.6 | 4.71699961 | 359.6 | 16.6124685 | 359.6 | 16.6124685 | 359.6 | 9.98317174 | | | | | 359.60 | 9.74781209 | | | 359.6 | 9.74781209 |
| 359.7 | 4.71699961 | 359.7 | 16.6124685 | 359.7 | 16.6124685 | 359.7 | 9.98317174 | | | | | 359.70 | 9.76742539 | | | 359.7 | 9.76742539 |
| 359.8 | 4.70719296 | 359.8 | 16.6320818 | 359.8 | 16.6320818 | 359.8 | 9.99297839 | | | | | 359.80 | 9.81645865 | | | 359.8 | 9.81645865 |
| 359.9 | 4.70719296 | 359.9 | 16.6418884 | 359.9 | 16.6418884 | 359.9 | 10.0223983 | | | | | 359.90 | 9.806652 | | | 359.9 | 9.806652 |
| 360 | 4.71699961 | 360 | 16.6516951 | 360 | 16.6516951 | 360 | 10.0223983 | | | | | 360.00 | 9.806652 | | | 360 | 9.806652 |
| 360.1 | 4.72680626 | 360.1 | 16.6713084 | 360.1 | 16.6713084 | 360.1 | 10.0125917 | | | | | 360.10 | 9.79684535 | | | 360.1 | 9.79684535 |
| 360.2 | 4.73661292 | 360.2 | 16.6811151 | 360.2 | 16.6811151 | 360.2 | 10.0125917 | | | | | 360.20 | 9.7870387 | | | 360.2 | 9.7870387 |
| 360.3 | 4.70719296 | 360.3 | 16.6713084 | 360.3 | 16.6713084 | 360.3 | 10.0125917 | | | | | 360.30 | 9.8262653 | | | 360.3 | 9.8262653 |
| 360.4 | 4.70719296 | 360.4 | 16.6713084 | 360.4 | 16.6713084 | 360.4 | 9.99297839 | | | | | 360.40 | 9.84587861 | | | 360.4 | 9.84587861 |
| 360.5 | 4.677773 | 360.5 | 16.6909217 | 360.5 | 16.6909217 | 360.5 | 10.002785 | | | | | 360.50 | 9.84587861 | | | 360.5 | 9.84587861 |
| 360.6 | 4.68757966 | 360.6 | 16.710535 | 360.6 | 16.710535 | 360.6 | 10.032205 | | | | | 360.60 | 9.83607196 | | | 360.6 | 9.83607196 |
| 360.7 | 4.72680626 | 360.7 | 16.7203417 | 360.7 | 16.7203417 | 360.7 | 10.0223983 | | | | | 360.70 | 9.8262653 | | | 360.7 | 9.8262653 |
| 360.8 | 4.74641957 | 360.8 | 16.710535 | 360.8 | 16.710535 | 360.8 | 10.0223983 | | | | | 360.80 | 9.806652 | | | 360.8 | 9.806652 |
| 360.9 | 4.75622622 | 360.9 | 16.7301483 | 360.9 | 16.7301483 | 360.9 | 10.002785 | | | | | 360.90 | 9.81645865 | | | 360.9 | 9.81645865 |
| 361 | 4.74641957 | 361 | 16.7497616 | 361 | 16.7497616 | 361 | 10.0125917 | | | | | 361.00 | 9.83607196 | | | 361 | 9.83607196 |
| 361.1 | 4.73661292 | 361.1 | 16.7497616 | 361.1 | 16.7497616 | 361.1 | 10.0223983 | | | | | 361.10 | 9.8262653 | | | 361.1 | 9.8262653 |
| 361.2 | 4.70719296 | 361.2 | 16.7301483 | 361.2 | 16.7301483 | 361.2 | 10.002785 | | | | | 361.20 | 9.81645865 | | | 361.2 | 9.81645865 |
| 361.3 | 4.71699961 | 361.3 | 16.7497616 | 361.3 | 16.7497616 | 361.3 | 10.0125917 | | | | | 361.30 | 9.84587861 | | | 361.3 | 9.84587861 |
| 361.4 | 4.70719296 | 361.4 | 16.7693749 | 361.4 | 16.7693749 | 361.4 | 10.032205 | | | | | 361.40 | 9.84587861 | | | 361.4 | 9.84587861 |
| 361.5 | 4.73661292 | 361.5 | 16.7889882 | 361.5 | 16.7889882 | 361.5 | 10.032205 | | | | | 361.50 | 9.86549191 | | | 361.5 | 9.86549191 |
| 361.6 | 4.73661292 | 361.6 | 16.7987949 | 361.6 | 16.7987949 | 361.6 | 10.0125917 | | | | | 361.60 | 9.88510522 | | | 361.6 | 9.88510522 |
| 361.7 | 4.70719296 | 361.7 | 16.7987949 | 361.7 | 16.7987949 | 361.7 | 10.0420116 | | | | | 361.70 | 9.85568526 | | | 361.7 | 9.85568526 |
| 361.8 | 4.66796635 | 361.8 | 16.7889882 | 361.8 | 16.7889882 | 361.8 | 10.0420116 | | | | | 361.80 | 9.87529856 | | | 361.8 | 9.87529856 |
| 361.9 | 4.71699961 | 361.9 | 16.7987949 | 361.9 | 16.7987949 | 361.9 | 9.98317174 | | | | | 361.90 | 9.88510522 | | | 361.9 | 9.88510522 |
| 362 | 4.73661292 | 362 | 16.8282148 | 362 | 16.8282148 | 362 | 9.97336508 | | | | | 362.00 | 9.86549191 | | | 362 | 9.86549191 |
| 362.1 | 4.77583952 | 362.1 | 16.8380215 | 362.1 | 16.8380215 | 362.1 | 9.97336508 | | | | | 362.10 | 9.85568526 | | | 362.1 | 9.85568526 |
| 362.2 | 4.78564618 | 362.2 | 16.8380215 | 362.2 | 16.8380215 | 362.2 | 9.95375178 | | | | | 362.20 | 9.89491187 | | | 362.2 | 9.89491187 |
| 362.3 | 4.75622622 | 362.3 | 16.8282148 | 362.3 | 16.8282148 | 362.3 | 9.92433182 | | | | | 362.30 | 9.87529856 | | | 362.3 | 9.87529856 |
| 362.4 | 4.75622622 | 362.4 | 16.8576348 | 362.4 | 16.8576348 | 362.4 | 9.91452517 | | | | | 362.40 | 9.90471852 | | | 362.4 | 9.90471852 |
| 362.5 | 4.75622622 | 362.5 | 16.8576348 | 362.5 | 16.8576348 | 362.5 | 9.94394513 | | | | | 362.50 | 9.88510522 | | | 362.5 | 9.88510522 |
| 362.6 | 4.78564618 | 362.6 | 16.8576348 | 362.6 | 16.8576348 | 362.6 | 9.97336508 | | | | | 362.60 | 9.88510522 | | | 362.6 | 9.88510522 |
| 362.7 | 4.76603287 | 362.7 | 16.8870547 | 362.7 | 16.8870547 | 362.7 | 9.92433182 | | | | | 362.70 | 9.88510522 | | | 362.7 | 9.88510522 |
| 362.8 | 4.78564618 | 362.8 | 16.906668 | 362.8 | 16.906668 | 362.8 | 9.92433182 | | | | | 362.80 | 9.88510522 | | | 362.8 | 9.88510522 |
| 362.9 | 4.78564618 | 362.9 | 16.8968614 | 362.9 | 16.8968614 | 362.9 | 9.96355843 | | | | | 362.90 | 9.88510522 | | | 362.9 | 9.88510522 |
| 363 | 4.79545283 | 363 | 16.906668 | 363 | 16.906668 | 363 | 9.96355843 | | | | | 363.00 | 9.91452517 | | | 363 | 9.91452517 |
| 363.1 | 4.75622622 | 363.1 | 16.936088 | 363.1 | 16.936088 | 363.1 | 9.93413848 | | | | | 363.10 | 9.91452517 | | | 363.1 | 9.91452517 |
| 363.2 | 4.73661292 | 363.2 | 16.9262814 | 363.2 | 16.9262814 | 363.2 | 9.92433182 | | | | | 363.20 | 9.92433182 | | | 363.2 | 9.92433182 |

| | | | | | | | | | | | | | | | | | |
|---|---|---|---|---|---|---|---|---|---|---|---|---|---|---|---|---|---|
| 363.3 | 4.73661292 | 363.3 | 16.936088 | 363.3 | 16.936088 | 363.3 | 9.92433182 | | | | | 363.30 | 9.91452517 | | | 363.3 | 9.91452517 |
| 363.4 | 4.74641957 | 363.4 | 16.9557013 | 363.4 | 16.9557013 | 363.4 | 9.94394513 | | | | | 363.40 | 9.93413848 | | | 363.4 | 9.93413848 |
| 363.5 | 4.77583952 | 363.5 | 16.965508 | 363.5 | 16.965508 | 363.5 | 9.96355843 | | | | | 363.50 | 9.92433182 | | | 363.5 | 9.92433182 |
| 363.6 | 4.78564618 | 363.6 | 16.965508 | 363.6 | 16.965508 | 363.6 | 9.94394513 | | | | | 363.60 | 9.91452517 | | | 363.6 | 9.91452517 |
| 363.7 | 4.77583952 | 363.7 | 16.965508 | 363.7 | 16.965508 | 363.7 | 9.96355843 | | | | | 363.70 | 9.94394513 | | | 363.7 | 9.94394513 |
| 363.8 | 4.76603287 | 363.8 | 16.9753146 | 363.8 | 16.9753146 | 363.8 | 9.95375178 | | | | | 363.80 | 9.94394513 | | | 363.8 | 9.94394513 |
| 363.9 | 4.79545283 | 363.9 | 16.9949279 | 363.9 | 16.9949279 | 363.9 | 9.97336508 | | | | | 363.90 | 9.95375178 | | | 363.9 | 9.95375178 |
| 364 | 4.80525948 | 364 | 16.965508 | 364 | 16.965508 | 364 | 10.002785 | | | | | 364.00 | 9.95375178 | | | 364 | 9.95375178 |
| 364.1 | 4.80525948 | 364.1 | 16.9753146 | 364.1 | 16.9753146 | 364.1 | 9.96355843 | | | | | 364.10 | 9.95375178 | | | 364.1 | 9.95375178 |
| 364.2 | 4.78564618 | 364.2 | 16.9949279 | | | 364.2 | 9.94394513 | | | | | 364.20 | 9.94394513 | | | 364.2 | 9.94394513 |
| 364.3 | 4.81506613 | 364.3 | 16.9753146 | | | 364.3 | 9.95375178 | | | | | 364.30 | 9.92433182 | | | 364.3 | 9.92433182 |
| 364.4 | 4.80525948 | 364.4 | 16.9949279 | | | 364.4 | 9.95375178 | | | | | 364.40 | 9.93413848 | | | 364.4 | 9.93413848 |
| 364.5 | 4.78564618 | 364.5 | 17.0145412 | | | 364.5 | 9.97336508 | | | | | 364.50 | 9.95375178 | | | 364.5 | 9.95375178 |
| 364.6 | 4.77583952 | 364.6 | 17.0341545 | | | 364.6 | 9.96355843 | | | | | 364.60 | 9.98317174 | | | 364.6 | 9.98317174 |
| 364.7 | 4.79545283 | 364.7 | 17.0243479 | | | 364.7 | 9.96355843 | | | | | 364.70 | 9.97336508 | | | 364.7 | 9.97336508 |
| 364.8 | 4.79545283 | 364.8 | 17.0439612 | | | 364.8 | 9.96355843 | | | | | 364.80 | 9.98317174 | | | 364.8 | 9.98317174 |
| 364.9 | 4.80525948 | 364.9 | 17.0243479 | | | 364.9 | 9.94394513 | | | | | 364.90 | 9.99297839 | | | 364.9 | 9.99297839 |
| 365 | 4.81506613 | 365 | 17.0047346 | | | 365 | 9.96355843 | | | | | 365.00 | 9.97336508 | | | 365 | 9.97336508 |
| 365.1 | 4.78564618 | 365.1 | 16.9949279 | | | 365.1 | 9.98317174 | | | | | 365.10 | 9.98317174 | | | 365.1 | 9.98317174 |
| 365.2 | 4.80525948 | 365.2 | 17.0145412 | | | 365.2 | 9.98317174 | | | | | 365.20 | 9.98317174 | | | 365.2 | 9.98317174 |
| 365.3 | 4.78564618 | 365.3 | 17.0537678 | | | 365.3 | 9.98317174 | | | | | 365.30 | 9.97336508 | | | 365.3 | 9.97336508 |
| 365.4 | 4.76603287 | 365.4 | 17.0537678 | | | 365.4 | 10.0125917 | | | | | 365.40 | 9.97336508 | | | 365.4 | 9.97336508 |
| 365.5 | 4.75622622 | 365.5 | 17.0733811 | | | 365.5 | 9.99297839 | | | | | 365.50 | 9.98317174 | | | 365.5 | 9.98317174 |
| 365.6 | 4.78564618 | 365.6 | 17.0733811 | | | 365.6 | 9.98317174 | | | | | 365.60 | 10.002785 | | | 365.6 | 10.002785 |
| 365.7 | 4.79545283 | 365.7 | 17.0733811 | | | 365.7 | 9.97336508 | | | | | 365.70 | 10.0518183 | | | 365.7 | 10.0518183 |
| 365.8 | 4.81506613 | 365.8 | 17.0635745 | | | 365.8 | 10.0125917 | | | | | 365.80 | 10.0125917 | | | 365.8 | 10.0125917 |
| 365.9 | 4.82487278 | 365.9 | 17.0635745 | | | 365.9 | 10.0125917 | | | | | 365.90 | 10.0125917 | | | 365.9 | 10.0125917 |
| 366 | 4.82487278 | 366 | 17.0733811 | | | 366 | 9.99297839 | | | | | 366.00 | 10.0125917 | | | 366 | 10.0125917 |
| 366.1 | 4.78564618 | 366.1 | 17.0831878 | | | 366.1 | 10.002785 | | | | | 366.10 | 10.032205 | | | 366.1 | 10.032205 |
| 366.2 | 4.77583952 | 366.2 | 17.1028011 | | | 366.2 | 10.0223983 | | | | | 366.20 | 10.0518183 | | | 366.2 | 10.0518183 |
| 366.3 | 4.80525948 | 366.3 | 17.132221 | | | 366.3 | 10.0125917 | | | | | 366.30 | 10.0518183 | | | 366.3 | 10.0518183 |
| 366.4 | 4.80525948 | 366.4 | 17.132221 | | | 366.4 | 10.0125917 | | | | | 366.40 | 10.0223983 | | | 366.4 | 10.0223983 |
| 366.5 | 4.83467944 | 366.5 | 17.132221 | | | 366.5 | 10.0223983 | | | | | 366.50 | 10.0420116 | | | 366.5 | 10.0420116 |
| 366.6 | 4.84448609 | 366.6 | 17.132221 | | | 366.6 | 10.0125917 | | | | | 366.60 | 10.0714316 | | | 366.6 | 10.0714316 |
| 366.7 | 4.82487278 | 366.7 | 17.1224144 | | | 366.7 | 10.0125917 | | | | | 366.70 | 10.0812383 | | | 366.7 | 10.0812383 |
| 366.8 | 4.82487278 | 366.8 | 17.132221 | | | 366.8 | 10.032205 | | | | | 366.80 | 10.061625 | | | 366.8 | 10.061625 |
| 366.9 | 4.81506613 | 366.9 | 17.1420277 | | | 366.9 | 10.0223983 | | | | | 366.90 | 10.061625 | | | 366.9 | 10.061625 |
| 367 | 4.81506613 | 367 | 17.1714477 | | | 367 | 10.002785 | | | | | 367.00 | 10.0812383 | | | 367 | 10.0812383 |
| 367.1 | 4.84448609 | 367.1 | 17.161641 | | | 367.1 | 10.0223983 | | | | | 367.10 | 10.0714316 | | | 367.1 | 10.0714316 |
| 367.2 | 4.85429274 | 367.2 | 17.1714477 | | | 367.2 | 10.0125917 | | | | | 367.20 | 10.0714316 | | | 367.2 | 10.0714316 |
| 367.3 | 4.84448609 | 367.3 | 17.191061 | | | 367.3 | 10.0125917 | | | | | 367.30 | 10.0910449 | | | 367.3 | 10.0910449 |
| 367.4 | 4.81506613 | 367.4 | 17.191061 | | | 367.4 | 10.032205 | | | | | 367.40 | 10.0812383 | | | 367.4 | 10.0812383 |
| 367.5 | 4.81506613 | 367.5 | 17.191061 | | | 367.5 | 10.032205 | | | | | 367.50 | 10.061625 | | | 367.5 | 10.061625 |
| 367.6 | 4.83467944 | 367.6 | 17.2204809 | | | 367.6 | 10.032205 | | | | | 367.60 | 10.0420116 | | | 367.6 | 10.0420116 |
| 367.7 | 4.84448609 | 367.7 | 17.2204809 | | | 367.7 | 10.061625 | | | | | 367.70 | 10.0910449 | | | 367.7 | 10.0910449 |
| 367.8 | 4.82487278 | 367.8 | 17.2008676 | | | 367.8 | 10.0518183 | | | | | 367.80 | 10.0812383 | | | 367.8 | 10.0812383 |
| 367.9 | 4.87390604 | 367.9 | 17.2008676 | | | 367.9 | 10.0812383 | | | | | 367.90 | 10.0420116 | | | 367.9 | 10.0420116 |
| 368 | 4.87390604 | 368 | 17.2204809 | | | 368 | 10.0714316 | | | | | 368.00 | 10.0812383 | | | 368 | 10.0812383 |
| 368.1 | 4.84448609 | 368.1 | 17.2106743 | | | 368.1 | 10.0518183 | | | | | 368.10 | 10.0812383 | | | 368.1 | 10.0812383 |
| 368.2 | 4.86409939 | 368.2 | 17.2302876 | | | 368.2 | 10.061625 | | | | | 368.20 | 10.0910449 | | | 368.2 | 10.0910449 |
| 368.3 | 4.8837127 | 368.3 | 17.2597075 | | | 368.3 | 10.061625 | | | | | 368.30 | 10.1008516 | | | 368.3 | 10.1008516 |
| 368.4 | 4.87390604 | 368.4 | 17.2793208 | | | 368.4 | 10.061625 | | | | | 368.40 | 10.1302715 | | | 368.4 | 10.1302715 |
| 368.5 | 4.87390604 | 368.5 | 17.2989341 | | | 368.5 | 10.0714316 | | | | | 368.50 | 10.1400782 | | | 368.5 | 10.1400782 |
| 368.6 | 4.87390604 | 368.6 | 17.3185474 | | | 368.6 | 10.0812383 | | | | | 368.60 | 10.1106582 | | | 368.6 | 10.1106582 |
| 368.7 | 4.83467944 | 368.7 | 17.2891275 | | | 368.7 | 10.0812383 | | | | | 368.70 | 10.1106582 | | | 368.7 | 10.1106582 |
| 368.8 | 4.84448609 | 368.8 | 17.2891275 | | | 368.8 | 10.0714316 | | | | | 368.80 | 10.1204649 | | | 368.8 | 10.1204649 |
| 368.9 | 4.86409939 | 368.9 | 17.3087408 | | | 368.9 | 10.0518183 | | | | | 368.90 | 10.1400782 | | | 368.9 | 10.1400782 |
| 369 | 4.86409939 | 369 | 17.3283541 | | | 369 | 10.061625 | | | | | 369.00 | 10.1302715 | | | 369 | 10.1302715 |
| 369.1 | 4.87390604 | 369.1 | 17.3283541 | | | 369.1 | 10.0518183 | | | | | 369.10 | 10.1400782 | | | 369.1 | 10.1400782 |
| 369.2 | 4.87390604 | 369.2 | 17.3479674 | | | 369.2 | 10.032205 | | | | | 369.20 | 10.1302715 | | | 369.2 | 10.1302715 |
| 369.3 | 4.87390604 | 369.3 | 17.387194 | | | 369.3 | 10.032205 | | | | | 369.30 | 10.1106582 | | | 369.3 | 10.1106582 |

| | | | | | | | | | | | | | | | | | |
|---|---|---|---|---|---|---|---|---|---|---|---|---|---|---|---|---|---|
| 369.4 | 4.86409939 | 369.4 | 17.3970006 | | | 369.4 | 10.0518183 | | | | | 369.40 | 10.1400782 | | | 369.4 | 10.1400782 |
| 369.5 | 4.84448609 | 369.5 | 17.3675807 | | | 369.5 | 10.032205 | | | | | 369.50 | 10.1498848 | | | 369.5 | 10.1498848 |
| 369.6 | 4.84448609 | 369.6 | 17.3773873 | | | 369.6 | 10.002785 | | | | | 369.60 | 10.1400782 | | | 369.6 | 10.1400782 |
| 369.7 | 4.86409939 | 369.7 | 17.3970006 | | | 369.7 | 10.002785 | | | | | 369.70 | 10.1694981 | | | 369.7 | 10.1694981 |
| 369.8 | 4.8837127 | 369.8 | 17.4362273 | | | 369.8 | 10.0125917 | | | | | 369.80 | 10.2087247 | | | 369.8 | 10.2087247 |
| 369.9 | 4.89351935 | 369.9 | 17.416614 | | | 369.9 | 9.95375178 | | | | | 369.90 | 10.1596915 | | | 369.9 | 10.1596915 |
| 370 | 4.8837127 | 370 | 17.4068073 | | | 370 | 9.83607196 | | | | | 370.00 | 10.1596915 | | | 370 | 10.1596915 |
| 370.1 | 4.903326 | 370.1 | 17.4264206 | | | 370.1 | 9.77723204 | | | | | 370.10 | 10.1596915 | | | 370.1 | 10.1596915 |
| 370.2 | 4.8837127 | 370.2 | 17.4460339 | | | 370.2 | 9.72819878 | | | | | 370.20 | 10.1302715 | | | 370.2 | 10.1302715 |
| 370.3 | 4.89351935 | 370.3 | 17.4460339 | | | 370.3 | 9.70858548 | | | | | 370.30 | 10.1498848 | | | 370.3 | 10.1498848 |
| 370.4 | 4.91313265 | 370.4 | 17.4558406 | | | 370.4 | 9.70858548 | | | | | 370.40 | 10.1989181 | | | 370.4 | 10.1989181 |
| 370.5 | 4.85429274 | 370.5 | 17.4460339 | | | 370.5 | 9.75761874 | | | | | 370.50 | 10.2087247 | | | 370.5 | 10.2087247 |
| 370.6 | 4.86409939 | 370.6 | 17.4656472 | | | 370.6 | 9.76742539 | | | | | 370.60 | 10.228338 | | | 370.6 | 10.228338 |
| 370.7 | 4.87390604 | 370.7 | 17.4852605 | | | 370.7 | 9.77723204 | | | | | 370.70 | 10.2381447 | | | 370.7 | 10.2381447 |
| 370.8 | 4.8837127 | 370.8 | 17.4754539 | | | 370.8 | 9.7870387 | | | | | 370.80 | 10.2381447 | | | 370.8 | 10.2381447 |
| 370.9 | 4.91313265 | 370.9 | 17.4754539 | | | 370.9 | 9.75761874 | | | | | 370.90 | 10.2185314 | | | 370.9 | 10.2185314 |
| 371 | 4.89351935 | 371 | 17.4656472 | | | 371 | 9.7870387 | | | | | 371.00 | 10.228338 | | | 371 | 10.228338 |
| 371.1 | 4.89351935 | 371.1 | 17.4754539 | | | 371.1 | 9.7870387 | | | | | 371.10 | 10.228338 | | | 371.1 | 10.228338 |
| 371.2 | 4.89351935 | 371.2 | 17.5048738 | | | 371.2 | 9.76742539 | | | | | 371.20 | 10.2381447 | | | 371.2 | 10.2381447 |
| 371.3 | 4.89351935 | 371.3 | 17.5539071 | | | 371.3 | 9.79684535 | | | | | 371.30 | 10.2381447 | | | 371.3 | 10.2381447 |
| 371.4 | 4.89351935 | 371.4 | 17.5244871 | | | 371.4 | 9.79684535 | | | | | 371.40 | 10.2381447 | | | 371.4 | 10.2381447 |
| 371.5 | 4.91313265 | 371.5 | 17.4950672 | | | 371.5 | 9.83607196 | | | | | 371.50 | 10.257758 | | | 371.5 | 10.257758 |
| 371.6 | 4.91313265 | 371.6 | 17.5342938 | | | 371.6 | 9.86549191 | | | | | 371.60 | 10.2675646 | | | 371.6 | 10.2675646 |
| 371.7 | 4.9229393 | 371.7 | 17.5048738 | | | 371.7 | 9.85568526 | | | | | 371.70 | 10.257758 | | | 371.7 | 10.257758 |
| 371.8 | 4.903326 | 371.8 | 17.5441004 | | | 371.8 | 9.83607196 | | | | | 371.80 | 10.257758 | | | 371.8 | 10.257758 |
| 371.9 | 4.91313265 | 371.9 | 17.5637137 | | | 371.9 | 9.81645865 | | | | | 371.90 | 10.257758 | | | 371.9 | 10.257758 |
| 372 | 4.93274596 | 372 | 17.5637137 | | | 372 | 9.83607196 | | | | | 372.00 | 10.257758 | | | 372 | 10.257758 |
| 372.1 | 4.93274596 | 372.1 | 17.5931337 | | | 372.1 | 9.87529856 | | | | | 372.10 | 10.2773713 | | | 372.1 | 10.2773713 |
| 372.2 | 4.94255261 | 372.2 | 17.583327 | | | 372.2 | 9.87529856 | | | | | 372.20 | 10.2969846 | | | 372.2 | 10.2969846 |
| 372.3 | 4.93274596 | 372.3 | 17.583327 | | | 372.3 | 9.87529856 | | | | | 372.30 | 10.3067913 | | | 372.3 | 10.3067913 |
| 372.4 | 4.93274596 | 372.4 | 17.5931337 | | | 372.4 | 9.89491187 | | | | | 372.40 | 10.3165979 | | | 372.4 | 10.3165979 |
| 372.5 | 4.95235926 | 372.5 | 17.5931337 | | | 372.5 | 9.89491187 | | | | | 372.50 | 10.3067913 | | | 372.5 | 10.3067913 |
| 372.6 | 4.95235926 | 372.6 | 17.6029403 | | | 372.6 | 9.91452517 | | | | | 372.60 | 10.3165979 | | | 372.6 | 10.3165979 |
| 372.7 | 4.98177922 | 372.7 | 17.5931337 | | | 372.7 | 9.88510522 | | | | | 372.70 | 10.3362112 | | | 372.7 | 10.3362112 |
| 372.8 | 4.97197256 | 372.8 | 17.5539071 | | | 372.8 | 9.90471852 | | | | | 372.80 | 10.3067913 | | | 372.8 | 10.3067913 |
| 372.9 | 4.94255261 | 372.9 | 17.5735204 | | | 372.9 | 9.93413848 | | | | | 372.90 | 10.3165979 | | | 372.9 | 10.3165979 |
| 373 | 4.94255261 | 373 | 17.5735204 | | | 373 | 9.92433182 | | | | | 373.00 | 10.3264046 | | | 373 | 10.3264046 |
| 373.1 | 4.96216591 | 373.1 | 17.583327 | | | 373.1 | 9.94394513 | | | | | 373.10 | 10.3165979 | | | 373.1 | 10.3165979 |
| 373.2 | 4.96216591 | 373.2 | 17.583327 | | | 373.2 | 9.93413848 | | | | | 373.20 | 10.3067913 | | | 373.2 | 10.3067913 |
| 373.3 | 4.98177922 | 373.3 | 17.5441004 | | | 373.3 | 9.93413848 | | | | | 373.30 | 10.2969846 | | | 373.3 | 10.2969846 |
| 373.4 | 4.95235926 | 373.4 | 17.5539071 | | | 373.4 | 9.92433182 | | | | | 373.40 | 10.3165979 | | | 373.4 | 10.3165979 |
| 373.5 | 4.94255261 | 373.5 | 17.5637137 | | | 373.5 | 9.91452517 | | | | | 373.50 | 10.3460179 | | | 373.5 | 10.3460179 |
| 373.6 | 4.93274596 | 373.6 | 17.5637137 | | | 373.6 | 9.93413848 | | | | | 373.60 | 10.3558245 | | | 373.6 | 10.3558245 |
| 373.7 | 4.94255261 | 373.7 | 17.583327 | | | 373.7 | 9.93413848 | | | | | 373.70 | 10.3656312 | | | 373.7 | 10.3656312 |
| 373.8 | 4.94255261 | 373.8 | 17.6029403 | | | 373.8 | 9.92433182 | | | | | 373.80 | 10.3558245 | | | 373.8 | 10.3558245 |
| 373.9 | 4.95235926 | 373.9 | 17.5637137 | | | 373.9 | 9.91452517 | | | | | 373.90 | 10.3460179 | | | 373.9 | 10.3460179 |
| 374 | 4.94255261 | 374 | 17.5637137 | | | 374 | 9.93413848 | | | | | 374.00 | 10.3558245 | | | 374 | 10.3558245 |
| 374.1 | 4.9229393 | 374.1 | 17.5735204 | | | 374.1 | 9.97336508 | | | | | 374.10 | 10.3656312 | | | 374.1 | 10.3656312 |
| 374.2 | 4.95235926 | 374.2 | 17.5637137 | | | 374.2 | 9.98317174 | | | | | 374.20 | 10.3656312 | | | 374.2 | 10.3656312 |
| 374.3 | 4.96216591 | 374.3 | 17.5539071 | | | 374.3 | 9.97336508 | | | | | 374.30 | 10.3558245 | | | 374.3 | 10.3558245 |
| 374.4 | 4.95235926 | 374.4 | 17.5735204 | | | 374.4 | 9.97336508 | | | | | 374.40 | 10.3264046 | | | 374.4 | 10.3264046 |
| 374.5 | 4.95235926 | 374.5 | 17.612747 | | | 374.5 | 9.98317174 | | | | | 374.50 | 10.3264046 | | | 374.5 | 10.3264046 |
| 374.6 | 4.99158587 | 374.6 | 17.5931337 | | | 374.6 | 9.98317174 | | | | | 374.60 | 10.3362112 | | | 374.6 | 10.3362112 |
| 374.7 | 5.00139252 | 374.7 | 17.583327 | | | 374.7 | 10.0125917 | | | | | 374.70 | 10.3460179 | | | 374.7 | 10.3460179 |
| 374.8 | 5.01119917 | 374.8 | 17.6029403 | | | 374.8 | 10.002785 | | | | | 374.80 | 10.3460179 | | | 374.8 | 10.3460179 |
| 374.9 | 5.00139252 | 374.9 | 17.5637137 | | | 374.9 | 10.0125917 | | | | | 374.90 | 10.3362112 | | | 374.9 | 10.3362112 |
| 375 | 5.00139252 | 375 | 17.583327 | | | 375 | 10.032205 | | | | | 375.00 | 10.3362112 | | | 375 | 10.3362112 |
| 375.1 | 4.98177922 | 375.1 | 17.5441004 | | | 375.1 | 10.0420116 | | | | | 375.10 | 10.3460179 | | | 375.1 | 10.3460179 |
| 375.2 | 4.98177922 | 375.2 | 17.5539071 | | | 375.2 | 10.0223983 | | | | | 375.20 | 10.3754378 | | | 375.2 | 10.3754378 |
| 375.3 | 4.99158587 | 375.3 | 17.5441004 | | | 375.3 | 10.0223983 | | | | | 375.30 | 10.3852445 | | | 375.3 | 10.3852445 |
| 375.4 | 5.00139252 | 375.4 | 17.5441004 | | | 375.4 | 10.032205 | | | | | 375.40 | 10.3754378 | | | 375.4 | 10.3754378 |

| | | | | | | | | | | | | | | | | | |
|---|---|---|---|---|---|---|---|---|---|---|---|---|---|---|---|---|---|
| 375.5 | 5.00139252 | 375.5 | 17.5735204 | | | 375.5 | 10.0518183 | | | | | 375.50 | 10.3754378 | | | 375.5 | 10.3754378 |
| 375.6 | 4.99158587 | 375.6 | 17.5931337 | | | 375.6 | 10.032205 | | | | | 375.60 | 10.3852445 | | | 375.6 | 10.3852445 |
| 375.7 | 4.99158587 | 375.7 | 17.5735204 | | | 375.7 | 10.032205 | | | | | 375.70 | 10.3656312 | | | 375.7 | 10.3656312 |
| 375.8 | 4.97197256 | 375.8 | 17.5735204 | | | 375.8 | 10.0420116 | | | | | 375.80 | 10.3852445 | | | 375.8 | 10.3852445 |
| 375.9 | 4.98177922 | 375.9 | 17.583327 | | | 375.9 | 10.032205 | | | | | 375.90 | 10.3950511 | | | 375.9 | 10.3950511 |
| 376 | 5.00139252 | 376 | 17.5735204 | | | 376 | 10.0518183 | | | | | 376.00 | 10.4048578 | | | 376 | 10.4048578 |
| 376.1 | 5.03081248 | 376.1 | 17.5539071 | | | 376.1 | 10.0714316 | | | | | 376.10 | 10.4146644 | | | 376.1 | 10.4146644 |
| 376.2 | 4.99158587 | 376.2 | 17.5342938 | | | 376.2 | 10.0714316 | | | | | 376.20 | 10.3852445 | | | 376.2 | 10.3852445 |
| 376.3 | 4.98177922 | 376.3 | 17.5441004 | | | 376.3 | 10.0714316 | | | | | 376.30 | 10.4244711 | | | 376.3 | 10.4244711 |
| 376.4 | 5.00139252 | 376.4 | 17.5244871 | | | 376.4 | 10.061625 | | | | | 376.40 | 10.4146644 | | | 376.4 | 10.4146644 |
| 376.5 | 4.99158587 | 376.5 | 17.5441004 | | | 376.5 | 10.0518183 | | | | | 376.50 | 10.4244711 | | | 376.5 | 10.4244711 |
| 376.6 | 4.97197256 | 376.6 | 17.5735204 | | | 376.6 | 10.0518183 | | | | | 376.60 | 10.4146644 | | | 376.6 | 10.4146644 |
| 376.7 | 4.99158587 | 376.7 | 17.5735204 | | | 376.7 | 10.0518183 | | | | | 376.70 | 10.4244711 | | | 376.7 | 10.4244711 |
| 376.8 | 4.98177922 | 376.8 | 17.5539071 | | | 376.8 | 10.0518183 | | | | | 376.80 | 10.4342777 | | | 376.8 | 10.4342777 |
| 376.9 | 4.98177922 | 376.9 | 17.5342938 | | | 376.9 | 10.0910449 | | | | | 376.90 | 10.4440844 | | | 376.9 | 10.4440844 |
| 377 | 5.01119917 | 377 | 17.5441004 | | | 377 | 10.0910449 | | | | | 377.00 | 10.4735043 | | | 377 | 10.4735043 |
| 377.1 | 5.01119917 | 377.1 | 17.5342938 | | | 377.1 | 10.1008516 | | | | | 377.10 | 10.4931176 | | | 377.1 | 10.4931176 |
| 377.2 | 5.00139252 | 377.2 | 17.5342938 | | | 377.2 | 10.1106582 | | | | | 377.20 | 10.453891 | | | 377.2 | 10.453891 |
| 377.3 | 5.01119917 | 377.3 | 17.5441004 | | | 377.3 | 10.1106582 | | | | | 377.30 | 10.4342777 | | | 377.3 | 10.4342777 |
| 377.4 | 5.03081248 | 377.4 | 17.5735204 | | | 377.4 | 10.1400782 | | | | | 377.40 | 10.4244711 | | | 377.4 | 10.4244711 |
| 377.5 | 5.01119917 | 377.5 | 17.5735204 | | | 377.5 | 10.1498848 | | | | | 377.50 | 10.483311 | | | 377.5 | 10.483311 |
| 377.6 | 5.00139252 | 377.6 | 17.5637137 | | | 377.6 | 10.1204649 | | | | | 377.60 | 10.4440844 | | | 377.6 | 10.4440844 |
| 377.7 | 5.02100582 | 377.7 | 17.5244871 | | | 377.7 | 10.1008516 | | | | | 377.70 | 10.4735043 | | | 377.7 | 10.4735043 |
| 377.8 | 5.03081248 | 377.8 | 17.5146805 | | | 377.8 | 10.1008516 | | | | | 377.80 | 10.483311 | | | 377.8 | 10.483311 |
| 377.9 | 5.01119917 | 377.9 | 17.5244871 | | | 377.9 | 10.1008516 | | | | | 377.90 | 10.483311 | | | 377.9 | 10.483311 |
| 378 | 5.03081248 | 378 | 17.5637137 | | | 378 | 10.1106582 | | | | | 378.00 | 10.4931176 | | | 378 | 10.4931176 |
| 378.1 | 5.05042578 | 378.1 | 17.5539071 | | | 378.1 | 10.1204649 | | | | | 378.10 | 10.4735043 | | | 378.1 | 10.4735043 |
| 378.2 | 5.02100582 | 378.2 | 17.5342938 | | | 378.2 | 10.1498848 | | | | | 378.20 | 10.4735043 | | | 378.2 | 10.4735043 |
| 378.3 | 5.00139252 | 378.3 | 17.5342938 | | | 378.3 | 10.1498848 | | | | | 378.30 | 10.5323442 | | | 378.3 | 10.5323442 |
| 378.4 | 5.03081248 | 378.4 | 17.5637137 | | | 378.4 | 10.1498848 | | | | | 378.40 | 10.5323442 | | | 378.4 | 10.5323442 |
| 378.5 | 5.06023243 | 378.5 | 17.5735204 | | | 378.5 | 10.1694981 | | | | | 378.50 | 10.5127309 | | | 378.5 | 10.5127309 |
| 378.6 | 5.07003908 | 378.6 | 17.5637137 | | | 378.6 | 10.1302715 | | | | | 378.60 | 10.5127309 | | | 378.6 | 10.5127309 |
| 378.7 | 5.04061913 | 378.7 | 17.5539071 | | | 378.7 | 10.1498848 | | | | | 378.70 | 10.5029243 | | | 378.7 | 10.5029243 |
| 378.8 | 5.03081248 | 378.8 | 17.5441004 | | | 378.8 | 10.1400782 | | | | | 378.80 | 10.5127309 | | | 378.8 | 10.5127309 |
| 378.9 | 5.02100582 | 378.9 | 17.5342938 | | | 378.9 | 10.1498848 | | | | | 378.90 | 10.5421509 | | | 378.9 | 10.5421509 |
| 379 | 5.03081248 | 379 | 17.5048738 | | | 379 | 10.1694981 | | | | | 379.00 | 10.5225376 | | | 379 | 10.5225376 |
| 379.1 | 5.05042578 | 379.1 | 17.5048738 | | | 379.1 | 10.1694981 | | | | | 379.10 | 10.5127309 | | | 379.1 | 10.5127309 |
| 379.2 | 5.06023243 | 379.2 | 17.5244871 | | | 379.2 | 10.1498848 | | | | | 379.20 | 10.5127309 | | | 379.2 | 10.5127309 |
| 379.3 | 5.05042578 | 379.3 | 17.5048738 | | | 379.3 | 10.1694981 | | | | | 379.30 | 10.5225376 | | | 379.3 | 10.5225376 |
| 379.4 | 5.05042578 | 379.4 | 17.5342938 | | | 379.4 | 10.1793048 | | | | | 379.40 | 10.5421509 | | | 379.4 | 10.5421509 |
| 379.5 | 5.03081248 | 379.5 | 17.5342938 | | | 379.5 | 10.1793048 | | | | | 379.50 | 10.5225376 | | | 379.5 | 10.5225376 |
| 379.6 | 5.03081248 | 379.6 | 17.5244871 | | | 379.6 | 10.1498848 | | | | | 379.60 | 10.5127309 | | | 379.6 | 10.5127309 |
| 379.7 | 5.05042578 | 379.7 | 17.5146805 | | | 379.7 | 10.1498848 | | | | | 379.70 | 10.5323442 | | | 379.7 | 10.5323442 |
| 379.8 | 5.07003908 | 379.8 | 17.5342938 | | | 379.8 | 10.1694981 | | | | | 379.80 | 10.5421509 | | | 379.8 | 10.5421509 |
| 379.9 | 5.10926569 | 379.9 | 17.5244871 | | | 379.9 | 10.1891114 | | | | | 379.90 | 10.5127309 | | | 379.9 | 10.5127309 |
| 380 | 5.07003908 | 380 | 17.5342938 | | | 380 | 10.1694981 | | | | | 380.00 | 10.5421509 | | | 380 | 10.5421509 |
| 380.1 | 5.07003908 | 380.1 | 17.5441004 | | | 380.1 | 10.1596915 | | | | | 380.10 | 10.5519576 | | | 380.1 | 10.5519576 |
| 380.2 | 5.07984574 | 380.2 | 17.5146805 | | | 380.2 | 10.1793048 | | | | | 380.20 | 10.5617642 | | | 380.2 | 10.5617642 |
| 380.3 | 5.08965239 | 380.3 | 17.5342938 | | | 380.3 | 10.1989181 | | | | | 380.30 | 10.5813775 | | | 380.3 | 10.5813775 |
| 380.4 | 5.08965239 | 380.4 | 17.5637137 | | | 380.4 | 10.1989181 | | | | | 380.40 | 10.5715709 | | | 380.4 | 10.5715709 |
| 380.5 | 5.07003908 | 380.5 | 17.5342938 | | | 380.5 | 10.1793048 | | | | | 380.50 | 10.5715709 | | | 380.5 | 10.5715709 |
| 380.6 | 5.07984574 | 380.6 | 17.5244871 | | | 380.6 | 10.1891114 | | | | | 380.60 | 10.5911842 | | | 380.6 | 10.5911842 |
| 380.7 | 5.07003908 | 380.7 | 17.5048738 | | | 380.7 | 10.1793048 | | | | | 380.70 | 10.5715709 | | | 380.7 | 10.5715709 |
| 380.8 | 5.07984574 | 380.8 | 17.5048738 | | | 380.8 | 10.1989181 | | | | | 380.80 | 10.5519576 | | | 380.8 | 10.5519576 |
| 380.9 | 5.10926569 | 380.9 | 17.4852605 | | | 380.9 | 10.2087247 | | | | | 380.90 | 10.5715709 | | | 380.9 | 10.5715709 |
| 381 | 5.08965239 | 381 | 17.4656472 | | | 381 | 10.2185314 | | | | | 381.00 | 10.6009908 | | | 381 | 10.6009908 |
| 381.1 | 5.06023243 | 381.1 | 17.4460339 | | | 381.1 | 10.2185314 | | | | | 381.10 | 10.5617642 | | | 381.1 | 10.5617642 |
| 381.2 | 5.07003908 | 381.2 | 17.4656472 | | | 381.2 | 10.2185314 | | | | | 381.20 | 10.5617642 | | | 381.2 | 10.5617642 |
| 381.3 | 5.08965239 | 381.3 | 17.4852605 | | | 381.3 | 10.228338 | | | | | 381.30 | 10.5715709 | | | 381.3 | 10.5715709 |
| 381.4 | 5.09945904 | 381.4 | 17.4754539 | | | 381.4 | 10.2185314 | | | | | 381.40 | 10.5911842 | | | 381.4 | 10.5911842 |
| 381.5 | 5.07003908 | 381.5 | 17.4754539 | | | 381.5 | 10.1989181 | | | | | 381.50 | 10.6304108 | | | 381.5 | 10.6304108 |

| | | | | | | | | | | | | | | | | | |
|---|---|---|---|---|---|---|---|---|---|---|---|---|---|---|---|---|---|
| 381.6 | 5.06023243 | 381.6 | 17.4852605 | | | 381.6 | 10.2087247 | | | | | 381.60 | 10.6107975 | | | 381.6 | 10.6107975 |
| 381.7 | 5.07003908 | 381.7 | 17.4950672 | | | 381.7 | 10.228338 | | | | | 381.70 | 10.6206041 | | | 381.7 | 10.6206041 |
| 381.8 | 5.08965239 | 381.8 | 17.5539071 | | | 381.8 | 10.228338 | | | | | 381.80 | 10.6402174 | | | 381.8 | 10.6402174 |
| 381.9 | 5.09945904 | 381.9 | 17.5146805 | | | 381.9 | 10.2185314 | | | | | 381.90 | 10.6304108 | | | 381.9 | 10.6304108 |
| 382 | 5.10926569 | 382 | 17.4852605 | | | 382 | 10.228338 | | | | | 382.00 | 10.6206041 | | | 382 | 10.6206041 |
| 382.1 | 5.10926569 | 382.1 | 17.4950672 | | | 382.1 | 10.228338 | | | | | 382.10 | 10.6304108 | | | 382.1 | 10.6304108 |
| 382.2 | 5.11907234 | 382.2 | 17.4852605 | | | 382.2 | 10.2381447 | | | | | 382.20 | 10.679444 | | | 382.2 | 10.679444 |
| 382.3 | 5.128879 | 382.3 | 17.4754539 | | | 382.3 | 10.228338 | | | | | 382.30 | 10.6696374 | | | 382.3 | 10.6696374 |
| 382.4 | 5.11907234 | 382.4 | 17.5048738 | | | 382.4 | 10.2087247 | | | | | 382.40 | 10.6402174 | | | 382.4 | 10.6402174 |
| 382.5 | 5.128879 | 382.5 | 17.4950672 | | | 382.5 | 10.2185314 | | | | | 382.50 | 10.6598307 | | | 382.5 | 10.6598307 |
| 382.6 | 5.13868565 | 382.6 | 17.5048738 | | | 382.6 | 10.257758 | | | | | 382.60 | 10.6500241 | | | 382.6 | 10.6500241 |
| 382.7 | 5.13868565 | 382.7 | 17.4852605 | | | 382.7 | 10.2381447 | | | | | 382.70 | 10.6696374 | | | 382.7 | 10.6696374 |
| 382.8 | 5.15829895 | 382.8 | 17.4950672 | | | 382.8 | 10.257758 | | | | | 382.80 | 10.6696374 | | | 382.8 | 10.6696374 |
| 382.9 | 5.13868565 | 382.9 | 17.4852605 | | | 382.9 | 10.2479513 | | | | | 382.90 | 10.6206041 | | | 382.9 | 10.6206041 |
| 383 | 5.11907234 | 383 | 17.4656472 | | | 383 | 10.2675646 | | | | | 383.00 | 10.6206041 | | | 383 | 10.6206041 |
| 383.1 | 5.13868565 | 383.1 | 17.4852605 | | | 383.1 | 10.257758 | | | | | 383.10 | 10.6598307 | | | 383.1 | 10.6598307 |
| 383.2 | 5.15829895 | 383.2 | 17.4950672 | | | 383.2 | 10.2675646 | | | | | 383.20 | 10.6598307 | | | 383.2 | 10.6598307 |
| 383.3 | 5.13868565 | 383.3 | 17.4754539 | | | 383.3 | 10.2969846 | | | | | 383.30 | 10.6598307 | | | 383.3 | 10.6598307 |
| 383.4 | 5.10926569 | 383.4 | 17.4950672 | | | 383.4 | 10.2675646 | | | | | 383.40 | 10.6500241 | | | 383.4 | 10.6500241 |
| 383.5 | 5.13868565 | 383.5 | 17.4754539 | | | 383.5 | 10.2871779 | | | | | 383.50 | 10.6598307 | | | 383.5 | 10.6598307 |
| 383.6 | 5.15829895 | 383.6 | 17.4754539 | | | 383.6 | 10.2969846 | | | | | 383.60 | 10.6892507 | | | 383.6 | 10.6892507 |
| 383.7 | 5.1484923 | 383.7 | 17.5048738 | | | 383.7 | 10.257758 | | | | | 383.70 | 10.679444 | | | 383.7 | 10.679444 |
| 383.8 | 5.13868565 | 383.8 | 17.5146805 | | | 383.8 | 10.2773713 | | | | | 383.80 | 10.6696374 | | | 383.8 | 10.6696374 |
| 383.9 | 5.11907234 | 383.9 | 17.4852605 | | | 383.9 | 10.2969846 | | | | | 383.90 | 10.6696374 | | | 383.9 | 10.6696374 |
| 384 | 5.10926569 | 384 | 17.4656472 | | | 384 | 10.2773713 | | | | | 384.00 | 10.6892507 | | | 384 | 10.6892507 |
| 384.1 | 5.10926569 | 384.1 | 17.4852605 | | | 384.1 | 10.2969846 | | | | | 384.10 | 10.679444 | | | 384.1 | 10.679444 |
| 384.2 | 5.13868565 | 384.2 | 17.5048738 | | | 384.2 | 10.2969846 | | | | | 384.20 | 10.6892507 | | | 384.2 | 10.6892507 |
| 384.3 | 5.13868565 | 384.3 | 17.4852605 | | | 384.3 | 10.2969846 | | | | | 384.30 | 10.6892507 | | | 384.3 | 10.6892507 |
| 384.4 | 5.1484923 | 384.4 | 17.4558406 | | | 384.4 | 10.2969846 | | | | | 384.40 | 10.6696374 | | | 384.4 | 10.6696374 |
| 384.5 | 5.15829895 | 384.5 | 17.4852605 | | | 384.5 | 10.2969846 | | | | | 384.50 | 10.6892507 | | | 384.5 | 10.6892507 |
| 384.6 | 5.1484923 | 384.6 | 17.4950672 | | | 384.6 | 10.2773713 | | | | | 384.60 | 10.6990573 | | | 384.6 | 10.6990573 |
| 384.7 | 5.128879 | 384.7 | 17.4754539 | | | 384.7 | 10.2871779 | | | | | 384.70 | 10.7186706 | | | 384.7 | 10.7186706 |
| 384.8 | 5.17791226 | 384.8 | 17.4852605 | | | 384.8 | 10.2969846 | | | | | 384.80 | 10.7186706 | | | 384.8 | 10.7186706 |
| 384.9 | 5.17791226 | 384.9 | 17.4852605 | | | 384.9 | 10.2871779 | | | | | 384.90 | 10.6892507 | | | 384.9 | 10.6892507 |
| 385 | 5.1681056 | 385 | 17.4852605 | | | 385 | 10.3067913 | | | | | 385.00 | 10.679444 | | | 385 | 10.679444 |
| 385.1 | 5.1484923 | 385.1 | 17.4754539 | | | 385.1 | 10.3558245 | | | | | 385.10 | 10.708864 | | | 385.1 | 10.708864 |
| 385.2 | 5.128879 | 385.2 | 17.4656472 | | | 385.2 | 10.3362112 | | | | | 385.20 | 10.708864 | | | 385.2 | 10.708864 |
| 385.3 | 5.15829895 | 385.3 | 17.4754539 | | | 385.3 | 10.3362112 | | | | | 385.30 | 10.708864 | | | 385.3 | 10.708864 |
| 385.4 | 5.19752556 | 385.4 | 17.5048738 | | | 385.4 | 10.3460179 | | | | | 385.40 | 10.7186706 | | | 385.4 | 10.7186706 |
| 385.5 | 5.19752556 | 385.5 | 17.4754539 | | | 385.5 | 10.3362112 | | | | | 385.50 | 10.7284773 | | | 385.5 | 10.7284773 |
| 385.6 | 5.1681056 | 385.6 | 17.4460339 | | | 385.6 | 10.3362112 | | | | | 385.60 | 10.6990573 | | | 385.6 | 10.6990573 |
| 385.7 | 5.15829895 | 385.7 | 17.4656472 | | | 385.7 | 10.3460179 | | | | | 385.70 | 10.708864 | | | 385.7 | 10.708864 |
| 385.8 | 5.15829895 | 385.8 | 17.4754539 | | | 385.8 | 10.3558245 | | | | | 385.80 | 10.7382839 | | | 385.8 | 10.7382839 |
| 385.9 | 5.13868565 | 385.9 | 17.4852605 | | | 385.9 | 10.3558245 | | | | | 385.90 | 10.7480906 | | | 385.9 | 10.7480906 |
| 386 | 5.15829895 | 386 | 17.4754539 | | | 386 | 10.3558245 | | | | | 386.00 | 10.7284773 | | | 386 | 10.7284773 |
| 386.1 | 5.1681056 | 386.1 | 17.4754539 | | | 386.1 | 10.3558245 | | | | | 386.10 | 10.7677039 | | | 386.1 | 10.7677039 |
| 386.2 | 5.17791226 | 386.2 | 17.4852605 | | | 386.2 | 10.3460179 | | | | | 386.20 | 10.7578972 | | | 386.2 | 10.7578972 |
| 386.3 | 5.15829895 | 386.3 | 17.4950672 | | | 386.3 | 10.3656312 | | | | | 386.30 | 10.7677039 | | | 386.3 | 10.7677039 |
| 386.4 | 5.17791226 | 386.4 | 17.5048738 | | | 386.4 | 10.3754378 | | | | | 386.40 | 10.7677039 | | | 386.4 | 10.7677039 |
| 386.5 | 5.15829895 | 386.5 | 17.4950672 | | | 386.5 | 10.3950511 | | | | | 386.50 | 10.7677039 | | | 386.5 | 10.7677039 |
| 386.6 | 5.1484923 | 386.6 | 17.4852605 | | | 386.6 | 10.3656312 | | | | | 386.60 | 10.7873172 | | | 386.6 | 10.7873172 |
| 386.7 | 5.17791226 | 386.7 | 17.4852605 | | | 386.7 | 10.3558245 | | | | | 386.70 | 10.7873172 | | | 386.7 | 10.7873172 |
| 386.8 | 5.18771891 | 386.8 | 17.4852605 | | | 386.8 | 10.3754378 | | | | | 386.80 | 10.7578972 | | | 386.8 | 10.7578972 |
| 386.9 | 5.1681056 | 386.9 | 17.4950672 | | | 386.9 | 10.3656312 | | | | | 386.90 | 10.7775105 | | | 386.9 | 10.7775105 |
| 387 | 5.17791226 | 387 | 17.4950672 | | | 387 | 10.3754378 | | | | | 387.00 | 10.7677039 | | | 387 | 10.7677039 |
| 387.1 | 5.17791226 | 387.1 | 17.4754539 | | | 387.1 | 10.3950511 | | | | | 387.10 | 10.7775105 | | | 387.1 | 10.7775105 |
| 387.2 | 5.19752556 | 387.2 | 17.4852605 | | | 387.2 | 10.3950511 | | | | | 387.20 | 10.7578972 | | | 387.2 | 10.7578972 |
| 387.3 | 5.21713886 | 387.3 | 17.4558406 | | | 387.3 | 10.3852445 | | | | | 387.30 | 10.7480906 | | | 387.3 | 10.7480906 |
| 387.4 | 5.21713886 | 387.4 | 17.4362273 | | | 387.4 | 10.3754378 | | | | | 387.40 | 10.7677039 | | | 387.4 | 10.7677039 |
| 387.5 | 5.22694552 | 387.5 | 17.4656472 | | | 387.5 | 10.3852445 | | | | | 387.50 | 10.7775105 | | | 387.5 | 10.7775105 |
| 387.6 | 5.20733221 | 387.6 | 17.4852605 | | | 387.6 | 10.4048578 | | | | | 387.60 | 10.7873172 | | | 387.6 | 10.7873172 |

| | | | | | | | | | | | | | | | | | |
|---|---|---|---|---|---|---|---|---|---|---|---|---|---|---|---|---|---|
| 387.7 | 5.20733221 | 387.7 | 17.4754539 | | | 387.7 | 10.4048578 | | | | | 387.70 | 10.7873172 | | | 387.7 | 10.7873172 |
| 387.8 | 5.22694552 | 387.8 | 17.4656472 | | | 387.8 | 10.4048578 | | | | | 387.80 | 10.7873172 | | | 387.8 | 10.7873172 |
| 387.9 | 5.21713886 | 387.9 | 17.4656472 | | | 387.9 | 10.4048578 | | | | | 387.90 | 10.7873172 | | | 387.9 | 10.7873172 |
| 388 | 5.20733221 | 388 | 17.4852605 | | | 388 | 10.4048578 | | | | | 388.00 | 10.8167372 | | | 388 | 10.8167372 |
| 388.1 | 5.24655882 | 388.1 | 17.4558406 | | | 388.1 | 10.3950511 | | | | | 388.10 | 10.7677039 | | | 388.1 | 10.7677039 |
| 388.2 | 5.21713886 | 388.2 | 17.4754539 | | | 388.2 | 10.4244711 | | | | | 388.20 | 10.7578972 | | | 388.2 | 10.7578972 |
| 388.3 | 5.19752556 | 388.3 | 17.4754539 | | | 388.3 | 10.4342777 | | | | | 388.30 | 10.7775105 | | | 388.3 | 10.7775105 |
| 388.4 | 5.19752556 | 388.4 | 17.4558406 | | | 388.4 | 10.4048578 | | | | | 388.40 | 10.7971239 | | | 388.4 | 10.7971239 |
| 388.5 | 5.21713886 | 388.5 | 17.4558406 | | | 388.5 | 10.3950511 | | | | | 388.50 | 10.7775105 | | | 388.5 | 10.7775105 |
| 388.6 | 5.23675217 | 388.6 | 17.4558406 | | | 388.6 | 10.3950511 | | | | | 388.60 | 10.7873172 | | | 388.6 | 10.7873172 |
| 388.7 | 5.23675217 | 388.7 | 17.4362273 | | | 388.7 | 10.4440844 | | | | | 388.70 | 10.8265438 | | | 388.7 | 10.8265438 |
| 388.8 | 5.25636547 | 388.8 | 17.4460339 | | | 388.8 | 10.4440844 | | | | | 388.80 | 10.8559638 | | | 388.8 | 10.8559638 |
| 388.9 | 5.24655882 | 388.9 | 17.4362273 | | | 388.9 | 10.4440844 | | | | | 388.90 | 10.8265438 | | | 388.9 | 10.8265438 |
| 389 | 5.23675217 | 389 | 17.4460339 | | | 389 | 10.4440844 | | | | | 389.00 | 10.8069305 | | | 389 | 10.8069305 |
| 389.1 | 5.23675217 | 389.1 | 17.4460339 | | | 389.1 | 10.4342777 | | | | | 389.10 | 10.8167372 | | | 389.1 | 10.8167372 |
| 389.2 | 5.21713886 | 389.2 | 17.4656472 | | | 389.2 | 10.4636977 | | | | | 389.20 | 10.8265438 | | | 389.2 | 10.8265438 |
| 389.3 | 5.22694552 | 389.3 | 17.4656472 | | | 389.3 | 10.453891 | | | | | 389.30 | 10.8069305 | | | 389.3 | 10.8069305 |
| 389.4 | 5.23675217 | 389.4 | 17.4558406 | | | 389.4 | 10.453891 | | | | | 389.40 | 10.8069305 | | | 389.4 | 10.8069305 |
| 389.5 | 5.23675217 | 389.5 | 17.4656472 | | | 389.5 | 10.4735043 | | | | | 389.50 | 10.8069305 | | | 389.5 | 10.8069305 |
| 389.6 | 5.22694552 | 389.6 | 17.4950672 | | | 389.6 | 10.453891 | | | | | 389.60 | 10.8069305 | | | 389.6 | 10.8069305 |
| 389.7 | 5.26617212 | 389.7 | 17.5048738 | | | 389.7 | 10.4636977 | | | | | 389.70 | 10.8167372 | | | 389.7 | 10.8167372 |
| 389.8 | 5.26617212 | 389.8 | 17.4852605 | | | 389.8 | 10.4735043 | | | | | 389.80 | 10.8657704 | | | 389.8 | 10.8657704 |
| 389.9 | 5.25636547 | 389.9 | 17.4460339 | | | 389.9 | 10.4735043 | | | | | 389.90 | 10.8853837 | | | 389.9 | 10.8853837 |
| 390 | 5.25636547 | 390 | 17.4460339 | | | 390 | 10.4735043 | | | | | 390.00 | 10.8363505 | | | 390 | 10.8363505 |
| 390.1 | 5.23675217 | 390.1 | 17.4362273 | | | 390.1 | 10.4735043 | | | | | 390.10 | 10.8363505 | | | 390.1 | 10.8363505 |
| 390.2 | 5.22694552 | 390.2 | 17.416614 | | | 390.2 | 10.4636977 | | | | | 390.20 | 10.8559638 | | | 390.2 | 10.8559638 |
| 390.3 | 5.23675217 | 390.3 | 17.416614 | | | 390.3 | 10.4636977 | | | | | 390.30 | 10.8657704 | | | 390.3 | 10.8657704 |
| 390.4 | 5.26617212 | 390.4 | 17.4068073 | | | 390.4 | 10.483311 | | | | | 390.40 | 10.904997 | | | 390.4 | 10.904997 |
| 390.5 | 5.26617212 | 390.5 | 17.3970006 | | | 390.5 | 10.483311 | | | | | 390.50 | 10.8951904 | | | 390.5 | 10.8951904 |
| 390.6 | 5.24655882 | 390.6 | 17.4362273 | | | 390.6 | 10.4931176 | | | | | 390.60 | 10.8853837 | | | 390.6 | 10.8853837 |
| 390.7 | 5.24655882 | 390.7 | 17.4558406 | | | 390.7 | 10.5225376 | | | | | 390.70 | 10.904997 | | | 390.7 | 10.904997 |
| 390.8 | 5.23675217 | 390.8 | 17.4852605 | | | 390.8 | 10.4931176 | | | | | 390.80 | 10.9246103 | | | 390.8 | 10.9246103 |
| 390.9 | 5.22694552 | 390.9 | 17.4950672 | | | 390.9 | 10.4636977 | | | | | 390.90 | 10.8951904 | | | 390.9 | 10.8951904 |
| 391 | 5.22694552 | 391 | 17.4950672 | | | 391 | 10.5029243 | | | | | 391.00 | 10.904997 | | | 391 | 10.904997 |
| 391.1 | 5.23675217 | 391.1 | 17.4852605 | | | 391.1 | 10.4931176 | | | | | 391.10 | 10.934417 | | | 391.1 | 10.934417 |
| 391.2 | 5.25636547 | 391.2 | 17.4852605 | | | 391.2 | 10.4636977 | | | | | 391.20 | 10.904997 | | | 391.2 | 10.904997 |
| 391.3 | 5.23675217 | 391.3 | 17.4852605 | | | 391.3 | 10.4636977 | | | | | 391.30 | 10.8755771 | | | 391.3 | 10.8755771 |
| 391.4 | 5.25636547 | 391.4 | 17.4558406 | | | 391.4 | 10.453891 | | | | | 391.40 | 10.904997 | | | 391.4 | 10.904997 |
| 391.5 | 5.27597878 | 391.5 | 17.4558406 | | | 391.5 | 10.4440844 | | | | | 391.50 | 10.9148037 | | | 391.5 | 10.9148037 |
| 391.6 | 5.28578543 | 391.6 | 17.4754539 | | | 391.6 | 10.4636977 | | | | | 391.60 | 10.8951904 | | | 391.6 | 10.8951904 |
| 391.7 | 5.26617212 | 391.7 | 17.4558406 | | | 391.7 | 10.4342777 | | | | | 391.70 | 10.9246103 | | | 391.7 | 10.9246103 |
| 391.8 | 5.27597878 | 391.8 | 17.4656472 | | | 391.8 | 10.4244711 | | | | | 391.80 | 10.9540303 | | | 391.8 | 10.9540303 |
| 391.9 | 5.29559208 | 391.9 | 17.4460339 | | | 391.9 | 10.4342777 | | | | | 391.90 | 10.9442236 | | | 391.9 | 10.9442236 |
| 392 | 5.27597878 | 392 | 17.4264206 | | | 392 | 10.4440844 | | | | | 392.00 | 10.9246103 | | | 392 | 10.9246103 |
| 392.1 | 5.28578543 | 392.1 | 17.4460339 | | | 392.1 | 10.453891 | | | | | 392.10 | 10.9442236 | | | 392.1 | 10.9442236 |
| 392.2 | 5.29559208 | 392.2 | 17.4656472 | | | 392.2 | 10.4440844 | | | | | 392.20 | 10.9540303 | | | 392.2 | 10.9540303 |
| 392.3 | 5.31520538 | 392.3 | 17.4558406 | | | 392.3 | 10.4342777 | | | | | 392.30 | 10.9246103 | | | 392.3 | 10.9246103 |
| 392.4 | 5.28578543 | 392.4 | 17.4656472 | | | 392.4 | 10.4244711 | | | | | 392.40 | 10.8951904 | | | 392.4 | 10.8951904 |
| 392.5 | 5.29559208 | 392.5 | 17.4754539 | | | 392.5 | 10.453891 | | | | | 392.50 | 10.9246103 | | | 392.5 | 10.9246103 |
| 392.6 | 5.30539873 | 392.6 | 17.4852605 | | | 392.6 | 10.4636977 | | | | | 392.60 | 10.934417 | | | 392.6 | 10.934417 |
| 392.7 | 5.28578543 | 392.7 | 17.4852605 | | | 392.7 | 10.453891 | | | | | 392.70 | 10.9442236 | | | 392.7 | 10.9442236 |
| 392.8 | 5.28578543 | 392.8 | 17.4852605 | | | 392.8 | 10.453891 | | | | | 392.80 | 10.9442236 | | | 392.8 | 10.9442236 |
| 392.9 | 5.31520538 | 392.9 | 17.4558406 | | | 392.9 | 10.4440844 | | | | | 392.90 | 10.9638369 | | | 392.9 | 10.9638369 |
| 393 | 5.31520538 | 393 | 17.4852605 | | | 393 | 10.4244711 | | | | | 393.00 | 10.9834502 | | | 393 | 10.9834502 |
| 393.1 | 5.29559208 | 393.1 | 17.4558406 | | | 393.1 | 10.4440844 | | | | | 393.10 | 10.9736436 | | | 393.1 | 10.9736436 |
| 393.2 | 5.29559208 | 393.2 | 17.4460339 | | | 393.2 | 10.4342777 | | | | | 393.20 | 10.9638369 | | | 393.2 | 10.9638369 |
| 393.3 | 5.29559208 | 393.3 | 17.4950672 | | | 393.3 | 10.453891 | | | | | 393.30 | 10.9736436 | | | 393.3 | 10.9736436 |
| 393.4 | 5.29559208 | 393.4 | 17.4852605 | | | 393.4 | 10.453891 | | | | | 393.40 | 11.0030635 | | | 393.4 | 11.0030635 |
| 393.5 | 5.30539873 | 393.5 | 17.4950672 | | | 393.5 | 10.4342777 | | | | | 393.50 | 10.9932569 | | | 393.5 | 10.9932569 |
| 393.6 | 5.30539873 | 393.6 | 17.4950672 | | | 393.6 | 10.4440844 | | | | | 393.60 | 10.9736436 | | | 393.6 | 10.9736436 |
| 393.7 | 5.32501204 | 393.7 | 17.4754539 | | | 393.7 | 10.4735043 | | | | | 393.70 | 11.0128702 | | | 393.7 | 11.0128702 |

| | | | | | | | | | | | | | | | | | |
|---|---|---|---|---|---|---|---|---|---|---|---|---|---|---|---|---|---|
| 393.8 | 5.31520538 | 393.8 | 17.4362273 | | | 393.8 | 10.4735043 | | | | | 393.80 | 11.0226768 | | | 393.8 | 11.0226768 |
| 393.9 | 5.31520538 | 393.9 | 17.4362273 | | | 393.9 | 10.4735043 | | | | | 393.90 | 10.9932569 | | | 393.9 | 10.9932569 |
| 394 | 5.33481869 | 394 | 17.4656472 | | | 394 | 10.4735043 | | | | | 394.00 | 11.0226768 | | | 394 | 11.0226768 |
| 394.1 | 5.35443199 | 394.1 | 17.4558406 | | | 394.1 | 10.4931176 | | | | | 394.10 | 11.0226768 | | | 394.1 | 11.0226768 |
| 394.2 | 5.31520538 | 394.2 | 17.4558406 | | | 394.2 | 10.4735043 | | | | | 394.20 | 11.0226768 | | | 394.2 | 11.0226768 |
| 394.3 | 5.29559208 | 394.3 | 17.4362273 | | | 394.3 | 10.4735043 | | | | | 394.30 | 11.0030635 | | | 394.3 | 11.0030635 |
| 394.4 | 5.31520538 | 394.4 | 17.4656472 | | | 394.4 | 10.453891 | | | | | 394.40 | 11.0422902 | | | 394.4 | 11.0422902 |
| 394.5 | 5.31520538 | 394.5 | 17.4558406 | | | 394.5 | 10.4440844 | | | | | 394.50 | 11.0422902 | | | 394.5 | 11.0422902 |
| 394.6 | 5.34462534 | 394.6 | 17.4264206 | | | 394.6 | 10.4342777 | | | | | 394.60 | 11.0128702 | | | 394.6 | 11.0128702 |
| 394.7 | 5.33481869 | 394.7 | 17.4754539 | | | 394.7 | 10.4342777 | | | | | 394.70 | 11.0128702 | | | 394.7 | 11.0128702 |
| 394.8 | 5.30539873 | 394.8 | 17.4754539 | | | 394.8 | 10.453891 | | | | | 394.80 | 11.0226768 | | | 394.8 | 11.0226768 |
| 394.9 | 5.31520538 | 394.9 | 17.4558406 | | | 394.9 | 10.453891 | | | | | 394.90 | 11.0030635 | | | 394.9 | 11.0030635 |
| 395 | 5.32501204 | 395 | 17.4362273 | | | 395 | 10.4931176 | | | | | 395.00 | 11.0226768 | | | 395 | 11.0226768 |
| 395.1 | 5.33481869 | 395.1 | 17.4068073 | | | 395.1 | 10.4735043 | | | | | 395.10 | 11.0128702 | | | 395.1 | 11.0128702 |
| 395.2 | 5.31520538 | 395.2 | 17.4264206 | | | 395.2 | 10.4636977 | | | | | 395.20 | 11.0324835 | | | 395.2 | 11.0324835 |
| 395.3 | 5.31520538 | 395.3 | 17.416614 | | | 395.3 | 10.453891 | | | | | 395.30 | 11.0520968 | | | 395.3 | 11.0520968 |
| 395.4 | 5.31520538 | 395.4 | 17.3970006 | | | 395.4 | 10.453891 | | | | | 395.40 | 11.0422902 | | | 395.4 | 11.0422902 |
| 395.5 | 5.33481869 | 395.5 | 17.387194 | | | 395.5 | 10.4244711 | | | | | 395.50 | 11.0422902 | | | 395.5 | 11.0422902 |
| 395.6 | 5.36423864 | 395.6 | 17.3970006 | | | 395.6 | 10.4440844 | | | | | 395.60 | 11.0619035 | | | 395.6 | 11.0619035 |
| 395.7 | 5.35443199 | 395.7 | 17.3675807 | | | 395.7 | 10.4636977 | | | | | 395.70 | 11.0717101 | | | 395.7 | 11.0717101 |
| 395.8 | 5.36423864 | 395.8 | 17.357774 | | | 395.8 | 10.4735043 | | | | | 395.80 | 11.0619035 | | | 395.8 | 11.0619035 |
| 395.9 | 5.35443199 | 395.9 | 17.4068073 | | | 395.9 | 10.4636977 | | | | | 395.90 | 11.0520968 | | | 395.9 | 11.0520968 |
| 396 | 5.33481869 | 396 | 17.4068073 | | | 396 | 10.4735043 | | | | | 396.00 | 11.0619035 | | | 396 | 11.0619035 |
| 396.1 | 5.38385195 | 396.1 | 17.387194 | | | 396.1 | 10.4735043 | | | | | 396.10 | 11.1011301 | | | 396.1 | 11.1011301 |
| 396.2 | 5.35443199 | 396.2 | 17.387194 | | | 396.2 | 10.4735043 | | | | | 396.20 | 11.0913234 | | | 396.2 | 11.0913234 |
| 396.3 | 5.35443199 | 396.3 | 17.416614 | | | 396.3 | 10.453891 | | | | | 396.30 | 11.1403567 | | | 396.3 | 11.1403567 |
| 396.4 | 5.35443199 | 396.4 | 17.387194 | | | 396.4 | 10.4931176 | | | | | 396.40 | 11.1501633 | | | 396.4 | 11.1501633 |
| 396.5 | 5.35443199 | 396.5 | 17.387194 | | | 396.5 | 10.483311 | | | | | 396.50 | 11.1403567 | | | 396.5 | 11.1403567 |
| 396.6 | 5.36423864 | 396.6 | 17.4264206 | | | 396.6 | 10.483311 | | | | | 396.60 | 11.1697766 | | | 396.6 | 11.1697766 |
| 396.7 | 5.36423864 | 396.7 | 17.416614 | | | 396.7 | 10.4735043 | | | | | 396.70 | 11.15997 | | | 396.7 | 11.15997 |
| 396.8 | 5.35443199 | 396.8 | 17.4264206 | | | 396.8 | 10.4735043 | | | | | 396.80 | 11.1991966 | | | 396.8 | 11.1991966 |
| 396.9 | 5.35443199 | 396.9 | 17.4264206 | | | 396.9 | 10.4636977 | | | | | 396.90 | 11.1893899 | | | 396.9 | 11.1893899 |
| 397 | 5.33481869 | 397 | 17.3970006 | | | 397 | 10.4735043 | | | | | 397.00 | 11.1795833 | | | 397 | 11.1795833 |
| 397.1 | 5.34462534 | 397.1 | 17.416614 | | | 397.1 | 10.453891 | | | | | 397.10 | 11.15997 | | | 397.1 | 11.15997 |
| 397.2 | 5.35443199 | 397.2 | 17.4460339 | | | 397.2 | 10.4440844 | | | | | 397.20 | 11.1501633 | | | 397.2 | 11.1501633 |
| 397.3 | 5.32501204 | 397.3 | 17.4068073 | | | 397.3 | 10.4636977 | | | | | 397.30 | 11.15997 | | | 397.3 | 11.15997 |
| 397.4 | 5.35443199 | 397.4 | 17.4068073 | | | 397.4 | 10.483311 | | | | | 397.40 | 11.1991966 | | | 397.4 | 11.1991966 |
| 397.5 | 5.34462534 | 397.5 | 17.3675807 | | | 397.5 | 10.4735043 | | | | | 397.50 | 11.2286165 | | | 397.5 | 11.2286165 |
| 397.6 | 5.35443199 | 397.6 | 17.416614 | | | 397.6 | 10.4636977 | | | | | 397.60 | 11.2286165 | | | 397.6 | 11.2286165 |
| 397.7 | 5.36423864 | 397.7 | 17.4362273 | | | 397.7 | 10.483311 | | | | | 397.70 | 11.2286165 | | | 397.7 | 11.2286165 |
| 397.8 | 5.35443199 | 397.8 | 17.4068073 | | | 397.8 | 10.483311 | | | | | 397.80 | 11.2384232 | | | 397.8 | 11.2384232 |
| 397.9 | 5.3740453 | 397.9 | 17.3970006 | | | 397.9 | 10.5029243 | | | | | 397.90 | 11.2286165 | | | 397.9 | 11.2286165 |
| 398 | 5.38385195 | 398 | 17.4362273 | | | 398 | 10.4931176 | | | | | 398.00 | 11.2384232 | | | 398 | 11.2384232 |
| 398.1 | 5.3740453 | 398.1 | 17.4558406 | | | 398.1 | 10.483311 | | | | | 398.10 | 11.2384232 | | | 398.1 | 11.2384232 |
| 398.2 | 5.35443199 | 398.2 | 17.416614 | | | 398.2 | 10.4735043 | | | | | 398.20 | 11.2482298 | | | 398.2 | 11.2482298 |
| 398.3 | 5.35443199 | 398.3 | 17.4068073 | | | 398.3 | 10.4636977 | | | | | 398.30 | 11.2384232 | | | 398.3 | 11.2384232 |
| 398.4 | 5.3740453 | 398.4 | 17.4068073 | | | 398.4 | 10.4636977 | | | | | 398.40 | 11.2384232 | | | 398.4 | 11.2384232 |
| 398.5 | 5.34462534 | 398.5 | 17.4264206 | | | 398.5 | 10.453891 | | | | | 398.50 | 11.2580365 | | | 398.5 | 11.2580365 |
| 398.6 | 5.3740453 | 398.6 | 17.4460339 | | | 398.6 | 10.4342777 | | | | | 398.60 | 11.2384232 | | | 398.6 | 11.2384232 |
| 398.7 | 5.36423864 | 398.7 | 17.4068073 | | | 398.7 | 10.4342777 | | | | | 398.70 | 11.2678431 | | | 398.7 | 11.2678431 |
| 398.8 | 5.33481869 | 398.8 | 17.4068073 | | | 398.8 | 10.453891 | | | | | 398.80 | 11.2678431 | | | 398.8 | 11.2678431 |
| 398.9 | 5.32501204 | 398.9 | 17.4460339 | | | 398.9 | 10.4636977 | | | | | 398.90 | 11.2972631 | | | 398.9 | 11.2972631 |
| 399 | 5.35443199 | 399 | 17.4558406 | | | 399 | 10.4440844 | | | | | 399.00 | 11.2874565 | | | 399 | 11.2874565 |
| 399.1 | 5.3740453 | 399.1 | 17.4460339 | | | 399.1 | 10.4440844 | | | | | 399.10 | 11.2776498 | | | 399.1 | 11.2776498 |
| 399.2 | 5.38385195 | 399.2 | 17.4264206 | | | 399.2 | 10.453891 | | | | | 399.20 | 11.2972631 | | | 399.2 | 11.2972631 |
| 399.3 | 5.40346525 | 399.3 | 17.4460339 | | | 399.3 | 10.4735043 | | | | | 399.30 | 11.2874565 | | | 399.3 | 11.2874565 |
| 399.4 | 5.40346525 | 399.4 | 17.4754539 | | | 399.4 | 10.4931176 | | | | | 399.40 | 11.2776498 | | | 399.4 | 11.2776498 |
| 399.5 | 5.3936586 | 399.5 | 17.4460339 | | | 399.5 | 10.5127309 | | | | | 399.50 | 11.2874565 | | | 399.5 | 11.2874565 |
| 399.6 | 5.40346525 | 399.6 | 17.4362273 | | | 399.6 | 10.5127309 | | | | | 399.60 | 11.2776498 | | | 399.6 | 11.2776498 |
| 399.7 | 5.36423864 | 399.7 | 17.4558406 | | | 399.7 | 10.5323442 | | | | | 399.70 | 11.2972631 | | | 399.7 | 11.2972631 |
| 399.8 | 5.40346525 | 399.8 | 17.4460339 | | | 399.8 | 10.5323442 | | | | | 399.80 | 11.2776498 | | | 399.8 | 11.2776498 |

| | | | | | | | | | | | | | | | | |
|---|---|---|---|---|---|---|---|---|---|---|---|---|---|---|---|---|
| 399.9 | 5.40346525 | 399.9 | 17.4656472 | | | 399.9 | 10.5519576 | | | | 399.90 | 11.2776498 | | | 399.9 | 11.2776498 |
| 400 | 5.40346525 | 400 | 17.4558406 | | | 400 | 10.5715709 | | | | 400.00 | 11.3070698 | | | 400 | 11.3070698 |
| 400.1 | 5.3936586 | 400.1 | 17.416614 | | | 400.1 | 10.5421509 | | | | 400.10 | 11.2874565 | | | 400.1 | 11.2874565 |
| 400.2 | 5.38385195 | 400.2 | 17.4068073 | | | 400.2 | 10.5323442 | | | | 400.20 | 11.2972631 | | | 400.2 | 11.2972631 |
| 400.3 | 5.4132719 | 400.3 | 17.3970006 | | | 400.3 | 10.5127309 | | | | 400.30 | 11.3462964 | | | 400.3 | 11.3462964 |
| 400.4 | 5.4132719 | 400.4 | 17.3970006 | | | 400.4 | 10.5225376 | | | | 400.40 | 11.356103 | | | 400.4 | 11.356103 |
| 400.5 | 5.3936586 | 400.5 | 17.387194 | | | 400.5 | 10.5421509 | | | | 400.50 | 11.3168764 | | | 400.5 | 11.3168764 |
| 400.6 | 5.40346525 | 400.6 | 17.3970006 | | | 400.6 | 10.5323442 | | | | 400.60 | 11.3168764 | | | 400.6 | 11.3168764 |
| 400.7 | 5.42307856 | 400.7 | 17.3970006 | | | 400.7 | 10.5029243 | | | | 400.70 | 11.3364897 | | | 400.7 | 11.3364897 |
| 400.8 | 5.40346525 | 400.8 | 17.3479674 | | | 400.8 | 10.5323442 | | | | 400.80 | 11.3168764 | | | 400.8 | 11.3168764 |
| 400.9 | 5.40346525 | 400.9 | 17.2989341 | | | 400.9 | 10.5225376 | | | | 400.90 | 11.3266831 | | | 400.9 | 11.3266831 |
| 401 | 5.3936586 | 401 | 17.2793208 | | | 401 | 10.5029243 | | | | 401.00 | 11.3266831 | | | 401 | 11.3266831 |
| 401.1 | 5.4132719 | 401.1 | 17.2891275 | | | 401.1 | 10.5127309 | | | | 401.10 | 11.3168764 | | | 401.1 | 11.3168764 |
| 401.2 | 5.42307856 | 401.2 | 17.2891275 | | | 401.2 | 10.5127309 | | | | 401.20 | 11.3168764 | | | 401.2 | 11.3168764 |
| 401.3 | 5.40346525 | 401.3 | 17.3185474 | | | 401.3 | 10.5225376 | | | | 401.30 | 11.3364897 | | | 401.3 | 11.3364897 |
| 401.4 | 5.40346525 | 401.4 | 17.3381607 | | | 401.4 | 10.5421509 | | | | 401.40 | 11.3364897 | | | 401.4 | 11.3364897 |
| 401.5 | 5.3936586 | 401.5 | 17.3185474 | | | 401.5 | 10.5225376 | | | | 401.50 | 11.3364897 | | | 401.5 | 11.3364897 |
| 401.6 | 5.4132719 | 401.6 | 17.3381607 | | | 401.6 | 10.5421509 | | | | 401.60 | 11.3462964 | | | 401.6 | 11.3462964 |
| 401.7 | 5.42307856 | 401.7 | 17.3283541 | | | 401.7 | 10.5421509 | | | | 401.70 | 11.3364897 | | | 401.7 | 11.3364897 |
| 401.8 | 5.4132719 | 401.8 | 17.3087408 | | | 401.8 | 10.5519576 | | | | 401.80 | 11.3364897 | | | 401.8 | 11.3364897 |
| 401.9 | 5.40346525 | 401.9 | 17.3283541 | | | 401.9 | 10.5715709 | | | | 401.90 | 11.3462964 | | | 401.9 | 11.3462964 |
| 402 | 5.4132719 | 402 | 17.3381607 | | | 402 | 10.5715709 | | | | 402.00 | 11.3462964 | | | 402 | 11.3462964 |
| 402.1 | 5.3936586 | 402.1 | 17.3283541 | | | 402.1 | 10.5715709 | | | | 402.10 | 11.3462964 | | | 402.1 | 11.3462964 |
| 402.2 | 5.42307856 | 402.2 | 17.3479674 | | | 402.2 | 10.5813775 | | | | 402.20 | 11.3659097 | | | 402.2 | 11.3659097 |
| 402.3 | 5.44269186 | 402.3 | 17.3479674 | | | 402.3 | 10.5715709 | | | | 402.30 | 11.385523 | | | 402.3 | 11.385523 |
| 402.4 | 5.43288521 | 402.4 | 17.3381607 | | | 402.4 | 10.5519576 | | | | 402.40 | 11.356103 | | | 402.4 | 11.356103 |
| 402.5 | 5.44269186 | 402.5 | 17.3479674 | | | 402.5 | 10.5617642 | | | | 402.50 | 11.356103 | | | 402.5 | 11.356103 |
| 402.6 | 5.42307856 | 402.6 | 17.387194 | | | 402.6 | 10.5911842 | | | | 402.60 | 11.3462964 | | | 402.6 | 11.3462964 |
| 402.7 | 5.43288521 | 402.7 | 17.3675807 | | | 402.7 | 10.6009908 | | | | 402.70 | 11.356103 | | | 402.7 | 11.356103 |
| 402.8 | 5.42307856 | 402.8 | 17.3479674 | | | 402.8 | 10.5813775 | | | | 402.80 | 11.3462964 | | | 402.8 | 11.3462964 |
| 402.9 | 5.44269186 | 402.9 | 17.3479674 | | | 402.9 | 10.5911842 | | | | 402.90 | 11.356103 | | | 402.9 | 11.356103 |
| 403 | 5.45249851 | 403 | 17.3185474 | | | 403 | 10.6107975 | | | | 403.00 | 11.385523 | | | 403 | 11.385523 |
| 403.1 | 5.45249851 | 403.1 | 17.2989341 | | | 403.1 | 10.6107975 | | | | 403.10 | 11.3953296 | | | 403.1 | 11.3953296 |
| 403.2 | 5.45249851 | 403.2 | 17.2891275 | | | 403.2 | 10.6206041 | | | | 403.20 | 11.3953296 | | | 403.2 | 11.3953296 |
| 403.3 | 5.42307856 | 403.3 | 17.3185474 | | | 403.3 | 10.6107975 | | | | 403.30 | 11.385523 | | | 403.3 | 11.385523 |
| 403.4 | 5.43288521 | 403.4 | 17.3283541 | | | 403.4 | 10.6206041 | | | | 403.40 | 11.3659097 | | | 403.4 | 11.3659097 |
| 403.5 | 5.42307856 | 403.5 | 17.3185474 | | | 403.5 | 10.6107975 | | | | 403.50 | 11.385523 | | | 403.5 | 11.385523 |
| 403.6 | 5.44269186 | 403.6 | 17.3479674 | | | 403.6 | 10.6107975 | | | | 403.60 | 11.385523 | | | 403.6 | 11.385523 |
| 403.7 | 5.44269186 | 403.7 | 17.3773873 | | | 403.7 | 10.6009908 | | | | 403.70 | 11.3953296 | | | 403.7 | 11.3953296 |
| 403.8 | 5.43288521 | 403.8 | 17.357774 | | | 403.8 | 10.6009908 | | | | 403.80 | 11.4051363 | | | 403.8 | 11.4051363 |
| 403.9 | 5.42307856 | 403.9 | 17.3479674 | | | 403.9 | 10.6009908 | | | | 403.90 | 11.4051363 | | | 403.9 | 11.4051363 |
| 404 | 5.42307856 | 404 | 17.3675807 | | | 404 | 10.5911842 | | | | 404.00 | 11.385523 | | | 404 | 11.385523 |
| 404.1 | 5.43288521 | 404.1 | 17.3773873 | | | 404.1 | 10.5911842 | | | | 404.10 | 11.4051363 | | | 404.1 | 11.4051363 |
| 404.2 | 5.46230516 | 404.2 | 17.3675807 | | | 404.2 | 10.5911842 | | | | 404.20 | 11.4051363 | | | 404.2 | 11.4051363 |
| 404.3 | 5.46230516 | 404.3 | 17.3479674 | | | 404.3 | 10.6009908 | | | | 404.30 | 11.3953296 | | | 404.3 | 11.3953296 |
| 404.4 | 5.43288521 | 404.4 | 17.3381607 | | | 404.4 | 10.6402174 | | | | 404.40 | 11.4149429 | | | 404.4 | 11.4149429 |
| 404.5 | 5.4132719 | 404.5 | 17.3185474 | | | 404.5 | 10.6402174 | | | | 404.50 | 11.4149429 | | | 404.5 | 11.4149429 |
| 404.6 | 5.43288521 | 404.6 | 17.3283541 | | | 404.6 | 10.6206041 | | | | 404.60 | 11.4345562 | | | 404.6 | 11.4345562 |
| 404.7 | 5.44269186 | 404.7 | 17.3283541 | | | 404.7 | 10.6206041 | | | | 404.70 | 11.4443629 | | | 404.7 | 11.4443629 |
| 404.8 | 5.47211182 | 404.8 | 17.3381607 | | | 404.8 | 10.6009908 | | | | 404.80 | 11.4345562 | | | 404.8 | 11.4345562 |
| 404.9 | 5.45249851 | 404.9 | 17.3479674 | | | 404.9 | 10.5715709 | | | | 404.90 | 11.4345562 | | | 404.9 | 11.4345562 |
| 405 | 5.46230516 | 405 | 17.3773873 | | | 405 | 10.5127309 | | | | 405.00 | 11.4345562 | | | 405 | 11.4345562 |
| 405.1 | 5.46230516 | 405.1 | 17.3479674 | | | 405.1 | 10.4735043 | | | | 405.10 | 11.4247496 | | | 405.1 | 11.4247496 |
| 405.2 | 5.44269186 | 405.2 | 17.3185474 | | | 405.2 | 10.483311 | | | | 405.20 | 11.4345562 | | | 405.2 | 11.4345562 |
| 405.3 | 5.47211182 | 405.3 | 17.3381607 | | | 405.3 | 10.483311 | | | | 405.30 | 11.4639762 | | | 405.3 | 11.4639762 |
| 405.4 | 5.48191847 | 405.4 | 17.3185474 | | | 405.4 | 10.4636977 | | | | 405.40 | 11.4639762 | | | 405.4 | 11.4639762 |
| 405.5 | 5.49172512 | 405.5 | 17.3381607 | | | 405.5 | 10.5029243 | | | | 405.50 | 11.4541695 | | | 405.5 | 11.4541695 |
| 405.6 | 5.46230516 | 405.6 | 17.357774 | | | 405.6 | 10.483311 | | | | 405.60 | 11.4835895 | | | 405.6 | 11.4835895 |
| 405.7 | 5.47211182 | 405.7 | 17.3675807 | | | 405.7 | 10.4931176 | | | | 405.70 | 11.4835895 | | | 405.7 | 11.4835895 |
| 405.8 | 5.48191847 | 405.8 | 17.3381607 | | | 405.8 | 10.5323442 | | | | 405.80 | 11.5032028 | | | 405.8 | 11.5032028 |
| 405.9 | 5.48191847 | 405.9 | 17.3675807 | | | 405.9 | 10.5225376 | | | | 405.90 | 11.4933961 | | | 405.9 | 11.4933961 |

| | | | | | | | | | | | | | | | | | |
|---|---|---|---|---|---|---|---|---|---|---|---|---|---|---|---|---|---|
| 406 | 5.48191847 | 406 | 17.3773873 | | | 406 | 10.5225376 | | | | | 406.00 | 11.4835895 | | | 406 | 11.4835895 |
| 406.1 | 5.52114508 | 406.1 | 17.3675807 | | | 406.1 | 10.5029243 | | | | | 406.10 | 11.5130094 | | | 406.1 | 11.5130094 |
| 406.2 | 5.51133842 | 406.2 | 17.3479674 | | | 406.2 | 10.5127309 | | | | | 406.20 | 11.4933961 | | | 406.2 | 11.4933961 |
| 406.3 | 5.46230516 | 406.3 | 17.3381607 | | | 406.3 | 10.5127309 | | | | | 406.30 | 11.5032028 | | | 406.3 | 11.5032028 |
| 406.4 | 5.47211182 | 406.4 | 17.3675807 | | | 406.4 | 10.5029243 | | | | | 406.40 | 11.4933961 | | | 406.4 | 11.4933961 |
| 406.5 | 5.51133842 | 406.5 | 17.3970006 | | | 406.5 | 10.5029243 | | | | | 406.50 | 11.4933961 | | | 406.5 | 11.4933961 |
| 406.6 | 5.53095173 | 406.6 | 17.3773873 | | | 406.6 | 10.5225376 | | | | | 406.60 | 11.5032028 | | | 406.6 | 11.5032028 |
| 406.7 | 5.51133842 | 406.7 | 17.357774 | | | 406.7 | 10.5225376 | | | | | 406.70 | 11.5130094 | | | 406.7 | 11.5130094 |
| 406.8 | 5.49172512 | 406.8 | 17.3970006 | | | 406.8 | 10.5127309 | | | | | 406.80 | 11.5228161 | | | 406.8 | 11.5228161 |
| 406.9 | 5.46230516 | 406.9 | 17.387194 | | | 406.9 | 10.5323442 | | | | | 406.90 | 11.5620427 | | | 406.9 | 11.5620427 |
| 407 | 5.45249851 | 407 | 17.3970006 | | | 407 | 10.5225376 | | | | | 407.00 | 11.5522361 | | | 407 | 11.5522361 |
| 407.1 | 5.49172512 | 407.1 | 17.3970006 | | | 407.1 | 10.5323442 | | | | | 407.10 | 11.5424294 | | | 407.1 | 11.5424294 |
| 407.2 | 5.49172512 | 407.2 | 17.3773873 | | | 407.2 | 10.5323442 | | | | | 407.20 | 11.5326228 | | | 407.2 | 11.5326228 |
| 407.3 | 5.49172512 | 407.3 | 17.357774 | | | 407.3 | 10.5323442 | | | | | 407.30 | 11.5424294 | | | 407.3 | 11.5424294 |
| 407.4 | 5.46230516 | 407.4 | 17.357774 | | | 407.4 | 10.5421509 | | | | | 407.40 | 11.581656 | | | 407.4 | 11.581656 |
| 407.5 | 5.47211182 | 407.5 | 17.3675807 | | | 407.5 | 10.5225376 | | | | | 407.50 | 11.5718494 | | | 407.5 | 11.5718494 |
| 407.6 | 5.51133842 | 407.6 | 17.387194 | | | 407.6 | 10.5127309 | | | | | 407.60 | 11.5424294 | | | 407.6 | 11.5424294 |
| 407.7 | 5.50153177 | 407.7 | 17.3773873 | | | 407.7 | 10.5127309 | | | | | 407.70 | 11.5522361 | | | 407.7 | 11.5522361 |
| 407.8 | 5.50153177 | 407.8 | 17.3479674 | | | 407.8 | 10.5323442 | | | | | 407.80 | 11.581656 | | | 407.8 | 11.581656 |
| 407.9 | 5.48191847 | 407.9 | 17.357774 | | | 407.9 | 10.5617642 | | | | | 407.90 | 11.5718494 | | | 407.9 | 11.5718494 |
| 408 | 5.53095173 | 408 | 17.3087408 | | | 408 | 10.5421509 | | | | | 408.00 | 11.581656 | | | 408 | 11.581656 |
| 408.1 | 5.53095173 | 408.1 | 17.2793208 | | | 408.1 | 10.5225376 | | | | | 408.10 | 11.581656 | | | 408.1 | 11.581656 |
| 408.2 | 5.52114508 | 408.2 | 17.2891275 | | | 408.2 | 10.5519576 | | | | | 408.20 | 11.6012693 | | | 408.2 | 11.6012693 |
| 408.3 | 5.50153177 | 408.3 | 17.2989341 | | | 408.3 | 10.5519576 | | | | | 408.30 | 11.581656 | | | 408.3 | 11.581656 |
| 408.4 | 5.51133842 | 408.4 | 17.3087408 | | | 408.4 | 10.5617642 | | | | | 408.40 | 11.5914627 | | | 408.4 | 11.5914627 |
| 408.5 | 5.51133842 | 408.5 | 17.3087408 | | | 408.5 | 10.5715709 | | | | | 408.50 | 11.611076 | | | 408.5 | 11.611076 |
| 408.6 | 5.51133842 | 408.6 | 17.3087408 | | | 408.6 | 10.5715709 | | | | | 408.60 | 11.6012693 | | | 408.6 | 11.6012693 |
| 408.7 | 5.52114508 | 408.7 | 17.3283541 | | | 408.7 | 10.5813775 | | | | | 408.70 | 11.611076 | | | 408.7 | 11.611076 |
| 408.8 | 5.56037168 | 408.8 | 17.3185474 | | | 408.8 | 10.5617642 | | | | | 408.80 | 11.6404959 | | | 408.8 | 11.6404959 |
| 408.9 | 5.54075838 | 408.9 | 17.3087408 | | | 408.9 | 10.5617642 | | | | | 408.90 | 11.6306893 | | | 408.9 | 11.6306893 |
| 409 | 5.53095173 | 409 | 17.3087408 | | | 409 | 10.5617642 | | | | | 409.00 | 11.611076 | | | 409 | 11.611076 |
| 409.1 | 5.55056503 | 409.1 | 17.2989341 | | | 409.1 | 10.5715709 | | | | | 409.10 | 11.5914627 | | | 409.1 | 11.5914627 |
| 409.2 | 5.54075838 | 409.2 | 17.2989341 | | | 409.2 | 10.5715709 | | | | | 409.20 | 11.611076 | | | 409.2 | 11.611076 |
| 409.3 | 5.54075838 | 409.3 | 17.2989341 | | | 409.3 | 10.5617642 | | | | | 409.30 | 11.6404959 | | | 409.3 | 11.6404959 |
| 409.4 | 5.54075838 | 409.4 | 17.3087408 | | | 409.4 | 10.5715709 | | | | | 409.40 | 11.6601092 | | | 409.4 | 11.6601092 |
| 409.5 | 5.52114508 | 409.5 | 17.3185474 | | | 409.5 | 10.5421509 | | | | | 409.50 | 11.6404959 | | | 409.5 | 11.6404959 |
| 409.6 | 5.53095173 | 409.6 | 17.3283541 | | | 409.6 | 10.5715709 | | | | | 409.60 | 11.6012693 | | | 409.6 | 11.6012693 |
| 409.7 | 5.53095173 | 409.7 | 17.3185474 | | | 409.7 | 10.5715709 | | | | | 409.70 | 11.6208826 | | | 409.7 | 11.6208826 |
| 409.8 | 5.53095173 | 409.8 | 17.2891275 | | | 409.8 | 10.5617642 | | | | | 409.80 | 11.6699159 | | | 409.8 | 11.6699159 |
| 409.9 | 5.51133842 | 409.9 | 17.2891275 | | | 409.9 | 10.5715709 | | | | | 409.90 | 11.6601092 | | | 409.9 | 11.6601092 |
| 410 | 5.54075838 | 410 | 17.2891275 | | | 410 | 10.5715709 | | | | | 410.00 | 11.6503026 | | | 410 | 11.6503026 |
| 410.1 | 5.55056503 | 410.1 | 17.2891275 | | | 410.1 | 10.5617642 | | | | | 410.10 | 11.6601092 | | | 410.1 | 11.6601092 |
| 410.2 | 5.55056503 | 410.2 | 17.2989341 | | | 410.2 | 10.5813775 | | | | | 410.20 | 11.6404959 | | | 410.2 | 11.6404959 |
| 410.3 | 5.55056503 | 410.3 | 17.3087408 | | | 410.3 | 10.6107975 | | | | | 410.30 | 11.6503026 | | | 410.3 | 11.6503026 |
| 410.4 | 5.49172512 | 410.4 | 17.2891275 | | | 410.4 | 10.6009908 | | | | | 410.40 | 11.6699159 | | | 410.4 | 11.6699159 |
| 410.5 | 5.50153177 | 410.5 | 17.3087408 | | | 410.5 | 10.5715709 | | | | | 410.50 | 11.6404959 | | | 410.5 | 11.6404959 |
| 410.6 | 5.52114508 | 410.6 | 17.3087408 | | | 410.6 | 10.5617642 | | | | | 410.60 | 11.6503026 | | | 410.6 | 11.6503026 |
| 410.7 | 5.53095173 | 410.7 | 17.2891275 | | | 410.7 | 10.5911842 | | | | | 410.70 | 11.6699159 | | | 410.7 | 11.6699159 |
| 410.8 | 5.54075838 | 410.8 | 17.2695142 | | | 410.8 | 10.6206041 | | | | | 410.80 | 11.6306893 | | | 410.8 | 11.6306893 |
| 410.9 | 5.54075838 | 410.9 | 17.2891275 | | | 410.9 | 10.5715709 | | | | | 410.90 | 11.6503026 | | | 410.9 | 11.6503026 |
| 411 | 5.51133842 | 411 | 17.3087408 | | | 411 | 10.6107975 | | | | | 411.00 | 11.6601092 | | | 411 | 11.6601092 |
| 411.1 | 5.51133842 | 411.1 | 17.3087408 | | | 411.1 | 10.6206041 | | | | | 411.10 | 11.6601092 | | | 411.1 | 11.6601092 |
| 411.2 | 5.52114508 | 411.2 | 17.2891275 | | | 411.2 | 10.6107975 | | | | | 411.20 | 11.6699159 | | | 411.2 | 11.6699159 |
| 411.3 | 5.56037168 | 411.3 | 17.2695142 | | | 411.3 | 10.6009908 | | | | | 411.30 | 11.6895292 | | | 411.3 | 11.6895292 |
| 411.4 | 5.55056503 | 411.4 | 17.2695142 | | | 411.4 | 10.5813775 | | | | | 411.40 | 11.6895292 | | | 411.4 | 11.6895292 |
| 411.5 | 5.55056503 | 411.5 | 17.3185474 | | | 411.5 | 10.5911842 | | | | | 411.50 | 11.6895292 | | | 411.5 | 11.6895292 |
| 411.6 | 5.54075838 | 411.6 | 17.2989341 | | | 411.6 | 10.6009908 | | | | | 411.60 | 11.6993358 | | | 411.6 | 11.6993358 |
| 411.7 | 5.55056503 | 411.7 | 17.2891275 | | | 411.7 | 10.6009908 | | | | | 411.70 | 11.7091425 | | | 411.7 | 11.7091425 |
| 411.8 | 5.55056503 | 411.8 | 17.3185474 | | | 411.8 | 10.5911842 | | | | | 411.80 | 11.6895292 | | | 411.8 | 11.6895292 |
| 411.9 | 5.56037168 | 411.9 | 17.3675807 | | | 411.9 | 10.5911842 | | | | | 411.90 | 11.7091425 | | | 411.9 | 11.7091425 |
| 412 | 5.56037168 | 412 | 17.3675807 | | | 412 | 10.6009908 | | | | | 412.00 | 11.7287558 | | | 412 | 11.7287558 |

| | | | | | | | | | | | | | | | | | |
|---|---|---|---|---|---|---|---|---|---|---|---|---|---|---|---|---|---|
| 412.1 | 5.57017834 | 412.1 | 17.3381607 | | | 412.1 | 10.6107975 | | | | | 412.10 | 11.7385624 | | | 412.1 | 11.7385624 |
| 412.2 | 5.57998499 | 412.2 | 17.3381607 | | | 412.2 | 10.6009908 | | | | | 412.20 | 11.7091425 | | | 412.2 | 11.7091425 |
| 412.3 | 5.53095173 | 412.3 | 17.3479674 | | | 412.3 | 10.5911842 | | | | | 412.30 | 11.7189491 | | | 412.3 | 11.7189491 |
| 412.4 | 5.55056503 | 412.4 | 17.357774 | | | 412.4 | 10.6107975 | | | | | 412.40 | 11.7679824 | | | 412.4 | 11.7679824 |
| 412.5 | 5.57017834 | 412.5 | 17.3479674 | | | 412.5 | 10.6009908 | | | | | 412.50 | 11.7581757 | | | 412.5 | 11.7581757 |
| 412.6 | 5.57017834 | 412.6 | 17.3185474 | | | 412.6 | 10.5813775 | | | | | 412.60 | 11.7483691 | | | 412.6 | 11.7483691 |
| 412.7 | 5.57017834 | 412.7 | 17.3087408 | | | 412.7 | 10.6009908 | | | | | 412.70 | 11.7483691 | | | 412.7 | 11.7483691 |
| 412.8 | 5.59959829 | 412.8 | 17.3283541 | | | 412.8 | 10.5911842 | | | | | 412.80 | 11.7189491 | | | 412.8 | 11.7189491 |
| 412.9 | 5.58979164 | 412.9 | 17.3479674 | | | 412.9 | 10.6107975 | | | | | 412.90 | 11.7483691 | | | 412.9 | 11.7483691 |
| 413 | 5.57017834 | 413 | 17.3381607 | | | 413 | 10.5911842 | | | | | 413.00 | 11.7385624 | | | 413 | 11.7385624 |
| 413.1 | 5.57017834 | 413.1 | 17.3479674 | | | 413.1 | 10.6304108 | | | | | 413.10 | 11.7287558 | | | 413.1 | 11.7287558 |
| 413.2 | 5.57998499 | 413.2 | 17.357774 | | | 413.2 | 10.6402174 | | | | | 413.20 | 11.7385624 | | | 413.2 | 11.7385624 |
| 413.3 | 5.57017834 | 413.3 | 17.3675807 | | | 413.3 | 10.6402174 | | | | | 413.30 | 11.7483691 | | | 413.3 | 11.7483691 |
| 413.4 | 5.57017834 | 413.4 | 17.3283541 | | | 413.4 | 10.6107975 | | | | | 413.40 | 11.7385624 | | | | |
| 413.5 | 5.59959829 | 413.5 | 17.3381607 | | | 413.5 | 10.6009908 | | | | | 413.50 | 11.7287558 | | | | |
| 413.6 | 5.59959829 | 413.6 | 17.3381607 | | | 413.6 | 10.6009908 | | | | | 413.60 | 11.7287558 | | | | |
| 413.7 | 5.58979164 | 413.7 | 17.3381607 | | | 413.7 | 10.6107975 | | | | | 413.70 | 11.7581757 | | | | |
| 413.8 | 5.59959829 | 413.8 | 17.3283541 | | | 413.8 | 10.6304108 | | | | | 413.80 | 11.7581757 | | | | |
| 413.9 | 5.59959829 | 413.9 | 17.3381607 | | | 413.9 | 10.6892507 | | | | | 413.90 | 11.7483691 | | | | |
| 414 | 5.58979164 | 414 | 17.3381607 | | | 414 | 10.7480906 | | | | | 414.00 | 11.7875957 | | | | |
| 414.1 | 5.59959829 | 414.1 | 17.3479674 | | | 414.1 | 10.7873172 | | | | | 414.10 | 11.7974024 | | | | |
| 414.2 | 5.58979164 | 414.2 | 17.3283541 | | | 414.2 | 10.7971239 | | | | | 414.20 | 11.7875957 | | | | |
| 414.3 | 5.58979164 | 414.3 | 17.2891275 | | | 414.3 | 10.6500241 | | | | | 414.30 | 11.7679824 | | | | |
| 414.4 | 5.60940494 | 414.4 | 17.2400942 | | | 414.4 | 10.6009908 | | | | | 414.40 | 11.7679824 | | | | |
| 414.5 | 5.6192116 | 414.5 | 17.2302876 | | | 414.5 | 10.4636977 | | | | | 414.50 | 11.7679824 | | | | |
| 414.6 | 5.62901825 | 414.6 | 17.2302876 | | | 414.6 | 10.3067913 | | | | | 414.60 | 11.7679824 | | | | |
| 414.7 | 5.6584382 | 414.7 | 17.2400942 | | | 414.7 | 10.2087247 | | | | | 414.70 | 11.7679824 | | | | |
| 414.8 | 5.6388249 | 414.8 | 17.2597075 | | | 414.8 | 10.1694981 | | | | | 414.80 | 11.7679824 | | | | |
| 414.9 | 5.62901825 | 414.9 | 17.2793208 | | | 414.9 | 10.1302715 | | | | | 414.90 | 11.807209 | | | | |
| 415 | 5.6388249 | 415 | 17.2695142 | | | 415 | 10.0910449 | | | | | 415.00 | 11.8170157 | | | | |
| 415.1 | 5.58979164 | 415.1 | 17.2499009 | | | 415.1 | 10.0714316 | | | | | 415.10 | 11.7777891 | | | | |
| 415.2 | 5.58979164 | 415.2 | 17.2302876 | | | 415.2 | 10.032205 | | | | | 415.20 | 11.7974024 | | | | |
| 415.3 | 5.58979164 | 415.3 | 17.2400942 | | | 415.3 | 9.98317174 | | | | | 415.30 | 11.8268223 | | | | |
| 415.4 | 5.6192116 | 415.4 | 17.2499009 | | | 415.4 | 9.93413848 | | | | | 415.40 | 11.8170157 | | | | |
| 415.5 | 5.62901825 | 415.5 | 17.2499009 | | | 415.5 | 10.0223983 | | | | | 415.50 | 11.8170157 | | | | |
| 415.6 | 5.62901825 | 415.6 | 17.2989341 | | | 415.6 | 10.0125917 | | | | | 415.60 | 11.8170157 | | | | |
| 415.7 | 5.6192116 | 415.7 | 17.3087408 | | | 415.7 | 9.99297839 | | | | | 415.70 | 11.807209 | | | | |
| 415.8 | 5.6584382 | 415.8 | 17.2793208 | | | 415.8 | 9.96355843 | | | | | 415.80 | 11.8170157 | | | | |
| 415.9 | 5.64863155 | 415.9 | 17.2891275 | | | 415.9 | 9.94394513 | | | | | 415.90 | 11.8170157 | | | | |
| 416 | 5.6388249 | 416 | 17.3087408 | | | 416 | 9.95375178 | | | | | 416.00 | 11.7974024 | | | | |
| 416.1 | 5.62901825 | 416.1 | 17.2989341 | | | 416.1 | 9.95375178 | | | | | 416.10 | 11.8268223 | | | | |
| 416.2 | 5.62901825 | 416.2 | 17.2989341 | | | 416.2 | 9.96355843 | | | | | 416.20 | 11.8464356 | | | | |
| 416.3 | 5.64863155 | 416.3 | 17.3185474 | | | 416.3 | 9.90471852 | | | | | 416.30 | 11.836629 | | | | |
| 416.4 | 5.62901825 | 416.4 | 17.2891275 | | | 416.4 | 9.86549191 | | | | | 416.40 | 11.8268223 | | | | |
| 416.5 | 5.60940494 | 416.5 | 17.2793208 | | | 416.5 | 9.90471852 | | | | | 416.50 | 11.8464356 | | | | |
| 416.6 | 5.6388249 | 416.6 | 17.2793208 | | | 416.6 | 9.89491187 | | | | | 416.60 | 11.8464356 | | | | |
| 416.7 | 5.64863155 | 416.7 | 17.2597075 | | | 416.7 | 9.91452517 | | | | | 416.70 | 11.8268223 | | | | |
| 416.8 | 5.67805151 | 416.8 | 17.2793208 | | | 416.8 | 9.91452517 | | | | | 416.80 | 11.8562423 | | | | |
| 416.9 | 5.67805151 | 416.9 | 17.2695142 | | | 416.9 | 9.89491187 | | | | | 416.90 | 11.8562423 | | | | |
| 417 | 5.66824486 | 417 | 17.2597075 | | | 417 | 9.86549191 | | | | | 417.00 | 11.8954689 | | | | |
| 417.1 | 5.66824486 | 417.1 | 17.2793208 | | | 417.1 | 9.88510522 | | | | | 417.10 | 11.9150822 | | | | |
| 417.2 | 5.6584382 | 417.2 | 17.2793208 | | | 417.2 | 9.88510522 | | | | | 417.20 | 11.9346955 | | | | |
| 417.3 | 5.6192116 | 417.3 | 17.2793208 | | | 417.3 | 9.88510522 | | | | | 417.30 | 11.9543088 | | | | |
| 417.4 | 5.62901825 | 417.4 | 17.2695142 | | | 417.4 | 9.86549191 | | | | | 417.40 | 11.9543088 | | | | |
| 417.5 | 5.64863155 | 417.5 | 17.2793208 | | | 417.5 | 9.83607196 | | | | | 417.50 | 11.9543088 | | | | |
| 417.6 | 5.66824486 | 417.6 | 17.2891275 | | | 417.6 | 9.83607196 | | | | | 417.60 | 11.9445021 | | | | |
| 417.7 | 5.68785816 | 417.7 | 17.2989341 | | | 417.7 | 9.87529856 | | | | | 417.70 | 11.9935354 | | | | |
| 417.8 | 5.69766481 | 417.8 | 17.2891275 | | | 417.8 | 9.94394513 | | | | | 417.80 | 11.9837287 | | | | |
| 417.9 | 5.66824486 | 417.9 | 17.2989341 | | | 417.9 | 9.93413848 | | | | | 417.90 | 11.9837287 | | | | |
| 418 | 5.6584382 | 418 | 17.2891275 | | | 418 | 9.91452517 | | | | | 418.00 | 12.0131487 | | | | |
| 418.1 | 5.6388249 | 418.1 | 17.2597075 | | | 418.1 | 9.87529856 | | | | | 418.10 | 12.0131487 | | | | |

| | | | | | | | | | | | | | | | | |
|---|---|---|---|---|---|---|---|---|---|---|---|---|---|---|---|---|
| 418.2 | 5.6388249 | 418.2 | 17.2302876 | | | 418.2 | 9.87529856 | | | | | 418.20 | 12.0229554 | | | |
| 418.3 | 5.6388249 | 418.3 | 17.2400942 | | | 418.3 | 9.90471852 | | | | | 418.30 | 12.0425687 | | | |
| 418.4 | 5.6388249 | 418.4 | 17.2302876 | | | 418.4 | 9.98317174 | | | | | 418.40 | 12.0425687 | | | |
| 418.5 | 5.66824486 | 418.5 | 17.2302876 | | | 418.5 | 10.0223983 | | | | | 418.50 | 12.0523753 | | | |
| 418.6 | 5.6584382 | 418.6 | 17.2400942 | | | 418.6 | 10.032205 | | | | | 418.60 | 12.0425687 | | | |
| 418.7 | 5.6584382 | 418.7 | 17.2499009 | | | 418.7 | 10.0420116 | | | | | 418.70 | 12.0229554 | | | |
| 418.8 | 5.67805151 | 418.8 | 17.2597075 | | | 418.8 | 10.0812383 | | | | | 418.80 | 12.0229554 | | | |
| 418.9 | 5.67805151 | 418.9 | 17.2793208 | | | 418.9 | 10.1106582 | | | | | 418.90 | 12.0425687 | | | |
| 419 | 5.69766481 | 419 | 17.2793208 | | | 419 | 10.061625 | | | | | 419.00 | 12.0523753 | | | |
| 419.1 | 5.69766481 | 419.1 | 17.2597075 | | | 419.1 | 10.0125917 | | | | | 419.10 | 12.0523753 | | | |
| 419.2 | 5.6584382 | 419.2 | 17.2597075 | | | 419.2 | 9.96355843 | | | | | 419.20 | 12.0425687 | | | |
| 419.3 | 5.6584382 | 419.3 | 17.2695142 | | | 419.3 | 9.96355843 | | | | | 419.30 | 12.0425687 | | | |
| 419.4 | 5.70747146 | 419.4 | 17.2793208 | | | 419.4 | 9.97336508 | | | | | 419.40 | 12.032762 | | | |
| 419.5 | 5.69766481 | 419.5 | 17.2989341 | | | 419.5 | 9.98317174 | | | | | 419.50 | 12.0425687 | | | |
| 419.6 | 5.68785816 | 419.6 | 17.2793208 | | | 419.6 | 10.032205 | | | | | 419.60 | 12.0523753 | | | |
| 419.7 | 5.69766481 | 419.7 | 17.2302876 | | | 419.7 | 10.1302715 | | | | | 419.70 | 12.032762 | | | |
| 419.8 | 5.70747146 | 419.8 | 17.2400942 | | | 419.8 | 10.1694981 | | | | | 419.80 | 12.0719886 | | | |
| 419.9 | 5.66824486 | 419.9 | 17.2499009 | | | 419.9 | 10.1302715 | | | | | 419.90 | 12.0425687 | | | |
| 420 | 5.66824486 | 420 | 17.2597075 | | | 420 | 10.1400782 | | | | | 420.00 | 12.062182 | | | |
| 420.1 | 5.70747146 | 420.1 | 17.2499009 | | | 420.1 | 10.1498848 | | | | | 420.10 | 12.0523753 | | | |
| 420.2 | 5.70747146 | 420.2 | 17.2499009 | | | 420.2 | 10.1008516 | | | | | 420.20 | 12.062182 | | | |
| 420.3 | 5.69766481 | 420.3 | 17.2400942 | | | 420.3 | 10.1302715 | | | | | 420.30 | 12.0719886 | | | |
| 420.4 | 5.69766481 | 420.4 | 17.2597075 | | | 420.4 | 10.1106582 | | | | | 420.40 | 12.062182 | | | |
| 420.5 | 5.69766481 | 420.5 | 17.2597075 | | | 420.5 | 10.1008516 | | | | | 420.50 | 12.0523753 | | | |
| 420.6 | 5.67805151 | 420.6 | 17.2499009 | | | 420.6 | 10.1008516 | | | | | 420.60 | 12.0719886 | | | |
| 420.7 | 5.67805151 | 420.7 | 17.2302876 | | | 420.7 | 10.0714316 | | | | | 420.70 | 12.0523753 | | | |
| 420.8 | 5.70747146 | 420.8 | 17.1812543 | | | 420.8 | 9.96355843 | | | | | 420.80 | 12.0719886 | | | |
| 420.9 | 5.69766481 | 420.9 | 17.1812543 | | | 420.9 | 9.806652 | | | | | 420.90 | 12.0719886 | | | |
| 421 | 5.70747146 | 421 | 17.1812543 | | | 421 | 9.71839213 | | | | | 421.00 | 12.0817953 | | | |
| 421.1 | 5.72708477 | 421.1 | 17.1714477 | | | 421.1 | 9.68897218 | | | | | 421.10 | 12.0916019 | | | |
| 421.2 | 5.73689142 | 421.2 | 17.1812543 | | | 421.2 | 9.70858548 | | | | | 421.20 | 12.0916019 | | | |
| 421.3 | 5.71727812 | 421.3 | 17.1812543 | | | 421.3 | 9.67916552 | | | | | 421.30 | 12.0817953 | | | |
| 421.4 | 5.72708477 | 421.4 | 17.1812543 | | | 421.4 | 9.72819878 | | | | | 421.40 | 12.0817953 | | | |
| 421.5 | 5.70747146 | 421.5 | 17.1812543 | | | 421.5 | 9.74781209 | | | | | 421.50 | 12.0916019 | | | |
| 421.6 | 5.71727812 | 421.6 | 17.2008676 | | | 421.6 | 9.71839213 | | | | | 421.60 | 12.0817953 | | | |
| 421.7 | 5.71727812 | 421.7 | 17.1812543 | | | 421.7 | 9.72819878 | | | | | 421.70 | 12.1014086 | | | |
| 421.8 | 5.70747146 | 421.8 | 17.191061 | | | 421.8 | 9.76742539 | | | | | 421.80 | 12.0916019 | | | |
| 421.9 | 5.73689142 | 421.9 | 17.1812543 | | | 421.9 | 9.77723204 | | | | | 421.90 | 12.0916019 | | | |
| 422 | 5.71727812 | 422 | 17.191061 | | | 422 | 9.76742539 | | | | | 422.00 | 12.1112152 | | | |
| 422.1 | 5.72708477 | 422.1 | 17.2106743 | | | 422.1 | 9.806652 | | | | | 422.10 | 12.1308285 | | | |
| 422.2 | 5.72708477 | 422.2 | 17.2499009 | | | 422.2 | 9.85568526 | | | | | 422.20 | 12.1014086 | | | |
| 422.3 | 5.68785816 | 422.3 | 17.2597075 | | | 422.3 | 9.87529856 | | | | | 422.30 | 12.1210219 | | | |
| 422.4 | 5.6584382 | 422.4 | 17.2400942 | | | 422.4 | 9.88510522 | | | | | 422.40 | 12.1308285 | | | |
| 422.5 | 5.67805151 | 422.5 | 17.2302876 | | | 422.5 | 9.89491187 | | | | | 422.50 | 12.1406352 | | | |
| 422.6 | 5.68785816 | 422.6 | 17.2400942 | | | 422.6 | 9.90471852 | | | | | 422.60 | 12.1308285 | | | |
| 422.7 | 5.73689142 | 422.7 | 17.2302876 | | | 422.7 | 9.92433182 | | | | | 422.70 | 12.1112152 | | | |
| 422.8 | 5.72708477 | 422.8 | 17.1812543 | | | 422.8 | 9.88510522 | | | | | 422.80 | 12.1406352 | | | |
| 422.9 | 5.71727812 | 422.9 | 17.1518343 | | | 422.9 | 9.91452517 | | | | | 422.90 | 12.1504418 | | | |
| 423 | 5.73689142 | 423 | 17.1714477 | | | 423 | 9.95375178 | | | | | 423.00 | 12.1504418 | | | |
| 423.1 | 5.74669807 | 423.1 | 17.1714477 | | | 423.1 | 9.96355843 | | | | | 423.10 | 12.1602485 | | | |
| 423.2 | 5.73689142 | 423.2 | 17.1812543 | | | 423.2 | 9.93413848 | | | | | 423.20 | 12.1406352 | | | |
| 423.3 | 5.73689142 | 423.3 | 17.191061 | | | 423.3 | 9.88510522 | | | | | 423.30 | 12.1308285 | | | |
| 423.4 | 5.74669807 | 423.4 | 17.2008676 | | | 423.4 | 9.86549191 | | | | | 423.40 | 12.1406352 | | | |
| 423.5 | 5.74669807 | 423.5 | 17.2008676 | | | 423.5 | 9.88510522 | | | | | 423.50 | 12.1602485 | | | |
| 423.6 | 5.73689142 | 423.6 | 17.1812543 | | | 423.6 | 9.90471852 | | | | | 423.60 | 12.1308285 | | | |
| 423.7 | 5.74669807 | 423.7 | 17.1812543 | | | 423.7 | 9.94394513 | | | | | 423.70 | 12.1308285 | | | |
| 423.8 | 5.74669807 | 423.8 | 17.2106743 | | | 423.8 | 9.94394513 | | | | | 423.80 | 12.1798618 | | | |
| 423.9 | 5.73689142 | 423.9 | 17.2106743 | | | 423.9 | 9.99297839 | | | | | 423.90 | 12.1896684 | | | |
| 424 | 5.72708477 | 424 | 17.2008676 | | | 424 | 9.99297839 | | | | | 424.00 | 12.1700551 | | | |
| 424.1 | 5.73689142 | 424.1 | 17.1714477 | | | 424.1 | 9.93413848 | | | | | 424.10 | 12.1602485 | | | |
| 424.2 | 5.75650472 | 424.2 | 17.1714477 | | | 424.2 | 9.88510522 | | | | | 424.20 | 12.1700551 | | | |

| | | | | | | | | | | | | | | | | | |
|---|---|---|---|---|---|---|---|---|---|---|---|---|---|---|---|---|---|
| 424.3 | 5.74669807 | 424.3 | 17.1812543 | | | 424.3 | 9.81645865 | | | | | 424.30 | 12.1798618 | | | | |
| 424.4 | 5.73689142 | 424.4 | 17.1812543 | | | 424.4 | 9.806652 | | | | | 424.40 | 12.1602485 | | | | |
| 424.5 | 5.75650472 | 424.5 | 17.1714477 | | | 424.5 | 9.85568526 | | | | | 424.50 | 12.1504418 | | | | |
| 424.6 | 5.75650472 | 424.6 | 17.1812543 | | | 424.6 | 9.87529856 | | | | | 424.60 | 12.1700551 | | | | |
| 424.7 | 5.78592468 | 424.7 | 17.1714477 | | | 424.7 | 9.84587861 | | | | | 424.70 | 12.1896684 | | | | |
| 424.8 | 5.78592468 | 424.8 | 17.191061 | | | 424.8 | 9.87529856 | | | | | 424.80 | 12.1896684 | | | | |
| 424.9 | 5.73689142 | 424.9 | 17.1714477 | | | 424.9 | 9.86549191 | | | | | 424.90 | 12.1700551 | | | | |
| 425 | 5.73689142 | 425 | 17.1420277 | | | 425 | 9.86549191 | | | | | 425.00 | 12.1896684 | | | | |
| 425.1 | 5.78592468 | 425.1 | 17.1518343 | | | 425.1 | 9.87529856 | | | | | 425.10 | 12.2092817 | | | | |
| 425.2 | 5.77611803 | 425.2 | 17.1714477 | | | 425.2 | 9.88510522 | | | | | 425.20 | 12.1994751 | | | | |
| 425.3 | 5.76631138 | 425.3 | 17.191061 | | | 425.3 | 9.88510522 | | | | | 425.30 | 12.2092817 | | | | |
| 425.4 | 5.77611803 | 425.4 | 17.191061 | | | 425.4 | 9.89491187 | | | | | 425.40 | 12.1896684 | | | | |
| 425.5 | 5.74669807 | 425.5 | 17.191061 | | | 425.5 | 9.90471852 | | | | | 425.50 | 12.2190884 | | | | |
| 425.6 | 5.73689142 | 425.6 | 17.1812543 | | | 425.6 | 9.89491187 | | | | | 425.60 | 12.228895 | | | | |
| 425.7 | 5.73689142 | 425.7 | 17.2008676 | | | 425.7 | 9.88510522 | | | | | 425.70 | 12.1994751 | | | | |
| 425.8 | 5.74669807 | 425.8 | 17.2106743 | | | 425.8 | 9.87529856 | | | | | 425.80 | 12.1896684 | | | | |
| 425.9 | 5.76631138 | 425.9 | 17.2106743 | | | 425.9 | 9.86549191 | | | | | 425.90 | 12.1994751 | | | | |
| 426 | 5.78592468 | 426 | 17.1714477 | | | 426 | 9.87529856 | | | | | 426.00 | 12.2485083 | | | | |
| 426.1 | 5.80553798 | 426.1 | 17.1714477 | | | 426.1 | 9.88510522 | | | | | 426.10 | 12.2387017 | | | | |
| 426.2 | 5.81534464 | 426.2 | 17.191061 | | | 426.2 | 9.86549191 | | | | | 426.20 | 12.258315 | | | | |
| 426.3 | 5.79573133 | 426.3 | 17.191061 | | | 426.3 | 9.87529856 | | | | | 426.30 | 12.2485083 | | | | |
| 426.4 | 5.76631138 | 426.4 | 17.1714477 | | | 426.4 | 9.89491187 | | | | | 426.40 | 12.258315 | | | | |
| 426.5 | 5.75650472 | 426.5 | 17.2106743 | | | 426.5 | 9.89491187 | | | | | 426.50 | 12.258315 | | | | |
| 426.6 | 5.77611803 | 426.6 | 17.2106743 | | | 426.6 | 9.94394513 | | | | | 426.60 | 12.2485083 | | | | |
| 426.7 | 5.78592468 | 426.7 | 17.1714477 | | | 426.7 | 9.92433182 | | | | | 426.70 | 12.2485083 | | | | |
| 426.8 | 5.79573133 | 426.8 | 17.1518343 | | | 426.8 | 9.90471852 | | | | | 426.80 | 12.2779283 | | | | |
| 426.9 | 5.81534464 | 426.9 | 17.161641 | | | 426.9 | 9.91452517 | | | | | 426.90 | 12.258315 | | | | |
| 427 | 5.80553798 | 427 | 17.1714477 | | | 427 | 9.89491187 | | | | | 427.00 | 12.287735 | | | | |
| 427.1 | 5.82515129 | 427.1 | 17.191061 | | | 427.1 | 9.87529856 | | | | | 427.10 | 12.287735 | | | | |
| 427.2 | 5.80553798 | 427.2 | 17.1812543 | | | 427.2 | 9.86549191 | | | | | 427.20 | 12.2975416 | | | | |
| 427.3 | 5.77611803 | 427.3 | 17.1714477 | | | 427.3 | 9.90471852 | | | | | 427.30 | 12.3073483 | | | | |
| 427.4 | 5.79573133 | 427.4 | 17.1714477 | | | 427.4 | 9.92433182 | | | | | 427.40 | 12.3171549 | | | | |
| 427.5 | 5.80553798 | 427.5 | 17.1812543 | | | 427.5 | 9.89491187 | | | | | 427.50 | 12.3269616 | | | | |
| 427.6 | 5.77611803 | 427.6 | 17.2008676 | | | 427.6 | 9.90471852 | | | | | 427.60 | 12.3073483 | | | | |
| 427.7 | 5.76631138 | 427.7 | 17.2008676 | | | 427.7 | 9.94394513 | | | | | 427.70 | 12.3171549 | | | | |
| 427.8 | 5.79573133 | 427.8 | 17.191061 | | | 427.8 | 9.91452517 | | | | | 427.80 | 12.3171549 | | | | |
| 427.9 | 5.80553798 | 427.9 | 17.1714477 | | | 427.9 | 9.92433182 | | | | | 427.90 | 12.3465749 | | | | |
| 428 | 5.79573133 | 428 | 17.161641 | | | 428 | 9.97336508 | | | | | 428.00 | 12.3367682 | | | | |
| 428.1 | 5.81534464 | 428.1 | 17.1420277 | | | 428.1 | 9.94394513 | | | | | 428.10 | 12.3367682 | | | | |
| 428.2 | 5.82515129 | 428.2 | 17.1714477 | | | 428.2 | 9.92433182 | | | | | 428.20 | 12.3269616 | | | | |
| 428.3 | 5.82515129 | 428.3 | 17.1518343 | | | 428.3 | 9.89491187 | | | | | 428.30 | 12.3367682 | | | | |
| 428.4 | 5.80553798 | 428.4 | 17.1518343 | | | 428.4 | 9.90471852 | | | | | 428.40 | 12.3563815 | | | | |
| 428.5 | 5.78592468 | 428.5 | 17.161641 | | | 428.5 | 9.89491187 | | | | | 428.50 | 12.3661882 | | | | |
| 428.6 | 5.77611803 | 428.6 | 17.1518343 | | | 428.6 | 9.89491187 | | | | | 428.60 | 12.3661882 | | | | |
| 428.7 | 5.76631138 | 428.7 | 17.1224144 | | | 428.7 | 9.96355843 | | | | | 428.70 | 12.3759948 | | | | |
| 428.8 | 5.78592468 | 428.8 | 17.132221 | | | 428.8 | 10.0518183 | | | | | 428.80 | 12.3661882 | | | | |
| 428.9 | 5.81534464 | 428.9 | 17.161641 | | | 428.9 | 10.0714316 | | | | | 428.90 | 12.3367682 | | | | |
| 429 | 5.82515129 | 429 | 17.161641 | | | 429 | 9.98317174 | | | | | 429.00 | 12.3465749 | | | | |
| 429.1 | 5.82515129 | 429.1 | 17.1714477 | | | 429.1 | 9.91452517 | | | | | 429.10 | 12.3563815 | | | | |
| 429.2 | 5.83495794 | 429.2 | 17.161641 | | | 429.2 | 9.94394513 | | | | | 429.20 | 12.3661882 | | | | |
| 429.3 | 5.82515129 | 429.3 | 17.161641 | | | 429.3 | 9.95375178 | | | | | 429.30 | 12.3956081 | | | | |
| 429.4 | 5.81534464 | 429.4 | 17.161641 | | | 429.4 | 9.95375178 | | | | | 429.40 | 12.3858015 | | | | |
| 429.5 | 5.81534464 | 429.5 | 17.1518343 | | | 429.5 | 9.88510522 | | | | | 429.50 | 12.3956081 | | | | |
| 429.6 | 5.82515129 | 429.6 | 17.1518343 | | | 429.6 | 9.89491187 | | | | | 429.60 | 12.3956081 | | | | |
| 429.7 | 5.82515129 | 429.7 | 17.132221 | | | 429.7 | 9.91452517 | | | | | 429.70 | 12.3858015 | | | | |
| 429.8 | 5.87418455 | 429.8 | 17.1420277 | | | 429.8 | 9.93413848 | | | | | 429.80 | 12.3858015 | | | | |
| 429.9 | 5.84476459 | 429.9 | 17.1224144 | | | 429.9 | 9.92433182 | | | | | 429.90 | 12.3759948 | | | | |
| 430 | 5.82515129 | 430 | 17.132221 | | | 430 | 9.91452517 | | | | | 430.00 | 12.4054148 | | | | |
| 430.1 | 5.85457124 | 430.1 | 17.1518343 | | | 430.1 | 9.91452517 | | | | | 430.10 | 12.3661882 | | | | |
| 430.2 | 5.87418455 | 430.2 | 17.1420277 | | | 430.2 | 9.91452517 | | | | | 430.20 | 12.3661882 | | | | |
| 430.3 | 5.8643779 | 430.3 | 17.161641 | | | 430.3 | 9.93413848 | | | | | 430.30 | 12.3759948 | | | | |

| | | | | | | | | | | | | | | | | | |
|---|---|---|---|---|---|---|---|---|---|---|---|---|---|---|---|---|---|
| 430.4 | 5.84476459 | 430.4 | 17.1518343 | | | 430.4 | 9.91452517 | | | | | 430.40 | 12.3661882 | | | | |
| 430.5 | 5.84476459 | 430.5 | 17.1518343 | | | 430.5 | 9.90471852 | | | | | 430.50 | 12.3661882 | | | | |
| 430.6 | 5.85457124 | 430.6 | 17.1714477 | | | 430.6 | 9.89491187 | | | | | 430.60 | 12.3759948 | | | | |
| 430.7 | 5.82515129 | 430.7 | 17.1518343 | | | 430.7 | 9.91452517 | | | | | 430.70 | 12.3563815 | | | | |
| 430.8 | 5.82515129 | 430.8 | 17.132221 | | | 430.8 | 9.92433182 | | | | | 430.80 | 12.3171549 | | | | |
| 430.9 | 5.83495794 | 430.9 | 17.161641 | | | 430.9 | 9.91452517 | | | | | 430.90 | 12.3367682 | | | | |
| 431 | 5.84476459 | 431 | 17.161641 | | | 431 | 9.93413848 | | | | | 431.00 | 12.3367682 | | | | |
| 431.1 | 5.8839912 | 431.1 | 17.1224144 | | | 431.1 | 9.94394513 | | | | | 431.10 | 12.3465749 | | | | |
| 431.2 | 5.8643779 | 431.2 | 17.1126077 | | | 431.2 | 9.95375178 | | | | | 431.20 | 12.3563815 | | | | |
| 431.3 | 5.85457124 | 431.3 | 17.1420277 | | | 431.3 | 9.93413848 | | | | | 431.30 | 12.3759948 | | | | |
| 431.4 | 5.83495794 | 431.4 | 17.1126077 | | | 431.4 | 9.93413848 | | | | | 431.40 | 12.3563815 | | | | |
| 431.5 | 5.85457124 | 431.5 | 17.132221 | | | 431.5 | 9.96355843 | | | | | 431.50 | 12.3367682 | | | | |
| 431.6 | 5.8643779 | 431.6 | 17.1518343 | | | 431.6 | 9.95375178 | | | | | 431.60 | 12.3661882 | | | | |
| 431.7 | 5.8643779 | 431.7 | 17.1420277 | | | 431.7 | 9.95375178 | | | | | 431.70 | 12.3465749 | | | | |
| 431.8 | 5.8839912 | 431.8 | 17.1420277 | | | 431.8 | 9.93413848 | | | | | 431.80 | 12.3661882 | | | | |
| 431.9 | 5.87418455 | 431.9 | 17.1224144 | | | 431.9 | 9.92433182 | | | | | 431.90 | 12.3956081 | | | | |
| 432 | 5.8643779 | 432 | 17.1126077 | | | 432 | 9.92433182 | | | | | 432.00 | 12.3858015 | | | | |
| 432.1 | 5.84476459 | 432.1 | 17.1224144 | | | 432.1 | 9.92433182 | | | | | 432.10 | 12.3858015 | | | | |
| 432.2 | 5.83495794 | 432.2 | 17.1420277 | | | 432.2 | 9.90471852 | | | | | 432.20 | 12.3956081 | | | | |
| 432.3 | 5.87418455 | 432.3 | 17.1420277 | | | 432.3 | 9.73800544 | | | | | 432.30 | 12.3759948 | | | | |
| 432.4 | 5.89379785 | 432.4 | 17.1420277 | | | 432.4 | 9.48303248 | | | | | 432.40 | 12.3073483 | | | | |
| 432.5 | 5.89379785 | 432.5 | 17.1420277 | | | 432.5 | 9.24767284 | | | | | 432.50 | 12.1994751 | | | | |
| 432.6 | 5.87418455 | 432.6 | 17.1224144 | | | 432.6 | 9.00250654 | | | | | 432.60 | 12.1308285 | | | | |
| 432.7 | 5.8839912 | 432.7 | 17.132221 | | | 432.7 | 8.77695354 | | | | | 432.70 | 12.0523753 | | | | |
| 432.8 | 5.9036045 | 432.8 | 17.0929944 | | | 432.8 | 8.48275398 | | | | | 432.80 | 12.0229554 | | | | |
| 432.9 | 5.91341116 | 432.9 | 17.1224144 | | | 432.9 | 7.78648169 | | | | | 432.90 | 12.0817953 | | | | |
| 433 | 5.87418455 | 433 | 17.132221 | | | 433 | 5.11907234 | | | | | 433.00 | 12.1700551 | | | | |
| 433.1 | 5.8839912 | 433.1 | 17.1028011 | | | 433.1 | 4.17763375 | | | | | 433.10 | 12.3171549 | | | | |
| 433.2 | 5.8839912 | 433.2 | 17.1126077 | | | 433.2 | 3.48136146 | | | | | 433.20 | 12.3858015 | | | | |
| 433.3 | 5.89379785 | 433.3 | 17.132221 | | | 433.3 | 2.95180225 | | | | | 433.30 | 12.2681217 | | | | |
| 433.4 | 5.9036045 | 433.4 | 17.1028011 | | | 433.4 | 2.54972952 | | | | | 433.40 | 12.003342 | | | | |
| 433.5 | 5.9036045 | 433.5 | 17.1126077 | | | 433.5 | 2.25552996 | | | | | 433.50 | 11.9248888 | | | | |
| 433.6 | 5.8839912 | 433.6 | 17.1224144 | | | 433.6 | 2.10843018 | | | | | 433.60 | 11.8954689 | | | | |
| 433.7 | 5.8839912 | 433.7 | 17.1224144 | | | 433.7 | 2.02997696 | | | | | 433.70 | 11.9052755 | | | | |
| 433.8 | 5.8643779 | 433.8 | 17.1028011 | | | 433.8 | 1.9417171 | | | | | 433.80 | 11.9248888 | | | | |
| 433.9 | 5.87418455 | 433.9 | 17.1224144 | | | 433.9 | 1.75539071 | | | | | 433.90 | 11.6699159 | | | | |
| 434 | 5.8839912 | 434 | 17.1224144 | | | 434 | 1.52003106 | | | | | 434.00 | 10.8657704 | | | | |
| 434.1 | 5.89379785 | 434.1 | 17.1126077 | | | 434.1 | 1.32389802 | | | | | 434.10 | 10.9736436 | | | | |
| 434.2 | 5.8839912 | 434.2 | 17.0831878 | | | 434.2 | 1.21602485 | | | | | 434.20 | 11.0520968 | | | | |
| 434.3 | 5.8839912 | 434.3 | 17.1126077 | | | 434.3 | 1.04931176 | | | | | 434.30 | 11.1011301 | | | | |
| 434.4 | 5.8643779 | 434.4 | 17.1028011 | | | 434.4 | 0.90221198 | | | | | 434.40 | 11.13055 | | | | |
| 434.5 | 5.85457124 | 434.5 | 17.0929944 | | | 434.5 | 0.74530555 | | | | | 434.50 | 11.1795833 | | | | |
| 434.6 | 5.9036045 | 434.6 | 17.1028011 | | | 434.6 | 0.63743238 | | | | | 434.60 | 11.2286165 | | | | |
| 434.7 | 5.91341116 | 434.7 | 17.1224144 | | | 434.7 | 0.58839912 | | | | | 434.70 | 11.1991966 | | | | |
| 434.8 | 5.8839912 | 434.8 | 17.1224144 | | | 434.8 | 0.52955921 | | | | | 434.80 | 11.1893899 | | | | |
| 434.9 | 5.89379785 | 434.9 | 17.1224144 | | | 434.9 | 0.44129934 | | | | | 434.90 | 11.2090032 | | | | |
| 435 | 5.9036045 | 435 | 17.1126077 | | | 435 | 0.37265278 | | | | | 435.00 | 11.2090032 | | | | |
| 435.1 | 5.93302446 | 435.1 | 17.0929944 | | | 435.1 | 0.35303947 | | | | | 435.10 | 11.2580365 | | | | |
| 435.2 | 5.95263776 | 435.2 | 17.1028011 | | | 435.2 | 0.30400621 | | | | | 435.20 | 11.2482298 | | | | |
| 435.3 | 5.91341116 | 435.3 | 17.1224144 | | | 435.3 | 0.21574634 | | | | | 435.30 | 11.2874565 | | | | |
| 435.4 | 5.91341116 | 435.4 | 17.1420277 | | | 435.4 | 0.16671308 | | | | | 435.40 | 11.3168764 | | | | |
| 435.5 | 5.91341116 | 435.5 | 17.132221 | | | 435.5 | 0.14709978 | | | | | 435.50 | 11.3070698 | | | | |
| 435.6 | 5.91341116 | 435.6 | 17.1420277 | | | 435.6 | 0.13729313 | | | | | 435.60 | 11.3070698 | | | | |
| 435.7 | 5.91341116 | 435.7 | 17.1224144 | | | 435.7 | 0.14709978 | | | | | 435.70 | 11.3168764 | | | | |
| 435.8 | 5.89379785 | 435.8 | 17.1126077 | | | 435.8 | 0.06864656 | | | | | 435.80 | 11.3168764 | | | | |
| 435.9 | 5.93302446 | 435.9 | 17.1420277 | | | 435.9 | 0.03922661 | | | | | 435.90 | 11.3168764 | | | | |
| 436 | 5.94283111 | 436 | 17.1224144 | | | 436 | 0.05883991 | | | | | 436.00 | 11.3266831 | | | | |
| 436.1 | 5.96244442 | 436.1 | 17.1126077 | | | 436.1 | -0.0196133 | | | | | 436.10 | 11.3364897 | | | | |
| 436.2 | 5.95263776 | 436.2 | 17.1126077 | | | 436.2 | -0.04903326 | | | | | 436.20 | 11.3364897 | | | | |
| 436.3 | 5.95263776 | 436.3 | 17.1224144 | | | 436.3 | -0.06864656 | | | | | 436.30 | 11.3659097 | | | | |
| 436.4 | 5.97225107 | 436.4 | 17.1126077 | | | 436.4 | -0.09806652 | | | | | 436.40 | 11.356103 | | | | |

| | | | | | | | | | | | | | | | | | |
|---|---|---|---|---|---|---|---|---|---|---|---|---|---|---|---|---|---|
| 436.5 | 5.94283111 | 436.5 | 17.1028011 | | | 436.5 | -0.19613304 | | | | | 436.50 | 11.3364897 | | | | |
| 436.6 | 5.96244442 | 436.6 | 17.1028011 | | | 436.6 | -0.25497295 | | | | | 436.60 | 11.3364897 | | | | |
| 436.7 | 5.93302446 | 436.7 | 17.0929944 | | | 436.7 | -0.27458626 | | | | | 436.70 | 11.3364897 | | | | |
| 436.8 | 5.93302446 | 436.8 | 17.0929944 | | | 436.8 | -0.32361952 | | | | | 436.80 | 11.356103 | | | | |
| 436.9 | 5.96244442 | 436.9 | 17.0831878 | | | 436.9 | -0.31381286 | | | | | 436.90 | 11.3757163 | | | | |
| 437 | 5.96244442 | 437 | 17.0831878 | | | | | | | | | 437.00 | 11.4149429 | | | | |
| 437.1 | 5.96244442 | 437.1 | 17.0929944 | | | | | | | | | 437.10 | 11.4051363 | | | | |
| 437.2 | 5.98205772 | 437.2 | 17.0733811 | | | | | | | | | 437.20 | 11.4051363 | | | | |
| 437.3 | 5.98205772 | 437.3 | 17.0831878 | | | | | | | | | 437.30 | 11.385523 | | | | |
| 437.4 | 5.96244442 | 437.4 | 17.1126077 | | | | | | | | | 437.40 | 11.4149429 | | | | |
| 437.5 | 5.96244442 | 437.5 | 17.1028011 | | | | | | | | | 437.50 | 11.4051363 | | | | |
| 437.6 | 5.97225107 | 437.6 | 17.0831878 | | | | | | | | | 437.60 | 11.4051363 | | | | |
| 437.7 | 5.99186437 | 437.7 | 17.1028011 | | | | | | | | | 437.70 | 11.4345562 | | | | |
| 437.8 | 5.99186437 | 437.8 | 17.1028011 | | | | | | | | | 437.80 | 11.4639762 | | | | |
| 437.9 | 5.98205772 | 437.9 | 17.0733811 | | | | | | | | | 437.90 | 11.4835895 | | | | |
| 438 | 6.00167102 | 438 | 17.0733811 | | | | | | | | | 438.00 | 11.4737828 | | | | |
| 438.1 | 5.97225107 | 438.1 | 17.0733811 | | | | | | | | | 438.10 | 11.4835895 | | | | |
| 438.2 | 5.98205772 | 438.2 | 17.0635745 | | | | | | | | | 438.20 | 11.4737828 | | | | |
| 438.3 | 5.99186437 | 438.3 | 17.0831878 | | | | | | | | | 438.30 | 11.4835895 | | | | |
| 438.4 | 6.00167102 | 438.4 | 17.0831878 | | | | | | | | | 438.40 | 11.5032028 | | | | |
| 438.5 | 6.00167102 | 438.5 | 17.0635745 | | | | | | | | | 438.50 | 11.5424294 | | | | |
| 438.6 | 5.98205772 | 438.6 | 17.0733811 | | | | | | | | | 438.60 | 11.4639762 | | | | |
| 438.7 | 5.98205772 | 438.7 | 17.0831878 | | | | | | | | | 438.70 | 11.4443629 | | | | |
| 438.8 | 5.97225107 | 438.8 | 17.0635745 | | | | | | | | | 438.80 | 11.4541695 | | | | |
| 438.9 | 5.97225107 | 438.9 | 17.0733811 | | | | | | | | | 438.90 | 11.4835895 | | | | |
| 439 | 6.01147768 | 439 | 17.0831878 | | | | | | | | | 439.00 | 11.5130094 | | | | |
| 439.1 | 6.02128433 | 439.1 | 17.0733811 | | | | | | | | | 439.10 | 11.5032028 | | | | |
| 439.2 | 6.02128433 | 439.2 | 17.0733811 | | | | | | | | | 439.20 | 11.5032028 | | | | |
| 439.3 | 6.01147768 | 439.3 | 17.0929944 | | | | | | | | | 439.30 | 11.4933961 | | | | |
| 439.4 | 6.02128433 | 439.4 | 17.0929944 | | | | | | | | | 439.40 | 11.5032028 | | | | |
| 439.5 | 6.03109098 | 439.5 | 17.0929944 | | | | | | | | | 439.50 | 11.5032028 | | | | |
| 439.6 | 6.04089763 | 439.6 | 17.0831878 | | | | | | | | | 439.60 | 11.5130094 | | | | |
| 439.7 | 6.03109098 | 439.7 | 17.1028011 | | | | | | | | | 439.70 | 11.5228161 | | | | |
| 439.8 | 6.04089763 | 439.8 | 17.0929944 | | | | | | | | | 439.80 | 11.5032028 | | | | |
| 439.9 | 6.05070428 | 439.9 | 17.0831878 | | | | | | | | | 439.90 | 11.5620427 | | | | |
| 440 | 6.02128433 | 440 | 17.0733811 | | | | | | | | | 440.00 | 11.5620427 | | | | |
| 440.1 | 6.01147768 | 440.1 | 17.0635745 | | | | | | | | | 440.10 | 11.5424294 | | | | |
| 440.2 | 6.03109098 | 440.2 | 17.0635745 | | | | | | | | | 440.20 | 11.5326228 | | | | |
| 440.3 | 6.02128433 | 440.3 | 17.0439612 | | | | | | | | | 440.30 | 11.5718494 | | | | |
| 440.4 | 6.04089763 | 440.4 | 17.0635745 | | | | | | | | | 440.40 | 11.5718494 | | | | |
| 440.5 | 6.04089763 | 440.5 | 17.0831878 | | | | | | | | | 440.50 | 11.5522361 | | | | |
| 440.6 | 6.01147768 | 440.6 | 17.0831878 | | | | | | | | | 440.60 | 11.6012693 | | | | |
| 440.7 | 6.01147768 | 440.7 | 17.0831878 | | | | | | | | | 440.70 | 11.6503026 | | | | |
| 440.8 | 6.04089763 | 440.8 | 17.0537678 | | | | | | | | | 440.80 | 11.6503026 | | | | |
| 440.9 | 6.02128433 | 440.9 | 17.0537678 | | | | | | | | | 440.90 | 11.6895292 | | | | |
| 441 | 6.05070428 | 441 | 17.0733811 | | | | | | | | | 441.00 | 11.7091425 | | | | |
| 441.1 | 6.03109098 | 441.1 | 17.0733811 | | | | | | | | | 441.10 | 11.7189491 | | | | |
| 441.2 | 6.05070428 | 441.2 | 17.0635745 | | | | | | | | | 441.20 | 11.6601092 | | | | |
| 441.3 | 6.04089763 | 441.3 | 17.0831878 | | | | | | | | | 441.30 | 11.6404959 | | | | |
| 441.4 | 6.04089763 | 441.4 | 17.0929944 | | | | | | | | | 441.40 | 11.6699159 | | | | |
| 441.5 | 6.07031759 | 441.5 | 17.0537678 | | | | | | | | | 441.50 | 11.6601092 | | | | |
| 441.6 | 6.04089763 | 441.6 | 17.0537678 | | | | | | | | | 441.60 | 11.6306893 | | | | |
| 441.7 | 6.03109098 | 441.7 | 17.0537678 | | | | | | | | | 441.70 | 11.5914627 | | | | |
| 441.8 | 6.04089763 | 441.8 | 17.0537678 | | | | | | | | | 441.80 | 11.5718494 | | | | |
| 441.9 | 6.03109098 | 441.9 | 17.0635745 | | | | | | | | | 441.90 | 11.581656 | | | | |
| 442 | 6.05070428 | 442 | 17.0733811 | | | | | | | | | 442.00 | 11.6208826 | | | | |
| 442.1 | 6.08012424 | 442.1 | 17.0635745 | | | | | | | | | 442.10 | 11.6208826 | | | | |
| 442.2 | 6.07031759 | 442.2 | 17.0439612 | | | | | | | | | 442.20 | 11.6601092 | | | | |
| 442.3 | 6.05070428 | 442.3 | 17.0929944 | | | | | | | | | 442.30 | 11.6797225 | | | | |
| 442.4 | 6.04089763 | 442.4 | 17.0635745 | | | | | | | | | 442.40 | 11.6503026 | | | | |
| 442.5 | 6.03109098 | 442.5 | 17.0635745 | | | | | | | | | 442.50 | 11.6601092 | | | | |

| | | | | | | | | | | | | | | | | | |
|---|---|---|---|---|---|---|---|---|---|---|---|---|---|---|---|---|---|
| 442.6 | 6.05070428 | 442.6 | 17.0733811 | | | | | | | | | 442.60 | 11.6993358 | | | | |
| 442.7 | 6.05070428 | 442.7 | 17.0537678 | | | | | | | | | 442.70 | 11.6699159 | | | | |
| 442.8 | 6.03109098 | 442.8 | 17.0439612 | | | | | | | | | 442.80 | 11.6601092 | | | | |
| 442.9 | 6.03109098 | 442.9 | 17.0537678 | | | | | | | | | 442.90 | 11.6503026 | | | | |
| 443 | 6.04089763 | 443 | 17.0439612 | | | | | | | | | 443.00 | 11.611076 | | | | |
| 443.1 | 6.08012424 | 443.1 | 17.0635745 | | | | | | | | | 443.10 | 11.5522361 | | | | |
| 443.2 | 6.07031759 | 443.2 | 17.0733811 | | | | | | | | | 443.20 | 11.5130094 | | | | |
| 443.3 | 6.03109098 | 443.3 | 17.0341545 | | | | | | | | | 443.30 | 11.5032028 | | | | |
| 443.4 | 6.05070428 | 443.4 | 17.0145412 | | | | | | | | | 443.40 | 11.4737828 | | | | |
| 443.5 | 6.05070428 | 443.5 | 17.0341545 | | | | | | | | | 443.50 | 11.5032028 | | | | |
| 443.6 | 6.09973754 | 443.6 | 17.0635745 | | | | | | | | | 443.60 | 11.5032028 | | | | |
| 443.7 | 6.06051094 | 443.7 | 17.0341545 | | | | | | | | | 443.70 | 11.5032028 | | | | |
| 443.8 | 6.08993089 | 443.8 | 17.0341545 | | | | | | | | | 443.80 | 11.5032028 | | | | |
| 443.9 | 6.04089763 | 443.9 | 17.0341545 | | | | | | | | | 443.90 | 11.5130094 | | | | |
| 444 | 6.03109098 | 444 | 17.0439612 | | | | | | | | | 444.00 | 11.5522361 | | | | |
| 444.1 | 6.06051094 | 444.1 | 17.0341545 | | | | | | | | | 444.10 | 11.5620427 | | | | |
| 444.2 | 6.06051094 | 444.2 | 17.0145412 | | | | | | | | | 444.20 | 11.5326228 | | | | |
| 444.3 | 6.07031759 | 444.3 | 17.0439612 | | | | | | | | | 444.30 | 11.5620427 | | | | |
| 444.4 | 6.07031759 | 444.4 | 17.0439612 | | | | | | | | | 444.40 | 11.5620427 | | | | |
| 444.5 | 6.07031759 | 444.5 | 17.0439612 | | | | | | | | | 444.50 | 11.5424294 | | | | |
| 444.6 | 6.08012424 | 444.6 | 17.0537678 | | | | | | | | | 444.60 | 11.6012693 | | | | |
| 444.7 | 6.08012424 | 444.7 | 17.0243479 | | | | | | | | | 444.70 | 11.581656 | | | | |
| 444.8 | 6.08993089 | 444.8 | 17.0145412 | | | | | | | | | 444.80 | 11.5522361 | | | | |
| 444.9 | 6.05070428 | 444.9 | 17.0145412 | | | | | | | | | 444.90 | 11.5228161 | | | | |
| 445 | 6.07031759 | 445 | 17.0243479 | | | | | | | | | 445.00 | 11.4835895 | | | | |
| 445.1 | 6.08993089 | 445.1 | 17.0635745 | | | | | | | | | 445.10 | 11.4149429 | | | | |
| 445.2 | 6.09973754 | 445.2 | 17.0635745 | | | | | | | | | 445.20 | 11.3462964 | | | | |
| 445.3 | 6.09973754 | 445.3 | 17.0439612 | | | | | | | | | 445.30 | 11.2580365 | | | | |
| 445.4 | 6.09973754 | 445.4 | 17.0537678 | | | | | | | | | 445.40 | 11.1893899 | | | | |
| 445.5 | 6.09973754 | 445.5 | 17.0243479 | | | | | | | | | 445.50 | 11.1403567 | | | | |
| 445.6 | 6.09973754 | 445.6 | 17.0145412 | | | | | | | | | 445.60 | 11.0324835 | | | | |
| 445.7 | 6.08993089 | 445.7 | 17.0047346 | | | | | | | | | 445.70 | 10.8951904 | | | | |
| 445.8 | 6.1291575 | 445.8 | 17.0537678 | | | | | | | | | 445.80 | 10.8755771 | | | | |
| 445.9 | 6.1095442 | 445.9 | 17.0733811 | | | | | | | | | 445.90 | 10.8265438 | | | | |
| 446 | 6.11935085 | 446 | 17.0635745 | | | | | | | | | 446.00 | 10.8069305 | | | | |
| 446.1 | 6.11935085 | 446.1 | 17.0537678 | | | | | | | | | 446.10 | 10.7971239 | | | | |
| 446.2 | 6.11935085 | 446.2 | 17.0635745 | | | | | | | | | 446.20 | 10.7284773 | | | | |
| 446.3 | 6.1095442 | 446.3 | 17.0341545 | | | | | | | | | 446.30 | 10.708864 | | | | |
| 446.4 | 6.08993089 | 446.4 | 17.0243479 | | | | | | | | | 446.40 | 10.6892507 | | | | |
| 446.5 | 6.07031759 | 446.5 | 17.0243479 | | | | | | | | | 446.50 | 10.6598307 | | | | |
| 446.6 | 6.08993089 | 446.6 | 17.0243479 | | | | | | | | | 446.60 | 10.5813775 | | | | |
| 446.7 | 6.1095442 | 446.7 | 17.0439612 | | | | | | | | | 446.70 | 10.4931176 | | | | |
| 446.8 | 6.08993089 | 446.8 | 17.0341545 | | | | | | | | | 446.80 | 10.4244711 | | | | |
| 446.9 | 6.08993089 | 446.9 | 17.0047346 | | | | | | | | | 446.90 | 10.3067913 | | | | |
| 447 | 6.09973754 | 447 | 17.0243479 | | | | | | | | | 447.00 | 10.2479513 | | | | |
| 447.1 | 6.11935085 | 447.1 | 17.0047346 | | | | | | | | | 447.10 | 10.1793048 | | | | |
| 447.2 | 6.1291575 | 447.2 | 16.9949279 | | | | | | | | | 447.20 | 10.1498848 | | | | |
| 447.3 | 6.1095442 | 447.3 | 17.0243479 | | | | | | | | | 447.30 | 10.0812383 | | | | |
| 447.4 | 6.11935085 | 447.4 | 17.0341545 | | | | | | | | | 447.40 | 10.0125917 | | | | |
| 447.5 | 6.1291575 | 447.5 | 17.0243479 | | | | | | | | | 447.50 | 9.99297839 | | | | |
| 447.6 | 6.13896415 | 447.6 | 17.0243479 | | | | | | | | | 447.60 | 9.99297839 | | | | |
| 447.7 | 6.1291575 | 447.7 | 17.0243479 | | | | | | | | | 447.70 | 9.92433182 | | | | |
| 447.8 | 6.1095442 | 447.8 | 17.0047346 | | | | | | | | | 447.80 | 9.93413848 | | | | |
| 447.9 | 6.1291575 | 447.9 | 17.0243479 | | | | | | | | | 447.90 | 9.76742539 | | | | |
| 448 | 6.1291575 | 448 | 17.0341545 | | | | | | | | | 448.00 | 9.10057306 | | | | |
| 448.1 | 6.11935085 | 448.1 | 17.0145412 | | | | | | | | | 448.10 | 8.48275398 | | | | |
| 448.2 | 6.13896415 | 448.2 | 17.0145412 | | | | | | | | | 448.20 | 8.37488081 | | | | |
| 448.3 | 6.13896415 | 448.3 | 17.0047346 | | | | | | | | | 448.30 | 8.01203468 | | | | |
| 448.4 | 6.1291575 | 448.4 | 17.0047346 | | | | | | | | | 448.40 | 7.65899521 | | | | |
| 448.5 | 6.13896415 | 448.5 | 17.0145412 | | | | | | | | | 448.50 | 7.15885596 | | | | |
| 448.6 | 6.13896415 | 448.6 | 17.0145412 | | | | | | | | | 448.60 | 6.58026349 | | | | |

| | | | | | | | | | | | | | | | | | |
|---|---|---|---|---|---|---|---|---|---|---|---|---|---|---|---|---|---|
| 448.7 | 6.13896415 | 448.7 | 17.0145412 | | | | | | | | | 448.70 | 5.93302446 | | | | |
| 448.8 | 6.16838411 | 448.8 | 17.0047346 | | | | | | | | | 448.80 | 5.15829895 | | | | |
| 448.9 | 6.17819076 | 448.9 | 17.0243479 | | | | | | | | | 448.90 | 4.12860049 | | | | |
| 449 | 6.17819076 | 449 | 17.0341545 | | | | | | | | | 449.00 | 2.58895613 | | | | |
| 449.1 | 6.17819076 | 449.1 | 17.0341545 | | | | | | | | | 449.10 | 0.97085855 | | | | |
| 449.2 | 6.15857746 | 449.2 | 17.0243479 | | | | | | | | | 449.20 | 0.04903326 | | | | |
| 449.3 | 6.13896415 | 449.3 | 17.0145412 | | | | | | | | | 449.30 | -0.35303947 | | | | |
| 449.4 | 6.16838411 | 449.4 | 17.0243479 | | | | | | | | | 449.40 | -0.34323282 | | | | |
| 449.5 | 6.16838411 | 449.5 | 17.0341545 | | | | | | | | | 449.50 | -0.34323282 | | | | |
| 449.6 | 6.16838411 | 449.6 | 17.0047346 | | | | | | | | | 449.60 | -0.35303947 | | | | |
| 449.7 | 6.15857746 | 449.7 | 17.0047346 | | | | | | | | | | | | | | |
| 449.8 | 6.15857746 | 449.8 | 16.9949279 | | | | | | | | | | | | | | |
| 449.9 | 6.17819076 | 449.9 | 16.9949279 | | | | | | | | | | | | | | |
| 450 | 6.1487708 | 450 | 17.0047346 | | | | | | | | | | | | | | |
| 450.1 | 6.15857746 | 450.1 | 16.9949279 | | | | | | | | | | | | | | |
| 450.2 | 6.18799741 | 450.2 | 17.0145412 | | | | | | | | | | | | | | |
| 450.3 | 6.17819076 | 450.3 | 17.0047346 | | | | | | | | | | | | | | |
| 450.4 | 6.17819076 | 450.4 | 16.9949279 | | | | | | | | | | | | | | |
| 450.5 | 6.15857746 | 450.5 | 16.9949279 | | | | | | | | | | | | | | |
| 450.6 | 6.16838411 | 450.6 | 17.0047346 | | | | | | | | | | | | | | |
| 450.7 | 6.16838411 | 450.7 | 16.9851213 | | | | | | | | | | | | | | |
| 450.8 | 6.17819076 | 450.8 | 16.9851213 | | | | | | | | | | | | | | |
| 450.9 | 6.13896415 | 450.9 | 17.0243479 | | | | | | | | | | | | | | |
| 451 | 6.11935085 | 451 | 17.0047346 | | | | | | | | | | | | | | |
| 451.1 | 6.16838411 | 451.1 | 16.9851213 | | | | | | | | | | | | | | |
| 451.2 | 6.17819076 | 451.2 | 17.0047346 | | | | | | | | | | | | | | |
| 451.3 | 6.17819076 | 451.3 | 16.9949279 | | | | | | | | | | | | | | |
| 451.4 | 6.17819076 | 451.4 | 16.9949279 | | | | | | | | | | | | | | |
| 451.5 | 6.17819076 | 451.5 | 16.9949279 | | | | | | | | | | | | | | |
| 451.6 | 6.19780406 | 451.6 | 16.9753146 | | | | | | | | | | | | | | |
| 451.7 | 6.21741737 | 451.7 | 16.9851213 | | | | | | | | | | | | | | |
| 451.8 | 6.17819076 | 451.8 | 16.9949279 | | | | | | | | | | | | | | |
| 451.9 | 6.15857746 | 451.9 | 17.0047346 | | | | | | | | | | | | | | |
| 452 | 6.15857746 | 452 | 17.0047346 | | | | | | | | | | | | | | |
| 452.1 | 6.18799741 | 452.1 | 17.0047346 | | | | | | | | | | | | | | |
| 452.2 | 6.17819076 | 452.2 | 17.0047346 | | | | | | | | | | | | | | |
| 452.3 | 6.19780406 | 452.3 | 16.9949279 | | | | | | | | | | | | | | |
| 452.4 | 6.21741737 | 452.4 | 16.9949279 | | | | | | | | | | | | | | |
| 452.5 | 6.19780406 | 452.5 | 17.0047346 | | | | | | | | | | | | | | |
| 452.6 | 6.19780406 | 452.6 | 17.0047346 | | | | | | | | | | | | | | |
| 452.7 | 6.21741737 | 452.7 | 16.9949279 | | | | | | | | | | | | | | |
| 452.8 | 6.18799741 | 452.8 | 17.0047346 | | | | | | | | | | | | | | |
| 452.9 | 6.18799741 | 452.9 | 16.9753146 | | | | | | | | | | | | | | |
| 453 | 6.24683732 | 453 | 16.9851213 | | | | | | | | | | | | | | |
| 453.1 | 6.23703067 | 453.1 | 17.0047346 | | | | | | | | | | | | | | |
| 453.2 | 6.21741737 | 453.2 | 17.0047346 | | | | | | | | | | | | | | |
| 453.3 | 6.22722402 | 453.3 | 16.9949279 | | | | | | | | | | | | | | |
| 453.4 | 6.22722402 | 453.4 | 17.0047346 | | | | | | | | | | | | | | |
| 453.5 | 6.23703067 | 453.5 | 17.0047346 | | | | | | | | | | | | | | |
| 453.6 | 6.21741737 | 453.6 | 16.9851213 | | | | | | | | | | | | | | |
| 453.7 | 6.22722402 | 453.7 | 16.9753146 | | | | | | | | | | | | | | |
| 453.8 | 6.22722402 | 453.8 | 17.0047346 | | | | | | | | | | | | | | |
| 453.9 | 6.24683732 | 453.9 | 16.965508 | | | | | | | | | | | | | | |
| 454 | 6.22722402 | 454 | 16.965508 | | | | | | | | | | | | | | |
| 454.1 | 6.21741737 | 454.1 | 16.9753146 | | | | | | | | | | | | | | |
| 454.2 | 6.23703067 | 454.2 | 16.9753146 | | | | | | | | | | | | | | |
| 454.3 | 6.22722402 | 454.3 | 16.9753146 | | | | | | | | | | | | | | |
| 454.4 | 6.23703067 | 454.4 | 16.9949279 | | | | | | | | | | | | | | |
| 454.5 | 6.24683732 | 454.5 | 16.9949279 | | | | | | | | | | | | | | |
| 454.6 | 6.26645063 | 454.6 | 16.9557013 | | | | | | | | | | | | | | |
| 454.7 | 6.25664398 | 454.7 | 16.9557013 | | | | | | | | | | | | | | |

| | | | | | | | | | | | | | | | | | |
|---|---|---|---|---|---|---|---|---|---|---|---|---|---|---|---|---|---|
| 454.8 | 6.25664398 | 454.8 | 16.965508 | | | | | | | | | | | | | | |
| 454.9 | 6.26645063 | 454.9 | 16.9458947 | | | | | | | | | | | | | | |
| 455 | 6.25664398 | 455 | 16.9753146 | | | | | | | | | | | | | | |
| 455.1 | 6.24683732 | 455.1 | 16.9949279 | | | | | | | | | | | | | | |
| 455.2 | 6.26645063 | 455.2 | 16.965508 | | | | | | | | | | | | | | |
| 455.3 | 6.24683732 | 455.3 | 16.9458947 | | | | | | | | | | | | | | |
| 455.4 | 6.26645063 | 455.4 | 16.965508 | | | | | | | | | | | | | | |
| 455.5 | 6.27625728 | 455.5 | 16.9753146 | | | | | | | | | | | | | | |
| 455.6 | 6.25664398 | 455.6 | 16.9753146 | | | | | | | | | | | | | | |
| 455.7 | 6.28606393 | 455.7 | 16.9851213 | | | | | | | | | | | | | | |
| 455.8 | 6.26645063 | 455.8 | 16.965508 | | | | | | | | | | | | | | |
| 455.9 | 6.27625728 | 455.9 | 16.965508 | | | | | | | | | | | | | | |
| 456 | 6.28606393 | 456 | 16.965508 | | | | | | | | | | | | | | |
| 456.1 | 6.25664398 | 456.1 | 16.965508 | | | | | | | | | | | | | | |
| 456.2 | 6.26645063 | 456.2 | 16.9458947 | | | | | | | | | | | | | | |
| 456.3 | 6.28606393 | 456.3 | 16.9557013 | | | | | | | | | | | | | | |
| 456.4 | 6.26645063 | 456.4 | 16.9557013 | | | | | | | | | | | | | | |
| 456.5 | 6.27625728 | 456.5 | 16.9262814 | | | | | | | | | | | | | | |
| 456.6 | 6.27625728 | 456.6 | 16.936088 | | | | | | | | | | | | | | |
| 456.7 | 6.26645063 | 456.7 | 16.9557013 | | | | | | | | | | | | | | |
| 456.8 | 6.29587058 | 456.8 | 16.9458947 | | | | | | | | | | | | | | |
| 456.9 | 6.27625728 | 456.9 | 16.9557013 | | | | | | | | | | | | | | |
| 457 | 6.26645063 | 457 | 16.9851213 | | | | | | | | | | | | | | |
| 457.1 | 6.29587058 | 457.1 | 16.965508 | | | | | | | | | | | | | | |
| 457.2 | 6.30567724 | 457.2 | 16.9458947 | | | | | | | | | | | | | | |
| 457.3 | 6.28606393 | 457.3 | 16.9557013 | | | | | | | | | | | | | | |
| 457.4 | 6.27625728 | 457.4 | 16.9557013 | | | | | | | | | | | | | | |
| 457.5 | 6.31548389 | 457.5 | 16.965508 | | | | | | | | | | | | | | |
| 457.6 | 6.29587058 | 457.6 | 16.965508 | | | | | | | | | | | | | | |
| 457.7 | 6.30567724 | 457.7 | 16.9458947 | | | | | | | | | | | | | | |
| 457.8 | 6.29587058 | 457.8 | 16.9458947 | | | | | | | | | | | | | | |
| 457.9 | 6.31548389 | 457.9 | 16.9557013 | | | | | | | | | | | | | | |
| 458 | 6.30567724 | 458 | 16.965508 | | | | | | | | | | | | | | |
| 458.1 | 6.32529054 | 458.1 | 16.9458947 | | | | | | | | | | | | | | |
| 458.2 | 6.31548389 | 458.2 | 16.936088 | | | | | | | | | | | | | | |
| 458.3 | 6.28606393 | 458.3 | 16.9557013 | | | | | | | | | | | | | | |
| 458.4 | 6.30567724 | 458.4 | 16.9458947 | | | | | | | | | | | | | | |
| 458.5 | 6.32529054 | 458.5 | 16.9753146 | | | | | | | | | | | | | | |
| 458.6 | 6.32529054 | 458.6 | 16.965508 | | | | | | | | | | | | | | |
| 458.7 | 6.33509719 | 458.7 | 16.965508 | | | | | | | | | | | | | | |
| 458.8 | 6.30567724 | 458.8 | 16.9458947 | | | | | | | | | | | | | | |
| 458.9 | 6.30567724 | 458.9 | 16.936088 | | | | | | | | | | | | | | |
| 459 | 6.32529054 | 459 | 16.936088 | | | | | | | | | | | | | | |
| 459.1 | 6.31548389 | 459.1 | 16.936088 | | | | | | | | | | | | | | |
| 459.2 | 6.34490384 | 459.2 | 16.936088 | | | | | | | | | | | | | | |
| 459.3 | 6.33509719 | 459.3 | 16.9458947 | | | | | | | | | | | | | | |
| 459.4 | 6.31548389 | 459.4 | 16.965508 | | | | | | | | | | | | | | |
| 459.5 | 6.29587058 | 459.5 | 16.9458947 | | | | | | | | | | | | | | |
| 459.6 | 6.28606393 | 459.6 | 16.936088 | | | | | | | | | | | | | | |
| 459.7 | 6.32529054 | 459.7 | 16.936088 | | | | | | | | | | | | | | |
| 459.8 | 6.33509719 | 459.8 | 16.965508 | | | | | | | | | | | | | | |
| 459.9 | 6.3743238 | 459.9 | 16.9458947 | | | | | | | | | | | | | | |
| 460 | 6.36451715 | 460 | 16.9458947 | | | | | | | | | | | | | | |
| 460.1 | 6.33509719 | 460.1 | 16.9458947 | | | | | | | | | | | | | | |
| 460.2 | 6.33509719 | 460.2 | 16.9164747 | | | | | | | | | | | | | | |
| 460.3 | 6.33509719 | 460.3 | 16.906668 | | | | | | | | | | | | | | |
| 460.4 | 6.33509719 | 460.4 | 16.9164747 | | | | | | | | | | | | | | |
| 460.5 | 6.36451715 | 460.5 | 16.936088 | | | | | | | | | | | | | | |
| 460.6 | 6.36451715 | 460.6 | 16.9262814 | | | | | | | | | | | | | | |
| 460.7 | 6.3547105 | 460.7 | 16.9262814 | | | | | | | | | | | | | | |
| 460.8 | 6.3547105 | 460.8 | 16.9164747 | | | | | | | | | | | | | | |

| | | | | | | | | | | | | | | | | | |
|---|---|---|---|---|---|---|---|---|---|---|---|---|---|---|---|---|---|
| 460.9 | 6.3547105 | 460.9 | 16.9164747 | | | | | | | | | | | | | | |
| 461 | 6.34490384 | 461 | 16.9458947 | | | | | | | | | | | | | | |
| 461.1 | 6.3547105 | 461.1 | 16.9458947 | | | | | | | | | | | | | | |
| 461.2 | 6.36451715 | 461.2 | 16.936088 | | | | | | | | | | | | | | |
| 461.3 | 6.3743238 | 461.3 | 16.9164747 | | | | | | | | | | | | | | |
| 461.4 | 6.36451715 | 461.4 | 16.9262814 | | | | | | | | | | | | | | |
| 461.5 | 6.36451715 | 461.5 | 16.9262814 | | | | | | | | | | | | | | |
| 461.6 | 6.3743238 | 461.6 | 16.9164747 | | | | | | | | | | | | | | |
| 461.7 | 6.36451715 | 461.7 | 16.9262814 | | | | | | | | | | | | | | |
| 461.8 | 6.36451715 | 461.8 | 16.936088 | | | | | | | | | | | | | | |
| 461.9 | 6.3939371 | 461.9 | 16.9262814 | | | | | | | | | | | | | | |
| 462 | 6.40374376 | 462 | 16.8870547 | | | | | | | | | | | | | | |
| 462.1 | 6.38413045 | 462.1 | 16.9164747 | | | | | | | | | | | | | | |
| 462.2 | 6.3743238 | 462.2 | 16.936088 | | | | | | | | | | | | | | |
| 462.3 | 6.3743238 | 462.3 | 16.8968614 | | | | | | | | | | | | | | |
| 462.4 | 6.3743238 | 462.4 | 16.8870547 | | | | | | | | | | | | | | |
| 462.5 | 6.3743238 | 462.5 | 16.9164747 | | | | | | | | | | | | | | |
| 462.6 | 6.40374376 | 462.6 | 16.9262814 | | | | | | | | | | | | | | |
| 462.7 | 6.3939371 | 462.7 | 16.9458947 | | | | | | | | | | | | | | |
| 462.8 | 6.40374376 | 462.8 | 16.9262814 | | | | | | | | | | | | | | |
| 462.9 | 6.42335706 | 462.9 | 16.9164747 | | | | | | | | | | | | | | |
| 463 | 6.42335706 | 463 | 16.8968614 | | | | | | | | | | | | | | |
| 463.1 | 6.3939371 | 463.1 | 16.8968614 | | | | | | | | | | | | | | |
| 463.2 | 6.3939371 | 463.2 | 16.9262814 | | | | | | | | | | | | | | |
| 463.3 | 6.40374376 | 463.3 | 16.9164747 | | | | | | | | | | | | | | |
| 463.4 | 6.38413045 | 463.4 | 16.9164747 | | | | | | | | | | | | | | |
| 463.5 | 6.3939371 | 463.5 | 16.9262814 | | | | | | | | | | | | | | |
| 463.6 | 6.3939371 | 463.6 | 16.9262814 | | | | | | | | | | | | | | |
| 463.7 | 6.41355041 | 463.7 | 16.8870547 | | | | | | | | | | | | | | |
| 463.8 | 6.3939371 | 463.8 | 16.8772481 | | | | | | | | | | | | | | |
| 463.9 | 6.40374376 | 463.9 | 16.906668 | | | | | | | | | | | | | | |
| 464 | 6.40374376 | 464 | 16.9164747 | | | | | | | | | | | | | | |
| 464.1 | 6.43316371 | 464.1 | 16.8968614 | | | | | | | | | | | | | | |
| 464.2 | 6.45277702 | 464.2 | 16.8772481 | | | | | | | | | | | | | | |
| 464.3 | 6.42335706 | 464.3 | 16.8576348 | | | | | | | | | | | | | | |
| 464.4 | 6.41355041 | 464.4 | 16.8772481 | | | | | | | | | | | | | | |
| 464.5 | 6.46258367 | 464.5 | 16.8870547 | | | | | | | | | | | | | | |
| 464.6 | 6.47239032 | 464.6 | 16.8870547 | | | | | | | | | | | | | | |
| 464.7 | 6.48219697 | 464.7 | 16.8968614 | | | | | | | | | | | | | | |
| 464.8 | 6.45277702 | 464.8 | 16.906668 | | | | | | | | | | | | | | |
| 464.9 | 6.40374376 | 464.9 | 16.8870547 | | | | | | | | | | | | | | |
| 465 | 6.42335706 | 465 | 16.8968614 | | | | | | | | | | | | | | |
| 465.1 | 6.44297036 | 465.1 | 16.9164747 | | | | | | | | | | | | | | |
| 465.2 | 6.43316371 | 465.2 | 16.9164747 | | | | | | | | | | | | | | |
| 465.3 | 6.43316371 | 465.3 | 16.8968614 | | | | | | | | | | | | | | |
| 465.4 | 6.44297036 | 465.4 | 16.8870547 | | | | | | | | | | | | | | |
| 465.5 | 6.45277702 | 465.5 | 16.8968614 | | | | | | | | | | | | | | |
| 465.6 | 6.43316371 | 465.6 | 16.906668 | | | | | | | | | | | | | | |
| 465.7 | 6.42335706 | 465.7 | 16.9262814 | | | | | | | | | | | | | | |
| 465.8 | 6.43316371 | 465.8 | 16.8968614 | | | | | | | | | | | | | | |
| 465.9 | 6.43316371 | 465.9 | 16.9164747 | | | | | | | | | | | | | | |
| 466 | 6.41355041 | 466 | 16.9262814 | | | | | | | | | | | | | | |
| 466.1 | 6.44297036 | 466.1 | 16.9262814 | | | | | | | | | | | | | | |
| 466.2 | 6.46258367 | 466.2 | 16.9164747 | | | | | | | | | | | | | | |
| 466.3 | 6.44297036 | 466.3 | 16.906668 | | | | | | | | | | | | | | |
| 466.4 | 6.47239032 | 466.4 | 16.8968614 | | | | | | | | | | | | | | |
| 466.5 | 6.46258367 | 466.5 | 16.906668 | | | | | | | | | | | | | | |
| 466.6 | 6.47239032 | 466.6 | 16.8870547 | | | | | | | | | | | | | | |
| 466.7 | 6.50181028 | 466.7 | 16.8674414 | | | | | | | | | | | | | | |
| 466.8 | 6.46258367 | 466.8 | 16.8772481 | | | | | | | | | | | | | | |
| 466.9 | 6.44297036 | 466.9 | 16.8968614 | | | | | | | | | | | | | | |

| | | | | | | | | | | | | | | | | | |
|---|---|---|---|---|---|---|---|---|---|---|---|---|---|---|---|---|---|
| 467 | 6.46258367 | 467 | 16.8968614 | | | | | | | | | | | | | | |
| 467.1 | 6.47239032 | 467.1 | 16.8772481 | | | | | | | | | | | | | | |
| 467.2 | 6.45277702 | 467.2 | 16.8772481 | | | | | | | | | | | | | | |
| 467.3 | 6.46258367 | 467.3 | 16.8674414 | | | | | | | | | | | | | | |
| 467.4 | 6.50181028 | 467.4 | 16.8576348 | | | | | | | | | | | | | | |
| 467.5 | 6.51161693 | 467.5 | 16.8772481 | | | | | | | | | | | | | | |
| 467.6 | 6.49200362 | 467.6 | 16.8772481 | | | | | | | | | | | | | | |
| 467.7 | 6.49200362 | 467.7 | 16.8674414 | | | | | | | | | | | | | | |
| 467.8 | 6.46258367 | 467.8 | 16.8968614 | | | | | | | | | | | | | | |
| 467.9 | 6.46258367 | 467.9 | 16.906668 | | | | | | | | | | | | | | |
| 468 | 6.46258367 | 468 | 16.8870547 | | | | | | | | | | | | | | |
| 468.1 | 6.48219697 | 468.1 | 16.8870547 | | | | | | | | | | | | | | |
| 468.2 | 6.45277702 | 468.2 | 16.8870547 | | | | | | | | | | | | | | |
| 468.3 | 6.45277702 | 468.3 | 16.8674414 | | | | | | | | | | | | | | |
| 468.4 | 6.49200362 | 468.4 | 16.8674414 | | | | | | | | | | | | | | |
| 468.5 | 6.48219697 | 468.5 | 16.8870547 | | | | | | | | | | | | | | |
| 468.6 | 6.49200362 | 468.6 | 16.8870547 | | | | | | | | | | | | | | |
| 468.7 | 6.50181028 | 468.7 | 16.8576348 | | | | | | | | | | | | | | |
| 468.8 | 6.53123023 | 468.8 | 16.8576348 | | | | | | | | | | | | | | |
| 468.9 | 6.51161693 | 468.9 | 16.8478281 | | | | | | | | | | | | | | |
| 469 | 6.51161693 | 469 | 16.8380215 | | | | | | | | | | | | | | |
| 469.1 | 6.50181028 | 469.1 | 16.8576348 | | | | | | | | | | | | | | |
| 469.2 | 6.50181028 | 469.2 | 16.8576348 | | | | | | | | | | | | | | |
| 469.3 | 6.53123023 | 469.3 | 16.8380215 | | | | | | | | | | | | | | |
| 469.4 | 6.55084354 | 469.4 | 16.8576348 | | | | | | | | | | | | | | |
| 469.5 | 6.57045684 | 469.5 | 16.8674414 | | | | | | | | | | | | | | |
| 469.6 | 6.53123023 | 469.6 | 16.8772481 | | | | | | | | | | | | | | |
| 469.7 | 6.51161693 | 469.7 | 16.8674414 | | | | | | | | | | | | | | |
| 469.8 | 6.50181028 | 469.8 | 16.8576348 | | | | | | | | | | | | | | |
| 469.9 | 6.51161693 | 469.9 | 16.8674414 | | | | | | | | | | | | | | |
| 470 | 6.54103688 | 470 | 16.8674414 | | | | | | | | | | | | | | |
| 470.1 | 6.54103688 | 470.1 | 16.8576348 | | | | | | | | | | | | | | |
| 470.2 | 6.53123023 | 470.2 | 16.8478281 | | | | | | | | | | | | | | |
| 470.3 | 6.56065019 | 470.3 | 16.8478281 | | | | | | | | | | | | | | |
| 470.4 | 6.56065019 | 470.4 | 16.8576348 | | | | | | | | | | | | | | |
| 470.5 | 6.53123023 | 470.5 | 16.8576348 | | | | | | | | | | | | | | |
| 470.6 | 6.55084354 | 470.6 | 16.8576348 | | | | | | | | | | | | | | |
| 470.7 | 6.55084354 | 470.7 | 16.8576348 | | | | | | | | | | | | | | |
| 470.8 | 6.53123023 | 470.8 | 16.8674414 | | | | | | | | | | | | | | |
| 470.9 | 6.53123023 | 470.9 | 16.8674414 | | | | | | | | | | | | | | |
| 471 | 6.55084354 | 471 | 16.8772481 | | | | | | | | | | | | | | |
| 471.1 | 6.52142358 | 471.1 | 16.8576348 | | | | | | | | | | | | | | |
| 471.2 | 6.55084354 | 471.2 | 16.8576348 | | | | | | | | | | | | | | |
| 471.3 | 6.57045684 | 471.3 | 16.8576348 | | | | | | | | | | | | | | |
| 471.4 | 6.59007014 | 471.4 | 16.8380215 | | | | | | | | | | | | | | |
| 471.5 | 6.57045684 | 471.5 | 16.8380215 | | | | | | | | | | | | | | |
| 471.6 | 6.55084354 | 471.6 | 16.8478281 | | | | | | | | | | | | | | |
| 471.7 | 6.56065019 | 471.7 | 16.8478281 | | | | | | | | | | | | | | |
| 471.8 | 6.59007014 | 471.8 | 16.8478281 | | | | | | | | | | | | | | |
| 471.9 | 6.57045684 | 471.9 | 16.8380215 | | | | | | | | | | | | | | |
| 472 | 6.55084354 | 472 | 16.8184082 | | | | | | | | | | | | | | |
| 472.1 | 6.52142358 | 472.1 | 16.8282148 | | | | | | | | | | | | | | |
| 472.2 | 6.52142358 | 472.2 | 16.8380215 | | | | | | | | | | | | | | |
| 472.3 | 6.57045684 | 472.3 | 16.8380215 | | | | | | | | | | | | | | |
| 472.4 | 6.60968345 | 472.4 | 16.8674414 | | | | | | | | | | | | | | |
| 472.5 | 6.5998768 | 472.5 | 16.8674414 | | | | | | | | | | | | | | |
| 472.6 | 6.57045684 | 472.6 | 16.8576348 | | | | | | | | | | | | | | |
| 472.7 | 6.58026349 | 472.7 | 16.8576348 | | | | | | | | | | | | | | |
| 472.8 | 6.59007014 | 472.8 | 16.8478281 | | | | | | | | | | | | | | |
| 472.9 | 6.56065019 | 472.9 | 16.8380215 | | | | | | | | | | | | | | |
| 473 | 6.56065019 | 473 | 16.8576348 | | | | | | | | | | | | | | |

| | | | | | | | | | | | | | | | | | |
|---|---|---|---|---|---|---|---|---|---|---|---|---|---|---|---|---|---|
| 473.1 | 6.5998768 | 473.1 | 16.8380215 | | | | | | | | | | | | | | |
| 473.2 | 6.6194901 | 473.2 | 16.8576348 | | | | | | | | | | | | | | |
| 473.3 | 6.5998768 | 473.3 | 16.8478281 | | | | | | | | | | | | | | |
| 473.4 | 6.59007014 | 473.4 | 16.8380215 | | | | | | | | | | | | | | |
| 473.5 | 6.59007014 | 473.5 | 16.8282148 | | | | | | | | | | | | | | |
| 473.6 | 6.59007014 | 473.6 | 16.8478281 | | | | | | | | | | | | | | |
| 473.7 | 6.6194901 | 473.7 | 16.8184082 | | | | | | | | | | | | | | |
| 473.8 | 6.6194901 | 473.8 | 16.7987949 | | | | | | | | | | | | | | |
| 473.9 | 6.5998768 | 473.9 | 16.7987949 | | | | | | | | | | | | | | |
| 474 | 6.5998768 | 474 | 16.7987949 | | | | | | | | | | | | | | |
| 474.1 | 6.60968345 | 474.1 | 16.8380215 | | | | | | | | | | | | | | |
| 474.2 | 6.62929675 | 474.2 | 16.8282148 | | | | | | | | | | | | | | |
| 474.3 | 6.6391034 | 474.3 | 16.8086015 | | | | | | | | | | | | | | |
| 474.4 | 6.62929675 | 474.4 | 16.8184082 | | | | | | | | | | | | | | |
| 474.5 | 6.5998768 | 474.5 | 16.8380215 | | | | | | | | | | | | | | |
| 474.6 | 6.62929675 | 474.6 | 16.8282148 | | | | | | | | | | | | | | |
| 474.7 | 6.6391034 | 474.7 | 16.8380215 | | | | | | | | | | | | | | |
| 474.8 | 6.65871671 | 474.8 | 16.8380215 | | | | | | | | | | | | | | |
| 474.9 | 6.6391034 | 474.9 | 16.8478281 | | | | | | | | | | | | | | |
| 475 | 6.5998768 | 475 | 16.8282148 | | | | | | | | | | | | | | |
| 475.1 | 6.60968345 | 475.1 | 16.8086015 | | | | | | | | | | | | | | |
| 475.2 | 6.62929675 | 475.2 | 16.8184082 | | | | | | | | | | | | | | |
| 475.3 | 6.60968345 | 475.3 | 16.8380215 | | | | | | | | | | | | | | |
| 475.4 | 6.62929675 | 475.4 | 16.8184082 | | | | | | | | | | | | | | |
| 475.5 | 6.64891006 | 475.5 | 16.8282148 | | | | | | | | | | | | | | |
| 475.6 | 6.6391034 | 475.6 | 16.8184082 | | | | | | | | | | | | | | |
| 475.7 | 6.62929675 | 475.7 | 16.8184082 | | | | | | | | | | | | | | |
| 475.8 | 6.62929675 | 475.8 | 16.7987949 | | | | | | | | | | | | | | |
| 475.9 | 6.64891006 | 475.9 | 16.7889882 | | | | | | | | | | | | | | |
| 476 | 6.6391034 | 476 | 16.8086015 | | | | | | | | | | | | | | |
| 476.1 | 6.65871671 | 476.1 | 16.8086015 | | | | | | | | | | | | | | |
| 476.2 | 6.65871671 | 476.2 | 16.7889882 | | | | | | | | | | | | | | |
| 476.3 | 6.67833001 | 476.3 | 16.7791816 | | | | | | | | | | | | | | |
| 476.4 | 6.66852336 | 476.4 | 16.7987949 | | | | | | | | | | | | | | |
| 476.5 | 6.66852336 | 476.5 | 16.8086015 | | | | | | | | | | | | | | |
| 476.6 | 6.65871671 | 476.6 | 16.8184082 | | | | | | | | | | | | | | |
| 476.7 | 6.65871671 | 476.7 | 16.7987949 | | | | | | | | | | | | | | |
| 476.8 | 6.66852336 | 476.8 | 16.8086015 | | | | | | | | | | | | | | |
| 476.9 | 6.65871671 | 476.9 | 16.8184082 | | | | | | | | | | | | | | |
| 477 | 6.6391034 | 477 | 16.8184082 | | | | | | | | | | | | | | |
| 477.1 | 6.66852336 | 477.1 | 16.8184082 | | | | | | | | | | | | | | |
| 477.2 | 6.65871671 | 477.2 | 16.8086015 | | | | | | | | | | | | | | |
| 477.3 | 6.66852336 | 477.3 | 16.7987949 | | | | | | | | | | | | | | |
| 477.4 | 6.67833001 | 477.4 | 16.7987949 | | | | | | | | | | | | | | |
| 477.5 | 6.68813666 | 477.5 | 16.8086015 | | | | | | | | | | | | | | |
| 477.6 | 6.69794332 | 477.6 | 16.7987949 | | | | | | | | | | | | | | |
| 477.7 | 6.69794332 | 477.7 | 16.7987949 | | | | | | | | | | | | | | |
| 477.8 | 6.66852336 | 477.8 | 16.8184082 | | | | | | | | | | | | | | |
| 477.9 | 6.67833001 | 477.9 | 16.8282148 | | | | | | | | | | | | | | |
| 478 | 6.68813666 | 478 | 16.8282148 | | | | | | | | | | | | | | |
| 478.1 | 6.70774997 | 478.1 | 16.8380215 | | | | | | | | | | | | | | |
| 478.2 | 6.70774997 | 478.2 | 16.8478281 | | | | | | | | | | | | | | |
| 478.3 | 6.70774997 | 478.3 | 16.8380215 | | | | | | | | | | | | | | |
| 478.4 | 6.68813666 | 478.4 | 16.8086015 | | | | | | | | | | | | | | |
| 478.5 | 6.70774997 | 478.5 | 16.7791816 | | | | | | | | | | | | | | |
| 478.6 | 6.70774997 | 478.6 | 16.7693749 | | | | | | | | | | | | | | |
| 478.7 | 6.68813666 | 478.7 | 16.7889882 | | | | | | | | | | | | | | |
| 478.8 | 6.69794332 | 478.8 | 16.7889882 | | | | | | | | | | | | | | |
| 478.9 | 6.71755662 | 478.9 | 16.7791816 | | | | | | | | | | | | | | |
| 479 | 6.71755662 | 479 | 16.7889882 | | | | | | | | | | | | | | |
| 479.1 | 6.70774997 | 479.1 | 16.7693749 | | | | | | | | | | | | | | |

| | | | | | | | | | | | | | | | | | |
|---|---|---|---|---|---|---|---|---|---|---|---|---|---|---|---|---|---|
| 479.2 | 6.73716992 | 479.2 | 16.7693749 | | | | | | | | | | | | | | |
| 479.3 | 6.73716992 | 479.3 | 16.7889882 | | | | | | | | | | | | | | |
| 479.4 | 6.72736327 | 479.4 | 16.7889882 | | | | | | | | | | | | | | |
| 479.5 | 6.71755662 | 479.5 | 16.7987949 | | | | | | | | | | | | | | |
| 479.6 | 6.72736327 | 479.6 | 16.8086015 | | | | | | | | | | | | | | |
| 479.7 | 6.72736327 | 479.7 | 16.8282148 | | | | | | | | | | | | | | |
| 479.8 | 6.71755662 | 479.8 | 16.8380215 | | | | | | | | | | | | | | |
| 479.9 | 6.72736327 | 479.9 | 16.8184082 | | | | | | | | | | | | | | |
| 480 | 6.74697658 | 480 | 16.739955 | | | | | | | | | | | | | | |
| 480.1 | 6.71755662 | 480.1 | 16.7595683 | | | | | | | | | | | | | | |
| 480.2 | 6.72736327 | 480.2 | 16.7693749 | | | | | | | | | | | | | | |
| 480.3 | 6.73716992 | 480.3 | 16.7791816 | | | | | | | | | | | | | | |
| 480.4 | 6.73716992 | 480.4 | 16.7889882 | | | | | | | | | | | | | | |
| 480.5 | 6.75678323 | 480.5 | 16.8086015 | | | | | | | | | | | | | | |
| 480.6 | 6.74697658 | 480.6 | 16.7987949 | | | | | | | | | | | | | | |
| 480.7 | 6.72736327 | 480.7 | 16.8086015 | | | | | | | | | | | | | | |
| 480.8 | 6.72736327 | 480.8 | 16.7693749 | | | | | | | | | | | | | | |
| 480.9 | 6.74697658 | 480.9 | 16.7693749 | | | | | | | | | | | | | | |
| 481 | 6.74697658 | 481 | 16.7987949 | | | | | | | | | | | | | | |
| 481.1 | 6.75678323 | 481.1 | 16.8086015 | | | | | | | | | | | | | | |
| 481.2 | 6.76658988 | 481.2 | 16.7791816 | | | | | | | | | | | | | | |
| 481.3 | 6.76658988 | 481.3 | 16.7693749 | | | | | | | | | | | | | | |
| 481.4 | 6.77639653 | 481.4 | 16.7595683 | | | | | | | | | | | | | | |
| 481.5 | 6.79600984 | 481.5 | 16.7595683 | | | | | | | | | | | | | | |
| 481.6 | 6.77639653 | 481.6 | 16.7595683 | | | | | | | | | | | | | | |
| 481.7 | 6.77639653 | 481.7 | 16.7791816 | | | | | | | | | | | | | | |
| 481.8 | 6.80581649 | 481.8 | 16.7987949 | | | | | | | | | | | | | | |
| 481.9 | 6.79600984 | 481.9 | 16.7987949 | | | | | | | | | | | | | | |
| 482 | 6.78620318 | 482 | 16.7791816 | | | | | | | | | | | | | | |
| 482.1 | 6.79600984 | 482.1 | 16.7497616 | | | | | | | | | | | | | | |
| 482.2 | 6.79600984 | 482.2 | 16.7497616 | | | | | | | | | | | | | | |
| 482.3 | 6.79600984 | 482.3 | 16.7595683 | | | | | | | | | | | | | | |
| 482.4 | 6.80581649 | 482.4 | 16.7693749 | | | | | | | | | | | | | | |
| 482.5 | 6.82542979 | 482.5 | 16.7497616 | | | | | | | | | | | | | | |
| 482.6 | 6.82542979 | 482.6 | 16.7497616 | | | | | | | | | | | | | | |
| 482.7 | 6.83523644 | 482.7 | 16.7595683 | | | | | | | | | | | | | | |
| 482.8 | 6.85484975 | 482.8 | 16.7693749 | | | | | | | | | | | | | | |
| 482.9 | 6.89407636 | 482.9 | 16.7791816 | | | | | | | | | | | | | | |
| 483 | 6.90388301 | 483 | 16.7889882 | | | | | | | | | | | | | | |
| 483.1 | 6.91368966 | 483.1 | 16.7889882 | | | | | | | | | | | | | | |
| 483.2 | 6.89407636 | 483.2 | 16.7595683 | | | | | | | | | | | | | | |
| 483.3 | 6.89407636 | 483.3 | 16.7497616 | | | | | | | | | | | | | | |
| 483.4 | 6.91368966 | 483.4 | 16.7497616 | | | | | | | | | | | | | | |
| 483.5 | 6.92349631 | 483.5 | 16.7693749 | | | | | | | | | | | | | | |
| 483.6 | 6.87446305 | 483.6 | 16.7693749 | | | | | | | | | | | | | | |
| 483.7 | 6.85484975 | 483.7 | 16.7497616 | | | | | | | | | | | | | | |
| 483.8 | 6.8646564 | 483.8 | 16.7595683 | | | | | | | | | | | | | | |
| 483.9 | 6.89407636 | 483.9 | 16.7595683 | | | | | | | | | | | | | | |
| 484 | 6.92349631 | 484 | 16.739955 | | | | | | | | | | | | | | |
| 484.1 | 6.91368966 | 484.1 | 16.7497616 | | | | | | | | | | | | | | |
| 484.2 | 6.89407636 | 484.2 | 16.7693749 | | | | | | | | | | | | | | |
| 484.3 | 6.91368966 | 484.3 | 16.7595683 | | | | | | | | | | | | | | |
| 484.4 | 6.90388301 | 484.4 | 16.7595683 | | | | | | | | | | | | | | |
| 484.5 | 6.91368966 | 484.5 | 16.7693749 | | | | | | | | | | | | | | |
| 484.6 | 6.93330296 | 484.6 | 16.7693749 | | | | | | | | | | | | | | |
| 484.7 | 6.92349631 | 484.7 | 16.7301483 | | | | | | | | | | | | | | |
| 484.8 | 6.94310962 | 484.8 | 16.7203417 | | | | | | | | | | | | | | |
| 484.9 | 6.94310962 | 484.9 | 16.7301483 | | | | | | | | | | | | | | |
| 485 | 6.91368966 | 485 | 16.7203417 | | | | | | | | | | | | | | |
| 485.1 | 6.89407636 | 485.1 | 16.7203417 | | | | | | | | | | | | | | |
| 485.2 | 6.92349631 | 485.2 | 16.7301483 | | | | | | | | | | | | | | |

| | | | | | | | | | | | | | | | | | |
|---|---|---|---|---|---|---|---|---|---|---|---|---|---|---|---|---|---|
| 485.3 | 6.90388301 | 485.3 | 16.739955 | | | | | | | | | | | | | | |
| 485.4 | 6.90388301 | 485.4 | 16.739955 | | | | | | | | | | | | | | |
| 485.5 | 6.91368966 | 485.5 | 16.7497616 | | | | | | | | | | | | | | |
| 485.6 | 6.91368966 | 485.6 | 16.7497616 | | | | | | | | | | | | | | |
| 485.7 | 6.90388301 | 485.7 | 16.7497616 | | | | | | | | | | | | | | |
| 485.8 | 6.89407636 | 485.8 | 16.7203417 | | | | | | | | | | | | | | |
| 485.9 | 6.92349631 | 485.9 | 16.710535 | | | | | | | | | | | | | | |
| 486 | 6.93330296 | 486 | 16.7301483 | | | | | | | | | | | | | | |
| 486.1 | 6.93330296 | 486.1 | 16.739955 | | | | | | | | | | | | | | |
| 486.2 | 6.95291627 | 486.2 | 16.739955 | | | | | | | | | | | | | | |
| 486.3 | 6.95291627 | 486.3 | 16.7301483 | | | | | | | | | | | | | | |
| 486.4 | 6.94310962 | 486.4 | 16.710535 | | | | | | | | | | | | | | |
| 486.5 | 6.95291627 | 486.5 | 16.7301483 | | | | | | | | | | | | | | |
| 486.6 | 6.95291627 | 486.6 | 16.7203417 | | | | | | | | | | | | | | |
| 486.7 | 6.93330296 | 486.7 | 16.710535 | | | | | | | | | | | | | | |
| 486.8 | 6.92349631 | 486.8 | 16.710535 | | | | | | | | | | | | | | |
| 486.9 | 6.92349631 | 486.9 | 16.710535 | | | | | | | | | | | | | | |
| 487 | 6.93330296 | 487 | 16.7007284 | | | | | | | | | | | | | | |
| 487.1 | 6.95291627 | 487.1 | 16.710535 | | | | | | | | | | | | | | |
| 487.2 | 6.93330296 | 487.2 | 16.7203417 | | | | | | | | | | | | | | |
| 487.3 | 6.92349631 | 487.3 | 16.7301483 | | | | | | | | | | | | | | |
| 487.4 | 6.94310962 | 487.4 | 16.739955 | | | | | | | | | | | | | | |
| 487.5 | 6.98233622 | 487.5 | 16.739955 | | | | | | | | | | | | | | |
| 487.6 | 6.95291627 | 487.6 | 16.739955 | | | | | | | | | | | | | | |
| 487.7 | 6.94310962 | 487.7 | 16.7497616 | | | | | | | | | | | | | | |
| 487.8 | 6.95291627 | 487.8 | 16.7595683 | | | | | | | | | | | | | | |
| 487.9 | 6.96272292 | 487.9 | 16.7497616 | | | | | | | | | | | | | | |
| 488 | 6.93330296 | 488 | 16.7497616 | | | | | | | | | | | | | | |
| 488.1 | 6.94310962 | 488.1 | 16.739955 | | | | | | | | | | | | | | |
| 488.2 | 6.95291627 | 488.2 | 16.7301483 | | | | | | | | | | | | | | |
| 488.3 | 6.95291627 | 488.3 | 16.7497616 | | | | | | | | | | | | | | |
| 488.4 | 6.94310962 | 488.4 | 16.7203417 | | | | | | | | | | | | | | |
| 488.5 | 6.97252957 | 488.5 | 16.710535 | | | | | | | | | | | | | | |
| 488.6 | 6.96272292 | 488.6 | 16.7007284 | | | | | | | | | | | | | | |
| 488.7 | 6.96272292 | 488.7 | 16.6909217 | | | | | | | | | | | | | | |
| 488.8 | 6.96272292 | 488.8 | 16.710535 | | | | | | | | | | | | | | |
| 488.9 | 6.97252957 | 488.9 | 16.739955 | | | | | | | | | | | | | | |
| 489 | 6.98233622 | 489 | 16.7203417 | | | | | | | | | | | | | | |
| 489.1 | 6.99214288 | 489.1 | 16.710535 | | | | | | | | | | | | | | |
| 489.2 | 6.96272292 | 489.2 | 16.710535 | | | | | | | | | | | | | | |
| 489.3 | 6.97252957 | 489.3 | 16.7203417 | | | | | | | | | | | | | | |
| 489.4 | 6.95291627 | 489.4 | 16.710535 | | | | | | | | | | | | | | |
| 489.5 | 6.96272292 | 489.5 | 16.7007284 | | | | | | | | | | | | | | |
| 489.6 | 6.95291627 | 489.6 | 16.6713084 | | | | | | | | | | | | | | |
| 489.7 | 6.94310962 | 489.7 | 16.6615017 | | | | | | | | | | | | | | |
| 489.8 | 6.94310962 | 489.8 | 16.6811151 | | | | | | | | | | | | | | |
| 489.9 | 6.98233622 | 489.9 | 16.7007284 | | | | | | | | | | | | | | |
| 490 | 7.00194953 | 490 | 16.7007284 | | | | | | | | | | | | | | |
| 490.1 | 6.99214288 | 490.1 | 16.710535 | | | | | | | | | | | | | | |
| 490.2 | 6.98233622 | 490.2 | 16.7203417 | | | | | | | | | | | | | | |
| 490.3 | 6.95291627 | 490.3 | 16.710535 | | | | | | | | | | | | | | |
| 490.4 | 6.97252957 | 490.4 | 16.6811151 | | | | | | | | | | | | | | |
| 490.5 | 6.99214288 | 490.5 | 16.6811151 | | | | | | | | | | | | | | |
| 490.6 | 6.98233622 | 490.6 | 16.7007284 | | | | | | | | | | | | | | |
| 490.7 | 7.00194953 | 490.7 | 16.7007284 | | | | | | | | | | | | | | |
| 490.8 | 7.01175618 | 490.8 | 16.7007284 | | | | | | | | | | | | | | |
| 490.9 | 7.01175618 | 490.9 | 16.7007284 | | | | | | | | | | | | | | |
| 491 | 7.00194953 | 491 | 16.6909217 | | | | | | | | | | | | | | |
| 491.1 | 6.99214288 | 491.1 | 16.6909217 | | | | | | | | | | | | | | |
| 491.2 | 6.97252957 | 491.2 | 16.6713084 | | | | | | | | | | | | | | |
| 491.3 | 6.99214288 | 491.3 | 16.6713084 | | | | | | | | | | | | | | |

| | | | | | | | | | | | | | | | | | |
|---|---|---|---|---|---|---|---|---|---|---|---|---|---|---|---|---|---|
| 491.4 | 7.02156283 | 491.4 | 16.6811151 | | | | | | | | | | | | | | |
| 491.5 | 6.99214288 | 491.5 | 16.7007284 | | | | | | | | | | | | | | |
| 491.6 | 6.99214288 | 491.6 | 16.6909217 | | | | | | | | | | | | | | |
| 491.7 | 6.99214288 | 491.7 | 16.6713084 | | | | | | | | | | | | | | |
| 491.8 | 7.00194953 | 491.8 | 16.6811151 | | | | | | | | | | | | | | |
| 491.9 | 7.02156283 | 491.9 | 16.6713084 | | | | | | | | | | | | | | |
| 492 | 7.03136948 | 492 | 16.7007284 | | | | | | | | | | | | | | |
| 492.1 | 7.05098279 | 492.1 | 16.6909217 | | | | | | | | | | | | | | |
| 492.2 | 7.04117614 | 492.2 | 16.6713084 | | | | | | | | | | | | | | |
| 492.3 | 7.01175618 | 492.3 | 16.6909217 | | | | | | | | | | | | | | |
| 492.4 | 7.01175618 | 492.4 | 16.6909217 | | | | | | | | | | | | | | |
| 492.5 | 7.03136948 | 492.5 | 16.6909217 | | | | | | | | | | | | | | |
| 492.6 | 7.03136948 | 492.6 | 16.6615017 | | | | | | | | | | | | | | |
| 492.7 | 7.02156283 | 492.7 | 16.6516951 | | | | | | | | | | | | | | |
| 492.8 | 7.05098279 | 492.8 | 16.6615017 | | | | | | | | | | | | | | |
| 492.9 | 7.06078944 | 492.9 | 16.6516951 | | | | | | | | | | | | | | |
| 493 | 7.05098279 | 493 | 16.6516951 | | | | | | | | | | | | | | |
| 493.1 | 7.04117614 | 493.1 | 16.6615017 | | | | | | | | | | | | | | |
| 493.2 | 7.02156283 | 493.2 | 16.6713084 | | | | | | | | | | | | | | |
| 493.3 | 7.04117614 | 493.3 | 16.6811151 | | | | | | | | | | | | | | |
| 493.4 | 7.05098279 | 493.4 | 16.6811151 | | | | | | | | | | | | | | |
| 493.5 | 7.06078944 | 493.5 | 16.6615017 | | | | | | | | | | | | | | |
| 493.6 | 7.05098279 | 493.6 | 16.6516951 | | | | | | | | | | | | | | |
| 493.7 | 7.02156283 | 493.7 | 16.6615017 | | | | | | | | | | | | | | |
| 493.8 | 7.06078944 | 493.8 | 16.6615017 | | | | | | | | | | | | | | |
| 493.9 | 7.04117614 | 493.9 | 16.6615017 | | | | | | | | | | | | | | |
| 494 | 7.05098279 | 494 | 16.6516951 | | | | | | | | | | | | | | |
| 494.1 | 7.07059609 | 494.1 | 16.6811151 | | | | | | | | | | | | | | |
| 494.2 | 7.05098279 | 494.2 | 16.6516951 | | | | | | | | | | | | | | |
| 494.3 | 7.07059609 | 494.3 | 16.6320818 | | | | | | | | | | | | | | |
| 494.4 | 7.05098279 | 494.4 | 16.6222751 | | | | | | | | | | | | | | |
| 494.5 | 7.05098279 | 494.5 | 16.6418884 | | | | | | | | | | | | | | |
| 494.6 | 7.06078944 | 494.6 | 16.6320818 | | | | | | | | | | | | | | |
| 494.7 | 7.06078944 | 494.7 | 16.6320818 | | | | | | | | | | | | | | |
| 494.8 | 7.06078944 | 494.8 | 16.6516951 | | | | | | | | | | | | | | |
| 494.9 | 7.07059609 | 494.9 | 16.6615017 | | | | | | | | | | | | | | |
| 495 | 7.08040274 | 495 | 16.6713084 | | | | | | | | | | | | | | |
| 495.1 | 7.08040274 | 495.1 | 16.6516951 | | | | | | | | | | | | | | |
| 495.2 | 7.10001605 | 495.2 | 16.6615017 | | | | | | | | | | | | | | |
| 495.3 | 7.08040274 | 495.3 | 16.6418884 | | | | | | | | | | | | | | |
| 495.4 | 7.07059609 | 495.4 | 16.6222751 | | | | | | | | | | | | | | |
| 495.5 | 7.08040274 | 495.5 | 16.6222751 | | | | | | | | | | | | | | |
| 495.6 | 7.08040274 | 495.6 | 16.6320818 | | | | | | | | | | | | | | |
| 495.7 | 7.0902094 | 495.7 | 16.6418884 | | | | | | | | | | | | | | |
| 495.8 | 7.10001605 | 495.8 | 16.6320818 | | | | | | | | | | | | | | |
| 495.9 | 7.10001605 | 495.9 | 16.6320818 | | | | | | | | | | | | | | |
| 496 | 7.08040274 | 496 | 16.6222751 | | | | | | | | | | | | | | |
| 496.1 | 7.1098227 | 496.1 | 16.6320818 | | | | | | | | | | | | | | |
| 496.2 | 7.05098279 | 496.2 | 16.6320818 | | | | | | | | | | | | | | |
| 496.3 | 7.0902094 | 496.3 | 16.6418884 | | | | | | | | | | | | | | |
| 496.4 | 7.14904931 | 496.4 | 16.6811151 | | | | | | | | | | | | | | |
| 496.5 | 7.11962935 | 496.5 | 16.6418884 | | | | | | | | | | | | | | |
| 496.6 | 7.08040274 | 496.6 | 16.6320818 | | | | | | | | | | | | | | |
| 496.7 | 7.10001605 | 496.7 | 16.6320818 | | | | | | | | | | | | | | |
| 496.8 | 7.10001605 | 496.8 | 16.6320818 | | | | | | | | | | | | | | |
| 496.9 | 7.0902094 | 496.9 | 16.6320818 | | | | | | | | | | | | | | |
| 497 | 7.1098227 | 497 | 16.6418884 | | | | | | | | | | | | | | |
| 497.1 | 7.10001605 | 497.1 | 16.6222751 | | | | | | | | | | | | | | |
| 497.2 | 7.08040274 | 497.2 | 16.6320818 | | | | | | | | | | | | | | |
| 497.3 | 7.0902094 | 497.3 | 16.6615017 | | | | | | | | | | | | | | |
| 497.4 | 7.13924266 | 497.4 | 16.6418884 | | | | | | | | | | | | | | |

| | | | | | | | | | | | | | | | | | |
|---|---|---|---|---|---|---|---|---|---|---|---|---|---|---|---|---|---|
| 497.5 | 7.14904931 | 497.5 | 16.6026618 | | | | | | | | | | | | | | |
| 497.6 | 7.129436 | 497.6 | 16.6124685 | | | | | | | | | | | | | | |
| 497.7 | 7.11962935 | 497.7 | 16.6418884 | | | | | | | | | | | | | | |
| 497.8 | 7.1098227 | 497.8 | 16.6418884 | | | | | | | | | | | | | | |
| 497.9 | 7.129436 | 497.9 | 16.6516951 | | | | | | | | | | | | | | |
| 498 | 7.13924266 | 498 | 16.6516951 | | | | | | | | | | | | | | |
| 498.1 | 7.14904931 | 498.1 | 16.6418884 | | | | | | | | | | | | | | |
| 498.2 | 7.129436 | 498.2 | 16.6222751 | | | | | | | | | | | | | | |
| 498.3 | 7.13924266 | 498.3 | 16.6516951 | | | | | | | | | | | | | | |
| 498.4 | 7.11962935 | 498.4 | 16.6320818 | | | | | | | | | | | | | | |
| 498.5 | 7.13924266 | 498.5 | 16.6222751 | | | | | | | | | | | | | | |
| 498.6 | 7.14904931 | 498.6 | 16.6124685 | | | | | | | | | | | | | | |
| 498.7 | 7.16866261 | 498.7 | 16.6418884 | | | | | | | | | | | | | | |
| 498.8 | 7.15885596 | 498.8 | 16.6418884 | | | | | | | | | | | | | | |
| 498.9 | 7.15885596 | 498.9 | 16.6124685 | | | | | | | | | | | | | | |
| 499 | 7.15885596 | 499 | 16.6222751 | | | | | | | | | | | | | | |
| 499.1 | 7.13924266 | 499.1 | 16.6222751 | | | | | | | | | | | | | | |
| 499.2 | 7.13924266 | 499.2 | 16.5830485 | | | | | | | | | | | | | | |
| 499.3 | 7.14904931 | 499.3 | 16.5830485 | | | | | | | | | | | | | | |
| 499.4 | 7.15885596 | 499.4 | 16.6026618 | | | | | | | | | | | | | | |
| 499.5 | 7.16866261 | 499.5 | 16.6124685 | | | | | | | | | | | | | | |
| 499.6 | 7.18827592 | 499.6 | 16.6124685 | | | | | | | | | | | | | | |
| 499.7 | 7.14904931 | 499.7 | 16.6026618 | | | | | | | | | | | | | | |
| 499.8 | 7.13924266 | 499.8 | 16.6124685 | | | | | | | | | | | | | | |
| 499.9 | 7.14904931 | 499.9 | 16.6320818 | | | | | | | | | | | | | | |
| 500 | 7.15885596 | 500 | 16.6026618 | | | | | | | | | | | | | | |
| 500.1 | 7.14904931 | 500.1 | 16.5830485 | | | | | | | | | | | | | | |
| 500.2 | 7.15885596 | 500.2 | 16.5830485 | | | | | | | | | | | | | | |
| 500.3 | 7.13924266 | 500.3 | 16.6026618 | | | | | | | | | | | | | | |
| 500.4 | 7.14904931 | 500.4 | 16.5928552 | | | | | | | | | | | | | | |
| 500.5 | 7.16866261 | 500.5 | 16.5732419 | | | | | | | | | | | | | | |
| 500.6 | 7.15885596 | 500.6 | 16.5830485 | | | | | | | | | | | | | | |
| 500.7 | 7.18827592 | 500.7 | 16.6222751 | | | | | | | | | | | | | | |
| 500.8 | 7.16866261 | 500.8 | 16.6222751 | | | | | | | | | | | | | | |
| 500.9 | 7.16866261 | 500.9 | 16.6124685 | | | | | | | | | | | | | | |
| 501 | 7.18827592 | 501 | 16.6026618 | | | | | | | | | | | | | | |
| 501.1 | 7.18827592 | 501.1 | 16.5830485 | | | | | | | | | | | | | | |
| 501.2 | 7.17846926 | 501.2 | 16.5928552 | | | | | | | | | | | | | | |
| 501.3 | 7.16866261 | 501.3 | 16.5928552 | | | | | | | | | | | | | | |
| 501.4 | 7.17846926 | 501.4 | 16.6124685 | | | | | | | | | | | | | | |
| 501.5 | 7.18827592 | 501.5 | 16.5928552 | | | | | | | | | | | | | | |
| 501.6 | 7.19808257 | 501.6 | 16.6026618 | | | | | | | | | | | | | | |
| 501.7 | 7.16866261 | 501.7 | 16.6026618 | | | | | | | | | | | | | | |
| 501.8 | 7.17846926 | 501.8 | 16.5732419 | | | | | | | | | | | | | | |
| 501.9 | 7.18827592 | 501.9 | 16.5732419 | | | | | | | | | | | | | | |
| 502 | 7.16866261 | 502 | 16.5536286 | | | | | | | | | | | | | | |
| 502.1 | 7.22750252 | 502.1 | 16.5732419 | | | | | | | | | | | | | | |
| 502.2 | 7.18827592 | 502.2 | 16.5830485 | | | | | | | | | | | | | | |
| 502.3 | 7.17846926 | 502.3 | 16.5732419 | | | | | | | | | | | | | | |
| 502.4 | 7.20788922 | 502.4 | 16.5732419 | | | | | | | | | | | | | | |
| 502.5 | 7.21769587 | 502.5 | 16.5438219 | | | | | | | | | | | | | | |
| 502.6 | 7.21769587 | 502.6 | 16.5536286 | | | | | | | | | | | | | | |
| 502.7 | 7.19808257 | 502.7 | 16.5830485 | | | | | | | | | | | | | | |
| 502.8 | 7.22750252 | 502.8 | 16.5928552 | | | | | | | | | | | | | | |
| 502.9 | 7.22750252 | 502.9 | 16.5732419 | | | | | | | | | | | | | | |
| 503 | 7.22750252 | 503 | 16.5634352 | | | | | | | | | | | | | | |
| 503.1 | 7.22750252 | 503.1 | 16.5634352 | | | | | | | | | | | | | | |
| 503.2 | 7.20788922 | 503.2 | 16.5634352 | | | | | | | | | | | | | | |
| 503.3 | 7.19808257 | 503.3 | 16.5634352 | | | | | | | | | | | | | | |
| 503.4 | 7.19808257 | 503.4 | 16.5732419 | | | | | | | | | | | | | | |
| 503.5 | 7.21769587 | 503.5 | 16.5732419 | | | | | | | | | | | | | | |

| | | | | | | | | | | | | | | | | | |
|---|---|---|---|---|---|---|---|---|---|---|---|---|---|---|---|---|---|
| 503.6 | 7.22750252 | 503.6 | 16.5634352 | | | | | | | | | | | | | | |
| 503.7 | 7.23730918 | 503.7 | 16.5634352 | | | | | | | | | | | | | | |
| 503.8 | 7.24711583 | 503.8 | 16.5634352 | | | | | | | | | | | | | | |
| 503.9 | 7.23730918 | 503.9 | 16.5732419 | | | | | | | | | | | | | | |
| 504 | 7.24711583 | 504 | 16.5732419 | | | | | | | | | | | | | | |
| 504.1 | 7.23730918 | 504.1 | 16.5438219 | | | | | | | | | | | | | | |
| 504.2 | 7.23730918 | 504.2 | 16.5634352 | | | | | | | | | | | | | | |
| 504.3 | 7.23730918 | 504.3 | 16.5928552 | | | | | | | | | | | | | | |
| 504.4 | 7.22750252 | 504.4 | 16.5732419 | | | | | | | | | | | | | | |
| 504.5 | 7.22750252 | 504.5 | 16.5732419 | | | | | | | | | | | | | | |
| 504.6 | 7.24711583 | 504.6 | 16.5928552 | | | | | | | | | | | | | | |
| 504.7 | 7.23730918 | 504.7 | 16.6026618 | | | | | | | | | | | | | | |
| 504.8 | 7.22750252 | 504.8 | 16.6418884 | | | | | | | | | | | | | | |
| 504.9 | 7.25692248 | 504.9 | 16.6615017 | | | | | | | | | | | | | | |
| 505 | 7.25692248 | 505 | 16.6615017 | | | | | | | | | | | | | | |
| 505.1 | 7.25692248 | 505.1 | 16.7007284 | | | | | | | | | | | | | | |
| 505.2 | 7.27653578 | 505.2 | 16.5438219 | | | | | | | | | | | | | | |
| 505.3 | 7.27653578 | 505.3 | 14.6119115 | | | | | | | | | | | | | | |
| 505.4 | 7.27653578 | 505.4 | 16.3574955 | | | | | | | | | | | | | | |
| 505.5 | 7.27653578 | 505.5 | 19.4563976 | | | | | | | | | | | | | | |
| 505.6 | 7.30595574 | 505.6 | 19.9761501 | | | | | | | | | | | | | | |
| 505.7 | 7.27653578 | 505.7 | 20.5841625 | | | | | | | | | | | | | | |
| 505.8 | 7.28634244 | 505.8 | 20.7312623 | | | | | | | | | | | | | | |
| 505.9 | 7.29614909 | 505.9 | 20.1624765 | | | | | | | | | | | | | | |
| 506 | 7.29614909 | 506 | 20.2703497 | | | | | | | | | | | | | | |
| 506.1 | 7.31576239 | 506.1 | 20.2997696 | | | | | | | | | | | | | | |
| 506.2 | 7.31576239 | 506.2 | 20.3095763 | | | | | | | | | | | | | | |
| 506.3 | 7.29614909 | 506.3 | 20.4468694 | | | | | | | | | | | | | | |
| 506.4 | 7.28634244 | 506.4 | 20.5351293 | | | | | | | | | | | | | | |
| 506.5 | 7.29614909 | 506.5 | 20.5743559 | | | | | | | | | | | | | | |
| 506.6 | 7.29614909 | 506.6 | 20.5743559 | | | | | | | | | | | | | | |
| 506.7 | 7.29614909 | 506.7 | 20.5743559 | | | | | | | | | | | | | | |
| 506.8 | 7.30595574 | 506.8 | 20.5645492 | | | | | | | | | | | | | | |
| 506.9 | 7.3353757 | 506.9 | 20.5057093 | | | | | | | | | | | | | | |
| 507 | 7.31576239 | 507 | 20.4762894 | | | | | | | | | | | | | | |
| 507.1 | 7.32556904 | 507.1 | 15.6318033 | | | | | | | | | | | | | | |
| 507.2 | 7.31576239 | 507.2 | 12.258315 | | | | | | | | | | | | | | |
| 507.3 | 7.30595574 | 507.3 | 19.0935514 | | | | | | | | | | | | | | |
| 507.4 | 7.28634244 | 507.4 | 15.8181297 | | | | | | | | | | | | | | |
| 507.5 | 7.27653578 | 507.5 | 7.8649349 | | | | | | | | | | | | | | |
| 507.6 | 7.28634244 | 507.6 | 13.7293128 | | | | | | | | | | | | | | |
| 507.7 | 7.29614909 | 507.7 | 19.4858175 | | | | | | | | | | | | | | |
| 507.8 | 7.31576239 | 507.8 | 20.2409297 | | | | | | | | | | | | | | |
| 507.9 | 7.31576239 | 507.9 | 20.3389962 | | | | | | | | | | | | | | |
| 508 | 7.32556904 | 508 | 20.2115098 | | | | | | | | | | | | | | |
| 508.1 | 7.36479565 | 508.1 | 20.3684162 | | | | | | | | | | | | | | |
| 508.2 | 7.34518235 | 508.2 | 20.4664827 | | | | | | | | | | | | | | |
| 508.3 | 7.354989 | 508.3 | 20.4468694 | | | | | | | | | | | | | | |
| 508.4 | 7.354989 | 508.4 | 20.4174495 | | | | | | | | | | | | | | |
| 508.5 | 7.3746023 | 508.5 | 20.4272561 | | | | | | | | | | | | | | |
| 508.6 | 7.354989 | 508.6 | 20.4174495 | | | | | | | | | | | | | | |
| 508.7 | 7.36479565 | 508.7 | 20.4174495 | | | | | | | | | | | | | | |
| 508.8 | 7.354989 | 508.8 | 20.4174495 | | | | | | | | | | | | | | |
| 508.9 | 7.36479565 | 508.9 | 20.4566761 | | | | | | | | | | | | | | |
| 509 | 7.3353757 | 509 | 20.4762894 | | | | | | | | | | | | | | |
| 509.1 | 7.354989 | 509.1 | 20.5057093 | | | | | | | | | | | | | | |
| 509.2 | 7.36479565 | 509.2 | 20.5057093 | | | | | | | | | | | | | | |
| 509.3 | 7.3746023 | 509.3 | 20.3291896 | | | | | | | | | | | | | | |
| 509.4 | 7.3746023 | 509.4 | 20.8489422 | | | | | | | | | | | | | | |
| 509.5 | 7.3746023 | 509.5 | 21.0548818 | | | | | | | | | | | | | | |
| 509.6 | 7.38440896 | 509.6 | 21.1333351 | | | | | | | | | | | | | | |

| | | | | | | | | | | | | | | | | | |
|---|---|---|---|---|---|---|---|---|---|---|---|---|---|---|---|---|---|
| 509.7 | 7.38440896 | 509.7 | 21.1039151 | | | | | | | | | | | | | | |
| 509.8 | 7.36479565 | 509.8 | 21.0744951 | | | | | | | | | | | | | | |
| 509.9 | 7.3746023 | 509.9 | 21.0744951 | | | | | | | | | | | | | | |
| 510 | 7.41382891 | 510 | 21.0254619 | | | | | | | | | | | | | | |
| 510.1 | 7.39421561 | 510.1 | 20.9764286 | | | | | | | | | | | | | | |
| 510.2 | 7.40402226 | 510.2 | 21.0156552 | | | | | | | | | | | | | | |
| 510.3 | 7.39421561 | 510.3 | 21.0352685 | | | | | | | | | | | | | | |
| 510.4 | 7.38440896 | 510.4 | 21.0254619 | | | | | | | | | | | | | | |
| 510.5 | 7.39421561 | 510.5 | 21.0156552 | | | | | | | | | | | | | | |
| 510.6 | 7.39421561 | 510.6 | 21.0352685 | | | | | | | | | | | | | | |
| 510.7 | 7.39421561 | 510.7 | 21.0941085 | | | | | | | | | | | | | | |
| 510.8 | 7.38440896 | 510.8 | 21.1039151 | | | | | | | | | | | | | | |
| 510.9 | 7.41382891 | 510.9 | 21.1039151 | | | | | | | | | | | | | | |
| 511 | 7.43344222 | 511 | 21.1529484 | | | | | | | | | | | | | | |
| 511.1 | 7.39421561 | 511.1 | 20.741069 | | | | | | | | | | | | | | |
| 511.2 | 7.3746023 | 511.2 | 19.7898237 | | | | | | | | | | | | | | |
| 511.3 | 7.40402226 | 511.3 | 20.2311231 | | | | | | | | | | | | | | |
| 511.4 | 7.44324887 | 511.4 | 20.0251834 | | | | | | | | | | | | | | |
| 511.5 | 7.45305552 | 511.5 | 19.9957634 | | | | | | | | | | | | | | |
| 511.6 | 7.43344222 | 511.6 | 19.9173102 | | | | | | | | | | | | | | |
| 511.7 | 7.41382891 | 511.7 | 19.5642707 | | | | | | | | | | | | | | |
| 511.8 | 7.43344222 | 511.8 | 17.8186867 | | | | | | | | | | | | | | |
| 511.9 | 7.40402226 | 511.9 | 12.513288 | | | | | | | | | | | | | | |
| 512 | 7.40402226 | 512 | 17.2302876 | | | | | | | | | | | | | | |
| 512.1 | 7.38440896 | 512.1 | 20.1722832 | | | | | | | | | | | | | | |
| 512.2 | 7.40402226 | 512.2 | 20.4076428 | | | | | | | | | | | | | | |
| 512.3 | 7.42363556 | 512.3 | 20.3291896 | | | | | | | | | | | | | | |
| 512.4 | 7.40402226 | 512.4 | 20.3095763 | | | | | | | | | | | | | | |
| 512.5 | 7.40402226 | 512.5 | 20.3488029 | | | | | | | | | | | | | | |
| 512.6 | 7.42363556 | 512.6 | 20.3684162 | | | | | | | | | | | | | | |
| 512.7 | 7.42363556 | 512.7 | 20.3880295 | | | | | | | | | | | | | | |
| 512.8 | 7.44324887 | 512.8 | 20.1820898 | | | | | | | | | | | | | | |
| 512.9 | 7.42363556 | 512.9 | 19.9663435 | | | | | | | | | | | | | | |
| 513 | 7.43344222 | 513 | 20.06441 | | | | | | | | | | | | | | |
| 513.1 | 7.41382891 | 513.1 | 20.1330566 | | | | | | | | | | | | | | |
| 513.2 | 7.42363556 | 513.2 | 20.1820898 | | | | | | | | | | | | | | |
| 513.3 | 7.45305552 | 513.3 | 20.2213164 | | | | | | | | | | | | | | |
| 513.4 | 7.45305552 | 513.4 | 20.2115098 | | | | | | | | | | | | | | |
| 513.5 | 7.42363556 | 513.5 | 20.2801563 | | | | | | | | | | | | | | |
| 513.6 | 7.46286217 | 513.6 | 20.3488029 | | | | | | | | | | | | | | |
| 513.7 | 7.48247548 | 513.7 | 20.1428632 | | | | | | | | | | | | | | |
| 513.8 | 7.47266882 | 513.8 | 20.1036366 | | | | | | | | | | | | | | |
| 513.9 | 7.43344222 | 513.9 | 20.0447967 | | | | | | | | | | | | | | |
| 514 | 7.47266882 | 514 | 20.1722832 | | | | | | | | | | | | | | |
| 514.1 | 7.46286217 | 514.1 | 20.2017031 | | | | | | | | | | | | | | |
| 514.2 | 7.49228213 | 514.2 | 20.1722832 | | | | | | | | | | | | | | |
| 514.3 | 7.47266882 | 514.3 | 20.0840233 | | | | | | | | | | | | | | |
| 514.4 | 7.45305552 | 514.4 | 20.0251834 | | | | | | | | | | | | | | |
| 514.5 | 7.45305552 | 514.5 | 20.0546033 | | | | | | | | | | | | | | |
| 514.6 | 7.47266882 | 514.6 | 20.1134433 | | | | | | | | | | | | | | |
| 514.7 | 7.46286217 | 514.7 | 20.1820898 | | | | | | | | | | | | | | |
| 514.8 | 7.47266882 | 514.8 | 20.0546033 | | | | | | | | | | | | | | |
| 514.9 | 7.50208878 | 514.9 | 19.2406512 | | | | | | | | | | | | | | |
| 515 | 7.52170208 | 515 | 19.0052916 | | | | | | | | | | | | | | |
| 515.1 | 7.51189543 | 515.1 | 19.3485244 | | | | | | | | | | | | | | |
| 515.2 | 7.47266882 | 515.2 | 19.838857 | | | | | | | | | | | | | | |
| 515.3 | 7.46286217 | 515.3 | 20.03499 | | | | | | | | | | | | | | |
| 515.4 | 7.49228213 | 515.4 | 20.3095763 | | | | | | | | | | | | | | |
| 515.5 | 7.47266882 | 515.5 | 19.9173102 | | | | | | | | | | | | | | |
| 515.6 | 7.48247548 | 515.6 | 19.5642707 | | | | | | | | | | | | | | |
| 515.7 | 7.47266882 | 515.7 | 19.7211772 | | | | | | | | | | | | | | |

| | | | |
|---|---|---|---|
| 515.8 | 7.47266882 | 515.8 | 19.5348508 |
| 515.9 | 7.50208878 | 515.9 | 19.191618 |
| 516 | 7.48247548 | 516 | 19.3092978 |
| 516.1 | 7.48247548 | 516.1 | 19.191618 |
| 516.2 | 7.48247548 | 516.2 | 19.3681377 |
| 516.3 | 7.52170208 | 516.3 | 19.6623373 |
| 516.4 | 7.51189543 | 516.4 | 19.868277 |
| 516.5 | 7.54131539 | 516.5 | 19.7800171 |
| 516.6 | 7.54131539 | 516.6 | 19.5740774 |
| 516.7 | 7.52170208 | 516.7 | 19.6034973 |
| 516.8 | 7.52170208 | 516.8 | 19.6034973 |
| 516.9 | 7.52170208 | 516.9 | 19.6034973 |
| 517 | 7.54131539 | 517 | 17.0537678 |
| 517.1 | 7.55112204 | 517.1 | 13.9352525 |
| 517.2 | 7.53150874 | 517.2 | 13.5429864 |
| 517.3 | 7.53150874 | 517.3 | 19.3387177 |
| 517.4 | 7.54131539 | 517.4 | 19.5348508 |
| 517.5 | 7.56092869 | 517.5 | 18.9758716 |
| 517.6 | 7.54131539 | 517.6 | 19.1229714 |
| 517.7 | 7.54131539 | 517.7 | 19.4269776 |
| 517.8 | 7.52170208 | 517.8 | 19.8486636 |
| 517.9 | 7.53150874 | 517.9 | 19.8976969 |
| 518 | 7.56092869 | 518 | 19.0445182 |
| 518.1 | 7.54131539 | 518.1 | 18.6718654 |
| 518.2 | 7.54131539 | 518.2 | 19.5740774 |
| 518.3 | 7.56092869 | 518.3 | 19.7604038 |
| 518.4 | 7.57073534 | 518.4 | 19.7015639 |
| 518.5 | 7.56092869 | 518.5 | 20.1624765 |
| 518.6 | 7.580542 | 518.6 | 19.5446574 |
| 518.7 | 7.6001553 | 518.7 | 13.1114937 |
| 518.8 | 7.59034865 | 518.8 | 8.14932781 |
| 518.9 | 7.580542 | 518.9 | 7.3746023 |
| 519 | 7.580542 | 519 | 4.51105992 |
| 519.1 | 7.57073534 | 519.1 | 5.54075838 |
| 519.2 | 7.56092869 | 519.2 | 5.76631138 |
| 519.3 | 7.6001553 | 519.3 | 4.38357344 |
| 519.4 | 7.580542 | 519.4 | 5.60940494 |
| 519.5 | 7.580542 | 519.5 | 4.45222001 |
| 519.6 | 7.580542 | 519.6 | 2.9616089 |
| 519.7 | 7.56092869 | 519.7 | 2.93218895 |
| 519.8 | 7.580542 | 519.8 | 2.9223823 |
| 519.9 | 7.6001553 | 519.9 | 2.9223823 |
| 520 | 7.57073534 | 520 | 2.9223823 |
| 520.1 | 7.57073534 | 520.1 | 2.9223823 |
| 520.2 | 7.6001553 | 520.2 | 2.93218895 |
| 520.3 | 7.60996195 | 520.3 | 2.93218895 |
| 520.4 | 7.59034865 | 520.4 | 2.9223823 |
| 520.5 | 7.6001553 | 520.5 | 2.30456322 |
| 520.6 | 7.60996195 | 520.6 | 0.44129934 |
| 520.7 | 7.59034865 | 520.7 | -0.16671308 |
| 520.8 | 7.6001553 | 520.8 | -0.225553 |
| 520.9 | 7.60996195 | 520.9 | -0.16671308 |
| 521 | 7.62957526 | 521 | -0.15690643 |
| 521.1 | 7.60996195 | 521.1 | -0.23535965 |
| 521.2 | 7.6001553 | 521.2 | -0.2451663 |
| 521.3 | 7.6001553 | 521.3 | -0.2451663 |
| 521.4 | 7.6197686 | 521.4 | -0.20593969 |
| 521.5 | 7.60996195 | 521.5 | -0.25497295 |
| 521.6 | 7.59034865 | 521.6 | -0.2451663 |
| 521.7 | 7.6001553 | 521.7 | -0.25497295 |
| 521.8 | 7.62957526 | 521.8 | -0.25497295 |

| | | | |
|---|---|---|---|
| 521.9 | 7.6197686 | 521.9 | -0.29419956 |
| 522 | 7.6197686 | 522 | -0.55897916 |
| 522.1 | 7.64918856 | 522.1 | -1.12776498 |
| 522.2 | 7.64918856 | 522.2 | -0.4903326 |
| 522.3 | 7.63938191 | 522.3 | -0.14709978 |
| 522.4 | 7.64918856 | 522.4 | -0.15690643 |
| 522.5 | 7.65899521 | 522.5 | -0.18632639 |
| 522.6 | 7.65899521 | 522.6 | -0.19613304 |
| 522.7 | 7.6197686 | 522.7 | -0.2451663 |
| 522.8 | 7.62957526 | 522.8 | -0.21574634 |
| 522.9 | 7.62957526 | 522.9 | -0.16671308 |
| 523 | 7.63938191 | 523 | -0.19613304 |
| 523.1 | 7.66880186 | 523.1 | -0.25497295 |
| 523.2 | 7.65899521 | 523.2 | -0.2647796 |
| 523.3 | 7.63938191 | 523.3 | -0.19613304 |
| 523.4 | 7.63938191 | 523.4 | -0.13729313 |
| 523.5 | 7.65899521 | 523.5 | -0.15690643 |
| 523.6 | 7.66880186 | 523.6 | -0.15690643 |
| 523.7 | 7.66880186 | 523.7 | -0.14709978 |
| 523.8 | 7.65899521 | 523.8 | -0.18632639 |
| 523.9 | 7.66880186 | 523.9 | -0.23535965 |
| 524 | 7.67860852 | 524 | -0.23535965 |
| 524.1 | 7.67860852 | 524.1 | -0.15690643 |
| 524.2 | 7.65899521 | 524.2 | -0.16671308 |
| 524.3 | 7.65899521 | 524.3 | -0.225553 |
| 524.4 | 7.68841517 | 524.4 | -0.2647796 |
| 524.5 | 7.67860852 | 524.5 | -0.2647796 |
| 524.6 | 7.68841517 | 524.6 | -0.225553 |
| 524.7 | 7.66880186 | 524.7 | -0.20593969 |
| 524.8 | 7.70802847 | 524.8 | -0.23535965 |
| 524.9 | 7.70802847 | 524.9 | 0.08825987 |
| 525 | 7.71783512 | 525 | 0.91201864 |
| 525.1 | 7.72764178 | 525.1 | 0.83356542 |
| 525.2 | 7.72764178 | 525.2 | 0.60801242 |
| 525.3 | 7.68841517 | 525.3 | 0.14709978 |
| 525.4 | 7.68841517 | 525.4 | -0.27458626 |
| 525.5 | 7.72764178 | 525.5 | -0.28439291 |
| 525.6 | 7.70802847 | 525.6 | -0.30400621 |
| 525.7 | 7.69822182 | 525.7 | -0.34323282 |
| 525.8 | 7.68841517 | 525.8 | -0.35303947 |
| 525.9 | 7.67860852 | 525.9 | -0.36284612 |

## Regeneration of Actuation Power in Yeast-Driven Inflated Membrane Actuators:

| Inflatable membrane actuator | | | | | | | | | | | |
|---|---|---|---|---|---|---|---|---|---|---|---|
| 12% | | | | | | | | | | | |
| T1 | | | T2 | | | T3 | | | T4 | | |
| t (s) | Y (mm) | F (kHz) | t (s) | Y (mm) | F (kHz) | t (s) | Y (mm) | F (kHz) | t (s) | Y (mm) | F (kHz) |
| 0.0000 | 0.0000 | 2.968 | 0.0000 | 0.0000 | 2.2840 | 0.0000 | 0.0000 | 2.2990 | 0.0000 | 0.0000 | 2.7740 |
| 225.8167 | 2.5000 | 3.487 | 209.3667 | 3.3957 | 2.8960 | 239.6833 | 3.3284 | 3.1550 | 181.7167 | 4.9552 | 3.5990 |
| 261.3667 | 2.9522 | 3.538 | 248.8167 | 5.7728 | 3.1490 | 254.3333 | 4.0906 | 3.2150 | 196.6000 | 6.3088 | 3.7080 |
| 287.3167 | 4.3016 | 3.827 | 260.6000 | 6.7066 | 3.2280 | 282.2833 | 6.4749 | 3.3550 | 206.0167 | 7.3594 | 3.7800 |
| 303.6667 | 5.5670 | 3.84 | 278.9500 | 7.9800 | 3.3590 | 296.9333 | 7.8850 | 3.4270 | 211.2333 | 7.9611 | 3.8380 |
| 318.0667 | 6.6631 | 3.849 | 293.3500 | 8.4045 | 3.4630 | 315.5833 | 8.4259 | 3.5410 | 213.3500 | 8.5611 | 3.8520 |
| 326.7167 | 7.4227 | 3.857 | 309.0667 | 9.0836 | 3.545 | 323.5667 | 8.9711 | 3.585 | 215.4167 | 8.5611 | 3.87 |
| 328.6167 | 6.9166 | 3.811 | 314.1667 | 8.8289 | 3.5590 | 325.1167 | 8.8607 | 3.5970 | 216.5333 | 7.3603 | 3.8590 |
| 328.9333 | 6.3264 | 3.814 | 314.6333 | 7.9800 | 3.5810 | 325.2667 | 8.3184 | 3.5910 | 216.7833 | 5.8564 | 3.8650 |
| 329.3000 | 5.9044 | 3.836 | 314.8667 | 7.2159 | 3.5980 | 325.4500 | 7.3422 | 3.6110 | 216.8000 | 6.4590 | 3.8900 |
| 329.6167 | 5.3981 | 3.84 | 315.0667 | 5.6030 | 3.6150 | 325.7333 | 6.1495 | 3.6170 | 216.8167 | 5.7080 | 3.9040 |
| 330.1000 | 4.7233 | 3.855 | 315.2500 | 3.4806 | 3.6300 | 325.8500 | 4.5220 | 3.6300 | 217.0167 | 4.5054 | 3.9110 |
| 332.5167 | 2.3620 | 3.9 | 315.3667 | 2.8015 | 3.6260 | 326.0000 | 2.8936 | 3.6720 | 217.0500 | 4.2041 | 3.9170 |

| | | | | | | | | | | | |
|---|---|---|---|---|---|---|---|---|---|---|---|
| 353.9667 | 3.2898 | 4.061 | 315.6667 | 1.8677 | 3.677 | 326.4667 | 0.0773 | 3.755 | 217.3167 | 2.7038 | 3.944 |
| 356.3000 | 3.5422 | 4.063 | 391.5667 | 2.9713 | 3.9670 | 403.4667 | 2.7880 | 3.8630 | 242.6167 | 4.8067 | 4.1470 |
| 387.2500 | 4.7235 | 4.065 | 411.2167 | 4.4145 | 4.0050 | 430.0833 | 4.5225 | 3.8000 | 261.4333 | 6.7603 | 4.2920 |
| 409.3500 | 5.7357 | 4.074 | 442.6333 | 6.6217 | 4.0970 | 446.0667 | 5.7142 | 3.8240 | 273.9833 | 8.5619 | 4.3780 |
| 420.8833 | 6.8322 | 4.079 | 480.6167 | 8.5742 | 4.1650 | 467.3667 | 7.5591 | 3.9010 | 279.2167 | 9.3130 | 4.4210 |
| 442.9833 | 8.0977 | 4.111 | 534.3167 | 9.9325 | 4.1770 | 491.3500 | 9.1866 | 3.9460 | 293.8500 | 10.8151 | 4.5590 |
| 444.0333 | 8.2660 | 4.114 | 606.3333 | 11.3757 | 4.2620 | 537.9500 | 10.8126 | 3.9770 | 302.2167 | 11.7155 | 4.7550 |
| 450.0667 | 7.9288 | 4.248 | 677.0500 | 12.2247 | 4.546 | 701.7333 | 13.7436 | 4.297 | 307.2500 | 10.6649 | 5.119 |
| 450.5000 | 7.3385 | 4.133 | 692.7667 | 11.1211 | 5.0350 | 751.8167 | 12.8745 | 4.6570 | 307.6500 | 9.6134 | 5.2480 |
| 451.0000 | 6.9164 | 4.195 | 694.5500 | 10.1872 | 5.0450 | 753.6167 | 11.8939 | 4.7390 | 308.0167 | 9.1619 | 5.1680 |
| 452.1167 | 5.3983 | 4.212 | 696.2667 | 9.5930 | 5.0750 | 756.7333 | 10.1652 | 4.6740 | 308.4333 | 7.9593 | 5.1860 |
| 452.5667 | 4.9764 | 4.322 | 697.1667 | 8.2347 | 5.2500 | 757.6500 | 9.5130 | 4.8880 | 308.9500 | 6.4590 | 5.2260 |
| 453.1833 | 4.2174 | 4.339 | 697.7833 | 7.0462 | 5.1530 | 758.6333 | 7.3422 | 4.8500 | 309.2833 | 5.2591 | 5.2420 |
| 454.0167 | 3.4581 | 4.282 | 698.6333 | 4.6691 | 5.1660 | 758.8500 | 6.1510 | 4.7450 | 309.7167 | 4.2067 | 5.3370 |
| 455.7000 | 2.6990 | 4.209 | 700.0167 | 2.2921 | 5.369 | 760.2833 | 2.2486 | 5.07 | 310.4833 | 2.2523 | 5.456 |
| 469.8833 | 3.4581 | 4.232 | 775.2833 | 3.1411 | 5.2730 | 792.2667 | 3.3323 | 4.8720 | 328.3667 | 4.0548 | 5.4940 |
| 492.9500 | 4.3861 | 4.248 | 790.9833 | 3.3957 | 5.2670 | 808.2500 | 3.8751 | 5.0080 | 347.1833 | 5.4075 | 5.3440 |
| 512.1667 | 5.1453 | 4.241 | 804.0833 | 3.9900 | 5.3250 | 844.2000 | 5.6072 | 5.1160 | 364.9667 | 7.2109 | 5.3460 |
| 528.5000 | 6.0731 | 4.281 | 814.5667 | 4.4145 | 5.4420 | 881.4833 | 7.6710 | 5.1420 | 383.7833 | 9.1628 | 5.3270 |
| 565.9833 | 7.8442 | 4.299 | 819.8000 | 4.7540 | 5.5520 | 916.1000 | 8.9706 | 5.2540 | 407.8333 | 11.4150 | 5.3210 |
| 594.8000 | 8.9408 | 4.405 | 831.5833 | 5.1785 | 5.5530 | 945.4000 | 9.8374 | 5.4840 | 426.6500 | 12.0176 | 5.4530 |
| 616.9000 | 9.6158 | 4.592 | 840.7500 | 5.6879 | 5.554 | 961.3833 | 10.3802 | 5.565 | 524.9500 | 14.8707 | 5.39 |
| 663.0333 | 8.5190 | 4.979 | 848.6167 | 5.0936 | 5.8040 | 974.4500 | 9.9468 | 5.8430 | 580.6167 | 13.0674 | 5.8500 |
| 663.5667 | 7.7601 | 4.917 | 849.0833 | 4.5843 | 5.8130 | 975.0833 | 9.4045 | 5.9690 | 582.7333 | 11.5653 | 6.0280 |
| 663.9167 | 6.7477 | 4.778 | 849.4000 | 4.1598 | 5.9460 | 975.8500 | 8.5348 | 5.9110 | 583.6000 | 10.3653 | 5.9130 |
| 664.1333 | 5.6512 | 4.959 | 849.8333 | 3.6504 | 5.9910 | 976.5000 | 7.6695 | 5.8800 | 584.4667 | 9.0117 | 6.0310 |
| 664.4167 | 4.7235 | 4.907 | 850.1833 | 3.3108 | 5.8660 | 977.4833 | 5.4987 | 5.9580 | 585.2833 | 7.5096 | 6.0350 |
| 664.7833 | 3.7115 | 4.993 | 850.6667 | 2.7166 | 5.9240 | 977.6667 | 4.5230 | 5.9240 | 586.2833 | 6.0084 | 6.1530 |
| 665.7000 | 2.1083 | 5.086 | 851.8167 | 1.7828 | 6.042 | 978.2500 | 2.1392 | 6.032 | 587.9500 | 3.4548 | 6.288 |
| 712.0333 | 3.1211 | 5.088 | 895.7500 | 2.2921 | 5.9220 | 997.3333 | 3.1124 | 6.0180 | 608.6167 | 4.5072 | 5.8800 |
| 734.1333 | 3.4581 | 5.129 | 923.2500 | 2.7166 | 5.9580 | 1055.9170 | 3.9807 | 6.0030 | 632.6667 | 6.0075 | 5.8950 |
| 789.8667 | 4.6390 | 5.215 | 944.2167 | 3.3108 | 5.8730 | 1082.5500 | 5.7152 | 6.1710 | 679.7167 | 8.7104 | 5.9060 |
| 818.7000 | 5.6512 | 5.221 | 973.0167 | 3.9900 | 5.4180 | 1114.5000 | 7.0177 | 6.2290 | 706.9167 | 10.3636 | 6.0110 |
| 841.7667 | 6.5792 | 5.219 | 997.9000 | 5.0087 | 5.5200 | 1166.4330 | 9.1866 | 6.3180 | 744.5667 | 11.5670 | 6.0140 |
| 884.0500 | 7.9288 | 5.398 | 1035.8830 | 6.2821 | 5.6620 | 1227.6830 | 10.3817 | 5.9680 | 799.9833 | 13.0691 | 6.1060 |
| 952.2667 | 8.9406 | 5.485 | 1058.1500 | 7.1311 | 5.803 | 1319.5670 | 11.4673 | 6.379 | 952.3480 | 14.9530 | 6.286 |
| | | | 1059.2830 | 6.9613 | 5.7440 | | | | | | |
| | | | 1065.4170 | 5.3483 | 5.9630 | | | | | | |
| | | | 1065.7000 | 5.0936 | 6.0120 | | | | | | |
| | | | 1065.9330 | 4.1598 | 6.1100 | | | | | | |
| | | | 1066.1170 | 3.1411 | 6.1710 | | | | | | |
| | | | 1066.4500 | 2.2921 | 6.0760 | | | | | | |
| | | | 1066.7500 | 1.7828 | 6.219 | | | | | | |
| | | | 1068.1170 | 2.4619 | 6.1980 | | | | | | |
| | | | 1105.2830 | 3.3957 | 6.0060 | | | | | | |
| | | | 1153.7500 | 4.8389 | 6.0900 | | | | | | |
| | | | 1194.3500 | 6.1972 | 6.0790 | | | | | | |
| | | | 1229.7000 | 7.0462 | 6.2960 | | | | | | |
| | | | 1246.7330 | 7.8102 | 6.3490 | | | | | | |
| | | | 1250.7000 | 7.6404 | 6.384 | | | | | | |
| | | | 1287.3170 | 6.5368 | 6.7070 | | | | | | |
| | | | 1288.6330 | 2.7166 | 6.8330 | | | | | | |
| | | | 1291.2500 | 1.2734 | 6.9740 | | | | | | |

## Soft limb motion:

| T-20 room | | | | | | | T-20 room | | | | | | | T-20 room | | | | | | |
|---|---|---|---|---|---|---|---|---|---|---|---|---|---|---|---|---|---|---|---|---|
| T2 | | | | | | | T5 | | | | | | | T6 | | | | | | |
| t (s) | x (mm) | y (mm) | angle (degree) | angle(rad) | dA/dt | F (Khz) | t (s) | x (mm) | y (mm) | angle (degree) | angle(rad) | dA/dt | F (Khz) | t (s) | x (mm) | y (mm) | angle (degree) | angle(rad) | dA/dt | F (Khz) |
| 0.0000 | 44.9823 | 29.0993 | 32.8991 | 0.57419777 | 0.04834839 | 1.7230 | 0.0000 | 46.1104 | 31.2282 | 34.1078 | 0.5953 | 0.0238 | 1.7400 | 0.0000 | 47.6157 | 25.0690 | 27.7662 | 0.4846 | 0.0325 | 1.7930 |
| 96.6500 | 43.1798 | 33.2193 | 37.5720 | 0.65575476 | 0.13981696 | 1.8440 | 74.7000 | 45.8664 | 33.1800 | 35.8821 | 0.6263 | 0.1084 | 1.8030 | 95.8167 | 47.3649 | 28.3303 | 30.8849 | 0.5390 | 0.1517 | 1.8990 |
| 135.7833 | 41.6348 | 38.8842 | 43.0435 | 0.75125043 | 0.33417223 | 1.9640 | 182.6333 | 43.9147 | 48.0622 | 47.5819 | 0.8305 | 0.5606 | 2.1010 | 222.6833 | 44.6053 | 53.4172 | 50.1369 | 0.8751 | 0.4576 | 1.9740 |
| 173.4167 | 33.6524 | 49.1841 | 55.6195 | 0.97074369 | 0.76801437 | 2.1160 | 212.3000 | 31.4722 | 65.1401 | 64.2127 | 1.1207 | 0.8048 | 2.2460 | 240.9000 | 37.0792 | 60.4415 | 58.4720 | 1.0205 | 1.0439 | 2.9640 |

| | | | | | | |
|---|---|---|---|---|---|---|
| 182.8333 | 27.7300 | 54.0765 | 62.8516 | 1.09696737 | 1.31506023 | 2.1770 |
| 194.3500 | 12.5377 | 58.9689 | 77.9968 | 1.36130017 | 2.13658796 | 2.2410 |
| 200.1167 | -0.3371 | 60.7714 | 90.3178 | -1.56524939 | 2.44045418 | 2.2740 |
| 205.8667 | -14.7569 | 57.6814 | 104.3504 | -1.32033411 | 1.62317343 | 2.2960 |
| 211.0167 | -22.7393 | 54.3340 | 112.7098 | -1.17443605 | 1.2004476 | 2.3280 |
| 216.4833 | -28.1467 | 50.2140 | 119.2721 | -1.05990114 | 0.70609621 | 2.3700 |
| 231.6333 | -35.6141 | 42.4891 | 129.9695 | -0.87319703 | 0.52740477 | 2.4080 |
| 239.8333 | -38.1891 | 39.1417 | 134.2942 | -0.79771644 | 0.33450689 | 2.4260 |
| 250.7500 | -40.2490 | 36.3092 | 137.9459 | -0.73398206 | 0.30723104 | 2.4860 |
| 273.8167 | -43.0815 | 30.1293 | 145.0327 | -0.61029395 | 0.03208227 | 2.7230 |
| 300.4500 | -43.3390 | 29.3568 | 145.8872 | -0.59538087 | 0.48556229 | 2.7500 |

| | | | | | | |
|---|---|---|---|---|---|---|
| 216.7667 | 27.5686 | 67.5798 | 67.8074 | 1.1835 | 1.3603 | 2.2510 |
| 221.8667 | 19.0297 | 69.7755 | 74.7449 | 1.3045 | 1.7530 | 2.2770 |
| 231.7667 | -2.6837 | 73.1911 | 92.0999 | -1.5341 | 1.1844 | 2.3200 |
| 241.3167 | -16.3460 | 68.5557 | 103.4109 | -1.3367 | 0.7398 | 2.3750 |
| 245.4500 | -19.7616 | 66.8479 | 106.4688 | -1.2834 | 3.4006 | 2.3560 |
| 245.7667 | -20.9815 | 66.3599 | 107.5457 | -1.2646 | 0.5948 | 2.3820 |
| 262.0333 | -30.4963 | 59.2848 | 117.2214 | -1.0957 | 0.3935 | 2.4380 |
| 267.7833 | -32.6920 | 57.8210 | 119.4839 | -1.0562 | 0.3323 | 2.4600 |
| 275.1000 | -34.6438 | 55.6252 | 121.9149 | -1.0138 | 0.4188 | 2.5240 |
| 280.2000 | -36.1076 | 53.4295 | 124.0508 | -0.9765 | 0.1919 | 2.5500 |
| 315.9000 | -39.5232 | 45.6224 | 130.9027 | -0.8569 | 0.4144 | 2.9330 |

| | | | | | | |
|---|---|---|---|---|---|---|
| 248.8833 | 29.5532 | 68.9710 | 66.8055 | 1.1660 | 0.8340 | 3.1800 |
| 249.5167 | 27.5462 | 65.9606 | 67.3338 | 1.1752 | 1.4530 | 3.2220 |
| 266.4500 | -2.5341 | 74.8706 | 91.9385 | -1.5370 | 1.2328 | 3.4050 |
| 274.1667 | -14.5134 | 71.6454 | 101.4516 | -1.3709 | 0.6935 | 3.5170 |
| 291.0500 | -27.1432 | 63.4519 | 113.1600 | -1.1666 | 0.9915 | 3.4880 |
| 292.5333 | -29.2571 | 63.8128 | 114.6307 | -1.1409 | 0.2887 | 3.6940 |
| 298.7333 | -30.4045 | 61.1941 | 116.4206 | -1.1097 | 0.7473 | 3.7410 |
| 302.2833 | -33.1734 | 59.6661 | 119.0734 | -1.0634 | 0.3244 | 3.8710 |
| 308.3167 | -34.7860 | 57.8232 | 121.0308 | -1.0292 | 0.3072 | 3.9570 |
| 315.1833 | -36.3986 | 55.7498 | 123.1402 | -0.9924 | -0.1912 | 3.9940 |
| 316.6167 | -36.1683 | 55.9802 | 122.8661 | -0.9972 | 0.3881 | 3.9650 |

| T-40 room | | | | | | |
|---|---|---|---|---|---|---|
| T4 | | | | | | |
| t (s) | x (mm) | y (mm) | angle (degree) | angle(rad) | dA/dt | F (Khz) |
| 0.0000 | 44.2205 | 41.1969 | 42.9727 | 0.7500 | 0.0378 | 1.7000 |
| 40.1833 | 43.0866 | 42.3307 | 44.4930 | 0.7765 | 0.1139 | 1.7770 |
| 61.9833 | 42.3307 | 45.3543 | 46.9749 | 0.8199 | 0.1809 | 1.8440 |
| 93.4000 | 38.9291 | 51.0236 | 52.6577 | 0.9191 | 0.2121 | 1.9950 |
| 102.4500 | 38.1732 | 53.6693 | 54.5770 | 0.9525 | 0.5946 | 2.0290 |
| 130.1833 | 22.2992 | 65.0079 | 71.0668 | 1.2404 | 1.4612 | 2.2260 |
| 136.7000 | 11.3386 | 68.4095 | 80.5890 | 1.4065 | 2.3841 | 2.2730 |
| 140.3833 | 0.7559 | 68.7874 | 89.3704 | 1.5598 | 2.2362 | 2.3150 |
| 145.7500 | -13.6063 | 67.6535 | 101.3715 | -1.3723 | 1.5646 | 2.3670 |
| 150.8500 | -22.2992 | 63.4961 | 109.3508 | -1.2331 | 0.9562 | 2.4020 |
| 159.3333 | -29.8583 | 57.4488 | 117.4626 | -1.0915 | 0.8104 | 2.4720 |
| 164.1500 | -33.6378 | 55.1811 | 121.3660 | -1.0234 | 0.4672 | 2.4990 |
| 172.1500 | -36.6614 | 52.1575 | 125.1033 | -0.9581 | 0.2435 | 2.5520 |
| 200.0833 | -39.6850 | 44.2205 | 131.9059 | -0.8394 | 0.1130 | 2.7090 |
| 282.4500 | -42.3307 | 34.0158 | 141.2156 | -0.6769 | 0.5000 | 3.9860 |

| T-40 room | | | | | | |
|---|---|---|---|---|---|---|
| T3 | | | | | | |
| t (s) | x (mm) | y (mm) | angle (degree) | angle(rad) | dA/dt | F (Khz) |
| 0.0000 | 54.8032 | 33.6378 | 31.5413 | 0.5505 | 0.1108 | 1.9020 |
| 64.2667 | 54.8032 | 43.8425 | 38.6598 | 0.6747 | 0.2413 | 2.0410 |
| 89.6833 | 52.5354 | 52.1575 | 44.7932 | 0.7818 | 0.3825 | 2.1610 |
| 100.6833 | 50.2677 | 57.8268 | 49.0002 | 0.8552 | 0.5284 | 2.2010 |
| 121.9833 | 39.3071 | 68.7874 | 60.2551 | 1.0517 | 1.0124 | 2.3340 |
| 130.9333 | 27.9685 | 74.0787 | 69.3159 | 1.2098 | 1.4717 | 2.4300 |
| 137.4500 | 15.1181 | 77.1024 | 78.9063 | 1.3772 | 1.6552 | 2.4300 |
| 143.3000 | 1.8898 | 76.7244 | 88.5891 | 1.5462 | 1.0660 | 2.4740 |
| 159.7833 | -19.2756 | 66.5197 | 106.1601 | -1.2887 | 0.4561 | 2.5600 |
| 166.3167 | -22.2992 | 64.2520 | 109.1398 | -1.2367 | 0.5354 | 2.6070 |
| 170.9500 | -24.5669 | 61.9843 | 111.6205 | -1.1934 | 0.2587 | 2.6490 |
| 206.6000 | -31.3701 | 52.5354 | 120.8424 | -1.0325 | 0.1370 | 3.0500 |
| 220.1000 | -32.5039 | 50.6457 | 122.6920 | -1.0002 | 0.1165 | 3.1510 |
| 259.2000 | -34.7717 | 45.7323 | 127.2468 | -0.9207 | 0.0861 | 3.8920 |
| 342.9500 | -39.6850 | 40.4409 | 134.4595 | -0.7948 | 0.3921 | 4.3500 |

| T-40 room | | | | | | |
|---|---|---|---|---|---|---|
| T1 | | | | | | |
| t (s) | x (mm) | y (mm) | angle (degree) | angle(rad) | dA/dt | F (Khz) |
| 0.0000 | 45.2361 | 39.3782 | 41.0397 | 0.7163 | -0.0246 | 1.7400 |
| 27.4500 | 45.5615 | 38.7273 | 40.3645 | 0.7045 | 0.0776 | 1.7520 |
| 100.5333 | 44.2597 | 45.8869 | 46.0341 | 0.8034 | 0.2540 | 1.9280 |
| 147.8333 | 37.7510 | 60.5317 | 58.0500 | 1.0132 | 0.6960 | 2.1440 |
| 171.4333 | 19.5264 | 70.2949 | 74.4759 | 1.2998 | 0.6464 | 2.2810 |
| 172.7000 | 18.8755 | 71.9221 | 75.2947 | 1.3141 | 1.2300 | 2.2790 |
| 175.7000 | 14.1658 | 72.7733 | 78.9848 | 1.3785 | 1.6250 | 2.3010 |
| 181.0500 | 2.9290 | 72.2475 | 87.6785 | 1.5303 | 2.0734 | 2.3260 |
| 187.8833 | -13.9939 | 66.7150 | 101.8464 | -1.3640 | 1.0223 | 2.3430 |
| 197.2000 | -23.4316 | 59.8808 | 111.3706 | -1.1978 | 0.5343 | 2.3800 |
| 225.1500 | -35.1474 | 47.8396 | 126.3045 | -0.9372 | 0.1456 | 2.5590 |
| 259.3167 | -37.4255 | 42.6325 | 131.2787 | -0.8503 | 0.0392 | 2.7170 |
| 275.4667 | -37.1001 | 41.3308 | 131.9123 | -0.8393 | 0.0851 | 2.9680 |
| 284.1667 | -38.0764 | 41.3308 | 132.6531 | -0.8264 | 0.0687 | 2.9960 |
| 307.7667 | -38.0764 | 39.0527 | 134.2748 | -0.7981 | 0.4363 | 3.1790 |

| T-80 | | | | | | |
|---|---|---|---|---|---|---|
| t (s) | x (mm) | y (mm) | angle (degree) | angle(rad) | dA/dt | F (Khz) |
| 0.0000 | 42.0554 | 31.4917 | 36.8265 | 0.6427 | 0.1594 | 2.0530 |
| 8.8667 | 40.9215 | 32.2477 | 38.2394 | 0.6674 | 0.0694 | 2.0260 |
| 110.4667 | 38.6538 | 39.0508 | 45.2927 | 0.7905 | 0.3335 | 2.1300 |
| 119.6000 | 36.7640 | 41.3185 | 48.3382 | 0.8437 | 0.2708 | 2.1910 |
| 132.6833 | 34.4963 | 43.9642 | 51.8806 | 0.9055 | 0.5443 | 2.2850 |
| 148.9500 | 28.4491 | 50.7673 | 60.7345 | 1.0600 | 1.3752 | 2.4040 |
| 159.0667 | 15.5987 | 56.8146 | 74.6475 | 1.3028 | 2.7802 | 2.4870 |
| 164.8167 | -0.6533 | 59.0823 | 90.6335 | -1.5597 | 3.9603 | 2.5420 |
| 167.7667 | -11.9919 | 54.9248 | 102.3163 | -1.3558 | 3.0262 | 2.5540 |
| 171.5667 | -22.5746 | 51.1453 | 113.8157 | -1.1551 | 2.0757 | 2.5740 |
| 174.9667 | -27.8659 | 46.6099 | 120.8733 | -1.0320 | 1.0299 | 2.6220 |
| 179.7500 | -30.8895 | 42.8303 | 125.7994 | -0.9460 | 1.7360 | 2.6430 |
| 183.2500 | -34.6690 | 38.6729 | 131.8753 | -0.8399 | 0.9269 | 2.6770 |

| T-80 | | | | | | |
|---|---|---|---|---|---|---|
| t (s) | x (mm) | y (mm) | angle (degree) | angle(rad) | dA/dt | F (Khz) |
| 0.0000 | 50.4376 | 30.1142 | 30.8397 | 0.5383 | 0.1869 | 2.2790 |
| 62.1500 | 47.7919 | 43.7205 | 42.4526 | 0.7409 | 0.0196 | 2.3110 |
| 125.8667 | 46.8799 | 44.8055 | 43.7039 | 0.7628 | 0.5096 | 2.4570 |
| 134.7000 | 44.5984 | 49.8898 | 48.2052 | 0.8413 | 0.7678 | 2.6720 |
| 137.7667 | 42.3163 | 51.4434 | 50.5599 | 0.8824 | 1.2035 | 2.8390 |
| 148.7000 | 30.2362 | 61.2284 | 63.7186 | 1.1121 | 2.3513 | 3.0490 |
| 154.6500 | 14.7402 | 67.6535 | 77.7086 | 1.3563 | 3.0047 | 3.1530 |
| 159.3833 | -2.2677 | 67.2756 | 91.9306 | -1.5371 | 3.0849 | 3.2460 |
| 164.7333 | -20.4095 | 61.2284 | 108.4349 | -1.2490 | 2.5423 | 3.4020 |
| 167.0000 | -26.8347 | 59.7165 | 114.1976 | -1.1485 | 1.3743 | 3.4680 |
| 171.2833 | -31.7480 | 54.8032 | 120.0842 | -1.0457 | 1.0284 | 3.4810 |
| 174.6000 | -34.0158 | 51.4016 | 123.4952 | -0.9862 | 0.4634 | 3.6110 |
| 178.7333 | -34.3937 | 48.3780 | 125.4104 | -0.9528 | 0.7865 | 3.7570 |

| T-80 | | | | | | |
|---|---|---|---|---|---|---|
| t (s) | x (mm) | y (mm) | angle (degree) | angle(rad) | dA/dt | F (Khz) |
| 0.0000 | 48.0569 | 25.8971 | 28.3195 | 0.4943 | 0.5112 | 1.8600 |
| 5.4667 | 45.4112 | 27.4089 | 31.1140 | 0.5430 | 0.0066 | 1.8260 |
| 35.6333 | 46.9230 | 28.5428 | 31.3117 | 0.5465 | 0.1279 | 1.8420 |
| 71.0667 | 45.7892 | 33.0782 | 35.8445 | 0.6256 | 0.2408 | 1.9470 |
| 104.2500 | 43.8994 | 42.1491 | 43.8347 | 0.7651 | 0.6303 | 2.1360 |
| 113.6167 | 40.4978 | 47.8184 | 49.7384 | 0.8681 | 0.7665 | 2.2190 |
| 131.3333 | 29.1593 | 58.0231 | 63.3184 | 1.1051 | 1.6983 | 2.3700 |
| 147.4167 | -0.6990 | 63.3145 | 90.6325 | -1.5598 | 2.9332 | 2.5470 |
| 150.1000 | -9.0140 | 60.2908 | 98.5032 | -1.4224 | 1.4920 | 2.5740 |
| 162.9000 | -26.7778 | 51.2200 | 117.6005 | -1.0891 | 0.8765 | 2.6690 |
| 169.1833 | -30.9352 | 47.4404 | 123.1078 | -0.9930 | 0.5282 | 2.8540 |
| 173.9000 | -32.0691 | 44.7948 | 125.5993 | -0.9495 | 0.5824 | 2.9740 |
| 185.5000 | -37.7384 | 41.3932 | 132.3556 | -0.8316 | 0.3219 | 3.3530 |

| | | | | | | | | | | | | | | | | | | | | |
|---|---|---|---|---|---|---|---|---|---|---|---|---|---|---|---|---|---|---|---|---|
| 186.7500 | -36.9368 | 36.7831 | 135.1194 | -0.7833 | 0.5162 | 2.7410 | 187.8500 | -38.5512 | 41.9528 | 132.5805 | -0.8276 | 0.2340 | 4.0360 | 189.4833 | -36.2266 | 37.9916 | 133.6377 | -0.8092 | 0.2330 | 3.3320 |
| 197.9333 | -39.2045 | 31.8697 | 140.8920 | -0.6826 | 0.7118 | 2.9160 | 205.2500 | -40.4409 | 38.1732 | 136.6523 | -0.7566 | 0.6658 | 4.1260 | 203.2167 | -38.4943 | 36.1019 | 136.8369 | -0.7533 | 0.6734 | 3.6330 |

| T-60 | | | | | | | T-60 | | | | | | | T-60 | | | | | | |
|---|---|---|---|---|---|---|---|---|---|---|---|---|---|---|---|---|---|---|---|---|
| t (s) | x (mm) | y (mm) | angle (degree) | angle(rad) | dA/dt | F (Khz) | t (s) | x (mm) | y (mm) | angle (degree) | angle(rad) | dA/dt | F (Khz) | t (s) | x (mm) | y (mm) | angle (degree) | angle(rad) | dA/dt | F (Khz) |
| 0.0000 | 40.7766 | 41.3420 | 45.3945 | 0.7923 | 0.1922 | 2.0020 | 0.0000 | 40.8189 | 47.6221 | 49.3987 | 0.8622 | 0.0458 | 2.1110 | 0.0000 | 45.3543 | 49.5118 | 47.5094 | 0.8292 | 0.0884 | 1.8290 |
| 24.5166 | 41.0880 | 49.1520 | 50.1065 | 0.8745 | 0.3140 | 2.1380 | 13.6000 | 41.1969 | 49.1339 | 50.0215 | 0.8730 | 0.0887 | 2.1530 | 12.3833 | 44.9764 | 51.0236 | 48.6044 | 0.8483 | 0.0824 | 1.8500 |
| 34.0666 | 41.5325 | 55.3262 | 53.1051 | 0.9269 | 0.2946 | 2.2410 | 30.2000 | 41.1969 | 51.7795 | 51.4935 | 0.8987 | 0.1184 | 2.1920 | 40.3333 | 44.2205 | 54.4252 | 50.9061 | 0.8885 | 0.1512 | 1.8960 |
| 41.4000 | 39.9360 | 57.6000 | 55.2651 | 0.9646 | 5.8345 | 2.2890 | 43.4833 | 41.1969 | 54.8032 | 53.0669 | 0.9262 | 0.1056 | 2.2440 | 58.2500 | 42.3307 | 57.4488 | 53.6156 | 0.9358 | 0.3245 | 1.9880 |
| 41.8833 | 37.7529 | 60.6176 | 58.0851 | 1.0138 | 0.2578 | 2.2860 | 79.7666 | 38.9291 | 59.7165 | 56.8997 | 0.9931 | 0.2421 | 2.4220 | 74.3333 | 38.1732 | 63.1181 | 58.8349 | 1.0269 | 0.8093 | 2.0840 |
| 45.5166 | 37.7529 | 62.8853 | 59.0217 | 1.0301 | 0.7983 | 2.3350 | 94.9666 | 36.6614 | 65.0079 | 60.5790 | 1.0573 | 0.4035 | 2.5160 | 93.9000 | 20.4095 | 74.4567 | 74.6711 | 1.3033 | 2.1601 | 2.2080 |
| 54.0666 | 30.5718 | 68.1766 | 65.8475 | 1.1493 | 1.8129 | 2.4220 | 119.2166 | 28.7244 | 80.5039 | 70.3632 | 1.2281 | 0.8255 | 2.7350 | 95.0000 | 17.3858 | 75.5906 | 77.0472 | 1.3447 | 1.8126 | 2.2690 |
| 64.6166 | 6.7608 | 76.8695 | 84.9737 | 1.4831 | 2.1565 | 2.5050 | 127.0166 | 20.0315 | 85.4173 | 76.8019 | 1.3404 | 1.2144 | 2.8840 | 101.2167 | 2.2677 | 77.1024 | 88.3153 | 1.5414 | 1.7405 | 2.3230 |
| 67.3500 | -1.1762 | 77.6255 | 90.8681 | -1.5556 | 1.7688 | 2.5040 | 139.9000 | -3.7795 | 88.4409 | 92.4470 | -1.5281 | 1.8071 | 3.2510 | 108.8833 | -14.7402 | 71.4331 | 101.6593 | -1.3673 | 1.1083 | 2.5320 |
| 84.0833 | -32.5463 | 55.3262 | 120.4666 | -1.0391 | 0.6459 | 2.5620 | 145.8000 | -19.2756 | 82.7717 | 103.1092 | -1.3420 | 1.1186 | 3.4250 | 114.7333 | -21.9213 | 66.8976 | 108.1431 | -1.2541 | 1.8771 | 2.6570 |
| 90.8000 | -35.5699 | 51.1688 | 124.8050 | -0.9633 | 0.3700 | 2.5900 | 152.7666 | -28.7244 | 75.2126 | 110.9023 | -1.2060 | 0.5818 | 3.5830 | 115.1000 | -22.2992 | 65.3858 | 108.8315 | -1.2421 | 0.7117 | 2.6700 |
| 99.7166 | -37.4597 | 47.7672 | 128.1040 | -0.9058 | 0.2734 | 2.6800 | 166.6833 | -36.6614 | 66.1417 | 118.9990 | -1.0647 | 0.2934 | 3.9030 | 120.7667 | -26.4567 | 62.7402 | 112.8645 | -1.1717 | 0.3880 | 2.8440 |
| 118.8166 | -39.3494 | 41.7199 | 133.3251 | -0.8146 | 0.2202 | 2.9910 | 181.2833 | -40.4409 | 61.6063 | 123.2826 | -0.9899 | 0.2818 | 4.0400 | 129.9167 | -28.7244 | 57.8268 | 116.4151 | -1.1098 | 0.4279 | 3.0320 |
| 126.0833 | -39.3494 | 39.4522 | 134.9253 | -0.7867 | 0.2549 | 3.1280 | 190.2000 | -40.0630 | 55.5591 | 125.7949 | -0.9461 | 0.0645 | 4.0440 | 146.9167 | -34.7717 | 52.1575 | 123.6901 | -0.9828 | 0.2156 | 3.3730 |
| 133.7333 | -40.1053 | 37.5625 | 136.8752 | -0.7527 | 1.0235 | 3.0840 | 204.8666 | -40.0630 | 53.6693 | 126.7406 | -0.9296 | 0.6186 | 4.1440 | 170.5000 | -37.0394 | 46.1102 | 128.7742 | -0.8941 | 0.7553 | 3.4700 |

## Tactile Sensor:

| T1 | | | | T2 | | | | T3 | | | | T4 | | | |
|---|---|---|---|---|---|---|---|---|---|---|---|---|---|---|---|
| time | force | pressure | frequency | time | force | pressure | frequency | time | force | pressure | frequency | time | force | pressure | frequency |
| 0 | 0 | 0 | 3.538 | 0 | 0 | 0 | 3.296 | 0 | 0 | 0 | 3.293 | 0 | 0 | 0 | 3.457 |
| 8.62 | 0 | 0.01 | 3.547 | 37.12 | 0 | 0.01 | 3.388 | 114.05 | 0 | 0.01 | 3.517 | 24.8 | 0 | 0.01 | 3.517 |
| 13.35 | 0 | 0.02 | 3.549 | 103.17 | 0 | 0.02 | 3.581 | 190.07 | 0 | 0.02 | 3.7 | 114 | 0 | 0.02 | 3.802 |
| 19.03 | 0 | 0.03 | 3.568 | 104.85 | 0 | 0.03 | 3.587 | 256.58 | 0 | 0.03 | 3.86 | 182 | 0 | 0.03 | 4.01 |
| 26.05 | 0 | 0.04 | 3.6 | 108.75 | 0 | 0.04 | 3.591 | 281.83 | 0 | 0.04 | 3.902 | 236 | 0 | 0.04 | 4.198 |
| 54.08 | 0 | 0.05 | 3.659 | 116.62 | 0 | 0.05 | 3.623 | 339.67 | 0 | 0.05 | 4.007 | 271 | 0 | 0.05 | 4.265 |
| 79.8 | 0 | 0.06 | 3.722 | 184.82 | 0 | 0.06 | 3.768 | 394.23 | 0 | 0.06 | 4.076 | 319 | 0 | 0.06 | 4.358 |
| 103.5 | 0 | 0.07 | 3.791 | 287.32 | 0 | 0.07 | 3.964 | 456.13 | 0 | 0.07 | 4.161 | 354 | 0 | 0.07 | 4.412 |
| 125.2 | 0 | 0.08 | 3.838 | 347.92 | 0 | 0.08 | 4.052 | 504.2 | 0 | 0.08 | 4.26 | 395 | 0 | 0.08 | 4.477 |
| 132.55 | 0 | 0.09 | 3.851 | 356.9 | 0 | 0.09 | 4.096 | 550.62 | 0 | 0.09 | 4.325 | 433 | 0 | 0.09 | 4.517 |
| 159.92 | 0 | 0.1 | 3.86 | 407.03 | 0.02 | 0.1 | 4.147 | 620.67 | 0 | 0.1 | 4.447 | 443 | 0.21 | 0.1 | 4.55 |
| 186.6 | 3.47 | 0.22 | 3.633 | 409.03 | 0.63 | 0.1 | 4.153 | 650.67 | 0.06 | 0.1 | 4.5 | 451 | 3.27 | 0.16 | 4.468 |
| 187.95 | 4.23 | 0.23 | 3.62 | 409.37 | 2.99 | 0.1 | 3.995 | 651.55 | 3.22 | 0.19 | 4.382 | 452 | 4.28 | 0.17 | 4.418 |
| 188.85 | 4.41 | 0.23 | 3.606 | 410.05 | 3.81 | 0.22 | 3.961 | 652.53 | 4.28 | 0.2 | 4.314 | 453 | 4.43 | 0.17 | 4.415 |
| 189.97 | 3.63 | 0.22 | 3.619 | 411.02 | 4.42 | 0.23 | 3.927 | 653.7 | 4.42 | 0.2 | 4.302 | 454 | 3.83 | 0.16 | 4.431 |
| 190.97 | 2.28 | 0.2 | 3.656 | 412.02 | 4.3 | 0.23 | 3.923 | 654.57 | 3.84 | 0.19 | 4.29 | 455 | 1.91 | 0.14 | 4.488 |
| 192.02 | 1.01 | 0.17 | 3.698 | 414.02 | 3.42 | 0.22 | 3.964 | 655.52 | 2.34 | 0.18 | 4.334 | 455 | 1.11 | 0.12 | 4.504 |
| 192.95 | 0.05 | 0.14 | 3.749 | 415.03 | 2.02 | 0.19 | 3.99 | 656.57 | 1.17 | 0.16 | 4.342 | 456 | 0.26 | 0.09 | 4.545 |
| 194.28 | 0.04 | 0.13 | 3.753 | 416.03 | 0.76 | 0.15 | 4.036 | 658.57 | 0.28 | 0.14 | 4.363 | 458 | 0.03 | 0.08 | 4.558 |
| 194.95 | 0.84 | 0.15 | 3.721 | 417.02 | 0.15 | 0.1 | 4.092 | 660.55 | 0.04 | 0.13 | 4.384 | 458 | 0.69 | 0.1 | 4.536 |
| 195.95 | 2.22 | 0.19 | 3.668 | 418.02 | 0.14 | 0.09 | 4.103 | 661.97 | 1.06 | 0.14 | 4.342 | 459 | 2.11 | 0.13 | 4.513 |
| 197 | 3.5 | 0.22 | 3.625 | 419.08 | 1.08 | 0.16 | 4.044 | 662.62 | 1.98 | 0.17 | 4.304 | 460 | 3.38 | 0.16 | 4.458 |
| 197.97 | 4.32 | 0.23 | 3.597 | 420.72 | 2.55 | 0.19 | 3.996 | 663.97 | 3.34 | 0.19 | 4.285 | 461 | 4.29 | 0.17 | 4.431 |
| 198.95 | 4.44 | 0.23 | 3.585 | 421.13 | 4.29 | 0.22 | 3.929 | 665.08 | 4.25 | 0.2 | 4.267 | 462 | 4.43 | 0.17 | 4.428 |
| 199.98 | 3.66 | 0.22 | 3.61 | 422.03 | 4.43 | 0.23 | 3.941 | 666.08 | 4.46 | 0.2 | 4.258 | 463 | 3.71 | 0.16 | 4.444 |
| 201 | 2.36 | 0.2 | 3.647 | 423.03 | 4.3 | 0.23 | 3.926 | 666.57 | 3.41 | 0.19 | 4.295 | 464 | 2.42 | 0.14 | 4.485 |
| 201.95 | 0.99 | 0.17 | 3.703 | 424.2 | 3.38 | 0.21 | 3.963 | 667.8 | 2.01 | 0.18 | 4.305 | 465 | 1.02 | 0.12 | 4.525 |
| 202.98 | 0.15 | 0.14 | 3.745 | 425.02 | 1.75 | 0.19 | 4.017 | 669.42 | 0.67 | 0.16 | 4.341 | 466 | 0.2 | 0.09 | 4.588 |
| 203.97 | 0.07 | 0.13 | 3.754 | 426.02 | 0.76 | 0.15 | 4.061 | 670 | 0.05 | 0.14 | 4.361 | 467 | 0.04 | 0.08 | 4.608 |

| | | | | | | | | | | | | | | | |
|---|---|---|---|---|---|---|---|---|---|---|---|---|---|---|---|
| 205 | 0.91 | 0.15 | 3.72 | 427.38 | 0.16 | 0.1 | 4.099 | 672.33 | 0.52 | 0.12 | 4.372 | 468 | 0.78 | 0.1 | 4.586 |
| 206.03 | 2.18 | 0.19 | 3.676 | 428.02 | 0.13 | 0.09 | 4.109 | 673.18 | 0.91 | 0.14 | 4.34 | 469 | 2.08 | 0.13 | 4.516 |
| 206.97 | 3.51 | 0.22 | 3.623 | 429.02 | 1.09 | 0.15 | 4.065 | 674.57 | 3.1 | 0.16 | 4.293 | 470 | 3.38 | 0.15 | 4.503 |
| 208 | 4.34 | 0.23 | 3.591 | 430.55 | 2.46 | 0.19 | 4.015 | 676.12 | 3.8 | 0.18 | 4.278 | 471 | 4.31 | 0.17 | 4.45 |
| 208.98 | 4.08 | 0.23 | 3.595 | 431 | 4.12 | 0.22 | 3.948 | 677.18 | 4.37 | 0.19 | 4.258 | 472 | 4.45 | 0.17 | 4.448 |
| 210.55 | 2.8 | 0.22 | 3.63 | 432 | 4.4 | 0.23 | 3.955 | 677.67 | 4.09 | 0.2 | 4.264 | 473 | 3.7 | 0.16 | 4.467 |
| 211.65 | 0.99 | 0.2 | 3.706 | 433.03 | 4.37 | 0.23 | 3.954 | 678.73 | 3.51 | 0.19 | 4.274 | 474 | 2.43 | 0.14 | 4.504 |
| 212.98 | 1.04 | 0.17 | 3.697 | 434 | 3.36 | 0.21 | 3.968 | 679.75 | 2.45 | 0.18 | 4.292 | 475 | 1.08 | 0.12 | 4.546 |
| 213.97 | 0.15 | 0.13 | 3.743 | 435 | 2.07 | 0.19 | 4.005 | 680.58 | 1.24 | 0.15 | 4.314 | 476 | 0.2 | 0.09 | 4.592 |
| 214.95 | 0.06 | 0.13 | 3.757 | 436.03 | 0.81 | 0.15 | 4.072 | 681.58 | 0.07 | 0.13 | 4.36 | 477 | 0.04 | 0.08 | 4.634 |
| 215.97 | 0.82 | 0.15 | 3.726 | 437.02 | 0.15 | 0.1 | 4.114 | 682.57 | 0.31 | 0.12 | 4.371 | 478 | 0.84 | 0.1 | 4.587 |
| 216.77 | 1.89 | 0.19 | 3.686 | 438.02 | 0.14 | 0.09 | 4.123 | 683.9 | 0.77 | 0.14 | 4.356 | 479 | 2.09 | 0.13 | 4.542 |
| 217.98 | 3.54 | 0.22 | 3.63 | 439 | 1.08 | 0.15 | 4.091 | 684.65 | 2.13 | 0.16 | 4.306 | 480 | 3.33 | 0.15 | 4.508 |
| 218.97 | 4.33 | 0.23 | 3.601 | 440 | 2.44 | 0.19 | 4.041 | 686.27 | 3.46 | 0.18 | 4.273 | 481 | 4.31 | 0.17 | 4.497 |
| 220.03 | 4.38 | 0.23 | 3.597 | 440.98 | 3.71 | 0.22 | 3.949 | 686.9 | 4.25 | 0.19 | 4.256 | 482 | 4.42 | 0.17 | 4.477 |
| 220.93 | 3.76 | 0.22 | 3.612 | 443 | 4.4 | 0.23 | 3.945 | 687.55 | 4.47 | 0.19 | 4.257 | 483 | 3.69 | 0.16 | 4.497 |
| 221.88 | 2.47 | 0.2 | 3.652 | 444.17 | 4.35 | 0.23 | 3.959 | 688.77 | 3.8 | 0.19 | 4.28 | 484 | 2.47 | 0.14 | 4.554 |
| 222.97 | 1.01 | 0.17 | 3.713 | 445.32 | 3.42 | 0.21 | 3.987 | 689.6 | 2.08 | 0.17 | 4.297 | 485 | 1.19 | 0.12 | 4.582 |
| 224.22 | 0.07 | 0.13 | 3.756 | 446.1 | 1.84 | 0.18 | 4.036 | 691.3 | 1.11 | 0.15 | 4.333 | 486 | 0.21 | 0.09 | 4.623 |
| 224.97 | 0.06 | 0.12 | 3.763 | 447 | 0.52 | 0.15 | 4.096 | 691.88 | 0.06 | 0.13 | 4.365 | 487 | 0.05 | 0.08 | 4.639 |
| 225.97 | 0.85 | 0.15 | 3.73 | 448 | 0.13 | 0.1 | 4.139 | 692.93 | 0.04 | 0.12 | 4.468 | 488 | 0.81 | 0.1 | 4.617 |
| 226.97 | 2.17 | 0.19 | 3.682 | 449.02 | 0.15 | 0.09 | 4.162 | 693.57 | 0.65 | 0.14 | 4.331 | 489 | 2.24 | 0.13 | 4.57 |
| 227.97 | 3.49 | 0.22 | 3.638 | 450.02 | 1.07 | 0.15 | 4.094 | 694.83 | 2.17 | 0.16 | 4.3 | 490 | 3.4 | 0.15 | 4.541 |
| 228.95 | 4.3 | 0.23 | 3.611 | 451.02 | 2.43 | 0.19 | 4.042 | 695.57 | 3.29 | 0.18 | 4.273 | 491 | 4.32 | 0.17 | 4.503 |
| 229.97 | 4.44 | 0.23 | 3.608 | 452 | 3.71 | 0.21 | 4.003 | 696.67 | 4.44 | 0.19 | 4.267 | 492 | 4.39 | 0.17 | 4.502 |
| 230.95 | 3.7 | 0.22 | 3.625 | 453.02 | 4.39 | 0.23 | 3.98 | 697.67 | 4.37 | 0.19 | 4.25 | 493 | 3.66 | 0.16 | 4.516 |
| 231.95 | 2.4 | 0.2 | 3.665 | 454.12 | 4.34 | 0.23 | 3.975 | 698.57 | 3.47 | 0.19 | 4.266 | 493 | 2.42 | 0.14 | 4.554 |
| 232.93 | 1.07 | 0.17 | 3.713 | 455.02 | 3.37 | 0.21 | 3.999 | 699.55 | 2.08 | 0.17 | 4.29 | 494 | 1.02 | 0.12 | 4.597 |
| 233.93 | 0.19 | 0.14 | 3.763 | 456 | 1.9 | 0.18 | 4.057 | 700.57 | 0.96 | 0.15 | 4.315 | 495 | 0.2 | 0.08 | 4.642 |
| 234.95 | 0.06 | 0.13 | 3.777 | 457.02 | 0.78 | 0.15 | 4.114 | 701.57 | 0.26 | 0.13 | 4.343 | 496 | 0.04 | 0.08 | 4.655 |
| 235.92 | 0.75 | 0.15 | 3.751 | 458.02 | 0.17 | 0.1 | 4.164 | 702.92 | 0.06 | 0.12 | 4.353 | 497 | 0.8 | 0.1 | 4.628 |
| 236.93 | 2.1 | 0.19 | 3.699 | 459.02 | 0.15 | 0.09 | 4.186 | 703.8 | 0.76 | 0.14 | 4.334 | 498 | 2.23 | 0.13 | 4.584 |
| 237.95 | 3.45 | 0.22 | 3.653 | 460 | 1.07 | 0.15 | 4.112 | 704.63 | 1.9 | 0.16 | 4.307 | 499 | 3.45 | 0.15 | 4.545 |
| 238.93 | 4.31 | 0.23 | 3.621 | 461 | 2.45 | 0.19 | 4.073 | 705.73 | 3.23 | 0.18 | 4.285 | 500 | 4.3 | 0.17 | 4.522 |
| 240.97 | 4.45 | 0.23 | 3.62 | 462.12 | 3.73 | 0.21 | 4.028 | 706.57 | 4.24 | 0.19 | 4.266 | 501 | 4.45 | 0.17 | 4.515 |
| 241.95 | 3.63 | 0.22 | 3.633 | 463.03 | 4.4 | 0.23 | 3.998 | 707.57 | 4.44 | 0.19 | 4.256 | 502 | 3.7 | 0.16 | 4.532 |
| 242.95 | 2.39 | 0.2 | 3.674 | 464.02 | 4.3 | 0.23 | 3.992 | 708.55 | 3.45 | 0.19 | 4.291 | 503 | 2.05 | 0.14 | 4.599 |
| 243.95 | 1.06 | 0.17 | 3.727 | 465.02 | 3.38 | 0.21 | 4.023 | 709.57 | 2.23 | 0.17 | 4.3 | 504 | 1.07 | 0.12 | 4.618 |
| 244.93 | 0.18 | 0.14 | 3.769 | 466.02 | 2.03 | 0.18 | 4.054 | 710.58 | 1.15 | 0.15 | 4.338 | 505 | 0.19 | 0.08 | 4.679 |
| 245.93 | 0.07 | 0.13 | 3.78 | 467 | 0.82 | 0.15 | 4.113 | 711.6 | 0.16 | 0.13 | 4.346 | 506 | 0.04 | 0.08 | 4.67 |
| 246.95 | 0.76 | 0.15 | 3.755 | 468.23 | 0.16 | 0.1 | 4.172 | 712.53 | 0.05 | 0.12 | 4.377 | 507 | 0.74 | 0.1 | 4.65 |
| 247.93 | 2.14 | 0.19 | 3.706 | 469.02 | 0.15 | 0.09 | 4.186 | 713.73 | 0.64 | 0.14 | 4.341 | 508 | 2.16 | 0.13 | 4.597 |
| 248.9 | 3.41 | 0.22 | 3.664 | 469.98 | 1.35 | 0.15 | 4.122 | 714.55 | 1.89 | 0.16 | 4.348 | 509 | 3.55 | 0.15 | 4.58 |
| 249.97 | 4.32 | 0.23 | 3.635 | 471 | 2.42 | 0.19 | 4.093 | 715.57 | 3.26 | 0.18 | 4.287 | 510 | 4.32 | 0.17 | 4.533 |
| 250.93 | 4.43 | 0.23 | 3.627 | 472.02 | 3.71 | 0.22 | 4.052 | 716.57 | 4.23 | 0.19 | 4.263 | 511 | 4.45 | 0.17 | 4.523 |
| 251.93 | 3.66 | 0.22 | 3.648 | 473.02 | 4.39 | 0.23 | 4.017 | 717.55 | 4.45 | 0.19 | 4.255 | 512 | 3.68 | 0.16 | 4.545 |
| 252.97 | 2.41 | 0.2 | 3.681 | 474.02 | 4.33 | 0.23 | 4.007 | 718.55 | 3.89 | 0.19 | 4.274 | 513 | 2.38 | 0.14 | 4.586 |
| 253.98 | 1.07 | 0.17 | 3.732 | 475 | 3.39 | 0.21 | 4.031 | 719.63 | 2.38 | 0.17 | 4.296 | 514 | 0.98 | 0.12 | 4.624 |
| 254.97 | 0.16 | 0.14 | 3.781 | 476 | 2.03 | 0.19 | 4.082 | 720.55 | 1.3 | 0.15 | 4.32 | 515 | 0.2 | 0.09 | 4.668 |
| 255.92 | 0.07 | 0.13 | 3.787 | 476.98 | 0.83 | 0.15 | 4.139 | 721.88 | 0.28 | 0.13 | 4.342 | 516 | 0.04 | 0.08 | 4.681 |
| 256.95 | 0.74 | 0.15 | 3.765 | 478.1 | 0.17 | 0.1 | 4.196 | 722.77 | 0.06 | 0.12 | 4.35 | 517 | 0.77 | 0.1 | 4.659 |
| 257.95 | 2.09 | 0.19 | 3.717 | 479.02 | 0.16 | 0.09 | 4.209 | 723.72 | 0.61 | 0.14 | 4.355 | 518 | 2.06 | 0.13 | 4.617 |
| 258.95 | 3.48 | 0.22 | 3.669 | 479.98 | 1.15 | 0.15 | 4.153 | 724.75 | 1.86 | 0.16 | 4.297 | 519 | 3.42 | 0.15 | 4.577 |
| 259.92 | 4.33 | 0.23 | 3.642 | 481.02 | 2.43 | 0.19 | 4.108 | 725.75 | 3.34 | 0.18 | 4.273 | 520 | 4.31 | 0.17 | 4.546 |
| 260.93 | 4.45 | 0.24 | 3.634 | 481.98 | 3.65 | 0.22 | 4.069 | 726.57 | 4.21 | 0.19 | 4.25 | 521 | 4.44 | 0.17 | 4.542 |
| 261.97 | 3.74 | 0.23 | 3.649 | 483.05 | 4.4 | 0.23 | 4.028 | 727.57 | 4.38 | 0.2 | 4.242 | 522 | 3.68 | 0.16 | 4.56 |
| 262.92 | 2.4 | 0.2 | 3.692 | 483.98 | 4.36 | 0.23 | 4.041 | 728.57 | 3.67 | 0.19 | 4.275 | 523 | 2.46 | 0.14 | 4.595 |
| 264.1 | 1.13 | 0.17 | 3.739 | 485.18 | 3.4 | 0.22 | 4.066 | 729.57 | 2.37 | 0.17 | 4.293 | 524 | 0.89 | 0.12 | 4.647 |
| 265.05 | 0.12 | 0.14 | 3.795 | 486.58 | 2.11 | 0.19 | 4.087 | 730.58 | 1.02 | 0.15 | 4.301 | 525 | 0.19 | 0.09 | 4.681 |
| 265.97 | 0.04 | 0.13 | 3.804 | 487 | 0.65 | 0.15 | 4.157 | 731.58 | 0.16 | 0.13 | 4.343 | 526 | 0.04 | 0.08 | 4.715 |
| 266.93 | 0.79 | 0.15 | 3.772 | 488.02 | 0.16 | 0.1 | 4.224 | 732.53 | 0.05 | 0.12 | 4.347 | 527 | 0.76 | 0.1 | 4.669 |

| | | | | | | | | | | | | | | | |
|---|---|---|---|---|---|---|---|---|---|---|---|---|---|---|---|
| 267.82 | 2.1 | 0.19 | 3.719 | 489.13 | 0.15 | 0.09 | 4.251 | 733.55 | 0.65 | 0.14 | 4.313 | 528 | 2.38 | 0.13 | 4.616 |
| 268.93 | 3.21 | 0.22 | 3.68 | 490 | 1.05 | 0.15 | 4.174 | 734.58 | 1.87 | 0.16 | 4.308 | 529 | 3.52 | 0.16 | 4.582 |
| 269.95 | 4.3 | 0.23 | 3.65 | 491 | 2.61 | 0.19 | 4.105 | 735.58 | 3.23 | 0.18 | 4.262 | 530 | 4.29 | 0.17 | 4.557 |
| 271.43 | 4.44 | 0.24 | 3.642 | 492 | 3.7 | 0.22 | 4.066 | 736.57 | 4.21 | 0.19 | 4.237 | 531 | 4.47 | 0.17 | 4.549 |
| 271.87 | 3.07 | 0.23 | 3.677 | 493 | 4.39 | 0.23 | 4.043 | 737.55 | 4.45 | 0.2 | 4.226 | 532 | 3.73 | 0.16 | 4.565 |
| 272.93 | 2.51 | 0.21 | 3.697 | 494 | 4.35 | 0.23 | 4.036 | 738.55 | 3.92 | 0.19 | 4.237 | 533 | 2.48 | 0.15 | 4.599 |
| 273.87 | 1.12 | 0.18 | 3.745 | 494.97 | 3.43 | 0.22 | 4.057 | 739.55 | 2.69 | 0.18 | 4.26 | 534 | 1.05 | 0.12 | 4.647 |
| 274.95 | 0.24 | 0.14 | 3.79 | 496.2 | 2.08 | 0.19 | 4.107 | 740.55 | 1.28 | 0.16 | 4.286 | 535 | 0.21 | 0.09 | 4.685 |
| 275.95 | 0.04 | 0.13 | 3.807 | 497.15 | 0.86 | 0.15 | 4.164 | 741.57 | 0.27 | 0.14 | 4.332 | 536 | 0.44 | 0.08 | 4.709 |
| 276.98 | 0.8 | 0.15 | 3.779 | 498.32 | 0.13 | 0.1 | 4.234 | 742.55 | 0.04 | 0.12 | 4.346 | 537 | 0.76 | 0.1 | 4.679 |
| 277.93 | 2.1 | 0.19 | 3.735 | 499 | 0.12 | 0.09 | 4.243 | 743.57 | 0.6 | 0.14 | 4.309 | 538 | 2.05 | 0.13 | 4.64 |
| 278.92 | 3.39 | 0.22 | 3.686 | 500.02 | 1.42 | 0.15 | 4.156 | 744.57 | 1.82 | 0.16 | 4.284 | 539 | 3.4 | 0.16 | 4.601 |
| 279.93 | 4.3 | 0.24 | 3.658 | 500.98 | 2.39 | 0.19 | 4.142 | 745.73 | 3.21 | 0.18 | 4.244 | 539 | 4.27 | 0.17 | 4.571 |
| 280.92 | 4.45 | 0.24 | 3.65 | 501.98 | 3.69 | 0.22 | 4.093 | 746.78 | 4.18 | 0.19 | 4.223 | 540 | 4.46 | 0.17 | 4.584 |
| 281.92 | 3.66 | 0.23 | 3.663 | 503 | 4.4 | 0.23 | 4.052 | 747.55 | 4.45 | 0.2 | 4.217 | 541 | 3.72 | 0.17 | 4.584 |
| 282.92 | 2.41 | 0.21 | 3.702 | 504.02 | 4.37 | 0.23 | 4.067 | 748.62 | 3.82 | 0.19 | 4.237 | 542 | 2.43 | 0.15 | 4.617 |
| 283.95 | 1.14 | 0.18 | 3.748 | 504.98 | 4.52 | 0.22 | 4.096 | 749.55 | 2.65 | 0.18 | 4.245 | 543 | 1.07 | 0.12 | 4.663 |
| 284.92 | 0.2 | 0.14 | 3.803 | 506.17 | 2.05 | 0.19 | 4.123 | 750.55 | 1.31 | 0.16 | 4.269 | 544 | 0.28 | 0.09 | 4.696 |
| 285.95 | 0.03 | 0.13 | 3.82 | 507.17 | 0.85 | 0.16 | 4.174 | 751.63 | 0.17 | 0.14 | 4.308 | 545 | 0.04 | 0.08 | 4.715 |
| 287.13 | 0.77 | 0.16 | 3.789 | 508.1 | 0.14 | 0.1 | 4.25 | 752.57 | 0.07 | 0.13 | 4.318 | 546 | 0.74 | 0.1 | 4.722 |
| 287.97 | 2.32 | 0.19 | 3.723 | 508.98 | 0.16 | 0.09 | 4.26 | 753.6 | 0.6 | 0.14 | 4.296 | 547 | 2.02 | 0.13 | 4.649 |
| 288.8 | 3.42 | 0.22 | 3.685 | 509.98 | 1.14 | 0.15 | 4.191 | 755.03 | 1.96 | 0.17 | 4.266 | 548 | 3.65 | 0.16 | 4.626 |
| 289.88 | 4.23 | 0.24 | 3.664 | 511 | 2.33 | 0.19 | 4.142 | 756.38 | 3.19 | 0.18 | 4.241 | 549 | 4.29 | 0.17 | 4.586 |
| 290.92 | 4.45 | 0.24 | 3.65 | 512 | 3.65 | 0.22 | 4.097 | 757.7 | 4.19 | 0.2 | 4.223 | 550 | 4.32 | 0.17 | 4.578 |
| 291.95 | 3.68 | 0.23 | 3.668 | 513.28 | 4.4 | 0.23 | 4.068 | 758.55 | 4.46 | 0.2 | 4.213 | 551 | 3.7 | 0.17 | 4.596 |
| 292.93 | 2.42 | 0.21 | 3.704 | 514.05 | 4.35 | 0.23 | 4.079 | 759.57 | 3.89 | 0.19 | 4.226 | 552 | 2.44 | 0.15 | 4.659 |
| 293.93 | 1.09 | 0.18 | 3.763 | 515.02 | 3.08 | 0.22 | 4.102 | 760.57 | 2.65 | 0.18 | 4.261 | 553 | 1.21 | 0.12 | 4.676 |
| 294.98 | 0.2 | 0.14 | 3.812 | 516 | 2.03 | 0.19 | 4.141 | 761.57 | 0.71 | 0.16 | 4.305 | 554 | 0.18 | 0.09 | 4.722 |
| 295.95 | 0.05 | 0.13 | 3.825 | 517.03 | 0.82 | 0.16 | 4.191 | 762.55 | 0.06 | 0.14 | 4.31 | 555 | 0.04 | 0.08 | 4.74 |
| 296.9 | 0.77 | 0.16 | 3.794 | 518.03 | 0.17 | 0.1 | 4.262 | 763.55 | 0.06 | 0.13 | 4.328 | 556 | 0.98 | 0.11 | 4.705 |
| 298.08 | 2.01 | 0.2 | 3.743 | 519.12 | 0.16 | 0.1 | 4.296 | 764.67 | 0.78 | 0.14 | 4.295 | 557 | 2.03 | 0.14 | 4.667 |
| 298.9 | 3.58 | 0.23 | 3.689 | 519.98 | 1.04 | 0.16 | 4.223 | 765.57 | 1.87 | 0.17 | 4.275 | 558 | 3.39 | 0.16 | 4.626 |
| 300 | 4.29 | 0.24 | 3.665 | 521.02 | 2.54 | 0.19 | 4.15 | 767 | 3.25 | 0.19 | 4.348 | 559 | 4.28 | 0.17 | 4.6 |
| 300.92 | 4.44 | 0.24 | 3.661 | 522.23 | 3.64 | 0.22 | 4.129 | 767.68 | 4.19 | 0.2 | 4.222 | 560 | 4.45 | 0.18 | 4.591 |
| 302 | 3.78 | 0.24 | 3.676 | 523.12 | 4.42 | 0.24 | 4.104 | 768.6 | 4.45 | 0.2 | 4.217 | 561 | 3.29 | 0.17 | 4.619 |
| 302.93 | 2.34 | 0.21 | 3.716 | 524.12 | 4.25 | 0.24 | 4.111 | 769.8 | 3.95 | 0.19 | 4.224 | 562 | 2.49 | 0.15 | 4.652 |
| 303.95 | 1.14 | 0.18 | 3.761 | 525.02 | 3.31 | 0.22 | 4.137 | 770.57 | 2.72 | 0.18 | 4.264 | 563 | 1.02 | 0.12 | 4.698 |
| 304.9 | 0.19 | 0.15 | 3.82 | 526.02 | 1.93 | 0.19 | 4.168 | 771.58 | 1.13 | 0.16 | 4.275 | 565 | 0.1 | 0.09 | 4.745 |
| 305.9 | 0.05 | 0.13 | 3.833 | 527.02 | 0.83 | 0.16 | 4.216 | 772.33 | 0.27 | 0.14 | 4.314 | 566 | 0.05 | 0.08 | 4.783 |
| 306.95 | 0.73 | 0.16 | 3.802 | 528.17 | 0.18 | 0.1 | 4.295 | 772.6 | 0.06 | 0.13 | 4.324 | 567 | 0.78 | 0.11 | 4.75 |
| 307.97 | 2.08 | 0.2 | 3.748 | 528.97 | 0.16 | 0.1 | 4.305 | 773.55 | 0.74 | 0.14 | 4.294 | 568 | 2.03 | 0.14 | 4.706 |
| 308.95 | 3.42 | 0.23 | 3.704 | 530 | 1.23 | 0.16 | 4.218 | 774.53 | 1.9 | 0.17 | 4.267 | 569 | 3.39 | 0.16 | 4.641 |
| 309.95 | 4.31 | 0.24 | 3.676 | 531 | 2.35 | 0.2 | 4.174 | 775.55 | 3.48 | 0.19 | 4.234 | 570 | 4.28 | 0.17 | 4.614 |
| 311.02 | 4.45 | 0.25 | 3.669 | 531.98 | 3.65 | 0.22 | 4.128 | 776.63 | 4.2 | 0.2 | 4.223 | 571 | 4.44 | 0.18 | 4.609 |
| 312.28 | 3.67 | 0.24 | 3.68 | 533.02 | 4.39 | 0.24 | 4.12 | 777.55 | 4.44 | 0.2 | 4.222 | 572 | 3.73 | 0.17 | 4.624 |
| 312.95 | 1.96 | 0.21 | 3.738 | 534.15 | 4.38 | 0.24 | 4.098 | 778.57 | 4.08 | 0.2 | 4.227 | 573 | 2.45 | 0.15 | 4.681 |
| 313.98 | 1.09 | 0.18 | 3.77 | 535 | 3.48 | 0.22 | 4.12 | 779.57 | 3.82 | 0.18 | 4.25 | 574 | 1.13 | 0.13 | 4.7 |
| 314.93 | 0.19 | 0.15 | 3.824 | 536 | 1.93 | 0.19 | 4.187 | 780.57 | 1.35 | 0.16 | 4.278 | 575 | 0.21 | 0.09 | 4.75 |
| 315.95 | 0.04 | 0.13 | 3.839 | 536.97 | 0.84 | 0.16 | 4.237 | 781.55 | 0.31 | 0.14 | 4.302 | 576 | 0.05 | 0.08 | 4.763 |
| 316.93 | 0.75 | 0.16 | 3.806 | 538 | 0.18 | 0.1 | 4.303 | 782.57 | 0.07 | 0.13 | 4.308 | 577 | 0.72 | 0.11 | 4.734 |
| 317.92 | 2.04 | 0.2 | 3.755 | 539.02 | 0.16 | 0.1 | 4.297 | 783.55 | 0.58 | 0.15 | 4.299 | 578 | 2.06 | 0.14 | 4.712 |
| 318.93 | 3.33 | 0.23 | 3.709 | 540.32 | 0.98 | 0.16 | 4.238 | 784.58 | 1.89 | 0.17 | 4.275 | 579 | 3.35 | 0.16 | 4.639 |
| 319.93 | 4.26 | 0.25 | 3.683 | 541.12 | 2.42 | 0.2 | 4.18 | 786.2 | 3.25 | 0.19 | 4.252 | 580 | 4.29 | 0.18 | 4.625 |
| 321.13 | 4.45 | 0.25 | 3.674 | 541.98 | 3.95 | 0.22 | 4.121 | 786.58 | 4.21 | 0.2 | 4.234 | 581 | 4.44 | 0.18 | 4.611 |
| 321.95 | 3.51 | 0.24 | 3.697 | 543.02 | 4.4 | 0.24 | 4.111 | 787.55 | 4.45 | 0.2 | 4.232 | 582 | 3.54 | 0.17 | 4.635 |
| 322.93 | 2.44 | 0.22 | 3.73 | 544.02 | 4.38 | 0.24 | 4.127 | 788.57 | 3.92 | 0.2 | 4.234 | 583 | 2.46 | 0.15 | 4.664 |
| 323.98 | 1.12 | 0.19 | 3.77 | 545.05 | 3.48 | 0.22 | 4.128 | 790.07 | 2.67 | 0.18 | 4.257 | 584 | 1.18 | 0.13 | 4.704 |
| 325.03 | 0.18 | 0.15 | 3.833 | 546.02 | 2.13 | 0.2 | 4.175 | 790.62 | 1.31 | 0.16 | 4.303 | 585 | 0.19 | 0.1 | 4.755 |
| 325.92 | 0.05 | 0.14 | 3.848 | 547.07 | 0.18 | 0.16 | 4.251 | 791.53 | 0.05 | 0.14 | 4.323 | 586 | 0.04 | 0.08 | 4.77 |
| 326.92 | 0.72 | 0.16 | 3.816 | 547.07 | 0.17 | 0.1 | 4.297 | 792.55 | 0.04 | 0.13 | 4.321 | 587 | 0.71 | 0.11 | 4.748 |
| 327.93 | 1.99 | 0.2 | 3.763 | 547.07 | 0.15 | 0.1 | 4.315 | 793.55 | 0.6 | 0.15 | 4.31 | 588 | 2.06 | 0.14 | 4.727 |

| | | | | | | | | | | | | | | | |
|---|---|---|---|---|---|---|---|---|---|---|---|---|---|---|---|
| 328.92 | 3.35 | 0.23 | 3.719 | 547.07 | 1.02 | 0.16 | 4.259 | 794.57 | 1.86 | 0.17 | 4.286 | 589 | 3.36 | 0.16 | 4.659 |
| 329.97 | 4.25 | 0.25 | 3.686 | 547.07 | 2.33 | 0.2 | 4.197 | 795.57 | 3.8 | 0.19 | 4.249 | 590 | 4.26 | 0.18 | 4.657 |
| 330.85 | 4.45 | 0.25 | 3.679 | 547.07 | 3.95 | 0.21 | 4.186 | 796.55 | 4.24 | 0.2 | 4.242 | 591 | 4.45 | 0.18 | 4.624 |
| 332.9 | 3.83 | 0.24 | 3.69 | 547.07 | 4.4 | 0.22 | 4.121 | 797.55 | 4.46 | 0.2 | 4.239 | 592 | 3.73 | 0.17 | 4.645 |
| 333.9 | 2.48 | 0.22 | 3.731 | 547.07 | 4.38 | 0.24 | 4.111 | 798.55 | 3.87 | 0.2 | 4.265 | 593 | 2.55 | 0.15 | 4.675 |
| 334.9 | 1.2 | 0.19 | 3.78 | 547.07 | 3.48 | 0.22 | 4.128 | 799.55 | 2.69 | 0.18 | 4.289 | 594 | 1.21 | 0.13 | 4.725 |
| 335.9 | 0.83 | 0.15 | 3.8 | 547.07 | 2.13 | 0.2 | 4.175 | 800.55 | 1.33 | 0.16 | 4.298 | 595 | 0.24 | 0.1 | 4.771 |
| 336.9 | 0.63 | 0.14 | 3.823 | 547.07 | 0.18 | 0.16 | 4.251 | 801.55 | 0.3 | 0.14 | 4.342 | 596 | 0.05 | 0.09 | 4.786 |
