# Supplementary material for: Yeast-Driven and Bioimpedance-Sensitive Biohybrid Soft Robots
Source: Cyborg Bionic Syst. 2025 Apr 25;6:0233. doi: 10.34133/cbsystems.0233 (PMC12022396; doi:10.34133/cbsystems.0233)
Supplement: Supplementary 1 — Figs. S1 to S9 Data File S1 Movies S1 and S2 [file cbsystems.0233.f1.zip › Supplimentry Materials.pdf]

Yeast-driven and Bioimpedance-sensitive Biohybrid Soft Robots
Supplementary Material

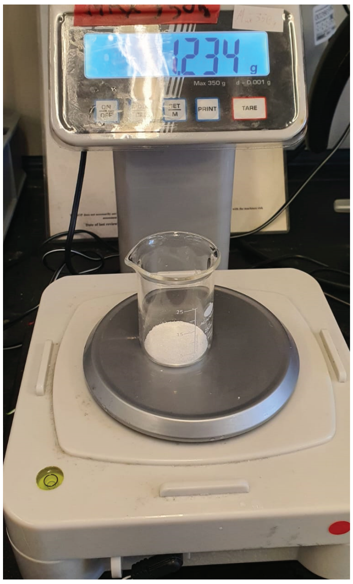

(1)

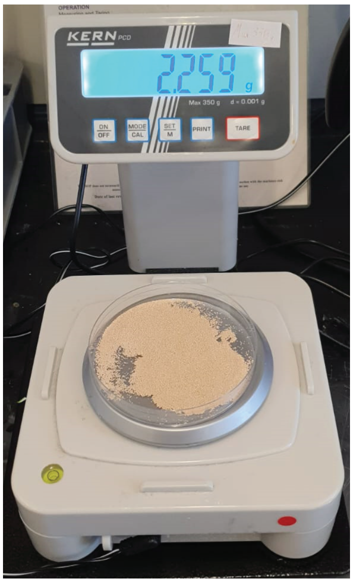

(2)

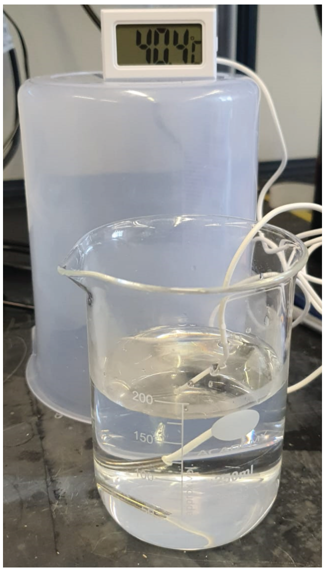

(3)

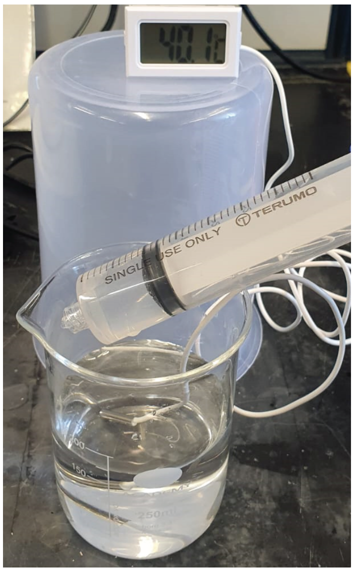

(4)

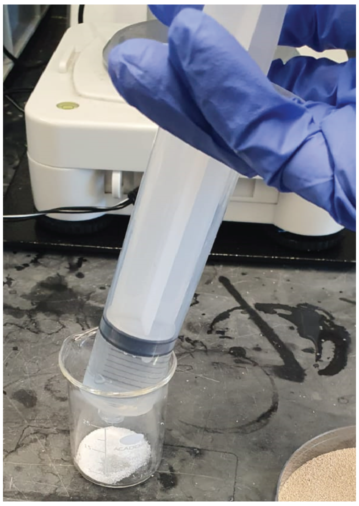

(5)

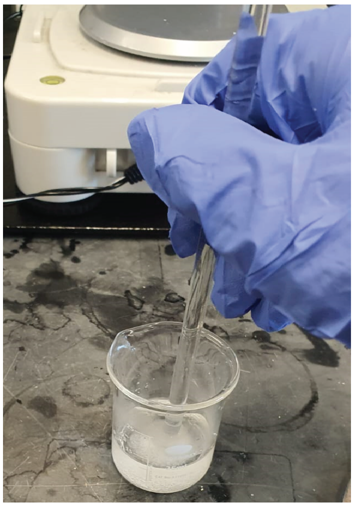

(6)

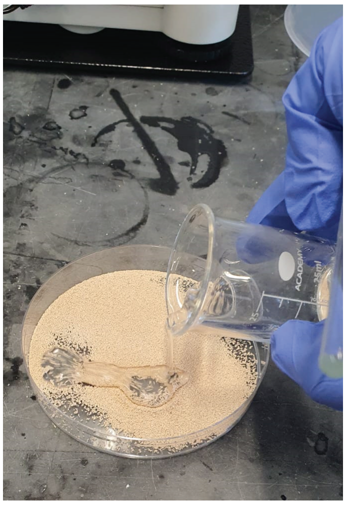

(7)

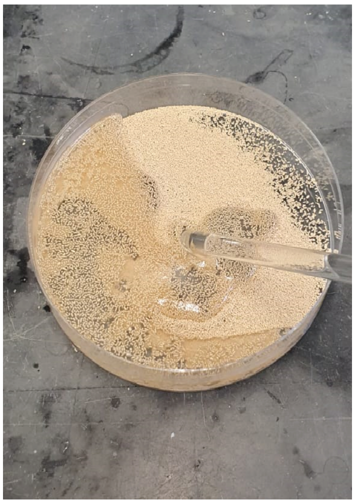

(8)

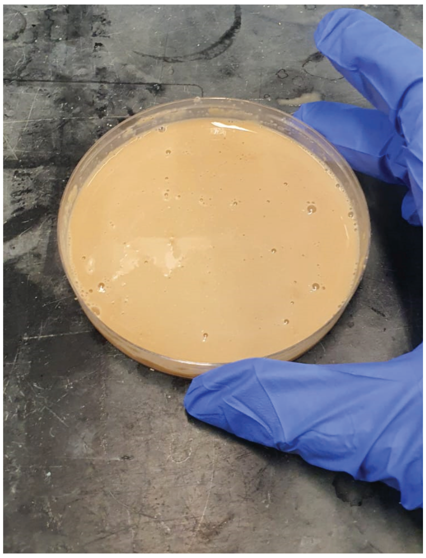

(9)

Fig.S1: Preparing Yeast Mixture. 1-Weight the sugar. 2- Weight the yeast. 3-Warm the water till it is approximately 40 degrees. 4- Take 10 ml of water. 5-,6. Dissolve the sugar with water. 7 Add sugar solution over yeast. 8- Mix yeast with sugar solution. A 9- The yeast mixture is added inside the yeast chamber.

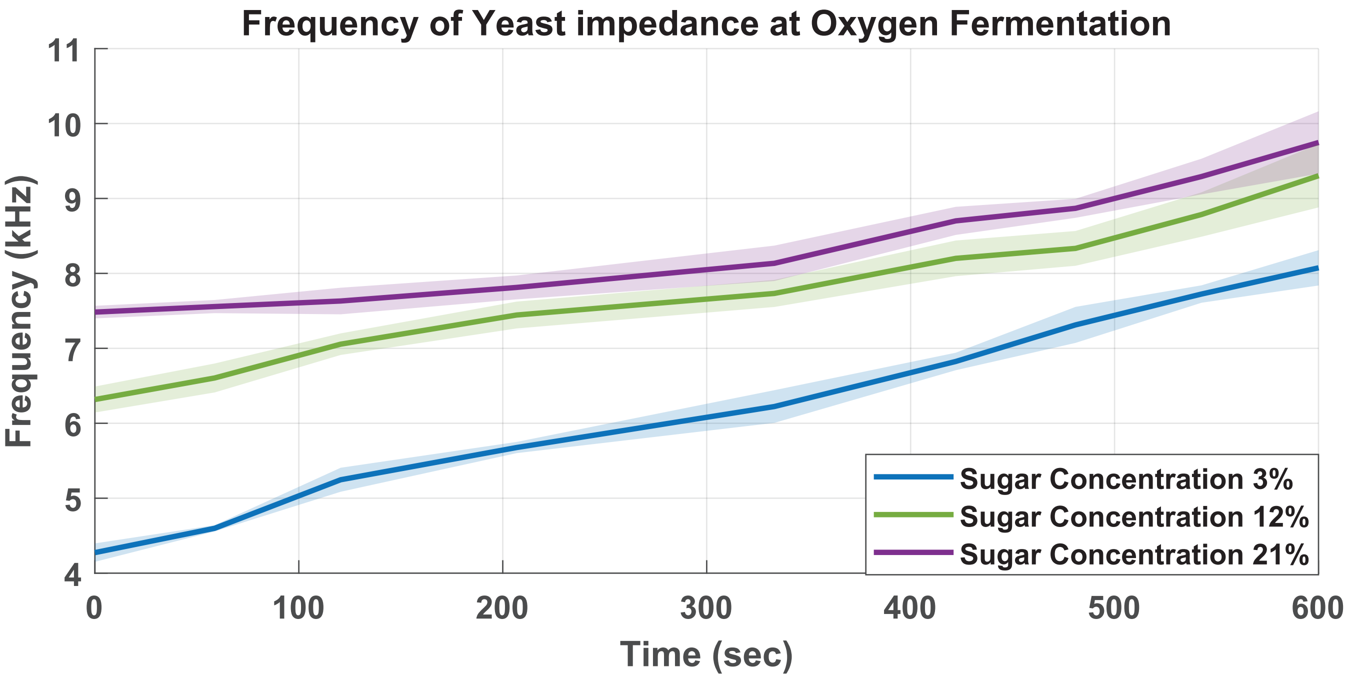

Fig.S2: Frequency of Yeast impedance at Oxygen Fermentation

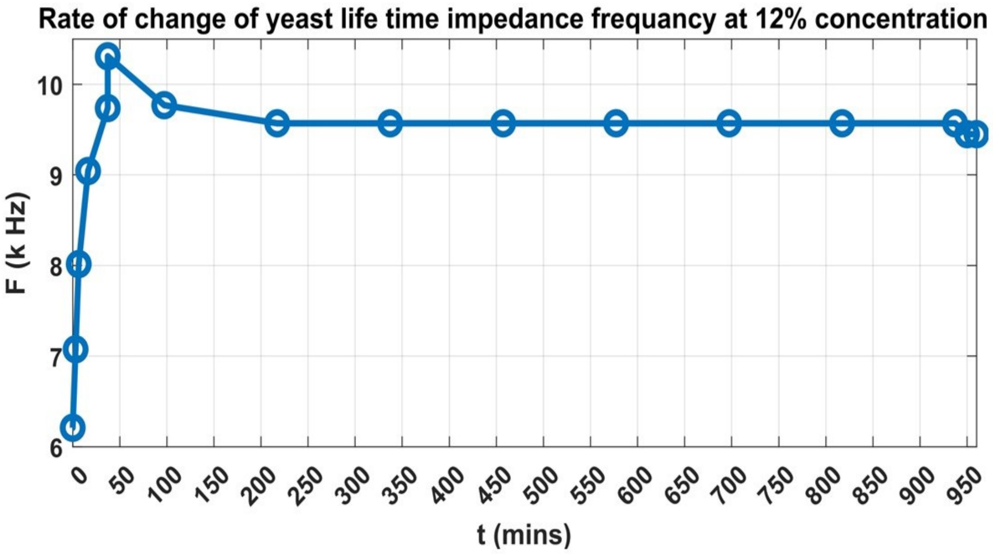

(a)

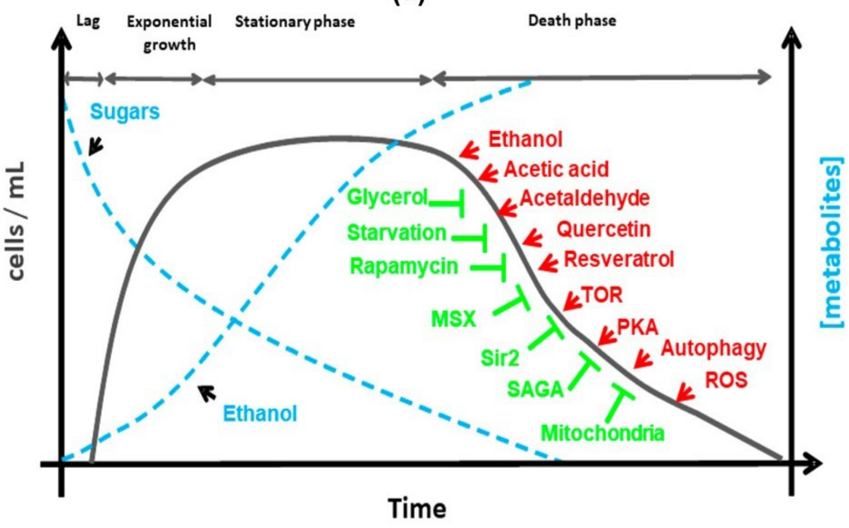

(b)

Figure S3: Yeast Growth Dynamics. (a) Impedance frequency change over 16 hours in a petri dish, reflecting yeast metabolic activity. (b) Growth curve modeled using Equations 1 and 2, highlighting the exponential phase of yeast growth. [Aranda, Agustín, et al."Yeast life span and its impact on food fermentations." Fermentation 5.2 (2019): 37.]

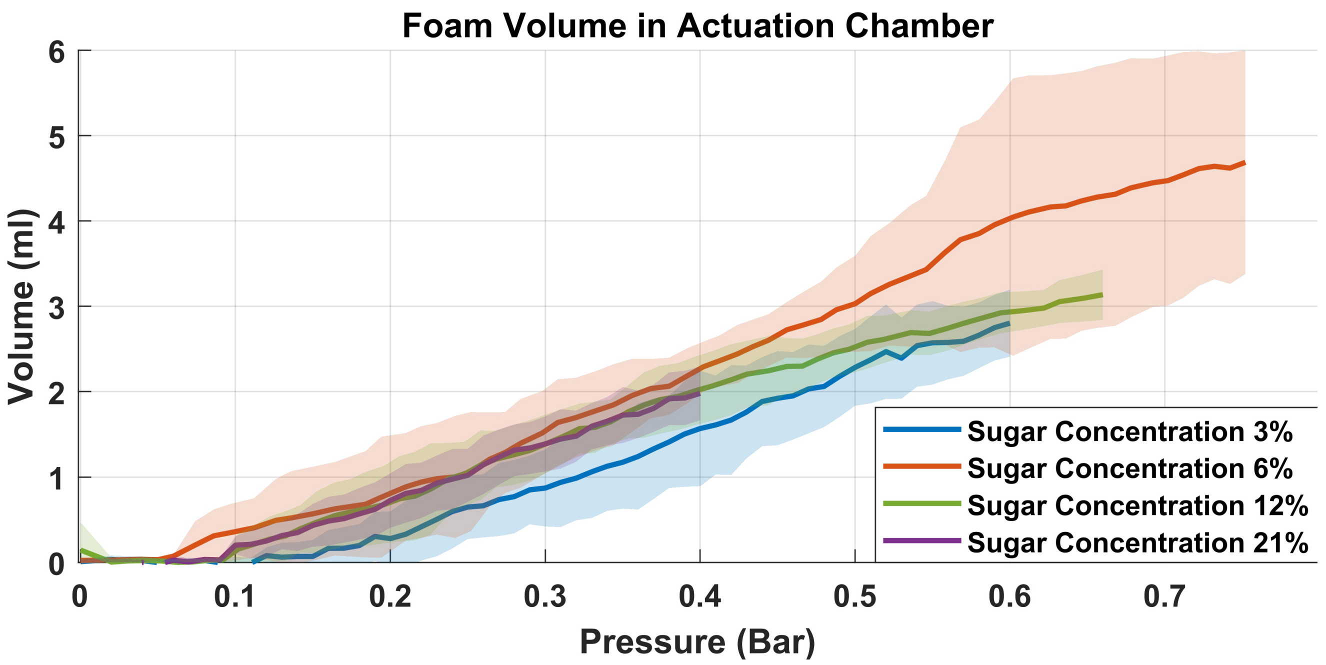

Fig.S4:Foam volume inside the actutation chamber during pressure experment.

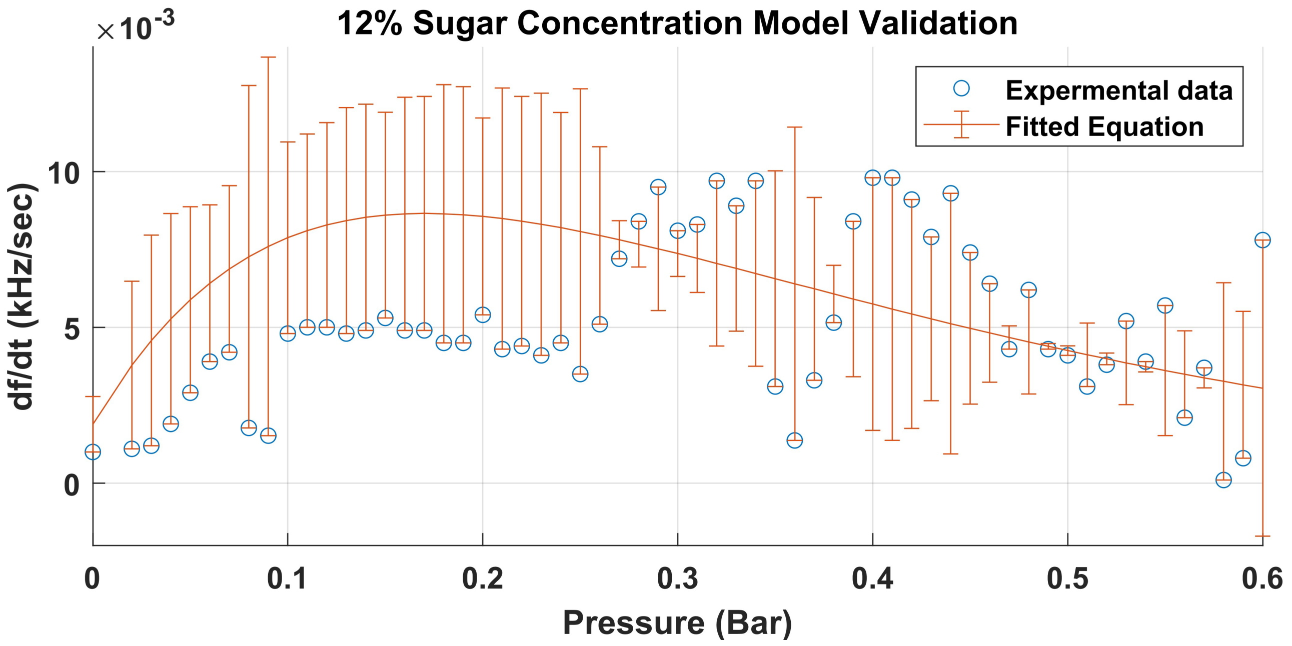

Fig.S5: Yeast impedance rate of change vs pressure of 12% yeast mixture equation validation.

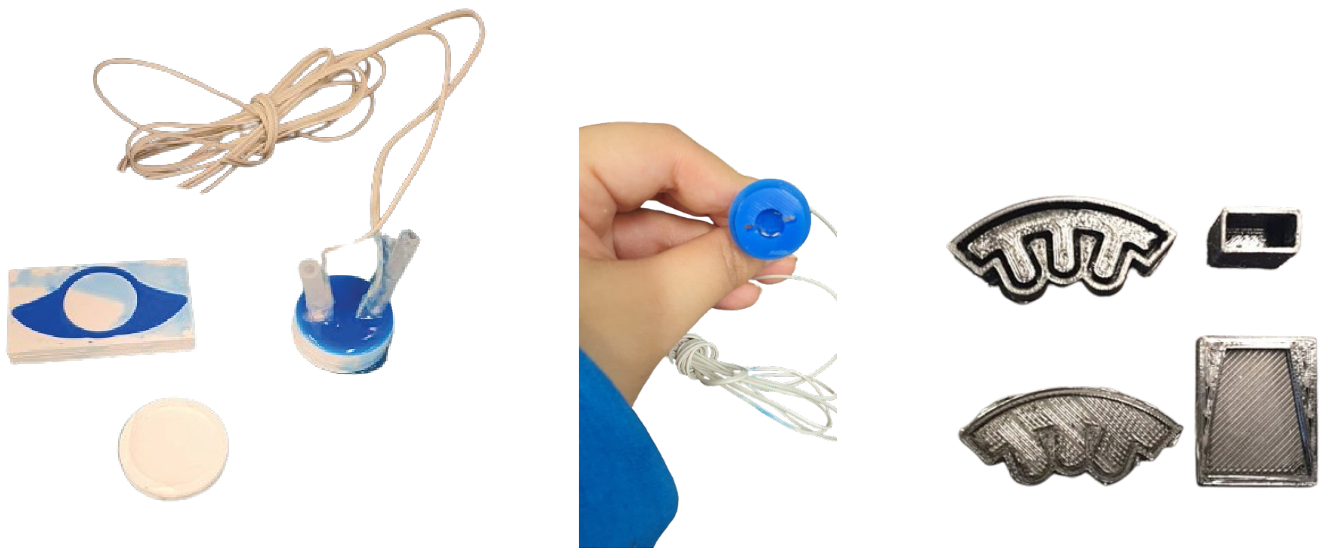

(a) (b) (c)

Fig.S6: Actuators mold.(a) Inflatable actuator molds with impedance electrode. (b) The impedance electrode . (c) Soft limb actuator.

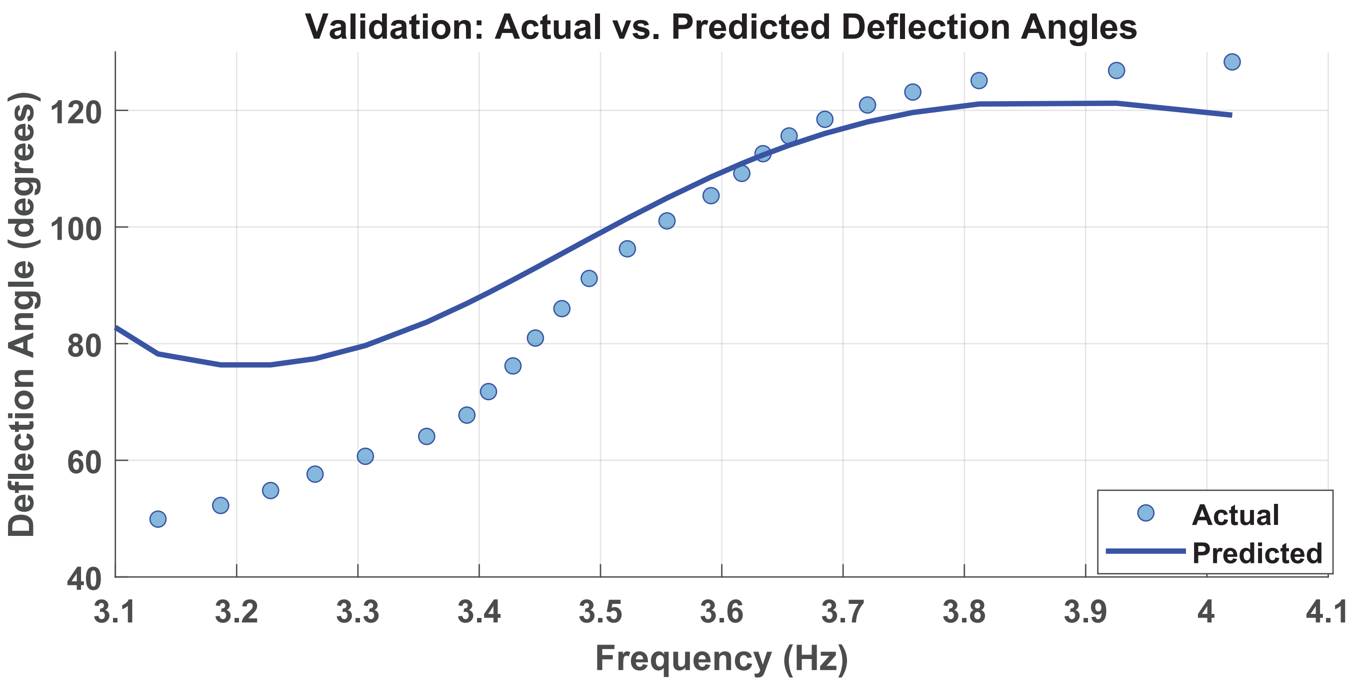

Fig.S7: Validation: Actual vs. Predicted Deflection Angles at 20 degree.

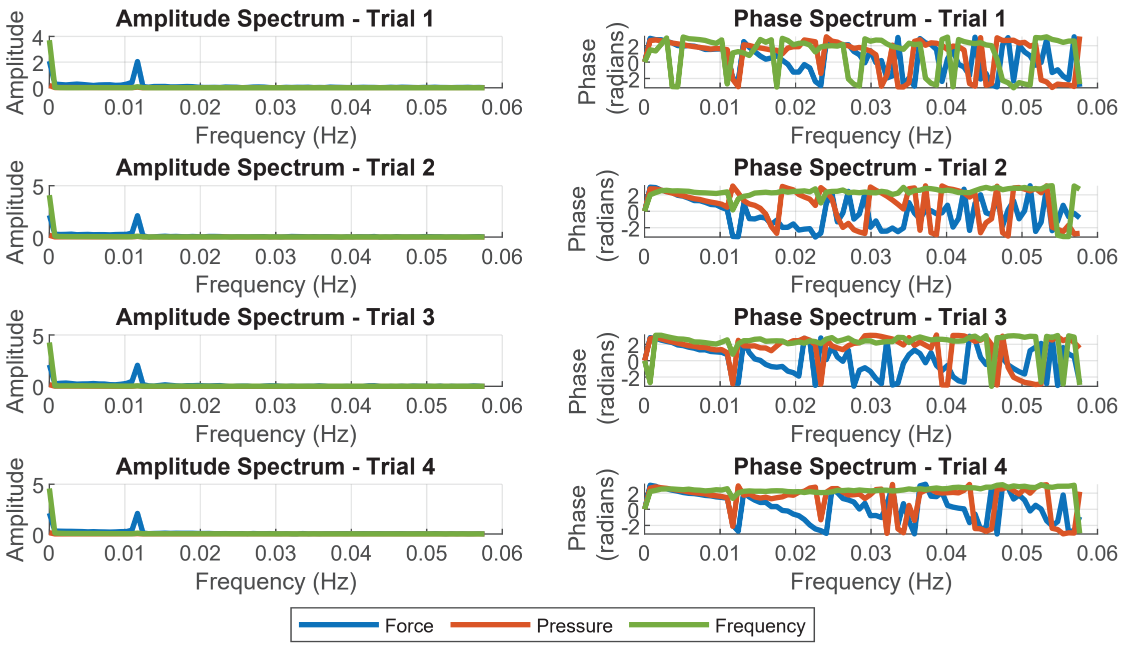

Fig.S8: FFT amplitude and phase spectrum between force , pressure and frequency of tactile experiment.

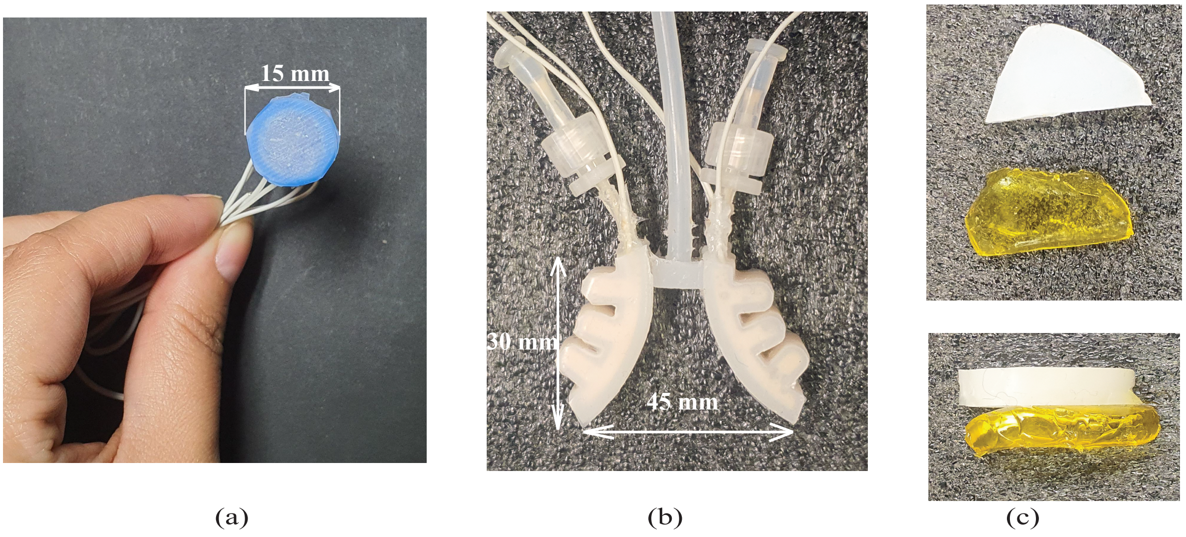

Fig. S9: Yeast-Driven Gripper and Probe.(a) Dimensions of the yeast-driven probe. (b) Dimensions of the yeast-driven gripper. (c) Object gripped by the gripper, weighing approximately 3.11 g and with a thickness of approximately 5 mm.

Movie S1.Illustration and Experiments Video

Movie S2.Yeast-Driven Systems for Tissue Palpation and Gripping Applications
